# Genomic landscape of autism spectrum disorder in Brazil

**DOI:** 10.1590/1678-4685-GMB-2025-0247

**Published:** 2026-09-07

**Authors:** Gabriele da Silva Campos, Claudia Ismania Samogy Costa, Jaqueline Yu Ting Wang, Ana Laura Ferraz, Ana Luiza Filippo, Victor Hugo Calegari de Toledo, Mayla Cristine Fortunata Silva, Carlos Henrique Passos, Diogo Meyer, Flavia Imbroisi Valle Errera, Helena Paula Brentani, Maria Rita Passos-Bueno

**Affiliations:** 1 Universidade de São Paulo, Instituto de Biociências, Departamento de Genética e Biologia Evolutiva, São Paulo, SP, Brazil.; 2 Universidade de São Paulo, Centro de Pesquisa sobre o Genoma Humano e Células Tronco, São Paulo, SP, Brazil.; 3 Universidade Federal do Espírito Santo, Centro de Ciências Humanas e Naturais, Departamento de Ciências Biológicas, Vitória, ES, Brazil.; 4 Universidade de São Paulo, Instituto de Psiquiatria, Departamento de Psiquiatria, São Paulo, SP, Brazil.

**Keywords:** Autism, exome sequencing, genomics

## Abstract

Genomic studies of autism spectrum disorder (ASD) have largely excluded admixed populations. To address this gap, we characterized the genomic landscape of ASD in Brazil by combining a systematic literature review with whole-exome sequencing analysis of 441 Brazilian individuals and their families. Our analysis revealed a conclusive molecular diagnosis in 13.1% of probands. The diagnostic yield was higher among individuals with clinical features, particularly comorbid signs of intellectual disability, hypotonia, and seizures, providing a basis for prioritizing genetic testing. The sample presented a diverse ancestry, with major European, African, and Native American contributions. Notably, more than half of the identified rare risk variants were located on non-European haplotypes. Both *de novo* and inherited variants contributed to ASD risk, and we reinforce *NPAS3* as a candidate ASD risk gene. This study provides the first comprehensive genomic overview of ASD in a large Brazilian cohort, reinforcing the critical need to include diversely admixed populations in genomic research to expand the understanding of ASD architecture and improve diagnostic strategies in resource-limited settings.

## Introduction

Autism spectrum disorder (ASD) is a neurodevelopmental disorder (NDD) characterized by persistent deficits in social communication and interaction, combined with restricted and repetitive patterns of behavior, interests, and/or activities (American Psychiatric Association, 2013). The estimated global prevalence of ASD is 1-2%, being four times more frequent in males than in females (Lord et al., 2020). The interest in ASD genetics dates back to at least the mid-20th century (Folstein and Rutter, 1977). While the definition of “autism” has evolved, the relevance of genetics has never been in doubt, with an estimated heritability of 80% in a population-based study (Bai et al., 2019) and 64-91% for twin studies (Tick et al., 2016).

The genetic architecture of ASD is complex and heterogeneous. Large-scale genomic initiatives were established, such as the Autism Sequencing Consortium (Buxbaum et al., 2012); Simons Simplex Collection (Fischbach and Lord, 2010) and the Simons Foundation Powering Autism Research (SPARK, Feliciano et al., 2018), both from the Simons Foundation Autism Research Initiative (SFARI); the Integrative Psychiatric Research (iPSYCH, Pedersen et al., 2018); and Autism Speaks’s MSSNG (Yuen et al., 2016). These initiatives have provided critical insights by assembling tens of thousands of samples and generating comprehensive datasets based on whole-exome sequencing (WES), whole-genome sequencing (WGS), and genome-wide genotyping arrays, leading to the discovery of causal and risk factors, and supporting multifactorial architecture models. Rare, highly penetrant variants, such as *de novo* copy number variants (CNVs) or damaging sequence variants (single-nucleotide variants - SNVs, and small deletions and insertions - indels), with large effect sizes are identified in approximately 10-20% of autistic individuals, following a monogenic pattern (Yuen *et al.*, 2015; Srivastava et al., 2019). These involve hundreds of genes and genomic regions, with recurrent examples including *FMR1*, *SCN2A*, *CHD8*, *ADNP*, *FOXP1*, and *SYNGAP1,* as well as CNVs at *loci* such as 22q13.3 (associated with the *SHANK3* gene) (Sebat et al., 2007; Pinto et al., 2010; Fu et al., 2022; Zhou et al., 2022). Beyond these, risk is likely shaped by rare variants with moderate effect sizes (oligogenic) and common variants with small effect sizes (polygenic) (Wang et al., 2022). Together, these genetic factors contribute additively, often in interaction with environmental influences, with common variants driving population-level liability and rare variants conferring the greatest risk to individual carriers (Weiner et al., 2017).

While polygenic risk scores (PRS) derived from large-scale genome-wide association studies (GWAS) demonstrate that common variants cumulatively explain a proportion of ASD heritability, each variant individually confers only a modest increase in risk (single SNP OR up to 1.1; Grove et al., 2019). The contribution of rare inherited variants and the oligogenic model is increasingly recognized, suggesting roles for both known and uncharacterized biological pathways (Wilfert et al., 2021).

Although it has a relatively low diagnostic yield, molecular testing in ASD plays a critical role in genetic counseling and is increasingly important for improving clinical guidelines that prioritize individuals most likely to benefit from genomic analyses.

Notably, most genomic discoveries in ASD have been derived from cohorts of predominantly European ancestry (Martin et al., 2019; Fatumo et al., 2022), leaving Latin American and other underrepresented populations largely excluded (Bruxel et al., 2025). The largest ASD study from Latin America, including Brazilian participants, demonstrated their value in identifying novel variants and candidate genes (Avila et al., 2025). Moreover, polygenic risk scores based mostly on European-ancestry GWAS show limited transferability to admixed populations mainly due to differences in linkage disequilibrium and environmental exposures (Martin *et al.*, 2019; Bruxel *et al.*, 2025).

The Brazilian population, shaped by a complex admixture of Native American, European, and African ancestries (Kehdy et al., 2015; Pena et al., 2020; Nunes et al., 2025), offers a valuable opportunity to identify ancestry-enriched risk variants and to expand our understanding of ASD genetics beyond European cohorts. Yet, genomic research on ASD in Brazil remains limited, with most studies relying on small samples, targeted panels, or case reports (Quaio et al., 2025), despite the estimated 2.4 million individuals diagnosed with ASD nationwide (IBGE, 2022).

To address this gap, we review existing literature on genomic analysis in Brazilian ASD individuals and present the largest WES dataset to date, comprising 441 exomes from individuals recruited in São Paulo, including 94 unpublished cases. Therefore, this study provides the first comprehensive overview of the landscape of ASD genomics in Brazil, with emphasis on rare variants and ancestry-related contributions.

## Material and Methods

### Review of ASD genetic and genomics in Brazil

A systematic literature review was performed using the Medline (PubMed) database. The terms were selected based on the Medical Subject Headings (MeSH) vocabulary. The search targeted studies that did not focus on a single locus and reported diagnostic yield, using the search syntax: (((Genomics) OR (Genetics)) AND (Autism)) AND (Brazil). Screening was independently conducted by two reviewers, with disagreements resolved by consensus with a third reviewer. Eligible studies included those published in English, with available abstracts and full texts, analyzing CNV or WES data from individuals with ASD in the Brazilian population. No publication date limits were applied. Data extraction followed a standardized form covering: (1) general article information (first author, publication year, and study design); (2) recruitment details regarding ASD phenotype, state, population, and sample size; and (3) analytical methods, genome assembly, CNV characteristics (cytogenetic location, genomic position, type, and size), implicated genes, and main findings. Only studies addressing most items in categories (2) and (3) were included. Exclusion criteria comprised duplicate publications, preprints, reviews, abstracts, protocols, posters, letters, non-open-access papers, and studies on non-Brazilian individuals or non-human organisms, as well as case reports and single-gene studies. Possible sample duplication across studies was evaluated when sufficient information was available. For WES studies with access to raw sequencing data or detailed sample identifiers, direct cross-study comparisons were performed to assess potential sample overlap. The search was conducted in October of 2025 and the review process followed the Preferred Reporting Items for Systematic Reviews and Meta-Analyses (PRISMA) guidelines (Page et al., 2021) and CNVs identified are presented in Table S1.

### Sample and clinical data

All families were recruited at the Centro de Estudos do Genoma Humano (CEGH; N=1196 individuals) or at the Programa do Transtorno do Espectro Autista do Instituto de Psiquiatria (PROTEA; N=99 individuals) ambulatories, both part of the Universidade de São Paulo (USP) in São Paulo, Brazil. DNA samples were obtained from whole blood or saliva. Blood samples were processed using the QIAsymphony automated system following manufacturer’s instructions. Saliva samples collected with Oragene kits, were processed with prepIT L2P kit following manufacturer’s instructions. Only probands with a prior diagnosis or diagnostic hypothesis of ASD were included. Clinical information was retrieved from clinical records or provided by parents.

Clinical data collected included age at first evaluation, speech development, parental ages, and previous occurrence of seizures or hypotonia. Information was also obtained regarding any additional neuropsychiatric diagnoses in the proband. Presence of comorbid signs of ID was defined as either a formal diagnosis of ID or an evaluator’s indication based on literacy level and ability to perform age-appropriate tasks.

The clinical features included in the association analysis of variant number and genotype-phenotype were defined as: lack of speech (defined as a non-verbal individual that was at least 3 years old by the time of evaluation); presence of hypotonia; presence of seizures; presence of comorbid signs of ADHD; and presence of comorbid signs of ID. The proportion of individuals evaluated for each feature was 80.5% (355/441) for lack of speech, 71.7% (316/441) for hypotonia, 78.9% (348/441) for seizures, 78.9% (348/441) for ADHD comorbid sign, and 42.4% (187/441) for ID comorbid sign. Analysis of sex differences in the frequency of clinical features was performed with Fisher’s exact test.

Family recurrence was defined as the reported presence of a first- or second-degree relative with a prior diagnosis or diagnostic hypothesis of ASD. Parental age at conception was calculated based on the birth dates of the parents and the proband.

CNV information was retrieved from prior genetic test reports when available, including karyotyping, multiplex ligation-dependent probe amplification (MLPA), comparative genomic hybridization microarray (aCGH), and low-pass whole-genome sequencing (LP-WGS). Karyotype was performed in a subset of individuals (N=121) in a clinical setting. MLPA analyses using the SALSA MLPA Probemix P343-C3 Autism-1 assay were performed for research purposes in a subset of individuals (N=131; of those, 65 included in Moreira et al., 2014), which targets clinically relevant chromosomal regions associated with ASD, including 15q11-q13, 16p11.2, and 22q13. Considering different platforms, aCGH was performed in 66 individuals, with either clinical or research purposes (N=40 included in Costa et al., 2022). LP-WGS data from a subset of the sample had been previously analyzed with a focus on clinical cytogenetics (N=177, Mazzonetto et al., 2024). Some individuals had more than one CNV analysis performed, as detailed in Table S2. No additional analyses were performed to identify CNVs via WES in the present study.

The total cohort of 441 probands includes 94 previously unpublished cases and 347 individuals whose sequencing data were part of previous studies (Montenegro et al., 2020; Neri de Souza Reis et al., 2021; Fu et al., 2022; Costa et al., 2023; Avila et al., 2025), with some overlap between cohorts (see Table S2 for details).

### Sequencing

Whole-exome sequencing (WES) data included ASD probands (N=441) and their relatives (N=854), including 812 non-affected parents, 3 affected parents, 24 affected siblings, 8 affected cousins or uncles, and 4 non-affected brothers or uncles, encompassing 1,295 individuals. Sequencing was conducted using Illumina HiSeq 2500 or NovaSeq 6000 platforms (Illumina, Inc., California, USA), in paired-end reads of approximately 100 bp, following the manufacturer’s recommendations by the Autism Sequencing Consortium (N=908), CEGH-CEL (N=288) or PROTEA (N=99) facilities. Sequencing reads were aligned to the human reference genome (hg38). All included samples passed quality control, which comprised reported sex concordance check, mean sequencing coverage ≥20x, and kinship analysis. Briefly, kinship analysis was performed as described in Costa et al. (2025) to identify rare variants most likely inherited by descent, by calculating the proportion of shared rare variants (minor allele frequency <1% in public databases) across high-quality loci, defined as loci successfully genotyped in at least 80% of the samples. Individuals sharing ≥5% of rare variants were considered related to some degree; for parent-offspring relationships, a minimum relatedness threshold of 33% was applied.

### Variant filtering and annotation

WES data processing and variant calling were performed using Burrows-Wheeler Aligner, Picard, and the Genome Analysis Toolkit (GATK, McKenna et al., 2010). Single-nucleotide variants (SNVs) and small insertions or deletions (indels) located in exons or within 50 bp of the splicing junction were called in individual samples using GATK HaplotypeCaller, and were jointly genotyped using GATK GenotypeGVCFs. Multiallelic sites were subsequently split and normalized (left-aligned). A total of 2,762,304 variants were identified prior to multiallelic splitting, which increased to 2,898,862 variants after this step. The mean coverage of exome data from the 441 probands was 79x (±19x), with 95% of the exome being covered by at least 10 reads, and 90% by at least 20 reads, on average.

Variant annotation with Annovar (Wang et al., 2010) included (but was not limited to) variants’ allele frequency in the general population (gnomAD, ABraOM, and 1000 Genomes databases), predictive tools for deleteriousness of the alternate allele (CADD score, REVEL, SpliceAI), gene probability of being loss of function intolerant (pLI) or missense intolerant (Z-score) scores (gnomAD database), and gene-disease association databases (OMIM, ClinVar, SFARI).

Only variants that fit the quality criteria were included in the analysis, including a minimum read depth of 10, an alternate allele read count ≥5, and an allele balance of between 0.3-0.7 for heterozygous variants or ≥0.9 for homozygous variants. Stopgain, stoploss, frameshift insertion, frameshift deletion, and splicing variants (in canonical sites or with spliceAI prediction as VUS or PP3) were classified as loss of function variants (LoF). Missense variants with a CADD score threshold of ≥20 were considered damaging (Schubach et al., 2024), and a stringent score of ≥30 was applied for rare risk variant analysis.

### *De novo* variants analysis

Rare missense, synonymous, and LoF variants (frequency <1% in gnomAD and 1000G databases) with minimum quality criteria were filtered to identify high-confidence *de novo* variants (DNVs) using PossibleDeNovo (Van der Auwera et al., 2013) and DeNovoGear (Ramu et al., 2013). Variants called by both methods were kept for further analysis, considering those classified as low or high confidence by PossibleDeNovo and with a posterior probability of being a *de novo* mutation (ppDNM) from DeNovoGear ≥0.69. Sex chromosome variants were excluded. Only variants that passed GATK’s Variant Quality Score Recalibration (VQSR) filter were retained. To estimate the false positive rate of DNVs, 20% of identified DNVs were visually inspected using the Integrative Genomics Viewer (IGV, Robinson et al., 2011).

To investigate the association between DNV number and a set of predictor variables, the analysis included paternal age at conception, maternal age at conception, sex, and clinical features (as defined in the “Sample and clinical data”). Negative Binomial generalized linear models (GLMs) were applied to account for data overdispersion. To maximize sample retention, each predictor was assessed independently rather than in a single multivariate model. Sequencing kit was first identified as a significant technical confounder and was therefore included as a covariate in all models. p-values from each independent variable test were then collectively adjusted for multiple comparisons using the Benjamini-Hochberg procedure. An association was considered statistically significant when the adjusted p<0.05.

### Rare risk variants analysis

Rare Risk Variants (RRVs) were defined as family private variants with frequency <1% in gnomAD and 1000G populational databases, classified as LoF or damaging missense (as defined in the “Variant filtering and annotation” section) in a neurodevelopment-associated geneset (NDD geneset, N=4,934 genes), with minimum quality criteria defined in the “Variant filtering” section (Costa et al., 2025). Only variants that passed GATK’s VQSR filter were retained. Further analyses were restricted to variants detected in probands. Novel RRVs were defined as variants that were absent from the populational databases. To identify variables associated with the number of RRVs, two distinct analyses were conducted. First, the number of RRVs was analyzed in the proband sample against predictor variables including paternal and maternal age at conception, sex, and clinical features (as defined in the “Sample and clinical data”). Negative Binomial GLMs were employed to account for the count nature and overdispersion of the data, with the sequencing kit included as a technical covariate. p-values from these independent tests were adjusted for multiple comparisons using the Benjamini-Hochberg method. Second, due to the non-normal distribution of the data, the Wilcoxon-Mann-Whitney test was used for direct group comparisons of RRV counts between: (1) probands and parents (mothers and fathers combined), (2) males and females, and (3) affected and unaffected individuals across the entire sample. For each model, the significance of the primary predictor variable was assessed after adjusting for the sequencing kit as a covariate.

Protein-protein interactions (PPI) analysis of the genes harboring RRVs was performed using STRING (Szklarczyk et al., 2023, version 12.0) with a minimum required interaction score of 0.7 at 5% false discovery rate (FDR) stringency, and the NDD geneset as background. The minimum interaction score of 0.7 corresponds to the “high confidence” threshold defined by the STRING consortium (Szklarczyk *et al.*, 2023). This cutoff prioritizes PPI supported by strong experimental evidence and curated database annotations, thereby reducing the inclusion of indirect or purely predicted associations. Given that the analysis focuses on RRVs, a stringent confidence threshold was applied to minimize false-positive interactions and to ensure robust biological interpretation of the resulting network. Gene Ontology (GO) enrichment analysis was also performed using the g:GOSt tool from g:Profiler (Kolberg et al., 2023; version e113_eg59_p19_f6a03c19), with the NDD geneset as background. Multiple testing corrections were applied using the Benjamini-Hochberg FDR method set at 0.05 significance level. Results were reported for biological processes, cellular components, and molecular functions.

### Clinically relevant variants, rate of conclusive cases, and genotype-phenotype correlation analysis

Conclusive cases were classified as those harboring a pathogenic or likely pathogenic (P/LP) sequence variant (SNV or indel) identified by WES or a CNV previously identified by other genetic tests. Pathogenicity annotations were refined using the Franklin Genoox platform and subjected to manual curation. All P/LP variants were classified according to the American College of Medical Genetics and Genomics guidelines (Richards et al., 2015), with a focus on phenotypes related to ASD and other NDD, including intellectual disability (ID), developmental delay, and speech delay. The total rate of conclusive cases was defined as the proportion of individuals carrying a P/LP alteration (either sequence variant or CNV), while the P/LP variant rate was defined as the proportion of individuals carrying a P/LP sequence variant detected by WES. Both rates were calculated by dividing by the total number of probands (N=441) or by the total number of affected individuals (N=476).

Given the limited availability of resources for genetic testing in Brazil, a binary logistic regression model was applied to assess which clinical features were associated with the presence of a P/LP variant detected by WES in probands. Aiming to generate evidence to guide case prioritization by identifying clinical features more likely to yield a molecular diagnosis, the binary outcome variable was defined as the presence of at least one P/LP sequence variant per individual (N=441 probands) The predictor variables included sex and the number of clinical features (as defined in the “Sample and clinical data”). Clinical features were also tested as individual variables, with p-values adjusted for multiple comparisons using the Benjamini-Hochberg procedure. Model’s coefficients were used to calculate odds ratios (OR) and their corresponding 95% confidence interval to quantify the effect size of each predictor.

### Global and local ancestry analysis

*Global ancestry inference*

Global ancestry components (European - EUR, African - AFR, Native American - NAM, and East Asian - EAS) were determined using ADMIXTURE (Alexander et al., 2009) through supervised analysis (K=4, 2000 bootstrap replicates) under a maximum likelihood framework. Parental population references included AFR, EUR, and EAS samples from the 1000 Genomes Project (1000 Genomes Project Consortium *et al.*, 2015), and NAM from published datasets (Human Genome Diversity Project - HGDP, Bergström et al, 2020). An individual was considered to present a given ancestry component when it accounted for at least 5% of the estimated ancestry, and to show predominance of that component when it represented ≥75% of the total ancestry.

*Local ancestry inference*

For local ancestry inference, HGDP samples (Bergström et al., 2020) were used to represent AFR, EUR, NAM, and EAS populations as reference panels. Briefly, each subpopulation was filtered for Hardy-Weinberg equilibrium (p<1e-8) using PLINK v1.9 (Chang et al., 2015). Indels were excluded, keeping only biallelic markers with bcftools (Danecek et al., 2021). Related individuals were removed using KING 2.3.2 (Manichaikul et al., 2010). Additional QC included filtering markers and individuals with excessive missingness using PLINK. Admixed individuals were identified via unsupervised ADMIXTURE (95% ancestry threshold) (Alexander et al., 2009) and excluded (N=32). To balance reference panels, larger subpopulations were downsampled to 40 individuals per continental group. For the ASD dataset, analogous QC steps were applied, and phasing was performed with SHAPEIT5 (Hofmeister et al., 2023). Local ancestry proportions were estimated with G-Nomix (Hilmarsson et al., 2021), intersecting phased VCFs for admixed and reference samples to ensure 100% overlap. Model training used UCSC genetic maps, and phasing errors were corrected with Gnofix, leveraging ancestry information.

### Statistical analysis

All statistical analyses and visualizations were conducted using R software (version 4.5.0). Detailed specific statistical analysis is described above sections. Statistical significance was defined as a p<0.05.

## Results

### Review of ASD genetic and genomics in Brazil

**Figure 1 -:**
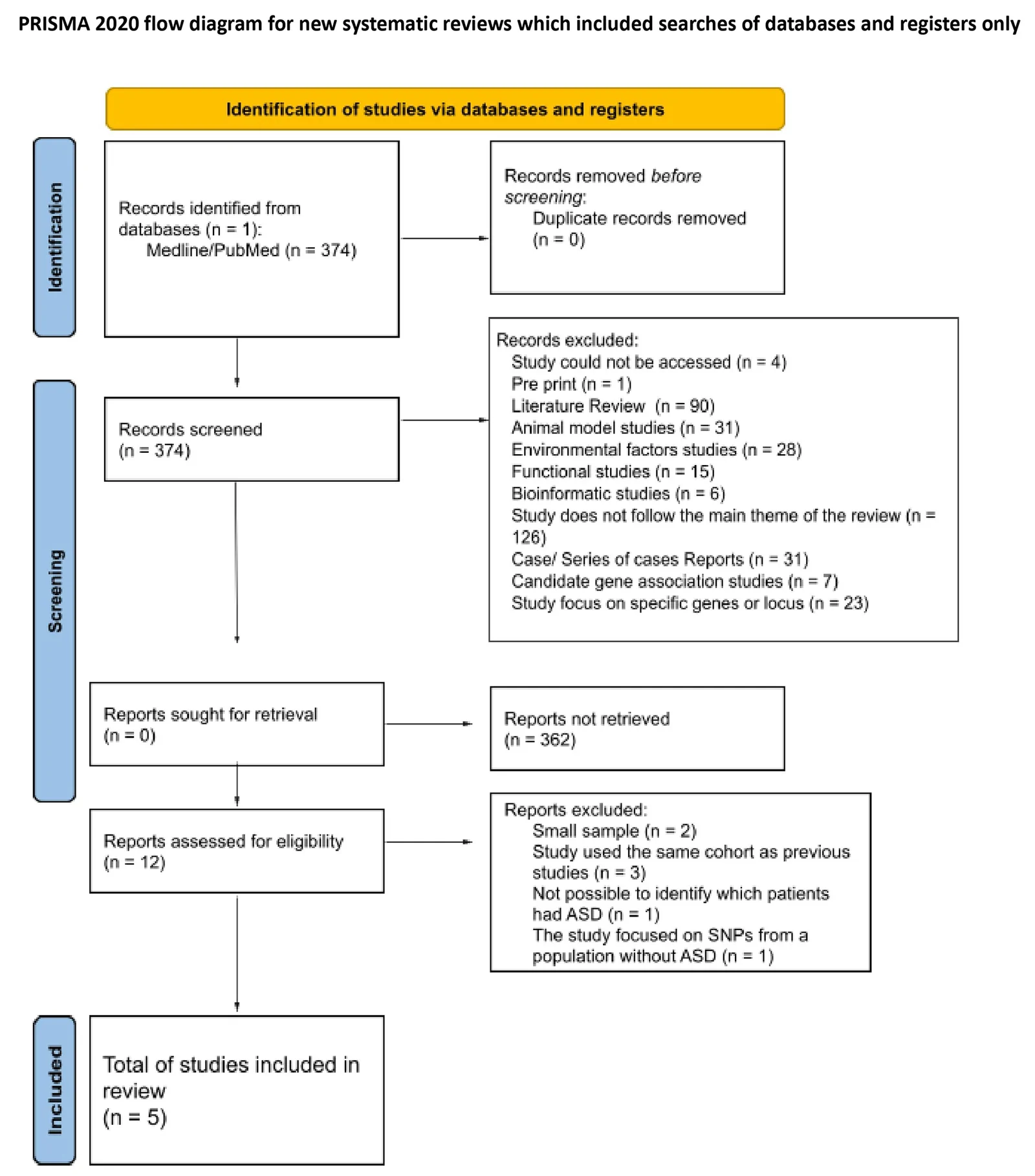
PRISMA flow diagram of the systematic literature review process. The diagram illustrates the different phases of study identification, screening, eligibility assessment, and final inclusion. It details the number of records identified from the PubMed/Medline database, the number of records excluded based on predefined criteria (e.g., duplicates, reviews, case reports, non-Brazilian cohorts), and the final number of studies included in the qualitative synthesis summarizing the diagnostic yield of CNV and WES analyses in Brazilian ASD cohorts.

A total of 374 records were systematically retrieved from the PubMed/Medline database. Based on the exclusion criteria, 362 records were removed (Figure 1). Of the 12 studies selected for detailed evaluation, five used a genomic approach and met the eligibility criteria. Two focused on CNV analysis and three on WES analysis (Table 1). The studies encompassed cohorts ranging from a few dozen to hundreds affected individuals. CNV analyses using chromosomal microarray or aCGH revealed several recurrent CNVs associated with ASD, particularly 16p11.2-12.2 region (4.2%, 6/143 individuals with CNV evaluation, five deletions and one duplication, detailed in Table S1), and yielded diagnostic rates between 10% and 30%. The use of WES in Brazilian ASD cohorts remains relatively recent and limited in scale, with sample sizes of approximately 30 individuals and diagnostic yields of 12% to 23%. Across studies, diagnostic performance varied considerably, reflecting methodological differences, cohort characteristics, and the inclusion of participants with comorbid phenotypes such as ID. Importantly, because no date restriction was applied to the literature search, changes in diagnostic standards and guidelines over time, particularly the transition from DSM-IV to DSM-5, may have influenced the clinical profiles of the cohorts (Matson et al., 2012; Kulage et al., 2020) and, consequently, the molecular diagnostic yield reported across studies. No sample overlap was identified among the reviewed WES studies (Table 1).

**Table 1 -:** Articles included in the review.

PMID	Author	Type	Title	Year	Diagnostic Tools	ASD Sample	Method	Sex	Age (years)	N (included)	Diagnostic yield
38355898	Chaves *et al*.	CNVs	A cohort study of neurodevelopmental disorders and/or congenital anomalies using high resolution chromosomal microarrays in southern Brazil highlighting the significance of ASD	2024	DSM-5	333	Chromosomal microarray	F (71) M (262)	0-34	77	10% (33/333)
31161481	Schuch *et al*.	CNVs	Analysis of a protein network related to copy number variations in autism spectrum disorder	2019	DSM-IV/ASQ/ CARS	40	Array Comparative Genomic Hybridization (aCGH)	F (4) M (36)	-	12	30% (12/40)
31696658	Montenegro *et al*.	CNVs and SNVs	Meta-Analyses support previous and novel autism candidate genes: outcomes of an unexplored brazilian cohort	2020	DSM-5/CARS	30	Whole Exome Sequencing	F (9) M (21)	2-34	30	23% (7/30)
34573415	Neri de Souza Reis *et al*.	SNVs	Environmental influences measured by epigenetic clock and vulnerability components at birth impact clinical ASD heterogeneity	2021	DSM-IV/ ADI-R/ SON-R 2½-7/Vineland II/ CARS	33	Whole Exome Sequencing	NA	NA	33	NA
37280359	Costa *et al*.	SNVs	Three generation families: Analysis of de novo variants in autism	2023	DSM-5	33	Whole Exome Sequencing	9 (F) 24 (M)	1-12	33	12% (4/33)

### WES dataset description

WES was performed on 476 Brazilian ASD affected individuals, including 441 probands (male-to-female ratio ~4:1) and 27 affected relatives (detailed in Table S2, and Supplementary Material). Most probands were isolated cases, with only 15.6% (69/441) reporting familial recurrence. The mean age at evaluation was 6.3±4.6 years. Among evaluated probands, comorbid signs of ID was the most frequent clinical feature (46%; 86/187), followed by lack of speech (45.6%;162/355), hypotonia (19.9%; 63/316), seizures (13.2%; 46/348), and comorbid signs of ADHD (11.9%; 42/352). Comorbidity burden in ASD has been shown to be disproportionately higher among females and closely associated with age at first diagnosis and clinical severity (Rødgaard et al., 2021); however, sex was not broadly associated with differences in most clinical features, except for a higher prevalence of seizures among females (2.6-fold higher; 19/73 females; 27/275 males, p=0.002). Overall, 65.8% of probands had at least one comorbid feature, and 27.7% had two or more. A summary of collected variables is presented in Table 2.

**Table 2 -:** Sample description (N = 441 probands).

Demographic characteristics	
Sex, n (%)	441 evaluated
*M*	348 (78.9%)
*F*	93 (21.1%)
Age at evaluation (years), mean (± SD)	6.3 (± 4.5)
Maternal age at conception, mean (± SD)	30.3 (± 5.3)
Paternal age at conception, mean (± SD)	32.8 (± 5.7)
Consanguinity, n (%)	441 evaluated
*no*	436 (98.9%)
*yes*	5 (1.2%)
Family recurrence, n (%)	387 evaluated
*no*	318 (82.2%)
*yes*	69 (17.8%)
Ancestry components, mean (± SD)	422 evaluated
*European (%)*	73.6 (± 18.7)
*African (%)*	13.9 (± 12.6)
*Native American (%)*	8.4 (± 6.3)
*East Asian (%)*	4.1 (± 15.1)
Clinical characteristics	
ADHD comorbid sign, n (%)	352 evaluated
*no*	310 (88.1%)
*yes*	42 (11.9%)
ID comorbid sign, n (%)	187 evaluated
*no*	101 (54.0%)
*yes*	86 (46.0%)
Speech development, n (%)	390 evaluated
*<3 years*	35 (9.0%)
*non-verbal*	162 (41.5%)
*verbal*	193 (49.5%)
Hypotonia, n (%)	316 evaluated
*no*	253 (80.1%)
*yes*	63 (19.9%)
Seizures, n (%)	348 evaluated
*no*	287 (82.5%)
*yes*	46 (13.2%)
*yes, febrile*	15 (4.3%)
Number of clinical features, n (%)	401 evaluated
*0*	137 (34.2%)
*1*	153 (38.2%)
*2*	83 (20.7%)
*3*	21 (5.2%)
*4*	6 (1.5%)
*5*	1 (0.2%)

Values presented as n (%) or mean ± Standard Deviation (SD). Percentages for categorical variables are calculated based on the number of individuals with available data.

### *De novo* variants analysis

**Figure 2 -:**
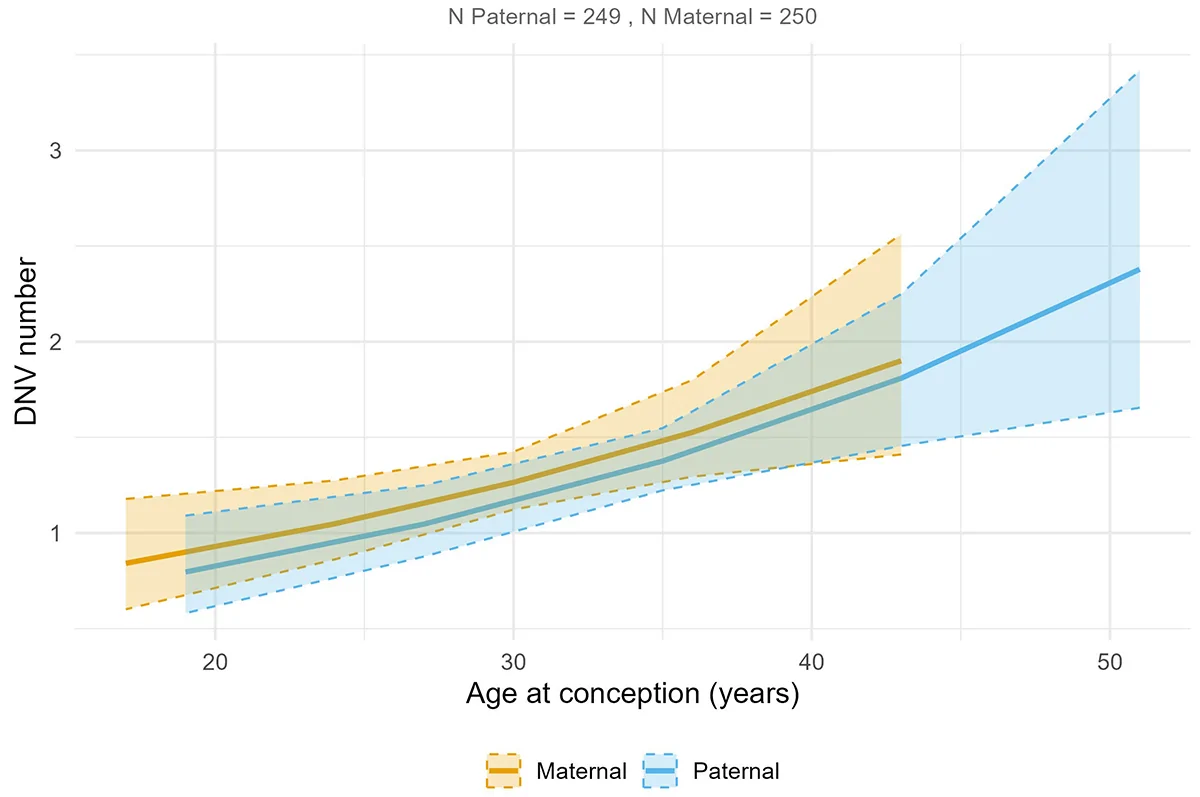
Association between the number of *de novo* variants (DNVs) and parental age at conception. The plot shows the number of DNVs identified per proband (Y-axis) as a function of parental age (X-axis). The analysis revealed a statistically significant positive correlation for both maternal age (yellow; p=0.026) and paternal age (blue; p=0.006), indicating that the number of DNVs increases with advancing parental age.

In 386 parent-offspring trios, 453 DNVs were identified (mean 1.17 per proband; 5.5% false-positive rate; Table S3). DNV numbers were positively associated with paternal (p=0.006) and maternal (p=0.026) age (Figure 2), but not with proband sex or clinical features (Supplementary Material). Among the DNVs, 21.4% (97/453) were missense or LoF variants detected in constrained genes (pLI≥0.9 for LoF and z-score ≥3 for missense variants). Of those, 22 (4.8%; 22/453) were protein-truncating variants (PTVs; nonsense, frameshift, and canonical splice site variants).

### Inherited and *de novo* rare risk variants analysis

*Rare risk variants number and characterization*

A total of 1,836 RRVs were identified in 1,097 of the 1,295 individuals included in the analysis. Among these, 912 RRVs were detected in 375 of the 441 probands, corresponding to a mean of 2.1 RRVs per proband (912/441). Of the 796/912 RRVs with a determined parental origin, the majority were inherited (96.2%, 766/796), with 53.1% maternally inherited (407/766) and 46.9% paternally inherited (359/766). The remaining 30 RRVs were classified as DNVs. Among the 912 RRVs identified in probands, 442 were putative LoF variants (118 frameshift, 98 stop-gain or stop-loss, and 226 splicing variants), and 470 were missense variants.

Association analysis of sex and clinical features with the number of RRVs revealed no statistically significant predictors in the proband sample (Supplementary Material). Also, there was no significant difference in the number of RRVs between probands (912/441; 2.05±1.54) and parents (1,647/815; 2.02 ± 1.54, p = 0.565), nor between affected (998/476; 2.10±1.54) and unaffected (1,412/683; 2.07±1.54) individuals (p=0.725).

*Gene ontology and protein-protein interactions enrichment analysis*

A total of 784 genes were included in the analysis of genes harboring RRVs identified in probands. The resulting PPI network consisted of 758 nodes and 1,296 edges, with a significant enrichment (PPI enrichment p<0.001). Gene Ontology (GO) enrichment analysis revealed several significantly enriched terms (Supplementary Material). Among the top enriched Cellular Component (CC) terms were cell periphery, plasma membrane, endomembrane system, and supramolecular fiber. The most significant Biological Process (BP) terms included signal transduction, cell adhesion, regulation of transport, skeletal system morphogenesis, membrane depolarization, collagen fibril organization, cell-substrate junction assembly, actin filament-based process, and regulation of transporter activity. In the Molecular Function (MF) category, enriched terms included structural molecule activity, calcium ion binding, and GTPase regulator activity. Notably, cell periphery and plasma membrane were the most strongly enriched terms, consistent across both PPI and GO analyses.

*Global ancestry*

**Figure 3 -:**
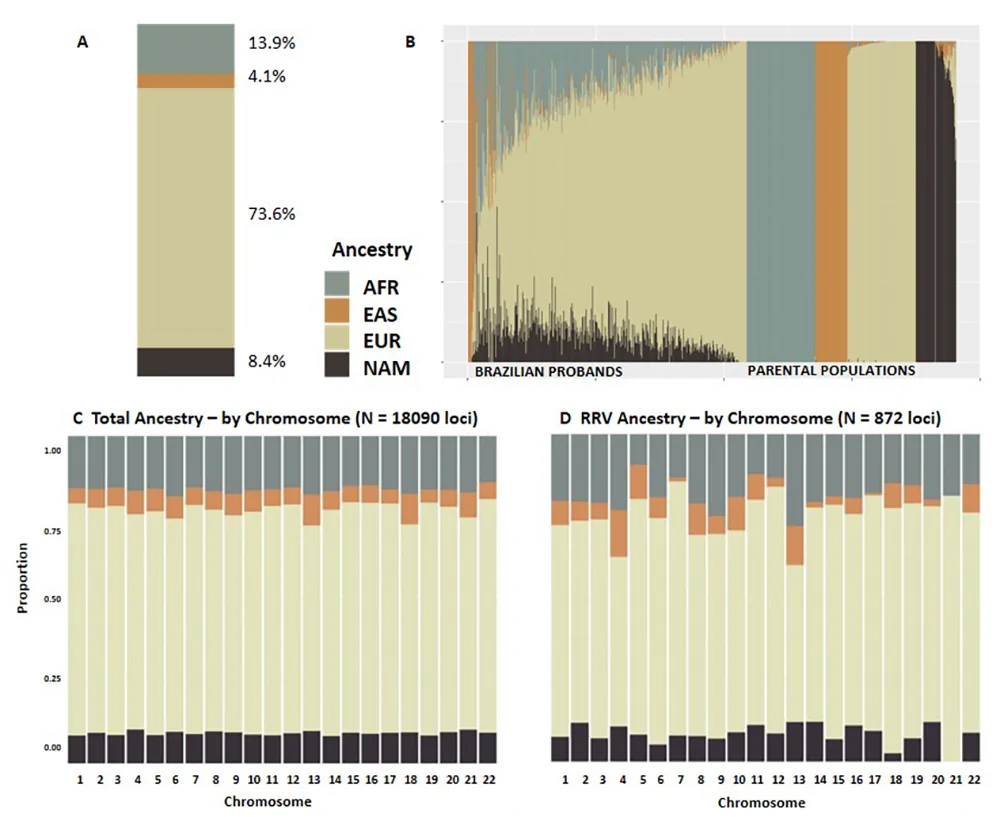
Global and local ancestry analysis of the Brazilian ASD cohort. (a) Bar plot showing the mean proportions of global ancestry for the 422 probands, indicating a predominance of European (EUR) ancestry, followed by African (AFR), Native American (NAM), and East Asian (EAS) components. (b) Individual ancestry composition estimated by ADMIXTURE (K=4). Each vertical bar represents a single proband, with colors corresponding to the proportion of each ancestral component. Parental reference populations used in the supervised analysis are displayed on the right. (c) Proportions of local ancestry across all autosomes. The plot illustrates the distribution of genomic segments assigned to each ancestral population along the chromosomes. (d) Local ancestry proportions of Rare Risk Variants (RRVs) by genomic *loci*, showing the ancestral background of the specific genomic regions where RRVs were identified.

Global ancestry was successfully estimated for 422 of 441 probands (95.7% of the sample; ancestry proportions could not be inferred for 19 individuals due to excessive missing genotypes in the SNP panel used for ADMIXTURE analysis) and showed a predominance of the European component, with 51.2% (226/422) exhibiting ≥75% European ancestry proportion (Figure 3A, 3B). The mean ancestry proportions were estimated as 73.6%±18.7% European, 13.9%±12.6% African, 8.4%±6.3% Native American, and 4.1%±15.1% East Asian. Of note, considering a minimum threshold of 5% to define the presence of an ancestry component, most individuals exhibited admixture from two or more ancestries, representing 84.6% of the probands (25% [N=110/422] with two components; 57% [N=240/422] with three components; and 1,6% [N=7/422] with four components).

*Local ancestry inference*

Given the uniquely admixed background of the sample, local ancestry inference analysis was performed to determine the distribution of RRVs across different ancestry components. Local ancestry could be assigned to 95.6% of the RRVs (872 out of 912, Table S4). For the remaining 40 variants, the number of informative SNPs was insufficient to enable haplotype determination during the phasing step. Consequently, these variants could not be matched between probands and reference datasets and were excluded from both phasing and local ancestry estimation. Because local ancestry inference relies on contiguous genomic ancestry segments, the removal of these variants resulted in gaps between ancestry segments, precluding reliable local ancestry assignment. Among the 872 RRVs with assigned local ancestry, 34.5% (303/872) were absent from major genomic databases, including gnomAD and the 1000G (Table S4). Notably, 13.1% of all RRVs (114/872) were located in genomic regions where both alleles were inferred to be of non-European ancestry based on local ancestry analysis. The majority of the RRVs (52.4%, 457/872) were located in regions where at least one allele was assigned to non-European ancestry. The remaining 47.6% (415/872) were located in genomic regions where both alleles were assigned to European ancestry, which is consistent with the high proportion of European global ancestry observed in the sample.

### Clinically relevant variants

*Pathogenic and likely pathogenic alterations*

Among 1,295 individuals, 51 P/LP variants were identified in NDD-associated AD or XL genes in 58 individuals. Of these, 41 (80.4%) occurred only in probands (37 confirmed as *de novo*), six (11.8%) were inherited from unaffected parents, three (7.9%) were found only in affected relatives, and one (2%) in a non-affected father (Table 3). Of the 51 P/LP variants, 31 were putative LoF variants (12 frameshift insertions or deletions, 17 stop-gain, and two splice-site variants; 27 in probands and four only detected in other individuals, including one non-affected parent), while the remaining 20 were missense variants (all detected in probands) predicted to be damaging by *in silico* analysis (CADD score ≥20).

**Table 3 -:** Pathogenic and likely pathogenic variants identified in all individuals (N = 1,295).

Variant (hg38)	HGVS	Gene	Type	ABraoM	gnomad4.0	1000G	CADD score	SpliceAI	DNV	SFARI	ID	Status	Affected status
chr1:2306188C>T	NM_003036.4:SKI:c.1936C>T	SKI	stopgain	0	0	0	50	PP3	yes	yes	P_115	proband	yes
chr1:6146759GGAGGGAdel	NM_015557:CHD5:c.1490_1496del	CHD5	frameshift	0	0	0	0	NA	no		F_329	father	no
chr1:211018934G>A	NM_172362.3:KCNH1:c.881C>T	KCNH1	missense	0	0	0	27.1	BP7	yes		P_70	proband	yes
chr2:159448260G>A	NM_013450.4:BAZ2B:c.484C>T	BAZ2B	stopgain	0	6.58E-06	0	36	BP7	no	yes	P_17; M_17	proband; mother	yes; no
chr2:165374775G>A	NM_001040142.2:SCN2A:c.4063G>A	SCN2A	missense	0	0	0	29.7	BP7	yes	yes	P_153	proband	yes
chr2:165991772G>A	NM_001165963.4:SCN1A:c.5503C>T	SCN1A	missense	0	0	0	24.7	NA	yes	yes	P_404	proband	yes
chr2:165996121T>A	NM_001165963.4:SCN1A:c.4477-4A>T	SCN1A	splicing	0	0	0	0	PP3	yes	yes	P_71	proband	yes
chr2:199349027G>A	NM_001172509.2:SATB2:c.847C>T	SATB2	stopgain	0	0	0	36	NA	yes	yes	P_24	proband	yes
chr2:224513582G>C	NM_003590.5:CUL3:c.596C>G	CUL3	stopgain	0	0	0	38	BP7	yes	yes	P_332	proband	yes
chr3:11017846C>A	NM_003042.4:SLC6A1:c.242C>A	SLC6A1	missense	0	0	0	26.3	BP7	yes	yes	P_101	proband	yes
chr3:70972667G>A	NM_001349338.3:FOXP1:c.1540C>T	FOXP1	missense	0	0	0	33	BP7	yes	yes	P_238	proband	yes
chr3:71046957G>A	NM_001349338.3:FOXP1:c.649C>T	FOXP1	stopgain	0	0	0	39	BP7	yes	yes	P_333	proband	yes
chr3:114339286G>T	NM_001348800.3:ZBTB20:c.1945C>A	ZBTB20	missense	0	0	0	26.8	NA	no	yes	P_361	proband	yes
chr3:192335434C>T	NM_004113.6:FGF12:c.155G>A	FGF12	missense	0	0	0	27.5	NA	yes		P_163	proband	yes
chr6:33440913C>T	NM_006772.3:SYNGAP1:c.1861C>T	SYNGAP1	stopgain	0	0	0	36	BP7	yes	yes	P_300	proband	yes
chr8:23006057C>T	NM_015178.3:RHOBTB2:c.394C>T	RHOBTB2	stopgain	0	0	0	37	BP7	yes		O1_370	cousin	yes
chr8:41940843T>C	NM_006766.5:KAT6A:c.3038A>G	KAT6A	missense	0	0	0	33	PP3	yes	yes	P_301	proband	yes
chr9:93660384Cdel	NM_005392.4:PHF2:c.1526del	PHF2	frameshift	0	0	0	0	NA	no	yes	P_399	proband	yes
chr9:98306245A>G	NM_005458.8:GABBR2:c.2105T>C	GABBR2	missense	0	0	0	24.7	NA	yes	yes	P_439	proband	yes
chr9:127668159C>T	NM_001032221.6:STXBP1:c.874C>T	STXBP1	missense	0	0	0	29.3	BP7	yes	yes	P_346	proband	yes
chr10:87957915C>T	NM_000314.8:PTEN:c.697C>T	PTEN	stopgain	0	0	0	36	BP7	no	yes	P_193; M_193	proband; mother	yes; no
chr10:87958020G>A	NM_000314.8:PTEN:c.801+1G>A	PTEN	splicing	0	0	0	34	PP3	no	yes	P_189	proband	yes
chr11:686950T>G	NM_021008.4:DEAF1:c.712A>C	DEAF1	missense	0	0	0	25	NA	yes	yes	P_239	proband	yes
chr11:1445436insT	NM_001256627:BRSK2:c.954_955insT	BRSK2	frameshift	0	0	0	0	BP7	yes	yes	P_16	proband	yes
chr11:124924872AGTCdel	NM_152722:HEPACAM:c.280_283del	HEPACAM	frameshift	0	0	0	0	BP7	no	yes	P_149; F_149	proband; father	yes; no
chr12:6593101T>C	NM_001273.5:CHD4:c.2642A>G	CHD4	missense	0	1.97E-05	0	26.8	BP7	yes		P_5	proband	yes
chr12:56184921G>A	NM_001330288.2:SMARCC2:c.415C>T	SMARCC2	stopgain	0	0	0	37	VUS	no	yes	P_71; F_71	proband; father	yes; NA
chr12:112477701T>G	NM_002834.5:PTPN11:c.904T>G	PTPN11	missense	0	0	0	22.1	NA	yes	yes	P_154	proband	yes
chr14:28768031A>G	NM_005249.5:FOXG1:c.752A>G	FOXG1	missense	0	0	0	31	NA	yes	yes	P_374	proband	yes
chr14:105368112G>A	NM_001100913.3:PACS2:c.625G>A	PACS2	missense	0	0	0	28.9	BP7	yes	yes	P_164	proband	yes
chr16:3731217G>A	NM_004380.3:CREBBP:c.5147C>T	CREBBP	missense	0	0	0	31	NA	no	yes	P_167	proband	yes
chr16:30710078C>T	NM_006662.3:SRCAP:c.1084C>T	SRCAP	stopgain	0	0	0	29.1	BP7	no	yes	P_219	proband	yes
chr16:30979979CCCGGCGCCG CCGCCCTCCGCCCCCACCdel	NM_014712:SETD1A:c.4193_4220del	SETD1A	frameshift	0	0	0	0	NA	yes	yes	P_172	proband	yes
chr17:31181482T>C	NM_001042492.3:NF1:c.647T>C	NF1	missense	0	0	0	24.3	NA	yes	yes	P_88	proband	yes
chr17:62481156C>T	NM_006852.6:TLK2:c.31C>T	TLK2	stopgain	0	0	0	36	BP7	yes	yes	P_132	proband	yes
chr18:33744172Gdel	NM_030632.3:ASXL3:c.4326del	ASXL3	frameshift	0	0	0	0	NA	yes	yes	P_21	proband	yes
chr18:33746172insAAAGA	NM_030632.3:ASXL3:c..6326_6330dup	ASXL3	frameshift	0	0	0	0	NA	yes	yes	P_120	proband	yes
chr20:492282T>C	NM_177559.3:CSNK2A1:c.593A>G	CSNK2A1	missense	0	0	0	24.9	NA	yes	yes	P_134	proband	yes
chr20:49374644G>A	NM_004975.4:KCNB1:c.916C>T	KCNB1	missense	0	0	0	29	NA	yes	yes	P_348	proband	yes
chr20:63495062C>T	NM_001958.5:EEF1A2:c.364G>A	EEF1A2	missense	0	0	0	25.7	BP7	yes	yes	P_177	proband	yes
chr21:37478281insT	NM_001347721.2:DYRK1A:c.281_282insT	DYRK1A	frameshift	0	0	0	0	BP7	no	yes	O_417	cousin	yes
chr22:20990442C>A	NM_006767.4:LZTR1:c.708C>A	LZTR1	stopgain	0	0	0	35	NA	no	yes	P_248; F_248	proband; father	yes; no
chr22:50721585TG CCCAGCCCCCGdel	NM_001372044.2:c.3977_3989del	SHANK3	frameshift	0	0	0	0	NA	no	yes	P_365	proband	yes
chr22:50721898TGdel	NM_001372044.2:c.4290_4291del	SHANK3	frameshift	0	0	0	0	NA	yes	yes	P_264	proband	yes
chrX:15852371G>A	NM_001272071.2:AP1S2:c.154C>T	AP1S2	stopgain	0	0	0	35	PP3	no	yes	O1_398; M_398; O2_398	cousin; mother; uncle	yes; no; yes
chrX:41342831G>T	NM_001356.5:DDX3X:c.538G>T	DDX3X	stopgain	0	0	0	37	BP7	yes	yes	P_39	proband	yes
chrX:53250732ACAGAGC CGCGATCTGAGCGGTCdel	NM_001111125.3:c.1822_1844del	IQSEC2	frameshift	0	0	0	0	NA	yes	yes	P_258	proband	yes
chrX:154030621GGGGGCTG GTGGGGTCCTCGGAGCTC TCGGGCTCAGGTGGAGGTdel	NM_001110792.2:c.1200_1243del ACCTCCACCTGAGCCCGAGAG CTCCGAGGACCCCACCAGCCCCC	MECP2	stopgain	0	0	0	0	NA	no	yes	P_60	proband	yes
chrX:154030690insG	NM_001110792.2:MECP2:c.1173dup	MECP2	frameshift	0	0	0	0	NA	yes	yes	P_32	proband	yes
chrX:154030948G>A	NM_001110792.2:MECP2:c.916C>T	MECP2	stopgain	0	0	0	37	VUS	yes	yes	P_65	proband	yes
chrX:154031065G>A	NM_001110792.2.MECP2:c.916C>T	MECP2	stopgain	0	0	0	36	NA	yes	yes	P_128	proband	yes

Considering different methodologies, CNV analysis was performed in 432 individuals (including 402 probands). ASD-associated CNVs were identified in 3% (12/401) of the probands and one affected sibling. Recurrent CNVs included duplications at 16p11.2-16p12 (3 probands), 15q11-q13.1 (3 probands), and a balanced translocation t(5;7)(p15.2;p15.2) shared by two affected siblings. Additional single occurrences involved duplications at 22q11.21, Y chromosome, 1q21.1, 16p11.2, and 18q21.2, and deletions at Xp22.33p22.31 and 22q13, all of them in affected individuals. Two male probands presented dual molecular diagnosis: one with P/LP variants in *SCN1A* and *SMARCC2*, and another with a 16p11.2 duplication plus a P/LP variant in *LZTR1*.

Taken together, conclusive molecular diagnoses were obtained in 13.1% of probands (58/441), including 46 with pathogenic or likely pathogenic (P/LP) sequence variants, 12 with CNVs, and one with both. A similar diagnostic yield (13.2%) was estimated among affected individuals (63/476). The WES-only diagnostic yield was 10.4% (46/441). Most variants were identified in well-established ASD genes, while several involved genes more recently implicated in the disorder (*BAZ2B*, *BRSK2*, and *PHF2*).

Additionally, variants in genes not previously associated with a defined clinical phenotype were systematically assessed. Among these, a variant in *NPAS3* (Table S3), a gene recently added to the SFARI Gene database (Score 3), which encodes a transcription factor with basic helix-loop-helix (bHLH) motifs involved in key neurodevelopmental processes, including astrogenesis during cortical development (Li et al., 2022), maintenance of radial glial cell stemness, and neuronal migration (Liu et al., 2022). In our sample, a *de novo* stop-gain variant was identified in a female with ASD. *NPAS3* has previously been implicated in schizophrenia (Kamnasaran et al., 2003; Pickard *et al.*, 2005) and developmental delay (Rossi et al., 2021), and recent evidence from disruptive non-coding alterations further supports its role as a candidate ASD susceptibility gene (Chen et al., 2025). However, as no well-defined clinical phenotype has been established for NPAS3 haploinsufficiency, the variant identified in this study was classified as a variant of uncertain significance. Although not classified as P/LP, we highlight the gene’s emerging evidence for involvement in neurodevelopment and ASD risk.

*Genotype-phenotype correlations*

**Figure 4 -:**
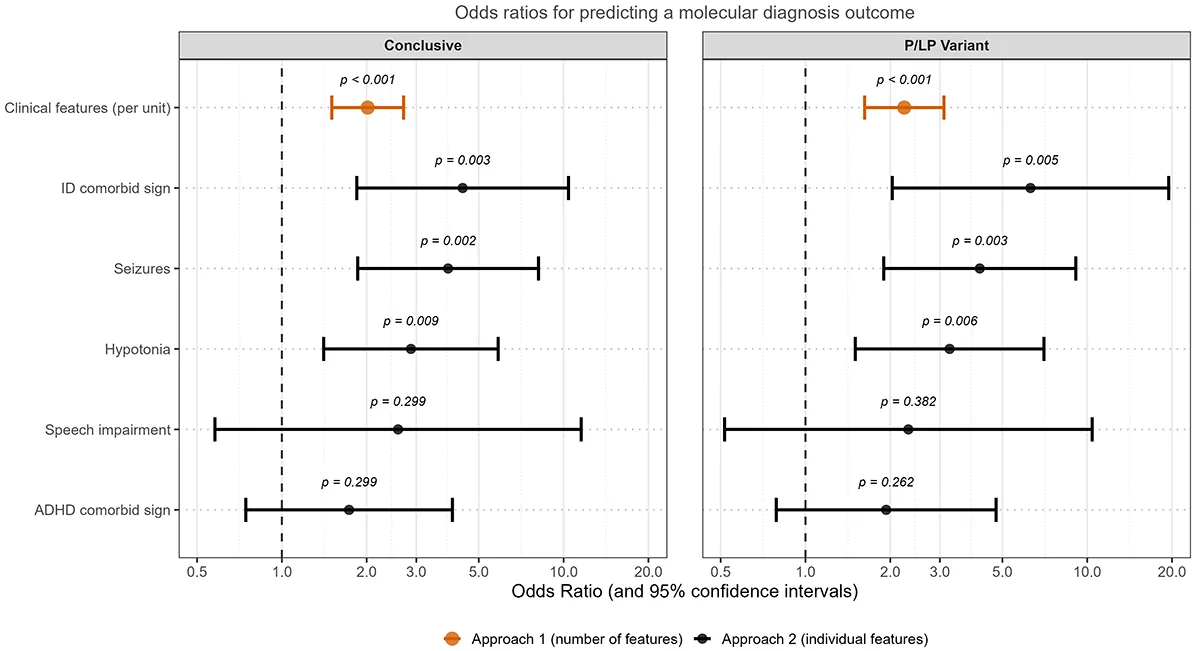
Forest plots of the association between clinical features and diagnostic yield. The plot displays the Odds Ratios (OR) and 95% confidence intervals from binary logistic regression models. Two outcomes were tested: obtaining a conclusive molecular diagnosis (from either WES or CNV analysis; left panel) and identifying a pathogenic/likely pathogenic (P/LP) sequence variant by WES (right panel). The predictor variables include the cumulative number of clinical features (hypotonia, seizures, lack of speech, comorbid signs of ID and ADHD) and each feature individually. An OR greater than 1 indicates that the feature is associated with a higher likelihood of a positive diagnostic outcome.

Two logistic regression approaches were applied to evaluate predictor variables of diagnostic yield. In the first approach, the cumulative number of clinical features (ranging from 0 to 5) was considered. Each additional feature increased the odds of obtaining a conclusive result or identifying a P/LP variant by 2-fold (Figure 4; OR=2.02, 95% CI [1.50-2.70], p<0.001 for conclusive and OR=2.24, 95% CI [1.62-3.10], p<0.001 for a P/LP sequence variant). Proband’s sex was not a significant predictor in either model (p=0.715 and p=0.860, respectively). In the second approach, individual clinical features were analyzed separately. Comorbid signs of ID showed the strongest effect, with affected probands presenting 4-fold higher odds of a conclusive result (OR=4.38, 95% CI [1.84-10.40], p=0.003) and 6-fold higher odds of a P/LP variant (OR=6.29, 95% CI [2.03-19.47], p=0.005). Hypotonia was also significantly associated with increased diagnostic yield (OR=2.87 for conclusive results, p=0.009; OR=3.25 for P/LP variants, p=0.006), as were seizures (OR=3.89 and OR=4.15, both p=0.006). In contrast, lack of speech and comorbid signs of ADHD were not significantly associated after multiple testing correction (p>0.05).

### Ethical issues

Informed consent was obtained from all subjects, and the study was approved by the Ethics Committee of the Instituto de Biociências, Universidade de São Paulo (CAEE 56459522.0.0000.5464).

## Discussion

Brazil has the seventh largest population in the world (United Nations, 2024), yet remains underrepresented in genomic databases (Nunes et al., 2025), with large national initiatives still underway. This gap extends to ASD genomics, as indicated by the small number of Brazilian studies available. In the past decades, ASD genetics research in Brazil focused on candidate gene association studies and case reports (Quaio et al., 2025). By aggregating available WES data, this study established the first Brazilian ASD genomic dataset and assessed the diagnostic yield and contribution of rare variants to ASD. The sample followed expected patterns, including a ~4:1 male-to-female ratio (Brugha et al., 2016) and frequent comorbidity with ID and ADHD (Khachadourian et al., 2023). Mean DNV rate was 1.17 per proband, encompassing all sequence variant types. Although methodological differences explain some variation, prior studies reported comparable rates, ranging from 1.25 to 1.49 (Iossifov et al., 2014; Satterstrom et al., 2020; Zhou et al., 2022); and similar estimates were previously found in Brazilian samples (Costa et al., 2023, 2025). Paternal age showed the strongest association with DNV count, consistent with earlier reports (Kong et al., 2012; Iossifov *et al.*, 2014; Deciphering Developmental Disorders Study, 2017; Costa *et al.*, 2023).

Family-specific rare variants, as previously shown by Wilfert et al. (2021), can also influence ASD risk. Although the number of RRVs did not differ between ASD probands and their parents, the enriched Gene Ontology (GO) terms provide evidence that the genes affected by such variants are involved in three important pathways altered in ASD neurobiology: synaptic signaling, cytoskeletal dynamics, and disrupted neurodevelopmental architecture. These processes, associated with gene-environmental responses, represent highly relevant molecular signatures in the context of ASD research (Gandal et al., 2022).

The ancestry profile was consistent with previous cohorts from São Paulo state, showing a predominantly European background (Naslavsky et al., 2022; Nunes et al., 2025). Nevertheless, more than half of the RRV loci (52.4%, 457/872) harbored at least one non-European allele, including rare risk variants located on African and Native American ancestry haplotypes. These findings highlights an important challenge for ASD genomics in admixed populations, as variants that are rare or classified as pathogenic in predominantly European-based reference databases may occur at higher frequencies in underrepresented populations, increasing the risk of pathogenicity misclassification (Xiang et al., 2020; Naslavsky *et al.*, 2022; He et al., 2025). Indeed, studies incorporating larger and more ancestrally diverse datasets have shown substantial reclassification of pathogenic or likely pathogenic variants, particularly those whose initial assertions were derived from limited ancestral representation (Xiang *et al.*, 2020; Naslavsky *et al.*, 2022). Although rare variants with large effect sizes are expected to be at least partially shared across ancestries (Avila et al., 2025), differences in allele frequencies and population-specific genomic architectures may influence their interpretation, and the contribution of ancestry-specific rare variants to ASD risk remains incompletely understood.

Among P/LP variants, all genes with an OMIM characterized phenotype were also linked to ID or developmental delay, which is expected given the sample’s clinical profile. Three of these genes (*BAZ2B*, *BRSK2*, *PHF2*) correspond to recently described syndromes or poorly characterized SFARI genes (Hiatt et al., 2019; Scott et al., 2020; Sertié et al., 2025). Thus, these findings expand the phenotypic spectrum and further confirm their relevance in ASD. While most P/LP variants arise *de novo*, six inherited variants were observed; the lack of clinical symptoms in carrier parents may reflect incomplete penetrance or mild, subclinical phenotypes, already reported for the three of five genes (*HEPACAM*, *PTEN* and *SMARCC2*; López-Hernández et al., 2011; Leslie and Longy, 2016; Machol et al., 2019) and suggested for *BAZ2B* (Scott *et al.*, 2020).

We also highlight *NPAS3* (OMIM #609430), a gene recently added to the SFARI Gene database (Score 3). Given the gene’s high mutational constraint (pLI=1; LOEUF=0.29), absence of loss of function variants in population databases, and its central role in neurodevelopment, supported by behavioral abnormalities observed in Npas3 mutant mice (Li et al., 2022), we suggest that *NPAS3* disruption may plausibly contribute to ASD risk.

The overall diagnostic yield (13.1%), considering P/LP sequence variants and CNVs, is consistent with that reported in the Brazilian literature review, as well as in other studies (Fu et al., 2022). Notably, 16p11.2-16p12 duplications were the most frequent CNVs observed in our sample (0.75%, 3/402 probands with CNV analysis). In contrast, the Brazilian literature review showed a predominance of deletions within the 16p11.2 region (4.2%, 6/143 individuals reported in the studies), a known ASD risk factor. This discrepancy likely reflects differences in cohort composition and ascertainment strategies across studies, rather than true population-level prevalence. Importantly, these proportions represent frequencies among clinically ascertained individuals who underwent CNV testing. Consistent with this interpretation, population-based estimates reported by Auwerx et al. (2024) indicate low prevalence for both duplications (0.032%) and deletions (0.026%), based on frequency calculations derived from Collins et al. (2022).

Two dual molecular diagnosis cases were identified, an expected frequency (Spedicati et al., 2022). One proband carried a *de novo* pathogenic variant in *SCN1A* and a maternally inherited variant in *SMARCC2*, consistent with previous reports of asymptomatic maternal transmission (Machol et al., 2019). The second proband presented a pathogenic *LZTR1* variant combined with a 16p11.2 duplication. LZTR1 might modulate the RAS-MAPK signaling cascade through CUL3 signaling (Steklov et al., 2018). Interestingly, the 16p11.2 region harbors *MAPK3* and *KCTD13* genes, which regulate the CUL3/GTPase network (Sundberg et al., 2021). Importantly, 16p11.2 deletion is known to disrupt the Ras Homolog Family Member A (RHOA) pathway by reducing *KCTD13* expression, leading to increased RHOA levels, which drive neuronal hyperactivity and increased excitability in dopaminergic neurons (Sundberg *et al.*, 2021). The presence of both the *LZTR1* variant and the 16p11.2 duplication may suggest a complex interaction within the shared CUL3 regulatory axis, potentially modifying the neurodevelopmental trajectory. Although further evidence and functional validation is needed, this may represent a multi-hit interaction acting in an oligogenic manner, as previously suggested (Pizzo et al., 2021).

The genotype-phenotype model indicated that the number of clinical features best predicted a conclusive genetic diagnosis (OR=2, p>0.001), thus signs such as hypotonia, seizures, and comorbid ID may help prioritize individuals for testing in resource-limited settings common in Brazil.

In summary, the review was limited by the scope of the available literature, which frequently presented limited clinical characterization, partial reporting of ASD evaluation methods, and the absence of variant classification. The WES analysis was constrained by the restricted availability of Brazilian genomic datasets, variable phenotyping, unequal access to genetic testing, and heterogeneity in sequencing. Despite these limitations, the findings reinforce the contribution of rare variants and ancestry to ASD in Brazil. Most of the existing genomic data originate from consortia funded by non-Brazilian institutions, which have been essential in providing sequencing access but also reinforces the pressing need for locally assembled cohorts. These results may serve as a foundation for the development of larger and clinically detailed national studies. Although initiatives such as GALA (Avila et al., 2025) and Genomas Brasil (Brasil, 2020) represent important advances, broader and more coordinated national efforts remain necessary to expand genomic research in ASD. Finally, the genotype-phenotype model suggests that clinical burden, particularly hypotonia, seizures, and comorbid intellectual disability, can help prioritize genetic testing in resource-limited settings.

## Supplementary material

The following online material is available for this article:

Supplementary Material - This document provides detailed statistical tests, methodologies, and additional results for the study on the genetic landscape of autism spectrum disorder (ASD) in Brazil.

Table S1 - Copy Number Variation identified in the review, including Variant of Uncertain Significance (VUS), Likely Pathogenic, and Pathogenic alterations.

Table S2 - Sample sheet describing all individuals included in the analysis (N=1,295).

Table S3 - *De novo* variants identified in the probands (N=386 trios).

Table S4 - Rare risk variants identified in all individuals included in the analysis (N = 1,295).

Figure S1 - Study flowchart describing the main steps of the study.

Figure S2 - Diagram of the different family structure types included in the study, such as quartets, trios, duos, singleton, and multiplex families.

Figure S3 - Scatter plot illustrating the sequencing quality (mean coverage) for all samples included in the study, with samples colored by the sequencing kit used.

Figure S4 - Regression analysis results for the number of *de novo* variants (DNVs) against various factors, including parental age, sex, and clinical features.

Figure S5 - Radar chart showing the difference in the distribution of clinical and ancestry features between individuals who have a pathogenic/likely pathogenic (P/LP) variant (grey line) and those who do not (blue line).

Figure S6 - Regression analysis results for the number of rare risk variants (RRVs) against different variables like parental age and clinical signs.

Figure S7 - Bar chart illustrating the transmission bias analysis of Rare Risk Variants (RRVs) from maternal (yellow) and paternal (blue) sources in 386 complete trios.

Figure S8 - Results of the STRING protein-protein interaction (PPI) network analysis for the Rare Risk Variants (RRVs) identified in the probands. Top: network characteristics. Bottom: GO enrichment.

Data Availability

 Data available upon reasonable request.
