# Supplementary material for: Genomic landscape of autism spectrum disorder in Brazil
Source: Genet Mol Biol. 2026 Sep 7;49(Suppl 1):e20250247. doi: 10.1590/1678-4685-GMB-2025-0247 (PMC13561403; doi:10.1590/1678-4685-GMB-2025-0247)
Supplement: Supplementary file 1 [file 1415-4757-GMB-49-s1-e20250247-s1.pdf]

Genetics and Molecular Biology
https://doi.org/10.1590/1678-4685-GMB-2025-0247

Supplementary Material to "Genomic landscape of autism spectrum disorder in Brazil"

Dataset

A total of 441 Brazilian probands were included in the WES analysis, with a ~4:1 male-to-female ratio (348:93). For 27 of these families, WES data were also available from additional affected relatives (two fathers, one mother, 21 siblings, three half-siblings, seven cousins, and one uncle), bringing the total to 476 affected individuals analyzed. Notably, six families had more than two affected members (Supplementary Table 1, Supplementary Figure 2).

ID comorbid sign was the most frequent feature, observed in 46% of the evaluated probands (86/187), followed by lack of speech in 45.6% (162/355), hypotonia in 19.9% (63/316), seizures in 13.2% (46/348), and ADHD comorbid sign in 11.9% (42/352). Seizures is the only clinical feature that differs across sexes (2.6-fold higher in females, 19/73 females versus 27/275 males, Fisher's exact test p=0,002), while no sex differences were observed for other clinical features (lack of speech: 35/76 versus 127/279, p=1; hypotonia: 14/60 versus 49/256, p=0.47; ADHD comorbid sign: 11/75 versus 31/277, p=0.42; ID comorbid sign: 21/38 versus 65/149, p=0.21, for females and males, respectively). Considering the clinical features assessed in 401/441 probands, 65.8% (264/401) of the probands presented at least one feature, and 27.7% (111/401) presented two or more.

Whole exome data were integrated from different facilities, but all samples were processed on the Illumina platform. Sequencing quality was assessed for all samples, with a minimum mean sequencing coverage of 20x defined as inclusion criteria (Supplementary Figure 3).

Statistical analysis

De Novo Variants

Analysis of 386 parent-offspring trios identified 453 *de novo* variants (DNVs). Regression analyses were performed to investigate the association of variables with DNV number, controlling for the technical effects of the sequencing kit (Supplementary Figure 4). The results showed a significant positive association between the DNV count and advanced paternal age (adjusted p = 0.06), as well as with maternal age (adjusted p=0.026). In contrast, no significant associations were found for the proband's sex or clinical features, as shown below.

Variable	N	p_value	p_value_adjustedBH
age_conception_father	249	0.000713	0.005703
age_conception_mother	250	0.006462	0.025849
Sex	386	0.483827	0.552945
ADHD_comorbid_sign	310	0.152803	0.244485
ID_comorbid_sign	161	0.036609	0.097624
Hypotonia	277	0.895237	0.895237
Seizures	310	0.250981	0.334641
Speech	338	0.152067	0.244485

Genotype-phenotype correlation analysis

Logistic regression analyses revealed that the number of clinical features was the strongest predictor of both a conclusive WES result and the presence of a pathogenic or likely pathogenic (P/LP) variant. For each additional feature present (approach 1, ranging from 0 to 5), the odds of obtaining a conclusive molecular result increased by 2.02 times (OR=2.02, 95% CI [1.50-2.70], $p=2.82\times10^{-6}$), and the odds of identifying a P/LP variant increased by 2.24 times (OR=2.24, 95% CI [1.62-3.10], $p=1.04\times10^{-6}$). Proband sex was not a significant predictor in either model (p=0.09 and p=0.27, respectively). When individual clinical features were analyzed separately (approach 2), ID comorbid sign, hypotonia, and seizures were significantly associated with higher diagnostic yield. ID comorbid sign showed the strongest effect, with affected probands presenting 4.8-fold higher odds of a conclusive result (OR=4.81, 95% CI [2.05-11.32], p=0.0003) and 6.5-fold higher odds of a P/LP variant (OR=6.51, 95% CI [2.12-20.06], p=0.001). Hypotonia was also significantly associated with increased diagnostic yield (OR=2.91, 95% CI [1.43-5.91], p=0.003 for conclusive results; OR=3.26, 95% CI [1.52-6.99], p=0.002 for P/LP variants), as were seizures (OR=3.89, 95% CI [2.22-9.21], $p < 0.001$ for conclusive results; OR=4.16, 95% CI [2.07-9.48], $p < 0.001$ for P/LP variants). In contrast, speech impairment and ADHD comorbid sign were not significantly associated with diagnostic yield after multiple testing correction (p>0.3), and proband sex showed no significant effect across models (p>0.4), as shown below. A radar chart showing the difference in the distribution of clinical and ancestry features between individuals who have a pathogenic/likely pathogenic (P/LP) variant and those who do not is presented in Supplementary Figure 5.

Approach	Model	N	Term	Odds Ratio	Lower 95% CI	Upper 95% CI	P_value	P_value (Adj)
1	Concl.	397	(Intercept)	0.065	0.030	0.142	9.08E-12	NA
1	Concl.	397	clinical_features_number	2.016	1.503	2.704	2.82E-06	2.82E-06
1	Concl.	397	sexM	0.876	0.430	1.786	0.715286024	NA
1	P/LP	397	(Intercept)	0.039	0.016	0.096	2.10E-12	NA
1	P/LP	397	clinical_features_number	2.243	1.622	3.101	1.04E-06	1.04E-06
1	P/LP	397	sexM	0.930	0.418	2.072	0.859718349	NA
2	Concl.	352	(Intercept)	0.140	0.070	0.279	2.18E-08	NA
2	Concl.	352	ADHD_comorbid_signyes	1.732	0.745	4.030	0.202157819	0.299234429
2	Concl.	352	sexM	0.961	0.451	2.049	0.917459998	NA
2	Concl.	187	(Intercept)	0.119	0.043	0.327	3.64E-05	NA
2	Concl.	187	ID_comorbid_signyes	4.381	1.844	10.407	0.00081778	0.00286223
2	Concl.	187	sexM	0.669	0.271	1.655	0.384742224	NA
2	Concl.	316	(Intercept)	0.095	0.041	0.222	5.25E-08	NA
2	Concl.	316	Hypotoniayes	2.871	1.407	5.858	0.003747506	0.00874418
2	Concl.	316	sexM	1.187	0.491	2.869	0.703608533	NA

2	Concl.	348	(Intercept)	0.122	0.059	0.250	9.96E-09	NA
2	Concl.	348	Seizuresyes	3.890	1.858	8.144	0.000313893	0.002197253
2	Concl.	348	seizuresyes, febrile	1.224	0.264	5.673	0.79638892	0.79638892
2	Concl.	348	sexM	1.039	0.489	2.205	0.9209096	NA
2	Concl.	390	(Intercept)	0.073	0.016	0.336	0.000772748	NA
2	Concl.	390	speechnon-verbal	2.585	0.579	11.547	0.213738878	0.299234429
2	Concl.	390	Speechverbal	2.338	0.527	10.376	0.264153756	0.308179382
2	Concl.	390	sexM	0.789	0.390	1.598	0.510589845	NA
2	P/LP	352	(Intercept)	0.106	0.049	0.227	7.58E-09	NA
2	P/LP	352	ADHD_comorbid_signyes	1.934	0.788	4.750	0.149950219	0.262412883
2	P/LP	352	sexM	0.968	0.420	2.230	0.938601261	NA
2	P/LP	187	(Intercept)	0.052	0.014	0.191	8.53E-06	NA
2	P/LP	187	ID_comorbid_signyes	6.291	2.033	19.467	0.001418228	0.0049638
2	P/LP	187	sexM	0.755	0.263	2.167	0.601317471	NA

2	P/LP	316	(Intercept)	0.062	0.023	0.165	3.14E-08	NA
2	P/LP	316	Hypotoniayes	3.249	1.503	7.023	0.002740451	0.006394386
2	P/LP	316	sexM	1.392	0.504	3.840	0.523230348	NA
2	P/LP	348	(Intercept)	0.093	0.042	0.204	3.21E-09	NA
2	P/LP	348	Seizuresyes	4.156	1.896	9.111	0.000374857	0.002624
2	P/LP	348	seizuresyes, febrile	1.610	0.344	7.546	0.545593607	0.606974878
2	P/LP	348	sexM	1.029	0.456	2.324	0.945319589	NA
2	P/LP	390	(Intercept)	0.068	0.014	0.323	0.000711301	NA
2	P/LP	390	speechnon-verbal	2.319	0.516	10.416	0.272513767	0.381519274
2	P/LP	390	Speechverbal	1.489	0.327	6.783	0.606974878	0.606974878
2	P/LP	390	sexM	0.862	0.390	1.905	0.713242086	NA

Rare Risk Variants

A total of 1,836 rare risk variants (RRVs) were identified in 1,097 of the 1,295 individuals included in the analysis. Among these, 912 RRVs were detected in 375 of the 441 probands, corresponding to a mean of 2.1 RRVs per proband (912/441). Of the 796/912 RRVs with a determined parental origin, the majority were inherited (96.2%, 766/796), with 53.1% maternally inherited (407/766) and 46.9% paternally inherited (359/766). The remaining 30 RRVs were classified as DNVs. Among the 912 RRVs identified in probands, 442 were putative loss-of-function variants, including 118 frameshift, 98 stop-gain or stop-loss, and 226 splicing variants. The remaining 470 were missense variants.

The analysis of variables associated with the number of RRVs revealed no statistically significant predictors in the proband sample (Supplementary Figure 6).

Variable	N	p_value	p_value_adjusted BH
age_conception_father	271	0.178134	0.475025
age_conception_mother	284	0.892542	0.897436
Sex	441	0.10156	0.475025
ADHD_comorbid_sign	352	0.897436	0.897436
ID_comorbid_sign	187	0.318586	0.509738
Hypotonia	316	0.249634	0.499268
Seizures	348	0.138489	0.475025
Speech	390	0.777071	0.897436

Direct group comparisons (probands versus parents) using the Wilcoxon-Mann-Whitney test did not show significant differences in RRV number. There was no significant difference in the number of RRVs between probands (912/441; 2.05±1.54) and parents (1647/815; 2.02±1.54, p = 0.565), nor between affected (998/476; 2.10±1.54) and unaffected (1412/683; 2.07±1.54) individuals (p = 0.725). Analysis of sex showed no significant difference in RRV number between males (1636/771; 2.12±1.54) and females (1022/524; 1.95±1.54), including probands and parents (p = 0.079). No sex-based difference was also observed when restricting the analysis to probands only (males 741/348; 2.13±1.54 *versus* females 171/93; 1.84±1.54, p = 0.089).

Comparison	Group	N	Mean	p_value
Probands vs. Parents	Parent	815	2.02	0.565086
Probands vs. Parents	Proband	441	2.07	0.565086
Males vs. Females (Full Cohort)	F	524	1.95	0.078804
Males vs. Females (Full Cohort)	M	771	2.12	0.078804
Affected vs. Unaffected	No	683	2.07	0.725248
Affected vs. Unaffected	Yes	476	2.1	0.725248
Males vs. Females (Probands Only)	F	93	1.84	0.08892
Males vs. Females (Probands Only)	M	348	2.13	0.08892

Based on variant transmission analysis with 386 trios, no RRV transmission bias was detected (Supplementary Figure 7).

Local Ancestry Inference

For local ancestry inference, we used samples from the Human Genome Diversity Project (HGDP; ftp://ngs.sanger.ac.uk/production/hgdp/) to represent four major continental groups: African, European, Native American, and East Asian populations, which served as reference panels.

Component	Population	N
AFR	BantuKenya	3
AFR	BantuSouthAfrica	5
AFR	Biaka	9
AFR	Mandenka	9
AFR	Mbuti	7
AFR	Yoruba	7
NAM	Colombian	7
NAM	Karitiana	8
NAM	Maya	14
NAM	Pima	9
NAM	Surui	2
EAS	Cambodian	2
EAS	Dai	2
EAS	Daur	2
EAS	Han	9
EAS	Hezhen	1
EAS	Japanese	4
EAS	Lahu	1
EAS	Miao	3
EAS	Naxi	1

EAS	NorthernHan	3
EAS	Oroqen	1
EAS	She	4
EAS	Tu	2
EAS	Tujia	2
EAS	Xibo	1
EAS	Yi	2
EUR	Adygei	3
EUR	Basque	7
EUR	BergamoItalian	3
EUR	French	8
EUR	Orcadian	5
EUR	Russian	8
EUR	Sardinian	2
EUR	Tuscan	4

Each subpopulation (populations within each continental group) was filtered for Hardy-Weinberg equilibrium (HWE) with a threshold of $p < 1e-8$ using PLINK v1.9 (Chang *et al.*, 2015). We excluded InDels and kept only biallelic markers with bcftools (Danecek *et al.*, 2021). Individuals with relatedness degree (measured by the kinship coefficient) greater than 0.0884 were removed (up to the 2nd-degree relatedness), using KING version 2.3.2 (Manichaikul *et al.*, 2010). Additional quality control steps included filtering out markers and individuals with excessive missingness using the --geno and --mind options in PLINK v1.9. Admixed individuals were identified through an unsupervised ADMIXTURE v1.3 analysis (Alexander *et al.*, 2009), applying a 95% ancestry threshold, and subsequently excluded (n = 32). To balance ancestral representation across reference panels, we randomly downsampled larger subpopulations, retaining 40 individuals per continental group.

For the WES-ASD dataset, we applied analogous quality control procedures, including filtering for HWE, biallelic markers, and missingness, with half-calls treated as missing genotypes in PLINK v1.9. After filtering, 990384 variants remained. Phasing was performed with SHAPEIT5 (phase_common pipeline) (Hofmeister *et al.*, 2023), incorporating allele count information in the VCF and using a recombination rate of 1 cM/Mb. Both related and unrelated individuals were phased jointly.

To estimate local ancestry proportions, we used G-Nomix (Hilmarsson *et al.*, 2021), which requires phased VCFs for both admixed and reference samples. To ensure compatibility, we intersected the queried and reference VCFs to guarantee 100% positional overlap. Model training was performed de novo using genetic map files from UCSC. In addition, we used the phasing error correction provided by Gnofix, implemented in the same

software, which leverages ancestry information to correct phasing errors.

The overall ancestry inference from both analyses (global and local) was consistent, with no significant differences observed between ADMIXTURE and G-Nomix when ancestry was averaged across all positions.

Gene ontology and protein-protein interactions enrichment analysis

Results of the STRING protein-protein interaction (PPI) network analysis for the RRVs identified in the probands are presented in Supplementary Figure 8, and the enriched terms results from Gene Ontology analysis are presented below.

References

Alexander DH, Novembre J and Lange K (2009). Fast model-based estimation of ancestry in unrelated individuals. Genome Res 19:1655–1664.

Chang CC, Chow CC, Tellier LC, Vattikuti S, Purcell SM and Lee JJ (2015) Second-generation PLINK: Rising to the challenge of larger and richer datasets. Gigascience 4:7.

Danecek P, Bonfield JK, Liddle J, Marshall J, Ohan V, Pollard MO, Whitwham A, Keane T, McCarthy SA, Davies RM and Li H (2021) Twelve years of SAMtools and BCFtools. Gigascience 10:giab008.

Hilmarsson H, Gudbjartsson DF and Halldorsson BV (2021) High resolution ancestry deconvolution for next generation genomic data. bioRxiv 2021.09.13.460144. doi: 10.1101/2021.09.19.460980

Hofmeister RJ, Ribeiro DM, Rubinacci S and Delaneau O (2023). Accurate rare variant phasing of whole-genome and whole-exome sequencing data in the UK Biobank. Nat Genet 55:1243–1249.

Manichaikul A, Mychaleckyj JC, Rich SS, Daly K, Sale M and Chen WM (2010) Robust relationship inference in genome-wide association studies. Bioinformatics 26:2867–2873.

source	term	term_id	adjusted p_value	term size	intersection size	intersections
GO:MF	structural molecule activity	GO:0005198	0.021907	222	58	IFFO2,MACF1,NDC1,COL11A1,LMNA,LAMC1,ACTN2,SPTBN1,MAP2,ARPC2,MAP4,ERC2,ANK2,FBN2,SPARC,RPS10,DST,COL12A1,EPB41L2,THBS2,HIP1,PCLO,COL1A2,ACTL6B,NUP205,ARC,CLTA,COL27A1,SPTAN1,NUP188,COL5A1,TUBB4B,MRPL41,ANK3,EIF3A,AHNAK,NUMA1,KRT18,PPFIA2,SPTB,ACTN1,THBS1,TLN2,ACAN,MYH11,RPL26,RPL23A,RPL27,COL1A1,MRPL27,CLTC,NUP85,RPS9,JAG1,RPL3,SHANK3,DMD,COL4A6
GO:MF	calcium ion binding	GO:0005509	0.021907	175	49	MACF1,LRP8,CELSR2,MAN1A2,NOTCH2,ACTN2,LRP2,SNED1,CELSR3,CBLB,PCDH10,CDH6,FBN2,PCDHGC4,SPARC,SLIT3,SCUBE3,DST,THBS2,PCLO,SVEP1,SPTAN1,CACNA1B,ITGB1,SLIT1,STIM1,LRP4,LTBP3,LRP1,HSP90B1,TPT1,PCDH17,PCDH20,PCDH9,ACTN1,RYR3,THBS1,ACAN,IQGAP1,RAB11FIP3,ZZEF1,RHOT1,EPS15L1,ACTN4,MEGF8,PLCB1,JAG1,ITSN1,SCUBE1
GO:MF	nucleoside-triphosphatase regulator activity	GO:0060589	0.047478	183	48	RCC2,RAP1GAP,ARHGAP29,ARHGEF11,RGL1,RAB3GAP2,ASAP2,CCDC88A,RAPGEF4,AGAP1,SRGAP3,ADRA2C,RASGEF1B,TRIO,RAPGEF6,PDGFRB,SYNGAP1,DEF6,SMAP1,STXBP5,CYTH3,GAPVD1,RAPGEF1,ARHGAP21,ARHGEF17,SRGAP1,TBC1D30,ARHGAP5,HERC2,HERC1,AKAP13,IQGAP1,TSC2,RAP1GAP2,ARHGAP44,FLCN,GIT1,NF1,RUNDC3A,CYTH1,EVI5L,PLCB1,ARFGEF2,TIAM1,ITSN1,BCR,SBF1,IQSEC2
GO:MF	GTPase regulator activity	GO:0030695	0.047478	183	48	RCC2,RAP1GAP,ARHGAP29,ARHGEF11,RGL1,RAB3GAP2,ASAP2,CCDC88A,RAPGEF4,AGAP1,SRGAP3,ADRA2C,RASGEF1B,TRIO,RAPGEF6,PDGFRB,SYNGAP1,DEF6,SMAP1,STXBP5,CYTH3,GAPVD1,RAPGEF1,ARHGAP21,ARHGEF17,SRGAP1,TBC1D30,ARHGAP5,HERC2,HERC1,AKAP13,IQGAP1,TSC2,RAP1GAP2,ARHGAP44,FLCN,GIT1,NF1,RUNDC3A,CYTH1,EVI5L,PLCB1,ARFGEF2,TIAM1,ITSN1,BCR,SBF1,IQSEC2
GO:BP	signal transduction	GO:0007165	0.005746	1769	337	PRKCZ,SKI,PARK7,PIK3CD,UBE4B,RCC2,RAP1GAP,EPHB2,RPA2,EYA3,GRIK3,MACF1,RNF220,FAF1,LRP8,ARHGAP29,CELSR2,SORT1,CSF1,RBM15,RHOC,NOTCH2,ASH1L,LMNA,ARHGEF11,DDR2,RC3H1,LAMC1,RGL1,PIK3C2B,MAPKAPK2,PPP2R5A,CNIH3,TRIM67,ACTN2,FMN2,SOX11,YWHAQ,DDX1,MSH2,SPTBN1,CCDC88A,CYP26B1,INPP4A,MBD5,LRP2,TLK1,RAPGEF4,CALCRL,HSPD1,CUL3,ITM2C,NCL,SRGAP3,RAF1,NR2C2,PTH1R,CELSR3,GNAI2,NISCH,CACNA1D,SLMAP,FOXP1,ROBO2,EPHA6,CBLB,ADCY5,PLXNA1,TOPBP1,MRAS,MECOM,OPA1,HTT,ADRA2C,PDGFRA,RASGEF1B,MAPK10,BMPR1B,NFKB1,ANK2,CAMK2D,JADE1,HHIP,ADCY2,TRIO,CDH6,PRLR,PTGER4,MAP3K1,PDE8B,AP3B1,JMY,APC,FBN2,RAPGEF6,FNIP1,NDFIP1,PDGFRB,CAMK2A,GRIA1,SLIT3,GMDS,RANBP9,AGPAT1,SYNGAP1,SCUBE3,DEF6,UBR2,RUNX2,DST,PTPRK,IGF2R,CYTH3,AHR,TBX20,CDK13,HECW1,VWC2,AUTS2,HIP1,HGF,GRM3,COL1A2,RELN,SRPK2,PLXNA4,PTK2B,DUSP4,NRG1,FGFR1,KAT6A,PRKDC,SOX17,LYN,MOS,GDF6,YWHAZ,RIMS2,EXT1,TRIB1,KCNQ3,PTK2,ARC,FAM83H,PTPRD,MLLT3,ZFAND5,RORB,TLE1,DAPK1,SEMA4D,WNK2,NR4A3,SVEP1,RABGAP1,SCAI,RAPGEF1,TRAF2,ARHGAP21,ABI1,ZEB1,ITGB1,CREM,ANK3,BMPR1A,PTEN,NFKB2,SUFU,WBP1L,EIF3A,GRK5,BRSK2,DUSP8,CDKN1C,APBB1,MAPK8IP1,LRP4,NR1H3,GPR137,LTBP3,RBM4,LRP5,ANO1,NUMA1,ARHGEF17,TENM4,GRM5,WNK1,PTPN6,EPS8,STRAP,RASSF8,STK38L,HDAC7,KRT18,RARG,STAT2,LRP1,SRGAP1,GRIP1,EEA1,CUX2,KSR2,NCOR2,AKAP11,TSC22D1,TPT1,NDFIP2,RAP2A,MYO16,ARHGAP5,AKAP6,SSTR1,MAP4K5,PPM1A,SLC39A9,SYNJ2BP,TGFB3,TRAF3,PPP1R13B,CYFIP1,HERC2,RYR3,THBS1,USP8,LEO1,SMAD3,TLE3,PARP6,AKAP13,IQGAP1,CRTC3,NR2F2,MEF2A,MAPK8IP3,TSC2,PKD1,CREBBP,CDIP1,GRIN2A,PAGR1,VPS35,BRD7,MMP2,HERPUD1,GLG1,BCAR1,PLCG2,USP10,RAP1GAP2,MINK1,TNFSF13,RPL26,ARHGAP44,NCOR1,FLCN,GIT1,NF1,RHOT1,ASIC2,STAT5B,STAT3,RUNDC3A,FZD2,COL1A1,MTMR4,TLK2,MAP3K3,ERN1,DDX5,SOX9,SEC14L1,SOCS3,CYTH1,EIF4A3,THOC1,PTPRM,PPP4R1,LDLRAD4,MIB1,DTNA,SKOR2,MALT1,BCL2,MADCAM1,PALM,PTBP1,CSNK1G2,PIP5K1C,MAP2K2,SAFB2,C3,PIN1,PDE4A,SMARCA4,PRKACA,BABAM1,FCHO1,HOMER3,ZNF536,ACTN4,AKT2,GRIK5,MEGF8,PPP5C,FLT3LG,EPN1,PLCB1,JAG1,BCL2L1,CBFA2T2,ITCH,SRC,NCOA3,ARFGEF2,SPATA2,USP25,TIAM1,ITSN1,PI4KA,BCR,PDGFB,SCUBE1,GRAMD4,PLXNB2,SHANK3,GPR143,GLRA2,DMD,DDX3X,KDM6A,MAGED1,IQSEC2,MED12,COL4A6,IRS4,MECP2
GO:BP	cell adhesion	GO:0007155	0.005746	506	118	PRKCZ,RCC2,EPHB2,RUNX3,MACF1,FAF1,CELSR2,CSF1,DDR2,RC3H1,LAMC1,NFASC,ACTN2,KIF26B,RAB10,CLASP1,CNTNAP5,HSPD1,ADAM23,ARPC2,SNED1,CLASP2,CELSR3,ROBO2,CBLB,LSAMP,PLXNA1,PODXL2,OPA1,LIMCH1,PDGFRA,PCDH10,CDH6,PDZD2,PRLR,AP3B1,APC,PCDHGC4,NDFIP1,BRD2,DST,COL12A1,PTPRK,ARID1B,THBS2,ELFN1,CYTH3,VWC2,ACTL6B,RELN,LRRC4,PLXNA4,SLC4A2,DMTN,PTK2B,NRG1,LYN,EXT1,PTK2,PTPRD,SEMA4D,NR4A3,SVEP1,RAPGEF1,COL5A1,ITGB1,ANK3,PTEN,LRP5,TENM4,HEPACAM,IGSF9B,WNK1,PTPN6,KRT18,SMARCC2,PPFIA2,FNDC3A,PCDH17,PCDH20,PCDH9,ARHGAP5,ZBTB1,ACTN1,THBS1,TLN2,SMAD3,ACAN,PKD1,BRD7,MMP2,ZFHX3,BCAR1,MINK1,MYH10,NF1,STAT5B,COL1A1,SOX9,CYTH1,PTPRM,MALT1,BCL2,MADCAM1,PIP5K1C,SMARCA4,FCHO1,ACTN4,SYMPK,JAG1,ITCH,SRC,TIAM1,BCR,PDGFB,PLXNB2,DMD,COL4A6
GO:BP	regulation of transport	GO:0051049	0.006196	575	130	PRKCZ,KCNAB2,PARK7,EPHB2,RNF220,NOS1AP,DHX9,CNIH3,ACTN2,SOX11,YWHAQ,CLASP1,IWS1,RAPGEF4,SESTD1,MAP2,PER2,CLASP2,PDCD6IP,SCN5A,SETD2,GNAI2,CACNA1D,ERC2,SLMAP,CBLB,ADCY5,DNAJC13,HTT,ADRA2C,NFKB1,ANK2,CAMK2D,PDE8B,KIF3A,DIAPH1,NDFIP1,YIPF5,PDGFRB,CAMK2A,GRIA1,SMAP1,SNAP91,STXBP5,HIP1,CACNA2D1,PCLO,RELN,PTK2B,NRG1,FGFR1,LYN,RIMS2,ARC,DAPK1,WNK2,NR4A3,GAPVD1,RAPGEF1,ABCA2,FRMD4A,ITGB1,ANK3,GLUD1,PTEN,SUFU,BRSK2,STIM1,APBB1,SLC1A2,NR1H3,OSBP,AHNAK,RBM4,LRP5,ANO1,NUMA1,WNK1,PTPN6,ASIC1,SLC4A8,LRP1,PPFIA2,ATXN2,NDFIP2,AKAP6,PPM1A,SYNJ2BP,TGFB3,HSP90AA1,CYFIP1,THBS1,SMAD3,MEF2A,RAB11FIP3,TSC2,GRIN2A,VPS35,AP1G1,PLCG2,MINK1,MYH10,ARHGAP44,ALKBH5,MYO18A,GIT1,NF1,ASIC2,CACNB1,MTMR4,MIB1,NEDD4L,BCL2,MAP2K2,C3,PRKACA,HOMER3,ACTN4,AKT2,GRIK5

						,PLCB1,BCL2L1,SRC,ATP9A,TIAM1,BCR,PDGFB,GPR143,DMD,MECP2
GO:BP	regulation of localization	GO:0032879	0.010593	743	157	PRKCZ,KCNAB2,PARK7,EPHB2,RNF220,CELSR2,LMNA,NOS1AP,DHX9,PPP2R5A,CNIH3,TRIM67,ACTN2,SOX11,YWHAQ,SPTBN1,CCDC88A,CLASP1,IWS1,RAPGEF4,SESTD1,MAP2,PER2,CLASP2,PDCD6IP,SCN5A,SETD2,ARIH2,GNAI2,CACNA1D,ERC2,SLMAP,CBLB,ADCY5,DNAJC13,EIF4G1,HTT,ADRA2C,NFKB1,ANK2,CAMK2D,PDE8B,APC,KIF3A,DIAPH1,NDFIP1,YIPF5,PDGFRB,CAMK2A,GRIA1,SMAP1,SNAP91,EPB41L2,STXBP5,TCP1,HIP1,CACNA2D1,PCLO,RELN,DMTN,PTK2B,NRG1,FGFR1,LYN,CCNE2,YWHAZ,RIMS2,ARC,DAPK1,WNK2,NR4A3,GAPVD1,RAPGEF1,ABCA2,FRMD4A,ITGB1,ANK3,GLUD1,PTEN,SUFU,BRSK2,STIM1,APBB1,SLC1A2,LRP4,NR1H3,OSBP,AHNAK,RBM4,LRP5,ANO1,NUMA1,WNK1,PTPN6,ASIC1,SLC4A8,LRP1,CCT2,PPFIA2,ATXN2,NBEA,PCDH17,NDFIP2,AKAP6,PPM1A,SYNJ2BP,TGFB3,HSP90AA1,CYFIP1,RYR3,THBS1,USP8,SMAD3,MEF2A,RAB11FIP3,TSC2,CREBBP,GRIN2A,VPS35,AP1G1,PLCG2,MINK1,MYH10,ARHGAP44,ALKBH5,MYO18A,GIT1,NF1,ASIC2,CACNB1,NPEPPS,MTMR4,CLTC,MIB1,NEDD4L,BCL2,MAP2K2,C3,PIN1,PRKACA,HOMER3,ACTN4,AKT2,GRIK5,PLCB1,BCL2L1,SRC,ATP9A,TIAM1,BCR,PDGFB,SHANK3,GPR143,DMD,MAGED1,IQSEC2,MECP2
GO:BP	cell-matrix adhesion	GO:0007160	0.010593	87	30	PRKCZ,RCC2,MACF1,CSF1,ACTN2,CLASP1,SNED1,CLASP2,LIMCH1,PTPRK,DMTN,PTK2B,PTK2,SVEP1,ITGB1,PTEN,PPFIA2,ACTN1,THBS1,SMAD3,PKD1,MINK1,NF1,BCL2,MADCAM1,JAG1,SRC,TIAM1,BCR,DMD
GO:BP	cellular localization	GO:0051641	0.010593	1229	241	PRKCZ,KCNAB2,PARK7,TMEM201,PIK3CD,KIF1B,RCC2,EPHB2,STX12,RPA2,MACF1,RNF220,NDC1,CELSR2,SORT1,LMNA,DHX9,PPP2R5A,RAB3GAP2,TRIM67,ACTN2,FMN2,YWHAQ,DDX1,RAB10,SIX2,MSH2,SPTBN1,CCDC88A,CLASP1,IWS1,KIF5C,SCN1A,LRP2,GORASP2,TLK1,RAPGEF4,CALCRL,HSPD1,MAP2,CUL3,ATG16L1,AGAP1,PER2,RAF1,CLASP2,PDCD6IP,SETD2,MAP4,ARIH2,ERC2,SLMAP,CBLB,ADCY5,DNAJC13,TOPBP1,EIF4G1,OPA1,HTT,ADRA2C,ANK2,CAMK2D,ABCE1,ZNF827,PDE8B,AP3B1,SCAMP1,APC,FBN2,RAPGEF6,KIF3A,HSPA4,HSPA9,DIAPH1,NDFIP1,YIPF5,CAMK2A,E2F3,BRD2,SYNGAP1,DEF6,TRAM2,DST,SNAP91,PTPRK,EPB41L2,STXBP5,TCP1,IGF2R,HNRNPA2B1,VWC2,HIP1,PCLO,RELN,LRRC4,NUP205,XPO7,DMTN,PTK2B,NRG1,LYN,MOS,CCNE2,YWHAZ,RIMS2,KCNQ3,ARC,FAM83H,NOL6,UBAP1,CLTA,NR4A3,GAPVD1,GOLGA2,NUP188,ABCA2,FRMD4A,ARHGAP21,ITGB1,ANK3,GLUD1,PTEN,LGI1,SLIT1,SUFU,BRSK2,APBB1,SLC1A2,LRP4,NR1H3,OSBP,RBM4,LRP5,ANO1,NUMA1,C1QTNF5,HEPACAM,WNK1,PTPN6,ASIC1,SLC4A8,KRT18,LRP1,GRIP1,CCT2,PPFIA2,EEA1,HSP90B1,NBEA,AKAP11,COG3,PCDH17,NDFIP2,RAP2A,AKAP6,PPM1A,SYNJ2BP,TGFB3,TTC7B,HSP90AA1,TECPR2,CYFIP1,HERC2,APBA2,RYR3,EMC4,SRP14,USP8,SNX1,SMAD3,AP3B2,RAB11FIP3,MAPK8IP3,TSC2,PKD1,CREBBP,GRIN2A,XPO6,VPS35,HERPUD1,AP1G1,PLCG2,EMC8,BANP,MYH10,ARHGAP44,ALKBH5,MYO18A,GIT1,NF1,RHOT1,ASIC2,SYNRG,CASC3,STAT3,ATP6V0A1,NPEPPS,COL1A1,MTMR4,CLTC,SOX9,NUP85,EIF4A3,THOC1,CHMP1B,NEDD4L,MALT1,BCL2,MADCAM1,PALM,PIP5K1C,MAP2K2,PIN1,PRKACA,EPS15L1,HOMER3,ATP13A1,ACTN4,AKT2,GRIK5,LIN7B,PPFIA3,PLCB1,BCL2L1,SRC,ARFGEF2,ATP9A,TIAM1,ITSN1,MORC3,PEX26,BCR,GGA1,SHANK3,GPR143,AP1S2,DMD,DDX3X,MAGED1,IQSEC2,MECP2
GO:BP	intracellular signaling cassette	GO:0141124	0.010593	723	154	PRKCZ,SKI,PARK7,PIK3CD,RAP1GAP,EPHB2,ARHGAP29,CSF1,RHOC,NOTCH2,ASH1L,ARHGEF11,DDR2,RC3H1,RGL1,PIK3C2B,MAPKAPK2,TRIM67,YWHAQ,DDX1,SPTBN1,CCDC88A,LRP2,RAPGEF4,CUL3,SRGAP3,RAF1,GNAI2,NISCH,MRAS,MECOM,HTT,ADRA2C,PDGFRA,RASGEF1B,MAPK10,NFKB1,ANK2,CAMK2D,ADCY2,TRIO,PTGER4,MAP3K1,PDE8B,RAPGEF6,NDFIP1,PDGFRB,GRIA1,RANBP9,SYNGAP1,DEF6,RUNX2,CYTH3,AHR,TBX20,AUTS2,HIP1,HGF,COL1A2,RELN,PTK2B,DUSP4,NRG1,FGFR1,LYN,MOS,GDF6,YWHAZ,TRIB1,PTK2,TLE1,SEMA4D,WNK2,SCAI,RAPGEF1,TRAF2,ARHGAP21,ITGB1,BMPR1A,PTEN,NFKB2,EIF3A,DUSP8,MAPK8IP1,LRP4,ARHGEF17,GRM5,PTPN6,EPS8,HDAC7,LRP1,KSR2,NDFIP2,RAP2A,MYO16,ARHGAP5,AKAP6,MAP4K5,PPM1A,SYNJ2BP,TGFB3,TRAF3,CYFIP1,RYR3,THBS1,USP8,SMAD3,AKAP13,IQGAP1,MEF2A,MAPK8IP3,TSC2,CREBBP,GRIN2A,PLCG2,USP10,RAP1GAP2,MINK1,ARHGAP44,NCOR1,FLCN,GIT1,NF1,RHOT1,STAT3,RUNDC3A,MAP3K3,ERN1,SOX9,CYTH1,LDLRAD4,MALT1,PTBP1,PIP5K1C,MAP2K2,PIN1,PDE4A,PRKACA,HOMER3,ACTN4,PPP5C,PLCB1,ITCH,SRC,ARFGEF2,TIAM1,ITSN1,BCR,PDGFB,SHANK3,GPR143,DMD,DDX3X,IQSEC2
GO:BP	cell surface receptor signaling pathway	GO:0007166	0.010593	952	195	PRKCZ,SKI,PARK7,PIK3CD,RCC2,EPHB2,EYA3,GRIK3,MACF1,RNF220,FAF1,LRP8,CELSR2,SORT1,CSF1,RBM15,NOTCH2,LMNA,DDR2,RC3H1,LAMC1,MAPKAPK2,SOX11,SPTBN1,CCDC88A,MBD5,LRP2,CALCRL,CUL3,ITM2C,NCL,RAF1,PTH1R,CELSR3,GNAI2,FOXP1,ROBO2,EPHA6,CBLB,ADCY5,PLXNA1,HTT,PDGFRA,MAPK10,BMPR1B,NFKB1,JADE1,HHIP,TRIO,CDH6,PRLR,MAP3K1,APC,FBN2,PDGFRB,CAMK2A,GRIA1,SLIT3,GMDS,AGPAT1,SCUBE3,UBR2,RUNX2,DST,PTPRK,IGF2R,TBX20,HECW1,VWC2,HIP1,HGF,GRM3,COL1A2,RELN,PLXNA4,PTK2B,NRG1,FGFR1,SOX17,LYN,GDF6,RIMS2,EXT1,TRIB1,PTK2,PTPRD,MLLT3,ZFAND5,TLE1,DAPK1,SEMA4D,WNK2,NR4A3,SVEP1,RABGAP1,RAPGEF1,TRAF2,ABI1,ZEB1,ITGB1,BMPR1A,PTEN,SUFU,WBP1L,GRK5,CDKN1C,LRP4,NR1H3,LTBP3,RBM4,LRP5,NUMA1,GRM5,WNK1,PTPN6,STRAP,KRT18,RARG,STAT2,LRP1,EEA1,CUX2,TSC22D1,SSTR1,PPM1A,SYNJ2BP,TGFB3,TRAF3,CYFIP1,HERC2,THBS1,USP8,LEO1,SMAD3,TLE3,IQGAP1,TSC2,PKD1,CREBBP,GRIN2A,VPS35,BRD7,MMP2,GLG1,BCAR1,PLCG2,FLCN,GIT1,NF1,STAT5B,STAT3,FZD2,COL1A1,MTMR4,DDX5,SOX9,SOCS3,EIF4A3,LDLRAD4,MIB1,SKOR2,MALT1,BCL2,MADCAM1,CSNK1G2,MAP2K2,C3,PIN1,SMARCA4,PRKACA,FCHO1,HOMER3,ACTN4,AKT2,GRIK5,MEGF8,EPN1,PLCB1,JAG1,BCL2L1,CBFA2T2,SRC,SPATA2,USP25,TIAM1,PDGFB,SCUBE1,PLXNB2,SHANK3,GLRA2,DDX3X,MED12,COL4A6,IRS4,MECP2
GO:BP	regulation of signal transduction	GO:0009966	0.010593	1121	223	PRKCZ,SKI,PARK7,RAP1GAP,EPHB2,RPA2,EYA3,MACF1,RNF220,FAF1,ARHGAP29,CSF1,RHOC,NOTCH2,ASH1L,LMNA,ARHGEF11,DDR2,RC3H1,LAMC1,MAPKAPK2,CNIH3,TRIM67,SOX11,DDX1,SPTBN1,CCDC88A,CYP26B1,MBD5,LRP2,CUL3,ITM2C,NCL,SRGAP3,RAF1,GNAI2,SLMAP,FOXP1,ROBO2,CBLB,MECOM,OPA1,HTT,ADRA2C,PDGFRA,BMPR1B,NFKB1,JADE1,HHIP,TRIO,PRLR,PDE8B,APC,FBN2,FNIP1,NDFIP1,PDGFRB,CAMK2A,GRIA1,SLIT3,RANBP9,AGPAT1,SYNGAP1,SCUBE3,DEF6,UBR2,RUNX2,CYTH3,TBX20,CDK13,HECW1,VWC2,AUTS2,HIP1,HGF,RELN,PTK2B,DUSP4,NRG1,FGFR1,KAT6A,PRKDC,SOX17,LYN,MOS,GDF6,YWHAZ,RIMS2,EXT1,TRIB1,PTK2,ARC,PTPRD,MLLT3,TLE1,DAPK1,SEMA4D,WNK2,RABGAP1,SCAI,RAPGEF1,TRAF2,ARHGAP21,ZEB1,ITGB1,BMPR1A,

						PTEN,SUFU,EIF3A,GRK5,DUSP8,CDKN1C,MAPK8IP1,LRP4,NR1H3,GPR137,RBM4,NUMA1,ARHGEF17,GRM5,WNK1,PTPN6,EPS8,STRAP,HDAC7,STAT2,LRP1,CUX2,KSR2,NCOR2,TSC22D1,TPT1,NDFIP2,RAP2A,ARHGAP5,AKAP6,PPM1A,SYNJ2BP,TGFB3,TRAF3,CYFIP1,HERC2,THBS1,USP8,SMAD3,TLE3,AKAP13,IQGAP1,CRTC3,MAPK8IP3,TSC2,CREBBP,GRIN2A,PAGR1,VPS35,MMP2,HERPUD1,GLG1,PLCG2,USP10,RAP1GAP2,MINK1,RPL26,ARHGAP44,NCOR1,FLCN,GIT1,NF1,STAT3,RUNDC3A,COL1A1,MTMR4,MAP3K3,ERN1,DDX5,SOX9,SEC14L1,SOCS3,CYTH1,EIF4A3,LDLRAD4,SKOR2,MALT1,BCL2,PALM,PTBP1,CSNK1G2,PIP5K1C,MAP2K2,SAFB2,C3,PIN1,PDE4A,SMARCA4,PRKACA,HOMER3,ZNF536,ACTN4,MEGF8,PPP5C,PLCB1,JAG1,BCL2L1,CBFA2T2,ITCH,SRC,NCOA3,ARFGEF2,SPATA2,USP25,TIAM1,ITSN1,BCR,PDGFB,SCUBE1,GRAMD4,SHANK3,GPR143,DMD,DDX3X,KDM6A,MAGED1,IQSEC2
GO:BP	regulation of cell communication	GO:0010646	0.010593	1308	255	PRKCZ,SKI,PARK7,RAP1GAP,EPHB2,RPA2,EYA3,GRIK3,MACF1,RNF220,FAF1,LRP8,ARHGAP29,CSF1,RHOC,NOTCH2,ASH1L,LMNA,ARHGEF11,DDR2,RC3H1,LAMC1,MAPKAPK2,CNIH3,TRIM67,SOX11,DDX1,SPTBN1,CCDC88A,CYP26B1,MBD5,LRP2,RAPGEF4,CUL3,ITM2C,NCL,PER2,SRGAP3,RAF1,GNAI2,ERC2,SLMAP,FOXP1,ROBO2,CBLB,ADCY5,MECOM,OPA1,HTT,ADRA2C,PDGFRA,BMPR1B,NFKB1,ANK2,CAMK2D,JADE1,HHIP,TRIO,PRLR,PDE8B,APC,FBN2,FNIP1,NDFIP1,PDGFRB,CAMK2A,GRIA1,SLIT3,RANBP9,AGPAT1,SYNGAP1,SCUBE3,DEF6,UBR2,RUNX2,STXBP5,CYTH3,TBX20,CDK13,HECW1,VWC2,AUTS2,HIP1,HGF,GRM3,RELN,LRRC4,PTK2B,DUSP4,NRG1,FGFR1,KAT6A,PRKDC,SOX17,LYN,MOS,GDF6,YWHAZ,RIMS2,EXT1,TRIB1,KCNQ3,PTK2,ARC,PTPRD,MLLT3,TLE1,DAPK1,SEMA4D,WNK2,RABGAP1,SCAI,RAPGEF1,TRAF2,CACNA1B,ARHGAP21,ZEB1,ITGB1,ANK3,BMPR1A,GLUD1,PTEN,LGI1,SUFU,EIF3A,GRK5,BRSK2,DUSP8,CDKN1C,MAPK8IP1,LRP4,NR1H3,OSBP,GPR137,RBM4,LRP5,ANO1,NUMA1,ARHGEF17,GRM5,KMT2A,WNK1,PTPN6,EPS8,STRAP,HDAC7,ASIC1,SLC4A8,STAT2,LRP1,PPFIA2,CUX2,KSR2,NCOR2,TSC22D1,TPT1,PCDH17,NDFIP2,RAP2A,ARHGAP5,AKAP6,PPM1A,SYNJ2BP,TGFB3,TRAF3,CYFIP1,HERC2,APBA2,THBS1,USP8,SMAD3,TLE3,AKAP13,IQGAP1,CRTC3,RAB11FIP3,MAPK8IP3,TSC2,CREBBP,GRIN2A,PAGR1,VPS35,IRX3,MMP2,HERPUD1,GLG1,PLCG2,USP10,RAP1GAP2,MINK1,RPL26,ARHGAP44,NCOR1,FLCN,GIT1,NF1,STAT3,RUNDC3A,COL1A1,MTMR4,MAP3K3,ERN1,DDX5,SOX9,SEC14L1,SOCS3,CYTH1,EIF4A3,LDLRAD4,SKOR2,MALT1,BCL2,PALM,PTBP1,CSNK1G2,PIP5K1C,MAP2K2,SAFB2,C3,PIN1,PDE4A,SMARCA4,PRKACA,HOMER3,ZNF536,ACTN4,GRIK5,MEGF8,PPP5C,PPFIA3,PLCB1,JAG1,BCL2L1,CBFA2T2,ITCH,SRC,NCOA3,ARFGEF2,SPATA2,USP25,TIAM1,ITSN1,BCR,PDGFB,SCUBE1,GRAMD4,SHANK3,GPR143,GLRA2,DMD,DDX3X,KDM6A,MAGED1,IQSEC2,MECP2
GO:BP	cell communication	GO:0007154	0.01687	1986	364	PRKCZ,SKI,PARK7,PIK3CD,UBE4B,KIF1B,RCC2,RAP1GAP,EPHB2,RPA2,EYA3,GRIK3,MACF1,RNF220,FAF1,LRP8,ARHGAP29,CELSR2,SORT1,CSF1,RBM15,RHOC,NOTCH2,ASH1L,LMNA,ARHGEF11,DDR2,RC3H1,LAMC1,RGL1,PIK3C2B,MAPKAPK2,PPP2R5A,CNIH3,TRIM67,ACTN2,FMN2,SOX11,YWHAQ,DDX1,MSH2,SPTBN1,CCDC88A,CYP26B1,INPP4A,MBD5,SCN1A,LRP2,TLK1,RAPGEF4,CALCRL,HSPD1,CUL3,ITM2C,NCL,PER2,SRGAP3,RAF1,NR2C2,SCN5A,PTH1R,CELSR3,GNAI2,NISCH,CACNA1D,ERC2,SLMAP,FOXP1,ROBO2,EPHA6,CBLB,ADCY5,PLXNA1,TOPBP1,MRAS,MECOM,OPA1,HTT,ADRA2C,PDGFRA,RASGEF1B,MAPK10,BMPR1B,NFKB1,ANK2,CAMK2D,JADE1,HHIP,ADCY2,TRIO,CDH6,PRLR,PTGER4,MAP3K1,PDE8B,AP3B1,JMY,APC,FBN2,RAPGEF6,FNIP1,NDFIP1,PDGFRB,CAMK2A,GRIA1,SLIT3,GMDS,RANBP9,AGPAT1,SYNGAP1,SCUBE3,DEF6,UBR2,RUNX2,DST,PTPRK,STXBP5,IGF2R,CYTH3,AHR,TBX20,CDK13,HECW1,VWC2,AUTS2,HIP1,HGF,CACNA2D1,PCLO,GRM3,COL1A2,RELN,SRPK2,LRRC4,PLXNA4,PTK2B,DUSP4,NRG1,FGFR1,KAT6A,PRKDC,SOX17,LYN,MOS,GDF6,YWHAZ,RIMS2,EXT1,TRIB1,KCNQ3,PTK2,ARC,FAM83H,PTPRD,MLLT3,ZFAND5,RORB,TLE1,DAPK1,SEMA4D,WNK2,NR4A3,SVEP1,RABGAP1,SCAI,RAPGEF1,TRAF2,CACNA1B,ARHGAP21,ABI1,ZEB1,ITGB1,CREM,ANK3,BMPR1A,GLUD1,PTEN,LGI1,NFKB2,SUFU,WBP1L,EIF3A,GRK5,BRSK2,DUSP8,CDKN1C,APBB1,SLC1A2,MAPK8IP1,LRP4,NR1H3,OSBP,GPR137,LTBP3,RBM4,LRP5,ANO1,NUMA1,ARHGEF17,TENM4,GRM5,KMT2A,WNK1,PTPN6,EPS8,STRAP,RASSF8,STK38L,HDAC7,ASIC1,SLC4A8,KRT18,RARG,STAT2,LRP1,SRGAP1,GRIP1,PPFIA2,EEA1,CUX2,KSR2,NCOR2,AKAP11,TSC22D1,TPT1,PCDH17,NDFIP2,RAP2A,MYO16,ARHGAP5,AKAP6,SSTR1,MAP4K5,PPM1A,SLC39A9,SYNJ2BP,TGFB3,TRAF3,PPP1R13B,CYFIP1,HERC2,APBA2,RYR3,THBS1,USP8,LEO1,FOXB1,SMAD3,TLE3,PARP6,AKAP13,IQGAP1,CRTC3,NR2F2,MEF2A,RAB11FIP3,MAPK8IP3,TSC2,PKD1,CREBBP,CDIP1,GRIN2A,PAGR1,VPS35,BRD7,IRX3,MMP2,HERPUD1,GLG1,BCAR1,PLCG2,USP10,RAP1GAP2,MINK1,TNFSF12-TNFSF13,TNFSF13,RPL26,ARHGAP44,NCOR1,FLCN,GIT1,NF1,RHOT1,ASIC2,CACNB1,STAT5B,STAT3,RUNDC3A,FZD2,COL1A1,MTMR4,TLK2,MAP3K3,ERN1,DDX5,SOX9,SEC14L1,SOCS3,CYTH1,EIF4A3,THOC1,PTPRM,PPP4R1,LDLRAD4,MIB1,DTNA,SKOR2,MALT1,BCL2,MADCAM1,PALM,PTBP1,CSNK1G2,PIP5K1C,MAP2K2,SAFB2,C3,PIN1,PDE4A,SMARCA4,PRKACA,BABAM1,FCHO1,HOMER3,ZNF536,ACTN4,AKT2,GRIK5,MEGF8,PPP5C,LIN7B,PPFIA3,FLT3LG,EPN1,PLCB1,JAG1,BCL2L1,CBFA2T2,ITCH,SRC,NCOA3,ARFGEF2,SPATA2,USP25,TIAM1,ITSN1,PI4KA,BCR,PDGFB,SCUBE1,GRAMD4,PLXNB2,SHANK3,GPR143,GLRA2,DMD,DDX3X,KDM6A,MAGED1,IQSEC2,MED12,COL4A6,IRS4,MECP2
GO:BP	skeletal system morphogenesis	GO:0048705	0.018636	113	35	SKI,COL11A1,SOX11,SIX2,CYP26B1,SATB2,SETD2,PDGFRA,BMPR1B,HHIP,FBN2,RUNX2,FGFR1,EXT1,PAX5,ZFAND5,COL27A1,ZEB1,LTBP3,LRP5,RARG,TMEM119,TGFB3,SMAD3,ACAN,PKD1,MMP2,GLG1,ANKRD11,HOXB3,COL1A1,SOX9,EIF4A3,MEGF8,MED12
GO:BP	regulation of signaling	GO:0023051	0.020378	1303	251	PRKCZ,SKI,PARK7,RAP1GAP,EPHB2,RPA2,EYA3,GRIK3,MACF1,RNF220,FAF1,LRP8,ARHGAP29,CSF1,RHOC,NOTCH2,ASH1L,LMNA,ARHGEF11,DDR2,RC3H1,LAMC1,MAPKAPK2,CNIH3,TRIM67,SOX11,DDX1,SPTBN1,CCDC88A,CYP26B1,MBD5,LRP2,RAPGEF4,CUL3,ITM2C,NCL,PER2,SRGAP3,RAF1,GNAI2,ERC2,SLMAP,FOXP1,ROBO2,CBLB,ADCY5,MECOM,OPA1,HTT,ADRA2C,PDGFRA,BMPR1B,NFKB1,JADE1,HHIP,TRIO,PRLR,PDE8B,APC,FBN2,FNIP1,NDFIP1,PDGFRB,CAMK2A,GRIA1,SLIT3,RANBP9,AGPAT1,SYNGAP1,SCUBE3,DEF6,UBR2,RUNX2,STXBP5,CYTH3,TBX20,CDK13,HECW1,VWC2,AUTS2,HIP1,HGF,GRM3,RELN,LRRC4,PTK2B,DUSP4,NRG1,FGFR1,KAT6A,PRKDC,SOX17,LYN,MOS,GDF6,YWHAZ,RIMS2,EXT1,TRIB1,KCNQ3,PTK2,ARC,PTPRD,MLLT3,TLE1,DAPK1,SEMA4D,WNK2,RABGAP1,SCAI,RAPGEF1,TRAF2,CACNA1B,ARHGAP21,ZEB1,ITGB1,BMPR1A,GLUD1,PTEN,LGI1,SUFU,EIF3A,GRK5,BRSK2,DUSP8,CDKN1C,MAPK8IP1,LRP4,NR1H3,OSBP,GPR137,RBM4,LRP5,ANO1,NUMA1,ARHGEF17,GRM5,KMT2A,WNK1,PTPN6,EPS8,STR

						AP,HDAC7,ASIC1,SLC4A8,STAT2,LRP1,PPFIA2,CUX2,KSR2,NCOR2,TSC22D1,TPT1,PCDH17,NDFIP2,RAP2A,ARHGAP5,AKAP6,PPM1A,SYNJ2BP,TGFB3,TRAF3,CYFIP1,HERC2,APBA2,THBS1,USP8,SMAD3,TLE3,AKAP13,IQGAP1,CRTC3,RAB11FIP3,MAPK8IP3,TSC2,CREBBP,GRIN2A,PAGR1,VPS35,MMP2,HERPUD1,GLG1,PLCG2,USP10,RAP1GAP2,MINK1,RPL26,ARHGAP44,NCOR1,FLCN,GIT1,NF1,STAT3,RUNDC3A,COL1A1,MTMR4,MAP3K3,ERN1,DDX5,SOX9,SEC14L1,SOCS3,CYTH1,EIF4A3,LDLRAD4,SKOR2,MALT1,BCL2,PALM,PTBP1,CSNK1G2,PIP5K1C,MAP2K2,SAFB2,C3,PIN1,PDE4A,SMARCA4,PRKACA,HOMER3,ZNF536,ACTN4,GRIK5,MEGF8,PPP5C,PPFIA3,PLCB1,JAG1,BCL2L1,CBFA2T2,ITCH,SRC,NCOA3,ARFGEF2,SPATA2,USP25,TIAM1,ITSN1,BCR,PDGFB,SCUBE1,GRAMD4,SHANK3,GPR143,GLRA2,DMD,DDX3X,KDM6A,MAGED1,IQSEC2,MECP2
GO:BP	signaling	GO:0023052	0.021182	1976	361	PRKCZ,SKI,PARK7,PIK3CD,UBE4B,KIF1B,RCC2,RAP1GAP,EPHB2,RPA2,EYA3,GRIK3,MACF1,RNF220,FAF1,LRP8,ARHGAP29,CELSR2,SORT1,CSF1,RBM15,RHOC,NOTCH2,ASH1L,LMNA,ARHGEF11,DDR2,RC3H1,LAMC1,RGL1,PIK3C2B,MAPKAPK2,PPP2R5A,CNIH3,TRIM67,ACTN2,FMN2,SOX11,YWHAQ,DDX1,MSH2,SPTBN1,CCDC88A,CYP26B1,INPP4A,MBD5,LRP2,TLK1,RAPGEF4,CALCRL,HSPD1,CUL3,ITM2C,NCL,PER2,SRGAP3,RAF1,NR2C2,SCN5A,PTH1R,CELSR3,GNAI2,NISCH,CACNA1D,ERC2,SLMAP,FOXP1,ROBO2,EPHA6,CBLB,ADCY5,PLXNA1,TOPBP1,MRAS,MECOM,OPA1,HTT,ADRA2C,PDGFRA,RASGEF1B,MAPK10,BMPR1B,NFKB1,ANK2,CAMK2D,JADE1,HHIP,ADCY2,TRIO,CDH6,PRLR,PTGER4,MAP3K1,PDE8B,AP3B1,JMY,APC,FBN2,RAPGEF6,FNIP1,NDFIP1,PDGFRB,CAMK2A,GRIA1,SLIT3,GMDS,RANBP9,AGPAT1,SYNGAP1,SCUBE3,DEF6,UBR2,RUNX2,DST,PTPRK,STXBP5,IGF2R,CYTH3,AHR,TBX20,CDK13,HECW1,VWC2,AUTS2,HIP1,HGF,CACNA2D1,PCLO,GRM3,COL1A2,RELN,SRPK2,LRRC4,PLXNA4,PTK2B,DUSP4,NRG1,FGFR1,KAT6A,PRKDC,SOX17,LYN,MOS,GDF6,YWHAZ,RIMS2,EXT1,TRIB1,KCNQ3,PTK2,ARC,FAM83H,PTPRD,MLLT3,ZFAND5,RORB,TLE1,DAPK1,SEMA4D,WNK2,NR4A3,SVEP1,RABGAP1,SCAI,RAPGEF1,TRAF2,CACNA1B,ARHGAP21,ABI1,ZEB1,ITGB1,CREM,ANK3,BMPR1A,GLUD1,PTEN,LGI1,NFKB2,SUFU,WBP1L,EIF3A,GRK5,BRSK2,DUSP8,CDKN1C,APBB1,SLC1A2,MAPK8IP1,LRP4,NR1H3,OSBP,GPR137,LTBP3,RBM4,LRP5,ANO1,NUMA1,ARHGEF17,TENM4,GRM5,KMT2A,WNK1,PTPN6,EPS8,STRAP,RASSF8,STK38L,HDAC7,ASIC1,SLC4A8,KRT18,RARG,STAT2,LRP1,SRGAP1,GRIP1,PPFIA2,EEA1,CUX2,KSR2,NCOR2,AKAP11,TSC22D1,TPT1,PCDH17,NDFIP2,RAP2A,MYO16,ARHGAP5,AKAP6,SSTR1,MAP4K5,PPM1A,SLC39A9,SYNJ2BP,TGFB3,TRAF3,PPP1R13B,CYFIP1,HERC2,APBA2,RYR3,THBS1,USP8,LEO1,SMAD3,TLE3,PARP6,AKAP13,IQGAP1,CRTC3,NR2F2,MEF2A,RAB11FIP3,MAPK8IP3,TSC2,PKD1,CREBBP,CDIP1,GRIN2A,PAGR1,VPS35,BRD7,MMP2,HERPUD1,GLG1,BCAR1,PLCG2,USP10,RAP1GAP2,MINK1,TNFSF12-TNFSF13,TNFSF13,RPL26,ARHGAP44,NCOR1,FLCN,GIT1,NF1,RHOT1,ASIC2,CACNB1,STAT5B,STAT3,RUNDC3A,FZD2,COL1A1,MTMR4,TLK2,MAP3K3,ERN1,DDX5,SOX9,SEC14L1,SOCS3,CYTH1,EIF4A3,THOC1,PTPRM,PPP4R1,LDLRAD4,MIB1,DTNA,SKOR2,MALT1,BCL2,MADCAM1,PALM,PTBP1,CSNK1G2,PIP5K1C,MAP2K2,SAFB2,C3,PIN1,PDE4A,SMARCA4,PRKACA,BABAM1,FCHO1,HOMER3,ZNF536,ACTN4,AKT2,GRIK5,MEGF8,PPP5C,LIN7B,PPFIA3,FLT3LG,EPN1,PLCB1,JAG1,BCL2L1,CBFA2T2,ITCH,SRC,NCOA3,ARFGEF2,SPATA2,USP25,TIAM1,ITSN1,PI4KA,BCR,PDGFB,SCUBE1,GRAMD4,PLXNB2,SHANK3,GPR143,GLRA2,DMD,DDX3X,KDM6A,MAGED1,IQSEC2,MED12,COL4A6,IRS4,MECP2
GO:BP	small GTPase-mediated signal transduction	GO:0007264	0.025158	221	57	PARK7,RAP1GAP,EPHB2,ARHGAP29,CSF1,RHOC,NOTCH2,ARHGEF11,RGL1,TRIM67,YWHAQ,CCDC88A,RAPGEF4,CUL3,SRGAP3,RAF1,NISCH,MRAS,RASGEF1B,TRIO,RAPGEF6,SYNGAP1,DEF6,CYTH3,AUTS2,COL1A2,RELN,SEMA4D,SCAI,RAPGEF1,ARHGAP21,ITGB1,LRP4,ARHGEF17,EPS8,KSR2,RAP2A,ARHGAP5,SYNJ2BP,CYFIP1,USP8,AKAP13,TSC2,RAP1GAP2,ARHGAP44,FLCN,GIT1,NF1,RHOT1,RUNDC3A,CYTH1,SRC,ARFGEF2,TIAM1,ITSN1,BCR,IQSEC2
GO:BP	membrane depolarization	GO:0051899	0.025158	37	16	PRKCZ,PARK7,SCN3A,SCN1A,SCN5A,CACNA1D,SLMAP,ANK2,CAMK2D,CACNA2D1,KCNQ3,ANK3,KMT2A,NEDD4L,BCL2,SRC
GO:BP	collagen fibril organization	GO:0030199	0.025158	23	12	COL11A1,DDR2,ADAMTS2,COL12A1,COL1A2,EXT1,COL27A1,COL5A1,LOXL1,ACAN,NF1,COL1A1
GO:BP	protein localization	GO:0008104	0.026032	942	188	PRKCZ,KCNAB2,PARK7,TMEM201,RCC2,EPHB2,STX12,RPA2,MACF1,NDC1,CELSR2,SORT1,LMNA,DHX9,PPP2R5A,RAB3GAP2,TRIM67,ACTN2,FMN2,YWHAQ,DDX1,RAB10,SIX2,MSH2,SPTBN1,CCDC88A,KIF5C,LRP2,GORASP2,TLK1,RAPGEF4,CALCRL,HSPD1,MAP2,ATG16L1,AGAP1,PER2,RAF1,CLASP2,PDCD6IP,SETD2,ARIH2,SLMAP,CBLB,ADCY5,DNAJC13,TOPBP1,EIF4G1,ADRA2C,ANK2,ZNF827,PDE8B,AP3B1,SCAMP1,APC,FBN2,RAPGEF6,KIF3A,HSPA4,HSPA9,DIAPH1,NDFIP1,YIPF5,E2F3,BRD2,SYNGAP1,TRAM2,SNAP91,PTPRK,EPB41L2,STXBP5,TCP1,VWC2,HIP1,PCLO,RELN,LRRC4,NUP205,XPO7,DMTN,CCNE2,YWHAZ,RIMS2,KCNQ3,FAM83H,UBAP1,CLTA,GAPVD1,GOLGA2,NUP188,ABCA2,FRMD4A,ITGB1,ANK3,GLUD1,LGI1,SUFU,BRSK2,APBB1,LRP4,NR1H3,OSBP,RBM4,LRP5,ANO1,NUMA1,C1QTNF5,HEPACAM,WNK1,SLC4A8,KRT18,LRP1,GRIP1,CCT2,HSP90B1,NBEA,AKAP11,COG3,NDFIP2,RAP2A,AKAP6,PPM1A,SYNJ2BP,TGFB3,TTC7B,HSP90AA1,TECPR2,HERC2,APBA2,EMC4,SRP14,USP8,SNX1,SMAD3,AP3B2,RAB11FIP3,MAPK8IP3,TSC2,PKD1,CREBBP,GRIN2A,XPO6,VPS35,HERPUD1,AP1G1,EMC8,BANP,MYH10,ARHGAP44,MYO18A,NF1,ASIC2,SYNRG,STAT3,NPEPPS,COL1A1,CLTC,SOX9,NUP85,CHMP1B,NEDD4L,BCL2,MADCAM1,PALM,PIN1,PRKACA,HOMER3,ATP13A1,ACTN4,AKT2,LIN7B,PLCB1,BCL2L1,SRC,ARFGEF2,TIAM1,ITSN1,MORC3,PEX26,BCR,GGA1,SHANK3,AP1S2,DMD,DDX3X,MAGED1,IQSEC2,MECP2
GO:BP	localization	GO:0051179	0.026032	1682	312	PRKCZ,KCNAB2,PARK7,TMEM201,PIK3CD,RBP7,KIF1B,RCC2,EPHB2,STX12,RPA2,GRIK3,MACF1,RNF220,OSBPL9,LRP8,NDC1,CELSR2,SORT1,SLC6A17,LMNA,NOS1AP,DHX9,MAPKAPK2,PPP2R5A,RAB3GAP2,CNIH3,TRIM67,ACTN2,FMN2,SOX11,YWHAQ,DDX1,RAB10,CAD,SIX2,MSH2,SPTBN1,CCDC88A,CLASP1,IWS1,KIF5C,SCN3A,SCN1A,LRP2,GORASP2,TLK1,RAPGEF4,SESTD1,CALCRL,HSPD1,MAP2,CUL3,ATG16L1,AGAP1,PER2,HDLBP,RAF1,SLC4A7,CLASP2,PDCD6IP,SCN5A,SETD2,MAP4,ARIH2,GNAI2,CACNA1D,CACNA2D3,ERC2,SLMAP,CBLB,ADCY5,DNAJC13,TOPBP1,EIF4G1,OPA1,HTT,ADRA2C,NFKB1,ANK2,CAMK2D,ABCE1,ZNF827,PRLR,PDE8B

						,AP3B1,SCAMP1,APC,FBN2,RAPGEF6,KIF3A,HSPA4,HSPA9,NDUFA2,DIAPH1,NDFIP1,YIPF5,PDGFRB,CAMK2A,GRIA1,PDLIM7,E2F3,BRD2,SYNGAP1,DEF6,TFEB,TRAM2,DST,SMAP1,SNAP91,PTPRK,EPB41L2,STXBP5,TCP1,IGF2R,CYTH3,HNRNPA2B1,VWC2,HIP1,CACNA2D1,PCLO,CUX1,RELN,LRRC4,NUP205,SLC4A2,XPO7,DMTN,PTK2B,NRG1,FGFR1,LYN,MOS,CCNE2,YWHAZ,RIMS2,EXT1,KCNQ3,PTK2,ARC,FAM83H,NOL6,UBAP1,CLTA,DAPK1,WNK2,NR4A3,GAPVD1,GOLGA2,NUP188,RAPGEF1,ABCA2,CACNA1B,FRMD4A,ARHGAP21,ITGB1,ANK3,GLUD1,PTEN,LGI1,SLIT1,SUFU,BRSK2,STIM1,APBB1,SLC1A2,MAPK8IP1,LRP4,NR1H3,OSBP,AHNAK,RBM4,LRP5,ANO1,NUMA1,C1QTNF5,HEPACAM,WNK1,PTPN6,SLC2A13,ASIC1,SLC4A8,KRT18,LRP1,GRIP1,CCT2,PPFIA2,EEA1,HSP90B1,ATXN2,NCOR2,NBEA,AKAP11,TPT1,COG3,PCDH17,NDFIP2,RAP2A,AKAP6,PPM1A,SLC39A9,SYNJ2BP,TGFB3,TTC7B,HSP90AA1,TECPR2,CYFIP1,HERC2,APBA2,RYR3,EMC4,THBS1,SRP14,USP8,FOXB1,SNX1,SMAD3,AP3B2,MEF2A,RAB11FIP3,MAPK8IP3,TSC2,PKD1,CREBBP,GRIN2A,XPO6,VPS35,HERPUD1,DYNC1LI2,AP1G1,PLCG2,EMC8,BANP,PITPNA,MINK1,MYH10,ARHGAP44,FLCN,ALKBH5,MYO18A,GIT1,NF1,RHOT1,ASIC2,SYNRG,CACNB1,CASC3,KCNH4,STAT5B,STAT3,ATP6V0A1,NPEPPS,COL1A1,MTMR4,CLTC,TLK2,MRC2,SOX9,NUP85,SEC14L1,CYTH1,EIF4A3,THOC1,CHMP1B,MIB1,NEDD4L,MALT1,BCL2,MADCAM1,PALM,CSNK1G2,PIP5K1C,MAP2K2,C3,PIN1,PRKACA,EPS15L1,ANO8,FCHO1,HOMER3,ATP13A1,ACTN4,AKT2,GRIK5,POU2F2,LIN7B,PPFIA3,EPN1,PLCB1,SLC24A3,BCL2L1,ITCH,SRC,ARFGEF2,ATP9A,TIAM1,ITSN1,MORC3,MICAL3,PEX26,BCR,GGA1,PDGFB,SHANK3,GPR143,GLRA2,AP1S2,DMD,DDX3X,MAGED1,IQSEC2,MECP2
GO:BP	cell-substrate junction assembly	GO:0007044	0.026078	49	19	RCC2,MACF1,LAMC1,ACTN2,CLASP1,CLASP2,LIMCH1,DST,PTPRK,DMTN,PTK2B,PTK2,PTEN,ACTN1,THBS1,SMAD3,BCL2,SRC,BCR
GO:BP	actin filament-based process	GO:0030029	0.026078	320	76	GRHL3,RHOC,ARHGEF11,NOS1AP,CDC42BPA,ACTN2,FMN2,SPTBN1,CCDC88A,CLASP1,SCN3A,SCN1A,ARPC2,CUL3,CLASP2,PDCD6IP,SCN5A,NISCH,CACNA1D,MRAS,LIMCH1,PDGFRA,ANK2,CAMK2D,PTGER4,JMY,DIAPH1,PDGFRB,PDLIM7,EPB41L2,AUTS2,HIP1,CACNA2D1,PCLO,FLNC,CALD1,SLC4A2,DMTN,PTK2B,SPTAN1,ABI1,ITGB1,BRSK2,ARHGEF17,EPS8,LRP1,HSP90B1,AKAP11,RAP2A,SPTB,ACTN1,TGFB3,CYFIP1,TLN2,SMAD3,AKAP13,IQGAP1,MEF2A,MYH11,BCAR1,MINK1,MYH10,ARHGAP44,MYO18A,NF1,FMNL1,NEDD4L,BCL2,PIP5K1C,ACTN4,SRC,PHACTR3,MICAL3,BCR,SHANK3,IQSEC2
GO:BP	cellular macromolecule localization	GO:0070727	0.026171	951	189	PRKCZ,KCNAB2,PARK7,TMEM201,RCC2,EPHB2,STX12,RPA2,MACF1,NDC1,CELSR2,SORT1,LMNA,DHX9,PPP2R5A,RAB3GAP2,TRIM67,ACTN2,FMN2,YWHAQ,DDX1,RAB10,SIX2,MSH2,SPTBN1,CCDC88A,KIF5C,LRP2,GORASP2,TLK1,RAPGEF4,CALCRL,HSPD1,MAP2,ATG16L1,AGAP1,PER2,RAF1,CLASP2,PDCD6IP,SETD2,ARIH2,SLMAP,CBLB,ADCY5,DNAJC13,TOPBP1,EIF4G1,ADRA2C,ANK2,ZNF827,PDE8B,AP3B1,SCAMP1,APC,FBN2,RAPGEF6,KIF3A,HSPA4,HSPA9,DIAPH1,NDFIP1,YIPF5,E2F3,BRD2,SYNGAP1,TRAM2,SNAP91,PTPRK,EPB41L2,STXBP5,TCP1,VWC2,HIP1,PCLO,RELN,LRRC4,NUP205,XPO7,DMTN,CCNE2,YWHAZ,RIMS2,KCNQ3,FAM83H,UBAP1,CLTA,GAPVD1,GOLGA2,NUP188,ABCA2,FRMD4A,ITGB1,ANK3,GLUD1,LGI1,SUFU,BRSK2,APBB1,LRP4,NR1H3,OSBP,RBM4,LRP5,ANO1,NUMA1,C1QTNF5,HEPACAM,WNK1,SLC4A8,KRT18,LRP1,GRIP1,CCT2,HSP90B1,NBEA,AKAP11,COG3,NDFIP2,RAP2A,AKAP6,PPM1A,SYNJ2BP,TGFB3,TTC7B,HSP90AA1,TECPR2,HERC2,APBA2,EMC4,SRP14,USP8,SNX1,SMAD3,AP3B2,RAB11FIP3,MAPK8IP3,TSC2,PKD1,CREBBP,GRIN2A,XPO6,VPS35,HERPUD1,AP1G1,EMC8,BANP,MYH10,ARHGAP44,MYO18A,NF1,ASIC2,SYNRG,CASC3,STAT3,NPEPPS,COL1A1,CLTC,SOX9,NUP85,CHMP1B,NEDD4L,BCL2,MADCAM1,PALM,PIN1,PRKACA,HOMER3,ATP13A1,ACTN4,AKT2,LIN7B,PLCB1,BCL2L1,SRC,ARFGEF2,TIAM1,ITSN1,MORC3,PEX26,BCR,GGA1,SHANK3,AP1S2,DMD,DDX3X,MAGED1,IQSEC2,MECP2
GO:BP	establishment of localization	GO:0051234	0.026171	1450	273	PRKCZ,KCNAB2,PARK7,TMEM201,PIK3CD,RBP7,KIF1B,RCC2,EPHB2,STX12,GRIK3,MACF1,RNF220,OSBPL9,LRP8,NDC1,CELSR2,SORT1,SLC6A17,LMNA,NOS1AP,DHX9,MAPKAPK2,RAB3GAP2,CNIH3,ACTN2,FMN2,SOX11,YWHAQ,RAB10,CAD,SIX2,SPTBN1,CCDC88A,CLASP1,IWS1,KIF5C,SCN3A,SCN1A,LRP2,GORASP2,TLK1,RAPGEF4,SESTD1,CALCRL,HSPD1,MAP2,CUL3,ATG16L1,AGAP1,PER2,HDLBP,RAF1,SLC4A7,CLASP2,PDCD6IP,SCN5A,SETD2,MAP4,ARIH2,GNAI2,CACNA1D,CACNA2D3,ERC2,SLMAP,CBLB,ADCY5,DNAJC13,OPA1,HTT,ADRA2C,NFKB1,ANK2,CAMK2D,ABCE1,ZNF827,PRLR,PDE8B,AP3B1,SCAMP1,APC,KIF3A,HSPA4,HSPA9,NDUFA2,DIAPH1,NDFIP1,YIPF5,PDGFRB,CAMK2A,GRIA1,PDLIM7,E2F3,DEF6,TRAM2,DST,SMAP1,SNAP91,STXBP5,TCP1,IGF2R,CYTH3,HNRNPA2B1,HIP1,CACNA2D1,PCLO,CUX1,RELN,NUP205,SLC4A2,XPO7,DMTN,PTK2B,NRG1,FGFR1,LYN,MOS,YWHAZ,RIMS2,EXT1,KCNQ3,PTK2,ARC,NOL6,UBAP1,CLTA,DAPK1,WNK2,NR4A3,GAPVD1,GOLGA2,NUP188,RAPGEF1,ABCA2,CACNA1B,FRMD4A,ARHGAP21,ITGB1,ANK3,GLUD1,PTEN,SLIT1,SUFU,BRSK2,STIM1,APBB1,SLC1A2,MAPK8IP1,LRP4,NR1H3,OSBP,AHNAK,RBM4,LRP5,ANO1,NUMA1,C1QTNF5,WNK1,PTPN6,SLC2A13,ASIC1,SLC4A8,KRT18,LRP1,GRIP1,PPFIA2,EEA1,HSP90B1,ATXN2,NCOR2,TPT1,COG3,PCDH17,NDFIP2,RAP2A,AKAP6,PPM1A,SLC39A9,SYNJ2BP,TGFB3,HSP90AA1,TECPR2,CYFIP1,HERC2,APBA2,RYR3,EMC4,THBS1,SRP14,FOXB1,SNX1,SMAD3,AP3B2,MEF2A,RAB11FIP3,MAPK8IP3,TSC2,PKD1,GRIN2A,XPO6,VPS35,HERPUD1,AP1G1,PLCG2,EMC8,PITPNA,MINK1,MYH10,ARHGAP44,ALKBH5,MYO18A,GIT1,NF1,RHOT1,ASIC2,SYNRG,CACNB1,CASC3,KCNH4,STAT5B,STAT3,ATP6V0A1,NPEPPS,COL1A1,MTMR4,CLTC,MRC2,NUP85,SEC14L1,CYTH1,EIF4A3,THOC1,CHMP1B,MIB1,NEDD4L,MALT1,BCL2,CSNK1G2,PIP5K1C,MAP2K2,C3,PRKACA,EPS15L1,ANO8,FCHO1,HOMER3,ATP13A1,ACTN4,AKT2,GRIK5,POU2F2,LIN7B,PPFIA3,EPN1,PLCB1,SLC24A3,BCL2L1,ITCH,SRC,ARFGEF2,ATP9A,TIAM1,ITSN1,MICAL3,PEX26,BCR,GGA1,PDGFB,GPR143,GLRA2,AP1S2,DMD,MECP2
GO:BP	regulation of transporter activity	GO:0032409	0.026171	78	26	EPHB2,NOS1AP,CNIH3,ACTN2,CACNA1D,SLMAP,HTT,ANK2,CAMK2D,NDFIP1,RELN,ARC,DAPK1,WNK2,ITGB1,ANK3,STIM1,NR1H3,AHNAK,NDFIP2,AKAP6,MINK1,NEDD4L,BCL2,ACTN4,DMD
GO:BP	intracellular	GO:0035556	0.027256	1078	210	PRKCZ,SKI,PARK7,PIK3CD,RAP1GAP,EPHB2,RPA2,ARHGAP29,CSF1,RHOC,NOTCH2,ASH1L,ARHGEF11,DDR2,RC3H1,RGL1,PIK3C2

	signal transduction					B,MAPKAPK2,TRIM67,FMN2,SOX11,YWHAQ,DDX1,MSH2,SPTBN1,CCDC88A,CYP26B1,LRP2,TLK1,RAPGEF4,CUL3,SRGAP3,RAF1,NR2C2,GNAI2,NISCH,SLMAP,FOXP1,CBLB,ADCY5,TOPBP1,MRAS,MECOM,OPA1,HTT,ADRA2C,PDGFRA,RASGEF1B,MAPK10,NFKB1,ANK2,CAMK2D,ADCY2,TRIO,PTGER4,MAP3K1,PDE8B,JMY,APC,RAPGEF6,FNIP1,NDFIP1,PDGFRB,GRIA1,RANBP9,SYNGAP1,DEF6,UBR2,RUNX2,CYTH3,AHR,TBX20,AUTS2,HIP1,HGF,COL1A2,RELN,SRPK2,PTK2B,DUSP4,NRG1,FGFR1,KAT6A,PRKDC,LYN,MOS,GDF6,YWHAZ,TRIB1,PTK2,RORB,TLE1,DAPK1,SEMA4D,WNK2,NR4A3,SCAI,RAPGEF1,TRAF2,ARHGAP21,ITGB1,BMPR1A,PTEN,NFKB2,EIF3A,BRSK2,DUSP8,MAPK8IP1,LRP4,NR1H3,GPR137,ARHGEF17,GRM5,WNK1,PTPN6,EPS8,STK38L,HDAC7,RARG,LRP1,GRIP1,KSR2,NCOR2,AKAP11,TPT1,NDFIP2,RAP2A,MYO16,ARHGAP5,AKAP6,MAP4K5,PPM1A,SYNJ2BP,TGFB3,TRAF3,PPP1R13B,CYFIP1,RYR3,THBS1,USP8,SMAD3,PARP6,AKAP13,IQGAP1,NR2F2,MEF2A,MAPK8IP3,TSC2,CREBBP,CDIP1,GRIN2A,PAGR1,MMP2,HERPUD1,PLCG2,USP10,RAP1GAP2,MINK1,RPL26,ARHGAP44,NCOR1,FLCN,GIT1,NF1,RHOT1,STAT3,RUNDC3A,TLK2,MAP3K3,ERN1,DDX5,SOX9,SEC14L1,SOCS3,CYTH1,LDLRAD4,MALT1,BCL2,PTBP1,PIP5K1C,MAP2K2,SAFB2,PIN1,PDE4A,SMARCA4,PRKACA,BABAM1,HOMER3,ZNF536,ACTN4,AKT2,PPP5C,PLCB1,BCL2L1,ITCH,SRC,NCOA3,ARFGEF2,TIAM1,ITSN1,PI4KA,BCR,PDGFB,GRAMD4,SHANK3,GPR143,DMD,DDX3X,KDM6A,IQSEC2
GO:BP	cytoskeleton organization	GO:0007010	0.027256	523	113	PRKCZ,GRHL3,MAP7D1,MACF1,RHOC,LMNA,ARHGEF11,NOS1AP,CDC42BPA,ACTN2,FMN2,SPTBN1,CCDC88A,CLASP1,PRPF40A,MAP2,ARPC2,CUL3,RAF1,CLASP2,PDCD6IP,SETD2,MAP4,NISCH,ATXN7,MRAS,HTT,LIMCH1,PDGFRA,ANK2,PTGER4,JMY,APC,KIF3A,DIAPH1,PDGFRB,PDLIM7,RANBP9,CUL9,DST,EPB41L2,AUTS2,HIP1,PCLO,ACTL6B,FLNC,CALD1,SLC4A2,DMTN,PTK2B,MOS,PTK2,ARC,FAM83H,GOLGA2,SPTAN1,TUBB4B,ABI1,ITGB1,ANK3,CCSER2,PTEN,BRSK2,NUMA1,ARHGEF17,EPS8,NCKAP5L,KRT18,LRP1,NAV3,HSP90B1,KATNAL1,AKAP11,SLAIN1,RAP2A,SPTB,ACTN1,TGFB3,CYFIP1,TLN2,SMAD3,AKAP13,IQGAP1,MEF2A,PKD1,MYH11,DYNC1LI2,BCAR1,MINK1,MYH10,ARHGAP44,NCOR1,MYO18A,GIT1,NF1,FMNL1,CLTC,SOX9,CHMP1B,BCL2,PALM,PIP5K1C,ACTN4,TPX2,SRC,PHACTR3,BRWD1,MICAL3,BCR,SHANK3,DMD,IQSEC2,MECP2
GO:BP	actin cytoskeleton organization	GO:0030036	0.027256	282	68	GRHL3,RHOC,ARHGEF11,NOS1AP,CDC42BPA,ACTN2,FMN2,SPTBN1,CCDC88A,CLASP1,ARPC2,CUL3,CLASP2,PDCD6IP,NISCH,MRAS,LIMCH1,PDGFRA,PTGER4,JMY,DIAPH1,PDGFRB,PDLIM7,EPB41L2,AUTS2,HIP1,PCLO,FLNC,CALD1,SLC4A2,DMTN,PTK2B,SPTAN1,ABI1,ITGB1,BRSK2,ARHGEF17,EPS8,LRP1,HSP90B1,AKAP11,RAP2A,SPTB,ACTN1,TGFB3,CYFIP1,TLN2,SMAD3,AKAP13,IQGAP1,MEF2A,MYH11,BCAR1,MINK1,MYH10,ARHGAP44,MYO18A,NF1,FMNL1,BCL2,PIP5K1C,ACTN4,SRC,PHACTR3,MICAL3,BCR,SHANK3,IQSEC2
GO:BP	enzyme-linked receptor protein signaling pathway	GO:0007167	0.027256	413	93	PRKCZ,SKI,PIK3CD,EPHB2,SORT1,CSF1,NOTCH2,DDR2,MAPKAPK2,SOX11,SPTBN1,CCDC88A,MBD5,LRP2,NCL,RAF1,GNAI2,EPHA6,CBLB,PDGFRA,BMPR1B,HHIP,TRIO,PRLR,APC,FBN2,PDGFRB,SCUBE3,RUNX2,PTPRK,IGF2R,TBX20,VWC2,HIP1,HGF,COL1A2,PTK2B,NRG1,FGFR1,LYN,GDF6,EXT1,PTK2,PTPRD,ZFAND5,NR4A3,SVEP1,RABGAP1,RAPGEF1,ABI1,ZEB1,ITGB1,BMPR1A,CDKN1C,LRP4,LTBP3,RBM4,NUMA1,STRAP,LRP1,TSC22D1,PPM1A,TGFB3,CYFIP1,THBS1,SMAD3,IQGAP1,TSC2,CREBBP,MMP2,GLG1,BCAR1,FLCN,GIT1,STAT5B,STAT3,COL1A1,MTMR4,DDX5,SOX9,SOCS3,LDLRAD4,SKOR2,MAP2K2,PIN1,AKT2,MEGF8,PLCB1,SRC,TIAM1,PDGFB,COL4A6,IRS4
GO:BP	transport	GO:0006810	0.027256	1363	258	PRKCZ,KCNAB2,PARK7,TMEM201,PIK3CD,RBP7,KIF1B,EPHB2,STX12,GRIK3,MACF1,RNF220,OSBPL9,LRP8,NDC1,CELSR2,SORT1,SLC6A17,LMNA,NOS1AP,DHX9,MAPKAPK2,RAB3GAP2,CNIH3,ACTN2,FMN2,SOX11,YWHAQ,RAB10,CAD,SIX2,SPTBN1,CCDC88A,CLASP1,IWS1,KIF5C,SCN3A,SCN1A,LRP2,TLK1,RAPGEF4,SESTD1,CALCRL,HSPD1,MAP2,CUL3,ATG16L1,AGAP1,PER2,HDLBP,RAF1,SLC4A7,CLASP2,PDCD6IP,SCN5A,SETD2,GNAI2,CACNA1D,CACNA2D3,ERC2,SLMAP,CBLB,ADCY5,DNAJC13,OPA1,HTT,ADRA2C,NFKB1,ANK2,CAMK2D,ABCE1,PRLR,PDE8B,AP3B1,SCAMP1,KIF3A,HSPA4,HSPA9,NDUFA2,DIAPH1,NDFIP1,YIPF5,PDGFRB,CAMK2A,GRIA1,PDLIM7,E2F3,DEF6,TRAM2,DST,SMAP1,SNAP91,STXBP5,IGF2R,CYTH3,HNRNPA2B1,HIP1,CACNA2D1,PCLO,CUX1,RELN,NUP205,SLC4A2,XPO7,PTK2B,NRG1,FGFR1,LYN,RIMS2,EXT1,KCNQ3,PTK2,ARC,NOL6,UBAP1,CLTA,DAPK1,WNK2,NR4A3,GAPVD1,GOLGA2,NUP188,RAPGEF1,ABCA2,CACNA1B,FRMD4A,ARHGAP21,ITGB1,ANK3,GLUD1,PTEN,SLIT1,SUFU,BRSK2,STIM1,APBB1,SLC1A2,MAPK8IP1,LRP4,NR1H3,OSBP,AHNAK,RBM4,LRP5,ANO1,NUMA1,C1QTNF5,WNK1,PTPN6,SLC2A13,ASIC1,SLC4A8,KRT18,LRP1,GRIP1,PPFIA2,EEA1,HSP90B1,ATXN2,NCOR2,TPT1,COG3,PCDH17,NDFIP2,AKAP6,PPM1A,SLC39A9,SYNJ2BP,TGFB3,HSP90AA1,TECPR2,CYFIP1,HERC2,APBA2,RYR3,THBS1,FOXB1,SNX1,SMAD3,AP3B2,MEF2A,RAB11FIP3,MAPK8IP3,TSC2,PKD1,GRIN2A,XPO6,VPS35,HERPUD1,AP1G1,PLCG2,PITPNA,MINK1,MYH10,ARHGAP44,ALKBH5,MYO18A,GIT1,NF1,RHOT1,ASIC2,SYNRG,CACNB1,CASC3,KCNH4,STAT5B,STAT3,ATP6V0A1,COL1A1,MTMR4,CLTC,MRC2,NUP85,SEC14L1,CYTH1,EIF4A3,THOC1,CHMP1B,MIB1,NEDD4L,MALT1,BCL2,CSNK1G2,PIP5K1C,MAP2K2,C3,PRKACA,EPS15L1,ANO8,FCHO1,HOMER3,ATP13A1,ACTN4,AKT2,GRIK5,POU2F2,LIN7B,PPFIA3,EPN1,PLCB1,SLC24A3,BCL2L1,ITCH,SRC,ARFGEF2,ATP9A,TIAM1,ITSN1,MICAL3,PEX26,BCR,GGA1,PDGFB,GPR143,GLRA2,AP1S2,DMD,MECP2
GO:BP	regulation of response to stimulus	GO:0048583	0.027956	1419	267	PRKCZ,SKI,PARK7,PIK3CD,RAP1GAP,EPHB2,RPA2,EYA3,MACF1,RNF220,FAF1,LRP8,ARHGAP29,CSF1,RHOC,NOTCH2,ASH1L,UBQLN4,LMNA,ARHGEF11,DDR2,RC3H1,DHX9,LAMC1,MAPKAPK2,CNIH3,TRIM67,FMN2,SOX11,DDX1,MSH2,SPTBN1,CCDC88A,CYP26B1,CLASP1,MBD5,RIF1,LRP2,HSPD1,CUL3,ITM2C,NCL,SRGAP3,RAF1,CLASP2,SCN5A,SETD2,USP19,GNAI2,SLMAP,ATXN7,FOXP1,ROBO2,CBLB,MECOM,EIF4G1,OPA1,HTT,ADRA2C,PDGFRA,MAPK10,BMPR1B,NFKB1,CAMK2D,JADE1,HHIP,TRIO,PRLR,PTGER4,MAP3K1,PDE8B,AP3B1,APC,FBN2,FNIP1,NDFIP1,PDGFRB,CAMK2A,GRIA1,SLIT3,RANBP9,AGPAT1,BRD2,SYNGAP1,SCUBE3,DEF6,UBR2,RUNX2,ARID1B,CYTH3,AHR,TBX20,CDK13,HECW1,VWC2,AUTS2,HIP1,HGF,ACTL6B,RELN,PLXNA4,DMTN,PTK2B,DUSP4,NRG1,FGFR1,KAT6A,PRKDC,SOX17,LYN,MOS,GDF6,YWHAZ,RIMS2,EXT1,TRIB1,PTK2,ARC,PTPRD,MLLT3,TLE1,DAPK1,SEMA4D,WNK2,NR4A3,SVEP1,RABGAP1,SCAI,RAPGEF1,TRAF2,ARHGAP21,ZEB1,ITGB1,BMPR1A,PTEN,SLIT1,SUFU,EIF3A,GRK5,DUSP8,CDKN1C,MAPK8IP1,LRP4,NR1H3,GPR137,RBM4,NUMA1,ARHGEF17,GRM5,WNK1,PTPN6,EPS8,STRAP,HDAC7,SMARCC2,STAT2,LRP1,CUX2,KS

						R2,NCOR2,EP400,TSC22D1,TPT1,NDFIP2,RAP2A,ARHGAP5,AKAP6,PPM1A,ZBTB1,SLC39A9,SYNJ2BP,TGFB3,HSP90AA1,TRAF3,CYFIP1,HERC2,THBS1,USP8,SMAD3,TLE3,AKAP13,IQGAP1,CRTC3,MEF2A,MAPK8IP3,TSC2,CREBBP,GRIN2A,PAGR1,VPS35,BRD7,MMP2,HERPUD1,AP1G1,GLG1,BCAR1,PLCG2,USP10,RAP1GAP2,MINK1,TNFSF13,RPL26,ARHGAP44,NCOR1,FLCN,GIT1,NF1,STAT5B,STAT3,RUNDC3A,COL1A1,MTMR4,MAP3K3,ERN1,DDX5,SOX9,SEC14L1,SOCS3,CYTH1,EIF4A3,LDLRAD4,SKOR2,MALT1,BCL2,PALM,PTBP1,CSNK1G2,PIP5K1C,MAP2K2,SAFB2,C3,PIN1,PDE4A,SMARCA4,PRKACA,BABAM1,FCHO1,HOMER3,ZNF536,ACTN4,MEGF8,PPP5C,PLCB1,JAG1,BCL2L1,CBFA2T2,ITCH,GGT7,SRC,NCOA3,ARFGEF2,SPATA2,USP25,TIAM1,ITSN1,BCR,PDGFB,XRCC6,SCUBE1,GRAMD4,SHANK3,GPR143,DMD,DDX3X,KDM6A,MAGED1,IQSEC2
GO:BP	establishment of localization in cell	GO:0051649	0.028571	732	150	PRKCZ,PARK7,TMEM201,PIK3CD,KIF1B,RCC2,STX12,RNF220,NDC1,SORT1,LMNA,DHX9,RAB3GAP2,ACTN2,FMN2,RAB10,SIX2,CCDC88A,CLASP1,IWS1,KIF5C,SCN1A,TLK1,RAPGEF4,MAP2,CUL3,ATG16L1,PER2,RAF1,CLASP2,PDCD6IP,SETD2,MAP4,ERC2,CBLB,ADCY5,DNAJC13,OPA1,HTT,ABCE1,PDE8B,AP3B1,SCAMP1,APC,KIF3A,HSPA4,HSPA9,NDFIP1,YIPF5,CAMK2A,E2F3,TRAM2,DST,SNAP91,STXBP5,IGF2R,HNRNPA2B1,HIP1,PCLO,NUP205,XPO7,NRG1,LYN,MOS,YWHAZ,RIMS2,KCNQ3,ARC,NOL6,UBAP1,CLTA,NR4A3,GOLGA2,NUP188,ABCA2,ARHGAP21,ITGB1,PTEN,SLIT1,SUFU,BRSK2,SLC1A2,OSBP,RBM4,LRP5,ANO1,NUMA1,WNK1,ASIC1,SLC4A8,LRP1,GRIP1,PPFIA2,EEA1,HSP90B1,COG3,PCDH17,NDFIP2,PPM1A,HSP90AA1,TECPR2,CYFIP1,HERC2,SNX1,SMAD3,AP3B2,RAB11FIP3,MAPK8IP3,TSC2,PKD1,XPO6,VPS35,HERPUD1,AP1G1,ARHGAP44,ALKBH5,GIT1,NF1,RHOT1,ASIC2,SYNRG,CASC3,STAT3,ATP6V0A1,MTMR4,CLTC,NUP85,EIF4A3,THOC1,CHMP1B,MALT1,BCL2,PIP5K1C,MAP2K2,PRKACA,EPS15L1,ACTN4,GRIK5,LIN7B,PPFIA3,SRC,ATP9A,TIAM1,ITSN1,PEX26,BCR,GGA1,GPR143,AP1S2,MECP2
GO:BP	regulation of small GTPase mediated signal transduction	GO:0051056	0.02911	139	39	RAP1GAP,EPHB2,ARHGAP29,CSF1,NOTCH2,ARHGEF11,TRIM67,CUL3,SRGAP3,RAF1,TRIO,SYNGAP1,DEF6,CYTH3,AUTS2,RELN,SEMA4D,SCAI,RAPGEF1,ARHGAP21,ITGB1,LRP4,ARHGEF17,EPS8,ARHGAP5,AKAP13,TSC2,RAP1GAP2,ARHGAP44,FLCN,GIT1,NF1,CYTH1,SRC,ARFGEF2,TIAM1,ITSN1,BCR,IQSEC2
GO:BP	vesicle-mediated transport	GO:0016192	0.034257	544	116	PARK7,PIK3CD,KIF1B,EPHB2,STX12,MACF1,RNF220,LRP8,SORT1,MAPKAPK2,CNIH3,ACTN2,FMN2,RAB10,SPTBN1,CLASP1,LRP2,RAPGEF4,CALCRL,CUL3,CLASP2,PDCD6IP,GNAI2,ERC2,CBLB,DNAJC13,HTT,ANK2,AP3B1,SCAMP1,KIF3A,YIPF5,GRIA1,PDLIM7,DEF6,SMAP1,SNAP91,STXBP5,IGF2R,CYTH3,HIP1,PCLO,CUX1,NRG1,LYN,RIMS2,KCNQ3,PTK2,ARC,UBAP1,CLTA,NR4A3,GAPVD1,GOLGA2,RAPGEF1,ABCA2,ITGB1,ANK3,PTEN,BRSK2,MAPK8IP1,LRP4,NR1H3,LRP5,SLC4A8,KRT18,LRP1,GRIP1,PPFIA2,EEA1,ATXN2,COG3,PCDH17,SYNJ2BP,CYFIP1,THBS1,SNX1,AP3B2,RAB11FIP3,MAPK8IP3,TSC2,VPS35,AP1G1,PLCG2,ARHGAP44,MYO18A,GIT1,SYNRG,ATP6V0A1,MTMR4,CLTC,MRC2,CYTH1,CHMP1B,MIB1,NEDD4L,CSNK1G2,PIP5K1C,MAP2K2,C3,EPS15L1,FCHO1,GRIK5,LIN7B,PPFIA3,EPN1,BCL2L1,ITCH,SRC,ARFGEF2,ATP9A,ITSN1,MICAL3,BCR,GGA1,AP1S2
GO:BP	macromolecule localization	GO:0033036	0.036672	1062	206	PRKCZ,KCNAB2,PARK7,TMEM201,RBP7,RCC2,EPHB2,STX12,RPA2,MACF1,OSBPL9,NDC1,CELSR2,SORT1,LMNA,DHX9,PPP2R5A,RAB3GAP2,TRIM67,ACTN2,FMN2,YWHAQ,DDX1,RAB10,SIX2,MSH2,SPTBN1,CCDC88A,IWS1,KIF5C,LRP2,GORASP2,TLK1,RAPGEF4,CALCRL,HSPD1,MAP2,ATG16L1,AGAP1,PER2,HDLBP,RAF1,CLASP2,PDCD6IP,SETD2,ARIH2,SLMAP,CBLB,ADCY5,DNAJC13,TOPBP1,EIF4G1,ADRA2C,NFKB1,ANK2,ZNF827,PDE8B,AP3B1,SCAMP1,APC,FBN2,RAPGEF6,KIF3A,HSPA4,HSPA9,DIAPH1,NDFIP1,YIPF5,E2F3,BRD2,SYNGAP1,TRAM2,SNAP91,PTPRK,EPB41L2,STXBP5,TCP1,HNRNPA2B1,VWC2,HIP1,PCLO,RELN,LRRC4,NUP205,XPO7,DMTN,LYN,CCNE2,YWHAZ,RIMS2,KCNQ3,ARC,FAM83H,NOL6,UBAP1,CLTA,GAPVD1,GOLGA2,NUP188,ABCA2,FRMD4A,ITGB1,ANK3,GLUD1,LGI1,SUFU,BRSK2,APBB1,LRP4,NR1H3,OSBP,RBM4,LRP5,ANO1,NUMA1,C1QTNF5,HEPACAM,WNK1,SLC4A8,KRT18,LRP1,GRIP1,CCT2,HSP90B1,ATXN2,NBEA,AKAP11,COG3,NDFIP2,RAP2A,AKAP6,PPM1A,SYNJ2BP,TGFB3,TTC7B,HSP90AA1,TECPR2,HERC2,APBA2,EMC4,THBS1,SRP14,USP8,SNX1,SMAD3,AP3B2,RAB11FIP3,MAPK8IP3,TSC2,PKD1,CREBBP,GRIN2A,XPO6,VPS35,HERPUD1,AP1G1,EMC8,BANP,PITPNA,MYH10,ARHGAP44,ALKBH5,MYO18A,NF1,ASIC2,SYNRG,CASC3,STAT3,NPEPPS,COL1A1,CLTC,SOX9,NUP85,EIF4A3,THOC1,CHMP1B,NEDD4L,BCL2,MADCAM1,PALM,C3,PIN1,PRKACA,HOMER3,ATP13A1,ACTN4,AKT2,LIN7B,PLCB1,BCL2L1,SRC,ARFGEF2,ATP9A,TIAM1,ITSN1,MORC3,PEX26,BCR,GGA1,SHANK3,AP1S2,DMD,DDX3X,MAGED1,IQSEC2,MECP2
GO:BP	cell-substrate adhesion	GO:0031589	0.038354	127	36	PRKCZ,RCC2,MACF1,CSF1,LAMC1,ACTN2,CLASP1,ARPC2,SNED1,CLASP2,LIMCH1,PTPRK,VWC2,DMTN,PTK2B,PTK2,SVEP1,ITGB1,PTEN,PPFIA2,ACTN1,THBS1,SMAD3,PKD1,MINK1,NF1,COL1A1,BCL2,MADCAM1,ACTN4,JAG1,SRC,TIAM1,BCR,PDGFB,DMD
GO:CC	cell periphery	GO:0071944	3.10E-08	1519	318	PRKCZ,KCNAB2,PARK7,TMEM201,PIK3CD,KAZN,RCC2,EPHB2,GRIK3,MACF1,FAF1,LRP8,NDC1,COL11A1,CELSR2,SORT1,CSF1,SLC6A17,RHOC,NOTCH2,ARHGEF11,NOS1AP,DDR2,LAMC1,RGL1,PIK3C2B,NFASC,PPP2R5A,RAB3GAP2,CNIH3,ACTN2,FMN2,SOX11,ASAP2,RAB10,SPTBN1,CCDC88A,INPP4A,CLASP1,RIF1,SCN3A,SCN1A,LRP2,RAPGEF4,CALCRL,HSPD1,ADAM23,ARPC2,CUL3,ITM2C,NCL,SNED1,HDLBP,LRRN1,RAF1,SLC4A7,CLASP2,PDCD6IP,SCN5A,PTH1R,MAP4,CELSR3,GNAI2,NISCH,CACNA1D,CACNA2D3,ERC2,SLMAP,ROBO2,EPHA6,CBLB,LSAMP,ADCY5,PLXNA1,PODXL2,DNAJC13,TOPBP1,MRAS,ADRA2C,PDS5A,PDGFRA,RASGEF1B,MAPK10,BMPR1B,ANK2,CAMK2D,JADE1,PCDH10,HHIP,ADCY2,CDH6,PDZD2,PRLR,PTGER4,SCAMP1,APC,FBN2,RAPGEF6,PCDHGC4,DIAPH1,NDFIP1,PDGFRB,DCTN4,SPARC,GRIA1,ADAMTS2,RANBP9,SYNGAP1,SCUBE3,DEF6,DST,SMAP1,COL12A1,SNAP91,PTPRK,EPB41L2,STXBP5,ARID1B,IGF2R,THBS2,ELFN1,CYTH3,VWC2,HIP1,CACNA2D1,PCLO,GRM3,COL1A2,RELN,LRRC4,FLNC,PLXNA4,CALD1,KIAA1549,SLC4A2,DMTN,PTK2B,NRG1,FGFR1,LYN,RIMS2,KCNQ3,PTK2,ARC,PTPRD,UBAP1,CLTA,DAPK1,SEMA4D,WNK2,FAM120A,COL27A1,RABGAP1,GAPVD1,SPTAN1,RAPGEF1,COL5A1,TRAF2,ABCA2,CACNA1B,SEPHS1,ARHGAP21,ITGB1,ANK3,BMPR1A,PTEN,SLIT1,GRK5,STIM1,APBB1,SLC1A2,MAPK8IP1,LRP4,OSBP,AHNAK,LTBP3,LRP5,PPP6R3,ANO1,NUMA1,TENM4,GRM5,PAFAH1B2,C1QTNF5,HEPACAM,IGSF9B,TSPAN9,PTPN6,EPS8,SLC2A13,ASIC1,SLC4A8,KRT18,STAT2,LRP1,TBC1D30,GRIP1,PTPRB,PPF

						IA2,HSP90B1,TMEM119,KSR2,FRY,NBEA,AKAP11,TSC22D1,COG3,PCDH17,PCDH20,PCDH9,RAP2A,MYO16,ACIN1,ARHGAP5,AKAP6,SSTR1,MAP4K5,PPM1A,HSPA2,SPTB,ACTN1,SLC39A9,TGFB3,TTC7B,HSP90AA1,TRAF3,PPP1R13B,HERC2,APBA2,RYR3,THBS1,USP8,TLN2,SMAD3,PKM,LOXL1,AKAP13,ACAN,IQGAP1,RAB11FIP3,MAPK8IP3,PKD1,GRIN2A,XPO6,MMP2,GLG1,BCAR1,PLCG2,RAP1GAP2,MYH10,ARHGAP44,FLCN,NF1,ASIC2,CACNB1,KCNH4,STAT3,ATP6V0A1,FZD2,FMNL1,COL1A1,CLTC,SOCS3,CYTH1,PTPRM,CHMP1B,MIB1,DTNA,NEDD4L,MADCAM1,PALM,CSNK1G2,PIP5K1C,MAP2K2,C3,ADAMTS10,PDE4A,PRKACA,EPS15L1,ANO8,FCHO1,HOMER3,ACTN4,AKT2,GRIK5,SYMPK,PPP5C,LIN7B,PPFIA3,FLT3LG,PTOV1,EPN1,ATRN,JAG1,SLC24A3,ITCH,GGT7,SRC,ATP9A,TIAM1,ITSN1,BRWD1,MICAL3,PI4KA,BCR,GAL3ST1,TIMP3,PDGFB,SCUBE1,PLXNB2,SHANK3,GPR143,GLRA2,DMD,DDX3X,MAGED1,COL4A6,IRS4
GO:CC	plasma membrane	GO:0005886	5.14E-06	1420	290	PRKCZ,KCNAB2,PARK7,PIK3CD,KAZN,RCC2,EPHB2,GRIK3,MACF1,FAF1,LRP8,NDC1,CELSR2,SORT1,CSF1,SLC6A17,RHOC,NOTCH2,ARHGEF11,NOS1AP,DDR2,RGL1,PIK3C2B,NFASC,PPP2R5A,RAB3GAP2,CNIH3,ACTN2,FMN2,SOX11,ASAP2,RAB10,SPTBN1,CCDC88A,INPP4A,RIF1,SCN3A,SCN1A,LRP2,RAPGEF4,CALCRL,HSPD1,ADAM23,ARPC2,CUL3,ITM2C,NCL,HDLBP,RAF1,SLC4A7,CLASP2,PDCD6IP,SCN5A,PTH1R,MAP4,CELSR3,GNAI2,NISCH,CACNA1D,CACNA2D3,ERC2,SLMAP,ROBO2,EPHA6,CBLB,LSAMP,ADCY5,PLXNA1,PODXL2,DNAJC13,TOPBP1,MRAS,ADRA2C,PDS5A,PDGFRA,RASGEF1B,MAPK10,BMPR1B,ANK2,CAMK2D,JADE1,PCDH10,HHIP,ADCY2,CDH6,PDZD2,PRLR,PTGER4,SCAMP1,APC,RAPGEF6,PCDHGC4,DIAPH1,PDGFRB,SPARC,GRIA1,RANBP9,SYNGAP1,SCUBE3,DEF6,DST,SMAP1,SNAP91,PTPRK,EPB41L2,STXBP5,ARID1B,IGF2R,ELFN1,CYTH3,VWC2,HIP1,CACNA2D1,GRM3,RELN,LRRC4,FLNC,PLXNA4,CALD1,KIAA1549,SLC4A2,DMTN,PTK2B,NRG1,FGFR1,LYN,RIMS2,KCNQ3,PTK2,ARC,PTPRD,UBAP1,CLTA,DAPK1,SEMA4D,WNK2,FAM120A,RABGAP1,GAPVD1,SPTAN1,RAPGEF1,TRAF2,ABCA2,CACNA1B,SEPHS1,ARHGAP21,ITGB1,ANK3,BMPR1A,PTEN,SLIT1,GRK5,STIM1,APBB1,SLC1A2,MAPK8IP1,LRP4,OSBP,AHNAK,LRP5,PPP6R3,ANO1,NUMA1,TENM4,GRM5,PAFAH1B2,C1QTNF5,HEPACAM,IGSF9B,TSPAN9,PTPN6,EPS8,SLC2A13,ASIC1,SLC4A8,STAT2,LRP1,TBC1D30,GRIP1,PTPRB,PPFIA2,HSP90B1,TMEM119,KSR2,NBEA,AKAP11,TSC22D1,COG3,PCDH17,PCDH20,PCDH9,RAP2A,MYO16,ACIN1,ARHGAP5,AKAP6,SSTR1,MAP4K5,PPM1A,HSPA2,SPTB,ACTN1,SLC39A9,TGFB3,TTC7B,HSP90AA1,TRAF3,PPP1R13B,HERC2,APBA2,RYR3,THBS1,USP8,TLN2,SMAD3,IQGAP1,RAB11FIP3,MAPK8IP3,PKD1,GRIN2A,XPO6,MMP2,GLG1,BCAR1,PLCG2,RAP1GAP2,MYH10,ARHGAP44,FLCN,NF1,ASIC2,CACNB1,KCNH4,STAT3,ATP6V0A1,FZD2,FMNL1,CLTC,SOCS3,CYTH1,PTPRM,CHMP1B,MIB1,DTNA,NEDD4L,MADCAM1,PALM,CSNK1G2,PIP5K1C,MAP2K2,C3,PDE4A,PRKACA,EPS15L1,ANO8,FCHO1,HOMER3,ACTN4,AKT2,GRIK5,SYMPK,PPP5C,LIN7B,FLT3LG,PTOV1,EPN1,ATRN,JAG1,SLC24A3,ITCH,GGT7,SRC,ATP9A,TIAM1,ITSN1,BRWD1,MICAL3,PI4KA,BCR,GAL3ST1,PDGFB,SCUBE1,PLXNB2,SHANK3,GPR143,GLRA2,DMD,DDX3X,MAGED1,IRS4
GO:CC	supramolecular polymer	GO:0099081	0.002285	311	78	KCNAB2,KIF1B,RCC2,IFFO2,MACF1,COL11A1,LMNA,NOS1AP,PPP2R5A,ACTN2,FMN2,KIF26B,SPTBN1,DNAH6,CLASP1,KIF5C,SCN1A,MAP2,CUL3,CLASP2,SCN5A,MAP4,CACNA1D,SLMAP,OPA1,ANK2,MTMR12,APC,FBN2,KIF3A,DIAPH1,DCTN4,PDLIM7,DST,TCP1,COL1A2,FLNC,CALD1,DMTN,FAM83H,COL27A1,GOLGA2,COL5A1,TUBB4B,ANK3,EIF3A,STIM1,AHNAK,NUMA1,NCKAP5L,KRT18,CCT2,NAV3,KATNAL1,TPT1,SLAIN1,ACTN1,RYR3,IQGAP1,MYH11,MMP2,DYNC1LI2,MYH10,MYO18A,COL1A1,CLTC,TLK2,CHMP1B,MAP2K2,DPP9,ADAMTS10,ACTN4,TPX2,SRC,ARFGEF2,TIAM1,DMD,COL4A6
GO:CC	supramolecular fiber	GO:0099512	0.002285	308	77	KCNAB2,KIF1B,RCC2,IFFO2,MACF1,COL11A1,LMNA,NOS1AP,PPP2R5A,ACTN2,FMN2,KIF26B,SPTBN1,DNAH6,CLASP1,KIF5C,SCN1A,MAP2,CUL3,CLASP2,SCN5A,MAP4,CACNA1D,SLMAP,OPA1,ANK2,MTMR12,APC,FBN2,KIF3A,DIAPH1,DCTN4,PDLIM7,DST,TCP1,COL1A2,FLNC,CALD1,DMTN,FAM83H,COL27A1,GOLGA2,COL5A1,TUBB4B,ANK3,EIF3A,STIM1,AHNAK,NUMA1,NCKAP5L,KRT18,CCT2,NAV3,KATNAL1,TPT1,SLAIN1,ACTN1,RYR3,IQGAP1,MYH11,MMP2,DYNC1LI2,MYH10,MYO18A,COL1A1,CLTC,TLK2,CHMP1B,MAP2K2,DPP9,ADAMTS10,ACTN4,TPX2,SRC,ARFGEF2,TIAM1,DMD
GO:CC	cell cortex	GO:0005938	0.005695	140	41	PRKCZ,TMEM201,ACTN2,FMN2,RAB10,SPTBN1,CLASP1,NCL,CLASP2,ERC2,NDFIP1,DCTN4,DST,EPB41L2,HIP1,PCLO,CALD1,DMTN,PTK2B,RIMS2,PTK2,ARC,CLTA,SPTAN1,TRAF2,STIM1,NUMA1,EPS8,FRY,SPTB,ACTN1,AKAP13,IQGAP1,MYH10,FMNL1,CSNK1G2,ACTN4,AKT2,PPFIA3,ITCH,MICAL3
GO:CC	membrane	GO:0016020	0.009029	2436	433	PRKCZ,KCNAB2,DNAJC11,PARK7,TMEM201,PIK3CD,KIF1B,KAZN,RCC2,RAP1GAP,EPHB2,STX12,PSMB2,GRIK3,MACF1,LRRC41,FAF1,OSBPL9,LRP8,NDC1,CELSR2,SORT1,CSF1,SLC6A17,RBM15,RHOC,MAN1A2,NOTCH2,UBQLN4,LMNA,ARHGEF11,NOS1AP,DDR2,DHX9,RGL1,PIK3C2B,NFASC,PPP2R5A,RAB3GAP2,CNIH3,CDC42BPA,ACTN2,FMN2,SOX11,ASAP2,YWHAQ,DDX1,RAB10,CAD,HNRNPLL,MSH2,SPTBN1,CCDC88A,CYP26B1,SNRNP200,INPP4A,CLASP1,CNTNAP5,RIF1,PRPF40A,GALNT13,SCN3A,SCN1A,LRP2,GORASP2,RAPGEF4,AGPS,SESTD1,CALCRL,HSPD1,ADAM23,ARPC2,CUL3,ITM2C,B3GNT7,NCL,ATG16L1,HDLBP,LRRN1,RAF1,SLC4A7,CLASP2,PDCD6IP,SCN5A,SACM1L,PTH1R,MAP4,CELSR3,USP19,GNAI2,NISCH,CACNA1D,CACNA2D3,ERC2,SLMAP,ROBO2,EPHA6,CBLB,LSAMP,ADCY5,PLXNA1,PODXL2,DNAJC13,TOPBP1,MRAS,PSMD2,EIF4G1,OPA1,HTT,ADRA2C,PDS5A,PDGFRA,RASGEF1B,MAPK10,BMPR1B,ANK2,CAMK2D,JADE1,PCDH10,HHIP,ABCE1,ADCY2,TRIO,CDH6,PDZD2,PRLR,PTGER4,AP3B1,SCAMP1,JMY,APC,RAPGEF6,FNIP1,HSPA9,NDUFA2,PCDHGC4,DIAPH1,NDFIP1,YIPF5,PDGFRB,CAMK2A,SPARC,GRIA1,MGAT4B,CDYL,RANBP9,AGPAT1,SYNGAP1,RPS10,SCUBE3,DEF6,FKBP5,TFEB,TMEM151B,TRAM2,DST,SMAP1,SNAP91,PTPRK,EPB41L2,STXBP5,ARID1B,IGF2R,ELFN1,TNRC18,CYTH3,HNRNPA2B1,VWC2,TMEM248,HIP1,HGF,CACNA2D1,PCLO,GRM3,CUX1,RELN,LRRC4,FLNC,PLXNA4,CALD1,NUP205,KIAA1549,SLC4A2,DMTN,PTK2B,NRG1,FGFR1,PRKDC,LYN,RIMS2,EXT1,KCNQ3,PTK2,ARC,PTPRD,UBAP1,CLTA,DAPK1,SEMA4D,WNK2,FAM120A,SVEP1,RABGAP1,SCAI,GAPVD1,GOLGA2,SPTAN1,NUP188,RAPGEF1,TRAF2,ABCA2,MRPL41,CACNA1B,SEPHS1,ARHGAP21,ITGB1,ANK3,BMPR1A,PTEN,SLIT1,WBP1L,EIF3A,GRK5,STIM1,APBB1,SLC1A2,MAPK8IP1,LRP4,OSBP,AHNAK,GPR137,LRP5,PPP6R3,ANO1,NUMA1,TENM4,GRM5,DCUN1D5,PAFAH1B2,C1QTNF5,HEPACAM,STT3A,IGSF9B,WNK1,TSPAN9,PTPN6,EPS8,STK38L,SLC2A13,ASIC1,SLC4A8,RARG,STAT2,LRP1,TBC1D30,GRIP1,PTPRB,NAV3,PPFIA2,EEA1,HSP90B1,TMEM119,ATXN2,KSR2,NC

						OR2,DHX37,NBEA,AKAP11,TSC22D1,COG3,FNDC3A,PCDH17,PCDH20,PCDH9,NDFIP2,RAP2A,MYO16,ACIN1,ARHGAP5,AKAP6,SSTR1,MAP4K5,PPM1A,ZBTB1,HSPA2,SPTB,ACTN1,SLC39A9,SYNJ2BP,TGFB3,TTC7B,HSP90AA1,TRAF3,PPP1R13B,TMEM179,HERC2,APBA2,RYR3,EMC4,THBS1,USP8,TLN2,HERC1,SNX1,SMAD3,PARP6,AP3B2,AKAP13,IQGAP1,RAB11FIP3,MAPK8IP3,TSC2,PKD1,CDIP1,GRIN2A,XPO6,VPS35,MMP2,HERPUD1,DYNC1LI2,EDC4,AP1G1,GLG1,BCAR1,PLCG2,EMC8,PRPF8,RAP1GAP2,TNFSF12-TNFSF13,TNFSF13,RPL26,MYH10,ARHGAP44,NCOR1,FLCN,MYO18A,GIT1,NF1,RHOT1,ASIC2,SYNRG,CACNB1,CASC3,KCNH4,STAT3,ATP6V0A1,RPL27,FZD2,FMNL1,NPEPPS,MRPL27,MTMR4,CLTC,MRC2,ERN1,DDX5,NUP85,SOCS3,CYTH1,EIF4A3,PTPRM,CHMP1B,LDLRAD4,MIB1,DTNA,ST8SIA3,NEDD4L,BCL2,MADCAM1,PALM,PTBP1,CSNK1G2,PIP5K1C,MAP2K2,C3,PNPLA6,EVI5L,PDE4A,SMARCA4,PRKACA,EPS15L1,ANO8,FCHO1,HOMER3,ATP13A1,ACTN4,AKT2,GRIK5,MEGF8,SYMPK,PPP5C,LMTK3,LIN7B,FLT3LG,PTOV1,RPS9,EPN1,ATRN,PLCB1,JAG1,SLC24A3,BCL2L1,ITCH,GGT7,SRC,ARFGEF2,ATP9A,TIAM1,ITSN1,BRWD1,MICAL3,PEX26,PI4KA,BCR,GAL3ST1,GGA1,PDGFB,XRCC6,SCUBE1,GRAMD4,PLXNB2,SBF1,SHANK3,GPR143,GLRA2,AP1S2,DMD,DDX3X,MAGED1,MED12,COL4A6,IRS4,DDX3Y
GO:CC	collagen-containing extracellular matrix	GO:0062023	0.01218	74	25	COL11A1,LAMC1,FBN2,SPARC,ADAMTS2,COL12A1,THBS2,VWC2,COL1A2,COL27A1,COL5A1,LTBP3,HSP90B1,TGFB3,HSP90AA1,THBS1,PKM,LOXL1,ACAN,MMP2,COL1A1,ADAMTS10,TIMP3,PDGFB,COL4A6
GO:CC	endomembrane system	GO:0012505	0.027871	1366	256	PRKCZ,KCNAB2,PARK7,TMEM201,KIF1B,RCC2,RAP1GAP,STX12,MACF1,RNF220,FAF1,OSBPL9,NDC1,COL11A1,SORT1,CSF1,SLC6A17,RBM15,RHOC,MAN1A2,NOTCH2,ASH1L,UBQLN4,LMNA,NOS1AP,LAMC1,PIK3C2B,NFASC,RAB3GAP2,CNIH3,ACTN2,FMN2,ASAP2,RAB10,CCDC88A,CYP26B1,INPP4A,CLASP1,RIF1,GALNT13,LRP2,GORASP2,CALCRL,HSPD1,ARPC2,CUL3,ITM2C,B3GNT7,NCL,ATG16L1,AGAP1,RAF1,CLASP2,PDCD6IP,SCN5A,SACM1L,USP19,GNAI2,NISCH,SLMAP,PODXL2,DNAJC13,PSMD2,HTT,ADRA2C,PDGFRA,RASGEF1B,NFKB1,ANK2,CAMK2D,PDZD2,MTMR12,PRLR,AP3B1,SCAMP1,JMY,APC,DIAPH1,NDFIP1,YIPF5,PDGFRB,SPARC,GRIA1,MGAT4B,AGPAT1,TRAM2,DST,COL12A1,SNAP91,STXBP5,TCP1,IGF2R,THBS2,TNRC18,CYTH3,CDK13,HIP1,HGF,CACNA2D1,COL1A2,CUX1,AHCYL2,NUP205,XPO7,DMTN,LYN,EXT1,ARC,UBAP1,CLTA,COL27A1,SCAI,GAPVD1,GOLGA2,SPTAN1,NUP188,RAPGEF1,COL5A1,TRAF2,ABCA2,TUBB4B,SEPHS1,ARHGAP21,ABI1,ITGB1,WDFY4,ANK3,CCAR1,GLUD1,LGI1,SLIT1,GRK5,BRSK2,STIM1,APBB1,MAPK8IP1,OSBP,LRP5,PAFAH1B2,C1QTNF5,STT3A,PTPN6,ASIC1,SLC4A8,LRP1,GRIP1,CCT2,PTPRB,NAV3,PPFIA2,EEA1,HSP90B1,TMEM119,ATXN2,DHX37,NBEA,TPT1,COG3,FNDC3A,NDFIP2,RAP2A,ARHGAP5,AKAP6,ZBTB1,ACTN1,SLC39A9,TGFB3,HSP90AA1,TRAF3,CYFIP1,RYR3,EMC4,THBS1,SRP14,USP8,HERC1,SNX1,SMAD3,PKM,PARP6,LOXL1,AP3B2,ACAN,IQGAP1,RAB11FIP3,MAPK8IP3,TSC2,PKD1,CDIP1,GRIN2A,METTL9,SRCAP,VPS35,HERPUD1,DYNC1LI2,AP1G1,GLG1,USP10,EMC8,RAP1GAP2,MINK1,ARHGAP44,ALKBH5,MYO18A,SYNRG,CASC3,ATP6V0A1,RPL27,COL1A1,MTMR4,CLTC,ERN1,NUP85,SEC14L1,CYTH1,CHMP1B,LDLRAD4,ST8SIA3,NEDD4L,BCL2,PIP5K1C,MAP2K2,C3,PNPLA6,PRKACA,EPS15L1,ANO8,FCHO1,ATP13A1,ACTN4,AKT2,GRIK5,LMTK3,PPFIA3,EPN1,ATRN,PLCB1,BCL2L1,ITCH,SRC,ARFGEF2,ATP9A,USP25,ITSN1,GAL3ST1,TIMP3,GGA1,PDGFB,XRCC6,GRAMD4,SBF1,GPR143,AP1S2,DMD,DDX3X,COL4A6
GO:CC	cytoskeleton	GO:0005856	0.027871	760	153	PRKCZ,SKI,KCNAB2,TMEM201,KIF1B,KAZN,RCC2,IFFO2,EYA3,MAP7D1,MACF1,LRP8,NDC1,LMNA,NOS1AP,DDR2,DHX9,MAPKAPK2,PPP2R5A,CDC42BPA,TRIM67,ACTN2,FMN2,KIF26B,RAB10,SPTBN1,CCDC88A,DNAH6,CLASP1,KIF5C,RIF1,SESTD1,MAP2,ARPC2,CUL3,ATG16L1,CLASP2,PDCD6IP,MAP4,GNAI2,NISCH,ERC2,SLMAP,ATXN7,TOPBP1,YEATS2,OPA1,HTT,LIMCH1,ANK2,JADE1,PDZD2,JMY,APC,CCDC112,RAPGEF6,KIF3A,DIAPH1,DCTN4,PDLIM7,RANBP9,DEF6,DST,EPB41L2,TCP1,AUTS2,HIP1,PCLO,FLNC,CALD1,DMTN,PTK2B,CCNE2,RIMS2,PTK2,ARC,FAM83H,CLTA,DAPK1,GOLGA2,SPTAN1,ABCA2,TUBB4B,FRMD4A,ARHGAP21,ABI1,ANK3,CCSER2,SH3PXD2A,EIF3A,BRSK2,STIM1,AHNAK,NUMA1,DCUN1D5,WNK1,STK38L,NCKAP5L,KRT18,LRP1,TBC1D30,CCT2,NAV3,KATNAL1,FRY,AKAP11,TPT1,SLAIN1,MYO16,HSPA2,SPTB,ACTN1,HERC2,LEO1,TLN2,AKAP13,IQGAP1,RAB11FIP3,MYH11,DYNC1LI2,GLG1,BCAR1,USP10,RAP1GAP2,NEURL4,MYH10,NCOR1,FLCN,MYO18A,GIT1,CLTC,TLK2,NUP85,CHMP1B,MIB1,DTNA,MAP2K2,DPP9,PIN1,PRKACA,ACTN4,SYMPK,BCL2L1,TPX2,RBM39,SRC,ARFGEF2,TIAM1,BRWD1,MICAL3,DMD,DDX3X,MECP2
GO:CC	cell cortex region	GO:0099738	0.035999	17	9	PRKCZ,CLASP1,CLASP2,ERC2,PCLO,RIMS2,CLTA,NUMA1,PPFIA3
GO:CC	cytoplasmic region	GO:0099568	0.035999	94	28	PRKCZ,KIF1B,GRIK3,DNAH6,CLASP1,KIF5C,MAP2,ATG16L1,CLASP2,MAP4,ERC2,OPA1,AP3B1,KIF3A,DST,PCLO,RIMS2,ARC,CLTA,TUBB4B,NUMA1,ZC3H14,AP3B2,MAPK8IP3,PRKACA,PPFIA3,ARFGEF2,BRWD1
GO:CC	external encapsulating structure	GO:0030312	0.041658	110	31	COL11A1,LAMC1,SNED1,LRRN1,FBN2,SPARC,ADAMTS2,DST,COL12A1,THBS2,ELFN1,VWC2,COL1A2,RELN,COL27A1,COL5A1,LTBP3,HSP90B1,TGFB3,HSP90AA1,THBS1,PKM,LOXL1,ACAN,MMP2,GLG1,COL1A1,ADAMTS10,TIMP3,PDGFB,COL4A6
GO:CC	extracellular matrix	GO:0031012	0.041658	110	31	COL11A1,LAMC1,SNED1,LRRN1,FBN2,SPARC,ADAMTS2,DST,COL12A1,THBS2,ELFN1,VWC2,COL1A2,RELN,COL27A1,COL5A1,LTBP3,HSP90B1,TGFB3,HSP90AA1,THBS1,PKM,LOXL1,ACAN,MMP2,GLG1,COL1A1,ADAMTS10,TIMP3,PDGFB,COL4A6
GO:CC	cortical	GO:0030863	0.041658	52	18	ACTN2,SPTBN1,CLASP1,CLASP2,ERC2,EPB41L2,HIP1,PCLO,CALD1,DMTN,RIMS2,SPTAN1,NUMA1,SPTB,ACTN1,AKAP13,IQGAP1,ACTN4

	cytoskeleton					
REAC	Extracellular matrix organization	REAC:R-HSA-1474244	0.021817	74	26	COL11A1,DDR2,LAMC1,FBN2,SPARC,ADAMTS2,SCUBE3,COL12A1,COL1A2,COL27A1,COL5A1,ITGB1,SH3PXD2A,LRP4,LTBP3,ACTN1,TGFB3,THBS1,LOXL1,ACAN,MMP2,COL1A1,PDGFB,SCUBE1,DMD,COL4A6
REAC	MET activates PTK2 signaling	REAC:R-HSA-8874081	0.021817	16	10	COL11A1,LAMC1,HGF,COL1A2,PTK2,COL27A1,COL5A1,ITGB1,COL1A1,SRC
TF	Factor: Sp2; motif: TGGGCGCGCCCA; match class: 1	TF:M06948_1	0.016102	2003	374	TMEM201,PIK3CD,RBP7,UBE4B,PGD,CASZ1,RCC2,EPHB2,RUNX3,MAP7D1,GRIK3,LRRC41,LRP8,ARHGAP29,CELSR2,SORT1,CSF1,SLC6A17,RHOC,MAN1A2,POGZ,UBQLN4,NOS1AP,DHX9,LAMC1,PPP2R5A,CNIH3,CDC42BPA,TRIM67,ASAP2,RAB10,HNRNPLL,SIX3,SPTBN1,CCDC88A,CYP26B1,SNRNP200,INPP4A,MCM6,KIF5C,RIF1,PRPF40A,GALNT13,LRP2,SESTD1,SPATS2L,KANSL1L,ARPC2,SPEG,ITM2C,B3GNT7,NCL,ATG16L1,AGAP1,PER2,SNED1,LRRN1,CLASP2,SCN5A,MAP4,ARIH2,USP19,CACNA2D3,SLMAP,FOXP1,EPHA6,CBLB,ADCY5,PLXNA1,PODXL2,DNAJC13,TOPBP1,MRAS,YEATS2,ADRA2C,HMX1,PDS5A,LIMCH1,PDGFRA,NFKB1,CAMK2D,JADE1,HHIP,ZNF827,ADCY2,TRIO,PDZD2,MTMR12,PTGER4,SCAMP1,JMY,ELL2,APC,CCDC112,FBN2,FNIP1,KIF3A,JADE2,HSPA9,NDFIP1,YIPF5,TCOF1,SLIT3,PDLIM7,ADAMTS2,MGAT4B,GMDS,E2F3,DHX16,BRD2,SCUBE3,TFEB,TMEM151B,TRAM2,COL12A1,SNAP91,VGLL2,PTPRK,ARID1B,TCP1,IGF2R,ELFN1,FOXK1,TNRC18,CYTH3,HNRNPA2B1,TBX20,CDK13,HECW1,AUTS2,HIP1,CUX1,SRPK2,FOXP2,LRRC4,FLNC,PLXNA4,FAM131B,SLC4A2,XPO7,PTK2B,KAT6A,LYN,CCNE2,RIMS2,EXT1,ATAD2,TRIB1,KCNQ3,PTK2,FAM83H,PUF60,MLLT3,NOL6,PAX5,GDA,ZFAND5,TLE1,WNK2,FAM120A,PHF2,COL27A1,SCAI,LMX1B,SPTAN1,RAPGEF1,COL5A1,TRAF2,ABCA2,TUBB4B,CACNA1B,SEPHS1,MLLT10,ARHGAP21,ITGB1,C10ORF53,CCAR1,BMPR1A,GLUD1,PTEN,NFKB2,SH3PXD2A,EIF3A,BRSK2,DUSP8,CDKN1C,STIM1,APBB1,SLC1A2,MAPK8IP1,LRP4,AHNAK,GPR137,LTBP3,RBM14-RBM4,LRP5,PPP6R3,TENM4,PCF11,GRM5,KMT2A,C1QTNF5,IGSF9B,TSPAN9,RASSF8,SLC2A13,HDAC7,ASIC1,LRP1,PTPRB,EEA1,C12ORF75,CUX2,ATXN2,KATNAL1,NBEA,TSC22D1,TPT1,FNDC3A,NDFIP2,RAP2A,ARHGAP5,PPM1A,ZBTB1,HSPA2,ACTN1,SRSF5,TTC7B,CCDC85C,HSP90AA1,PPP1R13B,TMEM179,CYFIP1,HERC2,RYR3,EMC4,BAHD1,FOXB1,SMAD3,TLE3,PKM,PARP6,LOXL1,AP3B2,ACAN,IDH2,CRTC3,NR2F2,MEF2A,MAPK8IP3,PKD1,CREBBP,GRIN2A,METTL9,XPO6,SRCAP,BRD7,MMP2,HERPUD1,DYNC1LI2,AP1G1,ZFHX3,BCAR1,PLCG2,USP10,GSE1,EMC8,ZCCHC14,ANKRD11,PITPNA,PRPF8,RAP1GAP2,MINK1,TNFSF12-TNFSF13,TNFSF13,RPL26,MYH10,ARHGAP44,FLCN,ALKBH5,MYO18A,GIT1,NF1,SYNRG,CASC3,KCNH4,STAT5B,FMNL1,NPEPPS,COL1A1,MTMR4,MRC2,DCAF7,MAP3K3,ERN1,SOX9,SEC14L1,SOCS3,CBX2,EIF4A3,THOC1,PTPRM,PPP4R1,LDLRAD4,MIB1,ASXL3,MBD1,ELAC1,ST8SIA3,NEDD4L,FBXO15,ADNP2,PALM,PTBP1,REXO1,CSNK1G2,BTBD2,PIP5K1C,DPP9,SAFB2,EVI5L,ADAMTS10,PIN1,RAVER1,PDE4A,SMARCA4,DCAF15,PRKACA,FCHO1,SUGP2,SUGP1,ATP13A1,ZNF536,ZNF850,ACTN4,AKT2,POU2F2,MEGF8,SYMPK,PPP5C,LMTK3,FLT3LG,PTOV1,ZNF813,CENPB,JAG1,SLC24A3,BCL2L1,NCOA3,ATP9A,PHACTR3,USP25,ITSN1,BRWD1,PCBP3,MICAL3,PEX26,DGCR8,PI4KA,BCR,GAL3ST1,TIMP3,GGA1,PDGFB,RPL3,XRCC6,SCUBE1,PLXNB2,SBF1,SHANK3,AP1S2,DDX3X,KDM6A,IQSEC2
TF	Factor: AP-2alpha; motif: NGCCYSNNGSN; match class: 1	TF:M01857_1	0.032631	1687	319	PRKCZ,SKI,ACOT7,RERE,TMEM201,PIK3CD,RBP7,UBE4B,KIF1B,PGD,CASZ1,KAZN,RCC2,IFFO2,RAP1GAP,EPHB2,MAP7D1,LRRC41,LRP8,CELSR2,SORT1,RBM15,RHOC,NOTCH2,POGZ,UBQLN4,ARHGEF11,LAMC1,MAPKAPK2,CDC42BPA,ACTN2,KIF26B,SOX11,ASAP2,YWHAQ,ATAD2B,ZNF513,WDR43,SIX2,SPTBN1,CCDC88A,CYP26B1,EIF5B,CNTNAP5,LRP2,RAPGEF4,SESTD1,HSPD1,ADAM23,ARPC2,CUL3,B3GNT7,NCL,AGAP1,PER2,LRRN1,SLC4A7,CLASP2,SCN5A,SETD2,MAP4,CELSR3,CACNA2D3,ERC2,FOXP1,ADCY5,PLXNA1,GMPS,EIF4G1,ADRA2C,HMX1,LIMCH1,RASGEF1B,CAMK2D,ZNF827,ADCY2,TRIO,PTGER4,MAP3K1,PDE8B,SCAMP1,JMY,ELL2,FBN2,JADE2,ADAMTS2,MGAT4B,GMDS,CDYL,RANBP9,E2F3,RING1,RPS10-NUDT3,RPS10,SCUBE3,TFEB,TMEM151B,RUNX2,TRAM2,DST,STXBP5,IGF2R,ELFN1,FOXK1,TNRC18,CYTH3,TBX20,CDK13,TMEM248,AUTS2,GRM3,COL1A2,ACTL6B,CUX1,RELN,SRPK2,LRRC4,FLNC,PLXNA4,KIAA1549,FAM131B,SLC4A2,DUSP4,NRG1,FGFR1,KAT6A,SOX17,LYN,GDF6,ATAD2,TRIB1,PTK2,FAM83H,ZFAND5,WNK2,FAM120A,PHF2,SVEP1,COL27A1,LMX1B,RAPGEF1,COL5A1,ABCA2,TUBB4B,CACNA1B,MLLT10,ARHGAP21,ITGB1,CREM,CCAR1,ZNF503,CCSER2,PTEN,SUFU,SH3PXD2A,GRK5,BRSK2,DUSP8,CDKN1C,STIM1,LRP4,NR1H3,OSBP,MACROD1,GPR137,LTBP3,LRP5,PPP6R3,ARHGEF17,KMT2A,C1QTNF5,IGSF9B,WNK1,TSPAN9,PTPN6,PHC1,RASSF8,SLC2A13,HDAC7,PRPF40B,NCKAP5L,ASIC1,KRT18,SMARCC2,LRP1,SRGAP1,PTPRB,PPFIA2,EEA1,HSP90B1,CUX2,HECTD4,MSI1,KDM2B,EP400,FRY,NBEA,LRCH1,FNDC3A,SLAIN1,ZIC5,NPAS3,PPM1A,ACTN1,DCAF5,TTC7B,CCDC85C,HSP90AA1,TECPR2,PPP1R13B,CYFIP1,HERC2,RYR3,THBS1,BAHD1,SLTM,SMAD3,TLE3,LOXL1,ACAN,IDH2,CRTC3,NR2F2,RAB11FIP3,MAPK8IP3,PKD1,CREBBP,MYH11,METTL9,XPO6,PAGR1,VPS35,BRD7,IRX3,MMP2,HERPUD1,DYNC1LI2,EDC4,ZFHX3,GLG1,BCAR1,PLCG2,USP10,GSE1,ZCCHC14,ANKRD11,TNFSF12-TNFSF13,MYH10,ALKBH5,MYO18A,GIT1,CASC3,FZD2,FMNL1,SPOP,TLK2,MAP3K3,SOX9,SOCS3,CBX2,EIF4A3,PTPRM,PPP4R1,ESCO1,MIB1,ASXL3,ST8SIA3,NEDD4L,BCL2,ADNP2,MADCAM1,PALM,PTBP1,REXO1,CSNK1G2,BTBD2,PIP5K1C,MAP2K2,DPP9,SAFB2,EVI5L,ADAMTS10,PIN1,PDE4A,SMARCA4,ANO8,SUGP2,ZNF536,AKT2,MEGF8,SYMPK,LIN7B,PTOV1,ATRN,CENPB,PLCB1,JAG1,SLC24A3,BCL2L1,CHD6,ATP9A,BACH1,BRWD1,PCBP3,DGCR8,PI4KA,BCR,GAL3ST1,TIMP3,GGA1,PDGFB,SCUBE1,SBF1,SHANK3,AP1S2,KDM6A,IQSEC2,MED12,MECP2
