# Supplementary material for: Genomic landscape of autism spectrum disorder in Brazil
Source: Genet Mol Biol. 2026 Sep 7;49(Suppl 1):e20250247. doi: 10.1590/1678-4685-GMB-2025-0247 (PMC13561403; doi:10.1590/1678-4685-GMB-2025-0247)
Supplement: Supplementary file 2 [file 1415-4757-GMB-49-s1-e20250247-s10.pdf]

Genetics and Molecular Biology
https://doi.org/10.1590/1678-4685-GMB-2025-0247

Supplementary Material to "Genomic landscape of autism spectrum disorder in Brazil"

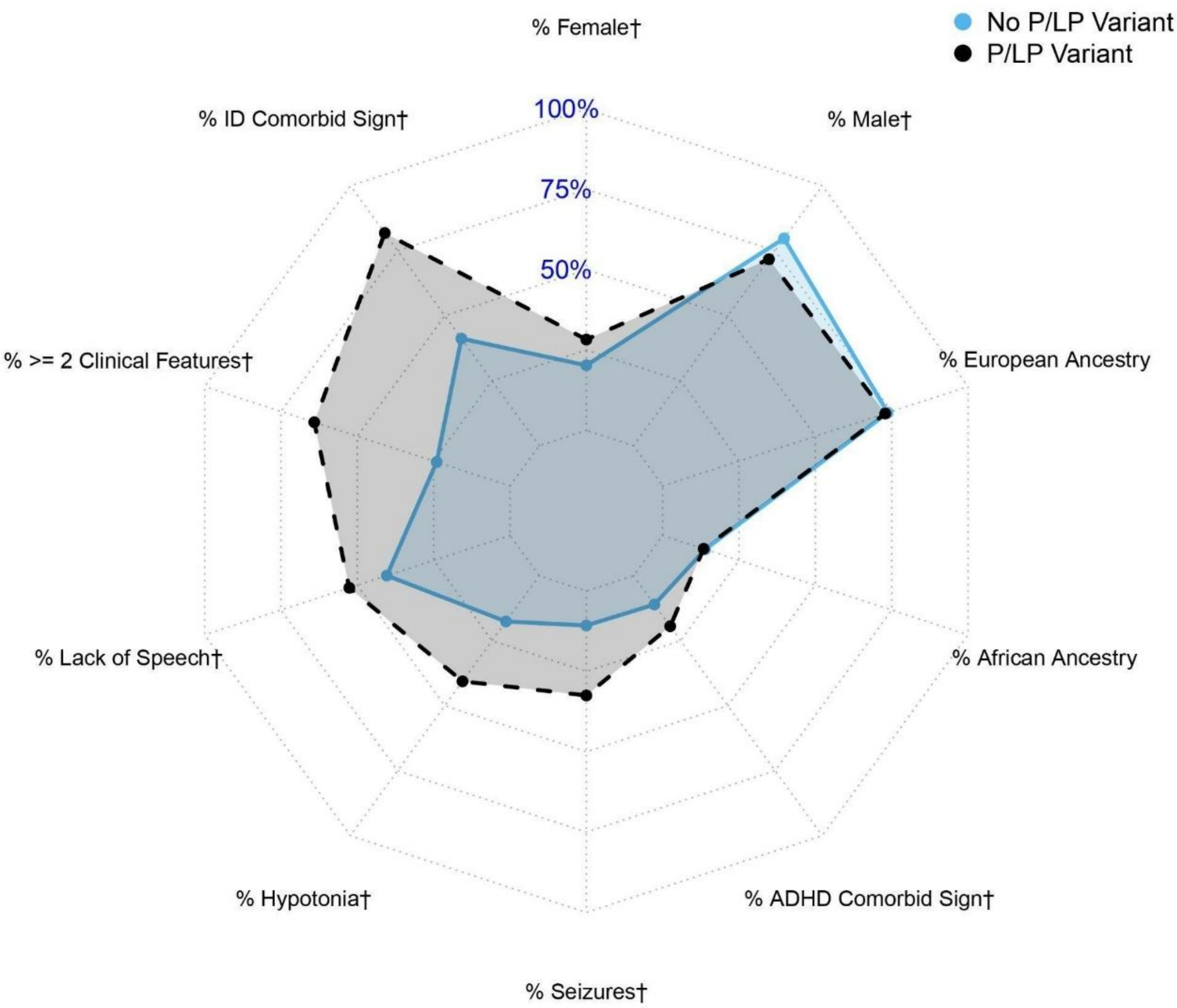

Figure S5 - Radar chart showing the difference in the distribution of clinical and ancestry features between individuals who have a pathogenic/likely pathogenic (P/LP) variant (grey line) and those who do not (blue line).
