# Supplementary material for: Genomic landscape of autism spectrum disorder in Brazil
Source: Genet Mol Biol. 2026 Sep 7;49(Suppl 1):e20250247. doi: 10.1590/1678-4685-GMB-2025-0247 (PMC13561403; doi:10.1590/1678-4685-GMB-2025-0247)
Supplement: Supplementary file 5 [file 1415-4757-GMB-49-s1-e20250247-s13.pdf]

*Genetics and Molecular Biology*
https://doi.org/10.1590/1678-4685-GMB-2025-0247

# Supplementary Material to "Genomic landscape of autism spectrum disorder in Brazil"

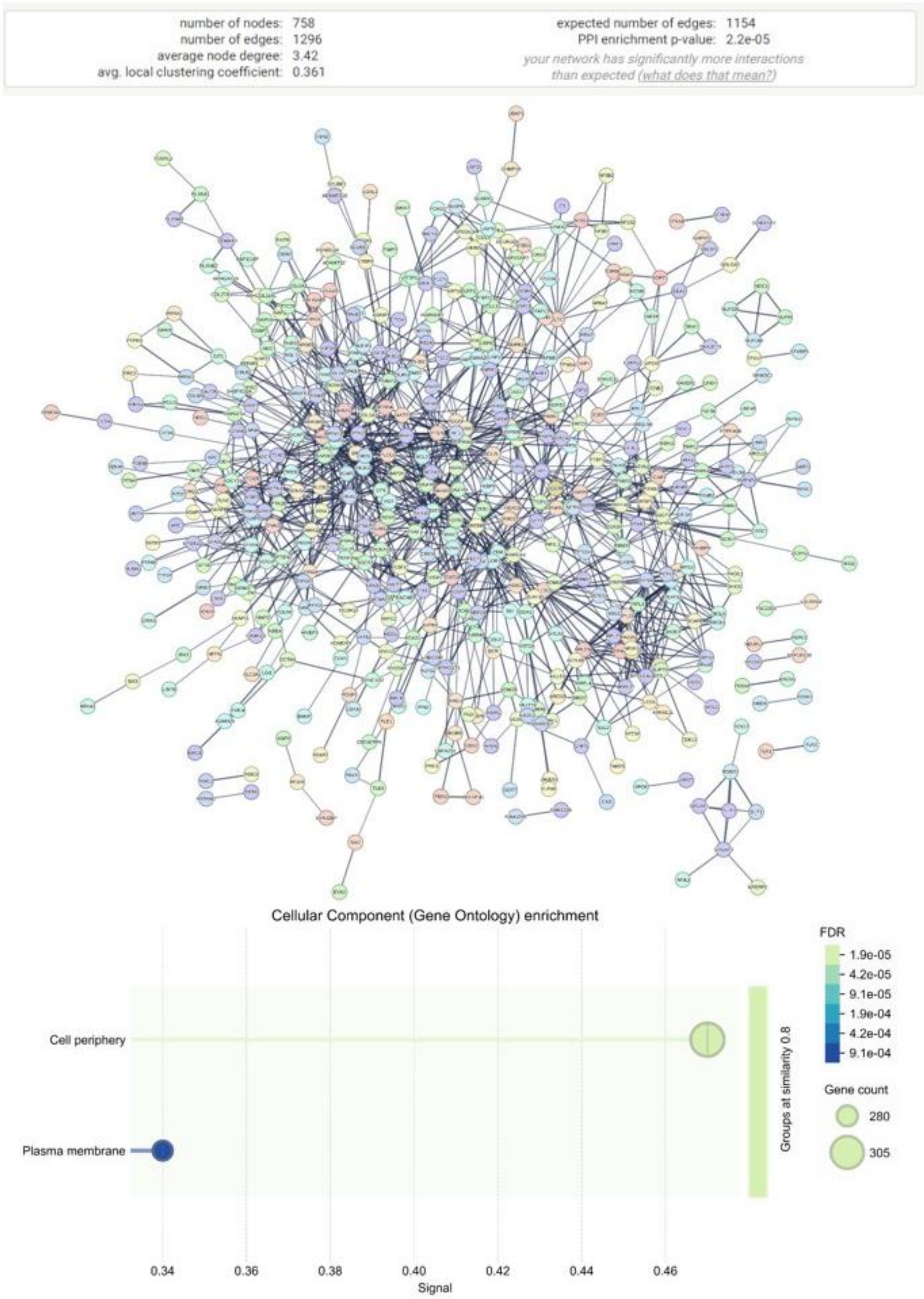


**Figure S8 -** Results of the STRING protein-protein interaction (PPI) network analysis for the Rare Risk Variants (RRVs) identified in the probands. Top: network characteristics. Bottom: GO enrichment.
