# Supplementary material for: Genomic landscape of autism spectrum disorder in Brazil
Source: Genet Mol Biol. 2026 Sep 7;49(Suppl 1):e20250247. doi: 10.1590/1678-4685-GMB-2025-0247 (PMC13561403; doi:10.1590/1678-4685-GMB-2025-0247)
Supplement: Supplementary file 6 [file 1415-4757-GMB-49-s1-e20250247-s2.pdf]

Genetics and Molecular Biology
https://doi.org/10.1590/1678-4685-GMB-2025-0247

Supplementary Material to "Genomic landscape of autism spectrum disorder in Brazil"

Table S1 - Copy Number Variation identified in the review, including Variant of Uncertain Significance (VUS), Likely Pathogenic and Pathogenic alterations.

Reference	Case	Sex	CNV Type	ACMG Classification	Cytogenetic location	Genomic Position	Size (Kbp)	Phenotype	Genes Involved
Chaves *et al.*, 2024	#1006	M	Loss	VUS	15q11.2	15q11.2(22,770,421-23,214,655)x1	444	ASD	*CYFIP1, NIPA2*
Chaves *et al.*, 2024	#1026	M	Gain	Pathogenic	2q33.1	2q33.1(200,182,545–201,185,809)x3	1003	ASD, ID and DF	*SATB2*
Chaves *et al.*, 2024	#1050	M	Loss	Pathogenic	9p24.3p24.1	9p24.3p24.1(208,454–5,222,238)x1	5013	ASD, ID, pectus excavatum and FD	*DMRT1, DMRT2, DMRT3*
Chaves *et al.*, 2024	#1053	M	Loss	VUS	7q31.1	7q31.1(111,483,652-111,922,578)x1	439	ASD, midID, obesity and gynecomastia	*DOCK4, ZNF277*
Chaves *et al.*, 2024	#1091	M	Gain	VUS	Xq11.2q12	Xq11.2q12(64,008,668-64,866,293)x2	858	ASD	*ZC4H2*
Chaves *et al.*, 2024	#1099	F	Gain	VUS	16q24.1	16q24.1(84,439,871-84,676,492)x3	237	Obesity, ASD	*ATP2C2*
Chaves *et al.*, 2024	#1100	F	Loss	Pathogenic	15q13.2q13.3	15q13.2q13.3(31,098,690–32,444,261)x1	1346	Asperger's syndrome	*CHRNA7*
Chaves *et al.*, 2024	#1107	M	Loss	Pathogenic	9p24.3p22.3	9p24.3p22.3(208,454–15,424,987)x1	15.216	ASD	*NFIB, FREM1*
Chaves *et al.*, 2024	#1120	M	Gain	VUS	15q21.1	15q21.1(45,225,243-45,806,154)x3	581	Mild ID	-
Chaves *et al.*, 2024	#1127	M	Loss	VUS	2q31.2	2q31.2(179,396,924-179,629,278)x1	232	Epilepsy	*TTN*
Chaves *et al.*, 2024	#13	M	Loss	VUS	11q14.1	11q14.1(84,050,388-84,415,990)x1	365	LDO, motor difficulties, FD and SLD	*DLG2*
Chaves *et al.*, 2024	#148	F	Gain	Pathogenic	Xp22.3q28	Xp22.3q28(1–247,249,719)x3 ou arr(X)x3	155.27	DD and schizophrenia	–
Chaves *et al.*, 2024	#15	M	Loss	Pathogenic	16p11.2	16p11.2(28,689,085–29,043,863)x1	355	DD	*SH2B1*
Chaves *et al.*, 2024	#16	M	Loss	Pathogenic	16p11.2	16p11.2(28,689,085–29,388,495)x1	362	DD	*SH2B1*

Reference	Case	Sex	CNV Type	ACMG Classification	Cytogenetic location	Genomic Position	Size (Kbp)	Phenotype	Genes Involved
Chaves *et al.*, 2024	#178	M	Gain	VUS	11q23.3	11q23.3(117,000,284-117,312,611)x3	312.327	ASD, FD, macrocephaly	*DSCAML1, CEP164, BACE1*
Chaves *et al.*, 2024	#184	M	Loss	Pathogenic	15q11.2q13.1	15q11.2q13.1(22,770,421–28,823,722)x1	6053	DD, ID, epilepsy, ASD and ADHD	*UBE3A, SNRPN*
Chaves *et al.*, 2024	#215	M	Loss	VUS	3q26.33	3q26.33(179,508,262-179,621,954)x1	113.692	Motor delay, DD, ID, ASD and ADHD	*PEX5L*
Chaves *et al.*, 2024	#235	F	Gain	Pathogenic	17p11.2	17p11.2(16,591,260–20,473,937)x3	3882	Slender build, DD, SLD, ModID, ASD and FD	*RAI*
Chaves *et al.*, 2024	#255	M	Gain	Pathogenic	22q11.21q11.23	22q11.21q11.23(18,493,187–24,313,652)x3	5820	DD, ASD and FD	*TBX1*
Chaves *et al.*, 2024	#268	M	Loss	VUS	2q13	2q13(110,504,318-111,365,996)x1	861.678	ASD	*NPH1*
Chaves *et al.*, 2024	#299	M	Gain	VUS	17q11.2	17q11.2(28,952,286-29,150,025)x3	197.739	DD, ASD and behavioural disorder	*CRLF3*
Chaves *et al.*, 2024	#345	F	Loss	Pathogenic	14q32.2q32.31	14q32.2q32.31(100,095,248–102,755,064)x1	2660	Low weight, short stature, prematurity, IUGR, ataxia, scoliosis, DD, SLD, SevID, ASD, FD and early puberty	*PEGS (DLK1 and RTL1), MEGS (MEG3 and MEG8)*
Chaves *et al.*, 2024	#346	M	Loss	VUS	7q31.1	arr[hg19] 7q31.1(111,485,313-111,922,531)x1	437.218	Low weight, slender build, motor delay, DD, SLD, SevID and ASD.	*DOCK4*
Chaves *et al.*, 2024	#383	M	Gain	VUS	10q11.23	arr[hg19] 10q11.23(51,250,417-51,755,110)x3	504.693	Convulsions, DD, SLD, ASD, behavioural disorder and gluten intolerance	*PARG , MSMBP , NCOA4 , TIMM23*
Chaves *et al.*, 2024	#385	M	Loss	Pathogenic	21q22.12q22.2	arr[hg19] 21q22.12q22.2(35,834,713–39,831,660)x1	3997	Convulsions, DD, ID, SevID, ASD, cardiomyopathy, CAs (abnormal external genitalia) and thrombocytopenia	*DYRK1A*

Reference	Case	Sex	CNV Type	ACMG Classification	Cytogenetic location	Genomic Position	Size (Kbp)	Phenotype	Genes Involved
Chaves *et al.*, 2024	#397	M	Gain	VUS	16p12.2	arr[hg19] 16p12.2(21,817,921-22,431,357)x3	613.436	DD, ASD and FD	*UQCRC2, EEF2K, CDR2*
Chaves *et al.*, 2024	#40	M	Loss	VUS	14q24.2	arr[hg19] 14q24.2(73,590,938-73,776,190)x1	185	ASD and SLD	*PSEN1, NUMBP1*
Chaves *et al.*, 2024	#401	F	Gain	VUS	2q11.1	arr[hg19] 2q11.1(95,733,867-96,279,208)x3	545.341	ASD, DF	*ZNF2, MRPS5, KCNIP3*
Chaves *et al.*, 2024	#416	M	Loss	Pathogenic	18q21.32q23	arr[hg19] 18q21.32q23(58,921,746–78,013,728)x1	19.092	Obesity, CASs, DD, ID, deafness, ASD, FD, and thrombocytopenia	*PIGN*
Chaves *et al.*, 2024	#43	M	Loss	VUS	16q23.2	arr[hg19] 16q23.2(80,260,131-80,701,060)x1	440	MildID, ASD, motor difficulties, SLD, hyperactivity and FD	*DYNLRB2, CDYL2*
Chaves *et al.*, 2024	#443	M	Gain	Pathogenic	22q12.3q13.1	arr[hg19] 22q12.3q13.1(35,888,588–38,692,765)x4	2804	Low weight, short stature, DD, SLD, ASD, behavioral disorder DF and mongolian spots	*45 OMIMs*
Chaves *et al.*, 2024	#444	M	Loss	VUS	16p13.3	arr[hg19] 16p13.3(6,644,079-6,675,606)x1	31	ASD	*RBFOX1*
Chaves *et al.*, 2024	#455	M	Gain	Pathogenic	Yp11.31p11.2-Yq11.23	arr[hg19] Yp11.31p11.2-Yq11.23(2,650,140–28,799,937)x2	26.149	ASD and tall stature	*39 OMIMs*
Chaves *et al.*, 2024	#456	M	Gain	VUS	8q24.3	arr[hg19] 8q24.3(144,262,042-144,486,369)x3	224	ASD and SLD	*GPIHBP1*
Chaves *et al.*, 2024	#470	F	Loss	Pathogenic	2q37.3	arr[hg19] 2q37.3(238,092,121–242,782,258)x1	4690	Asperger's syndrome	*HDAC4*
Chaves *et al.*, 2024	#477	M	Gain	VUS	7p15.3	arr[hg19] 7p15.3(24,133,960-24,671,640)x3	538	ASD and ID	*NPY, MPP6*
Chaves *et al.*, 2024	#492	M	Gain	VUS	Yq11.221q11.222	arr[hg19] Yq11.221q11.222(19,563,599-21,028,944)x4	1.465	ASD and ID	*XKRY, HSFY1, CDY2A*
Chaves *et al.*, 2024	#511	M	Gain	Pathogenic	2q11.2	arr[hg19] 2q11.2(99,222,915–101,919,539)x3	2696	ASD, ID, tall stature, CAs and FD	*13 OMIMs*

Reference	Case	Sex	CNV Type	ACMG Classification	Cytogenetic location	Genomic Position	Size (Kbp)	Phenotype	Genes Involved
Chaves *et al.*, 2024	#511	M	Gain	Pathogenic	2q11.1q11.2	arr[hg19] 2q11.1q11.2(95,327,873–98,719,140)x4	3391	ASD, ID, tall stature, CAs and FD	*24 OMIMs*
Chaves *et al.*, 2024	#52	M	Loss	Pathogenic	22q13.33	arr[hg19] 22q13.33(50,788,193–51,115,526)x1	327	SevID, ASD, motor difficulties, FD, CAs and epilepsy	*SHANK3*
Chaves *et al.*, 2024	#586	M	Loss	Pathogenic	15q21.3	arr[hg19] 15q21.3(57,289,688–57,510,425)x1	221	ASD, hyperactivity and FD (Asymmetric facies)	*TCF12*
Chaves *et al.*, 2024	#592	F	Gain	VUS	9q33.1	arr[hg19] 9q33.1(119,677,859-120,377,754)x3	700	ASD, ID, SLD and hemiparesis	*ASTN2*
Chaves *et al.*, 2024	#594	F	Gain	Pathogenic	1q32.3q41-1q43q44	1q32.3q41-1q43q44(212,011,806–249,181,598)x3	36.743	ASD, ID, CAs and FD	*169 OMIMs*
Chaves *et al.*, 2024	#594	F	Loss	Pathogenic	22q13.31q13.33	arr[hg19] 22q13.31q13.33(47,771,299–51,197,766)x1	3426	ASD, ID, CAs and FD	*29 OMIMs*
Chaves *et al.*, 2024	#595	M	Gain	VUS	14q12	arr[hg19] 14q12(32,110,535-32,560,537)x3	450	ASD, seizures	*NUBPL*
Chaves *et al.*, 2024	#658	M	Gain	VUS	9q22.31	arr[hg19] 9q22.31(94,665,153-94,812,523)x3	147	ASD	*ROR2, SPTLC1*
Chaves *et al.*, 2024	#658	M	Gain	VUS	13q12.3	arr[hg19] 13q12.3(31,764,937-32,190,263)x3	425	ASD	*B3GLCT*
Chaves *et al.*, 2024	#66	M	Gain	Pathogenic	15q25.1q26.3	arr[hg19] 15q25.1q26.3(80,304,866–102,429,040)x3	22.124	SevID, ASD, convulsions, SLD, hyperactivity, CAs (one kidney) and FD	*IGFR1, AKAP13, CPEB1, NTRK3, WDR73*
Chaves *et al.*, 2024	#661	M	Gain	VUS	6q14.3	arr[hg19] 6q14.3(87,410,108-87,911,748)x3	502	ASD	*HTR1E, CGA, ZNF292*
Chaves *et al.*, 2024	#667	M	Loss	Pathogenic	18q12.3	arr[hg19] 18q12.3(42,453,211–42,988,420)x1	535	ASD	*SETBP1 ,SLC14A2*

Reference	Case	Sex	CNV Type	ACMG Classification	Cytogenetic location	Genomic Position	Size (Kbp)	Phenotype	Genes Involved
Chaves *et al.*, 2024	#69	M	Loss	Pathogenic	16p12.2p11.2	arr[hg19] 16p12.2p11.2(21,405,327–29,388,495)x1	7983	MildID, ASD, SLD, hyperactivity and FD	*SH2B1*
Chaves *et al.*, 2024	#695	M	Loss	VUS	16p13.3	arr[hg19] 16p13.3(6,887,840-6,966,572)x1	79	ASD	*RBFOX1*
Chaves *et al.*, 2024	#7	M	Gain	VUS	1q44	1q44(246,324,898-246,688,599)x3	363	ASD, motor difficulties, convulsions and FD	*SMYD3*
Chaves *et al.*, 2024	#70	M	Gain	Pathogenic	7q11.23	arr[hg19] 7q11.23(72,732,834–74,155,067)x3	1422	ModID, ASD and hyperactivity	*WBSCR27, WBSCR28*
Chaves *et al.*, 2024	#714	M	Loss	Pathogenic	16p11.2	arr[hg19] 16p11.2(29,591,326–30,190,029)x1	598	Asperger's syndrome	*20 OMIMs*
Chaves *et al.*, 2024	#727	F	Gain	VUS	2q13	arr[hg19] 2q13(110,504,318-111,365,996)x3	862	ASD, DD, abnormal growth, FD, CAs, macrocephaly and hirsutism	*NPHP1*
Chaves *et al.*, 2024	#737	F	Gain	Pathogenic	16p13.3p12.3	arr[hg19] 16p13.3p12.3(85,880–18,242,713)x3	18.156	ASD, FD and CAs	*CREBBP*
Chaves *et al.*, 2024	#737	F	Loss	Pathogenic	Xq27.3q28	arr[hg19] Xq27.3q28(145,443,311–155,233,098)x1	9723	ASD, FD and CAs	*FMR1, AFF2*
Chaves *et al.*, 2024	#738	M	Gain	VUS	15q13.3	arr[hg19] 15q13.3(31,999,631-32,444,043)x3	444	ASD, ID, FD, SLD, abnormal growth, dyslalia, motor difficulties and ADHD	*CHRNA7*
Chaves *et al.*, 2024	#744	M	Gain	VUS	17p13.3	arr[hg19] 17p13.3(791,201-977,024)x3	186	Hypertension, dyslipidemia	*TIMM22, ABR*
Chaves *et al.*, 2024	#751	M	Loss	Pathogenic	18q12.2q21.1	arr[hg19] 18q12.2q21.1(36,210,635–44,530,609)x1	8319	ASD, FD and CAs	*SETBP1*
Chaves *et al.*, 2024	#76	M	Gain	Pathogenic	7q11.23	arr[hg19] 7q11.23(72,556,215–74,245,599)x3	1689	MildID, ASD	*WBSCR27, WBSCR28*

Reference	Case	Sex	CNV Type	ACMG Classification	Cytogenetic location	Genomic Position	Size (Kbp)	Phenotype	Genes Involved
Chaves *et al.*, 2024	#77	M	Loss	Pathogenic	15q13.2q13.3	arr[hg19] 15q13.2q13.3(31,073,735–32,446,830)x1	1373	MildID, ASD and hyperactivity	*CHRNA7*
Chaves *et al.*, 2024	#791	F	Loss	Pathogenic	14q12	arr[hg19] 14q12(29,197,241–29,514,397)x1	317	ASD, DD, SLD, FD, CAs and seizures	*FOXG1*
Chaves *et al.*, 2024	#809	M	Gain	Pathogenic	Xq28	arr[hg19] Xq28(153,123,879–153,621,056)x2	497	ASD and CAs	*MECP2*
Chaves *et al.*, 2024	#810	M	Gain	VUS	7p11.2	arr[hg19] 7p11.2(57,233,082-57,906,704)x3	674	ASD	-
Chaves *et al.*, 2024	#814	M	Gain	VUS	15q11.2	arr[hg19] 15q11.2(22,770,421-23,288,350)x3	518	ASD	*NIPA1*
Chaves *et al.*, 2024	#830	M	Loss	VUS	11p14.2p14.1	arr[hg19] 11p14.2p14.1(26,997,314-27,233,664)x1	236	ASD	*BBOX1*
Chaves *et al.*, 2024	#853	M	Loss	Pathogenic	16p11.2	arr[hg19] 16p11.2(29,591,326–30,176,508)x1	585	ASD, SLD, FD, dyslalias and motor difficulties	*20 OMIMs*
Chaves *et al.*, 2024	#855	F	Gain	VUS	14q12	arr[hg19] 14q12(26,490,666-27,520,832)x3	1.057	ASD	*NOVA1*
Chaves *et al.*, 2024	#873	F	Loss	Pathogenic	13q33.2q34	arr[hg19] 13q33.2q34(105,020,842–115,107,733)x1	10.086	ASD, ID, CAs, FD and microcephaly	*EFNB2, LIG4, SOX1*
Chaves *et al.*, 2024	#880	M	Loss	VUS	16p13.3	arr[hg19] 16p13.3(6,967,626-6,998,141)x1	30.5	ASD	*RBFOX1*
Chaves *et al.*, 2024	#89	F	Gain	VUS	9p24.3	arr[hg19] 9p24.3(319,876-517,446)x3	198	ASD, SLD, motor difficulties and FD	*DOCK8 , KANK1*
Chaves *et al.*, 2024	#904	M	Loss	VUS	7q31.1	arr[hg19] 7q31.1(110,863,879-111,283,978)x1	420	ASD and DD	*IMMP2L*
Chaves *et al.*, 2024	#913	M	Gain	Pathogenic	15q24.1q24.2	arr[hg19] 15q24.1q24.2(72,899,646–75,567,198)x3	2667	ASD, FD and CAs	*32 OMIMs*
Chaves *et al.*, 2024	#928	M	Loss	VUS	1q21.2	1q21.2(147,723,034-147,830,830)x1	108	ASD, FD, SLD, CAs and macrocephaly	*NBPF8*
Chaves *et al.*, 2024	#937	M	Gain	VUS	1q41q42.12	1q41q42.12(221,795,858-225,581,420)x3	3.786	ASD	*WDR26*

Reference	Case	Sex	CNV Type	ACMG Classification	Cytogenetic location	Genomic Position	Size (Kbp)	Phenotype	Genes Involved
Chaves *et al.*, 2024	#937	M	Gain	VUS	Xq21.1	arr[hg19] Xq21.1(76,993,262-77,392,096)x2	399	ASD	-
Chaves *et al.*, 2024	#937	M	Loss	VUS	1q41	1q41(219,090,413-219,734,998)x1	645	ASD	*LYPLAL1*
Chaves *et al.*, 2024	#941	M	Gain	VUS	Xq28	arr[hg19] Xq28(152,927,530-152,991,389)x2	64	ASD, MildID, SLD and hyperkinetic disorder	*ABCD1*
Chaves *et al.*, 2024	#954	M	Loss	VUS	16p13.3	arr[hg19] 16p13.3(3,315,091-3,432,025)x1	117	ASD, DD, Abnormal growth, FD, CAs, abnormal brain structure, motor difficulties and epilepsy	-
Chaves *et al.*, 2024	#970	F	Gain	Pathogenic	1q21.1q21.2	1q21.1q21.2(146,106,723–147,830,830)x3	1724	ASD	*SATB2*
Chaves *et al.*, 2024	#984	M	Loss	VUS	13q22.2q31.1	arr[hg19] 13q22.2q31.1(76,555,343-80,068,154)x1	3.513	ASD, motor agitation and use of corrective lenses	-
Schuch *et al.*, 2019	P1	M	Loss	LP	1q21.3	1q21.3 (152,538,937-152,595,196)	56	autism disorder. Epilepsy, mood instability, repetitive movements.	*LCE3E, LCE3D, LCE3C, LCE3B*
Schuch *et al.*, 2019	P10	M	Loss	LP	18q22.3	18q22.3 (70,382,640-71,091,974)	709	autism. Psychomotor agitation, self-injury, echolalia, sleep disorder.	*NETO1*
Schuch *et al.*, 2019	P11	M	Loss	LP	22q11.23	22q11.23 (25,621,922-25,799,582)	178	PDD-NOS. Psychomotor agitation, echolalia, mood instability.	*CRYBB2, IGLL3, LRP5L*
Schuch *et al.*, 2019	P12	M	Gain	LP	Xp22.31	Xp22.31 (7,946,477-8,115,153)	150	PDD-NOS. Delayed NPMD, epilepsy, echolalia, mood instability, repetitive movements.	*MIR651*

Reference	Case	Sex	CNV Type	ACMG Classification	Cytogenetic location	Genomic Position	Size (Kbp)	Phenotype	Genes Involved
Schuch *et al.*, 2019	P12	M	Gain	LP	Xp22.31	Xp22.31 (6,552,712-7,033,316)	481	PDD-NOS. Delayed NPMD, epilepsy, echolalia, mood instability, repetitive movements.	*HDHD1A*
Schuch *et al.*, 2019	P2	F	Gain	LP	2q37.3	2q37.3 (242,886,386-243,007,359)	121	PDD-NOS. Seizure, psychomotor agitation, self-injury, echolalia, mood instability, sleep disorder.	
Schuch *et al.*, 2019	P2	F	Gain	LP	1q44	1q44 (247,712,471-2,488,040,516)	328	PDD-NOS. Seizure, psychomotor agitation, self-injury, echolalia, mood instability, sleep disorder.	*C1orf150, TRIM58, OR genes*
Schuch *et al.*, 2019	P3	M	Loss	LP	2p11.2	2p11.2 (90,012,337-90,234,023)	222	PDD-NOS. Mood instability, echolalia, repetitive movements, sleep disorder.	
Schuch *et al.*, 2019	P4	M	Gain	LP	4q35.2	4q35.2 (187,831,029-189,968,308)	2137	autism disorder. Psychomotor agitation, mood instability, repetitive movements, sleep disorder.	*ZFP42, TRIML2, TRIML1*
Schuch *et al.*, 2019	P5	M	Gain	LP	7q11–23	7q11–23 (76,787,292-77,062,787)	275	autism disorder. Delayed NPMD, epilepsy, aggression, mood instability, repetitive movements, sleep disorder.	*CCDC146, FGL2, PION*

Reference	Case	Sex	CNV Type	ACMG Classification	Cytogenetic location	Genomic Position	Size (Kbp)	Phenotype	Genes Involved
Schuch *et al.*, 2019	P6	M	Gain	LP	7p21.3	7p21.3 (8,066,780-8,717,542)	631	PDD-NOS. Aggression, mood instability, echolalia, repetitive movements, sleep disorder.	*GLCCI1, ICA1, NXPH1*
Schuch *et al.*, 2019	P7	M	Loss	LP	15q11.2	15q11.2 (22,874,523-23,179,948)	395	autism disorder. Delayed NPMD, seizure, psychomotor agitation, aggression, echolalia, repetitive movements.	*TUBGCP5, CYF1P1, NIPA1, NIPA2, G100513, G100543*
Schuch *et al.*, 2019	P8	M	Loss	LP	15q25.2-q25.3	15q25.2-q25.3 (85,124,542-85,682,231)	558	autism. Self-injury, mood instability, repetitive movements.	*SCAND2, WDR73, NMB, SEC11A, ZNF592, ALPK3, SLC28A1, PDE8A, AL357213*
Schuch *et al.*, 2019	P9	M	Loss	LP	16p11.2	16p11.2 (29,673,954-30,196,600)	525	PDD-NOS. Delayed NPMD, psychomotor agitation aggression, mood instability.	*SPN, QPRT, C19orf54, ZG16, KIF22, MAZ, PRRT2, C16orf53, MVP, CDIPT, LOC440356, SEZ6L2, ASPHD1, KTCD13, TMEM219, TAOK2, HIRIP3, INO80E, DOC2A, C16orf92, FAM57B, ALDOA, PP4C, TBX6, YPEL3, GDPD3, MAPK3, CORO1A*

References

Chaves TF, Ocampos M, Barbato IT, de Camargo Pinto LL, de Luca GR, Barbato Filho JH, Bernardi P, Costa Netto Muniz Y and Francesca Maris A (2024) A cohort study of neurodevelopmental disorders and/or congenital anomalies using high resolution chromosomal microarrays in southern Brazil highlighting the significance of ASD. Sci Rep 14:3762.

Schuch JB, Paixão-Côrtes VR, Longo D, Roman T, Riesgo RDS, Ranzan J, Becker MM, Riegel M and Schuler-Faccini L (2019) Analysis of a protein network related to copy number variations in autism spectrum disorder. J Mol Neurosci 69:140–149.
