# Supplementary material for: Genomic landscape of autism spectrum disorder in Brazil
Source: Genet Mol Biol. 2026 Sep 7;49(Suppl 1):e20250247. doi: 10.1590/1678-4685-GMB-2025-0247 (PMC13561403; doi:10.1590/1678-4685-GMB-2025-0247)
Supplement: Supplementary file 7 [file 1415-4757-GMB-49-s1-e20250247-s3.pdf]

Genetics and Molecular Biology
https://doi.org/10.1590/1678-4685-GMB-2025-0247

Supplementary Material to “Genomic landscape of autism spectrum disorder in Brazil”

Table S2 - Sample sheet describing all individuals included in the analysis (N = 1,295).

ID	group	status	affected_status	origin	kit	kinship_mother
P_1	trio	proband	yes	ASC	ASC	0,47
M_1	trio	mother	no	ASC	ASC	NA
F_1	trio	father	no	ASC	ASC	NA
P_2	trio	proband	yes	ASC	ASC	0,44
M_2	trio	mother	no	ASC	ASC	NA
F_2	trio	father	no	ASC	ASC	NA
P_3	trio	proband	yes	ASC	ASC	0,43
M_3	trio	mother	no	ASC	ASC	NA
F_3	trio	father	no	ASC	ASC	NA
P_4	trio	proband	yes	ASC	ASC	0,43
M_4	trio	mother	no	ASC	ASC	NA
F_4	trio	father	no	ASC	ASC	NA
P_5	trio	proband	yes	ASC	ASC	0,38
M_5	trio	mother	no	ASC	ASC	NA
F_5	trio	father	no	ASC	ASC	NA
P_6	trio	proband	yes	ASC	ASC	0,42
M_6	trio	mother	no	ASC	ASC	NA
F_6	trio	father	no	ASC	ASC	NA
P_7	trio	proband	yes	ASC	ASC	0,38
M_7	trio	mother	no	ASC	ASC	NA
F_7	trio	father	no	ASC	ASC	NA
P_8	trio	proband	yes	ASC	ASC	0,42
M_8	trio	mother	no	ASC	ASC	NA
F_8	trio	father	no	ASC	ASC	NA
P_9	trio	proband	yes	ASC	ASC	0,39
M_9	trio	mother	no	ASC	ASC	NA
F_9	trio	father	no	ASC	ASC	NA
P_10	trio	proband	yes	ASC	ASC	0,46
M_10	trio	mother	no	ASC	ASC	NA
F_10	trio	father	no	ASC	ASC	NA
P_11	trio	proband	yes	ASC	ASC	0,36
M_11	trio	mother	no	ASC	ASC	NA
F_11	trio	father	no	ASC	ASC	NA
P_12	trio	proband	yes	ASC	ASC	0,43
M_12	trio	mother	no	ASC	ASC	NA
F_12	trio	father	no	ASC	ASC	NA
P_13	trio	proband	yes	ASC	ASC	0,4
M_13	trio	mother	no	ASC	ASC	NA
F_13	trio	father	yes	ASC	ASC	NA
P_14	trio	proband	yes	ASC	ASC	0,4
M_14	trio	mother	no	ASC	ASC	NA
F_14	trio	father	no	ASC	ASC	NA
P_15	trio	proband	yes	ASC	ASC	0,39
M_15	trio	mother	no	ASC	ASC	NA
F_15	trio	father	no	ASC	ASC	NA
P_16	trio	proband	yes	ASC	ASC	0,48
M_16	trio	mother	no	ASC	ASC	NA
F_16	trio	father	no	ASC	ASC	NA
P_17	trio	proband	yes	ASC	ASC	0,43
M_17	trio	mother	no	ASC	ASC	NA

P_17	trio	proband	yes	ASC	ASC		0,43
M_17	trio	mother	no	ASC	ASC	NA	
F_17	trio	father	no	ASC	ASC	NA	
P_18	trio	proband	yes	ASC	ASC		0,38
M_18	trio	mother	no	ASC	ASC	NA	
F_18	trio	father	no	ASC	ASC	NA	
M_19	trio	mother	no	ASC	ASC	NA	
F_19	trio	father	no	ASC	ASC	NA	
P_19	trio	proband	yes	ASC	ASC		0,43
P_20	trio	proband	yes	ASC	ASC		0,38
M_20	trio	mother	no	ASC	ASC	NA	
F_20	trio	father	no	ASC	ASC	NA	
P_21	trio	proband	yes	ASC	ASC		0,35
M_21	trio	mother	no	ASC	ASC	NA	
F_21	trio	father	no	ASC	ASC	NA	
P_22	trio	proband	yes	ASC	ASC		0,38
M_22	trio	mother	no	ASC	ASC	NA	
F_22	trio	father	no	ASC	ASC	NA	
P_23	trio	proband	yes	ASC	ASC		0,37
M_23	trio	mother	no	ASC	ASC	NA	
F_23	trio	father	no	ASC	ASC	NA	
P_24	trio	proband	yes	ASC	ASC		0,41
M_24	trio	mother	no	ASC	ASC	NA	
F_24	trio	father	no	ASC	ASC	NA	
P_25	trio	proband	yes	ASC	ASC		0,36
M_25	trio	mother	no	ASC	ASC	NA	
F_25	trio	father	no	ASC	ASC	NA	
P_26	trio	proband	yes	ASC	ASC		0,41
M_26	trio	mother	no	ASC	ASC	NA	
F_26	trio	father	no	ASC	ASC	NA	
P_27	trio	proband	yes	ASC	ASC		0,37
M_27	trio	mother	no	ASC	ASC	NA	
F_27	trio	father	no	ASC	ASC	NA	
P_28	quartet	proband	yes	ASC	ASC		0,44
M_28	quartet	mother	no	ASC	ASC	NA	
F_28	quartet	father	no	ASC	ASC	NA	
P_29	trio	proband	yes	ASC	ASC		0,45
M_29	trio	mother	no	ASC	ASC	NA	
F_29	trio	father	no	ASC	ASC	NA	
P_30	trio	proband	yes	ASC	ASC		0,34
M_30	trio	mother	no	ASC	ASC	NA	
F_30	trio	father	no	ASC	ASC	NA	
P_31	trio	proband	yes	ASC	ASC		0,39
M_31	trio	mother	no	ASC	ASC	NA	
F_31	trio	father	no	ASC	ASC	NA	
P_32	trio	proband	yes	ASC	ASC		0,42
M_32	trio	mother	no	ASC	ASC	NA	
F_32	trio	father	no	ASC	ASC	NA	
P_33	trio	proband	yes	ASC	ASC		0,44
M_33	trio	mother	no	ASC	ASC	NA	
F_33	trio	father	no	ASC	ASC	NA	
P_34	trio	proband	yes	ASC	ASC		0,46
M_34	trio	mother	no	ASC	ASC	NA	
F_34	trio	father	no	ASC	ASC	NA	

P_35	trio	proband	yes	ASC	ASC		0,44
M_35	trio	mother	no	ASC	ASC	NA	
F_35	trio	father	no	ASC	ASC	NA	
P_36	trio	proband	yes	ASC	ASC		0,42
M_36	trio	mother	no	ASC	ASC	NA	
F_36	trio	father	no	ASC	ASC	NA	
P_37	trio	proband	yes	ASC	ASC		0,44
M_37	trio	mother	no	ASC	ASC	NA	
F_37	trio	father	no	ASC	ASC	NA	
P_38	trio	proband	yes	ASC	ASC		0,45
M_38	trio	mother	no	ASC	ASC	NA	
F_38	trio	father	no	ASC	ASC	NA	
P_39	trio	proband	yes	ASC	ASC		0,41
M_39	trio	mother	no	ASC	ASC	NA	
F_39	trio	father	no	ASC	ASC	NA	
P_40	trio	proband	yes	ASC	ASC		0,43
M_40	trio	mother	no	ASC	ASC	NA	
F_40	trio	father	no	ASC	ASC	NA	
P_41	trio	proband	yes	ASC	ASC		0,45
M_41	trio	mother	no	ASC	ASC	NA	
F_41	trio	father	no	ASC	ASC	NA	
P_42	trio	proband	yes	ASC	ASC		0,47
M_42	trio	mother	no	ASC	ASC	NA	
F_42	trio	father	no	ASC	ASC	NA	
P_43	trio	proband	yes	ASC	ASC		0,4
M_43	trio	mother	no	ASC	ASC	NA	
F_43	trio	father	no	ASC	ASC	NA	
P_44	trio	proband	yes	ASC	ASC		0,41
M_44	trio	mother	no	ASC	ASC	NA	
F_44	trio	father	no	ASC	ASC	NA	
P_45	trio	proband	yes	ASC	ASC		0,45
M_45	trio	mother	no	ASC	ASC	NA	
F_45	trio	father	no	ASC	ASC	NA	
P_46	trio	proband	yes	ASC	ASC		0,43
M_46	trio	mother	no	ASC	ASC	NA	
F_46	trio	father	no	ASC	ASC	NA	
P_47	trio	proband	yes	ASC	ASC		0,47
M_47	trio	mother	no	ASC	ASC	NA	
F_47	trio	father	no	ASC	ASC	NA	
P_48	trio	proband	yes	ASC	ASC		0,48
M_48	trio	mother	no	ASC	ASC	NA	
F_48	trio	father	no	ASC	ASC	NA	
P_49	trio	proband	yes	ASC	ASC		0,4
M_49	trio	mother	no	ASC	ASC	NA	
F_49	trio	father	no	ASC	ASC	NA	
P_50	trio	proband	yes	ASC	ASC		0,44
M_50	trio	mother	no	ASC	ASC	NA	
F_50	trio	father	no	ASC	ASC	NA	
P_51	trio	proband	yes	ASC	ASC		0,4
M_51	trio	mother	no	ASC	ASC	NA	
F_51	trio	father	no	ASC	ASC	NA	
P_52	trio	proband	yes	ASC	ASC		0,46

M_52	trio	mother	no	ASC	ASC	NA	
F_52	trio	father	NA	ASC	ASC	NA	
P_53	trio	proband	yes	ASC	ASC		0,47
M_53	trio	mother	no	ASC	ASC	NA	
F_53	trio	father	no	ASC	ASC	NA	
M_54	trio	mother	no	ASC	ASC	NA	
F_54	trio	father	no	ASC	ASC	NA	
P_54	trio	proband	yes	ASC	ASC		0,41
P_55	trio	proband	yes	ASC	ASC		0,39
M_55	trio	mother	no	ASC	ASC	NA	
F_55	trio	father	no	ASC	ASC	NA	
P_56	trio	proband	yes	ASC	ASC		0,46
M_56	trio	mother	no	ASC	ASC	NA	
F_56	trio	father	no	ASC	ASC	NA	
P_57	trio	proband	yes	HB	SureSelectXT		0,37
P_58	trio	proband	yes	HB	SureSelectXT		0,41
M_59	trio	mother	NA	HB	SureSelectXT	NA	
F_59	trio	father	NA	HB	SureSelectXT	NA	
P_59	trio	proband	yes	HB	SureSelectXT		0,45
P_60	trio	proband	yes	HB	SureSelectXT		0,35
M_60	trio	mother	NA	HB	SureSelectXT	NA	
S1_61	complex	sibling	yes	CEGH	TruSeq	NA	
F_60	trio	father	NA	HB	SureSelectXT	NA	
S_62	quartet	sibling	yes	CEGH	TruSeq	NA	
P_63	trio	proband	yes	HB	SureSelectXT		0,42
F_63	trio	father	NA	HB	SureSelectXT	NA	
M_63	trio	mother	NA	HB	SureSelectXT	NA	
F_57	trio	father	NA	HB	SureSelectXT	NA	
M_57	trio	mother	NA	HB	SureSelectXT	NA	
P_64	duo	proband	yes	ASC	ASC		0,38
M_64	duo	mother	NA	ASC	ASC	NA	
P_65	trio	proband	yes	HB	SureSelectXT		0,36
F_65	trio	father	NA	HB	SureSelectXT	NA	
M_65	trio	mother	NA	HB	SureSelectXT	NA	
M_66	trio	mother	NA	HB	SureSelectXT	NA	
P_66	trio	proband	yes	HB	SureSelectXT		0,43
F_66	trio	father	NA	HB	SureSelectXT	NA	
F_58	trio	father	NA	HB	SureSelectXT	NA	
M_58	trio	mother	NA	HB	SureSelectXT	NA	
P_67	trio	proband	yes	HB	SureSelectXT		0,41
M_67	trio	mother	NA	HB	SureSelectXT	NA	
F_67	trio	father	NA	HB	SureSelectXT	NA	
F_68	trio	father	NA	HB	SureSelectXT	NA	
M_68	trio	mother	NA	HB	SureSelectXT	NA	
P_69	trio	proband	yes	HB	SureSelectXT		0,35
M_69	trio	mother	NA	HB	SureSelectXT	NA	
F_69	trio	father	NA	HB	SureSelectXT	NA	
P_70	trio	proband	yes	HB	SureSelectXT		0,34
M_70	trio	mother	NA	HB	SureSelectXT	NA	
F_70	trio	father	NA	HB	SureSelectXT	NA	
P_71	trio	proband	yes	HB	SureSelectXT		0,38
M_71	trio	mother	NA	HB	SureSelectXT	NA	

F_71	trio	father	NA	HB	SureSelectXT	NA	
P_72	trio	proband	yes	HB	SureSelectXT		0,26
F_72	trio	father	NA	HB	SureSelectXT	NA	
M_72	trio	mother	NA	HB	SureSelectXT	NA	
P_73	trio	proband	yes	HB	SureSelectXT		0,37
F_73	trio	father	NA	HB	SureSelectXT	NA	
M_73	trio	mother	NA	HB	SureSelectXT	NA	
P_74	trio	proband	yes	HB	SureSelectXT		0,4
M_74	trio	mother	NA	HB	SureSelectXT	NA	
F_74	trio	father	NA	HB	SureSelectXT	NA	
P_75	trio	proband	yes	HB	SureSelectXT		0,39
F_75	trio	father	NA	HB	SureSelectXT	NA	
M_75	trio	mother	NA	HB	SureSelectXT	NA	
P_68	trio	proband	yes	HB	SureSelectXT		0,39
P_76	trio	proband	yes	HB	SureSelectXT		0,37
M_76	trio	mother	NA	HB	SureSelectXT	NA	
F_76	trio	father	NA	HB	SureSelectXT	NA	
P_77	trio	proband	yes	HB	SureSelectXT		0,41
M_77	trio	mother	NA	HB	SureSelectXT	NA	
F_77	trio	father	NA	HB	SureSelectXT	NA	
P_78	trio	proband	yes	HB	SureSelectXT		0,35
M_78	trio	mother	NA	HB	SureSelectXT	NA	
P_79	trio	proband	yes	HB	SureSelectXT		0,39
F_79	trio	father	NA	HB	SureSelectXT	NA	
M_79	trio	mother	NA	HB	SureSelectXT	NA	
P_80	trio	proband	yes	ASC	ASC		0,49
M_80	trio	mother	no	ASC	ASC	NA	
F_80	trio	father	NA	ASC	ASC	NA	
P_81	trio	proband	yes	HB	SureSelectXT		0,41
M_81	trio	mother	NA	HB	SureSelectXT	NA	
P_82	trio	proband	yes	HB	SureSelectXT		0,37
M_82	trio	mother	NA	HB	SureSelectXT	NA	
F_82	trio	father	NA	HB	SureSelectXT	NA	
P_83	trio	proband	yes	ASC	ASC		0,42
M_83	trio	mother	no	ASC	ASC	NA	
F_83	trio	father	no	ASC	ASC	NA	
F_78	trio	father	NA	HB	SureSelectXT	NA	
F_81	trio	father	NA	HB	SureSelectXT	NA	
P_84	trio	proband	yes	ASC	ASC		0,38
M_84	trio	mother	no	ASC	ASC	NA	
F_84	trio	father	no	ASC	ASC	NA	
P_85	trio	proband	yes	ASC	ASC		0,44
M_85	trio	mother	no	ASC	ASC	NA	
F_85	trio	father	no	ASC	ASC	NA	
P_86	trio	proband	yes	ASC	ASC		0,4
M_86	trio	mother	no	ASC	ASC	NA	
F_86	trio	father	no	ASC	ASC	NA	
P_87	trio	proband	yes	ASC	ASC		0,36
M_87	trio	mother	no	ASC	ASC	NA	
F_87	trio	father	no	ASC	ASC	NA	
P_88	trio	proband	yes	ASC	ASC		0,39
M_88	trio	mother	no	ASC	ASC	NA	

F_88	trio	father	no	ASC	ASC	NA	
P_89	trio	proband	yes	ASC	ASC		0,36
M_89	trio	mother	no	ASC	ASC	NA	
F_89	trio	father	no	ASC	ASC	NA	
P_90	trio	proband	yes	ASC	ASC		0,36
M_90	trio	mother	no	ASC	ASC	NA	
F_90	trio	father	no	ASC	ASC	NA	
P_91	trio	proband	yes	ASC	ASC		0,43
M_91	trio	mother	no	ASC	ASC	NA	
F_91	trio	father	no	ASC	ASC	NA	
P_92	trio	proband	yes	ASC	ASC		0,39
M_92	trio	mother	no	ASC	ASC	NA	
F_92	trio	father	no	ASC	ASC	NA	
P_93	trio	proband	yes	ASC	ASC		0,39
M_93	trio	mother	no	ASC	ASC	NA	
F_93	trio	father	no	ASC	ASC	NA	
P_94	trio	proband	yes	ASC	ASC		0,38
M_94	trio	mother	no	ASC	ASC	NA	
F_94	trio	father	no	ASC	ASC	NA	
P_95	trio	proband	yes	ASC	ASC		0,41
M_95	trio	mother	no	ASC	ASC	NA	
F_95	trio	father	no	ASC	ASC	NA	
P_96	trio	proband	yes	ASC	ASC		0,4
M_96	trio	mother	no	ASC	ASC	NA	
F_96	trio	father	no	ASC	ASC	NA	
P_97	trio	proband	yes	ASC	ASC		0,4
M_97	trio	mother	no	ASC	ASC	NA	
F_97	trio	father	no	ASC	ASC	NA	
P_98	trio	proband	yes	ASC	ASC		0,42
M_98	trio	mother	no	ASC	ASC	NA	
F_98	trio	father	no	ASC	ASC	NA	
P_99	trio	proband	yes	ASC	ASC		0,49
M_99	trio	mother	no	ASC	ASC	NA	
F_99	trio	father	no	ASC	ASC	NA	
P_100	trio	proband	yes	ASC	ASC		0,42
M_100	trio	mother	no	ASC	ASC	NA	
F_100	trio	father	no	ASC	ASC	NA	
P_101	quartet	proband	yes	ASC	ASC		0,38
M_101	quartet	mother	NA	ASC	ASC	NA	
F_101	quartet	father	NA	ASC	ASC	NA	
P_102	trio	proband	yes	ASC	ASC		0,43
M_102	trio	mother	no	ASC	ASC	NA	
F_102	trio	father	no	ASC	ASC	NA	
P_103	trio	proband	yes	ASC	ASC		0,34
M_103	trio	mother	no	ASC	ASC	NA	
F_103	trio	father	no	ASC	ASC	NA	
P_104	trio	proband	yes	ASC	ASC		0,39
M_104	trio	mother	no	ASC	ASC	NA	
F_104	trio	father	no	ASC	ASC	NA	
P_105	trio	proband	yes	ASC	ASC		0,44
M_105	trio	mother	no	ASC	ASC	NA	
F_105	trio	father	no	ASC	ASC	NA	

P_106	duo	proband	yes	ASC	ASC		0,37
M_106	duo	mother	no	ASC	ASC	NA	
P_107	trio	proband	yes	ASC	ASC		0,47
M_107	trio	mother	no	ASC	ASC	NA	
F_107	trio	father	no	ASC	ASC	NA	
P_108	trio	proband	yes	ASC	ASC		0,44
M_108	trio	mother	no	ASC	ASC	NA	
F_108	trio	father	no	ASC	ASC	NA	
P_109	trio	proband	yes	ASC	ASC		0,37
M_109	trio	mother	no	ASC	ASC	NA	
F_109	trio	father	no	ASC	ASC	NA	
P_110	trio	proband	yes	ASC	ASC		0,39
M_110	trio	mother	no	ASC	ASC	NA	
F_110	trio	father	no	ASC	ASC	NA	
P_111	trio	proband	yes	ASC	ASC		0,46
M_111	trio	mother	no	ASC	ASC	NA	
F_111	trio	father	no	ASC	ASC	NA	
P_112	trio	proband	yes	ASC	ASC		0,36
M_112	trio	mother	no	ASC	ASC	NA	
F_112	trio	father	no	ASC	ASC	NA	
P_113	trio	proband	yes	ASC	ASC		0,41
M_113	trio	mother	no	ASC	ASC	NA	
F_113	trio	father	no	ASC	ASC	NA	
P_114	trio	proband	yes	ASC	ASC		0,44
M_114	trio	mother	no	ASC	ASC	NA	
F_114	trio	father	no	ASC	ASC	NA	
P_115	trio	proband	yes	ASC	ASC		0,37
M_115	trio	mother	no	ASC	ASC	NA	
F_115	trio	father	no	ASC	ASC	NA	
P_116	trio	proband	yes	ASC	ASC		0,44
M_116	trio	mother	no	ASC	ASC	NA	
F_116	trio	father	no	ASC	ASC	NA	
P_117	duo	proband	yes	ASC	ASC		0,41
M_117	duo	mother	no	ASC	ASC	NA	
P_118	trio	proband	yes	ASC	ASC		0,46
M_118	trio	mother	no	ASC	ASC	NA	
F_118	trio	father	no	ASC	ASC	NA	
P_119	trio	proband	yes	ASC	ASC		0,4
M_119	trio	mother	no	ASC	ASC	NA	
F_119	trio	father	no	ASC	ASC	NA	
P_120	trio	proband	yes	ASC	ASC		0,37
M_120	trio	mother	no	ASC	ASC	NA	
F_120	trio	father	no	ASC	ASC	NA	
P_121	trio	proband	yes	ASC	ASC		0,41
M_121	trio	mother	no	ASC	ASC	NA	
F_121	trio	father	no	ASC	ASC	NA	
P_122	trio	proband	yes	ASC	ASC		0,44
M_122	trio	mother	no	ASC	ASC	NA	
F_122	trio	father	no	ASC	ASC	NA	
P_123	trio	proband	yes	ASC	ASC		0,35
M_123	trio	mother	no	ASC	ASC	NA	
F_123	trio	father	no	ASC	ASC	NA	

P_124	trio	proband	yes	ASC	ASC		0,38
M_124	trio	mother	no	ASC	ASC	NA	
F_124	trio	father	no	ASC	ASC	NA	
P_125	trio	proband	yes	ASC	ASC		0,46
M_125	trio	mother	no	ASC	ASC	NA	
F_125	trio	father	no	ASC	ASC	NA	
P_126	trio	proband	yes	ASC	ASC		0,41
M_126	trio	mother	no	ASC	ASC	NA	
F_126	trio	father	no	ASC	ASC	NA	
P_127	trio	proband	yes	ASC	ASC		0,42
M_127	trio	mother	no	ASC	ASC	NA	
F_127	trio	father	no	ASC	ASC	NA	
P_128	trio	proband	yes	ASC	ASC		0,34
M_128	trio	mother	no	ASC	ASC	NA	
F_128	trio	father	no	ASC	ASC	NA	
P_129	trio	proband	yes	ASC	ASC		0,4
M_129	trio	mother	no	ASC	ASC	NA	
F_129	trio	father	no	ASC	ASC	NA	
P_130	trio	proband	yes	ASC	ASC		0,46
M_130	trio	mother	no	ASC	ASC	NA	
F_130	trio	father	no	ASC	ASC	NA	
P_131	trio	proband	yes	ASC	ASC		0,39
M_131	trio	mother	no	ASC	ASC	NA	
F_131	trio	father	no	ASC	ASC	NA	
P_132	trio	proband	yes	ASC	ASC		0,35
M_132	trio	mother	no	ASC	ASC	NA	
F_132	trio	father	no	ASC	ASC	NA	
P_133	trio	proband	yes	ASC	ASC		0,34
M_133	trio	mother	no	ASC	ASC	NA	
F_133	trio	father	no	ASC	ASC	NA	
P_134	trio	proband	yes	ASC	ASC		0,34
M_134	trio	mother	no	ASC	ASC	NA	
F_134	trio	father	no	ASC	ASC	NA	
P_135	trio	proband	yes	ASC	ASC		0,4
M_135	trio	mother	no	ASC	ASC	NA	
F_135	trio	father	no	ASC	ASC	NA	
P_136	complex	proband	yes	ASC	ASC		0,41
M_136	complex	mother	no	ASC	ASC	NA	
F_136	complex	father	no	ASC	ASC	NA	
P_137	trio	proband	yes	ASC	ASC		0,37
M_137	trio	mother	no	ASC	ASC	NA	
F_137	trio	father	no	ASC	ASC	NA	
P_138	trio	proband	yes	ASC	ASC		0,36
M_138	trio	mother	no	ASC	ASC	NA	
F_138	trio	father	no	ASC	ASC	NA	
P_139	duo	proband	yes	ASC	ASC	NA	
F_139	duo	father	no	ASC	ASC	NA	
P_140	trio	proband	yes	ASC	ASC		0,4
M_140	trio	mother	no	ASC	ASC	NA	
F_140	trio	father	no	ASC	ASC	NA	
P_141	trio	proband	yes	ASC	ASC		0,42
M_141	trio	mother	no	ASC	ASC	NA	

F_141	trio	father	no	ASC	ASC	NA	
P_142	trio	proband	yes	ASC	ASC		0,38
M_142	trio	mother	no	ASC	ASC	NA	
F_142	trio	father	no	ASC	ASC	NA	
P_143	trio	proband	yes	ASC	ASC		0,36
M_143	trio	mother	no	ASC	ASC	NA	
F_143	trio	father	no	ASC	ASC	NA	
P_144	trio	proband	yes	ASC	ASC		0,37
M_144	trio	mother	no	ASC	ASC	NA	
F_144	trio	father	no	ASC	ASC	NA	
P_145	trio	proband	yes	ASC	ASC		0,43
M_145	trio	mother	no	ASC	ASC	NA	
F_145	trio	father	no	ASC	ASC	NA	
P_146	trio	proband	yes	ASC	ASC		0,36
M_146	trio	mother	no	ASC	ASC	NA	
F_146	trio	father	no	ASC	ASC	NA	
P_147	single	proband	yes	CEGH	SureSelectXTV6	NA	
P_148	trio	proband	yes	ASC	ASC		0,47
M_148	trio	mother	NA	ASC	ASC	NA	
F_148	trio	father	NA	ASC	ASC	NA	
P_61	complex	proband	yes	CEGH	TruSeq		0,32
P_62	quartet	proband	yes	CEGH	TruSeq		0,29
P_149	trio	proband	yes	ASC	ASC		0,42
M_149	trio	mother	no	ASC	ASC	NA	
F_149	trio	father	no	ASC	ASC	NA	
P_150	mother + sons	proband	yes	CEGH	TruSeq		0,37
S_150	mother + sons	sibling	yes	CEGH	TruSeq	NA	
M_151	trio	mother	NA	CEGH	SureSelectXTV6	NA	
F_151	trio	father	NA	CEGH	SureSelectXTV6	NA	
P_152	duo	proband	yes	ASC	ASC		0,4
M_152	duo	mother	no	ASC	ASC	NA	
P_153	trio	proband	yes	ASC	ASC		0,41
M_153	trio	mother	no	ASC	ASC	NA	
F_153	trio	father	no	ASC	ASC	NA	
P_154	quartet	proband	yes	ASC	ASC		0,45
M_154	quartet	mother	NA	ASC	ASC	NA	
F_154	quartet	father	no	ASC	ASC	NA	
S_154	quartet	sibling	yes	ASC	ASC	NA	
P_155	duo	proband	yes	ASC	ASC	NA	
F_155	duo	father	no	ASC	ASC	NA	
P_156	trio	proband	yes	CEGH	SureSelectXTV6		0,41
M_156	trio	mother	no	CEGH	SureSelectXTV6	NA	
F_156	trio	father	no	CEGH	SureSelectXTV6	NA	
P_157	trio	proband	yes	ASC	ASC		0,42
M_157	trio	mother	no	ASC	ASC	NA	
F_157	trio	father	no	ASC	ASC	NA	
P_158	duo	proband	yes	ASC	ASC		0,47
M_158	duo	mother	no	ASC	ASC	NA	
M_159	trio	mother	no	CEGH	xgenV2	NA	
F_159	trio	father	no	CEGH	xgenV2	NA	
P_159	trio	proband	yes	CEGH	xgenV2		0,44
P_160	trio	proband	yes	ASC	ASC		0,46

M_160	trio	mother	no	ASC	ASC	NA		
F_160	trio	father	no	ASC	ASC	NA		
P_161	duo	proband	yes	ASC	ASC			0,39
M_161	duo	mother	no	ASC	ASC	NA		
P_162	duo	proband	yes	CEGH	SureSelectXTV6			0,44
M_162	duo	mother	no	CEGH	SureSelectXTV6	NA		
P_163	trio	proband	yes	ASC	ASC			0,47
M_163	trio	mother	no	ASC	ASC	NA		
F_163	trio	father	no	ASC	ASC	NA		
P_164	trio	proband	yes	ASC	ASC			0,37
M_164	trio	mother	no	ASC	ASC	NA		
F_164	trio	father	no	ASC	ASC	NA		
P_165	trio	proband	yes	ASC	ASC			0,46
M_165	trio	mother	no	ASC	ASC	NA		
F_165	trio	father	no	ASC	ASC	NA		
S_166	quartet	sibling	yes	ASC	ASC	NA		
S_101	quartet	sibling	yes	ASC	ASC	NA		
P_167	duo	proband	yes	ASC	ASC			0,46
M_167	duo	mother	no	ASC	ASC	NA		
P_168	trio	proband	yes	ASC	ASC			0,47
M_168	trio	mother	no	ASC	ASC	NA		
F_168	trio	father	no	ASC	ASC	NA		
P_169	trio	proband	yes	CEGH	xgenV2			0,4
M_169	trio	mother	NA	CEGH	xgenV2	NA		
F_169	trio	father	NA	CEGH	xgenV2	NA		
P_170	duo	proband	yes	ASC	ASC			0,43
M_170	duo	mother	no	ASC	ASC	NA		
P_171	trio	proband	yes	ASC	ASC			0,47
M_171	trio	mother	no	ASC	ASC	NA		
F_171	trio	father	no	ASC	ASC	NA		
O1_136	complex	cousin	yes	ASC	ASC	NA		
O2_136	complex	aunt	no	ASC	ASC	NA		
O3_136	complex	uncle	no	ASC	ASC	NA		
P_172	trio	proband	yes	ASC	ASC			0,4
M_172	trio	mother	no	ASC	ASC	NA		
F_172	trio	father	no	ASC	ASC	NA		
F_173	trio	father	NA	CEGH	xgenV2	NA		
P_174	trio	proband	yes	ASC	ASC			0,47
M_174	trio	mother	no	ASC	ASC	NA		
F_174	trio	father	no	ASC	ASC	NA		
P_175	trio	proband	yes	ASC	ASC			0,45
M_175	trio	mother	no	ASC	ASC	NA		
F_175	trio	father	no	ASC	ASC	NA		
P_176	complex	proband	yes	ASC	ASC			0,44
M_176	complex	mother	no	ASC	ASC	NA		
F_176	complex	father	no	ASC	ASC	NA		
S_176	complex	sibling	yes	ASC	ASC	NA		
O1_176	complex	cousin 1	yes	ASC	ASC	NA		
O2_176	complex	cousin 2	yes	ASC	ASC	NA		
P_177	trio	proband	yes	ASC	ASC			0,41
M_177	trio	mother	no	ASC	ASC	NA		
F_177	trio	father	no	ASC	ASC	NA		

P_178	trio	proband	yes	CEGH	xgenV2		0,43
M_178	trio	mother	no	CEGH	xgenV2	NA	
F_178	trio	father	no	CEGH	xgenV2	NA	
P_179	trio	proband	yes	ASC	ASC		0,46
M_179	trio	mother	no	ASC	ASC	NA	
F_179	trio	father	no	ASC	ASC	NA	
P_180	trio	proband	yes	ASC	ASC		0,48
M_180	trio	mother	no	ASC	ASC	NA	
F_180	trio	father	no	ASC	ASC	NA	
P_181	trio	proband	yes	ASC	ASC		0,36
M_181	trio	mother	no	ASC	ASC	NA	
F_181	trio	father	no	ASC	ASC	NA	
P_182	trio	proband	yes	ASC	ASC		0,41
M_182	trio	mother	no	ASC	ASC	NA	
F_182	trio	father	no	ASC	ASC	NA	
P_183	duo	proband	yes	ASC	ASC		0,37
M_183	duo	mother	no	ASC	ASC	NA	
P_184	trio	proband	yes	ASC	ASC		0,47
M_184	trio	mother	no	ASC	ASC	NA	
F_184	trio	father	no	ASC	ASC	NA	
P_185	duo	proband	yes	ASC	ASC		0,42
M_185	duo	mother	no	ASC	ASC	NA	
P_186	duo	proband	yes	ASC	ASC		0,41
M_186	duo	mother	no	ASC	ASC	NA	
P_187	trio	proband	yes	ASC	ASC		0,45
M_187	trio	mother	no	ASC	ASC	NA	
F_187	trio	father	no	ASC	ASC	NA	
S_28	quartet	sibling	yes	CEGH	xgenV1	NA	
P_188	trio	proband	yes	ASC	ASC		0,46
M_188	trio	mother	no	ASC	ASC	NA	
F_188	trio	father	no	ASC	ASC	NA	
P_189	duo	proband	yes	ASC	ASC		0,38
M_189	duo	mother	no	ASC	ASC	NA	
P_190	trio	proband	yes	ASC	ASC		0,42
M_190	trio	mother	no	ASC	ASC	NA	
F_190	trio	father	no	ASC	ASC	NA	
P_191	trio	proband	yes	ASC	ASC		0,53
M_191	trio	mother	no	ASC	ASC	NA	
F_191	trio	father	no	ASC	ASC	NA	
P_192	trio	proband	yes	ASC	ASC		0,43
M_192	trio	mother	no	ASC	ASC	NA	
F_192	trio	father	no	ASC	ASC	NA	
P_193	duo	proband	yes	ASC	ASC		0,45
M_193	duo	mother	no	ASC	ASC	NA	
P_194	duo	proband	yes	ASC	ASC		0,43
M_194	duo	mother	no	ASC	ASC	NA	
P_195	duo	proband	yes	ASC	ASC	NA	
F_195	duo	father	no	ASC	ASC	NA	
P_196	duo	proband	yes	ASC	ASC		0,44
M_196	duo	mother	no	ASC	ASC	NA	
P_197	duo	proband	yes	ASC	ASC		0,41
M_197	duo	mother	NA	ASC	ASC	NA	

P_198	trio	proband	yes	ASC	ASC		0,49
M_198	trio	mother	no	ASC	ASC	NA	
F_198	trio	father	NA	ASC	ASC	NA	
P_199	single	proband	yes	ASC	ASC	NA	
P_200	duo	proband	yes	ASC	ASC		0,42
M_200	duo	mother	no	ASC	ASC	NA	
P_201	duo	proband	yes	ASC	ASC		0,46
M_201	duo	mother	no	ASC	ASC	NA	
P_202	trio	proband	yes	ASC	ASC		0,43
M_202	trio	mother	no	ASC	ASC	NA	
F_202	trio	father	no	ASC	ASC	NA	
P_203	trio	proband	yes	ASC	ASC		0,38
M_203	trio	mother	no	ASC	ASC	NA	
F_203	trio	father	no	ASC	ASC	NA	
P_204	duo	proband	yes	ASC	ASC		0,4
M_204	duo	mother	no	ASC	ASC	NA	
P_205	duo	proband	yes	ASC	ASC		0,44
M_205	duo	mother	no	ASC	ASC	NA	
P_206	trio	proband	yes	ASC	ASC		0,46
M_206	trio	mother	no	ASC	ASC	NA	
F_206	trio	father	no	ASC	ASC	NA	
P_207	trio	proband	yes	ASC	ASC		0,51
M_207	trio	mother	no	ASC	ASC	NA	
F_207	trio	father	no	ASC	ASC	NA	
P_208	single	proband	yes	ASC	ASC	NA	
P_209	mother + sons	proband	yes	ASC	ASC		0,4
M_209	mother + sons	mother	no	ASC	ASC	NA	
S_209	mother + sons	sibling	yes	ASC	ASC	NA	
P_210	trio	proband	yes	ASC	ASC		0,42
M_210	trio	mother	no	ASC	ASC	NA	
F_210	trio	father	no	ASC	ASC	NA	
P_211	duo	proband	yes	ASC	ASC		0,49
M_211	duo	mother	no	ASC	ASC	NA	
P_212	trio	proband	yes	ASC	ASC		0,47
M_212	trio	mother	no	ASC	ASC	NA	
F_212	trio	father	no	ASC	ASC	NA	
F_213	trio	father	NA	CEGH	xgenV1	NA	
P_214	single	proband	yes	CEGH	xgenV1	NA	
P_215	trio	proband	yes	ASC	ASC		0,42
M_215	trio	mother	no	ASC	ASC	NA	
F_215	trio	father	no	ASC	ASC	NA	
P_216	trio	proband	yes	ASC	ASC		0,46
M_216	trio	mother	no	ASC	ASC	NA	
F_216	trio	father	no	ASC	ASC	NA	
P_217	duo	proband	yes	ASC	ASC	NA	
F_217	duo	father	no	ASC	ASC	NA	
P_218	single	proband	yes	ASC	ASC	NA	
P_219	duo	proband	yes	ASC	ASC		0,45
M_219	duo	mother	no	ASC	ASC	NA	
P_220	trio	proband	yes	ASC	ASC		0,39
M_220	trio	mother	no	ASC	ASC	NA	
F_220	trio	father	no	ASC	ASC	NA	

P_221	trio	proband	yes	ASC	ASC		0,45
M_221	trio	mother	no	ASC	ASC	NA	
F_221	trio	father	no	ASC	ASC	NA	
P_222	duo	proband	yes	ASC	ASC		0,44
M_222	duo	mother	NA	ASC	ASC	NA	
P_223	trio	proband	yes	ASC	ASC		0,49
M_223	trio	mother	no	ASC	ASC	NA	
F_223	trio	father	no	ASC	ASC	NA	
P_224	trio	proband	yes	ASC	ASC		0,51
M_224	trio	mother	no	ASC	ASC	NA	
F_224	trio	father	no	ASC	ASC	NA	
P_225	trio	proband	yes	ASC	ASC		0,44
M_225	trio	mother	no	ASC	ASC	NA	
F_225	trio	father	no	ASC	ASC	NA	
P_226	trio	proband	yes	ASC	ASC		0,46
M_226	trio	mother	no	ASC	ASC	NA	
F_226	trio	father	no	ASC	ASC	NA	
P_227	trio	proband	yes	ASC	ASC		0,46
M_227	trio	mother	no	ASC	ASC	NA	
F_227	trio	father	no	ASC	ASC	NA	
P_228	trio	proband	yes	ASC	ASC		0,45
M_228	trio	mother	no	ASC	ASC	NA	
F_228	trio	father	no	ASC	ASC	NA	
P_229	trio	proband	yes	ASC	ASC		0,49
M_229	trio	mother	no	ASC	ASC	NA	
F_229	trio	father	no	ASC	ASC	NA	
P_230	trio	proband	yes	ASC	ASC		0,47
M_230	trio	mother	no	ASC	ASC	NA	
F_230	trio	father	no	ASC	ASC	NA	
P_231	trio	proband	yes	ASC	ASC		0,42
M_231	trio	mother	no	ASC	ASC	NA	
F_231	trio	father	no	ASC	ASC	NA	
P_232	trio	proband	yes	ASC	ASC		0,46
M_232	trio	mother	no	ASC	ASC	NA	
F_232	trio	father	no	ASC	ASC	NA	
P_233	duo	proband	yes	ASC	ASC		0,43
M_233	duo	mother	no	ASC	ASC	NA	
M_234	mother + sons	mother	yes	ASC	ASC	NA	
S_234	mother + sons	sibling	no	ASC	ASC	NA	
P_234	mother + sons	proband	yes	ASC	ASC		0,48
P_235	single	proband	yes	CEGH	xgenV2	NA	
P_236	trio	proband	yes	ASC	Nextera		0,36
F_236	trio	father	no	ASC	Nextera	NA	
M_236	trio	mother	no	ASC	Nextera	NA	
P_237	trio	proband	yes	ASC	Nextera		0,38
M_237	trio	mother	no	ASC	Nextera	NA	
F_237	trio	father	no	ASC	Nextera	NA	
P_238	trio	proband	yes	ASC	Nextera		0,44
M_238	trio	mother	no	ASC	Nextera	NA	
F_238	trio	father	no	ASC	Nextera	NA	
P_239	trio	proband	yes	ASC	Nextera		0,39
M_239	trio	mother	no	ASC	Nextera	NA	

F_239	trio	father	NA	ASC	Nextera	NA	
P_240	trio	proband	yes	ASC	Nextera		0,42
M_240	trio	mother	no	ASC	Nextera	NA	
F_240	trio	father	no	ASC	Nextera	NA	
P_241	trio	proband	yes	ASC	Nextera		0,34
M_241	trio	mother	no	ASC	Nextera	NA	
F_241	trio	father	no	ASC	Nextera	NA	
P_242	trio	proband	yes	ASC	Nextera		0,5
M_242	trio	mother	no	ASC	Nextera	NA	
F_242	trio	father	no	ASC	Nextera	NA	
P_243	trio	proband	yes	ASC	Nextera		0,36
M_243	trio	mother	no	ASC	Nextera	NA	
F_243	trio	father	no	ASC	Nextera	NA	
P_244	trio	proband	yes	ASC	Nextera		0,41
M_244	trio	mother	no	ASC	Nextera	NA	
F_244	trio	father	no	ASC	Nextera	NA	
M_245	trio	mother	no	ASC	Nextera	NA	
F_245	trio	father	no	ASC	Nextera	NA	
P_245	trio	proband	yes	ASC	Nextera		0,36
P_246	trio	proband	yes	ASC	ASC		0,45
M_246	trio	mother	no	ASC	ASC	NA	
F_246	trio	father	no	ASC	ASC	NA	
P_247	trio	proband	yes	ASC	Nextera		0,4
M_247	trio	mother	no	ASC	Nextera	NA	
F_247	trio	father	no	ASC	Nextera	NA	
P_248	trio	proband	yes	ASC	Nextera		0,4
M_248	trio	mother	no	ASC	Nextera	NA	
F_248	trio	father	no	ASC	Nextera	NA	
P_249	trio	proband	yes	ASC	ASC		0,38
M_249	trio	mother	no	ASC	ASC	NA	
F_249	trio	father	no	ASC	ASC	NA	
P_250	trio	proband	yes	ASC	Nextera		0,33
M_250	trio	mother	no	ASC	Nextera	NA	
F_250	trio	father	no	ASC	Nextera	NA	
P_251	trio	proband	yes	ASC	Nextera		0,48
M_251	trio	mother	no	ASC	Nextera	NA	
F_251	trio	father	no	ASC	Nextera	NA	
P_252	trio	proband	yes	ASC	ASC		0,4
M_252	trio	mother	no	ASC	ASC	NA	
F_252	trio	father	no	ASC	ASC	NA	
P_253	trio	proband	yes	CEGH	SureSelectXTV6		0,39
M_253	trio	mother	no	CEGH	SureSelectXTV6	NA	
F_253	trio	father	no	CEGH	SureSelectXTV6	NA	
P_254	trio	proband	yes	ASC	ASC		0,4
M_254	trio	mother	no	ASC	ASC	NA	
F_254	trio	father	no	ASC	ASC	NA	
P_255	trio	proband	yes	ASC	Nextera		0,33
M_255	trio	mother	no	ASC	Nextera	NA	
F_255	trio	father	no	ASC	Nextera	NA	
P_256	trio	proband	yes	ASC	Nextera		0,43
M_256	trio	mother	no	ASC	Nextera	NA	
F_256	trio	father	no	ASC	Nextera	NA	

P_257	trio	proband	yes	ASC	Nextera		0,44
M_257	trio	mother	no	ASC	Nextera	NA	
F_257	trio	father	no	ASC	Nextera	NA	
P_258	trio	proband	yes	ASC	ASC		0,43
M_258	trio	mother	no	ASC	ASC	NA	
F_258	trio	father	no	ASC	ASC	NA	
P_259	trio	proband	yes	ASC	Nextera		0,43
M_259	trio	mother	no	ASC	Nextera	NA	
F_259	trio	father	no	ASC	Nextera	NA	
P_260	trio	proband	yes	CEGH	SureSelectXTV6		0,33
M_260	trio	mother	no	CEGH	SureSelectXTV6	NA	
F_260	trio	father	NA	CEGH	SureSelectXTV6	NA	
P_261	trio	proband	yes	ASC	Nextera		0,41
M_261	trio	mother	no	ASC	Nextera	NA	
F_261	trio	father	no	ASC	Nextera	NA	
P_262	trio	proband	yes	ASC	Nextera		0,37
M_262	trio	mother	no	ASC	Nextera	NA	
F_262	trio	father	no	ASC	Nextera	NA	
P_263	trio	proband	yes	ASC	Nextera		0,42
M_263	trio	mother	no	ASC	Nextera	NA	
F_263	trio	father	no	ASC	Nextera	NA	
P_264	trio	proband	yes	ASC	ASC		0,44
M_264	trio	mother	no	ASC	ASC	NA	
F_264	trio	father	no	ASC	ASC	NA	
P_265	trio	proband	yes	ASC	Nextera		0,37
M_265	trio	mother	no	ASC	Nextera	NA	
F_265	trio	father	no	ASC	Nextera	NA	
P_266	trio	proband	yes	ASC	Nextera		0,39
M_266	trio	mother	no	ASC	Nextera	NA	
F_266	trio	father	no	ASC	Nextera	NA	
P_267	quartet	proband	yes	ASC	Nextera		0,4
M_267	quartet	mother	no	ASC	Nextera	NA	
F_267	quartet	father	no	ASC	Nextera	NA	
S_267	quartet	sibling	yes	ASC	Nextera	NA	
P_268	trio	proband	yes	ASC	Nextera		0,36
M_268	trio	mother	no	ASC	Nextera	NA	
F_268	trio	father	no	ASC	Nextera	NA	
P_269	trio	proband	yes	ASC	Nextera		0,35
M_269	trio	mother	no	ASC	Nextera	NA	
F_269	trio	father	no	ASC	Nextera	NA	
P_270	trio	proband	yes	ASC	Nextera		0,44
M_270	trio	mother	no	ASC	Nextera	NA	
F_270	trio	father	no	ASC	Nextera	NA	
P_271	trio	proband	yes	ASC	Nextera		0,39
M_271	trio	mother	no	ASC	Nextera	NA	
F_271	trio	father	no	ASC	Nextera	NA	
P_272	trio	proband	yes	ASC	Nextera		0,48
M_272	trio	mother	no	ASC	Nextera	NA	
F_272	trio	father	no	ASC	Nextera	NA	
P_273	trio	proband	yes	ASC	Nextera		0,38
M_273	trio	mother	no	ASC	Nextera	NA	
F_273	trio	father	no	ASC	Nextera	NA	

P_274	trio	proband	yes	ASC	Nextera		0,37
M_274	trio	mother	no	ASC	Nextera	NA	
F_274	trio	father	no	ASC	Nextera	NA	
P_275	trio	proband	yes	ASC	Nextera		0,34
M_275	trio	mother	no	ASC	Nextera	NA	
F_275	trio	father	no	ASC	Nextera	NA	
P_276	trio	proband	yes	CEGH	SureSelectXTV6		0,37
M_276	trio	mother	no	CEGH	SureSelectXTV6	NA	
F_276	trio	father	no	CEGH	SureSelectXTV6	NA	
P_277	trio	proband	yes	ASC	ASC		0,44
M_277	trio	mother	no	ASC	ASC	NA	
F_277	trio	father	no	ASC	ASC	NA	
P_278	trio	proband	yes	ASC	ASC		0,36
M_278	trio	mother	no	ASC	ASC	NA	
F_278	trio	father	no	ASC	ASC	NA	
P_279	single	proband	yes	CEGH	xgenV1	NA	
P_280	trio	proband	yes	CEGH	SureSelectXTV6		0,37
M_280	trio	mother	no	CEGH	SureSelectXTV6	NA	
F_280	trio	father	no	CEGH	SureSelectXTV6	NA	
P_281	trio	proband	yes	CEGH	SureSelectXTV6		0,35
M_281	trio	mother	no	CEGH	SureSelectXTV6	NA	
F_281	trio	father	no	CEGH	SureSelectXTV6	NA	
P_282	trio	proband	yes	ASC	ASC		0,42
M_282	trio	mother	no	ASC	ASC	NA	
F_282	trio	father	no	ASC	ASC	NA	
P_283	duo	proband	yes	CEGH	SureSelectXTV6		0,44
M_283	duo	mother	no	CEGH	SureSelectXTV6	NA	
P_284	trio	proband	yes	CEGH	SureSelectXTV6		0,4
M_284	trio	mother	no	CEGH	SureSelectXTV6	NA	
F_284	trio	father	no	CEGH	SureSelectXTV6	NA	
P_285	trio	proband	yes	ASC	ASC		0,35
M_285	trio	mother	no	ASC	ASC	NA	
F_285	trio	father	no	ASC	ASC	NA	
P_286	trio	proband	yes	ASC	ASC		0,39
M_286	trio	mother	no	ASC	ASC	NA	
F_286	trio	father	no	ASC	ASC	NA	
P_287	trio	proband	yes	ASC	ASC		0,42
M_287	trio	mother	no	ASC	ASC	NA	
F_287	trio	father	no	ASC	ASC	NA	
P_288	trio	proband	yes	CEGH	SureSelectXTV6		0,47
M_288	trio	mother	no	CEGH	SureSelectXTV6	NA	
F_288	trio	father	no	CEGH	SureSelectXTV6	NA	
P_289	trio	proband	yes	CEGH	SureSelectXTV6		0,45
M_289	trio	mother	no	CEGH	SureSelectXTV6	NA	
F_289	trio	father	no	CEGH	SureSelectXTV6	NA	
P_290	trio	proband	yes	ASC	ASC		0,42
M_290	trio	mother	no	ASC	ASC	NA	
F_290	trio	father	no	ASC	ASC	NA	
P_291	trio	proband	yes	CEGH	SureSelectXTV6		0,37
M_291	trio	mother	no	CEGH	SureSelectXTV6	NA	
F_291	trio	father	no	CEGH	SureSelectXTV6	NA	
P_292	trio	proband	yes	ASC	ASC		0,45

M_292	trio	mother	no	ASC	ASC	NA	
F_292	trio	father	no	ASC	ASC	NA	
P_293	trio	proband	yes	ASC	ASC		0,4
M_293	trio	mother	no	ASC	ASC	NA	
F_293	trio	father	no	ASC	ASC	NA	
P_294	trio	proband	yes	ASC	ASC		0,41
M_294	trio	mother	no	ASC	ASC	NA	
F_294	trio	father	no	ASC	ASC	NA	
P_295	trio	proband	yes	ASC	ASC		0,4
M_295	trio	mother	no	ASC	ASC	NA	
F_295	trio	father	no	ASC	ASC	NA	
P_296	trio	proband	yes	CEGH	SureSelectXTV6		0,38
M_296	trio	mother	no	CEGH	SureSelectXTV6	NA	
F_296	trio	father	no	CEGH	SureSelectXTV6	NA	
P_297	trio	proband	yes	ASC	ASC		0,44
M_297	trio	mother	no	ASC	ASC	NA	
F_297	trio	father	no	ASC	ASC	NA	
P_298	trio	proband	yes	ASC	ASC		0,38
M_298	trio	mother	no	ASC	ASC	NA	
F_298	trio	father	no	ASC	ASC	NA	
P_299	trio	proband	yes	ASC	ASC		0,39
M_299	trio	mother	no	ASC	ASC	NA	
F_299	trio	father	no	ASC	ASC	NA	
P_300	trio	proband	yes	ASC	ASC		0,42
M_300	trio	mother	no	ASC	ASC	NA	
F_300	trio	father	no	ASC	ASC	NA	
P_301	trio	proband	yes	CEGH	SureSelectXTV6		0,38
M_301	trio	mother	no	CEGH	SureSelectXTV6	NA	
F_301	trio	father	no	CEGH	SureSelectXTV6	NA	
P_302	trio	proband	yes	ASC	ASC		0,4
M_302	trio	mother	no	ASC	ASC	NA	
F_302	trio	father	no	ASC	ASC	NA	
P_303	trio	proband	yes	CEGH	SureSelectXTV6		0,42
M_303	trio	mother	no	CEGH	SureSelectXTV6	NA	
F_303	trio	father	no	CEGH	SureSelectXTV6	NA	
P_304	trio	proband	yes	ASC	ASC		0,39
M_304	trio	mother	no	ASC	ASC	NA	
F_304	trio	father	no	ASC	ASC	NA	
P_305	trio	proband	yes	CEGH	SureSelectXTV6		0,38
M_305	trio	mother	no	CEGH	SureSelectXTV6	NA	
F_305	trio	father	no	CEGH	SureSelectXTV6	NA	
P_306	trio	proband	yes	CEGH	SureSelectXTV6		0,38
M_306	trio	mother	no	CEGH	SureSelectXTV6	NA	
F_306	trio	father	no	CEGH	SureSelectXTV6	NA	
P_307	trio	proband	yes	ASC	ASC		0,42
M_307	trio	mother	no	ASC	ASC	NA	
F_307	trio	father	no	ASC	ASC	NA	
P_308	trio	proband	yes	ASC	ASC		0,38
M_308	trio	mother	no	ASC	ASC	NA	
F_308	trio	father	no	ASC	ASC	NA	
P_309	trio	proband	yes	ASC	ASC		0,39
M_309	trio	mother	no	ASC	ASC	NA	

F_309	trio	father	no	ASC	ASC	NA	
P_310	trio	proband	yes	ASC	ASC		0,33
M_310	trio	mother	no	ASC	ASC	NA	
F_310	trio	father	no	ASC	ASC	NA	
P_311	trio	proband	yes	CEGH	SureSelectXTV6		0,36
M_311	trio	mother	no	CEGH	SureSelectXTV6	NA	
F_311	trio	father	no	CEGH	SureSelectXTV6	NA	
P_312	trio	proband	yes	ASC	ASC		0,41
M_312	trio	mother	no	ASC	ASC	NA	
F_312	trio	father	no	ASC	ASC	NA	
P_313	trio	proband	yes	ASC	ASC		0,41
M_313	trio	mother	no	ASC	ASC	NA	
F_313	trio	father	no	ASC	ASC	NA	
P_166	quartet	proband	yes	CEGH	SureSelectXTV6		0,41
M_166	quartet	mother	NA	CEGH	SureSelectXTV6	NA	
F_166	quartet	father	NA	CEGH	SureSelectXTV6	NA	
P_314	trio	proband	yes	CEGH	SureSelectXTV6		0,38
M_314	trio	mother	no	CEGH	SureSelectXTV6	NA	
F_314	trio	father	no	CEGH	SureSelectXTV6	NA	
P_315	trio	proband	yes	CEGH	SureSelectXTV6		0,39
M_315	trio	mother	no	CEGH	SureSelectXTV6	NA	
F_315	trio	father	no	CEGH	SureSelectXTV6	NA	
P_316	trio	proband	yes	CEGH	SureSelectXTV6		0,48
M_316	trio	mother	no	CEGH	SureSelectXTV6	NA	
F_316	trio	father	no	CEGH	SureSelectXTV6	NA	
P_317	trio	proband	yes	CEGH	SureSelectXTV6		0,4
M_317	trio	mother	no	CEGH	SureSelectXTV6	NA	
F_317	trio	father	no	CEGH	SureSelectXTV6	NA	
P_318	trio	proband	yes	CEGH	SureSelectXTV6		0,4
M_318	trio	mother	no	CEGH	SureSelectXTV6	NA	
F_318	trio	father	no	CEGH	SureSelectXTV6	NA	
P_319	trio	proband	yes	CEGH	SureSelectXTV6		0,44
M_319	trio	mother	no	CEGH	SureSelectXTV6	NA	
F_319	trio	father	no	CEGH	SureSelectXTV6	NA	
P_320	trio	proband	yes	CEGH	SureSelectXTV6		0,42
M_320	trio	mother	no	CEGH	SureSelectXTV6	NA	
F_320	trio	father	no	CEGH	SureSelectXTV6	NA	
P_321	trio	proband	yes	CEGH	SureSelectXTV6		0,3
M_321	trio	mother	no	CEGH	SureSelectXTV6	NA	
F_321	trio	father	no	CEGH	SureSelectXTV6	NA	
P_322	trio	proband	yes	CEGH	SureSelectXTV6		0,38
M_322	trio	mother	no	CEGH	SureSelectXTV6	NA	
F_322	trio	father	no	CEGH	SureSelectXTV6	NA	
P_173	trio	proband	yes	CEGH	xgenV2		0,34
M_173	trio	mother	NA	CEGH	xgenV2	NA	
P_323	trio	proband	yes	CEGH	SureSelectXTV6		0,37
M_323	trio	mother	no	CEGH	SureSelectXTV6	NA	
F_323	trio	father	no	CEGH	SureSelectXTV6	NA	
P_324	trio	proband	yes	CEGH	SureSelectXTV6		0,36
M_324	trio	mother	no	CEGH	SureSelectXTV6	NA	
F_324	trio	father	no	CEGH	SureSelectXTV6	NA	
P_325	trio	proband	yes	CEGH	SureSelectXTV6		0,37

M_325	trio	mother	no	CEGH	SureSelectXTV6	NA	
F_325	trio	father	no	CEGH	SureSelectXTV6	NA	
P_326	trio	proband	yes	CEGH	SureSelectXTV6		0,35
M_326	trio	mother	no	CEGH	SureSelectXTV6	NA	
F_326	trio	father	no	CEGH	SureSelectXTV6	NA	
P_327	trio	proband	yes	CEGH	SureSelectXTV6		0,38
M_327	trio	mother	no	CEGH	SureSelectXTV6	NA	
F_327	trio	father	no	CEGH	SureSelectXTV6	NA	
P_328	quartet	proband	yes	CEGH	SureSelectXTV6		0,49
M_328	quartet	mother	NA	CEGH	SureSelectXTV6	NA	
F_328	quartet	father	NA	CEGH	SureSelectXTV6	NA	
S_328	quartet	sibling	yes	CEGH	SureSelectXTV6	NA	
P_329	trio	proband	yes	CEGH	SureSelectXTV6		0,32
M_329	trio	mother	no	CEGH	SureSelectXTV6	NA	
F_329	trio	father	no	CEGH	SureSelectXTV6	NA	
P_213	trio	proband	yes	CEGH	xgenV1		0,44
M_213	trio	mother	NA	CEGH	xgenV1	NA	
P_330	single	proband	yes	CEGH	xgenV1	NA	
P_331	single	proband	yes	CEGH	SureSelectXTV6	NA	
P_332	trio	proband	yes	CEGH	SureSelectXTV6		0,4
M_332	trio	mother	no	CEGH	SureSelectXTV6	NA	
F_332	trio	father	no	CEGH	SureSelectXTV6	NA	
P_333	trio	proband	yes	ASC	Nextera		0,47
M_333	trio	mother	no	ASC	Nextera	NA	
F_333	trio	father	no	ASC	Nextera	NA	
P_334	trio	proband	yes	ASC	Nextera		0,42
M_334	trio	mother	no	ASC	Nextera	NA	
F_334	trio	father	no	ASC	Nextera	NA	
P_335	trio	proband	yes	CEGH	SureSelectXTV6		0,38
M_335	trio	mother	no	CEGH	SureSelectXTV6	NA	
F_335	trio	father	no	CEGH	SureSelectXTV6	NA	
P_336	trio	proband	yes	CEGH	SureSelectXTV6		0,41
M_336	trio	mother	no	CEGH	SureSelectXTV6	NA	
F_336	trio	father	no	CEGH	SureSelectXTV6	NA	
P_337	trio	proband	yes	CEGH	TruSeq		0,39
M_337	trio	mother	no	CEGH	TruSeq	NA	
F_337	trio	father	no	CEGH	TruSeq	NA	
P_338	trio	proband	yes	CEGH	SureSelectXTV6		0,35
M_338	trio	mother	no	CEGH	SureSelectXTV6	NA	
F_338	trio	father	no	CEGH	SureSelectXTV6	NA	
P_339	mother + sons	proband	yes	ASC	ASC		0,39
S_339	mother + sons	sibling	yes	ASC	ASC	NA	
M_339	mother + sons	mother	no	ASC	ASC	NA	
P_340	trio	proband	yes	ASC	Nextera		0,48
M_340	trio	mother	no	ASC	Nextera	NA	
F_340	trio	father	no	ASC	Nextera	NA	
P_341	duo	proband	yes	ASC	ASC		0,4
M_341	duo	mother	NA	ASC	ASC	NA	
P_342	trio	proband	yes	ASC	Nextera		0,41
M_342	trio	mother	no	ASC	Nextera	NA	
F_342	trio	father	no	ASC	Nextera	NA	
P_343	trio	proband	yes	ASC	Nextera		0,37

M_343	trio	mother	no	ASC	Nextera	NA	
F_343	trio	father	no	ASC	Nextera	NA	
P_344	trio	proband	yes	ASC	Nextera		0,38
M_344	trio	mother	no	ASC	Nextera	NA	
F_344	trio	father	no	ASC	Nextera	NA	
P_345	trio	proband	yes	ASC	Nextera		0,45
M_345	trio	mother	no	ASC	Nextera	NA	
F_345	trio	father	no	ASC	Nextera	NA	
P_346	trio	proband	yes	CEGH	TruSeq		0,41
M_346	trio	mother	no	CEGH	TruSeq	NA	
F_346	trio	father	no	CEGH	TruSeq	NA	
P_347	single	proband	yes	CEGH	TruSeq	NA	
P_348	trio	proband	yes	CEGH	SureSelectXTV6		0,33
M_348	trio	mother	no	CEGH	SureSelectXTV6	NA	
F_348	trio	father	no	CEGH	SureSelectXTV6	NA	
P_349	trio	proband	yes	CEGH	SureSelectXTV6		0,42
M_349	trio	mother	no	CEGH	SureSelectXTV6	NA	
F_349	trio	father	no	CEGH	SureSelectXTV6	NA	
P_350	trio	proband	yes	CEGH	Nextera		0,3
M_350	trio	mother	NA	CEGH	Nextera	NA	
F_350	trio	father	NA	CEGH	Nextera	NA	
P_351	trio	proband	yes	CEGH	TruSeq		0,39
M_351	trio	mother	NA	CEGH	TruSeq	NA	
F_351	trio	father	NA	CEGH	TruSeq	NA	
P_352	trio	proband	yes	CEGH	Nextera		0,44
M_352	trio	mother	NA	CEGH	Nextera	NA	
F_352	trio	father	NA	CEGH	Nextera	NA	
P_353	duo	proband	yes	CEGH	Nextera	NA	
F_353	duo	father	NA	CEGH	Nextera	NA	
P_354	duo	proband	yes	CEGH	Nextera		0,37
M_354	duo	mother	NA	CEGH	Nextera	NA	
P_355	trio	proband	yes	CEGH	Nextera		0,39
M_355	trio	mother	NA	CEGH	Nextera	NA	
F_355	trio	father	NA	CEGH	Nextera	NA	
P_356	trio	proband	yes	CEGH	Nextera		0,32
M_356	trio	mother	NA	CEGH	Nextera	NA	
F_356	trio	father	NA	CEGH	Nextera	NA	
P_357	quartet	proband	yes	CEGH	Nextera		0,39
M_357	quartet	mother	NA	CEGH	Nextera	NA	
F_357	quartet	father	NA	CEGH	Nextera	NA	
S_357	quartet	sibling	yes	CEGH	Nextera	NA	
P_358	duo	proband	yes	ASC	ASC	NA	
F_358	duo	father	NA	ASC	ASC	NA	
P_359	complex	proband	yes	CEGH	TruSeq		0,32
M_359	complex	mother	no	CEGH	SureSelectXTV6	NA	
F_359	complex	father	no	CEGH	SureSelectXTV6	NA	
O1_359	complex	cousin	yes	CEGH	TruSeq	NA	
O2_359	complex	aunt	no	CEGH	SureSelectXTV6	NA	
O3_359	complex	uncle	no	CEGH	SureSelectXTV6	NA	
P_360	trio	proband	yes	CEGH	Nextera		0,34
M_360	trio	mother	NA	CEGH	Nextera	NA	
F_360	trio	father	NA	CEGH	Nextera	NA	

P_361	trio	proband	yes	CEGH	SureSelectXTV6		0,31
M_361	trio	mother	no	CEGH	SureSelectXTV6	NA	
F_361	trio	father	no	CEGH	SureSelectXTV6	NA	
P_362	trio	proband	yes	CEGH	SureSelectXTV6		0,33
M_362	trio	mother	no	CEGH	SureSelectXTV6	NA	
F_362	trio	father	no	CEGH	SureSelectXTV6	NA	
P_363	trio	proband	yes	CEGH	SureSelectXTV6		0,31
M_363	trio	mother	no	CEGH	SureSelectXTV6	NA	
F_363	trio	father	no	CEGH	SureSelectXTV6	NA	
P_364	trio	proband	yes	CEGH	SureSelectXTV6		0,37
M_364	trio	mother	no	CEGH	SureSelectXTV6	NA	
F_364	trio	father	no	CEGH	SureSelectXTV6	NA	
P_365	trio	proband	yes	CEGH	SureSelectXTV6		0,32
M_365	trio	mother	no	CEGH	SureSelectXTV6	NA	
F_365	trio	father	no	CEGH	SureSelectXTV6	NA	
P_366	trio	proband	yes	ASC	Nextera		0,41
M_366	trio	mother	no	ASC	Nextera	NA	
F_366	trio	father	no	ASC	Nextera	NA	
P_367	quartet	proband	yes	CEGH	SureSelectXTV6		0,37
M_367	quartet	mother	NA	CEGH	SureSelectXTV6	NA	
F_367	quartet	father	NA	CEGH	SureSelectXTV6	NA	
S1_368	quartet	sibling	yes	CEGH	Nextera	NA	
P_369	trio	proband	yes	CEGH	SureSelectXTV6		0,28
M_369	trio	mother	no	CEGH	SureSelectXTV6	NA	
F_369	trio	father	no	CEGH	SureSelectXTV6	NA	
P_370	complex	proband	yes	CEGH	TruSeq		0,31
M_370	complex	mother	no	CEGH	SureSelectXTV6	NA	
O1_370	complex	cousin	yes	CEGH	TruSeq	NA	
O2_370	complex	aunt	no	CEGH	SureSelectXTV6	NA	
P_371	trio	proband	yes	CEGH	SureSelectXTV6		0,44
M_371	trio	mother	no	CEGH	SureSelectXTV6	NA	
F_371	trio	father	no	CEGH	SureSelectXTV6	NA	
P_372	trio	proband	yes	CEGH	SureSelectXTV6		0,42
M_372	trio	mother	no	CEGH	SureSelectXTV6	NA	
F_372	trio	father	no	CEGH	SureSelectXTV6	NA	
P_373	trio	proband	yes	ASC	Nextera		0,41
M_373	trio	mother	no	ASC	Nextera	NA	
F_373	trio	father	no	ASC	Nextera	NA	
P_374	trio	proband	yes	ASC	Nextera		0,4
M_374	trio	mother	no	ASC	Nextera	NA	
F_374	trio	father	no	ASC	Nextera	NA	
P_375	trio	proband	yes	CEGH	SureSelectXTV6		0,47
M_375	trio	mother	no	CEGH	SureSelectXTV6	NA	
F_375	trio	father	no	CEGH	SureSelectXTV6	NA	
P_368	quartet	proband	yes	ASC	Nextera		0,46
M_368	quartet	mother	NA	ASC	Nextera	NA	
F_368	quartet	father	NA	ASC	Nextera	NA	
S2_368	quartet	sibling	yes	ASC	Nextera	NA	
P_376	quartet	proband	yes	CEGH	SureSelectXTV6		0,51
M_376	quartet	mother	NA	CEGH	SureSelectXTV6	NA	
F_376	quartet	father	NA	CEGH	SureSelectXTV6	NA	
S_376	quartet	sibling	yes	CEGH	TruSeq	NA	

P_377	trio	proband	yes	CEGH	SureSelectXTV6		0,38
M_377	trio	mother	no	CEGH	SureSelectXTV6	NA	
F_377	trio	father	no	CEGH	SureSelectXTV6	NA	
P_378	trio	proband	yes	CEGH	Nextera		0,33
M_378	trio	mother	NA	CEGH	Nextera	NA	
F_378	trio	father	NA	CEGH	Nextera	NA	
P_379	trio	proband	yes	CEGH	Nextera		0,33
M_379	trio	mother	NA	CEGH	Nextera	NA	
F_379	trio	father	NA	CEGH	Nextera	NA	
P_380	trio	proband	yes	ASC	Nextera		0,4
M_380	trio	mother	no	ASC	Nextera	NA	
F_380	trio	father	no	ASC	Nextera	NA	
P_381	trio	proband	yes	ASC	Nextera		0,43
M_381	trio	mother	no	ASC	Nextera	NA	
F_381	trio	father	no	ASC	Nextera	NA	
P_382	trio	proband	yes	CEGH	SureSelectXTV6		0,35
M_382	trio	mother	no	CEGH	SureSelectXTV6	NA	
F_382	trio	father	no	CEGH	SureSelectXTV6	NA	
P_383	trio	proband	yes	CEGH	SureSelectXTV6		0,36
M_383	trio	mother	no	CEGH	SureSelectXTV6	NA	
F_383	trio	father	no	CEGH	SureSelectXTV6	NA	
P_384	trio	proband	yes	CEGH	SureSelectXTV6		0,41
M_384	trio	mother	no	CEGH	SureSelectXTV6	NA	
F_384	trio	father	no	CEGH	SureSelectXTV6	NA	
P_385	trio	proband	yes	CEGH	SureSelectXTV6		0,35
M_385	trio	mother	no	CEGH	SureSelectXTV6	NA	
F_385	trio	father	no	CEGH	SureSelectXTV6	NA	
P_386	trio	proband	yes	CEGH	TruSeq		0,43
M_386	trio	mother	NA	CEGH	TruSeq	NA	
F_386	trio	father	NA	CEGH	TruSeq	NA	
P_387	duo	proband	yes	CEGH	Nextera		0,26
M_387	duo	mother	no	CEGH	Nextera	NA	
P_388	trio	proband	yes	ASC	Nextera		0,42
M_388	trio	mother	no	ASC	Nextera	NA	
F_388	trio	father	no	ASC	Nextera	NA	
P_389	duo	proband	yes	ASC	ASC		0,39
M_389	duo	mother	NA	ASC	ASC	NA	
P_390	trio	proband	yes	ASC	Nextera		0,39
M_390	trio	mother	no	ASC	Nextera	NA	
F_390	trio	father	no	ASC	Nextera	NA	
P_391	trio	proband	yes	CEGH	SureSelectXTV6		0,34
M_391	trio	mother	no	CEGH	SureSelectXTV6	NA	
F_391	trio	father	NA	CEGH	SureSelectXTV6	NA	
P_392	trio	proband	yes	CEGH	SureSelectXTV6		0,45
M_392	trio	mother	no	CEGH	SureSelectXTV6	NA	
F_392	trio	father	no	CEGH	SureSelectXTV6	NA	
P_393	trio	proband	yes	ASC	Nextera		0,35
M_393	trio	mother	no	ASC	Nextera	NA	
F_393	trio	father	no	ASC	Nextera	NA	
P_394	trio	proband	yes	ASC	Nextera		0,44
M_394	trio	mother	no	ASC	Nextera	NA	
F_394	trio	father	no	ASC	Nextera	NA	

S1_395	complex	half-sibling	yes	CEGH	TruSeq	NA	
M_395	complex	mother	no	CEGH	SureSelectXTV6	NA	
F_395	complex	father	no	CEGH	SureSelectXTV6	NA	
P_395	complex	proband	yes	CEGH	TruSeq		0,29
S2_395	complex	sibling	yes	CEGH	TruSeq	NA	
M_61	complex	mother	no	CEGH	SureSelectXTV6	NA	
F_61	complex	father	no	CEGH	SureSelectXTV6	NA	
S2_61	complex	sibling 2	no	CEGH	SureSelectXTV6	NA	
M_62	quartet	mother	NA	CEGH	SureSelectXTV6	NA	
F_62	quartet	father	NA	CEGH	SureSelectXTV6	NA	
F_396	complex	father	NA	CEGH	TruSeq	NA	
P_396	complex	proband	yes	CEGH	TruSeq		0,29
M_396	complex	mother	no	CEGH	SureSelectXTV6	NA	
S1_396	complex	half-sibling	yes	CEGH	TruSeq	NA	
S2_396	complex	half-sibling 2	yes	CEGH	TruSeq	NA	
P_397	trio	proband	yes	CEGH	Nextera		0,31
M_397	trio	mother	no	CEGH	Nextera	NA	
F_397	trio	father	NA	CEGH	Nextera	NA	
P_398	complex	proband	yes	ASC	Nextera		0,4
O1_398	complex	cousin	yes	CEGH	Nextera	NA	
M_398	complex	mother	no	ASC	Nextera	NA	
F_398	complex	father	no	ASC	Nextera	NA	
O2_398	complex	uncle	yes	ASC	ASC	NA	
P_399	single	proband	yes	ASC	ASC	NA	
M_150	mother + sons	mother	NA	CEGH	SureSelectXTV6	NA	
P_400	complex	proband	yes	ASC	Nextera		0,34
M_400	complex	mother	NA	ASC	Nextera	NA	
F_400	complex	father	NA	ASC	Nextera	NA	
S1_400	complex	sibling	yes	CEGH	SureSelectXTV6	NA	
S2_400	complex	sibling 2	yes	CEGH	SureSelectXTV6	NA	
P_401	trio	proband	yes	ASC	ASC		0,38
M_401	trio	mother	no	ASC	ASC	NA	
F_401	trio	father	no	ASC	ASC	NA	
P_402	trio	proband	yes	ASC	Nextera		0,37
M_402	trio	mother	no	ASC	Nextera	NA	
F_402	trio	father	no	ASC	Nextera	NA	
P_403	trio	proband	yes	ASC	Nextera		0,36
M_403	trio	mother	no	ASC	Nextera	NA	
F_403	trio	father	no	ASC	Nextera	NA	
P_404	trio	proband	yes	ASC	Nextera		0,45
M_404	trio	mother	no	ASC	Nextera	NA	
F_404	trio	father	no	ASC	Nextera	NA	
P_405	trio	proband	yes	ASC	Nextera		0,44
M_405	trio	mother	no	ASC	Nextera	NA	
F_405	trio	father	no	ASC	Nextera	NA	
P_406	trio	proband	yes	ASC	Nextera		0,39
M_406	trio	mother	no	ASC	Nextera	NA	
F_406	trio	father	no	ASC	Nextera	NA	
P_407	trio	proband	yes	ASC	Nextera		0,39
M_407	trio	mother	no	ASC	Nextera	NA	
F_407	trio	father	NA	ASC	Nextera	NA	
P_408	trio	proband	yes	ASC	Nextera		0,43

M_408	trio	mother	NA	ASC	Nextera	NA	
F_408	trio	father	NA	ASC	Nextera	NA	
P_409	trio	proband	yes	ASC	ASC		0,46
M_409	trio	mother	no	ASC	ASC	NA	
F_409	trio	father	no	ASC	ASC	NA	
P_410	trio	proband	yes	ASC	Nextera		0,36
M_410	trio	mother	no	ASC	Nextera	NA	
F_410	trio	father	no	ASC	Nextera	NA	
P_411	trio	proband	yes	ASC	Nextera		0,47
M_411	trio	mother	no	ASC	Nextera	NA	
F_411	trio	father	no	ASC	Nextera	NA	
P_412	quartet	proband	yes	ASC	Nextera		0,41
M_412	quartet	mother	no	ASC	Nextera	NA	
F_412	quartet	father	yes	ASC	Nextera	NA	
S_412	quartet	sibling	yes	ASC	ASC	NA	
P_413	trio	proband	yes	ASC	Nextera		0,41
M_413	trio	mother	no	ASC	Nextera	NA	
F_413	trio	father	no	ASC	Nextera	NA	
P_414	trio	proband	yes	ASC	Nextera		0,4
M_414	trio	mother	no	ASC	Nextera	NA	
F_414	trio	father	no	ASC	Nextera	NA	
P_415	trio	proband	yes	ASC	Nextera		0,47
M_415	trio	mother	no	ASC	Nextera	NA	
F_415	trio	father	no	ASC	Nextera	NA	
P_416	trio	proband	yes	ASC	Nextera		0,43
M_416	trio	mother	no	ASC	Nextera	NA	
F_416	trio	father	no	ASC	Nextera	NA	
P_151	trio	proband	yes	CEGH	SureSelectXTV6		0,36
P_417	cousins	proband	yes	CEGH	SureSelectXTV6	NA	
O_417	cousins	cousin	yes	CEGH	SureSelectXTV6	NA	
P_418	trio	proband	yes	ASC	ASC		0,41
M_418	trio	mother	no	ASC	ASC	NA	
F_418	trio	father	no	ASC	ASC	NA	
P_419	duo	proband	yes	ASC	ASC	NA	
F_419	duo	father	NA	ASC	ASC	NA	
P_420	duo	proband	yes	ASC	Nextera		0,43
M_420	duo	mother	no	ASC	Nextera	NA	
P_421	trio	proband	yes	ASC	Nextera		0,42
M_421	trio	mother	no	ASC	Nextera	NA	
F_421	trio	father	no	ASC	Nextera	NA	
P_422	trio	proband	yes	ASC	Nextera		0,39
M_422	trio	mother	no	ASC	Nextera	NA	
F_422	trio	father	no	ASC	Nextera	NA	
P_423	trio	proband	yes	ASC	Nextera		0,38
M_423	trio	mother	no	ASC	Nextera	NA	
F_423	trio	father	no	ASC	Nextera	NA	
P_424	trio	proband	yes	ASC	Nextera		0,41
M_424	trio	mother	no	ASC	Nextera	NA	
F_424	trio	father	no	ASC	Nextera	NA	
P_425	trio	proband	yes	ASC	ASC		0,38
M_425	trio	mother	no	ASC	ASC	NA	
F_425	trio	father	no	ASC	ASC	NA	

P_426	quartet	proband	yes	ASC	Nextera		0,41
M_426	quartet	mother	NA	ASC	Nextera	NA	
F_426	quartet	father	NA	ASC	Nextera	NA	
S_426	quartet	sibling	yes	CEGH	SureSelectXTV6	NA	
P_427	trio	proband	yes	ASC	Nextera		0,41
M_427	trio	mother	no	ASC	Nextera	NA	
F_427	trio	father	no	ASC	Nextera	NA	
P_428	trio	proband	yes	ASC	Nextera		0,4
M_428	trio	mother	no	ASC	Nextera	NA	
F_428	trio	father	no	ASC	Nextera	NA	
P_429	trio	proband	yes	ASC	Nextera		0,36
M_429	trio	mother	no	ASC	Nextera	NA	
F_429	trio	father	no	ASC	Nextera	NA	
P_430	trio	proband	yes	ASC	Nextera		0,43
M_430	trio	mother	no	ASC	Nextera	NA	
F_430	trio	father	no	ASC	Nextera	NA	
P_431	trio	proband	yes	HB	SureSelectXT		0,45
P_432	trio	proband	yes	HB	SureSelectXT		0,3
M_433	trio	mother	NA	HB	SureSelectXT	NA	
F_433	trio	father	NA	HB	SureSelectXT	NA	
P_433	trio	proband	yes	HB	SureSelectXT		0,41
P_434	trio	proband	yes	HB	SureSelectXT		0,42
M_434	trio	mother	NA	HB	SureSelectXT	NA	
F_434	trio	father	NA	HB	SureSelectXT	NA	
P_435	trio	proband	yes	HB	SureSelectXT		0,4
P_436	trio	proband	yes	HB	SureSelectXT		0,33
M_436	trio	mother	NA	HB	SureSelectXT	NA	
F_436	trio	father	NA	HB	SureSelectXT	NA	
F_435	trio	father	NA	HB	SureSelectXT	NA	
M_435	trio	mother	NA	HB	SureSelectXT	NA	
F_437	trio	father	NA	HB	SureSelectXT	NA	
M_437	trio	mother	NA	HB	SureSelectXT	NA	
P_437	trio	proband	yes	HB	SureSelectXT		0,41
M_438	trio	mother	NA	HB	SureSelectXT	NA	
P_438	trio	proband	yes	HB	SureSelectXT		0,47
F_438	trio	father	NA	HB	SureSelectXT	NA	
P_439	trio	proband	yes	HB	SureSelectXT		0,43
F_439	trio	father	NA	HB	SureSelectXT	NA	
M_439	trio	mother	NA	HB	SureSelectXT	NA	
P_440	trio	proband	yes	HB	SureSelectXT		0,37
M_440	trio	mother	NA	HB	SureSelectXT	NA	
F_440	trio	father	NA	HB	SureSelectXT	NA	
P_441	trio	proband	yes	HB	SureSelectXT		0,44
M_441	trio	mother	NA	HB	SureSelectXT	NA	
F_441	trio	father	NA	HB	SureSelectXT	NA	
F_432	trio	father	NA	HB	SureSelectXT	NA	
F_431	trio	father	NA	HB	SureSelectXT	NA	
M_431	trio	mother	NA	HB	SureSelectXT	NA	
M_432	trio	mother	NA	HB	SureSelectXT	NA	

kinship_father	consanguinity	DNV_analysis	sex	total_SRY	mean_SRY
0,38	no	yes	M	42533	69,27
NA	no	no	F	0	0
NA	no	no	M	41352	67,35
0,42	no	yes	M	45151	73,54
NA	no	no	F	0	0
NA	no	no	M	39070	63,63
0,35	no	yes	M	59201	96,42
NA	no	no	F	0	0
NA	no	no	M	74797	121,82
0,37	no	yes	M	72658	118,34
NA	no	no	F	0	0
NA	no	no	M	83662	136,26
0,38	no	yes	M	70231	114,38
NA	no	no	F	0	0
NA	no	no	M	82363	134,14
0,38	no	yes	M	56620	92,21
NA	no	no	F	0	0
NA	no	no	M	66019	107,52
0,4	no	yes	M	51823	84,4
NA	no	no	F	0	0
NA	no	no	M	85712	139,6
0,36	no	yes	M	72285	117,73
NA	no	no	F	0	0
NA	no	no	M	76510	124,61
0,4	no	yes	M	79035	128,72
NA	no	no	F	0	0
NA	no	no	M	85164	138,7
0,38	no	yes	M	56500	92,02
NA	no	no	F	0	0
NA	no	no	M	70211	114,35
0,43	no	yes	F	0	0
NA	no	no	F	0	0
NA	no	no	M	61648	100,4
0,39	no	yes	M	78948	128,58
NA	no	no	F	0	0
NA	no	no	M	59351	96,66
0,45	no	yes	M	38274	62,34
NA	no	no	F	0	0
NA	no	no	M	38780	63,16
0,37	no	yes	M	87496	142,5
NA	no	no	F	0	0
NA	no	no	M	77240	125,8
0,44	no	yes	F	0	0
NA	no	no	F	0	0
NA	no	no	M	63018	102,64
0,36	no	yes	M	59053	96,18
NA	no	no	F	0	0
NA	no	no	M	62712	102,14
0,39	no	yes	M	41327	67,31
NA	no	no	F	0	0

NA	no	no	M	59177	96,38
0,4	no	yes	F	0	0
NA	no	no	F	0	0
NA	no	no	M	72093	117,42
NA	no	no	F	0	0
NA	no	no	M	41943	68,31
0,43	no	yes	M	45160	73,55
0,45	no	yes	M	82244	133,95
NA	no	no	F	0	0
NA	no	no	M	69709	113,53
0,43	no	yes	M	80165	130,56
NA	no	no	F	220	0,36
NA	no	no	M	73311	119,4
0,47	no	yes	F	0	0
NA	no	no	F	0	0
NA	no	no	M	79375	129,28
0,45	no	yes	F	0	0
NA	no	no	F	0	0
NA	no	no	M	67352	109,69
0,4	no	yes	M	63999	104,23
NA	no	no	F	151	0,25
NA	no	no	M	76515	124,62
0,43	no	yes	M	73849	120,28
NA	no	no	F	0	0
NA	no	no	M	75346	122,71
0,36	no	yes	M	67725	110,3
NA	no	no	F	70	0,11
NA	no	no	M	77657	126,48
0,42	no	yes	M	70947	115,55
NA	no	no	F	0	0
NA	no	no	M	69935	113,9
0,43	no	yes	M	37580	61,21
NA	no	no	F	0	0
NA	no	no	M	36507	59,46
0,36	no	yes	M	60320	98,24
NA	no	no	F	0	0
NA	no	no	M	93358	152,05
0,4	no	yes	M	70506	114,83
NA	no	no	F	0	0
NA	no	no	M	86799	141,37
0,36	no	yes	M	85578	139,38
NA	no	no	F	0	0
NA	no	no	M	67193	109,43
0,44	no	yes	F	0	0
NA	no	no	F	0	0
NA	no	no	M	29403	47,89
0,43	no	yes	F	0	0
NA	no	no	F	0	0
NA	no	no	M	41208	67,11
0,45	no	yes	M	39476	64,29
NA	no	no	F	0	0
NA	no	no	M	38744	63,1

	0,44	no	yes	M	40445	65,87
NA		no	no	F	0	0
NA		no	no	M	39488	64,31
	0,47	no	yes	M	34413	56,05
NA		no	no	F	0	0
NA		no	no	M	38737	63,09
	0,43	no	yes	F	0	0
NA		no	no	F	0	0
NA		no	no	M	35087	57,14
	0,41	no	yes	M	55121	89,77
NA		no	no	F	0	0
NA		no	no	M	37557	61,17
	0,45	no	yes	F	0	0
NA		no	no	F	0	0
NA		no	no	M	34003	55,38
	0,47	no	yes	M	34405	56,03
NA		no	no	F	0	0
NA		no	no	M	39882	64,95
	0,44	no	yes	M	45666	74,37
NA		no	no	F	0	0
NA		no	no	M	34854	56,77
	0,38	no	yes	M	32594	53,08
NA		no	no	F	0	0
NA		no	no	M	44669	72,75
	0,48	no	yes	M	45947	74,83
NA		no	no	F	0	0
NA		no	no	M	39556	64,42
	0,42	no	yes	M	43736	71,23
NA		no	no	F	0	0
NA		no	no	M	47952	78,1
	0,4	no	yes	M	39170	63,79
NA		no	no	F	0	0
NA		no	no	M	36965	60,2
	0,45	no	yes	M	41388	67,41
NA		no	no	F	0	0
NA		no	no	M	37656	61,33
	0,44	no	yes	M	43236	70,42
NA		no	no	F	0	0
NA		no	no	M	40998	66,77
	0,38	no	yes	F	0	0
NA		no	no	F	0	0
NA		no	no	M	42597	69,38
	0,47	no	yes	F	0	0
NA		no	no	F	0	0
NA		no	no	M	45537	74,16
	0,4	no	yes	M	44071	71,78
NA		no	no	F	0	0
NA		no	no	M	38811	63,21
	0,44	no	yes	M	36575	59,57
NA		no	no	F	0	0
NA		no	no	M	42867	69,82
	0,42	no	yes	M	48732	79,37

NA	no	no	F	0	0
NA	no	no	M	42026	68,45
0,41	no	yes	M	41004	66,78
NA	no	no	F	0	0
NA	no	no	M	34133	55,59
NA	no	no	F	0	0
NA	no	no	M	40028	65,19
0,44	no	yes	M	72283	117,72
0,49	no	yes	F	0	0
NA	no	no	F	0	0
NA	no	no	M	40922	66,65
0,41	no	yes	M	52228	85,06
NA	no	no	F	0	0
NA	no	no	M	38117	62,08
0,48	no	yes	M	45899	74,75
0,33	no	yes	M	67553	110,02
NA	no	no	F	0	0
NA	no	no	M	63921	104,11
0,45	no	yes	M	75286	122,62
0,35	no	yes	F	397	0,65
NA	no	no	F	0	0
NA	no	yes	M	32934	53,64
NA	no	no	M	33220	54,1
NA	no	yes	F	0	0
0,32	no	yes	F	337	0,55
NA	no	no	M	37770	61,51
NA	no	no	F	727	1,18
NA	no	no	M	33365	54,34
NA	no	no	F	0	0
NA	no	no	M	59400	96,74
NA	no	no	F	0	0
0,43	no	yes	F	0	0
NA	no	no	M	17772	28,94
NA	no	no	F	0	0
NA	no	no	F	0	0
0,35	no	yes	M	49203	80,14
NA	no	no	M	62692	102,1
NA	no	no	M	65412	106,53
NA	no	no	F	0	0
0,28	no	yes	M	55202	89,91
NA	no	no	F	0	0
NA	no	no	M	43143	70,27
NA	no	no	M	41775	68,04
NA	no	no	F	0	0
0,34	no	yes	M	43414	70,71
NA	no	no	F	0	0
NA	no	no	M	37678	61,36
0,36	no	yes	F	0	0
NA	no	no	F	0	0
NA	no	no	M	39362	64,11
0,32	no	yes	M	43474	70,8
NA	no	no	F	0	0

NA	no	no	M	47533	77,42
0,4	no	yes	M	67210	109,46
NA	no	no	M	25038	40,78
NA	no	no	F	0	0
0,32	no	yes	M	44853	73,05
NA	no	no	M	29054	47,32
NA	no	no	F	0	0
0,33	no	yes	M	35153	57,25
NA	no	no	F	0	0
NA	no	no	M	40025	65,19
0,37	no	yes	M	47362	77,14
NA	no	no	M	45009	73,3
NA	no	no	F	0	0
0,39	no	yes	M	26214	42,69
0,31	no	yes	M	55346	90,14
NA	no	no	F	0	0
NA	no	no	M	31730	51,68
0,31	no	yes	M	33552	54,64
NA	no	no	F	0	0
NA	no	no	M	57411	93,5
0,4	no	yes	F	0	0
NA	no	no	F	0	0
0,32	no	yes	F	0	0
NA	no	no	M	17588	28,64
NA	no	no	F	0	0
0,44	no	yes	M	39337	64,07
NA	no	no	F	0	0
NA	no	no	M	37881	61,7
0,29	no	yes	M	26796	43,64
NA	no	no	F	0	0
0,32	no	yes	M	49650	80,86
NA	no	no	F	0	0
NA	no	no	M	31221	50,85
0,43	no	yes	M	28349	46,17
NA	no	no	F	0	0
NA	no	no	M	37488	61,06
NA	no	no	M	37252	60,67
NA	no	no	M	53756	87,55
0,46	no	yes	F	0	0
NA	no	no	F	0	0
NA	no	no	M	41055	66,86
0,36	no	yes	M	51149	83,3
NA	no	no	F	0	0
NA	no	no	M	56548	92,1
0,39	no	yes	M	63010	102,62
NA	no	no	F	0	0
NA	no	no	M	72095	117,42
0,46	no	yes	M	78276	127,49
NA	no	no	F	0	0
NA	no	no	M	76868	125,19
0,42	no	yes	M	67972	110,7
NA	no	no	F	0	0

NA		no	no	M	76992	125,39
	0,45	no	yes	M	68691	111,87
NA		no	no	F	0	0
NA		no	no	M	73014	118,92
	0,41	no	yes	M	62790	102,26
NA		no	no	F	0	0
NA		no	no	M	79548	129,56
	0,38	no	yes	M	74230	120,9
NA		no	no	F	0	0
NA		no	no	M	69342	112,93
	0,38	no	yes	M	61226	99,72
NA		no	no	F	0	0
NA		no	no	M	77296	125,89
	0,42	no	yes	M	76455	124,52
NA		no	no	F	0	0
NA		no	no	M	81878	133,35
	0,39	no	yes	M	56997	92,83
NA		no	no	F	0	0
NA		no	no	M	58678	95,57
	0,42	no	yes	M	71627	116,66
NA		no	no	F	0	0
NA		no	no	M	67084	109,26
	0,39	no	yes	M	83258	135,6
NA		no	no	F	0	0
NA		no	no	M	62469	101,74
	0,39	no	yes	M	72933	118,78
NA		no	no	F	0	0
NA		no	no	M	62171	101,26
	0,36	no	yes	M	69242	112,77
NA		no	no	F	0	0
NA		no	no	M	47103	76,71
	0,32	no	yes	M	60232	98,1
NA		no	no	F	0	0
NA		no	no	M	78243	127,43
	0,37	no	yes	M	75884	123,59
NA		no	no	F	151	0,25
NA		no	no	M	85904	139,91
	0,39	no	yes	M	79392	129,3
NA		no	no	F	0	0
NA		no	no	M	69768	113,63
	0,34	no	yes	M	83008	135,19
NA		no	no	F	0	0
NA		no	no	M	72278	117,72
	0,45	no	yes	M	70999	115,63
NA		no	no	F	0	0
NA		no	no	M	60195	98,04
	0,39	no	yes	M	58401	95,12
NA		no	no	F	0	0
NA		no	no	M	63967	104,18
	0,34	no	yes	F	0	0
NA		no	no	F	0	0
NA		no	no	M	78469	127,8

NA		no	no	M	70787	115,29
NA		no	no	F	0	0
	0,35	no	yes	M	52920	86,19
NA		no	no	F	0	0
NA		no	no	M	49426	80,5
	0,39	no	yes	M	72013	117,29
NA		no	no	F	0	0
NA		no	no	M	55154	89,83
	0,39	no	yes	M	77940	126,94
NA		no	no	F	0	0
NA		no	no	M	77147	125,65
	0,36	no	yes	M	82628	134,57
NA		no	no	F	0	0
NA		no	no	M	80070	130,41
	0,39	no	yes	M	55802	90,88
NA		no	no	F	0	0
NA		no	no	M	65521	106,71
	0,45	no	yes	M	80550	131,19
NA		no	no	F	0	0
NA		no	no	M	69724	113,56
	0,43	no	yes	M	57566	93,76
NA		no	no	F	0	0
NA		no	no	M	65000	105,86
	0,34	no	yes	F	0	0
NA		no	no	F	0	0
NA		no	no	M	72196	117,58
	0,46	no	yes	M	71323	116,16
NA		no	no	F	0	0
NA		no	no	M	78700	128,18
	0,37	no	yes	M	62756	102,21
NA		no	no	F	0	0
NA		no	no	M	87318	142,21
NA		no	no	M	67970	110,7
NA		no	no	F	0	0
	0,39	no	yes	M	74532	121,39
NA		no	no	F	64	0,1
NA		no	no	M	81563	132,84
	0,36	no	yes	M	73326	119,42
NA		no	no	F	0	0
NA		no	no	M	69666	113,46
	0,41	no	yes	M	85838	139,8
NA		no	no	F	0	0
NA		no	no	M	65744	107,07
	0,37	no	yes	M	76819	125,11
NA		no	no	F	0	0
NA		no	no	M	74428	121,22
	0,36	no	yes	M	72230	117,64
NA		no	no	F	0	0
NA		no	no	M	84570	137,74
	0,48	no	yes	M	77962	126,97
NA		no	no	F	0	0
NA		no	no	M	50135	81,65

	0,41	no	yes	M	77059	125,5
NA		no	no	F	0	0
NA		no	no	M	78899	128,5
	0,35	no	yes	M	62863	102,38
NA		no	no	F	0	0
NA		no	no	M	76073	123,9
	0,41	no	yes	M	76744	124,99
NA		no	no	F	0	0
NA		no	no	M	59641	97,14
	0,39	no	yes	M	77099	125,57
NA		no	no	F	0	0
NA		no	no	M	68927	112,26
	0,4	no	yes	F	0	0
NA		no	no	F	0	0
NA		no	no	M	65570	106,79
	0,39	no	yes	M	85230	138,81
NA		no	no	F	0	0
NA		no	no	M	75969	123,73
	0,36	no	yes	M	65812	107,19
NA		no	no	F	0	0
NA		no	no	M	79658	129,74
	0,4	no	yes	M	70294	114,49
NA		no	no	F	0	0
NA		no	no	M	71346	116,2
	0,45	no	yes	M	68933	112,27
NA		no	no	F	0	0
NA		no	no	M	75458	122,9
	0,44	no	yes	M	87662	142,77
NA		no	no	F	0	0
NA		no	no	M	76506	124,6
	0,43	no	yes	M	84637	137,85
NA		no	no	F	0	0
NA		no	no	M	84949	138,35
	0,37	no	yes	M	70777	115,27
NA		no	no	F	0	0
NA		no	no	M	87511	142,53
	0,38	no	yes	F	121	0,2
NA		no	no	F	0	0
NA		no	no	M	73373	119,5
	0,42	no	yes	M	60355	98,3
NA		no	no	F	0	0
NA		no	no	M	60872	99,14
	0,43	no	yes	M	70474	114,78
NA		no	no	F	0	0
NA		no	no	M	65486	106,65
	0,36	no	no	M	74067	120,63
NA		no	no	M	75550	123,05
	0,42	no	yes	M	66922	108,99
NA		no	no	F	0	0
NA		no	no	M	78541	127,92
	0,43	no	yes	M	80540	131,17
NA		no	no	F	0	0

NA		no	no	M	67769	110,37
	0,43	no	yes	M	71222	116
NA		no	no	F	300	0,49
NA		no	no	M	86414	140,74
	0,41	no	yes	F	0	0
NA		no	no	F	0	0
NA		no	no	M	76015	123,8
	0,45	no	yes	M	74861	121,92
NA		no	no	F	0	0
NA		no	no	M	71819	116,97
	0,34	no	yes	M	81281	132,38
NA		no	no	F	0	0
NA		no	no	M	75855	123,54
	0,44	no	yes	M	85164	138,7
NA		no	no	F	0	0
NA		no	no	M	71827	116,98
NA		no	no	M	46121	75,12
	0,42	no	yes	M	55541	90,46
NA		no	no	F	296	0,48
NA		no	no	M	37150	60,5
	0,29	no	yes	M	31615	51,49
	0,31	no	yes	M	26595	43,31
	0,45	no	yes	M	47238	76,93
NA		no	no	F	0	0
NA		no	no	M	41780	68,05
NA		no	no	M	25083	40,85
NA		no	no	M	27291	44,45
NA		no	no	F	0	0
NA		no	no	M	41003	66,78
NA		no	no	M	46561	75,83
NA		no	no	F	0	0
	0,46	no	yes	M	38262	62,32
NA		no	no	F	0	0
NA		no	no	M	39981	65,12
	0,42	no	yes	M	39593	64,48
NA		no	no	F	0	0
NA		no	no	M	40947	66,69
NA		no	yes	F	0	0
	0,44	no	no	M	44364	72,25
NA		no	no	M	55331	90,12
	0,38	no	yes	M	39563	64,43
NA		no	no	F	246	0,4
NA		no	no	M	57976	94,42
	0,41	no	yes	M	42090	68,55
NA		no	no	F	0	0
NA		no	no	M	35276	57,45
NA		no	no	M	49419	80,49
NA		no	no	F	0	0
NA		no	no	F	0	0
NA		no	no	M	40451	65,88
	0,4	no	yes	F	0	0
	0,39	no	yes	F	0	0

NA	no	no	F	0	0
NA	no	no	M	44803	72,97
NA	no	no	M	45586	74,24
NA	no	no	F	0	0
NA	no	no	M	51355	83,64
NA	no	no	F	0	0
0,42	no	yes	M	34918	56,87
NA	no	no	F	0	0
NA	no	no	M	42665	69,49
0,5	no	yes	F	0	0
NA	no	no	F	0	0
NA	no	no	M	42853	69,79
0,39	no	yes	M	43719	71,2
NA	no	no	F	0	0
NA	no	no	M	53435	87,03
NA	no	yes	M	46030	74,97
NA	no	yes	M	42890	69,85
NA	no	no	M	38279	62,34
NA	no	no	F	0	0
0,42	no	yes	F	0	0
NA	no	no	F	0	0
NA	no	no	M	39118	63,71
0,4	no	yes	M	37474	61,03
NA	no	no	F	0	0
NA	no	no	M	35967	58,58
NA	no	no	M	45213	73,64
NA	no	no	F	0	0
0,43	no	yes	F	0	0
NA	no	no	F	0	0
NA	no	no	M	48160	78,44
NA	no	yes	F	0	0
NA	no	no	F	0	0
NA	no	no	M	37999	61,89
0,48	no	yes	F	0	0
NA	no	no	F	0	0
NA	no	no	M	34842	56,75
NA	no	no	M	60045	97,79
0,48	no	yes	M	32753	53,34
NA	yes	no	F	0	0
NA	yes	no	M	31878	51,92
0,45	no	yes	M	41203	67,11
NA	no	no	F	0	0
NA	no	no	M	54467	88,71
0,4	no	yes	M	36969	60,21
NA	no	no	F	0	0
NA	no	no	M	33827	55,09
NA	no	yes	M	23263	37,89
NA	no	no	M	42746	69,62
NA	no	no	M	39783	64,79
0,45	no	yes	M	42098	68,56
NA	no	no	F	0	0
NA	no	no	M	39870	64,93

	0,4	no	yes	F	0	0
NA		no	no	F	0	0
NA		no	no	M	45577	74,23
	0,41	no	yes	M	41859	68,17
NA		no	no	F	0	0
NA		no	no	M	31973	52,07
	0,39	no	yes	M	43420	70,72
NA		yes	no	F	0	0
NA		yes	no	M	38512	62,72
	0,53	no	yes	M	34477	56,15
NA		no	no	F	0	0
NA		no	no	M	39308	64,02
	0,43	no	yes	F	0	0
NA		no	no	F	0	0
NA		no	no	M	41718	67,94
NA		no	no	M	48646	79,23
NA		no	no	F	0	0
	0,38	no	yes	F	0	0
NA		no	no	F	0	0
NA		no	no	M	41678	67,88
NA		no	no	F	0	0
NA		no	no	F	0	0
NA		no	no	M	50475	82,21
NA		no	no	F	0	0
	0,43	no	yes	F	0	0
NA		no	no	F	0	0
NA		no	no	M	39488	64,31
NA		no	yes	M	24572	40,02
	0,46	no	yes	M	49391	80,44
NA		yes	no	F	0	0
NA		yes	no	M	44941	73,19
NA		no	no	M	42096	68,56
NA		no	no	F	0	0
	0,44	no	yes	M	49233	80,18
NA		no	no	F	0	0
NA		no	no	M	49731	81
	0,38	no	yes	M	41920	68,27
NA		no	no	F	0	0
NA		no	no	M	38033	61,94
	0,46	no	yes	M	43769	71,29
NA		no	no	F	0	0
NA		no	no	M	41578	67,72
NA		no	no	M	39003	63,52
NA		no	no	F	0	0
NA		no	no	F	0	0
NA		no	no	F	0	0
	0,41	no	no	M	46552	75,82
NA		no	no	M	50730	82,62
NA		no	no	M	42030	68,45
NA		no	no	F	0	0
NA		no	no	M	46708	76,07
NA		no	no	F	0	0

	0,34	no	yes	M	43749	71,25
NA		no	no	F	0	0
NA		no	no	M	36014	58,65
NA		no	no	M	46925	76,43
NA		no	no	M	37396	60,91
NA		no	no	F	0	0
NA		no	no	F	0	0
NA		no	no	F	0	0
	0,47	no	yes	M	40125	65,35
NA		no	no	F	0	0
NA		no	no	M	47925	78,05
	0,46	no	yes	F	0	0
NA		no	no	F	0	0
NA		no	no	M	42712	69,56
NA		no	no	M	48441	78,89
NA		no	no	F	0	0
NA		no	no	F	0	0
NA		no	no	F	0	0
	0,43	no	yes	M	60311	98,23
NA		no	no	F	0	0
NA		no	no	M	52703	85,84
	0,37	no	yes	M	40643	66,19
NA		no	no	F	0	0
NA		no	no	M	40767	66,4
NA		no	no	F	0	0
NA		no	no	M	38457	62,63
NA		no	no	F	0	0
NA		no	no	M	46297	75,4
	0,47	no	yes	M	49673	80,9
NA		no	no	F	0	0
NA		no	no	M	52133	84,91
NA		no	no	M	54229	88,32
NA		no	no	F	0	0
	0,42	no	yes	F	0	0
NA		no	no	F	0	0
NA		no	no	M	49216	80,16
NA		no	no	M	37222	60,62
NA		no	no	M	44853	73,05
	0,46	no	yes	M	43399	70,68
NA		no	no	F	0	0
NA		no	no	M	48514	79,01
	0,46	no	yes	M	39690	64,64
NA		no	no	F	0	0
NA		no	no	M	53520	87,17
	0,49	no	no	M	34887	56,82
NA		no	no	M	42876	69,83
NA		no	no	F	0	0
NA		no	no	M	41396	67,42
NA		no	no	F	0	0
	0,51	no	yes	F	0	0
NA		no	no	F	0	0
NA		no	no	M	46194	75,23

	0,42	no	yes	F	0	0
NA		no	no	F	0	0
NA		no	no	M	48131	78,39
NA		no	no	F	0	0
NA		no	no	F	0	0
	0,4	no	yes	M	47811	77,87
NA		no	no	F	0	0
NA		no	no	M	46315	75,43
	0,39	no	yes	M	44677	72,76
NA		no	no	F	0	0
NA		no	no	M	46876	76,35
	0,45	no	yes	F	0	0
NA		no	no	F	0	0
NA		no	no	M	43240	70,42
	0,47	no	yes	M	47407	77,21
NA		no	no	F	0	0
NA		no	no	M	42084	68,54
	0,43	no	yes	M	49367	80,4
NA		no	no	F	0	0
NA		no	no	M	50413	82,11
	0,43	no	yes	M	51590	84,02
NA		no	no	F	0	0
NA		no	no	M	39426	64,21
	0,36	no	yes	F	0	0
NA		no	no	F	0	0
NA		no	no	M	37975	61,85
	0,39	no	yes	M	58244	94,86
NA		no	no	F	0	0
NA		no	no	M	51383	83,69
	0,43	no	yes	F	0	0
NA		no	no	F	0	0
NA		no	no	M	39242	63,91
	0,43	no	yes	M	43595	71
NA		no	no	F	0	0
NA		no	no	M	46407	75,58
NA		no	no	M	35877	58,43
NA		no	no	F	0	0
NA		no	no	F	0	0
NA		no	no	M	49614	80,8
NA		no	no	M	40536	66,02
NA		no	no	M	51417	83,74
	0,44	no	yes	F	0	0
NA		no	no	M	85834	139,79
NA		no	no	F	0	0
	0,42	no	yes	M	94429	153,79
NA		no	no	F	0	0
NA		no	no	M	80186	130,6
	0,42	no	yes	M	65260	106,29
NA		no	no	F	0	0
NA		no	no	M	73477	119,67
	0,42	no	yes	M	66949	109,04
NA		no	no	F	0	0

NA	no	no	M	65568	106,79
0,35	no	yes	M	75967	123,72
NA	no	no	F	0	0
NA	no	no	M	88335	143,87
0,41	no	yes	M	79064	128,77
NA	no	no	F	0	0
NA	no	no	M	94064	153,2
0,32	no	yes	M	71601	116,61
NA	no	no	F	0	0
NA	no	no	M	74656	121,59
0,47	no	yes	M	61670	100,44
NA	no	no	F	0	0
NA	no	no	M	80634	131,33
0,43	no	yes	M	72527	118,12
NA	no	no	F	0	0
NA	no	no	M	90995	148,2
NA	no	no	F	0	0
NA	no	no	M	82967	135,13
0,39	no	yes	M	97143	158,21
0,37	no	yes	M	82582	134,5
NA	no	no	F	0	0
NA	no	no	M	75511	122,98
0,39	no	yes	F	0	0
NA	no	no	F	0	0
NA	no	no	M	70704	115,15
0,42	no	yes	M	78804	128,35
NA	no	no	F	0	0
NA	no	no	M	76841	125,15
0,42	no	yes	F	0	0
NA	no	no	F	0	0
NA	no	no	M	74315	121,03
0,52	no	yes	M	61051	99,43
NA	no	no	F	0	0
NA	no	no	M	77752	126,63
0,37	no	yes	M	82447	134,28
NA	no	no	F	151	0,25
NA	no	no	M	82175	133,84
0,46	no	yes	M	71792	116,93
NA	no	no	F	0	0
NA	no	no	M	76928	125,29
0,32	no	yes	M	57870	94,25
NA	no	no	F	0	0
NA	no	no	M	48563	79,09
0,4	no	yes	M	75522	123
NA	no	no	F	0	0
NA	no	no	M	82062	133,65
0,44	no	yes	M	91003	148,21
NA	no	no	F	0	0
NA	no	no	M	86154	140,32
0,37	no	yes	M	88922	144,82
NA	no	no	F	121	0,2
NA	no	no	M	61946	100,89

	0,35	no	yes	F	0	0
NA		no	no	F	0	0
NA		no	no	M	88164	143,59
	0,37	no	yes	F	0	0
NA		no	no	F	0	0
NA		no	no	M	70899	115,47
	0,36	no	yes	M	73629	119,92
NA		no	no	F	0	0
NA		no	no	M	75976	123,74
	0,35	no	yes	M	49768	81,06
NA		no	no	F	0	0
NA		no	no	M	30613	49,86
	0,39	no	yes	M	67283	109,58
NA		no	no	F	0	0
NA		no	no	M	81398	132,57
	0,38	no	yes	M	88958	144,88
NA		no	no	F	0	0
NA		no	no	M	57867	94,25
	0,36	no	yes	M	75691	123,28
NA		no	no	F	0	0
NA		no	no	M	81983	133,52
	0,38	no	yes	F	0	0
NA		no	no	F	0	0
NA		no	no	M	80290	130,77
	0,39	no	yes	M	81583	132,87
NA		no	no	F	0	0
NA		no	no	M	75530	123,01
	0,39	no	yes	M	81391	132,56
NA		no	no	F	0	0
NA		no	no	M	73932	120,41
	0,42	no	yes	M	103479	168,53
NA		no	no	F	294	0,48
NA		no	no	M	52615	85,69
NA		no	yes	M	66063	107,59
	0,39	no	yes	F	0	0
NA		no	no	F	0	0
NA		no	no	M	92734	151,03
	0,44	no	yes	M	88110	143,5
NA		no	no	F	0	0
NA		no	no	M	90188	146,89
	0,35	no	yes	M	88753	144,55
NA		no	no	F	0	0
NA		no	no	M	78444	127,76
	0,37	no	yes	M	83453	135,92
NA		no	no	F	0	0
NA		no	no	M	93165	151,73
	0,36	no	yes	M	75705	123,3
NA		no	no	F	0	0
NA		no	no	M	73991	120,51
	0,37	no	yes	M	91962	149,78
NA		no	no	F	0	0
NA		no	no	M	90557	147,49

	0,37	no	yes	M	82755	134,78
NA		no	no	F	0	0
NA		no	no	M	85898	139,9
	0,41	no	yes	M	75692	123,28
NA		no	no	F	0	0
NA		no	no	M	87994	143,31
	0,36	no	yes	F	0	0
NA		no	no	F	0	0
NA		no	no	M	51989	84,67
	0,41	no	yes	M	63032	102,66
NA		no	no	F	0	0
NA		no	no	M	70154	114,26
	0,48	no	yes	M	72928	118,78
NA		no	no	F	0	0
NA		no	no	M	82204	133,88
NA		no	no	F	0	0
	0,42	no	yes	F	0	0
NA		no	no	F	0	0
NA		no	no	M	47343	77,11
	0,38	no	yes	M	88923	144,83
NA		no	no	F	251	0,41
NA		no	no	M	99187	161,54
	0,4	no	yes	M	71439	116,35
NA		no	no	F	0	0
NA		no	no	M	64209	104,57
NA		no	no	M	21134	34,42
NA		no	no	F	0	0
	0,37	no	yes	M	21863	35,61
NA		no	no	F	0	0
NA		no	no	M	23099	37,62
	0,5	no	yes	M	85468	139,2
NA		no	no	F	0	0
NA		no	no	M	70164	114,27
	0,41	no	yes	M	85073	138,56
NA		no	no	F	0	0
NA		no	no	M	53084	86,46
	0,42	no	yes	M	78710	128,19
NA		no	no	F	200	0,33
NA		no	no	M	87867	143,11
	0,32	no	yes	M	36952	60,18
NA		no	no	F	0	0
NA		no	no	M	31847	51,87
	0,37	no	yes	M	35630	58,03
NA		no	no	F	0	0
NA		no	no	M	52262	85,12
	0,4	no	yes	M	83385	135,81
NA		no	no	F	0	0
NA		no	no	M	64520	105,08
	0,37	no	yes	M	48643	79,22
NA		no	no	F	0	0
NA		no	no	M	64687	105,35
	0,38	no	yes	M	69655	113,44

NA	no	no	F	0	0
NA	no	no	M	79314	129,18
0,37	no	yes	M	74369	121,12
NA	no	no	F	0	0
NA	no	no	M	77611	126,4
0,4	no	yes	M	84762	138,05
NA	no	no	F	0	0
NA	no	no	M	75758	123,38
0,46	no	yes	M	76200	124,1
NA	no	no	F	151	0,25
NA	no	no	M	74215	120,87
0,36	no	yes	F	0	0
NA	no	no	F	0	0
NA	no	no	M	63369	103,21
0,41	no	yes	M	67238	109,51
NA	no	no	F	0	0
NA	no	no	M	80736	131,49
0,45	no	yes	M	71693	116,76
NA	no	no	F	0	0
NA	no	no	M	77346	125,97
0,4	no	yes	M	67727	110,3
NA	no	no	F	0	0
NA	no	no	M	85663	139,52
0,37	no	yes	M	77515	126,25
NA	no	no	F	40	0,07
NA	no	no	M	75004	122,16
0,37	no	yes	M	32360	52,7
NA	no	no	F	0	0
NA	no	no	M	35718	58,17
0,48	no	yes	F	0	0
NA	no	no	F	0	0
NA	no	no	M	82764	134,79
0,36	no	yes	M	42527	69,26
NA	no	no	F	0	0
NA	no	no	M	48226	78,54
0,43	no	yes	M	84946	138,35
NA	no	no	F	0	0
NA	no	no	M	81229	132,29
0,35	no	yes	M	70980	115,6
NA	no	no	F	149	0,24
NA	no	no	M	37003	60,27
0,37	no	yes	M	42864	69,81
NA	no	no	F	0	0
NA	no	no	M	45001	73,29
0,43	no	yes	F	0	0
NA	no	no	F	90	0,15
NA	no	no	M	90245	146,98
0,45	no	yes	M	85085	138,57
NA	no	no	F	0	0
NA	no	no	M	85435	139,14
0,39	no	yes	F	0	0
NA	no	no	F	0	0

NA		no	no	M	77304	125,9
	0,5	no	yes	M	76135	124
NA		no	no	F	0	0
NA		no	no	M	89408	145,62
	0,44	no	yes	M	25778	41,98
NA		no	no	F	0	0
NA		no	no	M	34590	56,34
	0,38	no	yes	M	75900	123,62
NA		no	no	F	0	0
NA		no	no	M	76198	124,1
	0,4	no	yes	M	83758	136,41
NA		no	no	F	0	0
NA		no	no	M	93350	152,04
	0,43	no	yes	M	51666	84,15
NA		no	no	F	0	0
NA		no	no	M	43852	71,42
	0,37	no	yes	M	37408	60,93
NA		no	no	F	0	0
NA		no	no	M	38525	62,74
	0,37	no	yes	M	53190	86,63
NA		no	no	F	234	0,38
NA		no	no	M	54812	89,27
	0,35	no	yes	F	0	0
NA		no	no	F	378	0,62
NA		no	no	M	36191	58,94
	0,39	no	yes	M	33934	55,27
NA		no	no	F	182	0,3
NA		no	no	M	28319	46,12
	0,36	no	yes	M	32597	53,09
NA		no	no	F	112	0,18
NA		no	no	M	31330	51,03
	0,37	no	yes	F	0	0
NA		no	no	F	252	0,41
NA		no	no	M	35772	58,26
	0,34	no	yes	M	43142	70,26
NA		no	no	F	0	0
NA		no	no	M	48630	79,2
	0,46	no	yes	M	21934	35,72
NA		no	no	F	0	0
NA		no	no	M	27670	45,07
	0,35	no	yes	M	40756	66,38
NA		no	no	F	0	0
NA		no	no	M	18465	30,07
	0,4	no	yes	M	66549	108,39
NA		no	no	F	0	0
	0,4	no	yes	M	44017	71,69
NA		no	no	F	120	0,2
NA		no	no	M	26580	43,29
	0,38	no	yes	M	21089	34,35
NA		no	no	F	0	0
NA		no	no	M	23712	38,62
	0,38	no	yes	M	20274	33,02

NA	no	no	F	0	0
NA	no	no	M	19022	30,98
0,36	no	yes	M	6612	10,77
NA	no	no	F	0	0
NA	no	no	M	11039	17,98
0,39	no	yes	F	0	0
NA	no	no	F	0	0
NA	no	no	M	40604	66,13
0,3	no	yes	F	499	0,81
NA	no	no	F	111	0,18
NA	no	no	M	34406	56,04
NA	no	yes	M	32616	53,12
0,45	no	yes	F	0	0
NA	no	no	F	0	0
NA	no	no	M	50329	81,97
0,45	no	yes	F	0	0
NA	no	no	F	0	0
NA	no	no	M	37449	60,99
NA	no	no	F	83	0,14
0,39	no	yes	F	0	0
NA	no	no	F	0	0
NA	no	no	M	33462	54,5
0,38	no	yes	M	82483	134,34
NA	no	no	F	0	0
NA	no	no	M	78169	127,31
0,4	no	yes	M	63655	103,67
NA	no	no	F	0	0
NA	no	no	M	68084	110,89
0,41	no	yes	M	59734	97,29
NA	no	no	F	200	0,33
NA	no	no	M	24919	40,58
0,41	no	yes	M	27227	44,34
NA	no	no	F	792	1,29
NA	no	no	M	33182	54,04
0,43	no	yes	M	33268	54,18
NA	no	no	F	272	0,44
NA	no	no	M	26783	43,62
0,51	no	yes	F	0	0
NA	no	no	F	0	0
NA	no	no	M	27746	45,19
NA	no	no	F	0	0
NA	no	no	M	80949	131,84
NA	no	no	F	0	0
0,37	no	yes	M	72320	117,79
NA	no	no	F	0	0
NA	no	no	M	69660	113,45
NA	no	no	M	68741	111,96
NA	no	no	F	0	0
0,38	no	yes	F	0	0
NA	no	no	F	0	0
NA	no	no	M	64397	104,88
0,44	no	yes	M	69254	112,79

NA	no	no	F	0	0
NA	no	no	M	67627	110,14
0,44	no	yes	M	75045	122,22
NA	no	no	F	0	0
NA	no	no	M	68186	111,05
0,36	no	yes	M	74029	120,57
NA	no	no	F	0	0
NA	no	no	M	98832	160,96
0,42	no	yes	F	0	0
NA	no	no	F	199	0,32
NA	no	no	M	33821	55,08
NA	no	no	M	38380	62,51
0,34	no	yes	M	37559	61,17
NA	no	no	F	72	0,12
NA	no	no	M	39058	63,61
0,38	no	yes	M	23363	38,05
NA	no	no	F	0	0
NA	no	no	M	19690	32,07
0,45	no	yes	M	16745	27,27
NA	no	no	F	0	0
NA	no	no	M	15116	24,62
0,42	no	yes	M	21935	35,72
NA	no	no	F	0	0
NA	no	no	M	31932	52,01
0,3	no	yes	M	17956	29,24
NA	no	no	F	0	0
NA	no	no	M	18388	29,95
0,29	no	no	M	21316	34,72
NA	no	no	M	17306	28,19
NA	no	no	M	16105	26,23
NA	no	no	F	0	0
0,32	no	yes	F	91	0,15
NA	no	no	F	0	0
NA	no	no	M	23444	38,18
0,33	no	yes	M	20737	33,77
NA	yes	no	F	519	0,85
NA	yes	no	M	11572	18,85
0,37	no	yes	M	23758	38,69
NA	yes	no	F	0	0
NA	yes	no	M	21531	35,07
NA	no	yes	M	25875	42,14
0,41	no	no	M	71966	117,21
NA	no	no	M	67483	109,91
0,31	no	yes	M	37787	61,54
NA	no	no	F	0	0
NA	no	no	M	30829	50,21
NA	no	yes	M	43323	70,56
NA	yes	no	F	156	0,25
NA	yes	no	M	52358	85,27
0,31	no	yes	F	0	0
NA	no	no	F	0	0
NA	no	no	M	19784	32,22

	0,35	no	yes	M	16980	27,65
NA		no	no	F	0	0
NA		no	no	M	11847	19,29
	0,34	no	yes	M	15182	24,73
NA		no	no	F	0	0
NA		no	no	M	15744	25,64
	0,37	no	yes	M	18591	30,28
NA		no	no	F	69	0,11
NA		no	no	M	15827	25,78
	0,28	no	yes	F	0	0
NA		no	no	F	0	0
NA		no	no	M	11785	19,19
	0,32	no	yes	F	0	0
NA		no	no	F	0	0
NA		no	no	M	8654	14,09
	0,43	no	yes	M	81109	132,1
NA		no	no	F	0	0
NA		no	no	M	81500	132,74
	0,3	no	yes	M	35735	58,2
NA		no	no	F	560	0,91
NA		no	no	M	13588	22,13
NA		no	yes	M	35619	58,01
	0,33	no	yes	F	266	0,43
NA		no	no	F	245	0,4
NA		no	no	M	39796	64,81
NA		no	no	F	126	0,21
NA		no	no	F	0	0
NA		no	no	M	46596	75,89
NA		no	no	F	126	0,21
	0,38	no	yes	M	29382	47,85
NA		no	no	F	0	0
NA		no	no	M	26762	43,59
	0,41	no	yes	M	27994	45,59
NA		no	no	F	0	0
NA		no	no	M	30985	50,46
	0,4	no	yes	M	72209	117,6
NA		no	no	F	0	0
NA		no	no	M	63755	103,84
	0,39	no	yes	M	75873	123,57
NA		no	no	F	0	0
NA		no	no	M	65464	106,62
	0,37	no	yes	M	31509	51,32
NA		no	no	F	198	0,32
NA		no	no	M	36182	58,93
	0,34	no	yes	M	53045	86,39
NA		no	no	F	0	0
NA		no	no	M	66116	107,68
NA		no	yes	M	67273	109,57
	0,34	no	yes	F	0	0
NA		no	no	F	0	0
NA		no	no	M	22728	37,02
NA		no	yes	F	86	0,14

	0,33	no	yes	M	13807	22,49
NA		no	no	F	0	0
NA		no	no	M	16121	26,26
	0,35	no	yes	M	21516	35,04
NA		no	no	F	126	0,21
NA		no	no	M	30095	49,01
	0,4	no	yes	M	16413	26,73
NA		no	no	F	0	0
NA		no	no	M	15817	25,76
	0,41	no	yes	M	64714	105,4
NA		no	no	F	0	0
NA		no	no	M	58160	94,72
	0,45	no	yes	M	60856	99,11
NA		no	no	F	0	0
NA		no	no	M	46550	75,81
	0,33	no	yes	F	0	0
NA		no	no	F	280	0,46
NA		no	no	M	16933	27,58
	0,34	no	yes	M	18108	29,49
NA		no	no	F	0	0
NA		no	no	M	16543	26,94
	0,38	no	yes	F	0	0
NA		no	no	F	94	0,15
NA		no	no	M	15726	25,61
	0,35	no	yes	F	185	0,3
NA		no	no	F	0	0
NA		no	no	M	16240	26,45
	0,38	no	yes	M	31746	51,7
NA		no	no	F	0	0
NA		no	no	M	29181	47,53
NA		no	no	M	45677	74,39
NA		no	no	F	311	0,51
	0,38	no	yes	M	47430	77,25
NA		no	no	F	0	0
NA		no	no	M	70531	114,87
NA		no	no	M	44707	72,81
NA		no	no	F	0	0
	0,4	no	yes	M	77550	126,3
NA		no	no	F	0	0
NA		no	no	M	65930	107,38
	0,37	no	yes	M	17763	28,93
NA		no	no	F	92	0,15
NA		no	no	M	19407	31,61
	0,38	no	yes	M	27638	45,01
NA		no	no	F	0	0
NA		no	no	M	31851	51,87
	0,43	no	yes	M	91667	149,29
NA		no	no	F	0	0
NA		no	no	M	101350	165,07
	0,37	no	yes	M	53807	87,63
NA		no	no	F	0	0
NA		no	no	M	61157	99,6

NA	no	no	F	125	0,2
NA	no	no	F	0	0
NA	no	no	M	35825	58,35
0,36	no	yes	M	37688	61,38
NA	no	yes	M	39183	63,82
NA	no	no	F	250	0,41
NA	no	no	M	34290	55,85
NA	no	yes	F	0	0
NA	no	no	F	0	0
NA	no	no	M	37313	60,77
NA	no	no	M	33723	54,92
0,4	no	yes	M	36245	59,03
NA	no	no	F	0	0
NA	no	no	M	36169	58,91
NA	no	no	M	30762	50,1
0,28	no	yes	M	27196	44,29
NA	no	no	F	0	0
NA	no	no	M	25887	42,16
0,4	no	yes	M	61061	99,45
NA	no	no	M	14709	23,96
NA	no	no	F	0	0
NA	no	no	M	61808	100,66
NA	no	no	M	67651	110,18
NA	no	no	M	45205	73,62
NA	no	no	F	0	0
0,41	no	yes	M	63353	103,18
NA	no	no	F	0	0
NA	no	no	M	83373	135,79
NA	no	yes	M	66257	107,91
NA	no	yes	M	57485	93,62
0,36	no	yes	M	80656	131,36
NA	no	no	F	0	0
NA	no	no	M	41432	67,48
0,42	no	yes	M	85884	139,88
NA	no	no	F	0	0
NA	no	no	M	78545	127,92
0,44	no	yes	M	98028	159,65
NA	no	no	F	0	0
NA	no	no	M	74073	120,64
0,38	no	yes	M	60090	97,87
NA	no	no	F	0	0
NA	no	no	M	84259	137,23
0,35	no	yes	M	73897	120,35
NA	no	no	F	0	0
NA	no	no	M	57862	94,24
0,44	no	yes	F	0	0
NA	no	no	F	0	0
NA	no	no	M	57259	93,26
0,44	no	yes	M	78597	128,01
NA	no	no	F	0	0
NA	no	no	M	61307	99,85
0,35	no	yes	F	0	0

NA	no	no	F	0	0
NA	no	no	M	88647	144,38
0,38	no	yes	M	69437	113,09
NA	no	no	F	0	0
NA	no	no	M	79793	129,96
0,48	no	yes	M	69014	112,4
NA	no	no	F	0	0
NA	no	no	M	73991	120,51
0,39	no	yes	F	0	0
NA	no	no	F	0	0
NA	no	no	M	61815	100,68
0,46	no	yes	F	0	0
NA	no	no	F	0	0
NA	no	no	M	84297	137,29
NA	no	yes	M	70311	114,51
0,41	no	yes	M	58468	95,22
NA	no	no	F	0	0
NA	no	no	M	73401	119,55
0,43	no	yes	F	0	0
NA	no	no	F	0	0
NA	no	no	M	50663	82,51
0,33	no	yes	M	91956	149,77
NA	no	no	F	0	0
NA	no	no	M	96885	157,79
0,41	no	yes	F	0	0
NA	no	no	F	0	0
NA	no	no	M	96925	157,86
0,4	no	yes	M	45911	74,77
NA	no	no	M	123908	201,8
NA	no	no	M	116204	189,26
0,4	no	yes	M	74301	121,01
NA	no	no	F	0	0
NA	no	no	M	61113	99,53
0,39	no	no	M	74581	121,47
NA	no	no	M	73747	120,11
NA	no	no	M	64895	105,69
NA	no	no	F	0	0
0,39	no	yes	M	71947	117,18
NA	no	no	F	0	0
NA	no	no	M	69468	113,14
0,43	no	yes	M	75967	123,72
NA	no	no	F	0	0
NA	no	no	M	66945	109,03
0,43	no	yes	F	0	0
NA	no	no	F	0	0
NA	no	no	M	67808	110,44
0,37	no	yes	M	74440	121,24
NA	no	no	F	0	0
NA	no	no	M	62903	102,45
0,41	no	yes	M	75789	123,43
NA	no	no	F	0	0
NA	no	no	M	86828	141,41

	0,38	no	yes	M	61523	100,2
NA		no	no	F	0	0
NA		no	no	M	75276	122,6
NA		no	yes	M	114606	186,65
	0,39	no	yes	F	0	0
NA		no	no	F	0	0
NA		no	no	M	87987	143,3
	0,39	no	yes	M	84855	138,2
NA		no	no	F	0	0
NA		no	no	M	81628	132,94
	0,42	no	yes	M	78379	127,65
NA		no	no	F	143	0,23
NA		no	no	M	86513	140,9
	0,38	no	yes	M	70697	115,14
NA		no	no	F	0	0
NA		no	no	M	69945	113,92
	0,35	no	yes	M	27623	44,99
	0,37	no	yes	M	22464	36,59
NA		no	no	F	0	0
NA		no	no	M	46755	76,15
	0,34	no	yes	M	30770	50,11
	0,35	no	yes	M	25606	41,7
NA		no	no	F	0	0
NA		no	no	M	29267	47,67
	0,41	no	yes	M	21846	35,58
	0,52	no	yes	M	31606	51,48
NA		no	no	F	377	0,61
NA		no	no	M	35060	57,1
NA		no	no	M	23350	38,03
NA		no	no	F	0	0
NA		no	no	M	18682	30,43
NA		no	no	F	101	0,16
	0,4	no	yes	M	33847	55,13
NA		no	no	F	0	0
	0,42	no	yes	M	26089	42,49
NA		no	no	M	28838	46,97
	0,4	no	yes	M	23395	38,1
NA		no	no	M	23570	38,39
NA		no	no	F	0	0
	0,41	no	yes	F	0	0
NA		no	no	F	82	0,13
NA		no	no	M	30452	49,6
	0,41	no	yes	M	37542	61,14
NA		no	no	F	0	0
NA		no	no	M	28470	46,37
NA		no	no	M	27167	44,25
NA		no	no	M	36809	59,95
NA		no	no	F	21	0,03
NA		no	no	F	0	0

%_bases_above_10_SRY	%_bases_above_20_SRY	total_coverage	mean_coverage
100	100	2349482636	67,03
0	0	2409888263	68,76
100	100	2341519232	66,81
100	100	2203330284	62,86
0	0	2256284572	64,37
100	100	2243633520	64,01
100	100	2482296201	70,82
0	0	2733630809	77,99
100	100	2992861933	85,39
100	100	2656114806	75,78
0	0	2838941946	81
100	100	3483370678	99,39
100	100	2948684348	84,13
0	0	3004818312	85,73
100	100	3123124851	89,11
100	100	2734169636	78,01
0	0	3153049119	89,96
100	100	2686701318	76,66
100	100	2203331575	62,86
0	0	3138606908	89,55
100	100	3219199729	91,85
100	100	3301977433	94,21
0	0	2062051226	58,83
100	100	3268591722	93,26
100	100	2924181439	83,43
0	0	3558018192	101,52
100	100	3668705964	104,67
100	100	2558670650	73
0	0	3210726319	91,61
100	100	2731643006	77,94
0	0	2612329764	74,53
0	0	2587640621	73,83
100	100	2724937085	77,75
100	100	3323146821	94,81
0	0	2883515531	82,27
100	100	2474663301	70,61
100	100	2106937985	60,11
0	0	2300421090	65,63
100	100	2170014994	61,91
100	100	3317864050	94,66
0	0	3321331917	94,76
100	100	2984850536	85,16
0	0	3370042945	96,15
0	0	2979030483	85
100	100	2664455534	76,02
100	100	2769457503	79,02
0	0	3370880430	96,18
100	100	2853049296	81,4
100	100	1964251166	56,04
0	0	3168942481	90,41

100	100	2550531440	72,77
0	0	3001358965	85,63
0	0	3168742294	90,41
100	100	2994375645	85,43
0	0	2487403918	70,97
100	100	2467110025	70,39
100	100	2363632963	67,44
100	100	3747212369	106,91
0	0	2650115085	75,61
100	100	2798239210	79,84
100	100	3118640437	88,98
0	0	3069396574	87,57
100	100	2915336124	83,18
0	0	2662142961	75,95
0	0	3316456327	94,62
100	100	3494998334	99,72
0	0	3606473593	102,9
0	0	3000016568	85,59
100	100	2999483370	85,58
100	100	2768352090	78,98
0	0	2534258079	72,31
100	100	3334452147	95,14
100	100	3194972113	91,16
0	0	2731899891	77,94
100	100	2967493312	84,67
100	100	2924957623	83,45
0	0	2856073231	81,49
100	100	3464682013	98,85
100	100	3081049235	87,91
0	0	3125515146	89,18
100	100	3000915212	85,62
100	100	2270750880	64,79
0	0	2294870438	65,48
100	100	2078490031	59,3
100	100	2601463913	74,22
0	0	1906781580	54,4
100	100	3639730649	103,85
100	100	2842624397	81,1
0	0	2233068299	63,71
100	100	3378731236	96,4
100	100	3448902949	98,4
0	0	2724256835	77,73
100	100	2999598384	85,58
0	0	2119466683	60,47
0	0	2204283758	62,89
100	100	2051697638	58,54
0	0	1997101960	56,98
0	0	2730028730	77,89
100	100	2257313719	64,4
100	100	2578953876	73,58
0	0	2666095224	76,07
100	100	2304714253	65,76

100	100	2236032388	63,8
0	0	2012079725	57,41
100	100	2244288071	64,03
100	100	2173045423	62
0	0	2185657486	62,36
100	100	2322889605	66,28
0	0	2191793539	62,53
0	0	1968466100	56,16
100	100	2123331886	60,58
100	100	3083075161	87,96
0	0	2461268181	70,22
100	100	2352690685	67,13
0	0	2103729418	60,02
0	0	2166210738	61,8
100	100	2413487942	68,86
100	100	2111363344	60,24
0	0	2235883451	63,79
100	100	2538804088	72,44
100	100	2428513547	69,29
0	0	2058002856	58,72
100	100	2104670262	60,05
100	100	2085905961	59,51
0	0	2411068618	68,79
100	100	2293881154	65,45
100	100	2732683241	77,97
0	0	2504096678	71,45
100	100	2183704163	62,3
100	100	2228782371	63,59
0	0	2511552811	71,66
100	100	2606096218	74,36
100	100	2176802192	62,11
0	0	2298235989	65,57
100	100	2432337095	69,4
100	100	2368436519	67,57
0	0	2545612442	72,63
100	100	2277174245	64,97
100	100	2604661367	74,31
0	0	2352675708	67,13
100	100	2840470806	81,04
0	0	2272372354	64,83
0	0	2317924912	66,13
100	100	2485436404	70,91
0	0	2507925138	71,55
0	0	2246465030	64,09
100	100	2400061175	68,48
100	100	2347064152	66,96
0	0	2265224629	64,63
100	100	2258841859	64,45
100	100	2083266510	59,44
0	0	2322703714	66,27
100	100	2478417221	70,71
100	100	2539278931	72,45

0	0	2279828609	65,05
100	100	2598649109	74,14
100	100	2288571634	65,3
0	0	2052090266	58,55
100	100	2284784203	65,19
0	0	2366388280	67,52
100	100	2279218004	65,03
100	100	2015566450	57,51
0	0	2470585363	70,49
0	0	2100939713	59,94
100	100	2133025397	60,86
100	100	2549551702	72,74
0	0	2390721118	68,21
100	100	2330781326	66,5
100	100	2134831278	60,91
100	100	5911552302	168,66
0	0	3122352601	89,09
100	100	2876076475	82,06
100	100	4192801621	119,63
0	0	3804747698	108,55
0	0	3279759455	93,58
100	100	2463889641	70,3
100	100	3049565845	87,01
0	0	2517470132	71,83
0	0	2384589688	68,04
100	100	3078226598	87,83
0	0	2484957505	70,9
100	100	1623309871	46,32
0	0	3279123745	93,56
100	100	5476239323	156,24
0	0	6007377263	171,4
0	0	3237252383	92,36
100	93,6	1830670215	52,23
0	0	935972942	26,7
0	0	4307275011	122,89
100	100	4000728091	114,15
100	100	5215653808	148,81
100	100	5603617247	159,88
0	0	4467855050	127,47
100	100	4642371359	132,45
0	0	3721237687	106,17
100	100	3501179610	99,89
100	100	3178783940	90,7
0	0	5160357044	147,23
100	100	3629058701	103,54
0	0	3963969584	113,1
100	100	3351390267	95,62
0	0	3967006250	113,18
0	0	3927779211	112,06
100	100	3595440409	102,58
100	100	3656071924	104,31
0	0	4036302107	115,16

100	100	4047559265	115,48
100	100	5504450070	157,05
100	95	2666632475	76,08
0	0	4503114988	128,48
100	100	3433409557	97,96
100	95	3382519460	96,51
0	0	1997671241	57
100	100	2828951163	80,71
0	0	4371741168	124,73
100	100	3913591972	111,66
100	100	3753314678	107,09
100	100	4199134994	119,81
0	0	5413613928	154,46
100	100	2203960592	62,88
100	100	4512454364	128,75
0	0	3489942390	99,57
100	100	2608327844	74,42
100	100	2914281097	83,15
0	0	4493789971	128,21
100	100	4603270463	131,34
0	0	3453270562	98,53
0	0	4773647099	136,2
0	0	3156979490	90,07
100	82,1	1761373108	50,25
0	0	3380696907	96,46
100	100	2432362814	69,4
0	0	2221760464	63,39
100	100	2215144699	63,2
100	93,8	2058660042	58,74
0	0	1735557115	49,52
100	100	4657398348	132,88
0	0	2108143352	60,15
100	100	3120934600	89,04
100	100	1954375231	55,76
0	0	2247755056	64,13
100	100	2307015940	65,82
100	100	3001121248	85,63
100	100	4679856034	133,52
0	0	2331926061	66,53
0	0	2493949399	71,16
100	100	2537685723	72,4
100	100	2304245252	65,74
0	0	3324843281	94,86
100	100	2341285726	66,8
100	100	2774902919	79,17
0	0	3007252450	85,8
100	100	3123816927	89,13
100	100	3033000341	86,54
0	0	2940573811	83,9
100	100	3206590051	91,49
100	100	3048111193	86,97
0	0	2859013765	81,57

100	100	2913042200	83,11
100	100	3152653631	89,95
0	0	3176354491	90,63
100	100	3068513541	87,55
100	100	3040423773	86,75
0	0	3197629362	91,23
100	100	2934406766	83,72
100	100	3399594298	97
0	0	2659682837	75,88
100	100	2935500867	83,75
100	100	2486290662	70,94
0	0	3250769899	92,75
100	100	3127357048	89,23
100	100	3107042243	88,65
0	0	3235996922	92,33
100	100	3348424322	95,54
100	100	2509245995	71,59
0	0	3316671006	94,63
100	100	2544133584	72,59
100	100	3027628089	86,38
0	0	2957381424	84,38
100	100	2974362317	84,86
100	100	3390789954	96,74
0	0	2917214452	83,23
100	100	2611379063	74,51
100	100	3294177837	93,99
0	0	3428297821	97,81
100	100	2671890426	76,23
100	100	3307005028	94,35
0	0	2820096134	80,46
100	100	1890480910	53,94
100	100	2747303172	78,38
0	0	2880156560	82,17
100	100	3214289601	91,71
100	100	2933876240	83,71
0	0	2465889240	70,36
100	100	3251047834	92,76
100	100	3247920432	92,67
0	0	3074572854	87,72
100	100	2547183914	72,67
100	100	3024335125	86,29
0	0	3587196076	102,35
100	100	3110293427	88,74
100	100	3066828971	87,5
0	0	2981719379	85,07
100	100	2915032370	83,17
100	100	2592954963	73,98
0	0	2592424391	73,97
100	100	2729346249	77,87
0	0	3410403063	97,3
0	0	3371282194	96,19
100	100	3284548323	93,71

100	100	3336712383	95,2
0	0	2894560606	82,59
100	100	2352195974	67,11
0	0	2832362787	80,81
100	100	2236140880	63,8
100	100	2723377264	77,7
0	0	2705114290	77,18
100	100	2371713479	67,67
100	100	3415702278	97,45
0	0	2982711008	85,1
100	100	2781564220	79,36
100	100	3287835459	93,81
0	0	3533886607	100,83
100	100	3075714091	87,75
100	100	2301679462	65,67
0	0	2983295021	85,12
100	100	2589214852	73,87
100	100	2951922114	84,22
0	0	3958320968	112,94
100	100	3048485651	86,98
100	100	2493252852	71,14
0	0	3330464534	95,02
100	100	2733212129	77,98
0	0	2859544351	81,59
0	0	3610450898	103,01
100	100	2726793128	77,8
100	100	2998305773	85,55
0	0	3163330122	90,25
100	100	2991596369	85,35
100	100	2991981770	85,37
0	0	2847723533	81,25
100	100	3841948409	109,62
100	100	2698930560	77
0	0	3094766433	88,3
100	100	3174664652	90,58
0	0	3004789073	85,73
100	100	3652780657	104,22
100	100	2993022561	85,4
0	0	2754456255	78,59
100	100	2951336920	84,21
100	100	3552173845	101,35
0	0	3355388381	95,73
100	100	2779469041	79,3
100	100	3144365280	89,71
0	0	3407233998	97,21
100	100	2790243825	79,61
100	100	2689709632	76,74
0	0	2797953842	79,83
100	100	3196137956	91,19
100	100	3463233916	98,81
0	0	2767345310	78,96
100	100	2130989648	60,8

100	100	2917412984	83,24
0	0	2987671778	85,24
100	100	3027763404	86,39
100	100	2972592869	84,81
0	0	2480156300	70,76
100	100	3460204723	98,72
100	100	3107901939	88,67
0	0	3379317664	96,42
100	100	2700686642	77,05
100	100	3006202779	85,77
0	0	3456575343	98,62
100	100	2736724430	78,08
0	0	3131145572	89,34
0	0	2839659854	81,02
100	100	2703230128	77,13
100	100	3522296586	100,5
0	0	2399979148	68,47
100	100	2789121057	79,58
100	100	2690996541	76,78
0	0	2303888676	65,73
100	100	3089906463	88,16
100	100	2953100309	84,26
0	0	2776421245	79,22
100	100	2896799972	82,65
100	100	2681765911	76,51
0	0	2865904027	81,77
100	100	2956537296	84,35
100	100	3567776297	101,79
0	0	3003945566	85,71
100	100	2631946186	75,09
100	100	3115534329	88,89
0	0	2027850191	57,86
100	100	3563930644	101,68
100	100	3078804311	87,84
0	0	3308857289	94,41
100	100	3179976976	90,73
0	0	2458037089	70,13
0	0	2592624086	73,97
100	100	3251647589	92,77
100	100	2618478076	74,71
0	0	3303670345	94,26
100	100	2622695619	74,83
100	100	3174592024	90,58
0	0	2945376286	84,04
100	100	3154587756	90
100	100	3007384830	85,8
100	100	3248827437	92,69
100	100	2938070094	83,83
0	0	2660016875	75,89
100	100	3125573506	89,18
100	100	3280608779	93,6
0	0	2832008730	80,8

100	100	2726613512	77,79
100	100	3048549237	86,98
0	0	3020536353	86,18
100	100	3126434571	89,2
0	0	2878059364	82,12
0	0	2690318994	76,76
100	100	3124656738	89,15
100	100	3257656169	92,95
0	0	2670464237	76,19
100	100	2949464137	84,15
100	100	3406343955	97,19
0	0	3211635061	91,63
100	100	2977921764	84,96
100	100	3613147863	103,09
0	0	2894989137	82,6
100	100	2877317889	82,09
100	99,8	2415974848	68,93
100	100	3686237009	105,17
0	0	2522034473	71,96
100	100	2537212274	72,39
100	100	2415569699	68,92
100	100	2334349913	66,6
100	100	2513498707	71,71
0	0	1842000670	52,55
100	100	2404949758	68,62
100	96,4	2191958300	62,54
100	100	2118251040	60,44
0	0	4379673619	124,96
100	99,2	3129252315	89,28
100	100	2794921847	79,74
0	0	2910374080	83,04
100	100	2443723071	69,72
0	0	2373465060	67,72
100	100	2507445295	71,54
100	100	2465768529	70,35
0	0	2052353523	58,56
100	100	2259081349	64,45
0	0	3548160156	101,23
100	100	2729057167	77,86
100	100	3400059966	97,1
100	100	3360629324	95,88
0	0	2997188661	85,51
100	100	3465990481	98,89
100	100	2305955175	65,79
0	0	2290800067	65,36
100	100	2025791923	57,8
100	100	2895336282	82,61
0	0	2230387538	63,64
0	0	4213330353	120,21
100	100	5119887682	146,08
0	0	4349864643	124,11
0	0	2631373636	75,08

0	0	2488798750	71,01
100	100	2677313886	76,39
100	100	2525363760	72,05
0	0	2263816285	64,59
100	99,8	3637957417	103,8
0	0	4030809981	115
100	100	2048231305	58,44
0	0	1981966888	56,55
100	100	2410498751	68,77
0	0	2672952434	76,26
0	0	2162036329	61,69
100	100	2133171117	60,86
100	100	2768311497	78,98
0	0	2898426142	82,7
100	100	2484228182	70,88
100	100	2754215442	78,58
100	100	2454575137	70,03
100	100	2216435333	63,24
0	0	2482876986	70,84
0	0	2906821438	82,94
0	0	2213655894	63,16
100	100	2157841205	61,57
100	100	3971459980	113,31
0	0	4651528627	132,71
100	100	3774127225	107,68
100	100	2589753649	73,89
0	0	2180712210	62,22
0	0	2044802837	58,34
0	0	2327436560	66,4
100	100	2549437338	72,74
0	0	1945951610	55,52
0	0	2207704314	62,99
100	100	2167431416	61,84
0	0	2146712015	61,25
0	0	2232311645	63,69
100	100	2159347496	61,61
100	100	6364359033	181,58
100	100	1924541415	54,91
0	0	2470584750	70,49
100	100	2076826281	59,25
100	100	2389617770	68,18
0	0	1565552029	44,67
100	100	2871481054	81,93
100	100	2239642498	63,9
0	0	2349778884	67,04
100	100	1993503036	56,88
100	100	1468588352	41,9
100	100	2338895701	66,73
100	100	2217107966	63,26
100	100	2403384549	68,57
0	0	2567431756	73,25
100	100	2372238096	67,68

0	0	4431790623	126,45
0	0	4435903921	126,56
100	100	4617087680	131,73
100	100	2477485482	70,69
0	0	3038613010	86,7
100	100	1727490169	49,29
100	100	2515744515	71,78
0	0	2260213157	64,49
100	100	2358224399	67,28
100	100	1970577597	56,22
0	0	2207441183	62,98
100	100	2523259571	71,99
0	0	2727558985	77,82
0	0	2904466513	82,87
100	100	2522794129	71,98
100	100	2648399681	75,56
0	0	1991160110	56,81
0	0	2450557992	69,92
0	0	2432405533	69,4
100	100	2291611580	65,38
0	0	2292760286	65,42
0	0	2185010107	62,34
100	100	2810260916	80,18
0	0	2480809871	70,78
0	0	2094625604	59,76
0	0	2354536898	67,18
100	100	2224897597	63,48
100	97,9	2266957556	64,68
100	100	2342341948	66,83
0	0	1965705581	56,08
100	100	2496281999	71,22
100	100	2151249607	61,38
0	0	2497388288	71,25
100	100	2764339173	78,87
0	0	2606048864	74,35
100	100	2754648082	78,59
100	100	2764807936	78,88
0	0	2628652310	75
100	100	2378328535	67,86
100	100	2468789483	70,44
0	0	2198290119	62,72
100	100	2480924686	70,78
100	100	2173158820	62
0	0	2626820080	74,95
0	0	2723017500	77,69
0	0	2487566370	70,97
100	100	2782359178	79,38
100	100	2671167707	76,21
100	100	2243602247	64,01
0	0	2170427292	61,93
100	100	2456636289	70,09
0	0	2529887873	72,18

100	100	2429813008	69,33
0	0	2247114315	64,11
100	100	2233042464	63,71
100	100	2568665167	73,29
100	100	2141057408	61,09
0	0	2252939310	64,28
0	0	2407954227	68,7
0	0	2250434839	64,21
100	100	2227165997	63,54
0	0	2191726997	62,53
100	100	2801293688	79,92
0	0	2565077840	73,19
0	0	2513193244	71,7
100	100	2506013618	71,5
100	100	2472102599	70,53
0	0	2083632471	59,45
0	0	2345467901	66,92
0	0	2097733396	59,85
100	100	2842059608	81,09
0	0	3095837449	88,33
100	100	2967947404	84,68
100	100	2729243185	77,87
0	0	2070872445	59,08
100	100	2249416567	64,18
0	0	2329530904	66,46
100	100	2073561166	59,16
0	0	2280438438	65,06
100	100	2621049322	74,78
100	100	2891115562	82,49
0	0	2852201408	81,38
100	100	2812198772	80,24
100	100	2530042319	72,19
0	0	2622840698	74,83
0	0	2345088080	66,91
0	0	2289826636	65,33
100	100	2949655697	84,16
100	100	3840842536	109,58
100	100	3652892649	104,22
100	100	2419469830	69,03
0	0	2332607885	66,55
100	100	2794995499	79,75
100	100	2435206590	69,48
0	0	2380261082	67,91
100	100	3030612156	86,47
100	100	2112510421	60,27
100	100	2652399152	75,68
0	0	2428147344	69,28
100	100	2282182581	65,11
0	0	2469318254	70,45
0	0	2486042588	70,93
0	0	2301441099	65,66
100	100	2540022170	72,47

0	0	2282869835	65,13
0	0	2715109213	77,47
100	100	2799958760	79,89
0	0	2877130503	82,09
0	0	2442225849	69,68
100	100	2645398561	75,48
0	0	2342701201	66,84
100	100	2438333116	69,57
100	100	2337570366	66,69
0	0	2542396327	72,54
100	100	2750109156	78,46
0	0	3019757538	86,16
0	0	2345662566	66,93
100	100	2458836562	70,15
100	100	2460237775	70,19
0	0	2383425373	68
100	100	2068802431	59,03
100	100	2564569268	73,17
0	0	3026414433	86,35
100	100	2717564202	77,54
100	100	2642250565	75,39
0	0	2447508097	69,83
100	100	2141607153	61,1
0	0	2860346440	81,61
0	0	2587582905	73,83
100	100	2362847114	67,42
100	100	3109230709	88,71
0	0	2579982519	73,61
100	100	2802240126	79,95
0	0	2983333574	85,12
0	0	2889233408	82,43
100	100	2598078382	74,13
100	100	3229494230	92,14
0	0	3083071733	87,96
100	100	2860751489	81,62
100	100	2126380603	60,67
0	0	3309733876	94,43
0	0	2767654126	78,97
100	100	3230831652	92,18
100	100	2402434644	68,54
100	100	1830821677	52,24
0	0	3535272778	100,87
100	100	3972096213	113,33
0	0	3315088958	94,58
100	100	3742198728	106,77
0	0	3287550413	93,8
100	100	3454524268	98,56
100	100	2616277711	74,65
0	0	3415984681	97,46
100	100	3193006223	91,1
100	100	2635867405	75,2
0	0	3336423832	95,19

100	100	2609971521	74,47
100	100	3234628755	92,29
0	0	3569855150	101,85
100	100	3591929640	102,48
100	100	3491431853	99,62
0	0	4250155987	121,26
100	100	3782824169	107,93
100	100	3026451227	86,35
0	0	2827581399	80,67
100	100	2919858246	83,31
100	100	2362747185	67,41
0	0	2970668415	84,76
100	100	2917193380	83,23
100	100	3006004053	85,77
0	0	3508520180	100,1
100	100	3491607366	99,62
0	0	3684309198	105,12
100	100	3667729418	104,65
100	100	3678593827	104,96
100	100	3259427226	93
0	0	3193597712	91,12
100	100	3018349633	86,12
0	0	2245797096	64,08
0	0	2875596798	82,04
100	100	2896670507	82,65
100	100	3364979746	96,01
0	0	3053275415	87,11
100	100	2799545296	79,87
0	0	3729114842	106,4
0	0	3222338796	91,94
100	100	2901835777	82,79
100	100	2295940658	65,51
0	0	2778510345	79,27
100	100	3260861780	93,04
100	100	3229192744	92,13
0	0	3386283649	96,62
100	100	3147539042	89,8
100	100	3075394700	87,75
0	0	3110034102	88,73
100	100	3145699350	89,75
100	100	3591311512	102,47
0	0	4309406779	122,95
100	95,4	3139698818	89,58
100	100	2857339391	81,52
0	0	3090891010	88,19
100	100	3020760168	86,19
100	100	3549621167	101,28
0	0	3861152748	110,16
100	100	3486459458	99,47
100	100	3587988347	102,37
0	0	2637657476	75,26
100	100	2523425875	72

0	0	3618178571	103,23
0	0	3310807766	94,46
100	100	3268093906	93,24
0	0	2968068080	84,68
0	0	2509083740	71,59
100	100	2965379438	84,61
100	100	3282061972	93,64
0	0	3741713572	106,76
100	100	3049159639	87
100	100	3423880582	97,69
0	0	1685320194	48,08
100	100	2181838764	62,25
100	100	2806481770	80,07
0	0	2955547233	84,33
100	100	3161926613	90,21
100	100	3718078222	106,08
0	0	3513663833	100,25
100	100	2468111422	70,42
100	100	3052517679	87,09
0	0	4009634530	114,4
100	100	3633581374	103,67
0	0	3515657552	100,31
0	0	3097438323	88,37
100	100	3526130532	100,61
100	100	3729547262	106,41
0	0	4293934059	122,51
100	100	3400150680	97,01
100	100	3842773611	109,64
0	0	3587019794	102,34
100	100	3184134925	90,85
100	100	4081308488	116,45
0	0	2676992628	76,38
100	100	2180779572	62,22
100	100	2830876768	80,77
0	0	3357340413	95,79
0	0	3512854481	100,23
100	100	3807348109	108,63
100	100	3648538941	104,1
0	0	3488806703	99,54
100	100	3743362712	106,8
100	100	3613342037	103,09
0	0	3516578197	100,33
100	100	3251174696	92,76
100	100	3562587506	101,65
0	0	3567369365	101,78
100	100	3498821304	99,83
100	100	2817324683	80,38
0	0	3040893452	86,76
100	100	2587709921	73,83
100	100	4150728527	118,43
0	0	3799274823	108,4
100	100	3798190985	108,37

100	100	3465002628	98,86
0	0	3764988360	107,42
100	100	3478207413	99,24
100	100	3363404698	95,96
0	0	3035811713	86,62
100	100	3728753773	106,39
0	0	4933895921	140,77
0	0	3149133062	89,85
100	100	4223677976	120,51
100	100	2398723440	68,44
0	0	2640312375	75,33
100	100	2625938410	74,92
100	100	2490760014	71,06
0	0	3092471706	88,23
100	100	2966797143	84,65
0	0	3487572364	99,51
0	0	2317691163	66,13
0	0	1957411867	55,85
100	97,2	2720379902	77,62
100	100	2511395724	71,65
0	0	2729205688	77,87
100	100	2900328121	82,75
100	100	2807572716	80,1
0	0	2773073668	79,12
100	100	2749921633	78,46
100	89,7	2157102315	61,55
0	0	2166806479	61,82
100	81,8	1963513904	56,02
0	0	2373886301	67,73
100	95,4	2030359431	57,93
100	100	2943842173	83,99
0	0	3282448597	93,65
100	100	2842204544	81,09
100	100	3218047898	91,82
0	0	2928738244	83,56
100	100	1975403387	56,36
100	100	3014293836	86
0	0	3390863586	96,75
100	100	3238930062	92,41
100	99,2	3002678089	85,67
0	0	2742158743	78,24
100	99	2600997375	74,21
100	100	3453707701	98,54
0	0	2743450394	78,27
100	100	3976423883	113,45
100	100	3096464644	88,35
0	0	3254166375	92,85
100	100	2546226674	72,65
100	100	3275397991	93,45
0	0	3644540377	103,98
100	100	4005340936	114,28
100	100	2666488187	76,08

0	0	2749695907	78,45
100	100	2984664351	85,16
100	100	2913965544	83,14
0	0	2842281837	81,09
100	100	2784131124	79,44
100	100	3087385690	88,09
0	0	2859218383	81,58
100	100	2751967188	78,52
100	100	2569036299	73,3
0	0	2853390512	81,41
100	100	2745833299	78,34
0	0	3095009015	88,3
0	0	1905475514	54,37
100	100	3639932377	103,85
100	100	2431807130	69,38
0	0	3031862413	86,5
100	100	2967663842	84,67
100	100	2644636047	75,46
0	0	2792899654	79,69
100	100	2777364862	79,24
100	100	2458714934	70,15
0	0	2674016862	76,29
100	100	2895464492	82,61
100	100	2836446931	80,93
0	0	2948257941	84,12
100	100	2615125549	74,61
100	100	2289243995	65,32
0	0	2006074798	57,24
100	95	2721148166	77,64
0	0	2900904979	82,77
0	0	3131531505	89,35
100	100	2958408824	84,41
100	99,7	3083615273	87,98
0	0	3456790188	98,63
100	100	3825586199	109,15
100	100	2980270187	85,03
0	0	3056845505	87,22
100	100	2954024487	84,28
100	100	3882235613	110,77
0	0	1897878308	54,15
100	97,2	1985826553	56,66
100	99,7	2711894515	77,37
0	0	2244682546	64,04
100	100	3008150875	85,83
0	0	2721472992	77,65
0	0	3007473153	85,81
100	100	3102817942	88,53
100	100	3171316018	90,48
0	0	2880413586	82,18
100	100	2940488113	83,9
0	0	2939542194	83,87
0	0	2804519616	80,02

100	100	2740303382	78,18
100	100	2877575407	82,1
0	0	3060761230	87,33
100	100	3179662888	90,72
100	95,4	2485916018	70,93
0	0	2320569027	66,21
100	97,2	2645292770	75,47
100	100	2942332196	83,95
0	0	2712282923	77,39
100	100	2846789511	81,22
100	100	2882782415	82,25
0	0	2861694603	81,65
100	100	3004657174	85,73
100	98,2	2850329218	81,32
0	0	2203759561	62,88
100	95,4	2394124761	68,31
100	99,7	2103270309	60,01
0	0	2714164477	77,44
100	98,7	2325903434	66,36
100	97,2	3035099075	86,6
0	0	2144593522	61,19
100	100	3151381606	89,91
0	0	2670449655	76,19
0	0	2281367695	65,09
100	98,2	2052476475	58,56
100	100	2462056330	70,25
0	0	2887809189	82,39
100	97,1	2288541967	65,3
100	100	2026411975	57,82
0	0	2633754277	75,14
100	95,6	1850007502	52,78
0	0	2300078438	65,62
0	0	2945779716	84,05
100	97,2	2260251059	64,49
100	100	2453156686	69,99
0	0	1645261550	46,94
100	100	2282970509	65,14
100	91	1859292722	53,05
0	0	1840251142	52,5
100	99,7	2213911308	63,17
100	100	2440304261	69,63
0	0	2348842645	67,02
97,2	94,5	1282099440	36,58
100	100	6900977976	196,89
0	0	8380651286	239,11
100	100	2988962179	85,28
0	0	2176714773	62,1
100	93,6	2173771031	62,02
100	92,3	1906318911	54,39
0	0	1492988932	42,6
100	88,3	1963653523	56,03
100	70,7	1800131589	51,36

0	0	1713883579	48,9
97,1	70,8	1722026487	49,13
56,7	6,4	811888813	23,16
0	0	1011579217	28,86
95,6	43,2	1272935865	36,32
0	0	2072075387	59,12
0	0	2977753792	84,96
100	100	2335465163	66,63
0	0	2885497925	82,33
0	0	1690102695	48,22
100	100	2201923024	62,82
100	100	1945531022	55,51
0	0	2926650971	83,5
0	0	1976834219	56,4
100	100	4444899373	126,82
0	0	2598773788	74,15
0	0	2746374181	78,36
100	100	3208116407	91,53
0	0	3529495756	100,7
0	0	2212165826	63,12
0	0	2138851770	61,02
100	100	2557771025	72,98
100	100	3523366990	100,53
0	0	2966459849	84,64
100	100	3080931455	87,9
100	100	2585040417	73,75
0	0	2935755674	83,76
100	100	3019587431	86,15
100	100	4614714733	131,66
0	0	2648309720	75,56
100	100	2130020098	60,77
100	99,5	2381303480	67,94
0	0	2479302063	70,74
100	100	2328534919	66,44
100	100	2608508561	74,42
0	0	2319064899	66,17
100	100	2101701297	59,96
0	0	2235710097	63,79
0	0	2096237135	59,81
100	100	2163081212	61,72
0	0	3132357236	89,37
100	100	3169957589	90,44
0	0	3410938375	97,32
100	100	2854959064	81,46
0	0	4010711610	114,43
100	100	2957353040	84,38
100	100	2616149992	74,64
0	0	3349629439	95,57
0	0	2812548847	80,25
0	0	2971968486	84,79
100	100	2928409189	83,55
100	100	2778836668	79,28

0	0	3146587515	89,78
100	100	2672575133	76,25
100	100	3416923330	97,49
0	0	3379181002	96,41
100	100	2592149860	73,96
100	100	3004612006	85,73
0	0	3330329761	95,02
100	100	3898493186	111,23
0	0	2041703838	58,25
0	0	2230786896	63,65
100	100	2555288162	72,91
100	100	2355728268	67,21
100	100	3397962290	96,95
0	0	3188034791	90,96
100	91,7	3384255194	96,56
100	97,4	1448863611	41,34
0	0	1355856446	38,68
100	91	1483068909	42,31
88,8	52,9	1847286753	52,71
0	0	1716710916	48,98
68,9	46,9	1752250601	49,99
100	89,7	1436334107	40,98
0	0	2817915152	80,4
100	100	2301530097	65,67
97,1	59,1	1763552973	50,32
0	0	1620851706	46,25
81,4	53,4	2009434138	57,33
92,5	61,7	2190784926	62,51
86	55,4	2123360065	60,58
82,7	48	2185552906	62,36
0	0	1436305680	40,98
0	0	2187273705	62,41
0	0	2516335041	71,79
99,8	81,9	2584772524	73,75
100	66,3	2352695172	67,13
0	0	2506306502	71,51
63,2	24,3	1264483110	36,08
98,9	70,5	2407414832	68,69
0	0	2600737121	74,2
92,7	64,3	2273944998	64,88
100	78,8	2715888805	77,49
100	100	2818030476	80,4
100	100	2520764034	71,92
100	100	2469222279	70,45
0	0	1444762520	41,22
100	100	1622248902	46,29
100	100	2760106003	78,75
0	0	2390039649	68,19
100	100	3372700534	96,23
0	0	2208150559	63
0	0	2204690596	62,9
100	67,8	2518053447	71,84

91,5	55,4	1879226841	53,62
0	0	1409377289	40,21
83,4	27	1427395080	40,73
79,5	46,3	1543504231	44,04
0	0	1289295930	36,79
100	50,7	1641277193	46,83
85,7	54,9	2096571964	59,82
0	0	1993561829	56,88
80,9	45,9	1633322885	46,6
0	0	1963537978	56,02
0	0	1165507644	33,25
62,7	33,2	1346991352	38,43
0	0	1364974041	38,94
0	0	1432041874	40,86
48,9	21,2	1219986716	34,81
100	100	3210783458	91,61
0	0	3045610253	86,9
100	100	3595506182	102,58
100	98,9	3356220963	95,76
0	0	2249776790	64,19
74,4	36,8	1448768026	41,34
99	88,1	3266830429	93,21
0	0	3578289206	102,09
0	0	3150884089	89,9
100	99,3	3327640019	94,94
0	0	2568970208	73,3
0	0	2758387958	78,7
100	100	3228508161	92,11
0	0	2431027609	69,36
100	100	2069710429	59,05
0	0	2134725334	60,91
100	100	2142091754	61,12
100	100	2288143006	65,28
0	0	2342221933	66,83
100	100	2442838751	69,7
100	100	2942570770	83,96
0	0	2176183611	62,09
100	100	2562250090	73,1
100	100	2920759782	83,33
0	0	3476841479	99,2
100	100	2632934412	75,12
100	100	2269421788	64,75
0	0	2293110016	65,43
100	100	2382113157	67,96
100	100	1859176591	53,04
0	0	2741956751	78,23
100	100	2683797544	76,57
100	100	2462210373	70,25
0	0	1914192898	54,61
0	0	1883979579	53,75
100	97,1	1842475819	52,57
0	0	2020919631	57,66

94,1	68,6	1743707799	49,75
0	0	1574445374	44,92
100	76,5	1537657435	43,87
100	82,2	2247994803	64,14
0	0	1192414350	34,02
100	97,9	2396674408	68,38
100	85,8	1695588062	48,38
0	0	1613071327	46,02
100	74,4	1592379669	45,43
100	100	2475445704	70,63
0	0	2575468489	73,48
100	100	2412729931	68,84
100	100	2521159976	71,93
0	0	2305193851	65,77
100	100	1862963999	53,15
0	0	1712245221	48,85
0	0	1678733598	47,9
100	84,9	1784085634	50,9
100	91,7	1692292551	48,28
0	0	1085342790	30,97
100	91	1634669161	46,64
0	0	1590804512	45,39
0	0	1693114361	48,31
100	66,1	1688498357	48,18
0	0	1639357529	46,77
0	0	975218055	27,82
100	84,9	1582144742	45,14
100	100	2415331703	68,91
0	0	1988369144	56,73
100	100	2234809466	63,76
100	100	3758749191	107,24
0	0	3815884435	108,87
100	100	2232765144	63,7
0	0	2852273112	81,38
100	100	3182253993	90,79
100	100	2006270364	57,24
0	0	2810707971	80,19
100	100	2798210541	79,84
0	0	2782947472	79,4
100	100	2562280070	73,11
100	88,1	1686051158	48,11
0	0	1659545121	47,35
100	92,3	1647855940	47,02
100	100	2258692247	64,44
0	0	2521498005	71,94
100	100	2691217612	76,78
100	100	3811337429	108,74
0	0	3410689340	97,31
100	100	4052590380	115,63
100	100	2105752393	60,08
0	0	2869653528	81,88
100	100	2711849691	77,37

0	0	2569421495	73,31
0	0	1597296640	45,57
100	100	2110457520	60,21
100	100	2408110274	68,71
100	100	2769318652	79,01
0	0	1986579444	56,68
100	95,4	1910864119	54,52
0	0	1944360013	55,48
0	0	2049898396	58,49
100	98,2	1784235551	50,91
100	100	1896145482	54,1
100	100	2114911912	60,34
0	0	2759205319	78,72
100	100	1776482802	50,69
100	100	1792702334	51,15
89,4	68,6	3209265420	91,56
0	0	1717835852	49,01
96,3	69,4	2669989623	76,18
100	100	2553094255	72,84
89,3	47,6	1439143141	41,06
0	0	2188612572	62,44
100	100	2194205456	62,6
100	100	2821208774	80,49
100	100	2714600749	77,45
0	0	2392837644	68,27
100	100	2624922351	74,89
0	0	3297329238	94,08
100	100	3388448015	96,68
100	100	3806713989	108,61
100	100	3785748923	108,01
100	100	3528680045	100,68
0	0	3480256960	99,3
100	100	1802070102	51,42
100	100	3247941443	92,67
0	0	3551416292	101,33
100	100	3304177705	94,27
100	100	3904660200	111,41
0	0	2904213647	82,86
100	100	3372268225	96,22
100	100	2482665368	70,83
0	0	3276363394	93,48
100	100	3238417947	92,4
100	100	3471876141	99,06
0	0	2721191713	77,64
100	100	2410098641	68,76
0	0	2396548836	68,38
0	0	2510482486	71,63
100	100	2360181334	67,34
100	100	3395448616	96,88
0	0	2493975362	71,16
100	100	2802301519	79,95
0	0	3194621953	91,15

0	0	3921276990	111,88
100	100	3678758105	104,96
100	100	2866537028	81,79
0	0	2483862009	70,87
100	100	2970317614	84,75
100	100	2652817553	75,69
0	0	2653560724	75,71
100	100	3226473700	92,06
0	0	3111221226	88,77
0	0	2974916689	84,88
100	100	2815112556	80,32
0	0	3002105140	85,65
0	0	3162407922	90,23
100	100	3309305216	94,42
100	100	2563029796	73,13
100	100	2547020144	72,67
0	0	2298606454	65,58
100	100	3057667282	87,24
0	0	2910513311	83,04
0	0	2547365523	72,68
100	100	1785022232	50,93
100	100	4045217441	115,42
0	0	3621134147	103,32
100	100	3563538862	101,67
0	0	2963335207	84,55
0	0	2997607901	85,53
100	100	3588591540	102,39
100	100	2576986910	73,53
100	100	3565650271	101,73
100	100	3182353706	90,8
100	100	3141960162	89,64
0	0	3252413132	92,8
100	100	2630258605	75,04
100	100	2765744017	78,91
100	100	2960847829	84,48
100	100	2749487450	78,45
0	0	2566473670	73,23
100	100	2435587092	69,49
0	0	3007257866	85,8
100	100	2616943265	74,67
100	100	3020176970	86,17
0	0	2834326776	80,87
100	100	2707716773	77,25
0	0	2904232413	82,86
0	0	3213260029	91,68
100	100	3206700669	91,49
100	100	2843669807	81,13
0	0	3535581100	100,88
100	100	2769357602	79,01
100	100	2856101284	81,49
0	0	3132998516	89,39
100	100	3465265254	98,87

100	100	2495491143	71,2
0	0	2936350771	83,78
100	100	2812509034	80,24
100	100	3264313452	93,14
0	0	3531296146	100,75
0	0	3899832635	111,27
100	100	3332196746	95,07
100	100	3539066076	100,97
0	0	3542823666	101,08
100	100	3057140016	87,22
100	100	3611015692	103,03
0	0	3849485457	109,83
100	100	3529134997	100,69
100	100	2775047044	79,18
0	0	3235136827	92,3
100	100	3023267026	86,26
100	100	1561867884	44,56
100	100	1312823253	37,46
0	0	2313780306	66,02
100	100	3075874945	87,76
100	100	2030237190	57,93
100	100	1999240335	57,04
0	0	2960817625	84,48
100	100	2035021046	58,06
100	100	1766277766	50,39
100	100	2369415196	67,6
0	0	3067232193	87,51
100	100	2721734598	77,65
100	100	1664247643	47,48
0	0	2270737547	64,79
100	97,2	1342902086	38,31
0	0	2543161620	72,56
100	100	2199184409	62,75
0	0	2113004168	60,29
100	100	2164994825	61,77
100	98,4	2645485047	75,48
100	100	1755113259	50,08
100	100	1900843756	54,23
0	0	1742744246	49,72
0	0	2169058625	61,89
0	0	2472427664	70,54
100	100	2365854565	67,5
100	100	2481751155	70,81
0	0	2381809920	67,96
100	100	2077470563	59,27
100	100	1632960604	46,59
100	100	2216020297	63,23
0	0	2901611739	82,79
0	0	2832653523	80,82

%_bases_above_10	%_bases_above_20	%_bases_above_30	%_bases_above_40
97,2	96	92,4	82,4
97,2	96,1	92,7	82,8
97,1	95,8	92,1	82
97,2	95,7	90,6	77,8
97,1	95,9	91,7	79,8
97,1	95,8	91,5	80
97	92	82,3	70,5
97,1	92,1	82,6	71,9
97	93,2	85,4	75,7
97,1	92,8	84,4	73,5
97	91,7	82,3	72,2
97,4	94,9	89,6	82
97,3	93,2	85,1	75,2
97,4	94	87	77,7
97,6	94,6	88,9	80,8
97,2	92,4	83,5	72,9
97,3	93,6	86,5	77,5
97,1	91,4	81,7	71
96,7	89,6	77,2	64,3
97,4	93,5	85,9	76,8
97,5	94,3	87,8	79,2
97,6	95	89,7	82,1
96,4	88,3	74,8	61,2
97,7	95,3	90,1	82,3
97,3	93,5	85,9	76,1
97,4	94,6	88,8	81,1
97,6	94,5	88,7	81,3
96,6	89,6	78,8	67,9
97,4	94,1	87,3	78,5
97,2	93,2	85,1	74,6
97	92,5	83,3	72,2
96,7	90,5	80,3	69,4
97,1	92,8	84,3	73,7
97,6	94,7	88,7	80,4
97,2	93,9	86,9	77,1
96,8	90,4	79,4	67,7
97	95,4	89,8	75,9
96,9	95,8	92,2	81,4
97,1	95,6	90,7	78
97,5	94	87,3	78,8
97,5	94,8	89	80,8
97,3	93,6	86,2	76,6
97,4	95	89,8	82,1
97,3	94,1	87,3	78
97,2	93,2	85	74,3
97	91,6	82	71,6
97,6	94,6	88,5	80,4
97,2	92,4	83,7	73,6
96	87	72,7	58,8
97,5	94,1	87,4	78,5

96,9	91,1	80,8	69,6
97,1	93,5	86,2	76,7
97,3	94,6	88,7	80,2
97,4	93,4	85,7	76,2
97,1	96,1	93,5	85,5
97	95,8	93	85,5
97,2	96,1	92,4	82,7
97,8	95,4	90,4	83,6
97,2	93,2	84,8	73,9
97,4	93,6	85,9	75,6
97,5	93,9	86,8	77,6
97,4	94,4	88,1	79,2
97,3	93,1	85	75,1
97,1	92	82,6	71,9
97,2	94,4	88,4	80,2
97,7	95	89,4	81,8
97,7	95,7	91,2	84,3
97,3	94,2	87,5	78,1
97,3	93,1	85,2	75,5
97,2	91,8	82,3	71,8
96,9	90,6	79,9	68,5
97,7	94,5	88,3	79,9
97,6	94,4	87,6	78,8
97,1	92,9	84,3	73,7
97,6	94,4	87,6	78,3
97,4	93,2	85	75,1
97,1	92,7	84,1	73,9
97,5	94,6	88,6	80,6
97,4	93	85,1	75,7
97,4	93,3	85,5	76,2
97,6	94,5	88	78,9
97,3	96	91,5	80,6
97,1	95,9	92,4	82,6
96,9	95,2	89,8	77
96,9	91,1	80,9	69,8
95,9	86,9	72,2	58
97,5	94,8	89,5	82,2
97,3	93,6	86,1	76,2
96,5	90,5	79,1	66,2
97,6	94,7	88,9	81,1
97,8	95,4	90,5	83,1
97,1	92,7	83,8	73,1
97,2	92,5	84	74,4
97,1	95,6	90,9	78,4
97,1	95,8	91,6	80,5
97,2	95,3	88,5	73,6
97,1	95,2	87,4	70,9
97,2	96,4	94,7	90
97,3	95,9	91,2	80
97,1	96,1	93,9	88,3
97	96	94,2	89,5
97,1	95,8	92,2	83,2

97,1	95,6	91,5	81,3
96,8	95,1	89,6	75,6
97	95,5	91,3	81
97,3	95,7	90,7	78,5
96,9	95,6	91,3	80
97,3	96,1	91,9	81,6
97,2	95,8	90,8	78
96,9	94,8	87	70,4
97,2	95,4	89,4	75,7
96,9	96	94,7	92,2
97,3	96,4	93,8	85,8
97,2	96	92,6	83,9
97,2	95,7	90	76,1
97,2	95,9	90,8	77,8
97,2	96,1	93,1	85,4
97,3	95,5	89,5	75,9
97,2	95,9	91,5	80,1
97,4	96,5	93,9	86,8
97,3	96,3	93,2	84,9
97	95,4	89,1	74,4
97,2	95,3	89,1	75,2
97,1	95,3	88,7	74,3
97,1	96	93,2	85,4
96,9	95,5	91,9	83
96,9	95,9	94	89,7
97	96	93,6	86,8
97,2	95,5	90,1	77,5
97,2	95,7	90,7	78,9
97,1	96,1	93,8	87,2
97,2	96,3	94,1	88,5
97,1	95,5	90,2	78
96,9	95,6	91,8	81,4
97,2	96	93	85,4
96,7	95,3	92,2	84,3
97,1	96,1	94	88
96,8	95,4	91,6	82,3
97	96	93,8	88,5
97	95,8	92,6	83,8
97,1	96,2	94,6	90,8
96,9	95,6	92,5	83,7
97,1	95,8	92,1	82
97,3	96,2	93,2	85,6
97,1	96,1	93,5	85,7
97	95,8	91,8	80,1
97	95,8	92,5	83,6
97,2	96	92,5	82,8
97	95,8	92	80,9
97,1	95,7	91,3	79,9
97,1	95,4	89,3	74,7
96,9	95,7	91,9	81,1
97,1	96	93,1	85,3
97,2	96,2	93,8	87,1

97,1	95,9	92	80,5
97,1	96,1	93,6	86,8
97,1	95,7	91,6	80,7
96,9	95,4	89,7	74,8
97,1	95,8	91,8	81,2
97,1	96,1	92,5	82,1
97,1	95,8	91,9	81,2
97,1	95,1	87,8	71,5
97,2	96,3	93,6	85
97,1	95,7	89,8	74,9
97	95,5	90,5	77,8
97,1	96	93,5	86,5
97,1	96	92,4	82
97,2	96	92,1	81,7
79,3	74,3	68,3	60,9
97,5	96,5	95,1	93,2
84,2	80,9	76,9	71,7
84,1	80,1	75,1	68,5
85,5	82,9	80,5	77,6
97,1	95,3	92,5	88,5
96,9	94,5	90,5	84,9
92	85,9	74,8	61,3
96,5	93,3	88,3	81,5
91,9	85,8	74,2	60,6
96,2	91,9	84,3	74,4
96,8	93,8	89	82,3
96,2	92	84,8	75,4
79,8	73,4	64,8	54,1
83,3	80,3	77	72,8
97,2	95,8	93,7	91,1
97,4	96,5	95	93
96,8	94,3	90,2	84,3
94,8	85,8	72,7	58,6
86,4	61	36,6	19,7
97,1	95,5	93	89,5
97,1	95,2	92,2	88,1
97,4	96,2	94,4	91,9
97,4	96,3	94,8	92,7
97,2	95,6	93,2	89,9
97,1	95,4	92,9	89,6
96,7	94,4	91	86,3
96,6	93,8	89,6	84
96,5	93,3	88,5	82,1
97,3	95,9	94,1	91,7
96,8	94,3	90,5	85,5
96,8	94,6	91,5	87,4
96,5	93,5	89,1	83,3
96,9	94,9	92,1	88,2
96,8	94,6	91,5	87,4
96,8	94,3	90,5	85,5
96,7	94,2	90,5	85,6
96,9	94,8	91,8	87,7

97	94,8	91,7	87,5
97,5	96,3	94,5	92,3
95,8	91,2	84,3	75,6
97	95,3	92,9	89,6
96,5	93,7	89,6	84,1
96,4	93,5	89,2	83,5
94,7	87,4	76,3	63,4
96	92	86	78,1
97	95,3	92,8	89,4
96,8	94,6	91,4	87,1
96,9	94,6	91,1	86,4
97,1	95,4	92,7	88,9
97,4	96,4	95	93,1
95,1	88,6	78,9	67,4
97,1	95,4	93	89,6
96,6	94	90,1	84,9
95,8	91,2	84,2	75,2
96,1	92,3	86,5	79
97	95,3	93	89,9
97,1	95,4	93	89,8
96,5	93,8	89,8	84,4
97,1	95,6	93,4	90,4
96,2	93	88,2	81,8
93,7	84	70,3	55,8
96,5	93,9	89,8	84,2
97	95,8	92,8	84,7
96,6	95,2	91,1	79,5
97,1	95,7	91,1	79,4
94,5	87,2	76,5	64,2
93,9	84,2	70,3	55,5
97,3	95,8	93,6	90,7
95,3	88,7	78,4	66
96,6	93,5	88,5	81,9
96,8	94,5	86,3	69,2
96,8	95,5	91,5	79,9
97,2	95,9	92,3	82,4
96,2	92,5	86,8	79,3
97,4	95,9	93,9	91
97,3	96,1	92,2	81,7
97,2	96,3	93,8	86,7
97,3	96,3	93,7	86,6
96,3	88,7	76,7	64,6
97,4	94,3	88,1	79,8
96,7	90,3	79,1	66,8
97,1	92,6	84,3	74,2
97,3	93,2	85,3	75,7
97,2	93,2	85,7	76,6
97,4	93,5	85,8	76,5
97,3	93,4	85,7	76,1
97,6	94,5	88,2	79,7
97,2	92,8	84,9	75,5
97,3	92,6	83,9	73,9

97,1	92,9	85	75,2
97,6	93,9	86,9	77,8
97,1	92,6	84,9	76
97,2	93,4	86,1	76,9
97,5	94,4	87,9	78,8
97,4	94,5	88,5	79,9
97,3	94,1	87,6	78,3
97,7	94,7	88,8	80,9
97	91,3	81,5	70,8
97,5	93,7	86,1	76,4
97,1	92,4	82,9	71,1
97,5	94,7	88,6	80,1
97,4	94	87,2	78,2
97,4	93,9	86,9	77,7
97,5	94,9	89,4	81,2
97,6	94,7	88,7	80,5
96,8	91,7	81,9	70,4
97,2	93,8	87,1	78,8
96,8	91	80,8	69,4
97,4	93,5	85,9	76,5
97,2	93,1	85,1	75,5
97,3	92,9	84,8	75,1
97,5	94,9	89,7	82,1
97,5	94,4	87,8	78,5
97	92,6	83,7	72,6
97,6	94,8	89,1	81,1
97,5	94,8	89,1	81,4
97,2	91,9	82,3	71,5
97,5	93,9	86,9	78,2
97,1	92,8	84,4	74,2
94,3	81,6	67	54,3
97,3	93,6	86	75,8
97,3	92,2	83,1	73,1
97,6	94,4	87,8	79
97,2	92,7	84,4	74,5
97	92,2	82,4	70,6
97,5	94,9	89,6	81,6
97,4	94,9	89,7	81,9
97,3	94	87,1	77,9
97	92,1	82,6	71,1
97,3	93,3	85,7	76,2
97,7	94,6	88,7	81
97,6	94,1	87	77,9
97,6	94,6	88,3	79,5
97,1	92,6	84,3	74,8
97,6	94,3	87,3	77,7
97,2	92,7	83,7	72,6
97	92,4	83,1	71,7
97	92	82,9	72,5
97,5	94,6	88,8	81,1
97,5	93,9	86,8	78,3
97,5	94	87,2	78,7

97,7	94,2	87,3	78,7
97,3	94,2	87,5	78
96,9	91,6	81,2	68,8
97,2	92,3	83,5	73,2
96,7	89,9	78	65,2
97,3	93,2	84,9	74,4
96,8	92,6	84,2	73,5
97	91,3	80,5	68,2
97,6	94,2	87,7	79,6
97,5	94,7	89	80,4
97,2	93,3	85,5	75,3
97,7	94,7	88,4	80,1
97,7	95,3	90,1	82,6
97,3	93,6	86	76,7
96,8	90,9	80,2	67,7
97,6	94,7	88,3	79
97,2	92,4	83,2	72,1
97,3	92,8	84,4	74,6
97,8	96	92,2	86,5
97,4	93,6	86,1	76,7
97	92	82,6	71
97,5	94,6	88,5	80,3
97,4	93,8	86,4	76,3
97,3	93,9	87	77,5
97,4	95,1	90	82,7
97,3	93,1	84,8	74,3
97,4	93,3	85,6	76,1
97,2	92,9	85	75,9
97,4	93,2	85,3	75,7
97,2	92,7	84,3	74,6
97,3	93,2	85	75
97,8	95,7	91,6	85,2
97,3	92,9	84,4	73,8
97,5	94,9	89,4	81,1
97,5	94,6	88,7	80,2
97,5	94,9	89,3	80,7
97,8	95,6	91	84
97,6	94,4	87,8	78,6
97,4	94,4	87,8	78
97,2	93,3	85,6	76,1
97,8	95,3	90,2	82,9
97,5	94,2	87,5	79,1
97,5	93,6	85,6	75,3
97	93,5	86,8	77,9
97,7	94,7	88,6	80,5
97,3	93,5	86	75,7
97,1	92,6	83,8	73
97,2	93,9	86,6	76,6
97,6	93,9	86,8	77,9
97,8	95,3	90	82,4
97,2	92,4	83,1	72,6
96,4	88,9	76,1	62,7

97,3	92,8	84,5	74,5
97,2	92,9	85	75,4
97,5	94,1	87	77,7
97,3	93,4	85,7	76,1
96,7	89,6	78,2	66,8
97,6	94,5	88,4	80,5
97,4	93,5	86	76,7
97,5	94,2	87,6	79,2
97,1	92,5	83,6	72,9
97,4	93,7	86,3	76,8
97,4	95,1	90,1	82,7
97,2	92,4	83,6	73,1
97,6	94,4	88,1	79,4
97,2	92,7	83,8	73,5
97,2	92,1	82,8	71,9
97,6	94,6	89	81,7
96,7	90,4	79,5	67,6
97,4	93,9	86,6	76,6
97,4	93,3	85,1	74,4
96,4	88,8	76,6	64,6
97,4	94,2	87,6	78,6
97,3	92,3	83,5	73,6
97,3	93,7	86,4	76,4
97	92,9	84,8	74,8
97,3	93,5	85,7	75,1
97,3	93,5	85,6	75,5
97,6	94,6	88,4	79,4
97,8	95,6	91	84,2
97,3	93,6	86,4	77,3
96,9	90,9	80,6	69,7
97,4	93,4	85,7	76,4
96,3	88,3	74,6	61
97,7	95	89,6	82,1
97,2	93,9	87,1	78,1
97,6	94,6	88,2	79,7
96,3	90,7	82,8	74,5
96,8	91,6	81,5	69,6
97,1	92,3	83	71,8
97,4	93,8	86,8	78
97,2	92,6	83,6	72,6
97,7	95	89,4	81,5
97,2	92,1	82,5	71,4
97,4	93,4	85,9	76,9
97,3	93,6	86,1	76,4
97,5	94,5	88,4	79,9
97,4	93,7	86,4	76,9
97,3	93,3	85,9	77
97,5	93,2	84,9	74,9
97	93	84,6	73,8
97,5	94	87,4	78,7
97,6	95,1	89,9	82,1
97,3	94	86,9	77

97,1	92,4	83,6	73,1
97,4	93,4	85,9	76,7
97,4	94,7	89	80,4
97,6	94,4	87,7	78,8
97,3	93,9	86,6	76,8
97	91,8	82,2	71,5
97,4	93,9	86,8	77,7
97,6	94,8	89,1	80,9
97,1	92,6	83,7	72,9
97,3	93,3	85,4	75,6
97,1	94,6	89,4	81,7
97,5	93,9	86,7	77,8
97,4	93,4	85,5	75,8
97,6	95,1	90,2	83,2
97,2	93	85	75,1
97,4	93,6	85,9	76,1
95,6	89,1	80,5	70,9
97,2	96,5	95,6	94,5
96,9	95,9	93,7	86,8
97,1	95,9	93,1	85,3
91,7	85,2	73,6	60
91,7	84,4	71,6	57,3
96,4	94,8	91	83,2
95,3	89,6	78,5	63,8
96,9	95,3	91,2	82,8
91,4	83,4	69,7	55,2
91,2	82,5	68,3	53,6
93,2	86,9	80,8	74,8
93,6	86,3	78,5	70,8
96,8	95,9	94,2	90,1
96,7	95,8	94,5	91,5
97,4	96,4	92,9	83,6
97,3	96,3	92,2	81,3
97,3	96,3	93	84,1
96,7	95,5	92,9	86,1
96,9	94,8	87,1	70,1
97	95,4	90	76,9
97,2	96,7	95,9	94,7
96,6	95,5	93,7	89,2
96,9	96,2	95,1	93,3
95,4	90	83,9	77,3
95,8	90,2	83,4	76
94,4	88,9	83	76,9
97,3	95,8	90,9	79,2
97,2	95,7	90,3	77,7
97,4	94,5	84,2	67,3
97	96,2	94,7	91,4
96,8	95,3	90,3	76,9
97	96,4	95,7	94,5
97,3	96,7	96,1	95,3
97	96,4	95,8	94,8
96,6	95,6	93,7	88,6

96,6	95,4	93	86,1
96,6	95,5	93,5	88,4
97,5	96,5	93,2	84,5
97,3	96,1	91	78,3
96,8	93,1	88,4	82,9
96,6	93	88,4	83,3
97,3	95,6	88,4	72,8
97,2	95,3	87,1	69,7
97,4	96,4	92,9	83,7
97	96,1	93,7	86,9
97,2	95,6	89,2	74,4
97,4	95,6	88,2	73,4
96,7	95,7	93,9	89,7
96,7	95,8	94,5	91,6
96,3	94,9	92,3	86,2
96,9	95,9	94,2	89,8
96,6	95,4	92,6	85,1
97,4	95,9	89,9	76,6
97,3	96,5	93,5	84,6
96,9	96,2	94,7	90,6
96,8	95,5	90,8	78,6
97,3	95,7	89,1	74,9
97,2	96,5	95,5	93,5
97,2	96,5	95,9	95,1
97,3	96,5	95,2	92,6
97,4	96,6	93,8	86,2
97,3	96	90,5	76,9
97,2	95,4	87,3	70,7
97,3	96,2	92	80,8
97,5	96,5	93,4	85,1
97,2	94,4	82,9	64,5
97,2	95,9	90,1	76,4
97,4	95,8	89,8	76
97,3	95,9	90,2	76,1
97,3	96,1	91,1	78,4
97,3	95,7	89,5	75,6
97	96,4	96	95,5
96,8	93,8	83,3	65,7
97,4	96,4	92,5	82,5
97,4	95	86,1	70
96,4	94,9	91,6	83,3
96,1	90,7	72	48,5
96,8	95,8	94,3	90,7
97,3	96	90,6	77,9
97,3	96,1	91,8	80,4
96,6	93,9	84,6	68
96,4	86,6	64,2	44,1
97,4	96,2	92,2	81,9
97,4	96	90,2	77
97,4	96,4	92,7	83,1
97,3	96,6	94	86,3
97,4	96,3	92,2	81,8

97,2	96,6	95,9	94,8
97,1	96,5	95,8	94,8
97,3	96,6	96	94,9
97,4	96,4	93,2	84,7
97,3	96,6	95,2	91
96,9	92	76,5	56,1
97,3	96,3	93,1	84,6
97,3	96	90,6	77,6
97,4	96,3	92,1	81,6
97,3	95	85,7	68,1
97,3	95,8	89,4	74,9
97,4	96,5	93,2	84,6
96,6	95,6	94,1	90,2
96,6	95,7	94,3	91
96,8	95,6	93	86,2
97,2	96,2	93,3	86
97,2	94,7	84,6	66,8
97,2	96,2	92,9	83,8
96	94,6	92	85
97,4	96,1	91,3	79,5
96,4	95	91,9	82,9
96,6	94,9	89,2	75
97,3	96,5	94,4	88,9
97,3	96,3	92,8	83,1
97,2	94,3	83	66,2
97,3	96,1	91,5	79,5
97,3	95,6	89,1	74,8
98,1	93,6	84,6	73,1
96,9	95,4	90,6	79,4
96,9	94,4	84,6	66,7
96,6	95,4	92,2	84,2
97,3	95,5	89,1	75
97,2	96,2	93	84
97,4	96,6	94,6	89
97,2	96,4	94,2	87,3
97,3	96,5	94,3	88,6
97,5	96,6	93,4	85,6
97,2	96,3	93,5	85,5
97,3	96	91,5	80,6
97,4	96,3	92,3	82,1
97,2	95,6	89,1	74,5
97,4	96,2	91,7	80,9
97,2	95,4	88,8	74,5
97,4	96,5	93,4	84,5
96,7	95,7	94	89,2
96,4	95,2	93	86,5
97,5	96,7	94,1	87,3
97,2	96,3	93,6	86,6
97,3	95,7	89,9	76,7
97,1	95,6	89	74,1
97,4	96,2	92	81,6
97,3	96,4	93	83,6

96,5	95	91,1	81,9
96,9	95,4	89,9	77,3
97,4	95,8	89,7	76,1
97,5	96,5	92,9	83,8
97,3	95,2	87,4	72,2
97,1	95,7	89,9	76,4
97,2	96,1	92,3	81,7
97,2	95,7	89,7	75,8
97,3	95,7	89,4	75,3
97,2	95,7	89,1	74,4
97,5	96,7	94,5	88,5
97,3	96,5	93,4	84,3
97,2	96,2	92,4	82,3
97,3	96,3	92,9	84
97,4	96,2	92,3	82,3
97,2	95,1	86,1	69,2
97,3	96	90,9	78,2
97,2	95,3	87,2	70,8
97,4	96,6	94,3	88,1
97,4	96,8	95,4	91
97,5	96,7	94,4	88,2
97,4	96,6	94,3	87,9
97,2	95,1	86,4	69,1
97,3	95,7	89,4	75,5
97,3	96	90,9	78
97,3	95,4	88	72,4
97,1	95,8	90,7	78,4
97,3	96,3	93,5	86,1
97,4	96,7	94,9	90
97,3	96,6	94,5	88,4
97,3	96,5	94,1	87,9
97,4	96,3	93,1	84,4
97,4	96,5	93,7	85,3
97,3	96,1	91,8	80,2
97,3	96	90,4	76,9
97,5	96,9	95,1	90,2
98,7	98,1	96,4	93,3
98,6	98	96,5	93,4
97,3	96,1	91,5	80,5
97,3	96	90,5	77,4
97,5	96,7	94,3	87,6
97,4	96,3	92,3	81,8
97,3	96,4	93,3	83,8
97,5	96,8	95	90
97,2	95,3	88	73
97,4	96,7	94,6	88,9
96,7	95,5	91,3	80,6
97,2	95,6	90,2	77,5
96,9	95,8	92	81,9
97,1	96,1	92,4	82,3
96,9	95,6	90,8	78,7
97,3	96,3	92,9	83,9

97,1	95,7	90,4	77,5
97,4	96,6	94,1	86,5
97,5	96,6	93,7	86,3
97,4	96,6	94,3	87,5
97,1	96,1	92,7	83
97,4	96,5	93,7	86,3
97,3	96	90,5	77,7
97,4	96,2	91,9	81,3
97,3	95,9	90,9	79,2
97,1	96,1	93,1	84,4
97,5	96,7	94,1	87
97,4	96,8	95	89,6
97,1	95,9	91,6	80,2
97,3	96,1	92,5	83,1
97,4	96,3	92,7	83
97,2	96,1	92	80,7
97,3	95	86,1	69,3
97,4	96,3	92,9	83,8
97,4	96,8	95,1	90
97,5	96,2	92	82,8
97,2	96,2	93,3	85,7
97,1	96,1	92,4	82,2
97,2	95,2	87,6	72,4
96,7	95,7	94,3	90,8
96,6	95,5	93,6	88,1
96,7	95,3	91,8	82,6
96,9	96,1	94,9	92,5
96,8	95,7	93,5	86,9
96,9	96	94,3	90,1
96,9	96,1	94,8	92
96,7	95,9	94,4	90,7
96,7	95,6	93,5	88,2
97	96,3	95,2	93,2
96,9	96,1	94,9	92,3
96,7	95,8	94,2	90,8
96,6	94,6	87,9	72,9
96,9	96,2	95,2	93,4
96,8	95,8	94,2	89,9
96,9	96,1	94,9	92,5
96,6	95,3	92,3	84,4
95,5	90,2	80,2	66
97,8	95,3	90,2	82,9
97,9	95,9	91,7	85,7
97,8	95,3	89,7	81,8
98	95,9	91,7	85,4
97,6	94,2	87,7	79,5
97,9	96,1	92,3	85,8
96	89,9	80,2	69,3
97	93,4	87,3	79,4
96,6	91,4	83,6	74,9
96	89,4	79,4	68,8
96,7	91,8	84,4	76,1

96	89,1	78,9	68,1
97,6	94,3	87,8	79,5
97,6	94,7	89	81,5
97,9	95,6	90,5	83,2
97,9	95,4	90,2	82,7
97,9	96,1	92,5	87
97,8	95	89,6	82,5
96,6	91,4	83,2	74
96,6	91,3	82,5	72,5
96,5	91	82,3	72,7
95,6	87,1	75,4	63,6
96,3	90,3	81,4	72
96	89,4	80,4	70,9
96,7	92,5	85,4	76,4
96,7	92,5	85,8	77,9
97	93	86,5	78,7
97,7	95,1	89,9	82,9
97,9	95,5	90,4	83,3
98	95,9	91,8	85,6
96,3	90,7	82,9	74,4
96,6	92,3	85,2	76,6
96,4	91,5	83,7	74,3
95,4	86,5	74	61,9
96,4	90,9	82	71,9
96,6	91	82,2	72,4
97,1	93,1	86,4	78,2
96,6	91,3	83,1	73,9
96,5	90,7	81,5	71,3
96,9	93	86,9	79,7
97	93,3	86,9	78,6
96,4	90,6	81,8	72,2
95,3	86,1	74	62,3
96,3	90,1	80,6	70,4
96,2	90,3	82,4	73,9
96,7	91,9	84,4	75,8
96,8	92,7	85,7	77,3
97,2	92,9	85,6	76,7
96,5	91,1	82,8	73,7
97	92	84	74,7
96,7	91,8	83,9	74,9
97,6	94,7	90,3	85
97,5	94,7	91	86,6
97	93,2	88,1	81,9
95,7	88,6	79,2	69,6
96,6	92,2	84,9	75,9
96,4	91,5	83,7	74,4
97,9	95,9	91,4	84,6
97,9	96,1	92,1	85,9
97,7	94,7	88,9	81,3
97,1	93,2	86,9	79,4
96,1	90,3	81	70,2
96,1	89,6	79,4	68,1

97,7	95,1	90,2	83,4
97,7	94,9	89,3	81,6
97,7	94,9	89,1	81,2
96,7	91,6	83,3	73,6
96,1	89,9	79,8	68,4
96,6	92	84,4	75,1
97,7	95	89,3	81,1
97,7	95,2	90,3	83,5
97,6	94	87,1	78,3
97,2	93,8	89,2	83,7
92,6	81,6	68,3	54,5
95,1	87,9	78,6	68,1
96,3	89,9	80,5	70,5
96,8	92,1	84,1	74,4
96,7	91,9	84,3	75,5
97,9	95,9	91,4	84,7
97,7	94,9	89,4	82
97	91,1	80,9	69,6
97,8	94,9	88,8	80,1
97,8	95,8	91,6	85,6
97,9	95,6	90,6	83,5
96,5	91,3	84,1	76,3
96,9	93	86	77
96,7	91,9	85	77,2
98,1	96,2	92,1	85,6
98	96,5	93,5	88,6
98	96	91,3	84
97,9	95,5	90,8	84,4
97,7	95,2	89,9	82,8
97,5	93,8	86,9	78,4
97,1	93,2	87,5	80,8
96,2	90	80,3	69,7
95,2	87,4	75,5	62,4
96,5	91,4	83,1	73,2
97,6	94,8	89,2	81,5
97,6	94,7	89,1	81,7
98	96,2	92,5	86,4
98	95,7	91,2	84,5
97,6	94,7	88,9	81,5
97,8	95,2	90,1	83
98,1	96	91,7	84,9
97,8	95,4	90,3	83,1
97,7	94,3	87,7	79,3
97,5	95	89,8	82,4
97,7	95,3	90,1	82,6
97,9	95,2	89,7	82,2
97,2	92,5	83,9	73,8
97,2	92,3	84,2	75,1
97,1	91,8	82	70,9
97,9	96	92,2	86,6
97,6	95	90	83,4
98	96	91,9	85,7

97,8	94,9	89,3	81,7
97,8	95,4	90,7	84,1
97,8	95	89,5	82
97,7	94,5	88,3	80,3
97,7	94,6	88	79
97,8	95,1	90,1	83,2
97,5	95,3	92,3	88,7
93,7	86,3	78,6	71
96,2	91,7	86,5	81
96,7	90	78,8	67
97,2	92,6	83,6	72,7
97	91,1	81,1	70,3
97,3	92	81,9	70,2
97,5	94,3	87,6	78,6
97,5	93,5	85,8	76,2
97,9	96,3	93,1	88,1
93,9	87,1	78,9	70
91,7	82,8	72,7	62
94,1	88,1	81,5	74,3
93,9	87,5	80,4	72,5
94	88,3	82,4	75,6
94,5	89,1	83,3	77,1
97,3	92,9	84,6	74,4
97,3	93,1	84,8	74,5
97,1	92,4	83,7	73,2
89	76,7	65,3	55,6
90	78,7	67,7	57,8
89,4	77,1	65,1	54,3
91,9	82,4	72,4	62,8
89,2	76,8	64,9	54,4
97,1	92,3	83,9	74,2
97,3	92,8	85,4	77
97,5	93,4	85,3	75,3
97,4	93	85,5	76,8
97,5	93,6	85,8	76,1
96,1	86,7	72,5	58,7
97,5	93,7	86,2	76,8
97,5	93,6	86,6	78,6
97,7	94,5	88,2	79,7
95,2	89,6	83,3	76,3
94,5	88,3	81,2	73,4
94,7	88,1	80,5	72,1
96	90,9	84,8	78,3
94,8	88,2	80,6	72,3
96,8	93,1	88,5	83,4
97,7	94,6	88,1	79,3
97,7	94,9	88,7	80,3
97,3	92,2	82,7	71,3
97	92,8	87,1	80,6
97,3	94,4	90,3	85,2
97,5	95	91,4	87,1
97,1	91,4	81,7	71

97,4	93	84,3	73,8
97,2	92,4	84	74,5
97,4	93	84,8	74,9
97,2	92,8	84,5	74,4
97,3	92,7	84,1	73,9
97,5	93,7	86,3	77,1
97,2	93	84,7	74,7
97,2	92,9	84,3	73,8
96,9	91,1	80,9	69,8
97,2	92,7	84,2	74,3
97,2	92,6	83,9	73,4
96,8	92,8	87,2	80,5
93,3	82,9	71	58,8
97,3	94,3	90	84,9
97	91,2	80,7	68,8
97,5	94,4	87,4	77,9
97,7	94,5	87,8	78,6
97,2	92,2	82,8	71,8
97,4	93,1	84,8	74,6
97,1	91,5	82,2	72
97	91,4	81,1	69,3
97,3	92,7	83,9	73,2
97,3	92,3	83,6	73,7
97,3	92,7	84,2	74
97,5	93,9	86,5	76,9
97,1	91,8	82,1	71,1
87,3	77	67,3	58,5
85,1	73	62,5	53,6
89,5	80,3	71,7	63,7
97,3	92,8	84,3	74,5
97,5	94,5	88,1	79,4
97,4	93,3	85,4	75,9
95,9	90,8	84,4	77,4
96	91,3	85,7	79,5
96,9	93,1	88,3	82,8
97,5	93,7	86,2	76,6
97,5	93,8	86,4	77,2
97,4	93,6	86,1	76,5
97,4	94,6	90,9	86,4
94	84,3	72,6	60,3
94,4	85,7	74,8	63,2
95,8	90,2	83,2	75,2
95	87,8	78,6	68,3
96,4	91,7	85,7	78,9
97,1	91,8	82,5	72
97,3	93	85	75,6
97,4	93,7	86,4	77,4
97,6	94,1	87,1	78,2
97,2	92,6	84,1	74,2
97,2	92,6	84,4	74,7
97,3	92,8	84,4	74,7
97,3	93,1	84,7	74,5

97,2	92,7	84	73,5
97,5	93,4	85,4	75,5
97,5	93,7	86,4	77,2
97,5	93,9	86,9	78,1
93,8	85,5	76,1	66,3
95,2	88,3	79,5	69,5
96,3	91	83,6	74,9
97,5	93,5	85,7	76
97,2	92,3	83,1	72,4
97,2	92,6	84,1	74
97,3	93,1	85,1	75,2
97,3	93,2	85,1	75,2
97,4	93,3	85,5	76
96,5	91,5	84,8	77,1
94,7	86,8	77,2	66,8
95,4	88,7	80,2	70,6
94,7	87	77,1	66,1
96,4	91,3	84,4	76,5
95,7	88,9	79,9	70
97	92,8	87	80,1
94,7	86,7	76,8	66,1
96,9	92,7	86,9	80,2
96,5	91,2	83,8	75,2
96,1	89,3	79,9	69,4
95,3	87	76	64
89,1	79,4	70,2	61,7
90,4	82,3	74,7	67,3
85,3	73,8	63,9	55,5
95,2	87	75,8	63,7
96,5	90,9	83,2	74,4
94,2	83,7	70,8	57,7
95	87,5	78,4	68,5
96,3	90,9	84,1	76,7
95,2	87,6	78,1	67,8
95,1	88,3	79,9	70,8
92,2	80,1	66,2	52,2
95,4	88,5	79,5	69,5
83,8	70,7	59,4	50,2
83,7	71,1	59,7	50,2
86,4	75,5	65,6	56,7
95,7	89,1	80,8	71,5
95,5	88,7	79,9	70,2
88,8	72,2	54,6	38,9
97,3	96,7	96,2	95,8
97	96,5	96,1	95,8
95,5	89,1	81,6	73,6
86,2	75,2	65,2	56,2
83,7	71,2	60,8	52,4
82,1	69,1	58,2	49,4
79	63,4	51,3	42
83,7	71	60,1	50,9
83,5	69,7	58	48,6

81,1	66,7	55,2	46,3
81,4	67,1	55,5	46,5
69	46,6	31,1	19,6
78,3	57,8	41	27,9
78,3	60,5	46,8	36,3
95,4	87,4	76,6	64,8
97	92,6	86,3	78,8
95,9	88,6	79	68,6
97	92,6	86,1	78,5
93,7	82,3	68,2	53,8
94,8	86	75,3	64,3
94,5	84,6	72,5	60
94,5	87,5	79,8	71,9
90,2	78,6	67	56,3
94,3	88,9	83,3	77,8
98,5	96,9	92,5	84,8
98,5	97,3	93,9	87,3
98,6	97,1	93,3	87,2
97,3	94,1	89,5	83,7
92	85,9	74,7	60,9
92,1	85,9	74	59,5
92,6	88	79,3	67,7
97	93,5	87,6	80
96,7	92,3	84,8	75,4
96,7	91,7	83,6	74,4
96,4	90,4	80,6	69,5
96,8	93	86,3	77,2
97	92,8	85,6	76,4
92,9	91,1	88,5	85,1
92,3	88,7	82,5	73,4
91,7	84,3	71,4	56,9
92,1	86,4	75,9	63
92,3	87,3	77,6	64,9
92	86,1	75,3	62,1
92,6	88,2	79,8	68,3
92,2	86,9	76,3	62,6
91,5	83,7	70,1	55,4
91,8	85,7	74	59,8
91,5	84,7	71,8	56,9
91,6	84,5	72	57,7
96,7	91,7	83,8	74,8
96,6	92	84,4	75,6
97	92,9	86,1	77,9
96,2	90,3	81,3	71,5
97,2	93,9	88,3	81,5
96,5	91,1	82,6	73,1
96,1	88,8	78,5	67,7
96,8	92,6	85,7	77,4
96,5	90,9	81,8	71,5
96,6	91,5	83,1	73,4
96,9	92,1	84,2	74,5
96,4	90,6	81,3	70,9

96,9	92,7	85,4	76,4
96,5	91	82	71,4
97,1	92,6	85,3	76,9
96,9	92,8	85,8	77,5
96,2	89,4	79	68,1
96,5	90,9	82,3	72,8
96,7	92,5	85,3	76,9
96,7	92,3	85,8	78,6
91,3	83,6	69,7	54,5
91,7	85,3	73,4	59,2
92,2	87,2	77,9	65,9
92,1	86,5	76,8	64,4
91,4	87,3	82,2	76,6
91,1	86,7	81,4	75,3
90,1	85,2	79,7	73,9
90,2	80,6	65,1	47,3
87,8	68,2	47,6	34,1
90,1	80,5	65,3	48,1
77,7	63	51,4	41,9
76,5	61,7	49,9	40,2
76,2	61,3	49,5	40
90,1	80,5	65	47,3
92,8	89,5	82,8	72,8
92,3	86	74,6	61
78,5	63,5	51	41
73	58,3	47,1	38,1
79,2	65,5	54,2	44,9
80,7	67,8	56,9	47,8
80,7	67,6	56,4	47
80	67,1	56,3	47,3
75	57,5	43,8	33,5
81	68,3	57,5	48,3
82,1	70,8	61,1	52,7
84	73,2	63,3	54,5
82,6	70,7	60,1	51
83,8	72,7	62,7	53,8
71,1	53,3	40	30,2
83,2	71,6	61,1	52
83,1	72,2	62,6	54,2
82,3	70,1	59,2	49,8
83,6	73	63,7	55,5
96,4	91	82,2	72
96,2	90	79,8	68,2
92,6	87,7	79	67,3
93,1	80,3	63,7	46,7
94,4	83,8	69,4	54
92,9	89,1	82,2	72,4
97,1	92,8	85,7	76,3
97,4	93,8	88,9	83
81,3	68,7	57,7	48,4
80,9	68,2	57,4	48,1
84	73	62,7	53,7

77,3	62,2	50	40,3
74,4	55,8	41,6	31,6
75,1	56,2	41,8	31,7
73,3	56,1	43,2	33,7
73,1	53,1	38,4	28,7
78,4	61,3	47,3	36,7
78,9	65,3	54	44,7
79,9	66	54,1	44,3
74,4	58,1	45,5	36
75,9	62,3	51,5	42,6
66,2	47,3	34,3	25,8
68,1	50,9	38,5	29,7
74,6	55,6	41,1	30,9
71,5	55	42,4	32,9
70,2	50,8	36,8	27,5
96,8	92,4	85,1	76,4
96,5	91,6	83,6	74,2
97	93,5	87,6	80,2
91,6	87,7	83	77,6
89	81,8	73,4	64,5
85,2	72,3	58,4	45,2
90,9	86,5	81,3	75,6
91,8	88,2	83,9	78,8
91,2	86,9	81,6	75,5
91,2	86,9	81,8	76
92,7	88,7	81	70,1
96,9	93,1	87,6	80,4
93,3	90,5	85,3	77,7
96,6	92,1	85,3	76,4
91,9	85	72,9	58,5
92,2	86,2	74,5	59,9
92,2	85,5	73,4	58,9
92,3	86,7	76,6	63,5
92,3	87,3	77,8	65
92,5	87,7	78,9	67
96,4	90,3	81,4	71,7
95	86,3	73,6	60,9
96,2	90	80,1	68,8
96,3	90,3	81,6	72
96,6	92,2	85,5	77,8
96,5	90,4	80,8	69,9
92,2	86,5	76	62,7
92,2	86,7	76	62,4
92,3	86,8	76,5	63,6
94,2	82,6	67,1	53,1
96,4	90,8	81,6	71,1
96,6	91,1	82	71,3
95,8	88,4	77,3	65,5
91,5	83,5	69,2	53,4
91,2	82,1	66,5	50,7
91	81,3	65,5	49,8
91,6	84	70,3	54,9

81,3	59,9	44,2	34,8
79,6	56,6	40,9	32,1
78,7	55,7	40,3	31,8
91	84,1	75,2	65,2
84,4	66,6	48,3	33,8
91,4	85,4	77,4	68,2
78,9	56,7	42	33,6
78,9	56,1	41	32,7
78,7	56,1	41,1	32,6
96	89,7	79,6	68
96,2	90,7	81,4	70,2
95,9	89,4	79,4	67,6
96	89,3	79	67,5
95,6	88,8	78,5	66,3
94	83,3	69	54,8
80,9	59,3	43,8	34,6
80,7	58,8	43,2	34,1
81,6	60,4	44,8	35,3
81,1	59,6	43,8	34,3
68,6	42,7	30,6	24,8
79,4	57,1	41,9	33,1
80,5	58,3	42,5	33,3
80,7	58,9	43,4	34,3
80,6	58,9	43,4	34,2
80,9	58,9	43	33,7
65	39,2	28,3	22,8
79,3	56,5	41,2	32,4
92,4	86,9	76,6	63,7
91,7	83,6	69,2	53,6
92,1	85,7	74	60,1
91,9	88,4	84,2	79,2
91,5	88	83,7	78,9
95,5	88	76,7	64
96,5	90,8	81,7	71,7
97	92,2	84,4	75,4
95	86,4	74,1	60,9
96,3	90,1	81	71
96,6	91,5	82,8	72,5
96,5	91,2	82,4	72,1
96,1	89,7	79,5	68,4
80,2	58,2	43	34,1
80,2	58	42,6	33,7
79,6	57,5	42,4	33,6
91,7	84,7	72,3	58,3
92	86,9	76,8	64,1
92,3	87,6	78,8	67,1
97,9	95,5	90,5	83,5
97,6	94,9	89,2	81,6
97,9	95,7	91,4	85,3
94,7	86	73,9	61
96,5	91,4	82,7	72,7
93,5	85,9	77,2	68,4

92,7	88,4	80,3	69
93,4	83,9	70,6	55,2
96	90	81	69,8
92,6	87,7	78,8	66,9
93	89,3	82,4	72,5
96	89,6	79,6	67,3
95,4	88,1	77,5	64,9
94,5	86,8	77	65,5
96,1	90,1	80,8	69
95,4	87,2	74,9	60,6
91,1	82,5	69,3	54,9
91,5	84,6	74,3	62,3
97,5	94,3	89	81,7
90,4	80,2	65,7	50,8
90,7	81	66,7	51,8
86,1	80	74,6	69,4
81,7	71,6	62	52,7
85,5	78,8	72,3	66,1
96,2	90,1	80,4	69
86,7	73,3	58	43,9
94,4	86,1	74,8	62,8
94,9	86,7	75,1	62,7
96,7	91,5	82,9	72,7
96,2	90,7	82	71,8
96,3	91,5	84,5	75,7
97,2	93,5	87	77,7
98	95,9	91,1	83,4
97,9	96,1	92,3	86,1
97,5	94,7	90,8	86
97,5	94,6	90,4	85,3
98	95,7	90,9	83,7
97,8	95,8	91,5	84,7
93,4	83,1	70	56,3
97,9	95,9	91,2	83,8
97,9	96,1	92	85,2
97,9	95,4	90,1	82,2
97,3	94,5	89,5	82,8
96,2	90,7	82,1	72,4
96,8	93	86,8	79
96,1	89,2	78,4	66,9
96,6	92,4	85,4	76,9
96,9	92,4	85,1	76,3
97,1	93	86,3	78,2
96,5	90,8	81,4	70,7
95,9	88,2	76,8	65,2
95,8	88,2	76,9	65,1
96,1	89,3	78,8	67,4
95,6	88,9	78,7	66,9
96,9	92,8	86	77,9
96	89	78,2	66,6
95,9	89,3	79,9	70
97,8	95,6	90,5	82,6

97,8	95,4	90,7	84,2
97,8	95,3	90,4	83,6
96,3	90,6	81,8	72
95,8	89,1	78,7	67,3
95,8	89,2	80,6	71,5
96,3	89,6	79,6	69
95,9	89,3	79,4	68,8
97	93,3	87	78,8
96,6	91,7	84	75
96,7	91,7	83,4	73,7
96,3	90,9	82,5	72,5
96,5	92	84,5	75,3
96,4	91,2	83,2	74,3
96,8	92,1	84,9	76,5
96,1	89,7	79,6	68,5
95,9	89,1	78,8	67,7
96,1	89,3	78,1	65,5
96,9	92,5	85,1	76
96,5	91	82,3	72,6
96,2	90,6	81,7	70,6
93,7	82	66,6	52,2
97,9	95,7	91,3	85,3
97,7	95	89,8	82,8
97,6	94,7	89,4	82,4
96,4	91,3	83,1	73,7
96,8	92,2	84,3	74,8
97	92,7	86,2	78,6
95,9	90,2	83	74,7
96,2	92,7	88,3	83,5
95,7	91,5	86,5	80,9
96,7	93	86,7	78,4
96,8	92,4	85,2	76,6
96,2	90,6	81,6	70,8
96,5	91	82,2	72
96,4	90,7	82,2	72,7
96,3	91,1	82,5	72,1
95,9	88,8	78,1	67,1
95,8	87,8	76,4	64,9
96,6	91,9	84,1	74,6
96,2	90,4	81	70,1
96,6	91,5	83,4	74,1
96,5	91,3	82,6	72,6
95,9	89,4	79,8	69,5
96	89,7	80,7	71,2
96,5	91,3	83,5	74,8
96,5	91,2	83,4	74,8
96,7	91,6	83,1	73
96	90,7	83,5	75,8
96,5	91,3	82,7	72,4
96,4	91	82,3	72,4
96,6	91,9	84,2	75,2
96,8	92,2	85,2	77,3

96	88,6	77,8	66,6
96,4	92,2	85,1	75,9
96,2	90	80,9	70,9
96	92,2	87,3	81,8
97,6	94,8	89,4	82
97,9	96,2	92,4	86,3
97,9	95,3	89,7	81,7
98	95,8	91	83,8
97,8	95,3	90	82,4
97,3	93,1	85,5	76,5
97,9	95,5	90,3	83
97,8	96	92	85,7
97,8	95,3	90,3	83,1
96,2	89,7	80,2	70,1
97	93	86,1	77,4
96,6	91,1	82,7	73,3
77,4	70,3	61,1	50,4
75,4	66,2	54,8	42,3
84	79,8	74,6	67,6
85	81,9	78,7	74,8
80,6	76	70,4	63,3
83,7	78,7	72,1	63,6
86,1	83,1	79,7	75,1
81,2	76,3	70,2	62,3
84	78	69,4	58,4
85,4	81,5	76,4	69,5
88,2	85,4	82	77,3
87,5	84,3	80	74,1
82,3	75,6	66,3	54,6
85,8	81,6	75,6	67,3
80,9	72,2	59,5	44,4
84,7	81	76,7	71
83,8	79,3	73,5	66,1
87	82,6	76,2	66,9
85,4	80,8	74,5	66
88	84,5	79,7	73
84,7	78,9	70,4	59,2
85	79,9	72,6	62,8
85,6	76,7	64,1	50,8
83,6	79,3	73,7	65,8
86	82,4	77,6	70,9
86,3	82,3	76,6	69
83,6	80	75,9	70,5
86	82,1	76,9	69,6
84	79	72,3	63,6
74,2	68,3	61,4	52,9
79,4	75,1	70,3	64,4
80,7	77,4	74,2	70,5
80,3	76,8	73,3	69,1

admixture_EUR	admixture_NAT	admixture_AFR	admixture_EAS	age_conception_mother	
0,938828	0,0343	0,0269	1,00E-05		34
NA	NA	NA	NA	NA	
NA	NA	NA	NA	NA	
0,623636	0,16322	0,207949	0,0052		23
NA	NA	NA	NA	NA	
NA	NA	NA	NA	NA	
0,736355	0,0602	0,201709	0,00178		29
NA	NA	NA	NA	NA	
NA	NA	NA	NA	NA	
0,743428	0,0937	0,152837	0,0101		22
NA	NA	NA	NA	NA	
NA	NA	NA	NA	NA	
0,895507	0,0599	0,0257	0,0189		39
NA	NA	NA	NA	NA	
NA	NA	NA	NA	NA	
0,799524	0,0472	0,153313	1,00E-05		27
NA	NA	NA	NA	NA	
NA	NA	NA	NA	NA	
0,849958	0,111024	0,039	1,00E-05		33
NA	NA	NA	NA	NA	
NA	NA	NA	NA	NA	
0,683753	0,126774	0,154161	0,0353		39
NA	NA	NA	NA	NA	
NA	NA	NA	NA	NA	
0,798053	0,0711	0,128053	0,00278		20
NA	NA	NA	NA	NA	
NA	NA	NA	NA	NA	
0,51011	0,0152	1,00E-05	0,474647		34
NA	NA	NA	NA	NA	
NA	NA	NA	NA	NA	
0,540474	0,10139	0,358127	1,00E-05		27
NA	NA	NA	NA	NA	
NA	NA	NA	NA	NA	
0,490056	0,100642	0,409292	1,00E-05		32
NA	NA	NA	NA	NA	
NA	NA	NA	NA	NA	
0,790048	0,128455	0,0596	0,0219		27
NA	NA	NA	NA	NA	
NA	NA	NA	NA	NA	
0,931042	0,0171	0,0518	1,00E-05		24
NA	NA	NA	NA	NA	
NA	NA	NA	NA	NA	
0,686394	0,0828	0,218852	0,0119		35
NA	NA	NA	NA	NA	
NA	NA	NA	NA	NA	
0,488333	0,171074	0,319626	0,021		35
NA	NA	NA	NA	NA	
NA	NA	NA	NA	NA	
0,937222	0,0255	0,0372	1,00E-05		26
NA	NA	NA	NA	NA	

NA	NA	NA	NA	NA	
0,854523	0,109019	0,0364	1,00E-05		35
NA	NA	NA	NA	NA	
NA	NA	NA	NA	NA	
NA	NA	NA	NA	NA	
NA	NA	NA	NA	NA	
0,645766	0,163499	0,17832	0,0124	NA	
0,59109	0,059	0,345644	0,00429		22
NA	NA	NA	NA	NA	
NA	NA	NA	NA	NA	
0,770145	0,0738	0,156014	1,00E-05		28
NA	NA	NA	NA	NA	
NA	NA	NA	NA	NA	
0,37303	0,0984	0,528518	1,00E-05		36
NA	NA	NA	NA	NA	
NA	NA	NA	NA	NA	
0,612125	0,133413	0,253585	0,000877		38
NA	NA	NA	NA	NA	
NA	NA	NA	NA	NA	
0,539171	0,078	0,381707	0,00114		27
NA	NA	NA	NA	NA	
NA	NA	NA	NA	NA	
0,773343	0,0119	0,206777	0,00797		30
NA	NA	NA	NA	NA	
NA	NA	NA	NA	NA	
0,822952	0,132002	0,0413	0,0037	NA	
NA	NA	NA	NA	NA	
NA	NA	NA	NA	NA	
0,916389	0,0392	0,0444	1,00E-05		28
NA	NA	NA	NA	NA	
NA	NA	NA	NA	NA	
0,770373	0,171538	0,0581	1,00E-05		36
NA	NA	NA	NA	NA	
NA	NA	NA	NA	NA	
0,709106	0,0656	0,22529	1,00E-05		35
NA	NA	NA	NA	NA	
NA	NA	NA	NA	NA	
0,623106	0,19759	0,174499	0,00481		23
NA	NA	NA	NA	NA	
NA	NA	NA	NA	NA	
0,742655	0,0775	0,17036	0,00952		37
NA	NA	NA	NA	NA	
NA	NA	NA	NA	NA	
0,902895	0,0486	0,0485	1,00E-05		35
NA	NA	NA	NA	NA	
NA	NA	NA	NA	NA	
0,90784	0,051	0,0359	0,00531		28
NA	NA	NA	NA	NA	
NA	NA	NA	NA	NA	
0,764197	0,0486	0,152114	0,0351		20
NA	NA	NA	NA	NA	
NA	NA	NA	NA	NA	

0,991331	0,00861	1,00E-05	4,80E-05		33
NA	NA	NA	NA	NA	
NA	NA	NA	NA	NA	
0,24389	0,028	0,726038	0,0021		35
NA	NA	NA	NA	NA	
NA	NA	NA	NA	NA	
0,705564	0,0643	0,21694	0,0132		35
NA	NA	NA	NA	NA	
NA	NA	NA	NA	NA	
0,799261	0,0665	0,113676	0,0206		36
NA	NA	NA	NA	NA	
NA	NA	NA	NA	NA	
0,824292	0,0594	0,116265	1,00E-05		30
NA	NA	NA	NA	NA	
NA	NA	NA	NA	NA	
0,734384	0,0585	0,193423	0,0137		35
NA	NA	NA	NA	NA	
NA	NA	NA	NA	NA	
0,676229	0,153676	0,165073	0,00502		29
NA	NA	NA	NA	NA	
NA	NA	NA	NA	NA	
0,735733	0,164581	0,0997	1,00E-05		36
NA	NA	NA	NA	NA	
NA	NA	NA	NA	NA	
0,378112	0,0872	0,534655	1,00E-05		28
NA	NA	NA	NA	NA	
NA	NA	NA	NA	NA	
0,807535	0,0609	0,117607	0,014		31
NA	NA	NA	NA	NA	
NA	NA	NA	NA	NA	
0,841542	0,0312	0,1272	1,00E-05	NA	
NA	NA	NA	NA	NA	
NA	NA	NA	NA	NA	
0,753667	0,0735	0,1625	0,0103		20
NA	NA	NA	NA	NA	
NA	NA	NA	NA	NA	
0,55411	0,111646	0,322389	0,0119		20
NA	NA	NA	NA	NA	
NA	NA	NA	NA	NA	
0,604112	0,17716	0,212628	0,0061		29
NA	NA	NA	NA	NA	
NA	NA	NA	NA	NA	
0,672	0,102102	0,207238	0,0187		27
NA	NA	NA	NA	NA	
NA	NA	NA	NA	NA	
0,895953	0,0336	0,0695	0,001		32
NA	NA	NA	NA	NA	
NA	NA	NA	NA	NA	
0,786542	0,134655	0,0667	0,0121	NA	
NA	NA	NA	NA	NA	
NA	NA	NA	NA	NA	
0,92666	0,0381	0,0352	1,00E-05	NA	

NA	NA	NA	NA	NA	
NA	NA	NA	NA	NA	
0,731738	0,0601	0,184238	0,0239	NA	
NA	NA	NA	NA	NA	
NA	NA	NA	NA	NA	
NA	NA	NA	NA	NA	
NA	NA	NA	NA	NA	
0,936293	0,0426	1,00E-05	0,0211	NA	
0,991544	0,00745	1,00E-05	0,001		29
NA	NA	NA	NA	NA	
NA	NA	NA	NA	NA	
0,969398	0,0306	1,00E-05	1,00E-05		36
NA	NA	NA	NA	NA	
NA	NA	NA	NA	NA	
NA	NA	NA	NA	NA	
0,828644	0,0429	0,12844	1,00E-05	NA	
NA	NA	NA	NA	NA	
NA	NA	NA	NA	NA	
0,762089	0,0542	0,164008	0,0197	NA	
0,99997	1,00E-05	1,00E-05	1,00E-05	NA	
NA	NA	NA	NA	NA	
NA	NA	NA	NA	NA	
NA	NA	NA	NA	NA	
NA	NA	NA	NA	NA	
0,45252	0,165421	0,381387	0,000672	NA	
NA	NA	NA	NA	NA	
NA	NA	NA	NA	NA	
NA	NA	NA	NA	NA	
NA	NA	NA	NA	NA	
0,448735	0,0973	0,443393	0,0106	NA	
NA	NA	NA	NA	NA	
0,566715	0,0802	0,347768	0,00529	NA	
NA	NA	NA	NA	NA	
NA	NA	NA	NA	NA	
NA	NA	NA	NA	NA	
0,482152	0,125727	0,333118	0,059	NA	
NA	NA	NA	NA	NA	
NA	NA	NA	NA	NA	
NA	NA	NA	NA	NA	
0,664526	0,126167	0,181575	0,0277	NA	
NA	NA	NA	NA	NA	
NA	NA	NA	NA	NA	
NA	NA	NA	NA	NA	
NA	NA	NA	NA	NA	
0,729418	0,096	0,17453	1,00E-05	NA	
NA	NA	NA	NA	NA	
NA	NA	NA	NA	NA	
0,618104	0,197481	0,167315	0,0171	NA	
NA	NA	NA	NA	NA	
NA	NA	NA	NA	NA	
0,776823	0,110489	0,104451	0,00824	NA	
NA	NA	NA	NA	NA	

NA	NA	NA	NA	NA	
0,788788	0,0977	0,106919	0,00659	NA	
NA	NA	NA	NA	NA	
NA	NA	NA	NA	NA	
0,936399	0,0109	0,00518	0,0475	NA	
NA	NA	NA	NA	NA	
NA	NA	NA	NA	NA	
0,701823	0,103407	0,179323	0,0154	NA	
NA	NA	NA	NA	NA	
NA	NA	NA	NA	NA	
0,547166	0,105247	0,346099	0,00149	NA	
NA	NA	NA	NA	NA	
NA	NA	NA	NA	NA	
1,00E-05	1,00E-05	1,00E-05	0,99997	NA	
0,87906	0,0465	0,0602	0,0143	NA	
NA	NA	NA	NA	NA	
NA	NA	NA	NA	NA	
0,685757	0,107792	0,206441	1,00E-05	NA	
NA	NA	NA	NA	NA	
NA	NA	NA	NA	NA	
0,593166	0,099	0,307826	1,00E-05	NA	
NA	NA	NA	NA	NA	
0,658389	0,0648	0,276823	1,00E-05	NA	
NA	NA	NA	NA	NA	
NA	NA	NA	NA	NA	
0,172735	0,0228	1,00E-05	0,804429	NA	
NA	NA	NA	NA	NA	
NA	NA	NA	NA	NA	
0,604525	0,149437	0,22912	0,0169	NA	
NA	NA	NA	NA	NA	
0,762352	0,0701	0,167523	1,00E-05	NA	
NA	NA	NA	NA	NA	
NA	NA	NA	NA	NA	
0,886895	0,0687	0,0444	1,00E-05	NA	
NA	NA	NA	NA	NA	
NA	NA	NA	NA	NA	
NA	NA	NA	NA	NA	
NA	NA	NA	NA	NA	
0,743389	0,13131	0,12529	1,00E-05	NA	
NA	NA	NA	NA	NA	
NA	NA	NA	NA	NA	
0,84362	0,0474	0,108471	0,000522		34
NA	NA	NA	NA	NA	
NA	NA	NA	NA	NA	
0,846574	0,0767	0,0767	1,00E-05		36
NA	NA	NA	NA	NA	
NA	NA	NA	NA	NA	
0,629645	0,105601	0,264744	1,00E-05		29
NA	NA	NA	NA	NA	
NA	NA	NA	NA	NA	
0,872504	0,0802	0,0473	1,00E-05		31
NA	NA	NA	NA	NA	

NA	NA	NA	NA	NA	
0,662509	0,101751	0,195205	0,0405		26
NA	NA	NA	NA	NA	
NA	NA	NA	NA	NA	
0,870465	0,0511	0,0641	0,0143		32
NA	NA	NA	NA	NA	
NA	NA	NA	NA	NA	
0,683576	0,0657	0,234923	0,0158		34
NA	NA	NA	NA	NA	
NA	NA	NA	NA	NA	
0,99997	1,00E-05	1,00E-05	1,00E-05		34
NA	NA	NA	NA	NA	
NA	NA	NA	NA	NA	
0,909019	0,0561	0,0216	0,0133		28
NA	NA	NA	NA	NA	
NA	NA	NA	NA	NA	
0,892991	0,0496	0,0574	1,00E-05		25
NA	NA	NA	NA	NA	
NA	NA	NA	NA	NA	
0,642235	0,268511	0,0825	0,00671		21
NA	NA	NA	NA	NA	
NA	NA	NA	NA	NA	
0,866853	0,043	0,0851	0,00513		31
NA	NA	NA	NA	NA	
NA	NA	NA	NA	NA	
0,735193	0,0851	0,176918	0,00275		40
NA	NA	NA	NA	NA	
NA	NA	NA	NA	NA	
0,858229	0,0667	0,0751	1,00E-05		32
NA	NA	NA	NA	NA	
NA	NA	NA	NA	NA	
0,643383	0,122299	0,234308	1,00E-05		27
NA	NA	NA	NA	NA	
NA	NA	NA	NA	NA	
0,569403	0,096	0,334623	1,00E-05		20
NA	NA	NA	NA	NA	
NA	NA	NA	NA	NA	
0,995655	0,00432	1,00E-05	1,00E-05		29
NA	NA	NA	NA	NA	
NA	NA	NA	NA	NA	
0,845171	0,0365	0,118352	1,00E-05		29
NA	NA	NA	NA	NA	
NA	NA	NA	NA	NA	
0,667293	0,113371	0,198344	0,021		33
NA	NA	NA	NA	NA	
NA	NA	NA	NA	NA	
0,737087	0,111057	0,134818	0,017		37
NA	NA	NA	NA	NA	
NA	NA	NA	NA	NA	
0,808686	0,0905	0,100788	1,00E-05		35
NA	NA	NA	NA	NA	
NA	NA	NA	NA	NA	

0,827598	0,129314	0,0365	0,00659		33
NA	NA	NA	NA	NA	
0,690944	0,0234	0,267726	0,018		35
NA	NA	NA	NA	NA	
NA	NA	NA	NA	NA	
0,330052	0,0464	0,62351	1,00E-05		23
NA	NA	NA	NA	NA	
NA	NA	NA	NA	NA	
0,72873	0,108902	0,155112	0,00726		28
NA	NA	NA	NA	NA	
NA	NA	NA	NA	NA	
0,929381	0,0342	0,0315	0,00488		28
NA	NA	NA	NA	NA	
NA	NA	NA	NA	NA	
0,479651	0,168364	0,3508	0,00119		36
NA	NA	NA	NA	NA	
NA	NA	NA	NA	NA	
0,772995	0,116824	0,106035	0,00415		38
NA	NA	NA	NA	NA	
NA	NA	NA	NA	NA	
0,916792	0,0457	0,0375	1,00E-05		28
NA	NA	NA	NA	NA	
NA	NA	NA	NA	NA	
0,711191	0,100611	0,183124	0,00507	NA	
NA	NA	NA	NA	NA	
NA	NA	NA	NA	NA	
0,368925	0,0567	0,571064	0,0033		27
NA	NA	NA	NA	NA	
NA	NA	NA	NA	NA	
0,519634	0,119942	0,343186	0,0172		37
NA	NA	NA	NA	NA	
NA	NA	NA	NA	NA	
0,742381	0,166892	0,0907	1,00E-05		27
NA	NA	NA	NA	NA	
0,622857	0,0673	0,0556	0,254234		34
NA	NA	NA	NA	NA	
NA	NA	NA	NA	NA	
0,90453	0,0386	0,0518	0,00503		33
NA	NA	NA	NA	NA	
NA	NA	NA	NA	NA	
0,886145	0,03	0,0469	0,0369		28
NA	NA	NA	NA	NA	
NA	NA	NA	NA	NA	
0,938046	0,00459	0,0515	0,00585		32
NA	NA	NA	NA	NA	
NA	NA	NA	NA	NA	
0,846046	0,0786	0,0753	1,00E-05		31
NA	NA	NA	NA	NA	
NA	NA	NA	NA	NA	
0,796611	0,0337	0,169658	1,00E-05		26
NA	NA	NA	NA	NA	
NA	NA	NA	NA	NA	

0,800959	0,0289	0,170179	1,00E-05		40
NA	NA	NA	NA	NA	
NA	NA	NA	NA	NA	
0,528517	0,33427	0,131464	0,00575		24
NA	NA	NA	NA	NA	
NA	NA	NA	NA	NA	
0,630818	0,109564	0,259607	1,00E-05		33
NA	NA	NA	NA	NA	
NA	NA	NA	NA	NA	
0,847732	0,0461	0,105395	0,000754		32
NA	NA	NA	NA	NA	
NA	NA	NA	NA	NA	
0,762969	0,110454	0,122103	0,00447		38
NA	NA	NA	NA	NA	
NA	NA	NA	NA	NA	
0,776121	0,0628	0,161035	1,00E-05		36
NA	NA	NA	NA	NA	
NA	NA	NA	NA	NA	
0,75275	0,104939	0,133767	0,00854		35
NA	NA	NA	NA	NA	
NA	NA	NA	NA	NA	
0,96971	0,0303	1,00E-05	1,00E-05		30
NA	NA	NA	NA	NA	
NA	NA	NA	NA	NA	
0,823554	0,0385	0,12432	0,0136		32
NA	NA	NA	NA	NA	
NA	NA	NA	NA	NA	
0,763438	0,0732	0,155845	0,00751		25
NA	NA	NA	NA	NA	
NA	NA	NA	NA	NA	
0,819806	0,067	0,113208	1,00E-05		17
NA	NA	NA	NA	NA	
NA	NA	NA	NA	NA	
0,990412	1,00E-05	1,00E-05	0,00957		36
NA	NA	NA	NA	NA	
NA	NA	NA	NA	NA	
0,680123	0,236537	0,0647	0,0186		36
NA	NA	NA	NA	NA	
NA	NA	NA	NA	NA	
0,744046	0,147732	0,106832	0,00139		21
NA	NA	NA	NA	NA	
NA	NA	NA	NA	NA	
0,717909	0,100135	0,178043	0,00391		34
NA	NA	NA	NA	NA	
NA	NA	NA	NA	NA	
0,817428	0,105452	0,0771	1,00E-05		44
NA	NA	NA	NA	NA	
0,777304	0,0896	0,133046	1,00E-05		24
NA	NA	NA	NA	NA	
NA	NA	NA	NA	NA	
0,153993	0,0632	0,77526	0,0076		28
NA	NA	NA	NA	NA	

NA	NA	NA	NA	NA	
0,837693	0,0361	0,126176	1,00E-05		36
NA	NA	NA	NA	NA	
NA	NA	NA	NA	NA	
0,663404	0,1001	0,233698	0,0028		23
NA	NA	NA	NA	NA	
NA	NA	NA	NA	NA	
0,620373	0,118286	0,236615	0,0247		43
NA	NA	NA	NA	NA	
NA	NA	NA	NA	NA	
0,735628	0,0989	0,165431	1,00E-05		32
NA	NA	NA	NA	NA	
NA	NA	NA	NA	NA	
0,61605	0,125376	0,251879	0,0067		33
NA	NA	NA	NA	NA	
NA	NA	NA	NA	NA	
0,915977	0,0607	0,0234	1,00E-05	NA	
0,459141	0,361406	0,147384	0,0321	NA	
NA	NA	NA	NA	NA	
NA	NA	NA	NA	NA	
0,607381	0,063	0,329588	1,00E-05	NA	
0,865615	0,112078	0,0173	0,00501	NA	
1,00E-05	1,10E-05	1,00E-05	0,999969	NA	
NA	NA	NA	NA	NA	
NA	NA	NA	NA	NA	
0,359016	0,157684	0,476156	0,00714	NA	
NA	NA	NA	NA	NA	
NA	NA	NA	NA	NA	
NA	NA	NA	NA	NA	
0,744536	0,127443	0,125443	0,00258		35
NA	NA	NA	NA	NA	
0,732218	0,140314	0,110424	0,017		24
NA	NA	NA	NA	NA	
NA	NA	NA	NA	NA	
0,891743	0,0579	0,0148	0,0356		28
NA	NA	NA	NA	NA	
NA	NA	NA	NA	NA	
NA	NA	NA	NA	NA	
0,961492	0,035	0,00347	1,00E-05		31
NA	NA	NA	NA	NA	
0,59145	0,0861	0,322394	1,00E-05		22
NA	NA	NA	NA	NA	
NA	NA	NA	NA	NA	
0,890911	0,0746	0,0345	1,00E-05		39
NA	NA	NA	NA	NA	
NA	NA	NA	NA	NA	
0,780743	0,0671	0,152176	1,00E-05		36
NA	NA	NA	NA	NA	
NA	NA	NA	NA	NA	
NA	NA	NA	NA	NA	
0,908811	0,0541	0,0371	1,00E-05		30
0,807698	0,0603	0,12108	0,0109		27

NA	NA	NA	NA	NA	
NA	NA	NA	NA	NA	
0,712161	0,183659	0,104171	1,00E-05		32
NA	NA	NA	NA	NA	
0,547523	0,191151	0,237822	0,0235		27
NA	NA	NA	NA	NA	
0,602905	0,186798	0,171194	0,0391		26
NA	NA	NA	NA	NA	
NA	NA	NA	NA	NA	
0,840677	0,00772	0,14257	0,00904		31
NA	NA	NA	NA	NA	
NA	NA	NA	NA	NA	
0,95851	0,014	0,00299	0,0245		34
NA	NA	NA	NA	NA	
NA	NA	NA	NA	NA	
NA	NA	NA	NA	NA	
NA	NA	NA	NA	NA	
0,810556	0,0912	0,0667	0,0316		29
NA	NA	NA	NA	NA	
0,696612	0,0694	0,233955	1,00E-05		33
NA	NA	NA	NA	NA	
NA	NA	NA	NA	NA	
0,667925	0,183831	0,148234	1,00E-05		34
NA	NA	NA	NA	NA	
NA	NA	NA	NA	NA	
0,891314	0,0532	0,0424	0,013		37
NA	NA	NA	NA	NA	
0,637253	0,178735	0,181606	0,00241		22
NA	NA	NA	NA	NA	
NA	NA	NA	NA	NA	
NA	NA	NA	NA		25
NA	NA	NA	NA	NA	
NA	NA	NA	NA	NA	
0,728421	0,0819	0,189642	1,00E-05		30
NA	NA	NA	NA	NA	
NA	NA	NA	NA	NA	
NA	NA	NA	NA	NA	
0,853716	1,00E-05	1,00E-05	0,146264		27
NA	NA	NA	NA	NA	
NA	NA	NA	NA	NA	
0,908588	0,0286	0,0602	0,00254		37
NA	NA	NA	NA	NA	
NA	NA	NA	NA	NA	
0,791036	0,0453	0,163667	1,00E-05		39
NA	NA	NA	NA	NA	
NA	NA	NA	NA	NA	
NA	NA	NA	NA	NA	
NA	NA	NA	NA	NA	
NA	NA	NA	NA	NA	
0,667845	0,0678	0,249878	0,0144	NA	
NA	NA	NA	NA	NA	
NA	NA	NA	NA	NA	

0,881739	0,0659	0,0481	0,00428		29
NA	NA	NA	NA	NA	
NA	NA	NA	NA	NA	
0,848671	0,089	0,0336	0,0288		19
NA	NA	NA	NA	NA	
NA	NA	NA	NA	NA	
0,605776	0,0841	0,292399	0,0177		26
NA	NA	NA	NA	NA	
NA	NA	NA	NA	NA	
0,487964	1,50E-05	1,00E-05	0,512011		29
NA	NA	NA	NA	NA	
NA	NA	NA	NA	NA	
0,76326	0,0821	0,154595	1,00E-05		34
NA	NA	NA	NA	NA	
NA	NA	NA	NA	NA	
0,775078	0,122781	0,089	0,0132		33
NA	NA	NA	NA	NA	
0,825104	0,0456	0,100257	0,029		29
NA	NA	NA	NA	NA	
NA	NA	NA	NA	NA	
0,651392	0,170089	0,178509	1,00E-05		32
NA	NA	NA	NA	NA	
0,707543	0,112503	0,173994	0,00596		24
NA	NA	NA	NA	NA	
0,857786	0,0202	0,087	0,0351		25
NA	NA	NA	NA	NA	
NA	NA	NA	NA	NA	
NA	NA	NA	NA		39
0,822147	0,0985	0,0749	0,00449		18
NA	NA	NA	NA	NA	
NA	NA	NA	NA	NA	
0,716102	0,15922	0,109255	0,0154		31
NA	NA	NA	NA	NA	
0,717845	0,138194	0,123005	0,021		31
NA	NA	NA	NA	NA	
NA	NA	NA	NA	NA	
0,564987	0,0836	0,350314	0,00112		28
NA	NA	NA	NA	NA	
NA	NA	NA	NA	NA	
0,960502	0,026	0,0135	1,00E-05		29
NA	NA	NA	NA	NA	
NA	NA	NA	NA	NA	
0,369936	0,103285	0,52627	0,000509		20
NA	NA	NA	NA	NA	
0,93386	0,0566	0,00949	1,00E-05		28
NA	NA	NA	NA	NA	
0,674542	0,0778	0,247633	1,00E-05		39
NA	NA	NA	NA	NA	
0,737492	0,0583	0,192693	0,0116		28
NA	NA	NA	NA	NA	
0,918708	0,0598	0,016	0,00549		34
NA	NA	NA	NA	NA	

0,617968	0,0634	0,30381	0,0149		23
NA	NA	NA	NA	NA	
NA	NA	NA	NA	NA	
0,538508	0,17505	0,280421	0,00602	NA	
0,704296	0,105681	0,0894	0,100604	NA	
NA	NA	NA	NA	NA	
0,673682	0,264889	0,0614	1,00E-05		36
NA	NA	NA	NA	NA	
0,716536	0,12255	0,15205	0,00886		36
NA	NA	NA	NA	NA	
NA	NA	NA	NA	NA	
0,70184	0,12814	0,151305	0,0187		32
NA	NA	NA	NA	NA	
NA	NA	NA	NA	NA	
0,540304	0,0502	0,409445	1,00E-05		34
NA	NA	NA	NA	NA	
0,914486	0,0579	0,0177	0,00988		27
NA	NA	NA	NA	NA	
0,919309	0,0336	0,0262	0,0208		36
NA	NA	NA	NA	NA	
NA	NA	NA	NA	NA	
0,852839	0,0666	0,0791	0,00142	NA	
NA	NA	NA	NA	NA	
NA	NA	NA	NA	NA	
0,644645	0,142106	0,164793	0,0485		24
0,956801	0,0231	0,011	0,00906		38
NA	NA	NA	NA	NA	
NA	NA	NA	NA	NA	
0,748159	0,126219	0,107074	0,0185		26
NA	NA	NA	NA	NA	
NA	NA	NA	NA	NA	
0,721725	0,0403	0,229772	0,00817		32
NA	NA	NA	NA	NA	
0,98042	0,0196	1,00E-05	1,00E-05		32
NA	NA	NA	NA	NA	
NA	NA	NA	NA	NA	
NA	NA	NA	NA	NA	
0,925972	0,0545	0,0195	1,00E-05		35
0,709719	0,170841	0,110501	0,00894		29
NA	NA	NA	NA	NA	
NA	NA	NA	NA	NA	
0,624386	0,0745	0,281397	0,0197		35
NA	NA	NA	NA	NA	
NA	NA	NA	NA	NA	
1,00E-05	1,70E-05	1,00E-05	0,999963		32
NA	NA	NA	NA	NA	
0,870311	0,051	0,0787	1,00E-05	NA	
0,911439	0,0483	0,0402	1,00E-05		30
NA	NA	NA	NA	NA	
0,582465	0,0739	0,338908	0,00477		32
NA	NA	NA	NA	NA	
NA	NA	NA	NA	NA	

0,874103	0,0647	0,0612	1,00E-05		36
NA	NA	NA	NA	NA	
NA	NA	NA	NA	NA	
0,942243	0,0563	1,00E-05	0,00145	NA	
NA	NA	NA	NA	NA	
0,616976	0,219027	0,163987	1,00E-05		33
NA	NA	NA	NA	NA	
NA	NA	NA	NA	NA	
0,741224	0,0381	0,209835	0,0108		38
NA	NA	NA	NA	NA	
NA	NA	NA	NA	NA	
0,818413	0,0751	0,104506	0,00195		28
NA	NA	NA	NA	NA	
NA	NA	NA	NA	NA	
1,00E-05	1,60E-05	1,00E-05	0,999964		31
NA	NA	NA	NA	NA	
NA	NA	NA	NA	NA	
0,822182	0,0905	0,0873	1,00E-05		33
NA	NA	NA	NA	NA	
NA	NA	NA	NA	NA	
0,968408	0,0192	1,00E-05	0,0124		33
NA	NA	NA	NA	NA	
NA	NA	NA	NA	NA	
0,654483	0,117143	0,212418	0,016		29
NA	NA	NA	NA	NA	
NA	NA	NA	NA	NA	
0,56273	0,143535	0,284372	0,00936		33
NA	NA	NA	NA	NA	
NA	NA	NA	NA	NA	
0,75321	0,0994	0,135236	0,0122		29
NA	NA	NA	NA	NA	
NA	NA	NA	NA	NA	
0,945962	0,0154	0,0246	0,014		31
NA	NA	NA	NA	NA	
NA	NA	NA	NA	NA	
0,7373	0,0949	0,157705	0,0101		30
NA	NA	NA	NA	NA	
NA	NA	NA	NA	NA	
0,332456	0,276252	0,126818	0,264474	NA	
NA	NA	NA	NA	NA	
0,659366	0,159867	0,180245	0,000521		20
0,781697	0,0482	0,170113	1,00E-05		21
NA	NA	NA	NA	NA	
NA	NA	NA	NA	NA	
0,769973	0,0734	0,156665	1,00E-05		26
NA	NA	NA	NA	NA	
NA	NA	NA	NA	NA	
0,514591	0,485129	1,00E-05	0,00027		20
NA	NA	NA	NA	NA	
NA	NA	NA	NA	NA	
0,99997	1,00E-05	1,00E-05	1,00E-05		32
NA	NA	NA	NA	NA	

NA	NA	NA	NA	NA	
0,726522	0,109221	0,146811	0,0174		27
NA	NA	NA	NA	NA	
NA	NA	NA	NA	NA	
0,696899	0,128327	0,174763	1,00E-05		37
NA	NA	NA	NA	NA	
NA	NA	NA	NA	NA	
0,693529	0,160888	0,145573	1,00E-05		23
NA	NA	NA	NA	NA	
NA	NA	NA	NA	NA	
0,77225	0,0831	0,144648	1,00E-05		31
NA	NA	NA	NA	NA	
NA	NA	NA	NA	NA	
0,99997	1,00E-05	1,00E-05	1,00E-05		33
NA	NA	NA	NA	NA	
NA	NA	NA	NA	NA	
NA	NA	NA	NA	NA	
NA	NA	NA	NA	NA	
0,906761	0,0832	0,00999	1,00E-05		29
0,66443	0,119494	0,216067	1,00E-05		24
NA	NA	NA	NA	NA	
NA	NA	NA	NA	NA	
0,893556	0,0287	0,0778	1,00E-05		34
NA	NA	NA	NA	NA	
NA	NA	NA	NA	NA	
0,835094	0,0614	0,0901	0,0134		36
NA	NA	NA	NA	NA	
NA	NA	NA	NA	NA	
0,797258	0,0656	0,132331	0,0048		27
NA	NA	NA	NA	NA	
NA	NA	NA	NA	NA	
0,473066	1,00E-05	1,00E-05	0,526914		33
NA	NA	NA	NA	NA	
NA	NA	NA	NA	NA	
0,406456	0,307608	0,034	0,251975		29
NA	NA	NA	NA	NA	
NA	NA	NA	NA	NA	
0,68809	0,146814	0,165086	1,00E-05		27
NA	NA	NA	NA	NA	
NA	NA	NA	NA	NA	
0,854381	0,0553	0,0903	1,00E-05		28
NA	NA	NA	NA	NA	
NA	NA	NA	NA	NA	
0,92348	0,0372	0,0393	1,00E-05		34
NA	NA	NA	NA	NA	
NA	NA	NA	NA	NA	
0,554211	0,167123	0,0562	0,222469		35
NA	NA	NA	NA	NA	
NA	NA	NA	NA	NA	
0,692104	0,126427	0,181044	0,000425		34
NA	NA	NA	NA	NA	
NA	NA	NA	NA	NA	

0,751058	0,107961	0,134991	0,00599		24
NA	NA	NA	NA	NA	
NA	NA	NA	NA	NA	
0,467215	0,265407	0,23666	0,0307		20
NA	NA	NA	NA	NA	
NA	NA	NA	NA	NA	
0,902802	0,031	0,0586	0,00753		32
NA	NA	NA	NA	NA	
NA	NA	NA	NA	NA	
0,778784	0,0624	0,022	0,136748		29
NA	NA	NA	NA	NA	
NA	NA	NA	NA	NA	
0,739265	0,132393	0,0881	0,0402		25
NA	NA	NA	NA	NA	
NA	NA	NA	NA	NA	
0,973415	0,0133	0,0133	1,00E-05		36
NA	NA	NA	NA	NA	
NA	NA	NA	NA	NA	
0,906855	0,0316	0,0555	0,00609		27
NA	NA	NA	NA	NA	
NA	NA	NA	NA	NA	
0,804352	0,0474	0,1482	1,00E-05		30
NA	NA	NA	NA	NA	
NA	NA	NA	NA	NA	
0,829937	0,0918	0,0765	0,00178		41
NA	NA	NA	NA	NA	
NA	NA	NA	NA	NA	
0,954759	0,0419	1,00E-05	0,00335		23
NA	NA	NA	NA	NA	
NA	NA	NA	NA	NA	
0,901825	0,0599	0,0346	0,00368		30
NA	NA	NA	NA	NA	
NA	NA	NA	NA	NA	
NA	NA	NA	NA	NA	
0,965232	0,0292	1,00E-05	0,00552		30
NA	NA	NA	NA	NA	
NA	NA	NA	NA	NA	
0,775882	0,056	0,129061	0,039		37
NA	NA	NA	NA	NA	
NA	NA	NA	NA	NA	
0,744	0,0629	0,169838	0,0232		33
NA	NA	NA	NA	NA	
NA	NA	NA	NA	NA	
0,965816	0,0117	0,0177	0,00472		33
NA	NA	NA	NA	NA	
NA	NA	NA	NA	NA	
0,62257	0,0277	0,0843	0,265398		30
NA	NA	NA	NA	NA	
NA	NA	NA	NA	NA	
0,99997	1,00E-05	1,00E-05	1,00E-05		40
NA	NA	NA	NA	NA	
NA	NA	NA	NA	NA	

0,722243	0,13063	0,136222	0,0109		30
NA	NA	NA	NA	NA	
NA	NA	NA	NA	NA	
0,664293	0,082	0,242118	0,0116		25
NA	NA	NA	NA	NA	
NA	NA	NA	NA	NA	
0,775541	0,168697	0,0456	0,0101		40
NA	NA	NA	NA	NA	
NA	NA	NA	NA	NA	
0,651684	0,106182	0,230048	0,0121		31
NA	NA	NA	NA	NA	
NA	NA	NA	NA	NA	
0,844055	0,0183	0,127696	0,00997		29
NA	NA	NA	NA	NA	
NA	NA	NA	NA	NA	
0,664645	0,0895	0,213157	0,0327		38
0,766677	0,191038	0,0316	0,0107		32
NA	NA	NA	NA	NA	
NA	NA	NA	NA	NA	
0,937358	0,0202	0,0151	0,0273		30
NA	NA	NA	NA	NA	
NA	NA	NA	NA	NA	
0,821555	0,0522	0,109886	0,0163		27
NA	NA	NA	NA	NA	
NA	NA	NA	NA	NA	
0,482646	1,30E-05	1,00E-05	0,517331		37
NA	NA	NA	NA	NA	
0,99997	1,00E-05	1,00E-05	1,00E-05		34
NA	NA	NA	NA	NA	
NA	NA	NA	NA	NA	
0,51122	0,116908	0,356328	0,0155		24
NA	NA	NA	NA	NA	
NA	NA	NA	NA	NA	
0,905045	0,0698	0,0251	1,00E-05		21
NA	NA	NA	NA	NA	
NA	NA	NA	NA	NA	
0,272229	0,0656	0,6622	1,00E-05		29
NA	NA	NA	NA	NA	
NA	NA	NA	NA	NA	
0,508276	0,152298	0,33525	0,00418		29
NA	NA	NA	NA	NA	
NA	NA	NA	NA	NA	
0,592619	0,0818	0,296604	0,029		35
NA	NA	NA	NA	NA	
NA	NA	NA	NA	NA	
0,784476	0,0532	0,158398	0,00391		27
NA	NA	NA	NA	NA	
NA	NA	NA	NA	NA	
0,764176	0,034	0,201802	1,00E-05		28
NA	NA	NA	NA	NA	
NA	NA	NA	NA	NA	
0,875807	0,0358	0,0884	1,00E-05		28

NA	NA	NA	NA	NA	
NA	NA	NA	NA	NA	
0,816412	0,122812	0,0461	0,0146		25
NA	NA	NA	NA	NA	
NA	NA	NA	NA	NA	
0,99997	1,00E-05	1,00E-05	1,00E-05		30
NA	NA	NA	NA	NA	
NA	NA	NA	NA	NA	
0,811046	0,0567	0,110272	0,022		29
NA	NA	NA	NA	NA	
NA	NA	NA	NA	NA	
0,924159	0,068	0,00779	1,00E-05		33
NA	NA	NA	NA	NA	
NA	NA	NA	NA	NA	
0,51544	0,0823	0,396514	0,00577		18
NA	NA	NA	NA	NA	
NA	NA	NA	NA	NA	
0,89288	0,0233	0,0838	1,00E-05		40
NA	NA	NA	NA	NA	
NA	NA	NA	NA	NA	
0,806568	0,115769	0,0777	1,00E-05		33
NA	NA	NA	NA	NA	
NA	NA	NA	NA	NA	
0,848744	0,0466	0,0888	0,0159		26
NA	NA	NA	NA	NA	
NA	NA	NA	NA	NA	
0,902251	0,0386	0,0414	0,0177		23
NA	NA	NA	NA	NA	
NA	NA	NA	NA	NA	
0,48268	0,0927	0,161477	0,263187		21
NA	NA	NA	NA	NA	
NA	NA	NA	NA	NA	
0,878466	0,104679	0,0164	0,000431		35
NA	NA	NA	NA	NA	
NA	NA	NA	NA	NA	
0,735373	0,143822	0,105843	0,015		26
NA	NA	NA	NA	NA	
NA	NA	NA	NA	NA	
0,902691	0,0409	0,0483	0,00806		31
NA	NA	NA	NA	NA	
NA	NA	NA	NA	NA	
0,777969	0,0837	0,131287	0,00703		28
NA	NA	NA	NA	NA	
NA	NA	NA	NA	NA	
0,96938	1,00E-05	1,00E-05	0,0306		38
NA	NA	NA	NA	NA	
NA	NA	NA	NA	NA	
0,818028	0,0959	0,0628	0,0233		37
NA	NA	NA	NA	NA	
NA	NA	NA	NA	NA	
0,896603	0,0565	0,0469	1,00E-05		31
NA	NA	NA	NA	NA	

NA	NA	NA	NA	NA
0,434457	0,0521	0,513416	1,00E-05	38
NA	NA	NA	NA	NA
NA	NA	NA	NA	NA
0,75902	0,0831	0,15788	1,00E-05	26
NA	NA	NA	NA	NA
NA	NA	NA	NA	NA
0,878457	0,048	0,0736	1,00E-05	26
NA	NA	NA	NA	NA
NA	NA	NA	NA	NA
0,910945	0,0547	0,0343	1,00E-05	34
NA	NA	NA	NA	NA
NA	NA	NA	NA	NA
1,00E-05	1,20E-05	1,00E-05	0,999968	32
NA	NA	NA	NA	NA
NA	NA	NA	NA	NA
0,958295	0,00225	0,0256	0,0138	30
NA	NA	NA	NA	NA
NA	NA	NA	NA	NA
0,873606	0,0846	0,0418	1,00E-05	24
NA	NA	NA	NA	NA
NA	NA	NA	NA	NA
0,283157	0,465215	0,235728	0,0159	19
NA	NA	NA	NA	NA
NA	NA	NA	NA	NA
0,602532	0,173749	0,223709	1,00E-05	26
NA	NA	NA	NA	NA
NA	NA	NA	NA	NA
0,670649	0,0954	0,22922	0,00476	21
NA	NA	NA	NA	NA
NA	NA	NA	NA	NA
0,728518	0,0394	0,21202	0,0201	33
NA	NA	NA	NA	NA
NA	NA	NA	NA	NA
0,802904	0,119029	0,0319	0,0461	34
NA	NA	NA	NA	NA
NA	NA	NA	NA	NA
0,470222	0,0143	1,00E-05	0,515498	32
NA	NA	NA	NA	NA
NA	NA	NA	NA	NA
0,795048	0,104626	0,0693	0,031	31
NA	NA	NA	NA	NA
NA	NA	NA	NA	NA
0,948065	0,0259	0,0261	1,00E-05	24
NA	NA	NA	NA	NA
0,549749	0,0796	0,349878	0,0208	33
NA	NA	NA	NA	NA
NA	NA	NA	NA	NA
0,873542	0,102162	0,0201	0,00422	NA
NA	NA	NA	NA	NA
NA	NA	NA	NA	NA
0,768356	0,184809	0,0349	0,119	NA

NA	NA	NA	NA	NA	
NA	NA	NA	NA	NA	
0,742658	0,2362	0,0211	1,00E-05	NA	
NA	NA	NA	NA	NA	
NA	NA	NA	NA	NA	
0,831236	0,151314	0,0174	1,00E-05	NA	
NA	NA	NA	NA	NA	
NA	NA	NA	NA	NA	
0,648416	0,139517	0,212058	1,00E-05	NA	
NA	NA	NA	NA	NA	
NA	NA	NA	NA	NA	
NA	NA	NA	NA	NA	
0,810535	0,0818	0,105553	0,00215		28
NA	NA	NA	NA	NA	
NA	NA	NA	NA	NA	
1,00E-05	1,20E-05	1,00E-05	0,999968		30
NA	NA	NA	NA	NA	
0,897491	0,045	0,0329	0,0246	NA	
0,667709	0,0558	0,276488	1,00E-05		22
0,996818	1,00E-05	1,00E-05	0,00316	NA	
NA	NA	NA	NA	NA	
NA	NA	NA	NA	NA	
0,822296	0,0728	0,0888	0,0161		30
NA	NA	NA	NA	NA	
NA	NA	NA	NA	NA	
0,708843	0,0784	0,212779	1,00E-05		23
NA	NA	NA	NA	NA	
NA	NA	NA	NA	NA	
0,703731	0,119105	0,171571	0,00559	NA	
NA	NA	NA	NA	NA	
NA	NA	NA	NA	NA	
0,99997	1,00E-05	1,00E-05	1,00E-05		33
NA	NA	NA	NA	NA	
NA	NA	NA	NA	NA	
0,98947	1,00E-05	0,0105	1,00E-05		28
NA	NA	NA	NA	NA	
NA	NA	NA	NA	NA	
0,440166	0,216735	0,32482	0,0183		39
NA	NA	NA	NA	NA	
NA	NA	NA	NA	NA	
0,998159	1,00E-05	0,00182	1,00E-05	NA	
NA	NA	NA	NA	NA	
NA	NA	NA	NA	NA	
0,814418	0,0216	0,116639	0,0473	NA	
NA	NA	NA	NA	NA	
NA	NA	NA	NA	NA	
0,647913	0,0865	0,262679	0,00289		36
NA	NA	NA	NA	NA	
0,922793	0,0266	0,0479	0,00274		28
NA	NA	NA	NA	NA	
NA	NA	NA	NA	NA	
0,723473	0,0922	0,161647	0,227	NA	

NA	NA	NA	NA	NA
NA	NA	NA	NA	NA
0,683108	0,0971	0,219771	1,00E-05	NA
NA	NA	NA	NA	NA
NA	NA	NA	NA	NA
0,727076	0,0946	0,175162	0,00318	NA
NA	NA	NA	NA	NA
NA	NA	NA	NA	NA
0,821751	0,115611	0,0479	0,0147	NA
NA	NA	NA	NA	NA
NA	NA	NA	NA	NA
0,769338	0,178876	0,0261	0,0257	NA
0,134447	0,022	0,00831	0,835224	NA
NA	NA	NA	NA	NA
NA	NA	NA	NA	NA
0,809414	0,0583	0,132276	1,00E-05	NA
NA	NA	NA	NA	NA
NA	NA	NA	NA	NA
NA	NA	NA	NA	NA
NA	NA	NA	NA	NA
NA	NA	NA	NA	NA
0,93532	0,0397	0,025	1,00E-05	NA
NA	NA	NA	NA	NA
NA	NA	NA	NA	NA
NA	NA	NA	NA	NA
NA	NA	NA	NA	NA
NA	NA	NA	NA	NA
NA	NA	NA	NA	NA
NA	NA	NA	NA	NA
NA	NA	NA	NA	NA
NA	NA	NA	NA	NA
NA	NA	NA	NA	NA
NA	NA	NA	NA	NA
NA	NA	NA	NA	NA
0,894759	0,0749	0,0304	1,00E-05	NA
NA	NA	NA	NA	NA
NA	NA	NA	NA	NA
0,630219	0,160805	0,208966	1,00E-05	NA
NA	NA	NA	NA	NA
NA	NA	NA	NA	NA
NA	NA	NA	NA	NA
0,878751	0,0498	0,0714	1,00E-05	NA
NA	NA	NA	NA	NA
0,691203	0,0738	0,230907	0,00407	NA
NA	NA	NA	NA	NA
NA	NA	NA	NA	NA
NA	NA	NA	NA	NA
NA	NA	NA	NA	NA
NA	NA	NA	NA	NA
NA	NA	NA	NA	NA
NA	NA	NA	NA	NA
NA	NA	NA	NA	NA

NA	NA	NA	NA	NA
NA	NA	NA	NA	NA
NA	NA	NA	NA	NA
NA	NA	NA	NA	NA
NA	NA	NA	NA	NA
NA	NA	NA	NA	NA
NA	NA	NA	NA	NA
NA	NA	NA	NA	NA
NA	NA	NA	NA	NA
NA	NA	NA	NA	NA
NA	NA	NA	NA	NA
NA	NA	NA	NA	NA
NA	NA	NA	NA	NA
NA	NA	NA	NA	NA
NA	NA	NA	NA	NA
0,993041	1,00E-05	1,00E-05	0,00694	NA
NA	NA	NA	NA	NA
NA	NA	NA	NA	NA
0,690917	0,120139	0,184613	0,00433	NA
NA	NA	NA	NA	NA
NA	NA	NA	NA	NA
NA	NA	NA	NA	NA
0,880996	0,0543	0,0474	0,0174	NA
NA	NA	NA	NA	NA
NA	NA	NA	NA	NA
0,758967	0,152503	0,0885	1,00E-05	NA
NA	NA	NA	NA	NA
NA	NA	NA	NA	NA
NA	NA	NA	NA	NA
0,859061	0,0818	0,0445	0,0146	NA
NA	NA	NA	NA	NA
NA	NA	NA	NA	NA
0,96736	0,0326	1,00E-05	1,00E-05	NA
NA	NA	NA	NA	NA
NA	NA	NA	NA	NA
0,662921	0,0847	0,248456	0,0039	NA
NA	NA	NA	NA	NA
NA	NA	NA	NA	NA
0,85983	0,109598	0,023	0,00753	NA
NA	NA	NA	NA	NA
NA	NA	NA	NA	NA
0,654532	0,116703	0,0845	0,144238	NA
NA	NA	NA	NA	NA
NA	NA	NA	NA	NA
0,610442	0,0969	0,287337	0,00528	NA
NA	NA	NA	NA	NA
NA	NA	NA	NA	NA
NA	NA	NA	NA	NA
0,768375	0,0757	0,155944	1,00E-05	NA
NA	NA	NA	NA	NA
NA	NA	NA	NA	NA
NA	NA	NA	NA	NA

0,822254	0,0495	0,127054	0,00121	NA
NA	NA	NA	NA	NA
NA	NA	NA	NA	NA
0,953244	1,00E-05	0,0263	0,0204	NA
NA	NA	NA	NA	NA
NA	NA	NA	NA	NA
0,878234	0,00911	0,0965	0,0161	NA
NA	NA	NA	NA	NA
NA	NA	NA	NA	NA
0,99997	1,00E-05	1,00E-05	1,00E-05	NA
NA	NA	NA	NA	NA
NA	NA	NA	NA	NA
0,0855	0,0153	0,0798	0,81943	NA
NA	NA	NA	NA	NA
NA	NA	NA	NA	NA
0,844055	0,0565	0,0995	1,00E-05	NA
NA	NA	NA	NA	NA
NA	NA	NA	NA	NA
0,660056	0,119906	0,205119	0,0149	NA
NA	NA	NA	NA	NA
NA	NA	NA	NA	NA
0,273127	0,0364	0,172402	0,5181	NA
NA	NA	NA	NA	NA
NA	NA	NA	NA	NA
0,68375	0,140936	0,169359	0,00596	NA
NA	NA	NA	NA	NA
NA	NA	NA	NA	NA
0,825636	0,0542	0,116534	0,00364	NA
NA	NA	NA	NA	NA
NA	NA	NA	NA	NA
0,638586	0,120524	0,24088	1,00E-05	NA
NA	NA	NA	NA	NA
0,762157	0,0655	0,136284	0,036	NA
NA	NA	NA	NA	NA
NA	NA	NA	NA	NA
0,783497	0,0822	0,1343	1,00E-05	NA
NA	NA	NA	NA	NA
0,871205	0,0448	0,0839	1,00E-05	NA
NA	NA	NA	NA	NA
NA	NA	NA	NA	NA
0,831331	0,109491	0,0592	1,00E-05	NA
NA	NA	NA	NA	NA
NA	NA	NA	NA	NA
0,860467	0,0739	0,0656	1,00E-05	NA
NA	NA	NA	NA	NA
NA	NA	NA	NA	NA
0,816394	0,0141	0,164628	0,0049	NA
NA	NA	NA	NA	NA
NA	NA	NA	NA	NA
0,586042	0,117713	0,0832	0,213025	NA
NA	NA	NA	NA	NA
NA	NA	NA	NA	NA

NA	NA	NA	NA		NA
NA	NA	NA	NA		NA
NA	NA	NA	NA		NA
	0,387904	0,107573	0,504513	1,00E-05	NA
NA	NA	NA	NA		NA
NA	NA	NA	NA		NA
NA	NA	NA	NA		NA
NA	NA	NA	NA		NA
NA	NA	NA	NA		NA
NA	NA	NA	NA		NA
NA	NA	NA	NA		NA
	0,806517	0,0209	0,148082	0,0245	NA
NA	NA	NA	NA		NA
NA	NA	NA	NA		NA
NA	NA	NA	NA		NA
NA	NA	NA	NA		NA
NA	NA	NA	NA		NA
NA	NA	NA	NA		NA
	0,806863	0,0816	0,099	0,0126	NA
	0,750556	0,0767	0,156701	0,016	NA
NA	NA	NA	NA		NA
NA	NA	NA	NA		NA
NA	NA	NA	NA		NA
	0,534619	0,0645	0,389493	0,0114	NA
NA	NA	NA	NA		NA
	0,847149	0,0891	0,0517	0,0121	NA
NA	NA	NA	NA		NA
NA	NA	NA	NA		NA
NA	NA	NA	NA		NA
NA	NA	NA	NA		NA
	0,901385	0,0372	0,0571	0,00432	NA
NA	NA	NA	NA		NA
NA	NA	NA	NA		NA
	0,680411	0,119999	0,19958	1,00E-05	NA
NA	NA	NA	NA		NA
NA	NA	NA	NA		NA
	0,54721	0,129509	0,296431	0,0268	NA
NA	NA	NA	NA		NA
NA	NA	NA	NA		NA
	0,674083	0,0804	0,245484	1,00E-05	NA
NA	NA	NA	NA		NA
NA	NA	NA	NA		NA
	0,63179	0,0992	0,254553	0,0145	NA
NA	NA	NA	NA		NA
NA	NA	NA	NA		NA
	0,755046	0,0911	0,113968	0,0399	NA
NA	NA	NA	NA		NA
NA	NA	NA	NA		NA
	0,756191	0,0812	0,150618	0,012	NA
NA	NA	NA	NA		NA
NA	NA	NA	NA		NA
	0,748182	0,123698	0,117748	0,0104	NA

NA	NA	NA	NA	NA	
NA	NA	NA	NA	NA	
	0,501479	0,0864	0,412097	1,00E-05	NA
NA	NA	NA	NA	NA	
NA	NA	NA	NA	NA	
	0,814118	0,066	0,105788	0,0141	NA
NA	NA	NA	NA	NA	
NA	NA	NA	NA	NA	
	0,458042	0,0605	0,481449	1,00E-05	NA
NA	NA	NA	NA	NA	
NA	NA	NA	NA	NA	
	0,298265	0,0319	0,18053	0,489316	NA
NA	NA	NA	NA	NA	
NA	NA	NA	NA	NA	
NA	NA	NA	NA	NA	
	0,61159	0,106093	0,278875	0,00344	NA
NA	NA	NA	NA	NA	
NA	NA	NA	NA	NA	
	0,618684	0,0938	0,27064	0,0169	NA
NA	NA	NA	NA	NA	
NA	NA	NA	NA	NA	
	0,615009	0,075	0,294602	0,0154	NA
NA	NA	NA	NA	NA	
NA	NA	NA	NA	NA	
	0,982226	0,00621	1,00E-05	0,0116	NA
NA	NA	NA	NA	NA	
NA	NA	NA	NA	NA	
	0,821122	0,120713	0,0473	0,0108	NA
	0,898861	0,0561	0,0267	0,0184	NA
NA	NA	NA	NA	NA	
	0,99997	1,00E-05	1,00E-05	1,00E-05	NA
NA	NA	NA	NA	NA	
NA	NA	NA	NA	NA	
	0,863009	0,0409	0,087	0,0091	NA
NA	NA	NA	NA	NA	
	0,591562	0,077	0,316931	0,0145	NA
NA	NA	NA	NA	NA	
	0,727052	0,0724	0,185479	0,0151	NA
NA	NA	NA	NA	NA	
NA	NA	NA	NA	NA	
	0,851985	0,12945	0,0132	0,00539	NA
NA	NA	NA	NA	NA	
NA	NA	NA	NA	NA	
	0,650503	0,116107	0,20502	0,0284	NA
NA	NA	NA	NA	NA	
NA	NA	NA	NA	NA	
	0,931137	0,0337	0,0293	0,00592	NA
NA	NA	NA	NA	NA	
NA	NA	NA	NA	NA	
	0,937453	0,0467	0,0158	1,00E-05	NA
NA	NA	NA	NA	NA	
NA	NA	NA	NA	NA	

0,918801	0,048	0,0332	1,00E-05	NA
NA	NA	NA	NA	NA
NA	NA	NA	NA	NA
NA	NA	NA	NA	NA
0,680007	0,102857	0,208627	0,00851	NA
NA	NA	NA	NA	NA
NA	NA	NA	NA	NA
0,840921	0,0436	0,110204	0,00528	NA
NA	NA	NA	NA	NA
NA	NA	NA	NA	NA
0,638069	0,202432	0,159489	1,00E-05	NA
NA	NA	NA	NA	NA
NA	NA	NA	NA	NA
0,51778	0,262257	0,175372	0,0446	NA
NA	NA	NA	NA	NA
NA	NA	NA	NA	NA
NA	NA	NA	NA	NA
NA	NA	NA	NA	NA
NA	NA	NA	NA	NA
NA	NA	NA	NA	NA
NA	NA	NA	NA	NA
0,795308	0,192701	0,012	1,00E-05	NA
NA	NA	NA	NA	NA
NA	NA	NA	NA	NA
0,875434	0,0747	0,0499	1,00E-05	NA
0,442293	0,137648	0,420049	1,00E-05	NA
NA	NA	NA	NA	NA
NA	NA	NA	NA	NA
NA	NA	NA	NA	NA
NA	NA	NA	NA	NA
NA	NA	NA	NA	NA
NA	NA	NA	NA	NA
0,891788	0,0329	0,0502	0,0251	NA
NA	NA	NA	NA	NA
0,791066	0,0821	0,124372	0,00243	NA
NA	NA	NA	NA	NA
0,738978	0,084	0,138244	0,0388	NA
NA	NA	NA	NA	NA
NA	NA	NA	NA	NA
NA	NA	NA	NA	NA
NA	NA	NA	NA	NA
NA	NA	NA	NA	NA
NA	NA	NA	NA	NA
NA	NA	NA	NA	NA
NA	NA	NA	NA	NA
NA	NA	NA	NA	NA
NA	NA	NA	NA	NA
NA	NA	NA	NA	NA
NA	NA	NA	NA	NA

age_conception_father	age_evaluation	diagnosis	ADHD_comorbid_sign
45	9	ASD	no
NA	NA	NA	NA
NA	NA	NA	NA
28	5	ASD	no
NA	NA	NA	NA
NA	NA	NA	NA
30	3	ASD	no
NA	NA	NA	NA
NA	NA	NA	NA
27	10	ASD	no
NA	NA	NA	NA
NA	NA	NA	NA
43	10	ASD	no
NA	NA	NA	NA
NA	NA	NA	NA
34	3	DH ASD	no
NA	NA	NA	NA
NA	NA	NA	NA
29	13	ASD	yes
NA	NA	NA	NA
NA	NA	NA	NA
45	6	DH ASD	no
NA	NA	NA	NA
NA	NA	NA	NA
31	3	ASD	yes
NA	NA	NA	NA
NA	NA	NA	NA
34	4	DH ASD	no
NA	NA	NA	NA
NA	NA	NA	NA
28	12	ASD	yes
NA	NA	NA	NA
NA	NA	NA	NA
36	2	ASD	no
NA	NA	NA	NA
NA	NA	NA	NA
27	4	ASD	no
NA	NA	NA	NA
NA	NA	NA	NA
27	8	ASD	no
NA	NA	NA	NA
NA	NA	NA	NA
32	6	ASD	no
NA	NA	NA	NA
NA	NA	NA	NA
36	7	ASD	no
NA	NA	NA	NA
NA	NA	NA	NA
25	9	ASD	no
NA	NA	NA	NA

NA		NA	NA	NA
	29	11	ASD	no
NA		NA	NA	NA
NA		NA	NA	NA
NA		NA	NA	NA
NA		NA	NA	NA
NA		4	ASD	no
	27	14	ASD	yes
NA		NA	NA	NA
NA		NA	NA	NA
	28	15	ASD	no
NA		NA	NA	NA
NA		NA	NA	NA
	37	4	DH ASD	no
NA		NA	NA	NA
NA		NA	NA	NA
	37	5	ASD	yes
NA		NA	NA	NA
NA		NA	NA	NA
	32	6	ASD	no
NA		NA	NA	NA
NA		NA	NA	NA
	43	6	ASD	no
NA		NA	NA	NA
NA		NA	NA	NA
	43	3	ASD	no
NA		NA	NA	NA
NA		NA	NA	NA
	29	5	ASD	no
NA		NA	NA	NA
NA		NA	NA	NA
	32	6	DH ASD	no
NA		NA	NA	NA
NA		NA	NA	NA
	38	4	DH ASD	no
NA		NA	NA	NA
NA		NA	NA	NA
	28	23	ASD	no
NA		NA	NA	NA
NA		NA	NA	NA
	35	9	ASD	no
NA		NA	NA	NA
NA		NA	NA	NA
	37	4	ASD	yes
NA		NA	NA	NA
NA		NA	NA	NA
	34	3	DH ASD	no
NA		NA	NA	NA
NA		NA	NA	NA
	28	3	DH ASD	no
NA		NA	NA	NA
NA		NA	NA	NA

	34	4	ASD	no
NA		NA	NA	NA
NA		NA	NA	NA
	38	8	ASD	yes
NA		NA	NA	NA
NA		NA	NA	NA
	32	2	ASD	no
NA		NA	NA	NA
NA		NA	NA	NA
	33	3	DH ASD	no
NA		NA	NA	NA
NA		NA	NA	NA
	47	9	ASD	no
NA		NA	NA	NA
NA		NA	NA	NA
	28	4	ASD	no
NA		NA	NA	NA
NA		NA	NA	NA
	34	2	DH ASD	no
NA		NA	NA	NA
NA		NA	NA	NA
	32	2	ASD	no
NA		NA	NA	NA
NA		NA	NA	NA
	38	6	ASD	no
NA		NA	NA	NA
NA		NA	NA	NA
	32	5	DH ASD	no
NA		NA	NA	NA
NA		NA	NA	NA
NA		16	ASD	no
NA		NA	NA	NA
NA		NA	NA	NA
	22	10	ASD	no
NA		NA	NA	NA
NA		NA	NA	NA
	25	3	ASD	no
NA		NA	NA	NA
NA		NA	NA	NA
	31	5	ASD	no
NA		NA	NA	NA
NA		NA	NA	NA
	37	7	ASD	no
NA		NA	NA	NA
NA		NA	NA	NA
	38	12	ASD	yes
NA		NA	NA	NA
NA		NA	NA	NA
NA		NA	NA	NA
NA		NA	NA	NA
NA		NA	NA	NA
NA		6	ASD	no

NA		NA	NA	NA
NA		NA	NA	NA
NA		3	DH ASD	no
NA		NA	NA	NA
NA		NA	NA	NA
NA		NA	NA	NA
NA		NA	NA	NA
NA		7	ASD	no
	33	13	DH ASD	no
NA		NA	NA	NA
NA		NA	NA	NA
	43	22	ASD	no
NA		NA	NA	NA
NA		NA	NA	NA
NA		NA	NA	NA
NA		NA	NA	NA
NA		NA	NA	NA
NA		NA	NA	NA
NA		NA	NA	NA
NA		NA	NA	NA
NA		NA	NA	NA
NA		NA	NA	NA
NA		NA	NA	NA
NA		NA	NA	NA
NA		NA	NA	NA
NA		NA	NA	NA
NA		NA	NA	NA
NA		NA	NA	NA
NA		NA	NA	NA
NA		NA	NA	NA
NA		NA	NA	NA
NA		NA	NA	NA
NA		NA	NA	NA
NA		NA	NA	NA
NA		NA	NA	NA
NA		NA	NA	NA
NA		NA	NA	NA
NA		NA	NA	NA
NA		NA	NA	NA
NA		NA	NA	NA
NA		NA	NA	NA
NA		NA	NA	NA
NA		NA	NA	NA
NA		NA	NA	NA
NA		NA	NA	NA
NA		NA	NA	NA
NA		NA	NA	NA
NA		NA	NA	NA
NA		NA	NA	NA
NA		NA	NA	NA
NA		NA	NA	NA
NA		NA	NA	NA

NA	NA	NA	NA
NA	NA	NA	NA
NA	NA	NA	NA
NA	NA	NA	NA
NA	NA	NA	NA
NA	NA	NA	NA
NA	NA	NA	NA
NA	NA	NA	NA
NA	NA	NA	NA
NA	NA	NA	NA
NA	NA	NA	NA
NA	NA	NA	NA
NA	NA	NA	NA
NA	NA	NA	NA
NA	NA	NA	NA
NA	NA	NA	NA
NA	NA	NA	NA
NA	NA	NA	NA
NA	NA	NA	NA
NA	NA	NA	NA
NA	NA	NA	NA
NA	NA	NA	NA
NA	NA	NA	NA
NA	NA	NA	NA
NA	NA	NA	NA
NA	NA	NA	no
NA	NA	NA	NA
NA	NA	NA	NA
NA	NA	NA	NA
NA	NA	NA	NA
NA	NA	NA	NA
NA	NA	NA	NA
NA	NA	NA	NA
NA	NA	NA	no
NA	NA	NA	NA
NA	NA	NA	NA
NA	NA	NA	NA
NA	NA	NA	NA
NA	NA	NA	no
NA	NA	NA	NA
NA	NA	NA	NA
35	5	ASD	no
NA	NA	NA	NA
NA	NA	NA	NA
33	2	ASD	no
NA	NA	NA	NA
NA	NA	NA	NA
43	2	ASD	no
NA	NA	NA	NA
NA	NA	NA	NA
40	5	ASD	no
NA	NA	NA	NA

NA		NA	NA	NA
	25	4	ASD	no
NA		NA	NA	NA
NA		NA	NA	NA
	43	2	ASD	yes
NA		NA	NA	NA
NA		NA	NA	NA
	33	7	DH ASD	no
NA		NA	NA	NA
NA		NA	NA	NA
	34	4	ASD	no
NA		NA	NA	NA
NA		NA	NA	NA
	31	4	ASD	no
NA		NA	NA	NA
NA		NA	NA	NA
	24	9	ASD	no
NA		NA	NA	NA
NA		NA	NA	NA
	34	3	DH ASD	no
NA		NA	NA	NA
NA		NA	NA	NA
	38	2	DH ASD	no
NA		NA	NA	NA
NA		NA	NA	NA
	42	12	ASD	yes
NA		NA	NA	NA
NA		NA	NA	NA
	31	4	ASD	no
NA		NA	NA	NA
NA		NA	NA	NA
	28	2	DH ASD	no
NA		NA	NA	NA
NA		NA	NA	NA
	25	10	ASD	no
NA		NA	NA	NA
NA		NA	NA	NA
	43	3	DH ASD	no
NA		NA	NA	NA
NA		NA	NA	NA
	28	9	ASD	no
NA		NA	NA	NA
NA		NA	NA	NA
	32	5	ASD	yes
NA		NA	NA	NA
NA		NA	NA	NA
	33	8	ASD	no
NA		NA	NA	NA
NA		NA	NA	NA
	41	5	ASD	no
NA		NA	NA	NA
NA		NA	NA	NA

	30	6	ASD	yes
NA		NA	NA	NA
	32	3	ASD	no
NA		NA	NA	NA
NA		NA	NA	NA
	29	3	DH ASD	no
NA		NA	NA	NA
NA		NA	NA	NA
	35	7	ASD	no
NA		NA	NA	NA
NA		NA	NA	NA
	31	6	ASD	no
NA		NA	NA	NA
NA		NA	NA	NA
	38	2	DH ASD	no
NA		NA	NA	NA
NA		NA	NA	NA
	40	6	DH ASD	no
NA		NA	NA	NA
NA		NA	NA	NA
	29	6	DH ASD	no
NA		NA	NA	NA
NA		NA	NA	NA
NA		2	DH ASD	no
NA		NA	NA	NA
NA		NA	NA	NA
	38	20	ASD	yes
NA		NA	NA	NA
NA		NA	NA	NA
	29	10	ASD	no
NA		NA	NA	NA
NA		NA	NA	NA
	36	6	ASD	no
NA		NA	NA	NA
	36	4	DH ASD	no
NA		NA	NA	NA
NA		NA	NA	NA
	33	6	ASD	no
NA		NA	NA	NA
NA		NA	NA	NA
	33	9	ASD	yes
NA		NA	NA	NA
NA		NA	NA	NA
	40	3	DH ASD	no
NA		NA	NA	NA
NA		NA	NA	NA
	32	5	ASD	NA
NA		NA	NA	NA
NA		NA	NA	NA
	27	6	ASD	no
NA		NA	NA	NA
NA		NA	NA	NA

	36	4	ASD	no
NA	NA		NA	NA
NA	NA		NA	NA
	27	3	ASD	no
NA	NA		NA	NA
NA	NA		NA	NA
	34	3	ASD	no
NA	NA		NA	NA
NA	NA		NA	NA
	38	5	ASD	no
NA	NA		NA	NA
NA	NA		NA	NA
	32	3	ASD	no
NA	NA		NA	NA
NA	NA		NA	NA
	31	6	ASD	no
NA	NA		NA	NA
NA	NA		NA	NA
	35	3	ASD	no
NA	NA		NA	NA
NA	NA		NA	NA
	32	9	ASD	no
NA	NA		NA	NA
NA	NA		NA	NA
	36	2	ASD	yes
NA	NA		NA	NA
NA	NA		NA	NA
	34	6	ASD	no
NA	NA		NA	NA
NA	NA		NA	NA
	21	8	ASD	no
NA	NA		NA	NA
NA	NA		NA	NA
	39	2	ASD	no
NA	NA		NA	NA
NA	NA		NA	NA
	30	3	ASD	no
NA	NA		NA	NA
NA	NA		NA	NA
NA		3	ASD	no
NA	NA		NA	NA
NA	NA		NA	NA
	36	5	ASD	no
NA	NA		NA	NA
NA	NA		NA	NA
NA		4	ASD	no
NA	NA		NA	NA
	26	5	ASD	yes
NA	NA		NA	NA
NA	NA		NA	NA
	34	10	DH ASD	no
NA	NA		NA	NA

NA	NA		NA	NA
	40	4	ASD	no
NA	NA		NA	NA
NA	NA		NA	NA
	23	7	DH ASD	no
NA	NA		NA	NA
NA	NA		NA	NA
	41	5	DH ASD	no
NA	NA		NA	NA
NA	NA		NA	NA
	31	3	ASD	no
NA	NA		NA	NA
NA	NA		NA	NA
	33	7	ASD	no
NA	NA		NA	NA
NA	NA		NA	NA
NA	NA		NA	NA
NA	NA		NA	no
NA	NA		NA	NA
NA	NA		NA	NA
NA	NA		NA	NA
NA	NA		NA	no
NA	NA		NA	no
NA	NA		NA	NA
NA	NA		NA	NA
NA	NA		NA	NA
NA	NA		NA	NA
NA	NA		NA	NA
NA	NA		NA	NA
	35	5	ASD	no
NA	NA		NA	NA
	27	5	ASD	no
NA	NA		NA	NA
NA	NA		NA	NA
	30	4	ASD	no
NA	NA		NA	NA
NA	NA		NA	NA
NA		7	ASD	no
	32	5	DH ASD	no
NA	NA		NA	NA
	29	4	ASD	yes
NA	NA		NA	NA
NA	NA		NA	NA
	31	11	DH ASD	no
NA	NA		NA	NA
NA	NA		NA	NA
NA		13	DH ASD	no
NA	NA		NA	NA
NA	NA		NA	NA
NA	NA		NA	NA
	34	6	ASD	yes
	31	3	ASD	yes

NA		NA	NA	NA
NA		NA	NA	NA
	34	22	ASD	yes
NA		NA	NA	NA
	28	6	ASD	no
NA		NA	NA	NA
	26	9	ASD	no
NA		NA	NA	NA
NA		NA	NA	NA
	34	4	ASD	no
NA		NA	NA	NA
NA		NA	NA	NA
	34	3	ASD	yes
NA		NA	NA	NA
NA		NA	NA	NA
NA		2	DH ASD	no
NA		8	ASD	no
	23	19	ASD	yes
NA		NA	NA	NA
NA		10	ASD	no
NA		NA	NA	NA
NA		NA	NA	NA
	37	6	ASD	NA
NA		NA	NA	NA
NA		NA	NA	NA
	38	3	DH ASD	no
NA		NA	NA	NA
	25	12	DH ASD	yes
NA		NA	NA	NA
NA		NA	NA	NA
	37	4	ASD	no
NA		NA	NA	NA
NA		NA	NA	NA
	36	3	ASD	no
NA		NA	NA	NA
NA		NA	NA	NA
NA		NA	NA	NA
	39	10	ASD	no
NA		NA	NA	NA
NA		NA	NA	NA
	37	3	ASD	no
NA		NA	NA	NA
NA		NA	NA	NA
	31	9	ASD	no
NA		NA	NA	NA
NA		NA	NA	NA
NA		6	ASD	no
NA		6	ASD	no
NA		4	ASD	no
	40	2	DH ASD	no
NA		NA	NA	NA
NA		NA	NA	NA

	29	3	ASD	no
NA		NA	NA	NA
NA		NA	NA	NA
	25	3	ASD	yes
NA		NA	NA	NA
NA		NA	NA	NA
	29	4	DH ASD	no
NA		NA	NA	NA
NA		NA	NA	NA
	34	5	ASD	no
NA		NA	NA	NA
NA		NA	NA	NA
	34	4	ASD	no
NA		NA	NA	NA
NA		NA	NA	NA
NA		6	ASD	no
NA		NA	NA	NA
	48	9	ASD	no
NA		NA	NA	NA
NA		NA	NA	NA
NA		4	ASD	no
NA		NA	NA	NA
NA		7	ASD	no
NA		NA	NA	NA
	32	3	ASD	no
NA		NA	NA	NA
NA		NA	NA	NA
	34	4	ASD	NA
	20	6	DH ASD	no
NA		NA	NA	NA
NA		NA	NA	NA
	38	8	ASD	no
NA		NA	NA	NA
	31	11	DH ASD	no
NA		NA	NA	NA
NA		NA	NA	NA
	28	4	DH ASD	no
NA		NA	NA	NA
NA		NA	NA	NA
	32	2	DH ASD	no
NA		NA	NA	NA
NA		NA	NA	NA
NA		14	ASD	yes
NA		NA	NA	NA
NA		16	DH ASD	yes
NA		NA	NA	NA
	35	7	DH ASD	no
NA		NA	NA	NA
NA		2	ASD	no
NA		NA	NA	NA
	35	5	DH ASD	no
NA		NA	NA	NA

24		3	ASD	no
NA	NA		NA	NA
NA	NA		NA	NA
NA		8	ASD	no
NA		4	ASD	no
NA	NA		NA	NA
NA		4	DH ASD	no
NA	NA		NA	NA
34		1	ASD	no
NA	NA		NA	NA
NA	NA		NA	NA
32		1	ASD	no
NA	NA		NA	NA
NA	NA		NA	NA
NA		6	ASD	no
NA	NA		NA	NA
NA		6	ASD	no
NA	NA		NA	NA
36		1	ASD	no
NA	NA		NA	NA
NA	NA		NA	NA
NA		3	ASD	no
NA	NA		NA	NA
NA	NA		NA	NA
32		8	ASD	yes
34		15	DH ASD	yes
NA	NA		NA	NA
NA		15	ASD	no
35		4	ASD	no
NA	NA		NA	NA
NA	NA		NA	NA
34		5	ASD	no
NA	NA		NA	NA
32		5	ASD	no
NA	NA		NA	NA
NA	NA		NA	NA
NA	NA		NA	NA
38		4	ASD	NA
35		7	DH ASD	no
NA	NA		NA	NA
NA	NA		NA	NA
39		1	DH ASD	no
NA	NA		NA	NA
NA	NA		NA	NA
33		5	ASD	no
NA	NA		NA	NA
NA		27	DH ASD	no
26		5	ASD	no
NA	NA		NA	NA
35		2	DH ASD	no
NA	NA		NA	NA
NA	NA		NA	NA

	26	9	ASD	yes
NA		NA	NA	NA
NA		NA	NA	NA
	32	4	DH ASD	no
NA		NA	NA	NA
	33	6	ASD	no
NA		NA	NA	NA
NA		NA	NA	NA
	40	2	DH ASD	no
NA		NA	NA	NA
NA		NA	NA	NA
	31	NA	DH ASD	no
NA		NA	NA	NA
NA		NA	NA	NA
	33	11	ASD	yes
NA		NA	NA	NA
NA		NA	NA	NA
	51	6	ASD	no
NA		NA	NA	NA
NA		NA	NA	NA
	33	2	ASD	no
NA		NA	NA	NA
NA		NA	NA	NA
	27	10	ASD	no
NA		NA	NA	NA
NA		NA	NA	NA
	33	2	ASD	no
NA		NA	NA	NA
NA		NA	NA	NA
	27	6	ASD	no
NA		NA	NA	NA
NA		NA	NA	NA
	29	3	ASD	no
NA		NA	NA	NA
NA		NA	NA	NA
NA		8	DH ASD	no
NA		NA	NA	NA
NA		43	ASD	no
NA		NA	NA	NA
NA		6	ASD	no
	21	6	ASD	NA
	26	3	ASD	no
NA		NA	NA	NA
NA		NA	NA	NA
	27	3	ASD	no
NA		NA	NA	NA
NA		NA	NA	NA
	41	5	ASD	yes
NA		NA	NA	NA
NA		NA	NA	NA
	36	3	ASD	no
NA		NA	NA	NA

NA	NA		NA	NA
	30	6	ASD	no
NA	NA		NA	NA
NA	NA		NA	NA
	30	9	ASD	no
NA	NA		NA	NA
NA	NA		NA	NA
	43	5	ASD	no
NA	NA		NA	NA
NA	NA		NA	NA
	30	6	ASD	no
NA	NA		NA	NA
NA	NA		NA	NA
	35	7	ASD	no
NA	NA		NA	NA
NA	NA		NA	NA
NA	NA		NA	NA
NA	NA		NA	NA
	28	4	ASD	no
	30	2	DH ASD	yes
NA	NA		NA	NA
NA	NA		NA	NA
	34	1	DH ASD	no
NA	NA		NA	NA
NA	NA		NA	NA
	35	2	ASD	no
NA	NA		NA	NA
NA	NA		NA	NA
	30	8	ASD	no
NA	NA		NA	NA
NA	NA		NA	NA
	43	2	ASD	no
NA	NA		NA	NA
NA	NA		NA	NA
	31	6	ASD	no
NA	NA		NA	NA
NA	NA		NA	NA
	30	4	ASD	no
NA	NA		NA	NA
NA	NA		NA	NA
	29	9	ASD	no
NA	NA		NA	NA
NA	NA		NA	NA
	39	13	ASD	no
NA	NA		NA	NA
NA	NA		NA	NA
	46	2	ASD	no
NA	NA		NA	NA
NA	NA		NA	NA
	37	6	ASD	no
NA	NA		NA	NA
NA	NA		NA	NA

	27	6	ASD	no
NA	NA		NA	NA
NA	NA		NA	NA
	22	11	ASD	no
NA	NA		NA	NA
NA	NA		NA	NA
	37	4	ASD	yes
NA	NA		NA	NA
NA	NA		NA	NA
	32	6	ASD	no
NA	NA		NA	NA
NA	NA		NA	NA
	25	4	ASD	no
NA	NA		NA	NA
NA	NA		NA	NA
	44	3	DH ASD	no
NA	NA		NA	NA
NA	NA		NA	NA
	26	7	ASD	yes
NA	NA		NA	NA
NA	NA		NA	NA
	33	5	ASD	no
NA	NA		NA	NA
NA	NA		NA	NA
	45	3	ASD	no
NA	NA		NA	NA
NA	NA		NA	NA
	31	9	ASD	no
NA	NA		NA	NA
NA	NA		NA	NA
	35	7	ASD	no
NA	NA		NA	NA
NA	NA		NA	NA
NA		5	DH ASD	no
	30	13	DH ASD	no
NA	NA		NA	NA
NA	NA		NA	NA
	37	6	ASD	no
NA	NA		NA	NA
NA	NA		NA	NA
	45	4	ASD	no
NA	NA		NA	NA
NA	NA		NA	NA
	35	2	ASD	no
NA	NA		NA	NA
NA	NA		NA	NA
	36	6	ASD	no
NA	NA		NA	NA
NA	NA		NA	NA
	37	6	ASD	no
NA	NA		NA	NA
NA	NA		NA	NA

	30	10	ASD	no
NA	NA		NA	NA
NA	NA		NA	NA
	29	17	ASD	yes
NA	NA		NA	NA
NA	NA		NA	NA
	33	3	ASD	no
NA	NA		NA	NA
NA	NA		NA	NA
	32	10	DH ASD	no
NA	NA		NA	NA
NA	NA		NA	NA
	33	3	DH ASD	no
NA	NA		NA	NA
NA	NA		NA	NA
	40	7	DH ASD	NA
	33	3	DH ASD	yes
NA	NA		NA	NA
NA	NA		NA	NA
	32	6	ASD	NA
NA	NA		NA	NA
NA	NA		NA	NA
	32	10	ASD	no
NA	NA		NA	NA
NA	NA		NA	NA
	47	7	ASD	no
NA	NA		NA	NA
	31	6	ASD	NA
NA	NA		NA	NA
NA	NA		NA	NA
	23	10	ASD	yes
NA	NA		NA	NA
NA	NA		NA	NA
	24	18	ASD	yes
NA	NA		NA	NA
NA	NA		NA	NA
	28	6	ASD	no
NA	NA		NA	NA
NA	NA		NA	NA
	32	3	ASD	NA
NA	NA		NA	NA
NA	NA		NA	NA
	30	2	ASD	no
NA	NA		NA	NA
NA	NA		NA	NA
	36	6	ASD	no
NA	NA		NA	NA
NA	NA		NA	NA
	30	2	ASD	NA
NA	NA		NA	NA
NA	NA		NA	NA
	38	3	ASD	no

NA	NA	NA	NA
NA	NA	NA	NA
25	6	ASD	no
NA	NA	NA	NA
NA	NA	NA	NA
32	7	ASD	no
NA	NA	NA	NA
NA	NA	NA	NA
36	7	DH ASD	no
NA	NA	NA	NA
NA	NA	NA	NA
31	4	ASD	no
NA	NA	NA	NA
NA	NA	NA	NA
21	12	ASD	no
NA	NA	NA	NA
NA	NA	NA	NA
30	6	ASD	no
NA	NA	NA	NA
NA	NA	NA	NA
34	3	ASD	no
NA	NA	NA	NA
NA	NA	NA	NA
26	5	ASD	no
NA	NA	NA	NA
NA	NA	NA	NA
30	4	DH ASD	NA
NA	NA	NA	NA
NA	NA	NA	NA
24	6	ASD	no
NA	NA	NA	NA
NA	NA	NA	NA
40	10	ASD	NA
NA	NA	NA	NA
NA	NA	NA	NA
24	6	ASD	no
NA	NA	NA	NA
NA	NA	NA	NA
31	2	ASD	NA
NA	NA	NA	NA
NA	NA	NA	NA
26	5	ASD	no
NA	NA	NA	NA
NA	NA	NA	NA
32	6	ASD	no
NA	NA	NA	NA
NA	NA	NA	NA
48	4	ASD	no
NA	NA	NA	NA
NA	NA	NA	NA
31	5	ASD	no
NA	NA	NA	NA

NA	NA	NA		NA
	31	11	ASD	no
NA	NA	NA		NA
NA	NA	NA		NA
	26	5	ASD	yes
NA	NA	NA		NA
NA	NA	NA		NA
	38	6	ASD	no
NA	NA	NA		NA
NA	NA	NA		NA
	38	3	ASD	no
NA	NA	NA		NA
NA	NA	NA		NA
	32	4	ASD	no
NA	NA	NA		NA
NA	NA	NA		NA
	34	4	ASD	no
NA	NA	NA		NA
NA	NA	NA		NA
	26	5	ASD	no
NA	NA	NA		NA
NA	NA	NA		NA
	19	4	ASD	no
NA	NA	NA		NA
NA	NA	NA		NA
	28	3	ASD	yes
NA	NA	NA		NA
NA	NA	NA		NA
	22	5	DH ASD	yes
NA	NA	NA		NA
NA	NA	NA		NA
	35	3	ASD	no
NA	NA	NA		NA
NA	NA	NA		NA
	35	6	ASD	no
NA	NA	NA		NA
NA	NA	NA		NA
	31	2	ASD	no
NA	NA	NA		NA
NA	NA	NA		NA
	32	4	ASD	no
NA	NA	NA		NA
NA	NA	NA		NA
	30	29	ASD	NA
NA	NA	NA		NA
	27	4	ASD	no
NA	NA	NA		NA
NA	NA	NA		NA
NA		2	ASD	no
NA	NA	NA		NA
NA	NA	NA		NA
NA		2	ASD	no

NA		NA	NA	NA
NA		NA	NA	NA
NA		2	ASD	no
NA		NA	NA	NA
NA		NA	NA	NA
NA		4	ASD	no
NA		NA	NA	NA
NA		NA	NA	NA
NA		4	ASD	no
NA		NA	NA	NA
NA		NA	NA	NA
NA		9	ASD	no
	26	12	ASD	yes
NA		NA	NA	NA
NA		NA	NA	NA
	32	12	DH ASD	NA
NA		NA	NA	NA
NA		NA	NA	NA
	31	26	ASD	NA
NA		3	ASD	NA
NA		NA	NA	NA
NA		NA	NA	NA
	33	13	ASD	no
NA		NA	NA	NA
NA		NA	NA	NA
NA		21	DH ASD	no
NA		NA	NA	NA
NA		NA	NA	NA
NA		8	ASD	NA
NA		NA	NA	NA
NA		NA	NA	NA
	34	4	ASD	no
NA		NA	NA	NA
NA		NA	NA	NA
	21	15	ASD	NA
NA		NA	NA	NA
NA		NA	NA	NA
	42	3	ASD	no
NA		NA	NA	NA
NA		NA	NA	NA
NA		14	ASD	no
NA		30	ASD	no
NA		NA	NA	NA
NA		NA	ASD	no
NA		NA	NA	NA
NA		NA	NA	NA
NA		6	DH ASD	no
NA		NA	NA	NA
	38	2	ASD	no
NA		NA	NA	NA
NA		NA	NA	NA
NA		NA	NA	no

NA	NA	NA	NA
NA	NA	NA	NA
NA	NA	NA	no
NA	NA	NA	NA
NA	NA	NA	NA
NA	NA	NA	no
NA	NA	NA	NA
NA	NA	NA	NA
NA	NA	NA	NA
NA	NA	NA	NA
NA	NA	NA	NA
NA	NA	NA	NA
NA	NA	NA	NA
NA	NA	NA	NA
NA	NA	NA	NA
NA	NA	NA	NA
NA	NA	NA	NA
NA	NA	NA	NA
NA	NA	NA	NA
NA	NA	NA	NA
NA	NA	NA	NA
NA	NA	NA	no
NA	NA	NA	NA
NA	NA	NA	NA
NA	NA	NA	NA
NA	NA	NA	NA
NA	NA	NA	NA
NA	NA	NA	NA
NA	NA	NA	NA
NA	NA	NA	NA
NA	NA	NA	NA
NA	NA	NA	NA
NA	NA	NA	NA
NA	NA	NA	NA
NA	NA	NA	NA
NA	NA	NA	NA
NA	NA	NA	NA
NA	NA	NA	NA
NA	NA	NA	NA
NA	NA	NA	NA
NA	NA	NA	NA
NA	NA	NA	no
NA	NA	NA	NA
NA	NA	NA	NA
NA	NA	NA	NA
NA	NA	NA	NA
NA	NA	NA	no
NA	NA	NA	NA
NA	NA	NA	NA
NA	NA	NA	NA
NA	NA	NA	NA
NA	NA	NA	NA

NA	NA	NA	NA
NA	NA	NA	NA
NA	NA	NA	NA
NA	NA	NA	NA
NA	NA	NA	NA
NA	NA	NA	NA
NA	NA	NA	NA
NA	NA	NA	NA
NA	NA	NA	NA
NA	NA	NA	NA
NA	NA	NA	NA
NA	NA	NA	NA
NA	NA	NA	no
NA	NA	NA	NA
NA	NA	NA	NA
NA	NA	NA	no
NA	NA	NA	NA
NA	NA	NA	NA
NA	NA	NA	NA
NA	NA	NA	NA
NA	NA	NA	NA
NA	NA	NA	NA
NA	NA	NA	no
NA	NA	NA	NA
NA	NA	NA	NA
NA	NA	NA	NA
NA	NA	NA	NA
NA	NA	NA	NA
NA	NA	NA	NA
NA	NA	NA	NA
NA	NA	NA	NA
NA	NA	NA	NA
NA	NA	NA	no
NA	NA	NA	NA
NA	NA	NA	NA
NA	NA	NA	no
NA	NA	NA	NA
NA	NA	NA	NA
NA	NA	NA	no
NA	NA	NA	NA
NA	NA	NA	NA
NA	NA	NA	no
NA	NA	NA	NA
NA	NA	NA	NA
NA	NA	NA	no
NA	NA	NA	NA
NA	NA	NA	NA
NA	NA	NA	no
NA	NA	NA	no
NA	NA	NA	NA
NA	NA	NA	NA
NA	NA	NA	NA

NA	NA	NA	no
NA	NA	NA	NA
NA	NA	NA	NA
NA	NA	NA	NA
NA	NA	NA	NA
NA	NA	NA	NA
NA	NA	NA	NA
NA	NA	NA	NA
NA	NA	NA	NA
NA	NA	NA	no
NA	NA	NA	NA
NA	NA	NA	NA
NA	NA	NA	no
NA	NA	NA	NA
NA	NA	NA	NA
NA	NA	NA	no
NA	NA	NA	NA
NA	NA	NA	NA
NA	NA	NA	no
NA	NA	NA	NA
NA	NA	NA	NA
NA	NA	NA	no
NA	NA	NA	NA
NA	NA	NA	NA
NA	NA	NA	NA
NA	NA	NA	NA
NA	NA	NA	NA
NA	NA	NA	no
NA	NA	NA	NA
NA	NA	NA	NA
NA	NA	NA	NA
NA	NA	NA	NA
NA	NA	NA	no
NA	NA	NA	NA
NA	NA	NA	NA
NA	NA	NA	no
NA	NA	NA	NA
NA	NA	NA	no
NA	NA	NA	NA
NA	NA	NA	NA
NA	NA	NA	no
NA	NA	NA	NA
NA	NA	NA	NA
NA	NA	NA	NA
NA	NA	NA	NA
NA	NA	NA	NA
NA	NA	NA	no
NA	NA	NA	NA
NA	NA	NA	NA
NA	NA	NA	no
NA	NA	NA	NA
NA	NA	NA	NA

NA	NA	NA	no
NA	NA	NA	NA
NA	NA	NA	NA
NA	NA	NA	no
NA	NA	NA	no
NA	NA	NA	NA
NA	NA	NA	NA
NA	NA	NA	NA
NA	NA	NA	NA
NA	NA	NA	NA
NA	NA	NA	NA
NA	NA	NA	NA
NA	NA	NA	NA
NA	NA	NA	NA
NA	NA	NA	NA
NA	NA	NA	NA
NA	NA	NA	NA
NA	NA	NA	NA
NA	NA	NA	no
NA	NA	NA	NA
NA	NA	NA	NA
NA	NA	NA	NA
NA	NA	NA	no
NA	NA	NA	no
NA	NA	NA	NA
NA	NA	NA	no
NA	NA	NA	NA
NA	NA	NA	NA
NA	NA	NA	NA
NA	NA	NA	NA
NA	NA	NA	no
NA	NA	NA	NA
NA	NA	NA	NA
NA	NA	NA	no
NA	NA	NA	NA
NA	NA	NA	NA
NA	NA	NA	no
NA	NA	NA	NA
NA	NA	NA	NA
NA	NA	NA	no
NA	NA	NA	NA
NA	NA	NA	NA
NA	NA	NA	no
NA	NA	NA	NA
NA	NA	NA	NA
NA	NA	NA	no
NA	NA	NA	NA
NA	NA	NA	NA
NA	NA	NA	no
NA	NA	NA	NA
NA	NA	NA	NA
NA	NA	NA	NA

NA	NA	NA	NA
NA	NA	NA	NA
NA	NA	NA	no
NA	NA	NA	NA
NA	NA	NA	NA
NA	NA	NA	no
NA	NA	NA	NA
NA	NA	NA	NA
NA	NA	NA	no
NA	NA	NA	NA
NA	NA	NA	NA
NA	NA	NA	no
NA	NA	NA	NA
NA	NA	NA	NA
NA	NA	NA	yes
NA	NA	NA	NA
NA	NA	NA	NA
NA	NA	NA	NA
NA	NA	NA	no
NA	NA	NA	NA
NA	NA	NA	NA
NA	NA	NA	no
NA	NA	NA	NA
NA	NA	NA	NA
NA	NA	NA	no
NA	NA	NA	NA
NA	NA	NA	NA
NA	NA	NA	no
NA	NA	NA	no
NA	NA	NA	no
NA	NA	NA	no
NA	NA	NA	NA
NA	NA	NA	NA
NA	NA	NA	no
NA	NA	NA	NA
NA	NA	NA	no
NA	NA	NA	NA
NA	NA	NA	no
NA	NA	NA	NA
NA	NA	NA	NA
NA	NA	NA	no
NA	NA	NA	NA
NA	NA	NA	NA
NA	NA	NA	no
NA	NA	NA	NA
NA	NA	NA	NA
NA	NA	NA	no
NA	NA	NA	NA
NA	NA	NA	NA
NA	NA	NA	no
NA	NA	NA	NA
NA	NA	NA	NA

NA	NA	NA	no
NA	NA	NA	NA
NA	NA	NA	NA
NA	NA	NA	NA
NA	NA	NA	no
NA	NA	NA	NA
NA	NA	NA	NA
NA	NA	NA	NA
NA	NA	NA	NA
NA	NA	NA	NA
NA	NA	NA	NA
NA	NA	NA	NA
NA	NA	NA	NA
NA	NA	NA	NA
NA	NA	NA	NA
NA	NA	NA	NA
NA	NA	NA	NA
NA	NA	NA	NA
NA	NA	NA	NA
NA	NA	NA	NA
NA	NA	NA	NA
NA	NA	NA	NA
NA	NA	NA	NA
NA	NA	NA	NA
NA	NA	NA	NA
NA	NA	NA	NA
NA	NA	NA	NA
NA	NA	NA	NA
NA	NA	NA	NA
NA	NA	NA	NA
NA	NA	NA	NA
NA	NA	NA	NA
NA	NA	NA	NA
NA	NA	NA	NA
NA	NA	NA	NA
NA	NA	NA	NA
NA	NA	NA	NA
NA	NA	NA	NA
NA	NA	NA	NA
NA	NA	NA	NA
NA	NA	NA	NA
NA	NA	NA	NA
NA	NA	NA	NA
NA	NA	NA	NA
NA	NA	NA	NA
NA	NA	NA	NA
NA	NA	NA	NA
NA	NA	NA	NA
NA	NA	NA	NA

psychiatric_comorbidity_type	speech	hypotonia
no	non-verbal	no
NA	NA	NA
NA	NA	NA
no	verbal	no
NA	NA	NA
NA	NA	NA
no	verbal	no
NA	NA	NA
NA	NA	NA
no	verbal	no
NA	NA	NA
NA	NA	NA
no	verbal	NA
NA	NA	NA
NA	NA	NA
no	non-verbal	no
NA	NA	NA
NA	NA	NA
ADHD	verbal	no
NA	NA	NA
NA	NA	NA
no	verbal	no
NA	NA	NA
NA	NA	NA
ADHD	verbal	yes
NA	NA	NA
NA	NA	NA
no	non-verbal	no
NA	NA	NA
NA	NA	NA
ADHD	verbal	no
NA	NA	NA
NA	NA	NA
no	verbal	yes
NA	NA	NA
NA	NA	NA
no	non-verbal	no
NA	NA	NA
NA	NA	NA
no	non-verbal	no
NA	NA	NA
NA	NA	NA
no	verbal	no
NA	NA	NA
NA	NA	NA
no	verbal	no
NA	NA	NA
NA	NA	NA
ODD	verbal	yes
NA	NA	NA

NA	NA	NA
no	verbal	no
NA	NA	NA
NA	NA	NA
NA	NA	NA
NA	NA	NA
no	non-verbal	yes
ADHD	verbal	no
NA	NA	NA
NA	NA	NA
no	non-verbal	no
NA	NA	NA
NA	NA	NA
no	verbal	no
NA	NA	NA
NA	NA	NA
ADHD	verbal	no
NA	NA	NA
NA	NA	NA
no	non-verbal	no
NA	NA	NA
NA	NA	NA
no	verbal	no
NA	NA	NA
NA	NA	NA
no	non-verbal	no
NA	NA	NA
NA	NA	NA
no	verbal	no
NA	NA	NA
NA	NA	NA
no	non-verbal	no
NA	NA	NA
NA	NA	NA
no	non-verbal	NA
NA	NA	NA
NA	NA	NA
no	verbal	no
NA	NA	NA
NA	NA	NA
no	verbal	yes
NA	NA	NA
NA	NA	NA
ADHD	verbal	yes
NA	NA	NA
NA	NA	NA
no	non-verbal	yes
NA	NA	NA
NA	NA	NA
no	non-verbal	no
NA	NA	NA
NA	NA	NA

no	verbal	no
NA	NA	NA
NA	NA	NA
ADHD	verbal	no
NA	NA	NA
NA	NA	NA
no	<3years	NA
NA	NA	NA
NA	NA	NA
no	verbal	NA
NA	NA	NA
NA	NA	NA
no	non-verbal	NA
NA	NA	NA
NA	NA	NA
no	non-verbal	yes
NA	NA	NA
NA	NA	NA
no	<3years	no
NA	NA	NA
NA	NA	NA
no	verbal	yes
NA	NA	NA
NA	NA	NA
no	verbal	no
NA	NA	NA
NA	NA	NA
no	verbal	no
NA	NA	NA
NA	NA	NA
no	non-verbal	no
NA	NA	NA
NA	NA	NA
no	verbal	no
NA	NA	NA
NA	NA	NA
no	non-verbal	no
NA	NA	NA
NA	NA	NA
no	verbal	no
NA	NA	NA
NA	NA	NA
no	verbal	no
NA	NA	NA
NA	NA	NA
ADHD	verbal	no
NA	NA	NA
NA	NA	NA
NA	NA	NA
NA	NA	NA
NA	NA	NA
no	verbal	no

NA	NA	NA
NA	NA	NA
no	verbal	no
NA	NA	NA
NA	NA	NA
NA	NA	NA
NA	NA	NA
no	verbal	no
no	verbal	no
NA	NA	NA
NA	NA	NA
no	verbal	no
NA	NA	NA
NA	NA	NA
NA	NA	NA
NA	NA	NA
NA	NA	NA
NA	NA	NA
NA	NA	NA
NA	NA	NA
NA	NA	NA
NA	NA	NA
NA	NA	NA
NA	verbal	yes
NA	NA	NA
NA	NA	NA
NA	NA	NA
NA	NA	NA
NA	NA	NA
NA	NA	NA
NA	NA	NA
NA	NA	NA
NA	NA	NA
NA	NA	NA
NA	NA	NA
NA	NA	NA
NA	NA	NA
NA	NA	NA
NA	NA	NA
NA	NA	NA
NA	NA	NA
NA	NA	NA
NA	NA	NA
NA	NA	NA
NA	NA	NA
NA	NA	NA
NA	NA	NA
NA	NA	NA
NA	NA	NA
NA	NA	NA
NA	NA	NA
NA	NA	NA

NA	NA	NA
NA	NA	NA
NA	NA	NA
NA	NA	NA
NA	NA	NA
NA	NA	NA
NA	NA	NA
NA	NA	NA
NA	NA	NA
NA	NA	NA
NA	NA	NA
NA	NA	NA
NA	NA	NA
NA	NA	NA
NA	NA	NA
NA	NA	NA
NA	NA	NA
NA	NA	NA
NA	NA	NA
NA	NA	NA
NA	NA	NA
NA	NA	NA
NA	NA	NA
NA	NA	NA
NA	NA	NA
NA	verbal	no
NA	NA	NA
NA	NA	NA
NA	NA	NA
NA	NA	NA
NA	NA	NA
NA	NA	NA
NA	NA	NA
NA	non-verbal	no
NA	NA	NA
NA	NA	NA
NA	NA	NA
NA	NA	NA
NA	verbal	NA
NA	NA	NA
NA	NA	NA
no	verbal	no
NA	NA	NA
NA	NA	NA
no	<3years	no
NA	NA	NA
NA	NA	NA
no	verbal	no
NA	NA	NA
NA	NA	NA
no	verbal	yes
NA	NA	NA

NA	NA	NA
no	verbal	no
NA	NA	NA
NA	NA	NA
ADHD	<3years	no
NA	NA	NA
NA	NA	NA
no	verbal	no
NA	NA	NA
NA	NA	NA
no	non-verbal	no
NA	NA	NA
NA	NA	NA
no	verbal	no
NA	NA	NA
NA	NA	NA
no	verbal	no
NA	NA	NA
NA	NA	NA
no	non-verbal	no
NA	NA	NA
NA	NA	NA
no	verbal	no
NA	NA	NA
NA	NA	NA
ADHD	non-verbal	no
NA	NA	NA
NA	NA	NA
no	verbal	no
NA	NA	NA
NA	NA	NA
no	<3years	no
NA	NA	NA
NA	NA	NA
no	non-verbal	yes
NA	NA	NA
NA	NA	NA
OCD	verbal	no
NA	NA	NA
NA	NA	NA
no	non-verbal	no
NA	NA	NA
NA	NA	NA
ADHD	verbal	no
NA	NA	NA
NA	NA	NA
no	non-verbal	yes
NA	NA	NA
NA	NA	NA
no	verbal	yes
NA	NA	NA
NA	NA	NA

ADHD	non-verbal	no
NA	NA	NA
no	non-verbal	no
NA	NA	NA
NA	NA	NA
no	verbal	yes
NA	NA	NA
NA	NA	NA
no	verbal	yes
NA	NA	NA
NA	NA	NA
no	non-verbal	no
NA	NA	NA
NA	NA	NA
no	verbal	no
NA	NA	NA
NA	NA	NA
no	verbal	no
NA	NA	NA
NA	NA	NA
no	verbal	no
NA	NA	NA
NA	NA	NA
no	<3years	NA
NA	NA	NA
NA	NA	NA
ADHD	verbal	no
NA	NA	NA
NA	NA	NA
no	non-verbal	no
NA	NA	NA
NA	NA	NA
no	verbal	no
NA	NA	NA
no	verbal	no
NA	NA	NA
NA	NA	NA
no	non-verbal	no
NA	NA	NA
NA	NA	NA
ADHD	non-verbal	no
NA	NA	NA
NA	NA	NA
no	verbal	no
NA	NA	NA
NA	NA	NA
NA	non-verbal	no
NA	NA	NA
NA	NA	NA
no	verbal	yes
NA	NA	NA
NA	NA	NA

no	verbal	NA
NA	NA	NA
NA	NA	NA
no	non-verbal	no
NA	NA	NA
NA	NA	NA
no	non-verbal	no
NA	NA	NA
NA	NA	NA
no	verbal	yes
NA	NA	NA
NA	NA	NA
no	non-verbal	no
NA	NA	NA
NA	NA	NA
no	verbal	no
NA	NA	NA
NA	NA	NA
no	non-verbal	yes
NA	NA	NA
NA	NA	NA
no	verbal	yes
NA	NA	NA
NA	NA	NA
ADHD	<3years	yes
NA	NA	NA
NA	NA	NA
no	non-verbal	no
NA	NA	NA
NA	NA	NA
no	verbal	no
NA	NA	NA
NA	NA	NA
no	<3years	no
NA	NA	NA
NA	NA	NA
no	non-verbal	no
NA	NA	NA
NA	NA	NA
no	non-verbal	no
NA	NA	NA
NA	NA	NA
no	verbal	yes
NA	NA	NA
NA	NA	NA
no	verbal	no
NA	NA	NA
ADHD	verbal	no
NA	NA	NA
NA	NA	NA
no	non-verbal	yes
NA	NA	NA

NA	NA	NA
no	non-verbal	NA
NA	NA	NA
NA	NA	NA
no	verbal	no
NA	NA	NA
NA	NA	NA
no	NA	NA
NA	NA	NA
NA	NA	NA
no	non-verbal	no
NA	NA	NA
NA	NA	NA
no	verbal	no
NA	NA	NA
NA	NA	NA
NA	NA	NA
NA	verbal	NA
NA	NA	NA
NA	NA	NA
NA	non-verbal	NA
NA	verbal	no
NA	verbal	yes
NA	NA	NA
NA	NA	NA
NA	non-verbal	NA
NA	verbal	NA
NA	NA	NA
NA	NA	NA
no	verbal	no
NA	NA	NA
no	non-verbal	yes
NA	NA	NA
NA	NA	NA
no	non-verbal	yes
NA	NA	NA
NA	NA	NA
no	verbal	no
no	verbal	NA
NA	NA	NA
ADHD	non-verbal	no
NA	NA	NA
NA	NA	NA
no	non-verbal	yes
NA	NA	NA
NA	NA	NA
no	verbal	no
NA	NA	NA
NA	NA	NA
NA	NA	NA
ADHD	non-verbal	no
ADHD	verbal	no

NA	NA	NA
NA	NA	NA
ADHD	verbal	no
NA	NA	NA
no	non-verbal	no
NA	NA	NA
no	non-verbal	yes
NA	NA	NA
NA	NA	NA
no	non-verbal	no
NA	NA	NA
NA	NA	NA
ADHD	non-verbal	no
NA	NA	NA
NA	NA	NA
no	<3years	no
no	verbal	no
ADHD; OCD	verbal	no
NA	NA	NA
no	non-verbal	yes
NA	NA	NA
NA	NA	NA
NA	verbal	no
NA	NA	NA
NA	NA	NA
no	non-verbal	no
NA	NA	NA
ADHD	verbal	no
NA	NA	NA
NA	NA	NA
no	verbal	no
NA	NA	NA
NA	NA	NA
no	non-verbal	yes
NA	NA	NA
NA	NA	NA
NA	NA	NA
no	non-verbal	no
NA	NA	NA
NA	NA	NA
no	verbal	yes
NA	NA	NA
NA	NA	NA
no	non-verbal	no
NA	NA	NA
NA	NA	NA
no	non-verbal	no
no	non-verbal	no
no	non-verbal	no
no	<3years	yes
NA	NA	NA
NA	NA	NA

no	verbal	yes
NA	NA	NA
NA	NA	NA
ADHD	non-verbal	yes
NA	NA	NA
NA	NA	NA
no	non-verbal	no
NA	NA	NA
NA	NA	NA
no	verbal	no
NA	NA	NA
NA	NA	NA
no	verbal	no
NA	NA	NA
NA	NA	NA
no	verbal	yes
NA	NA	NA
no	verbal	NA
NA	NA	NA
NA	NA	NA
no	non-verbal	yes
NA	NA	NA
no	verbal	no
NA	NA	NA
no	verbal	no
NA	NA	NA
NA	NA	NA
NA	verbal	no
no	non-verbal	no
NA	NA	NA
NA	NA	NA
no	verbal	no
NA	NA	NA
no	verbal	no
NA	NA	NA
NA	NA	NA
no	non-verbal	no
NA	NA	NA
NA	NA	NA
no	verbal	no
NA	NA	NA
NA	NA	NA
ADHD	verbal	no
NA	NA	NA
ADHD	verbal	no
NA	NA	NA
no	verbal	yes
NA	NA	NA
no	<3years	no
NA	NA	NA
no	verbal	NA
NA	NA	NA

no	non-verbal	no
NA	NA	NA
NA	NA	NA
no	verbal	yes
no	non-verbal	no
NA	NA	NA
no	verbal	no
NA	NA	NA
no	<3years	no
NA	NA	NA
NA	NA	NA
no	<3years	yes
NA	NA	NA
NA	NA	NA
no	verbal	yes
NA	NA	NA
no	non-verbal	no
NA	NA	NA
no	<3years	no
NA	NA	NA
NA	NA	NA
no	verbal	no
NA	NA	NA
NA	NA	NA
ADHD	verbal	yes
ADHD	verbal	no
NA	NA	NA
no	verbal	no
no	verbal	no
NA	NA	NA
NA	NA	NA
no	verbal	no
NA	NA	NA
no	non-verbal	no
NA	NA	NA
NA	NA	NA
NA	NA	NA
NA	verbal	no
no	verbal	yes
NA	NA	NA
NA	NA	NA
no	<3years	no
NA	NA	NA
NA	NA	NA
no	verbal	no
NA	NA	NA
no	verbal	NA
no	non-verbal	no
NA	NA	NA
no	<3years	no
NA	NA	NA
NA	NA	NA

ADHD; ODD	verbal	no
NA	NA	NA
NA	NA	NA
no	verbal	yes
NA	NA	NA
no	non-verbal	NA
NA	NA	NA
NA	NA	NA
no	<3years	yes
NA	NA	NA
NA	NA	NA
no	NA	NA
NA	NA	NA
NA	NA	NA
ADHD	non-verbal	yes
NA	NA	NA
NA	NA	NA
no	non-verbal	no
NA	NA	NA
NA	NA	NA
no	<3years	no
NA	NA	NA
NA	NA	NA
no	verbal	no
NA	NA	NA
NA	NA	NA
no	<3years	no
NA	NA	NA
NA	NA	NA
no	verbal	NA
NA	NA	NA
NA	NA	NA
no	non-verbal	no
NA	NA	NA
NA	NA	NA
no	verbal	yes
NA	NA	NA
no	verbal	no
NA	NA	NA
no	verbal	no
NA	verbal	NA
no	verbal	no
NA	NA	NA
NA	NA	NA
no	non-verbal	no
NA	NA	NA
NA	NA	NA
ADHD	non-verbal	yes
NA	NA	NA
NA	NA	NA
no	non-verbal	no
NA	NA	NA

NA	NA	NA
no	non-verbal	no
NA	NA	NA
NA	NA	NA
no	verbal	no
NA	NA	NA
NA	NA	NA
no	verbal	no
NA	NA	NA
NA	NA	NA
no	non-verbal	no
NA	NA	NA
NA	NA	NA
no	non-verbal	no
NA	NA	NA
NA	NA	NA
NA	NA	NA
NA	NA	NA
no	verbal	no
ADHD	<3years	no
NA	NA	NA
NA	NA	NA
no	verbal	no
NA	NA	NA
NA	NA	NA
no	verbal	no
NA	NA	NA
NA	NA	NA
no	verbal	no
NA	NA	NA
NA	NA	NA
no	<3years	no
NA	NA	NA
NA	NA	NA
no	non-verbal	no
NA	NA	NA
NA	NA	NA
no	verbal	NA
NA	NA	NA
NA	NA	NA
no	verbal	NA
NA	NA	NA
NA	NA	NA
no	non-verbal	no
NA	NA	NA
NA	NA	NA
no	verbal	no
NA	NA	NA
NA	NA	NA
no	verbal	no
NA	NA	NA
NA	NA	NA

ODD	verbal	no
NA	NA	NA
NA	NA	NA
no	non-verbal	no
NA	NA	NA
NA	NA	NA
ADHD	non-verbal	no
NA	NA	NA
NA	NA	NA
no	verbal	yes
NA	NA	NA
NA	NA	NA
no	verbal	yes
NA	NA	NA
NA	NA	NA
no	verbal	yes
NA	NA	NA
NA	NA	NA
ADHD	non-verbal	no
NA	NA	NA
NA	NA	NA
no	verbal	NA
NA	NA	NA
NA	NA	NA
no	non-verbal	no
NA	NA	NA
NA	NA	NA
no	non-verbal	no
NA	NA	NA
NA	NA	NA
no	verbal	NA
NA	NA	NA
NA	NA	NA
no	verbal	no
no	verbal	no
NA	NA	NA
NA	NA	NA
no	verbal	no
NA	NA	NA
NA	NA	NA
no	non-verbal	no
NA	NA	NA
NA	NA	NA
no	<3years	yes
NA	NA	NA
NA	NA	NA
no	NA	NA
NA	NA	NA
NA	NA	NA
no	verbal	no
NA	NA	NA
NA	NA	NA

no	non-verbal	no
NA	NA	NA
NA	NA	NA
ADHD	verbal	no
NA	NA	NA
NA	NA	NA
no	non-verbal	no
NA	NA	NA
NA	NA	NA
no	non-verbal	no
NA	NA	NA
NA	NA	NA
no	non-verbal	NA
NA	NA	NA
NA	NA	NA
NA	verbal	NA
ADHD	non-verbal	no
NA	NA	NA
NA	NA	NA
NA	verbal	NA
NA	NA	NA
NA	NA	NA
no	verbal	no
NA	NA	NA
NA	NA	NA
no	verbal	no
NA	NA	NA
NA	verbal	NA
NA	NA	NA
NA	NA	NA
ADHD	verbal	yes
NA	NA	NA
NA	NA	NA
ADHD	verbal	no
NA	NA	NA
NA	NA	NA
no	non-verbal	no
NA	NA	NA
NA	NA	NA
NA	non-verbal	no
NA	NA	NA
NA	NA	NA
no	<3years	no
NA	NA	NA
NA	NA	NA
no	verbal	yes
NA	NA	NA
NA	NA	NA
NA	<3years	yes
NA	NA	NA
NA	NA	NA
no	non-verbal	no

NA	NA	NA
NA	NA	NA
no	verbal	no
NA	NA	NA
NA	NA	NA
no	non-verbal	no
NA	NA	NA
NA	NA	NA
no	verbal	no
NA	NA	NA
NA	NA	NA
no	non-verbal	yes
NA	NA	NA
NA	NA	NA
no	non-verbal	no
NA	NA	NA
NA	NA	NA
no	non-verbal	no
NA	NA	NA
NA	NA	NA
no	verbal	no
NA	NA	NA
NA	NA	NA
no	non-verbal	no
NA	NA	NA
NA	NA	NA
NA	non-verbal	NA
NA	NA	NA
NA	NA	NA
no	non-verbal	NA
NA	NA	NA
NA	NA	NA
NA	verbal	NA
NA	NA	NA
NA	NA	NA
no	non-verbal	no
NA	NA	NA
NA	NA	NA
NA	<3years	NA
NA	NA	NA
NA	NA	NA
no	non-verbal	no
NA	NA	NA
NA	NA	NA
no	non-verbal	no
NA	NA	NA
NA	NA	NA
no	verbal	no
NA	NA	NA
NA	NA	NA
no	non-verbal	no
NA	NA	NA

NA	NA	NA
no	NA	NA
NA	NA	NA
NA	NA	NA
ADHD	non-verbal	no
NA	NA	NA
NA	NA	NA
no	non-verbal	yes
NA	NA	NA
NA	NA	NA
no	verbal	no
NA	NA	NA
NA	NA	NA
no	verbal	no
NA	NA	NA
NA	NA	NA
no	verbal	no
NA	NA	NA
NA	NA	NA
no	non-verbal	no
NA	NA	NA
NA	NA	NA
no	non-verbal	yes
NA	NA	NA
NA	NA	NA
ADHD	non-verbal	no
NA	NA	NA
NA	NA	NA
ADHD	verbal	NA
NA	NA	NA
NA	NA	NA
no	non-verbal	no
NA	NA	NA
NA	NA	NA
OCD	non-verbal	NA
NA	NA	NA
NA	NA	NA
no	<3years	no
NA	NA	NA
NA	NA	NA
no	non-verbal	no
NA	NA	NA
NA	NA	NA
NA	verbal	NA
NA	NA	NA
no	verbal	no
NA	NA	NA
NA	NA	NA
no	<3years	no
NA	NA	NA
NA	NA	NA
no	<3years	no

NA	NA	NA
NA	NA	NA
no	<3years	no
NA	NA	NA
NA	NA	NA
no	non-verbal	no
NA	NA	NA
NA	NA	NA
no	non-verbal	no
NA	NA	NA
NA	NA	NA
no	verbal	no
ADHD; ODD	verbal	no
NA	NA	NA
NA	NA	NA
NA	non-verbal	yes
NA	NA	NA
NA	NA	NA
NA	non-verbal	NA
NA	non-verbal	NA
NA	NA	NA
NA	NA	NA
no	non-verbal	yes
NA	NA	NA
NA	NA	NA
no	verbal	NA
NA	NA	NA
NA	NA	NA
NA	NA	NA
NA	NA	NA
NA	NA	NA
no	non-verbal	no
NA	NA	NA
NA	NA	NA
NA	non-verbal	NA
NA	NA	NA
NA	NA	NA
no	non-verbal	NA
NA	NA	NA
NA	NA	NA
no	non-verbal	NA
no	NA	NA
NA	NA	NA
no	NA	NA
NA	NA	NA
NA	NA	NA
no	verbal	no
NA	NA	NA
no	<3years	NA
NA	NA	NA
NA	NA	NA
NA	non-verbal	NA

NA	NA	NA
NA	NA	NA
NA	verbal	NA
NA	NA	NA
NA	NA	NA
NA	NA	NA
NA	NA	NA
NA	NA	NA
NA	non-verbal	NA
NA	NA	NA
NA	NA	NA
NA	verbal	NA
NA	NA	NA
NA	NA	NA
NA	NA	NA
NA	verbal	no
NA	NA	NA
NA	NA	NA
NA	NA	NA
NA	NA	NA
NA	NA	NA
NA	verbal	NA
NA	NA	NA
NA	NA	NA
NA	<3years	NA
NA	NA	NA
NA	NA	NA
NA	non-verbal	NA
NA	NA	NA
NA	non-verbal	NA
NA	NA	NA
NA	non-verbal	NA
NA	NA	NA
NA	NA	NA
NA	non-verbal	NA
NA	NA	NA
NA	NA	NA
NA	non-verbal	NA
NA	NA	NA
NA	NA	NA
NA	non-verbal	NA
NA	non-verbal	yes
NA	NA	NA
NA	NA	NA
NA	NA	NA
NA	NA	NA
NA	non-verbal	NA
NA	NA	NA
NA	NA	NA
NA	NA	NA
NA	NA	NA
NA	NA	NA

NA	NA	NA
NA	NA	NA
NA	NA	NA
NA	non-verbal	NA
NA	NA	NA
NA	NA	NA
NA	<3years	NA
NA	NA	NA
NA	NA	NA
NA	non-verbal	NA
NA	NA	NA
NA	NA	NA
NA	NA	NA
NA	NA	NA
NA	NA	NA
NA	non-verbal	no
NA	NA	NA
NA	NA	NA
NA	non-verbal	NA
NA	NA	NA
NA	NA	NA
NA	<3years	NA
NA	<3years	no
NA	NA	NA
NA	NA	NA
NA	verbal	NA
NA	NA	NA
NA	non-verbal	NA
NA	NA	NA
NA	verbal	no
NA	NA	NA
NA	NA	NA
NA	non-verbal	no
NA	NA	NA
NA	NA	NA
NA	non-verbal	NA
NA	NA	NA
NA	NA	NA
NA	verbal	yes
NA	NA	NA
NA	NA	NA
NA	non-verbal	no
NA	NA	NA
NA	NA	NA
NA	non-verbal	no
NA	NA	NA
NA	NA	NA
NA	non-verbal	NA
NA	non-verbal	no
NA	NA	NA
NA	NA	NA
NA	non-verbal	NA

NA	verbal	no
NA	NA	NA
NA	NA	NA
NA	<3years	NA
NA	NA	NA
NA	NA	NA
NA	non-verbal	NA
NA	NA	NA
NA	NA	NA
NA	verbal	no
NA	NA	NA
NA	NA	NA
NA	non-verbal	NA
NA	NA	NA
NA	NA	NA
NA	non-verbal	no
NA	NA	NA
NA	NA	NA
NA	verbal	no
NA	NA	NA
NA	NA	NA
NA	non-verbal	no
NA	NA	NA
NA	NA	NA
NA	non-verbal	NA
NA	NA	NA
NA	NA	NA
NA	verbal	no
NA	NA	NA
NA	NA	NA
NA	non-verbal	no
NA	NA	NA
NA	non-verbal	no
NA	NA	NA
NA	NA	NA
NA	non-verbal	NA
NA	NA	NA
NA	verbal	yes
NA	NA	NA
NA	NA	NA
NA	verbal	no
NA	NA	NA
NA	NA	NA
NA	non-verbal	no
NA	NA	NA
NA	NA	NA
NA	non-verbal	NA
NA	NA	NA
NA	NA	NA
NA	non-verbal	no
NA	NA	NA
NA	NA	NA

NA	non-verbal	NA
NA	NA	NA
NA	NA	NA
NA	non-verbal	NA
NA	non-verbal	no
NA	NA	NA
NA	NA	NA
NA	NA	NA
NA	NA	NA
NA	NA	NA
NA	NA	NA
NA	verbal	NA
NA	NA	NA
NA	verbal	NA
NA	verbal	NA
NA	verbal	NA
NA	NA	NA
NA	NA	NA
NA	verbal	NA
NA	NA	NA
NA	NA	NA
NA	NA	NA
NA	NA	NA
NA	verbal	no
NA	NA	NA
NA	non-verbal	no
NA	NA	NA
NA	NA	NA
NA	NA	no
NA	NA	no
NA	<3years	no
NA	NA	NA
NA	NA	NA
NA	non-verbal	no
NA	NA	NA
NA	NA	NA
NA	non-verbal	NA
NA	NA	NA
NA	NA	NA
NA	non-verbal	no
NA	NA	NA
NA	NA	NA
NA	verbal	no
NA	NA	NA
NA	NA	NA
NA	verbal	no
NA	NA	NA
NA	NA	NA
NA	verbal	no
NA	NA	NA
NA	NA	NA
NA	verbal	no

NA	NA	NA
NA	NA	NA
NA	verbal	no
NA	NA	NA
NA	NA	NA
NA	verbal	NA
NA	NA	NA
NA	NA	NA
NA	verbal	NA
NA	NA	NA
NA	NA	NA
NA	verbal	NA
NA	NA	NA
NA	NA	NA
NA	verbal	no
NA	verbal	no
NA	NA	NA
NA	NA	NA
NA	verbal	no
NA	NA	NA
NA	NA	NA
NA	verbal	no
NA	NA	NA
NA	NA	NA
NA	non-verbal	yes
NA	NA	NA
NA	NA	NA
NA	verbal	no
NA	verbal	no
NA	non-verbal	no
NA	verbal	yes
NA	NA	NA
NA	NA	NA
NA	non-verbal	no
NA	NA	NA
NA	non-verbal	no
NA	NA	NA
NA	non-verbal	no
NA	NA	NA
NA	NA	NA
NA	verbal	no
NA	NA	NA
NA	NA	NA
NA	<3years	NA
NA	NA	NA
NA	NA	NA
NA	non-verbal	NA
NA	NA	NA
NA	NA	NA
NA	verbal	NA
NA	NA	NA
NA	NA	NA

NA	non-verbal	no
NA	NA	NA
NA	NA	NA
NA	NA	NA
NA	verbal	no
NA	NA	NA
NA	NA	NA
NA	NA	no
NA	NA	NA
NA	NA	NA
NA	verbal	no
NA	NA	NA
NA	NA	NA
NA	non-verbal	no
NA	NA	NA
NA	NA	NA
NA	NA	NA
NA	NA	NA
NA	NA	NA
NA	NA	NA
NA	NA	NA
NA	NA	NA
NA	NA	NA
NA	NA	NA
NA	NA	NA
NA	NA	NA
NA	NA	NA
NA	NA	NA
NA	NA	NA
NA	NA	NA
NA	NA	NA
NA	NA	NA
NA	NA	NA
NA	NA	NA
NA	NA	NA
NA	NA	NA
NA	NA	NA
NA	NA	NA
NA	NA	NA
NA	NA	NA
NA	NA	NA
NA	NA	NA
NA	NA	NA
NA	NA	NA
NA	NA	NA
NA	NA	NA
NA	NA	NA
NA	NA	NA
NA	NA	NA

ID_comorbid_sign	seizures	clinical_features_number
yes	no	2
NA	NA	NA
NA	NA	NA
NA	no	0
NA	NA	NA
NA	NA	NA
no	no	0
NA	NA	NA
NA	NA	NA
NA	no	0
NA	NA	NA
NA	NA	NA
NA	no	0
NA	NA	NA
NA	NA	NA
NA	no	1
NA	NA	NA
NA	NA	NA
yes	no	2
NA	NA	NA
NA	NA	NA
no	no	0
NA	NA	NA
NA	NA	NA
no	no	2
NA	NA	NA
NA	NA	NA
NA	yes, febrile	1
NA	NA	NA
NA	NA	NA
yes	no	2
NA	NA	NA
NA	NA	NA
NA	no	1
NA	NA	NA
NA	NA	NA
yes	no	2
NA	NA	NA
NA	NA	NA
yes	no	2
NA	NA	NA
NA	NA	NA
yes	yes	2
NA	NA	NA
NA	NA	NA
yes	yes, febrile	1
NA	NA	NA
NA	NA	NA
no	no	1
NA	NA	NA

NA	NA	NA	
yes	no		1
NA	NA	NA	
NA	NA	NA	
NA	NA	NA	
NA	NA	NA	
yes	no		3
no	no		1
NA	NA	NA	
NA	NA	NA	
yes	no		2
NA	NA	NA	
NA	NA	NA	
NA	no		0
NA	NA	NA	
NA	NA	NA	
NA	no		1
NA	NA	NA	
NA	NA	NA	
yes	no		2
NA	NA	NA	
NA	NA	NA	
yes	no		1
NA	NA	NA	
NA	NA	NA	
NA	no		1
NA	NA	NA	
NA	NA	NA	
no	no		0
NA	NA	NA	
NA	NA	NA	
yes	no		2
NA	NA	NA	
NA	NA	NA	
no	yes, febrile		1
NA	NA	NA	
NA	NA	NA	
NA	yes		1
NA	NA	NA	
NA	NA	NA	
NA	no		1
NA	NA	NA	
NA	NA	NA	
no	yes		3
NA	NA	NA	
NA	NA	NA	
NA	no		2
NA	NA	NA	
NA	NA	NA	
NA	no		1
NA	NA	NA	
NA	NA	NA	

no	no		0
NA	NA	NA	
NA	NA	NA	
no	no		1
NA	NA	NA	
NA	NA	NA	
NA	NA		0
NA	NA	NA	
NA	NA	NA	
NA	no		0
NA	NA	NA	
NA	NA	NA	
yes	no		2
NA	NA	NA	
NA	NA	NA	
yes	no		3
NA	NA	NA	
NA	NA	NA	
NA	no		0
NA	NA	NA	
NA	NA	NA	
NA	yes		2
NA	NA	NA	
NA	NA	NA	
no	yes, febrile		0
NA	NA	NA	
NA	NA	NA	
NA	no		0
NA	NA	NA	
NA	NA	NA	
yes	NA		2
NA	NA	NA	
NA	NA	NA	
no	NA		0
NA	NA	NA	
NA	NA	NA	
NA	no		1
NA	NA	NA	
NA	NA	NA	
no	NA		0
NA	NA	NA	
NA	NA	NA	
NA	yes, febrile		0
NA	NA	NA	
NA	NA	NA	
no	no		1
NA	NA	NA	
NA	NA	NA	
NA	NA	NA	
NA	NA	NA	
NA	NA	NA	
yes	no		1

NA	NA	NA	
NA	NA	NA	
NA	NA		0
NA	NA	NA	
NA	NA	NA	
NA	NA	NA	
NA	NA	NA	
no	no		0
NA	no		0
NA	NA	NA	
NA	NA	NA	
no	yes, febrile		0
NA	NA	NA	
NA	NA	NA	
NA	NA	NA	
NA	NA	NA	
NA	NA	NA	
NA	NA	NA	
NA	NA	NA	
NA	NA	NA	
NA	NA	NA	
NA	NA	NA	
NA	NA	NA	
no	no		1
NA	NA	NA	
NA	NA	NA	
NA	NA	NA	
NA	NA	NA	
NA	NA	NA	
NA	NA	NA	
NA	NA	NA	
NA	NA	NA	
NA	NA	NA	
NA	NA	NA	
NA	NA	NA	
NA	NA	NA	
NA	NA	NA	
NA	NA	NA	
NA	NA	NA	
NA	NA	NA	
NA	NA	NA	
NA	NA	NA	
NA	NA	NA	
NA	NA	NA	
NA	NA	NA	
NA	NA	NA	
NA	NA	NA	
NA	NA	NA	
NA	NA	NA	
NA	NA	NA	
NA	NA	NA	
NA	NA	NA	

NA	NA	NA	
NA	NA	NA	
NA	NA	NA	
NA	NA	NA	
NA	NA	NA	
NA	NA	NA	
NA	NA	NA	
NA	NA	NA	
NA	NA	NA	
NA	NA	NA	
NA	NA	NA	
NA	NA	NA	
NA	NA	NA	
NA	NA	NA	
NA	NA	NA	
NA	NA	NA	
NA	NA	NA	
NA	NA	NA	
NA	NA	NA	
NA	NA	NA	
NA	NA	NA	
NA	NA	NA	
NA	NA	NA	
NA	NA	NA	
NA	NA	NA	
no	no		0
NA	NA	NA	
NA	NA	NA	
NA	NA	NA	
NA	NA	NA	
NA	NA	NA	
NA	NA	NA	
NA	NA	NA	
no	no		1
NA	NA	NA	
NA	NA	NA	
NA	NA	NA	
NA	NA	NA	
NA	NA		0
NA	NA	NA	
NA	NA	NA	
no	no		0
NA	NA	NA	
NA	NA	NA	
no	no		0
NA	NA	NA	
NA	NA	NA	
NA	no		0
NA	NA	NA	
NA	NA	NA	
NA	yes		2
NA	NA	NA	

NA	NA	NA	
no	no		0
NA	NA	NA	
NA	NA	NA	
NA	no		1
NA	NA	NA	
NA	NA	NA	
NA	yes		1
NA	NA	NA	
NA	NA	NA	
no	no		1
NA	NA	NA	
NA	NA	NA	
NA	no		0
NA	NA	NA	
NA	NA	NA	
no	no		0
NA	NA	NA	
NA	NA	NA	
yes	no		2
NA	NA	NA	
NA	NA	NA	
no	no		0
NA	NA	NA	
NA	NA	NA	
yes	no		3
NA	NA	NA	
NA	NA	NA	
NA	no		0
NA	NA	NA	
NA	NA	NA	
NA	no		0
NA	NA	NA	
NA	NA	NA	
yes	no		3
NA	NA	NA	
NA	NA	NA	
yes	yes		2
NA	NA	NA	
NA	NA	NA	
no	no		1
NA	NA	NA	
NA	NA	NA	
NA	no		1
NA	NA	NA	
NA	NA	NA	
no	no		2
NA	NA	NA	
NA	NA	NA	
NA	no		1
NA	NA	NA	
NA	NA	NA	

no	no		2
NA	NA	NA	
NA	yes		2
NA	NA	NA	
NA	NA	NA	
NA	no		1
NA	NA	NA	
NA	NA	NA	
NA	no		1
NA	NA	NA	
NA	NA	NA	
yes	no		2
NA	NA	NA	
NA	NA	NA	
NA	no		0
NA	NA	NA	
NA	NA	NA	
NA	no		0
NA	NA	NA	
NA	NA	NA	
no	no		0
NA	NA	NA	
NA	NA	NA	
no	no		0
NA	NA	NA	
NA	NA	NA	
yes	no		2
NA	NA	NA	
NA	NA	NA	
NA	no		1
NA	NA	NA	
NA	NA	NA	
NA	no		0
NA	NA	NA	
no	no		0
NA	NA	NA	
NA	NA	NA	
yes	no		2
NA	NA	NA	
NA	NA	NA	
yes	yes		4
NA	NA	NA	
NA	NA	NA	
no	no		0
NA	NA	NA	
NA	NA	NA	
NA	no		1
NA	NA	NA	
NA	NA	NA	
NA	no		1
NA	NA	NA	
NA	NA	NA	

yes	no		1
NA	NA	NA	
NA	NA	NA	
NA	no		1
NA	NA	NA	
NA	NA	NA	
NA	no		1
NA	NA	NA	
NA	NA	NA	
NA	no		1
NA	NA	NA	
NA	NA	NA	
yes	no		2
NA	NA	NA	
NA	NA	NA	
NA	no		0
NA	NA	NA	
NA	NA	NA	
NA	no		2
NA	NA	NA	
NA	NA	NA	
NA	no		1
NA	NA	NA	
NA	NA	NA	
NA	no		2
NA	NA	NA	
NA	NA	NA	
NA	no		1
NA	NA	NA	
NA	NA	NA	
yes	no		1
NA	NA	NA	
NA	NA	NA	
NA	no		0
NA	NA	NA	
NA	NA	NA	
NA	no		1
NA	NA	NA	
NA	NA	NA	
NA	no		1
NA	NA	NA	
NA	NA	NA	
NA	no		1
NA	NA	NA	
NA	NA	NA	
NA	no		0
NA	NA	NA	
no	no		1
NA	NA	NA	
NA	NA	NA	
yes	no		3
NA	NA	NA	

NA	NA	NA	
NA	no		1
NA	NA	NA	
NA	NA	NA	
NA	no		0
NA	NA	NA	
NA	NA	NA	
NA	NA		0
NA	NA	NA	
NA	NA	NA	
NA	no		1
NA	NA	NA	
NA	NA	NA	
no	no		0
NA	NA	NA	
NA	NA	NA	
yes	NA		1
NA	NA		0
NA	NA	NA	
NA	NA	NA	
NA	NA		2
yes	no		1
NA	no		1
NA	NA	NA	
NA	NA	NA	
NA	NA		1
NA	NA		0
NA	NA	NA	
NA	NA	NA	
NA	no		0
NA	NA	NA	
NA	yes		3
NA	NA	NA	
NA	NA	NA	
NA	no		2
NA	NA	NA	
NA	NA	NA	
no	no		0
NA	no		0
NA	NA	NA	
yes	no		3
NA	NA	NA	
NA	NA	NA	
yes	no		3
NA	NA	NA	
NA	NA	NA	
yes	yes, febrile		1
NA	NA	NA	
NA	NA	NA	
NA	NA	NA	
NA	no		2
no	no		1

NA	NA	NA	
NA	NA	NA	
no	no		1
NA	NA	NA	
no	no		1
NA	NA	NA	
NA	yes		3
NA	NA	NA	
NA	NA	NA	
NA	yes		2
NA	NA	NA	
NA	NA	NA	
NA	no		2
NA	NA	NA	
NA	NA	NA	
NA	no		0
no	no		0
yes	no		2
NA	NA	NA	
yes	yes		4
NA	NA	NA	
NA	NA	NA	
NA	no		0
NA	NA	NA	
NA	NA	NA	
NA	no		1
NA	NA	NA	
yes	NA		2
NA	NA	NA	
NA	NA	NA	
no	no		0
NA	NA	NA	
NA	NA	NA	
yes	no		3
NA	NA	NA	
NA	NA	NA	
NA	NA	NA	
NA	no		1
NA	NA	NA	
NA	NA	NA	
NA	no		1
NA	NA	NA	
NA	NA	NA	
NA	no		1
NA	NA	NA	
NA	NA	NA	
NA	no		1
yes	no		2
yes	no		2
yes	yes		3
NA	NA	NA	
NA	NA	NA	

NA	no		1
NA	NA	NA	
NA	NA	NA	
yes	yes		5
NA	NA	NA	
NA	NA	NA	
yes	no		2
NA	NA	NA	
NA	NA	NA	
no	no		0
NA	NA	NA	
NA	NA	NA	
NA	no		0
NA	NA	NA	
NA	NA	NA	
yes	no		2
NA	NA	NA	
NA	no		0
NA	NA	NA	
NA	NA	NA	
NA	no		2
NA	NA	NA	
no	yes		1
NA	NA	NA	
NA	no		0
NA	NA	NA	
NA	NA	NA	
NA	no		0
yes	no		2
NA	NA	NA	
NA	NA	NA	
yes	no		1
NA	NA	NA	
yes	no		1
NA	NA	NA	
NA	NA	NA	
NA	no		1
NA	NA	NA	
NA	NA	NA	
no	no		0
NA	NA	NA	
NA	NA	NA	
yes	no		2
NA	NA	NA	
NA	no		1
NA	NA	NA	
NA	NA		1
NA	NA	NA	
no	no		0
NA	NA	NA	
NA	no		0
NA	NA	NA	

yes	yes		3
NA	NA	NA	
NA	NA	NA	
yes	no		2
yes	NA		2
NA	NA	NA	
yes	NA		1
NA	NA	NA	
NA	no		0
NA	NA	NA	
NA	NA	NA	
NA	yes		2
NA	NA	NA	
NA	NA	NA	
NA	NA		1
NA	NA	NA	
yes	yes		3
NA	NA	NA	
yes	no		1
NA	NA	NA	
NA	NA	NA	
no	no		0
NA	NA	NA	
NA	NA	NA	
yes	no		3
yes	no		2
NA	NA	NA	
no	no		0
no	no		0
NA	NA	NA	
NA	NA	NA	
NA	no		0
NA	NA	NA	
NA	yes		2
NA	NA	NA	
NA	NA	NA	
NA	NA	NA	
NA	no		0
NA	no		1
NA	NA	NA	
NA	NA	NA	
NA	no		0
NA	NA	NA	
NA	NA	NA	
NA	no		0
NA	NA	NA	
NA	NA		0
NA	no		1
NA	NA	NA	
NA	no		0
NA	NA	NA	
NA	NA	NA	

yes	no		2
NA	NA	NA	
NA	NA	NA	
NA	no		1
NA	NA	NA	
NA	no		1
NA	NA	NA	
NA	NA	NA	
NA	no		1
NA	NA	NA	
NA	NA	NA	
NA	NA		0
NA	NA	NA	
NA	NA	NA	
NA	yes		4
NA	NA	NA	
NA	NA	NA	
NA	no		1
NA	NA	NA	
NA	NA	NA	
NA	no		0
NA	NA	NA	
NA	NA	NA	
yes	no		1
NA	NA	NA	
NA	NA	NA	
NA	no		0
NA	NA	NA	
NA	NA	NA	
no	NA		0
NA	NA	NA	
NA	NA	NA	
NA	no		1
NA	NA	NA	
NA	NA	NA	
yes	no		2
NA	NA	NA	
no	no		0
NA	NA	NA	
no	yes		1
NA	no		0
NA	no		0
NA	NA	NA	
NA	NA	NA	
no	no		1
NA	NA	NA	
NA	NA	NA	
yes	no		4
NA	NA	NA	
NA	NA	NA	
NA	yes		2
NA	NA	NA	

NA	NA	NA	
NA	no		1
NA	NA	NA	
NA	NA	NA	
NA	no		0
NA	NA	NA	
NA	NA	NA	
no	no		0
NA	NA	NA	
NA	NA	NA	
yes	no		2
NA	NA	NA	
NA	NA	NA	
no	no		1
NA	NA	NA	
NA	NA	NA	
NA	NA	NA	
NA	NA	NA	
no	no		0
NA	no		1
NA	NA	NA	
NA	NA	NA	
no	yes		1
NA	NA	NA	
NA	NA	NA	
no	no		0
NA	NA	NA	
NA	NA	NA	
NA	no		0
NA	NA	NA	
NA	NA	NA	
no	no		0
NA	NA	NA	
NA	NA	NA	
NA	no		1
NA	NA	NA	
NA	NA	NA	
NA	no		0
NA	NA	NA	
NA	NA	NA	
NA	NA		0
NA	NA	NA	
NA	NA	NA	
yes	no		2
NA	NA	NA	
NA	NA	NA	
no	no		0
NA	NA	NA	
NA	NA	NA	
no	no		0
NA	NA	NA	
NA	NA	NA	

NA	no		0
NA	NA	NA	
NA	NA	NA	
yes	yes		3
NA	NA	NA	
NA	NA	NA	
NA	no		2
NA	NA	NA	
NA	NA	NA	
NA	NA		1
NA	NA	NA	
NA	NA	NA	
NA	no		1
NA	NA	NA	
NA	NA	NA	
no	no		1
NA	NA	NA	
NA	NA	NA	
NA	no		2
NA	NA	NA	
NA	NA	NA	
no	no		0
NA	NA	NA	
NA	NA	NA	
no	no		1
NA	NA	NA	
NA	NA	NA	
NA	no		1
NA	NA	NA	
NA	NA	NA	
NA	NA		0
NA	NA	NA	
NA	NA	NA	
no	no		0
yes	no		1
NA	NA	NA	
NA	NA	NA	
no	no		0
NA	NA	NA	
NA	NA	NA	
yes	no		2
NA	NA	NA	
NA	NA	NA	
yes	no		2
NA	NA	NA	
NA	NA	NA	
NA	no		0
NA	NA	NA	
NA	NA	NA	
NA	yes, febrile		0
NA	NA	NA	
NA	NA	NA	

NA	yes, febrile		1
NA	NA	NA	
NA	NA	NA	
yes	yes, febrile		2
NA	NA	NA	
NA	NA	NA	
no	yes		2
NA	NA	NA	
NA	NA	NA	
NA	no		1
NA	NA	NA	
NA	NA	NA	
NA	no		1
NA	NA	NA	
NA	NA	NA	
NA	yes		1
NA	no		2
NA	NA	NA	
NA	NA	NA	
NA	no		0
NA	NA	NA	
NA	NA	NA	
no	no		0
NA	NA	NA	
NA	NA	NA	
no	no		0
NA	NA	NA	
NA	NA		0
NA	NA	NA	
NA	NA	NA	
yes	yes		4
NA	NA	NA	
NA	NA	NA	
no	no		1
NA	NA	NA	
NA	NA	NA	
NA	no		1
NA	NA	NA	
NA	NA	NA	
NA	no		1
NA	NA	NA	
NA	NA	NA	
yes	no		1
NA	NA	NA	
NA	NA	NA	
NA	no		1
NA	NA	NA	
NA	NA	NA	
NA	no		1
NA	NA	NA	
NA	NA	NA	
NA	no		1

NA	NA	NA	
NA	NA	NA	
NA	no		0
NA	NA	NA	
NA	NA	NA	
NA	no		1
NA	NA	NA	
NA	NA	NA	
NA	NA		0
NA	NA	NA	
NA	NA	NA	
NA	yes		3
NA	NA	NA	
NA	NA	NA	
NA	no		1
NA	NA	NA	
NA	NA	NA	
NA	no		1
NA	NA	NA	
NA	NA	NA	
NA	no		0
NA	NA	NA	
NA	NA	NA	
NA	yes		2
NA	NA	NA	
NA	NA	NA	
NA	yes		2
NA	NA	NA	
NA	NA	NA	
yes	NA		2
NA	NA	NA	
NA	NA	NA	
NA	no		0
NA	NA	NA	
NA	NA	NA	
NA	no		1
NA	NA	NA	
NA	NA	NA	
NA	NA	NA	
NA	NA	NA	
NA	NA	NA	
NA	no		1
NA	NA	NA	
NA	NA	NA	
NA	no		1
NA	NA	NA	
NA	NA	NA	
NA	no		0
NA	NA	NA	
NA	NA	NA	
NA	no		1
NA	NA	NA	

NA	NA	NA	
NA	no		0
NA	NA	NA	
NA	NA	NA	
no	no		2
NA	NA	NA	
NA	NA	NA	
NA	yes, febrile		2
NA	NA	NA	
NA	NA	NA	
no	no		0
NA	NA	NA	
NA	NA	NA	
no	no		0
NA	NA	NA	
NA	NA	NA	
no	no		0
NA	NA	NA	
NA	NA	NA	
no	no		1
NA	NA	NA	
NA	NA	NA	
no	no		2
NA	NA	NA	
NA	NA	NA	
no	no		2
NA	NA	NA	
NA	NA	NA	
no	no		1
NA	NA	NA	
NA	NA	NA	
no	no		1
NA	NA	NA	
NA	NA	NA	
no	yes, febrile		1
NA	NA	NA	
NA	NA	NA	
no	no		0
NA	NA	NA	
NA	NA	NA	
no	no		1
NA	NA	NA	
NA	NA	NA	
NA	NA		0
NA	NA	NA	
NA	no		0
NA	NA	NA	
NA	NA	NA	
NA	no		0
NA	NA	NA	
NA	NA	NA	
no	no		0

NA	NA	NA	
NA	NA	NA	
no	no		0
NA	NA	NA	
NA	NA	NA	
no	yes		2
NA	NA	NA	
NA	NA	NA	
no	yes		2
NA	NA	NA	
NA	NA	NA	
NA	no		0
yes	no		2
NA	NA	NA	
NA	NA	NA	
yes	yes		4
NA	NA	NA	
NA	NA	NA	
NA	no		1
NA	no		1
NA	NA	NA	
NA	NA	NA	
yes	no		3
NA	NA	NA	
NA	NA	NA	
no	no		0
NA	NA	NA	
NA	NA	NA	
NA	NA	NA	
NA	NA	NA	
NA	NA	NA	
no	no		1
NA	NA	NA	
NA	NA	NA	
yes	NA		2
NA	NA	NA	
NA	NA	NA	
yes	no		2
NA	NA	NA	
NA	NA	NA	
yes	NA		2
yes	yes		2
NA	NA	NA	
NA	NA		0
NA	NA	NA	
NA	NA	NA	
no	no		0
NA	NA	NA	
NA	NA		0
NA	NA	NA	
NA	NA	NA	
NA	NA		1

NA	NA	NA	
NA	NA	NA	
NA	NA		0
NA	NA	NA	
NA	NA	NA	
NA	NA		0
NA	NA	NA	
NA	NA	NA	
NA	no		1
NA	NA	NA	
NA	NA	NA	
NA	NA		0
NA	yes, febrile	NA	
NA	NA	NA	
NA	NA	NA	
no	no		1
NA	NA	NA	
NA	NA	NA	
NA	NA	NA	
NA	NA	NA	
NA	NA	NA	
no	NA		0
NA	NA	NA	
NA	NA	NA	
NA	no		0
NA	NA	NA	
NA	NA	NA	
NA	no		2
NA	NA	NA	
yes	NA		2
NA	NA	NA	
NA	no		2
NA	NA	NA	
NA	NA	NA	
NA	yes		2
NA	NA	NA	
NA	NA	NA	
NA	NA		1
NA	NA	NA	
NA	NA	NA	
NA	NA		1
NA	NA		2
NA	NA	NA	
NA	NA	NA	
NA	NA	NA	
NA	NA	NA	
NA	NA		1
NA	NA	NA	
NA	NA	NA	
NA	yes		1
NA	NA	NA	
NA	NA	NA	

NA	NA	NA	
NA	NA	NA	
NA	NA	NA	
NA	no		1
NA	NA	NA	
NA	NA	NA	
NA	NA	NA	
NA	NA	NA	
NA	NA	NA	
NA	no		1
NA	NA	NA	
NA	NA	NA	
NA	no		0
NA	NA	NA	
NA	NA	NA	
yes	yes		3
NA	NA	NA	
NA	NA	NA	
NA	no		1
NA	NA	NA	
NA	NA	NA	
NA	NA	NA	
no	no		0
NA	NA	NA	
NA	NA	NA	
NA	NA		0
NA	NA	NA	
NA	NA		1
NA	NA	NA	
no	no		1
NA	NA	NA	
NA	NA	NA	
no	no		1
NA	NA	NA	
NA	NA	NA	
NA	no		1
NA	NA	NA	
NA	NA	NA	
NA	no		1
NA	NA	NA	
NA	NA	NA	
no	no		1
NA	NA	NA	
NA	NA	NA	
yes	no		2
NA	NA	NA	
NA	NA	NA	
yes	no		2
no	no		1
NA	NA	NA	
NA	NA	NA	
yes	yes		3

no	no		0
NA	NA	NA	
NA	NA	NA	
NA	NA	NA	
NA	NA	NA	
NA	NA	NA	
NA	NA		1
NA	NA	NA	
NA	NA	NA	
yes	no		1
NA	NA	NA	
NA	NA	NA	
NA	no		1
NA	NA	NA	
NA	NA	NA	
no	no		1
NA	NA	NA	
NA	NA	NA	
NA	no		0
NA	NA	NA	
NA	NA	NA	
no	no		1
NA	NA	NA	
NA	NA	NA	
NA	NA		1
NA	NA	NA	
NA	NA	NA	
no	no		0
NA	NA	NA	
NA	NA	NA	
no	NA		2
NA	NA	NA	
no	no		1
NA	NA	NA	
NA	NA	NA	
NA	NA		1
NA	NA	NA	
NA	no		1
NA	NA	NA	
NA	NA	NA	
no	no		0
NA	NA	NA	
NA	NA	NA	
no	NA		2
NA	NA	NA	
NA	NA	NA	
NA	no		1
NA	NA	NA	
NA	NA	NA	
NA	no		1
NA	NA	NA	
NA	NA	NA	

NA	NA		1
NA	NA	NA	
NA	NA	NA	
NA	NA		1
no	NA		1
NA	NA	NA	
NA	NA	NA	
NA	NA	NA	
NA	NA	NA	
NA	NA	NA	
NA	NA	NA	
NA	NA		0
NA	NA	NA	
NA	no		0
NA	no		0
NA	NA		0
NA	NA	NA	
NA	NA	NA	
yes	no		1
NA	NA	NA	
NA	NA	NA	
NA	NA	NA	
NA	NA		0
yes	no		1
NA	NA	NA	
NA	no		1
NA	NA	NA	
NA	NA	NA	
no	yes		1
no	no		0
yes	no		1
NA	NA	NA	
NA	NA	NA	
NA	no		1
NA	NA	NA	
NA	NA	NA	
NA	NA		1
NA	NA	NA	
NA	NA	NA	
NA	yes		2
NA	NA	NA	
NA	NA	NA	
NA	no		0
NA	NA	NA	
NA	NA	NA	
no	no		0
NA	NA	NA	
NA	NA	NA	
NA	yes		1
NA	NA	NA	
NA	NA	NA	
NA	no		1

NA	NA	NA	
NA	NA	NA	
NA	no		0
NA	NA	NA	
NA	NA	NA	
NA	no		0
NA	NA	NA	
NA	NA	NA	
NA	yes		1
NA	NA	NA	
NA	NA	NA	
NA	yes		1
NA	NA	NA	
NA	NA	NA	
NA	no		1
yes	no		2
NA	NA	NA	
NA	NA	NA	
NA	no		0
NA	NA	NA	
NA	NA	NA	
yes	yes		2
NA	NA	NA	
NA	NA	NA	
yes	yes, febrile		3
NA	NA	NA	
NA	NA	NA	
no	no		0
NA	no		0
NA	yes		2
no	yes		2
NA	NA	NA	
NA	NA	NA	
NA	NA		1
NA	NA	NA	
yes	no		2
NA	NA	NA	
NA	yes		2
NA	NA	NA	
NA	NA	NA	
no	no		0
NA	NA	NA	
NA	NA	NA	
NA	yes		1
NA	NA	NA	
NA	NA	NA	
NA	yes		2
NA	NA	NA	
NA	NA	NA	
no	yes, febrile		0
NA	NA	NA	
NA	NA	NA	

NA	NA		1
NA	NA	NA	
NA	NA	NA	
NA	NA	NA	
NA	no		0
NA	NA	NA	
NA	NA	NA	
no	no		1
NA	NA	NA	
NA	NA	NA	
NA	no		1
NA	NA	NA	
NA	NA	NA	
yes	no		3
NA	NA	NA	
NA	NA	NA	
NA	NA	NA	
NA	NA	NA	
NA	NA	NA	
NA	NA	NA	
NA	NA	NA	
NA	NA	NA	
NA	NA	NA	
NA	NA	NA	
NA	NA	NA	
NA	NA	NA	
NA	NA	NA	
NA	NA	NA	
NA	NA	NA	
NA	NA	NA	
NA	NA	NA	
NA	NA	NA	
NA	NA	NA	
NA	NA	NA	
NA	NA	NA	
NA	NA	NA	
NA	NA	NA	
NA	NA	NA	
NA	NA	NA	
NA	NA	NA	
NA	NA	NA	
NA	NA	NA	
NA	NA	NA	
NA	NA	NA	
NA	NA	NA	
NA	NA	NA	
NA	NA	NA	
NA	NA	NA	
NA	NA	NA	

family_recurrence	affected_member	CARS_score	Vineland_score
no	no	35,5	NA
NA	NA	NA	NA
NA	NA	NA	NA
no	no	30,5	NA
NA	NA	NA	NA
NA	NA	NA	NA
no	no	NA	NA
NA	NA	NA	NA
NA	NA	NA	NA
yes	Brother	32	NA
NA	NA	NA	NA
NA	NA	NA	NA
no	no	31	NA
NA	NA	NA	NA
NA	NA	NA	NA
no	no	31	76
NA	NA	NA	NA
NA	NA	NA	NA
no	no	32	65
NA	NA	NA	NA
NA	NA	NA	NA
no	no	35	57
NA	NA	NA	NA
NA	NA	NA	NA
no	no	NA	NA
NA	NA	NA	NA
NA	NA	NA	NA
no	no	27,5	NA
NA	NA	NA	NA
NA	NA	NA	NA
no	no	27,5	NA
NA	NA	NA	NA
NA	NA	NA	NA
no	no	35,5	NA
NA	NA	NA	NA
NA	NA	NA	NA
yes	Father	40	NA
NA	NA	NA	NA
NA	NA	NA	NA
no	no	NA	NA
NA	NA	NA	NA
NA	NA	NA	NA
no	no	37,5	45
NA	NA	NA	NA
NA	NA	NA	NA
no	no	30	NA
NA	NA	NA	NA
NA	NA	NA	NA
no	no	34	66
NA	NA	NA	NA

NA	NA	NA	NA
no	no	33,5	50
NA	NA	NA	NA
NA	NA	NA	NA
NA	NA	NA	NA
NA	NA	NA	NA
yes	Brother	NA	NA
no	no	31,5	75
NA	NA	NA	NA
NA	NA	NA	NA
no	no	NA	NA
NA	NA	NA	NA
NA	NA	NA	NA
no	no	33	NA
NA	NA	NA	NA
NA	NA	NA	NA
no	no	NA	NA
NA	NA	NA	NA
NA	NA	NA	NA
no	no	37	NA
NA	NA	NA	NA
NA	NA	NA	NA
no	no	NA	NA
NA	NA	NA	NA
NA	NA	NA	NA
no	no	NA	NA
NA	NA	NA	NA
NA	NA	NA	NA
no	no	NA	NA
NA	NA	NA	NA
NA	NA	NA	NA
yes	Brother	42,5	NA
NA	NA	NA	NA
NA	NA	NA	NA
no	no	NA	NA
NA	NA	NA	NA
NA	NA	NA	NA
no	no	33	NA
NA	NA	NA	NA
NA	NA	NA	NA
no	no	36	NA
NA	NA	NA	NA
NA	NA	NA	NA
no	no	30	NA
NA	NA	NA	NA
NA	NA	NA	NA
no	no	29,5	NA
NA	NA	NA	NA
NA	NA	NA	NA
no	no	30	NA
NA	NA	NA	NA
NA	NA	NA	NA

no	no	NA	NA
NA	NA	NA	NA
NA	NA	NA	NA
no	no	34	NA
NA	NA	NA	NA
NA	NA	NA	NA
no	no	36	NA
NA	NA	NA	NA
NA	NA	NA	NA
no	no	28	NA
NA	NA	NA	NA
NA	NA	NA	NA
no	no	NA	NA
NA	NA	NA	NA
NA	NA	NA	NA
no	no	36	NA
NA	NA	NA	NA
NA	NA	NA	NA
yes	Sister	38	NA
NA	NA	NA	NA
NA	NA	NA	NA
no	no	NA	NA
NA	NA	NA	NA
NA	NA	NA	NA
yes	Cousin	NA	NA
NA	NA	NA	NA
NA	NA	NA	NA
no	no	NA	NA
NA	NA	NA	NA
NA	NA	NA	NA
no	no	NA	NA
NA	NA	NA	NA
NA	NA	NA	NA
no	no	30,5	NA
NA	NA	NA	NA
NA	NA	NA	NA
no	no	36,5	NA
NA	NA	NA	NA
NA	NA	NA	NA
no	no	NA	NA
NA	NA	NA	NA
NA	NA	NA	NA
no	no	36,5	NA
NA	NA	NA	NA
NA	NA	NA	NA
no	no	NA	NA
NA	NA	NA	NA
NA	NA	NA	NA
NA	NA	NA	NA
NA	NA	NA	NA
NA	NA	NA	NA
yes	Brother	NA	NA

NA	NA	NA	NA
NA	NA	NA	NA
no	no	NA	NA
NA	NA	NA	NA
NA	NA	NA	NA
NA	NA	NA	NA
NA	NA	NA	NA
yes	Brother	NA	NA
no	no	33	NA
NA	NA	NA	NA
NA	NA	NA	NA
no	no	NA	NA
NA	NA	NA	NA
NA	NA	NA	NA
NA	NA	NA	NA
NA	NA	NA	NA
NA	NA	NA	NA
NA	NA	NA	NA
NA	NA	NA	NA
NA	NA	NA	NA
NA	NA	NA	NA
yes	Brother	NA	NA
NA	NA	NA	NA
yes	Brother	NA	NA
NA	NA	NA	NA
NA	NA	NA	NA
NA	NA	NA	NA
NA	NA	NA	NA
NA	NA	NA	NA
NA	NA	NA	NA
NA	NA	NA	NA
NA	NA	NA	NA
NA	NA	NA	NA
NA	NA	NA	NA
NA	NA	NA	NA
NA	NA	NA	NA
NA	NA	NA	NA
NA	NA	NA	NA
NA	NA	NA	NA
NA	NA	NA	NA
NA	NA	NA	NA
NA	NA	NA	NA
NA	NA	NA	NA
NA	NA	NA	NA
NA	NA	NA	NA
NA	NA	NA	NA
NA	NA	NA	NA
NA	NA	NA	NA
NA	NA	NA	NA
NA	NA	NA	NA
NA	NA	NA	NA
NA	NA	NA	NA

NA	NA	NA	NA
NA	NA	NA	NA
NA	NA	NA	NA
NA	NA	NA	NA
NA	NA	NA	NA
NA	NA	NA	NA
NA	NA	NA	NA
NA	NA	NA	NA
NA	NA	NA	NA
NA	NA	NA	NA
NA	NA	NA	NA
NA	NA	NA	NA
NA	NA	NA	NA
NA	NA	NA	NA
NA	NA	NA	NA
NA	NA	NA	NA
NA	NA	NA	NA
NA	NA	NA	NA
NA	NA	NA	NA
NA	NA	NA	NA
NA	NA	NA	NA
NA	NA	NA	NA
NA	NA	NA	NA
NA	NA	NA	NA
NA	NA	NA	NA
yes	Brother	NA	NA
NA	NA	NA	NA
NA	NA	NA	NA
NA	NA	NA	NA
NA	NA	NA	NA
NA	NA	NA	NA
NA	NA	NA	NA
NA	NA	NA	NA
no	no	NA	NA
NA	NA	NA	NA
NA	NA	NA	NA
NA	NA	NA	NA
NA	NA	NA	NA
no	no	NA	NA
NA	NA	NA	NA
NA	NA	NA	NA
yes	Cousin	32,5	63
NA	NA	NA	NA
NA	NA	NA	NA
no	no	NA	NA
NA	NA	NA	NA
NA	NA	NA	NA
no	no	34,5	77
NA	NA	NA	NA
NA	NA	NA	NA
no	no	30	66
NA	NA	NA	NA

NA	NA	NA	NA
no	no	31	61
NA	NA	NA	NA
NA	NA	NA	NA
no	no	32	69
NA	NA	NA	NA
NA	NA	NA	NA
no	no	28,5	NA
NA	NA	NA	NA
NA	NA	NA	NA
no	no	NA	NA
NA	NA	NA	NA
NA	NA	NA	NA
no	no	NA	NA
NA	NA	NA	NA
NA	NA	NA	NA
no	no	32,5	74
NA	NA	NA	NA
NA	NA	NA	NA
no	no	36,5	57
NA	NA	NA	NA
NA	NA	NA	NA
no	no	27	86
NA	NA	NA	NA
NA	NA	NA	NA
no	no	42,5	38
NA	NA	NA	NA
NA	NA	NA	NA
no	no	NA	NA
NA	NA	NA	NA
NA	NA	NA	NA
no	no	31,5	70
NA	NA	NA	NA
NA	NA	NA	NA
no	no	NA	NA
NA	NA	NA	NA
NA	NA	NA	NA
yes	Cousin	33	71
NA	NA	NA	NA
NA	NA	NA	NA
no	no	NA	NA
NA	NA	NA	NA
NA	NA	NA	NA
no	no	NA	NA
NA	NA	NA	NA
NA	NA	NA	NA
no	no	36	46
NA	NA	NA	NA
NA	NA	NA	NA
no	no	33	73
NA	NA	NA	NA
NA	NA	NA	NA

no	no	NA	NA
NA	NA	NA	NA
no	no	34	69
NA	NA	NA	NA
NA	NA	NA	NA
no	no	NA	NA
NA	NA	NA	NA
NA	NA	NA	NA
no	no	31	74
NA	NA	NA	NA
NA	NA	NA	NA
no	no	35,5	50
NA	NA	NA	NA
NA	NA	NA	NA
no	no	35	63
NA	NA	NA	NA
NA	NA	NA	NA
no	no	35,5	62
NA	NA	NA	NA
NA	NA	NA	NA
no	no	34,5	65
NA	NA	NA	NA
NA	NA	NA	NA
yes	Brother	30	71
NA	NA	NA	NA
NA	NA	NA	NA
no	no	33	38
NA	NA	NA	NA
NA	NA	NA	NA
no	no	NA	NA
NA	NA	NA	NA
NA	NA	NA	NA
no	no	33	73
NA	NA	NA	NA
no	no	32	67
NA	NA	NA	NA
NA	NA	NA	NA
no	no	34,5	53
NA	NA	NA	NA
NA	NA	NA	NA
no	no	39	52
NA	NA	NA	NA
NA	NA	NA	NA
no	no	33	NA
NA	NA	NA	NA
NA	NA	NA	NA
no	no	NA	NA
NA	NA	NA	NA
NA	NA	NA	NA
no	no	NA	NA
NA	NA	NA	NA
NA	NA	NA	NA

no	no	34	61
NA	NA	NA	NA
NA	NA	NA	NA
no	no	NA	NA
NA	NA	NA	NA
NA	NA	NA	NA
no	no	NA	NA
NA	NA	NA	NA
NA	NA	NA	NA
no	no	NA	NA
NA	NA	NA	NA
NA	NA	NA	NA
no	no	38	52
NA	NA	NA	NA
NA	NA	NA	NA
no	no	34	63
NA	NA	NA	NA
NA	NA	NA	NA
no	no	33	62
NA	NA	NA	NA
NA	NA	NA	NA
no	no	NA	NA
NA	NA	NA	NA
NA	NA	NA	NA
no	no	34	NA
NA	NA	NA	NA
NA	NA	NA	NA
no	no	37,5	NA
NA	NA	NA	NA
NA	NA	NA	NA
no	no	38	NA
NA	NA	NA	NA
NA	NA	NA	NA
no	no	NA	NA
NA	NA	NA	NA
NA	NA	NA	NA
yes	Cousin	NA	NA
NA	NA	NA	NA
NA	NA	NA	NA
no	no	30,5	NA
NA	NA	NA	NA
NA	NA	NA	NA
no	no	NA	NA
NA	NA	NA	NA
NA	NA	NA	NA
no	no	NA	NA
NA	NA	NA	NA
no	no	NA	NA
NA	NA	NA	NA
NA	NA	NA	NA
no	no	35	47
NA	NA	NA	NA

NA	NA	NA	NA
no	no	30	67
NA	NA	NA	NA
NA	NA	NA	NA
no	no	34	59
NA	NA	NA	NA
NA	NA	NA	NA
NA	NA	32	62
NA	NA	NA	NA
NA	NA	NA	NA
no	no	34	57
NA	NA	NA	NA
NA	NA	NA	NA
no	no	31	74
NA	NA	NA	NA
NA	NA	NA	NA
no	no	NA	NA
no	no	NA	NA
NA	NA	NA	NA
NA	NA	NA	NA
yes	Brother	NA	NA
yes	Sister	NA	NA
yes	Cousin	NA	NA
NA	NA	NA	NA
NA	NA	NA	NA
yes	Brother	NA	NA
yes	Brother	NA	NA
NA	NA	NA	NA
NA	NA	NA	NA
no	no	NA	NA
NA	NA	NA	NA
no	no	NA	NA
NA	NA	NA	NA
NA	NA	NA	NA
yes	Sister	35,5	54
NA	NA	NA	NA
NA	NA	NA	NA
yes	Brother	34	NA
no	no	21,5	73
NA	NA	NA	NA
no	no	35,5	53
NA	NA	NA	NA
NA	NA	NA	NA
no	no	36,5	44
NA	NA	NA	NA
NA	NA	NA	NA
no	no	NA	NA
NA	NA	NA	NA
NA	NA	NA	NA
NA	NA	NA	NA
yes	Sister	NA	NA
no	no	NA	NA

NA	NA	NA	NA
NA	NA	NA	NA
no	no	NA	NA
NA	NA	NA	NA
no	no	36	45
NA	NA	NA	NA
no	no	NA	NA
NA	NA	NA	NA
NA	NA	NA	NA
no	no	NA	NA
NA	NA	NA	NA
NA	NA	NA	NA
no	no	34,5	62
NA	NA	NA	NA
NA	NA	NA	NA
yes	Brother	NA	NA
yes	Brother	NA	NA
no	no	26	61
NA	NA	NA	NA
no	no	32,5	62
NA	NA	NA	NA
NA	NA	NA	NA
yes	Cousin	NA	NA
NA	NA	NA	NA
NA	NA	NA	NA
no	no	NA	NA
NA	NA	NA	NA
yes	Brother	NA	NA
NA	NA	NA	NA
NA	NA	NA	NA
yes	Cousin	33,5	NA
NA	NA	NA	NA
NA	NA	NA	NA
no	no	NA	NA
NA	NA	NA	NA
NA	NA	NA	NA
NA	NA	NA	NA
no	no	NA	NA
NA	NA	NA	NA
NA	NA	NA	NA
no	no	NA	NA
NA	NA	NA	NA
NA	NA	NA	NA
yes	Brother; cousins	NA	NA
NA	NA	NA	NA
NA	NA	NA	NA
yes	Brother; cousins	NA	NA
yes	Brother; cousins	NA	NA
yes	Brother; cousins	NA	NA
no	no	NA	NA
NA	NA	NA	NA
NA	NA	NA	NA

yes	Brother	NA	NA
NA	NA	NA	NA
NA	NA	NA	NA
no	no	NA	NA
NA	NA	NA	NA
NA	NA	NA	NA
no	no	NA	NA
NA	NA	NA	NA
NA	NA	NA	NA
no	no	NA	NA
NA	NA	NA	NA
NA	NA	NA	NA
no	no	27,5	62
NA	NA	NA	NA
NA	NA	NA	NA
no	no	NA	NA
NA	NA	NA	NA
no	no	NA	NA
NA	NA	NA	NA
NA	NA	NA	NA
no	no	34	61
NA	NA	NA	NA
no	no	NA	NA
NA	NA	NA	NA
no	no	NA	NA
NA	NA	NA	NA
NA	NA	NA	NA
yes	Brother	36	NA
no	no	37,5	NA
NA	NA	NA	NA
NA	NA	NA	NA
no	no	34,5	NA
NA	NA	NA	NA
no	no	32	NA
NA	NA	NA	NA
NA	NA	NA	NA
no	no	NA	NA
NA	NA	NA	NA
NA	NA	NA	NA
no	no	NA	NA
NA	NA	NA	NA
NA	NA	NA	NA
no	no	NA	NA
NA	NA	NA	NA
no	no	32	NA
NA	NA	NA	NA
no	no	NA	NA
NA	NA	NA	NA
no	no	29	NA
NA	NA	NA	NA
yes	Brother	30	NA
NA	NA	NA	NA

yes	Sister		38,5	NA
NA	NA	NA		NA
NA	NA	NA		NA
NA	NA		34	NA
no	no		38,5	NA
NA	NA	NA		NA
no	no		32,5	NA
NA	NA	NA		NA
no	no	NA		NA
NA	NA	NA		NA
NA	NA	NA		NA
no	no		30	NA
NA	NA	NA		NA
NA	NA	NA		NA
no	no		34,5	NA
NA	NA	NA		NA
no	no	NA		NA
NA	NA	NA		NA
no	no		30	NA
NA	NA	NA		NA
NA	NA	NA		NA
no	no	NA		NA
NA	NA	NA		NA
NA	NA	NA		NA
yes	Sister	NA		NA
yes	Brother	NA		NA
NA	NA	NA		NA
yes	Brother	NA		NA
no	no		35,5	NA
NA	NA	NA		NA
NA	NA	NA		NA
no	no		30,5	NA
NA	NA	NA		NA
yes	Sister		36	NA
NA	NA	NA		NA
NA	NA	NA		NA
NA	NA	NA		NA
no	no	NA		NA
no	no	NA		NA
NA	NA	NA		NA
NA	NA	NA		NA
yes	Brother		33	NA
NA	NA	NA		NA
NA	NA	NA		NA
no	no	NA		NA
NA	NA	NA		NA
no	no	NA		NA
yes	Brother	NA		NA
NA	NA	NA		NA
no	no		30,5	NA
NA	NA	NA		NA
NA	NA	NA		NA

no	no	NA	NA	
NA	NA	NA	NA	
NA	NA	NA	NA	
yes	Sister	NA	NA	
NA	NA	NA	NA	
no	no	NA	NA	
NA	NA	NA	NA	
NA	NA	NA	NA	
no	no	NA	NA	
NA	NA	NA	NA	
NA	NA	NA	NA	
no	no	NA	NA	
NA	NA	NA	NA	
NA	NA	NA	NA	
no	no	NA	NA	
NA	NA	NA	NA	
NA	NA	NA	NA	
no	no	NA	NA	
NA	NA	NA	NA	
NA	NA	NA	NA	
no	no	NA	NA	
NA	NA	NA	NA	
NA	NA	NA	NA	
no	no	NA	NA	
NA	NA	NA	NA	
NA	NA	NA	NA	
no	no	NA	NA	
NA	NA	NA	NA	
NA	NA	NA	NA	
no	no	NA	NA	
NA	NA	NA	NA	
NA	NA	NA	NA	
no	no	NA	NA	
NA	NA	NA	NA	
NA	NA	NA	NA	
no	no	NA	NA	
NA	NA	NA	NA	
yes	Son	NA	NA	
NA	NA	NA	NA	
yes	Mother	NA	NA	
no	no	NA	NA	
no	no	NA	NA	
NA	NA	NA	NA	
NA	NA	NA	NA	
no	no	31	NA	
NA	NA	NA	NA	
NA	NA	NA	NA	
no	no	35,5		52
NA	NA	NA	NA	
NA	NA	NA	NA	
yes	Father	34,5		50
NA	NA	NA	NA	

NA	NA	NA	NA
yes	Uncle; aunt	NA	NA
NA	NA	NA	NA
NA	NA	NA	NA
no	no	NA	NA
NA	NA	NA	NA
NA	NA	NA	NA
no	no	NA	NA
NA	NA	NA	NA
NA	NA	NA	NA
no	no	NA	NA
NA	NA	NA	NA
NA	NA	NA	NA
no	no	NA	NA
NA	NA	NA	NA
NA	NA	NA	NA
NA	NA	NA	NA
NA	NA	NA	NA
yes	Brother	NA	NA
no	no	31,5	65
NA	NA	NA	NA
NA	NA	NA	NA
no	no	30,5	82
NA	NA	NA	NA
NA	NA	NA	NA
yes	Uncle; aunt	29	59
NA	NA	NA	NA
NA	NA	NA	NA
yes	Cousin	NA	NA
NA	NA	NA	NA
NA	NA	NA	NA
no	no	NA	NA
NA	NA	NA	NA
NA	NA	NA	NA
no	no	NA	NA
NA	NA	NA	NA
NA	NA	NA	NA
no	no	29,5	NA
NA	NA	NA	NA
NA	NA	NA	NA
no	no	33,5	56
NA	NA	NA	NA
NA	NA	NA	NA
no	no	38	41
NA	NA	NA	NA
NA	NA	NA	NA
no	no	30	77
NA	NA	NA	NA
NA	NA	NA	NA
no	no	29	NA
NA	NA	NA	NA
NA	NA	NA	NA

no	no		32,5	NA	
NA	NA	NA		NA	
NA	NA	NA		NA	
no	no		37,5		32
NA	NA	NA		NA	
NA	NA	NA		NA	
no	no	NA		NA	
NA	NA	NA		NA	
NA	NA	NA		NA	
no	no		34,5		61
NA	NA	NA		NA	
NA	NA	NA		NA	
no	no		31	NA	
NA	NA	NA		NA	
NA	NA	NA		NA	
no	no		31		75
NA	NA	NA		NA	
NA	NA	NA		NA	
no	no	NA		NA	
NA	NA	NA		NA	
NA	NA	NA		NA	
no	no	NA		NA	
NA	NA	NA		NA	
NA	NA	NA		NA	
no	no	NA		NA	
NA	NA	NA		NA	
NA	NA	NA		NA	
no	no	NA		NA	
NA	NA	NA		NA	
NA	NA	NA		NA	
yes	Brother	NA		NA	
NA	NA	NA		NA	
NA	NA	NA		NA	
yes	Brother	NA		NA	
no	no		31,5	NA	
NA	NA	NA		NA	
NA	NA	NA		NA	
no	no		28		76
NA	NA	NA		NA	
NA	NA	NA		NA	
no	no		31		63
NA	NA	NA		NA	
NA	NA	NA		NA	
no	no		29		79
NA	NA	NA		NA	
NA	NA	NA		NA	
no	no		32		61
NA	NA	NA		NA	
NA	NA	NA		NA	
no	no	NA		NA	
NA	NA	NA		NA	
NA	NA	NA		NA	

no	no	32	44
NA	NA	NA	NA
NA	NA	NA	NA
no	no	NA	NA
NA	NA	NA	NA
NA	NA	NA	NA
no	no	33	NA
NA	NA	NA	NA
NA	NA	NA	NA
no	no	31	NA
NA	NA	NA	NA
NA	NA	NA	NA
no	no	NA	NA
NA	NA	NA	NA
NA	NA	NA	NA
no	no	NA	NA
NA	NA	34	71
NA	NA	NA	NA
NA	NA	NA	NA
yes	Brother	NA	NA
NA	NA	NA	NA
NA	NA	NA	NA
no	no	NA	NA
NA	NA	NA	NA
NA	NA	NA	NA
no	no	36	54
NA	NA	NA	NA
no	no	33,5	NA
NA	NA	NA	NA
NA	NA	NA	NA
no	no	37	48
NA	NA	NA	NA
NA	NA	NA	NA
no	no	NA	NA
NA	NA	NA	NA
NA	NA	NA	NA
no	no	NA	NA
NA	NA	NA	NA
NA	NA	NA	NA
no	no	NA	NA
NA	NA	NA	NA
NA	NA	NA	NA
no	no	31	60
NA	NA	NA	NA
NA	NA	NA	NA
no	no	NA	NA
NA	NA	NA	NA
NA	NA	NA	NA
no	no	NA	NA
NA	NA	NA	NA
NA	NA	NA	NA
no	no	35,5	59

NA	NA	NA	NA
NA	NA	NA	NA
no	no	31	NA
NA	NA	NA	NA
NA	NA	NA	NA
no	no	NA	NA
NA	NA	NA	NA
NA	NA	NA	NA
no	no	31,5	NA
NA	NA	NA	NA
NA	NA	NA	NA
NA	NA	NA	NA
NA	NA	NA	NA
NA	NA	NA	NA
yes	Uncle; aunt	NA	NA
NA	NA	NA	NA
NA	NA	NA	NA
no	no	32	52
NA	NA	NA	NA
NA	NA	NA	NA
no	no	NA	NA
NA	NA	NA	NA
NA	NA	NA	NA
no	no	34,5	47
NA	NA	NA	NA
NA	NA	NA	NA
NA	NA	31	57
NA	NA	NA	NA
NA	NA	NA	NA
no	no	28	53
NA	NA	NA	NA
NA	NA	NA	NA
NA	NA	NA	NA
NA	NA	NA	NA
NA	NA	NA	NA
no	no	NA	NA
NA	NA	NA	NA
NA	NA	NA	NA
no	no	NA	NA
NA	NA	NA	NA
NA	NA	NA	NA
no	no	NA	NA
NA	NA	NA	NA
NA	NA	NA	NA
no	no	34,5	49
NA	NA	NA	NA
NA	NA	NA	NA
yes	Brother	NA	NA
NA	NA	NA	NA
NA	NA	NA	NA
no	no	NA	NA
NA	NA	NA	NA

NA	NA	NA	NA
no	no	NA	NA
NA	NA	NA	NA
NA	NA	NA	NA
no	no	32	73
NA	NA	NA	NA
NA	NA	NA	NA
no	no	NA	NA
NA	NA	NA	NA
NA	NA	NA	NA
no	no	24,5	76
NA	NA	NA	NA
NA	NA	NA	NA
yes	Brother	NA	NA
NA	NA	NA	NA
NA	NA	NA	NA
no	no	NA	NA
NA	NA	NA	NA
NA	NA	NA	NA
no	no	55	NA
NA	NA	NA	NA
NA	NA	NA	NA
no	no	NA	NA
NA	NA	NA	NA
NA	NA	NA	NA
no	no	34	60
NA	NA	NA	NA
NA	NA	NA	NA
yes	Brother	NA	NA
NA	NA	NA	NA
NA	NA	NA	NA
no	no	NA	NA
NA	NA	NA	NA
NA	NA	NA	NA
no	no	32	NA
NA	NA	NA	NA
NA	NA	NA	NA
yes	Cousin	NA	NA
NA	NA	NA	NA
NA	NA	NA	NA
no	no	NA	NA
NA	NA	NA	NA
NA	NA	NA	NA
yes	Father	NA	NA
NA	NA	NA	NA
no	no	33	73
NA	NA	NA	NA
NA	NA	NA	NA
NA	NA	NA	NA
NA	NA	NA	NA
NA	NA	NA	NA
NA	NA	NA	NA

NA	NA	NA	NA	
NA	NA	NA	NA	
NA	NA	NA	NA	
NA	NA	NA	NA	
NA	NA	NA	NA	
NA	NA	NA	NA	
NA	NA	NA	NA	
NA	NA	NA	NA	
yes	Brother	NA	NA	
NA	NA	NA	NA	
NA	NA	NA	NA	
yes	Sister	NA	NA	
no	no	38,5	NA	
NA	NA	NA	NA	
NA	NA	NA	NA	
no	no	46	NA	
NA	NA	NA	NA	
NA	NA	NA	NA	
no	no	NA	NA	
no	no	NA	NA	
NA	NA	NA	NA	
NA	NA	NA	NA	
no	no	35,5		31
NA	NA	NA	NA	
NA	NA	NA	NA	
no	no	NA	NA	
NA	NA	NA	NA	
NA	NA	NA	NA	
no	no	NA	NA	
NA	NA	NA	NA	
NA	NA	NA	NA	
no	no	NA	NA	
NA	NA	NA	NA	
NA	NA	NA	NA	
no	no	NA	NA	
NA	NA	NA	NA	
NA	NA	NA	NA	
no	no	44	NA	
NA	NA	NA	NA	
NA	NA	NA	NA	
yes	Brother	NA	NA	
yes	Sister	NA	NA	
NA	NA	NA	NA	
no	no	NA	NA	
NA	NA	NA	NA	
NA	NA	NA	NA	
yes	Mother	NA	NA	
NA	NA	NA	NA	
no	no	NA	NA	
NA	NA	NA	NA	
NA	NA	NA	NA	
no	no	NA	NA	

NA	NA	NA	NA
NA	NA	NA	NA
no	no	NA	NA
NA	NA	NA	NA
NA	NA	NA	NA
no	no	NA	NA
NA	NA	NA	NA
NA	NA	NA	NA
no	no	NA	NA
NA	NA	NA	NA
NA	NA	NA	NA
no	no	NA	NA
NA	NA	NA	NA
NA	NA	NA	NA
NA	NA	NA	NA
no	no	NA	NA
NA	NA	NA	NA
NA	NA	NA	NA
NA	NA	NA	NA
NA	NA	NA	NA
NA	NA	NA	NA
no	no	NA	NA
NA	NA	NA	NA
NA	NA	NA	NA
no	no	NA	NA
NA	NA	NA	NA
NA	NA	NA	NA
no	no	NA	NA
NA	NA	NA	NA
yes	Brother	NA	NA
NA	NA	NA	NA
NA	NA	NA	NA
NA	NA	NA	NA
NA	NA	NA	NA
no	no	NA	NA
NA	NA	NA	NA
NA	NA	NA	NA
yes	Brother	NA	NA
NA	NA	NA	NA
NA	NA	NA	NA
yes	Brother	NA	NA
yes	Brother	NA	NA
NA	NA	NA	NA
yes	Cousin	NA	NA
NA	NA	NA	NA
NA	NA	NA	NA
yes	Cousin	NA	NA
NA	NA	NA	NA
NA	NA	NA	NA
NA	NA	NA	NA
NA	NA	NA	NA
NA	NA	NA	NA

no	no	NA	NA
NA	NA	NA	NA
NA	NA	NA	NA
no	no	NA	NA
NA	NA	NA	NA
NA	NA	NA	NA
NA	NA	NA	NA
NA	NA	NA	NA
NA	NA	NA	NA
no	no	NA	NA
NA	NA	NA	NA
NA	NA	NA	NA
no	no	NA	NA
NA	NA	NA	NA
NA	NA	NA	NA
no	no	NA	NA
NA	NA	NA	NA
NA	NA	NA	NA
yes	Brother	NA	NA
NA	NA	NA	NA
NA	NA	NA	NA
yes	Brother	NA	NA
no	no	NA	NA
NA	NA	NA	NA
NA	NA	NA	NA
yes	Cousin	NA	NA
NA	NA	NA	NA
yes	Cousin	NA	NA
NA	NA	NA	NA
yes	NA	NA	NA
NA	NA	NA	NA
NA	NA	NA	NA
yes	Brother	NA	NA
NA	NA	NA	NA
NA	NA	NA	NA
no	no	NA	NA
NA	NA	NA	NA
NA	NA	NA	NA
no	no	NA	NA
NA	NA	NA	NA
NA	NA	NA	NA
no	no	NA	NA
NA	NA	NA	NA
NA	NA	NA	NA
yes	Brother	NA	NA
NA	NA	NA	NA
NA	NA	NA	NA
yes	Brother	NA	NA
yes	Sister	NA	NA
NA	NA	NA	NA
NA	NA	NA	NA
yes	Sister	NA	NA

no	no	NA	NA
NA	NA	NA	NA
NA	NA	NA	NA
no	no	NA	NA
NA	NA	NA	NA
NA	NA	NA	NA
no	no	NA	NA
NA	NA	NA	NA
NA	NA	NA	NA
no	no	NA	NA
NA	NA	NA	NA
NA	NA	NA	NA
no	no	NA	NA
NA	NA	NA	NA
NA	NA	NA	NA
no	no	NA	NA
NA	NA	NA	NA
NA	NA	NA	NA
NA	NA	NA	NA
NA	NA	NA	NA
NA	NA	NA	NA
no	no	NA	NA
NA	NA	NA	NA
NA	NA	NA	NA
no	no	NA	NA
NA	NA	NA	NA
NA	NA	NA	NA
NA	NA	NA	NA
NA	NA	NA	NA
NA	NA	NA	NA
no	no	NA	NA
NA	NA	NA	NA
no	no	NA	NA
NA	NA	NA	NA
NA	NA	NA	NA
no	no	NA	NA
NA	NA	NA	NA
no	no	NA	NA
NA	NA	NA	NA
NA	NA	NA	NA
yes	Father; brother	NA	NA
NA	NA	NA	NA
NA	NA	NA	NA
no	no	NA	NA
NA	NA	NA	NA
NA	NA	NA	NA
no	no	NA	NA
NA	NA	NA	NA
NA	NA	NA	NA
no	no	NA	NA
NA	NA	NA	NA
NA	NA	NA	NA

yes	Brother	NA	NA
NA	NA	NA	NA
NA	NA	NA	NA
yes	Sister	NA	NA
yes	Brother	NA	NA
NA	NA	NA	NA
NA	NA	NA	NA
NA	NA	NA	NA
NA	NA	NA	NA
NA	NA	NA	NA
NA	NA	NA	NA
yes	Son	NA	NA
NA	NA	NA	NA
yes	Father; brother	NA	NA
yes	Father; brother	NA	NA
NA	NA	NA	NA
NA	NA	NA	NA
NA	NA	NA	NA
yes	Uncle; aunt	NA	NA
yes	Cousin	NA	NA
NA	NA	NA	NA
NA	NA	NA	NA
yes	Cousin	NA	NA
no	no	NA	NA
NA	NA	NA	NA
yes	Brother	NA	NA
NA	NA	NA	NA
NA	NA	NA	NA
yes	Brother	NA	NA
yes	Brother	NA	NA
no	no	NA	NA
NA	NA	NA	NA
NA	NA	NA	NA
no	no	NA	NA
NA	NA	NA	NA
NA	NA	NA	NA
no	no	NA	NA
NA	NA	NA	NA
NA	NA	NA	NA
no	no	NA	NA
NA	NA	NA	NA
NA	NA	NA	NA
no	no	NA	NA
NA	NA	NA	NA
NA	NA	NA	NA
no	no	NA	NA
NA	NA	NA	NA
NA	NA	NA	NA
no	no	NA	NA
NA	NA	NA	NA
NA	NA	NA	NA
no	no	NA	NA

NA	NA	NA	NA
NA	NA	NA	NA
no	no	NA	NA
NA	NA	NA	NA
NA	NA	NA	NA
yes	Brother	NA	NA
NA	NA	NA	NA
NA	NA	NA	NA
no	no	NA	NA
NA	NA	NA	NA
NA	NA	NA	NA
yes	Father; brother	NA	NA
NA	NA	NA	NA
yes	Son; daughter	NA	NA
yes	Father; sister	NA	NA
no	no	NA	NA
NA	NA	NA	NA
NA	NA	NA	NA
yes	Uncle; aunt	NA	NA
NA	NA	NA	NA
NA	NA	NA	NA
no	no	NA	NA
NA	NA	NA	NA
NA	NA	NA	NA
no	no	NA	NA
NA	NA	NA	NA
NA	NA	NA	NA
no	no	NA	NA
yes	Cousin	NA	NA
yes	Cousin	NA	NA
no	no	NA	NA
NA	NA	NA	NA
NA	NA	NA	NA
no	no	NA	NA
NA	NA	NA	NA
no	no	NA	NA
NA	NA	NA	NA
no	no	NA	NA
NA	NA	NA	NA
NA	NA	NA	NA
no	no	NA	NA
NA	NA	NA	NA
NA	NA	NA	NA
no	no	NA	NA
NA	NA	NA	NA
NA	NA	NA	NA
no	no	NA	NA
NA	NA	NA	NA
NA	NA	NA	NA
no	no	NA	NA
NA	NA	NA	NA
NA	NA	NA	NA

yes	Brother	NA	NA
NA	NA	NA	NA
NA	NA	NA	NA
yes	Brother	NA	NA
no	no	NA	NA
NA	NA	NA	NA
NA	NA	NA	NA
yes	Sister	NA	NA
NA	NA	NA	NA
NA	NA	NA	NA
no	no	NA	NA
NA	NA	NA	NA
NA	NA	NA	NA
no	no	NA	NA
NA	NA	NA	NA
NA	NA	NA	NA
NA	NA	NA	NA
NA	NA	NA	NA
NA	NA	NA	NA
NA	NA	NA	NA
NA	NA	NA	NA
NA	NA	NA	NA
NA	NA	NA	NA
NA	NA	NA	NA
NA	NA	NA	NA
NA	NA	NA	NA
NA	NA	NA	NA
NA	NA	NA	NA
NA	NA	NA	NA
NA	NA	NA	NA
NA	NA	NA	NA
NA	NA	NA	NA
NA	NA	NA	NA
NA	NA	NA	NA
NA	NA	NA	NA
NA	NA	NA	NA
NA	NA	NA	NA
NA	NA	NA	NA
NA	NA	NA	NA
NA	NA	NA	NA
NA	NA	NA	NA
NA	NA	NA	NA
NA	NA	NA	NA
NA	NA	NA	NA
NA	NA	NA	NA
NA	NA	NA	NA
NA	NA	NA	NA
NA	NA	NA	NA
NA	NA	NA	NA

karyotype	fragile-X	MLPA	aCGH	LP-WGS	CNV_analys	conclusive_	alteration
no	nl	no	no	nl	yes	no	NA
NA	NA	NA	NA	NA	no	NA	NA
NA	NA	NA	NA	NA	no	NA	NA
no	no	no	no	nl	yes	no	NA
NA	NA	NA	NA	NA	no	NA	NA
NA	NA	NA	NA	NA	no	NA	NA
no	nl	nl	no	nl	yes	no	NA
NA	NA	NA	NA	NA	no	NA	NA
NA	NA	NA	NA	NA	no	NA	NA
no	nl	no	no	nl	yes	no	NA
NA	NA	NA	NA	NA	no	NA	NA
NA	NA	NA	NA	NA	no	NA	NA
no	nl	no	no	nl	yes	no	NA
NA	NA	NA	NA	NA	no	NA	NA
NA	NA	NA	NA	NA	no	NA	NA
no	nl	no	no	nl	yes	no	NA
NA	NA	NA	NA	NA	no	NA	NA
NA	NA	NA	NA	NA	no	NA	NA
nl	nl	nl	nl	no	yes	no	NA
NA	NA	NA	NA	NA	no	NA	NA
NA	NA	NA	NA	NA	no	NA	NA
nl	nl	no	no	no	yes	no	NA
NA	NA	NA	NA	NA	no	NA	NA
NA	NA	NA	NA	NA	no	NA	NA
no	nl	no	no	no	yes	no	NA
NA	NA	NA	NA	NA	no	NA	NA
NA	NA	NA	NA	NA	no	NA	NA
nl	nl	no	no	no	yes	no	NA
NA	NA	NA	NA	NA	no	NA	NA
NA	NA	NA	NA	NA	no	NA	NA
no	no	nl	no	no	yes	no	NA
NA	NA	NA	NA	NA	no	NA	NA
NA	NA	NA	NA	NA	no	NA	NA
no	nl	nl	nl	no	yes	no	NA
NA	NA	NA	NA	NA	no	NA	NA
NA	NA	NA	NA	NA	no	NA	NA
nl	nl	no	nl	no	yes	no	NA
NA	NA	NA	NA	NA	no	NA	NA
NA	NA	NA	NA	NA	no	NA	NA
no	nl	nl	no	no	yes	no	NA
NA	NA	NA	NA	NA	no	NA	NA
NA	NA	NA	NA	NA	no	NA	NA
nl	nl	nl	no	no	yes	no	NA
NA	NA	NA	NA	NA	no	NA	NA
NA	NA	NA	NA	NA	no	NA	NA
nl	nl	no	nl	no	yes	no	NA
NA	NA	NA	NA	NA	no	NA	NA
NA	NA	NA	NA	NA	no	NA	NA
nl	nl	nl	nl	no	yes	no	NA
NA	NA	NA	NA	NA	no	NA	NA

NA	NA	NA	NA	NA	no	NA	NA
no	no	nl	nl	no	yes	no	NA
NA	NA	NA	NA	NA	no	NA	NA
NA	NA	NA	NA	NA	no	NA	NA
NA	NA	NA	NA	NA	no	NA	NA
NA	NA	NA	NA	NA	no	NA	NA
no	no	nl	no	nl	yes	no	NA
no	nl	no	no	no	yes	no	NA
NA	NA	NA	NA	NA	no	NA	NA
NA	NA	NA	NA	NA	no	NA	NA
nl	nl	nl	no	no	yes	no	NA
NA	NA	NA	NA	NA	no	NA	NA
NA	NA	NA	NA	NA	no	NA	NA
nl	no	nl	no	no	yes	no	NA
NA	NA	NA	NA	NA	no	NA	NA
NA	NA	NA	NA	NA	no	NA	NA
nl	no	no	no	no	yes	no	NA
NA	NA	NA	NA	NA	no	NA	NA
NA	NA	NA	NA	NA	no	NA	NA
nl	nl	nl	no	no	yes	no	NA
NA	NA	NA	NA	NA	no	NA	NA
NA	NA	NA	NA	NA	no	NA	NA
no	nl	no	no	no	yes	no	NA
NA	NA	NA	NA	NA	no	NA	NA
NA	NA	NA	NA	NA	no	NA	NA
nl	nl	nl	no	no	yes	no	NA
NA	NA	NA	NA	NA	no	NA	NA
NA	NA	NA	NA	NA	no	NA	NA
no	no	no	no	no	yes	no	NA
NA	NA	NA	NA	NA	no	NA	NA
NA	NA	NA	NA	NA	no	NA	NA
nl	nl	no	no	no	yes	no	NA
NA	NA	NA	NA	NA	no	NA	NA
NA	NA	NA	NA	NA	no	NA	NA
no	nl	no	no	no	yes	no	NA
NA	NA	NA	NA	NA	no	NA	NA
NA	NA	NA	NA	NA	no	NA	NA
no	nl	no	no	no	yes	no	NA
NA	NA	NA	NA	NA	no	NA	NA
NA	NA	NA	NA	NA	no	NA	NA
nl	no	no	no	no	yes	no	NA
NA	NA	NA	NA	NA	no	NA	NA
NA	NA	NA	NA	NA	no	NA	NA
nl	nl	no	no	no	yes	no	NA
NA	NA	NA	NA	NA	no	NA	NA
NA	NA	NA	NA	NA	no	NA	NA
nl	no	no	no	no	yes	no	NA
NA	NA	NA	NA	NA	no	NA	NA
NA	NA	NA	NA	NA	no	NA	NA
nl	nl	nl	no	no	yes	no	NA
NA	NA	NA	NA	NA	no	NA	NA
NA	NA	NA	NA	NA	no	NA	NA

no	nl	no	no	no	yes	no	NA
NA	NA	NA	NA	NA	no	NA	NA
NA	NA	NA	NA	NA	no	NA	NA
nl	nl	no	no	no	yes	no	NA
NA	NA	NA	NA	NA	no	NA	NA
NA	NA	NA	NA	NA	no	NA	NA
no	no	nl	no	no	yes	no	NA
NA	NA	NA	NA	NA	no	NA	NA
NA	NA	NA	NA	NA	no	NA	NA
no	nl	no	LP	no	yes	yes, monog	22q11.21du
NA	NA	NA	NA	NA	no	NA	NA
NA	NA	NA	NA	NA	no	NA	NA
nl	no	no	nl	no	yes	no	NA
NA	NA	NA	NA	NA	no	NA	NA
NA	NA	NA	NA	NA	no	NA	NA
nl	nl	no	no	no	yes	no	NA
NA	NA	NA	NA	NA	no	NA	NA
NA	NA	NA	NA	NA	no	NA	NA
no	nl	no	no	no	yes	no	NA
NA	NA	NA	NA	NA	no	NA	NA
NA	NA	NA	NA	NA	no	NA	NA
no	no	no	nl	no	yes	no	NA
NA	NA	NA	NA	NA	no	NA	NA
NA	NA	NA	NA	NA	no	NA	NA
nl	nl	no	no	no	yes	no	NA
NA	NA	NA	NA	NA	no	NA	NA
NA	NA	NA	NA	NA	no	NA	NA
no	nl	no	no	no	yes	no	NA
NA	NA	NA	NA	NA	no	NA	NA
NA	NA	NA	NA	NA	no	NA	NA
nl	no	no	no	no	yes	no	NA
NA	NA	NA	NA	NA	no	NA	NA
NA	NA	NA	NA	NA	no	NA	NA
no	nl	no	no	no	yes	no	NA
NA	NA	NA	NA	NA	no	NA	NA
NA	NA	NA	NA	NA	no	NA	NA
no	nl	no	no	no	yes	no	NA
NA	NA	NA	NA	NA	no	NA	NA
NA	NA	NA	NA	NA	no	NA	NA
nl	nl	no	no	no	yes	no	NA
NA	NA	NA	NA	NA	no	NA	NA
NA	NA	NA	NA	NA	no	NA	NA
no	no	no	no	no	yes	no	NA
NA	NA	NA	NA	NA	no	NA	NA
NA	NA	NA	NA	NA	no	NA	NA
no	no	no	nl	no	yes	no	NA
NA	NA	NA	NA	NA	no	NA	NA
NA	NA	NA	NA	NA	no	NA	NA
NA	NA	NA	NA	NA	no	NA	NA
NA	NA	NA	NA	NA	no	NA	NA
NA	NA	NA	NA	NA	no	NA	NA
no	no	no	no	nl	yes	no	NA

NA	NA	NA	NA	NA	no	NA	NA
NA	NA	NA	NA	NA	no	NA	NA
no	no	no	no	no	yes	no	NA
NA	NA	NA	NA	NA	no	NA	NA
NA	NA	NA	NA	NA	no	NA	NA
NA	NA	NA	NA	NA	no	NA	NA
NA	NA	NA	NA	NA	no	NA	NA
no	no	no	no	P	yes	yes, ASD su	Ydup
no	no	no	no	no	yes	no	NA
NA	NA	NA	NA	NA	no	NA	NA
NA	NA	NA	NA	NA	no	NA	NA
no	no	no	no	no	yes	no	NA
NA	NA	NA	NA	NA	no	NA	NA
NA	NA	NA	NA	NA	no	NA	NA
NA	NA	NA	NA	NA	no	NA	NA
NA	NA	NA	NA	NA	no	NA	NA
NA	NA	NA	NA	NA	no	NA	NA
NA	NA	NA	NA	NA	no	NA	NA
NA	NA	NA	NA	NA	no	NA	NA
NA	NA	NA	NA	NA	no	NA	NA
NA	NA	NA	NA	NA	no	NA	NA
NA	NA	NA	NA	NA	no	NA	NA
NA	NA	NA	NA	NA	no	NA	NA
NA	NA	NA	NA	NA	no	NA	NA
NA	NA	NA	NA	NA	no	NA	NA
NA	NA	NA	NA	NA	no	NA	NA
NA	NA	NA	NA	NA	no	NA	NA
NA	NA	NA	NA	NA	no	NA	NA
NA	NA	NA	NA	NA	no	NA	NA
NA	NA	NA	NA	NA	no	NA	NA
NA	NA	NA	NA	NA	no	NA	NA
NA	NA	NA	NA	NA	no	NA	NA
NA	NA	NA	NA	NA	no	NA	NA
NA	NA	NA	NA	NA	no	NA	NA
NA	NA	NA	NA	NA	no	NA	NA
NA	NA	NA	NA	NA	no	NA	NA
NA	NA	NA	NA	NA	no	NA	NA
NA	NA	NA	NA	NA	no	NA	NA
NA	NA	NA	NA	NA	no	NA	NA
NA	NA	NA	NA	NA	no	NA	NA
NA	NA	NA	NA	NA	no	NA	NA
NA	NA	NA	NA	NA	no	NA	NA
NA	NA	NA	NA	NA	no	NA	NA
NA	NA	NA	NA	NA	no	NA	NA
NA	NA	NA	NA	NA	no	NA	NA
NA	NA	NA	NA	NA	no	NA	NA
NA	NA	NA	NA	NA	no	NA	NA
NA	NA	NA	NA	NA	no	NA	NA
NA	NA	NA	NA	NA	no	NA	NA
NA	NA	NA	NA	NA	no	NA	NA
NA	NA	NA	NA	NA	no	NA	NA
NA	NA	NA	NA	NA	no	NA	NA

NA	NA	NA	NA	NA	no	NA	NA
NA	NA	NA	NA	NA	no	NA	NA
NA	NA	NA	NA	NA	no	NA	NA
NA	NA	NA	NA	NA	no	NA	NA
NA	NA	NA	NA	NA	no	NA	NA
NA	NA	NA	NA	NA	no	NA	NA
NA	NA	NA	NA	NA	no	NA	NA
NA	NA	NA	NA	NA	no	NA	NA
NA	NA	NA	NA	NA	no	NA	NA
NA	NA	NA	NA	NA	no	NA	NA
NA	NA	NA	NA	NA	no	NA	NA
NA	NA	NA	NA	NA	no	NA	NA
NA	NA	NA	NA	NA	no	NA	NA
NA	NA	NA	NA	NA	no	NA	NA
NA	NA	NA	NA	NA	no	NA	NA
NA	NA	NA	NA	NA	no	NA	NA
NA	NA	NA	NA	NA	no	NA	NA
NA	NA	NA	NA	NA	no	NA	NA
NA	NA	NA	NA	NA	no	NA	NA
NA	NA	NA	NA	NA	no	NA	NA
NA	NA	NA	NA	NA	no	NA	NA
NA	NA	NA	NA	NA	no	NA	NA
NA	NA	NA	NA	NA	no	NA	NA
NA	NA	NA	NA	NA	no	NA	NA
NA	NA	NA	NA	NA	no	NA	NA
NA	NA	nl	NA	NA	yes	NA	NA
NA	NA	NA	NA	NA	no	NA	NA
NA	NA	NA	NA	NA	no	NA	NA
NA	NA	NA	NA	NA	no	NA	NA
NA	NA	NA	NA	NA	no	NA	NA
NA	NA	NA	NA	NA	no	NA	NA
NA	NA	NA	NA	NA	no	NA	NA
NA	NA	NA	NA	NA	no	NA	NA
NA	NA	nl	NA	NA	yes	NA	NA
NA	NA	NA	NA	NA	no	NA	NA
NA	NA	NA	NA	NA	no	NA	NA
NA	NA	NA	NA	NA	no	NA	NA
NA	NA	NA	NA	NA	no	NA	NA
NA	NA	no	NA	NA	yes	NA	NA
NA	NA	NA	NA	NA	no	NA	NA
NA	NA	NA	NA	NA	no	NA	NA
no	nl	no	no	nl	yes	no	NA
NA	NA	NA	NA	NA	no	NA	NA
NA	NA	NA	NA	NA	no	NA	NA
no	nl	no	no	nl	yes	no	NA
NA	NA	NA	NA	NA	no	NA	NA
NA	NA	NA	NA	NA	no	NA	NA
no	nl	no	no	nl	yes	no	NA
NA	NA	NA	NA	NA	no	NA	NA
NA	NA	NA	NA	NA	no	NA	NA
nl	nl	nl	nl	no	yes	no	NA
NA	NA	NA	NA	NA	no	NA	NA

NA	NA	NA	NA	NA	no	NA	NA
no	nl	no	no	nl	yes	no	NA
NA	NA	NA	NA	NA	no	NA	NA
NA	NA	NA	NA	NA	no	NA	NA
no	nl	no	no	nl	yes	no	NA
NA	NA	NA	NA	NA	no	NA	NA
NA	NA	NA	NA	NA	no	NA	NA
no	nl	nl	no	nl	yes	no	NA
NA	NA	NA	NA	NA	no	NA	NA
NA	NA	NA	NA	NA	no	NA	NA
nl	nl	no	nl	nl	yes	no	NA
NA	NA	NA	NA	NA	no	NA	NA
NA	NA	NA	NA	NA	no	NA	NA
no	nl	no	no	nl	yes	no	NA
NA	NA	NA	NA	NA	no	NA	NA
NA	NA	NA	NA	NA	no	NA	NA
no	no	no	no	nl	yes	no	NA
NA	NA	NA	NA	NA	no	NA	NA
NA	NA	NA	NA	NA	no	NA	NA
no	nl	nl	no	nl	yes	no	NA
NA	NA	NA	NA	NA	no	NA	NA
NA	NA	NA	NA	NA	no	NA	NA
nl	nl	no	nl	nl	yes	no	NA
NA	NA	NA	NA	NA	no	NA	NA
NA	NA	NA	NA	NA	no	NA	NA
nl	nl	no	no	nl	yes	no	NA
NA	NA	NA	NA	NA	no	NA	NA
NA	NA	NA	NA	NA	no	NA	NA
nl	nl	no	no	nl	yes	no	NA
NA	NA	NA	NA	NA	no	NA	NA
NA	NA	NA	NA	NA	no	NA	NA
no	nl	no	no	nl	yes	no	NA
NA	NA	NA	NA	NA	no	NA	NA
NA	NA	NA	NA	NA	no	NA	NA
no	nl	nl	no	nl	yes	no	NA
NA	NA	NA	NA	NA	no	NA	NA
NA	NA	NA	NA	NA	no	NA	NA
nl	nl	no	no	no	yes	no	NA
NA	NA	NA	NA	NA	no	NA	NA
NA	NA	NA	NA	NA	no	NA	NA
nl	nl	no	no	nl	yes	no	NA
NA	NA	NA	NA	NA	no	NA	NA
NA	NA	NA	NA	NA	no	NA	NA
nl	nl	no	no	nl	yes	no	NA
NA	NA	NA	NA	NA	no	NA	NA
NA	NA	NA	NA	NA	no	NA	NA
nl	nl	no	no	nl	yes	no	NA
NA	NA	NA	NA	NA	no	NA	NA
NA	NA	NA	NA	NA	no	NA	NA
nl	no	nl	no	no	yes	no	NA
NA	NA	NA	NA	NA	no	NA	NA
NA	NA	NA	NA	NA	no	NA	NA

nl	nl	no	no	nl	yes	no	NA
NA	NA	NA	NA	NA	no	NA	NA
nl	nl	no	no	nl	yes	no	NA
NA	NA	NA	NA	NA	no	NA	NA
NA	NA	NA	NA	NA	no	NA	NA
no	no	no	no	nl	yes	no	NA
NA	NA	NA	NA	NA	no	NA	NA
NA	NA	NA	NA	NA	no	NA	NA
no	nl	no	no	nl	yes	no	NA
NA	NA	NA	NA	NA	no	NA	NA
NA	NA	NA	NA	NA	no	NA	NA
no	nl	no	no	nl	yes	no	NA
NA	NA	NA	NA	NA	no	NA	NA
NA	NA	NA	NA	NA	no	NA	NA
no	nl	no	no	nl	yes	no	NA
NA	NA	NA	NA	NA	no	NA	NA
NA	NA	NA	NA	NA	no	NA	NA
nl	nl	no	no	nl	yes	no	NA
NA	NA	NA	NA	NA	no	NA	NA
NA	NA	NA	NA	NA	no	NA	NA
no	nl	no	no	nl	yes	no	NA
NA	NA	NA	NA	NA	no	NA	NA
NA	NA	NA	NA	NA	no	NA	NA
no	no	nl	no	nl	yes	no	NA
NA	NA	NA	NA	NA	no	NA	NA
NA	NA	NA	NA	NA	no	NA	NA
no	nl	nl	nl	no	yes	no	NA
NA	NA	NA	NA	NA	no	NA	NA
NA	NA	NA	NA	NA	no	NA	NA
no	nl	nl	no	nl	yes	no	NA
NA	NA	NA	NA	NA	no	NA	NA
NA	NA	NA	NA	NA	no	NA	NA
nl	nl	no	no	nl	yes	no	NA
NA	NA	NA	NA	NA	no	NA	NA
nl	nl	no	no	nl	yes	no	NA
NA	NA	NA	NA	NA	no	NA	NA
NA	NA	NA	NA	NA	no	NA	NA
nl	nl	no	nl	nl	yes	no	NA
NA	NA	NA	NA	NA	no	NA	NA
NA	NA	NA	NA	NA	no	NA	NA
nl	nl	no	nl	no	yes	no	NA
NA	NA	NA	NA	NA	no	NA	NA
NA	NA	NA	NA	NA	no	NA	NA
no	no	no	nl	nl	yes	no	NA
NA	NA	NA	NA	NA	no	NA	NA
NA	NA	NA	NA	NA	no	NA	NA
nl	nl	no	no	nl	yes	no	NA
NA	NA	NA	NA	NA	no	NA	NA
NA	NA	NA	NA	NA	no	NA	NA
no	nl	no	no	nl	yes	no	NA
NA	NA	NA	NA	NA	no	NA	NA
NA	NA	NA	NA	NA	no	NA	NA

nl	nl	no	no	nl	yes	no	NA
NA	NA	NA	NA	NA	no	NA	NA
NA	NA	NA	NA	NA	no	NA	NA
no	nl	no	no	nl	yes	no	NA
NA	NA	NA	NA	NA	no	NA	NA
NA	NA	NA	NA	NA	no	NA	NA
no	nl	no	no	nl	yes	no	NA
NA	NA	NA	NA	NA	no	NA	NA
NA	NA	NA	NA	NA	no	NA	NA
no	nl	no	no	nl	yes	no	NA
NA	NA	NA	NA	NA	no	NA	NA
NA	NA	NA	NA	NA	no	NA	NA
nl	nl	no	no	no	yes	no	NA
NA	NA	NA	NA	NA	no	NA	NA
NA	NA	NA	NA	NA	no	NA	NA
no	nl	no	no	nl	yes	no	NA
NA	NA	NA	NA	NA	no	NA	NA
NA	NA	NA	NA	NA	no	NA	NA
no	nl	nl	no	nl	yes	no	NA
NA	NA	NA	NA	NA	no	NA	NA
NA	NA	NA	NA	NA	no	NA	NA
no	nl	nl	no	nl	yes	no	NA
NA	NA	NA	NA	NA	no	NA	NA
NA	NA	NA	NA	NA	no	NA	NA
no	no	no	no	no	yes	no	NA
NA	NA	NA	NA	NA	no	NA	NA
NA	NA	NA	NA	NA	no	NA	NA
no	nl	no	no	nl	yes	no	NA
NA	NA	NA	NA	NA	no	NA	NA
NA	NA	NA	NA	NA	no	NA	NA
nl	nl	no	no	no	yes	no	NA
NA	NA	NA	NA	NA	no	NA	NA
NA	NA	NA	NA	NA	no	NA	NA
no	nl	no	no	nl	yes	no	NA
NA	NA	NA	NA	NA	no	NA	NA
NA	NA	NA	NA	NA	no	NA	NA
no	no	no	no	nl	yes	no	NA
NA	NA	NA	NA	NA	no	NA	NA
NA	NA	NA	NA	NA	no	NA	NA
no	no	no	no	nl	yes	no	NA
NA	NA	NA	NA	NA	no	NA	NA
NA	NA	NA	NA	NA	no	NA	NA
no	nl	nl	no	nl	yes	no	NA
NA	NA	NA	NA	NA	no	NA	NA
NA	NA	NA	NA	NA	no	NA	NA
no	nl	no	no	no	yes	no	NA
NA	NA	NA	NA	NA	no	NA	NA
nl	nl	no	no	nl	yes	no	NA
NA	NA	NA	NA	NA	no	NA	NA
NA	NA	NA	NA	NA	no	NA	NA
nl	nl	no	no	nl	yes	no	NA
NA	NA	NA	NA	NA	no	NA	NA

NA	NA	NA	NA	NA	no	NA	NA
no	nl	no	no	nl	yes	no	NA
NA	NA	NA	NA	NA	no	NA	NA
NA	NA	NA	NA	NA	no	NA	NA
nl	no	nl	no	nl	yes	no	NA
NA	NA	NA	NA	NA	no	NA	NA
NA	NA	NA	NA	NA	no	NA	NA
no	nl	no	no	nl	yes	no	NA
NA	NA	NA	NA	NA	no	NA	NA
NA	NA	NA	NA	NA	no	NA	NA
no	nl	nl	no	nl	yes	no	NA
NA	NA	NA	NA	NA	no	NA	NA
NA	NA	NA	NA	NA	no	NA	NA
nl	nl	no	no	P	yes	yes, monog	1q21.1dup
NA	NA	NA	NA	NA	no	NA	NA
NA	NA	NA	NA	NA	no	NA	NA
NA	NA	P	NA	NA	yes	yes, ASD su	15q11-13.3
NA	NA	no	NA	NA	yes	NA	NA
NA	NA	NA	NA	NA	no	NA	NA
NA	NA	NA	NA	NA	no	NA	NA
NA	NA	NA	NA	NA	no	NA	NA
NA	NA	NA	NA	NA	no	NA	NA
NA	NA	nl	NA	NA	yes	NA	NA
NA	NA	NA	NA	NA	no	NA	NA
NA	NA	NA	NA	NA	no	NA	NA
NA	NA	NA	NA	NA	no	NA	NA
NA	NA	NA	NA	NA	no	NA	NA
NA	NA	NA	NA	NA	no	NA	NA
NA	NA	NA	NA	NA	no	NA	NA
nl	nl	no	no	nl	yes	no	NA
NA	NA	NA	NA	NA	no	NA	NA
no	nl	nl	no	nl	yes	no	NA
NA	NA	NA	NA	NA	no	NA	NA
NA	NA	NA	NA	NA	no	NA	NA
nl	nl	nl	no	nl	yes	no	NA
NA	NA	NA	NA	NA	no	NA	NA
NA	NA	NA	NA	NA	no	NA	NA
no	no	no	no	no	yes	no	NA
nl	nl	no	nl	nl	yes	no	NA
NA	NA	NA	NA	NA	no	NA	NA
nl	nl	nl	nl	no	yes	no	NA
NA	NA	NA	NA	NA	no	NA	NA
NA	NA	NA	NA	NA	no	NA	NA
nl	no	no	no	nl	yes	no	NA
NA	NA	NA	NA	NA	no	NA	NA
NA	NA	NA	NA	NA	no	NA	NA
nl	nl	nl	no	nl	yes	no	NA
NA	NA	NA	NA	NA	no	NA	NA
NA	NA	NA	NA	NA	no	NA	NA
NA	NA	NA	NA	NA	no	NA	NA
nl	no	no	no	no	yes	no	NA
no	no	nl	no	nl	yes	no	NA

NA	NA	NA	NA	NA	no	NA	NA
NA	NA	NA	NA	NA	no	NA	NA
nl	nl	no	no	nl	yes	no	NA
NA	NA	NA	NA	NA	no	NA	NA
nl	nl	no	nl	no	yes	no	NA
NA	NA	NA	NA	NA	no	NA	NA
no	nl	nl	no	nl	yes	no	NA
NA	NA	NA	NA	NA	no	NA	NA
NA	NA	NA	NA	NA	no	NA	NA
no	no	nl	nl	no	yes	no	NA
NA	NA	NA	NA	NA	no	NA	NA
NA	NA	NA	NA	NA	no	NA	NA
no	nl	no	no	nl	yes	no	NA
NA	NA	NA	NA	NA	no	NA	NA
NA	NA	NA	NA	NA	no	NA	NA
nl	nl	no	no	no	yes	no	NA
no	no	no	no	nl	yes	no	NA
no	nl	no	no	nl	yes	no	NA
NA	NA	NA	NA	NA	no	NA	NA
no	no	no	no	nl	yes	no	NA
NA	NA	NA	NA	NA	no	NA	NA
NA	NA	NA	NA	NA	no	NA	NA
no	no	no	no	no	yes	no	NA
NA	NA	NA	NA	NA	no	NA	NA
NA	NA	NA	NA	NA	no	NA	NA
no	nl	no	no	nl	yes	no	NA
NA	NA	NA	NA	NA	no	NA	NA
no	no	no	no	nl	yes	no	NA
NA	NA	NA	NA	NA	no	NA	NA
NA	NA	NA	NA	NA	no	NA	NA
no	no	no	no	no	yes	no	NA
NA	NA	NA	NA	NA	no	NA	NA
NA	NA	NA	NA	NA	no	NA	NA
nl	no	nl	no	nl	yes	no	NA
NA	NA	NA	NA	NA	no	NA	NA
NA	NA	NA	NA	NA	no	NA	NA
NA	NA	NA	NA	NA	no	NA	NA
no	nl	no	no	nl	yes	no	NA
NA	NA	NA	NA	NA	no	NA	NA
NA	NA	NA	NA	NA	no	NA	NA
no	nl	nl	no	nl	yes	no	NA
NA	NA	NA	NA	NA	no	NA	NA
NA	NA	NA	NA	NA	no	NA	NA
no	nl	no	no	nl	yes	no	NA
NA	NA	NA	NA	NA	no	NA	NA
NA	NA	NA	NA	NA	no	NA	NA
no	nl	no	no	no	yes	no	NA
no	nl	no	no	no	yes	no	NA
no	nl	no	no	no	yes	no	NA
no	no	no	no	nl	yes	no	NA
NA	NA	NA	NA	NA	no	NA	NA
NA	NA	NA	NA	NA	no	NA	NA

nl	no	nl	no	no	yes	no	NA
NA	NA	NA	NA	NA	no	NA	NA
NA	NA	NA	NA	NA	no	NA	NA
nl	nl	no	nl	nl	yes	no	NA
NA	NA	NA	NA	NA	no	NA	NA
NA	NA	NA	NA	NA	no	NA	NA
no	nl	nl	no	nl	yes	no	NA
NA	NA	NA	NA	NA	no	NA	NA
NA	NA	NA	NA	NA	no	NA	NA
nl	gray zone	no	nl	nl	yes	no	NA
NA	NA	NA	NA	NA	no	NA	NA
NA	NA	NA	NA	NA	no	NA	NA
no	no	no	no	nl	yes	no	NA
NA	NA	NA	NA	NA	no	NA	NA
NA	NA	NA	NA	NA	no	NA	NA
nl	nl	nl	no	no	yes	no	NA
NA	NA	NA	NA	NA	no	NA	NA
no	no	no	nl	nl	yes	no	NA
NA	NA	NA	NA	NA	no	NA	NA
NA	NA	NA	NA	NA	no	NA	NA
nl	no	nl	no	nl	yes	no	NA
NA	NA	NA	NA	NA	no	NA	NA
no	nl	no	no	nl	yes	no	NA
NA	NA	NA	NA	NA	no	NA	NA
no	no	no	no	nl	yes	no	NA
NA	NA	NA	NA	NA	no	NA	NA
NA	NA	NA	NA	NA	no	NA	NA
no	nl	no	no	no	yes	no	NA
nl	no	no	no	nl	yes	no	NA
NA	NA	NA	NA	NA	no	NA	NA
NA	NA	NA	NA	NA	no	NA	NA
no	nl	no	no	no	yes	no	NA
NA	NA	NA	NA	NA	no	NA	NA
no	nl	no	no	nl	yes	no	NA
NA	NA	NA	NA	NA	no	NA	NA
NA	NA	NA	NA	NA	no	NA	NA
no	no	no	no	nl	yes	no	NA
NA	NA	NA	NA	NA	no	NA	NA
NA	NA	NA	NA	NA	no	NA	NA
no	no	no	nl	nl	yes	no	NA
NA	NA	NA	NA	NA	no	NA	NA
NA	NA	NA	NA	NA	no	NA	NA
no	nl	no	no	nl	yes	no	NA
NA	NA	NA	NA	NA	no	NA	NA
no	no	no	no	nl	yes	no	NA
NA	NA	NA	NA	NA	no	NA	NA
nl	nl	no	no	nl	yes	no	NA
NA	NA	NA	NA	NA	no	NA	NA
no	nl	no	no	nl	yes	no	NA
NA	NA	NA	NA	NA	no	NA	NA
no	nl	no	no	nl	yes	no	NA
NA	NA	NA	NA	NA	no	NA	NA

nl	nl	no	no	nl	yes	no	NA
NA	NA	NA	NA	NA	no	NA	NA
NA	NA	NA	NA	NA	no	NA	NA
no	nl	no	no	nl	yes	no	NA
no	nl	no	no	nl	yes	no	NA
NA	NA	NA	NA	NA	no	NA	NA
no	no	no	no	nl	yes	no	NA
NA	NA	NA	NA	NA	no	NA	NA
no	nl	no	nl	nl	yes	no	NA
NA	NA	NA	NA	NA	no	NA	NA
NA	NA	NA	NA	NA	no	NA	NA
no	no	no	no	no	yes	no	NA
NA	NA	NA	NA	NA	no	NA	NA
NA	NA	NA	NA	NA	no	NA	NA
nl	nl	no	no	nl	yes	no	NA
NA	NA	NA	NA	NA	no	NA	NA
nl	nl	no	nl	nl	yes	no	NA
NA	NA	NA	NA	NA	no	NA	NA
nl	no	no	nl	no	yes	no	NA
NA	NA	NA	NA	NA	no	NA	NA
NA	NA	NA	NA	NA	no	NA	NA
nl	nl	no	LP	LP	yes	yes, ASD su	16p12.2-11
NA	NA	NA	NA	NA	no	NA	NA
NA	NA	NA	NA	NA	no	NA	NA
no	no	no	no	nl	yes	no	NA
no	no	no	no	nl	yes	no	NA
NA	NA	NA	NA	NA	no	NA	NA
no	no	no	no	no	yes	no	NA
no	nl	no	no	nl	yes	no	NA
NA	NA	NA	NA	NA	no	NA	NA
NA	NA	NA	NA	NA	no	NA	NA
nl	nl	no	no	nl	yes	no	NA
NA	NA	NA	NA	NA	no	NA	NA
no	no	no	no	nl	yes	no	NA
NA	NA	NA	NA	NA	no	NA	NA
NA	NA	NA	NA	NA	no	NA	NA
NA	NA	NA	NA	NA	no	NA	NA
nl	nl	no	nl	no	yes	no	NA
no	no	no	no	nl	yes	no	NA
NA	NA	NA	NA	NA	no	NA	NA
NA	NA	NA	NA	NA	no	NA	NA
no	no	no	no	nl	yes	no	NA
NA	NA	NA	NA	NA	no	NA	NA
NA	NA	NA	NA	NA	no	NA	NA
no	no	no	no	nl	yes	no	NA
NA	NA	NA	NA	NA	no	NA	NA
no	no	no	no	nl	yes	no	NA
no	no	no	no	nl	yes	no	NA
NA	NA	NA	NA	NA	no	NA	NA
no	no	no	no	no	yes	no	NA
NA	NA	NA	NA	NA	no	NA	NA
NA	NA	NA	NA	NA	no	NA	NA

no	no	no	no	nl	yes	no	NA
NA	NA	NA	NA	NA	no	NA	NA
NA	NA	NA	NA	NA	no	NA	NA
no	no	no	no	nl	yes	no	NA
NA	NA	NA	NA	NA	no	NA	NA
no	no	nl	no	nl	yes	no	NA
NA	NA	NA	NA	NA	no	NA	NA
NA	NA	NA	NA	NA	no	NA	NA
no	nl	no	no	nl	yes	no	NA
NA	NA	NA	NA	NA	no	NA	NA
NA	NA	NA	NA	NA	no	NA	NA
nl	no	no	no	nl	yes	no	NA
NA	NA	NA	NA	NA	no	NA	NA
NA	NA	NA	NA	NA	no	NA	NA
nl	nl	no	nl	nl	yes	no	NA
NA	NA	NA	NA	NA	no	NA	NA
NA	NA	NA	NA	NA	no	NA	NA
no	no	no	no	nl	yes	no	NA
NA	NA	NA	NA	NA	no	NA	NA
NA	NA	NA	NA	NA	no	NA	NA
nl	nl	no	no	nl	yes	no	NA
NA	NA	NA	NA	NA	no	NA	NA
NA	NA	NA	NA	NA	no	NA	NA
no	no	no	no	P	yes	yes, monog	Xp22.33p22
NA	NA	NA	NA	NA	no	NA	NA
NA	NA	NA	NA	NA	no	NA	NA
no	no	no	no	nl	yes	no	NA
NA	NA	NA	NA	NA	no	NA	NA
NA	NA	NA	NA	NA	no	NA	NA
no	nl	no	no	nl	yes	no	NA
NA	NA	NA	NA	NA	no	NA	NA
NA	NA	NA	NA	NA	no	NA	NA
no	no	no	no	nl	yes	no	NA
NA	NA	NA	NA	NA	no	NA	NA
NA	NA	NA	NA	NA	no	NA	NA
no	nl	no	no	nl	yes	no	NA
NA	NA	NA	NA	NA	no	NA	NA
no	no	no	no	nl	yes	no	NA
NA	NA	NA	NA	NA	no	NA	NA
no	no	no	no	no	yes	no	NA
nl	nl	no	nl	no	yes	no	NA
no	no	nl	no	no	yes	no	NA
NA	NA	NA	NA	NA	no	NA	NA
NA	NA	NA	NA	NA	no	NA	NA
no	nl	no	no	no	yes	no	NA
NA	NA	NA	NA	NA	no	NA	NA
NA	NA	NA	NA	NA	no	NA	NA
no	no	no	nl	no	yes	no	NA
NA	NA	NA	NA	NA	no	NA	NA
NA	NA	NA	NA	NA	no	NA	NA
no	nl	no	nl	no	yes	no	NA
NA	NA	NA	NA	NA	no	NA	NA

NA	NA	NA	NA	NA	no	NA	NA
nl	nl	no	nl	no	yes	no	NA
NA	NA	NA	NA	NA	no	NA	NA
NA	NA	NA	NA	NA	no	NA	NA
no	nl	no	no	no	yes	no	NA
NA	NA	NA	NA	NA	no	NA	NA
NA	NA	NA	NA	NA	no	NA	NA
no	nl	no	no	nl	yes	no	NA
NA	NA	NA	NA	NA	no	NA	NA
NA	NA	NA	NA	NA	no	NA	NA
nl	nl	nl	no	nl	yes	no	NA
NA	NA	NA	NA	NA	no	NA	NA
NA	NA	NA	NA	NA	no	NA	NA
nl	nl	nl	no	nl	yes	no	NA
NA	NA	NA	NA	NA	no	NA	NA
NA	NA	NA	NA	NA	no	NA	NA
NA	NA	NA	NA	NA	no	NA	NA
NA	NA	NA	NA	NA	no	NA	NA
no	nl	no	no	no	yes	no	NA
nl	nl	nl	no	nl	yes	no	NA
NA	NA	NA	NA	NA	no	NA	NA
NA	NA	NA	NA	NA	no	NA	NA
no	no	nl	no	nl	yes	no	NA
NA	NA	NA	NA	NA	no	NA	NA
NA	NA	NA	NA	NA	no	NA	NA
nl	nl	no	no	P	yes	yes, ASD su	16p11.2dup
NA	NA	NA	NA	NA	no	NA	NA
NA	NA	NA	NA	NA	no	NA	NA
no	no	nl	no	nl	yes	no	NA
NA	NA	NA	NA	NA	no	NA	NA
NA	NA	NA	NA	NA	no	NA	NA
nl	nl	no	no	nl	yes	no	NA
NA	NA	NA	NA	NA	no	NA	NA
NA	NA	NA	NA	NA	no	NA	NA
no	nl	no	no	nl	yes	no	NA
NA	NA	NA	NA	NA	no	NA	NA
NA	NA	NA	NA	NA	no	NA	NA
no	no	no	no	nl	yes	no	NA
NA	NA	NA	NA	NA	no	NA	NA
NA	NA	NA	NA	NA	no	NA	NA
no	nl	no	no	nl	yes	no	NA
NA	NA	NA	NA	NA	no	NA	NA
NA	NA	NA	NA	NA	no	NA	NA
no	no	no	nl	nl	yes	no	NA
NA	NA	NA	NA	NA	no	NA	NA
NA	NA	NA	NA	NA	no	NA	NA
no	nl	no	no	no	yes	no	NA
NA	NA	NA	NA	NA	no	NA	NA
NA	NA	NA	NA	NA	no	NA	NA
no	nl	no	no	nl	yes	no	NA
NA	NA	NA	NA	NA	no	NA	NA
NA	NA	NA	NA	NA	no	NA	NA

no	no	no	nl	no	yes	no	NA
NA	NA	NA	NA	NA	no	NA	NA
NA	NA	NA	NA	NA	no	NA	NA
no	no	nl	no	no	yes	no	NA
NA	NA	NA	NA	NA	no	NA	NA
NA	NA	NA	NA	NA	no	NA	NA
nl	no	no	no	no	yes	no	NA
NA	NA	NA	NA	NA	no	NA	NA
NA	NA	NA	NA	NA	no	NA	NA
nl	nl	nl	nl	no	yes	no	NA
NA	NA	NA	NA	NA	no	NA	NA
NA	NA	NA	NA	NA	no	NA	NA
no	nl	no	no	nl	yes	no	NA
NA	NA	NA	NA	NA	no	NA	NA
NA	NA	NA	NA	NA	no	NA	NA
no	nl	nl	no	no	yes	no	NA
NA	NA	NA	NA	NA	no	NA	NA
NA	NA	NA	NA	NA	no	NA	NA
nl	nl	nl	no	no	yes	no	NA
NA	NA	NA	NA	NA	no	NA	NA
NA	NA	NA	NA	NA	no	NA	NA
no	no	nl	no	nl	yes	no	NA
NA	NA	NA	NA	NA	no	NA	NA
NA	NA	NA	NA	NA	no	NA	NA
nl	nl	no	no	no	yes	no	NA
NA	NA	NA	NA	NA	no	NA	NA
NA	NA	NA	NA	NA	no	NA	NA
nl	nl	no	no	no	yes	no	NA
NA	NA	NA	NA	NA	no	NA	NA
NA	NA	NA	NA	NA	no	NA	NA
nl	nl	no	no	nl	yes	no	NA
NA	NA	NA	NA	NA	no	NA	NA
NA	NA	NA	NA	NA	no	NA	NA
nl	nl	no	no	no	yes	no	NA
no	no	nl	no	no	yes	no	NA
NA	NA	NA	NA	NA	no	NA	NA
NA	NA	NA	NA	NA	no	NA	NA
no	nl	no	no	no	yes	no	NA
NA	NA	NA	NA	NA	no	NA	NA
NA	NA	NA	NA	NA	no	NA	NA
no	nl	nl	no	no	yes	no	NA
NA	NA	NA	NA	NA	no	NA	NA
NA	NA	NA	NA	NA	no	NA	NA
no	nl	no	no	no	yes	no	NA
NA	NA	NA	NA	NA	no	NA	NA
NA	NA	NA	NA	NA	no	NA	NA
no	nl	nl	no	nl	yes	no	NA
NA	NA	NA	NA	NA	no	NA	NA
NA	NA	NA	NA	NA	no	NA	NA
no	nl	nl	no	no	yes	no	NA
NA	NA	NA	NA	NA	no	NA	NA
NA	NA	NA	NA	NA	no	NA	NA

no	nl	nl	no	no	yes	no	NA
NA	NA	NA	NA	NA	no	NA	NA
NA	NA	NA	NA	NA	no	NA	NA
no	nl	no	no	no	yes	no	NA
NA	NA	NA	NA	NA	no	NA	NA
NA	NA	NA	NA	NA	no	NA	NA
nl	no	nl	nl	no	yes	no	NA
NA	NA	NA	NA	NA	no	NA	NA
NA	NA	NA	NA	NA	no	NA	NA
no	nl	nl	no	nl	yes	no	NA
NA	NA	NA	NA	NA	no	NA	NA
NA	NA	NA	NA	NA	no	NA	NA
nl	nl	nl	no	nl	yes	no	NA
NA	NA	NA	NA	NA	no	NA	NA
NA	NA	NA	NA	NA	no	NA	NA
nl	no	no	nl	no	yes	no	NA
no	no	nl	nl	no	yes	no	NA
NA	NA	NA	NA	NA	no	NA	NA
NA	NA	NA	NA	NA	no	NA	NA
no	no	no	nl	no	yes	no	NA
NA	NA	NA	NA	NA	no	NA	NA
NA	NA	NA	NA	NA	no	NA	NA
no	nl	no	no	nl	yes	no	NA
NA	NA	NA	NA	NA	no	NA	NA
NA	NA	NA	NA	NA	no	NA	NA
nl	gray zone	nl	nl	no	yes	no	NA
NA	NA	NA	NA	NA	no	NA	NA
nl	no	no	nl	no	yes	no	NA
NA	NA	NA	NA	NA	no	NA	NA
NA	NA	NA	NA	NA	no	NA	NA
nl	nl	P	no	LP	yes	yes, ASD su	15q11-13.1
NA	NA	NA	NA	NA	no	NA	NA
NA	NA	NA	NA	NA	no	NA	NA
nl	nl	nl	no	nl	yes	no	NA
NA	NA	NA	NA	NA	no	NA	NA
NA	NA	NA	NA	NA	no	NA	NA
no	nl	no	no	nl	yes	no	NA
NA	NA	NA	NA	NA	no	NA	NA
NA	NA	NA	NA	NA	no	NA	NA
nl	nl	nl	nl	no	yes	no	NA
NA	NA	NA	NA	NA	no	NA	NA
NA	NA	NA	NA	NA	no	NA	NA
no	nl	nl	nl	no	yes	no	NA
NA	NA	NA	NA	NA	no	NA	NA
NA	NA	NA	NA	NA	no	NA	NA
no	nl	nl	no	nl	yes	no	NA
NA	NA	NA	NA	NA	no	NA	NA
NA	NA	NA	NA	NA	no	NA	NA
no	nl	nl	nl	no	yes	no	NA
NA	NA	NA	NA	NA	no	NA	NA
NA	NA	NA	NA	NA	no	NA	NA
no	nl	no	no	nl	yes	no	NA

NA	NA	NA	NA	NA	no	NA	NA
NA	NA	NA	NA	NA	no	NA	NA
no	nl	nl	no	nl	yes	no	NA
NA	NA	NA	NA	NA	no	NA	NA
NA	NA	NA	NA	NA	no	NA	NA
nl	nl	no	no	nl	yes	no	NA
NA	NA	NA	NA	NA	no	NA	NA
NA	NA	NA	NA	NA	no	NA	NA
no	nl	nl	no	nl	yes	no	NA
NA	NA	NA	NA	NA	no	NA	NA
NA	NA	NA	NA	NA	no	NA	NA
no	no	no	nl	no	yes	no	NA
NA	NA	NA	NA	NA	no	NA	NA
NA	NA	NA	NA	NA	no	NA	NA
nl	nl	nl	no	nl	yes	no	NA
NA	NA	NA	NA	NA	no	NA	NA
NA	NA	NA	NA	NA	no	NA	NA
no	no	nl	no	nl	yes	no	NA
NA	NA	NA	NA	NA	no	NA	NA
NA	NA	NA	NA	NA	no	NA	NA
nl	nl	no	no	nl	yes	no	NA
NA	NA	NA	NA	NA	no	NA	NA
NA	NA	NA	NA	NA	no	NA	NA
no	nl	nl	no	no	yes	no	NA
NA	NA	NA	NA	NA	no	NA	NA
NA	NA	NA	NA	NA	no	NA	NA
nl	nl	no	no	no	yes	no	NA
NA	NA	NA	NA	NA	no	NA	NA
NA	NA	NA	NA	NA	no	NA	NA
nl	no	no	no	nl	yes	no	NA
NA	NA	NA	NA	NA	no	NA	NA
NA	NA	NA	NA	NA	no	NA	NA
nl	nl	no	nl	no	yes	no	NA
NA	NA	NA	NA	NA	no	NA	NA
NA	NA	NA	NA	NA	no	NA	NA
no	nl	no	no	nl	yes	no	NA
NA	NA	NA	NA	NA	no	NA	NA
NA	NA	NA	NA	NA	no	NA	NA
nl	nl	no	nl	no	yes	no	NA
NA	NA	NA	NA	NA	no	NA	NA
NA	NA	NA	NA	NA	no	NA	NA
no	nl	no	nl	no	yes	no	NA
NA	NA	NA	NA	NA	no	NA	NA
NA	NA	NA	NA	NA	no	NA	NA
nl	nl	no	no	nl	yes	no	NA
NA	NA	NA	NA	NA	no	NA	NA
NA	NA	NA	NA	NA	no	NA	NA
nl	nl	no	no	no	yes	no	NA
NA	NA	NA	NA	NA	no	NA	NA
NA	NA	NA	NA	NA	no	NA	NA
no	no	no	no	nl	yes	no	NA
NA	NA	NA	NA	NA	no	NA	NA

NA	NA	NA	NA	NA	no	NA	NA
no	nl	no	no	nl	yes	no	NA
NA	NA	NA	NA	NA	no	NA	NA
NA	NA	NA	NA	NA	no	NA	NA
no	nl	no	no	no	yes	no	NA
NA	NA	NA	NA	NA	no	NA	NA
NA	NA	NA	NA	NA	no	NA	NA
no	nl	no	no	nl	yes	no	NA
NA	NA	NA	NA	NA	no	NA	NA
NA	NA	NA	NA	NA	no	NA	NA
no	nl	no	no	nl	yes	no	NA
NA	NA	NA	NA	NA	no	NA	NA
NA	NA	NA	NA	NA	no	NA	NA
nl	nl	no	nl	nl	yes	no	NA
NA	NA	NA	NA	NA	no	NA	NA
NA	NA	NA	NA	NA	no	NA	NA
nl	nl	no	no	no	yes	no	NA
NA	NA	NA	NA	NA	no	NA	NA
NA	NA	NA	NA	NA	no	NA	NA
nl	nl	no	nl	no	yes	no	NA
NA	NA	NA	NA	NA	no	NA	NA
NA	NA	NA	NA	NA	no	NA	NA
no	no	nl	no	no	yes	no	NA
NA	NA	NA	NA	NA	no	NA	NA
NA	NA	NA	NA	NA	no	NA	NA
no	nl	no	no	no	yes	no	NA
NA	NA	NA	NA	NA	no	NA	NA
NA	NA	NA	NA	NA	no	NA	NA
nl	nl	no	nl	no	yes	no	NA
NA	NA	NA	NA	NA	no	NA	NA
NA	NA	NA	NA	NA	no	NA	NA
no	no	no	nl	no	yes	no	NA
NA	NA	NA	NA	NA	no	NA	NA
NA	NA	NA	NA	NA	no	NA	NA
no	nl	no	no	nl	yes	no	NA
NA	NA	NA	NA	NA	no	NA	NA
NA	NA	NA	NA	NA	no	NA	NA
nl	nl	no	nl	no	yes	no	NA
NA	NA	NA	NA	NA	no	NA	NA
NA	NA	NA	NA	NA	no	NA	NA
nl	nl	no	no	no	yes	no	NA
NA	NA	NA	NA	NA	no	NA	NA
NA	NA	NA	NA	NA	no	NA	NA
no	no	no	no	no	yes	no	NA
NA	NA	NA	NA	NA	no	NA	NA
no	nl	no	nl	nl	yes	no	NA
NA	NA	NA	NA	NA	no	NA	NA
NA	NA	NA	NA	NA	no	NA	NA
no	nl	no	no	no	yes	no	NA
NA	NA	NA	NA	NA	no	NA	NA
NA	NA	NA	NA	NA	no	NA	NA
no	nl	no	no	no	yes	no	NA

NA	NA	NA	NA	NA	no	NA	NA
NA	NA	NA	NA	NA	no	NA	NA
no	nl	no	no	no	yes	no	NA
NA	NA	NA	NA	NA	no	NA	NA
NA	NA	NA	NA	NA	no	NA	NA
nl	no	no	no	no	yes	no	NA
NA	NA	NA	NA	NA	no	NA	NA
NA	NA	NA	NA	NA	no	NA	NA
nl	no	no	no	no	yes	no	NA
NA	NA	NA	NA	NA	no	NA	NA
NA	NA	NA	NA	NA	no	NA	NA
no	no	no	no	no	yes	no	NA
no	no	no	no	no	yes	no	NA
NA	NA	NA	NA	NA	no	NA	NA
NA	NA	NA	NA	NA	no	NA	NA
nl	nl	P	P	no	yes	yes, monog	22q13.3del
NA	NA	NA	NA	NA	no	NA	NA
NA	NA	NA	NA	NA	no	NA	NA
nl	no	no	nl	no	yes	no	NA
nl	no	no	nl	no	yes	no	NA
NA	NA	NA	NA	NA	no	NA	NA
NA	NA	NA	NA	NA	no	NA	NA
no	no	no	no	no	yes	no	NA
NA	NA	NA	NA	NA	no	NA	NA
NA	NA	NA	NA	NA	no	NA	NA
no	nl	no	no	nl	yes	no	NA
NA	NA	NA	NA	NA	no	NA	NA
NA	NA	NA	NA	NA	no	NA	NA
no	nl	no	LP	no	yes	yes, monog	18q21.2dup
NA	NA	NA	NA	NA	no	NA	NA
NA	NA	NA	NA	NA	no	NA	NA
nl	nl	no	nl	no	yes	no	NA
NA	NA	NA	NA	NA	no	NA	NA
NA	NA	NA	NA	NA	no	NA	NA
nl	nl	no	nl	no	yes	no	NA
NA	NA	NA	NA	NA	no	NA	NA
NA	NA	NA	NA	NA	no	NA	NA
nl	no	no	nl	nl	yes	no	NA
NA	NA	NA	NA	NA	no	NA	NA
NA	NA	NA	NA	NA	no	NA	NA
no	no	no	P	P	yes	yes, monog	t(5;7)(p15.2
no	no	no	P	no	yes	yes, monog	t(5;7)(p15.2
NA	NA	NA	NA	NA	no	NA	NA
no	nl	no	no	no	yes	no	NA
NA	NA	NA	NA	NA	no	NA	NA
NA	NA	NA	NA	NA	no	NA	NA
no	nl	no	no	P	yes	yes, ASD su	16p12.2dup
NA	NA	NA	NA	NA	no	NA	NA
no	no	no	no	nl	yes	no	NA
NA	NA	NA	NA	NA	no	NA	NA
NA	NA	NA	NA	NA	no	NA	NA
NA	NA	no	NA	NA	yes	NA	NA

NA	NA	NA	NA	NA	no	NA	NA
NA	NA	NA	NA	NA	no	NA	NA
NA	NA	no	NA	NA	yes	NA	NA
NA	NA	NA	NA	NA	no	NA	NA
NA	NA	NA	NA	NA	no	NA	NA
NA	NA	no	NA	NA	yes	NA	NA
NA	NA	NA	NA	NA	no	NA	NA
NA	NA	NA	NA	NA	no	NA	NA
NA	NA	nl	NA	NA	yes	NA	NA
NA	NA	NA	NA	NA	no	NA	NA
NA	NA	NA	NA	NA	no	NA	NA
NA	NA	nl	NA	NA	yes	NA	NA
NA	NA	no	NA	NA	yes	NA	NA
NA	NA	NA	NA	NA	no	NA	NA
NA	NA	NA	NA	NA	no	NA	NA
NA	NA	nl	NA	NA	yes	NA	NA
NA	NA	NA	NA	NA	no	NA	NA
NA	NA	NA	NA	NA	no	NA	NA
NA	NA	nl	NA	NA	yes	NA	NA
NA	NA	NA	NA	NA	no	NA	NA
NA	NA	NA	NA	NA	no	NA	NA
NA	NA	no	NA	NA	yes	NA	NA
NA	NA	NA	NA	NA	no	NA	NA
NA	NA	NA	NA	NA	no	NA	NA
NA	NA	nl	NA	NA	yes	NA	NA
NA	NA	NA	NA	NA	no	NA	NA
NA	NA	NA	NA	NA	no	NA	NA
NA	NA	nl	NA	NA	yes	NA	NA
NA	NA	NA	NA	NA	no	NA	NA
NA	NA	no	NA	NA	yes	NA	NA
NA	NA	NA	NA	NA	no	NA	NA
NA	NA	nl	NA	NA	yes	NA	NA
NA	NA	NA	NA	NA	no	NA	NA
NA	NA	NA	NA	NA	no	NA	NA
NA	NA	nl	NA	NA	yes	NA	NA
NA	NA	NA	NA	NA	no	NA	NA
NA	NA	NA	NA	NA	no	NA	NA
NA	NA	nl	NA	NA	yes	NA	NA
NA	NA	NA	NA	NA	no	NA	NA
NA	NA	NA	NA	NA	no	NA	NA
NA	NA	no	NA	NA	yes	NA	NA
NA	NA	nl	NA	NA	yes	NA	NA
NA	NA	NA	NA	NA	no	NA	NA
NA	NA	no	NA	NA	yes	NA	NA
NA	NA	NA	NA	NA	no	NA	NA
NA	NA	NA	NA	NA	no	NA	NA
NA	NA	no	NA	NA	yes	NA	NA
NA	NA	NA	NA	NA	no	NA	NA
NA	NA	NA	NA	NA	no	NA	NA
NA	NA	nl	NA	NA	yes	NA	NA
NA	NA	NA	NA	NA	no	NA	NA
NA	NA	NA	NA	NA	no	NA	NA

NA	NA	no	NA	NA	yes	NA	NA
NA	NA	NA	NA	NA	no	NA	NA
NA	NA	NA	NA	NA	no	NA	NA
NA	NA	no	NA	NA	yes	NA	NA
NA	NA	NA	NA	NA	no	NA	NA
NA	NA	NA	NA	NA	no	NA	NA
NA	NA	no	NA	NA	yes	NA	NA
NA	NA	NA	NA	NA	no	NA	NA
NA	NA	NA	NA	NA	no	NA	NA
NA	NA	no	NA	NA	yes	NA	NA
NA	NA	NA	NA	NA	no	NA	NA
NA	NA	NA	NA	NA	no	NA	NA
NA	NA	no	NA	NA	yes	NA	NA
NA	NA	NA	NA	NA	no	NA	NA
NA	NA	NA	NA	NA	no	NA	NA
NA	NA	no	NA	NA	yes	NA	NA
NA	NA	NA	NA	NA	no	NA	NA
NA	NA	NA	NA	NA	no	NA	NA
NA	NA	no	NA	NA	yes	NA	NA
NA	NA	NA	NA	NA	no	NA	NA
NA	NA	NA	NA	NA	no	NA	NA
NA	NA	no	NA	NA	yes	NA	NA
NA	NA	no	NA	NA	yes	NA	NA
NA	NA	NA	NA	NA	no	NA	NA
NA	NA	NA	NA	NA	no	NA	NA
NA	NA	no	NA	NA	yes	NA	NA
NA	NA	NA	NA	NA	no	NA	NA
NA	NA	no	NA	NA	yes	NA	NA
NA	NA	NA	NA	NA	no	NA	NA
NA	NA	no	NA	NA	yes	NA	NA
NA	NA	NA	NA	NA	no	NA	NA
NA	NA	NA	NA	NA	no	NA	NA
NA	NA	no	NA	NA	yes	NA	NA
NA	NA	NA	NA	NA	no	NA	NA
NA	NA	NA	NA	NA	no	NA	NA
NA	NA	no	NA	NA	yes	NA	NA
NA	NA	NA	NA	NA	no	NA	NA
NA	NA	NA	NA	NA	no	NA	NA
NA	NA	nl	NA	NA	yes	NA	NA
NA	NA	NA	NA	NA	no	NA	NA
NA	NA	NA	NA	NA	no	NA	NA
NA	NA	no	NA	NA	yes	NA	NA
NA	NA	NA	NA	NA	no	NA	NA
NA	NA	NA	NA	NA	no	NA	NA
NA	NA	nl	NA	NA	yes	NA	NA
NA	NA	NA	NA	NA	no	NA	NA
NA	NA	NA	NA	NA	no	NA	NA
NA	NA	no	NA	NA	yes	NA	NA
NA	NA	no	NA	NA	yes	NA	NA
NA	NA	NA	NA	NA	no	NA	NA
NA	NA	NA	NA	NA	no	NA	NA
NA	NA	no	NA	NA	yes	NA	NA

NA	NA	no	NA	NA	yes	NA	NA
NA	NA	NA	NA	NA	no	NA	NA
NA	NA	NA	NA	NA	no	NA	NA
NA	NA	no	NA	NA	yes	NA	NA
NA	NA	NA	NA	NA	no	NA	NA
NA	NA	NA	NA	NA	no	NA	NA
NA	NA	nl	NA	NA	yes	NA	NA
NA	NA	NA	NA	NA	no	NA	NA
NA	NA	NA	NA	NA	no	NA	NA
NA	NA	P	NA	NA	yes	yes, ASD su	15q11-13.1
NA	NA	NA	NA	NA	no	NA	NA
NA	NA	NA	NA	NA	no	NA	NA
NA	NA	no	NA	NA	yes	NA	NA
NA	NA	NA	NA	NA	no	NA	NA
NA	NA	NA	NA	NA	no	NA	NA
NA	NA	nl	NA	NA	yes	NA	NA
NA	NA	NA	NA	NA	no	NA	NA
NA	NA	NA	NA	NA	no	NA	NA
NA	NA	nl	NA	NA	yes	NA	NA
NA	NA	NA	NA	NA	no	NA	NA
NA	NA	NA	NA	NA	no	NA	NA
NA	NA	nl	NA	NA	yes	NA	NA
NA	NA	NA	NA	NA	no	NA	NA
NA	NA	NA	NA	NA	no	NA	NA
NA	NA	nl	NA	NA	yes	NA	NA
NA	NA	NA	NA	NA	no	NA	NA
NA	NA	NA	NA	NA	no	NA	NA
NA	NA	no	NA	NA	yes	NA	NA
NA	NA	NA	NA	NA	no	NA	NA
NA	NA	NA	NA	NA	no	NA	NA
NA	NA	nl	NA	NA	yes	NA	NA
NA	NA	NA	NA	NA	no	NA	NA
NA	NA	nl	NA	NA	yes	NA	NA
NA	NA	NA	NA	NA	no	NA	NA
NA	NA	NA	NA	NA	no	NA	NA
NA	NA	nl	NA	NA	yes	NA	NA
NA	NA	NA	NA	NA	no	NA	NA
NA	NA	nl	NA	NA	yes	NA	NA
NA	NA	NA	NA	NA	no	NA	NA
NA	NA	NA	NA	NA	no	NA	NA
NA	NA	nl	NA	NA	yes	NA	NA
NA	NA	NA	NA	NA	no	NA	NA
NA	NA	NA	NA	NA	no	NA	NA
NA	NA	nl	NA	NA	yes	NA	NA
NA	NA	NA	NA	NA	no	NA	NA
NA	NA	NA	NA	NA	no	NA	NA
NA	NA	nl	NA	NA	yes	NA	NA
NA	NA	NA	NA	NA	no	NA	NA
NA	NA	NA	NA	NA	no	NA	NA
NA	NA	nl	NA	NA	yes	NA	NA
NA	NA	NA	NA	NA	no	NA	NA
NA	NA	NA	NA	NA	no	NA	NA

NA	NA	nl	NA	NA	yes	NA	NA
NA	NA	NA	NA	NA	no	NA	NA
NA	NA	NA	NA	NA	no	NA	NA
NA	NA	nl	NA	NA	yes	NA	NA
NA	NA	no	NA	NA	yes	NA	NA
NA	NA	NA	NA	NA	no	NA	NA
NA	NA	NA	NA	NA	no	NA	NA
NA	NA	NA	NA	NA	no	NA	NA
NA	NA	NA	NA	NA	no	NA	NA
NA	NA	NA	NA	NA	no	NA	NA
NA	NA	NA	NA	NA	no	NA	NA
NA	NA	no	NA	NA	yes	NA	NA
NA	NA	NA	NA	NA	no	NA	NA
NA	NA	no	NA	NA	yes	NA	NA
NA	NA	no	NA	NA	yes	NA	NA
NA	NA	no	NA	NA	yes	NA	NA
NA	NA	NA	NA	NA	no	NA	NA
NA	NA	NA	NA	NA	no	NA	NA
NA	NA	nl	NA	NA	yes	NA	NA
NA	NA	no	NA	NA	yes	NA	NA
NA	NA	NA	NA	NA	no	NA	NA
NA	NA	NA	NA	NA	no	NA	NA
NA	NA	no	NA	NA	yes	NA	NA
NA	NA	nl	NA	NA	yes	NA	NA
NA	NA	NA	NA	NA	no	NA	NA
NA	NA	no	NA	NA	yes	NA	NA
NA	NA	NA	NA	NA	no	NA	NA
NA	NA	NA	NA	NA	no	NA	NA
NA	NA	no	NA	NA	yes	NA	NA
NA	NA	no	NA	NA	yes	NA	NA
NA	NA	no	NA	NA	yes	NA	NA
NA	NA	NA	NA	NA	no	NA	NA
NA	NA	NA	NA	NA	no	NA	NA
NA	NA	no	NA	NA	yes	NA	NA
NA	NA	NA	NA	NA	no	NA	NA
NA	NA	NA	NA	NA	no	NA	NA
NA	NA	nl	NA	NA	yes	NA	NA
NA	NA	NA	NA	NA	no	NA	NA
NA	NA	NA	NA	NA	no	NA	NA
NA	NA	nl	NA	NA	yes	NA	NA
NA	NA	NA	NA	NA	no	NA	NA
NA	NA	NA	NA	NA	no	NA	NA
NA	NA	nl	NA	NA	yes	NA	NA
NA	NA	NA	NA	NA	no	NA	NA
NA	NA	NA	NA	NA	no	NA	NA
NA	NA	nl	NA	NA	yes	NA	NA
NA	NA	NA	NA	NA	no	NA	NA
NA	NA	NA	NA	NA	no	NA	NA
NA	NA	nl	NA	NA	yes	NA	NA
NA	NA	NA	NA	NA	no	NA	NA
NA	NA	NA	NA	NA	no	NA	NA
NA	NA	no	NA	NA	yes	NA	NA

NA	NA	NA	NA	NA	no	NA	NA
NA	NA	NA	NA	NA	no	NA	NA
NA	NA	nl	NA	NA	yes	NA	NA
NA	NA	NA	NA	NA	no	NA	NA
NA	NA	NA	NA	NA	no	NA	NA
NA	NA	nl	NA	NA	yes	NA	NA
NA	NA	NA	NA	NA	no	NA	NA
NA	NA	NA	NA	NA	no	NA	NA
NA	NA	nl	NA	NA	yes	NA	NA
NA	NA	NA	NA	NA	no	NA	NA
NA	NA	NA	NA	NA	no	NA	NA
NA	NA	nl	NA	NA	yes	NA	NA
NA	NA	NA	NA	NA	no	NA	NA
NA	NA	NA	NA	NA	no	NA	NA
NA	NA	no	NA	NA	yes	NA	NA
NA	NA	nl	NA	NA	yes	NA	NA
NA	NA	NA	NA	NA	no	NA	NA
NA	NA	NA	NA	NA	no	NA	NA
NA	NA	nl	NA	NA	yes	NA	NA
NA	NA	NA	NA	NA	no	NA	NA
NA	NA	NA	NA	NA	no	NA	NA
NA	NA	nl	NA	NA	yes	NA	NA
NA	NA	NA	NA	NA	no	NA	NA
NA	NA	NA	NA	NA	no	NA	NA
NA	NA	nl	NA	NA	yes	NA	NA
NA	NA	NA	NA	NA	no	NA	NA
NA	NA	NA	NA	NA	no	NA	NA
NA	NA	nl	NA	NA	yes	NA	NA
NA	NA	no	NA	NA	yes	NA	NA
NA	NA	no	NA	NA	yes	NA	NA
NA	NA	nl	NA	NA	yes	NA	NA
NA	NA	NA	NA	NA	no	NA	NA
NA	NA	NA	NA	NA	no	NA	NA
NA	NA	no	NA	NA	yes	NA	NA
NA	NA	NA	NA	NA	no	NA	NA
NA	NA	no	NA	NA	yes	NA	NA
NA	NA	NA	NA	NA	no	NA	NA
NA	NA	nl	NA	NA	yes	NA	NA
NA	NA	NA	NA	NA	no	NA	NA
NA	NA	NA	NA	NA	no	NA	NA
NA	NA	no	NA	NA	yes	NA	NA
NA	NA	NA	NA	NA	no	NA	NA
NA	NA	NA	NA	NA	no	NA	NA
NA	NA	nl	NA	NA	yes	NA	NA
NA	NA	NA	NA	NA	no	NA	NA
NA	NA	NA	NA	NA	no	NA	NA
NA	NA	nl	NA	NA	yes	NA	NA
NA	NA	NA	NA	NA	no	NA	NA
NA	NA	NA	NA	NA	no	NA	NA
NA	NA	no	NA	NA	yes	NA	NA
NA	NA	NA	NA	NA	no	NA	NA
NA	NA	NA	NA	NA	no	NA	NA

NA	NA	no	NA	NA	yes	NA	NA
NA	NA	NA	NA	NA	no	NA	NA
NA	NA	NA	NA	NA	no	NA	NA
NA	NA	no	NA	NA	yes	NA	NA
NA	NA	nl	NA	NA	yes	NA	NA
NA	NA	NA	NA	NA	no	NA	NA
NA	NA	NA	NA	NA	no	NA	NA
NA	NA	no	NA	NA	yes	NA	NA
NA	NA	NA	NA	NA	no	NA	NA
NA	NA	NA	NA	NA	no	NA	NA
NA	NA	nl	NA	NA	yes	NA	NA
NA	NA	NA	NA	NA	no	NA	NA
NA	NA	NA	NA	NA	no	NA	NA
NA	NA	no	NA	NA	yes	NA	NA
NA	NA	NA	NA	NA	no	NA	NA
NA	NA	NA	NA	NA	no	NA	NA
NA	NA	NA	NA	NA	no	NA	NA
NA	NA	NA	NA	NA	no	NA	NA
NA	NA	NA	NA	NA	no	NA	NA
NA	NA	NA	NA	NA	no	NA	NA
NA	NA	NA	NA	NA	no	NA	NA
NA	NA	NA	NA	NA	no	NA	NA
NA	NA	NA	NA	NA	no	NA	NA
NA	NA	NA	NA	NA	no	NA	NA
NA	NA	NA	NA	NA	no	NA	NA
NA	NA	NA	NA	NA	no	NA	NA
NA	NA	NA	NA	NA	no	NA	NA
NA	NA	NA	NA	NA	no	NA	NA
NA	NA	NA	NA	NA	no	NA	NA
NA	NA	NA	NA	NA	no	NA	NA
NA	NA	NA	NA	NA	no	NA	NA
NA	NA	NA	NA	NA	no	NA	NA
NA	NA	NA	NA	NA	no	NA	NA
NA	NA	NA	NA	NA	no	NA	NA
NA	NA	NA	NA	NA	no	NA	NA
NA	NA	NA	NA	NA	no	NA	NA
NA	NA	NA	NA	NA	no	NA	NA
NA	NA	NA	NA	NA	no	NA	NA
NA	NA	NA	NA	NA	no	NA	NA
NA	NA	NA	NA	NA	no	NA	NA
NA	NA	NA	NA	NA	no	NA	NA
NA	NA	NA	NA	NA	no	NA	NA
NA	NA	NA	NA	NA	no	NA	NA
NA	NA	NA	NA	NA	no	NA	NA
NA	NA	NA	NA	NA	no	NA	NA
NA	NA	NA	NA	NA	no	NA	NA
NA	NA	NA	NA	NA	no	NA	NA
NA	NA	NA	NA	NA	no	NA	NA
NA	NA	NA	NA	NA	no	NA	NA

exome_PLP_number	exome_PLP_gene	DNV_count	conclusive	conclusive_is_DNV
0	NA	3	no	0
0	NA	NA	no	0
0	NA	NA	no	0
0	NA	0	no	0
0	NA	NA	no	0
0	NA	NA	no	0
0	NA	1	no	0
0	NA	NA	no	0
0	NA	NA	no	0
0	NA	0	no	0
0	NA	NA	no	0
0	NA	NA	no	0
1	CHD4	2	yes	1
0	NA	NA	no	0
0	NA	NA	no	0
0	NA	1	no	0
0	NA	NA	no	0
0	NA	NA	no	0
0	NA	1	no	0
0	NA	NA	no	0
0	NA	NA	no	0
0	NA	4	no	0
0	NA	NA	no	0
0	NA	NA	no	0
0	NA	0	no	0
0	NA	NA	no	0
0	NA	NA	no	0
0	NA	2	no	0
0	NA	NA	no	0
0	NA	NA	no	0
0	NA	0	no	0
0	NA	NA	no	0
0	NA	NA	no	0
0	NA	1	no	0
0	NA	NA	no	0
0	NA	NA	no	0
0	NA	1	no	0
0	NA	NA	no	0
0	NA	NA	no	0
0	NA	1	no	0
0	NA	NA	no	0
0	NA	NA	no	0
0	NA	1	no	0
0	NA	NA	no	0
0	NA	NA	no	0
1	BRSK2	2	yes	1
0	NA	NA	no	0
0	NA	NA	no	0
1	BAZ2B	1	yes	0
1	BAZ2B	NA	no	0

0	NA	NA	no	0
0	NA	4	no	0
0	NA	NA	no	0
0	NA	NA	no	0
0	NA	NA	no	0
0	NA	NA	no	0
0	NA	1	no	0
0	NA	2	no	0
0	NA	NA	no	0
0	NA	NA	no	0
1	ASXL3	1	yes	1
0	NA	NA	no	0
0	NA	NA	no	0
0	NA	3	no	0
0	NA	NA	no	0
0	NA	NA	no	0
0	NA	0	no	0
0	NA	NA	no	0
0	NA	NA	no	0
1	SATB2	5	yes	0
1	SATB2	NA	no	0
0	NA	NA	no	0
0	NA	2	no	0
0	NA	NA	no	0
0	NA	NA	no	0
0	NA	2	no	0
0	NA	NA	no	0
0	NA	NA	no	0
0	NA	1	no	0
0	NA	NA	no	0
0	NA	NA	no	0
0	NA	0	no	0
0	NA	NA	no	0
0	NA	NA	no	0
0	NA	2	no	0
0	NA	NA	no	0
0	NA	NA	no	0
0	NA	1	no	0
0	NA	NA	no	0
0	NA	NA	no	0
0	NA	0	no	0
0	NA	NA	no	0
0	NA	NA	no	0
1	MECP2	1	yes	1
0	NA	NA	no	0
0	NA	NA	no	0
0	NA	0	no	0
0	NA	NA	no	0
0	NA	NA	no	0
0	NA	1	no	0
0	NA	NA	no	0
0	NA	NA	no	0

0	NA		1	no	0
0	NA	NA		no	0
0	NA	NA		no	0
0	NA		0	no	0
0	NA	NA		no	0
0	NA	NA		no	0
0	NA		2	no	0
0	NA	NA		no	0
0	NA	NA		no	0
0	NA		1	yes	0
0	NA	NA		no	0
0	NA	NA		no	0
1	DDX3X		3	yes	1
0	NA	NA		no	0
0	NA	NA		no	0
0	NA		0	no	0
0	NA	NA		no	0
0	NA	NA		no	0
0	NA		0	no	0
0	NA	NA		no	0
0	NA	NA		no	0
0	NA		1	no	0
0	NA	NA		no	0
0	NA	NA		no	0
0	NA		0	no	0
0	NA	NA		no	0
0	NA	NA		no	0
0	NA		1	no	0
0	NA	NA		no	0
0	NA	NA		no	0
0	NA		1	no	0
0	NA	NA		no	0
0	NA	NA		no	0
0	NA		0	no	0
0	NA	NA		no	0
0	NA	NA		no	0
0	NA		0	no	0
0	NA	NA		no	0
0	NA	NA		no	0
0	NA		0	no	0
0	NA	NA		no	0
0	NA	NA		no	0
0	NA		0	no	0
0	NA	NA		no	0
0	NA	NA		no	0
0	NA		1	no	0
0	NA	NA		no	0
0	NA	NA		no	0
0	NA		2	no	0
0	NA	NA		no	0
0	NA	NA		no	0
0	NA		1	no	0

0	NA	NA		no	0
0	NA	NA		no	0
0	NA		0	no	0
0	NA	NA		no	0
0	NA	NA		no	0
0	NA	NA		no	0
0	NA	NA		no	0
0	NA		0	yes	0
0	NA		2	no	0
0	NA	NA		no	0
0	NA	NA		no	0
0	NA		1	no	0
0	NA	NA		no	0
0	NA	NA		no	0
0	NA		0	no	0
0	NA		0	no	0
0	NA	NA		no	0
0	NA	NA		no	0
0	NA		0	no	0
1	MECP2		2	yes	0
0	NA	NA		no	0
0	NA	NA		no	0
0	NA	NA		no	0
0	NA	NA		no	0
0	NA		1	no	0
0	NA	NA		no	0
0	NA	NA		no	0
0	NA	NA		no	0
0	NA	NA		no	0
0	NA	NA		no	0
0	NA	NA		no	0
1	MECP2		1	yes	1
0	NA	NA		no	0
0	NA	NA		no	0
0	NA	NA		no	0
0	NA		0	no	0
0	NA	NA		no	0
0	NA	NA		no	0
0	NA	NA		no	0
0	NA		0	no	0
0	NA	NA		no	0
0	NA	NA		no	0
0	NA	NA		no	0
0	NA	NA		no	0
0	NA		1	no	0
0	NA	NA		no	0
0	NA	NA		no	0
1	KCNH1		1	yes	1
0	NA	NA		no	0
0	NA	NA		no	0
2	SCN1A; SMARCC2		0	yes	0
0	NA	NA		no	0

1	SMARCC2	NA		no	0
0	NA		0	no	0
0	NA	NA		no	0
0	NA	NA		no	0
0	NA		1	no	0
0	NA	NA		no	0
0	NA	NA		no	0
0	NA		4	no	0
0	NA	NA		no	0
0	NA	NA		no	0
0	NA		2	no	0
0	NA	NA		no	0
0	NA	NA		no	0
0	NA		2	no	0
0	NA		2	no	0
0	NA	NA		no	0
0	NA	NA		no	0
0	NA		0	no	0
0	NA	NA		no	0
0	NA	NA		no	0
0	NA		1	no	0
0	NA	NA		no	0
0	NA		1	no	0
0	NA	NA		no	0
0	NA	NA		no	0
0	NA		1	no	0
0	NA	NA		no	0
0	NA	NA		no	0
0	NA		2	no	0
0	NA	NA		no	0
0	NA		0	no	0
0	NA	NA		no	0
0	NA	NA		no	0
0	NA		0	no	0
0	NA	NA		no	0
0	NA	NA		no	0
0	NA	NA		no	0
0	NA	NA		no	0
0	NA		1	no	0
0	NA	NA		no	0
0	NA	NA		no	0
0	NA		1	no	0
0	NA	NA		no	0
0	NA	NA		no	0
0	NA		0	no	0
0	NA	NA		no	0
0	NA	NA		no	0
0	NA		3	no	0
0	NA	NA		no	0
0	NA	NA		no	0
1	NF1		4	yes	1
0	NA	NA		no	0

0	NA	NA		no	0
0	NA		1	no	0
0	NA	NA		no	0
0	NA	NA		no	0
0	NA		0	no	0
0	NA	NA		no	0
0	NA	NA		no	0
0	NA		3	no	0
0	NA	NA		no	0
0	NA	NA		no	0
0	NA		1	no	0
0	NA	NA		no	0
0	NA	NA		no	0
0	NA		5	no	0
0	NA	NA		no	0
0	NA	NA		no	0
0	NA		3	no	0
0	NA	NA		no	0
0	NA	NA		no	0
0	NA		0	no	0
0	NA	NA		no	0
0	NA	NA		no	0
0	NA		1	no	0
0	NA	NA		no	0
0	NA	NA		no	0
0	NA		1	no	0
0	NA	NA		no	0
0	NA	NA		no	0
0	NA		0	no	0
0	NA	NA		no	0
0	NA	NA		no	0
0	NA		0	no	0
0	NA	NA		no	0
0	NA	NA		no	0
0	NA		0	no	0
0	NA	NA		no	0
0	NA	NA		no	0
1	SLC6A1		2	yes	1
0	NA	NA		no	0
0	NA	NA		no	0
0	NA		0	no	0
0	NA	NA		no	0
0	NA	NA		no	0
0	NA		1	no	0
0	NA	NA		no	0
0	NA	NA		no	0
0	NA		1	no	0
0	NA	NA		no	0
0	NA	NA		no	0
0	NA		1	no	0
0	NA	NA		no	0
0	NA	NA		no	0

0	NA	NA		no	0
0	NA	NA		no	0
0	NA		0	no	0
0	NA	NA		no	0
0	NA	NA		no	0
0	NA		1	no	0
0	NA	NA		no	0
0	NA	NA		no	0
0	NA		1	no	0
0	NA	NA		no	0
0	NA	NA		no	0
0	NA		3	no	0
0	NA	NA		no	0
0	NA	NA		no	0
0	NA		1	no	0
0	NA	NA		no	0
0	NA	NA		no	0
0	NA		3	no	0
0	NA	NA		no	0
0	NA	NA		no	0
0	NA		3	no	0
0	NA	NA		no	0
0	NA	NA		no	0
0	NA		1	no	0
0	NA	NA		no	0
0	NA	NA		no	0
1	SKI		3	yes	1
0	NA	NA		no	0
0	NA	NA		no	0
0	NA		1	no	0
0	NA	NA		no	0
0	NA	NA		no	0
0	NA	NA		no	0
0	NA	NA		no	0
0	NA		0	no	0
0	NA	NA		no	0
0	NA	NA		no	0
0	NA		0	no	0
0	NA	NA		no	0
0	NA	NA		no	0
1	ASXL3		3	yes	1
0	NA	NA		no	0
0	NA	NA		no	0
0	NA		1	no	0
0	NA	NA		no	0
0	NA	NA		no	0
0	NA		1	no	0
0	NA	NA		no	0
0	NA	NA		no	0
0	NA		0	no	0
0	NA	NA		no	0
0	NA	NA		no	0

0	NA		0	no	0
0	NA	NA		no	0
0	NA	NA		no	0
0	NA		1	no	0
0	NA	NA		no	0
0	NA	NA		no	0
0	NA		3	no	0
0	NA	NA		no	0
0	NA	NA		no	0
0	NA		0	no	0
0	NA	NA		no	0
0	NA	NA		no	0
1	MECP2		1	yes	1
0	NA	NA		no	0
0	NA	NA		no	0
0	NA		1	no	0
0	NA	NA		no	0
0	NA	NA		no	0
0	NA		0	no	0
0	NA	NA		no	0
0	NA	NA		no	0
0	NA		1	no	0
0	NA	NA		no	0
0	NA	NA		no	0
1	TLK2		1	yes	1
0	NA	NA		no	0
0	NA	NA		no	0
0	NA		0	no	0
0	NA	NA		no	0
0	NA	NA		no	0
1	CSNK2A1		3	yes	1
0	NA	NA		no	0
0	NA	NA		no	0
0	NA		2	no	0
0	NA	NA		no	0
0	NA	NA		no	0
0	NA		1	no	0
0	NA	NA		no	0
0	NA	NA		no	0
0	NA		2	no	0
0	NA	NA		no	0
0	NA	NA		no	0
0	NA		2	no	0
0	NA	NA		no	0
0	NA	NA		no	0
0	NA	NA		no	0
0	NA	NA		no	0
0	NA		1	no	0
0	NA	NA		no	0
0	NA	NA		no	0
0	NA		1	no	0
0	NA	NA		no	0

0	NA	NA	no	0
0	NA	1	no	0
0	NA	NA	no	0
0	NA	NA	no	0
0	NA	0	no	0
0	NA	NA	no	0
0	NA	NA	no	0
0	NA	2	no	0
0	NA	NA	no	0
0	NA	NA	no	0
0	NA	2	no	0
0	NA	NA	no	0
0	NA	NA	no	0
0	NA	1	yes	0
0	NA	NA	no	0
0	NA	NA	no	0
0	NA	NA	yes	0
0	NA	0	no	0
0	NA	NA	no	0
0	NA	NA	no	0
0	NA	1	no	0
0	NA	2	no	0
1	HEPACAM	0	yes	0
0	NA	NA	no	0
1	HEPACAM	NA	no	0
0	NA	NA	no	0
0	NA	NA	no	0
0	NA	NA	no	0
0	NA	NA	no	0
0	NA	NA	no	0
0	NA	NA	no	0
1	SCN2A	4	yes	1
0	NA	NA	no	0
0	NA	NA	no	0
1	PTPN11	1	yes	1
0	NA	NA	no	0
0	NA	NA	no	0
0	NA	NA	no	0
0	NA	NA	no	0
0	NA	NA	no	0
0	NA	1	no	0
0	NA	NA	no	0
0	NA	NA	no	0
0	NA	1	no	0
0	NA	NA	no	0
0	NA	NA	no	0
0	NA	NA	no	0
0	NA	NA	no	0
0	NA	NA	no	0
0	NA	NA	no	0
0	NA	1	no	0
0	NA	0	no	0

0	NA	NA	no	0
0	NA	NA	no	0
0	NA	NA	no	0
0	NA	NA	no	0
0	NA	NA	no	0
0	NA	NA	no	0
1	FGF12	1	yes	1
0	NA	NA	no	0
0	NA	NA	no	0
1	PACS2	2	yes	1
0	NA	NA	no	0
0	NA	NA	no	0
0	NA	0	no	0
0	NA	NA	no	0
0	NA	NA	no	0
0	NA	NA	no	0
0	NA	NA	no	0
1	CREBBP	NA	yes	0
0	NA	NA	no	0
0	NA	0	no	0
0	NA	NA	no	0
0	NA	NA	no	0
0	NA	1	no	0
0	NA	NA	no	0
0	NA	NA	no	0
0	NA	NA	no	0
0	NA	NA	no	0
0	NA	1	no	0
0	NA	NA	no	0
0	NA	NA	no	0
0	NA	NA	no	0
0	NA	NA	no	0
0	NA	NA	no	0
1	SETD1A	2	yes	1
0	NA	NA	no	0
0	NA	NA	no	0
0	NA	NA	no	0
0	NA	1	no	0
0	NA	NA	no	0
0	NA	NA	no	0
0	NA	0	no	0
0	NA	NA	no	0
0	NA	NA	no	0
0	NA	2	no	0
0	NA	NA	no	0
0	NA	NA	no	0
0	NA	NA	no	0
0	NA	NA	no	0
0	NA	NA	no	0
1	EEF1A2	1	yes	1
0	NA	NA	no	0
0	NA	NA	no	0

0	NA		0	no	0
0	NA	NA		no	0
0	NA	NA		no	0
0	NA		0	no	0
0	NA	NA		no	0
0	NA	NA		no	0
0	NA		4	no	0
0	NA	NA		no	0
0	NA	NA		no	0
0	NA		2	no	0
0	NA	NA		no	0
0	NA	NA		no	0
0	NA		1	no	0
0	NA	NA		no	0
0	NA	NA		no	0
0	NA	NA		no	0
0	NA	NA		no	0
0	NA		2	no	0
0	NA	NA		no	0
0	NA	NA		no	0
0	NA	NA		no	0
0	NA	NA		no	0
0	NA	NA		no	0
0	NA	NA		no	0
0	NA		1	no	0
0	NA	NA		no	0
0	NA	NA		no	0
0	NA	NA		no	0
0	NA		0	no	0
0	NA	NA		no	0
0	NA	NA		no	0
1	PTEN	NA		yes	0
0	NA	NA		no	0
0	NA		2	no	0
0	NA	NA		no	0
0	NA	NA		no	0
0	NA		0	no	0
0	NA	NA		no	0
0	NA	NA		no	0
0	NA		4	no	0
0	NA	NA		no	0
0	NA	NA		no	0
1	PTEN	NA		yes	0
1	PTEN	NA		no	0
0	NA	NA		no	0
0	NA	NA		no	0
0	NA	NA		no	0
0	NA	NA		no	0
0	NA	NA		no	0
0	NA	NA		no	0
0	NA	NA		no	0
0	NA	NA		no	0

0	NA		0	no	0
0	NA	NA		no	0
0	NA	NA		no	0
0	NA	NA		no	0
0	NA	NA		no	0
0	NA	NA		no	0
0	NA	NA		no	0
0	NA	NA		no	0
0	NA		2	no	0
0	NA	NA		no	0
0	NA	NA		no	0
0	NA		5	no	0
0	NA	NA		no	0
0	NA	NA		no	0
0	NA	NA		no	0
0	NA	NA		no	0
0	NA	NA		no	0
0	NA	NA		no	0
0	NA		0	no	0
0	NA	NA		no	0
0	NA	NA		no	0
0	NA		1	yes	0
0	NA	NA		no	0
0	NA	NA		no	0
0	NA	NA		no	0
0	NA	NA		no	0
0	NA	NA		no	0
0	NA	NA		no	0
0	NA		0	no	0
0	NA	NA		no	0
0	NA	NA		no	0
0	NA	NA		no	0
0	NA	NA		no	0
0	NA		2	no	0
0	NA	NA		no	0
0	NA	NA		no	0
0	NA	NA		no	0
0	NA	NA		no	0
0	NA		4	no	0
0	NA	NA		no	0
0	NA	NA		no	0
0	NA		2	no	0
0	NA	NA		no	0
0	NA	NA		no	0
0	NA	NA		no	0
0	NA	NA		no	0
0	NA	NA		no	0
1	SRCAP	NA		yes	0
0	NA	NA		no	0
0	NA		0	no	0
0	NA	NA		no	0
0	NA	NA		no	0

0	NA		1	no	0
0	NA	NA		no	0
0	NA	NA		no	0
0	NA	NA		no	0
0	NA	NA		no	0
0	NA		2	no	0
0	NA	NA		no	0
0	NA	NA		no	0
0	NA		5	no	0
0	NA	NA		no	0
0	NA	NA		no	0
0	NA		0	no	0
0	NA	NA		no	0
0	NA	NA		no	0
0	NA		3	no	0
0	NA	NA		no	0
0	NA	NA		no	0
0	NA		2	no	0
0	NA	NA		no	0
0	NA	NA		no	0
0	NA		2	no	0
0	NA	NA		no	0
0	NA	NA		no	0
0	NA		0	yes	0
0	NA	NA		no	0
0	NA	NA		no	0
0	NA		0	no	0
0	NA	NA		no	0
0	NA	NA		no	0
0	NA		2	no	0
0	NA	NA		no	0
0	NA	NA		no	0
0	NA		0	no	0
0	NA	NA		no	0
0	NA	NA		no	0
0	NA	NA		no	0
0	NA	NA		no	0
0	NA	NA		no	0
0	NA	NA		no	0
0	NA	NA		no	0
0	NA	NA		no	0
0	NA		0	no	0
0	NA	NA		no	0
0	NA	NA		no	0
0	NA		2	no	0
0	NA	NA		no	0
0	NA	NA		no	0
1	FOXP1		2	yes	1
0	NA	NA		no	0
0	NA	NA		no	0
1	DEAF1		1	yes	1
0	NA	NA		no	0

0	NA	NA		no	0
0	NA		2	no	0
0	NA	NA		no	0
0	NA	NA		no	0
0	NA		3	no	0
0	NA	NA		no	0
0	NA	NA		no	0
0	NA		0	no	0
0	NA	NA		no	0
0	NA	NA		no	0
0	NA		2	no	0
0	NA	NA		no	0
0	NA	NA		no	0
0	NA		0	no	0
0	NA	NA		no	0
0	NA	NA		no	0
0	NA	NA		no	0
0	NA	NA		no	0
0	NA		0	no	0
0	NA		0	no	0
0	NA	NA		no	0
0	NA	NA		no	0
0	NA		0	no	0
0	NA	NA		no	0
0	NA	NA		no	0
1	LZTR1		2	yes	0
0	NA	NA		no	0
1	LZTR1	NA		no	0
0	NA		6	no	0
0	NA	NA		no	0
0	NA	NA		no	0
0	NA		0	no	0
0	NA	NA		no	0
0	NA	NA		no	0
0	NA		1	no	0
0	NA	NA		no	0
0	NA	NA		no	0
0	NA		1	no	0
0	NA	NA		no	0
0	NA	NA		no	0
0	NA		1	no	0
0	NA	NA		no	0
0	NA	NA		no	0
0	NA		4	no	0
0	NA	NA		no	0
0	NA	NA		no	0
0	NA		3	no	0
0	NA	NA		no	0
0	NA	NA		no	0
0	NA		0	no	0
0	NA	NA		no	0
0	NA	NA		no	0

0	NA		2	no	0
0	NA	NA		no	0
0	NA	NA		no	0
1	IQSEC2		1	yes	1
0	NA	NA		no	0
0	NA	NA		no	0
0	NA		2	no	0
0	NA	NA		no	0
0	NA	NA		no	0
0	NA		1	no	0
0	NA	NA		no	0
0	NA	NA		no	0
0	NA		0	no	0
0	NA	NA		no	0
0	NA	NA		no	0
0	NA		2	no	0
0	NA	NA		no	0
0	NA	NA		no	0
0	NA		1	no	0
0	NA	NA		no	0
0	NA	NA		no	0
1	SHANK3		2	yes	1
0	NA	NA		no	0
0	NA	NA		no	0
0	NA		2	no	0
0	NA	NA		no	0
0	NA	NA		no	0
0	NA		1	no	0
0	NA	NA		no	0
0	NA	NA		no	0
0	NA		0	no	0
0	NA	NA		no	0
0	NA	NA		no	0
0	NA	NA		no	0
0	NA		1	no	0
0	NA	NA		no	0
0	NA	NA		no	0
0	NA		1	no	0
0	NA	NA		no	0
0	NA	NA		no	0
0	NA		3	no	0
0	NA	NA		no	0
0	NA	NA		no	0
0	NA		1	no	0
0	NA	NA		no	0
0	NA	NA		no	0
0	NA		0	no	0
0	NA	NA		no	0
0	NA	NA		no	0
0	NA		4	no	0
0	NA	NA		no	0
0	NA	NA		no	0

0	NA		1	no	0
0	NA	NA		no	0
0	NA	NA		no	0
0	NA		0	no	0
0	NA	NA		no	0
0	NA	NA		no	0
0	NA		2	no	0
0	NA	NA		no	0
0	NA	NA		no	0
0	NA		0	no	0
0	NA	NA		no	0
0	NA	NA		no	0
0	NA		0	no	0
0	NA	NA		no	0
0	NA	NA		no	0
0	NA	NA		no	0
0	NA		0	no	0
0	NA	NA		no	0
0	NA	NA		no	0
0	NA		2	no	0
0	NA	NA		no	0
0	NA	NA		no	0
0	NA		1	no	0
0	NA	NA		no	0
0	NA	NA		no	0
0	NA	NA		no	0
0	NA	NA		no	0
0	NA		2	no	0
0	NA	NA		no	0
0	NA	NA		no	0
0	NA		0	yes	0
0	NA	NA		no	0
0	NA	NA		no	0
0	NA		0	no	0
0	NA	NA		no	0
0	NA	NA		no	0
0	NA		0	no	0
0	NA	NA		no	0
0	NA	NA		no	0
0	NA		1	no	0
0	NA	NA		no	0
0	NA	NA		no	0
0	NA		2	no	0
0	NA	NA		no	0
0	NA	NA		no	0
0	NA		0	no	0
0	NA	NA		no	0
0	NA	NA		no	0
0	NA		0	no	0
0	NA	NA		no	0
0	NA	NA		no	0
0	NA		2	no	0

0	NA	NA		no	0
0	NA	NA		no	0
0	NA		0	no	0
0	NA	NA		no	0
0	NA	NA		no	0
0	NA		2	no	0
0	NA	NA		no	0
0	NA	NA		no	0
0	NA		3	no	0
0	NA	NA		no	0
0	NA	NA		no	0
0	NA		3	no	0
0	NA	NA		no	0
0	NA	NA		no	0
0	NA		2	no	0
0	NA	NA		no	0
0	NA	NA		no	0
0	NA		1	no	0
0	NA	NA		no	0
0	NA	NA		no	0
0	NA		3	no	0
0	NA	NA		no	0
0	NA	NA		no	0
1	SYNGAP1		2	yes	1
0	NA	NA		no	0
0	NA	NA		no	0
1	KAT6A		1	yes	1
0	NA	NA		no	0
0	NA	NA		no	0
0	NA		1	no	0
0	NA	NA		no	0
0	NA	NA		no	0
0	NA		1	no	0
0	NA	NA		no	0
0	NA	NA		no	0
0	NA		2	no	0
0	NA	NA		no	0
0	NA	NA		no	0
0	NA		0	no	0
0	NA	NA		no	0
0	NA	NA		no	0
0	NA		4	no	0
0	NA	NA		no	0
0	NA	NA		no	0
0	NA		1	no	0
0	NA	NA		no	0
0	NA	NA		no	0
0	NA		2	no	0
0	NA	NA		no	0
0	NA	NA		no	0
0	NA		1	no	0
0	NA	NA		no	0

0	NA	NA	no	0
0	NA	2	no	0
0	NA	NA	no	0
0	NA	NA	no	0
0	NA	3	no	0
0	NA	NA	no	0
0	NA	NA	no	0
0	NA	0	no	0
0	NA	NA	no	0
0	NA	NA	no	0
0	NA	1	no	0
0	NA	NA	no	0
0	NA	NA	no	0
0	NA	1	no	0
0	NA	NA	no	0
0	NA	NA	no	0
0	NA	1	no	0
0	NA	NA	no	0
0	NA	NA	no	0
0	NA	0	no	0
0	NA	NA	no	0
0	NA	NA	no	0
0	NA	1	no	0
0	NA	NA	no	0
0	NA	NA	no	0
0	NA	1	no	0
0	NA	NA	no	0
0	NA	NA	no	0
0	NA	1	no	0
0	NA	NA	no	0
0	NA	NA	no	0
0	NA	2	no	0
0	NA	NA	no	0
0	NA	NA	no	0
0	NA	3	no	0
0	NA	NA	no	0
0	NA	NA	no	0
0	NA	0	no	0
0	NA	NA	no	0
0	NA	NA	no	0
0	NA	1	no	0
0	NA	NA	no	0
0	NA	NA	no	0
0	NA	0	no	0
0	NA	NA	no	0
0	NA	0	no	0
0	NA	NA	no	0
0	NA	NA	no	0
0	NA	3	no	0
0	NA	NA	no	0
0	NA	NA	no	0
0	NA	2	no	0

0	NA	NA		no	0
0	NA	NA		no	0
0	NA		0	no	0
0	NA	NA		no	0
0	NA	NA		no	0
0	NA		2	no	0
0	NA	NA		no	0
0	NA	NA		no	0
0	NA		3	no	0
0	NA	NA		no	0
0	NA	NA		no	0
0	NA	NA		no	0
0	NA		2	no	0
0	NA	NA		no	0
1	CHD5	NA		no	0
0	NA		2	yes	0
0	NA	NA		no	0
0	NA	NA		no	0
0	NA	NA		no	0
1	CUL3		2	yes	1
0	NA	NA		no	0
0	NA	NA		no	0
1	FOXP1		3	yes	1
0	NA	NA		no	0
0	NA	NA		no	0
0	NA		0	no	0
0	NA	NA		no	0
0	NA	NA		no	0
0	NA		2	yes	0
0	NA	NA		no	0
0	NA	NA		no	0
0	NA		1	no	0
0	NA	NA		no	0
0	NA	NA		no	0
0	NA		1	no	0
0	NA	NA		no	0
0	NA	NA		no	0
0	NA		3	no	0
0	NA	NA		no	0
0	NA	NA		no	0
0	NA	NA		yes	0
0	NA	NA		yes	0
0	NA	NA		no	0
0	NA		2	no	0
0	NA	NA		no	0
0	NA	NA		no	0
0	NA	NA		yes	0
0	NA	NA		no	0
0	NA		1	no	0
0	NA	NA		no	0
0	NA	NA		no	0
0	NA		3	no	0

0	NA	NA	no	0
0	NA	NA	no	0
0	NA	0	no	0
0	NA	NA	no	0
0	NA	NA	no	0
0	NA	2	no	0
0	NA	NA	no	0
0	NA	NA	no	0
1	STXBP1	1	yes	1
0	NA	NA	no	0
0	NA	NA	no	0
0	NA	NA	no	0
1	KCNB1	2	yes	1
0	NA	NA	no	0
0	NA	NA	no	0
0	NA	1	no	0
0	NA	NA	no	0
0	NA	NA	no	0
0	NA	1	no	0
0	NA	NA	no	0
0	NA	NA	no	0
0	NA	0	no	0
0	NA	NA	no	0
0	NA	NA	no	0
0	NA	0	no	0
0	NA	NA	no	0
0	NA	NA	no	0
0	NA	NA	no	0
0	NA	NA	no	0
0	NA	NA	no	0
0	NA	NA	no	0
0	NA	1	no	0
0	NA	NA	no	0
0	NA	NA	no	0
0	NA	0	no	0
0	NA	NA	no	0
0	NA	NA	no	0
0	NA	2	no	0
0	NA	NA	no	0
0	NA	NA	no	0
0	NA	NA	no	0
0	NA	NA	no	0
0	NA	NA	no	0
0	NA	1	no	0
0	NA	NA	no	0
0	NA	NA	no	0
0	NA	NA	no	0
0	NA	NA	no	0
0	NA	NA	no	0
0	NA	0	no	0
0	NA	NA	no	0
0	NA	NA	no	0

1	ZBTB20		0	yes	0
0	NA	NA		no	0
0	NA	NA		no	0
0	NA		1	no	0
0	NA	NA		no	0
0	NA	NA		no	0
0	NA		1	no	0
0	NA	NA		no	0
0	NA	NA		no	0
0	NA		2	no	0
0	NA	NA		no	0
0	NA	NA		no	0
1	SHANK3		0	yes	0
0	NA	NA		no	0
0	NA	NA		no	0
0	NA		0	no	0
0	NA	NA		no	0
0	NA	NA		no	0
0	NA		0	no	0
0	NA	NA		no	0
0	NA	NA		no	0
0	NA	NA		no	0
0	NA		1	no	0
0	NA	NA		no	0
0	NA	NA		no	0
0	NA	NA		no	0
0	NA	NA		no	0
1	RHOBTB2	NA		yes	1
0	NA	NA		no	0
0	NA		1	no	0
0	NA	NA		no	0
0	NA	NA		no	0
0	NA		1	no	0
0	NA	NA		no	0
0	NA	NA		no	0
0	NA		4	no	0
0	NA	NA		no	0
0	NA	NA		no	0
1	FOXG1		1	yes	1
0	NA	NA		no	0
0	NA	NA		no	0
0	NA		1	no	0
0	NA	NA		no	0
0	NA	NA		no	0
0	NA		2	no	0
0	NA	NA		no	0
0	NA	NA		no	0
0	NA	NA		no	0
0	NA		1	no	0
0	NA	NA		no	0
0	NA	NA		no	0
0	NA	NA		no	0

0	NA		0	no	0
0	NA	NA		no	0
0	NA	NA		no	0
0	NA		1	no	0
0	NA	NA		no	0
0	NA	NA		no	0
0	NA		1	no	0
0	NA	NA		no	0
0	NA	NA		no	0
0	NA		0	yes	0
0	NA	NA		no	0
0	NA	NA		no	0
0	NA		1	no	0
0	NA	NA		no	0
0	NA	NA		no	0
0	NA		1	no	0
0	NA	NA		no	0
0	NA	NA		no	0
0	NA		0	no	0
0	NA	NA		no	0
0	NA	NA		no	0
0	NA		0	no	0
0	NA	NA		no	0
0	NA	NA		no	0
0	NA		1	no	0
0	NA	NA		no	0
0	NA	NA		no	0
0	NA		0	no	0
0	NA	NA		no	0
0	NA	NA		no	0
0	NA	NA		no	0
0	NA	NA		no	0
0	NA		0	no	0
0	NA	NA		no	0
0	NA	NA		no	0
0	NA	NA		no	0
0	NA	NA		no	0
0	NA		1	no	0
0	NA	NA		no	0
0	NA	NA		no	0
0	NA		0	no	0
0	NA	NA		no	0
0	NA	NA		no	0
0	NA		0	no	0
0	NA	NA		no	0
0	NA	NA		no	0
0	NA		1	no	0
0	NA	NA		no	0
0	NA	NA		no	0
0	NA		0	no	0
0	NA	NA		no	0
0	NA	NA		no	0

0	NA	NA	no	0
0	NA	NA	no	0
0	NA	NA	no	0
0	NA	0	no	0
0	NA	NA	no	0
0	NA	NA	no	0
0	NA	NA	no	0
0	NA	NA	no	0
0	NA	NA	no	0
0	NA	NA	no	0
0	NA	NA	no	0
0	NA	0	no	0
0	NA	NA	no	0
0	NA	NA	no	0
0	NA	NA	no	0
0	NA	0	no	0
0	NA	NA	no	0
0	NA	NA	no	0
0	NA	2	no	0
1	AP1S2	NA	yes	0
1	AP1S2	NA	no	0
0	NA	NA	no	0
1	AP1S2	NA	yes	0
1	PHF2	NA	yes	0
0	NA	NA	no	0
0	NA	2	no	0
0	NA	NA	no	0
0	NA	NA	no	0
0	NA	NA	no	0
0	NA	NA	no	0
0	NA	0	no	0
0	NA	NA	no	0
0	NA	NA	no	0
0	NA	2	no	0
0	NA	NA	no	0
0	NA	NA	no	0
0	NA	0	no	0
0	NA	NA	no	0
0	NA	NA	no	0
1	SCN1A	2	yes	1
0	NA	NA	no	0
0	NA	NA	no	0
0	NA	0	no	0
0	NA	NA	no	0
0	NA	NA	no	0
0	NA	0	no	0
0	NA	NA	no	0
0	NA	NA	no	0
0	NA	0	no	0
0	NA	NA	no	0
0	NA	NA	no	0
0	NA	3	no	0

0	NA	NA		no	0
0	NA	NA		no	0
0	NA		3	no	0
0	NA	NA		no	0
0	NA	NA		no	0
0	NA		1	no	0
0	NA	NA		no	0
0	NA	NA		no	0
0	NA		0	no	0
0	NA	NA		no	0
0	NA	NA		no	0
0	NA		1	no	0
0	NA	NA		no	0
0	NA	NA		no	0
0	NA	NA		no	0
0	NA		0	no	0
0	NA	NA		no	0
0	NA	NA		no	0
0	NA		1	no	0
0	NA	NA		no	0
0	NA	NA		no	0
0	NA		0	no	0
0	NA	NA		no	0
0	NA	NA		no	0
0	NA		4	no	0
0	NA	NA		no	0
0	NA	NA		no	0
0	NA		1	no	0
0	NA	NA		no	0
1	DYRK1A	NA		yes	0
0	NA		3	no	0
0	NA	NA		no	0
0	NA	NA		no	0
0	NA	NA		no	0
0	NA	NA		no	0
0	NA	NA		no	0
0	NA	NA		no	0
0	NA		0	no	0
0	NA	NA		no	0
0	NA	NA		no	0
0	NA		3	no	0
0	NA	NA		no	0
0	NA	NA		no	0
0	NA		0	no	0
0	NA	NA		no	0
0	NA	NA		no	0
0	NA		0	no	0
0	NA	NA		no	0
0	NA	NA		no	0
0	NA		1	no	0
0	NA	NA		no	0
0	NA	NA		no	0

0	NA		3	no	0
0	NA	NA		no	0
0	NA	NA		no	0
0	NA	NA		no	0
0	NA		0	no	0
0	NA	NA		no	0
0	NA	NA		no	0
0	NA		0	no	0
0	NA	NA		no	0
0	NA	NA		no	0
0	NA		1	no	0
0	NA	NA		no	0
0	NA	NA		no	0
0	NA		1	no	0
0	NA	NA		no	0
0	NA	NA		no	0
0	NA		0	no	0
0	NA		1	no	0
0	NA	NA		no	0
0	NA	NA		no	0
0	NA		2	no	0
0	NA		0	no	0
0	NA	NA		no	0
0	NA	NA		no	0
0	NA		0	no	0
0	NA		2	no	0
0	NA	NA		no	0
0	NA	NA		no	0
0	NA	NA		no	0
0	NA	NA		no	0
0	NA	NA		no	0
0	NA	NA		no	0
0	NA		0	no	0
0	NA	NA		no	0
0	NA		0	no	0
0	NA	NA		no	0
1	GABBR2		1	yes	1
0	NA	NA		no	0
0	NA	NA		no	0
0	NA		0	no	0
0	NA	NA		no	0
0	NA	NA		no	0
0	NA		0	no	0
0	NA	NA		no	0
0	NA	NA		no	0
0	NA	NA		no	0
0	NA	NA		no	0
0	NA	NA		no	0
0	NA	NA		no	0

recurrent_geneorCNV	RRV_number_genese	RRV_number_noXY	RRV_number_phased	in_Moreira2014
no	2	2	2	no
no	1	1	0	no
no	1	1	0	no
no	4	4	4	no
no	2	2	0	no
no	4	4	0	no
no	1	1	0	no
no	1	1	0	no
no	0	0	0	no
no	4	4	2	no
no	4	4	0	no
no	2	2	0	no
no	3	3	3	no
no	2	2	0	no
no	1	1	0	no
no	0	0	0	no
no	0	0	0	no
no	1	1	0	no
no	0	0	0	no
no	0	0	0	no
no	2	2	0	no
no	2	2	2	no
no	1	1	0	no
no	1	1	0	no
no	2	2	1	no
no	5	5	0	no
no	3	3	0	no
no	0	0	0	no
no	0	0	0	no
no	2	2	0	no
no	0	0	0	no
no	0	0	0	no
no	3	3	0	no
no	3	2	2	no
no	3	2	0	no
no	1	1	0	no
no	6	6	5	no
no	8	8	0	no
no	5	5	0	no
no	2	2	1	no
no	2	2	0	no
no	2	2	0	no
no	1	1	1	no
no	2	2	0	no
no	0	0	0	no
no	4	4	3	no
no	5	4	0	no
no	4	4	0	no
no	4	4	4	no
no	2	2	0	no

no	5	5	0	no
no	2	2	2	no
no	0	0	0	no
no	1	1	0	no
no	1	1	0	no
no	1	1	0	no
no	0	0	0	no
no	4	4	3	no
no	2	2	0	no
no	4	4	0	no
yes	3	3	2	no
no	1	1	0	no
no	1	1	0	no
no	1	1	1	no
no	2	2	0	no
no	2	2	0	no
no	1	1	1	no
no	2	2	0	no
no	0	0	0	no
no	3	3	2	no
no	1	1	0	no
no	3	4	0	no
no	2	3	3	no
no	2	2	0	no
no	2	3	0	no
no	1	1	1	no
no	0	0	0	no
no	1	1	0	no
no	4	4	4	no
no	2	2	0	no
no	3	3	0	no
no	1	1	1	no
no	3	3	0	no
no	2	2	0	no
no	0	0	0	no
no	0	0	0	no
no	0	0	0	no
no	1	1	1	no
no	4	4	0	no
no	1	1	0	no
no	2	2	2	no
no	2	2	0	no
no	3	3	0	no
yes	2	2	1	no
no	0	0	0	no
no	2	2	0	no
no	2	2	2	no
no	2	2	0	no
no	3	4	0	no
no	1	1	1	no
no	1	1	0	no
no	2	2	0	no

no	4	5	4	no
no	3	4	0	no
no	3	3	0	no
no	3	3	3	no
no	2	2	0	no
no	1	1	0	no
no	1	1	1	no
no	0	0	0	no
no	2	2	0	no
no	1	1	1	no
no	0	0	0	no
no	2	2	0	no
no	3	2	2	no
no	4	4	0	no
no	5	5	0	no
no	4	4	3	no
no	2	2	0	no
no	4	4	0	no
no	2	2	0	no
no	2	2	0	no
no	3	3	0	no
no	1	1	1	no
no	4	4	0	no
no	1	1	0	no
no	4	4	3	no
no	5	5	0	no
no	1	1	0	no
no	4	5	4	no
no	2	2	0	no
no	7	8	0	no
no	5	5	5	no
no	6	6	0	no
no	0	0	0	no
no	4	4	4	no
no	4	4	0	no
no	2	2	0	no
no	5	5	5	no
no	4	4	0	no
no	4	4	0	no
no	3	3	3	no
no	7	7	0	no
no	2	2	0	no
no	4	4	3	no
no	2	2	0	no
no	2	2	0	no
no	6	6	6	no
no	6	6	0	no
no	1	1	0	no
no	3	3	3	no
no	4	4	0	no
no	4	4	0	no
no	2	2	2	no

no	2	2	0	no
no	1	1	0	no
no	2	2	1	no
no	3	3	0	no
no	2	2	0	no
no	1	1	0	no
no	1	1	0	no
no	1	1	1	no
no	1	1	1	no
no	2	2	0	no
no	1	1	0	no
no	1	1	1	no
no	2	2	0	no
no	1	1	0	no
no	0	0	0	no
no	0	0	0	no
no	2	2	0	no
no	2	1	0	no
no	1	1	1	no
yes	0	0	0	no
no	0	0	0	no
no	1	1	0	no
no	2	2	0	no
no	2	2	0	no
no	3	3	1	no
no	2	3	0	no
no	4	4	0	no
no	0	0	0	no
no	0	0	0	no
no	3	3	1	no
no	1	1	0	no
yes	2	1	1	no
no	0	0	0	no
no	0	0	0	no
no	4	4	0	no
no	1	1	1	no
no	0	0	0	no
no	2	2	0	no
no	1	1	0	no
no	5	5	4	no
no	4	4	0	no
no	5	5	0	no
no	1	1	0	no
no	4	4	0	no
no	3	3	3	no
no	3	3	0	no
no	1	1	0	no
no	1	1	1	no
no	2	2	0	no
no	0	0	0	no
yes	2	2	2	no
no	1	1	0	no

no	1	1	0	no
no	3	3	2	no
no	1	1	0	no
no	2	2	0	no
no	2	2	1	no
no	0	0	0	no
no	3	3	0	no
no	0	0	0	no
no	0	0	0	no
no	0	0	0	no
no	5	5	4	no
no	1	1	0	no
no	6	6	0	no
no	3	3	3	no
no	1	1	1	no
no	0	0	0	no
no	4	4	0	no
no	4	4	4	no
no	1	1	0	no
no	3	3	0	no
no	4	5	5	no
no	3	3	0	no
no	1	1	1	no
no	2	2	0	no
no	1	1	0	no
no	2	2	2	no
no	4	4	0	no
no	2	2	0	no
no	1	1	1	no
no	0	0	0	no
no	2	2	2	no
no	0	0	0	no
no	4	4	0	no
no	5	5	5	no
no	4	4	0	no
no	3	3	0	no
no	3	4	0	no
no	2	2	0	no
no	0	0	0	no
no	1	0	0	no
no	0	0	0	no
no	2	2	2	no
no	3	3	0	no
no	3	3	0	no
no	1	1	1	no
no	0	0	0	no
no	3	3	0	no
no	3	3	2	no
no	3	3	0	no
no	4	4	0	no
no	2	2	2	no
no	2	2	0	no

no	0	0	0	no
no	2	2	2	no
no	1	1	0	no
no	1	1	0	no
no	2	2	2	no
no	2	2	0	no
no	0	0	0	no
no	1	1	1	no
no	4	4	0	no
no	2	2	0	no
no	1	1	1	no
no	0	0	0	no
no	3	3	0	no
no	1	1	1	no
no	1	1	0	no
no	2	2	0	no
no	4	4	4	no
no	3	3	0	no
no	4	4	0	no
no	0	0	0	no
no	0	0	0	no
no	0	0	0	no
no	1	1	1	no
no	2	2	0	no
no	0	0	0	no
no	3	3	3	no
no	5	5	0	no
no	3	3	0	no
no	1	1	1	no
no	0	0	0	no
no	2	2	0	no
no	1	1	1	no
no	1	1	0	no
no	2	2	0	no
no	2	2	2	no
no	5	5	0	no
no	0	0	0	no
no	2	2	1	no
no	1	1	0	no
no	2	2	0	no
no	2	2	2	no
no	2	2	0	no
no	5	5	0	no
no	3	3	3	no
no	2	2	0	no
no	1	1	0	no
no	4	4	4	no
no	3	3	0	no
no	4	4	0	no
no	3	3	3	no
no	2	2	0	no
no	4	4	0	no

no	2	2	2	no
no	1	1	0	no
no	1	1	1	no
no	0	0	0	no
no	1	1	0	no
no	2	2	1	no
no	2	2	0	no
no	4	4	0	no
no	3	3	3	no
no	0	0	0	no
no	3	3	0	no
no	3	3	3	no
no	2	2	0	no
no	3	3	0	no
no	4	4	1	no
no	3	3	0	no
no	2	2	0	no
no	4	4	4	no
no	1	1	0	no
no	5	5	0	no
no	2	2	2	no
no	2	2	0	no
no	0	0	0	no
no	4	4	4	no
no	2	2	0	no
no	2	2	0	no
no	1	1	1	no
no	2	2	0	no
no	0	0	0	no
no	4	4	4	no
no	3	3	0	no
no	5	5	0	no
no	2	2	1	no
no	3	3	0	no
no	3	3	3	no
no	2	2	0	no
no	4	4	0	no
no	0	0	0	no
no	1	1	0	no
no	2	2	0	no
yes	3	3	3	no
no	4	4	0	no
no	0	0	0	no
no	0	0	0	no
no	2	2	0	no
no	0	0	0	no
no	6	6	5	no
no	4	4	0	no
no	3	3	0	no
no	1	1	1	no
no	1	1	0	no
no	1	1	0	no

no	0	0	0	no
no	1	1	0	no
no	2	2	0	no
no	2	2	2	no
no	2	2	0	no
no	2	2	0	no
no	0	0	0	no
no	1	1	0	no
no	1	1	0	no
no	4	4	4	no
no	3	3	0	no
no	2	1	0	no
yes	3	2	2	no
no	0	0	0	no
no	2	2	0	no
no	2	3	3	no
no	3	3	0	no
no	2	3	0	no
no	3	3	3	no
no	1	1	0	no
no	4	4	0	no
no	1	1	1	no
no	1	1	0	no
no	1	1	0	no
no	2	2	1	no
no	2	2	0	no
no	0	0	0	no
no	2	2	2	no
no	1	1	0	no
no	3	3	0	no
no	3	3	2	no
no	1	1	0	no
no	3	2	0	no
no	2	2	2	no
no	1	1	0	no
no	0	0	0	no
no	3	4	4	no
no	1	1	0	no
no	3	4	0	no
no	2	2	2	no
no	1	1	0	no
no	1	1	0	no
no	4	4	1	no
no	4	4	0	no
no	4	4	0	no
no	3	3	3	no
no	5	5	0	no
no	2	1	1	no
no	2	1	0	no
no	2	2	0	no
no	3	2	2	no
no	3	2	0	no

no	4	4	0	no
no	0	0	0	no
no	1	1	0	no
no	1	1	0	no
no	1	1	1	no
no	1	1	0	no
no	2	2	0	no
no	2	2	2	no
no	0	0	0	no
no	4	4	0	no
no	1	1	1	no
no	1	1	0	no
no	1	1	0	no
no	0	0	0	no
no	1	1	0	no
no	3	3	0	no
yes	0	0	0	yes
no	2	2	2	yes
no	2	2	0	no
no	5	5	0	no
no	3	3	2	no
no	2	2	2	no
no	3	3	3	no
no	4	4	0	no
no	3	3	0	no
no	3	3	2	no
no	2	2	0	no
no	2	2	0	no
no	0	0	0	no
no	3	3	1	no
no	2	2	0	no
no	5	5	5	no
no	3	3	0	no
no	3	3	0	no
no	1	1	1	no
no	1	1	0	no
no	1	1	0	no
no	1	1	0	no
no	1	1	1	no
no	1	1	0	no
no	5	5	4	no
no	4	4	0	no
no	2	2	0	no
no	3	3	3	no
no	3	3	0	no
no	1	1	0	no
no	1	1	1	no
no	1	1	0	no
no	0	0	0	no
no	7	7	0	no
no	4	4	4	no
no	3	3	2	no

no	2	2	0	no
no	1	1	0	no
no	4	4	0	no
no	5	5	0	no
no	2	2	1	no
no	2	2	0	no
no	0	0	0	no
no	2	2	0	no
no	1	1	0	no
no	4	4	3	no
no	4	4	0	no
no	1	1	0	no
no	2	2	2	no
no	2	2	0	no
no	5	5	0	no
no	3	3	0	no
no	2	2	0	no
no	6	6	4	no
no	5	5	0	no
no	2	2	2	no
no	2	2	0	no
no	2	2	0	no
no	1	1	1	no
no	2	2	0	no
no	2	2	0	no
no	1	2	1	no
no	1	1	0	no
no	5	5	4	no
no	3	3	0	no
no	4	4	0	no
no	5	5	0	no
no	2	3	0	no
no	4	4	0	no
no	4	3	3	no
no	3	2	0	no
no	4	5	0	no
no	6	5	0	no
no	4	4	4	no
no	3	3	0	no
no	5	5	0	no
no	4	4	3	no
no	2	2	0	no
no	2	2	0	no
no	3	3	2	no
no	2	2	0	no
no	2	2	0	no
no	0	0	0	no
no	3	3	0	no
no	4	4	0	no
no	4	4	4	no
no	3	3	0	no
no	3	3	0	no

no	0	0	0	no
no	0	0	0	no
no	4	4	0	no
no	1	1	1	no
no	3	3	0	no
no	1	1	0	no
no	1	1	1	no
no	4	4	0	no
no	0	0	0	no
no	1	1	1	no
no	1	1	0	no
no	4	3	0	no
no	3	3	3	no
no	2	3	0	no
no	2	2	0	no
no	1	1	1	no
no	1	1	0	no
no	5	5	5	no
no	4	4	0	no
no	2	2	0	no
no	6	6	6	no
no	4	4	0	no
no	1	1	1	no
no	2	2	0	no
no	2	2	2	no
no	6	5	0	no
no	2	2	0	no
no	1	1	0	no
no	2	2	2	no
no	3	3	0	no
no	1	1	0	no
yes	5	5	2	no
no	2	2	0	no
no	3	3	2	no
no	5	5	0	no
no	2	2	0	no
no	1	1	1	no
no	1	1	0	no
no	0	0	0	no
no	0	0	0	no
no	0	0	0	no
no	0	0	0	no
yes	1	1	1	no
no	2	2	0	no
no	0	0	0	no
no	0	0	0	no
no	2	2	1	no
no	2	2	0	no
no	2	2	1	no
no	1	1	0	no
no	0	0	0	no
no	0	0	0	no

no	0	0	0	no
no	2	2	0	no
no	1	1	0	no
no	2	2	1	no
no	3	3	1	no
no	1	1	0	no
no	2	2	0	no
no	4	4	0	no
no	0	0	0	no
no	0	0	0	no
no	0	0	0	no
no	5	5	3	no
no	2	3	0	no
no	8	8	0	no
no	2	2	1	no
no	4	4	0	no
no	5	4	3	no
no	5	4	0	no
no	4	4	4	no
no	2	2	0	no
no	6	6	0	no
yes	3	3	3	no
no	1	1	0	no
no	3	3	0	no
no	0	1	0	no
no	1	1	1	no
no	2	2	0	no
no	0	0	0	no
no	2	2	2	no
no	4	4	0	no
no	5	4	0	no
no	1	1	0	no
no	3	3	0	no
no	3	3	3	no
no	4	4	0	no
no	5	5	0	no
no	4	4	0	no
no	3	3	3	no
no	6	6	6	no
no	2	2	0	no
no	4	4	0	no
no	3	3	3	no
no	3	2	0	no
no	3	4	0	no
no	1	1	1	no
no	0	0	0	no
no	3	3	2	no
no	2	2	2	no
no	2	2	0	no
no	5	5	5	no
no	4	4	0	no
no	6	6	0	no

no	2	2	1	no
no	2	2	0	no
no	2	2	0	no
no	0	0	0	no
no	0	0	0	no
no	4	4	3	no
no	2	3	0	no
no	5	5	0	no
no	0	0	0	no
no	0	0	0	no
no	0	0	0	no
no	3	3	3	no
no	4	4	0	no
no	1	1	0	no
no	2	1	1	no
no	3	1	0	no
no	3	3	0	no
no	2	2	2	no
no	2	2	0	no
no	2	2	0	no
no	7	7	7	no
no	2	2	0	no
no	7	7	0	no
no	1	1	1	no
no	2	1	0	no
no	4	4	0	no
no	4	4	4	no
no	1	1	0	no
no	4	4	0	no
no	1	1	1	no
no	5	5	0	no
no	1	2	0	no
no	3	3	3	no
no	2	2	0	no
no	3	3	0	no
no	2	2	1	no
no	1	1	0	no
no	3	3	0	no
no	4	4	0	no
no	5	5	0	no
no	3	3	1	no
no	1	1	0	no
no	2	2	0	no
no	1	1	0	no
no	1	1	1	no
no	1	1	0	no
no	2	2	0	no
yes	4	4	2	no
no	1	1	0	no
no	2	2	0	no
no	3	3	3	no
no	2	2	0	no

no	4	4	0	no
no	6	6	5	no
no	3	3	0	no
no	2	2	0	no
no	1	1	1	no
no	2	2	0	no
no	1	2	0	no
no	3	3	2	no
no	3	3	0	no
no	1	1	0	no
no	1	1	1	no
no	1	1	0	no
no	3	3	0	no
no	2	2	1	no
no	0	0	0	no
no	4	3	0	no
no	0	0	0	no
no	3	2	0	no
no	1	1	1	no
no	0	0	0	no
no	0	0	0	no
no	1	1	0	no
no	0	0	0	no
no	0	0	0	no
no	1	1	0	no
yes	6	6	5	no
no	5	4	0	no
no	2	2	0	no
no	1	1	1	no
no	0	0	0	no
no	2	2	0	no
no	0	1	1	no
no	1	2	0	no
no	3	3	0	no
no	0	0	0	no
no	1	1	0	no
no	4	4	0	no
no	1	1	1	no
no	1	1	0	no
no	2	2	0	no
no	3	3	3	no
no	2	2	0	no
no	2	2	0	no
no	1	1	1	no
no	2	2	0	no
no	0	0	0	no
no	5	4	3	no
no	3	3	0	no
no	3	2	0	no
no	1	1	1	no
no	4	4	0	no
no	2	2	0	no

no	2	2	2	no
no	2	2	0	no
no	2	2	0	no
no	1	0	0	no
no	1	1	0	no
no	5	5	0	no
no	2	2	2	no
no	0	0	0	no
no	5	5	0	no
no	3	3	3	no
no	3	3	0	no
no	2	2	0	no
no	1	1	1	no
no	2	3	0	no
no	0	1	0	no
no	5	5	4	no
no	0	0	0	no
no	6	6	0	no
no	1	1	1	no
no	2	2	0	no
no	0	0	0	no
yes	1	1	0	no
no	1	1	0	no
no	1	1	0	no
no	2	2	2	no
no	1	1	0	no
no	3	3	0	no
no	2	2	2	no
no	2	2	0	no
no	1	1	0	no
no	2	2	2	no
no	1	1	0	no
no	5	5	0	no
no	2	2	0	no
no	0	0	0	no
no	0	0	0	no
no	2	2	0	no
no	1	1	1	no
no	2	2	0	no
no	5	5	0	no
no	2	2	1	no
no	2	2	0	no
no	2	2	0	no
no	2	2	2	no
no	1	1	0	no
no	2	2	0	no
no	3	3	0	no
no	4	4	0	no
no	1	1	0	no
no	3	3	3	no
no	3	3	0	no
no	2	2	0	no

no	2	2	2	no
no	0	0	0	no
no	1	1	0	no
no	0	0	0	no
no	2	2	0	no
no	1	1	0	no
no	1	1	1	no
no	2	2	0	no
no	1	1	0	no
no	6	6	4	no
no	4	4	0	no
no	6	6	0	no
no	2	2	2	no
no	3	3	0	no
no	1	1	0	no
no	1	1	0	no
no	0	0	0	no
no	0	0	0	no
no	0	0	0	no
no	3	3	3	no
no	2	2	0	no
no	2	2	0	no
no	5	5	5	no
no	3	4	0	no
no	4	4	0	no
no	2	2	1	no
no	1	1	0	no
no	2	2	2	no
no	1	1	0	no
no	2	2	0	no
yes	0	0	0	no
no	2	2	0	no
no	1	1	0	no
no	3	3	3	no
no	2	1	0	no
no	3	4	0	no
no	5	5	3	no
no	1	1	0	no
no	5	5	0	no
no	2	2	1	no
no	3	3	0	no
no	1	1	0	no
no	1	1	1	no
no	4	4	0	no
no	2	2	0	no
no	4	3	3	no
no	4	3	0	no
no	1	1	0	no
no	3	3	3	no
no	2	2	0	no
no	5	5	0	no
no	2	3	2	no

no	2	2	0	no
no	3	4	0	no
no	1	1	1	no
no	2	2	0	no
no	1	1	0	no
no	1	1	1	no
no	2	2	0	no
no	4	2	0	no
no	1	1	1	no
no	2	2	0	no
no	1	1	0	no
no	0	0	0	no
no	0	0	0	no
no	1	1	0	no
no	3	3	2	no
no	4	4	0	no
no	2	2	0	no
no	2	2	2	no
no	3	3	0	no
no	2	2	0	no
no	2	2	2	no
no	1	1	0	no
no	2	2	0	no
no	1	1	1	no
no	1	1	0	no
no	1	1	0	no
no	1	1	0	no
no	1	1	0	no
no	2	2	0	no
no	1	1	0	no
no	0	0	0	no
no	2	2	0	no
no	3	3	3	no
no	0	0	0	no
no	4	4	0	no
no	0	0	0	no
no	2	1	0	no
no	2	2	0	no
no	0	0	0	no
no	0	0	0	no
no	0	0	0	no
no	2	2	1	no
no	1	1	0	no
no	2	2	0	no
no	1	1	0	no
no	1	1	0	no
no	0	0	0	no
no	2	2	2	no
no	2	2	0	no
no	1	1	0	no
no	2	2	2	no
no	1	1	0	no

no	1	1	0	no
no	1	1	1	no
no	2	2	0	no
no	0	0	0	no
no	0	0	0	no
no	0	0	0	no
no	0	0	0	no
no	4	4	4	no
no	2	2	0	no
no	2	2	0	no
no	3	3	3	no
no	4	4	0	no
no	5	5	0	no
no	1	1	1	no
no	1	1	0	no
no	2	2	0	no
no	0	0	0	no
no	0	0	0	no
no	1	1	0	no
no	1	2	2	no
no	2	2	0	no
no	0	1	0	no
no	0	0	0	no
no	0	0	0	no
no	0	0	0	no
no	2	2	2	no
no	3	3	0	no
no	1	1	0	no
no	2	2	2	no
no	1	1	0	no
no	2	2	0	no
no	0	0	0	no
no	2	2	0	no
no	0	0	0	no
no	3	3	3	no
no	2	2	0	no
no	1	1	0	no
no	1	1	1	no
no	1	1	0	no
no	3	3	0	no
no	1	1	1	no
no	0	0	0	no
no	3	3	0	no
no	5	5	5	no
no	4	4	0	no
no	1	1	1	no
no	1	1	0	no
no	2	2	0	no
no	0	0	0	no
no	0	0	0	no
no	1	1	0	no
no	1	1	1	no

no	1	1	0	no
no	1	1	0	no
no	1	1	1	no
no	0	0	0	no
no	2	2	0	no
no	3	3	2	no
no	1	1	0	no
no	3	3	0	no
no	2	2	2	no
no	4	4	0	no
no	2	2	0	no
no	4	4	0	no
no	4	3	3	no
no	4	3	0	no
no	4	4	0	no
no	2	2	1	no
no	3	3	0	no
no	4	4	3	no
no	1	1	1	yes
no	1	1	1	yes
no	1	1	0	no
no	3	3	0	no
yes	5	5	3	yes
no	2	2	0	no
no	4	4	0	no
no	1	1	1	yes
no	2	2	0	no
no	2	2	0	no
no	4	4	3	yes
no	0	0	0	no
no	4	4	0	no
no	3	3	3	yes
no	3	3	0	no
no	3	3	0	no
no	3	3	3	yes
no	3	3	0	no
no	0	0	0	no
no	1	1	0	yes
no	1	1	0	no
no	4	4	0	no
no	3	3	2	no
no	3	3	0	no
no	2	2	0	no
no	1	0	0	yes
no	1	0	0	no
no	2	2	0	no
yes	5	5	2	yes
no	4	4	0	no
no	0	0	0	yes
no	1	1	0	no
no	2	2	0	no
no	2	2	2	yes

no	4	4	0	no
no	0	0	0	no
no	3	3	3	yes
no	1	1	0	no
no	6	6	0	no
no	1	1	1	yes
no	1	1	0	no
no	1	2	0	no
no	0	0	0	yes
no	2	2	0	no
no	1	1	0	no
no	0	0	0	yes
no	2	2	1	yes
no	1	1	0	no
no	3	3	0	no
no	1	1	1	yes
no	2	2	0	no
no	4	4	0	no
no	0	0	0	yes
no	0	0	0	no
no	4	4	0	no
no	2	2	2	yes
no	3	3	0	no
no	1	1	0	no
no	3	3	3	yes
no	5	5	0	no
no	1	1	0	no
no	3	3	2	yes
no	2	2	0	no
no	0	0	0	yes
no	1	1	0	no
no	1	1	1	yes
no	1	1	0	no
no	1	1	0	no
no	0	0	0	yes
no	0	0	0	no
no	1	1	0	no
no	2	2	1	yes
no	4	4	0	no
no	1	1	0	no
no	5	5	0	yes
no	2	2	2	yes
no	4	4	0	no
no	4	4	4	yes
no	5	5	0	no
no	2	2	0	no
no	3	3	0	yes
no	4	4	0	no
no	5	5	0	no
no	4	4	4	yes
no	5	5	0	no
no	4	4	0	no

no	4	4	4	yes
no	2	2	0	no
no	1	1	0	no
no	1	1	0	yes
no	0	0	0	no
no	4	4	0	no
no	3	3	2	yes
no	3	3	0	no
no	2	2	0	no
no	1	1	1	yes
no	1	1	0	no
no	1	1	0	no
yes	1	1	0	yes
no	0	0	0	no
no	1	0	0	no
no	1	1	1	yes
no	1	0	0	no
no	3	3	0	no
no	2	2	1	yes
no	0	0	0	no
no	1	2	0	no
no	1	2	0	yes
no	0	0	0	yes
no	2	3	0	no
no	0	0	0	no
no	0	0	0	yes
no	0	0	0	no
no	4	4	0	yes
no	4	4	0	no
no	2	2	1	yes
no	3	3	0	no
no	1	2	0	no
no	6	6	5	yes
no	8	8	0	no
no	2	2	0	no
no	1	1	0	yes
no	0	0	0	no
no	1	1	0	no
no	4	4	3	yes
no	2	2	0	no
no	1	1	0	no
no	4	4	3	yes
no	3	3	0	no
no	2	2	0	no
no	0	0	0	yes
no	1	1	0	no
no	0	0	0	no
no	2	2	0	yes
no	1	1	1	yes
no	1	1	0	no
no	1	1	0	no
no	2	2	0	yes

no	1	1	1	yes
no	0	0	0	no
no	2	1	0	no
no	2	2	2	yes
no	1	1	0	no
no	1	1	0	no
no	3	3	3	yes
no	3	3	0	no
no	1	1	0	no
yes	1	1	1	yes
no	0	0	0	no
no	3	3	0	no
no	6	7	7	yes
no	3	4	0	no
no	7	7	0	no
no	1	1	1	yes
no	1	1	0	no
no	1	1	0	no
no	1	1	1	yes
no	0	0	0	no
no	1	1	0	no
no	1	1	1	yes
no	2	2	0	no
no	1	1	0	no
no	3	2	2	yes
no	1	1	0	no
no	2	2	0	no
no	2	2	2	no
no	1	1	0	no
no	2	2	0	no
no	2	2	1	yes
no	2	2	0	no
no	0	0	0	yes
no	1	1	0	no
no	0	0	0	no
no	1	1	0	yes
no	2	2	0	no
no	3	3	3	no
no	2	1	0	no
no	4	4	0	no
no	0	0	0	no
no	0	0	0	no
no	0	0	0	no
no	1	1	1	no
no	1	1	0	no
no	4	3	0	no
no	2	3	3	no
no	3	3	0	no
no	2	3	0	no
no	0	0	0	no
no	0	0	0	no
no	0	0	0	no

no	3	3	0	no
no	2	2	0	no
no	4	4	0	no
no	0	0	0	no
no	1	1	0	no
no	2	2	0	no
no	1	1	0	no
no	1	1	0	no
no	5	5	0	no
no	1	1	0	no
no	1	1	0	no
no	2	2	2	no
no	2	2	0	no
no	2	2	0	no
no	0	0	0	no
no	3	3	3	no
no	4	4	0	no
no	3	3	0	no
no	0	0	0	no
no	6	5	0	no
no	1	0	0	no
no	0	0	0	no
no	2	1	0	no
no	2	2	0	no
no	2	2	0	no
no	2	2	2	no
no	1	1	0	no
no	1	1	0	no
no	1	1	0	no
no	1	1	0	no
no	4	4	4	no
no	3	3	0	no
no	2	2	0	no
no	1	1	1	no
no	1	1	0	no
no	1	1	0	no
no	0	0	0	no
no	1	1	0	no
no	1	1	0	no
yes	4	4	4	no
no	1	1	0	no
no	4	4	0	no
no	1	1	0	no
no	1	1	0	no
no	0	0	0	no
no	3	3	3	no
no	3	3	0	no
no	3	3	0	no
no	0	0	0	no
no	0	0	0	no
no	4	4	0	no
no	2	2	2	no

no	3	3	0	no
no	3	3	0	no
no	2	2	2	no
no	1	1	0	no
no	2	2	0	no
no	0	0	0	no
no	0	0	0	no
no	0	0	0	no
no	0	0	0	no
no	2	2	0	no
no	1	1	0	no
no	3	3	2	no
no	1	1	0	no
no	3	3	0	no
no	5	5	0	no
no	2	1	1	no
no	3	2	0	no
no	3	2	0	no
no	1	1	1	no
no	1	1	0	no
no	1	1	0	no
no	1	1	1	no
no	2	2	0	no
no	0	0	0	no
no	2	2	2	no
no	3	3	0	no
no	1	1	0	no
no	2	2	2	no
no	1	1	0	no
no	3	3	0	no
no	1	1	1	no
no	0	0	0	no
no	1	1	0	no
no	1	1	1	no
no	1	1	0	no
no	2	2	1	no
no	2	2	0	no
no	2	2	1	no
no	3	3	0	no
no	2	2	0	no
no	3	3	3	no
no	2	2	0	no
no	2	2	0	no
no	0	0	0	no
no	0	0	0	no
no	2	2	0	no
no	0	0	0	no
no	2	2	0	no
no	1	1	0	no
no	3	3	2	no
no	4	4	0	no
no	2	2	0	no

no	1	1	1	no
no	2	2	0	no
no	1	1	0	no
no	1	1	0	no
no	2	2	2	no
no	2	2	0	no
no	2	2	0	no
no	2	3	3	no
no	3	3	0	no
no	2	3	0	no
no	5	5	5	no
no	4	3	0	no
no	1	1	0	no
no	1	2	2	no
no	1	2	0	no
no	1	1	0	no
no	3	3	2	no
no	2	3	2	no
no	3	3	0	no
no	0	0	0	no
no	1	1	1	no
no	1	1	1	no
no	4	4	0	no
no	0	0	0	no
no	1	1	1	no
no	0	0	0	no
no	0	0	0	no
no	0	0	0	no
no	1	1	0	no
no	0	0	0	no
no	0	0	0	no
no	4	4	0	no
no	1	1	1	no
no	3	3	0	no
no	1	1	1	no
no	1	1	0	no
no	1	1	1	no
no	0	0	0	no
no	1	1	0	no
no	2	2	2	no
no	1	1	0	no
no	2	2	0	no
no	2	2	2	no
no	2	2	0	no
no	3	3	0	no
no	1	2	0	no
no	1	1	0	no
no	2	2	0	no
no	3	3	0	no

in_Montenegro2019	in_Satterstrom2020	in_Neri2021	in_Costa2022	in_Fu2022	in_Costa2023
no	no	no	no	yes	no
no	no	no	no	yes	no
no	no	no	no	yes	no
no	no	no	yes	yes	no
no	no	no	no	yes	no
no	no	no	no	yes	no
no	no	no	no	yes	no
no	no	no	no	yes	no
no	no	no	no	yes	no
no	no	no	no	yes	no
no	no	no	no	yes	no
no	no	no	no	yes	no
no	no	no	no	yes	no
no	no	no	no	yes	no
no	no	no	no	yes	no
no	no	no	no	yes	no
no	no	no	no	yes	no
no	no	no	no	yes	no
no	no	no	no	yes	no
no	no	no	no	yes	no
no	no	no	no	yes	no
no	no	no	no	yes	no
no	no	no	no	yes	no
no	no	no	no	yes	no
no	no	no	no	yes	no
no	no	no	no	yes	no
no	no	no	no	yes	no
no	no	no	yes	yes	no
no	no	no	no	yes	no
no	no	no	no	yes	no
no	no	no	no	no	no
no	no	no	no	no	no
no	no	no	no	no	no
no	no	no	yes	yes	no
no	no	no	no	yes	no
no	no	no	no	yes	no
no	no	no	yes	yes	no
no	no	no	no	yes	no
no	no	no	no	yes	no
no	no	no	no	no	no
no	no	no	no	no	no
no	no	no	no	no	no
no	no	no	yes	yes	no
no	no	no	no	yes	no
no	no	no	no	yes	no
no	no	no	no	yes	no
no	no	no	no	yes	no
no	no	no	no	yes	no
no	no	no	yes	yes	no
no	no	no	no	yes	no

no	no	no	no	yes	no
no	no	no	yes	yes	no
no	no	no	no	yes	no
no	no	no	no	yes	no
no	no	no	no	yes	no
no	no	no	no	yes	no
no	no	no	no	yes	no
no	no	no	no	yes	no
no	no	no	no	yes	no
no	no	no	no	yes	no
no	no	no	no	yes	no
no	no	no	no	yes	no
no	no	no	no	yes	no
no	no	no	no	yes	no
no	no	no	no	yes	no
no	no	no	no	yes	no
no	no	no	yes	yes	no
no	no	no	no	yes	no
no	no	no	no	yes	no
no	no	no	no	yes	no
no	no	no	no	yes	no
no	no	no	no	yes	no
no	no	no	no	yes	no
no	no	no	no	yes	no
no	no	no	no	yes	no
no	no	no	no	yes	no
no	no	no	no	yes	no
no	no	no	no	yes	no
no	no	no	no	yes	no
no	no	no	no	yes	no
no	no	no	no	yes	no
no	no	no	no	yes	no
no	no	no	no	yes	no
no	no	no	no	yes	no
no	no	no	no	yes	no
no	no	no	no	yes	no
no	no	no	no	yes	no
no	no	no	no	yes	no
no	no	no	no	yes	no
no	no	no	no	yes	no
no	no	no	no	yes	no
no	no	no	no	yes	no
no	no	no	no	yes	no
no	no	no	no	yes	no
no	no	no	no	yes	no
no	no	no	no	yes	no
no	no	no	no	yes	no
no	no	no	no	yes	no
no	no	no	no	yes	no
no	no	no	no	yes	no
no	no	no	no	yes	no
no	no	no	no	yes	no

no	no	no	no	yes	no
no	no	no	no	yes	no
no	no	no	no	yes	no
no	no	no	no	yes	no
no	no	no	no	yes	no
no	no	no	no	yes	no
no	no	no	no	yes	no
no	no	no	no	yes	no
no	no	no	no	yes	no
no	no	no	yes	yes	no
no	no	no	no	yes	no
no	no	no	no	yes	no
no	no	no	yes	yes	no
no	no	no	no	yes	no
no	no	no	no	yes	no
no	no	no	no	yes	no
no	no	no	no	yes	no
no	no	no	no	yes	no
no	no	no	no	yes	no
no	no	no	no	yes	no
no	no	no	no	yes	no
no	no	no	yes	yes	no
no	no	no	no	yes	no
no	no	no	no	yes	no
no	no	no	yes	yes	no
no	no	no	no	yes	no
no	no	no	no	yes	no
no	no	no	no	yes	no
no	no	no	no	yes	no
no	no	no	no	yes	no
no	no	no	no	yes	no
no	no	no	no	yes	no
no	no	no	no	yes	no
no	no	no	no	yes	no
no	no	no	no	yes	no
no	no	no	no	yes	no
no	no	no	no	yes	no
no	no	no	no	yes	no
no	no	no	no	yes	no
no	no	no	yes	yes	no
no	no	no	no	yes	no
no	no	no	no	yes	no
no	no	no	no	yes	no
no	no	no	no	yes	no
no	no	no	no	yes	no
no	no	no	no	yes	no
no	no	no	no	yes	no
no	no	no	no	yes	no
no	no	no	no	yes	no
no	no	no	no	yes	no
no	no	no	no	yes	no
no	no	no	no	yes	no

no	no	no	no	yes	no
no	no	no	no	yes	no
no	no	no	no	yes	no
no	no	no	no	yes	no
no	no	no	no	yes	no
no	no	no	no	yes	no
no	no	no	no	yes	no
no	no	no	no	yes	no
no	no	no	no	yes	no
no	no	no	no	yes	no
no	no	no	no	yes	no
no	no	no	no	yes	no
no	no	no	no	yes	no
no	no	no	no	yes	no
no	no	yes	no	no	no
no	no	yes	no	no	no
no	no	yes	no	no	no
no	no	yes	no	no	no
no	no	yes	no	no	no
no	no	yes	no	no	no
no	no	yes	no	no	no
no	no	no	no	no	no
no	no	yes	no	no	no
no	no	no	no	no	no
no	no	yes	no	no	no
no	no	yes	no	no	no
no	no	yes	no	no	no
no	no	yes	no	no	no
no	no	yes	no	no	no
no	no	no	no	no	no
no	no	no	no	no	no
no	no	yes	no	no	no
no	no	yes	no	no	no
no	no	yes	no	no	no
no	no	yes	no	no	no
no	no	yes	no	no	no
no	no	yes	no	no	no
no	no	yes	no	no	no
no	no	yes	no	no	no
no	no	yes	no	no	no
no	no	yes	no	no	no
no	no	yes	no	no	no
no	no	yes	no	no	no
no	no	yes	no	no	no
no	no	yes	no	no	no
no	no	yes	no	no	no
no	no	yes	no	no	no
no	no	yes	no	no	no
no	no	yes	no	no	no
no	no	yes	no	no	no
no	no	yes	no	no	no
no	no	yes	no	no	no

no	no	yes	no	no	no
no	no	yes	no	no	no
no	no	yes	no	no	no
no	no	yes	no	no	no
no	no	yes	no	no	no
no	no	yes	no	no	no
no	no	yes	no	no	no
no	no	yes	no	no	no
no	no	yes	no	no	no
no	no	yes	no	no	no
no	no	yes	no	no	no
no	no	yes	no	no	no
no	no	yes	no	no	no
no	no	yes	no	no	no
no	no	yes	no	no	no
no	no	yes	no	no	no
no	no	yes	no	no	no
no	no	yes	no	no	no
no	no	yes	no	no	no
no	no	yes	no	no	no
no	no	yes	no	no	no
no	no	yes	no	no	no
no	no	yes	no	no	no
no	no	yes	no	no	no
no	no	yes	no	no	no
no	no	no	no	yes	no
no	no	no	no	yes	no
no	no	no	no	yes	no
no	no	yes	no	no	no
no	no	yes	no	no	no
no	no	yes	no	no	no
no	no	yes	no	no	no
no	no	yes	no	no	no
no	no	no	no	yes	no
no	no	no	no	yes	no
no	no	no	no	yes	no
no	no	yes	no	no	no
no	no	yes	no	no	no
no	no	no	no	yes	no
no	no	no	no	yes	no
no	no	no	no	yes	no
no	no	no	no	yes	no
no	no	no	no	yes	no
no	no	no	no	yes	no
no	no	no	no	yes	no
no	no	no	no	yes	no
no	no	no	no	yes	no
no	no	no	no	yes	no
no	no	no	no	yes	no
no	no	no	no	yes	no
no	no	no	no	yes	no
no	no	no	no	yes	no

no	no	no	no	yes	no
no	no	no	no	yes	no
no	no	no	no	yes	no
no	no	no	no	yes	no
no	no	no	no	yes	no
no	no	no	no	yes	no
no	no	no	no	yes	no
no	no	no	no	yes	no
no	no	no	no	yes	no
no	no	no	no	yes	no
no	no	no	yes	yes	no
no	no	no	no	yes	no
no	no	no	no	yes	no
no	no	no	no	yes	no
no	no	no	no	yes	no
no	no	no	no	yes	no
no	no	no	yes	yes	no
no	no	no	no	yes	no
no	no	no	no	yes	no
no	no	no	no	yes	no
no	no	no	no	yes	no
no	no	no	no	yes	no
no	no	no	yes	yes	no
no	no	no	no	yes	no
no	no	no	no	yes	no
no	no	no	no	yes	no
no	no	no	no	yes	no
no	no	no	no	yes	no
no	no	no	no	yes	no
no	no	no	no	yes	no
no	no	no	no	yes	no
no	no	no	no	yes	no
no	no	no	no	yes	no
no	no	no	no	yes	no
no	no	no	yes	yes	no
no	no	no	no	yes	no
no	no	no	no	yes	no
no	no	no	no	no	no
no	no	no	no	no	no
no	no	no	no	no	no
no	no	no	no	yes	no
no	no	no	no	yes	no
no	no	no	no	yes	no
no	no	no	no	yes	no
no	no	no	no	yes	no
no	no	no	no	yes	no
no	no	no	no	yes	no
no	no	no	no	yes	no
no	no	no	no	yes	no
no	no	no	no	no	no
no	no	no	no	no	no
no	no	no	no	no	no

no	no	no	no	no	no
no	no	no	no	no	no
no	no	no	no	yes	no
no	no	no	no	yes	no
no	no	no	no	yes	no
no	no	no	no	yes	no
no	no	no	no	yes	no
no	no	no	no	yes	no
no	no	no	no	yes	no
no	no	no	no	yes	no
no	no	no	no	yes	no
no	no	no	no	yes	no
no	no	no	no	yes	no
no	no	no	no	yes	no
no	no	no	no	yes	no
no	no	no	no	yes	no
no	no	no	no	yes	no
no	no	no	no	yes	no
no	no	no	no	yes	no
no	no	no	no	yes	no
no	no	no	no	yes	no
no	no	no	no	yes	no
no	no	no	no	yes	no
no	no	no	yes	yes	no
no	no	no	no	yes	no
no	no	no	no	yes	no
no	no	no	yes	yes	no
no	no	no	no	yes	no
no	no	no	no	yes	no
no	no	no	yes	yes	no
no	no	no	no	yes	no
no	no	no	no	yes	no
no	no	no	no	no	no
no	no	no	no	no	no
no	no	no	no	yes	no
no	no	no	no	yes	no
no	no	no	no	yes	no
no	no	no	yes	yes	no
no	no	no	no	yes	no
no	no	no	no	yes	no
no	no	no	yes	no	no
no	no	no	no	no	no
no	no	no	no	no	no
no	no	no	yes	yes	no
no	no	no	no	yes	no
no	no	no	no	yes	no
no	no	no	no	yes	no
no	no	no	no	yes	no
no	no	no	no	yes	no
no	no	no	no	yes	no
no	no	no	no	yes	no
no	no	no	no	yes	no

no	no	no	no	yes	no
no	no	no	no	yes	no
no	no	no	no	yes	no
no	no	no	no	yes	no
no	no	no	no	yes	no
no	no	no	no	yes	no
no	no	no	no	yes	no
no	no	no	no	yes	no
no	no	no	no	yes	no
no	no	no	no	yes	no
no	no	no	no	yes	no
no	no	no	no	yes	no
no	no	no	no	yes	no
no	no	no	no	yes	no
no	no	no	no	yes	no
no	no	no	no	yes	no
no	no	no	no	yes	no
no	no	no	no	yes	no
no	no	no	no	yes	no
no	no	no	no	yes	no
no	no	no	no	yes	no
no	no	no	no	yes	no
no	no	no	no	yes	no
no	no	no	no	yes	no
no	no	no	yes	yes	no
no	no	no	no	yes	no
no	no	no	no	yes	no
no	no	no	no	yes	no
no	no	no	no	yes	no
no	no	no	no	yes	no
no	no	no	no	yes	no
no	no	no	no	yes	no
no	no	no	no	yes	no
no	no	no	no	yes	no
no	no	no	no	yes	no
no	no	no	no	yes	no
no	no	no	yes	yes	no
no	no	no	no	yes	no
no	no	no	no	yes	no
no	no	no	no	yes	no
no	no	no	no	yes	no
no	no	no	no	yes	no
no	no	no	no	yes	no
no	no	no	no	yes	no
no	no	no	no	yes	no
no	no	no	no	no	no
no	no	no	no	no	no
no	no	no	yes	yes	no
no	no	no	no	yes	no
no	no	no	no	yes	no
no	no	no	yes	yes	no
no	no	no	no	yes	no

no	no	no	no	yes	no
no	no	no	no	yes	no
no	no	no	no	yes	no
no	no	no	no	yes	no
no	no	no	yes	yes	no
no	no	no	no	yes	no
no	no	no	no	yes	no
no	no	no	no	yes	no
no	no	no	no	yes	no
no	no	no	no	yes	no
no	no	no	no	yes	no
no	no	no	no	yes	no
no	no	no	no	yes	no
no	no	no	no	yes	no
no	no	no	no	yes	no
no	no	no	no	yes	no
no	no	no	no	no	no
no	no	no	yes	no	no
no	no	no	no	no	no
no	no	no	no	no	no
no	no	no	yes	no	no
no	no	no	no	no	no
no	no	no	no	no	no
no	no	no	no	no	no
no	no	no	no	no	no
no	no	no	no	no	no
no	no	no	no	no	no
no	no	no	no	no	no
no	no	no	no	no	no
no	no	no	no	no	no
no	no	no	no	no	no
no	no	no	yes	no	no
no	no	no	no	no	no
no	no	no	no	no	no
no	no	no	no	no	no
no	no	no	no	no	no
no	no	no	no	no	no
no	no	no	no	no	no
no	no	no	no	no	no
no	no	no	no	no	no
no	no	no	yes	no	yes
no	no	no	no	no	yes
no	no	no	no	no	yes
no	no	no	yes	no	no
no	no	no	no	no	no
no	no	no	no	no	no
no	no	no	yes	no	no
no	no	no	no	no	no
no	no	no	no	no	no
no	no	no	no	no	no
no	no	no	no	no	no
no	no	no	yes	no	no

no	no	no	no	no	no
no	no	no	no	no	no
no	no	no	yes	no	no
no	no	no	no	no	no
no	no	no	yes	no	yes
no	no	no	no	no	yes
no	no	no	yes	no	no
no	no	no	no	no	no
no	no	no	no	no	no
no	no	no	yes	no	yes
no	no	no	no	no	yes
no	no	no	no	no	yes
no	no	no	no	no	no
no	no	no	no	no	no
no	no	no	no	no	no
no	no	no	no	no	no
no	no	no	no	no	no
no	no	no	no	no	no
no	no	no	no	no	no
no	no	no	yes	no	no
no	no	no	no	no	no
no	no	no	no	no	no
no	no	no	no	no	no
no	no	no	no	no	no
no	no	no	no	no	no
no	no	no	no	no	no
no	no	no	no	no	no
no	no	no	no	no	no
no	no	no	no	no	no
no	no	no	no	no	no
no	no	no	no	no	no
no	no	no	no	no	no
no	no	no	no	no	no
no	no	no	no	no	no
no	no	no	no	no	no
no	no	no	no	no	no
no	no	no	no	no	no
no	no	no	yes	no	no
no	no	no	no	no	no
no	no	no	no	no	no
no	no	no	no	no	no
no	no	no	no	no	no
no	no	no	no	no	no
no	no	no	yes	no	no
no	no	no	no	no	no
no	no	no	no	no	no
no	no	no	no	no	no
no	no	no	no	no	no
no	no	no	no	no	no
no	no	no	no	no	no
no	no	no	no	no	no
no	no	no	no	no	no

no	no	no	no	no	no
no	no	no	no	no	no
no	no	no	no	no	no
no	no	no	yes	no	no
no	no	no	no	no	no
no	no	no	no	no	no
no	no	no	yes	no	no
no	no	no	no	no	no
no	no	no	no	no	no
no	no	no	yes	no	no
no	no	no	no	no	no
no	no	no	no	no	no
no	no	no	no	no	no
no	no	no	no	no	no
no	no	no	no	no	no
no	no	no	no	no	no
no	no	no	no	no	no
no	no	no	yes	no	no
no	no	no	no	no	no
no	no	no	no	no	no
no	no	no	no	no	no
no	no	no	no	no	no
no	no	no	no	no	no
no	no	no	no	no	no
no	no	no	no	no	no
no	no	no	no	no	no
no	no	no	no	no	no
no	no	no	no	no	no
no	no	no	no	no	no
no	no	no	no	no	no
no	no	no	no	no	no
no	no	no	no	no	no
no	no	no	no	no	no
no	no	no	no	no	no
no	no	no	no	no	no
no	no	no	no	no	no
no	no	no	no	no	no
no	no	no	no	no	no
no	no	no	no	no	no
no	no	no	yes	no	no
no	no	no	no	no	no
no	no	no	no	no	no
no	no	no	no	no	no
no	no	no	no	no	no
no	no	no	no	no	no
no	no	no	no	no	no
no	no	no	no	no	no
no	no	no	no	no	no
no	no	no	no	no	no
no	no	no	no	no	no
no	no	no	no	no	no
no	no	no	no	no	no

no	no	no	no	no	no
no	no	no	no	no	no
no	no	no	no	no	no
no	no	no	no	no	no
no	no	no	no	no	no
no	no	no	no	no	no
no	no	no	no	no	no
no	no	no	no	no	no
no	no	no	yes	no	no
no	no	no	no	no	no
no	no	no	no	no	no
no	no	no	no	yes	no
no	no	no	no	yes	no
no	no	no	no	yes	no
no	no	no	no	no	no
no	no	no	no	no	no
no	no	no	no	no	no
no	no	no	no	no	no
no	no	no	no	no	no
no	no	no	no	no	no
no	no	no	no	no	no
no	no	no	no	no	no
no	no	no	no	no	no
no	no	no	no	no	no
no	no	no	no	no	no
no	no	no	no	no	no
no	no	no	no	no	no
no	no	no	no	no	no
no	no	no	no	no	no
no	no	no	no	no	no
no	no	no	no	no	no
no	no	no	no	no	no
no	no	no	no	no	no
no	no	no	no	no	no
no	no	no	no	no	no
no	no	no	no	no	no
no	no	no	no	no	no
no	no	no	no	no	no
no	no	no	no	no	no
no	no	no	no	no	no
no	no	no	no	no	no
no	no	no	no	no	no
no	no	no	no	no	no
no	no	no	no	no	no
no	no	no	no	no	no
no	no	no	no	no	no
no	no	no	no	no	no
no	no	no	no	no	no
no	no	no	no	no	no
no	no	no	no	no	no
no	no	no	no	no	no
no	no	no	no	no	no

no	no	no	no	no	no
no	no	no	no	no	no
no	no	no	no	no	no
no	no	no	no	no	no
no	no	no	no	no	no
no	no	no	no	no	no
no	no	no	no	no	no
no	no	no	no	no	no
no	no	no	no	no	no
no	no	no	no	no	no
no	no	no	no	no	no
no	no	no	no	no	no
no	no	no	no	no	no
no	no	no	no	no	no
no	no	no	no	no	no
no	no	no	no	no	no
no	no	no	no	no	no
no	no	no	no	no	no
no	no	no	no	no	no
no	no	no	no	no	no
no	no	no	no	no	no
no	no	no	no	no	no
no	no	no	no	no	no
no	no	no	no	no	no
no	no	no	no	no	no
no	no	no	no	no	no
no	no	no	no	no	no
no	no	no	no	no	no
no	no	no	no	no	no
no	no	no	no	no	no
no	no	no	no	no	no
no	no	no	no	no	no
no	no	no	no	no	no
no	no	no	no	no	no
no	no	no	no	no	no
no	no	no	no	no	no
no	no	no	no	no	no
no	no	no	no	no	no
no	no	no	no	no	no
no	no	no	no	no	no
no	no	no	no	no	no
no	yes	no	no	yes	no
no	yes	no	no	yes	no
no	yes	no	no	yes	no
no	yes	no	no	yes	no
no	yes	no	no	yes	no
no	yes	no	no	yes	no
no	yes	no	no	yes	no
no	yes	no	no	yes	no
no	yes	no	no	yes	no
no	yes	no	no	yes	no
no	yes	no	no	yes	no

no	yes	no	no	yes	no
no	yes	no	no	yes	no
no	yes	no	no	yes	no
no	yes	no	no	yes	no
no	yes	no	no	yes	no
no	yes	no	no	yes	no
no	yes	no	no	yes	no
no	yes	no	no	yes	no
no	yes	no	no	yes	no
no	yes	no	no	yes	no
no	yes	no	no	yes	no
no	yes	no	no	yes	no
no	yes	no	no	yes	no
no	yes	no	no	yes	no
no	yes	no	no	yes	no
no	yes	no	no	yes	no
no	yes	no	no	yes	no
no	yes	no	no	yes	no
no	yes	no	no	yes	no
no	no	no	no	yes	no
no	no	no	no	yes	no
no	no	no	no	yes	no
no	yes	no	no	yes	no
no	yes	no	no	yes	no
no	yes	no	no	yes	no
no	yes	no	no	yes	no
no	yes	no	no	yes	no
no	yes	no	no	yes	no
no	no	no	no	yes	no
no	no	no	no	yes	no
no	no	no	no	yes	no
no	yes	no	no	yes	no
no	yes	no	no	yes	no
no	yes	no	no	yes	no
no	yes	no	no	yes	no
no	yes	no	no	yes	no
no	yes	no	no	yes	no
no	no	no	no	yes	no
no	no	no	no	yes	no
no	no	no	no	yes	no
no	no	no	no	no	no
no	no	no	no	no	no
no	no	no	no	no	no
no	no	no	no	yes	no
no	no	no	no	yes	no
no	no	no	no	yes	no
no	yes	no	no	yes	no
no	yes	no	no	yes	no
no	yes	no	no	yes	no
no	yes	no	no	yes	no
no	yes	no	no	yes	no
no	yes	no	no	yes	no

no	yes	no	yes	yes	no
no	yes	no	no	yes	no
no	yes	no	no	yes	no
no	no	no	no	yes	no
no	no	no	no	yes	no
no	no	no	no	yes	no
no	yes	no	yes	yes	no
no	yes	no	no	yes	no
no	yes	no	no	yes	no
no	no	no	yes	no	no
no	no	no	no	no	no
no	no	no	no	no	no
no	yes	no	no	yes	no
no	yes	no	no	yes	no
no	yes	no	no	yes	no
no	yes	no	no	yes	no
no	yes	no	no	yes	no
no	yes	no	no	yes	no
no	yes	no	no	yes	no
no	yes	no	no	yes	no
no	yes	no	no	yes	no
no	no	no	no	yes	no
no	no	no	no	yes	no
no	no	no	no	yes	no
no	yes	no	no	yes	no
no	yes	no	no	yes	no
no	yes	no	no	yes	no
no	yes	no	no	yes	no
no	yes	no	no	yes	no
no	yes	no	no	yes	no
no	yes	no	no	yes	no
no	yes	no	no	yes	no
no	yes	no	no	yes	no
no	yes	no	no	yes	no
no	yes	no	no	yes	no
no	yes	no	no	yes	no
no	yes	no	no	yes	no
no	yes	no	no	yes	no
no	yes	no	no	yes	no
no	yes	no	no	yes	no
no	yes	no	no	yes	no
no	yes	no	no	yes	no
no	yes	no	no	yes	no
no	yes	no	no	yes	no
no	yes	no	no	yes	no
no	yes	no	no	yes	no
no	yes	no	no	no	no
no	yes	no	no	no	no
no	yes	no	no	no	no
no	yes	no	no	yes	no
no	yes	no	no	yes	no
no	yes	no	no	yes	no

no	yes	no	no	yes	no
no	yes	no	no	yes	no
no	yes	no	no	yes	no
no	yes	no	no	yes	no
no	yes	no	no	yes	no
no	yes	no	no	yes	no
no	no	no	no	no	yes
no	no	no	no	no	yes
no	no	no	no	no	yes
no	no	no	no	yes	no
no	no	no	no	yes	no
no	no	no	no	yes	no
no	no	no	no	yes	no
no	no	no	no	yes	no
no	no	no	no	yes	no
no	no	no	yes	no	no
no	no	no	yes	no	yes
no	no	no	no	no	yes
no	no	no	no	no	yes
yes	no	no	no	no	no
yes	no	no	no	no	no
no	no	no	no	no	no
no	no	no	no	yes	no
no	no	no	no	yes	no
no	no	no	no	yes	no
no	no	no	yes	no	no
no	no	no	no	no	no
no	no	no	yes	no	yes
no	no	no	no	no	yes
no	no	no	no	no	yes
no	no	no	no	yes	no
no	no	no	no	yes	no
no	no	no	no	yes	no
no	no	no	no	yes	no
no	no	no	no	yes	no
no	no	no	no	yes	no
no	no	no	no	yes	no
no	no	no	no	yes	no
no	no	no	no	yes	no
no	no	no	yes	no	yes
no	no	no	no	no	yes
no	no	no	no	no	yes
no	no	no	yes	no	yes
no	no	no	no	no	yes
no	no	no	no	no	yes
no	no	no	no	yes	no
no	no	no	no	yes	no
no	no	no	no	yes	no
no	no	no	yes	no	yes
no	no	no	no	no	yes
no	no	no	no	no	yes
no	no	no	no	yes	no

no	no	no	no	yes	no
no	no	no	no	yes	no
no	no	no	no	yes	no
no	no	no	no	yes	no
no	no	no	no	yes	no
no	no	no	no	yes	no
no	no	no	no	yes	no
no	no	no	no	yes	no
no	no	no	no	yes	no
no	no	no	no	yes	no
no	no	no	no	yes	no
no	no	no	yes	no	yes
no	no	no	no	no	yes
no	no	no	no	no	yes
no	no	no	no	yes	no
no	no	no	no	yes	no
no	no	no	no	yes	no
no	no	no	no	yes	no
no	no	no	no	yes	no
no	no	no	no	yes	no
no	no	no	no	yes	no
no	no	no	no	yes	no
no	no	no	no	yes	no
no	no	no	no	yes	no
no	no	no	no	yes	no
no	no	no	no	yes	no
no	no	no	no	no	yes
no	no	no	no	no	yes
no	no	no	no	no	yes
no	no	no	yes	yes	no
no	no	no	no	yes	no
no	no	no	no	yes	no
no	no	no	yes	no	yes
no	no	no	no	no	yes
no	no	no	no	no	yes
no	no	no	no	yes	no
no	no	no	no	yes	no
no	no	no	no	yes	no
no	no	no	yes	no	yes
no	no	no	no	no	yes
no	no	no	no	no	yes
no	no	no	yes	no	yes
no	no	no	no	no	yes
no	no	no	no	no	yes
no	no	no	no	yes	no
no	no	no	no	yes	no
no	no	no	no	yes	no
no	no	no	no	yes	no
no	no	no	no	yes	no
no	no	no	no	yes	no
no	no	no	no	yes	no
no	no	no	no	yes	no

no	no	no	no	yes	no
no	no	no	no	yes	no
no	no	no	no	yes	no
no	no	no	no	yes	no
no	no	no	yes	no	yes
no	no	no	no	no	yes
no	no	no	no	no	yes
no	no	no	no	yes	no
no	no	no	no	yes	no
no	no	no	no	yes	no
no	no	no	no	yes	no
no	no	no	no	yes	no
no	no	no	no	yes	no
no	no	no	yes	no	yes
no	no	no	no	no	yes
no	no	no	no	no	yes
no	no	no	yes	no	yes
no	no	no	no	no	yes
no	no	no	no	no	yes
no	no	no	no	no	yes
no	no	no	no	no	yes
no	no	no	no	no	yes
no	no	no	yes	no	yes
no	no	no	no	no	yes
no	no	no	no	no	yes
no	no	no	yes	no	yes
no	no	no	no	no	yes
no	no	no	no	no	yes
no	no	no	yes	no	yes
no	no	no	no	no	yes
no	no	no	no	no	yes
no	no	no	yes	no	yes
no	no	no	no	no	yes
no	no	no	no	no	yes
no	no	no	yes	no	yes
no	no	no	no	no	yes
no	no	no	no	no	yes
no	no	no	yes	no	yes
no	no	no	no	no	yes
no	no	no	no	no	yes
no	no	no	yes	no	yes
no	no	no	no	no	yes
no	no	no	no	no	yes
no	no	no	yes	no	no
no	no	no	no	no	no
no	no	no	yes	no	yes
no	no	no	no	no	yes
no	no	no	no	no	yes
no	no	no	yes	no	yes
no	no	no	no	no	yes
no	no	no	no	no	yes
no	no	no	yes	no	yes

no	no	no	no	no	yes
no	no	no	no	no	yes
no	no	no	no	no	yes
no	no	no	no	no	yes
no	no	no	no	no	yes
no	no	no	yes	no	yes
no	no	no	no	no	yes
no	no	no	no	no	yes
no	no	no	yes	no	yes
no	no	no	no	no	yes
no	no	no	no	no	yes
no	no	no	no	no	no
no	no	no	yes	no	yes
no	no	no	no	no	yes
no	no	no	no	no	yes
no	no	no	no	no	no
no	no	no	no	no	no
no	no	no	no	no	no
no	no	no	no	no	no
yes	no	no	yes	no	no
yes	no	no	no	no	no
yes	no	no	no	no	no
no	yes	no	no	yes	no
no	yes	no	no	yes	no
no	yes	no	no	yes	no
no	yes	no	no	yes	no
no	yes	no	no	yes	no
no	yes	no	no	yes	no
yes	no	no	no	no	no
yes	no	no	no	no	no
yes	no	no	no	no	no
yes	no	no	no	no	no
yes	no	no	no	no	no
yes	no	no	no	no	no
no	no	no	no	no	no
no	no	no	no	no	no
no	no	no	no	no	no
yes	no	no	no	no	no
yes	no	no	no	no	no
yes	no	no	no	no	no
no	no	no	no	no	no
no	no	no	no	no	no
no	no	no	no	no	no
no	yes	no	no	yes	no
no	yes	no	no	yes	no
no	yes	no	no	yes	no
no	no	no	no	no	no
no	no	no	no	no	no
no	yes	no	no	yes	no
no	yes	no	no	yes	no
no	yes	no	no	yes	no
no	yes	no	no	yes	no

no	yes	no	no	yes	no
no	yes	no	no	yes	no
no	yes	no	no	yes	no
no	yes	no	no	yes	no
no	yes	no	no	yes	no
no	yes	no	no	yes	no
no	yes	no	no	yes	no
no	yes	no	no	yes	no
no	no	no	no	no	no
no	no	no	no	no	no
no	no	no	no	no	no
yes	no	no	no	no	no
yes	no	no	no	no	no
yes	no	no	no	no	no
yes	no	no	no	no	no
yes	no	no	no	no	no
yes	no	no	no	no	no
yes	no	no	no	no	no
no	no	no	no	no	no
no	no	no	no	no	no
no	no	no	no	no	no
no	no	no	no	no	no
no	no	no	no	no	no
no	no	no	no	no	no
no	no	no	no	no	no
no	no	no	no	no	no
no	no	no	no	no	no
no	no	no	no	no	no
no	no	no	no	no	no
no	no	no	no	no	no
no	no	no	no	no	no
no	no	no	no	no	no
no	no	no	no	no	no
no	no	no	no	no	no
no	no	no	no	no	no
no	no	no	no	no	no
no	no	no	no	no	no
no	no	no	no	no	no
no	no	no	no	no	no
no	no	no	no	no	no
no	no	no	no	no	no
no	no	no	no	no	no
no	no	no	no	no	no
no	no	no	yes	no	no
no	no	no	no	no	no
no	no	no	no	no	no
no	no	no	no	no	no
no	no	no	no	no	no
no	no	no	no	no	no
no	no	no	no	no	no
no	no	no	no	no	no
no	no	no	no	no	no

yes	no	no	no	no	no
yes	no	no	no	no	no
yes	no	no	no	no	no
yes	no	no	no	no	no
yes	no	no	no	no	no
yes	no	no	no	no	no
yes	no	no	no	no	no
yes	no	no	no	no	no
yes	no	no	no	no	no
yes	no	no	no	no	no
yes	no	no	no	no	no
yes	no	no	no	no	no
yes	no	no	no	no	no
yes	no	no	no	no	no
yes	no	no	no	no	no
no	yes	no	no	yes	no
no	yes	no	no	yes	no
no	yes	no	no	yes	no
yes	no	no	yes	no	no
yes	no	no	no	no	no
yes	no	no	no	no	no
no	no	no	no	no	no
yes	no	no	no	no	no
yes	no	no	no	no	no
yes	no	no	no	no	no
no	no	no	no	no	no
no	no	no	no	no	no
no	no	no	no	no	no
no	no	no	no	no	no
yes	no	no	yes	no	no
yes	no	no	no	no	no
yes	no	no	no	no	no
yes	no	no	no	no	no
yes	no	no	no	no	no
yes	no	no	no	no	no
no	yes	no	no	yes	no
no	yes	no	no	yes	no
no	yes	no	no	yes	no
no	yes	no	no	yes	no
no	yes	no	no	yes	no
no	yes	no	no	yes	no
yes	no	no	no	no	no
yes	no	no	no	no	no
yes	no	no	no	no	no
yes	yes	no	no	yes	no
yes	yes	no	no	yes	no
yes	yes	no	no	yes	no
no	yes	no	yes	yes	no
yes	no	no	yes	no	no
yes	no	no	no	no	no
yes	no	no	no	no	no
no	no	no	no	no	no

yes	no	no	no	no	no
yes	no	no	no	no	no
yes	no	no	no	no	no
no	no	no	no	no	no
no	no	no	no	no	no
no	no	no	no	no	no
no	no	no	no	no	no
no	no	no	no	no	no
no	no	no	no	no	no
no	yes	no	no	yes	no
no	yes	no	no	yes	no
no	yes	no	no	yes	no
no	yes	no	no	yes	no
no	yes	no	no	yes	no
no	yes	no	no	yes	no
yes	no	no	no	no	no
yes	no	no	no	no	no
yes	no	no	no	no	no
yes	no	no	yes	no	no
yes	no	no	no	no	no
yes	no	no	no	no	no
yes	no	no	no	no	no
yes	no	no	no	no	no
yes	no	no	no	no	no
yes	no	no	no	no	no
yes	no	no	no	no	no
yes	no	no	no	no	no
no	no	no	yes	no	no
no	no	no	no	no	no
no	no	no	no	no	no
no	no	no	no	no	no
no	no	no	no	no	no
yes	yes	no	no	yes	no
yes	yes	no	no	yes	no
yes	yes	no	no	yes	no
no	no	no	no	no	no
no	no	no	no	no	no
no	yes	no	no	yes	no
no	yes	no	no	yes	no
no	yes	no	no	yes	no
yes	no	no	yes	no	no
yes	no	no	no	no	no
yes	no	no	no	no	no
yes	no	no	no	no	no
yes	no	no	no	no	no
yes	no	no	no	no	no
no	yes	no	no	yes	no
no	yes	no	no	yes	no
no	yes	no	no	yes	no
no	yes	no	no	yes	no
no	yes	no	no	yes	no
no	yes	no	no	yes	no

no	no	no	yes	no	no
no	no	no	no	no	no
no	no	no	no	no	no
no	no	no	yes	no	no
no	no	no	no	no	no
no	no	no	no	no	no
no	no	no	no	no	no
no	no	no	no	no	no
no	no	no	no	no	no
no	no	no	no	no	no
no	no	no	no	no	no
no	no	no	no	no	no
no	no	no	no	no	no
no	no	no	yes	no	no
no	no	no	yes	no	no
no	no	no	no	no	no
no	no	no	no	no	no
no	no	no	no	no	no
no	yes	no	yes	yes	no
no	no	no	yes	no	no
no	yes	no	no	yes	no
no	yes	no	no	yes	no
no	no	no	no	no	no
no	no	no	no	no	no
no	no	no	no	no	no
no	yes	no	no	yes	no
no	yes	no	no	yes	no
no	yes	no	no	yes	no
no	no	no	no	no	no
no	no	no	no	no	no
no	no	no	no	yes	no
no	no	no	no	yes	no
no	no	no	no	yes	no
no	yes	no	no	yes	no
no	yes	no	no	yes	no
no	yes	no	no	yes	no
no	yes	no	no	yes	no
no	yes	no	no	yes	no
no	yes	no	no	yes	no
no	yes	no	no	yes	no
no	yes	no	no	yes	no
no	yes	no	no	yes	no
no	yes	no	no	yes	no
no	yes	no	no	yes	no
no	yes	no	no	yes	no
no	yes	no	no	yes	no
no	yes	no	no	yes	no
no	yes	no	no	yes	no
no	yes	no	no	yes	no
no	yes	no	no	yes	no
no	yes	no	no	yes	no
no	yes	no	no	yes	no

no	yes	no	no	yes	no
no	yes	no	no	yes	no
no	no	no	no	yes	no
no	no	no	no	yes	no
no	no	no	no	no	no
no	yes	no	no	yes	no
no	yes	no	no	yes	no
no	yes	no	no	yes	no
no	yes	no	no	yes	no
no	yes	no	no	yes	no
no	yes	no	no	yes	no
no	yes	no	no	yes	no
no	yes	no	no	yes	no
no	yes	no	no	yes	no
no	no	no	no	yes	no
no	yes	no	no	yes	no
no	yes	no	no	yes	no
no	yes	no	no	yes	no
no	yes	no	no	yes	no
no	yes	no	no	yes	no
no	yes	no	no	yes	no
no	yes	no	no	yes	no
no	yes	no	no	yes	no
no	yes	no	no	yes	no
no	yes	no	no	yes	no
no	yes	no	no	yes	no
no	yes	no	no	yes	no
no	no	no	yes	no	no
no	no	no	yes	no	no
no	no	no	no	no	no
no	no	no	no	yes	no
no	no	no	no	yes	no
no	no	no	no	yes	no
no	no	no	no	no	no
no	no	no	no	no	no
no	yes	no	no	yes	no
no	yes	no	no	yes	no
no	yes	no	yes	yes	no
no	yes	no	no	yes	no
no	yes	no	no	yes	no
no	yes	no	no	yes	no
no	yes	no	no	yes	no
no	yes	no	no	yes	no
no	yes	no	no	yes	no
no	yes	no	no	yes	no
no	yes	no	no	yes	no
no	yes	no	no	yes	no
no	yes	no	no	yes	no
no	yes	no	no	yes	no
no	no	no	no	yes	no
no	no	no	no	yes	no
no	no	no	no	yes	no

no	yes	no	yes	yes	yes
no	yes	no	no	yes	yes
no	yes	no	no	yes	yes
no	no	no	no	no	no
no	yes	no	no	yes	no
no	yes	no	no	yes	no
no	yes	no	no	yes	no
no	yes	no	no	yes	no
no	yes	no	no	yes	no
no	yes	no	no	yes	no
no	yes	no	no	yes	no
no	yes	no	no	yes	no
no	yes	no	no	yes	no
no	yes	no	no	yes	no
no	yes	no	no	yes	no
no	yes	no	no	yes	no
no	no	yes	no	no	no
no	no	yes	no	no	no
no	no	yes	no	no	no
no	no	yes	no	no	no
no	no	yes	no	no	no
no	no	yes	no	no	no
no	no	yes	no	no	no
no	no	yes	no	no	no
no	no	yes	no	no	no
no	no	yes	no	no	no
no	no	yes	no	no	no
no	no	yes	no	no	no
no	no	yes	no	no	no
no	no	yes	no	no	no
no	no	yes	no	no	no
no	no	yes	no	no	no
no	no	yes	no	no	no
no	no	yes	no	no	no
no	no	yes	no	no	no
no	no	yes	no	no	no
no	no	yes	no	no	no
no	no	yes	no	no	no
no	no	yes	no	no	no
no	no	yes	no	no	no
no	no	yes	no	no	no
no	no	yes	no	no	no
no	no	yes	no	no	no
no	no	yes	no	no	no
no	no	yes	no	no	no
no	no	yes	no	no	no
no	no	yes	no	no	no
no	no	yes	no	no	no
no	no	yes	no	no	no

in_Avila2025
yes
yes
yes
yes
yes
yes
yes
yes
yes
yes
yes
yes
yes
yes
yes
yes
yes
yes
yes
yes
yes
yes
yes
yes
yes
yes
yes
yes
yes
yes
no
no
no
yes
yes
yes
yes
yes
yes
no
no
no
yes
yes
yes
yes
yes
yes
yes
yes

yes
yes
yes
yes
yes
yes
yes
yes
yes
yes
yes
yes
yes
yes
yes
yes
yes
yes
yes
yes
yes
yes
yes
yes
yes
yes
yes
yes
yes
yes
yes
yes
yes
yes
yes
yes
yes
yes
yes
yes
yes
yes
yes
yes
yes
yes
yes
yes
yes
yes
yes
yes

yes
yes
yes
yes
yes
yes
yes
yes
yes
yes
yes
yes
yes
yes
yes
yes
yes
yes
yes
yes
yes
yes
yes
yes
yes
yes
yes
yes
yes
yes
yes
yes
yes
yes
yes
yes
yes
yes
yes
yes
yes
yes
yes
yes
yes
yes
yes
yes
yes
yes
yes
yes

yes
yes
yes
yes
yes
yes
yes
yes
yes
yes
yes
yes
yes
yes
no
no
no
no
no
no
no
no
no
no
no
no
no
no
no
no
no
no
no
no
no
no
no
no
no
no
no
no
no
no
no
no
no
no
no
no
no
no

no
no
no
no
no
no
no
no
no
no
no
no
no
no
no
no
no
no
no
no
no
no
no
no
no
yes
yes
yes
no
no
no
no
no
yes
yes
yes
no
no
yes
yes
yes
yes
yes
yes
yes
yes
yes
yes
yes
yes
yes
yes

yes
yes
yes
yes
yes
yes
yes
yes
yes
yes
yes
yes
yes
yes
yes
yes
yes
yes
yes
yes
yes
yes
yes
yes
yes
yes
yes
yes
yes
yes
yes
yes
yes
yes
yes
yes
yes
no
no
no
yes
yes
yes
yes
yes
yes
yes
yes
yes
yes
yes
yes

no
no
yes
yes
yes
yes
yes
yes
yes
yes
yes
yes
yes
yes
yes
yes
yes
yes
yes
yes
yes
yes
yes
yes
yes
yes
yes
yes
yes
yes
yes
yes
no
no
yes
yes
yes
yes
yes
yes
no
no
no
yes
yes
yes
yes
yes
yes
yes
yes
yes

yes
yes
yes
yes
yes
yes
yes
yes
yes
yes
yes
yes
yes
yes
yes
yes
yes
yes
yes
yes
yes
yes
yes
yes
yes
yes
yes
yes
yes
yes
yes
yes
yes
yes
yes
yes
yes
yes
yes
yes
yes
yes
yes
yes
yes
no
no
yes
yes
yes
yes
yes

yes
yes
yes
yes
yes
yes
yes
yes
yes
yes
yes
yes
yes
yes
yes
yes
no
yes
yes
yes
no
no
no
no
no
no
no
no
no
no
no
yes
yes
yes
yes
yes
yes
no
no
no
no
no
no
yes
yes
yes
yes
yes
no
no
no
yes

yes
yes
no
no
no
no
yes
yes
yes
no
no
no
no
no
no
no
no
no
no
yes
yes
yes
no
no
no
no
no
yes
yes
yes
yes
yes
yes
yes
yes
yes
no
no
no
no
yes
yes
yes
yes
yes
yes
yes
no
no
yes
yes
yes

no
no
no
yes
yes
yes
yes
yes
yes
no
no
no
yes
yes
yes
no
no
yes
yes
yes
no
no
no
no
no
no
no
no
yes
yes
yes
no
no
yes
yes
yes
yes
yes
yes
no
no
no
no
no
no
no
no
no
no
no
no
no

yes
yes
yes
no
no
no
no
no
yes
yes
yes
yes
yes
yes
no
no
no
no
no
no
no
yes
yes
yes
no
no
no
no
yes
yes
yes
no
no
no
no
no
no
no
yes
yes
yes
yes
yes
yes
no
no
no
no
no
yes
yes
yes

no
no
no
no
no
yes
yes
yes
yes
yes
yes
yes
yes
yes
no
no
no
yes
yes
yes
no
no
no
yes
yes
yes
yes
yes
yes
yes
yes
yes
no
no
no
no
no
no
no
no
no
yes
yes
yes
yes
yes
yes
yes
yes
yes
yes
yes

yes
yes
yes
yes
yes
yes
yes
yes
yes
yes
yes
yes
yes
yes
yes
yes
yes
yes
yes
yes
yes
yes
yes
yes
yes
yes
yes
yes
yes
yes
yes
yes
yes
yes
yes
yes
yes
yes
yes
yes
no
no
no
yes
yes
yes
yes
yes
yes
yes
yes
yes

yes
yes
yes
yes
yes
yes
yes
yes
yes
no
no
no
yes
yes
yes
yes
yes
yes
yes
yes
yes
yes
yes
yes
yes
yes
yes
yes
yes
yes
yes
yes
yes
yes
yes
yes
yes
yes
yes
yes
yes
yes
yes
yes
yes
yes
no
no
no
yes
yes
yes

yes
yes
yes
yes
yes
yes
no
no
no
yes
yes
yes
yes
yes
yes
no
no
no
no
no
no
no
yes
yes
yes
no
no
no
no
no
yes
yes
yes
yes
yes
yes
yes
yes
yes
no
no
no
yes
yes
yes
yes
yes
yes
no
no
no
yes

yes
yes
yes
yes
yes
yes
yes
yes
yes
yes
yes
no
no
no
yes
yes
yes
yes
yes
yes
yes
yes
yes
yes
yes
yes
no
no
no
yes
yes
yes
no
no
no
yes
yes
yes
no
no
no
no
no
no
yes
yes
yes
yes
yes
yes
yes
yes

yes
yes
yes
yes
no
no
no
yes
yes
yes
yes
yes
yes
no
no
no
no
no
no
no
no
no
no
no
no
no
no
no
no
no
no
no
no
no
yes
yes
yes
no
no
no
no
no
no
no
no
yes
yes
yes
no
no
no
no

no
no
no
no
no
no
no
no
no
no
no
no
no
no
no
no
no
no
no
no
no
no
yes
yes
yes
yes
yes
yes
no
no
no
no
no
no
no
no
no
no
no
no
no
no
no
yes
yes
yes
no
no
yes
yes
yes
yes

yes
yes
yes
yes
yes
yes
yes
yes
no
no
no
no
no
no
no
no
no
no
no
no
no
no
no
no
no
no
no
no
no
no
no
no
no
no
no
no
no
no
no
no
no
no
no
no
no
no
no
no
no
no
no
no

no
no
no
no
no
no
no
no
no
no
no
no
no
no
no
yes
yes
yes
no
no
no
no
no
no
no
no
no
no
no
no
no
no
no
no
no
yes
yes
yes
yes
yes
yes
no
no
no
yes
yes
yes
yes
no
no
no
no

no
no
no
no
no
no
no
no
no
yes
yes
yes
yes
yes
yes
no
no
no
no
no
no
no
no
no
no
no
no
no
no
no
no
no
yes
yes
yes
no
no
yes
yes
yes
no
no
no
no
no
no
yes
yes
yes
yes
yes
yes

no
no
no
no
no
no
no
no
no
no
no
no
no
no
no
no
no
no
yes
no
yes
yes
no
no
no
yes
yes
yes
no
no
yes
yes
yes
yes
yes
yes
yes
yes
yes
yes
yes
yes
yes
yes
yes
yes
yes
yes
yes
yes
yes
yes

yes
yes
yes
yes
no
yes
yes
yes
yes
yes
yes
yes
yes
yes
yes
yes
yes
yes
yes
yes
yes
yes
yes
yes
yes
yes
yes
no
no
no
yes
yes
yes
no
no
yes
yes
yes
yes
yes
yes
yes
yes
yes
yes
yes
yes
yes
yes
yes
yes
yes

yes
yes
yes
no
yes
yes
yes
yes
yes
yes
yes
yes
yes
yes
yes
yes
no
no
no
no
no
no
no
no
no
no
no
no
no
no
no
no
no
no
no
no
no
no
no
no
no
no
no
no
no
no
no
no
no
