# Supplementary material for: Genomic landscape of autism spectrum disorder in Brazil
Source: Genet Mol Biol. 2026 Sep 7;49(Suppl 1):e20250247. doi: 10.1590/1678-4685-GMB-2025-0247 (PMC13561403; doi:10.1590/1678-4685-GMB-2025-0247)
Supplement: Supplementary file 8 [file 1415-4757-GMB-49-s1-e20250247-s4.pdf]

Genetics and Molecular Biology
https://doi.org/10.1590/1678-4685-GMB-2025-0247

Supplementary Material to "Genomic landscape of autism spectrum disorder in Brazil"

Table S3 - *De novo* variants identified in the probands (N = 386 trios).

Chr	Start	End	Ref	Alt	Canoni	Func.re	Gene.r	GeneD	Exonic	AACha	OMIM
chr1	1040862	1040862	C	-	NO	exonic	AGRN	0	frames	AGRN	Agrin (
chr1	1040864	1040864	C	-	NO	exonic	AGRN	0	frames	AGRN	Agrin (
chr1	1211584	1211584	C	T	NO	exonic	TNFRS	0	nonsyn	TNFRS	Tumor
chr1	1655480	1655480	T	A	NO	exonic;	CDK1	0	nonsyn	CDK1	Cyclin-
chr1	2193691	2193691	C	T	NO	exonic	FAAP2	0	nonsyn	FAAP2	Chrom
chr1	2306188	2306188	C	T	NO	exonic	SKI	0	stopgai	SKI:N	SKI pr
chr1	5986213	5986213	T	G	NO	exonic	NPHP4	0	nonsyn	NPHP4	Nephro
chr1	10660530	10660530	G	C	NO	exonic	CASZ1	0	nonsyn	CASZ1	Castor
chr1	11676850	11676850	G	C	NO	exonic;	MAD2	0	nonsyn	MAD2	Mitotic
chr1	27006680	27006680	C	A	NO	exonic	TENT5	0	nonsyn	TENT5	Termin
chr1	27362966	27362966	G	T	NO	exonic	MAP3	0	nonsyn	MAP3	Mitoge
chr1	28714462	28714462	T	C	NO	exonic	GMEB	0	synony	GMEB	Glucoc
chr1	32091818	32091818	C	T	NO	exonic	TMEM	0	nonsyn	TMEM39B:N	
chr1	37800156	37800156	C	T	NO	exonic	MANE	0	nonsyn	MANEAL:NM	
chr1	37946280	37946280	G	A	NO	exonic	INPP5	0	nonsyn	INPP5	Inosito
chr1	39360917	39360917	T	C	NO	exonic	MACF	0	synony	MACF	Microt
chr1	39684099	39684099	G	C	NO	exonic	HPCA	0	nonsyn	HPCA	Hippoc
chr1	44802829	44802829	G	A	NO	exonic	PLK3	0	synony	PLK3:	Polo-li
chr1	45003682	45003682	G	T	NO	exonic	HECT	0	nonsyn	HECT	HECT
chr1	66534359	66534359	A	G	NO	exonic	SGIP1	0	startlos	SGIP1:	SH3-do
chr1	70262791	70262791	C	T	NO	exonic	ANKR	0	nonsyn	ANKR	Ankyri
chr1	74636429	74636429	G	A	NO	exonic	ERICH	0	stopgai	ERICH3:NM_	
chr1	75794082	75794082	A	C	YES	splicin	RABG	NM_0	0	0	Rab ge
chr1	76868686	76868686	G	A	NO	exonic	ST6GA	0	nonsyn	ST6GA	ST6 al
chr1	95072764	95072764	G	A	NO	exonic;	ALG14	0	synony	ALG14	ALG14
chr1	102962695	102962695	-	CCAG	NO	exonic	COL11	0	nonfra	COL11	Collag
chr1	108922448	108922448	G	C	NO	exonic	GPSM	0	nonsyn	GPSM	G-prot
chr1	113673383	113673383	G	A	NO	exonic	MAGI	0	nonsyn	MAGI	Membr
chr1	113887301	113887301	T	C	NO	exonic	BCL2L	0	synony	BCL2L	BCL2-
chr1	116961390	116961390	G	T	NO	exonic	PTGFR	0	nonsyn	PTGFR	Prostag
chr1	147758680	147758680	T	A	NO	exonic	GJA5	0	nonsyn	GJA5:	Gap ju
chr1	149944917	149944917	T	C	NO	exonic	OTUD	0	nonsyn	OTUD	OTU d
chr1	151315926	151315926	T	C	NO	exonic	PI4KB	0	nonsyn	PI4KB	Phosph
chr1	152352585	152352585	C	T	NO	exonic	FLG2	0	nonsyn	FLG2:	Filaggr
chr1	155188615	155188692	GCCT	-	NO	exonic	MUC1	0	nonfra	MUC1	Mucin
chr1	156866906	156866906	G	T	NO	splicin	NTRK	NM_0	0	0	Neurot
chr1	158766614	158766614	G	A	NO	exonic	OR6N1	0	synony	OR6N1:NM_0	
chr1	175989477	175989477	G	A	NO	exonic;	COP1	0	nonsyn	COP1:	Ring fi
chr1	182488991	182488991	G	T	NO	exonic	RGSL1	0	nonsyn	RGSL1	Regula
chr1	200873248	200873248	C	T	NO	exonic	GPR25	0	nonsyn	GPR25	G prote
chr1	201211011	201211011	G	A	NO	exonic	IGFN1	0	nonsyn	IGFN1	Immun
chr1	202440736	202440736	C	T	NO	exonic	PPP1R	0	synony	PPP1R	Protein
chr1	211018934	211018934	G	A	NO	exonic	KCNH	0	nonsyn	KCNH	Potassi
chr1	220195110	220195110	C	T	NO	exonic	RAB3	0	synony	RAB3	RAB3
chr1	225411356	225411356	C	T	NO	exonic	LBR	0	nonsyn	LBR:N	Lamin
chr1	232461008	232461008	C	T	NO	exonic	SIPA1	0	nonsyn	SIPA1	SIPA1-
chr1	236862261	236862261	A	G	NO	exonic	MTR	0	nonsyn	MTR:N	5-meth
chr1	237806159	237806159	A	G	NO	exonic	RYR2	0	nonsyn	RYR2:	Ryanod

chr1	245155451	245155451	G	C	NO	exonic	KIF26	0	nonsyn	KIF26	Kinesi
chr2	9418690	9418690	C	T	NO	exonic	ITGB1	0	nonsyn	ITGB1	Integri
chr2	10441875	10441875	T	C	NO	exonic	ODC1	0	nonsyn	ODC1:	Ornithi
chr2	20278703	20278703	G	A	NO	exonic	PUM2	0	synony	PUM2:	Pumili
chr2	24299946	24299946	C	T	NO	exonic	ITSN2	0	nonsyn	ITSN2:	Interse
chr2	25243930	25243930	C	T	NO	exonic	DNMT	0	nonsyn	DNMT	DNA m
chr2	43852751	43852751	G	C	NO	exonic	ABCG	0	nonsyn	ABCG	ATP-bi
chr2	43852756	43852756	C	G	NO	exonic	ABCG	0	synony	ABCG	ATP-bi
chr2	47176489	47176489	A	G	NO	exonic	CALM	0	nonsyn	CALM	Calmo
chr2	63912627	63912627	G	A	NO	exonic	VPS54	0	synony	VPS54	VPS54
chr2	65268699	65268699	C	T	NO	exonic	ACTR	0	nonsyn	ACTR	Actin-r
chr2	69505663	69505663	G	T	NO	exonic	AAK1	0	synony	AAK1:	Adapto
chr2	70963247	70963247	C	T	NO	exonic	ATP6V	0	nonsyn	ATP6V	ATPas
chr2	71569839	71569839	G	A	NO	exonic	DYSF	0	nonsyn	DYSF:	Dysfer
chr2	73816579	73816579	C	G	NO	exonic	C2orf7	0	nonsyn	C2orf78:NM_0	
chr2	74461962	74461962	A	G	NO	exonic	MOGS	0	synony	MOGS	Manno
chr2	95410875	95410875	T	C	NO	exonic	FAHD	0	synony	FAHD2A:NM	
chr2	96304826	96304826	G	A	NO	exonic	SNRN	0	nonsyn	SNRN	Small n
chr2	102368073	102368073	G	A	NO	splicin	IL18R1NM_0		0	0	Interle
chr2	110160214	110160214	T	C	NO	exonic	NPHP1	0	synony	NPHP1	Nephro
chr2	121736850	121736850	T	C	NO	exonic	NIFK	0	startlos	NIFK:	Nucleo
chr2	127505011	127505011	G	A	NO	exonic	IWS1	0	stopgai	IWS1:NM_017	
chr2	127955179	127955179	C	T	NO	exonic	SAP13	0	synony	SAP13	Sin3A-
chr2	165374775	165374775	G	A	NO	exonic	SCN2A	0	nonsyn	SCN2A	Sodium
chr2	165991772	165991772	G	A	NO	exonic	SCN1A	0	nonsyn	SCN1A	Sodium
chr2	169211988	169211998	ATGG	-	NO	exonic	LRP2	0	frames	LRP2:	Low de
chr2	172562287	172562287	T	C	NO	exonic;	PDK1	0	nonsyn	PDK1:	Pyruva
chr2	178746404	178746404	T	G	NO	exonic	TTN	0	synony	TTN:N	Titin (
chr2	178868117	178868117	C	T	NO	exonic	CCDC	0	nonsyn	CCDC	Coiled-
chr2	198146802	198146802	A	G	NO	exonic	PLCL1	0	nonsyn	PLCL1	Phosph
chr2	199349027	199349027	G	A	NO	exonic	SATB2	0	stopgai	SATB2	Special
chr2	219489658	219489658	G	A	NO	exonic	SPEG	0	synony	SPEG:	SPEG
chr2	224513582	224513582	G	C	NO	exonic	CUL3	0	stopgai	CUL3:	Cullin
chr2	227991070	227991070	C	T	NO	exonic	SPHK	0	stopgai	SPHK	SPHK1
chr2	231271044	231271044	C	T	NO	exonic	ARMC	0	synony	ARMC	Armad
chr2	231458356	231458356	A	G	NO	exonic	NCL	0	nonsyn	NCL:N	Nucleo
chr2	233950032	233950032	G	C	NO	exonic	TRPM	0	synony	TRPM	Transie
chr2	240045383	240045383	C	T	NO	exonic	OR6B3	0	synony	OR6B3:NM_1	
chr2	241256337	241256337	C	T	NO	exonic	HDLB	0	synony	HDLB	High-d
chr2	241343018	241343018	C	T	NO	exonic	SEPTI	0	synony	SEPTI	Septin
chr3	9365151	9365151	G	A	NO	exonic	THUM	0	nonsyn	THUMPD3:N	
chr3	10042657	10042657	A	T	NO	exonic	FANC	0	synony	FANC	Fancon
chr3	11017846	11017846	C	A	NO	exonic;	SLC6A	0	nonsyn	SLC6A	Solute
chr3	12167238	12167238	A	G	NO	exonic;	SYN2	0	nonsyn	SYN2:	Synaps
chr3	14517789	14517789	C	A	NO	exonic	GRIP2	0	nonsyn	GRIP2:NM_00	
chr3	23499654	23499654	T	C	NO	exonic	UBE2E	0	synony	UBE2E	Ubiqui
chr3	42528094	42528094	G	A	NO	exonic	VIPR1	0	nonsyn	VIPR1	Vasoac
chr3	42686013	42686013	A	G	NO	exonic	KLHL	0	nonsyn	KLHL	Kelch-
chr3	46265360	46265360	A	T	NO	exonic	CCR3	0	nonsyn	CCR3:	Chemo
chr3	47418796	47418796	G	A	NO	exonic	SCAP	0	nonsyn	SCAP:	SREBP

chr3	70972667	70972667	G	A	NO	exonic	FOXP1	0	nonsyn	FOXP1	Forkhe
chr3	71046957	71046957	G	A	NO	exonic	FOXP1	0	stopgai	FOXP1	Forkhe
chr3	98593227	98593227	A	G	NO	exonic	CPOX	0	nonsyn	CPOX:	Coprop
chr3	100864867	100864867	C	T	NO	exonic	ABI3B	0	synony	ABI3B	Target
chr3	101332821	101332821	C	A	NO	exonic	SENP7	0	nonsyn	SENP7	Sentrin
chr3	101801403	101801403	C	A	NO	exonic	NXPE	0	nonsyn	NXPE3:NM_0	
chr3	108644304	108644304	C	T	NO	exonic	DZIP3	0	nonsyn	DZIP3:	DAZ-i
chr3	112619100	112619100	A	G	NO	exonic;	CCDC	0	synony	CCDC	Coiled-
chr3	128971340	128971340	G	A	NO	exonic	CFAP9	0	nonsyn	CFAP92:NM_	
chr3	136151239	136151239	C	G	NO	exonic	MSL2	0	nonsyn	MSL2:	MSL c
chr3	136151256	136151256	C	A	NO	exonic	MSL2	0	nonsyn	MSL2:	MSL c
chr3	138094906	138094906	C	G	NO	exonic	DZIP1	0	nonsyn	DZIP1	DAZ-i
chr3	138462117	138462117	A	G	NO	exonic	ESYT3	0	nonsyn	ESYT3	Extend
chr3	146160980	146160980	A	C	NO	exonic	PLOD2	0	nonsyn	PLOD2	Procoll
chr3	150660096	150660096	C	T	NO	exonic	ERICH	0	synony	ERICH6:NM_	
chr3	161242554	161242554	T	C	NO	exonic	NMD3	0	synony	NMD3	NMD3
chr3	170136434	170136434	G	C	NO	exonic	PHC3	0	nonsyn	PHC3:	Polyho
chr3	170423272	170423272	C	T	NO	exonic	CLDN	0	synony	CLDN	Claudi
chr3	170868110	170868110	T	C	NO	exonic	RPL22	0	nonsyn	RPL22L1:NM	
chr3	186080098	186080098	A	G	NO	exonic	ETV5	0	synony	ETV5:	ETS va
chr3	192335434	192335434	C	T	NO	exonic	FGF12	0	nonsyn	FGF12	Fibrobl
chr3	194642223	194642223	C	T	NO	exonic	LSG1	0	nonsyn	LSG1:	Large 6
chr3	195780142	195780142	C	T	NO	exonic	MUC4	0	nonsyn	MUC4	Mucin
chr3	195790542	195790542	C	T	NO	exonic	MUC4	0	synony	MUC4	Mucin
chr4	893906	893906	T	C	NO	exonic	GAK	0	nonsyn	GAK:N	Cyclin
chr4	2304470	2304470	G	A	NO	exonic	ZFYV	0	nonsyn	ZFYV	Zinc fi
chr4	3145168	3145168	C	G	NO	exonic	HTT	0	nonsyn	HTT:N	Huntin
chr4	5801974	5801974	G	T	NO	exonic	EVC	0	stopgai	EVC:N	EvC ci
chr4	15625757	15625757	C	T	NO	exonic	FBXL5	0	nonsyn	FBXL5	F-box a
chr4	23824313	23824313	G	A	NO	exonic	PPAR	0	nonsyn	PPAR	Peroxis
chr4	25156187	25156187	T	G	NO	exonic	SEPSE	0	nonsyn	SEPSE	O-phos
chr4	40436557	40436557	C	T	NO	exonic	RBM4	0	nonsyn	RBM4	RNA-b
chr4	78910727	78910727	C	G	NO	exonic	BMP2	0	nonsyn	BMP2	BMP2-
chr4	87615845	87615845	T	A	NO	exonic	DSPP	0	nonsyn	DSPP:	Dentin
chr4	105967247	105967247	G	T	NO	exonic	NPNT	0	nonsyn	NPNT:	Nephro
chr4	107614287	107614287	C	T	NO	exonic	PAPSS	0	nonsyn	PAPSS	3'-phos
chr4	128871522	128871522	C	T	NO	exonic	JADE1	0	stopgai	JADE1	PHD fi
chr4	134200923	134200923	C	G	NO	exonic	PABPC	0	nonsyn	PABPC4L:NM	
chr4	139349538	139349538	A	G	NO	exonic	NAA1	0	synony	NAA1	N-alph
chr4	140622580	140622580	G	A	NO	exonic	TBC1D	0	nonsyn	TBC1D	TBC1
chr4	140911274	140911274	A	T	NO	exonic	RNF15	0	synony	RNF150:NM_	
chr4	152770942	152770942	T	C	NO	exonic	TIGD4	0	synony	TIGD4:NM_1	
chr4	169117696	169117696	G	A	NO	exonic	SH3RF	0	nonsyn	SH3RF	SH3 do
chr4	186707093	186707093	A	G	NO	exonic	FAT1	0	nonsyn	FAT1:	FAT at
chr5	1279463	1279463	C	T	NO	exonic	TERT	0	nonsyn	TERT:	Telome
chr5	140695805	140695805	A	C	NO	exonic	HARS	0	nonsyn	HARS	Histidy
chr5	141179926	141179926	T	G	NO	exonic	PCDH	0	nonsyn	PCDH	Protoca
chr5	174104272	174104272	C	T	NO	exonic	NSG2	0	synony	NSG2:	Neuron
chr5	175513532	175513532	G	A	NO	exonic	SFXN1	0	synony	SFXN1	Siderof
chr6	7301332	7301332	T	C	NO	exonic	SSR1	0	nonsyn	SSR1:	Signal

chr6	7581366	7581366	A	C	NO	exonic	DSP	0	nonsyn	DSP:N	Desmo
chr6	12120365	12120365	C	G	NO	exonic	HIVEP	0	synony	HIVEP	Human
chr6	26199022	26199022	G	C	NO	exonic	H2AC7	0	nonsyn	H2AC7	H2A cl
chr6	28391198	28391198	G	A	NO	exonic	ZSCA	0	synony	ZSCA	Zinc fi
chr6	29723911	29723911	C	T	NO	exonic	HLA-F	0	synony	HLA-F	Major
chr6	31759452	31759452	C	T	NO	exonic	MSH5	0	nonsyn	MSH5:	mutS h
chr6	33440913	33440913	C	T	NO	exonic	SYNG	0	stopgai	SYNG	Synapt
chr6	33663751	33663751	G	A	NO	exonic	ITPR3	0	nonsyn	ITPR3:	Inosito
chr6	43451227	43451227	G	C	NO	exonic	DLK2	0	nonsyn	DLK2:NM_00	
chr6	52266089	52266089	C	T	NO	exonic	MCM3	0	synony	MCM3	Minich
chr6	84152567	84152567	G	C	NO	exonic	CEP16	0	nonsyn	CEP16	Centro
chr6	105373511	105373511	C	G	NO	exonic	PREP	0	synony	PREP:	Prolyl
chr6	109476844	109476844	C	T	NO	exonic	ZBTB2	0	nonsyn	ZBTB2	Zinc fi
chr6	116765258	116765258	G	A	NO	splicin	FAM1	NM_0	0	0	
chr6	117675709	117675709	C	T	NO	exonic	NUS1	0	synony	NUS1:	NUS1
chr6	130083695	130083695	C	T	NO	exonic	L3MB	0	nonsyn	L3MB	L3MB
chr6	138207113	138207113	C	T	NO	exonic	ARFG	0	nonsyn	ARFG	ADP-ri
chr6	138430424	138430424	C	T	NO	exonic	NHSL1	0	synony	NHSL1	NHS-li
chr6	152140036	152140036	C	G	NO	exonic	SYNE	0	nonsyn	SYNE	Spectri
chr6	167136496	167136496	C	T	NO	exonic	CCR6	0	nonsyn	CCR6:	Chemo
chr7	1438209	1438209	G	C	NO	exonic	MICA	0	nonsyn	MICALL2:NM	
chr7	2360861	2360861	C	A	NO	exonic	EIF3B	0	synony	EIF3B:	Eukary
chr7	5529230	5529230	G	T	NO	exonic	ACTB	0	synony	ACTB:	Actin,
chr7	10983028	10983028	G	A	NO	exonic	PHF14	0	nonsyn	PHF14	PHD fi
chr7	14682641	14682641	T	A	NO	exonic	DGKB	0	nonsyn	DGKB	Diacyl
chr7	18793382	18793382	C	G	NO	exonic	HDAC	0	nonsyn	HDAC	Histon
chr7	21545120	21545120	C	T	NO	exonic	DNAH	0	nonsyn	DNAH	Dynein
chr7	55939664	55939664	T	C	NO	exonic	ZNF71	0	synony	ZNF71	Zinc fi
chr7	70766316	70766316	G	A	NO	exonic	AUTS	0	synony	AUTS	KIAA0
chr7	73573326	73573326	T	C	NO	exonic	TBL2	0	nonsyn	TBL2:	Transd
chr7	87402305	87402305	A	G	NO	splicin	ABCB	NM_0	0	0	ATP-bi
chr7	92590280	92590280	C	T	NO	exonic	FAM1	0	synony	FAM133B:NM	
chr7	96189587	96189587	G	T	NO	exonic	SLC25	0	nonsyn	SLC25	Solute
chr7	96694852	96694852	C	T	NO	exonic	SEM1	0	stopgai	SEM1:	Proteas
chr7	99397983	99397983	C	A	NO	exonic	PDAP1	0	nonsyn	PDAP1	PDGF
chr7	100958215	100958215	T	A	NO	exonic	MUC3	0	nonsyn	MUC3	Mucin
chr7	100958237	100958237	C	T	NO	exonic	MUC3	0	nonsyn	MUC3	Mucin
chr7	100958239	100958239	A	C	NO	exonic	MUC3	0	nonsyn	MUC3	Mucin
chr7	103603478	103603478	C	T	NO	exonic	RELN	0	synony	RELN:	Reelin
chr7	128675752	128675752	C	T	NO	exonic	FAM7	0	synony	FAM71F2:NM	
chr7	141764551	141764551	A	G	NO	exonic	TAS2R	0	synony	TAS2R	Taste r
chr7	150993882	150993882	G	A	NO	exonic	NOS3	0	nonsyn	NOS3:	Nitric o
chr7	151675486	151675486	C	T	NO	exonic	PRKA	0	synony	PRKA	Protein
chr7	152224032	152224032	T	C	NO	exonic	KMT2	0	synony	KMT2	Lysine
chr7	158692850	158692850	A	G	NO	exonic	NCAP	0	nonsyn	NCAP	Non-S
chr8	1864426	1864426	C	T	NO	exonic	ARHG	0	nonsyn	ARHG	Rho gu
chr8	2002590	2002590	C	G	NO	exonic	KBTB	0	synony	KBTB	Kelch r
chr8	6621466	6621466	G	A	NO	exonic	MCPH	0	nonsyn	MCPH	Microc
chr8	8376413	8376413	A	T	NO	exonic	PRAG	0	nonsyn	PRAG	PEAK
chr8	8892768	8892768	G	A	NO	exonic	MFHA	0	synony	MFHA	Malign

chr8	18400763	18400763	G	A	NO	exonic	NAT2		0	nonsyn	NAT2:	Arylam
chr8	18871970	18871971	AT	-	NO	exonic	PSD3		0	frames	PSD3:	Pleckst
chr8	26643486	26643486	G	A	NO	exonic	DPYS		0	nonsyn	DPYS	Dihydr
chr8	37741802	37741802	G	A	NO	exonic	ERLIN		0	nonsyn	ERLIN	Endopl
chr8	41940843	41940843	T	C	NO	exonic;	KAT6		0	nonsyn	KAT6	K(lysin
chr8	56113911	56113911	G	T	NO	exonic	MOS		0	stopgai	MOS:N	MOS p
chr8	56113912	56113912	C	T	NO	exonic	MOS		0	nonsyn	MOS:N	MOS p
chr8	68533165	68533165	A	G	NO	exonic	C8orf3		0	synony	C8orf34:NM_0	
chr8	98951686	98951686	G	T	NO	exonic	OSR2		0	synony	OSR2:	Odd-sk
chr8	124070475	124070475	C	T	NO	exonic	FER1L		0	synony	FER1L6:NM_	
chr8	130115697	130115697	G	C	NO	exonic	ASAP1		0	synony	ASAP1	Arf GT
chr8	132929195	132929195	G	A	NO	splicin	TG	NM_0		0	0	Thyrog
chr8	142537009	142537009	G	A	NO	exonic	ADGR		0	nonsyn	ADGR	Adhesi
chr8	143921817	143921817	C	T	NO	exonic	PLEC		0	synony	PLEC:	Plectin
chr8	143925589	143925589	C	G	NO	exonic	PLEC		0	nonsyn	PLEC:	Plectin
chr8	144255541	144255541	G	A	NO	exonic	MROH		0	synony	MROH1:NM_	
chr9	2170415	2170415	G	A	NO	splicin	SMAR	NM_0		0	0	SWI/S
chr9	15474095	15474095	C	T	NO	exonic	PSIP1		0	nonsyn	PSIP1:	PC4- a
chr9	33030535	33030535	C	T	NO	exonic	DNAJ		0	stopgai	DNAJ	DNAJ/
chr9	33301330	33301330	C	T	NO	exonic	NFX1		0	synony	NFX1:	Nuclea
chr9	35906544	35906544	-	CCTC	NO	exonic	HRCT		0	nonfra	HRCT1:NM_0	
chr9	92847174	92847174	C	T	NO	exonic	ZNF48		0	nonsyn	ZNF484:NM_	
chr9	98306245	98306245	A	G	NO	exonic	GABB		0	nonsyn	GABB	Gamm
chr9	127668159	127668159	C	T	NO	exonic	STXBP		0	nonsyn	STXBP	Syntax
chr9	127802972	127802972	G	A	NO	exonic	FPGS		0	synony	FPGS:	Folylp
chr9	129097252	129097252	G	A	NO	exonic;	CRAT		0	nonsyn	CRAT:	Carniti
chr9	129098585	129098585	T	C	NO	exonic	CRAT		0	nonsyn	CRAT:	Carniti
chr9	130893527	130893527	C	T	NO	exonic	QRFP		0	synony	QRFP:	Pyrogl
chr9	132482326	132482326	C	T	NO	exonic	CFAP7		0	nonsyn	CFAP77:NM_	
chr9	134885316	134885316	C	T	NO	exonic	FCN2		0	nonsyn	FCN2:	Ficolin
chr9	136687312	136687312	G	A	NO	exonic	AGPA		0	synony	AGPA	1-acylg
chr9	137813075	137813075	G	A	NO	exonic	EHMT		0	synony	EHMT	Euchro
chr10	15064402	15064402	G	C	YES	splicin	OLAH	NM_0		0	0	
chr10	15064404	15064404	A	T	NO	exonic;	OLAH		0	nonsyn	OLAH:NM_00	
chr10	18675181	18675181	C	G	NO	exonic	ARL5B		0	nonsyn	ARL5B	ADP ri
chr10	26125511	26125511	G	A	NO	exonic	MYO3		0	nonsyn	MYO3	Myosin
chr10	49188309	49188309	T	C	NO	exonic	TMEM		0	nonsyn	TMEM273:NM	
chr10	50130954	50130954	G	C	NO	exonic	WASH		0	nonsyn	WASHC2A:N	
chr10	50820592	50820592	C	T	NO	exonic	A1CF		0	nonsyn	A1CF:	APOB
chr10	69186552	69186552	C	T	YES	splicin	SUPV3	NM_0		0	0	Suv3-li
chr10	71716294	71716294	C	T	NO	exonic	C10orf		0	nonsyn	C10orf105:NM	
chr10	99836264	99836264	T	G	NO	exonic	ABCC		0	nonsyn	ABCC	ATP-bi
chr10	103174859	103174859	G	T	NO	exonic;	NT5C2		0	synony	NT5C2	5' nucl
chr10	103174886	103174886	T	C	NO	exonic	NT5C2		0	nonsyn	NT5C2	5' nucl
chr10	116443095	116443095	A	G	NO	exonic	PNLIP		0	nonsyn	PNLIPRP3:NM	
chr10	118729329	118729329	G	T	NO	exonic	CACU		0	nonsyn	CACU	CDK2-
chr10	119042372	119042372	G	A	NO	exonic	EIF3A		0	stopgai	EIF3A:	Eukary
chr10	119577113	119577113	T	C	NO	exonic	TIAL1		0	synony	TIAL1	Tia1 cy
chr10	133355797	133355797	C	T	NO	exonic	FUOM		0	synony	FUOM	Fucose
chr11	397240	397240	G	C	NO	exonic	PKP3		0	nonsyn	PKP3:	Plakop

chr11	686950	686950	T	G	NO	exonic	DEAF	0	nonsyn	DEAF1	DEAF
chr11	1013542	1013542	C	T	NO	exonic	MUC6	0	nonsyn	MUC6	Mucin
chr11	1016288	1016288	G	T	NO	exonic	MUC6	0	nonsyn	MUC6	Mucin
chr11	1246223	1246223	A	T	NO	exonic	MUC5	0	nonsyn	MUC5	Mucin
chr11	1445436	1445436	-	T	NO	exonic	BRSK	0	frames	BRSK	BR ser
chr11	1454557	1454557	A	C	NO	exonic	BRSK	0	nonsyn	BRSK	BR ser
chr11	1621952	1621952	-	GGAG	NO	exonic	KRTA	0	nonfra	KRTAP5-4:N	
chr11	5268585	5268585	C	T	NO	exonic	HBE1	0	nonsyn	HBE1:	Hemog
chr11	5516155	5516155	A	T	NO	exonic	UBQL	0	nonsyn	UBQLNL:NM	
chr11	12258484	12258484	G	A	NO	exonic	MICA	0	synony	MICA	Microt
chr11	61770329	61770329	C	T	NO	exonic	MYRF	0	nonsyn	MYRF	Myelin
chr11	64854458	64854458	C	T	NO	exonic	EHD1	0	nonsyn	EHD1:	EH do
chr11	65540296	65540296	G	A	NO	exonic	LTBP3	0	nonsyn	LTBP3	Latent
chr11	66626576	66626576	G	A	NO	exonic	RBM1	0	nonsyn	RBM1	RNA-b
chr11	72235738	72235738	G	A	NO	exonic	INPPL	0	nonsyn	INPPL	Inosito
chr11	107815787	107815787	T	C	NO	splicin	SLC35	NM_0	0	0	Solute
chr11	108330233	108330233	C	T	NO	exonic	ATM	0	stopgai	ATM:	ATM s
chr11	113827352	113827352	A	T	NO	exonic	USP28	0	nonsyn	USP28	Ubiqui
chr11	118117382	118117382	C	T	NO	exonic	TMPR	0	synony	TMPR	Transm
chr11	119677850	119677850	G	A	NO	exonic	NECTI	0	synony	NECTI	Nectin
chr11	121589344	121589344	C	T	NO	exonic	SORL1	0	synony	SORL1	Sortilin
chr11	124029645	124029645	C	T	NO	exonic	OR10G	0	nonsyn	OR10G8:NM_	
chr11	129914927	129914927	C	G	NO	exonic	PRDM	0	nonsyn	PRDM	PR do
chr12	4278859	4278859	C	T	NO	exonic	CCND	0	nonsyn	CCND	Cyclin
chr12	4444204	4444204	C	T	NO	exonic	FGF6	0	nonsyn	FGF6:	Fibrobl
chr12	6593101	6593101	T	C	NO	exonic	CHD4	0	nonsyn	CHD4:	Chrom
chr12	6697311	6697311	A	G	NO	exonic	PIANP	0	nonsyn	PIANP	PILR-a
chr12	47794800	47794800	G	A	NO	exonic	HDAC	0	nonsyn	HDAC	Histon
chr12	52520158	52520158	C	T	NO	exonic	KRT5	0	nonsyn	KRT5:	Keratin
chr12	52772135	52772135	G	A	NO	exonic	KRT76	0	stopgai	KRT76	Keratin
chr12	54403761	54403761	T	C	NO	exonic	ITGA5	0	nonsyn	ITGA5	Integri
chr12	55681819	55681819	T	C	NO	exonic	METT	0	nonsyn	METTL7B:NM	
chr12	55961733	55961733	C	T	YES	splicin	PMEL	NM_0	0	0	Silver,
chr12	56428361	56428361	G	C	NO	exonic	TIMEL	0	nonsyn	TIMEL	Timele
chr12	75207969	75207969	C	G	NO	exonic	KCNC	0	nonsyn	KCNC	Potassi
chr12	75284965	75284965	C	T	NO	exonic;	CAPS2	0	nonsyn	CAPS2	Calcyp
chr12	99443680	99443680	C	T	NO	exonic	ANKS	0	nonsyn	ANKS	Ankyri
chr12	100142621	100142621	G	C	NO	exonic	UHRF	0	nonsyn	UHRF1BP1L:	
chr12	101648043	101648043	A	C	YES	splicin	MYBP	NM_0	0	0	Myosin
chr12	106814737	106814737	C	T	NO	exonic	RIC8B	0	synony	RIC8B	RIC8 g
chr12	108545243	108545243	C	T	NO	exonic	SART3	0	nonsyn	SART3	Spliceo
chr12	111452842	111452842	T	C	YES	splicin	ATXN	NM_0	0	0	Ataxin
chr12	112477701	112477701	T	G	NO	exonic	PTPN1	0	nonsyn	PTPN1	Protein
chr12	113388520	113388520	A	G	NO	exonic	PLBD2	0	nonsyn	PLBD2	Phosph
chr12	116007633	116007633	G	A	NO	exonic;	MED1	0	synony	MED1	Mediat
chr12	117222745	117222745	C	T	NO	exonic	NOS1	0	synony	NOS1:	Nitric o
chr12	120099139	120099139	G	T	NO	exonic	RAB35	0	synony	RAB35	Ras-ass
chr12	121444539	121444539	G	T	NO	splicin	KDM2	NM_0	0	0	Lysine
chr12	124913749	124913750	AG	-	NO	exonic	UBC	0	frames	UBC:N	Ubiqui
chr13	42166620	42166620	A	G	NO	exonic	DGKH	0	nonsyn	DGKH	Diacyl

chr13	96832764	96832764	A	G	NO	exonic	HS6ST	0	nonsyn	HS6ST	Hepara
chr13	100209369	100209369	A	G	NO	exonic	PCCA	0	nonsyn	PCCA:	Propio
chr13	113547346	113547346	G	C	NO	exonic	TMCO	0	nonsyn	TMCO	Transm
chr13	113820988	113820988	G	A	NO	exonic	GAS6	0	nonsyn	GAS6:	Growth
chr14	23396817	23396817	C	A	NO	exonic;	MYH6	0	nonsyn	MYH6	Myosin
chr14	23418370	23418370	G	A	NO	exonic	MYH7	0	nonsyn	MYH7	Myosin
chr14	23562056	23562056	G	T	NO	exonic	AP1G2	0	nonsyn	AP1G2	Adapto
chr14	28768031	28768031	A	G	NO	exonic	FOXG	0	nonsyn	FOXG	Forkhe
chr14	31175055	31175055	G	A	NO	exonic	HECT	0	synony	HECT	HECT
chr14	32093953	32093953	T	C	NO	exonic	ARHG	0	nonsyn	ARHG	RHO G
chr14	33367200	33367200	C	T	NO	exonic	NPAS3	0	stopgai	NPAS3	Neuron
chr14	50761906	50761906	C	G	NO	exonic	NIN	0	nonsyn	NIN:N	Ninein
chr14	54418310	54418310	C	T	NO	splicin	CDKN	NM_0	0	0	Cyclin-
chr14	58645319	58645319	T	C	NO	exonic	DACT	0	synony	DACT	Dapper
chr14	59330643	59330643	G	A	NO	exonic	DAAM	0	synony	DAAM	Dishev
chr14	68904669	68904669	A	C	NO	exonic	ACTN	0	nonsyn	ACTN	Actinin
chr14	73264328	73264328	C	A	NO	exonic	PAPLN	0	synony	PAPLN	Papilin
chr14	92651769	92651769	C	T	NO	exonic	RIN3	0	synony	RIN3:N	Ras an
chr14	93183303	93183303	G	A	NO	exonic	MOAP	0	nonsyn	MOAP	Modul
chr14	104988951	104988951	C	T	NO	exonic	CLBA	0	synony	CLBA1:NM_0	
chr14	105368112	105368112	G	A	NO	exonic	PACS2	0	nonsyn	PACS2	Phosph
chr15	22881890	22881890	A	C	NO	exonic	CYFIP	0	nonsyn	CYFIP	Cytopl
chr15	30140864	30140864	G	A	NO	exonic	GOLG	0	nonsyn	GOLGA8T:N	
chr15	40165114	40165114	A	G	NO	exonic	BUB1	0	nonsyn	BUB1	BUB1
chr15	43067901	43067902	TT	-	NO	exonic;	UBR1	0	frames	UBR1:	Ubiqui
chr15	55360075	55360075	A	G	NO	exonic	CCPG	0	synony	CCPG1	Cell cy
chr15	62013096	62013096	G	A	NO	exonic	VPS13	0	synony	VPS13	Vacuol
chr15	73322846	73322846	G	A	NO	exonic	HCN4	0	nonsyn	HCN4:	Hyperp
chr15	73740251	73740251	C	T	NO	exonic	INSYN	0	nonsyn	INSYN	Chrom
chr15	74134334	74134334	T	A	NO	exonic	ISLR2	0	nonsyn	ISLR2:	Immun
chr15	74821138	74821138	G	-	NO	exonic	LMAN	0	frames	LMAN	Lectin,
chr15	74821142	74821142	T	C	NO	exonic	LMAN	0	synony	LMAN	Lectin,
chr15	77018173	77018173	G	A	NO	exonic	PSTPI	0	nonsyn	PSTPI	Proline
chr15	83151234	83151234	A	G	NO	exonic	HDGF	0	nonsyn	HDGF	Hepato
chr15	100574031	100574031	G	C	NO	exonic	LINS1	0	nonsyn	LINS1:	Lines h
chr16	270763	270763	G	C	NO	exonic	RGS11	0	nonsyn	RGS11	Regula
chr16	563811	563811	G	A	NO	exonic	PRR35	0	nonsyn	PRR35:NM_1	
chr16	1207119	1207119	G	A	YES	splicin	CACN	NM_0	0	0	Calciu
chr16	2206107	2206107	G	C	NO	exonic	MLST	0	nonsyn	MLST	MTOR
chr16	2319737	2319737	C	T	NO	exonic	ABCA	0	synony	ABCA	ATP-bi
chr16	2853159	2853159	C	T	NO	exonic	PRSS2	0	synony	PRSS2	Proteas
chr16	8813039	8813039	A	G	NO	exonic	PMM2	0	nonsyn	PMM2	Phosph
chr16	8895712	8895712	A	G	NO	exonic	USP7	0	nonsyn	USP7:	Ubiqui
chr16	20944491	20944491	G	A	NO	splicin	DNAH	NM_0	0	0	Dynein
chr16	30979979	30980006	CCCG	-	NO	exonic	SETD1	0	frames	SETD1	SET do
chr16	30986894	30986894	G	A	NO	exonic	HSD3B	0	nonsyn	HSD3B	3-beta-
chr16	67200919	67200919	-	GGCC	NO	exonic	ELMO	0	nonfra	ELMO	Engulf
chr16	67200938	67200938	G	T	NO	exonic	ELMO	0	nonsyn	ELMO	Engulf
chr16	69925468	69925468	C	T	NO	exonic	WWP2	0	synony	WWP2	WW d
chr16	70840170	70840170	A	G	NO	exonic	HYDIN	0	nonsyn	HYDIN	HYDIN

chr16	72017025	72017025	T	C	NO	exonic;	DHOD	0	nonsyn	DHOD	Dihydr
chr16	72798478	72798478	G	A	NO	exonic	ZFHX3	0	nonsyn	ZFHX3	Zinc fi
chr16	75229930	75229930	G	A	NO	exonic	BCAR	0	stopgai	BCAR	BCAR
chr16	86578856	86578856	G	A	NO	exonic	FOXL	0	nonsyn	FOXL	Forkhe
chr16	88720235	88720235	C	G	NO	exonic	PIEZO	0	nonsyn	PIEZO	PIEZO
chr16	89281384	89281384	G	T	NO	exonic	ANKR	0	nonsyn	ANKR	Ankyri
chr16	89281385	89281385	C	T	NO	exonic	ANKR	0	nonsyn	ANKR	Ankyri
chr16	89529544	89529544	G	A	NO	exonic	SPG7	0	nonsyn	SPG7:	SPG7
chr16	89779902	89779902	G	A	NO	exonic	FANC	0	nonsyn	FANC	Fancon
chr17	1660505	1660505	A	C	NO	exonic	PRPF8	0	nonsyn	PRPF8	Pre-mR
chr17	4789888	4789888	G	T	NO	exonic	GLTP	0	synony	GLTPD2:NM_	
chr17	7309747	7309747	T	C	NO	exonic	EIF5A	0	nonsyn	EIF5A:	Eukary
chr17	18234405	18234405	-	A	NO	exonic;	LLGL1	0	stopgai	LLGL1	LLGL
chr17	20227575	20227578	CGGG	-	NO	exonic	SPECC	0	frames	SPECC	Sperm
chr17	20227581	20227581	-	A	NO	exonic	SPECC	0	frames	SPECC	Sperm
chr17	28901614	28901614	C	T	NO	exonic;	DHRS	0	synony	DHRS	Short-c
chr17	29562562	29562562	C	T	NO	exonic	ABHD	0	synony	ABHD	Abhyd
chr17	31181482	31181482	T	C	NO	exonic	NF1	0	nonsyn	NF1:N	Neurof
chr17	39274977	39274977	G	A	NO	exonic	FBXL2	0	nonsyn	FBXL2	F-box a
chr17	40356104	40356104	C	G	NO	exonic	RARA	0	nonsyn	RARA	Retinoi
chr17	41084531	41084531	T	C	NO	exonic	KRTA	0	nonsyn	LOC10099675	
chr17	50548407	50548407	G	C	NO	exonic	SPATA	0	nonsyn	SPATA	Sperma
chr17	58308621	58308621	G	A	NO	exonic	TSPOA	0	nonsyn	TSPOA	TSPO
chr17	58310724	58310724	T	A	NO	exonic	TSPOA	0	nonsyn	TSPOA	TSPO
chr17	62481156	62481156	C	T	NO	exonic	TLK2	0	stopgai	TLK2:	Tousle
chr17	68929691	68929691	C	A	NO	exonic	ABCA	0	nonsyn	ABCA	ATP-bi
chr17	74850233	74850233	G	A	NO	exonic	GRIN2	0	synony	GRIN2	Glutam
chr17	75502058	75502058	G	A	NO	exonic	CASKI	0	synony	CASKI	CASK-
chr17	76023650	76023650	C	T	NO	exonic;	EVPL	0	nonsyn	EVPL:	Envopl
chr17	78428653	78428656	CATA	-	NO	exonic	DNAH	0	frames	DNAH	Dynein
chr17	78437746	78437746	G	C	NO	exonic	DNAH	0	synony	DNAH	Dynein
chr17	80104655	80104655	C	T	NO	exonic	GAA	0	synony	GAA:N	Glucos
chr18	23534490	23534490	G	A	NO	exonic	NPC1	0	nonsyn	NPC1:	NPC in
chr18	23861714	23861714	A	G	NO	exonic	LAMA	0	synony	LAMA	Lamini
chr18	33744172	33744172	G	-	NO	exonic	ASXL	0	frames	ASXL	ASXL
chr18	33746172	33746172	-	AAAG	NO	exonic	ASXL	0	frames	ASXL	ASXL
chr18	46680488	46680488	G	A	NO	exonic	ST8SI	0	nonsyn	ST8SI	Sialyltr
chr18	61545029	61545029	G	A	NO	exonic;	CDH20	0	synony	CDH20	Cadher
chr19	685888	685888	C	T	NO	exonic	PRSS5	0	nonsyn	PRSS57:NM_0	
chr19	1220605	1220605	G	A	NO	exonic	STK11	0	nonsyn	STK11	Serine/
chr19	1399908	1399908	A	G	NO	exonic	GAMT	0	nonsyn	GAMT	Guanid
chr19	1785700	1785700	G	A	NO	exonic	ATP8B	0	synony	ATP8B	ATPas
chr19	3544826	3544826	G	A	NO	exonic	MFSD	0	nonsyn	MFSD	Major
chr19	3590106	3590106	C	T	NO	exonic	GIPC3	0	synony	GIPC3	GAIP
chr19	4548342	4548342	C	A	NO	exonic	SEMA	0	nonsyn	SEMA	Semap
chr19	5244382	5244382	G	A	NO	exonic	PTPRS	0	synony	PTPRS	Protein
chr19	5696264	5696264	G	A	NO	exonic	LONP	0	synony	LONP1	lon pep
chr19	6586171	6586171	C	T	NO	exonic	CD70	0	nonsyn	CD70:	CD70
chr19	7899392	7899392	G	A	NO	exonic	LRRC	0	synony	LRRC8	Leucin
chr19	8499547	8499618	CTCA	-	NO	exonic	PRAM	0	nonfra	PRAM	PML-R

chr19	8499551	8499559	GGCG	-	NO	exonic	PRAM	0	nonfra	PRAM	PML-R
chr19	8499564	8499590	TCTT	-	NO	exonic	PRAM	0	nonfra	PRAM	PML-R
chr19	12044926	12044926	G	A	NO	exonic	ZNF87	0	nonsyn	ZNF878:NM_	
chr19	12689432	12689432	G	A	NO	exonic	FBXW	0	synony	FBXW	F-box a
chr19	17436685	17436685	G	A	NO	exonic	TMEM	0	nonsyn	TMEM221:NM	
chr19	17460803	17460803	C	T	NO	exonic	NXNL	0	nonsyn	NXNL	Nucleo
chr19	17838373	17838373	C	T	NO	exonic	JAK3	0	nonsyn	JAK3:	Janus k
chr19	19542521	19542521	C	T	NO	exonic	CILP2	0	nonsyn	CILP2:	Cartila
chr19	23360788	23360788	T	C	NO	exonic	ZNF91	0	nonsyn	ZNF91	Zinc fi
chr19	30445133	30445133	G	A	NO	exonic	ZNF53	0	stopgai	ZNF53	Zinc fi
chr19	33109871	33109871	G	A	NO	exonic	GPAT	0	synony	GPATCH1:NM	
chr19	35033894	35033894	C	T	NO	exonic	SCN1B	0	synony	SCN1B	Sodium
chr19	35527793	35527793	A	G	NO	exonic	SBSN	0	synony	SBSN:	Suprab
chr19	39407807	39407807	C	T	NO	exonic	ZFP36	0	nonsyn	ZFP36:	Zfp36,
chr19	40617222	40617222	G	A	NO	exonic;	LTBP4	0	nonsyn	LTBP4	Latent
chr19	41721157	41721157	C	T	NO	exonic	CEAC	0	synony	CEAC	CEA c
chr19	44430435	44430435	A	G	NO	exonic	ZNF22	0	synony	ZNF229:NM_	
chr19	48599145	48599145	C	T	NO	exonic	SULT2	0	synony	SULT2	Sulfotr
chr19	48599330	48599330	G	A	NO	exonic	SULT2	0	nonsyn	SULT2	Sulfotr
chr19	48965528	48965528	G	A	NO	exonic	FTL	0	synony	FTL:N	Ferritin
chr19	49150096	49150096	T	C	NO	exonic	PPFIA	0	synony	PPFIA	PTPRF
chr19	50713881	50713881	C	T	NO	exonic	SHAN	0	nonsyn	SHAN	SH3 an
chr19	55231937	55231937	G	A	NO	exonic	PPP6R	0	nonsyn	PPP6R	SAPS
chr19	55401501	55401501	C	T	NO	exonic	UBE2S	0	nonsyn	UBE2S	Ubiqui
chr20	492282	492282	T	C	NO	exonic	CSNK	0	nonsyn	CSNK	Casein
chr20	1578414	1578414	G	A	NO	exonic	SIRPB	0	synony	SIRPB	Signal
chr20	17659145	17659145	-	CCCC	NO	exonic	RRBP1	0	nonfra	RRBP1	Riboso
chr20	20288767	20288767	T	C	NO	exonic	CFAP6	0	synony	CFAP6	Cilia- a
chr20	33668183	33668183	G	A	NO	exonic	ACTL	0	nonsyn	ACTL10:NM_	
chr20	33770154	33770154	A	G	NO	exonic	ZNF34	0	nonsyn	ZNF34	Zinc fi
chr20	35556122	35556122	G	A	NO	exonic	ERGIC	0	synony	ERGIC	Endopl
chr20	38138194	38138194	G	A	NO	exonic	TGM2	0	nonsyn	TGM2	Transg
chr20	46504292	46504292	T	G	NO	exonic	ZNF33	0	nonsyn	ZNF334:NM_	
chr20	49374644	49374644	G	A	NO	exonic	KCNB	0	nonsyn	KCNB	Potassi
chr20	49524233	49524233	T	C	NO	exonic	PTGIS	0	nonsyn	PTGIS	Prostag
chr20	61933091	61933091	G	C	NO	exonic	CDH4	0	nonsyn	CDH4:	Cadher
chr20	62064526	62064526	G	A	NO	exonic	TAF4	0	nonsyn	TAF4:	TAF4
chr20	62312423	62312423	C	T	NO	exonic	LAMA	0	nonsyn	LAMA	Lamini
chr20	63495062	63495062	C	T	NO	exonic	EEF1A	0	nonsyn	EEF1A	Eukary
chr20	63547122	63547122	C	T	NO	exonic	SRMS	0	synony	SRMS:	Src-rel
chr20	64106168	64106168	C	T	NO	exonic	NPBW	0	nonsyn	NPBW	G prote
chr21	41992302	41992302	G	A	NO	exonic	ZBTB2	0	synony	ZBTB2	Zinc fi
chr21	44289691	44289691	G	A	NO	exonic	AIRE	0	synony	AIRE:	Autoim
chr21	44895063	44895063	G	A	NO	splicin	ITGB2	NM_0	0	0	Integri
chr22	23160886	23160886	C	T	NO	exonic	RAB36	0	nonsyn	RAB36	Ras fa
chr22	29049687	29049687	C	A	NO	exonic	ZNRF3	0	nonsyn	ZNRF3	Zinc fi
chr22	30946442	30946442	T	C	NO	exonic	MORC	0	nonsyn	MORC	MORC
chr22	31822795	31822795	C	T	NO	splicin	DEPD	NM_0	0	0	DEP d
chr22	38081642	38081642	G	C	NO	exonic	SLC16	0	nonsyn	SLC16	Solute
chr22	46352283	46352283	G	T	NO	exonic	TRMU	0	nonsyn	TRMU	tRNA

chr22	46367791	46367791	G	A	NO	exonic	CELSR	0	synony	CELSR	Cadher
chr22	49884668	49884668	C	G	NO	exonic	ZBED4	0	nonsyn	ZBED4	Zinc fi
chr22	50289543	50289543	C	T	NO	exonic	PLXN	0	nonsyn	PLXN	Plexin
chr22	50679412	50679412	G	A	NO	exonic	SHAN	0	nonsyn	SHAN	SH3 an
chr22	50721898	50721899	TG	-	NO	exonic	SHAN	0	frames	SHAN	SH3 an

OMIM_num_03Apr2024	OMIM	Sfari_G	Sfari_S	Sfari_S	SABE1171_AC	SABE1171_AF	SABE1171_AN
103320	Myasthenic syndrome, congen				0	0	0
103320	Myasthenic syndrome, congen				0	0	0
600315	?Immunodeficiency 16, 61559				0	0	0
176873					0	0	0
615183					0	0	0
164780	Shprint	Rare Si	1	NO	0	0	0
607215	Senior-Loken syndrome 4, 60				0	0	0
609895		Rare Si	1	NO	0	0	0
604094	?Fanconi anemia, complemen				0	0	0
619069					0	0	0
604468					0	0	0
604409					0	0	0
M_001319678:exon4:c.C137T:p.T4		Rare Si	3	NO	0	0	0
_152496:exon2:c.C661T:p.R221C,MANEAL:NM_00111					0	0	0
147264					0	0	0
608271	Lissencephaly 9 with complex				0	0	0
619211					0	0	0
602913					0	0	0
618638					0	0	0
611540					0	0	0
615125					0	0	0
001002912:exon6:c.C454T:p.R152X					1	0,000427	2342
179080					0	0	0
610134					0	0	0
612866	Intellectual developmental dis				0	0	0
120280	Fibrochondrogenesis 1, 22852				0	0	0
609245	Chudley-McCullough syndro				0	0	0
615943					0	0	0
619660					0	0	0
601204					0	0	0
121013	Atrial fibrillation, familial, 11				0	0	0
611748					0	0	0
602758	Deafness, autosomal dominan				0	0	0
616284	Peeling skin syndrome 6, 618				0	0	0
158340	Tubulointerstitial kidney disea				0	0	0
191315	Insensi	Rare Si	3	NO	0	0	0
01005185:exon1:c.C69T:p.V23V					0	0	0
608067					0	0	0
611012					0	0	0
602174					0	0	0
617309					0	0	0
603768					0	0	0
603305	Zimmermann-Laband syndro				0	0	0
609275	Martsolf syndrome 1, 212720				0	0	0
600024	Pelger-Huet anomaly, 169400				0	0	0
611609					0	0	0
156570	{Neural tube defects, folate-se				0	0	0
180902	Ventricular tachycardia, catec				0	0	0

614026					0	0	0
607153					0	0	0
165640	Bachmann-Bupp syndrome, 6				0	0	0
607205					0	0	0
604464					0	0	0
602769	Tatton-	Rare Si	1	NO	1	0,000427	2342
605460	Sitosterolemia 1, 210250 (3),				0	0	0
605460	Sitosterolemia 1, 210250 (3),				0	0	0
114182	Long QT syndrome 15, 61624				0	0	0
614633					0	0	0
604221					0	0	0
616405					0	0	0
192132	Distal renal tubular acidosis 2				0	0	0
603009	Muscular dystrophy, limb-gird				0	0	0
01080474:exon3:c.C2356G:p.P786A,C2orf78:NM_0013					0	0	0
601336	Congenital disorder of glycos				0	0	0
_016044:exon5:c.T534C:p.A178A					2	0,000854	2342
601664	Retinitis pigmentosa 33, 6103				0	0	0
604494					0	0	0
607100	Joubert syndrome 4, 609583 (				0	0	0
611970					0	0	0
969:exon3:c.C892T:p.R298X					0	0	0
609697					0	0	0
182390	Seizure	Rare Si	1	NO	0	0	0
182389	Develo	Rare Si	1	NO	0	0	0
600073	Donnai	Rare Si	3	NO	0	0	0
602524					0	0	0
188840	Muscu	Rare Si	3	YES	0	0	0
616031					0	0	0
600597					0	0	0
608148	Glass s	Rare Si	3	YES	0	0	0
615950	Centronuclear myopathy 5, 61				0	0	0
603136	Neurod	Rare Si	1	NO	0	0	0
611646					0	0	0
617612	Joubert syndrome 30, 617622				0	0	0
164035					0	0	0
606678					0	0	0
73351:exon1:c.G690A:p.S230S					0	0	0
142695		Rare Si	1	NO	1	0,000427	2342
601506					0	0	0
M_001114092:exon2:c.G83A:p.S28N,THUMPD3:NM_01					0	0	0
613984	Fanconi anemia, complementa				0	0	0
137165	Myocl	Rare Si	1	NO	0	0	0
600755	{Schiz	Rare Si	3	NO	0	0	0
1080423:exon10:c.G1139T:p.G380V					0	0	0
602163					0	0	0
192321					0	0	0
615340	Nemaline myopathy 8, autoso				0	0	0
601268					0	0	0
601510					0	0	0

	605515	Intellec	Rare Si	1	NO	0	0	0
	605515	Intellec	Rare Si	1	NO	0	0	0
	612732	Coproporphyria, 121300 (3),				0	0	0
	606279					0	0	0
	612846					0	0	0
01134456:exon5:c.C262A:p.Q88K,NXPE3:NM_0013489						0	0	0
	608672					0	0	0
	608298					0	0	0
001348520:exon7:c.C242T:p.P81L,CFAP92:NM_001348						0	0	0
	614802					0	0	0
	614802					0	0	0
	617570	Polycystic kidney disease 5, 6				0	0	0
	616692					0	0	0
	601865	Bruck syndrome 2, 609220 (3				0	0	0
001308234:exon14:c.G1350A:p.L450L,ERICH6:NM_152						0	0	0
	611021					0	0	0
	620031					0	0	0
	601326	Leukodystrophy, hypomyelina				2	0,000854	2342
_001099645:exon3:c.A127G:p.K43E,RPL22L1:NM_0013						0	0	0
	601600					0	0	0
	601513	Developmental and epileptic e				0	0	0
	610780					0	0	0
	158372		Rare Si	3	NO	0	0	0
	158372		Rare Si	3	NO	0	0	0
	602052					0	0	0
	614176					0	0	0
	613004	Lopes-Maciel-Rodan syndrom				0	0	0
	604831	Ellis-van Creveld syndrome, 2				0	0	0
	605655					0	0	0
	604517					0	0	0
	613009	Pontocerebellar hypoplasia ty				0	0	0
	619104					0	0	0
	617648					0	0	0
	125485	Dentinogenesis imperfecta, Sh				0	0	0
	610306					0	0	0
	603262					0	0	0
	610514					0	0	0
_001114734:exon2:c.G97C:p.V33L,PABPC4L:NM_001						0	0	0
	608000	Intellec	Rare Si	1	NO	0	0	0
	618035					0	0	0
020724:exon6:c.T1068A:p.G356G						0	0	0
45720:exon2:c.A63G:p.L21L						0	0	0
	618642					0	0	0
	600976		Rare Si	3	NO	0	0	0
	187270	Dyskeratosis congenita, autos				0	0	0
	600783	Perrault syndrome 2, 614926 (				0	0	0
	606334					0	0	0
	616752					0	0	0
	615569					0	0	0
	600868					0	0	0

	125647	Arrhythmogenic right ventricu				0	0	0
	194540					0	0	0
	602792					0	0	0
	603978					0	0	0
	143110					0	0	0
	603382	?Premature ovarian failure 13				0	0	0
	603384	Intellec	Rare Si	1	NO	0	0	0
	147267	Charcot-Marie-Tooth disease,				0	0	0
1286655:exon6:c.C353G:p.A118G,DLK2:NM_00128665						0	0	0
	602693					0	0	0
	610201					0	0	0
	600400					0	0	0
	614064	Immunodeficiency-centromeri				0	0	0
						0	0	0
	610463	Intellectual developmental dis				0	0	0
	618844					0	0	0
	617411					0	0	0
	620171					0	0	0
	608441	Arthro	Rare Si	3	YES	0	0	0
	601835					0	0	0
_182924:exon12:c.C2199G:p.D733E						0	0	0
	603917					0	0	0
	102630	Baraits	Rare Si	1	YES	0	0	0
	619907					0	0	0
	604070					0	0	0
	606543	?Auriculocondylar syndrome				0	0	0
	603339	Ciliary dyskinesia, primary, 7,				0	0	0
	616181		Rare Si	3	NO	0	0	0
	607270	Intellec	Rare Si	1	NO	0	0	0
	605842					0	0	0
	171060	Gallbladder disease 1, 600803				0	0	0
_152789:exon1:c.G12A:p.R4R						0	0	0
	603859	Citrullinemia, type II, neonata				0	0	0
	601285					0	0	0
	607075					0	0	0
	158371					0	0	0
	158371					0	0	0
	158371					0	0	0
	600514	{Epilep	Rare Si	1	NO	0	0	0
_001012454:exon2:c.C258T:p.P86P,FAM71F2:NM_001						0	0	0
	604868					1	0,000427	2342
	163729	{Coronary artery spasm 1, sus				1	0,000427	2342
	602743	Glycogen storage disease of h				1	0,000427	2342
	606833	Kleefst	Rare Si	1	NO	0	0	0
	608532	Khan-Khan-Katsanis syndrom				0	0	0
	608136	?Slowe	Rare Si	3	NO	0	0	0
	618794					0	0	0
	607117	Microc	Rare Si	3	NO	0	0	0
	617344					0	0	0
	605352	Malignant fibrous histiocytom				0	0	0

	612182	[Acetylation, slow], 243400 (	0	0	0
	614440	Rare Si 3 NO	0	0	0
	602463	Rare Si 1 NO	0	0	0
	611605	Spastic paraplegia 18A, autos	0	0	0
	601408	Arbole Rare Si 2 YES	0	0	0
	190060	Oocyte/zygote/embryo matura	0	0	0
	190060	Oocyte/zygote/embryo matura	0	0	0
01349480:exon6:c.A992G:p.X331X,C8orf34:NM_00119			0	0	0
	611297		0	0	0
001039112:exon30:c.C3843T:p.D1281D			0	0	0
	605953		0	0	0
	188450	{Autoimmune thyroid disease	0	0	0
	602682		0	0	0
	601282	?Epidermolysis bullosa simpl	0	0	0
	601282	?Epidermolysis bullosa simpl	0	0	0
001288814:exon33:c.G3600A:p.T1200T,MROH1:NM_03			0	0	0
	600014	Nicolai Rare Si 1 YES	0	0	0
	603620		0	0	0
	602837		0	0	0
	603255		0	0	0
01039792:exon1:c.257_258insCCTCCACCACCACCAC			0	0	0
001007101:exon4:c.G1505A:p.R502Q,ZNF484:NM_0012			0	0	0
	607340	{NicotiRare Si 3 YES	0	0	0
	602926	Develo Rare Si 1 NO	0	0	0
	136510		0	0	0
	600184	?Neurodegeneration with brai	0	0	0
	600184	?Neurodegeneration with brai	0	0	0
	609795		0	0	0
207417:exon2:c.C212T:p.S71L			0	0	0
	601624		0	0	0
	603100	Lipodystrophy, congenital gen	0	0	0
	607001	Kleefst Rare Si 1 NO	0	0	0
			0	0	0
1039702:exon5:c.A304T:p.M102L,OLAH:NM_018324:e			0	0	0
	608909		0	0	0
	606808	Deafness, autosomal recessive	0	0	0
_001010863:exon1:c.A28G:p.I10V,TMEM273:NM_001			0	0	0
M_001330102:exon28:c.G3576C:p.K1192N,WASHC2A:			0	0	0
	618199		0	0	0
	605122		0	0	0
_001164375:exon2:c.G44A:p.S15N,C10orf105:NM_001			0	0	0
	601107	Dubin-Johnson syndrome, 237	0	0	0
	600417	Spastic paraplegia 45, autoso	0	0	0
	600417	Spastic paraplegia 45, autoso	0	0	0
_001011709:exon3:c.A245G:p.Y82C			0	0	0
	618764		0	0	0
	602039		0	0	0
	603413		0	0	0
	617725		0	0	0
	605561		0	0	0

	602635	Vulto-	Rare Si	1	NO	0	0	0
	158374					0	0	0
	158374					0	0	0
	600770	{Pulmonary fibrosis, idiopathi				0	0	0
	609236		Rare Si	1	NO	0	0	0
	609236		Rare Si	1	NO	0	0	0
M_001012709:exon1:c.141_142insGGCTGTGGGGGCTG						0	0	0
	142100					0	0	0
_145053:exon1:c.T287A:p.I96N						0	0	0
	608881					0	0	0
	608329	Encephalitis/encephalopathy,				0	0	0
	605888					0	0	0
	602090	Dental anomalies and short st				0	0	0
	612409					0	0	0
	600829	Opsismodysplasia, 258480 (3)				0	0	0
	620350					0	0	0
	607585	Lymphoma, B-cell non-Hodgk				0	0	0
	610748					0	0	0
	606565					0	0	0
	600644	Cleft lip/palate-ectodermal dy				0	0	0
	602005					0	0	0
001004464:exon2:c.C23T:p.T8I						0	0	0
	618319	?Birt-Hogg-Dube syndrome 2,				0	0	0
	123833	Megalencephaly-polymicrogy				0	0	0
	134921					0	0	0
	603277	Sifrim-Hitz-Weiss syndrome,				0	0	0
	616065					0	0	0
	606542					0	0	0
	148040	Epidermolysis bullosa simple				0	0	0
	616671					0	0	0
	135620					0	0	0
_152637:exon1:c.T65C:p.M22T						0	0	0
	155550					0	0	0
	603887	?Advance sleep phase syndro				0	0	0
	176256	Developmental and epileptic e				0	0	0
	607724					0	0	0
	607815		Rare Si	3	YES	0	0	0
NM_001006947:exon1:c.C15G:p.I5M,UHRF1BP1L:NM_						0	0	0
	160794	Congenital myopathy 16, 618				0	0	0
	609147					0	0	0
	611684					0	0	0
	601517	{Amyotrophic lateral sclerosi				0	0	0
	176876	Noona	Rare Si	1	NO	0	0	0
	620097					0	0	0
	608771	Impair	Rare Si	1	NO	0	0	0
	163731					0	0	0
	604199					0	0	0
	609078					0	0	0
	191340					0	0	0
	604071					0	0	0

609401		0	0	0
232000	Propio Rare Single GeYES	0	0	0
617134		0	0	0
600441		0	0	0
160710	{Sick sinus syndrome 3}, 614	0	0	0
160760	Laing distal myopathy, 16050	0	0	0
603534		0	0	0
164874	Rett sy Rare Si 1 NO	0	0	0
618649		0	0	0
602680	Rare Si 3 NO	0	0	0
609430		0	0	0
608684	?Seckel syndrome 7, 614851 (	0	0	0
123832		0	0	0
607861	Townes-Brocks syndrome 2, 6	0	0	0
606626		0	0	0
102575	Bleeding disorder, platelet-typ	0	0	0
617785		0	0	0
610223		0	0	0
609485		0	0	0
01364170:exon2:c.C432T:p.L144L,CLBA1:NM_174891		0	0	0
610423	Develo Rare Single GeYES	0	0	0
606322	Rare Si 2 NO	0	0	0
M_001355469:exon9:c.G614A:p.R205H		0	0	0
602860	Colorectal cancer, somatic, 11	0	0	0
605981	Johans Rare Si 1 NO	0	0	0
611326		0	0	0
608879	Parkinson disease 23, autosom	0	0	0
605206	Sick sinus syndrome 2, 16380	0	0	0
617128		0	0	0
614179		0	0	0
609548		0	0	0
609548		0	0	0
606347	Pyogenic sterile arthritis, pyod	0	0	0
616643		0	0	0
610350	Intellectual developmental dis	0	0	0
603895		0	0	0
45270:exon2:c.G517A:p.V173M		0	0	0
607904	{EpilepRare Si 2 NO	0	0	0
612190		0	0	0
601615	Surfactant metabolism dysfun	0	0	0
609343		0	0	0
601785	Congenital disorder of glycos	0	0	0
602519	Hao-FoRare Si 2 YES	0	0	0
603334	Rare Si 3 NO	0	0	0
611052	EpilepsRare Si 1 NO	0	0	0
607764	Bile acid synthesis defect, con	0	0	0
606422		0	0	0
606422		0	0	0
602308		0	0	0
610812	Ciliary Rare Si 3 NO	0	0	0

126064	Miller syndrome, 263750 (3),				0	0	0
104155	Prostate cancer, somatic, 1768				0	0	0
602941					0	0	0
603252	Otosclerosis 11, 620576 (3), A				0	0	0
611184	[ER blood group system], 620				0	0	0
611192	KBG s	Rare Si	1	NO	0	0	0
611192	KBG s	Rare Si	1	NO	0	0	0
602783	Spastic paraplegia 7, autosom				0	0	0
607139	Fanconi anemia, complementa				0	0	0
607300	Retinitis pigmentosa 13, 6000				0	0	0
001375801:exon3:c.G576T:p.A192A,GLTPD2:NM_001					0	0	0
600187	Faundes-Banka syndrome, 61				0	0	0
600966					0	0	0
608793					0	0	0
608793					0	0	0
616157					0	0	0
619894					0	0	0
613113	Watson	Rare Si	1	NO	0	0	0
609086					0	0	0
180240	Leukemia, acute promyelocyti				0	0	0
0:NM_001365252:exon1:c.T325C:p.C109R,KRTAP4-7:					0	0	0
613939					0	0	0
610764	Dyston	Rare Si	3	NO	0	0	0
610764	Dyston	Rare Si	3	NO	0	0	0
608439	Intellec	Rare Si	1	NO	0	0	0
612505					0	0	0
138254					0	0	0
612185					0	0	0
601590					0	0	0
610063	Sperma	Rare Si	3	NO	0	0	0
610063	Sperma	Rare Si	3	NO	0	0	0
606800	Glycogen storage disease II, 2				0	0	0
607623	Niemann-Pick disease, type C				0	0	0
600805	Epidermolysis bullosa, junctio				0	0	0
615115	Bainbr	Rare Si	1	NO	0	0	0
615115	Bainbr	Rare Si	1	NO	0	0	0
607162					0	0	0
605807					0	0	0
01308209:exon5:c.G677A:p.R226Q,PRSS57:NM_21471					0	0	0
602216	Melanoma, malignant, somati				0	0	0
601240	Cerebral creatine deficiency s				0	0	0
605866					0	0	0
617745					1	0,000427	2342
608792	Deafness, autosomal recessive				0	0	0
608873	Epilepsy, progressive myoclo				0	0	0
601576					0	0	0
605490	CODAS syndrome, 600373 (3				0	0	0
602840	Lymphoproliferative syndrom				0	0	0
612891					0	0	0
606466					0	0	0

606466		0	0	0
606466		0	0	0
001080404:exon4:c.C475T:p.R159C		0	0	0
609074		0	0	0
_001190844:exon3:c.C649T:p.R217W		0	0	0
608791		1	0,000427	2342
600173	SCID, autosomal recessive, T	0	0	0
612419		1	0,000427	2342
603971		0	0	0
618037		0	0	0
_018025:exon11:c.G1440A:p.A480A		1	0,000427	2342
600235	Generalized epilepsy with feb	0	0	0
609969		2	0,000857	2334
190700		0	0	0
604710	Cutis laxa, autosomal recessiv	0	0	0
114890		0	0	0
001278510:exon6:c.T328C:p.L110L,ZNF229:NM_01451		0	0	0
604125	Ichthyosis, congenital, autoso	0	0	0
604125	Ichthyosis, congenital, autoso	0	0	0
134790	Hyperferritinemia-cataract syn	0	0	0
603144		0	0	0
604999	Rare Si 2 NO	0	0	0
610875		0	0	0
610309		0	0	0
115440	Okur-CRare Si 1 NO	0	0	0
603889		0	0	0
601418		0	0	0
620381	Spermatogenic failure 84, 620	0	0	0
001024675:exon1:c.G686A:p.R229Q		0	0	0
618269	Hyper-IgE syndrome 3, autoso	0	0	0
616971		0	0	0
190196		0	0	0
001353814:exon3:c.A49C:p.K17Q,ZNF334:NM_0013538		0	0	0
600397	Develo Rare Si 1 NO	0	0	0
601699	Hypertension, essential, 1455	0	0	0
603006		0	0	0
601796	Intellectual developmental dis	1	0,000427	2342
601033	Nephrotic syndrome, type 26,	0	0	0
602959	Develo Rare Single GeYES	0	0	0
617797		0	0	0
600731		0	0	0
616485		0	0	0
607358	Autoimmune polyendocrinopa	0	0	0
600065	Leukocyte adhesion deficienc	0	0	0
605662		1	0,000427	2342
612062		0	0	0
616661	Charcot-Marie-Tooth disease,	0	0	0
614191	EpilepsRare Single GeYES	0	0	0
610409		0	0	0
610230	{Deafness, mitochondrial, mo	0	0	0

604523	Lymphatic malformation 9, 61			0	0	0
612552				0	0	0
604293				0	0	0
606230	Phelan Rare Si	1	NO	0	0	0
606230	Phelan Rare Si	1	NO	0	0	0

gnomad40_AF	gnomad40_AF_ra	gnomad40_AF_XX	gnomad40_AF_XY	gnomad40_AF_grp	gnomad40_faf95
0	0	0	0	0	0
0	0	0	0	0	0
0	0	0	0	0	0
0	0	0	0	0	0
0	0	0	0	0	0
0	0	0	0	0	0
0	0	0	0	0	0
0	0	0	0	0	0
0	0	0	0	0	0
0	0	0	0	0	0
0	0	0	0	0	0
0	0	0	0	0	0
0,00002626	0,00002624	0,00002569	0,00002685	0,0004	0,00000814
0,000006569	0,000006566	0	0,00001344	0,0000147	0
0,000006571	0,000006567	0	0,00001345	0,0000147	0
0	0	0	0	0	0
0	0	0	0	0	0
0,0005	0,0005	0,0003	0,0006	0,0035	0,0004
0	0	0	0	0	0
0	0	0	0	0	0
0,00001316	0,00001314	0,00001286	0,00001347	0,00002417	0,00000219
0,000006575	0,000006568	0,00001285	0	0,0000147	0
0	0	0	0	0	0
0	0	0	0	0	0
0,000006576	0,000006567	0	0,00001346	0,0000147	0
0	0	0	0	0	0
0,0003	0,0003	0,0004	0,0003	0,0008	0,0002
0	0	0	0	0	0
0	0	0	0	0	0
0	0	0	0	0	0
0	0	0	0	0	0
0	0	0	0	0	0
0	0	0	0	0	0
0	0	0	0	0	0
0	0,00003356	0	0	.	0
0	0	0	0	0	0
0	0	0	0	0	0
0	0	0	0	0	0
0	0	0	0	0	0
0	0	0	0	0	0
0,000006757	0,000006636	0,00001318	0	0,00006693	0
0,00003283	0,00003281	0,00001285	0,00005371	0,001	0,0000126
0	0	0	0	0	0
0	0	0	0	0	0
0,00002629	0,00002627	0,0000257	0,00002691	0,0004	0,00000814
0,00005911	0,00005909	0,00002568	0,00009411	0,0003	0,00003076
0	0	0	0	0	0
0	0	0	0	0	0

0	0	0	0	0	0
0,00001316	0,00001314	0,00001286	0,00001348	0,00001471	0,00000219
0,00000657	0,000006566	0,00001284	0	0,00002413	0
0	0	0	0	0	0
0,000006571	0,000006567	0	0,00001345	0,0002	0
0,00002629	0,00006567	0,0000257	0,0000269	0,0000147	0,00000814
0	0	0	0	0	0
0	0	0	0	0	0
0,00001318	0,00001314	0	0,00002698	0,00002944	0,00000219
0	0	0	0	0	0
0,000006574	0,000006567	0,00001285	0	0,0000147	0
0	0	0	0	0	0
0,00001314	0,00001312	0	0,00002687	0,0002	0,00000218
0,00001969	0,00001968	0,00001285	0,00002685	0,0004	0,00000524
0	0	0	0	0	0
0	0	0	0	0	0
0,0007	0,0007	0,0007	0,0007	0,0023	0,0006
0	0	0	0	0	0
0	0	0	0	0	0
0	0	0	0	0	0
0	0	0	0	0	0
0	0	0	0	0	0
0,00001316	0,00001314	0,00002572	0	0,00002417	0,00000219
0	0	0	0	0	0
0	0	0	0	0	0
0	0	0	0	0	0
0	0	0	0	0	0
0	0	0	0	0	0
0,0000197	0,00001969	0,00001285	0,00002687	0,00006538	0,00000524
0	0	0	0	0	0
0	0	0	0	0	0
0	0	0	0	0	0
0	0	0	0	0	0
0	0	0	0	0	0
0	0	0	0	0	0
0	0	0	0	0	0
0	0	0	0	0	0
0,00001972	0,0000197	0,00002571	0,00001346	0,00007244	0,00000524
0,000046	0,00004597	0,00003855	0,00005381	0,00006545	0,0000211
0	0	0	0	0	0
0	0	0	0	0	0
0	0	0	0	0	0
0	0	0	0	0	0
0	0	0	0	0	0
0	0	0	0	0	0
0	0	0	0	0	0
0,00001315	0,00001313	0,00001285	0,00001346	0,00002414	0,00000218
0,00001317	0,00001314	0,00001287	0,00001348	0,00002943	0,00000219
0	0	0	0	0	0
0	0	0	0	0	0

0	0	0	0	0	0
0	0	0	0	0	0
0	0	0	0	0	0
0,0002	0,0002	0,0001	0,0003	0,0062	0,0001
0	0	0	0	0	0
0	0	0	0	0	0
0	0	0	0	0	0
0	0	0	0	0	0
0	0	0	0	0	0
0	0	0	0	0	0
0	0	0	0	0	0
0	0	0	0	0	0
0	0	0	0	0	0
0	0	0	0	0	0
0	0	0	0	0	0
0	0	0	0	0	0
0	0	0	0	0	0
0,0005	0,0005	0,0004	0,0005	0,001	0,0004
0	0	0	0	0	0
0	0	0	0	0	0
0	0	0	0	0	0
0	0	0	0	0	0
0,0000661	0,0002	0,00006429	0,00006802	0,0002	0,00003237
0,00001973	0,00002627	0,00002571	0,00001346	0,0000294	0,00000524
0	0	0	0	0	0
0,00009197	0,00009194	0,0002	0,00001345	0,0002	0,00005526
0	0	0	0	0	0
0	0	0	0	0	0
0,00006566	0,00006562	0,0000514	0,00008057	0,0015	0,00003514
0,00001317	0,00001314	0,00001287	0,00001349	0,00002943	0,00000219
0	0	0	0	0	0
0,000006572	0,000006567	0,00001285	0	0,0000147	0
0	0	0	0	0	0
0	0	0	0	0	0
0	0	0	0	0	0
0,00001314	0,00001313	0	0,00002691	0,00002415	0,00000218
0	0	0	0	0	0
0	0	0	0	0	0
0	0	0	0	0	0
0	0	0	0	0	0
0	0	0	0	0	0
0,00001971	0,0000197	0,00001284	0,0000269	0,0002	0,00000524
0,0000591	0,00005906	0,00006423	0,00005374	0,0002	0,00003076
0,000006569	0,000006566	0	0,00001345	0,0000147	0
0,000006571	0,000006566	0	0,00001345	0,00002413	0
0	0	0	0	0	0
0,000006568	0,000006566	0	0,00001344	0,0000147	0
0,00003284	0,00003281	0,0000257	0,0000403	0,00009624	0,0000126
0,0001	0,0001	0,0001	0,0001	0,0004	0,00009151
0,0002	0,0002	0,0002	0,0002	0,0033	0,0001

0	0	0	0	0	0
0	0	0	0	0	0
0,0000197	0,00002625	0,00001285	0,00002687	0,0001	0,00000524
0	0	0	0	0	0
0	0	0	0	0	0
0	0	0	0	0	0
0	0	0	0	0	0
0	0	0	0	0	0
0	0	0	0	0	0
0	0	0	0	0	0
0	0	0	0	0	0
0	0	0	0	0	0
0,00001314	0,00001313	0	0,0000269	0,0002	0,00000218
0	0	0	0	0	0
0	0	0	0	0	0
0	0	0	0	0	0
0	0	0	0	0	0
0	0	0	0	0	0
0	0	0	0	0	0
0	0	0	0	0	0
0	0	0	0	0	0
0	0	0	0	0	0
0	0	0	0	0	0
0	0	0	0	0	0
0	0	0	0	0	0
0	0	0	0	0	0
0	0	0	0	0	0
0	0	0	0	0	0
0,00007888	0,0000788	0,00008996	0,00006728	0,0002	0,00004498
0	0	0	0	0	0
0,00001971	0,0000197	0,00001284	0,00002689	0,00004409	0,00000524
0	0	0	0	0	0
0	0,000006736	0	0 .		0
0	0	0	0	0	0
0	0	0	0	0	0
0,00002312	0,00004544	0,00002999	0,00001586	0,00005633	0,00000615
0,00001459	0,00004329	0,00001424	0,00001495	0,00002668	0,00000242
0,0003	0,0011	0,0003	0,0003	0,001	0,00005348
0,000006572	0,000006567	0	0,00001345	0,0002	0
0,00001315	0,00001313	0	0,00002692	0,0001	0,00000218
0,0002	0,0002	0,0002	0,0002	0,0003	0,0001
0,00002633	0,00002627	0,00001288	0,0000404	0,00004419	0,00000815
0,0000657	0,00006566	0,00001285	0,0001	0,0002	0,00003516
0,00003286	0,00003284	0,00003855	0,00002691	0,0008	0,00001261
0	0	0	0	0	0
0,000006572	0,000006567	0,00001285	0	0,0002	0
0,000006575	0,000006563	0	0,00001345	0,00002407	0
0	0	0	0	0	0
0	0	0	0	0	0
0	0	0	0	0	0

0,00001972	0,0000197	0,00001285	0,00002692	0,0000294	0,00000524
0,00001971	0,0000197	0	0,00004036	0,0002	0,00000524
0	0	0	0	0	0
0,00000657	0,000006567	0,00001285	0	0,00002413	0
0	0	0	0	0	0
0	0	0	0	0	0
0	0	0	0	0	0
0	0	0	0	0	0
0	0	0	0	0	0
0,00005259	0,00005254	0,00006426	0,00004037	0,0002	0,00002558
0	0	0	0	0	0
0	0	0	0	0	0
0,00002628	0,00002625	0,0000514	0	0,00006533	0,00000814
0	0	0	0	0	0
0	0	0	0	0	0
0	0	0	0	0	0
0,0001	0,0001	0,0001	0,0002	0,0004	0,00008661
0	0	0	0	0	0
0	0	0	0	0	0
0	0	0	0	0	0
0	0	0	0	0	0
0,00006583	0,00006568	0,00006437	0,00006734	0,0002	0,00003523
0	0	0	0	0	0
0	0	0	0	0	0
0,000006579	0,000006567	0	0,00001347	0,00002414	0
0,00002628	0,00002625	0,00002572	0,00002687	0,0008	0,00000814
0,00001976	0,00001971	0,00002573	0,00001349	0,00004413	0,00000525
0,00006564	0,00006561	0,00008993	0,00004026	0,0001	0,00003513
0,00003289	0,00003284	0,00001286	0,00005387	0,0002	0,00001262
0	0	0	0	0	0
0	0	0	0	0	0
0,00001313	0,00001312	0,00001285	0,00001343	0,0004	0,00000218
0,00001315	0,00001314	0,00002569	0	0,00002413	0,00000218
0	0	0	0	0	0
0	0	0	0	0	0
0,00000657	0,000006566	0	0,00001345	0,00002413	0
0	0	0	0	0	0
0	0	0	0	0	0
0,000006573	0,000006568	0	0,00001346	0,00002414	0
0	0	0	0	0	0
0	0	0	0	0	0
0	0	0	0	0	0
0	0	0	0	0	0
0	0	0	0	0	0
0	0	0	0	0	0
0	0	0	0	0	0
0	0	0	0	0	0
0	0	0	0	0	0
0	0	0	0	0	0
0	0	0	0	0	0

0	0	0	0	0	0
0,00002628	0,00002627	0,00003853	0,00001345	0,0002	0,00000814
0	0	0	0	0	0
0,0012	0,0013	0,0014	0,0011	0,0045	0,0011
0	0	0	0	0	0
0	0	0	0	0	0
0,00002938	0,0000762	0	0,00006094	0,0001	0,00000986
0,000006575	0,000006569	0,00001285	0	0,0002	0
0	0	0	0	0	0
0	0	0	0	0	0
0	0	0	0	0	0
0	0	0	0	0	0
0	0	0	0	0	0
0	0	0	0	0	0
0	0	0	0	0	0
0,000006576	0,000006568	0	0,00001346	0,0000147	0
0	0	0	0	0	0
0	0	0	0	0	0
0,00005256	0,0000525	0,00006428	0,00004031	0,0004	0,00002557
0	0	0	0	0	0
0,00001314	0,00001314	0,00001285	0,00001345	0,00006544	0,00000218
0	0	0	0	0	0
0	0	0	0	0	0
0,000006568	0,000006566	0	0,00001345	0,0000147	0
0,0006	0,0006	0,0003	0,0008	0,0053	0,0005
0,00001971	0,0000197	0,00002569	0,00001345	0,0000441	0,00000524
0,00001314	0,00001313	0,0000257	0	0,00002941	0,00000218
0	0	0	0	0	0
0,00009851	0,0001	0,0001	0,00006716	0,0002	0,00006004
0	0	0	0	0	0
0	0	0	0	0	0
0	0	0	0	0	0
0,000006581	0,00000657	0	0,00001348	0,0002	0
0	0	0	0	0	0
0	0	0	0	0	0
0	0	0	0	0	0
0,000006588	0,000006571	0,00001288	0	0,00001473	0
0	0	0	0	0	0
0	0	0	0	0	0
0,00001314	0,00001313	0	0,0000269	0,00006548	0,00000218
0	0	0	0	0	0
0	0	0	0	0	0
0	0	0	0	0	0
0	0	0	0	0	0
0,00004668	0,0003	0,00005195	0,00004051	0,0003	0,00001764
0,000006588	0,000006571	0,00001287	0	0,0000242	0
0	0	0	0	0	0
0	0	0	0	0	0
0	0	0	0	0	0
0	0	0	0	0	0

0	0	0	0	0	0
0	0	0	0	0	0
0	0	0	0	0	0
0,000006569	0,000006566	0,00001284	0	0,0000147	0
0	0	0	0	0	0
0,00000657	0,000006567	0	0,00001345	0,00002413	0
0	0	0	0	0	0
0	0	0	0	0	0
0	0	0	0	0	0
0,000006571	0,000006567	0	0,00001345	0,00002413	0
0	0	0	0	0	0
0	0	0	0	0	0
0	0	0	0	0	0
0	0	0	0	0	0
0	0	0	0	0	0
0	0	0	0	0	0
0	0	0	0	0	0
0,00002627	0,00002625	0	0,00005373	0,0006	0,00000814
0	0	0	0	0	0
0,0002	0,0002	0,0002	0,0002	0,0006	0,0001
0	0	0	0	0	0
0	0	0	0	0	0
0,00005109	0,00006744	0,00008538	0,00001498	0,00007468	0,0000232
0	0	0	0	0	0
0	0	0	0	0	0
0	0	0	0	0	0
0,00005929	0,00005913	0,00007725	0,00004047	0,00008848	0,00003084
0	0	0	0	0	0
0,000006571	0,000006566	0	0,00001345 .		0
0	0	0	0	0	0
0	0	0	0	0	0
0	0	0	0	0	0
0	0	0	0	0	0
0	0	0	0	0	0
0,00003285	0,00003283	0,00002569	0,00004034	0,00004825	0,00001261
0	0	0	0	0	0
0,0003	0,0003	0,0003	0,0003	0,0005	0,0002
0	0	0	0	0	0
0	0	0	0	0	0
0,00001971	0,00001969	0,00001286	0,00002688	0,00007222	0,00000524
0	0	0	0	0	0
0	0	0	0	0	0
0,000006572	0,000006567	0,00001285	0	0,00002415	0
0	0	0	0	0	0
0	0	0	0	0	0
0	0	0	0	0	0
0	0	0	0	0	0
0	0	0	0	0	0
0	0	0	0	0	0
0	0	0	0	0	0

0	0	0	0	0	0
0,00001314	0,00001314	0,00001285	0,00001345	0,0000294	0,00000218
0	0	0	0	0	0
0	0	0	0	0	0
0	0	0	0	0	0
0	0	0	0	0	0
0	0	0	0	0	0
0,00000657	0,000006566	0	0,00001345	0,00006545	0
0,0001	0,0001	0,00008991	0,0001	0,0001	0,00006511
0	0	0	0	0	0
0	0	0	0	0	0
0	0	0	0	0	0
0	0	0	0	0	0
0	0	0	0	0	0
0	0	0	0	0	0
0	0	0	0	0	0
0	0	0	0	0	0
0	0	0	0	0	0
0,00006573	0,00006568	0,00003855	0,00009418	0,00007241	0,00003518
0	0	0	0	0	0
0	0	0	0	0	0
0	0	0	0	0	0
0	0	0	0	0	0
0	0	0	0	0	0
0	0	0	0	0	0
0	0	0	0	0	0
0,00007884	0,00007874	0,00006427	0,00009406	0,0004	0,00004496
0	0	0	0	0	0
0,0000197	0,00001969	0,0000257	0,00001343	0,00002406	0,00000524
0	0	0	0	0	0
0	0	0	0	0	0
0,00002627	0,00003281	0,00003854	0,00001343	0,0002	0,00000814
0,00001971	0,0000197	0,00001285	0,00002689	0,0000294	0,00000524
0	0	0	0	0	0
0	0	0	0	0	0
0	0	0	0	0	0
0	0	0	0	0	0
0	0	0	0	0	0
0,00005258	0,00005253	0,0000514	0,00005382	0,0001	0,00002558
0	0	0	0	0	0
0,000006571	0,000006567	0,00001285	0	0,0000147	0
0,00009268	0,00009227	0,0001	0,00008137	0,0003	0,00005568
0,00003288	0,00003284	0,00001285	0,00005384	0,00007247	0,00001261
0,00001314	0,00001313	0	0,00002689	0,0002	0,00000218
0	0	0	0	0	0
0,0002	0,0002	0,0001	0,0003	0,005	0,0001
0,00001971	0,0000197	0,00003854	0	0,00004824	0,00000524
0,00001972	0,00002627	0,00001285	0,00002692	0,0000294	0,00000524
0,00006565	0,00006561	0,00002569	0,0001	0,0021	0,00003514
0,00008244	0,00008695	0,00008059	0,00008439	0,0003	0,00004688

0,00004738	0,00004681	0,0000396	0,00005556	0,0001	0,00002166
0,00004669	0,00004656	0,00003909	0,00005465	0,0001	0,00002138
0,000006582	0,00000657	0,00001286	0	0,00001471	0
0	0	0	0	0	0
0,00004601	0,00004597	0,00005138	0,00004037	0,0001	0,0000211
0,0002	0,0002	0,0003	0,0002	0,0008	0,0002
0	0	0	0	0	0
0,00005913	0,0000591	0,00007707	0,00004035	0,0001	0,00003077
0	0	0	0	0	0
0	0	0	0	0	0
0	0	0	0	0	0
0	0	0	0	0	0
0,0011	0,0011	0,001	0,0013	0,0014	0,001
0	0	0	0	0	0
0,00007879	0,00007874	0,0000257	0,0001	0,0006	0,00004493
0	0	0	0	0	0
0	0	0	0	0	0
0,0000329	0,00003284	0,00003857	0,00002695	0,00005883	0,00001262
0,00008545	0,00008533	0,00009	0,0000807	0,0002	0,00004959
0	0	0	0	0	0
0	0	0	0	0	0
0,000006573	0,000006567	0,00001285	0	0,0000147	0
0,0001	0,0001	0,0001	0,0001	0,0017	0,00009146
0,00003942	0,00003937	0,00005142	0,00002688	0,0002	0,00001715
0	0	0	0	0	0
0	0	0	0	0	0
0	0,00001835	0	0 .		0
0,00003285	0,00003283	0,00003854	0,00002689	0,0001	0,00001261
0	0	0	0	0	0
0	0	0	0	0	0
0,00001972	0,0000197	0,00003854	0	0,00006546	0,00000524
0	0	0	0	0	0
0	0	0	0	0	0
0	0	0	0	0	0
0	0	0	0	0	0
0	0	0	0	0	0
0	0	0	0	0	0
0	0	0	0	0	0
0	0	0	0	0	0
0,00003284	0,00003283	0,00001284	0,00005377	0,0002	0,0000126
0,00008544	0,00008537	0,00005139	0,0001	0,0002	0,00004958
0,00001971	0,0000197	0,00003855	0	0,0000294	0,00000524
0	0	0	0	0	0
0	0	0	0	0	0
0,0000657	0,00006566	0,00006422	0,00006725	0,0001	0,00003516
0	0	0	0	0	0
0,00004597	0,00004593	0,0000514	0,00004028	0,0004	0,00002108
0	0	0	0	0	0
0	0	0	0	0	0
0	0	0	0	0	0

0	0	0	0	0	0
0	0	0	0	0	0
0,000006567	0,000006566	0	0,00001344	0,00002411	0
0	0	0	0	0	0
0	0	0	0	0	0

gnomad40_faf99	gnomad40_fafmax	gnomad40_AF_afr	gnomad40_AF_am	gnomad40_AF_am	gnomad40_AF_asj
0	0	0	0	0	0
0	0	0	0	0	0
0	0	0	0	0	0
0	0	0	0	0	0
0	0	0	0	0	0
0	0	0	0	0	0
0	0	0	0	0	0
0	0	0	0	0	0
0	0	0	0	0	0
0	0	0	0	0	0
0	0	0	0	0	0
0	0	0	0	0	0
0,00000514	0,00002858	0,00002404	0	0	0
0	.	0	0	0	0
0	.	0	0	0	0
0	0	0	0	0	0
0	0	0	0	0	0
0,0003	0,0025	0,00004813	0	0,0035	0
0	0	0	0	0	0
0	0	0	0	0	0
0,00000082	.	0,00002417	0	0	0
0	.	0	0	0	0
0	0	0	0	0	0
0	0	0	0	0	0
0	.	0	0	0	0
0	0	0	0	0	0
0,0002	0,0004	0,0006	0	0,00006541	0
0	0	0	0	0	0
0	0	0	0	0	0
0	0	0	0	0	0
0	0	0	0	0	0
0	0	0	0	0	0
0	0	0	0	0	0
0	0	0	0	0	0
0	.	0	0	0	0
0	0	0	0	0	0
0	0	0	0	0	0
0	0	0	0	0	0
0	0	0	0	0	0
0	0	0	0	0	0
0	.	0	0	0,00006693	0
0,00000797	0,0002	0	0	0	0
0	0	0	0	0	0
0	0	0	0	0	0
0,00000514	0,00003034	0	0	0	0
0,00002209	0,00005384	0,00007234	0	0,0003	0

0	0	0	0	0	0
0,00000082	.	0	0	0	0
0	.	0,00002413	0	0	0
0	0	0	0	0	0
0	.	0	0	0	0
0,00000514	.	0	0	0	0,0006
0	0	0	0	0	0
0	0	0	0	0	0
0,00000082	0,00000183	0	0	0	0
0	0	0	0	0	0
0	.	0	0	0	0
0	0	0	0	0	0
0,00000082	.	0	0	0	0
0,00000245	0,00003034	0	0	0	0
0	0	0	0	0	0
0	0	0	0	0	0
0,0005	0,0018	0,0023	0	0,0003	0
0	0	0	0	0	0
0	0	0	0	0	0
0	0	0	0	0	0
0	0	0	0	0	0
0	0	0	0	0	0
0,00000082	.	0,00002417	0	0	0
0	0	0	0	0	0
0	0	0	0	0	0
0	0	0	0	0	0
0	0	0	0	0	0
0	0	0	0	0	0
0,00000245	0,00000298	0,00004813	0	0,00006538	0
0	0	0	0	0	0
0	0	0	0	0	0
0	0	0	0	0	0
0	0	0	0	0	0
0	0	0	0	0	0
0	0	0	0	0	0
0	0	0	0	0	0
0	0	0	0	0	0
0,00000246	0,00001032	0,00007244	0	0	0
0,00001527	0,00000625	0	0	0,00006545	0
0	0	0	0	0	0
0	0	0	0	0	0
0	0	0	0	0	0
0	0	0	0	0	0
0	0	0	0	0	0
0	0	0	0	0	0
0	0	0	0	0	0
0,00000082	.	0,00002414	0	0	0
0,00000082	0,00000183	0	0	0	0

0	0	0	0	0	0
0	0	0	0	0	0
0	0	0	0	0	0
0,0001	0,0039	0	0	0	0
0	0	0	0	0	0
0	0	0	0	0	0
0	0	0	0	0	0
0	0	0	0	0	0
0	0	0	0	0	0
0	0	0	0	0	0
0	0	0	0	0	0
0	0	0	0	0	0
0	0	0	0	0	0
0	0	0	0	0	0
0	0	0	0	0	0
0	0	0	0	0	0
0	0	0	0	0	0
0,0003	0,0007	0,001	0	0,0003	0
0	0	0	0	0	0
0	0	0	0	0	0
0	0	0	0	0	0
0	0	0	0	0	0
0,00002349	0,00007941	0,0002	0	0,0000847	0
0,00000246	0,00000183	0,00002415	0	0	0
0	0	0	0	0	0
0,00004364	0,00005994	0,00007235	0	0	0
0	0	0	0	0	0
0	0	0	0	0	0
0,00002615	0,0006	0,00002407	0	0	0
0,00000082	0,00000183	0	0	0	0
0	0	0	0	0	0
0	.	0	0	0	0
0	0	0	0	0	0
0	0	0	0	0	0
0	0	0	0	0	0
0,00000082	.	0,00002415	0	0	0
0	0	0	0	0	0
0	0	0	0	0	0
0	0	0	0	0	0
0	0	0	0	0	0
0	0	0	0	0	0
0,00000245	.	0	0	0	0,0006
0,00002209	0,00001124	0,00004812	0	0,00006534	0
0	.	0	0	0	0
0	.	0,00002413	0	0	0
0	0	0	0	0	0
0	.	0	0	0	0
0,00000798	0,0000191	0,00009624	0	0	0
0,00007706	0,0002	0,0004	0	0,0000654	0
0,0001	0,0017	0,0002	0	0	0,0014

0	0	0	0	0	0
0	0	0	0	0	0
0,00000245	0,00000906	0	0	0,0001	0
0	0	0	0	0	0
0	0	0	0	0	0
0	0	0	0	0	0
0	0	0	0	0	0
0	0	0	0	0	0
0	0	0	0	0	0
0	0	0	0	0	0
0	0	0	0	0	0
0	0	0	0	0	0
0,00000082	.	0	0	0,00006547	0
0	0	0	0	0	0
0	0	0	0	0	0
0	0	0	0	0	0
0	0	0	0	0	0
0	0	0	0	0	0
0	0	0	0	0	0
0	0	0	0	0	0
0	0	0	0	0	0
0	0	0	0	0	0
0	0	0	0	0	0
0	0	0	0	0	0
0	0	0	0	0	0
0	0	0	0	0	0
0	0	0	0	0	0
0	0	0	0	0	0
0,00003513	0,00008458	0,0002	0	0	0
0	0	0	0	0	0
0,00000245	0,00000625	0	0	0	0
0	0	0	0	0	0
0	.	0	0	0	0
0	0	0	0	0	0
0	0	0	0	0	0
0,00000271	0,00000349	0,00005633	0	0	0
0,00000091	.	0,00002668	0	0	0
0,00002145	.	0,001	0	0	0
0	.	0	0	0	0
0,00000082	0,00000908	0	0	0,0001	0
0,0001	0,0002	0,0002	0	0	0
0,00000515	0,00000625	0,00002413	0	0	0
0,00002616	0,00003052	0,0001	0	0,0002	0
0,00000798	0,0002	0,00002415	0	0	0
0	0	0	0	0	0
0	.	0	0	0	0
0	.	0,00002407	0	0	0
0	0	0	0	0	0
0	0	0	0	0	0
0	0	0	0	0	0

0,00000246	0,00000183	0,00002414	0	0	0
0,00000245	.	0,00002412	0	0	0
0	0	0	0	0	0
0	.	0,00002413	0	0	0
0	0	0	0	0	0
0	0	0	0	0	0
0	0	0	0	0	0
0	0	0	0	0	0
0	0	0	0	0	0
0,00001831	0,00001858	0,00004831	0	0	0
0	0	0	0	0	0
0	0	0	0	0	0
0,00000514	.	0	0	0,00006533	0
0	0	0	0	0	0
0	0	0	0	0	0
0	0	0	0	0	0
0,00007253	0,00005994	0,00007216	0	0,0002	0
0	0	0	0	0	0
0	0	0	0	0	0
0	0	0	0	0	0
0	0	0	0	0	0
0,00002621	0,00006969	0,0002	0	0	0
0	0	0	0	0	0
0	0	0	0	0	0
0	.	0,00002414	0	0	0
0,00000514	0,0002	0	0	0	0
0,00000246	0,00000625	0	0	0	0
0,00002614	0,00004289	0,0001	0	0,0001	0
0,00000799	0,000003	0,00004832	0	0,00006555	0
0	0	0	0	0	0
0	0	0	0	0	0
0,00000082	0,00003033	0	0	0	0
0,00000082	.	0,00002413	0	0	0
0	0	0	0	0	0
0	0	0	0	0	0
0	.	0,00002413	0	0	0
0	0	0	0	0	0
0	0	0	0	0	0
0	.	0,00002414	0	0	0
0	0	0	0	0	0
0	0	0	0	0	0
0	0	0	0	0	0
0	0	0	0	0	0
0	0	0	0	0	0
0	0	0	0	0	0
0	0	0	0	0	0
0	0	0	0	0	0
0	0	0	0	0	0
0	0	0	0	0	0
0	0	0	0	0	0

0	0	0	0	0	0
0,00000514	0,00000299	0,00004825	0	0	0
0	0	0	0	0	0
0,001	0,0037	0,0045	0	0	0
0	0	0	0	0	0
0	0	0	0	0	0
0,00000563	0,00002247	0,0001	0	0	0
0 .		0	0	0	0
0	0	0	0	0	0
0	0	0	0	0	0
0	0	0	0	0	0
0	0	0	0	0	0
0	0	0	0	0	0
0	0	0	0	0	0
0	0	0	0	0	0
0 .		0	0	0	0
0	0	0	0	0	0
0	0	0	0	0	0
0,0000183	0,00002863	0,00002408	0	0	0
0	0	0	0	0	0
0,00000082 .		0	0	0,00006544	0
0	0	0	0	0	0
0	0	0	0	0	0
0 .		0	0	0	0
0,0004	0,004	0	0	0,0053	0
0,00000245	0,00000625	0	0	0	0
0,00000082	0,00000183	0	0	0	0
0	0	0	0	0	0
0,00004877	0,00009916	0,0002	0	0,00006537	0
0	0	0	0	0	0
0	0	0	0	0	0
0	0	0	0	0	0
0 .		0	0	0	0
0	0	0	0	0	0
0	0	0	0	0	0
0	0	0	0	0	0
0 .		0	0	0	0
0	0	0	0	0	0
0	0	0	0	0	0
0,00000082 .		0	0	0,00006548	0
0	0	0	0	0	0
0	0	0	0	0	0
0	0	0	0	0	0
0	0	0	0	0	0
0,00001191	0,00000222	0,00003653	0	0,0001	0
0 .		0,0000242	0	0	0
0	0	0	0	0	0
0	0	0	0	0	0
0	0	0	0	0	0
0	0	0	0	0	0

0	0	0	0	0	0
0	0	0	0	0	0
0	0	0	0	0	0
0 .		0	0	0	0
0	0	0	0	0	0
0 .		0,00002413	0	0	0
0	0	0	0	0	0
0	0	0	0	0	0
0	0	0	0	0	0
0 .		0,00002413	0	0	0
0	0	0	0	0	0
0	0	0	0	0	0
0	0	0	0	0	0
0	0	0	0	0	0
0	0	0	0	0	0
0	0	0	0	0	0
0	0	0	0	0	0
0,00000514	0,00009003	0	0	0	0
0	0	0	0	0	0
0,0001	0,0004	0,0006	0	0	0
0	0	0	0	0	0
0	0	0	0	0	0
0,00001662	0,00001883	0,00003384	0	0,00006871	0
0	0	0	0	0	0
0	0	0	0	0	0
0	0	0	0	0	0
0,00002215	0,00002581	0,00004831	0	0,00006574	0
0	0	0	0	0	0
0 .		0	0	0	0
0	0	0	0	0	0
0	0	0	0	0	0
0	0	0	0	0	0
0	0	0	0	0	0
0	0	0	0	0	0
0,00000798	0,00000625	0,00004825	0	0	0
0	0	0	0	0	0
0,0002	0,0003	0,0002	0	0,0002	0
0	0	0	0	0	0
0	0	0	0	0	0
0,00000245	0,00001031	0,00007222	0	0	0
0	0	0	0	0	0
0	0	0	0	0	0
0 .		0,00002415	0	0	0
0	0	0	0	0	0
0	0	0	0	0	0
0	0	0	0	0	0
0	0	0	0	0	0
0	0	0	0	0	0
0	0	0	0	0	0
0	0	0	0	0	0

0	0	0	0	0	0
0,00000082	0,00000183	0	0	0	0
0	0	0	0	0	0
0	0	0	0	0	0
0	0	0	0	0	0
0	0	0	0	0	0
0	0	0	0	0	0
0 .		0	0	0,00006545	0
0,00005322	0,00003053	0,0001	0	0	0
0	0	0	0	0	0
0	0	0	0	0	0
0	0	0	0	0	0
0	0	0	0	0	0
0	0	0	0	0	0
0	0	0	0	0	0
0	0	0	0	0	0
0	0	0	0	0	0
0	0	0	0	0	0
0,00002617	0,00001032	0,00007241	0	0,00006551	0
0	0	0	0	0	0
0	0	0	0	0	0
0	0	0	0	0	0
0	0	0	0	0	0
0	0	0	0	0	0
0	0	0	0	0	0
0	0	0	0	0	0
0,00003512	0,00005088	0,00002408	0	0	0
0	0	0	0	0	0
0,00000245 .		0,00002406	0	0	0
0	0	0	0	0	0
0	0	0	0	0	0
0,00000514	0,00000625	0	0	0	0
0,00000245	0,00000183	0,00002412	0	0	0
0	0	0	0	0	0
0	0	0	0	0	0
0	0	0	0	0	0
0	0	0	0	0	0
0	0	0	0	0	0
0,00001831	0,00003054	0,0001	0	0	0
0	0	0	0	0	0
0 .		0	0	0	0
0,00004396	0,0001	0,0003	0	0,0001	0
0,00000798	0,00001033	0,00007247	0	0,00006546	0
0,00000082 .		0,00002412	0	0	0
0	0	0	0	0	0
0,0001	0,0029	0,00002408	0	0	0
0,00000245	0,00000299	0,00004824	0	0	0
0,00000245	0,00000183	0,00002414	0	0	0
0,00002614	0,0009	0	0	0	0
0,00003663	0,0001	0,0003	0	0	0

0,00001564	0,00003143	0,0001	0	0,0001	0
0,00001545	0,00003106	0,0001	0	0,0001	0
0 .		0	0	0	0
0	0	0	0	0	0
0,00001527	0,00001858	0	0	0,0001	0
0,0001	0,0005	0,0008	0	0,00006545	0
0	0	0	0	0	0
0,0000221	0,00005087	0	0	0	0
0	0	0	0	0	0
0	0	0	0	0	0
0	0	0	0	0	0
0	0	0	0	0	0
0,0009	0,0011	0,0006	0,0014	0,0012	0,0006
0	0	0	0	0	0
0,0000351	0,00008989	0,00009621	0	0	0,0003
0	0	0	0	0	0
0	0	0	0	0	0
0,00000799	0,00001125	0,00002417	0	0	0
0,00003964	0,00009921	0,0002	0	0	0
0	0	0	0	0	0
0	0	0	0	0	0
0 .		0	0	0	0
0,00007702	0,0006	0,00002407	0	0,0001	0
0,00001129	0,00001125	0,00002407	0	0	0
0	0	0	0	0	0
0	0	0	0	0	0
0 .		0	0	0	0
0,00000798	0,00003052	0,0001	0	0	0
0	0	0	0	0	0
0	0	0	0	0	0
0,00000245	0,00000183	0	0	0,00006546	0
0	0	0	0	0	0
0	0	0	0	0	0
0	0	0	0	0	0
0	0	0	0	0	0
0	0	0	0	0	0
0	0	0	0	0	0
0	0	0	0	0	0
0	0	0	0	0	0
0,00000798 .		0,00002411	0	0,00006539	0
0,00003964	0,00006964	0,0002	0	0	0
0,00000245	0,00000183	0,00002413	0	0	0
0	0	0	0	0	0
0	0	0	0	0	0
0,00002616	0,00004299	0,0001	0	0	0
0	0	0	0	0	0
0,00001526	0,00002858	0,00004811	0	0	0
0	0	0	0	0	0
0	0	0	0	0	0
0	0	0	0	0	0

0	0	0	0	0	0
0	0	0	0	0	0
0 .		0,00002411	0	0	0
0	0	0	0	0	0
0	0	0	0	0	0

gnomad40_AF_eas	gnomad40_AF_fin	gnomad40_AF_mi	gnomad40_AF_nfe	gnomad40_AF_re	gnomad40_AF_sas
0	0	0	0	0	0
0	0	0	0	0	0
0	0	0	0	0	0
0	0	0	0	0	0
0	0	0	0	0	0
0	0	0	0	0	0
0	0	0	0	0	0
0	0	0	0	0	0
0	0	0	0	0	0
0	0	0	0	0	0
0	0	0	0	0	0
0	0	0	0	0	0
0,0004	0	0	0,0000147	0	0
0	0	0	0,0000147	0	0
0	0	0	0,0000147	0	0
0	0	0	0	0	0
0	0	0	0	0	0
0	0	0	0,0002	0	0,0004
0	0	0	0	0	0
0	0	0	0	0	0
0	0,00009459	0	0	0	0
0	0	0	0,0000147	0	0
0	0	0	0	0	0
0	0	0	0	0	0
0	0	0	0,0000147	0	0
0	0	0	0	0	0
0,0008	0	0	0,0002	0,0005	0,0002
0	0	0	0	0	0
0	0	0	0	0	0
0	0	0	0	0	0
0	0	0	0	0	0
0	0	0	0	0	0
0	0	0	0	0	0
0	0	0	0	0	0
0	0	0	0	0	0
0	0	0	0	0	0
0	0	0	0	0	0
0	0	0	0	0	0
0	0	0	0	0	0
0	0	0	0	0	0
0	0	0	0	0	0
0,001	0	0	0	0	0
0	0	0	0	0	0
0	0	0	0	0	0
0,0002	0	0	0,0000147	0	0,0004
0	0	0	0,00002939	0	0
0	0	0	0	0	0
0	0	0	0	0	0

0	0	0	0	0	0
0	0,00009482	0	0,00001471	0	0
0	0	0	0	0	0
0	0	0	0	0	0
0	0	0	0	0	0,0002
0	0,00009418	0	0,0000147	0	0
0	0	0	0	0	0
0	0	0	0	0	0
0	0	0	0,00002944	0	0
0	0	0	0	0	0
0	0	0	0,0000147	0	0
0	0	0	0	0	0
0,0002	0	0	0,0000147	0	0
0	0	0	0,0000147	0	0,0004
0	0	0	0	0	0
0	0	0	0	0	0
0	0	0	0,0000147	0	0,0002
0	0	0	0	0	0
0	0	0	0	0	0
0	0	0	0	0	0
0	0	0	0	0	0
0	0	0	0	0	0
0	0	0	0,00001471	0	0
0	0	0	0	0	0
0	0	0	0	0	0
0	0	0	0	0	0
0	0	0	0	0	0
0	0	0	0	0	0
0	0	0	0	0	0
0	0	0	0	0	0
0	0	0	0	0	0
0	0	0	0	0	0
0	0	0	0	0	0
0	0	0	0	0	0
0	0	0	0	0	0
0	0	0	0	0	0
0	0	0	0	0	0
0	0	0	0	0	0
0	0,0002	0	0,00004409	0,0005	0
0	0	0	0	0	0
0	0	0	0	0	0
0	0	0	0	0	0
0	0	0	0	0	0
0	0	0	0	0	0
0	0	0	0	0	0
0	0	0	0	0	0
0	0	0	0,0000147	0	0
0	0	0	0,00002943	0	0
0	0	0	0	0	0
0	0	0	0	0	0

0	0	0	0	0	0
0	0	0	0	0	0
0	0	0	0	0	0
0	0	0	0,00001471	0	0,0062
0	0	0	0	0	0
0	0	0	0	0	0
0	0	0	0	0	0
0	0	0	0	0	0
0	0	0	0	0	0
0	0	0	0	0	0
0	0	0	0	0	0
0	0	0	0	0	0
0	0	0	0	0	0
0	0	0	0	0	0
0	0	0	0	0	0
0	0	0	0	0	0
0	0	0	0	0	0
0	0,00009422	0	0,0004	0	0
0	0	0	0	0	0
0	0	0	0	0	0
0	0	0	0	0	0
0	0	0	0	0	0
0	0	0	0	0	0
0	0	0	0,0000294	0	0
0	0	0	0	0	0
0,0002	0	0	0,0001	0	0
0	0	0	0	0	0
0	0	0	0	0	0
0,0015	0	0	0,0000147	0	0
0	0	0	0,00002943	0	0
0	0	0	0	0	0
0	0	0	0,0000147	0	0
0	0	0	0	0	0
0	0	0	0	0	0
0	0	0	0	0	0
0	0	0	0,0000147	0	0
0	0	0	0	0	0
0	0	0	0	0	0
0	0	0	0	0	0
0	0	0	0	0	0
0	0	0	0	0	0
0	0	0	0	0	0,0002
0,0002	0	0	0,0000588	0	0,0002
0	0	0	0,0000147	0	0
0	0	0	0	0	0
0	0	0	0	0	0
0	0	0	0,0000147	0	0
0	0	0	0,0000147	0	0
0	0	0	0,00004412	0	0
0	0	0	0,0000147	0	0,0033

0	0	0	0	0	0
0	0	0	0	0	0
0	0	0	0,0000147	0	0
0	0	0	0	0	0
0	0	0	0	0	0
0	0	0	0	0	0
0	0	0	0	0	0
0	0	0	0	0	0
0	0	0	0	0	0
0	0	0	0	0	0
0	0	0	0	0	0
0	0	0	0	0	0
0	0	0	0	0	0,0002
0	0	0	0	0	0
0	0	0	0	0	0
0	0	0	0	0	0
0	0	0	0	0	0
0	0	0	0	0	0
0	0	0	0	0	0
0	0	0	0	0	0
0	0	0	0	0	0
0	0	0	0	0	0
0	0	0	0	0	0
0	0	0	0	0	0
0	0	0	0	0	0
0	0	0	0	0	0
0	0	0	0	0	0
0	0	0	0	0	0
0	0	0	0,0000294	0,0005	0
0	0	0	0	0	0
0	0	0	0,00004409	0	0
0	0	0	0	0	0
0	0	0	0	0	0
0	0	0	0	0	0
0	0	0	0	0	0
0	0	0	0,00001684	0	0
0	0	0	0,00001586	0	0
0	0	0	0,0002	0	0
0,0002	0	0	0	0	0
0	0	0	0	0	0
0	0,00009425	0	0,0003	0	0
0	0	0	0,00004419	0	0
0	0	0	0,0000147	0,0005	0
0,0008	0	0	0	0	0
0	0	0	0	0	0
0	0	0	0	0	0,0002
0	0	0	0	0	0
0	0	0	0	0	0
0	0	0	0	0	0
0	0	0	0	0	0

0	0	0	0,0000294	0	0
0	0	0	0,0000147	0	0,0002
0	0	0	0	0	0
0	0	0	0	0	0
0	0	0	0	0	0
0	0	0	0	0	0
0	0	0	0	0	0
0	0	0	0	0	0
0	0	0	0	0	0
0	0	0	0,00007349	0	0,0002
0	0	0	0	0	0
0	0	0	0	0	0
0	0,0000942	0	0,00001471	0,0005	0
0	0	0	0	0	0
0	0	0	0	0	0
0	0	0	0	0	0
0,0002	0	0	0,0001	0,0005	0,0004
0	0	0	0	0	0
0	0	0	0	0	0
0	0	0	0	0	0
0	0	0	0	0	0
0	0	0	0,00002944	0	0
0	0	0	0	0	0
0	0	0	0	0	0
0	0	0	0	0	0
0,0008	0	0	0	0	0
0	0	0	0,00004413	0	0
0	0	0	0,0000294	0	0
0,0002	0	0	0,00001471	0	0
0	0	0	0	0	0
0	0	0	0	0	0
0	0	0	0	0	0,0004
0	0	0	0,0000147	0	0
0	0	0	0	0	0
0	0	0	0	0	0
0	0	0	0	0	0
0	0	0	0	0	0
0	0	0	0	0	0
0	0	0	0	0	0
0	0	0	0	0	0
0	0	0	0	0	0
0	0	0	0	0	0
0	0	0	0	0	0
0	0	0	0	0	0
0	0	0	0	0	0
0	0	0	0	0	0
0	0	0	0	0	0
0	0	0	0	0	0
0	0	0	0	0	0
0	0	0	0	0	0

0	0	0	0	0	0
0	0	0	0,0000147	0	0,0002
0	0	0	0	0	0
0	0	0,0034	0	0,0014	0
0	0	0	0	0	0
0	0	0	0	0	0
0	0	0	0	0	0
0,0002	0	0	0	0	0
0	0	0	0	0	0
0	0	0	0	0	0
0	0	0	0	0	0
0	0	0	0	0	0
0	0	0	0	0	0
0	0	0	0	0	0
0	0	0	0	0	0
0	0	0	0,0000147	0	0
0	0	0	0	0	0
0	0	0	0	0	0
0,0004	0	0	0,00005882	0	0,0002
0	0	0	0	0	0
0	0	0	0,0000147	0	0
0	0	0	0	0	0
0	0	0	0	0	0
0	0	0	0,0000147	0	0
0	0	0	0,00007349	0	0,0002
0	0	0	0,0000441	0	0
0	0	0	0,00002941	0	0
0	0	0	0	0	0
0	0	0	0,0000588	0	0
0	0	0	0	0	0
0	0	0	0	0	0
0	0	0	0	0	0
0	0	0	0	0	0,0002
0	0	0	0	0	0
0	0	0	0	0	0
0	0	0	0	0	0
0	0	0	0,00001473	0	0
0	0	0	0	0	0
0	0	0	0	0	0
0	0	0	0,0000147	0	0
0	0	0	0	0	0
0	0	0	0	0	0
0	0	0	0	0	0
0	0	0	0	0	0
0	0	0	0,00003578	0	0,0003
0	0	0	0	0	0
0	0	0	0	0	0
0	0	0	0	0	0
0	0	0	0	0	0
0	0	0	0	0	0

0	0	0	0	0	0
0	0	0	0	0	0
0	0	0	0	0	0
0	0	0	0,0000147	0	0
0	0	0	0	0	0
0	0	0	0	0	0
0	0	0	0	0	0
0	0	0	0	0	0
0	0	0	0	0	0
0	0	0	0	0	0
0	0	0	0	0	0
0	0	0	0	0	0
0	0	0	0	0	0
0	0	0	0	0	0
0	0	0	0	0	0
0	0	0	0	0	0
0	0	0	0	0	0
0	0	0	0,0000147	0	0,0006
0	0	0	0	0	0
0	0	0	0	0	0
0	0	0	0	0	0
0	0	0	0	0	0
0	0	0	0,00007468	0	0
0	0	0	0	0	0
0	0	0	0	0	0
0	0	0	0	0	0
0	0	0	0,00008848	0	0
0	0	0	0	0	0
0	0	0	0	0,0005	0
0	0	0	0	0	0
0	0	0	0	0	0
0	0	0	0	0	0
0	0	0	0	0	0
0	0	0	0	0	0
0	0	0	0,00004409	0	0
0	0	0	0	0	0
0	0	0	0,0005	0,0014	0
0	0	0	0	0	0
0	0	0	0	0	0
0	0	0	0	0	0
0	0	0	0	0	0
0	0	0	0	0	0
0	0	0	0	0	0
0	0	0	0	0	0
0	0	0	0	0	0
0	0	0	0	0	0
0	0	0	0	0	0
0	0	0	0	0	0
0	0	0	0	0	0
0	0	0	0	0	0

0	0	0	0	0	0
0	0	0	0,0000294	0	0
0	0	0	0	0	0
0	0	0	0	0	0
0	0	0	0	0	0
0	0	0	0	0	0
0	0	0	0	0	0
0	0	0	0	0	0
0	0,0009	0	0,0000147	0	0
0	0	0	0	0	0
0	0	0	0	0	0
0	0	0	0	0	0
0	0	0	0	0	0
0	0	0	0	0	0
0	0	0	0	0	0
0	0	0	0	0	0
0	0	0	0	0	0
0	0	0	0	0	0
0	0,0005	0	0,0000147	0	0
0	0	0	0	0	0
0	0	0	0	0	0
0	0	0	0	0	0
0	0	0	0	0	0
0	0	0	0	0	0
0	0	0	0	0	0
0	0	0	0	0	0
0	0	0	0,0001	0	0,0004
0	0	0	0	0	0
0	0	0	0,0000147	0,0005	0
0	0	0	0	0	0
0	0	0	0	0	0
0,0002	0	0	0,00004411	0	0
0	0	0	0,0000294	0	0
0	0	0	0	0	0
0	0	0	0	0	0
0	0	0	0	0	0
0	0	0	0	0	0
0	0	0	0	0	0
0	0,0000942	0	0,0000294	0	0
0	0	0	0	0	0
0	0	0	0,0000147	0	0
0	0	0	0	0	0
0	0	0	0,00001471	0	0
0	0	0	0	0	0,0002
0	0	0	0	0	0
0	0,00009414	0	0,00005881	0	0,005
0	0	0	0,00001471	0	0
0	0	0	0,0000294	0	0
0	0	0	0	0	0,0021
0	0	0	0	0	0

0	0	0	0	0	0
0	0	0	0	0	0
0	0	0	0,00001471	0	0
0	0	0	0	0	0
0	0	0	0,0000735	0	0
0	0	0	0	0,0005	0
0	0	0	0	0	0
0	0	0	0,0001	0	0
0	0	0	0	0	0
0	0	0	0	0	0
0	0	0	0	0	0
0	0	0	0	0	0
0,0002	0,002	0	0,0014	0,0017	0,0002
0	0	0	0	0	0
0	0	0	0,00005881	0	0,0006
0	0	0	0	0	0
0	0	0	0	0	0
0	0	0	0,00005883	0	0
0,0002	0	0	0,00002942	0	0
0	0	0	0	0	0
0	0	0	0	0	0
0	0	0	0,0000147	0	0
0	0	0	0,0001	0	0,0017
0,0002	0	0	0,00005881	0	0
0	0	0	0	0	0
0	0	0	0	0	0
0	0	0	0	0	0
0	0	0	0	0	0
0	0	0	0	0	0
0	0	0	0	0	0
0	0	0	0,0000294	0	0
0	0	0	0	0	0
0	0	0	0	0	0
0	0	0	0	0	0
0	0	0	0	0	0
0	0	0	0	0	0
0	0	0	0	0	0
0	0	0	0	0	0
0	0	0	0	0	0
0	0	0	0,0000147	0,0005	0,0002
0	0	0	0,00005882	0	0,0002
0	0	0	0,0000294	0	0
0	0	0	0	0	0
0	0	0	0	0	0
0	0	0	0,0000588	0	0
0	0	0	0	0	0
0,0004	0	0	0,00004411	0	0
0	0	0	0	0	0
0	0	0	0	0	0
0	0	0	0	0	0

0	0	0	0	0	0
0	0	0	0	0	0
0	0	0	0	0	0
0	0	0	0	0	0
0	0	0	0	0	0

gnomAD3.1_AC	gnomAD3.1_AF	gnomAD3.1_AN	gnomAD_exome_	gnomAD_exome_	gnomAD_exome_
0	0	0	0	0	0
0	0	0	0	0	0
0	0	0	0,000005894	0	0
0	0	0	0	0	0
0	0	0	0	0	0
0	0	0	0	0	0
0	0	0	0	0	0
0	0	0	0	0	0
0	0	0	0	0	0
0	0	0	0	0	0
0	0	0	0	0	0
0	0	0	0	0	0
4	2,62778E-05	152220	0,0000732	0,0001	0
1	6,56858E-06	152240	0,000008202	0	0
1	6,57065E-06	152192	0,000008264	0	0
0	0	0	0	0	0
0	0	0	0	0	0
69	0,00045341	152180	0,0004	0	0,0008
0	0	0	0	0	0
0	0	0	0,000004063	0	0
2	0,000013157	152010	0,00004068	0,00006537	0,0001
1	6,57523E-06	152086	0,000004109	0	0
0	0	0	0	0	0
0	0	0	0	0	0
1	6,57601E-06	152068	0	0	0
0	0	0	0	0	0
48	0,000315711	152038	0,0003	0,0006	0,00002978
0	0	0	0	0	0
0	0	0	0	0	0
0	0	0	0	0	0
0	0	0	0	0	0
0	0	0	0	0	0
0	0	0	0,00000407	0	0
0	0	0	0	0	0
0	0	0	0,00003171	0	0,00004891
0	0	0	0	0	0
0	0	0	0	0	0
0	0	0	0	0	0
0	0	0	0	0	0
0	0	0	0	0	0
1	6,75667E-06	148002	0	0	0
5	3,28567E-05	152176	0,0001	0	0
0	0	0	0	0	0
0	0	0	0,0000122	0	0
4	0,000026293	152132	0,0000203	0	0,00008933
9	5,91086E-05	152262	0,00006094	0,0001	0,0003

0	0	0	0	0	0
2	1,31633E-05	151938	0,00007309	0	0
1	6,57013E-06	152204	0	0	0
0	0	0	0,000004066	0	0
1	6,57099E-06	152184	0	0	0
4	2,62864E-05	152170	0,000006667	0	0
0	0	0	0	0	0
0	0	0	0	0	0
2	1,31763E-05	151788	0,000004108	0	0
0	0	0	0	0	0
1	6,57367E-06	152122	0,0000244	0	0,0001
0	0	0	0	0	0
2	0,000013146	152138	0	0	0
3	1,97096E-05	152210	0,00004472	0	0
0	0	0	0	0	0
0	0	0	0	0	0
101	0,000663557	152210	0,0003	0,0027	0,0007
0	0	0	0	0	0
0	0	0	0	0	0
0	0	0	0	0	0
0	0	0	0	0	0
0	0	0	0	0	0
2	1,31575E-05	152004	0,000008131	0	0,00005957
0	0	0	0	0	0
0	0	0	0	0	0
0	0	0	0	0	0
0	0	0	0	0	0
0	0	0	0	0	0
3	1,97189E-05	152138	0,00002851	0,0001	0,00002982
0	0	0	0	0	0
0	0	0	0	0	0
0	0	0	0	0	0
0	0	0	0	0	0
0	0	0	0	0	0
0	0	0	0	0	0
0	0	0	0	0	0
0	0	0	0	0	0
3	1,97246E-05	152094	0,00001625	0	0
7	4,60036E-05	152162	0,00006099	0	0
0	0	0	0	0	0
0	0	0	0	0	0
0	0	0	0	0	0
0	0	0	0	0	0
0	0	0	0,000008207	0	0
0	0	0	0	0	0
0	0	0	0	0	0
2	0,000013147	152126	0,00001219	0,00006534	0
2	1,31712E-05	151846	0,00001229	0	0
0	0	0	0	0	0
0	0	0	0,00001074	0	0

0	0	0	0	0	0
0	0	0	0	0	0
0	0	0	0	0	0
31	0,000203958	151992	0,0006	0	0,00003052
0	0	0	0	0	0
0	0	0	0	0	0
0	0	0	0	0	0
0	0	0	0	0	0
0	0	0	0	0	0
0	0	0	0	0	0
0	0	0	0	0	0
0	0	0	0	0	0
0	0	0	0	0	0
0	0	0	0	0	0
0	0	0	0	0	0
0	0	0	0	0	0
0	0	0	0	0	0
67	0,000440419	152128	0,0003	0,0012	0,0004
0	0	0	0	0	0
0	0	0	0	0	0
0	0	0	0	0	0
0	0	0	0	0	0
8	6,61474E-05	120942	0,00002782	0,0003	0,00004343
3	1,97265E-05	152080	0,00001625	0	0
0	0	0	0	0	0
14	9,19685E-05	152226	0,0002	0,00006554	0,00008938
0	0	0	0	0	0
0	0	0	0	0	0
10	6,57142E-05	152174	0,00006501	0	0
2	1,31704E-05	151856	0,00001628	0	0
0	0	0	0	0	0
1	6,57246E-06	152150	0,000008122	0	0
0	0	0	0	0	0
0	0	0	0	0	0
0	0	0	0	0	0
2	1,31446E-05	152154	0,0000244	0	0
0	0	0	0	0	0
0	0	0	0	0	0
0	0	0	0	0	0
0	0	0	0,00001627	0	0,00005957
0	0	0	0,000004063	0	0
3	1,97091E-05	152214	0,00002859	0	0
9	5,91467E-05	152164	0,00006501	0	0,00002978
1	6,56901E-06	152230	0,000004063	0	0
1	6,57091E-06	152186	0,000006972	0	0
0	0	0	0	0	0
1	6,56771E-06	152260	0	0	0
5	0,000032861	152156	0,00004061	0,0001	0,00002978
20	0,000131536	152050	0,00002843	0,0002	0
29	0,000190584	152164	0,0008	0,0002	0

0	0	0	0	0	0
0	0	0	0	0	0
3	1,97171E-05	152152	0,00001624	0	0
0	0	0	0	0	0
0	0	0	0	0	0
0	0	0	0,000008301	0	0
0	0	0	0	0	0
0	0	0	0,00002444	0	0
0	0	0	0	0	0
0	0	0	0	0	0
0	0	0	0	0	0
0	0	0	0	0	0
2	1,31434E-05	152168	0,000004066	0	0
0	0	0	0	0	0
0	0	0	0	0	0
0	0	0	0	0	0
0	0	0	0	0	0
0	0	0	0	0	0
0	0	0	0	0	0
0	0	0	0	0	0
0	0	0	0	0	0
0	0	0	0	0	0
0	0	0	0	0	0
0	0	0	0	0	0
0	0	0	0	0	0
0	0	0	0	0	0
0	0	0	0	0	0
0	0	0	0	0	0
12	7,88799E-05	152130	0,00004107	0,0002	0
0	0	0	0	0	0
3	1,97055E-05	152242	0	0	0
0	0	0	0	0	0
0	0	0	0	0	0
0	0	0	0	0	0
0	0	0	0	0	0
3	2,31385E-05	129654	0	0	0
2	1,45979E-05	137006	0	0	0
2	0,000302572	6610	0	0	0
1	6,57229E-06	152154	0,000004072	0	0
2	1,31468E-05	152128	0,00001219	0	0,00008933
28	0,000184106	152086	0,00009349	0,00006535	0,00005957
4	2,63508E-05	151798	0,00002745	0	0,00006401
10	6,57047E-05	152196	0,00001625	0,0001	0,00002978
5	3,28632E-05	152146	0,00009777	0	0
0	0	0	0	0	0
1	6,57212E-06	152158	0,00001218	0	0
1	6,57921E-06	151994	0,000004989	0,00009558	0
0	0	0	0,00004883	0	0
0	0	0	0	0	0
0	0	0	0,00000534	0	0

3	1,97241E-05	152098	0,00004083	0	0
3	1,97143E-05	152174	0,00002031	0,00006543	0
0	0	0	0	0	0
1	6,56961E-06	152216	0	0	0
0	0	0	0	0	0
0	0	0	0	0	0
0	0	0	0	0	0
0	0	0	0	0	0
0	0	0	0	0	0
8	0,000052588	152126	0,00002848	0	0
0	0	0	0	0	0
0	0	0	0	0	0
4	2,62961E-05	152114	0,0000549	0	0,0002
0	0	0	0	0	0
0	0	0	0	0	0
0	0	0	0,00002338	0	0,0001
20	0,000131441	152160	0,0005	0	0,0028
0	0	0	0	0	0
0	0	0	0	0	0
0	0	0	0	0	0
0	0	0	0	0	0
10	0,000065877	151798	0,00002438	0,0002	0
0	0	0	0	0	0
0	0	0	0	0	0
1	6,57877E-06	152004	0	0	0
4	2,63023E-05	152078	0	0	0
3	1,97553E-05	151858	0,000004331	0	0
8	5,25514E-05	152232	0,00004488	0,0002	0,00002985
5	3,28917E-05	152014	0,00005693	0,00006539	0
0	0	0	0,000008141	0	0
0	0	0	0	0	0
2	1,31423E-05	152180	0,000008132	0	0
2	1,31463E-05	152134	0,00001308	0	0
0	0	0	0	0	0
0	0	0	0	0	0
1	6,57022E-06	152202	0,00001219	0	0
0	0	0	0	0	0
0	0	0	0	0	0
1	6,57315E-06	152134	0,000004074	0,00006541	0
0	0	0	0	0	0
0	0	0	0	0	0
0	0	0	0	0	0
0	0	0	0	0	0
0	0	0	0	0	0
0	0	0	0	0	0
0	0	0	0	0	0
0	0	0	0	0	0
0	0	0	0	0	0
0	0	0	0	0	0
0	0	0	0,000004586	0	0

0	0	0	0	0	0
4	2,62809E-05	152202	0,00007005	0	0
0	0	0	0	0	0
187	0,00124415	150304	0,0002	0,0024	0,00005959
0	0	0	0	0	0
0	0	0	0	0	0
0	0	0	0,00004098	0,0004	0,00005963
1	6,57523E-06	152086	0,00001628	0,00006536	0
0	0	0	0	0	0
0	0	0	0	0	0
0	0	0	0	0	0
0	0	0	0	0	0
0	0	0	0	0	0
0	0	0	0	0	0
0	0	0	0,000004069	0	0
1	6,57557E-06	152078	0	0	0
0	0	0	0,000004068	0	0
0	0	0	0	0	0
8	5,25997E-05	152092	0,00006506	0,00006548	0
0	0	0	0	0	0
2	1,31444E-05	152156	0,00001624	0	0
0	0	0	0	0	0
0	0	0	0	0	0
1	6,56832E-06	152246	0	0	0
87	0,000571721	152172	0,0001	0	0,0009
3	1,97106E-05	152202	0	0	0
2	1,31444E-05	152156	0,000004078	0	0
0	0	0	0	0	0
15	9,85869E-05	152150	0,00006518	0,00006601	0,0001
0	0	0	0	0	0
0	0	0	0	0	0
0	0	0	0	0	0
1	6,58051E-06	151964	0	0	0
0	0	0	0	0	0
0	0	0	0	0	0
0	0	0	0,00001271	0	0,00003252
1	6,5884E-06	151782	0,000004086	0	0
0	0	0	0	0	0
0	0	0	0	0	0
2	1,31423E-05	152180	0,00001632	0	0,00002989
0	0	0	0,00001624	0	0
0	0	0	0,000004263	0	0
0	0	0	0	0	0
0	0	0	0	0	0
0	0	0	0,00001992	0	0
1	6,58779E-06	151796	0,000008127	0	0
0	0	0	0	0	0
0	0	0	0,000004063	0	0,00002978
0	0	0	0,000004063	0	0
0	0	0	0,000004181	0	0

0	0	0	0,000004062	0	0
0	0	0	0,000004063	0	0
0	0	0	0	0	0
1	6,56927E-06	152224	0,000008181	0	0
0	0	0	0	0	0
1	6,56996E-06	152208	0,000004079	0	0
0	0	0	0	0	0
0	0	0	0	0	0
0	0	0	0	0	0
1	6,57117E-06	152180	0,000008186	0	0
0	0	0	0	0	0
0	0	0	0	0	0
0	0	0	0,000008294	0	0
0	0	0	0	0	0
0	0	0	0	0	0
0	0	0	0	0	0
0	0	0	0	0	0
4	2,62919E-05	152138	0,00004468	0,00006534	0
0	0	0	0	0	0
26	0,000170814	152212	0,00003711	0,0005	0
0	0	0	0	0	0
0	0	0	0	0	0
7	5,10882E-05	137018	0,0002	0,0001	0,0007
0	0	0	0	0	0
0	0	0	0	0	0
0	0	0	0,000008186	0	0
9	5,92862E-05	151806	0,00004523	0	0
0	0	0	0	0	0
1	6,57117E-06	152180	0,000004074	0	0
0	0	0	0	0	0
0	0	0	0	0	0
0	0	0	0	0	0
0	0	0	0	0	0
0	0	0	0	0	0
5	3,28472E-05	152220	0,00006098	0	0
0	0	0	0	0	0
46	0,000302357	152138	0,0004	0,0001	0,00005184
0	0	0	0	0	0
0	0	0	0	0	0
3	1,97301E-05	152052	0,0000244	0,0002	0
0	0	0	0	0	0
0	0	0	0,000004061	0	0,00002978
1	6,57177E-06	152166	0	0	0
0	0	0	0	0	0
0	0	0	0	0	0
0	0	0	0	0	0
0	0	0	0	0	0
0	0	0	0	0	0
0	0	0	0	0	0
0	0	0	0	0	0

0	0	0	0	0	0
2	1,31435E-05	152166	0,00002032	0	0,00002978
0	0	0	0	0	0
0	0	0	0,000004067	0	0
0	0	0	0	0	0
0	0	0	0	0	0
0	0	0	0	0	0
1	6,57047E-06	152196	0,00002851	0	0,00005961
16	0,000105125	152200	0,00009353	0	0,00002978
0	0	0	0	0	0
0	0	0	0,000005951	0	0
0	0	0	0	0	0
0	0	0	0,000004116	0	0
0	0	0	0	0	0
0	0	0	0	0	0
0	0	0	0	0	0
0	0	0	0,000004084	0	0
0	0	0	0	0	0
10	6,57263E-05	152146	0,00002454	0	0
0	0	0	0	0	0
0	0	0	0	0	0
0	0	0	0	0	0
0	0	0	0,000008274	0	0
0	0	0	0	0	0
0	0	0	0	0	0
0	0	0	0	0	0
12	7,88965E-05	152098	0,00006098	0,00006539	0
0	0	0	0	0	0
2	0,000013143	152172	0,00000549	0	0
0	0	0	0	0	0
0	0	0	0	0	0
4	2,62864E-05	152170	0,00002445	0	0,00005957
3	1,97083E-05	152220	0,00001626	0	0
0	0	0	0	0	0
0	0	0	0	0	0
0	0	0	0	0	0
0	0	0	0,00001374	0,00007783	0
0	0	0	0	0	0
8	5,25845E-05	152136	0,00001341	0	0
0	0	0	0	0	0
1	6,57142E-06	152174	0,000004241	0	0
14	9,26833E-05	151052	0,00001808	0,0002	0
5	3,28753E-05	152090	0,00002909	0,0000665	0
2	1,31385E-05	152224	0,00001686	0	0
0	0	0	0	0	0
30	0,000197166	152156	0,0004	0	0
3	0,000019714	152176	0,00004485	0,0000658	0
3	1,97215E-05	152118	0,00002844	0	0
10	6,57013E-05	152204	0,0003	0	0,00002978
12	8,25048E-05	145446	0,00001932	0,0003	0

7	4,74088E-05	147652	0,000004467	0,00007239	0
7	4,67209E-05	149826	0,000008344	0,0001	0
1	6,58233E-06	151922	0,000004061	0	0
0	0	0	0,000004072	0	0
7	0,000046006	152154	0,00003867	0	0,0001
34	0,000223414	152184	0,00005688	0,0008	0,00002978
0	0	0	0	0	0
9	5,91327E-05	152200	0,00008539	0,00006549	0,00008935
0	0	0	0,000008568	0	0
0	0	0	0	0	0
0	0	0	0	0	0
0	0	0	0	0	0
151	0,00114207	132216	0,0017	0,0000658	0,0009
0	0	0	0	0	0
12	7,88498E-05	152188	0,00009838	0	0
0	0	0	0,000004062	0	0
0	0	0	0	0	0
5	3,28956E-05	151996	0,00004576	0	0
13	8,55207E-05	152010	0,00002819	0,0002	0,00009784
0	0	0	0	0	0
0	0	0	0	0	0
1	6,57298E-06	152138	0	0	0
21	0,000138036	152134	0,0002	0,00007051	0,0001
6	0,000039455	152072	0,00001677	0	0
0	0	0	0,000004063	0	0
0	0	0	0	0	0
0	0	0	0	0	0
5	3,28498E-05	152208	0	0	0
0	0	0	0	0	0
0	0	0	0	0	0
3	1,97179E-05	152146	0,00002843	0,00006534	0,00008935
0	0	0	0,00001275	0	0
0	0	0	0	0	0
0	0	0	0	0	0
0	0	0	0	0	0
0	0	0	0	0	0
0	0	0	0,000007731	0	0
0	0	0	0	0	0
0	0	0	0	0	0
5	3,28403E-05	152252	0,000008723	0	0,00003153
13	8,54398E-05	152154	0,00002451	0	0
3	1,97148E-05	152170	0,00001641	0	0
0	0	0	0	0	0
0	0	0	0	0	0
10	6,56987E-05	152210	0,0000163	0	0
0	0	0	0	0	0
7	4,60012E-05	152170	0,00003722	0	0
0	0	0	0	0	0
0	0	0	0	0	0
0	0	0	0	0	0

0	0	0	0,00002491	0	0
0	0	0	0	0	0
1	6,5672E-06	152272	0	0	0
0	0	0	0	0	0
0	0	0	0	0	0

gnomAD_exome_	gnomAD_exome_	gnomAD_exome_	gnomAD_exome_	gnomAD_exome_	gnomAD_exome_
0	0	0	0	0	0
0	0	0	0	0	0
0	0	0	0	0	0,00006044
0	0	0	0	0	0
0	0	0	0	0	0
0	0	0	0	0	0
0	0	0	0	0	0
0	0	0	0	0	0
0	0	0	0	0	0
0	0	0	0	0	0
0	0	0	0	0	0
0	0	0	0	0	0
0,0004	0,0002	0	0,00008077	0	0
0	0,00005808	0	0,000009081	0	0
0	0	0	0	0	0,00006665
0	0	0	0	0	0
0	0	0	0	0	0
0	0,00005798	0	0,0001	0,0002	0,0016
0	0	0	0	0	0
0	0	0	0,000008963	0	0
0	0	0,0001	0,00001797	0	0
0	0	0	0,000009048	0	0
0	0	0	0	0	0
0	0	0	0	0	0
0	0	0	0	0	0
0	0	0	0	0	0
0	0,0008	0	0,0004	0,0002	0,0003
0	0	0	0	0	0
0	0	0	0	0	0
0	0	0	0	0	0
0	0	0	0	0	0
0	0	0	0	0	0
0	0	0	0,000008984	0	0
0	0	0	0	0	0
0	0	0	0,00006243	0	0
0	0	0	0	0	0
0	0	0	0	0	0
0	0	0	0	0	0
0	0	0	0	0	0
0	0	0	0	0	0
0	0	0	0	0	0
0	0,0017	0	0	0	0
0	0	0	0	0	0
0	0	0	0,00001796	0	0,00003249
0	0	0	0,0000179	0	0
0	0,000058	0	0,00001792	0	0

0	0	0	0	0	0
0	0	0	0,0002	0	0,00003249
0	0	0	0	0	0
0	0	0	0,000008964	0	0
0	0	0	0	0	0
0	0	0,00006616	0	0	0
0	0	0	0	0	0
0	0	0	0	0	0
0	0	0	0,000009065	0	0
0	0	0	0	0	0
0	0	0	0,000008972	0	0,0000325
0	0	0	0	0	0
0	0	0	0	0	0
0	0	0	0,00002688	0	0,0003
0	0	0	0	0	0
0	0	0	0	0	0
0	0,0001	0	0,00006284	0,0002	0,0002
0	0	0	0	0	0
0	0	0	0	0	0
0	0	0	0	0	0
0	0	0	0	0	0
0	0	0	0	0	0
0	0	0	0	0	0
0	0	0	0	0	0
0	0	0	0	0	0
0	0	0	0	0	0
0	0	0	0	0	0
0	0	0	0	0	0
0	0,00005813	0	0,00002699	0	0
0	0	0	0	0	0
0	0	0	0	0	0
0	0	0	0	0	0
0	0	0	0	0	0
0	0	0	0	0	0
0	0	0	0	0	0
0	0	0	0	0	0
0	0	0	0	0	0
0	0	0	0,00002686	0	0,00003249
0	0	0,00008982	0,00009869	0	0,00006499
0	0	0	0	0	0
0	0	0	0	0	0
0	0	0	0	0	0
0	0	0	0	0	0
0	0	0	0,00000903	0,0002	0
0	0	0	0	0	0
0	0	0	0	0	0
0	0	0	0,000008958	0	0,00003249
0	0	0	0,000008964	0	0,00006497
0	0	0	0	0	0
0	0	0	0,00002296	0	0

0	0	0	0	0	0
0	0	0	0	0	0
0	0	0	0	0	0
0	0	0	0,00001826	0	0,0049
0	0	0	0	0	0
0	0	0	0	0	0
0	0	0	0	0	0
0	0	0	0	0	0
0	0	0	0	0	0
0	0	0	0	0	0
0	0	0	0	0	0
0	0	0	0	0	0
0	0	0	0	0	0
0	0	0	0	0	0
0	0	0	0	0	0
0	0	0	0	0	0
0	0	0	0	0	0
0	0	0	0,0003	0,0002	0,0002
0	0	0	0	0	0
0	0	0	0	0	0
0	0	0	0	0	0
0	0	0	0	0	0
0	0	0	0,00001837	0	0
0	0	0	0,00003582	0	0
0	0	0	0	0	0
0,0001	0,00005802	0	0,0003	0,0002	0,0001
0	0	0	0	0	0
0	0	0	0	0	0
0	0,0009	0	0	0	0
0	0,000058	0	0,00002697	0	0
0	0	0	0	0	0
0	0	0	0,000008951	0	0,00003249
0	0	0	0	0	0
0	0	0	0	0	0
0	0	0	0	0	0
0	0	0,0000897	0,000008974	0	0,0000975
0	0	0	0	0	0
0	0	0	0	0	0
0	0	0	0	0	0
0	0,0001	0	0	0	0
0	0	0	0,000008963	0	0
0,0005	0	0	0,000008995	0,0002	0
0	0,00005798	0	0,00008067	0	0,0002
0	0	0	0,000008958	0	0
0	0	0	0	0	0,0000439
0	0	0	0	0	0
0	0	0	0	0	0
0	0	0,00004484	0,00005371	0	0
0,0001	0,0001	0	0,000008952	0	0
0,0015	0	0	0	0,0005	0,0056

0	0	0	0	0	0
0	0	0	0	0	0
0,0001	0	0	0,00002685	0	0
0	0	0	0	0	0
0	0	0	0	0	0
0	0	0	0,000009242	0,0002	0
0	0	0	0	0	0
0	0	0	0,00003592	0	0,00006502
0	0	0	0	0	0
0	0	0	0	0	0
0	0	0	0	0	0
0	0	0	0	0	0
0	0,00005799	0	0	0	0
0	0	0	0	0	0
0	0	0	0	0	0
0	0	0	0	0	0
0	0	0	0	0	0
0	0	0	0	0	0
0	0	0	0	0	0
0	0	0	0	0	0
0	0	0	0	0	0
0	0	0	0	0	0
0	0	0	0	0	0
0	0	0	0	0	0
0	0	0	0	0	0
0	0	0	0	0	0
0	0	0	0	0	0
0	0	0	0	0	0
0	0	0	0,00004584	0	0,00006499
0	0	0	0	0	0
0	0	0	0	0	0
0	0	0	0	0	0
0	0	0	0	0	0
0	0	0	0	0	0
0	0	0	0	0	0
0	0	0	0	0	0
0	0	0	0	0	0
0	0	0	0	0	0
0	0,00005817	0	0	0	0
0	0	0	0	0	0
0	0	0	0,0002	0,0002	0
0	0	0	0,00002079	0	0,00006856
0	0	0	0,000008957	0	0
0	0,0012	0	0,00001799	0	0,00003258
0	0	0	0	0	0
0	0	0	0	0	0,00009746
0	0	0	0	0	0
0	0	0	0,000008984	0,0002	0,0003
0	0	0	0	0	0
0	0	0	0,00001261	0	0

0	0	0,00004549	0,00008105	0	0
0	0,00005799	0	0,00001791	0	0,00003249
0	0	0	0	0	0
0	0	0	0	0	0
0	0	0	0	0	0
0	0	0	0	0	0
0	0	0	0	0	0
0	0	0	0	0	0
0	0	0	0	0	0
0	0	0	0,0000359	0	0,0000975
0	0	0	0	0	0
0	0	0	0	0	0
0	0,0001	0,0002	0,00001007	0,0002	0
0	0	0	0	0	0
0	0	0	0	0	0
0	0	0	0	0	0
0	0,0002	0	0,00009852	0,0005	0,0002
0	0	0	0	0	0
0	0	0	0	0	0
0	0	0	0	0	0
0	0	0	0	0	0
0	0	0,0000449	0	0,0004	0
0	0	0	0	0	0
0	0	0	0	0	0
0	0	0	0	0	0
0	0	0	0	0	0
0	0	0	0,000009308	0	0
0	0	0	0,00001803	0,0002	0,0001
0	0,0006	0	0,00001792	0	0
0	0	0	0	0	0,000065
0	0	0	0	0	0
0	0	0	0	0	0,00006497
0	0	0	0,0000279	0	0
0	0	0	0	0	0
0	0	0	0	0	0
0	0,00005801	0	0,000008966	0	0,00003249
0	0	0	0	0	0
0	0	0	0	0	0
0	0	0	0	0	0
0	0	0	0	0	0
0	0	0	0	0	0
0	0	0	0	0	0
0	0	0	0	0	0
0	0	0	0	0	0
0	0	0	0	0	0
0	0	0	0	0	0
0	0	0	0	0	0
0	0	0	0	0	0
0	0	0	0	0	0
0	0	0	0	0,0002	0

0	0	0	0	0	0
0	0,0000769	0	0,00001251	0,0002	0,0004
0	0	0	0	0	0
0	0	0	0	0	0
0	0	0	0	0	0
0	0	0	0	0	0
0	0,00005852	0	0,00000905	0	0
0	0	0	0,00002696	0	0
0	0	0	0	0	0
0	0	0	0	0	0
0	0	0	0	0	0
0	0	0	0	0	0
0	0	0	0	0	0
0	0	0	0	0	0
0	0	0	0	0	0,00003249
0	0	0	0	0	0
0	0	0	0,000008977	0	0
0	0	0	0	0	0
0	0,0002	0	0,00008969	0	0,00006497
0	0	0	0	0	0
0	0	0	0,000008951	0,0002	0,00006497
0	0	0	0	0	0
0	0	0	0	0	0
0	0	0	0	0	0
0	0	0	0,00001791	0,0002	0,00003256
0	0	0	0	0	0
0	0	0	0,000009006	0	0
0	0	0	0	0	0
0	0	0,000045	0,00005395	0	0,00009748
0	0	0	0	0	0
0	0	0	0	0	0
0	0	0	0	0	0
0	0	0	0	0	0
0	0	0	0	0	0
0	0	0	0	0	0
0	0,00006043	0	0,000009208	0	0
0	0	0,00004511	0	0	0
0	0	0	0	0	0
0	0	0	0	0	0
0	0	0	0	0	0,00009818
0	0	0	0,00002686	0	0,00003249
0	0	0	0	0,0002	0
0	0	0	0	0	0
0	0	0	0	0	0
0	0,0000777	0	0	0,0002	0,00008464
0	0,00005798	0	0,000008962	0	0
0	0	0	0	0	0
0	0	0	0	0	0
0	0	0	0,000008958	0	0
0,0001	0	0	0	0	0

0	0	0	0,000008958	0	0
0	0	0	0,00000896	0	0
0	0	0	0	0	0
0	0	0	0,00001815	0	0
0	0	0	0	0	0
0	0	0	0	0	0,0000325
0	0	0	0	0	0
0	0	0	0	0	0
0	0	0	0	0	0
0	0	0	0,00001811	0	0
0	0	0	0	0	0
0	0	0	0	0	0
0	0	0	0	0	0,00004693
0	0	0	0	0	0
0	0	0	0	0	0
0	0	0	0	0	0
0	0	0	0	0	0
0	0	0	0,00001791	0	0,0003
0	0	0	0	0	0
0	0	0	0,000009031	0	0
0	0	0	0	0	0
0	0	0	0	0	0
0,0001	0	0	0,00005106	0	0,0002
0	0	0	0	0	0
0	0	0	0	0	0
0	0	0	0,00001817	0	0
0	0	0	0,00009946	0	0
0	0	0	0	0	0
0	0	0	0	0	0,00003249
0	0	0	0	0	0
0	0	0	0	0	0
0	0	0	0	0	0
0	0	0	0	0	0
0	0	0	0	0	0
0	0	0	0,0001	0	0
0	0	0	0	0	0
0	0	0	0,0009	0	0,00006898
0	0	0	0	0	0
0	0	0	0	0	0
0	0	0	0,000008966	0	0,00006497
0	0	0	0	0	0
0	0	0	0	0	0
0	0	0	0	0	0
0	0	0	0	0	0
0	0	0	0	0	0
0	0	0	0	0	0
0	0	0	0	0	0
0	0	0	0	0	0
0	0	0	0	0	0
0	0	0	0	0	0

0	0	0	0	0	0
0	0	0	0,00003585	0	0
0	0	0	0	0	0
0	0	0	0	0	0,00003249
0	0	0	0	0	0
0	0	0	0	0	0
0	0	0	0	0	0
0	0,00005806	0,00004533	0,00002694	0	0
0	0	0,0006	0,00001796	0	0,0002
0	0	0	0	0	0
0	0	0	0,00001458	0	0
0	0	0	0	0	0
0,0001	0	0	0	0	0
0	0	0	0	0	0
0	0	0	0	0	0
0	0	0	0	0	0
0	0	0	0,000009029	0	0
0	0	0	0	0	0
0	0	0,0001	0,00001797	0	0,00003323
0	0	0	0	0	0
0	0	0	0	0	0
0	0	0	0	0	0
0	0	0	0,000009151	0,0002	0
0	0	0	0	0	0
0	0	0	0	0	0
0	0	0	0	0	0
0,0001	0,000058	0	0,00007174	0,0002	0,00009746
0	0	0	0	0	0
0	0	0	0,00001297	0	0
0	0	0	0	0	0
0	0	0	0	0	0
0	0,0002	0	0,000008996	0	0
0	0,00005798	0	0,00001792	0	0,0000325
0	0	0	0	0	0
0	0	0	0	0	0
0	0	0	0	0	0
0	0	0	0,00001042	0	0,0000357
0	0	0	0	0	0
0	0,00009502	0	0,00001734	0	0
0	0	0	0	0	0
0	0	0	0,000009356	0	0
0	0	0	0,00001028	0	0
0,0001	0,0000583	0	0,00001853	0	0,00006509
0	0,00005916	0	0	0	0,00009954
0	0	0	0	0	0
0	0	0	0,000008997	0	0,0032
0	0,0002	0	0,00005407	0	0,00003249
0	0	0	0,00005375	0	0,00003249
0	0	0	0	0,0002	0,0023
0	0	0	0	0	0

0	0	0	0	0	0
0	0	0	0	0	0
0	0	0	0,000008952	0	0
0	0	0	0,000008986	0	0
0	0	0	0,00001993	0	0,00004444
0	0	0	0	0	0
0	0	0	0	0	0
0	0,000058	0	0,0001	0,0002	0
0	0	0	0,00001923	0	0
0	0	0	0	0	0
0	0	0	0	0	0
0	0	0	0	0	0
0,0021	0	0,0074	0,0016	0,0022	0,0009
0	0	0	0	0	0
0	0	0	0,00003632	0	0,0007
0	0	0	0	0	0,00003249
0	0	0	0	0	0
0	0,0001	0,00007398	0,00006903	0	0
0	0	0	0	0	0
0	0	0	0	0	0
0	0	0	0	0	0
0	0	0	0	0	0
0	0	0	0,0002	0	0,0011
0	0	0	0,00002802	0	0,00003258
0	0	0	0,00000896	0	0
0	0	0	0	0	0
0	0	0	0	0	0
0	0	0	0	0	0
0	0	0	0	0	0
0	0	0	0	0	0
0	0	0,00004485	0,00001791	0	0
0	0	0	0,0000188	0	0,00003369
0	0	0	0	0	0
0	0	0	0	0	0
0	0	0	0	0	0
0	0	0	0	0	0
0	0	0	0,00001895	0	0
0	0	0	0	0	0
0	0	0	0	0	0
0	0	0	0	0	0,00003561
0	0,00005812	0	0,00004516	0	0
0	0	0	0,00003662	0	0
0	0	0	0	0	0
0	0	0	0	0	0
0	0	0	0,00002698	0	0,00003258
0	0	0	0	0	0
0,0002	0,0001	0,00004498	0,00003613	0	0
0	0	0	0	0	0
0	0	0	0	0	0
0	0	0	0	0	0

0	0	0	0	0	0,0002
0	0	0	0	0	0
0	0	0	0	0	0
0	0	0	0	0	0
0	0	0	0	0	0

gnomAD_genome_	gnomAD_genome_	gnomAD_genome_	gnomAD_genome_	gnomAD_genome_	gnomAD_genome_
0	0	0	0	0	0
0	0	0	0	0	0
0	0	0	0	0	0
0	0	0	0	0	0
0	0	0	0	0	0
0	0	0	0	0	0
0	0	0	0	0	0
0	0	0	0	0	0
0	0	0	0	0	0
0	0	0	0	0	0
0	0	0	0	0	0
0	0	0	0	0	0
0,00003229	0	0	0	0	0
0,00003228	0	0	0	0	0
0	0	0	0	0	0
0	0	0	0	0	0
0	0	0	0	0	0
0	0	0	0	0	0
0	0	0	0	0	0
0	0	0	0	0	0
0	0	0	0	0	0
0	0	0	0	0	0
0	0	0	0	0	0
0	0	0	0	0	0
0	0	0	0	0	0
0	0	0	0	0	0
0,0002	0,0006	0	0	0	0
0	0	0	0	0	0
0	0	0	0	0	0
0	0	0	0	0	0
0	0	0	0	0	0
0	0	0	0	0	0
0	0	0	0	0	0
0	0	0	0	0	0
0	0	0	0	0	0
0	0	0	0	0	0
0	0	0	0	0	0
0	0	0	0	0	0
0	0	0	0	0	0
0	0	0	0	0	0
0	0	0	0	0	0
0,00009687	0	0	0	0,0018	0
0	0	0	0	0	0
0	0	0	0	0	0
0	0	0	0	0	0
0,00003228	0	0	0	0	0

0	0	0	0	0	0
0,00003237	0	0	0	0	0
0	0	0	0	0	0
0	0	0	0	0	0
0	0	0	0	0	0
0,00003231	0	0	0	0	0
0	0	0	0	0	0
0	0	0	0	0	0
0,00003238	0	0	0	0	0
0	0	0	0	0	0
0	0	0	0	0	0
0	0	0	0	0	0
0	0	0	0	0	0
0,00006459	0	0	0	0	0
0	0	0	0	0	0
0	0	0	0	0	0
0,0005	0,0016	0,0012	0	0	0
0	0	0	0	0	0
0	0	0	0	0	0
0	0	0	0	0	0
0	0	0	0	0	0
0	0	0	0	0	0
0	0	0	0	0	0
0	0	0	0	0	0
0	0	0	0	0	0
0	0	0	0	0	0
0	0	0	0	0	0
0	0	0	0	0	0
0,00003227	0,0001	0	0	0	0
0	0	0	0	0	0
0	0	0	0	0	0
0	0	0	0	0	0
0	0	0	0	0	0
0	0	0	0	0	0
0	0	0	0	0	0
0	0	0	0	0	0
0	0	0	0	0	0
0,0000323	0,0001	0	0	0	0
0,00006458	0	0	0	0	0,0003
0	0	0	0	0	0
0	0	0	0	0	0
0	0	0	0	0	0
0	0	0	0	0	0
0	0	0	0	0	0
0	0	0	0	0	0
0	0	0	0	0	0
0	0	0	0	0	0
0	0	0	0	0	0

0	0	0	0	0	0
0	0	0	0	0	0
0	0	0	0	0	0
0,00003233	0	0	0	0	0
0	0	0	0	0	0
0	0	0	0	0	0
0	0	0	0	0	0
0	0	0	0	0	0
0	0	0	0	0	0
0	0	0	0	0	0
0	0	0	0	0	0
0	0	0	0	0	0
0	0	0	0	0	0
0	0	0	0	0	0
0	0	0	0	0	0
0	0	0	0	0	0
0	0	0	0	0	0
0,0005	0,0008	0,0012	0	0	0
0	0	0	0	0	0
0	0	0	0	0	0
0	0	0	0	0	0
0	0	0	0	0	0
0,00009073	0,0003	0	0	0	0
0,00003231	0	0	0	0	0
0	0	0	0	0	0
0,00006465	0,0001	0	0	0	0
0	0	0	0	0	0
0	0	0	0	0	0
0,00009684	0	0	0	0,0012	0
0	0	0	0	0	0
0	0	0	0	0	0
0	0	0	0	0	0
0	0	0	0	0	0
0	0	0	0	0	0
0	0	0	0	0	0
0,00003228	0	0	0	0	0
0	0	0	0	0	0
0	0	0	0	0	0
0	0	0	0	0	0
0	0	0	0	0	0
0	0	0	0	0	0
0	0	0	0	0	0
0,0000646	0,0002	0	0	0	0
0	0	0	0	0	0
0	0	0	0	0	0
0	0	0	0	0	0
0	0	0	0	0	0
0,00003228	0,0001	0	0	0	0
0,0001	0,0005	0	0	0	0
0,00009685	0,0001	0	0,0033	0	0

0	0	0	0	0	0
0	0	0	0	0	0
0	0	0	0	0	0
0	0	0	0	0	0
0	0	0	0	0	0
0	0	0	0	0	0
0	0	0	0	0	0
0	0	0	0	0	0
0	0	0	0	0	0
0	0	0	0	0	0
0	0	0	0	0	0
0	0	0	0	0	0
0,0000323	0	0,0012	0	0	0
0	0	0	0	0	0
0	0	0	0	0	0
0	0	0	0	0	0
0	0	0	0	0	0
0	0	0	0	0	0
0	0	0	0	0	0
0	0	0	0	0	0
0	0	0	0	0	0
0	0	0	0	0	0
0	0	0	0	0	0
0	0	0	0	0	0
0	0	0	0	0	0
0	0	0	0	0	0
0	0	0	0	0	0
0	0	0	0	0	0
0,00006462	0,0002	0	0	0	0
0	0	0	0	0	0
0	0	0	0	0	0
0	0	0	0	0	0
0	0	0	0	0	0
0	0	0	0	0	0
0	0	0	0	0	0
0	0	0	0	0	0
0	0	0	0	0	0
0	0	0	0	0	0
0	0	0	0	0	0
0	0	0	0	0	0
0,0001	0,0002	0	0	0	0
0	0	0	0	0	0
0,00006466	0,0002	0	0	0	0
0,00009702	0	0	0	0,0019	0
0	0	0	0	0	0
0	0	0	0	0	0
0	0	0	0	0	0
0	0	0	0	0	0
0	0	0	0	0	0
0	0	0	0	0	0

0	0	0	0	0	0
0	0	0	0	0	0
0	0	0	0	0	0
0	0	0	0	0	0
0	0	0	0	0	0
0	0	0	0	0	0
0	0	0	0	0	0
0	0	0	0	0	0
0	0	0	0	0	0
0,00006457	0	0	0	0	0
0	0	0	0	0	0
0	0	0	0	0	0
0,00003239	0	0	0	0	0,0003
0	0	0	0	0	0
0	0	0	0	0	0
0	0	0	0	0	0
0,0002	0	0,0012	0	0,0006	0
0	0	0	0	0	0
0	0	0	0	0	0
0	0	0	0	0	0
0	0	0	0	0	0
0,00009698	0,0001	0	0	0	0
0	0	0	0	0	0
0	0	0	0	0	0
0,00003255	0,0001	0	0	0	0
0	0	0	0	0	0
0	0	0	0	0	0
0,00006458	0,0002	0	0	0	0
0,00003249	0	0	0	0,0006	0
0,00003231	0	0	0,0033	0	0
0	0	0	0	0	0
0	0	0	0	0	0
0	0	0	0	0	0
0	0	0	0	0	0
0	0	0	0	0	0
0,0000323	0,0001	0	0	0	0
0	0	0	0	0	0
0	0	0	0	0	0
0	0	0	0	0	0
0	0	0	0	0	0
0	0	0	0	0	0
0	0	0	0	0	0
0	0	0	0	0	0
0	0	0	0	0	0
0	0	0	0	0	0
0	0	0	0	0	0
0	0	0	0	0	0
0	0	0	0	0	0
0	0	0	0	0	0
0	0	0	0	0	0

0	0	0	0	0	0
0	0	0	0	0	0
0	0	0	0	0	0
0,0012	0,0045	0	0	0	0
0	0	0	0	0	0
0	0	0	0	0	0
0	0	0	0	0	0
0	0	0	0	0	0
0	0	0	0	0	0
0	0	0	0	0	0
0	0	0	0	0	0
0	0	0	0	0	0
0	0	0	0	0	0
0	0	0	0	0	0
0	0	0	0	0	0
0	0	0	0	0	0
0	0	0	0	0	0
0	0	0	0	0	0
0,00009706	0	0	0	0	0
0	0	0	0	0	0
0	0	0	0	0	0
0	0	0	0	0	0
0	0	0	0	0	0
0,00003227	0	0	0	0	0
0,0000323	0	0,0012	0	0	0
0,00003228	0	0	0	0	0
0	0	0	0	0	0
0	0	0	0	0	0
0,0002	0,0003	0	0	0	0
0	0	0	0	0	0
0	0	0	0	0	0
0	0	0	0	0	0
0	0	0	0	0	0
0	0	0	0	0	0
0	0	0	0	0	0
0	0	0	0	0	0
0	0	0	0	0	0
0	0	0	0	0	0
0	0	0	0	0	0
0	0	0	0	0	0
0	0	0	0	0	0
0	0	0	0	0	0
0	0	0	0	0	0
0	0	0	0	0	0
0	0	0	0	0	0
0	0	0	0	0	0
0	0	0	0	0	0
0	0	0	0	0	0
0	0	0	0	0	0
0	0	0	0	0	0

0	0	0	0	0	0
0	0	0	0	0	0
0	0	0	0	0	0
0,00003234	0	0	0	0	0
0	0	0	0	0	0
0	0	0	0	0	0
0	0	0	0	0	0
0	0	0	0	0	0
0	0	0	0	0	0
0	0	0	0	0	0
0	0	0	0	0	0
0	0	0	0	0	0
0	0	0	0	0	0
0	0	0	0	0	0
0	0	0	0	0	0
0	0	0	0	0	0
0	0	0	0	0	0
0	0	0	0	0	0
0	0	0	0	0	0
0,0002	0,0007	0	0	0	0
0	0	0	0	0	0
0	0	0	0	0	0
0,00003838	0	0,0013	0	0	0
0	0	0	0	0	0
0	0	0	0	0	0
0	0	0	0	0	0
0,00009709	0,0002	0	0	0	0
0	0	0	0	0	0
0	0	0	0	0	0
0	0	0	0	0	0
0	0	0	0	0	0
0	0	0	0	0	0
0	0	0	0	0	0
0	0	0	0	0	0
0,00003227	0,0001	0	0	0	0
0	0	0	0	0	0
0,0002	0,0001	0,0012	0	0	0
0	0	0	0	0	0
0	0	0	0	0	0
0	0	0	0	0	0
0	0	0	0	0	0
0	0	0	0	0	0
0	0	0	0	0	0
0	0	0	0	0	0
0	0	0	0	0	0
0	0	0	0	0	0
0	0	0	0	0	0
0	0	0	0	0	0
0	0	0	0	0	0
0	0	0	0	0	0

0	0	0	0	0	0
0,0000323	0	0	0	0	0
0	0	0	0	0	0
0	0	0	0	0	0
0	0	0	0	0	0
0	0	0	0	0	0
0	0	0	0	0	0
0	0	0	0	0	0
0,0002	0,0001	0	0	0	0,0009
0	0	0	0	0	0
0	0	0	0	0	0
0	0	0	0	0	0
0	0	0	0	0	0
0	0	0	0	0	0
0	0	0	0	0	0
0	0	0	0	0	0
0	0	0	0	0	0
0	0	0	0	0	0
0,00006457	0	0	0	0	0,0003
0	0	0	0	0	0
0	0	0	0	0	0
0	0	0	0	0	0
0	0	0	0	0	0
0	0	0	0	0	0
0	0	0	0	0	0
0	0	0	0	0	0
0,00006475	0	0	0	0	0
0	0	0	0	0	0
0,00003233	0	0	0	0	0
0	0	0	0	0	0
0	0	0	0	0	0
0	0	0	0	0	0
0,00003229	0,0001	0	0	0	0
0	0	0	0	0	0
0	0	0	0	0	0
0	0	0	0	0	0
0	0	0	0	0	0
0	0	0	0	0	0
0,00006477	0,0002	0	0	0	0
0	0	0	0	0	0
0	0	0	0	0	0
0,00003255	0,0001	0	0	0	0
0	0	0	0	0	0
0	0	0	0	0	0
0	0	0	0	0	0
0,0000323	0	0	0	0	0
0	0	0	0	0	0
0	0	0	0	0	0
0	0	0	0	0	0
0,00006828	0,0002	0	0	0	0

0,00003299	0,0001	0	0	0	0
0,00003257	0,0001	0	0	0	0
0	0	0	0	0	0
0	0	0	0	0	0
0,00003233	0	0	0	0	0
0,0002	0,0006	0	0	0	0
0	0	0	0	0	0
0,00006465	0	0	0	0	0
0	0	0	0	0	0
0	0	0	0	0	0
0	0	0	0	0	0
0	0	0	0	0	0
0,0025	0,0013	0	0,0035	0	0,0009
0	0	0	0	0	0
0,00006458	0,0001	0	0	0	0
0	0	0	0	0	0
0	0	0	0	0	0
0	0	0	0	0	0
0,00009713	0,0001	0	0	0	0
0	0	0	0	0	0
0	0	0	0	0	0
0	0	0	0	0	0
0	0	0	0	0	0
0	0	0	0	0	0
0	0	0	0	0	0
0	0	0	0	0	0
0	0	0	0	0	0
0,00003228	0,0001	0	0	0	0
0	0	0	0	0	0
0	0	0	0	0	0
0,00003235	0	0	0	0	0
0	0	0	0	0	0
0	0	0	0	0	0
0	0	0	0	0	0
0	0	0	0	0	0
0	0	0	0	0	0
0	0	0	0	0	0
0	0	0	0	0	0
0	0	0	0	0	0
0	0	0	0	0	0
0	0	0	0	0	0
0	0	0	0	0	0
0	0	0	0	0	0
0	0	0	0	0	0
0,00009699	0,0002	0	0	0	0
0	0	0	0	0	0
0,00003232	0,0001	0	0	0	0
0	0	0	0	0	0
0	0	0	0	0	0
0	0	0	0	0	0

0	0	0	0	0	0
0	0	0	0	0	0
0	0	0	0	0	0
0	0	0	0	0	0
0	0	0	0	0	0

gnomAD_genome_	gnomAD_genome_	1000g2	CADD	CADD	CADD	Splice	Splice	Splice	PossibleDeNovo
0	0	0	0	0	0	AGRN	NA	NA	hiConfDeNovo
0	0	0	0	0	0	AGRN	NA	NA	hiConfDeNovo
0	0	0	1,557	0,228	16,1	TNFRS	NA	NA	hiConfDeNovo
0	0	0	3,953	0,77	26,7	CDK1	NA	NA	hiConfDeNovo
0	0	0	0,499	0,091	6,491	FAAP2	Accept	BP7	hiConfDeNovo
0	0	0	9,452	0,999	50	SKI|0.0	Donor	PP3	hiConfDeNovo
0	0	0	1,948	0,296	18,86	NPHP4	Donor	BP7	hiConfDeNovo
0	0	0	3,088	0,54	23,6	CASZ1	NA	NA	hiConfDeNovo
0	0	0	2,623	0,439	22,7	MAD2	Donor	PP3	hiConfDeNovo
0	0	0	2,934	0,506	23,3	FAM4	NA	NA	hiConfDeNovo
0	0	0	1,809	0,27	17,8	MAP3	Accept	BP7	hiConfDeNovo
0	0	0	0	0	0	GMEB	NA	NA	hiConfDeNovo
0,00006664	0	0	3,381	0,611	24,3	TMEM	NA	NA	hiConfDeNovo
0,00006665	0	0	3,462	0,631	24,6	MANE	NA	NA	hiConfDeNovo
0	0	0	4,125	0,83	28	INPP5	Donor	BP7	hiConfDeNovo
0	0	0	0	0	0	MACF	NA	NA	hiConfDeNovo
0	0	0	3,59	0,664	25	HPCA	Accept	BP7	hiConfDeNovo
0	0	2E-04	0	0	0	PLK3|0	Donor	BP7	hiConfDeNovo
0	0	0	4,141	0,835	28,1	HECT	NA	NA	hiConfDeNovo
0	0	0	2,653	0,446	22,7	SGIP1|	NA	NA	hiConfDeNovo
0	0	0	2,35	0,379	21,9	ANKR	Accept	BP7	hiConfDeNovo
0	0	0	6,98	0,97	37	ERICH	Accept	PP3	hiConfDeNovo
0	0	0	5,88	0,955	35	RABG	Accept	PP3	hiConfDeNovo
0	0	0	1,427	0,208	15,26	ST6GA	NA	NA	hiConfDeNovo
0	0	0	0	0	0	ALG14	NA	NA	hiConfDeNovo
0	0	0	0	0	0	NA	NA	NA	hiConfDeNovo
0,00006665	0	0	2,937	0,507	23,3	GPSM	Accept	BP7	hiConfDeNovo
0	0	0	4,231	0,864	29	MAGI	NA	NA	hiConfDeNovo
0	0	0	0	0	0	BCL2L	NA	NA	hiConfDeNovo
0	0	0	2,269	0,362	21,4	PTGFR	NA	NA	hiConfDeNovo
0	0	0	2,726	0,462	22,9	GJA5|0	Accept	BP7	hiConfDeNovo
0	0	0	1,904	0,287	18,52	OTUD	Donor	BP7	hiConfDeNovo
0	0	0	2,287	0,366	21,5	PI4KB	NA	NA	hiConfDeNovo
0	0	0	-0,18	0,03	0,579	FLG2|0	NA	NA	hiConfDeNovo
0	0	0	0	0	0	NA	NA	NA	hiConfDeNovo
0	0	0	0	0	0	NTRK	Accept	PP3	hiConfDeNovo
0	0	0	0	0	0	OR6N1	Accept	BP7	hiConfDeNovo
0	0	0	4,11	0,824	27,8	RFWD	NA	NA	hiConfDeNovo
0	0	0	-0,14	0,032	0,676	RGSL1	Accept	BP7	hiConfDeNovo
0	0	0	3,27	0,583	24	GPR25	NA	NA	hiConfDeNovo
0	0	0	0,312	0,071	4,429	IGFN1	NA	NA	hiConfDeNovo
0	0	0	0	0	0	PPP1R	NA	NA	hiConfDeNovo
0	0	0	4,02	0,793	27,1	KCNH	Accept	BP7	hiConfDeNovo
0	0	0	0	0	0	RAB3	Donor	BP7	hiConfDeNovo
0	0	0	4,258	0,872	29,2	LBR|0.	NA	NA	hiConfDeNovo
0,00006662	0	0	3,924	0,761	26,5	SIPA1	NA	NA	hiConfDeNovo
0	0	0	4,348	0,892	30	MTR|0	NA	NA	hiConfDeNovo
0	0	0	3,913	0,757	26,4	RYR2|	Accept	BP7	hiConfDeNovo

0	0	0	2,608	0,436	22,7	KIF26	NA	NA	hiConfDeNovo
0	0,001	0	4,329	0,889	29,8	ITGB1	Accept	VUS	hiConfDeNovo
0	0	0	3,807	0,724	25,9	ODC1|	NA	NA	hiConfDeNovo
0	0	0	0	0	0	PUM2|	Accept	BP7	hiConfDeNovo
0	0	0	2,881	0,495	23,2	ITSN2|	NA	NA	hiConfDeNovo
0,00006672	0	0	4,178	0,847	28,4	DNMT	Donor	BP7	hiConfDeNovo
0	0	0	2,914	0,502	23,2	ABCG	Accept	BP7	hiConfDeNovo
0	0	0	0	0	0	ABCG	Accept	BP7	hiConfDeNovo
0,00006686	0	0	0,119	0,052	2,265	CALM	NA	NA	hiConfDeNovo
0	0	0	0	0	0	VPS54	Accept	BP7	hiConfDeNovo
0	0	0	3,707	0,696	25,4	ACTR	NA	NA	hiConfDeNovo
0	0	0	0	0	0	AAK1|	NA	NA	hiConfDeNovo
0	0	0	3,794	0,72	25,8	AC007	NA	NA	hiConfDeNovo
0,0001	0	2E-04	3,438	0,625	24,5	DYSF|	NA	NA	hiConfDeNovo
0	0	0	1,352	0,197	14,75	C2orf7	NA	NA	hiConfDeNovo
0	0	0	0	0	0	MOGS	NA	NA	hiConfDeNovo
0	0	1E-03	0	0	0	FAHD	NA	NA	hiConfDeNovo
0	0	0	4,6	0,919	32	SNRN	NA	NA	hiConfDeNovo
0	0	0	0	0	0	IL18R1	Donor	PP3	hiConfDeNovo
0	0	0	0	0	0	NPHP1	Accept	BP7	hiConfDeNovo
0	0	0	2,331	0,375	21,7	NIFK|0	NA	NA	hiConfDeNovo
0	0	0	7,141	0,973	37	IWS1|0	NA	NA	hiConfDeNovo
0	0	0	0	0	0	SAP13	NA	NA	hiConfDeNovo
0	0	0	4,318	0,886	29,7	SCN2A	Accept	BP7	hiConfDeNovo
0	0	0	3,493	0,639	24,7	SCN1A	NA	NA	hiConfDeNovo
0	0	0	0	0	0	NA	NA	NA	hiConfDeNovo
0	0	0	1,938	0,294	18,78	PDK1|	NA	NA	hiConfDeNovo
0	0	0	0	0	0	TTN|0.	Donor	BP7	hiConfDeNovo
0	0	2E-04	0,248	0,064	3,679	CCDC	Accept	BP7	hiConfDeNovo
0	0	0	1,647	0,242	16,68	PLCL1	NA	NA	hiConfDeNovo
0	0	0	6,573	0,965	36	SATB2	NA	NA	hiConfDeNovo
0	0	0	0	0	0	SPEG|	Donor	BP7	hiConfDeNovo
0	0	0	7,45	0,978	38	CUL3|	Accept	BP7	hiConfDeNovo
0	0	0	8,694	0,995	44	SPHK	NA	NA	hiConfDeNovo
0	0	0	0	0	0	ARMC	NA	NA	hiConfDeNovo
0	0	0	4,363	0,895	31	NCL|0.	Accept	BP7	hiConfDeNovo
0	0	0	0	0	0	TRPM	NA	NA	hiConfDeNovo
0	0	0	0	0	0	OR6B3	Accept	BP7	hiConfDeNovo
0	0,001	0	0	0	0	HDLB	NA	NA	hiConfDeNovo
0	0	0	0	0	0	SEPT2	NA	NA	hiConfDeNovo
0	0	0	1,789	0,266	17,65	THUM	NA	NA	hiConfDeNovo
0	0	0	0	0	0	FANC	NA	NA	hiConfDeNovo
0	0	0	3,887	0,749	26,3	SLC6A	Accept	BP7	hiConfDeNovo
0	0	0	4,278	0,877	29,4	SYN2|	NA	NA	hiConfDeNovo
0	0	0	3,353	0,604	24,3	GRIP2	Donor	BP7	hiConfDeNovo
0	0	0	0	0	0	UBE2E	NA	NA	hiConfDeNovo
0	0	0	0,835	0,13	9,749	VIPR1	Accept	BP7	hiConfDeNovo
0	0	0	4,077	0,813	27,6	KLHL	NA	NA	hiConfDeNovo
0	0	0	1,317	0,192	14,51	CCR3|	NA	NA	hiConfDeNovo
0	0	0	3,595	0,665	25	SCAP|	NA	NA	hiConfDeNovo

0	0	0	4,888	0,93	33	FOXP1	Accept	BP7	hiConfDeNovo
0	0	0	7,748	0,983	39	FOXP1	Accept	BP7	hiConfDeNovo
0	0	0	2,199	0,347	20,9	CPOX|	NA	NA	hiConfDeNovo
0	0,001	0,002	0	0	0	ABI3B	Donor	BP7	hiConfDeNovo
0	0	0	4,278	0,877	29,4	SENP7	Accept	VUS	hiConfDeNovo
0	0	0	1,487	0,217	15,65	NXPE	NA	NA	hiConfDeNovo
0	0	0	2,747	0,466	22,9	DZIP3|	NA	NA	hiConfDeNovo
0	0	0	0	0	0	CCDC	NA	NA	hiConfDeNovo
0	0	0	-1,25	0,003	0,002	KIAA1	NA	NA	hiConfDeNovo
0	0	0	2,101	0,326	20,1	MSL2|	NA	NA	hiConfDeNovo
0	0	0	3,522	0,647	24,8	MSL2|	NA	NA	hiConfDeNovo
0	0	0	1,132	0,167	13,07	DZIP1	NA	NA	hiConfDeNovo
0	0	0	2,944	0,508	23,3	ESYT3	Accept	BP7	hiConfDeNovo
0	0	0	0,389	0,079	5,307	PLOD2	NA	NA	hiConfDeNovo
0	0	0	0	0	0	ERICH	NA	NA	hiConfDeNovo
0	0	0	0	0	0	NMD3	Accept	BP7	hiConfDeNovo
0	0	0	2,368	0,383	21,9	PHC3|	Donor	BP7	hiConfDeNovo
0,0005	0,001	0	0	0	0	CLDN	NA	NA	hiConfDeNovo
0	0	0	4,3	0,882	29,6	RPL22	NA	NA	hiConfDeNovo
0	0	0	0	0	0	ETV5|	Accept	VUS	loConfDeNovo
0	0	0	4,072	0,811	27,5	FGF12	NA	NA	hiConfDeNovo
0	0	0	4,017	0,792	27,1	LSG1|0	Accept	BP7	hiConfDeNovo
0	0	0	0,46	0,087	6,083	MUC4	Accept	BP7	loConfDeNovo
0,0000667	0	0	0	0	0	MUC4	NA	NA	hiConfDeNovo
0	0	0	3,621	0,672	25,1	GAK|0	NA	NA	hiConfDeNovo
0,00006675	0	0	2,673	0,45	22,8	ZFYV	Donor	BP7	hiConfDeNovo
0	0	0	4,17	0,845	28,4	HTT|0.	Accept	BP7	hiConfDeNovo
0	0	0	9,425	0,998	49	EVC|0.	Accept	BP7	hiConfDeNovo
0,0000666	0	8E-04	0,791	0,125	9,301	FBXL5	NA	NA	hiConfDeNovo
0	0	0	4,188	0,851	28,5	PPAR	Accept	BP7	hiConfDeNovo
0	0	0	1,903	0,287	18,51	SEPSE	Donor	BP7	hiConfDeNovo
0	0	0	3,424	0,622	24,5	RBM4	NA	NA	hiConfDeNovo
0	0	0	-0,01	0,041	1,285	NA	NA	NA	hiConfDeNovo
0	0	0	2,143	0,335	20,4	DSPP|0	NA	NA	hiConfDeNovo
0	0	0	2,652	0,446	22,7	NPNT|	NA	NA	hiConfDeNovo
0,00006663	0	0	3,844	0,735	26	PAPSS	NA	NA	hiConfDeNovo
0	0	0	6,717	0,967	36	JADE1	Donor	BP7	hiConfDeNovo
0	0	0	2,341	0,377	21,8	PABPC	NA	NA	hiConfDeNovo
0	0	0	0	0	0	NAA1	Donor	BP7	hiConfDeNovo
0	0	0	3,513	0,644	24,7	TBC1D	NA	NA	hiConfDeNovo
0	0	0	0	0	0	RNF15	Donor	BP7	hiConfDeNovo
0	0	0	0	0	0	TIGD4	NA	NA	hiConfDeNovo
0	0	0	3,834	0,732	26	SH3RF	Accept	BP7	hiConfDeNovo
0	0	0	1,096	0,163	12,73	FAT1|0	NA	NA	hiConfDeNovo
0	0	0	-0,11	0,034	0,785	TERT|	NA	NA	hiConfDeNovo
0	0	0	4,04	0,8	27,3	HARS	NA	NA	hiConfDeNovo
0	0	0	2,691	0,454	22,8	PCDH	NA	NA	hiConfDeNovo
0	0	2E-04	0	0	0	HMP1	Accept	BP7	hiConfDeNovo
0	0	2E-04	0	0	0	SFXN1	Donor	BP7	hiConfDeNovo
0	0,001	1E-03	3,182	0,562	23,8	SSR1|0	NA	NA	hiConfDeNovo

0	0	0	2,145	0,335	20,4	DSP|0.	NA	NA	hiConfDeNovo
0	0	0	0	0	0	HIVEP	NA	NA	hiConfDeNovo
0	0	4E-04	3,906	0,755	26,4	HIST1	Accept	BP7	hiConfDeNovo
0	0	0	0	0	0	ZSCA	NA	NA	hiConfDeNovo
0	0	0	0	0	0	HLA-F	Donor	BP7	hiConfDeNovo
0	0	0	4,383	0,898	31	MSH5-	Accept	BP7	hiConfDeNovo
0	0	0	6,726	0,967	36	SYNG	Accept	BP7	hiConfDeNovo
0	0	0	2,932	0,506	23,3	ITPR3|	Accept	BP7	hiConfDeNovo
0	0	0	3,922	0,76	26,5	DLK2|	NA	NA	hiConfDeNovo
0	0	0	0	0	0	MCM3	Donor	BP7	hiConfDeNovo
0	0	0	1,182	0,174	13,49	CEP16	NA	NA	loConfDeNovo
0	0	0	0	0	0	PREP|0	NA	NA	hiConfDeNovo
0	0	0	3,863	0,741	26,1	ZBTB2	Donor	BP7	hiConfDeNovo
0	0	0	0	0	0	FAM1	Accept	BP7	hiConfDeNovo
0	0	0	0	0	0	NUS1|	NA	NA	hiConfDeNovo
0	0	0	3,879	0,746	26,2	L3MB	NA	NA	hiConfDeNovo
0	0	0	3,843	0,735	26	ARFG	Donor	PP3	hiConfDeNovo
0	0	0	0	0	0	NHSL1	Donor	BP7	hiConfDeNovo
0	0	0	1,981	0,302	19,11	NA	NA	NA	hiConfDeNovo
0	0	0	3,641	0,678	25,2	CCR6|	NA	NA	hiConfDeNovo
0	0	0	1,424	0,207	15,24	MICA	Accept	BP7	hiConfDeNovo
0	0	0	0	0	0	EIF3B|	NA	NA	hiConfDeNovo
0	0	0	0	0	0	ACTB|	NA	NA	hiConfDeNovo
0	0	0	2,699	0,456	22,8	PHF14	NA	NA	hiConfDeNovo
0	0	0	4,059	0,807	27,4	DGKB	Donor	PP3	hiConfDeNovo
0	0	0	3,278	0,585	24,1	HDAC	Accept	BP7	hiConfDeNovo
0	0	0	0,725	0,117	8,681	DNAH	NA	NA	hiConfDeNovo
0	0	0	0	0	0	RP11-1	NA	NA	hiConfDeNovo
0	0	0	0	0	0	AUTS	NA	NA	hiConfDeNovo
0	0	0	1,336	0,195	14,64	TBL2|0	NA	NA	hiConfDeNovo
0	0	0	0	0	0	ABCB	Accept	BP7	hiConfDeNovo
0	0	0	0	0	0	FAM1	Donor	BP7	hiConfDeNovo
0	0	0	2,218	0,351	21	SLC25	Donor	BP7	hiConfDeNovo
0	0	0	7,66	0,982	39	SHFM	NA	NA	hiConfDeNovo
0	0	0	0,83	0,13	9,693	PDAP1	NA	NA	hiConfDeNovo
0	0	0	0,516	0,093	6,673	NA	NA	NA	hiConfDeNovo
0	0	0	0,443	0,085	5,9	NA	NA	NA	hiConfDeNovo
0	0	0	0,845	0,131	9,844	NA	NA	NA	hiConfDeNovo
0	0	0	0	0	0	RELN|	Accept	BP7	hiConfDeNovo
0	0	0	0	0	0	FAM7	NA	NA	hiConfDeNovo
0,0001	0	0	0	0	0	TAS2R	Donor	PP3	hiConfDeNovo
0	0	2E-04	1,261	0,185	14,1	NOS3|	NA	NA	hiConfDeNovo
0	0	0	0	0	0	PRKA	NA	NA	hiConfDeNovo
0	0	0	0	0	0	KMT2	Donor	BP7	hiConfDeNovo
0	0	0	2,689	0,454	22,8	NCAP	NA	NA	hiConfDeNovo
0	0	0	1,803	0,269	17,75	ARHG	NA	NA	hiConfDeNovo
0	0	0	0	0	0	KBTB	NA	NA	hiConfDeNovo
0	0	0	1,195	0,176	13,6	MCPH	NA	NA	hiConfDeNovo
0	0	0	0,656	0,109	8,05	SGK22	NA	NA	hiConfDeNovo
0	0	0	0	0	0	MFHA	NA	NA	hiConfDeNovo

0	0	0	3,072	0,536	23,6	NAT2|	NA	NA	hiConfDeNovo
0	0	0	0	0	0	NA	NA	NA	hiConfDeNovo
0	0	0	4,277	0,876	29,4	DPYS	NA	NA	hiConfDeNovo
0	0	0	3,338	0,6	24,2	ERLIN	Accept	BP7	hiConfDeNovo
0	0	0	5,048	0,934	33	KAT6	Donor	PP3	hiConfDeNovo
0	0	0	6,571	0,965	36	MOS|0	NA	NA	hiConfDeNovo
0	0	0	3,768	0,713	25,7	MOS|0	NA	NA	hiConfDeNovo
0	0	0	0	0	0	C8orf3	NA	NA	hiConfDeNovo
0	0	0	0	0	0	OSR2|	NA	NA	hiConfDeNovo
0,0001	0	0	0	0	0	FER1L	NA	NA	hiConfDeNovo
0	0	0	0	0	0	ASAP1	NA	NA	hiConfDeNovo
0	0	0	0	0	0	TG|0.0	Donor	BP7	hiConfDeNovo
0	0	2E-04	4,384	0,898	31	ADGR	Accept	BP7	hiConfDeNovo
0	0	0	0	0	0	PLEC|	NA	NA	hiConfDeNovo
0	0	0	4,005	0,788	27	PLEC|	NA	NA	hiConfDeNovo
0	0	0	0	0	0	MROH	Accept	VUS	hiConfDeNovo
0,0002	0	2E-04	0	0	0	SMAR	Accept	BP7	hiConfDeNovo
0	0	0	2,761	0,469	22,9	PSIP1|	NA	NA	hiConfDeNovo
0	0	0	8,027	0,988	40	DNAJ	Donor	BP7	hiConfDeNovo
0	0	0	0	0	0	NFX1|	NA	NA	hiConfDeNovo
0	0	0	0	0	0	NA	NA	NA	hiConfDeNovo
0,0001	0	2E-04	2,254	0,359	21,3	ZNF48	NA	NA	hiConfDeNovo
0	0	0	3,494	0,639	24,7	GABB	NA	NA	hiConfDeNovo
0	0	0	4,269	0,875	29,3	STXBP	Donor	BP7	hiConfDeNovo
0	0	0	0	0	0	FPGS|0	NA	NA	hiConfDeNovo
0	0	2E-04	3,126	0,549	23,7	CRAT|	NA	NA	hiConfDeNovo
0	0	0	2,623	0,439	22,7	CRAT|	NA	NA	hiConfDeNovo
0	0	2E-04	0	0	0	QRFP|	Accept	BP7	hiConfDeNovo
0	0	0	-0,34	0,022	0,255	CFAP7	Accept	BP7	hiConfDeNovo
0	0	0	1,729	0,256	17,23	FCN2|	Donor	BP7	hiConfDeNovo
0	0	0	0	0	0	AGPA	Donor	BP7	hiConfDeNovo
0	0	2E-04	0	0	0	EHMT	NA	NA	hiConfDeNovo
0	0	0	4,249	0,869	29,1	OLAH	Accept	PP3	hiConfDeNovo
0	0	0	0,485	0,09	6,351	OLAH	Accept	BP7	hiConfDeNovo
0	0	0	3,498	0,64	24,7	ARL5B	Accept	BP7	hiConfDeNovo
0	0	0	4,203	0,855	28,7	MYO3	NA	NA	hiConfDeNovo
0	0	0	1,681	0,248	16,9	C10orf	NA	NA	hiConfDeNovo
0	0	0	3,437	0,625	24,5	FAM2	NA	NA	hiConfDeNovo
0	0	0	4,038	0,799	27,3	A1CF|	NA	NA	hiConfDeNovo
0	0	0	2,408	0,392	22,1	SUPV3	Donor	BP7	hiConfDeNovo
0	0	0	-0,32	0,023	0,286	C10orf	Accept	VUS	hiConfDeNovo
0	0	0	3,315	0,594	24,2	ABCC	Donor	BP7	hiConfDeNovo
0	0	0	0	0	0	NT5C2	NA	NA	hiConfDeNovo
0	0	0	2,697	0,455	22,8	NT5C2	NA	NA	hiConfDeNovo
0	0	0	3,028	0,527	23,5	PNLIP	Accept	BP7	hiConfDeNovo
0	0	0	3,037	0,528	23,5	CACU	NA	NA	hiConfDeNovo
0	0	0	6,963	0,97	36	EIF3A|	Donor	BP7	hiConfDeNovo
0	0	0	0	0	0	TIAL1	Donor	PP3	hiConfDeNovo
0	0	0	0	0	0	FUOM	Accept	BP7	hiConfDeNovo
0	0	0	4,097	0,82	27,7	PKP3|0	Accept	BP7	hiConfDeNovo

0	0	0	3,582	0,662	25	DEAF	NA	NA	hiConfDeNovo
0	0	0	3,054	0,532	23,5	MUC6	NA	NA	hiConfDeNovo
0	0	0	0,304	0,07	4,338	MUC6	NA	NA	hiConfDeNovo
0	0	0,002	0,028	0,044	1,519	MUC5	NA	NA	hiConfDeNovo
0	0	0	0	0	0	BRSK	Donor	BP7	hiConfDeNovo
0	0	0	2,195	0,346	20,8	BRSK	Donor	BP7	hiConfDeNovo
0	0	0	0	0	0	NA	NA	NA	hiConfDeNovo
0	0	0	1,798	0,268	17,71	HBE1|	NA	NA	hiConfDeNovo
0	0	0	3,818	0,727	25,9	HBG2|	NA	NA	hiConfDeNovo
0	0	0	0	0	0	MICA	Accept	BP7	hiConfDeNovo
0	0	0	2,62	0,439	22,7	MYRF	NA	NA	hiConfDeNovo
0	0	0	4,03	0,797	27,2	EHD1|	NA	NA	hiConfDeNovo
0	0	0	3,507	0,643	24,7	LTBP3	NA	NA	hiConfDeNovo
0	0	0	4,073	0,811	27,5	RBM1	NA	NA	hiConfDeNovo
0	0	0	1,817	0,271	17,85	INPPL	NA	NA	hiConfDeNovo
0	0	0	0	0	0	SLC35	Donor	PP3	hiConfDeNovo
0	0	0	7,415	0,977	38	ATM|0	Accept	PP3	hiConfDeNovo
0	0	0	3,936	0,764	26,6	USP28	Accept	BP7	hiConfDeNovo
0,0001	0,001	0	0	0	0	TMPR	NA	NA	hiConfDeNovo
0	0	0	0	0	0	PVRL1	NA	NA	hiConfDeNovo
0	0	0	0	0	0	SORL1	Donor	BP7	hiConfDeNovo
0	0	0	1,415	0,206	15,18	OR10G	Donor	BP7	hiConfDeNovo
0	0	0	3,604	0,668	25,1	PRDM	NA	NA	hiConfDeNovo
0,0000666	0	0	3,036	0,528	23,5	CCND	NA	NA	hiConfDeNovo
0	0	2E-04	3,807	0,724	25,9	FGF6|0	Donor	BP7	hiConfDeNovo
0,00006662	0	0	3,978	0,778	26,8	CHD4|	Donor	BP7	hiConfDeNovo
0	0	0	3,714	0,698	25,5	PIANP	NA	NA	hiConfDeNovo
0	0	0	1,488	0,217	15,66	HDAC	NA	NA	hiConfDeNovo
0,0001	0	0	1,443	0,21	15,37	KRT5|	NA	NA	hiConfDeNovo
0	0	0	7,799	0,984	39	KRT76	Donor	BP7	hiConfDeNovo
0	0	0	3,358	0,605	24,3	ITGA5	Accept	BP7	hiConfDeNovo
0	0	0	2,366	0,383	21,9	METT	NA	NA	hiConfDeNovo
0	0	0	5,227	0,938	33	PMEL|	Accept	PP3	hiConfDeNovo
0	0	0	3,615	0,671	25,1	TIMEL	Accept	BP7	hiConfDeNovo
0	0	0	1,586	0,232	16,28	KCNC	NA	NA	hiConfDeNovo
0	0	0	3,124	0,548	23,7	CAPS2	Donor	BP7	hiConfDeNovo
0	0	0	4,075	0,812	27,5	ANKS	NA	NA	hiConfDeNovo
0	0	0	3,179	0,561	23,8	UHRF	NA	NA	hiConfDeNovo
0	0	0	5,423	0,943	34	MYBP	Accept	PP3	hiConfDeNovo
0	0	0	0	0	0	RIC8B	NA	NA	hiConfDeNovo
0	0	0	3,659	0,683	25,3	SART3	NA	NA	hiConfDeNovo
0	0	0	5,526	0,946	34	ATXN	Accept	PP3	hiConfDeNovo
0	0	0	2,415	0,394	22,1	PTPN1	NA	NA	hiConfDeNovo
0	0	0	3,188	0,563	23,8	PLBD2	Accept	BP7	hiConfDeNovo
0	0	2E-04	0	0	0	MED1	Accept	BP7	loConfDeNovo
0	0	0	0	0	0	NOS1|	NA	NA	hiConfDeNovo
0	0	0	0	0	0	RAB35	NA	NA	hiConfDeNovo
0	0	0	0	0	0	KDM2	Accept	PP3	hiConfDeNovo
0	0	0	0	0	0	UBC|0	Accept	BP7	hiConfDeNovo
0	0	0	4,002	0,787	27	DGKH	Donor	BP7	hiConfDeNovo

0	0	0	2,388	0,388	22	HS6ST	NA	NA	hiConfDeNovo
0	0	0	2,388	0,388	22	PCCA|	NA	NA	hiConfDeNovo
0	0	0	0	0	0	TMCO	NA	NA	hiConfDeNovo
0,00006677	0	0	3,652	0,681	25,2	GAS6|	NA	NA	hiConfDeNovo
0	0	0	3,641	0,678	25,2	MYH6	Accept	BP7	hiConfDeNovo
0	0	0	4,216	0,859	28,8	MYH7	NA	NA	hiConfDeNovo
0	0	0	2,219	0,351	21	AP1G2	Accept	PP3	hiConfDeNovo
0	0	0	4,425	0,904	31	FOXG	NA	NA	hiConfDeNovo
0	0	0	0	0	0	HECT	Accept	PP3	hiConfDeNovo
0	0	0	1,782	0,265	17,6	ARHG	NA	NA	hiConfDeNovo
0	0	0	7,412	0,977	38	NPAS3	Accept	BP7	hiConfDeNovo
0	0	0	2,653	0,446	22,7	NIN|0.	NA	NA	hiConfDeNovo
0	0	0	0	0	0	CDKN	Donor	PP3	hiConfDeNovo
0	0	0	0	0	0	DACT	Accept	BP7	hiConfDeNovo
0	0	0	0	0	0	DAAM	Donor	BP7	hiConfDeNovo
0	0	0	4,372	0,897	31	ACTN	NA	NA	hiConfDeNovo
0	0	0	0	0	0	PAPLN	Donor	BP7	hiConfDeNovo
0	0	0	0	0	0	RIN3|0	Donor	BP7	hiConfDeNovo
0	0	0	1,329	0,194	14,6	MOAP	NA	NA	hiConfDeNovo
0	0	0	0	0	0	C14orf	NA	NA	hiConfDeNovo
0	0	0	4,228	0,863	28,9	PACS2	Donor	BP7	hiConfDeNovo
0	0	0	4,504	0,913	32	NA	NA	NA	hiConfDeNovo
0	0	0	-0,49	0,016	0,128	GOLG	NA	NA	hiConfDeNovo
0	0	0	2,659	0,447	22,7	BUB1	Accept	BP7	hiConfDeNovo
0	0	0	0	0	0	UBR1|	NA	NA	hiConfDeNovo
0	0	0	0	0	0	CCPG	NA	NA	hiConfDeNovo
0,00006684	0	0	0	0	0	VPS13	Accept	VUS	hiConfDeNovo
0	0	0	2,866	0,491	23,1	HCN4|	NA	NA	hiConfDeNovo
0	0	0	4,043	0,801	27,3	C15orf	Donor	BP7	hiConfDeNovo
0	0	0	3,795	0,721	25,8	ISLR2|	NA	NA	hiConfDeNovo
0	0	0	0	0	0	LMAN	NA	NA	hiConfDeNovo
0	0	0	0	0	0	LMAN	Accept	BP7	hiConfDeNovo
0	0	0	3,988	0,782	26,9	PSTPI	Accept	BP7	hiConfDeNovo
0	0	0	2,982	0,516	23,4	RP11-3	NA	NA	hiConfDeNovo
0	0	0	2,54	0,421	22,5	LINS1|	NA	NA	hiConfDeNovo
0	0	0	0,935	0,142	10,82	RGS11	Donor	BP7	hiConfDeNovo
0,0003	0	0	-0,19	0,029	0,529	PRR35	NA	NA	hiConfDeNovo
0	0	0	5,284	0,94	33	CACN	Donor	PP3	hiConfDeNovo
0	0	0	2,574	0,429	22,6	MLST	Accept	BP7	hiConfDeNovo
0	0	2E-04	0	0	0	ABCA	Donor	BP7	hiConfDeNovo
0	0	0	0	0	0	PRSS2	NA	NA	hiConfDeNovo
0	0	0	3,185	0,562	23,8	PMM2	NA	NA	hiConfDeNovo
0	0	0	2,641	0,443	22,7	USP7|0	NA	NA	hiConfDeNovo
0	0	0	0	0	0	DNAH	Donor	BP7	hiConfDeNovo
0	0	0	0	0	0	NA	NA	NA	hiConfDeNovo
0	0	0	4,439	0,906	31	HSD3B	Accept	PP3	hiConfDeNovo
0	0	0	0	0	0	NA	NA	NA	hiConfDeNovo
0	0	0	3,282	0,586	24,1	ELMO	Accept	BP7	hiConfDeNovo
0	0	0	0	0	0	WWP2	Accept	BP7	hiConfDeNovo
0	0	0	4,152	0,839	28,2	NA	NA	NA	hiConfDeNovo

0	0	0	3,861	0,741	26,1	DHOD	Accept	BP7	hiConfDeNovo
0,00006664	0	0	4,411	0,903	31	ZFHX3	NA	NA	hiConfDeNovo
0	0	0	7,89	0,985	40	BCAR	NA	NA	hiConfDeNovo
0	0	0	2,981	0,516	23,4	FOXL	NA	NA	hiConfDeNovo
0	0	0	2,7	0,456	22,8	PIEZO	Accept	PP3	hiConfDeNovo
0	0	0	3,217	0,57	23,9	ANKR	NA	NA	hiConfDeNovo
0	0	0	3,057	0,533	23,5	ANKR	NA	NA	hiConfDeNovo
0	0	0	2,243	0,356	21,2	SPG7|0	Donor	PP3	hiConfDeNovo
0,00006663	0	0	3,312	0,593	24,2	FANC	Donor	BP7	hiConfDeNovo
0	0	0	3,564	0,657	24,9	PRPF8	Accept	BP7	hiConfDeNovo
0	0	0	0	0	0	GLTP	NA	NA	hiConfDeNovo
0	0	0	3,874	0,745	26,2	EIF5A|	NA	NA	hiConfDeNovo
0	0	0	0	0	0	LLGL1	NA	NA	hiConfDeNovo
0	0	0	0	0	0	SPECC	Donor	BP7	hiConfDeNovo
0	0	0	0	0	0	SPECC	Donor	BP7	hiConfDeNovo
0	0	0	0	0	0	DHRS	Accept	BP7	hiConfDeNovo
0	0	0	0	0	0	ABHD	Donor	BP7	hiConfDeNovo
0	0	0	3,364	0,606	24,3	NF1|0.	NA	NA	hiConfDeNovo
0,00006662	0	0	3,638	0,677	25,2	FBXL2	Donor	PP3	hiConfDeNovo
0	0	0	2,092	0,325	19,96	RARA	Donor	VUS	hiConfDeNovo
0	0	0	3,001	0,521	23,4	KRTA	NA	NA	hiConfDeNovo
0	0	0	4,046	0,802	27,3	SPATA	Accept	BP7	hiConfDeNovo
0	0	0	0,953	0,145	11,03	BZRA	NA	NA	hiConfDeNovo
0	0	0	-0,48	0,016	0,132	BZRA	Donor	BP7	hiConfDeNovo
0	0	0	6,405	0,963	36	TLK2|	Accept	BP7	hiConfDeNovo
0	0	0	2,978	0,516	23,4	ABCA	NA	NA	loConfDeNovo
0,0001	0	4E-04	0	0	0	GRIN2	Donor	PP3	hiConfDeNovo
0	0	0	0	0	0	CASKI	NA	NA	hiConfDeNovo
0,00006676	0	0	1,067	0,159	12,43	EVPL|	Accept	BP7	hiConfDeNovo
0	0	0	0	0	0	DNAH	NA	NA	hiConfDeNovo
0	0	0	0	0	0	DNAH	Accept	BP7	hiConfDeNovo
0	0	0	0	0	0	GAA|0	NA	NA	hiConfDeNovo
0	0	0	4,19	0,851	28,6	NPC1|	Donor	BP7	hiConfDeNovo
0	0	0	0	0	0	LAMA	Accept	PP3	hiConfDeNovo
0	0	0	0	0	0	ASXL	NA	NA	hiConfDeNovo
0	0	0	0	0	0	NA	NA	NA	hiConfDeNovo
0	0	0	3,776	0,715	25,7	ST8SI	Accept	BP7	hiConfDeNovo
0	0	0	0	0	0	CDH20	Accept	BP7	hiConfDeNovo
0	0	0	1,289	0,188	14,3	PRSS5	NA	NA	hiConfDeNovo
0	0	0	4,184	0,849	28,5	STK11	Accept	BP7	hiConfDeNovo
0	0	0	4,008	0,789	27	GAMT	Accept	BP7	hiConfDeNovo
0	0	0	0	0	0	ATP8B	Accept	BP7	hiConfDeNovo
0	0	0	3,815	0,726	25,9	MFSD	Donor	BP7	hiConfDeNovo
0	0	0	0,74	0,119	8,819	GIPC3	NA	NA	hiConfDeNovo
0	0	0	2,345	0,378	21,8	SEMA	Accept	BP7	hiConfDeNovo
0,00006668	0	0,001	0	0	0	PTPRS	NA	NA	hiConfDeNovo
0	0	0	0	0	0	LONP	Donor	BP7	hiConfDeNovo
0	0	0	3,22	0,571	23,9	CD70|0	NA	NA	hiConfDeNovo
0	0	0	0	0	0	LRRC	NA	NA	hiConfDeNovo
0	0	0	0	0	0	NA	NA	NA	hiConfDeNovo

0	0	0	0	0	0	NA	NA	NA	hiConfDeNovo
0	0	0	0	0	0	NA	NA	NA	hiConfDeNovo
0	0	0	1,482	0,216	15,62	CTD-2	Donor	BP7	hiConfDeNovo
0	0	0	0	0	0	FBXW	Accept	PP3	hiConfDeNovo
0,00006675	0	0	1,839	0,275	18,02	CTD-2	NA	NA	hiConfDeNovo
0	0	2E-04	3,352	0,603	24,3	CTD-2	NA	NA	hiConfDeNovo
0	0	0	3,332	0,598	24,2	JAK3|0	Accept	BP7	hiConfDeNovo
0,0001	0	0	2,844	0,487	23,1	CILP2|	Accept	BP7	hiConfDeNovo
0	0	0	-0,23	0,027	0,448	ZNF91	NA	NA	hiConfDeNovo
0	0	0	8,019	0,987	40	ZNF53	NA	NA	hiConfDeNovo
0	0	0	0	0	0	GPAT	NA	NA	hiConfDeNovo
0	0	0	0	0	0	SCN1B	Accept	BP7	hiConfDeNovo
0,0039	0,0035	0,008	0	0	0	SBSN|	Donor	BP7	hiConfDeNovo
0	0	0	1,029	0,154	11,98	ZFP36|	NA	NA	hiConfDeNovo
0,00006661	0	6E-04	3,811	0,725	25,9	LTBP4	Donor	BP7	hiConfDeNovo
0	0	0	0	0	0	AC011	Donor	BP7	hiConfDeNovo
0	0	0	0	0	0	ZNF22	NA	NA	hiConfDeNovo
0	0	0	0	0	0	SULT2	NA	NA	hiConfDeNovo
0,0001	0	2E-04	0,069	0,047	1,827	SULT2	NA	NA	hiConfDeNovo
0	0	0	0	0	0	FTL|0.	NA	NA	hiConfDeNovo
0	0	0	0	0	0	PPFIA	Accept	BP7	hiConfDeNovo
0	0	0	4,028	0,796	27,2	SHAN	Accept	BP7	hiConfDeNovo
0	0	4E-04	-0,05	0,038	1,036	PPP6R	Accept	BP7	hiConfDeNovo
0	0	0	2,4	0,39	22,1	UBE2S	NA	NA	hiConfDeNovo
0	0	0	3,558	0,656	24,9	CSNK	NA	NA	hiConfDeNovo
0	0	0	0	0	0	SIRPB	Accept	BP7	hiConfDeNovo
0	0	0	0	0	0	NA	NA	NA	hiConfDeNovo
0	0	0	0	0	0	CFAP6	Accept	BP7	hiConfDeNovo
0	0	0	2,745	0,466	22,9	ACTL	NA	NA	hiConfDeNovo
0	0	0	1,964	0,299	18,98	ZNF34	Accept	BP7	hiConfDeNovo
0	0,001	0	0	0	0	ERGIC	NA	NA	hiConfDeNovo
0	0	0	3,316	0,594	24,2	TGM2|	NA	NA	hiConfDeNovo
0	0	0	3,257	0,58	24	ZNF33	Accept	BP7	hiConfDeNovo
0	0	0	4,236	0,865	29	KCNB	NA	NA	hiConfDeNovo
0	0	0	2,608	0,436	22,7	PTGIS	Accept	BP7	hiConfDeNovo
0	0	0	2,963	0,512	23,3	CDH4|	Donor	BP7	hiConfDeNovo
0	0	0	3,799	0,722	25,8	TAF4|0	NA	NA	loConfDeNovo
0	0	0	0,481	0,089	6,304	LAMA	Donor	BP7	hiConfDeNovo
0	0	0	3,776	0,715	25,7	EEF1A	Accept	BP7	hiConfDeNovo
0	0	0	0	0	0	SRMS|	NA	NA	hiConfDeNovo
0	0	0	-0,12	0,033	0,738	NPBW	Accept	BP7	hiConfDeNovo
0	0	0	0	0	0	ZBTB2	NA	NA	hiConfDeNovo
0	0	0	0	0	0	AIRE|0	Accept	BP7	hiConfDeNovo
0	0	0	0	0	0	ITGB2	Accept	BP7	hiConfDeNovo
0,00006679	0	0	0	0	0	RAB36	NA	NA	hiConfDeNovo
0	0	0	-1,17	0,003	0,003	ZNRF3	NA	NA	hiConfDeNovo
0	0	4E-04	3,786	0,718	25,8	MORC	Accept	BP7	hiConfDeNovo
0	0	0	0	0	0	DEPD	Donor	BP7	hiConfDeNovo
0	0	0	3,872	0,744	26,2	SLC16	Accept	BP7	hiConfDeNovo
0	0	0	3,997	0,785	27	TRMU	Accept	BP7	hiConfDeNovo

0	0	0	0	0	0	CELSR	NA	NA	hiConfDeNovo
0	0	0	2,045	0,315	19,6	ZBED4	NA	NA	hiConfDeNovo
0	0	0	1,788	0,266	17,65	PLXN	Donor	BP7	hiConfDeNovo
0	0	0	4,292	0,88	29,5	SHAN	NA	NA	hiConfDeNovo
0	0	0	0	0	0	SHAN	NA	NA	hiConfDeNovo

DeNovoGear	QUAL	FILTE	INFO	RRV	status	DNV	id
0,969948	1354	PASS	AC=1;	no	proban	yes	P_91
0,969948	1354	PASS	AC=1;	no	proban	yes	P_91
0,998485	257,2	PASS	AC=1;	no	proban	yes	P_134
0,999216	477,7	PASS	AC=1;	no	proban	yes	P_145
0:00:00	454,1	PASS	AC=1;	no	proban	yes	P_74
1	1494	PASS	AC=1;	yes	proban	yes	P_115
0,999994	554	PASS	AC=1;	no	proban	yes	P_39
1	1287	PASS	AC=1;	no	proban	yes	P_398
0,998093	494	PASS	AC=1;	no	proban	yes	P_342
1	2489	PASS	AC=1;	no	proban	yes	P_145
1	516	PASS	AC=1;	no	proban	yes	P_404
0,999995	2443	PASS	AC=1;	no	proban	yes	P_120
0,999998	1214	PASS	AC=1;	no	proban	yes	P_284
0,999994	1183	PASS	AC=1;	no	proban	yes	P_192
0,999995	681	PASS	AC=1;	no	proban	yes	P_311
0,999996	541	PASS	AC=1;	no	proban	yes	P_368
1	951	PASS	AC=1;	no	proban	yes	P_157
1	705	PASS	AC=1;	no	proban	yes	P_176
0,999997	1994	PASS	AC=1;	no	proban	yes	P_120
0,999997	516	PASS	AC=1;	no	proban	yes	P_39
0,999994	327,1	PASS	AC=1;	no	proban	yes	P_410
00:00,0	1610	PASS	AC=1;	no	proban	yes	P_73
00:00,0	750,4	PASS	AC=1;	no	proban	yes	P_75
00:00,0	1247	PASS	AC=1;	no	proban	yes	P_60
0,999997	636,1	PASS	AC=1;	no	proban	yes	P_110
0,872495	1382	PASS	AC=1;	no	proban	yes	P_142
1	937	PASS	AC=1;	no	proban	yes	P_418
0,999997	738	PASS	AC=1;	no	proban	yes	P_15
0,999994	441	PASS	AC=1;	no	proban	yes	P_51
0,999952	544	PASS	AC=1;	no	proban	yes	P_248
0,999997	3495	PASS	AC=1;	no	proban	yes	P_88
0,999997	1270	PASS	AC=1;	no	proban	yes	P_398
0,999995	788	PASS	AC=1;	no	proban	yes	P_430
0,999999	2190	PASS	AC=1;	no	proban	yes	P_224
0,969948	618,5	PASS	AC=1;	no	proban	yes	P_121
1	1522	PASS	AC=1;	no	proban	yes	P_416
1	806	PASS	AC=1;	no	proban	yes	P_97
0,999237	234,1	PASS	AC=1;	no	proban	yes	P_174
1	987	PASS	AC=1;	no	proban	yes	P_169
1	1572	PASS	AC=1;	no	proban	yes	P_171
1	21262	PASS	AC=1;	no	proban	yes	P_311
0,999997	332	PASS	AC=1;	no	proban	yes	P_393
0:00:00	2236	PASS	AC=1;	no	proban	yes	P_70
1	1173	PASS	AC=1;	no	proban	yes	P_144
1	867	PASS	AC=1;	no	proban	yes	P_343
0,999997	1027	PASS	AC=1;	no	proban	yes	P_409
1	1309	PASS	AC=1;	no	proban	yes	P_426
0:00:00	1941	PASS	AC=1;	no	proban	yes	P_69

00:00,0	1062	PASS	AC=1; no	proban yes	P_81
0,976515	223	PASS	AC=1; no	proban yes	P_385
0,999997	939	PASS	AC=1; no	proban yes	P_207
0,999994	859	PASS	AC=1; no	proban yes	P_337
0,999994	355,1	PASS	AC=1; no	proban yes	P_112
1	955	PASS	AC=1; no	proban yes	P_26
1	2519	PASS	AC=1; no	proban yes	P_422
1	2507	PASS	AC=1; no	proban yes	P_422
0,999994	150	PASS	AC=1; no	proban yes	P_368
0,999997	395	PASS	AC=1; no	proban yes	P_1
0,999806	496	PASS	AC=1; no	proban yes	P_416
0,999995	264	PASS	AC=1; no	proban yes	P_306
1	1265	PASS	AC=1; no	proban yes	P_238
0,976517	505	PASS	AC=1; no	proban yes	P_212
0,999997	1348	PASS	AC=1; no	proban yes	P_414
0,999995	1483	PASS	AC=1; no	proban yes	P_317
1	935	PASS	AC=1; no	proban yes	P_137
1	744	PASS	AC=1; yes	proban yes	P_292
1	498	PASS	AC=1; no	proban yes	P_112
0,999996	846	PASS	AC=1; no	proban yes	P_241
0,999999	422,1	PASS	AC=1; no	proban yes	P_340
0,999994	1182	PASS	AC=1; yes	proban yes	P_3
0,999999	1288	PASS	AC=1; no	proban yes	P_296
0,999997	1301	PASS	AC=1; no	proban yes	P_153
0,999997	2299	PASS	AC=1; no	proban yes	P_404
0,775606	890	PASS	AC=1; yes	proban yes	P_203
0,999967	619	PASS	AC=1; no	proban yes	P_327
0,999999	670	PASS	AC=1; no	proban yes	P_87
0,999997	806	PASS	AC=1; no	proban yes	P_180
0,999997	1221	PASS	AC=1; no	proban yes	P_328
0,999998	654	PASS	AC=1; yes	proban yes	P_24
0,999999	1602	PASS	AC=1; no	proban yes	P_297
0,992499	205,1	PASS	AC=1; yes	proban yes	P_332
1	1175	PASS	AC=1; yes	proban yes	P_320
0,999994	725	PASS	AC=1; no	proban yes	P_333
0,999995	211	PASS	AC=1; yes	proban yes	P_281
1	1145	PASS	AC=1; no	proban yes	P_416
1	1720	PASS	AC=1; no	proban yes	P_299
0,999998	1267	PASS	AC=1; no	proban yes	P_227
0,998637	368	PASS	AC=1; no	proban yes	P_141
0,999998	972	PASS	AC=1; no	proban yes	P_270
1	1222	PASS	AC=1; no	proban yes	P_25
0,999998	1225	PASS	AC=1; no	proban yes	P_101
1	458	PASS	AC=1; no	proban yes	P_254
0,999997	479,1	PASS	AC=1; no	proban yes	P_254
0,999995	270	PASS	AC=1; no	proban yes	P_241
00:00,0	557	PASS	AC=1; no	proban yes	P_68
0,999998	3081	PASS	AC=1; no	proban yes	P_266
0,999996	1908	PASS	AC=1; no	proban yes	P_408
0,999975	829,1	PASS	AC=1; no	proban yes	P_310

0,999998	470	PASS	AC=1;	yes	proban yes	P_238
1	2016	PASS	AC=1;	yes	proban yes	P_333
0,998482	455,2	PASS	AC=1;	no	proban yes	P_306
0,999901	1222	PASS	AC=1;	no	proban yes	P_192
0,952835	200,1	PASS	AC=1;	no	proban yes	P_18
1	1455	PASS	AC=1;	no	proban yes	P_297
0,999995	1380	PASS	AC=1;	no	proban yes	P_38
1	2214	PASS	AC=2;	no	proban yes	P_433
0,999996	996	PASS	AC=1;	no	proban yes	P_302
0,999995	796	PASS	AC=1;	no	proban yes	P_306
0,999995	817	PASS	AC=1;	no	proban yes	P_306
0,999994	892	PASS	AC=1;	no	proban yes	P_333
0,999998	373	PASS	AC=1;	no	proban yes	P_187
0,999996	9089	PASS	AC=1;	no	proban yes	P_5
0,999997	764	PASS	AC=1;	no	proban yes	P_8
0,97079	210,1	PASS	AC=1;	no	proban yes	P_62
0,999994	765	PASS	AC=1;	no	proban yes	P_226
0,999994	3374	PASS	AC=1;	no	proban yes	P_52
0,999995	700	PASS	AC=1;	no	proban yes	P_215
0,720888	356,4	PASS	AC=1;	no	proban yes	P_299
0,999994	535	PASS	AC=1;	no	proban yes	P_163
0,976505	462	PASS	AC=1;	no	proban yes	P_300
0,970615	19590	PASS	AC=1;	no	proban yes	P_122
1	1934	PASS	AC=1;	no	proban yes	P_273
0,999999	615	PASS	AC=1;	no	proban yes	P_138
1	2056	PASS	AC=1;	no	proban yes	P_203
0,999997	553	PASS	AC=1;	no	proban yes	P_262
0,999998	623	PASS	AC=1;	no	proban yes	P_296
0,965157	247	PASS	AC=1;	no	proban yes	P_324
1	899	PASS	AC=1;	no	proban yes	P_51
0,999995	603	PASS	AC=1;	no	proban yes	P_61
0,999998	981	PASS	AC=1;	no	proban yes	P_35
0,999998	896	PASS	AC=1;	no	proban yes	P_37
1	4009	PASS	AC=1;	no	proban yes	P_180
0,999996	1310	PASS	AC=1;	no	proban yes	P_8
0,999995	650	PASS	AC=1;	no	proban yes	P_231
0,999997	770	PASS	AC=1;	yes	proban yes	P_213
1	2667	PASS	AC=1;	no	proban yes	P_85
0,999997	459	PASS	AC=1;	no	proban yes	P_25
0,999999	1310	PASS	AC=1;	no	proban yes	P_292
0,999994	924	PASS	AC=1;	no	proban yes	P_10
00:00,0	2785	PASS	AC=1;	no	proban yes	P_65
1	1280	PASS	AC=1;	no	proban yes	P_1
1	1691	PASS	AC=1;	no	proban yes	P_202
1	1562	PASS	AC=1;	no	proban yes	P_93
1	3136	PASS	AC=1;	no	proban yes	P_112
0,999998	3385	PASS	AC=1;	no	proban yes	P_131
0,999998	425	PASS	AC=1;	no	proban yes	P_113
0,999994	433	PASS	AC=1;	no	proban yes	P_273
0,999997	635	PASS	AC=1;	no	proban yes	P_313

1	1618	PASS	AC=1; no	proban yes	P_115
0,999998	1452	PASS	AC=1; no	proban yes	P_276
0,999999	1536	PASS	AC=1; no	proban yes	P_402
1	1483	PASS	AC=1; no	proban yes	P_128
1	813,1	PASS	AC=1; no	proban yes	P_332
0,999997	1277	PASS	AC=1; no	proban yes	P_203
1	3036	PASS	AC=1; yes	proban yes	P_300
0,999997	581,1	PASS	AC=1; no	proban yes	P_295
0,999999	1102	PASS	AC=1; no	proban yes	P_45
0,999994	524	PASS	AC=1; no	proban yes	P_34
0,71165	326,1	PASS	AC=1; no	proban yes	P_308
1	1128	PASS	AC=1; no	proban yes	P_93
0,999995	704	PASS	AC=1; no	proban yes	P_338
0,999999	1042	PASS	AC=1; no	proban yes	P_91
1	1335	PASS	AC=1; no	proban yes	P_89
0,699732	102,1	PASS	AC=1; no	proban yes	P_24
0,999994	511	PASS	AC=1; no	proban yes	P_307
1	738	PASS	AC=1; no	proban yes	P_39
1	1947	PASS	AC=1; no	proban yes	P_24
0,999998	466	PASS	AC=1; no	proban yes	P_113
1	1589	PASS	AC=1; no	proban yes	P_22
0,999995	363	PASS	AC=1; no	proban yes	P_249
0,999997	1689	PASS	AC=1; no	proban yes	P_243
0,999998	1131	PASS	AC=1; no	proban yes	P_259
0,999994	1183	PASS	AC=1; no	proban yes	P_156
0,999997	607	PASS	AC=1; no	proban yes	P_426
0,988088	384	PASS	AC=1; no	proban yes	P_296
0,999997	1030	PASS	AC=1; no	proban yes	P_282
1	945,5	PASS	AC=1; no	proban yes	P_93
00:00,0	1192	PASS	AC=1; no	proban yes	P_75
0,999997	742,2	PASS	AC=1; no	proban yes	P_110
1	362	PASS	AC=1; no	proban yes	P_17
0,999997	445,8	PASS	AC=1; no	proban yes	P_180
0,777147	192	PASS	AC=1; no	proban yes	P_311
0,999998	755	PASS	AC=1; no	proban yes	P_252
1	4826	PASS	AC=1; no	proban yes	P_153
1	2349	PASS	AC=1; no	proban yes	P_153
1	2346	PASS	AC=1; no	proban yes	P_153
0,999994	474	PASS	AC=1; no	proban yes	P_215
1	1783	PASS	AC=1; no	proban yes	P_340
0,999998	678	PASS	AC=1; no	proban yes	P_325
0,999996	1113	PASS	AC=1; no	proban yes	P_295
0,999998	2794	PASS	AC=1; no	proban yes	P_254
0,999973	357,7	PASS	AC=1; no	proban yes	P_115
0,976335	533,1	PASS	AC=1; no	proban yes	P_336
1	2905	PASS	AC=1; no	proban yes	P_249
0,954193	20543	PASS	AC=1; no	proban yes	P_373
0,999997	719	PASS	AC=1; no	proban yes	P_221
1	1620	PASS	AC=1; no	proban yes	P_231
0,999806	174,2	PASS	AC=1; no	proban yes	P_316

0,999998	473	PASS	AC=1; no	proban	yes	P_268
0,995461	3366	PASS	AC=1; no	proban	yes	P_20
1	825	PASS	AC=1; no	proban	yes	P_308
1	815	PASS	AC=1; no	proban	yes	P_94
0,998482	381	PASS	AC=1; yes	proban	yes	P_301
0,999997	1495	PASS	AC=1; yes	proban	yes	P_240
0,999997	1495	PASS	AC=1; no	proban	yes	P_240
0,976514	272	PASS	AC=1; no	proban	yes	P_1
0,999974	650	PASS	AC=1; no	proban	yes	P_373
0,999998	301	PASS	AC=1; no	proban	yes	P_7
0:00:00	1925	PASS	AC=1; no	proban	yes	P_63
0,999999	428	PASS	AC=1; no	proban	yes	P_126
0,999998	471	PASS	AC=1; no	proban	yes	P_270
0,999997	3686	PASS	AC=1; no	proban	yes	P_29
1	2779	PASS	AC=1; no	proban	yes	P_144
1	2355	PASS	AC=1; no	proban	yes	P_24
0,999618	245	PASS	AC=1; no	proban	yes	P_30
0,999996	467,1	PASS	AC=1; no	proban	yes	P_14
0,999994	1197	PASS	AC=1; no	proban	yes	P_182
0,999986	711	PASS	AC=1; no	proban	yes	P_260
0,995461	5944	PASS	AC=1; no	proban	yes	P_135
0,999997	3101	PASS	AC=1; no	proban	yes	P_418
0,999995	1475	PASS	AC=1; no	proban	yes	P_439
0,999996	1078	PASS	AC=1; no	proban	yes	P_346
0,822999	524,4	PASS	AC=1; no	proban	yes	P_146
0,993245	197	PASS	AC=1; no	proban	yes	P_113
0,999998	1414	PASS	AC=1; no	proban	yes	P_166
0,999996	2398	PASS	AC=1; no	proban	yes	P_350
0,999994	588	PASS	AC=1; no	proban	yes	P_37
1	1002	PASS	AC=1; no	proban	yes	P_400
0,999997	930,7	PASS	AC=1; no	proban	yes	P_273
1	1730	PASS	AC=1; no	proban	yes	P_137
00:00,0	2425	PASS	AC=1; no	proban	yes	P_76
00:00,0	2425	PASS	AC=1; no	proban	yes	P_76
0,999998	674	PASS	AC=1; no	proban	yes	P_359
1	1074	PASS	AC=1; no	proban	yes	P_104
0,999975	155	PASS	AC=1; no	proban	yes	P_249
1	1151	PASS	AC=1; no	proban	yes	P_203
0,999996	499	PASS	AC=1; no	proban	yes	P_364
0,999994	621,1	PASS	AC=1; no	proban	yes	P_172
0,999995	1043	PASS	AC=1; no	proban	yes	P_55
0,999995	459	PASS	AC=1; no	proban	yes	P_56
0:00:00	2415	PASS	AC=1; no	proban	yes	P_74
0:00:00	2833	PASS	AC=1; no	proban	yes	P_74
0,954212	319	PASS	AC=1; no	proban	yes	P_289
0,999996	682	PASS	AC=1; no	proban	yes	P_304
0,999995	3235	PASS	AC=1; yes	proban	yes	P_429
0,999951	236	PASS	AC=1; no	proban	yes	P_13
0,999997	1690	PASS	AC=1; no	proban	yes	P_270
0,999997	1213	PASS	AC=1; no	proban	yes	P_375

1	1480	PASS	AC=1;	no	proban	yes	P_239
0,999995	649,1	PASS	AC=1;	no	proban	yes	P_258
0,999996	3205	PASS	AC=1;	no	proban	yes	P_190
0,999994	153	PASS	AC=1;	no	proban	yes	P_355
0,995461	3294	PASS	AC=1;	yes	proban	yes	P_16
0,999997	1251	PASS	AC=1;	no	proban	yes	P_289
0,980017	1194	PASS	AC=1;	no	proban	yes	P_44
0,999997	371,1	PASS	AC=1;	no	proban	yes	P_62
0,99924	299	PASS	AC=1;	no	proban	yes	P_338
0,999996	545	PASS	AC=1;	no	proban	yes	P_215
1	948,8	PASS	AC=1;	no	proban	yes	P_259
0,999997	2594	PASS	AC=1;	no	proban	yes	P_265
1	2788	PASS	AC=1;	no	proban	yes	P_136
0,999994	517	PASS	AC=1;	no	proban	yes	P_281
0,999998	1111	PASS	AC=1;	no	proban	yes	P_409
0,954111	357,1	PASS	AC=1;	no	proban	yes	P_309
1	1019	PASS	AC=1;	no	proban	yes	P_373
0,999996	553	PASS	AC=1;	no	proban	yes	P_255
0,999998	1230	PASS	AC=1;	no	proban	yes	P_255
0,980722	272,1	PASS	AC=1;	no	proban	yes	P_436
0,999994	983	PASS	AC=1;	no	proban	yes	P_363
0,999996	1156	PASS	AC=1;	no	proban	yes	P_412
0,999999	613	PASS	AC=1;	no	proban	yes	P_213
0,975792	201,3	PASS	AC=1;	no	proban	yes	P_12
1	414	PASS	AC=1;	no	proban	yes	P_408
1	621	PASS	AC=1;	no	proban	yes	P_5
0,999998	1638	PASS	AC=1;	no	proban	yes	P_224
0,999994	966,1	PASS	AC=1;	no	proban	yes	P_379
00:00,0	169	PASS	AC=1;	no	proban	yes	P_68
0,999999	996	PASS	AC=1;	no	proban	yes	P_284
0,999997	817	PASS	AC=1;	no	proban	yes	P_432
0,999995	1404	PASS	AC=1;	no	proban	yes	P_138
0,999997	335	PASS	AC=1;	no	proban	yes	P_408
0,999996	796	PASS	AC=1;	no	proban	yes	P_93
1	1680	PASS	AC=1;	no	proban	yes	P_212
00:00,0	1407	PASS	AC=2;	no	proban	yes	P_79
0,999998	394	PASS	AC=1;	no	proban	yes	P_372
0,999995	815	PASS	AC=1;	no	proban	yes	P_216
1	1165	PASS	AC=1;	no	proban	yes	P_381
0,999995	692	PASS	AC=1;	no	proban	yes	P_378
1	1756	PASS	AC=1;	no	proban	yes	P_426
0,999994	581,4	PASS	AC=1;	yes	proban	yes	P_176
0,999997	495,1	PASS	AC=1;	no	proban	yes	P_154
0,999998	499,1	PASS	AC=1;	no	proban	yes	P_224
0,98111	253,8	PASS	AC=1;	no	proban	yes	P_16
0,999997	537	PASS	AC=1;	no	proban	yes	P_249
0,999997	1978	PASS	AC=1;	no	proban	yes	P_134
0,999997	1317	PASS	AC=1;	yes	proban	yes	P_129
0,775606	1564	PASS	AC=1;	no	proban	yes	P_249
0,999994	819	PASS	AC=1;	no	proban	yes	P_50

0,999997	1300	PASS	AC=1; no	proban yes	P_18
0,993984	287	PASS	AC=1; no	proban yes	P_202
0,999998	2402	PASS	AC=1; no	proban yes	P_357
0,999995	1074	PASS	AC=1; no	proban yes	P_249
1	3342	PASS	AC=1; no	proban yes	P_26
1	922,1	PASS	AC=1; no	proban yes	P_227
0,999999	1055	PASS	AC=1; no	proban yes	P_364
1	2480	PASS	AC=1; yes	proban yes	P_374
0,999999	954	PASS	AC=1; no	proban yes	P_345
0,999996	1365	PASS	AC=1; no	proban yes	P_96
0,893028	416	PASS	AC=1; yes	proban yes	P_114
0,97079	203	PASS	AC=1; no	proban yes	P_422
0,999901	248,7	PASS	AC=1; no	proban yes	P_181
1	1178	PASS	AC=1; no	proban yes	P_111
0,999997	463	PASS	AC=1; no	proban yes	P_203
0,999997	226	PASS	AC=1; yes	proban yes	P_274
1	2364	PASS	AC=1; no	proban yes	P_108
0,999999	2184	PASS	AC=1; no	proban yes	P_190
0,999997	973,2	PASS	AC=1; no	proban yes	P_255
0,999806	700	PASS	AC=1; no	proban yes	P_184
0,999995	783	PASS	AC=1; no	proban yes	P_164
0,999998	731	PASS	AC=1; yes	proban yes	P_248
1	1680	PASS	AC=1; no	proban yes	P_294
1	1119	PASS	AC=1; no	proban yes	P_140
0,963006	1302	PASS	AC=1; no	proban yes	P_436
0,999996	1652	PASS	AC=1; no	proban yes	P_226
0,999997	905	PASS	AC=1; no	proban yes	P_369
0,999999	679,3	PASS	AC=1; no	proban yes	P_18
0,999999	1858	PASS	AC=1; no	proban yes	P_164
0,999998	414,1	PASS	AC=1; no	proban yes	P_318
0,769172	1016	PASS	AC=1; no	proban yes	P_237
0,999997	1016	PASS	AC=1; no	proban yes	P_237
0,999998	1056	PASS	AC=1; no	proban yes	P_159
0,999806	594	PASS	AC=1; no	proban yes	P_269
0,912608	168	PASS	AC=1; no	proban yes	P_324
0,999998	1365	PASS	AC=1; no	proban yes	P_303
1	1207	PASS	AC=1; no	proban yes	P_6
0,999995	872	PASS	AC=1; no	proban yes	P_349
0,999998	608	PASS	AC=1; no	proban yes	P_22
0,999997	1065	PASS	AC=1; no	proban yes	P_226
0,999998	830,2	PASS	AC=1; no	proban yes	P_310
1	1366	PASS	AC=1; no	proban yes	P_345
0,999997	419,1	PASS	AC=1; no	proban yes	P_19
0,999994	386,2	PASS	AC=1; no	proban yes	P_181
0,995461	1914	PASS	AC=1; yes	proban yes	P_172
0,999998	974	PASS	AC=1; no	proban yes	P_299
0,995461	2659	PASS	AC=1; no	proban yes	P_87
1	2585	PASS	AC=1; no	proban yes	P_87
0,999997	420	PASS	AC=1; no	proban yes	P_402
0,850498	199,1	PASS	AC=1; no	proban yes	P_298

0,887653	234,1	PASS	AC=1;	no	proban	yes	P_371
0,999998	960	PASS	AC=1;	yes	proban	yes	P_295
0,999998	477,1	PASS	AC=1;	yes	proban	yes	P_243
0,999996	2258	PASS	AC=1;	no	proban	yes	P_265
0,999996	1470	PASS	AC=1;	no	proban	yes	P_357
0,999995	1192	PASS	AC=1;	no	proban	yes	P_88
0,999995	1192	PASS	AC=1;	no	proban	yes	P_88
0,999997	2282	PASS	AC=1;	no	proban	yes	P_8
1	2046	PASS	AC=1;	no	proban	yes	P_254
0,999997	697	PASS	AC=1;	no	proban	yes	P_338
0,999982	566,1	PASS	AC=1;	no	proban	yes	P_328
0,999999	1700	PASS	AC=1;	no	proban	yes	P_241
0,963006	785,9	PASS	AC=1;	no	proban	yes	P_32
0,995461	1403	PASS	AC=1;	no	proban	yes	P_257
0,995461	1406	PASS	AC=1;	no	proban	yes	P_257
0,999807	142	PASS	AC=1;	no	proban	yes	P_320
1	1392	PASS	AC=1;	no	proban	yes	P_335
0,999996	454	PASS	AC=1;	no	proban	yes	P_88
0,999806	452,6	PASS	AC=1;	no	proban	yes	P_376
1	1365	PASS	AC=1;	no	proban	yes	P_92
1	97467	PASS	AC=1;	no	proban	yes	P_263
1	1039	PASS	AC=1;	no	proban	yes	P_224
1	1750	PASS	AC=1;	no	proban	yes	P_223
1	1510	PASS	AC=1;	no	proban	yes	P_223
0,999997	518	PASS	AC=1;	yes	proban	yes	P_132
0,724041	287	PASS	AC=1;	no	proban	yes	P_319
1	4572	PASS	AC=1;	no	proban	yes	P_192
0,999995	2099	PASS	AC=1;	no	proban	yes	P_273
1	2069	PASS	AC=1;	no	proban	yes	P_20
0,995461	2039	PASS	AC=1;	no	proban	yes	P_8
0,999995	1279	PASS	AC=1;	no	proban	yes	P_288
0,999996	4314	PASS	AC=1;	no	proban	yes	P_335
0,999999	2506	PASS	AC=1;	no	proban	yes	P_18
0,999997	520	PASS	AC=1;	no	proban	yes	P_314
0,698864	2666	PASS	AC=1;	yes	proban	yes	P_21
0,766198	2626	PASS	AC=1;	yes	proban	yes	P_120
0,999997	890	PASS	AC=1;	no	proban	yes	P_42
0,999999	575	PASS	AC=1;	no	proban	yes	P_416
0,999998	1154	PASS	AC=1;	no	proban	yes	P_228
0,999997	1042	PASS	AC=1;	no	proban	yes	P_271
1	687	PASS	AC=1;	no	proban	yes	P_224
00:00,0	1222	PASS	AC=1;	no	proban	yes	P_60
0,999997	780,1	PASS	AC=1;	no	proban	yes	P_101
0,999997	1502	PASS	AC=1;	no	proban	yes	P_390
0,999995	584	PASS	AC=1;	no	proban	yes	P_409
0,999994	1121	PASS	AC=1;	no	proban	yes	P_322
0,999999	1206	PASS	AC=1;	no	proban	yes	P_126
0,999996	948	PASS	AC=1;	no	proban	yes	P_80
0,999974	800,9	PASS	AC=2;	no	proban	yes	P_433
0,983607	60558	PASS	AC=1;	no	proban	yes	P_24

0,815262	59535	PASS	AC=1; no	proban yes	P_343
0,815262	57500	PASS	AC=1; no	proban yes	P_343
0:00:00	1373	PASS	AC=1; no	proban yes	P_78
0,999997	1231	PASS	AC=1; no	proban yes	P_425
1	1224	PASS	AC=1; no	proban yes	P_319
0,999997	948	PASS	AC=1; no	proban yes	P_192
0,999997	1938	PASS	AC=1; no	proban yes	P_329
1	2025	PASS	AC=1; no	proban yes	P_180
1	1272	PASS	AC=1; no	proban yes	P_22
0,999995	1156	PASS	AC=1; yes	proban yes	P_320
00:00,0	1072	PASS	AC=1; no	proban yes	P_74
1	1827	PASS	AC=1; no	proban yes	P_418
0,99522	34417	PASS	AC=1; no	proban yes	P_327
0,999997	2131	PASS	AC=1; no	proban yes	P_84
0,954193	530	PASS	AC=1; no	proban yes	P_373
1	1444	PASS	AC=1; no	proban yes	P_264
0,999997	1077	PASS	AC=1; no	proban yes	P_215
1	1743	PASS	AC=1; no	proban yes	P_262
1	1267	PASS	AC=1; no	proban yes	P_400
0,976153	476,1	PASS	AC=1; no	proban yes	P_362
0,999994	722	PASS	AC=1; no	proban yes	P_325
1	1009	PASS	AC=1; no	proban yes	P_304
0,999969	198	PASS	AC=1; no	proban yes	P_93
0,999996	1272	PASS	AC=1; no	proban yes	P_184
0,999807	502,1	PASS	AC=1; no	proban yes	P_134
1	3528	PASS	AC=1; no	proban yes	P_109
0,847462	970,3	PASS	AC=1; no	proban yes	P_276
0,999998	1122	PASS	AC=1; no	proban yes	P_294
0,999998	1832	PASS	AC=1; no	proban yes	P_253
0,999997	670	PASS	AC=1; no	proban yes	P_382
0,999998	2516	PASS	AC=1; no	proban yes	P_126
00:00,0	213	PASS	AC=1; no	proban yes	P_81
0,976514	395	PASS	AC=1; no	proban yes	P_125
0,999996	828	PASS	AC=1; no	proban yes	P_348
1	1983	PASS	AC=1; no	proban yes	P_116
0,999997	1874	PASS	AC=1; no	proban yes	P_329
0,717777	208,1	PASS	AC=1; no	proban yes	P_27
1	1601	PASS	AC=1; no	proban yes	P_55
0,999999	1559	PASS	AC=1; no	proban yes	P_177
0,999999	683,1	PASS	AC=1; no	proban yes	P_135
0,999999	2322	PASS	AC=1; no	proban yes	P_110
0,999999	1526	PASS	AC=1; no	proban yes	P_94
1	755	PASS	AC=1; no	proban yes	P_103
0,999995	1671	PASS	AC=1; no	proban yes	P_151
0,999997	514	PASS	AC=1; no	proban yes	P_105
0,999997	1104	PASS	AC=1; no	proban yes	P_29
0,999974	431	PASS	AC=1; no	proban yes	P_228
0,999996	532,3	PASS	AC=1; no	proban yes	P_10
0,990516	778,1	PASS	AC=1; no	proban yes	P_328
0,999997	897	PASS	AC=1; no	proban yes	P_216

0,999998	1432	PASS	AC=1;	no	proban yes	P_348
1	1686	PASS	AC=1;	no	proban yes	P_324
1	1772	PASS	AC=1;	no	proban yes	P_94
0,999997	504	PASS	AC=1;	no	proban yes	P_251
0,799488	5807	PASS	AC=1;	yes	proban yes	P_264
