# Supplementary material for: Genomic landscape of autism spectrum disorder in Brazil
Source: Genet Mol Biol. 2026 Sep 7;49(Suppl 1):e20250247. doi: 10.1590/1678-4685-GMB-2025-0247 (PMC13561403; doi:10.1590/1678-4685-GMB-2025-0247)
Supplement: Supplementary file 9 [file 1415-4757-GMB-49-s1-e20250247-s5.pdf]

Genetics and Molecular Biology
https://doi.org/10.1590/1678-4685-GMB-2025-0247

Supplementary Material to "Genomic landscape of autism spectrum disorder in Brazil"

Table S4 - Rare risk variants identified in all individuals included in the analysis (N = 1,295).

Chr	Start	End	Ref	Alt	Canonical.	Gene.refG	ExonicFun	AAChange	gnomAD_	gnomAD_z	OMIM_na	OMIM_nu	OMIM_dis
chr1	2055508	2055509	AT	-	NO	PRKCZ	frameshift	PRKCZ:NM	0,541	2,8049	Protein kin	176982	NA
chr1	2135263	2135266	ATCT	-	NO	PRKCZ	frameshift	PRKCZ:NM	0,541	2,8049	Protein kin	176982	NA
chr1	2306188	2306188	C	T	NO	SKI	stopgain	SKI:NM_00	0,99901	1,5143	SKI protoo	164780	Shprintzen
chr1	3730025	3730025	G	A	NO	TP73	nonsynony	TP73:NM_	0,99696	1,8527	p53-relate	601990	Ciliary dys
chr1	6082279	6082279	G	A	NO	KCNAB2	0	0	0,79978	2,5963	Potassium	601142	NA
chr1	6109790	6109790	C	T	NO	CHD5	0	0	1	5,3168	Chromodo	610771	Parenti-Mi
chr1	6143930	6143930	C	A	NO	CHD5	stopgain	CHD5:NM	1	5,3168	Chromodo	610771	Parenti-Mi
chr1	6146759	6146765	GGAGGGA	-	NO	CHD5	frameshift	CHD5:NM	1	5,3168	Chromodo	610771	Parenti-Mi
chr1	6234881	6234881	C	A	NO	ICMT	0	0	0,79377	2,4158	Isoprenylc	605851	NA
chr1	6281262	6281262	C	T	NO	ACOT7	nonsynony	ACOT7:NM	0,75926	1,9837	Acyl-CoA t	602587	NA
chr1	6667743	6667746	TCTC	-	NO	DNAJC11	frameshift	DNAJC11:	0,99893	1,8474	DNAJ/HSP	614827	NA
chr1	7969406	7969406	-	A	YES	PARK7	0	0	0,75335	0,16459	Oncogene	602533	Parkinson
chr1	8358584	8358593	CCGCTCTC	-	NO	RERE	frameshift	RERE:NM_	1	2,0321	Arginine-g	605226	Neurodeve
chr1	8358595	8358599	TCTCC	-	NO	RERE	frameshift	RERE:NM_	1	2,0321	Arginine-g	605226	Neurodeve
chr1	8792593	8792595	AAG	-	NO	RERE	0	0	1	2,0321	Arginine-g	605226	Neurodeve
chr1	9598557	9598557	C	T	NO	TMEM201	nonsynony	TMEM201	0,99004	1,3273	NA	NA	NA
chr1	9613079	9613079	G	A	NO	TMEM201	nonsynony	TMEM201	0,99004	1,3273	NA	NA	NA
chr1	9721592	9721592	C	T	NO	PIK3CD	0	0	0,99999	4,2747	Phosphati	602839	Immunode
chr1	9730605	9730605	G	A	NO	CLSTN1	nonsynony	CLSTN1:N	0,97757	1,0231	Calsynteni	611321	NA
chr1	10007630	10007633	AGAA	-	NO	RBP7	frameshift	RBP7:NM_	0,54133	1,0755	Retinol-bin	608604	NA
chr1	10106357	10106357	C	T	NO	UBE4B	stopgain	UBE4B:NM	1	3,726	Ubiquitina	613565	NA
chr1	10171306	10171306	G	A	NO	UBE4B	nonsynony	UBE4B:NM	1	3,726	Ubiquitina	613565	NA
chr1	10361776	10361776	C	T	NO	KIF1B	nonsynony	KIF1B:NM	1	3,599	Kinesin fa	605995	{Neurobla
chr1	10403089	10403089	G	T	NO	PGD	nonsynony	PGD:NM_	0,98788	1,3963	6-phospho	172200	NA
chr1	10639774	10639774	C	T	NO	CASZ1	nonsynony	CASZ1:NM	1	2,6057	Castor zinc	609895	NA
chr1	10649302	10649302	A	C	NO	CASZ1	nonsynony	CASZ1:NM	1	2,6057	Castor zinc	609895	NA
chr1	14960735	14960735	G	T	NO	KAZN	nonsynony	KAZN:NM_	0,59671	2,3109	Kazrin, per	618301	NA
chr1	15103378	15103378	C	-	NO	KAZN	frameshift	KAZN:NM_	0,59671	2,3109	Kazrin, per	618301	NA
chr1	15946317	15946317	C	G	YES	ZBTB17	0	0	0,97933	3,7316	Zinc finger	604084	NA
chr1	16315166	16315166	T	C	NO	FBXO42	0	0	0,99503	2,0948	F-box prot	609109	NA
chr1	17409196	17409196	-	G	YES	RCC2	0	0	0,95567	3,0895	Regulator	609587	NA
chr1	17413704	17413704	G	A	NO	RCC2	nonsynony	RCC2:NM_	0,95567	3,0895	Regulator	609587	NA
chr1	17422707	17422707	G	A	NO	RCC2	nonsynony	RCC2:NM_	0,95567	3,0895	Regulator	609587	NA

chr1	18910370	18910370	G	A	NO	IFFO2	nonsynony	IFFO2:NM	0,72617	2,4867	NA	NA	NA
chr1	19139167	19139167	G	A	NO	UBR4	nonsynony	UBR4:NM_	1	5,6833	Ubiquitin	609890	NA
chr1	21260309	21260309	C	A	NO	ECE1	stopgain	ECE1:NM_	0,95987	2,3066	Endothelin	600423	{Hyperten
chr1	21598451	21598451	T	C	NO	RAP1GAP	nonsynony	RAP1GAP:	0,84376	2,3001	RAP1, GTP	600278	NA
chr1	22908121	22908121	C	T	NO	EPHB2	nonsynony	EPHB2:NM	0,99997	2,4517	eph tyrosi	600997	?Bleeding
chr1	22910410	22910410	G	A	NO	EPHB2	nonsynony	EPHB2:NM	0,99997	2,4517	eph tyrosi	600997	?Bleeding
chr1	22910533	22910533	G	A	NO	EPHB2	nonsynony	EPHB2:NM	0,99997	2,4517	eph tyrosi	600997	?Bleeding
chr1	23055090	23055090	G	A	NO	KDM1A	nonsynony	KDM1A:N	0,99667	4,6784	Lysine dem	609132	Cleft palat
chr1	23081508	23081508	G	A	NO	KDM1A	nonsynony	KDM1A:N	0,99667	4,6784	Lysine dem	609132	Cleft palat
chr1	23093495	23093495	C	-	NO	LUZP1	frameshift	LUZP1:NM	0,52955	0,72117	Leucine zip	601422	NA
chr1	24347552	24347552	C	T	NO	GRHL3	nonsynony	GRHL3:NM	0,99292	1,4216	Grainyhea	608317	van der W
chr1	24652610	24652610	G	A	NO	SRRM1	nonsynony	SRRM1:NM	1	1,4554	Serine/arg	605975	NA
chr1	24929627	24929627	G	A	NO	RUNX3	nonsynony	RUNX3:NM	0,84295	2,6942	Runt-relat	600210	NA
chr1	26474569	26474569	C	T	NO	HMGN2	0	0	0,86341	3,09E-02	High-mobi	163910	NA
chr1	27113826	27113827	CA	-	NO	SLC9A1	frameshift	SLC9A1:N	0,79049	3,5504	Solute carr	107310	Lichtenste
chr1	27409698	27409698	G	A	NO	WASF2	nonsynony	WASF2:NM	0,97567	1,7698	WAS prote	605875	NA
chr1	27812257	27812257	C	T	NO	STX12	nonsynony	STX12:NM	0,95624	0,94585	Syntaxin 1	606892	NA
chr1	27914421	27914423	CCC	-	NO	RPA2	frameshift	RPA2:NM_	0,50461	0,77386	Replicatio	179836	NA
chr1	27997319	27997319	C	T	YES	EYA3	0	0	0,99702	1,9356	EYA transc	601655	NA
chr1	28710516	28710516	A	G	NO	GMEB1	0	0	0,98549	2,3665	Glucocorti	604409	NA
chr1	28737049	28737049	G	A	NO	YTHDF2	0	0	0,99943	2,2666	YTH N6-m	610640	NA
chr1	32222441	32222441	G	T	NO	EIF3I	0	0	0,9493	2,4625	Eukaryotic	603911	NA
chr1	32627775	32627775	A	G	YES	ZBTB8OS	0	0	0,70542	0,5925	Zinc finger	615891	NA
chr1	35269097	35269097	G	T	NO	ZMYM4	0	0	1	3,859	Zinc finger	613568	NA
chr1	35603379	35603380	AG	-	NO	PSMB2	0	0	0,91397	1,2561	Proteasom	602175	NA
chr1	36173438	36173438	G	T	NO	MAP7D1	nonsynony	MAP7D1:N	0,99199	1,0007	NA	NA	NA
chr1	36179268	36179268	G	A	NO	MAP7D1	nonsynony	MAP7D1:N	0,99199	1,0007	NA	NA	NA
chr1	36859958	36859958	-	TCTC	NO	GRIK3	frameshift	GRIK3:NM	0,99998	3,0056	Glutamate	138243	NA
chr1	39437792	39437792	G	A	NO	MACF1	nonsynony	MACF1:NM	1	3,4344	Microtubu	608271	Lissenceph
chr1	39452327	39452327	C	T	NO	MACF1	nonsynony	MACF1:NM	1	3,4344	Microtubu	608271	Lissenceph
chr1	39455035	39455035	C	T	NO	MACF1	nonsynony	MACF1:NM	1	3,4344	Microtubu	608271	Lissenceph
chr1	39461934	39461934	G	A	NO	MACF1	nonsynony	MACF1:NM	1	3,4344	Microtubu	608271	Lissenceph
chr1	39463619	39463619	G	C	NO	MACF1	nonsynony	MACF1:NM	1	3,4344	Microtubu	608271	Lissenceph
chr1	39479845	39479845	G	C	NO	MACF1	nonsynony	MACF1:NM	1	3,4344	Microtubu	608271	Lissenceph
chr1	39479909	39479909	G	A	NO	MACF1	nonsynony	MACF1:NM	1	3,4344	Microtubu	608271	Lissenceph
chr1	41510972	41510973	CT	-	NO	HIVEP3	frameshift	HIVEP3:N	0,90584	1,1744	Human im	606649	NA
chr1	41575571	41575571	G	A	NO	HIVEP3	nonsynony	HIVEP3:N	0,90584	1,1744	Human im	606649	NA

chr1	41583196	41583196	A	-	NO	HIVEP3	frameshift	HIVEP3:N	0,90584	1,1744	Human im	606649	NA
chr1	44636105	44636105	G	A	NO	RNF220	nonsynony	RNF220:N	0,99958	2,2243	RING finge	616136	Leukodyst
chr1	44776708	44776708	C	T	NO	RPS8	nonsynony	RPS8:NM_	0,99003	2,4175	Ribosomal	600357	NA
chr1	45618112	45618112	G	T	NO	NASP	stopgain	NASP:NM_	0,99553	0,6057	Nuclear au	603185	NA
chr1	46303124	46303124	C	T	NO	LRRC41	nonsynony	LRRC41:N	0,99986	3,0813	Leucine-ric	618753	NA
chr1	50705782	50705782	T	C	NO	FAF1	0	0	0,99895	2,1266	Fas-associ	604460	NA
chr1	51714080	51714080	G	A	YES	OSBPL9	0	0	0,97558	2,6559	Oxysterol-	606737	NA
chr1	51730005	51730005	A	T	NO	OSBPL9	0	0	0,97558	2,6559	Oxysterol-	606737	NA
chr1	53271350	53271350	G	C	NO	LRP8	0	0	0,99983	2,5202	Low densit	602600	{Myocardi
chr1	53327842	53327851	AGCAGCA	-	NO	LRP8	frameshift	LRP8:NM_	0,99983	2,5202	Low densit	602600	{Myocardi
chr1	53819083	53819083	-	G	NO	NDC1	0	0	0,99932	1,0942	Transmem	610115	NA
chr1	53894534	53894534	G	A	NO	DIO1	stopgain	DIO1:NM_	0,58399	0,47876	Deiodinas	147892	Thyroid ho
chr1	57062879	57062879	C	G	NO	DAB1	0	0	0,99915	1,5184	DAB adapt	603448	Spinocere
chr1	65408637	65408637	G	C	NO	DNAJC6	0	0	0,99986	1,6003	DNAJ heat	608375	Parkinson
chr1	65633151	65633151	G	A	YES	LEPR	0	0	0,99475	1,422	Leptin rec	601007	Obesity, m
chr1	67426159	67426159	T	C	NO	SERBP1	nonsynony	SERBP1:N	0,9995	1,6692	Serpine1 m	607378	NA
chr1	70053059	70053059	G	A	NO	LRRC7	nonsynony	LRRC7:NM	1	3,6044	Leucine-ric	614453	NA
chr1	84814107	84814107	C	-	NO	LPAR3	frameshift	LPAR3:NM	0,67935	0,2566	Lysophosp	605106	NA
chr1	84865430	84865430	G	A	NO	LPAR3	nonsynony	LPAR3:NM	0,67935	0,2566	Lysophosp	605106	NA
chr1	87331816	87331816	T	C	NO	LMO4	0	0	0,67667	2,1002	LIM domai	603129	NA
chr1	94174125	94174126	CT	-	NO	ARHGAP29	frameshift	ARHGAP29	0,99964	1,2111	RHO GTPa	610496	NA
chr1	96812856	96812856	T	C	NO	PTBP2	nonsynony	PTBP2:NM	0,99883	3,2024	Polypyrimi	608449	NA
chr1	1,03E+08	1,03E+08	T	A	NO	COL11A1	nonsynony	COL11A1:	1	1,0245	Collagen X	120280	Fibrochon
chr1	1,03E+08	1,03E+08	A	C	NO	COL11A1	0	0	1	1,0245	Collagen X	120280	Fibrochon
chr1	1,03E+08	1,03E+08	G	C	NO	COL11A1	nonsynony	COL11A1:	1	1,0245	Collagen X	120280	Fibrochon
chr1	1,09E+08	1,09E+08	CATGCGG	-	NO	CELSR2	stopgain	CELSR2:N	0,99994	2,176	Cadherin E	604265	NA
chr1	1,09E+08	1,09E+08	C	T	YES	SORT1	0	0	0,99987	2,1035	Sortilin (SO	602458	[Low densi
chr1	1,1E+08	1,1E+08	A	T	NO	CSF1	nonsynony	CSF1:NM_	0,99634	0,51416	Colony-sti	120420	NA
chr1	1,1E+08	1,1E+08	C	A	NO	SLC6A17	0	0	0,50353	2,3852	Solute carr	610299	Intellectua
chr1	1,1E+08	1,1E+08	G	T	NO	RBM15	nonsynony	RBM15:N	0,99474	1,7679	One-twent	606077	NA
chr1	1,1E+08	1,1E+08	-	CCGACGA	NO	RBM15	frameshift	RBM15:N	0,99474	1,7679	One-twent	606077	NA
chr1	1,13E+08	1,13E+08	G	T	NO	CAPZA1	nonsynony	CAPZA1:N	0,93292	1,2989	Capping pr	601580	NA
chr1	1,13E+08	1,13E+08	-	G	NO	RHOC	frameshift	RHOC:NM	0,76766	1,8491	RAS homo	165380	NA
chr1	1,17E+08	1,17E+08	G	T	YES	MAN1A2	0	0	0,99892	2,5642	Mannosid	604345	NA
chr1	1,2E+08	1,2E+08	G	A	NO	NOTCH2	stopgain	NOTCH2:N	1	3,5024	Notch rece	600275	Alagille sy
chr1	1,5E+08	1,5E+08	A	G	NO	RPRD2	0	0	1	2,1188	Regulation	614695	NA

chr1	1,51E+08	1,51E+08	G	A	NO	POGZ	nonsynony	POGZ:NM	1	3,5062	POGO tran	614787	White-Sut
chr1	1,52E+08	1,52E+08	G	A	NO	RORC	stopgain	RORC:NM	0,99985	2,2852	RAR-relate	602943	Immunode
chr1	1,55E+08	1,55E+08	-	GA	YES	UBE2Q1	0	0	0,99919	3,3549	Ubiquitin-	617429	NA
chr1	1,55E+08	1,55E+08	G	-	NO	KCNN3	frameshift	KCNN3:NM	0,97227	3,3258	Potassium	602983	Zimmerma
chr1	1,55E+08	1,55E+08	A	G	NO	CLK2	nonsynony	CLK2:NM_	0,99997	2,1973	CDC-like ki	602989	NA
chr1	1,55E+08	1,55E+08	G	A	NO	ASH1L	nonsynony	ASH1L:NM	1	3,4699	ASH1-like	607999	Intellectua
chr1	1,56E+08	1,56E+08	G	A	NO	ARHGEF2	nonsynony	ARHGEF2:	1	3,6056	RHO guani	607560	?Neurodev
chr1	1,56E+08	1,56E+08	G	A	NO	ARHGEF2	nonsynony	ARHGEF2:	1	3,6056	RHO guani	607560	?Neurodev
chr1	1,56E+08	1,56E+08	C	T	NO	UBQLN4	nonsynony	UBQLN4:N	0,98252	2,8804	Ubiquitin 4	605440	NA
chr1	1,56E+08	1,56E+08	G	A	NO	LMNA	0	0	0,9994	2,3673	Lamin A/C	150330	Mandibulo
chr1	1,56E+08	1,56E+08	T	C	NO	LMNA	0	0	0,9994	2,3673	Lamin A/C	150330	Mandibulo
chr1	1,56E+08	1,56E+08	CACT	-	NO	CCT3	stoploss	CCT3:NM_	0,99998	1,1592	Chaperoni	600114	NA
chr1	1,57E+08	1,57E+08	G	A	NO	ARHGEF11	nonsynony	ARHGEF11	1	1,6298	Rho guani	605708	NA
chr1	1,57E+08	1,57E+08	T	C	NO	ARHGEF11	nonsynony	ARHGEF11	1	1,6298	Rho guani	605708	NA
chr1	1,6E+08	1,6E+08	G	T	NO	COPA	0	0	1	3,558	Coatomer	601924	{Autoimm
chr1	1,6E+08	1,6E+08	G	A	NO	VANGL2	nonsynony	VANGL2:N	0,84283	1,4332	VANGL pla	600533	Neural tub
chr1	1,61E+08	1,61E+08	G	A	NO	DEDD	nonsynony	DEDD:NM	0,95912	2,2138	Death effe	606841	NA
chr1	1,61E+08	1,61E+08	G	A	NO	TOMM40L	nonsynony	TOMM40L	0,66713	1,4267	NA	NA	NA
chr1	1,62E+08	1,62E+08	A	G	NO	NOS1AP	nonsynony	NOS1AP:N	0,61424	2,0372	Nitric oxid	605551	Nephrotic
chr1	1,63E+08	1,63E+08	G	C	NO	UHMK1	nonsynony	UHMK1:N	0,83404	0,84423	Kinase-int	608849	NA
chr1	1,63E+08	1,63E+08	G	A	NO	DDR2	nonsynony	DDR2:NM	0,5503	2,2869	Discoidin d	191311	Warburg-C
chr1	1,66E+08	1,66E+08	A	G	NO	UCK2	0	0	0,72131	2,8122	Uridine/cy	609329	NA
chr1	1,67E+08	1,67E+08	T	C	NO	ILDR2	nonsynony	ILDR2:NM	0,95385	1,2559	Immunogl	618081	NA
chr1	1,74E+08	1,74E+08	-	A	NO	ZBTB37	frameshift	ZBTB37:N	0,95517	2,3551	NA	NA	NA
chr1	1,74E+08	1,74E+08	G	A	NO	RC3H1	nonsynony	RC3H1:NM	0,99309	2,7947	Roquin (RC	609424	?Immune
chr1	1,74E+08	1,74E+08	G	A	NO	RC3H1	nonsynony	RC3H1:NM	0,99309	2,7947	Roquin (RC	609424	?Immune
chr1	1,8E+08	1,8E+08	C	T	NO	CEP350	nonsynony	CEP350:N	1	1,3875	Centrosom	617870	NA
chr1	1,83E+08	1,83E+08	C	T	NO	DHX9	0	0	1	5,8433	DExH-box	603115	NA
chr1	1,83E+08	1,83E+08	G	A	NO	LAMC1	nonsynony	LAMC1:NM	1	1,5804	Laminin, g	150290	NA
chr1	1,83E+08	1,83E+08	A	-	NO	LAMC1	frameshift	LAMC1:NM	1	1,5804	Laminin, g	150290	NA
chr1	1,84E+08	1,84E+08	G	T	NO	RGL1	nonsynony	RGL1:NM_	0,95608	2,2238	Ral guanin	605667	NA
chr1	1,85E+08	1,85E+08	G	C	NO	TRMT1L	stopgain	TRMT1L:N	0,79653	1,8621	tRNA meth	611673	NA
chr1	1,85E+08	1,85E+08	G	A	NO	TRMT1L	0	0	0,79653	1,8621	tRNA meth	611673	NA
chr1	1,86E+08	1,86E+08	G	A	NO	TPR	nonsynony	TPR:NM_0	1	2,9916	Tumor pot	189940	?Intellectu
chr1	1,98E+08	1,98E+08	G	A	NO	LHX9	nonsynony	LHX9:NM_	0,98062	1,076	LIM home	606066	NA
chr1	2,02E+08	2,02E+08	C	T	NO	LMOD1	nonsynony	LMOD1:N	0,87057	1,1904	Leiomodin	602715	?Megacyst

chr1	2,03E+08	2,03E+08	TT	-	NO	MYOG	0	0	0,89602	0,55001	Myogenic	159980	NA
chr1	2,04E+08	2,04E+08	G	A	NO	SOX13	0	0	0,94185	1,741	SRY-box 1	604748	NA
chr1	2,04E+08	2,04E+08	G	A	NO	PIK3C2B	nonsynony	PIK3C2B:N	0,99651	2,5455	Phosphati	602838	NA
chr1	2,04E+08	2,04E+08	G	A	NO	PIK3C2B	nonsynony	PIK3C2B:N	0,99651	2,5455	Phosphati	602838	NA
chr1	2,04E+08	2,04E+08	G	A	NO	PIK3C2B	nonsynony	PIK3C2B:N	0,99651	2,5455	Phosphati	602838	NA
chr1	2,05E+08	2,05E+08	A	G	NO	NFASC	nonsynony	NFASC:NM	0,99999	2,5875	Neurofasci	609145	Neurodeve
chr1	2,05E+08	2,05E+08	T	A	NO	NFASC	stopgain	NFASC:NM	0,99999	2,5875	Neurofasci	609145	Neurodeve
chr1	2,05E+08	2,05E+08	G	A	NO	DSTYK	nonsynony	DSTYK:NM	0,51262	1,5861	Dual serin	612666	Spastic par
chr1	2,05E+08	2,05E+08	C	T	YES	DSTYK	0	0	0,51262	1,5861	Dual serin	612666	Spastic par
chr1	2,07E+08	2,07E+08	C	T	NO	MAPKAPK	nonsynony	MAPKAPK	0,99957	3,0509	Mitogen-a	602006	NA
chr1	2,11E+08	2,11E+08	G	C	NO	KCNH1	stopgain	KCNH1:NM	0,54625	3,8156	Potassium	603305	Zimmerma
chr1	2,12E+08	2,12E+08	G	A	NO	PPP2R5A	nonsynony	PPP2R5A:	0,81558	2,409	Protein ph	601643	NA
chr1	2,12E+08	2,12E+08	AGGTATG	-	NO	PPP2R5A	frameshift	PPP2R5A:	0,81558	2,409	Protein ph	601643	NA
chr1	2,2E+08	2,2E+08	G	A	NO	RAB3GAP2	nonsynony	RAB3GAP2	0,98674	0,86814	RAB3 GTPa	609275	Martsolf s
chr1	2,25E+08	2,25E+08	G	A	NO	CNIH3	nonsynony	CNIH3:NM	0,84533	0,68285	NA	NA	NA
chr1	2,27E+08	2,27E+08	C	T	YES	CDC42BPA	0	0	0,99309	2,3876	CDC42-bin	603412	NA
chr1	2,31E+08	2,31E+08	C	G	NO	TRIM67	0	0	0,96542	1,7402	Tripartite	610584	NA
chr1	2,37E+08	2,37E+08	C	T	NO	HEATR1	nonsynony	HEATR1:N	1	8,71E-02	HEAT repe	620390	NA
chr1	2,37E+08	2,37E+08	C	T	NO	HEATR1	nonsynony	HEATR1:N	1	8,71E-02	HEAT repe	620390	NA
chr1	2,37E+08	2,37E+08	G	A	NO	HEATR1	nonsynony	HEATR1:N	1	8,71E-02	HEAT repe	620390	NA
chr1	2,37E+08	2,37E+08	T	C	YES	ACTN2	0	0	0,99968	1,2925	Actinin, al	102573	Myopathy
chr1	2,37E+08	2,37E+08	G	A	NO	ACTN2	nonsynony	ACTN2:NM	0,99968	1,2925	Actinin, al	102573	Myopathy
chr1	2,38E+08	2,38E+08	C	T	NO	RYR2	nonsynony	RYR2:NM_	0,99999	5,7809	Ryanodine	180902	Ventricula
chr1	2,38E+08	2,38E+08	A	G	NO	RYR2	0	0	0,99999	5,7809	Ryanodine	180902	Ventricula
chr1	2,4E+08	2,4E+08	T	C	NO	FMN2	nonsynony	FMN2:NM	0,99772	0,31819	Formin 2 (	606373	Intellectua
chr1	2,43E+08	2,43E+08	T	C	NO	CEP170	nonsynony	CEP170:N	1	2,1974	Centrosom	613023	NA
chr1	2,46E+08	2,46E+08	T	C	NO	KIF26B	nonsynony	KIF26B:NM	0,99999	0,94702	Kinesin fa	614026	NA
chr1	2,46E+08	2,46E+08	G	C	NO	KIF26B	nonsynony	KIF26B:NM	0,99999	0,94702	Kinesin fa	614026	NA
chr1	2,47E+08	2,47E+08	G	A	NO	AHCTF1	nonsynony	AHCTF1:N	1	-0,3814	AT hook co	610853	NA
chr2	2284501	2284501	G	A	NO	MYT1L	0	0	1	4,7706	Myelin tra	613084	Intellectua
chr2	3580186	3580186	C	T	NO	RPS7	nonsynony	RPS7:NM_	0,95439	1,6271	Ribosomal	603658	Diamond-
chr2	3580208	3580208	G	C	NO	RPS7	nonsynony	RPS7:NM_	0,95439	1,6271	Ribosomal	603658	Diamond-
chr2	5693653	5693653	T	G	NO	SOX11	nonsynony	SOX11:NM	0,85844	2,2591	SRY-box 1	600898	Intellectua
chr2	8882557	8882557	G	A	NO	MBOAT2	nonsynony	MBOAT2:N	0,51629	1,7037	Membran	611949	NA
chr2	9391201	9391201	G	A	NO	ASAP2	0	0	0,99996	2,3661	Developm	603817	NA
chr2	9630397	9630397	T	C	NO	YWHAQ	nonsynony	YWHAQ:N	0,69755	1,6694	Tyrosine 3	609009	NA

chr2	10423088	10423088	G	A	NO	HPCAL1	nonsynony	HPCAL1:N	0,89103	2,4027	Hippocalci	600207	NA
chr2	15602546	15602546	A	G	YES	DDX1	0	0	0,99449	1,8179	DEAD-box	601257	NA
chr2	15620215	15620228	TTTGCTCT	-	NO	DDX1	frameshift	DDX1:NM	0,99449	1,8179	DEAD-box	601257	NA
chr2	20258234	20258234	T	A	NO	PUM2	0	0	0,99492	2,5887	Pumilio RN	607205	NA
chr2	20282991	20282992	AA	-	NO	PUM2	frameshift	PUM2:NM	0,99492	2,5887	Pumilio RN	607205	NA
chr2	23883593	23883593	C	-	YES	ATAD2B	0	0	1	3,9649	ATPase fa	615347	NA
chr2	25144969	25144969	G	A	NO	EFR3B	nonsynony	EFR3B:NM	2,41E-02	4,2618	EFR3 hom	616797	NA
chr2	26127238	26127238	A	G	NO	RAB10	0	0	0,97213	2,6317	Ras-associ	612672	NA
chr2	26848499	26848499	T	G	NO	DPYSL5	0	0	0,99991	3,0829	Dihydropy	608383	Ritscher-S
chr2	27223913	27223913	A	G	NO	CAD	0	0	0,99993	4,3365	CAD trifun	114010	Developm
chr2	27377808	27377808	G	A	NO	ZNF513	stopgain	ZNF513:N	0,81634	0,53015	Zinc finger	613598	?Retinitis p
chr2	27627067	27627067	G	A	NO	CCDC121	stopgain	CCDC121:	0,72977	0,55155	NA	NA	NA
chr2	27628631	27628631	T	A	NO	CCDC121	stopgain	CCDC121:	0,72977	0,55155	NA	NA	NA
chr2	28752033	28752033	T	C	NO	PPP1CB	0	0	0,99912	4,3319	Protein ph	600590	Noonan sy
chr2	28929594	28929594	C	T	NO	WDR43	nonsynony	WDR43:N	0,99997	0,7013	WD repeat	616195	NA
chr2	32477387	32477387	G	A	NO	BIRC6	nonsynony	BIRC6:NM	1	3,2081	Baculovira	605638	NA
chr2	32548017	32548017	A	G	NO	BIRC6	0	0	1	3,2081	Baculovira	605638	NA
chr2	36356625	36356625	-	A	YES	CRIM1	0	0	0,99971	0,19444	Cysteine-ri	606189	NA
chr2	36512349	36512349	G	A	NO	CRIM1	nonsynony	CRIM1:NM	0,99971	0,19444	Cysteine-ri	606189	NA
chr2	38582169	38582169	A	G	YES	HNRNPLL	0	0	0,91907	2,3619	Heterogen	611208	NA
chr2	38585640	38585640	-	T	NO	HNRNPLL	0	0	0,91907	2,3619	Heterogen	611208	NA
chr2	38751224	38751224	C	G	NO	SRSF7	0	0	0,98203	2,7201	Splicing fa	600572	NA
chr2	38989264	38989267	ACTA	-	NO	SOS1	0	0	1	3,0487	SOS Ras/R	182530	Noonan sy
chr2	38989315	38989315	C	T	YES	SOS1	0	0	1	3,0487	SOS Ras/R	182530	Noonan sy
chr2	44942814	44942814	G	A	NO	SIX3	nonsynony	SIX3:NM_0	0,94714	2,0739	SIX homeo	603714	Schizencep
chr2	45008615	45008615	T	C	NO	SIX2	nonsynony	SIX2:NM_0	0,93869	0,51896	SIX homeo	604994	NA
chr2	45008635	45008635	T	C	NO	SIX2	nonsynony	SIX2:NM_0	0,93869	0,51896	SIX homeo	604994	NA
chr2	47403319	47403319	A	G	NO	MSH2	nonsynony	MSH2:NM	0,89539	-2,4487	mutS hom	609309	Lynch synd
chr2	53887992	53887992	A	G	NO	PSME4	0	0	1	2,2691	Proteasom	607705	NA
chr2	53940003	53940003	G	C	NO	PSME4	0	0	1	2,2691	Proteasom	607705	NA
chr2	54622318	54622318	G	A	NO	SPTBN1	nonsynony	SPTBN1:N	1	4,5424	Spectrin, b	182790	Developm
chr2	55308874	55308874	T	C	NO	CCDC88A	nonsynony	CCDC88A:	1	2,3494	Coiled-coil	609736	?PEHO syn
chr2	55870721	55870721	C	T	NO	EFEMP1	nonsynony	EFEMP1:N	0,99992	1,8234	EGF-conta	601548	Doyne hon
chr2	61245287	61245287	A	G	NO	USP34	nonsynony	USP34:NM	1	2,3403	Ubiquitin-	615295	NA
chr2	61319280	61319280	C	T	NO	USP34	nonsynony	USP34:NM	1	2,3403	Ubiquitin-	615295	NA
chr2	69181795	69181795	G	T	NO	ANTXR1	nonsynony	ANTXR1:N	0,98631	1,5204	Anthrax to	606410	GAPO synd

chr2	70453313	70453313	C	T	NO	TGFA	nonsynony	TGFA:NM_	0,71981	0,75783	Transform	190170	NA
chr2	72132484	72132484	G	A	NO	CYP26B1	nonsynony	CYP26B1:N	0,97987	1,0492	Cytochrom	605207	Craniosyn
chr2	72135278	72135278	G	A	NO	CYP26B1	nonsynony	CYP26B1:N	0,97987	1,0492	Cytochrom	605207	Craniosyn
chr2	73260845	73260845	G	A	NO	FBXO41	nonsynony	FBXO41:N	0,99944	1,8463	F-box only	609108	NA
chr2	74457553	74457553	C	T	NO	INO80B	nonsynony	INO80B:N	0,75864	0,73116	INO80 com	616456	NA
chr2	74457730	74457730	G	-	NO	INO80B	frameshift	INO80B:N	0,75864	0,73116	INO80 com	616456	NA
chr2	84718215	84718215	G	A	NO	DNAH6	nonsynony	DNAH6:N	1,12E-36	3,5535	Dynein, ax	603336	NA
chr2	84813992	84813992	G	A	NO	DNAH6	nonsynony	DNAH6:N	1,12E-36	3,5535	Dynein, ax	603336	NA
chr2	96286311	96286311	C	T	NO	SNRNP200	nonsynony	SNRNP200	1	5,9363	Small nucl	601664	Retinitis pi
chr2	96304826	96304826	G	A	NO	SNRNP200	nonsynony	SNRNP200	1	5,9363	Small nucl	601664	Retinitis pi
chr2	98546050	98546050	G	T	NO	INPP4A	nonsynony	INPP4A:N	0,99952	3,3467	Inositol po	600916	NA
chr2	98548951	98548951	G	C	YES	INPP4A	0	0	0,99952	3,3467	Inositol po	600916	NA
chr2	98555774	98555774	-	C	NO	INPP4A	frameshift	INPP4A:N	0,99952	3,3467	Inositol po	600916	NA
chr2	99369484	99369484	G	A	NO	EIF5B	0	0	0,99981	2,6223	Eukaryotic	606086	NA
chr2	99390390	99390390	C	T	NO	EIF5B	stopgain	EIF5B:NM_	0,99981	2,6223	Eukaryotic	606086	NA
chr2	99451344	99451344	C	-	NO	REV1	0	0	0,99989	1,6026	REV1 DNA	606134	NA
chr2	1,01E+08	1,01E+08	TA	-	NO	NPAS2	frameshift	NPAS2:NM	1	2,5032	Neuronal P	603347	NA
chr2	1,02E+08	1,02E+08	G	A	NO	MAP4K4	nonsynony	MAP4K4:N	1	4,7404	Mitogen-a	604666	NA
chr2	1,09E+08	1,09E+08	G	A	NO	RANBP2	0	0	1	-0,77637	RAN-bindi	601181	{Encephal
chr2	1,11E+08	1,11E+08	C	T	NO	BCL2L11	stopgain	BCL2L11:N	0,88857	0,12116	BCL2-like 1	603827	NA
chr2	1,12E+08	1,12E+08	T	C	NO	ANAPC1	nonsynony	ANAPC1:N	0,94532	0,98694	Anaphase-	608473	Rothmund
chr2	1,13E+08	1,13E+08	G	A	NO	POLR1B	nonsynony	POLR1B:N	0,99946	2,9739	Polymeras	602000	Treacher-C
chr2	1,21E+08	1,21E+08	G	A	NO	GLI2	nonsynony	GLI2:NM_	0,97303	0,8168	GLI-Krupp	165230	Culler-Jon
chr2	1,21E+08	1,21E+08	G	A	NO	GLI2	nonsynony	GLI2:NM_	0,97303	0,8168	GLI-Krupp	165230	Culler-Jon
chr2	1,21E+08	1,21E+08	G	A	NO	CLASP1	nonsynony	CLASP1:N	1	3,6133	CLIP-assoc	605852	NA
chr2	1,25E+08	1,25E+08	G	A	NO	CNTNAP5	stopgain	CNTNAP5:	0,99167	0,38792	Contactin-	610519	NA
chr2	1,27E+08	1,27E+08	T	C	NO	IWS1	0	0	0,99727	2,3765	NA	NA	NA
chr2	1,28E+08	1,28E+08	G	A	NO	IWS1	stopgain	IWS1:NM_	0,99727	2,3765	NA	NA	NA
chr2	1,36E+08	1,36E+08	G	A	NO	R3HDM1	nonsynony	R3HDM1:N	0,86068	1,8465	R3H doma	619474	NA
chr2	1,36E+08	1,36E+08	G	A	NO	MCM6	nonsynony	MCM6:NM	0,97833	1,0681	Minichrom	601806	Lactase pe
chr2	1,48E+08	1,48E+08	G	A	NO	MBD5	nonsynony	MBD5:NM	1	0,85582	Methyl-Cp	611472	Intellectua
chr2	1,49E+08	1,49E+08	-	G	YES	KIF5C	0	0	1	4,0539	Kinesin fa	604593	Cortical dy
chr2	1,49E+08	1,49E+08	C	T	NO	KIF5C	nonsynony	KIF5C:NM	1	4,0539	Kinesin fa	604593	Cortical dy
chr2	1,51E+08	1,51E+08	A	G	NO	RIF1	0	0	1	-0,41404	Replicatio	608952	NA
chr2	1,51E+08	1,51E+08	G	A	NO	RIF1	nonsynony	RIF1:NM_	1	-0,41404	Replicatio	608952	NA
chr2	1,53E+08	1,53E+08	G	A	NO	PRPF40A	nonsynony	PRPF40A:N	3,91E-02	3,3321	Pre-mRNA	612941	NA

chr2	1,54E+08	1,54E+08	A	G	NO	GALNT13	0	0	0,99066	1,2685	UDP-N-ace	608369	NA
chr2	1,54E+08	1,54E+08	G	C	NO	GALNT13	nonsynony	GALNT13:	0,99066	1,2685	UDP-N-ace	608369	NA
chr2	1,54E+08	1,54E+08	A	G	NO	GALNT13	0	0	0,99066	1,2685	UDP-N-ace	608369	NA
chr2	1,59E+08	1,59E+08	C	T	NO	BAZ2B	nonsynony	BAZ2B:NM	0,98162	1,3314	Bromodom	605683	NA
chr2	1,59E+08	1,59E+08	G	A	NO	BAZ2B	stopgain	BAZ2B:NM	0,98162	1,3314	Bromodom	605683	NA
chr2	1,62E+08	1,62E+08	G	A	NO	SLC4A10	nonsynony	SLC4A10:N	0,13404	3,8352	Solute carr	605556	Neurodeve
chr2	1,65E+08	1,65E+08	T	C	NO	SCN3A	0	0	1	4,618	Sodium vo	182391	Epilepsy, f
chr2	1,66E+08	1,66E+08	T	A	NO	SCN1A	0	0	1	5,2206	Sodium vo	182389	Developm
chr2	1,69E+08	1,69E+08	T	C	NO	LRP2	nonsynony	LRP2:NM_	0,99999	2,0673	Low densit	600073	Donnai-Ba
chr2	1,69E+08	1,69E+08	C	A	NO	LRP2	nonsynony	LRP2:NM_	0,99999	2,0673	Low densit	600073	Donnai-Ba
chr2	1,69E+08	1,69E+08	ATGGTTTC	-	NO	LRP2	frameshift	LRP2:NM_	0,99999	2,0673	Low densit	600073	Donnai-Ba
chr2	1,71E+08	1,71E+08	C	T	NO	GORASP2	nonsynony	GORASP2:	0,88389	1,5927	Golgi reass	608693	NA
chr2	1,71E+08	1,71E+08	T	G	NO	TLK1	nonsynony	TLK1:NM_	0,99999	3,564	Tousled-lik	608438	NA
chr2	1,71E+08	1,71E+08	C	G	YES	TLK1	0	0	0,99999	3,564	Tousled-lik	608438	NA
chr2	1,72E+08	1,72E+08	G	A	NO	DLX2	nonsynony	DLX2:NM_	0,97847	0,94875	Distal-less	126255	NA
chr2	1,73E+08	1,73E+08	G	A	NO	RAPGEF4	nonsynony	RAPGEF4:	0,99218	1,8304	Rap guani	606058	NA
chr2	1,73E+08	1,73E+08	G	A	NO	RAPGEF4	nonsynony	RAPGEF4:	0,99218	1,8304	Rap guani	606058	NA
chr2	1,78E+08	1,78E+08	-	A	NO	AGPS	stopgain	AGPS:NM_	1	3,2294	Alkylglycer	603051	Rhizomelic
chr2	1,78E+08	1,78E+08	G	A	NO	OSBPL6	0	0	0,99999	1,7799	Oxysterol-	606734	NA
chr2	1,79E+08	1,79E+08	C	G	NO	SESTD1	nonsynony	SESTD1:N	0,99802	2,3071	NA	NA	NA
chr2	1,82E+08	1,82E+08	G	A	NO	NEUROD1	stopgain	NEUROD1:	0,77109	0,23293	Neurogeni	601724	{Type 2 dia
chr2	1,83E+08	1,83E+08	G	A	NO	NCKAP1	nonsynony	NCKAP1:N	1	4,2701	NCK-assoc	604891	NA
chr2	1,87E+08	1,87E+08	G	A	NO	CALCRL	nonsynony	CALCRL:N	0,9853	2,0933	Calcitonin	114190	?Lymphati
chr2	1,89E+08	1,89E+08	G	A	NO	COL5A2	nonsynony	COL5A2:N	1	2,4395	Collagen V	120190	Ehlers-Dan
chr2	1,91E+08	1,91E+08	-	A	NO	STAT1	0	0	0,99999	5,1492	Signal tran	600555	Immunode
chr2	1,96E+08	1,96E+08	A	G	NO	HECW2	0	0	0,9997	3,3052	HECT, C2,	617245	Neurodeve
chr2	1,97E+08	1,97E+08	TCTG	-	NO	HSPD1	frameshift	HSPD1:NM	0,99257	2,2563	Heat-shoc	118190	Spastic par
chr2	1,99E+08	1,99E+08	G	A	NO	SATB2	stopgain	SATB2:NM	0,99999	4,0511	Special AT	608148	Glass synd
chr2	2E+08	2E+08	T	-	NO	SPATS2L	frameshift	SPATS2L:N	0,55833	1,3115	Spermatog	613817	NA
chr2	2,06E+08	2,06E+08	G	T	NO	INO80D	0	0	0,99962	2,7432	INO80 com	619207	NA
chr2	2,07E+08	2,07E+08	G	T	NO	ADAM23	stopgain	ADAM23:N	0,99709	1,8992	ADAM me	603710	NA
chr2	2,1E+08	2,1E+08	G	A	NO	MAP2	nonsynony	MAP2:NM	1	0,88429	Microtubu	157130	NA
chr2	2,1E+08	2,1E+08	G	A	YES	MAP2	0	0	1	0,88429	Microtubu	157130	NA
chr2	2,1E+08	2,1E+08	G	A	NO	UNC80	nonsynony	UNC80:NM	5,23E-02	5,5319	Unc-80 ho	612636	Hypotonia
chr2	2,1E+08	2,1E+08	G	A	NO	UNC80	nonsynony	UNC80:NM	5,23E-02	5,5319	Unc-80 ho	612636	Hypotonia
chr2	2,1E+08	2,1E+08	-	T	NO	KANSL1L	frameshift	KANSL1L:N	0,65684	0,24511	KAT8 regul	613833	NA

chr2	2,1E+08	2,1E+08	C	A	NO	KANSL1L	nonsynony	KANSL1L:N	0,65684	0,24511	KAT8 regul	613833	NA
chr2	2,16E+08	2,16E+08	G	C	NO	XRCC5	nonsynony	XRCC5:NM	0,99999	2,8095	X-ray repa	194364	NA
chr2	2,18E+08	2,18E+08	G	A	NO	ARPC2	nonsynony	ARPC2:NM	0,99769	2,7043	Actin-relat	604224	NA
chr2	2,18E+08	2,18E+08	T	A	NO	USP37	nonsynony	USP37:NM	0,99999	2,464	Ubiquitin-	620226	NA
chr2	2,19E+08	2,19E+08	C	T	NO	SPEG	stopgain	SPEG:NM_	0,92315	2,7761	SPEG com	615950	Centronuc
chr2	2,19E+08	2,19E+08	C	T	NO	SPEG	nonsynony	SPEG:NM_	0,92315	2,7761	SPEG com	615950	Centronuc
chr2	2,19E+08	2,19E+08	C	T	NO	SPEG	stopgain	SPEG:NM_	0,92315	2,7761	SPEG com	615950	Centronuc
chr2	2,19E+08	2,19E+08	C	T	NO	SPEG	nonsynony	SPEG:NM_	0,92315	2,7761	SPEG com	615950	Centronuc
chr2	2,19E+08	2,19E+08	C	T	NO	SPEG	nonsynony	SPEG:NM_	0,92315	2,7761	SPEG com	615950	Centronuc
chr2	2,25E+08	2,25E+08	G	C	NO	CUL3	stopgain	CUL3:NM_	0,9996	4,7538	Cullin 3 (C	603136	Neurodeve
chr2	2,28E+08	2,28E+08	G	C	NO	AGFG1	0	0	0,99276	1,6605	ADP-ribos	600862	NA
chr2	2,28E+08	2,28E+08	C	T	NO	SPHKAP	stopgain	SPHKAP:N	0,93594	-0,75633	SPHK1 inte	611646	NA
chr2	2,28E+08	2,28E+08	T	C	NO	SPHKAP	0	0	0,93594	-0,75633	SPHK1 inte	611646	NA
chr2	2,3E+08	2,3E+08	A	T	NO	TRIP12	nonsynony	TRIP12:NM	1	4,6402	Thyroid ho	604506	Intellectua
chr2	2,3E+08	2,3E+08	G	A	NO	TRIP12	nonsynony	TRIP12:NM	1	4,6402	Thyroid ho	604506	Intellectua
chr2	2,31E+08	2,31E+08	G	A	NO	ITM2C	nonsynony	ITM2C:NM	0,70418	0,37992	Integral m	609554	NA
chr2	2,31E+08	2,31E+08	C	T	NO	ITM2C	nonsynony	ITM2C:NM	0,70418	0,37992	Integral m	609554	NA
chr2	2,31E+08	2,31E+08	-	T	NO	B3GNT7	frameshift	B3GNT7:N	0,70639	1,1902	Beta-1,3-N	615313	NA
chr2	2,31E+08	2,31E+08	A	G	NO	NCL	nonsynony	NCL:NM_0	0,99992	0,84885	Nucleolin (	164035	NA
chr2	2,32E+08	2,32E+08	TG	-	NO	DIS3L2	stopgain	DIS3L2:NM	0,944	0,94426	DIS3 like e	614184	Perlman sy
chr2	2,33E+08	2,33E+08	-	GC	NO	GIGYF2	frameshift	GIGYF2:N	1	2,244	GRB10-int	612003	{Parkinson
chr2	2,33E+08	2,33E+08	T	C	NO	ATG16L1	nonsynony	ATG16L1:N	0,96452	2,6338	Autophagy	610767	{Inflamma
chr2	2,36E+08	2,36E+08	A	G	NO	AGAP1	0	0	0,99984	1,2874	Arf GTPase	608651	NA
chr2	2,37E+08	2,37E+08	T	-	NO	COPS8	0	0	0,72485	0,91894	COP9 sign	616011	NA
chr2	2,38E+08	2,38E+08	T	A	NO	ILKAP	nonsynony	ILKAP:NM	0,98116	2,2097	ILK-associa	618909	NA
chr2	2,38E+08	2,38E+08	T	G	NO	PER2	0	0	0,7148	0,42662	Period circ	603426	?Advanced
chr2	2,41E+08	2,41E+08	G	A	NO	SNED1	nonsynony	SNED1:NM	0,66246	3,1323	SUSHI, NID	616634	NA
chr2	2,41E+08	2,41E+08	G	C	NO	SNED1	0	0	0,66246	3,1323	SUSHI, NID	616634	NA
chr2	2,41E+08	2,41E+08	G	A	NO	HDLBP	nonsynony	HDLBP:NM	1	4,0979	High-densi	142695	NA
chr3	3846238	3846238	C	T	NO	LRRN1	stopgain	LRRN1:NM	0,96996	0,3483	Leucine-ric	619623	NA
chr3	4691256	4691256	G	A	NO	ITPR1	nonsynony	ITPR1:NM	1	5,5951	Inositol 1,4	147265	Gillespie s
chr3	8985620	8985620	G	A	NO	SRGAP3	nonsynony	SRGAP3:N	0,9997	2,114	Slit-robo G	606525	NA
chr3	9013447	9013447	G	A	NO	SRGAP3	stopgain	SRGAP3:N	0,9997	2,114	Slit-robo G	606525	NA
chr3	9104702	9104702	T	C	NO	SRGAP3	nonsynony	SRGAP3:N	0,9997	2,114	Slit-robo G	606525	NA
chr3	9464667	9464667	T	G	NO	SETD5	nonsynony	SETD5:NM	0,99997	1,1326	SET domai	615743	Intellectua
chr3	12604200	12604200	G	C	NO	RAF1	nonsynony	RAF1:NM_	0,85295	2,4628	Oncogene	164760	Cardiomyo

chr3	14416408	14416408	A	G	NO	SLC6A6	0	0	0,98987	3,3854	Solute carr	186854	Hypotauri
chr3	15016180	15016180	G	A	NO	NR2C2	nonsynony	NR2C2:NM	0,99892	3,1793	Nuclear re	601426	NA
chr3	15714661	15714661	G	C	NO	ANKRD28	0	0	1	2,3872	Ankyrin re	611122	NA
chr3	15714661	15714661	G	T	NO	ANKRD28	0	0	1	2,3872	Ankyrin re	611122	NA
chr3	23921607	23921608	CA	-	NO	RPL15	frameshift	RPL15:NM	0,97146	1,8348	Ribosomal	604174	Diamond-
chr3	27394756	27394756	T	C	NO	SLC4A7	nonsynony	SLC4A7:N	0,73616	2,2455	Solute carr	603353	NA
chr3	27418637	27418637	G	C	NO	SLC4A7	0	0	0,73616	2,2455	Solute carr	603353	NA
chr3	27721932	27721932	G	-	NO	EOMES	frameshift	EOMES:N	0,98443	0,86068	Eomesode	604615	NA
chr3	33608625	33608625	G	A	NO	CLASP2	nonsynony	CLASP2:N	1	3,872	CLIP-assoc	605853	NA
chr3	33866510	33866510	C	A	NO	PDCD6IP	stopgain	PDCD6IP:N	0,70554	0,97635	Programm	608074	?Microcep
chr3	38165916	38165917	AG	-	NO	OXSR1	frameshift	OXSR1:NM	0,77444	2,6028	Oxidative	604046	NA
chr3	38598966	38598966	G	A	NO	SCN5A	nonsynony	SCN5A:NM	0,91291	2,7504	Sodium ch	600163	Ventricula
chr3	38622494	38622494	G	T	NO	SCN5A	0	0	0,91291	2,7504	Sodium ch	600163	Ventricula
chr3	40462062	40462063	AA	-	NO	RPL14	frameshift	RPL14:NM	0,89508	0,35716	Ribosomal	617414	NA
chr3	43347955	43347955	C	T	NO	SNRK	stopgain	SNRK:NM_	0,99961	3,4703	SNF-relate	612760	NA
chr3	45713197	45713198	GT	-	NO	SACM1L	0	0	0,69536	1,9903	SAC1 supp	606569	NA
chr3	46901860	46901860	G	A	NO	PTH1R	nonsynony	PTH1R:NM	0,57253	1,6514	Parathyroi	168468	Metaphys
chr3	46903270	46903270	G	A	NO	PTH1R	nonsynony	PTH1R:NM	0,57253	1,6514	Parathyroi	168468	Metaphys
chr3	47062158	47062158	-	T	NO	SETD2	0	0	1	3,0459	SET domai	612778	Luscan-Lu
chr3	47678297	47678297	C	T	NO	SMARCC1	nonsynony	SMARCC1:	1	2,4466	SWI/SNF r	601732	{Hydrocep
chr3	47870844	47870844	T	C	NO	MAP4	nonsynony	MAP4:NM	0,84782	0,69035	Microtubu	157132	NA
chr3	47921765	47921765	C	T	NO	MAP4	nonsynony	MAP4:NM	0,84782	0,69035	Microtubu	157132	NA
chr3	48412596	48412596	G	A	NO	PLXNB1	nonsynony	PLXNB1:N	3,83E-04	3,2333	Plexin B1 (	601053	NA
chr3	48642010	48642010	C	T	YES	CELSR3	0	0	0,99998	4,0766	Cadherin E	604264	NA
chr3	48645795	48645795	G	A	NO	CELSR3	nonsynony	CELSR3:N	0,99998	4,0766	Cadherin E	604264	NA
chr3	48651355	48651355	G	A	NO	CELSR3	0	0	0,99998	4,0766	Cadherin E	604264	NA
chr3	48981633	48981633	G	C	YES	ARIH2	0	0	0,99374	3,5771	Ariadne RB	605615	NA
chr3	49109190	49109190	G	T	NO	USP19	0	0	0,99999	1,9896	Ubiquitin-	614471	NA
chr3	49111957	49111957	G	A	NO	USP19	nonsynony	USP19:NM	0,99999	1,9896	Ubiquitin-	614471	NA
chr3	49119017	49119017	C	T	NO	USP19	0	0	0,99999	1,9896	Ubiquitin-	614471	NA
chr3	50061957	50061957	C	T	NO	RBM6	0	0	1	2,1708	RNA-bindi	606886	NA
chr3	50257553	50257553	-	TTTTTAA	NO	GNAI2	frameshift	GNAI2:NM	0,89194	3,1524	Guanine n	139360	Ventricula
chr3	50367445	50367445	G	A	NO	CACNA2D2	nonsynony	CACNA2D2	0,99997	2,9999	Calcium ch	607082	Cerebellar
chr3	51214170	51214170	G	A	NO	DOCK3	nonsynony	DOCK3:NM	1	3,9356	Dedicator	603123	Neurodeve
chr3	51338360	51338360	G	C	NO	DOCK3	nonsynony	DOCK3:NM	1	3,9356	Dedicator	603123	Neurodeve
chr3	51374486	51374486	G	T	NO	DOCK3	nonsynony	DOCK3:NM	1	3,9356	Dedicator	603123	Neurodeve

chr3	52480309	52480309	T	-	NO	NISCH	stopgain	NISCH:NM	0,99443	2,2993	Nischarin (	615507	NA
chr3	53722325	53722325	G	A	NO	CACNA1D	nonsynony	CACNA1D:	1	4,5817	Calcium ch	114206	Primary al
chr3	53723631	53723631	C	T	NO	CACNA1D	nonsynony	CACNA1D:	1	4,5817	Calcium ch	114206	Primary al
chr3	54899872	54899872	A	T	NO	CACNA2D3	0	0	0,57778	1,863	Calcium ch	606399	NA
chr3	55074131	55074131	T	G	NO	CACNA2D3	nonsynony	CACNA2D3	0,57778	1,863	Calcium ch	606399	NA
chr3	55683855	55683855	T	-	NO	ERC2	frameshift	ERC2:NM_	0,98931	2,2244	ELKS/RAB6	617250	NA
chr3	56010445	56010445	T	C	NO	ERC2	0	0	0,98931	2,2244	ELKS/RAB6	617250	NA
chr3	57268430	57268431	AG	-	NO	APPL1	frameshift	APPL1:NM	0,7952	0,71635	Adaptor pr	604299	{Maturity-
chr3	57861950	57861950	G	A	NO	SLMAP	nonsynony	SLMAP:NM	0,99976	1,7012	Sarcolemm	602701	NA
chr3	57925935	57925935	G	A	YES	SLMAP	0	0	0,99976	1,7012	Sarcolemm	602701	NA
chr3	63912711	63912712	AG	-	NO	ATXN7	frameshift	ATXN7:NM	0,9595	-0,62346	Ataxin 7 (A	607640	Spinocere
chr3	63912714	63912714	A	-	NO	ATXN7	frameshift	ATXN7:NM	0,9595	-0,62346	Ataxin 7 (A	607640	Spinocere
chr3	63913154	63913154	T	C	NO	ATXN7	0	0	0,9595	-0,62346	Ataxin 7 (A	607640	Spinocere
chr3	63997632	63997632	A	-	NO	ATXN7	frameshift	ATXN7:NM	0,9595	-0,62346	Ataxin 7 (A	607640	Spinocere
chr3	65478624	65478624	T	C	NO	MAGI1	nonsynony	MAGI1:NM	0,5597	1,3637	Membran	602625	NA
chr3	69064098	69064098	T	A	NO	UBA3	nonsynony	UBA3:NM	0,50413	1,1653	Ubiquitin-l	603172	NA
chr3	69080346	69080346	T	C	NO	UBA3	nonsynony	UBA3:NM	0,50413	1,1653	Ubiquitin-l	603172	NA
chr3	70972667	70972667	G	A	NO	FOXP1	nonsynony	FOXP1:NM	0,99998	2,2759	Forkhead	605515	Intellectua
chr3	71046957	71046957	G	A	NO	FOXP1	stopgain	FOXP1:NM	0,99998	2,2759	Forkhead	605515	Intellectua
chr3	77477498	77477498	G	A	NO	ROBO2	nonsynony	ROBO2:NM	1	1,6495	Roundabo	602431	Vesicouret
chr3	97646234	97646234	G	A	NO	EPHA6	stopgain	EPHA6:NM	0,67037	1,5033	Ephrin rec	600066	NA
chr3	1,06E+08	1,06E+08	G	A	NO	CBLB	nonsynony	CBLB:NM_	0,99825	1,2942	CAS-BR-M	604491	Autoimmu
chr3	1,06E+08	1,06E+08	G	A	NO	CBLB	nonsynony	CBLB:NM_	0,99825	1,2942	CAS-BR-M	604491	Autoimmu
chr3	1,08E+08	1,08E+08	T	A	NO	BBX	stopgain	BBX:NM_0	0,51752	1,0908	NA	NA	NA
chr3	1,16E+08	1,16E+08	C	T	NO	LSAMP	nonsynony	LSAMP:NM	0,98742	1,5698	Limbic syst	603241	NA
chr3	1,16E+08	1,16E+08	-	A	YES	LSAMP	0	0	0,98742	1,5698	Limbic syst	603241	NA
chr3	1,2E+08	1,2E+08	C	T	NO	FSTL1	nonsynony	FSTL1:NM	0,89403	0,7142	Follistatin-	605547	NA
chr3	1,23E+08	1,23E+08	A	G	NO	ADCY5	nonsynony	ADCY5:NM	0,99928	3,4043	Adenylate	600293	Dyskinesia
chr3	1,25E+08	1,25E+08	G	A	NO	KALRN	nonsynony	KALRN:NM	1	4,3604	Kalirin (KA	604605	NA
chr3	1,27E+08	1,27E+08	G	A	NO	PLXNA1	nonsynony	PLXNA1:N	0,99951	3,4482	Plexin A1 (	601055	Dworschak
chr3	1,27E+08	1,27E+08	T	G	NO	PLXNA1	0	0	0,99951	3,4482	Plexin A1 (	601055	Dworschak
chr3	1,27E+08	1,27E+08	G	T	NO	PLXNA1	nonsynony	PLXNA1:N	0,99951	3,4482	Plexin A1 (	601055	Dworschak
chr3	1,27E+08	1,27E+08	G	A	NO	PLXNA1	nonsynony	PLXNA1:N	0,99951	3,4482	Plexin A1 (	601055	Dworschak
chr3	1,28E+08	1,28E+08	A	G	NO	PODXL2	nonsynony	PODXL2:N	0,99936	0,90734	Podocalyxi	616627	NA
chr3	1,29E+08	1,29E+08	G	A	NO	ISY1;ISY1-R	nonsynony	ISY1:NM_0	9.9990e-0	1.2445e+0	ISY1 splicin	612764|	NA
chr3	1,3E+08	1,3E+08	G	T	NO	PLXND1	0	0	1	3,3465	Plexin D1 (	604282	Congenital

<table>
<tr><td>chr3</td><td>1,3E+08</td><td>1,3E+08</td><td>C</td><td>G</td><td>NO</td><td>PLXND1</td><td>0</td><td>0</td><td>1</td><td>3,3465</td><td>Plexin D1 (</td><td>604282</td><td>Congenital</td></tr>
<tr><td>chr3</td><td>1,31E+08</td><td>1,31E+08</td><td>C</td><td>-</td><td>NO</td><td>ASTE1;ATP</td><td>frameshift</td><td>ASTE1:NM</td><td>7.2103e-1</td><td>8.6288e-0</td><td>Asteroid h</td><td>620693|60|Hailey-Ha</td><td></td></tr>
<tr><td>chr3</td><td>1,32E+08</td><td>1,32E+08</td><td>G</td><td>A</td><td>NO</td><td>DNAJC13</td><td>nonsynony</td><td>DNAJC13:</td><td>1</td><td>2,2836</td><td>DNAJ/HSP</td><td>614334</td><td>NA</td></tr>
<tr><td>chr3</td><td>1,33E+08</td><td>1,33E+08</td><td>G</td><td>T</td><td>NO</td><td>DNAJC13</td><td>stopgain</td><td>DNAJC13:</td><td>1</td><td>2,2836</td><td>DNAJ/HSP</td><td>614334</td><td>NA</td></tr>
<tr><td>chr3</td><td>1,34E+08</td><td>1,34E+08</td><td>G</td><td>A</td><td>NO</td><td>CDV3</td><td>nonsynony</td><td>CDV3:NM_</td><td>0,76817</td><td>0,7649</td><td>CDV3 hom</td><td>618789</td><td>NA</td></tr>
<tr><td>chr3</td><td>1,34E+08</td><td>1,34E+08</td><td>G</td><td>A</td><td>NO</td><td>TOPBP1</td><td>0</td><td>0</td><td>1</td><td>1,971</td><td>DNA topoi</td><td>607760</td><td>NA</td></tr>
<tr><td>chr3</td><td>1,38E+08</td><td>1,38E+08</td><td>TCAAAGAC</td><td>-</td><td>NO</td><td>MRAS</td><td>frameshift</td><td>MRAS:NM</td><td>0,92111</td><td>2,3268</td><td>Muscle Ra</td><td>608435</td><td>Noonan sy</td></tr>
<tr><td>chr3</td><td>1,5E+08</td><td>1,5E+08</td><td>T</td><td>G</td><td>NO</td><td>WWTR1</td><td>0</td><td>0</td><td>0,93914</td><td>1,1165</td><td>WW doma</td><td>607392</td><td>NA</td></tr>
<tr><td>chr3</td><td>1,51E+08</td><td>1,51E+08</td><td>G</td><td>A</td><td>NO</td><td>MED12L</td><td>nonsynony</td><td>MED12L:N</td><td>1</td><td>3,132</td><td>Mediator c</td><td>611318</td><td>Nizon-Isid</td></tr>
<tr><td>chr3</td><td>1,56E+08</td><td>1,56E+08</td><td>G</td><td>A</td><td>NO</td><td>GMPS</td><td>nonsynony</td><td>GMPS:NM</td><td>0,99825</td><td>2,3686</td><td>Guanine m</td><td>600358</td><td>NA</td></tr>
<tr><td>chr3</td><td>1,69E+08</td><td>1,69E+08</td><td>C</td><td>T</td><td>NO</td><td>MECOM</td><td>nonsynony</td><td>MECOM:N</td><td>1</td><td>1,6191</td><td>MDS1 and</td><td>165215</td><td>Radioulnar</td></tr>
<tr><td>chr3</td><td>1,71E+08</td><td>1,71E+08</td><td>G</td><td>A</td><td>NO</td><td>TNIK</td><td>stopgain</td><td>TNIK:NM_</td><td>1</td><td>4,1047</td><td>TRAF2-and</td><td>610005</td><td>Intellectua</td></tr>
<tr><td>chr3</td><td>1,82E+08</td><td>1,82E+08</td><td>G</td><td>T</td><td>NO</td><td>SOX2</td><td>nonsynony</td><td>SOX2:NM_</td><td>0,70662</td><td>2,1215</td><td>SRY (sex d</td><td>184429</td><td>Optic nerv</td></tr>
<tr><td>chr3</td><td>1,84E+08</td><td>1,84E+08</td><td>A</td><td>T</td><td>NO</td><td>YEATS2</td><td>nonsynony</td><td>YEATS2:N</td><td>0,99639</td><td>0,97161</td><td>YEATS dom</td><td>613373</td><td>?Epilepsy,</td></tr>
<tr><td>chr3</td><td>1,84E+08</td><td>1,84E+08</td><td>C</td><td>T</td><td>NO</td><td>PSMD2</td><td>nonsynony</td><td>PSMD2:N</td><td>1</td><td>3,0867</td><td>Proteasom</td><td>606223</td><td>NA</td></tr>
<tr><td>chr3</td><td>1,84E+08</td><td>1,84E+08</td><td>C</td><td>T</td><td>NO</td><td>EIF4G1</td><td>nonsynony</td><td>EIF4G1:NM</td><td>1</td><td>2,2233</td><td>Eukaryotic</td><td>600495</td><td>{Parkinson</td></tr>
<tr><td>chr3</td><td>1,94E+08</td><td>1,94E+08</td><td>A</td><td>T</td><td>NO</td><td>OPA1</td><td>nonsynony</td><td>OPA1:NM</td><td>0,98636</td><td>1,9741</td><td>OPA1 mito</td><td>605290</td><td>Optic atro</td></tr>
<tr><td>chr3</td><td>1,94E+08</td><td>1,94E+08</td><td>C</td><td>T</td><td>NO</td><td>OPA1</td><td>nonsynony</td><td>OPA1:NM</td><td>0,98636</td><td>1,9741</td><td>OPA1 mito</td><td>605290</td><td>Optic atro</td></tr>
<tr><td>chr3</td><td>1,94E+08</td><td>1,94E+08</td><td>G</td><td>A</td><td>NO</td><td>OPA1</td><td>nonsynony</td><td>OPA1:NM</td><td>0,98636</td><td>1,9741</td><td>OPA1 mito</td><td>605290</td><td>Optic atro</td></tr>
<tr><td>chr3</td><td>1,94E+08</td><td>1,94E+08</td><td>G</td><td>A</td><td>NO</td><td>ATP13A3</td><td>nonsynony</td><td>ATP13A3:N</td><td>0,99998</td><td>3,0145</td><td>ATPase 13</td><td>610232</td><td>Pulmonary</td></tr>
<tr><td>chr3</td><td>1,97E+08</td><td>1,97E+08</td><td>G</td><td>A</td><td>NO</td><td>DLG1</td><td>nonsynony</td><td>DLG1:NM_</td><td>0,99393</td><td>1,4957</td><td>Discs large</td><td>601014</td><td>NA</td></tr>
<tr><td>chr4</td><td>737524</td><td>737524</td><td>G</td><td>T</td><td>NO</td><td>PCGF3</td><td>0</td><td>0</td><td>0,95326</td><td>2,1015</td><td>Polycomb</td><td>617543</td><td>NA</td></tr>
<tr><td>chr4</td><td>2926043</td><td>2926043</td><td>-</td><td>A</td><td>NO</td><td>ADD1</td><td>frameshift</td><td>ADD1:NM</td><td>0,9883</td><td>0,80446</td><td>Adducin-1</td><td>102680</td><td>{Hyperten</td></tr>
<tr><td>chr4</td><td>2928470</td><td>2928470</td><td>A</td><td>T</td><td>NO</td><td>ADD1</td><td>stopgain</td><td>ADD1:NM</td><td>0,9883</td><td>0,80446</td><td>Adducin-1</td><td>102680</td><td>{Hyperten</td></tr>
<tr><td>chr4</td><td>3074906</td><td>3074907</td><td>GC</td><td>-</td><td>NO</td><td>HTT</td><td>frameshift</td><td>HTT:NM_0</td><td>1</td><td>2,7799</td><td>Huntingtin</td><td>613004</td><td>Lopes-Mac</td></tr>
<tr><td>chr4</td><td>3074909</td><td>3074927</td><td>GCAGCAG</td><td>-</td><td>NO</td><td>HTT</td><td>frameshift</td><td>HTT:NM_0</td><td>1</td><td>2,7799</td><td>Huntingtin</td><td>613004</td><td>Lopes-Mac</td></tr>
<tr><td>chr4</td><td>3074912</td><td>3074913</td><td>GC</td><td>-</td><td>NO</td><td>HTT</td><td>frameshift</td><td>HTT:NM_0</td><td>1</td><td>2,7799</td><td>Huntingtin</td><td>613004</td><td>Lopes-Mac</td></tr>
<tr><td>chr4</td><td>3074915</td><td>3074927</td><td>GCAGCAG</td><td>-</td><td>NO</td><td>HTT</td><td>frameshift</td><td>HTT:NM_0</td><td>1</td><td>2,7799</td><td>Huntingtin</td><td>613004</td><td>Lopes-Mac</td></tr>
<tr><td>chr4</td><td>3074927</td><td>3074928</td><td>GC</td><td>-</td><td>NO</td><td>HTT</td><td>frameshift</td><td>HTT:NM_0</td><td>1</td><td>2,7799</td><td>Huntingtin</td><td>613004</td><td>Lopes-Mac</td></tr>
<tr><td>chr4</td><td>3074930</td><td>3074930</td><td>G</td><td>-</td><td>NO</td><td>HTT</td><td>frameshift</td><td>HTT:NM_0</td><td>1</td><td>2,7799</td><td>Huntingtin</td><td>613004</td><td>Lopes-Mac</td></tr>
<tr><td>chr4</td><td>3074938</td><td>3074938</td><td>-</td><td>CCGCC</td><td>NO</td><td>HTT</td><td>frameshift</td><td>HTT:NM_0</td><td>1</td><td>2,7799</td><td>Huntingtin</td><td>613004</td><td>Lopes-Mac</td></tr>
<tr><td>chr4</td><td>3105421</td><td>3105421</td><td>G</td><td>A</td><td>NO</td><td>HTT</td><td>nonsynony</td><td>HTT:NM_0</td><td>1</td><td>2,7799</td><td>Huntingtin</td><td>613004</td><td>Lopes-Mac</td></tr>
<tr><td>chr4</td><td>3249265</td><td>3249265</td><td>-</td><td>CTCACCCC</td><td>NO</td><td>MSANTD1</td><td>frameshift</td><td>MSANTD1</td><td>0,80456</td><td>0,74585</td><td>NA</td><td>NA</td><td>NA</td></tr>
<tr><td>chr4</td><td>3767780</td><td>3767780</td><td>G</td><td>A</td><td>NO</td><td>ADRA2C</td><td>nonsynony</td><td>ADRA2C:N</td><td>0,50159</td><td>1,6407</td><td>Adrenergic</td><td>104250</td><td>{Congestiv</td></tr>
<tr><td>chr4</td><td>7054133</td><td>7054133</td><td>C</td><td>A</td><td>NO</td><td>TADA2B</td><td>stopgain</td><td>TADA2B:N</td><td>0,72366</td><td>2,7966</td><td>Transcripti</td><td>608790</td><td>NA</td></tr>
<tr><td>chr4</td><td>8868236</td><td>8868315</td><td>CGCCAGCT</td><td>-</td><td>NO</td><td>HMX1</td><td>frameshift</td><td>HMX1:NM</td><td>0,72623</td><td>-0,10187</td><td>Homeobox</td><td>142992</td><td>Oculoauric</td></tr>
</table>

chr4	20524169	20524169	G	A	NO	SLIT2	nonsynony	SLIT2:NM_	1	2,0993	SLIT guida	603746	NA
chr4	39838052	39838052	G	A	NO	PDS5A	nonsynony	PDS5A:NM	1	5,0494	PDS5 cohe	613200	NA
chr4	41613094	41613094	-	G	NO	LIMCH1	frameshift	LIMCH1:N	0,94734	0,52339	LIM and ca	617750	NA
chr4	41619188	41619188	G	A	NO	LIMCH1	nonsynony	LIMCH1:N	0,94734	0,52339	LIM and ca	617750	NA
chr4	41633788	41633788	T	-	NO	LIMCH1	frameshift	LIMCH1:N	0,94734	0,52339	LIM and ca	617750	NA
chr4	41662989	41662989	A	G	NO	LIMCH1	0	0	0,94734	0,52339	LIM and ca	617750	NA
chr4	41670786	41670786	C	T	NO	LIMCH1	stopgain	LIMCH1:N	0,94734	0,52339	LIM and ca	617750	NA
chr4	47032412	47032412	G	A	NO	GABRB1	0	0	0,98116	2,6517	Gamma-a	137190	Developm
chr4	48420123	48420123	A	G	YES	SLAIN2	0	0	0,79312	0,71968	SLAIN mot	610492	NA
chr4	52658284	52658284	A	G	NO	USP46	0	0	0,79135	2,8667	Ubiquitin-	612849	NA
chr4	53459476	53459480	TAGTA	-	NO	FIP1L1	frameshift	FIP1L1:NM	0,7889	3,0652	Fip1-like 1	607686	NA
chr4	54289119	54289119	G	A	NO	PDGFRA	0	0	1	1,9401	Platelet-de	173490	Gastrointe
chr4	81456671	81456671	G	A	NO	RASGEF1B	stopgain	RASGEF1B	0,92073	1,474	RASGEF do	614532	NA
chr4	82970472	82970472	G	A	NO	LIN54	0	0	0,99994	2,6985	lin-54 DRE	613367	NA
chr4	84702385	84702385	T	C	NO	WDFY3	nonsynony	WDFY3:N	1	5,8174	WD repeat	617485	?Microcep
chr4	84721549	84721549	G	A	NO	WDFY3	nonsynony	WDFY3:N	1	5,8174	WD repeat	617485	?Microcep
chr4	84774985	84774985	T	C	NO	WDFY3	0	0	1	5,8174	WD repeat	617485	?Microcep
chr4	86101052	86101052	C	T	NO	MAPK10	nonsynony	MAPK10:N	0,27553	3,0399	Mitogen-a	602897	NA
chr4	95131511	95131511	A	G	NO	BMPR1B	nonsynony	BMPR1B:N	0,99863	0,27016	Bone morp	603248	Acromeso
chr4	1,03E+08	1,03E+08	A	G	NO	NFKB1	0	0	1	3,1933	Nuclear fa	164011	Immunode
chr4	1,06E+08	1,06E+08	-	A	NO	INTS12	frameshift	INTS12:NM	0,7537	1,347	Integrator	611355	NA
chr4	1,06E+08	1,06E+08	A	G	YES	INTS12	0	0	0,7537	1,347	Integrator	611355	NA
chr4	1,1E+08	1,1E+08	C	T	NO	ELOVL6	0	0	0,96581	3,0411	Elongation	611546	NA
chr4	1,13E+08	1,13E+08	G	A	NO	ANK2	nonsynony	ANK2:NM_	1	1,9641	Ankyrin-2,	106410	Long QT sy
chr4	1,13E+08	1,13E+08	CCAGCCTT	-	NO	ANK2	frameshift	ANK2:NM_	1	1,9641	Ankyrin-2,	106410	Long QT sy
chr4	1,13E+08	1,13E+08	G	C	NO	ANK2	nonsynony	ANK2:NM_	1	1,9641	Ankyrin-2,	106410	Long QT sy
chr4	1,14E+08	1,14E+08	C	T	NO	CAMK2D	nonsynony	CAMK2D:N	2,29E-03	3,1055	Calcium/c	607708	NA
chr4	1,29E+08	1,29E+08	C	T	NO	JADE1	stopgain	JADE1:NM	0,99885	3,1809	PHD finger	610514	NA
chr4	1,33E+08	1,33E+08	G	T	NO	PCDH10	nonsynony	PCDH10:N	0,89066	1,083	Protocadh	608286	NA
chr4	1,33E+08	1,33E+08	G	A	NO	PCDH10	nonsynony	PCDH10:N	0,89066	1,083	Protocadh	608286	NA
chr4	1,33E+08	1,33E+08	G	-	NO	PCDH10	frameshift	PCDH10:N	0,89066	1,083	Protocadh	608286	NA
chr4	1,39E+08	1,39E+08	C	T	NO	ELF2	0	0	0,99482	1,5919	E74-like ET	619798	NA
chr4	1,45E+08	1,45E+08	A	T	NO	HHIP	nonsynony	HHIP:NM_	0,95436	1,3631	Hedgehog	606178	NA
chr4	1,45E+08	1,45E+08	G	C	NO	ABCE1	nonsynony	ABCE1:NM	0,9738	4,1012	ATP-bindin	601213	NA
chr4	1,46E+08	1,46E+08	G	A	NO	ZNF827	nonsynony	ZNF827:N	0,99965	1,9033	Zinc finger	617962	NA
chr4	1,59E+08	1,59E+08	C	T	NO	FNIP2	0	0	1	1,2788	Folliculin-i	612768	NA

chr4	1,76E+08	1,76E+08	AGCCGCG	-	NO	GPM6A	frameshift	GPM6A:N	0,98962	1,9639	Glycoprote	601275	NA
chr5	7757568	7757568	-	ATGGCCGT	NO	ADCY2	frameshift	ADCY2:NM	0,99898	4,2471	Adenylyl c	103071	NA
chr5	7816984	7816984	C	T	NO	ADCY2	0	0	0,99898	4,2471	Adenylyl c	103071	NA
chr5	14394134	14394134	T	G	NO	TRIO	0	0	1	5,3161	Triple func	601893	Intellectua
chr5	14496985	14496985	C	T	NO	TRIO	nonsynony	TRIO:NM_	1	5,3161	Triple func	601893	Intellectua
chr5	14507971	14507971	A	G	NO	TRIO	nonsynony	TRIO:NM_	1	5,3161	Triple func	601893	Intellectua
chr5	31297289	31297289	G	A	NO	CDH6	nonsynony	CDH6:NM	0,91966	2,3866	Cadherin 6	603007	NA
chr5	31995573	31995573	A	G	NO	PDZD2	0	0	0,99694	1,6926	PDZ doma	610697	NA
chr5	31995652	31995652	G	A	NO	PDZD2	nonsynony	PDZD2:NM	0,99694	1,6926	PDZ doma	610697	NA
chr5	32059342	32059342	G	T	NO	PDZD2	nonsynony	PDZD2:NM	0,99694	1,6926	PDZ doma	610697	NA
chr5	32234999	32234999	A	G	NO	MTMR12	nonsynony	MTMR12:	0,99638	2,2957	Myotubula	606501	NA
chr5	32276681	32276681	C	G	YES	MTMR12	0	0	0,99638	2,2957	Myotubula	606501	NA
chr5	35068212	35068212	T	A	NO	PRLR	0	0	0,98732	0,681	Prolactin r	176761	Multiple fi
chr5	37303291	37303291	G	A	NO	NUP155	nonsynony	NUP155:N	0,65349	0,80795	Nucleopor	606694	?Atrial fibr
chr5	40681750	40681750	G	T	NO	PTGER4	nonsynony	PTGER4:N	0,9755	2,2056	Prostaglan	601586	NA
chr5	43624047	43624047	G	T	NO	NNT	nonsynony	NNT:NM_	0,97271	2,0839	Nicotinam	607878	Glucocorti
chr5	51387588	51387588	G	A	NO	ISL1	nonsynony	ISL1:NM_0	0,86857	1,8743	ISL1 transc	600366	NA
chr5	54519583	54519583	G	A	NO	SNX18	stopgain	SNX18:NM	0,95637	1,9702	NA	NA	NA
chr5	55785346	55785346	T	C	YES	DDX4	0	0	0,99826	2,0515	DEAD-box	605281	NA
chr5	56884701	56884701	A	T	NO	MAP3K1	nonsynony	MAP3K1:N	0,99998	1,6749	Mitogen-a	600982	46XY sex r
chr5	58975081	58975081	-	TAGT	YES	PDE4D	0	0	0,9998	3,7529	Phosphodi	600129	Acrodysos
chr5	77409019	77409019	G	A	NO	PDE8B	nonsynony	PDE8B:NM	0,99905	2,9973	Phosphodi	603390	Pigmented
chr5	78015405	78015405	C	A	NO	AP3B1	0	0	0,61532	0,88997	Adaptor-re	603401	Hermansk
chr5	78020716	78020716	C	A	NO	AP3B1	stopgain	AP3B1:NM	0,61532	0,88997	Adaptor-re	603401	Hermansk
chr5	78162819	78162819	C	T	NO	AP3B1	nonsynony	AP3B1:NM	0,61532	0,88997	Adaptor-re	603401	Hermansk
chr5	78388917	78388917	A	G	NO	SCAMP1	0	0	0,977	2,4181	Secretory	606911	NA
chr5	79306465	79306465	A	G	NO	JMY	0	0	0,96031	1,7085	Junction-m	604279	NA
chr5	79312485	79312485	G	A	NO	JMY	nonsynony	JMY:NM_1	0,96031	1,7085	Junction-m	604279	NA
chr5	81225757	81225757	G	A	NO	RASGRF2	nonsynony	RASGRF2:	0,15243	3,0285	Ras protei	606614	NA
chr5	83553433	83553433	G	A	NO	VCAN	nonsynony	VCAN:NM	1	0,13865	Versican (c	118661	Wagner sy
chr5	95891243	95891243	G	A	NO	ELL2	nonsynony	ELL2:NM_	0,99824	1,5461	Elongation	601874	NA
chr5	95913863	95913863	G	-	NO	ELL2	frameshift	ELL2:NM_	0,99824	1,5461	Elongation	601874	NA
chr5	95961586	95961586	G	A	NO	ELL2	stopgain	ELL2:NM_	0,99824	1,5461	Elongation	601874	NA
chr5	1,03E+08	1,03E+08	G	C	NO	PPIP5K2	nonsynony	PPIP5K2:N	8,58E-21	3,8022	Histidine a	611648	Deafness,
chr5	1,09E+08	1,09E+08	T	C	NO	FER	0	0	0,6633	1,2604	fer (fps/fe	176942	NA
chr5	1,09E+08	1,09E+08	G	A	NO	FER	nonsynony	FER:NM_0	0,6633	1,2604	fer (fps/fe	176942	NA

chr5	1,13E+08	1,13E+08	A	G	NO	APC	0	0	1	0,16764	APC regula	611731	Colorectal
chr5	1,14E+08	1,14E+08	T	A	NO	KCNN2	nonsynony	KCNN2:NM	0,99099	2,2857	Potassium	605879	?Dystonia
chr5	1,15E+08	1,15E+08	C	A	NO	CCDC112	nonsynony	CCDC112:	0,84869	-0,11443	NA	NA	NA
chr5	1,22E+08	1,22E+08	T	C	NO	LOX	nonsynony	LOX:NM_0	0,98769	0,65219	Lysyl oxida	153455	Aortic ane
chr5	1,24E+08	1,24E+08	A	G	NO	CSNK1G3	0	0	0,99947	2,7512	Casein kin	604253	NA
chr5	1,28E+08	1,28E+08	G	A	NO	FBN2	nonsynony	FBN2:NM_	1	1,5491	Fibrillin-2 (	612570	Macular d
chr5	1,31E+08	1,31E+08	G	A	NO	RAPGEF6	stopgain	RAPGEF6:	0,99644	1,5586	RAP guani	610499	NA
chr5	1,32E+08	1,32E+08	G	A	NO	FNIP1	stopgain	FNIP1:NM	0,99957	2,3526	Folliculin-i	610594	Immunode
chr5	1,32E+08	1,32E+08	A	C	NO	FNIP1	nonsynony	FNIP1:NM	0,99957	2,3526	Folliculin-i	610594	Immunode
chr5	1,32E+08	1,32E+08	G	A	NO	FNIP1	nonsynony	FNIP1:NM	0,99957	2,3526	Folliculin-i	610594	Immunode
chr5	1,32E+08	1,32E+08	G	A	NO	PDLIM4	nonsynony	PDLIM4:N	0,98283	0,71455	LIM domai	603422	{Osteopor
chr5	1,33E+08	1,33E+08	T	A	NO	KIF3A	nonsynony	KIF3A:NM	0,89775	3,0924	Kinesin fa	604683	NA
chr5	1,33E+08	1,33E+08	C	T	NO	HSPA4	stopgain	HSPA4:NM	0,99954	2,0572	Heat-shoc	601113	NA
chr5	1,35E+08	1,35E+08	G	A	NO	JADE2	nonsynony	JADE2:NM	0,99994	2,4622	PHD finger	610515	NA
chr5	1,35E+08	1,35E+08	G	A	NO	C5orf24	nonsynony	C5orf24:N	0,73025	0,94804	NA	NA	NA
chr5	1,39E+08	1,39E+08	T	C	NO	HSPA9	0	0	0,96852	1,0372	Heat-shoc	600548	Even-plus
chr5	1,39E+08	1,39E+08	-	AGTGGAG	YES	MATR3	0	0	1	2,7297	Matrin 3 (	164015	Amyotrop
chr5	1,4E+08	1,4E+08	A	T	NO	ANKHD1;A	nonsynony	ANKHD1:N	1.0000e+0	4.4679e+0	Ankyrin re	610500|	NA
chr5	1,4E+08	1,4E+08	G	A	NO	ANKHD1;A	nonsynony	ANKHD1:N	1.0000e+0	4.4679e+0	Ankyrin re	610500|	NA
chr5	1,41E+08	1,41E+08	C	T	NO	ANKHD1-E	stopgain	EIF4EBP3:	1.0000e+0	4.4679e+0	|Eukaryoti	|603483	NA
chr5	1,41E+08	1,41E+08	GAGGAGC	-	NO	ANKHD1-E	stopgain	EIF4EBP3:	1.0000e+0	4.4679e+0	|Eukaryoti	|603483	NA
chr5	1,41E+08	1,41E+08	GAAGGAG	-	NO	ANKHD1-E	frameshift	EIF4EBP3:	1.0000e+0	4.4679e+0	|Eukaryoti	|603483	NA
chr5	1,41E+08	1,41E+08	C	G	NO	NDUFA2	nonsynony	NDUFA2:N	0,77351	-0,24046	NADH-ubi	602137	Mitochond
chr5	1,41E+08	1,41E+08	G	T	NO	PCDHGA1;	nonsynony	PCDHGC3:	1.5784e-0	-5.2980e-0	Protocadh	606288|60	||||||||
chr5	1,42E+08	1,42E+08	C	T	NO	DIAPH1	nonsynony	DIAPH1:N	0,91507	1,6505	Diaphanou	602121	Deafness,
chr5	1,42E+08	1,42E+08	G	A	NO	DIAPH1	nonsynony	DIAPH1:N	0,91507	1,6505	Diaphanou	602121	Deafness,
chr5	1,42E+08	1,42E+08	G	A	NO	NDFIP1	nonsynony	NDFIP1:N	0,97747	1,4968	NEDD4 fa	612050	NA
chr5	1,43E+08	1,43E+08	G	A	NO	NR3C1	nonsynony	NR3C1:NM	0,96958	1,841	Nuclear re	138040	Glucocorti
chr5	1,44E+08	1,44E+08	A	C	NO	YIPF5	0	0	0,98529	0,67755	Yip1 doma	611483	Microceph
chr5	1,47E+08	1,47E+08	C	T	YES	PPP2R2B	0	0	0,98467	2,2458	Protein ph	604325	Spinocere
chr5	1,48E+08	1,48E+08	G	T	NO	FBXO38	nonsynony	FBXO38:N	0,99879	2,8126	F-box only	608533	Neuronop
chr5	1,5E+08	1,5E+08	C	T	NO	HMGXB3	nonsynony	HMGXB3:N	0,999	3,5086	HMG box-	619800	NA
chr5	1,5E+08	1,5E+08	A	G	NO	PDGFRB	0	0	0,9041	1,8417	Platelet-de	173410	Premature
chr5	1,5E+08	1,5E+08	C	T	NO	CAMK2A	nonsynony	CAMK2A:N	0,99845	4,6751	Calcium/c	114078	Intellectua
chr5	1,5E+08	1,5E+08	C	T	NO	TCOF1	0	0	0,9541	0,33899	Treacle (TC	606847	Treacher C
chr5	1,51E+08	1,51E+08	C	T	NO	DCTN4	nonsynony	DCTN4:NM	0,73221	1,4444	Dynactin 4	614758	NA

chr5	1,52E+08	1,52E+08	T	C	NO	SPARC	nonsynony	SPARC:NM	0,89148	1,0988	Osteonect	182120	Osteogene
chr5	1,52E+08	1,52E+08	C	T	NO	SPARC	0	0	0,89148	1,0988	Osteonect	182120	Osteogene
chr5	1,53E+08	1,53E+08	C	T	NO	GRIA1	stopgain	GRIA1:NM	0,99869	3,599	Glutamate	138248	?Intellectu
chr5	1,68E+08	1,68E+08	G	A	NO	TENM2	0	0	1	3,2996	Teneurin t	610119	NA
chr5	1,69E+08	1,69E+08	G	A	NO	SLIT3	stopgain	SLIT3:NM_	0,75233	1,9438	SLIT guida	603745	NA
chr5	1,69E+08	1,69E+08	C	T	NO	SLIT3	nonsynony	SLIT3:NM_	0,75233	1,9438	SLIT guida	603745	NA
chr5	1,69E+08	1,69E+08	G	A	NO	SLIT3	nonsynony	SLIT3:NM_	0,75233	1,9438	SLIT guida	603745	NA
chr5	1,69E+08	1,69E+08	G	A	NO	SLIT3	nonsynony	SLIT3:NM_	0,75233	1,9438	SLIT guida	603745	NA
chr5	1,77E+08	1,77E+08	T	A	NO	GPRIN1	nonsynony	GPRIN1:N	0,97001	0,33776	GPRIN fam	611239	NA
chr5	1,77E+08	1,77E+08	C	T	NO	UNC5A	nonsynony	UNC5A:NM	0,99983	2,7493	unc-5 netr	607869	NA
chr5	1,77E+08	1,77E+08	G	A	NO	PDLIM7	nonsynony	PDLIM7:N	0,70838	0,73094	PDZ and LI	605903	NA
chr5	1,79E+08	1,79E+08	G	C	NO	ADAMTS2	nonsynony	ADAMTS2:	0,9661	1,4291	ADAM me	604539	Ehlers-Dan
chr5	1,79E+08	1,79E+08	C	T	NO	ADAMTS2	nonsynony	ADAMTS2:	0,9661	1,4291	ADAM me	604539	Ehlers-Dan
chr5	1,79E+08	1,79E+08	C	T	YES	ADAMTS2	0	0	0,9661	1,4291	ADAM me	604539	Ehlers-Dan
chr5	1,8E+08	1,8E+08	G	A	YES	MGAT4B	0	0	0,69145	1,3098	Alpha-1,3-	604561	NA
chr5	1,81E+08	1,81E+08	G	A	NO	FLT4	0	0	1	2,8339	fms-relate	136352	Hemangio
chr6	1930201	1930201	G	A	NO	GMDS	nonsynony	GMDS:NM	0,99132	1,819	GDP-mann	602884	NA
chr6	4952413	4952413	T	C	NO	CDYL	0	0	0,99504	2,3517	Chromodo	603778	NA
chr6	4952413	4952413	T	G	NO	CDYL	0	0	0,99504	2,3517	Chromodo	603778	NA
chr6	13641311	13641311	T	C	NO	RANBP9	0	0	0,99999	1,4878	RAN-bindi	603854	NA
chr6	17787773	17787773	T	C	NO	KIF13A	0	0	0,99999	2,0467	Kinesin fa	605433	NA
chr6	17787876	17787876	C	T	YES	KIF13A	0	0	0,99999	2,0467	Kinesin fa	605433	NA
chr6	20402390	20402390	C	-	NO	E2F3	frameshift	E2F3:NM_	0,9458	1,5902	E2F transc	600427	NA
chr6	20402403	20402403	C	A	NO	E2F3	stopgain	E2F3:NM_	0,9458	1,5902	E2F transc	600427	NA
chr6	21595033	21595033	-	C	NO	SOX4	frameshift	SOX4:NM_	0,92656	1,1667	SRY (sex d	184430	Coffin-Siris
chr6	21595035	21595035	-	GC	NO	SOX4	frameshift	SOX4:NM_	0,92656	1,1667	SRY (sex d	184430	Coffin-Siris
chr6	30647559	30647559	-	GCGC	NO	C6orf136	frameshift	C6orf136:	0,94895	1,2564	NA	NA	NA
chr6	30657092	30657092	C	A	NO	DHX16	nonsynony	DHX16:NM	1,60E-12	3,0787	DEAH-box	603405	Neuromus
chr6	31640267	31640267	G	A	NO	BAG6	nonsynony	BAG6:NM_	1	2,7197	BAG cocha	142590	NA
chr6	32171030	32171030	C	T	NO	AGPAT1	nonsynony	AGPAT1:N	0,97215	2,6081	1-acylglyce	603099	NA
chr6	32973128	32973128	A	G	NO	BRD2	0	0	0,99957	0,59311	Bromodom	601540	NA
chr6	32973129	32973129	G	A	NO	BRD2	0	0	0,99957	0,59311	Bromodom	601540	NA
chr6	32976076	32976076	T	C	NO	BRD2	nonsynony	BRD2:NM_	0,99957	0,59311	Bromodom	601540	NA
chr6	32976866	32976866	A	G	NO	BRD2	nonsynony	BRD2:NM_	0,99957	0,59311	Bromodom	601540	NA
chr6	32978294	32978294	C	T	NO	BRD2	stopgain	BRD2:NM_	0,99957	0,59311	Bromodom	601540	NA
chr6	33208764	33208764	G	A	NO	RING1	0	0	0,64854	2,5593	RING finge	602045	NA

chr6	33289621	33289621	-	T	NO	PFDN6	0	0	0,93592	1,4066	Prefoldin 6	605660	NA
chr6	33440913	33440913	C	T	NO	SYNGAP1	stopgain	SYNGAP1:	1	5,6047	Synaptic R	603384	Intellectua
chr6	33684611	33684611	G	A	NO	ITPR3	nonsynony	ITPR3:NM	2,55E-22	4,5522	Inositol 1,4	147267	Charcot-M
chr6	33687512	33687512	T	G	NO	ITPR3	nonsynony	ITPR3:NM	2,55E-22	4,5522	Inositol 1,4	147267	Charcot-M
chr6	34028243	34028243	G	A	NO	GRM4	nonsynony	GRM4:NM	0,99307	2,6835	Glutamate	604100	NA
chr6	34341951	34341951	G	A	NO	NUDT3;RP	nonsynony	NUDT3:NM	4.2531e-0	1.3177e+0	Nudix hyd	609228|	NA
chr6	34425151	34425151	T	C	NO	RPS10;RPS	nonsynony	RPS10:NM	9.7108e-0	1.2401e+0	Ribosomal	603632|	Diamond-
chr6	34762651	34762651	-	T	NO	SNRPC	frameshift	SNRPC:NM	0,72303	2,5542	Small nucl	603522	NA
chr6	35243635	35243635	C	T	NO	SCUBE3	stopgain	SCUBE3:N	0,99939	2,6996	Signal pep	614708	Short statu
chr6	35244809	35244809	A	G	NO	SCUBE3	nonsynony	SCUBE3:N	0,99939	2,6996	Signal pep	614708	Short statu
chr6	35319938	35319938	-	TCAGTAAA	NO	DEF6	stopgain	DEF6:NM_	0,99768	3,0265	DEF6 guan	610094	Immunode
chr6	35321375	35321375	C	T	NO	DEF6	stopgain	DEF6:NM_	0,99768	3,0265	DEF6 guan	610094	Immunode
chr6	35469376	35469376	C	A	NO	RPL10A	0	0	0,85357	2,0633	Ribosomal	615660	NA
chr6	35475034	35475138	AGAGGGC	-	NO	TEAD3	frameshift	TEAD3:NM	0,99362	2,7612	TEA domai	603170	NA
chr6	35475159	35475159	-	GGGGGGT	YES	TEAD3	0	0	0,99362	2,7612	TEA domai	603170	NA
chr6	35580178	35580178	A	C	NO	FKBP5	nonsynony	FKBP5:NM	0,73393	1,6174	FK506-bin	602623	{Major de
chr6	36207437	36207437	G	A	NO	BRPF3	nonsynony	BRPF3:NM	0,99998	2,9063	Bromodom	616856	NA
chr6	37649131	37649131	A	G	NO	MDGA1	nonsynony	MDGA1:N	0,97574	2,5691	MAM dom	609626	NA
chr6	41686131	41686131	G	A	NO	TFEB	nonsynony	TFEB:NM_	0,89931	1,7693	Transcripti	600744	NA
chr6	41687804	41687804	C	T	NO	TFEB	nonsynony	TFEB:NM_	0,89931	1,7693	Transcripti	600744	NA
chr6	42658644	42658644	A	G	YES	UBR2	0	0	1	3,1456	Ubiquitin-	609134	NA
chr6	43206319	43206327	AGGAGGA	-	NO	CUL9	frameshift	CUL9:NM_	0,99191	3,0964	Cullin 9 (C	607489	NA
chr6	44273261	44273261	G	A	NO	TMEM151	nonsynony	TMEM151	0,13582	3,4198	NA	NA	NA
chr6	45422809	45422809	G	A	NO	RUNX2	nonsynony	RUNX2:NM	0,94884	1,6033	Runt-relat	600211	Metaphys
chr6	52507610	52507610	C	T	NO	TRAM2	nonsynony	TRAM2:N	0,94418	1,7609	Translocat	608485	NA
chr6	52516730	52516733	CTCA	-	NO	TRAM2	frameshift	TRAM2:N	0,94418	1,7609	Translocat	608485	NA
chr6	53269136	53269136	-	CG	NO	ELOVL5	frameshift	ELOVL5:N	0,93341	1,5155	Elongation	611805	Spinocere
chr6	53269167	53269167	A	-	NO	ELOVL5	frameshift	ELOVL5:N	0,93341	1,5155	Elongation	611805	Spinocere
chr6	56473968	56473968	T	C	NO	DST	nonsynony	DST:NM_0	1	2,2208	Dystonin (	113810	Neuropath
chr6	56573810	56573810	G	A	NO	DST	nonsynony	DST:NM_0	1	2,2208	Dystonin (	113810	Neuropath
chr6	56605079	56605079	G	C	NO	DST	stopgain	DST:NM_0	1	2,2208	Dystonin (	113810	Neuropath
chr6	56620044	56620044	A	-	NO	DST	stopgain	DST:NM_0	1	2,2208	Dystonin (	113810	Neuropath
chr6	56634463	56634463	T	C	NO	DST	nonsynony	DST:NM_0	1	2,2208	Dystonin (	113810	Neuropath
chr6	56699708	56699708	T	C	NO	DST	nonsynony	DST:NM_0	1	2,2208	Dystonin (	113810	Neuropath
chr6	56701968	56701968	A	T	NO	DST	0	0	1	2,2208	Dystonin (	113810	Neuropath
chr6	63709204	63709204	G	T	NO	PHF3	nonsynony	PHF3:NM_	0,99256	0,75532	PHD finger	607789	NA

chr6	70533793	70533793	C	T	NO	FAM135A	nonsynony	FAM135A:	0,99893	1,3308	NA	NA	NA
chr6	70859381	70859381	G	-	NO	SMAP1	frameshift	SMAP1:NM	0,99357	1,0973	Small ADP	611372	NA
chr6	71887055	71887055	G	A	NO	RIMS1	nonsynony	RIMS1:NM	0,9902	2,0083	Regulating	606629	NA
chr6	72250415	72250415	G	A	NO	RIMS1	nonsynony	RIMS1:NM	0,9902	2,0083	Regulating	606629	NA
chr6	75119427	75119427	A	G	NO	COL12A1	nonsynony	COL12A1:	0,97361	2,106	Collagen, t	120320	Bethlem m
chr6	75152127	75152127	T	C	NO	COL12A1	0	0	0,97361	2,106	Collagen, t	120320	Bethlem m
chr6	75152378	75152378	G	A	NO	COL12A1	nonsynony	COL12A1:	0,97361	2,106	Collagen, t	120320	Bethlem m
chr6	83656750	83656750	C	G	NO	SNAP91	0	0	0,99993	2,1577	Synaptoso	607923	NA
chr6	99402621	99402621	G	A	NO	PNISR	nonsynony	PNISR:NM	0,99998	1,6144	PNN-inter	616653	NA
chr6	1,06E+08	1,06E+08	G	A	NO	PRDM1	nonsynony	PRDM1:N	0,9565	1,7699	PR domain	603423	NA
chr6	1,11E+08	1,11E+08	G	A	NO	CDK19	0	0	0,99982	3,5632	Cyclin-dep	614720	Developm
chr6	1,12E+08	1,12E+08	G	A	NO	FYN	nonsynony	FYN:NM_1	0,99406	4,0323	FYN proto	137025	NA
chr6	1,17E+08	1,17E+08	A	G	NO	VGLL2	nonsynony	VGLL2:NM	0,70547	0,11739	Vestigial-li	609979	NA
chr6	1,18E+08	1,18E+08	C	T	NO	SLC35F1	stopgain	SLC35F1:N	0,95133	1,7774	Solute carr	620349	NA
chr6	1,27E+08	1,27E+08	C	T	NO	KIAA0408;	0	0	4.8741e-0	8.4277e-0	KIAA0408	619236|	NA
chr6	1,28E+08	1,28E+08	GCCGCAG	-	NO	SOGA3	frameshift	SOGA3:NM	0,67899	3,3234	NA	NA	NA
chr6	1,28E+08	1,28E+08	A	G	YES	PTPRK	0	0	0,90291	3,1702	Protein-tyr	602545	NA
chr6	1,31E+08	1,31E+08	C	T	NO	EPB41L2	nonsynony	EPB41L2:N	0,99318	0,68759	Erythrocyt	603237	NA
chr6	1,31E+08	1,31E+08	G	A	NO	EPB41L2	nonsynony	EPB41L2:N	0,99318	0,68759	Erythrocyt	603237	NA
chr6	1,36E+08	1,36E+08	G	C	NO	PDE7B	nonsynony	PDE7B:NM	0,54578	1,37	Phosphodi	604645	NA
chr6	1,38E+08	1,38E+08	T	C	NO	TNFAIP3	0	0	0,99972	1,452	Tumor nec	191163	Autoinflam
chr6	1,39E+08	1,39E+08	TG	-	NO	HECA	frameshift	HECA:NM_	0,61493	2,6087	HDC homo	607977	NA
chr6	1,39E+08	1,39E+08	GGCCGCC	-	NO	HECA	frameshift	HECA:NM_	0,61493	2,6087	HDC homo	607977	NA
chr6	1,47E+08	1,47E+08	G	A	NO	STXBP5	nonsynony	STXBP5:N	0,99956	2,9995	Syntaxin-b	604586	NA
chr6	1,47E+08	1,47E+08	G	T	NO	STXBP5	nonsynony	STXBP5:N	0,99956	2,9995	Syntaxin-b	604586	NA
chr6	1,55E+08	1,55E+08	T	-	NO	SCAF8	frameshift	SCAF8:NM	1	1,8985	SR-related	616024	NA
chr6	1,57E+08	1,57E+08	-	G	NO	ARID1B	frameshift	ARID1B:N	1	2,5854	AT-rich int	614556	Coffin-Siris
chr6	1,6E+08	1,6E+08	G	C	NO	TCP1	nonsynony	TCP1:NM_	0,99527	1,6049	T-complex	186980	NA
chr6	1,6E+08	1,6E+08	A	G	NO	IGF2R	0	0	1	2,0655	Insulin-like	147280	Hepatocell
chr6	1,69E+08	1,69E+08	G	A	NO	THBS2	nonsynony	THBS2:NM	0,55641	2,2148	Thrombos	188061	{Lumbar d
chr6	1,69E+08	1,69E+08	C	T	NO	THBS2	nonsynony	THBS2:NM	0,55641	2,2148	Thrombos	188061	{Lumbar d
chr7	892277	892277	G	T	YES	GET4	0	0	0,56164	0,16812	Guided en	612056	?Congenit
chr7	892423	892423	G	A	NO	GET4	0	0	0,56164	0,16812	Guided en	612056	?Congenit
chr7	1539959	1539959	G	A	NO	MAFK	nonsynony	MAFK:NM	0,71128	1,3619	MAF bZIP	600197	NA
chr7	1746231	1746231	C	-	NO	ELFN1	frameshift	ELFN1:NM	0,99882	2,4386	Extracellul	614964	NA
chr7	2355391	2355391	A	G	NO	EIF3B	nonsynony	EIF3B:NM_	1	1,8208	Eukaryotic	603917	NA

chr7	2924233	2924233	C	A	NO	CARD11	nonsynony	CARD11:N	0,99995	3,5439	Caspase re	607210	B-cell expa
chr7	4759580	4759580	G	T	NO	FOXK1	nonsynony	FOXK1:NM	0,80522	2,4946	Forkhead	616302	NA
chr7	5374208	5374208	-	GGGGGGG	NO	TNRC18	frameshift	TNRC18:N	0,99093	-0,26203	NA	NA	NA
chr7	5394500	5394500	G	A	NO	TNRC18	nonsynony	TNRC18:N	0,99093	-0,26203	NA	NA	NA
chr7	5604032	5604032	C	T	YES	FSCN1	0	0	0,86852	1,9831	Fascin hom	602689	NA
chr7	5605391	5605391	G	A	NO	FSCN1	nonsynony	FSCN1:NM	0,86852	1,9831	Fascin hom	602689	NA
chr7	6187045	6187045	C	G	NO	CYTH3	0	0	0,72252	2,5696	Cytohesin	605081	NA
chr7	11590457	11590457	T	C	NO	THSD7A	0	0	1	-0,59799	Thrombos	612249	NA
chr7	15686195	15686196	GG	-	NO	MEOX2	frameshift	MEOX2:N	0,91495	-0,21843	Mesenchy	600535	NA
chr7	15686198	15686198	G	-	NO	MEOX2	frameshift	MEOX2:N	0,91495	-0,21843	Mesenchy	600535	NA
chr7	17309987	17309994	TAGAGAC	-	NO	AHR	frameshift	AHR:NM_0	0,99999	0,73492	Aryl hydro	600253	?Retinitis p
chr7	18591516	18591516	G	A	NO	HDAC9	nonsynony	HDAC9:NM	0,99973	2,0616	Histone de	606543	?Auriculoc
chr7	21428258	21428258	G	A	NO	SP4	nonsynony	SP4:NM_0	0,99288	0,39538	Sp4 transc	600540	NA
chr7	22146896	22146896	C	A	YES	RAPGEF5	0	0	0,98099	1,6366	RAP guani	609527	NA
chr7	24573593	24573593	G	T	NO	MPP6	0	0	0,81619	2,1249	NA	NA	NA
chr7	24668598	24668598	G	A	NO	MPP6	nonsynony	MPP6:NM	0,81619	2,1249	NA	NA	NA
chr7	26196486	26196486	A	G	NO	HNRNPA2	0	0	0,99732	3,0018	Heterogen	600124	Oculophar
chr7	35202465	35202465	G	A	NO	TBX20	nonsynony	TBX20:NM	0,97302	1,6816	T-box 20 (	606061	Atrial sept
chr7	39987600	39987600	C	T	NO	CDK13	nonsynony	CDK13:NM	0,90753	2,3479	Cyclin-dep	603309	Congenital
chr7	42148222	42148222	T	C	NO	GLI3	0	0	0,99999	0,5222	GLI-Krupp	165240	Greig ceph
chr7	43456323	43456323	G	A	NO	HECW1	nonsynony	HECW1:N	0,99982	2,9523	HECT, C2,	610384	NA
chr7	44146108	44146108	C	A	NO	GCK	stopgain	GCK:NM_0	0,63464	3,069	Glucokinas	138079	MODY, typ
chr7	44760160	44760160	C	T	NO	ZMIZ2	stopgain	ZMIZ2:NM	0,99981	1,9188	Zinc finger	611196	NA
chr7	47297151	47297151	G	A	NO	TNS3	stopgain	TNS3:NM_	1	0,94464	Tensin 3 (T	606825	NA
chr7	49776118	49776118	T	C	NO	VWC2	nonsynony	VWC2:NM	0,97088	1,6745	von Willeb	611108	NA
chr7	56058520	56058617	AGGTGAG	-	NO	CCT6A	frameshift	CCT6A:NM	0,99532	1,2629	Chaperoni	104613	NA
chr7	66951116	66951116	T	G	NO	TMEM248	nonsynony	TMEM248	0,7422	1,2248	NA	NA	NA
chr7	70787350	70787350	G	A	NO	AUTS2	nonsynony	AUTS2:NM	0,99934	2,2242	KIAA0442	607270	Intellectua
chr7	74403841	74403841	A	G	NO	CLIP2	nonsynony	CLIP2:NM_	0,99997	2,3557	CAP-GLY d	603432	NA
chr7	74529921	74529921	C	T	NO	GTF2IRD1	0	0	0,90039	2,6604	GTF21 rep	604318	NA
chr7	75542978	75542978	C	T	NO	HIP1	0	0	0,76173	2,2662	Huntingtin	601767	NA
chr7	75568227	75568227	G	A	NO	HIP1	nonsynony	HIP1:NM_	0,76173	2,2662	Huntingtin	601767	NA
chr7	81702685	81702685	G	A	NO	HGF	nonsynony	HGF:NM_0	0,99947	1,8452	Hepatic gr	142409	Deafness,
chr7	81950394	81950394	A	G	NO	CACNA2D1	stoploss	CACNA2D1	0,99989	3,4906	Calcium ch	114204	Developm
chr7	82909010	82909010	T	C	NO	PCLO	nonsynony	PCLO:NM_	1	-1,461	Piccolo, m	604918	?Pontocer
chr7	86839448	86839448	G	A	NO	GRM3	nonsynony	GRM3:NM	0,78458	3,1962	Glutamate	601115	NA

chr7	94399076	94399076	G	-	NO	COL1A2	frameshift	COL1A2:N	1	2,1491	Collagen I,	120160	Osteogene
chr7	94408343	94408343	G	A	NO	COL1A2	nonsynony	COL1A2:N	1	2,1491	Collagen I,	120160	Osteogene
chr7	94416412	94416412	G	A	NO	COL1A2	nonsynony	COL1A2:N	1	2,1491	Collagen I,	120160	Osteogene
chr7	94420276	94420276	G	A	NO	COL1A2	nonsynony	COL1A2:N	1	2,1491	Collagen I,	120160	Osteogene
chr7	97006105	97006106	AA	-	NO	DLX6	frameshift	DLX6:NM_	0,92126	1,1694	Distal-less	600030	NA
chr7	97006108	97006108	A	-	NO	DLX6	frameshift	DLX6:NM_	0,92126	1,1694	Distal-less	600030	NA
chr7	98204112	98204112	G	A	NO	LMTK2	nonsynony	LMTK2:NM	0,99978	1,2855	Lemur tyro	610989	NA
chr7	98992232	98992232	G	A	NO	TRRAP	0	0	1	8,173	Transform	603015	?Deafness
chr7	1,01E+08	1,01E+08	G	A	NO	ACTL6B	nonsynony	ACTL6B:N	2,83E-04	3,2738	Actin-like	612458	Developm
chr7	1,01E+08	1,01E+08	-	GT	NO	GNB2	frameshift	GNB2:NM	0,98876	3,214	Guanine n	139390	Neurodeve
chr7	1,01E+08	1,01E+08	G	C	NO	SRRT	0	0	0,59362	2,9685	Serrate RN	614469	NA
chr7	1,02E+08	1,02E+08	G	A	NO	CUX1	nonsynony	CUX1:NM_	1	3,7492	Cut-like ho	116896	Global dev
chr7	1,04E+08	1,04E+08	C	T	NO	RELN	nonsynony	RELN:NM_	1	2,2497	Reelin (RE	600514	{Epilepsy,
chr7	1,05E+08	1,05E+08	G	A	NO	KMT2E	nonsynony	KMT2E:NM	1	1,4204	Lysine (K)-	608444	O'Donnell-
chr7	1,05E+08	1,05E+08	-	A	NO	KMT2E	frameshift	KMT2E:NM	1	1,4204	Lysine (K)-	608444	O'Donnell-
chr7	1,05E+08	1,05E+08	G	A	NO	SRPK2	stopgain	SRPK2:NM	0,99697	2,8073	SRSF prote	602980	NA
chr7	1,05E+08	1,05E+08	-	G	NO	SRPK2	0	0	0,99697	2,8073	SRSF prote	602980	NA
chr7	1,15E+08	1,15E+08	-	T	NO	FOXP2	frameshift	FOXP2:NM	0,99994	1,8963	Forkhead	605317	Speech-lan
chr7	1,22E+08	1,22E+08	T	A	YES	PTPRZ1	0	0	0,98997	0,85252	Protein-tyr	176891	NA
chr7	1,28E+08	1,28E+08	T	A	NO	LRRC4	0	0	0,99787	1,9588	Leucine-ric	610486	NA
chr7	1,29E+08	1,29E+08	G	A	NO	FLNC	nonsynony	FLNC:NM_	0,99994	2,789	Filamin C (	102565	Cardiomyo
chr7	1,29E+08	1,29E+08	G	A	NO	FLNC	nonsynony	FLNC:NM_	0,99994	2,789	Filamin C (	102565	Cardiomyo
chr7	1,29E+08	1,29E+08	A	G	NO	FLNC	nonsynony	FLNC:NM_	0,99994	2,789	Filamin C (	102565	Cardiomyo
chr7	1,29E+08	1,29E+08	G	A	NO	FLNC	nonsynony	FLNC:NM_	0,99994	2,789	Filamin C (	102565	Cardiomyo
chr7	1,29E+08	1,29E+08	G	A	NO	AHCYL2	nonsynony	AHCYL2:N	0,99127	2,7392	Adenosylh	616520	NA
chr7	1,32E+08	1,32E+08	T	C	NO	PLXNA4	0	0	0,99999	3,7998	Plexin A4 (	604280	NA
chr7	1,32E+08	1,32E+08	G	-	NO	PLXNA4	frameshift	PLXNA4:N	0,99999	3,7998	Plexin A4 (	604280	NA
chr7	1,32E+08	1,32E+08	C	T	NO	PLXNA4	nonsynony	PLXNA4:N	0,99999	3,7998	Plexin A4 (	604280	NA
chr7	1,35E+08	1,35E+08	G	A	NO	CALD1	nonsynony	CALD1:NM	0,99879	0,80166	Caldesmon	114213	NA
chr7	1,35E+08	1,35E+08	G	T	NO	CALD1	nonsynony	CALD1:NM	0,99879	0,80166	Caldesmon	114213	NA
chr7	1,36E+08	1,36E+08	C	T	NO	NUP205	stopgain	NUP205:N	1	1,3537	Nucleopor	614352	?Nephroti
chr7	1,36E+08	1,36E+08	C	-	NO	NUP205	frameshift	NUP205:N	1	1,3537	Nucleopor	614352	?Nephroti
chr7	1,36E+08	1,36E+08	G	T	NO	NUP205	nonsynony	NUP205:N	1	1,3537	Nucleopor	614352	?Nephroti
chr7	1,36E+08	1,36E+08	-	A	YES	NUP205	0	0	1	1,3537	Nucleopor	614352	?Nephroti
chr7	1,37E+08	1,37E+08	G	A	NO	DGKI	nonsynony	DGKI:NM_	0,93134	3,0849	Diacylglyce	604072	NA
chr7	1,39E+08	1,39E+08	G	A	NO	KIAA1549	nonsynony	KIAA1549:	0,98607	-9,23E-02	KIAA1549	613344	Retinitis pi

chr7	1,39E+08	1,39E+08	C	T	NO	KIAA1549	0	0	0,98607	-9,23E-02	KIAA1549	613344	Retinitis pi
chr7	1,4E+08	1,4E+08	A	G	YES	HIPK2	0	0	1	2,8624	Homeodo	606868	NA
chr7	1,43E+08	1,43E+08	C	G	NO	FAM131B	nonsynony	FAM131B:	0,99092	1,416	Family wit	619282	NA
chr7	1,51E+08	1,51E+08	G	A	NO	KCNH2	nonsynony	KCNH2:NM	0,99593	3,3724	Potassium	152427	Short QT s
chr7	1,51E+08	1,51E+08	G	A	NO	SLC4A2	nonsynony	SLC4A2:N	0,98521	2,9687	Solute carr	109280	?Osteopet
chr7	1,53E+08	1,53E+08	C	G	NO	ACTR3B	0	0	0,99911	2,9731	NA	NA	NA
chr8	3367032	3367032	C	G	NO	CSMD1	nonsynony	CSMD1:N	1	-6,8693	Cub and S	608397	NA
chr8	13088629	13088629	G	A	NO	DLC1	nonsynony	DLC1:NM_	0,99948	-2,931	DLC1 Rho	604258	Colorectal
chr8	19823938	19823938	G	T	NO	INTS10	nonsynony	INTS10:NM	0,99999	1,4436	Integrator	611353	NA
chr8	21984720	21984720	G	A	NO	XPO7	nonsynony	XPO7:NM_	1	4,8157	Exportin 7	606140	NA
chr8	22081153	22081153	G	A	NO	DMTN	nonsynony	DMTN:NM	0,90082	1,255	Erythrocyt	125305	NA
chr8	23246994	23246994	-	GTGA	NO	CHMP7	stopgain	CHMP7:N	0,86301	1,5029	Charged m	611130	NA
chr8	25861069	25861069	G	C	NO	EBF2	stopgain	EBF2:NM_	0,99976	2,11	Early B-cel	609934	NA
chr8	27445917	27445917	C	T	NO	PTK2B	nonsynony	PTK2B:NM	0,93536	1,5043	Protein tyr	601212	NA
chr8	27450747	27450749	AGG	-	NO	PTK2B	frameshift	PTK2B:NM	0,93536	1,5043	Protein tyr	601212	NA
chr8	27458324	27458324	G	A	NO	PTK2B	nonsynony	PTK2B:NM	0,93536	1,5043	Protein tyr	601212	NA
chr8	29345447	29345447	G	-	NO	DUSP4	frameshift	DUSP4:NM	0,88152	1,4902	Dual-speci	602747	NA
chr8	31640560	31640560	-	T	NO	NRG1	frameshift	NRG1:NM	0,99665	0,63667	Neuregulin	142445	{?Schizoph
chr8	35767065	35767065	A	C	NO	UNC5D	nonsynony	UNC5D:N	0,92929	2,2475	unc-5 netr	616466	NA
chr8	38418316	38418316	G	A	NO	FGFR1	nonsynony	FGFR1:NM	0,99984	2,4852	Fibroblast	136350	Pfeiffer sy
chr8	38440303	38440303	-	A	NO	FGFR1	0	0	0,99984	2,4852	Fibroblast	136350	Pfeiffer sy
chr8	38461091	38461091	C	T	NO	FGFR1	0	0	0,99984	2,4852	Fibroblast	136350	Pfeiffer sy
chr8	41265295	41265295	G	A	NO	SFRP1	nonsynony	SFRP1:NM	0,96457	1,3107	Secreted f	604156	NA
chr8	41308790	41308790	G	A	NO	SFRP1	nonsynony	SFRP1:NM	0,96457	1,3107	Secreted f	604156	NA
chr8	41692743	41692743	G	A	NO	ANK1	nonsynony	ANK1:NM_	1	2,0781	Ankyrin-1,	612641	Spherocyt
chr8	41694003	41694003	G	A	NO	ANK1	nonsynony	ANK1:NM_	1	2,0781	Ankyrin-1,	612641	Spherocyt
chr8	41940843	41940843	T	C	NO	KAT6A	nonsynony	KAT6A:NM	1	2,0718	K(lysine) a	601408	Arboleda-T
chr8	42405431	42405431	A	G	NO	VDAC3	nonsynony	VDAC3:NM	0,99454	1,0966	Voltage-de	610029	NA
chr8	47858990	47858990	C	T	NO	PRKDC	0	0	1	2,7576	Protein kin	600899	Immunode
chr8	52645868	52645868	C	T	YES	RB1CC1	0	0	1	0,81178	RB1-induci	606837	Breast can
chr8	54459825	54459825	G	T	NO	SOX17	nonsynony	SOX17:NM	0,88495	0,7745	SRY-box 1	610928	Vesicouret
chr8	55998433	55998434	AT	-	NO	LYN	frameshift	LYN:NM_0	0,99886	3,3381	LYN proto	165120	Autoinflam
chr8	56113911	56113911	G	T	NO	MOS	stopgain	MOS:NM_	0,58928	1,1461	MOS proto	190060	Oocyte/zy
chr8	63038660	63038660	C	G	NO	GGH	nonsynony	GGH:NM_	0,58132	0,45119	Gamma-gl	601509	NA
chr8	70121349	70121349	G	A	NO	NCOA2	stopgain	NCOA2:NM	1	2,2865	Nuclear re	601993	NA
chr8	74237036	74237036	C	T	YES	JPH1	0	0	0,76731	1,4637	Junctophil	605266	?Charcot-

chr8	86448477	86448486	TTCTGGTG	-	NO	WWP1	frameshift	WWP1:NM	0,99966	2,812	WW doma	602307	NA
chr8	91986223	91986223	G	A	NO	RUNX1T1	nonsynony	RUNX1T1:	0,97768	2,1674	unt-relate	133435	NA
chr8	92017347	92017347	-	TGCT	NO	RUNX1T1	frameshift	RUNX1T1:	0,97768	2,1674	unt-relate	133435	NA
chr8	92095346	92095346	C	T	YES	RUNX1T1	0	0	0,97768	2,1674	unt-relate	133435	NA
chr8	94882777	94882777	T	C	NO	CCNE2	0	0	0,86983	1,5004	Cyclin E2 (	603775	NA
chr8	96144837	96144837	T	G	NO	GDF6	nonsynony	GDF6:NM_	0,98833	0,9343	Growth/di	601147	Microphth
chr8	1,01E+08	1,01E+08	C	A	NO	YWHAZ	0	0	0,93965	3,0961	Tyrosine 3	601288	NA
chr8	1,04E+08	1,04E+08	T	C	NO	RIMS2	0	0	1	0,77596	Regulating	606630	Cone-rod s
chr8	1,04E+08	1,04E+08	-	G	NO	RIMS2	frameshift	RIMS2:NM	1	0,77596	Regulating	606630	Cone-rod s
chr8	1,07E+08	1,07E+08	G	-	YES	OXR1	0	0	0,84148	1,0161	Oxidation	605609	Cerebellar
chr8	1,18E+08	1,18E+08	C	T	NO	EXT1	stopgain	EXT1:NM_	0,99732	1,6661	Exostosin	608177	Exostoses,
chr8	1,18E+08	1,18E+08	G	A	NO	EXT1	nonsynony	EXT1:NM_	0,99732	1,6661	Exostosin	608177	Exostoses,
chr8	1,23E+08	1,23E+08	T	C	NO	ATAD2	stoploss	ATAD2:NM	0,9982	1,6583	ATPase fa	611941	NA
chr8	1,23E+08	1,23E+08	A	G	NO	ATAD2	0	0	0,9982	1,6583	ATPase fa	611941	NA
chr8	1,25E+08	1,25E+08	G	A	NO	TRIB1	nonsynony	TRIB1:NM	0,83343	1,2103	Tribbles ps	609461	NA
chr8	1,3E+08	1,3E+08	C	T	NO	ASAP1	nonsynony	ASAP1:NM	1	3,3052	Arf GTPase	605953	NA
chr8	1,32E+08	1,32E+08	C	T	NO	KCNQ3	nonsynony	KCNQ3:NM	0,80089	2,068	Potassium	602232	Seizures, b
chr8	1,32E+08	1,32E+08	A	G	YES	KCNQ3	0	0	0,80089	2,068	Potassium	602232	Seizures, b
chr8	1,41E+08	1,41E+08	GAC	-	NO	PTK2	0	0	0,99987	3,4369	PTK2 prote	600758	NA
chr8	1,41E+08	1,41E+08	C	T	YES	PTK2	0	0	0,99987	3,4369	PTK2 prote	600758	NA
chr8	1,43E+08	1,43E+08	G	-	NO	ARC	frameshift	ARC:NM_0	0,59433	2,5955	Activity-re	612461	NA
chr8	1,44E+08	1,44E+08	-	T	YES	FAM83H	0	0	0,89239	-0,41702	Family wit	611927	Amelogen
chr8	1,44E+08	1,44E+08	G	C	NO	SCRIB	0	0	0,92926	0,38071	Scribble pl	607733	NA
chr8	1,44E+08	1,44E+08	G	A	NO	SCRIB	nonsynony	SCRIB:NM	0,92926	0,38071	Scribble pl	607733	NA
chr8	1,44E+08	1,44E+08	G	A	NO	SCRIB	0	0	0,92926	0,38071	Scribble pl	607733	NA
chr8	1,44E+08	1,44E+08	G	C	NO	PUF60	nonsynony	PUF60:NM	0,99677	4,4358	Poly-U-bin	604819	Verheij syn
chr9	8319934	8319934	G	A	NO	PTPRD	nonsynony	PTPRD:NM	1	0,27314	Protein tyr	601598	NA
chr9	8497267	8497267	C	T	NO	PTPRD	stopgain	PTPRD:NM	1	0,27314	Protein tyr	601598	NA
chr9	20620651	20620651	T	C	NO	MLLT3	0	0	0,99964	1,8957	Myeloid/ly	159558	NA
chr9	33467228	33467228	G	A	NO	NOL6	nonsynony	NOL6:NM_	1	0,86416	Nucleolar	611532	NA
chr9	33469315	33469315	G	A	NO	NOL6	nonsynony	NOL6:NM_	1	0,86416	Nucleolar	611532	NA
chr9	34250721	34250721	G	T	NO	UBAP1	stopgain	UBAP1:NM	0,91168	0,86858	Ubiquitin-	609787	Spastic par
chr9	34251443	34251443	-	TGAAAGA	NO	UBAP1	frameshift	UBAP1:NM	0,91168	0,86858	Ubiquitin-	609787	Spastic par
chr9	35062976	35062976	A	G	YES	VCP	0	0	0,99999	5,4064	Valosin-co	601023	Frontotem
chr9	35699462	35699462	C	A	YES	TLN1	0	0	1	4,6725	Talin 1 (TL	186745	NA
chr9	36191187	36191187	C	T	NO	CLTA	nonsynony	CLTA:NM_	0,96319	0,79917	Clathrin, li	118960	NA

chr9	36882049	36882049	C	T	NO	PAX5	nonsynony	PAX5:NM_	0,99798	2,4521	Paired box	167414	{Leukemia
chr9	38068558	38068558	G	A	NO	SHB	stopgain	SHB:NM_0	0,96874	0,24113	SHB adapt	600314	NA
chr9	69456432	69456432	C	T	NO	APBA1	nonsynony	APBA1:NM	0,53701	2,2307	Amyloid b	602414	NA
chr9	72223151	72223151	G	A	NO	GDA	nonsynony	GDA:NM_	0,99867	1,4639	Guanine d	139260	NA
chr9	72250769	72250769	C	A	NO	GDA	stopgain	GDA:NM_	0,99867	1,4639	Guanine d	139260	NA
chr9	72250813	72250813	G	A	NO	GDA	stopgain	GDA:NM_	0,99867	1,4639	Guanine d	139260	NA
chr9	72364461	72364461	C	A	NO	ZFAND5	0	0	0,84575	1,443	Zinc finger	604761	NA
chr9	74662550	74662550	G	A	NO	RORB	nonsynony	RORB:NM	0,99991	3,0833	RAR-relate	601972	{Epilepsy, i
chr9	81584467	81584467	T	C	NO	TLE1	nonsynony	TLE1:NM_	0,99922	2,7371	TLE family	600189	NA
chr9	81585561	81585561	T	C	NO	TLE1	nonsynony	TLE1:NM_	0,99922	2,7371	TLE family	600189	NA
chr9	81591057	81591057	A	G	NO	TLE1	0	0	0,99922	2,7371	TLE family	600189	NA
chr9	81620560	81620560	G	C	NO	TLE1	0	0	0,99922	2,7371	TLE family	600189	NA
chr9	87649918	87649918	C	T	NO	DAPK1	0	0	0,99767	2,5678	Death-ass	600831	NA
chr9	89399342	89399342	T	C	NO	SEMA4D	0	0	0,97472	0,99698	Semaphor	601866	NA
chr9	92764663	92764663	G	A	NO	BICD2	nonsynony	BICD2:NM	0,98272	2,205	BICD carbo	609797	Spinal mus
chr9	93293167	93293167	G	A	NO	WNK2	nonsynony	WNK2:NM	1	2,5818	WNK lysin	606249	NA
chr9	93452252	93452252	G	A	NO	FAM120A;	nonsynony	FAM120A:	1.0000e+0	3.3485e+0	Family wit	612265|	NA
chr9	93660384	93660384	C	-	NO	PHF2	frameshift	PHF2:NM_	1	3,4944	PHD finger	604351	NA
chr9	93676725	93676725	-	CCTCCACC	NO	PHF2	frameshift	PHF2:NM_	1	3,4944	PHD finger	604351	NA
chr9	99832684	99832684	C	T	NO	NR4A3	0	0	0,9126	2,8061	Chondrosa	600542	Chondrosa
chr9	1E+08	1E+08	T	C	NO	TEX10	0	0	1	1,541	Testis-expr	616717	NA
chr9	1,04E+08	1,04E+08	G	A	NO	SMC2	nonsynony	SMC2:NM	0,9999	1,0589	Chromoso	605576	NA
chr9	1,09E+08	1,09E+08	C	A	NO	EPB41L4B	stopgain	EPB41L4B:	0,99972	1,2503	Erythrocyt	610340	NA
chr9	1,1E+08	1,1E+08	G	A	NO	SVEP1	nonsynony	SVEP1:NM	0,99995	1,8788	Sushi, von	611691	NA
chr9	1,1E+08	1,1E+08	-	G	NO	SVEP1	frameshift	SVEP1:NM	0,99995	1,8788	Sushi, von	611691	NA
chr9	1,1E+08	1,1E+08	C	T	NO	SVEP1	0	0	0,99995	1,8788	Sushi, von	611691	NA
chr9	1,11E+08	1,11E+08	G	A	NO	SVEP1	nonsynony	SVEP1:NM	0,99995	1,8788	Sushi, von	611691	NA
chr9	1,14E+08	1,14E+08	C	T	NO	COL27A1	nonsynony	COL27A1:	0,5864	0,6291	Collagen, t	608461	Steel synd
chr9	1,14E+08	1,14E+08	T	C	NO	COL27A1	0	0	0,5864	0,6291	Collagen, t	608461	Steel synd
chr9	1,14E+08	1,14E+08	C	T	NO	COL27A1	nonsynony	COL27A1:	0,5864	0,6291	Collagen, t	608461	Steel synd
chr9	1,14E+08	1,14E+08	A	G	NO	COL27A1	0	0	0,5864	0,6291	Collagen, t	608461	Steel synd
chr9	1,14E+08	1,14E+08	C	G	NO	COL27A1	0	0	0,5864	0,6291	Collagen, t	608461	Steel synd
chr9	1,22E+08	1,22E+08	C	T	NO	DAB2IP	nonsynony	DAB2IP:N	0,99995	2,3614	DAB2-inte	609205	NA
chr9	1,23E+08	1,23E+08	C	T	NO	RABGAP1	nonsynony	RABGAP1:	1	2,9624	RAB GTPas	615882	NA
chr9	1,25E+08	1,25E+08	A	T	NO	SCAI	0	0	0,99936	3,0105	Suppresso	619222	NA
chr9	1,25E+08	1,25E+08	A	-	NO	PPP6C	frameshift	PPP6C:NM	0,85535	2,5825	Protein ph	612725	NA

chr9	1,25E+08	1,25E+08	C	T	NO	GAPVD1	0	0	1	2,5854	GTPase-ac	611714	NA
chr9	1,25E+08	1,25E+08	G	A	NO	GAPVD1	nonsynony	GAPVD1:N	1	2,5854	GTPase-ac	611714	NA
chr9	1,25E+08	1,25E+08	G	A	NO	GAPVD1	nonsynony	GAPVD1:N	1	2,5854	GTPase-ac	611714	NA
chr9	1,27E+08	1,27E+08	C	T	NO	LMX1B	stopgain	LMX1B:NM	0,74637	2,0208	LIM home	602575	Focal segm
chr9	1,27E+08	1,27E+08	G	A	NO	LMX1B	0	0	0,74637	2,0208	LIM home	602575	Focal segm
chr9	1,28E+08	1,28E+08	G	A	NO	GOLGA2	0	0	0,98756	0,90253	Golgin A2	602580	Developm
chr9	1,29E+08	1,29E+08	C	T	NO	SPTAN1	nonsynony	SPTAN1:N	1	5,523	Spectrin, a	182810	Developm
chr9	1,29E+08	1,29E+08	C	T	NO	SPTAN1	nonsynony	SPTAN1:N	1	5,523	Spectrin, a	182810	Developm
chr9	1,29E+08	1,29E+08	G	A	NO	SPTAN1	nonsynony	SPTAN1:N	1	5,523	Spectrin, a	182810	Developm
chr9	1,29E+08	1,29E+08	G	C	NO	ZER1	nonsynony	ZER1:NM_	0,99992	3,7482	Zyg11-rela	617764	NA
chr9	1,29E+08	1,29E+08	C	T	NO	NUP188	nonsynony	NUP188:N	0,95428	1,1238	Nucleopor	615587	Sandestig-
chr9	1,29E+08	1,29E+08	G	A	NO	NUP188	nonsynony	NUP188:N	0,95428	1,1238	Nucleopor	615587	Sandestig-
chr9	1,29E+08	1,29E+08	TCACAACA	-	NO	NUP188	frameshift	NUP188:N	0,95428	1,1238	Nucleopor	615587	Sandestig-
chr9	1,29E+08	1,29E+08	T	C	NO	NUP188	nonsynony	NUP188:N	0,95428	1,1238	Nucleopor	615587	Sandestig-
chr9	1,3E+08	1,3E+08	-	T	YES	FNBP1	0	0	0,82844	2,0246	Formin-bin	606191	NA
chr9	1,32E+08	1,32E+08	G	-	NO	RAPGEF1	frameshift	RAPGEF1:	0,99995	3,13	RAP guani	600303	NA
chr9	1,32E+08	1,32E+08	G	A	NO	NTNG2	nonsynony	NTNG2:NM	0,99866	2,9337	Netrin G2	618689	Neurodeve
chr9	1,32E+08	1,32E+08	C	A	NO	SETX	0	0	0,95483	-0,11177	Senataxin	608465	Spinocere
chr9	1,33E+08	1,33E+08	A	G	NO	TSC1	nonsynony	TSC1:NM_	1	2,3217	Hamartin (	605284	Focal corti
chr9	1,34E+08	1,34E+08	C	T	NO	WDR5	0	0	0,99978	3,3923	WD repeat	609012	NA
chr9	1,35E+08	1,35E+08	G	A	NO	COL5A1	nonsynony	COL5A1:N	1	2,0682	Collagen V	120215	Ehlers-Dan
chr9	1,35E+08	1,35E+08	A	T	NO	COL5A1	nonsynony	COL5A1:N	1	2,0682	Collagen V	120215	Ehlers-Dan
chr9	1,35E+08	1,35E+08	A	G	NO	COL5A1	nonsynony	COL5A1:N	1	2,0682	Collagen V	120215	Ehlers-Dan
chr9	1,35E+08	1,35E+08	T	C	NO	OLFM1	nonsynony	OLFM1:NM	0,99679	3,4102	Olfactome	605366	NA
chr9	1,37E+08	1,37E+08	G	A	NO	RABL6	0	0	0,99817	-0,1161	RAB-like p	610615	NA
chr9	1,37E+08	1,37E+08	G	A	NO	RABL6	nonsynony	RABL6:NM	0,99817	-0,1161	RAB-like p	610615	NA
chr9	1,37E+08	1,37E+08	-	CTCTAGGC	NO	RABL6	stopgain	RABL6:NM	0,99817	-0,1161	RAB-like p	610615	NA
chr9	1,37E+08	1,37E+08	C	T	NO	TRAF2	nonsynony	TRAF2:NM	0,99979	1,5611	TNF recep	601895	NA
chr9	1,37E+08	1,37E+08	-	AT	NO	TRAF2	frameshift	TRAF2:NM	0,99979	1,5611	TNF recep	601895	NA
chr9	1,37E+08	1,37E+08	A	G	NO	TRAF2	nonsynony	TRAF2:NM	0,99979	1,5611	TNF recep	601895	NA
chr9	1,37E+08	1,37E+08	C	T	NO	TRAF2	stopgain	TRAF2:NM	0,99979	1,5611	TNF recep	601895	NA
chr9	1,37E+08	1,37E+08	C	T	NO	ABCA2	nonsynony	ABCA2:NM	1	4,9116	ATP-bindin	600047	Intellectua
chr9	1,37E+08	1,37E+08	T	-	NO	ABCA2	frameshift	ABCA2:NM	1	4,9116	ATP-bindin	600047	Intellectua
chr9	1,37E+08	1,37E+08	G	A	NO	ABCA2	stopgain	ABCA2:NM	1	4,9116	ATP-bindin	600047	Intellectua
chr9	1,37E+08	1,37E+08	ACCTGGTG	-	NO	TUBB4B	frameshift	TUBB4B:N	0,80877	4,4977	Tubulin, b	602660	Leber cong
chr9	1,38E+08	1,38E+08	C	A	NO	MRPL41	stopgain	MRPL41:N	0,55531	0,41859	Mitochond	611846	NA

chr9	1,38E+08	1,38E+08	G	A	NO	CACNA1B	nonsynony	CACNA1B:	0,99998	4,5165	Calcium ch	601012	Neurodeve
chr9	1,38E+08	1,38E+08	G	A	NO	CACNA1B	nonsynony	CACNA1B:	0,99998	4,5165	Calcium ch	601012	Neurodeve
chr10	344804	344804	C	T	NO	DIP2C	0	0	1	4,6684	Disco-inter	611380	NA
chr10	1379162	1379162	T	G	YES	ADARB2	0	0	0,85603	1,7325	Adenosine	602065	NA
chr10	7172089	7172089	G	A	NO	SFMBT2	nonsynony	SFMBT2:N	0,99939	1,2804	SCM-like p	615392	NA
chr10	13338719	13338719	C	T	NO	SEPHS1	nonsynony	SEPHS1:N	0,97637	3,2396	Selenopho	600902	NA
chr10	13654425	13654425	C	A	NO	FRMD4A	nonsynony	FRMD4A:N	1	2,4555	FERM dom	616305	?Corpus ca
chr10	14838464	14838464	G	C	NO	HSPA14;LO	0	0	6.7359e-0	1.1695e+0	Heat-shoc	610369|	NA
chr10	14878961	14878961	G	T	NO	SUV39H2	0	0	0,92921	3,2832	Suppresso	606503	NA
chr10	21727910	21727910	G	C	NO	MLLT10	nonsynony	MLLT10:N	1	1,5999	ALL1 fused	602409	Leukemia,
chr10	24598003	24598003	C	T	NO	ARHGAP21	nonsynony	ARHGAP21	1	0,95534	GTPase-ac	609870	NA
chr10	26755738	26755738	G	A	NO	ABI1	nonsynony	ABI1:NM_	0,63911	2,0949	ABL-intera	603050	NA
chr10	31526776	31526776	G	A	NO	ZEB1	nonsynony	ZEB1:NM_	0,96665	1,6042	Zinc finger	189909	Corneal dy
chr10	31527194	31527194	-	A	NO	ZEB1	frameshift	ZEB1:NM_	0,96665	1,6042	Zinc finger	189909	Corneal dy
chr10	32907071	32907071	A	G	NO	ITGB1	stoploss	ITGB1:NM	0,98208	3,4595	Integrin, b	135630	NA
chr10	32926040	32926040	G	A	NO	ITGB1	nonsynony	ITGB1:NM	0,98208	3,4595	Integrin, b	135630	NA
chr10	33202980	33202980	G	A	NO	NRP1	nonsynony	NRP1:NM_	0,98584	1,56	Neuropilin	602069	NA
chr10	34360142	34360142	G	A	NO	PARD3	nonsynony	PARD3:NM	0,95577	1,0399	PAR3 fami	606745	NA
chr10	34470086	34470086	T	C	NO	PARD3	nonsynony	PARD3:NM	0,95577	1,0399	PAR3 fami	606745	NA
chr10	35195216	35195216	-	A	YES	CREM	0	0	0,50878	0,46198	cAMP-resp	123812	NA
chr10	48779955	48779955	G	A	NO	WDFY4	nonsynony	WDFY4:N	0,34009	3,1236	WD repeat	613316	NA
chr10	49693871	49693871	C	A	NO	C10orf53	stopgain	C10orf53:	0,58427	0,4573	NA	NA	NA
chr10	58788421	58788421	C	T	NO	BICC1	nonsynony	BICC1:NM	0,83685	1,2467	Bicaudal C	614295	{Renal dys
chr10	59176962	59176962	A	G	NO	PHYHIPL	0	0	0,82096	1,6362	NA	NA	NA
chr10	60042669	60042669	C	T	NO	ANK3	0	0	1	2,7937	Ankyrin-3,	600465	Intellectua
chr10	63176425	63176425	G	A	NO	JMJD1C	nonsynony	JMJD1C:N	1	1,513	Jumonji do	604503	NA
chr10	68788274	68788274	G	A	NO	CCAR1	nonsynony	CCAR1:NM	0,99999	3,6371	Cell divisio	612569	NA
chr10	69171199	69171200	AG	-	NO	VPS26A	frameshift	VPS26A:N	0,61411	1,5574	VPS26 retr	605506	NA
chr10	69394945	69394945	C	G	NO	HK1	0	0	0,91459	3,2312	Hexokinas	142600	Retinitis pi
chr10	73479319	73479319	G	A	NO	PPP3CB	nonsynony	PPP3CB:N	0,99998	3,793	Protein ph	114106	NA
chr10	73797794	73797794	C	T	NO	ZSWIM8	nonsynony	ZSWIM8:N	1	5,1433	Zinc finger	619213	NA
chr10	75400019	75400019	G	A	NO	ZNF503	nonsynony	ZNF503:N	0,94589	0,68106	Zinc finger	613902	NA
chr10	79302103	79302103	G	T	NO	ZMIZ1	0	0	0,99937	3,3882	Zinc finger	607159	Neurodeve
chr10	79306125	79306125	G	A	NO	ZMIZ1	nonsynony	ZMIZ1:NM	0,99937	3,3882	Zinc finger	607159	Neurodeve
chr10	79311101	79311101	G	A	NO	ZMIZ1	nonsynony	ZMIZ1:NM	0,99937	3,3882	Zinc finger	607159	Neurodeve
chr10	84373790	84373790	A	G	NO	CCSER2	nonsynony	CCSER2:N	0,66698	-7,27E-02	Coiled-coil	619944	NA

chr10	84463940	84463940	G	T	NO	CCSER2	nonsynony	CCSER2:N	0,66698	-7,27E-02	Coiled-coil	619944	NA
chr10	84499959	84499959	A	T	NO	CCSER2	stopgain	CCSER2:N	0,66698	-7,27E-02	Coiled-coil	619944	NA
chr10	86921678	86921678	G	A	NO	BMPR1A	nonsynony	BMPR1A:N	0,90333	1,9176	Bone morp	601299	Polyposis s
chr10	87094733	87094733	G	A	NO	GLUD1	nonsynony	GLUD1:NM	7,05E-03	3,059	Glutamate	138130	Hyperinsul
chr10	87952143	87952143	G	A	NO	PTEN	nonsynony	PTEN:NM_	0,25651	3,4883	Phosphata	601728	{Glioma su
chr10	92027134	92027134	G	A	NO	BTAF1	nonsynony	BTAF1:NM	1	4,3771	BTAF1 RNA	605191	NA
chr10	93777401	93777401	G	A	NO	LGI1	nonsynony	LGI1:NM_	0,99914	2,7809	Leucine-ric	604619	Epilepsy, f
chr10	97019062	97019062	G	A	NO	SLIT1	nonsynony	SLIT1:NM_	0,99999	3,0887	SLIT guida	603742	NA
chr10	97021322	97021322	G	A	NO	SLIT1	nonsynony	SLIT1:NM_	0,99999	3,0887	SLIT guida	603742	NA
chr10	97064821	97064821	G	A	NO	SLIT1	nonsynony	SLIT1:NM_	0,99999	3,0887	SLIT guida	603742	NA
chr10	1E+08	1E+08	G	A	NO	CHUK	nonsynony	CHUK:NM	0,99403	2,7816	Componen	600664	?Popliteal
chr10	1,01E+08	1,01E+08	A	G	YES	TLX1	0	0	0,74161	1,9272	T-cell leuk	186770	NA
chr10	1,02E+08	1,02E+08	G	A	YES	PPRC1	0	0	1	0,24445	Peroxisom	617462	NA
chr10	1,02E+08	1,02E+08	-	T	NO	PPRC1	frameshift	PPRC1:NM	1	0,24445	Peroxisom	617462	NA
chr10	1,02E+08	1,02E+08	G	A	NO	NFKB2	0	0	1	3,9376	Nuclear fa	164012	Immunode
chr10	1,02E+08	1,02E+08	C	G	NO	NFKB2	0	0	1	3,9376	Nuclear fa	164012	Immunode
chr10	1,03E+08	1,03E+08	T	C	NO	SUFU	nonsynony	SUFU:NM_	0,99992	1,9341	Suppresso	607035	{Meningio
chr10	1,03E+08	1,03E+08	C	T	NO	SUFU	nonsynony	SUFU:NM_	0,99992	1,9341	Suppresso	607035	{Meningio
chr10	1,03E+08	1,03E+08	G	A	NO	SUFU	nonsynony	SUFU:NM_	0,99992	1,9341	Suppresso	607035	{Meningio
chr10	1,03E+08	1,03E+08	G	A	NO	WBP1L	nonsynony	WBP1L:NM	0,64011	1,0229	Chromoso	611129	NA
chr10	1,03E+08	1,03E+08	C	T	NO	CNNM2	nonsynony	CNNM2:N	0,99521	4,4105	Cyclin M2	607803	Hypomagn
chr10	1,04E+08	1,04E+08	T	C	NO	SH3PXD2A	0	0	0,98897	1,5586	SH3 and P	619455	NA
chr10	1,04E+08	1,04E+08	C	T	NO	SH3PXD2A	0	0	0,98897	1,5586	SH3 and P	619455	NA
chr10	1,07E+08	1,07E+08	T	A	NO	SORCS1	0	0	0,55937	1,3024	Sortilin-rel	606283	NA
chr10	1,1E+08	1,1E+08	G	T	NO	SMNDC1	0	0	0,63777	2,4844	Survival m	603519	NA
chr10	1,11E+08	1,11E+08	A	G	NO	SMC3	0	0	1	6,3999	Structural	606062	Cornelia d
chr10	1,17E+08	1,17E+08	G	A	NO	VAX1	nonsynony	VAX1:NM_	0,7705	0,8693	Ventral an	604294	?Micropht
chr10	1,19E+08	1,19E+08	G	A	NO	EIF3A	stopgain	EIF3A:NM	1	3,0406	Eukaryotic	602039	NA
chr10	1,19E+08	1,19E+08	G	A	NO	EIF3A	nonsynony	EIF3A:NM	1	3,0406	Eukaryotic	602039	NA
chr10	1,19E+08	1,19E+08	C	-	NO	GRK5	frameshift	GRK5:NM_	0,67567	2,1583	G protein-	600870	NA
chr10	1,22E+08	1,22E+08	A	G	NO	FGFR2	0	0	0,99731	2,402	Fibroblast	176943	Bent bone
chr10	1,23E+08	1,23E+08	G	A	NO	HMX3	nonsynony	HMX3:NM	0,91992	0,90769	H6 family	613380	NA
chr11	986805	986805	G	-	NO	AP2A2	frameshift	AP2A2:NM	1	3,1324	Adaptor-re	607242	NA
chr11	1442610	1442610	C	T	NO	BRSK2	0	0	0,95141	2,9466	BR serine/	609236	NA
chr11	1445436	1445436	-	T	NO	BRSK2	frameshift	BRSK2:NM	0,95141	2,9466	BR serine/	609236	NA
chr11	1565591	1565591	G	C	NO	DUSP8	0	0	0,69975	2,3291	Dual speci	602038	NA

chr11	2884009	2884009	G	T	NO	CDKN1C	stopgain	CDKN1C:N	0,81963	1,9346	Cyclin-dep	600856	IMAGE syn
chr11	2885264	2885264	G	A	NO	CDKN1C	nonsynony	CDKN1C:N	0,81963	1,9346	Cyclin-dep	600856	IMAGE syn
chr11	4086544	4086544	G	T	YES	STIM1	nonsynony	STIM1:NM	0,77534	2,1179	Stromal in	605921	Myopathy
chr11	6402113	6402113	G	A	NO	APBB1	nonsynony	APBB1:NM	0,96811	1,5605	Amyloid b	602709	NA
chr11	6611773	6611773	G	A	NO	TAF10	nonsynony	TAF10:NM	0,88076	0,83802	TAF10 RNA	600475	NA
chr11	13378319	13378320	CA	-	NO	ARNTL	0	0	0,99444	2,9244	NA	NA	NA
chr11	18325945	18325945	A	G	NO	GTF2H1	0	0	0,98982	1,7981	General tr	189972	NA
chr11	35418950	35418950	C	T	NO	SLC1A2	nonsynony	SLC1A2:N	0,71036	2,2846	Solute carr	600300	Developm
chr11	43493770	43493770	C	A	NO	TTC17	0	0	0,99996	1,3045	Tetratricop	619388	NA
chr11	45903428	45903428	G	A	NO	MAPK8IP1	nonsynony	MAPK8IP1	0,99983	1,4927	Mitogen-a	604641	{Diabetes
chr11	46312434	46312434	A	G	NO	CREB3L1	nonsynony	CREB3L1:N	0,98488	0,94937	cAMP resp	616215	Osteogene
chr11	46443602	46443602	A	G	NO	AMBRA1	0	0	1	2,948	Activating	611359	NA
chr11	46889946	46889946	G	A	NO	LRP4	nonsynony	LRP4:NM_	1,44E-06	3,0632	Low densit	604270	?Myasthen
chr11	47261941	47261941	G	A	NO	NR1H3	nonsynony	NR1H3:NM	0,90374	1,2978	Nuclear re	602423	NA
chr11	47268596	47268596	G	A	NO	NR1H3	nonsynony	NR1H3:NM	0,90374	1,2978	Nuclear re	602423	NA
chr11	57737600	57737614	TGTTCCAG	-	NO	TMX2	frameshift	TMX2:NM	0,64745	0,21872	Thioredoxi	616715	Neurodeve
chr11	57739186	57739193	CAAGGTG	-	NO	TMX2	frameshift	TMX2:NM	0,64745	0,21872	Thioredoxi	616715	Neurodeve
chr11	59576681	59576681	G	A	NO	OSBP	nonsynony	OSBP:NM_	0,99855	3,585	Oxysterol-	167040	NA
chr11	62516836	62516836	G	A	NO	AHNAK	stopgain	AHNAK:N	0,89827	-2,9677	AHNAK nu	103390	NA
chr11	62517693	62517693	C	-	NO	AHNAK	frameshift	AHNAK:N	0,89827	-2,9677	AHNAK nu	103390	NA
chr11	62529340	62529340	T	A	NO	AHNAK	stopgain	AHNAK:N	0,89827	-2,9677	AHNAK nu	103390	NA
chr11	62639928	62639928	A	G	NO	GANAB	0	0	0,99873	2,2404	Neutral alp	104160	Polycystic
chr11	62676778	62676790	GTTTCTAG	-	NO	UBXN1	frameshift	UBXN1:NM	0,72752	0,83765	UBX doma	616378	NA
chr11	62831266	62831266	T	C	NO	STX5	0	0	0,84297	0,40028	Syntaxin 5	603189	?Congenit
chr11	63998954	63998954	C	A	YES	MACROD1	0	0	0,56909	0,79367	Macro do	610400	NA
chr11	64165696	64165696	C	A	YES	MACROD1	0	0	0,56909	0,79367	Macro do	610400	NA
chr11	64287722	64287750	CTCGCTGT	-	NO	GPR137	frameshift	GPR137:N	0,66798	1,0419	NA	NA	NA
chr11	64287949	64287949	A	G	NO	GPR137	0	0	0,66798	1,0419	NA	NA	NA
chr11	64650448	64650448	C	T	NO	NRXN2	nonsynony	NRXN2:NM	0,99934	4,0235	Neurexin 2	600566	NA
chr11	64778218	64778218	G	-	NO	SF1	stopgain	SF1:NM_0	0,99943	3,6203	Splicing fa	601516	NA
chr11	65132740	65132740	C	T	NO	SYVN1	nonsynony	SYVN1:NM	0,9997	2,1821	Synovial a	608046	NA
chr11	65546793	65546793	C	G	NO	LTBP3	0	0	0,68647	2,849	Latent tran	602090	Dental ano
chr11	66643456	66643456	G	A	NO	RBM14-RB	nonsynony	RBM14-RB	9.8438e-0	1.9812e+0	|RNA-bind	|602571	NA
chr11	66853239	66853239	C	A	NO	PC	nonsynony	PC:NM_02	1,38E-02	3,0552	Pyruvate c	608786	Pyruvate c
chr11	68386583	68386583	G	A	NO	LRP5	nonsynony	LRP5:NM_	0,50992	1,6684	Low densit	603506	Osteopetr
chr11	68436930	68436930	T	C	NO	LRP5	nonsynony	LRP5:NM_	0,50992	1,6684	Low densit	603506	Osteopetr

chr11	68590674	68590674	G	A	NO	PPP6R3	nonsynony	PPP6R3:N	0,99487	1,5409	SAPS dom	610879	NA
chr11	69641357	69641357	G	A	NO	CCND1	nonsynony	CCND1:NM	0,89057	1,4216	Cyclin D1 (	168461	{von Hippe
chr11	70103108	70103108	T	-	NO	ANO1	frameshift	ANO1:NM	0,87426	2,77	Anoctamin	610108	?Moyamo
chr11	70124387	70124387	A	G	NO	ANO1	nonsynony	ANO1:NM	0,87426	2,77	Anoctamin	610108	?Moyamo
chr11	70167286	70167286	-	C	NO	ANO1	stopgain	ANO1:NM	0,87426	2,77	Anoctamin	610108	?Moyamo
chr11	70206440	70206440	G	A	NO	FADD	stopgain	FADD:NM	0,52595	0,83747	FAS-associ	602457	Immunode
chr11	72004083	72004083	G	A	NO	NUMA1	nonsynony	NUMA1:N	1	0,88132	Nuclear m	164009	Leukemia,
chr11	72006030	72006030	C	A	NO	NUMA1	0	0	1	0,88132	Nuclear m	164009	Leukemia,
chr11	72007368	72007368	G	A	NO	NUMA1	nonsynony	NUMA1:N	1	0,88132	Nuclear m	164009	Leukemia,
chr11	72024276	72024276	T	C	NO	NUMA1	nonsynony	NUMA1:N	1	0,88132	Nuclear m	164009	Leukemia,
chr11	73310077	73310077	C	G	NO	ARHGEF17	stopgain	ARHGEF17	0,98445	0,74953	Rho guani	617043	NA
chr11	73311135	73311135	C	T	NO	ARHGEF17	nonsynony	ARHGEF17	0,98445	0,74953	Rho guani	617043	NA
chr11	73355934	73355934	G	A	NO	ARHGEF17	nonsynony	ARHGEF17	0,98445	0,74953	Rho guani	617043	NA
chr11	73365696	73365696	G	A	NO	ARHGEF17	nonsynony	ARHGEF17	0,98445	0,74953	Rho guani	617043	NA
chr11	73367697	73367697	C	T	NO	ARHGEF17	stopgain	ARHGEF17	0,98445	0,74953	Rho guani	617043	NA
chr11	74171455	74171455	C	T	NO	PPME1	nonsynony	PPME1:NM	0,99904	1,7468	Protein ph	611117	NA
chr11	74492359	74492359	G	A	NO	LIPT2	nonsynony	LIPT2:NM_	0,62575	-0,11985	Lipoyl(octa	617659	Encephalo
chr11	75272891	75272891	T	C	NO	ARRB1	0	0	0,99394	2,3563	Arrestin, b	107940	NA
chr11	78658065	78658065	C	T	NO	TENM4	nonsynony	TENM4:N	1	2,7323	Teneurin t	610084	Essential t
chr11	78669648	78669648	G	A	NO	TENM4	nonsynony	TENM4:N	1	2,7323	Teneurin t	610084	Essential t
chr11	78778617	78778617	C	A	NO	TENM4	nonsynony	TENM4:N	1	2,7323	Teneurin t	610084	Essential t
chr11	78889820	78889820	G	A	NO	TENM4	nonsynony	TENM4:N	1	2,7323	Teneurin t	610084	Essential t
chr11	83157498	83157498	G	T	NO	PCF11	nonsynony	PCF11:NM	1	2,6592	PCF11, yea	608876	NA
chr11	88509032	88509032	G	A	NO	GRM5	nonsynony	GRM5:NM	0,99872	3,2078	Glutamate	604102	NA
chr11	92790075	92790075	A	T	NO	FAT3	stopgain	FAT3:NM_	0,99995	1,26	FAT atypic	612483	NA
chr11	92792767	92792767	G	T	NO	FAT3	nonsynony	FAT3:NM_	0,99995	1,26	FAT atypic	612483	NA
chr11	1,03E+08	1,03E+08	G	C	NO	DCUN1D5	0	0	0,99432	2,2757	DCN1 dom	616522	NA
chr11	1,15E+08	1,15E+08	A	G	YES	CADM1	0	0	0,66216	1,8385	Cell adhesi	605686	NA
chr11	1,17E+08	1,17E+08	G	-	NO	PAFAH1B2	frameshift	PAFAH1B2	0,93401	1,6503	Platelet-ac	602508	NA
chr11	1,17E+08	1,17E+08	G	T	NO	PAFAH1B2	stopgain	PAFAH1B2	0,93401	1,6503	Platelet-ac	602508	NA
chr11	1,17E+08	1,17E+08	C	T	NO	PAFAH1B2	stopgain	PAFAH1B2	0,93401	1,6503	Platelet-ac	602508	NA
chr11	1,17E+08	1,17E+08	T	C	NO	RNF214	nonsynony	RNF214:N	0,9096	1,1522	NA	NA	NA
chr11	1,17E+08	1,17E+08	C	T	NO	DSCAML1	nonsynony	DSCAML1:	1	3,15	DS cell adh	611782	NA
chr11	1,17E+08	1,17E+08	A	G	NO	DSCAML1	0	0	1	3,15	DS cell adh	611782	NA
chr11	1,18E+08	1,18E+08	G	A	NO	DSCAML1	nonsynony	DSCAML1:	1	3,15	DS cell adh	611782	NA
chr11	1,18E+08	1,18E+08	A	G	NO	KMT2A	nonsynony	KMT2A:N	1	6,2276	Lysine-spe	159555	Wiedeman

chr11	1,18E+08	1,18E+08	C	T	NO	KMT2A	nonsynony	KMT2A:N	1	6,2276	Lysine-spe	159555	Wiedeman
chr11	1,18E+08	1,18E+08	G	A	NO	KMT2A	nonsynony	KMT2A:N	1	6,2276	Lysine-spe	159555	Wiedeman
chr11	1,19E+08	1,19E+08	G	A	NO	KMT2A	nonsynony	KMT2A:N	1	6,2276	Lysine-spe	159555	Wiedeman
chr11	1,19E+08	1,19E+08	AGA	-	NO	DDX6	0	0	0,99964	3,7809	DEAD-box	600326	Intellectua
chr11	1,19E+08	1,19E+08	G	A	NO	HMBS	nonsynony	HMBS:NM	0,94884	0,64341	Hydroxym	609806	Leukoence
chr11	1,19E+08	1,19E+08	G	A	NO	C2CD2L	nonsynony	C2CD2L:N	0,98718	1,7789	C2 calcium	617582	NA
chr11	1,19E+08	1,19E+08	C	T	NO	C1QTNF5;	0	0	8.3565e-0	1.1564e+0	C1q- and t	608752|60	Retinal de
chr11	1,25E+08	1,25E+08	AGTC	-	NO	HEPACAM	frameshift	HEPACAM	0,86791	1,591	Hepatocyt	611642	Megalence
chr11	1,26E+08	1,26E+08	G	T	NO	STT3A	nonsynony	STT3A:NM	2,04E-03	3,6202	STT3A, sub	601134	Congenital
chr11	1,26E+08	1,26E+08	G	A	NO	STT3A	nonsynony	STT3A:NM	2,04E-03	3,6202	STT3A, sub	601134	Congenital
chr11	1,28E+08	1,28E+08	G	A	NO	ETS1	0	0	0,7816	2,5174	ETS protoo	164720	NA
chr11	1,3E+08	1,3E+08	G	A	NO	PRDM10	nonsynony	PRDM10:N	0,15304	3,2778	PR domain	618319	?Birt-Hogg
chr11	1,34E+08	1,34E+08	G	A	NO	IGSF9B	nonsynony	IGSF9B:NM	0,86551	2,5812	Immunogl	613773	NA
chr11	1,34E+08	1,34E+08	C	A	NO	IGSF9B	0	0	0,86551	2,5812	Immunogl	613773	NA
chr12	882027	882027	G	A	NO	WNK1	nonsynony	WNK1:NM	1	2,1626	WNK lysin	605232	Neuropath
chr12	897511	897511	G	A	NO	WNK1	nonsynony	WNK1:NM	1	2,1626	WNK lysin	605232	Neuropath
chr12	1566804	1566804	G	A	NO	FBXL14	stopgain	FBXL14:N	0,82537	2,9461	F-box and	609081	NA
chr12	2605076	2605076	C	T	NO	CACNA1C	0	0	1	6,4654	Calcium ch	114205	Timothy sy
chr12	3278568	3278568	G	A	NO	TSPAN9	nonsynony	TSPAN9:N	0,65913	1,3822	Tetraspani	613137	NA
chr12	6557256	6557256	G	A	NO	NOP2	stopgain	NOP2:NM	0,99996	0,63815	Nucleolar	164031	NA
chr12	6578141	6578141	A	G	NO	CHD4	0	0	1	6,3412	Chromodo	603277	Sifrim-Hitz
chr12	6602502	6602502	A	T	NO	CHD4	0	0	1	6,3412	Chromodo	603277	Sifrim-Hitz
chr12	6667909	6667909	C	-	NO	ZNF384	frameshift	ZNF384:N	0,99842	2,4396	Zinc finger	609951	NA
chr12	6954885	6954885	G	A	NO	PTPN6	nonsynony	PTPN6:NM	0,99999	2,8659	Protein tyr	176883	NA
chr12	7141249	7141249	A	T	NO	CLSTN3	nonsynony	CLSTN3:N	0,97016	1,4978	Calsynteni	611324	NA
chr12	7158061	7158061	-	C	NO	CLSTN3	frameshift	CLSTN3:N	0,97016	1,4978	Calsynteni	611324	NA
chr12	8921070	8921070	G	A	NO	PHC1	0	0	0,99938	1,9176	Polyhome	602978	?Microcep
chr12	10220551	10220551	T	C	NO	GABARAPL	nonsynony	GABARAPL	0,83532	1,1918	GABA-A re	607420	NA
chr12	15670851	15670851	C	T	NO	EPS8	0	0	0,97308	0,81071	Epidermal	600206	?Deafness
chr12	15882820	15882820	G	A	YES	STRAP	0	0	0,90143	2,0751	Serine/thr	605986	NA
chr12	26068692	26068692	T	A	NO	RASSF8	0	0	0,94158	1,1038	Ras associ	608231	NA
chr12	26123117	26123117	G	C	NO	BHLHE41	nonsynony	BHLHE41:	0,99212	0,82297	Basic helix	606200	[Short slee
chr12	26123629	26123629	C	G	YES	BHLHE41	0	0	0,99212	0,82297	Basic helix	606200	[Short slee
chr12	27309199	27309199	T	C	YES	STK38L	0	0	0,93427	2,9413	Serine/thr	615836	NA
chr12	27318022	27318022	T	A	NO	STK38L	0	0	0,93427	2,9413	Serine/thr	615836	NA
chr12	31399657	31399657	T	C	NO	DENND5B	nonsynony	DENND5B:	1	3,3888	DENN dom	617279	NA

chr12	39760257	39760257	A	G	NO	SLC2A13	0	0	0,83307	1,4822	Solute carr	611036	NA
chr12	42460156	42460156	T	-	NO	PRICKLE1	frameshift	PRICKLE1:	0,99999	1,8336	Prickle-like	608500	Epilepsy, p
chr12	44522155	44522155	G	A	NO	NELL2	nonsynony	NELL2:NM	0,99952	0,73015	Nel-like 2 (	602320	NA
chr12	45926403	45926403	G	A	NO	SCAF11	stopgain	SCAF11:N	1	0,53835	SR-related	603668	NA
chr12	46363746	46363746	A	G	NO	SLC38A2	nonsynony	SLC38A2:N	0,99709	0,79703	Solute carr	605180	NA
chr12	47791294	47791294	G	A	NO	HDAC7	nonsynony	HDAC7:NM	0,99969	2,8252	Histone de	606542	NA
chr12	47794937	47794937	G	A	NO	HDAC7	0	0	0,99969	2,8252	Histone de	606542	NA
chr12	49543273	49543274	AG	-	NO	KCNH3	0	0	0,99919	3,8464	Potassium	604527	NA
chr12	49557180	49557180	C	G	NO	KCNH3	0	0	0,99919	3,8464	Potassium	604527	NA
chr12	49636818	49636818	G	A	NO	PRPF40B	nonsynony	PRPF40B:N	0,99963	1,0695	NA	NA	NA
chr12	49793412	49793412	G	A	NO	NCKAP5L	nonsynony	NCKAP5L:	0,632	0,74066	NCK-assoc	615104	NA
chr12	50077223	50077223	G	A	NO	ASIC1	nonsynony	ASIC1:NM	0,93017	2,8206	Cation cha	602866	NA
chr12	50081111	50081133	GGGGCCA	-	NO	ASIC1	frameshift	ASIC1:NM	0,93017	2,8206	Cation cha	602866	NA
chr12	50695935	50695935	G	A	NO	DIP2B	nonsynony	DIP2B:NM	0,99924	3,1809	Disco-inter	611379	Intellectua
chr12	51480024	51480024	C	A	NO	SLC4A8	stopgain	SLC4A8:N	0,99849	4,0551	Solute carr	605024	NA
chr12	52950848	52950848	A	G	NO	KRT18	nonsynony	KRT18:NM	0,73477	1,0374	Keratin 18	148070	Cirrhosis, c
chr12	53220064	53220064	G	A	NO	RARG	stopgain	RARG:NM	0,99478	3,1331	Retinoic ac	180190	NA
chr12	53220164	53220164	T	-	NO	RARG	frameshift	RARG:NM	0,99478	3,1331	Retinoic ac	180190	NA
chr12	53486947	53486947	C	T	NO	MAP3K12	nonsynony	MAP3K12:	1	2,9896	Mitogen-a	600447	NA
chr12	56184921	56184921	G	A	NO	SMARCC2	stopgain	SMARCC2:	1	3,9085	SW1/SNF r	601734	Coffin-Siris
chr12	56254063	56254063	G	C	NO	ANKRD52	0	0	1	4,4928	NA	NA	NA
chr12	56319382	56319382	G	A	NO	PAN2	nonsynony	PAN2:NM_	0,93596	3,1762	PABP-depe	617447	NA
chr12	56322677	56322677	G	A	NO	PAN2	nonsynony	PAN2:NM_	0,93596	3,1762	PABP-depe	617447	NA
chr12	56324685	56324685	G	A	NO	PAN2	nonsynony	PAN2:NM_	0,93596	3,1762	PABP-depe	617447	NA
chr12	56349639	56349639	G	A	NO	STAT2	0	0	0,93065	2,5952	Signal tran	600556	Pseudo-TO
chr12	57093524	57093524	G	A	NO	NAB2	nonsynony	NAB2:NM	0,56734	3,0094	NGFIA-bin	602381	NA
chr12	57098501	57098501	T	C	NO	STAT6	0	0	0,98849	2,4144	Signal tran	601512	Hyper-IgE
chr12	57183847	57183847	G	A	NO	LRP1	nonsynony	LRP1:NM_	1	8,2463	Low densit	107770	?Keratosis
chr12	57199400	57199400	G	A	NO	LRP1	nonsynony	LRP1:NM_	1	8,2463	Low densit	107770	?Keratosis
chr12	57210880	57210883	GTGA	-	NO	LRP1	0	0	1	8,2463	Low densit	107770	?Keratosis
chr12	57528696	57528696	C	T	NO	MBD6	nonsynony	MBD6:NM	0,99937	-0,74132	Methyl-Cp	619458	NA
chr12	57529182	57529182	G	A	NO	MBD6	nonsynony	MBD6:NM	0,99937	-0,74132	Methyl-Cp	619458	NA
chr12	57741908	57741908	T	C	NO	AGAP2	0	0	0,99816	3,3883	ARF-GAP w	605476	NA
chr12	62648525	62648525	A	-	NO	PPM1H	stopgain	PPM1H:N	0,79216	1,5125	Protein ph	616016	NA
chr12	62788323	62788323	G	A	NO	PPM1H	stopgain	PPM1H:N	0,79216	1,5125	Protein ph	616016	NA
chr12	64043574	64043574	A	G	NO	SRGAP1	nonsynony	SRGAP1:N	0,99845	2,2072	Slit-robo G	606523	{Thyroid c

chr12	64108967	64108967	C	T	NO	SRGAP1	nonsynony	SRGAP1:N	0,99845	2,2072	Slit-robo G	606523	{Thyroid c
chr12	64425395	64425395	G	A	NO	XPOT	nonsynony	XPOT:NM_	0,99711	2,0457	Exportin, t	603180	NA
chr12	64828483	64828483	G	-	NO	TBC1D30	frameshift	TBC1D30:	0,88919	2,7005	TBC1 dom	615077	NA
chr12	64875211	64875211	G	A	NO	TBC1D30	nonsynony	TBC1D30:	0,88919	2,7005	TBC1 dom	615077	NA
chr12	66455460	66455460	G	A	NO	GRIP1	nonsynony	GRIP1:NM	0,50828	1,582	Glutamate	604597	Fraser syn
chr12	66517947	66517947	G	A	NO	GRIP1	nonsynony	GRIP1:NM	0,50828	1,582	Glutamate	604597	Fraser syn
chr12	68824552	68824552	T	G	NO	MDM2	0	0	0,99981	2,3326	MDM2 pro	164785	{Accelerat
chr12	69586765	69586765	G	A	NO	CCT2	nonsynony	CCT2:NM_	0,99853	1,4748	Chaperoni	605139	NA
chr12	69589670	69589670	A	G	NO	CCT2	nonsynony	CCT2:NM_	0,99853	1,4748	Chaperoni	605139	NA
chr12	70524534	70524534	G	A	NO	PTPRB	nonsynony	PTPRB:NM	0,50207	1,6562	Protein tyr	176882	NA
chr12	70538220	70538220	G	A	NO	PTPRB	stopgain	PTPRB:NM	0,50207	1,6562	Protein tyr	176882	NA
chr12	77831624	77831624	C	T	NO	NAV3	stopgain	NAV3:NM	1	1,3325	Neuron na	611629	NA
chr12	77941099	77941099	A	G	NO	NAV3	nonsynony	NAV3:NM	1	1,3325	Neuron na	611629	NA
chr12	78118210	78118210	C	T	NO	NAV3	stopgain	NAV3:NM	1	1,3325	Neuron na	611629	NA
chr12	78177634	78177634	G	A	NO	NAV3	stopgain	NAV3:NM	1	1,3325	Neuron na	611629	NA
chr12	78190222	78190222	A	G	NO	NAV3	0	0	1	1,3325	Neuron na	611629	NA
chr12	81284242	81284242	G	A	NO	PPFIA2	nonsynony	PPFIA2:NM	1	3,2597	PTPRF-inte	603143	NA
chr12	81295040	81295043	TAGA	-	NO	PPFIA2	0	0	1	3,2597	PTPRF-inte	603143	NA
chr12	81299298	81299298	T	C	NO	PPFIA2	0	0	1	3,2597	PTPRF-inte	603143	NA
chr12	92826290	92826290	A	C	NO	EEA1	0	0	0,92875	1,3779	Early endo	605070	NA
chr12	99825312	99825312	T	C	NO	ANKS1B	nonsynony	ANKS1B:N	0,99998	2,9457	Ankyrin re	607815	NA
chr12	1,04E+08	1,04E+08	G	A	NO	HSP90B1	nonsynony	HSP90B1:N	0,9972	2,1239	Heat-shoc	191175	NA
chr12	1,05E+08	1,05E+08	G	A	NO	C12orf75	nonsynony	C12orf75:	0,63977	0,12821	NA	|	NA
chr12	1,06E+08	1,06E+08	G	A	NO	POLR3B	nonsynony	POLR3B:N	1,68E-23	3,1978	Polymeras	614366	Leukodyst
chr12	1,07E+08	1,07E+08	G	A	NO	RFX4	nonsynony	RFX4:NM_	0,99999	2,2288	Regulatory	603958	NA
chr12	1,07E+08	1,07E+08	C	G	NO	BTBD11	nonsynony	BTBD11:N	0,99993	2,2234	NA	|	NA
chr12	1,08E+08	1,08E+08	G	A	NO	BTBD11	nonsynony	BTBD11:N	0,99993	2,2234	NA	|	NA
chr12	1,09E+08	1,09E+08	G	A	NO	SART3	nonsynony	SART3:NM	0,99661	0,80565	Spliceosom	611684	NA
chr12	1,09E+08	1,09E+08	G	A	NO	SART3	0	0	0,99661	0,80565	Spliceosom	611684	NA
chr12	1,09E+08	1,09E+08	G	A	NO	SART3	nonsynony	SART3:NM	0,99661	0,80565	Spliceosom	611684	NA
chr12	1,09E+08	1,09E+08	CACTGCTG	-	NO	TMEM119	frameshift	TMEM119	0,62503	0,29449	Transmem	618989	NA
chr12	1,09E+08	1,09E+08	G	A	NO	TMEM119	stopgain	TMEM119	0,62503	0,29449	Transmem	618989	NA
chr12	1,1E+08	1,1E+08	G	A	NO	ANAPC7	stopgain	ANAPC7:N	0,8485	2,1176	Anaphase-	606949	Ferguson-
chr12	1,11E+08	1,11E+08	C	A	YES	PPP1CC	0	0	0,95777	3,3595	Protein ph	176914	NA
chr12	1,11E+08	1,11E+08	A	C	NO	CUX2	nonsynony	CUX2:NM_	1	3,1818	Cut-like ho	610648	Developm
chr12	1,11E+08	1,11E+08	T	C	YES	ATXN2	0	0	0,85289	2,347	Ataxin-2 (A	601517	{Amyotrop

chr12	1,12E+08	1,12E+08	C	A	NO	ATXN2	nonsynony	ATXN2:NM	0,85289	2,347	Ataxin-2 (A	601517	{Amyotrop
chr12	1,12E+08	1,12E+08	GTTGCTGC	-	NO	ATXN2	frameshift	ATXN2:NM	0,85289	2,347	Ataxin-2 (A	601517	{Amyotrop
chr12	1,12E+08	1,12E+08	T	A	NO	HECTD4	0	0	1	6,9421	HECT dom	620209	Neurodeve
chr12	1,15E+08	1,15E+08	G	A	NO	TBX3	nonsynony	TBX3:NM_	0,98907	1,7339	T-box 3 (T	601621	Ulnar-mam
chr12	1,15E+08	1,15E+08	T	C	NO	TBX3	nonsynony	TBX3:NM_	0,98907	1,7339	T-box 3 (T	601621	Ulnar-mam
chr12	1,16E+08	1,16E+08	G	A	NO	MED13L	nonsynony	MED13L:N	1	3,691	Mediator c	608771	Impaired i
chr12	1,17E+08	1,17E+08	G	A	NO	NOS1	nonsynony	NOS1:NM	1	3,6832	Nitric oxid	163731	NA
chr12	1,18E+08	1,18E+08	G	A	NO	KSR2	nonsynony	KSR2:NM_	1	3,3393	Kinase sup	610737	NA
chr12	1,2E+08	1,2E+08	A	G	NO	CIT	nonsynony	CIT:NM_0	0,9997	3,8214	Citron rho	605629	Microceph
chr12	1,2E+08	1,2E+08	C	T	NO	CIT	nonsynony	CIT:NM_0	0,9997	3,8214	Citron rho	605629	Microceph
chr12	1,2E+08	1,2E+08	T	C	YES	MSI1	0	0	0,99357	2,7962	Musashi R	603328	NA
chr12	1,2E+08	1,2E+08	G	T	NO	SRSF9	0	0	0,63924	2,5277	Splicing fa	601943	NA
chr12	1,2E+08	1,2E+08	A	G	NO	DYNLL1	0	0	0,6983	2,0127	Dynein, lig	601562	NA
chr12	1,21E+08	1,21E+08	A	-	NO	UNC119B	frameshift	UNC119B:	0,78353	0,92603	UNC119 li	620513	NA
chr12	1,21E+08	1,21E+08	G	T	NO	KDM2B	0	0	1	3,4362	Lysine dem	609078	NA
chr12	1,23E+08	1,23E+08	T	C	YES	ZCCHC8	0	0	0,96584	2,0278	Zinc finger	616381	?Pulmonar
chr12	1,23E+08	1,23E+08	GGCCGAA	-	NO	PITPNM2	frameshift	PITPNM2:	1	4,13	Phosphati	608920	NA
chr12	1,23E+08	1,23E+08	C	T	NO	PITPNM2	nonsynony	PITPNM2:	1	4,13	Phosphati	608920	NA
chr12	1,24E+08	1,24E+08	G	A	NO	NCOR2	nonsynony	NCOR2:NM	1	2,0058	Nuclear re	600848	NA
chr12	1,25E+08	1,25E+08	G	A	NO	DHX37	stopgain	DHX37:NM	0,99252	1,8276	DEAH-box	617362	Neurodeve
chr12	1,32E+08	1,32E+08	T	A	YES	EP400	0	0	1	2,5604	p400 SWI2	606265	NA
chr12	1,32E+08	1,32E+08	G	A	NO	EP400	0	0	1	2,5604	p400 SWI2	606265	NA
chr12	1,32E+08	1,32E+08	T	C	NO	EP400	0	0	1	2,5604	p400 SWI2	606265	NA
chr12	1,32E+08	1,32E+08	-	G	NO	EP400	frameshift	EP400:NM	1	2,5604	p400 SWI2	606265	NA
chr13	20800827	20800827	T	C	NO	XPO4	0	0	1	4,0208	Exportin 4	611449	NA
chr13	20902718	20902718	C	-	NO	XPO4	frameshift	XPO4:NM_	1	4,0208	Exportin 4	611449	NA
chr13	20902723	20902724	GC	-	NO	XPO4	frameshift	XPO4:NM_	1	4,0208	Exportin 4	611449	NA
chr13	28014522	28014522	G	A	NO	FLT3	nonsynony	FLT3:NM_	0,60777	0,982	fms-relate	136351	Leukemia,
chr13	28368587	28368587	-	GATGATG	NO	FLT1	stoploss	FLT1:NM_	0,99983	1,4548	fms-relate	165070	NA
chr13	28368606	28368606	G	T	NO	FLT1	stopgain	FLT1:NM_	0,99983	1,4548	fms-relate	165070	NA
chr13	28672338	28672338	G	C	YES	POMP	0	0	0,90942	0,96629	Proteasom	613386	Proteasom
chr13	30231331	30231331	G	A	NO	KATNAL1	nonsynony	KATNAL1:	0,53169	1,6031	Katanin p6	614764	NA
chr13	31151173	31151173	C	A	NO	HSPH1	nonsynony	HSPH1:NM	0,99822	1,7199	Heat-shoc	610703	NA
chr13	32131719	32131719	G	A	NO	FRY	nonsynony	FRY:NM_0	1	4,1944	FRY microt	614818	NA
chr13	32147883	32147883	G	A	NO	FRY	nonsynony	FRY:NM_0	1	4,1944	FRY microt	614818	NA
chr13	35211179	35211179	G	C	NO	NBEA	nonsynony	NBEA:NM_	1	5,5108	Neurobeac	604889	Neurodeve

chr13	35645868	35645868	G	T	YES	NBEA	0	0	1	5,5108	Neurobeac	604889	Neurodeve
chr13	39012927	39012928	AG	-	NO	PROSER1	frameshift	PROSER1:	0,71143	0,13831	Proline- ad	620773	NA
chr13	42308524	42308524	G	T	NO	AKAP11	nonsynony	AKAP11:N	0,97878	0,31287	A-kinase a	604696	NA
chr13	44573166	44573166	T	C	NO	TSC22D1	nonsynony	TSC22D1:N	0,9599	-0,88133	TSC22 dom	607715	NA
chr13	45337481	45337481	G	-	NO	TPT1	frameshift	TPT1:NM_	0,80548	1,5254	Tumor pro	600763	NA
chr13	45337515	45337518	AGAC	-	NO	TPT1	frameshift	TPT1:NM_	0,80548	1,5254	Tumor pro	600763	NA
chr13	45491538	45491538	G	T	NO	COG3	nonsynony	COG3:NM	0,99993	1,5702	Componen	606975	Congenital
chr13	45509752	45509752	G	T	NO	COG3	nonsynony	COG3:NM	0,99993	1,5702	Componen	606975	Congenital
chr13	46553616	46553616	G	A	NO	LRCH1	nonsynony	LRCH1:NM	0,99552	1,388	Leucine-ric	610368	NA
chr13	46685900	46685900	T	G	NO	LRCH1	0	0	0,99552	1,388	Leucine-ric	610368	NA
chr13	49186102	49186102	G	A	NO	FNDC3A	nonsynony	FNDC3A:N	0,99998	1,5293	Fibronecti	615794	NA
chr13	49188592	49188592	C	T	NO	FNDC3A	nonsynony	FNDC3A:N	0,99998	1,5293	Fibronecti	615794	NA
chr13	57633682	57633682	G	C	NO	PCDH17	nonsynony	PCDH17:N	0,64056	1,345	Protocadh	611760	NA
chr13	61411944	61411944	C	-	NO	PCDH20	frameshift	PCDH20:N	0,86222	0,70607	Protocadh	614449	NA
chr13	61415083	61415083	G	-	NO	PCDH20	frameshift	PCDH20:N	0,86222	0,70607	Protocadh	614449	NA
chr13	66631254	66631254	G	A	NO	PCDH9	nonsynony	PCDH9:NM	0,80865	2,3295	Protocadh	603581	NA
chr13	71475180	71475180	G	A	NO	DACH1	nonsynony	DACH1:NM	0,99954	2,1853	Dachshun	603803	NA
chr13	71573439	71573439	G	A	NO	DACH1	stopgain	DACH1:NM	0,99954	2,1853	Dachshun	603803	NA
chr13	77719644	77719644	C	T	NO	SLAIN1	nonsynony	SLAIN1:NM	0,90094	1,224	SLAIN mot	610491	NA
chr13	77753290	77753290	G	A	NO	SLAIN1	nonsynony	SLAIN1:NM	0,90094	1,224	SLAIN mot	610491	NA
chr13	77760891	77760891	G	A	NO	SLAIN1	nonsynony	SLAIN1:NM	0,90094	1,224	SLAIN mot	610491	NA
chr13	79539677	79539677	T	G	NO	NDFIP2	0	0	0,52743	0,61857	NEDD4 fa	610041	NA
chr13	79543600	79543600	G	T	NO	NDFIP2	nonsynony	NDFIP2:N	0,52743	0,61857	NEDD4 fa	610041	NA
chr13	96833089	96833089	A	-	NO	HS6ST3	frameshift	HS6ST3:N	0,99001	1,191	Heparan s	609401	NA
chr13	97434694	97434694	G	T	NO	RAP2A	nonsynony	RAP2A:NM	0,85068	2,5609	RAP2, me	179540	NA
chr13	98829405	98829405	A	C	NO	DOCK9	nonsynony	DOCK9:NM	1	3,0983	Zizimin 1 (	607325	NA
chr13	99554323	99554323	G	A	NO	TM9SF2	nonsynony	TM9SF2:N	1	3,2749	Transmem	604678	NA
chr13	99971174	99971193	GGGGAGG	-	NO	ZIC5	frameshift	ZIC5:NM_0	0,76534	0,96265	Zic family,	617896	NA
chr13	99971262	99971262	G	T	NO	ZIC5	stopgain	ZIC5:NM_0	0,76534	0,96265	Zic family,	617896	NA
chr13	99982602	99982602	C	T	NO	ZIC2	nonsynony	ZIC2:NM_0	0,97362	3,2124	ZIC family,	603073	Holoprose
chr13	1,07E+08	1,07E+08	G	C	NO	ARGLU1	nonsynony	ARGLU1:N	0,86183	2,4801	Arginine- a	614046	NA
chr13	1,09E+08	1,09E+08	GTTAGAG	-	NO	MYO16	frameshift	MYO16:N	0,99851	1,0261	Myosin XV	615479	NA
chr13	1,1E+08	1,1E+08	T	C	YES	COL4A1	0	0	1	3,0194	Collagen IV	120130	?Retinal ar
chr14	22842463	22842463	G	A	NO	MMP14	nonsynony	MMP14:N	0,99522	1,4844	Matrix me	600754	Wincheste
chr14	22981801	22981801	G	A	NO	AJUBA	nonsynony	AJUBA:NM	0,79717	2,2495	AJUBA LIM	609066	NA
chr14	23061592	23061592	G	A	NO	ACIN1	nonsynony	ACIN1:NM	1	2,2011	Acinus (AC	604562	NA

chr14	23062417	23062417	G	A	NO	ACIN1	nonsynony	ACIN1:NM	1	2,2011	Acinus (AC	604562	NA
chr14	24164912	24164912	C	T	NO	IRF9	stopgain	IRF9:NM_	0,60773	1,5724	Interferon	147574	Immunode
chr14	24373769	24373769	G	C	NO	NFATC4	nonsynony	NFATC4:N	0,8679	1,4429	Nuclear fa	602699	NA
chr14	28768031	28768031	A	G	NO	FOXG1	nonsynony	FOXG1:NM	0,94355	3,4897	Forkhead	164874	Rett syndr
chr14	30929225	30929225	G	A	NO	STRN3	stopgain	STRN3:NM	0,82925	0,93473	Striatin, ca	614766	NA
chr14	32154621	32154621	G	C	NO	ARHGAP5	nonsynony	ARHGAP5:	0,99862	2,2104	RHO GTPa	602680	NA
chr14	32545438	32545438	A	-	NO	AKAP6	frameshift	AKAP6:NM	0,99983	-4,27E-02	A-kinase a	604691	NA
chr14	32678464	32678464	G	A	NO	AKAP6	0	0	0,99983	-4,27E-02	A-kinase a	604691	NA
chr14	33367200	33367200	C	T	NO	NPAS3	stopgain	NPAS3:NM	0,99974	3,1383	Neuronal P	609430	NA
chr14	33774491	33774491	G	A	NO	NPAS3	nonsynony	NPAS3:NM	0,99974	3,1383	Neuronal P	609430	NA
chr14	38210558	38210558	-	GCTCTGAG	NO	SSTR1	stopgain	SSTR1:NM	0,7473	1,2046	Somatosta	182451	NA
chr14	49894186	49894186	T	A	NO	ARF6	stopgain	ARF6:NM_	0,71536	2,7765	ADP-ribos	600464	NA
chr14	50456579	50456579	G	A	NO	MAP4K5	nonsynony	MAP4K5:N	0,99469	2,0578	Mitogen-a	604923	NA
chr14	50485641	50485641	G	A	NO	MAP4K5	nonsynony	MAP4K5:N	0,99469	2,0578	Mitogen-a	604923	NA
chr14	52872822	52872822	G	A	NO	FERMT2	nonsynony	FERMT2:N	0,99999	1,9559	FERM dom	607746	NA
chr14	53062980	53062980	G	A	NO	DDHD1	stopgain	DDHD1:N	0,902	1,1808	DDHD dom	614603	Spastic par
chr14	53152893	53152893	G	-	NO	DDHD1	frameshift	DDHD1:N	0,902	1,1808	DDHD dom	614603	Spastic par
chr14	54776460	54776460	G	A	NO	SAMD4A	nonsynony	SAMD4A:N	0,99958	1,8422	Sterile alp	610747	NA
chr14	54784441	54784441	C	T	NO	SAMD4A	stopgain	SAMD4A:N	0,99958	1,8422	Sterile alp	610747	NA
chr14	57246863	57246863	C	G	NO	EXOC5	0	0	0,99991	2,3445	Exocyst co	604469	NA
chr14	58267520	58267520	T	C	NO	PSMA3	nonsynony	PSMA3:NM	0,98462	1,7604	Proteasom	176843	NA
chr14	60292472	60292472	A	G	NO	PPM1A	nonsynony	PPM1A:N	0,99834	2,5758	Protein ph	606108	NA
chr14	64521580	64521580	T	-	NO	ZBTB1	frameshift	ZBTB1:NM	0,99407	2,9676	Zinc finger	616578	NA
chr14	64541057	64541057	G	T	NO	HSPA2	nonsynony	HSPA2:NM	4,86E-02	3,7216	Heat-shoc	140560	NA
chr14	64772678	64772678	C	A	NO	SPTB	stopgain	SPTB:NM_	1	0,75083	Spectrin, b	182870	Anemia, n
chr14	64779165	64779165	T	G	NO	SPTB	nonsynony	SPTB:NM_	1	0,75083	Spectrin, b	182870	Anemia, n
chr14	64787165	64787165	C	T	NO	SPTB	0	0	1	0,75083	Spectrin, b	182870	Anemia, n
chr14	64799829	64799829	G	A	NO	SPTB	nonsynony	SPTB:NM_	1	0,75083	Spectrin, b	182870	Anemia, n
chr14	68882589	68882589	G	A	NO	ACTN1	nonsynony	ACTN1:NM	0,99997	3,3621	Actinin, al	102575	Bleeding d
chr14	68904669	68904669	A	C	NO	ACTN1	nonsynony	ACTN1:NM	0,99997	3,3621	Actinin, al	102575	Bleeding d
chr14	69054903	69054903	G	A	NO	DCAF5	stopgain	DCAF5:NM	1	2,8802	DDB1- and	603812	NA
chr14	69424097	69424097	C	T	NO	SLC39A9	stopgain	SLC39A9:N	0,72946	1,9744	Solute carr	619116	NA
chr14	69441977	69441977	G	A	NO	SLC39A9	0	0	0,72946	1,9744	Solute carr	619116	NA
chr14	69768634	69768634	G	T	NO	SRSF5	nonsynony	SRSF5:NM	0,69255	1,3112	Splicing fa	600914	NA
chr14	70375717	70375717	G	A	NO	SYNJ2BP;S	nonsynony	SYNJ2BP-C	5.1218e-0	7.4506e-0	Synaptoja	609411|	NA
chr14	71705296	71705296	C	G	NO	SIPA1L1	nonsynony	SIPA1L1:N	1	2,9815	SIPA1-like	617504	NA

chr14	73112165	73112165	G	A	NO	RBM25	nonsynony	RBM25:N	1	4,3087	RNA-bindi	612427	NA
chr14	74811706	74811706	G	A	NO	YLPM1	nonsynony	YLPM1:NM	0,99965	0,76788	YLP motif-	619766	NA
chr14	74818258	74818258	C	T	NO	YLPM1	nonsynony	YLPM1:NM	0,99965	0,76788	YLP motif-	619766	NA
chr14	75971584	75971584	G	A	NO	TGFB3	nonsynony	TGFB3:NM	0,98727	1,6624	Transform	190230	Arrhythmo
chr14	78968335	78968335	G	A	NO	NRXN3	nonsynony	NRXN3:NM	1	3,438	Neurexin 3	600567	NA
chr14	79861215	79861215	C	T	NO	NRXN3	stopgain	NRXN3:NM	1	3,438	Neurexin 3	600567	NA
chr14	88563646	88563646	C	T	NO	ZC3H14	0	0	0,88291	1,4514	Zinc finger	613279	Intellectua
chr14	88609707	88609707	G	T	NO	ZC3H14	0	0	0,88291	1,4514	Zinc finger	613279	Intellectua
chr14	90260214	90260214	A	G	NO	PSMC1	0	0	0,99945	3,9473	Proteasom	602706	?Birk-Ahar
chr14	90541565	90541565	G	A	NO	TTC7B	nonsynony	TTC7B:NM	0,99998	2,0937	Tetratricop	620060	NA
chr14	90578134	90578134	G	A	NO	TTC7B	nonsynony	TTC7B:NM	0,99998	2,0937	Tetratricop	620060	NA
chr14	93586760	93586760	G	A	NO	UNC79	nonsynony	UNC79:NM	1	4,16	unc-79 ho	616884	NA
chr14	94057871	94057871	G	A	NO	DDX24	nonsynony	DDX24:NM	0,68465	8,74E-02	DEAD-box	606181	NA
chr14	94060588	94060588	G	A	NO	DDX24	nonsynony	DDX24:NM	0,68465	8,74E-02	DEAD-box	606181	NA
chr14	99515287	99515287	G	A	NO	CCDC85C	nonsynony	CCDC85C:	0,7501	1,3466	NA	NA	NA
chr14	99894627	99894629	AGA	-	NO	EML1	frameshift	EML1:NM	0,99608	2,4519	Echinoder	602033	Band hete
chr14	99897162	99897162	G	A	NO	EML1	nonsynony	EML1:NM	0,99608	2,4519	Echinoder	602033	Band hete
chr14	1,02E+08	1,02E+08	A	G	NO	DYNC1H1	nonsynony	DYNC1H1:	1	10,967	Dynein, cy	600112	Charcot-M
chr14	1,02E+08	1,02E+08	-	GTAAT	NO	DYNC1H1	frameshift	DYNC1H1:	1	10,967	Dynein, cy	600112	Charcot-M
chr14	1,02E+08	1,02E+08	TCTT	-	NO	HSP90AA1	frameshift	HSP90AA1	0,86025	1,0368	Heat-shoc	140571	NA
chr14	1,02E+08	1,02E+08	AC	-	NO	TECPR2	frameshift	TECPR2:N	0,58003	1,7378	Tectonin b	615000	Neuropath
chr14	1,02E+08	1,02E+08	G	A	NO	TECPR2	0	0	0,58003	1,7378	Tectonin b	615000	Neuropath
chr14	1,03E+08	1,03E+08	C	T	NO	TRAF3	0	0	0,99869	2,9892	TNF recep	601896	{?Encepha
chr14	1,04E+08	1,04E+08	A	G	NO	KLC1	nonsynony	KLC1:NM_	0,37095	3,2232	Kinesin, lig	600025	NA
chr14	1,04E+08	1,04E+08	T	C	NO	KLC1	nonsynony	KLC1:NM_	0,37095	3,2232	Kinesin, lig	600025	NA
chr14	1,04E+08	1,04E+08	G	A	NO	PPP1R13B	nonsynony	PPP1R13B	0,99811	1,5265	Protein ph	606455	NA
chr14	1,04E+08	1,04E+08	A	G	NO	PPP1R13B	0	0	0,99811	1,5265	Protein ph	606455	NA
chr14	1,04E+08	1,04E+08	C	-	NO	PPP1R13B	frameshift	PPP1R13B	0,99811	1,5265	Protein ph	606455	NA
chr14	1,05E+08	1,05E+08	AG	-	NO	TMEM179	frameshift	TMEM179	0,67923	0,93064	NA	NA	NA
chr14	1,05E+08	1,05E+08	C	T	NO	INF2	stopgain	INF2:NM_	0,97346	2,3077	Inverted fo	610982	Glomerulo
chr14	1,05E+08	1,05E+08	G	C	YES	INF2	0	0	0,97346	2,3077	Inverted fo	610982	Glomerulo
chr14	1,05E+08	1,05E+08	CAC	-	NO	CEP170B	0	0	0,99671	0,87788	Centrosom	620251	NA
chr15	22881890	22881890	A	C	NO	CYFIP1	nonsynony	CYFIP1:NM	0,96816	2,6545	Cytoplasm	606322	NA
chr15	22903707	22903707	G	A	NO	CYFIP1	nonsynony	CYFIP1:NM	0,96816	2,6545	Cytoplasm	606322	NA
chr15	22910764	22910764	A	G	NO	CYFIP1	nonsynony	CYFIP1:NM	0,96816	2,6545	Cytoplasm	606322	NA
chr15	25405480	25405480	C	T	NO	UBE3A	nonsynony	UBE3A:NM	0,9998	4,4026	Ubiquitin	601623	Angelman

chr15	28121341	28121341	G	A	NO	HERC2	0	0	1	4,4237	HECT dom	605837	Intellectua
chr15	28233562	28233562	C	T	YES	HERC2	0	0	1	4,4237	HECT dom	605837	Intellectua
chr15	28995809	28995809	A	G	NO	APBA2	0	0	0,74272	1,4594	Amyloid b	602712	NA
chr15	29761215	29761215	G	A	NO	TJP1	nonsynony	TJP1:NM_	1	1,5417	Tight junct	601009	NA
chr15	33566802	33566802	A	G	NO	RYR3	0	0	0,70566	1,8352	Ryanodine	180903	Congenital
chr15	33581542	33581542	G	C	NO	RYR3	nonsynony	RYR3:NM_	0,70566	1,8352	Ryanodine	180903	Congenital
chr15	33634700	33634700	G	A	NO	RYR3	nonsynony	RYR3:NM_	0,70566	1,8352	Ryanodine	180903	Congenital
chr15	33736270	33736270	G	A	NO	RYR3	nonsynony	RYR3:NM_	0,70566	1,8352	Ryanodine	180903	Congenital
chr15	33826695	33826695	G	A	NO	RYR3	nonsynony	RYR3:NM_	0,70566	1,8352	Ryanodine	180903	Congenital
chr15	33827280	33827280	G	A	NO	RYR3	nonsynony	RYR3:NM_	0,70566	1,8352	Ryanodine	180903	Congenital
chr15	33853091	33853091	T	C	NO	RYR3	0	0	0,70566	1,8352	Ryanodine	180903	Congenital
chr15	33853681	33853681	T	C	NO	RYR3	nonsynony	RYR3:NM_	0,70566	1,8352	Ryanodine	180903	Congenital
chr15	34225549	34225549	C	T	NO	EMC4	nonsynony	EMC4:NM	0,86097	1,2827	ER membr	616245	NA
chr15	39584360	39584360	G	A	NO	THBS1	nonsynony	THBS1:NM	0,99985	2,7177	Thrombos	188060	NA
chr15	39589066	39589066	C	T	NO	THBS1	stopgain	THBS1:NM	0,99985	2,7177	Thrombos	188060	NA
chr15	40036374	40036374	-	CTGTTGTT	NO	SRP14	frameshift	SRP14:NM	0,87497	0,84329	Signal reco	600708	NA
chr15	40458522	40458522	C	T	NO	BAHD1	stopgain	BAHD1:NM	0,98547	1,308	BROMO-a	613880	NA
chr15	42528419	42528419	C	T	NO	SNAP23	nonsynony	SNAP23:N	0,92412	0,83495	Synaptoso	602534	NA
chr15	43415696	43415696	G	A	NO	TP53BP1	nonsynony	TP53BP1:N	0,99985	1,8797	Tumor pro	605230	NA
chr15	43521113	43521113	G	T	NO	MAP1A	0	0	1	2,0101	Microtubu	600178	NA
chr15	43756751	43756751	G	C	NO	PDIA3	nonsynony	PDIA3:NM	0,99608	0,68368	Glucose re	602046	NA
chr15	47763944	47763944	G	A	NO	SEMA6D	nonsynony	SEMA6D:N	0,93948	1,0786	Semaphor	609295	NA
chr15	48159783	48159783	G	A	NO	MYEF2	nonsynony	MYEF2:NM	0,63452	1,5266	Myelin exp	619395	NA
chr15	48483813	48483813	C	T	NO	FBN1	0	0	1	5,0644	Fibrillin-1 (	134797	Geleophys
chr15	50424654	50424654	G	A	NO	USP8	0	0	1	1,8666	Ubiquitin-	603158	Pituitary a
chr15	50490260	50490260	A	G	NO	USP8	0	0	1	1,8666	Ubiquitin-	603158	Pituitary a
chr15	51966037	51966044	GCTTATCT	-	NO	LEO1	frameshift	LEO1:NM_	0,87094	2,3445	LEO1 RNA	610507	NA
chr15	51971741	51971741	G	A	NO	LEO1	nonsynony	LEO1:NM_	0,87094	2,3445	LEO1 RNA	610507	NA
chr15	56094235	56094235	G	A	NO	RFX7	nonsynony	RFX7:NM_	1	1,5996	Regulatory	612660	Intellectua
chr15	58894585	58894585	A	G	NO	SLTM	0	0	0,99785	2,5265	NA	NA	NA
chr15	60005096	60005096	G	A	NO	FOXB1	nonsynony	FOXB1:NM	0,90634	1,4834	Forkhead	619961	NA
chr15	62694329	62694329	A	T	NO	TLN2	nonsynony	TLN2:NM_	0,99231	1,4106	Talin 2 (TL	607349	NA
chr15	62740644	62740644	G	-	NO	TLN2	frameshift	TLN2:NM_	0,99231	1,4106	Talin 2 (TL	607349	NA
chr15	62755590	62755590	G	A	NO	TLN2	nonsynony	TLN2:NM_	0,99231	1,4106	Talin 2 (TL	607349	NA
chr15	63656362	63656362	C	A	NO	HERC1	0	0	1	4,9558	HECT dom	605109	Macrocep
chr15	63675100	63675100	G	A	NO	HERC1	nonsynony	HERC1:NM	1	4,9558	HECT dom	605109	Macrocep

chr15	63774689	63774689	C	T	NO	HERC1	0	0	1	4,9558	HECT dom	605109	Macrocep
chr15	64136403	64136403	G	A	NO	SNX1	nonsynony	SNX1:NM_	0,93179	0,80383	Sorting ne	601272	NA
chr15	66501390	66501390	A	G	NO	RPL4	nonsynony	RPL4:NM_	0,99984	1,2717	Ribosomal	180479	NA
chr15	67138095	67138095	G	-	NO	SMAD3	frameshift	SMAD3:N	0,79796	3,4782	SMAD fam	603109	Loeys-Diet
chr15	70057663	70057663	G	A	NO	TLE3	0	0	1	3,7217	TLE family	600190	NA
chr15	70095606	70095606	T	C	NO	TLE3	nonsynony	TLE3:NM_	1	3,7217	TLE family	600190	NA
chr15	70096676	70096676	C	T	YES	TLE3	0	0	1	3,7217	TLE family	600190	NA
chr15	72209668	72209668	C	A	NO	PKM	0	0	0,8549	2,0911	Pyruvate k	179050	NA
chr15	72250917	72250917	A	T	NO	PARP6	nonsynony	PARP6:NM	0,99996	3,176	Poly(ADP-r	619439	NA
chr15	72265472	72265472	C	T	NO	PARP6	nonsynony	PARP6:NM	0,99996	3,176	Poly(ADP-r	619439	NA
chr15	73947137	73947137	G	A	NO	LOXL1	nonsynony	LOXL1:NM	0,88677	1,125	Lysyl oxida	153456	{Exfoliatio
chr15	74801107	74801107	G	A	NO	CSK	0	0	0,99977	3,978	c-src tyros	124095	NA
chr15	77478288	77478290	TCC	-	NO	HMG20A	0	0	0,67769	1,4125	High mobi	605534	NA
chr15	77479325	77479325	A	G	NO	HMG20A	0	0	0,67769	1,4125	High mobi	605534	NA
chr15	78487816	78487816	A	G	NO	IREB2	nonsynony	IREB2:NM	0,99991	2,033	Iron-respo	147582	Neurodeg
chr15	82663559	82663559	C	G	YES	AP3B2	0	0	0,77198	2,9221	Adaptor-re	602166	Developm
chr15	82688453	82688454	GG	-	NO	AP3B2	frameshift	AP3B2:NM	0,77198	2,9221	Adaptor-re	602166	Developm
chr15	83257570	83257570	G	A	NO	BNC1	nonsynony	BNC1:NM_	0,96479	1,2039	Basonuclin	601930	?Prematur
chr15	85708021	85708021	T	C	NO	AKAP13	nonsynony	AKAP13:N	0,92909	-1,6419	A-kinase a	604686	NA
chr15	85719201	85719201	G	A	NO	AKAP13	nonsynony	AKAP13:N	0,92909	-1,6419	A-kinase a	604686	NA
chr15	85736090	85736090	G	C	NO	AKAP13	nonsynony	AKAP13:N	0,92909	-1,6419	A-kinase a	604686	NA
chr15	88838986	88838986	C	T	NO	ACAN	nonsynony	ACAN:NM	1	0,20716	Aggrecan (	155760	?Spondylo
chr15	90084812	90084812	G	A	NO	IDH2	0	0	0,88271	1,3418	Isocitrate	147650	D-2-hydro
chr15	90084911	90084911	A	T	NO	IDH2	0	0	0,88271	1,3418	Isocitrate	147650	D-2-hydro
chr15	90087258	90087258	T	C	NO	IDH2	nonsynony	IDH2:NM_	0,88271	1,3418	Isocitrate	147650	D-2-hydro
chr15	90388381	90388381	C	T	NO	IQGAP1	nonsynony	IQGAP1:N	0,99754	2,4441	RASGAP-li	603379	NA
chr15	90472931	90472931	G	A	NO	IQGAP1	nonsynony	IQGAP1:N	0,99754	2,4441	RASGAP-li	603379	NA
chr15	90487499	90487499	A	G	NO	IQGAP1	nonsynony	IQGAP1:N	0,99754	2,4441	RASGAP-li	603379	NA
chr15	90604407	90604407	G	A	NO	CRTC3	nonsynony	CRTC3:NM	0,86585	0,4005	CREB-regu	608986	NA
chr15	90617954	90617954	T	G	NO	CRTC3	nonsynony	CRTC3:NM	0,86585	0,4005	CREB-regu	608986	NA
chr15	90638735	90638735	G	T	NO	CRTC3	nonsynony	CRTC3:NM	0,86585	0,4005	CREB-regu	608986	NA
chr15	96332626	96332626	G	A	NO	NR2F2	0	0	0,98972	3,5964	Nuclear re	107773	46XX sex r
chr15	98891610	98891610	C	A	NO	IGF1R	stopgain	IGF1R:NM	0,96799	2,7286	Insulin-like	147370	Insulin-like
chr15	99690385	99690385	G	A	NO	MEF2A	nonsynony	MEF2A:NM	0,98706	1,5438	MADS box	600660	{Coronary
chr16	520766	520766	G	A	NO	RAB11FIP3	nonsynony	RAB11FIP3	0,9982	1,2666	RAB11 fam	608738	NA
chr16	553410	553410	G	A	NO	CAPN15	nonsynony	CAPN15:N	0,96857	1,7722	Calpain 15	603267	Oculogastr

chr16	1398905	1398905	G	-	NO	UNKL	frameshift	UNKL:NM_	0,81729	0,81625	Unkempt f	617463	NA
chr16	1729575	1729599	GGAGGTA	-	NO	MAPK8IP3	frameshift	MAPK8IP3	1	2,8844	Mitogen-a	605431	Neurodeve
chr16	1729583	1729583	G	A	NO	MAPK8IP3	0	0	1	2,8844	Mitogen-a	605431	Neurodeve
chr16	1762946	1762946	G	A	NO	MAPK8IP3	nonsynony	MAPK8IP3	1	2,8844	Mitogen-a	605431	Neurodeve
chr16	1998258	1998258	G	A	NO	ZNF598	stopgain	ZNF598:N	0,54824	-0,92384	Zinc finger	617508	NA
chr16	2077692	2077692	C	T	NO	TSC2	nonsynony	TSC2:NM_	1	-0,33027	Tuberin (tu	191092	Lymphang
chr16	2084229	2084229	C	T	NO	TSC2	nonsynony	TSC2:NM_	1	-0,33027	Tuberin (tu	191092	Lymphang
chr16	2106028	2106028	C	T	NO	PKD1	0	0	1	-4,3234	Polycystin-	601313	Polycystic
chr16	2106599	2106599	G	A	NO	PKD1	stopgain	PKD1:NM_	1	-4,3234	Polycystin-	601313	Polycystic
chr16	2181018	2181018	G	A	NO	CASKIN1	stopgain	CASKIN1:N	1	1,7876	CASK-inter	612184	NA
chr16	3731217	3731217	G	A	NO	CREBBP	nonsynony	CREBBP:N	1	3,8991	CREB bindi	600140	Menke-He
chr16	3740395	3740395	T	C	NO	CREBBP	0	0	1	3,8991	CREB bindi	600140	Menke-He
chr16	4260220	4260220	-	GGGG	NO	TFAP4	frameshift	TFAP4:NM	0,99731	1,5962	Transcripti	600743	NA
chr16	4513068	4513068	G	A	NO	CDIP1	0	0	0,87001	0,43798	Cell death-	610503	NA
chr16	4852882	4852882	C	G	NO	UBN1	0	0	0,99995	-0,78925	Ubinuclein	609771	NA
chr16	9092918	9092918	C	G	NO	C16orf72	0	0	0,98049	1,9479	NA	NA	NA
chr16	9840746	9840746	G	A	NO	GRIN2A	nonsynony	GRIN2A:N	0,99998	2,8278	Glutamate	138253	Epilepsy, f
chr16	11915461	11915462	GG	-	NO	GSPT1	frameshift	GSPT1:NM	0,99986	3,3179	G1 to S ph	139259	NA
chr16	11915478	11915479	GT	-	NO	GSPT1	frameshift	GSPT1:NM	0,99986	3,3179	G1 to S ph	139259	NA
chr16	15708816	15708816	G	A	NO	MYH11	stopgain	MYH11:N	0,77299	1,4389	Myosin, he	160745	Megacysti
chr16	15721591	15721591	T	C	NO	MYH11	nonsynony	MYH11:N	0,77299	1,4389	Myosin, he	160745	Megacysti
chr16	15750140	15750140	T	-	NO	MYH11	frameshift	MYH11:N	0,77299	1,4389	Myosin, he	160745	Megacysti
chr16	15750191	15750191	G	A	NO	MYH11	nonsynony	MYH11:N	0,77299	1,4389	Myosin, he	160745	Megacysti
chr16	15778831	15778831	G	A	NO	MYH11	nonsynony	MYH11:N	0,77299	1,4389	Myosin, he	160745	Megacysti
chr16	17134474	17134474	G	A	NO	XYLT1	stopgain	XYLT1:NM	0,92418	0,58588	Xylosyltran	608124	Desbuquoi
chr16	18838049	18838049	G	A	NO	SMG1	nonsynony	SMG1:NM	1	3,2976	SMG1 non	607032	NA
chr16	21597286	21597286	A	T	NO	METTL9	stopgain	METTL9:N	0,80352	2,6186	Methyltra	609388	NA
chr16	21599791	21599791	A	T	NO	METTL9	nonsynony	METTL9:N	0,80352	2,6186	Methyltra	609388	NA
chr16	23680940	23680940	G	A	NO	PLK1	nonsynony	PLK1:NM_	0,97851	2,5236	Polo-like k	602098	NA
chr16	28101573	28101573	G	A	NO	XPO6	nonsynony	XPO6:NM_	1	3,4849	Exportin 6	608411	NA
chr16	28169747	28169747	C	T	NO	XPO6	0	0	1	3,4849	Exportin 6	608411	NA
chr16	29817010	29817010	A	G	NO	PAGR1	0	0	0,74265	0,63229	PAXIP1-ass	612033	NA
chr16	29819551	29819551	C	G	NO	PAGR1	0	0	0,74265	0,63229	PAXIP1-ass	612033	NA
chr16	30710078	30710078	C	T	NO	SRCAP	stopgain	SRCAP:NM	1	2,1272	SNF2-relat	611421	Developm
chr16	30979979	30980006	CCCGGCG	-	NO	SETD1A	frameshift	SETD1A:N	1	1,8312	SET domai	611052	Epilepsy, e
chr16	31190398	31190398	C	T	NO	FUS	nonsynony	FUS:NM_0	0,9993	2,2053	FUS RNA-b	137070	Amyotrop

chr16	46683504	46683504	T	C	NO	VPS35	0	0	0,96921	3,5321	VPS35 retr	601501	{Parkinson
chr16	50340088	50340088	T	C	YES	BRD7	0	0	0,99774	0,48921	Bromodom	618489	NA
chr16	53490301	53490301	G	C	NO	RBL2	0	0	0,99835	1,2253	RB transcr	180203	Brunet-Wa
chr16	54285318	54285318	T	A	NO	IRX3	nonsynony	IRX3:NM_	0,89165	0,58069	Iroquois h	612985	NA
chr16	55489788	55489788	G	A	NO	MMP2	nonsynony	MMP2:NM	0,83878	0,70987	Matrix me	120360	Multicentr
chr16	56935396	56935396	T	A	NO	HERPUD1	0	0	0,99582	0,80041	Homocyst	608070	NA
chr16	64982306	64982306	C	A	NO	CDH11	0	0	0,9999	1,5868	Cadherin-1	600023	Teebi hype
chr16	66728211	66728211	G	A	NO	DYNC1LI2	nonsynony	DYNC1LI2:	0,98346	1,8932	Dynein, cy	611406	NA
chr16	67877354	67877354	G	A	NO	EDC4	nonsynony	EDC4:NM_	0,1061	3,2857	Enhancer	606030	NA
chr16	69568494	69568494	G	T	YES	NFAT5	0	0	1	3,1341	Nuclear fa	604708	NA
chr16	71753847	71753847	G	A	NO	AP1G1	nonsynony	AP1G1:NM	0,99998	2,9839	Adaptor-re	603533	Usmani-Ri
chr16	72798478	72798478	G	A	NO	ZFHX3	nonsynony	ZFHX3:NM	1	-1,2345	Zinc finger	104155	Prostate c
chr16	74462194	74462194	C	T	NO	GLG1	nonsynony	GLG1:NM_	1	2,0663	Golgi appa	600753	NA
chr16	74503528	74503528	T	C	NO	GLG1	0	0	1	2,0663	Golgi appa	600753	NA
chr16	75229930	75229930	G	A	NO	BCAR1	stopgain	BCAR1:NM	0,99808	-0,59449	BCAR1 sca	602941	NA
chr16	75236853	75236863	GCCCTGGC	-	NO	BCAR1	frameshift	BCAR1:NM	0,99808	-0,59449	BCAR1 sca	602941	NA
chr16	75242952	75242952	G	A	NO	BCAR1	nonsynony	BCAR1:NM	0,99808	-0,59449	BCAR1 sca	602941	NA
chr16	81893771	81893771	G	A	NO	PLCG2	nonsynony	PLCG2:NM	0,99562	0,81508	Phospholi	600220	Autoinflam
chr16	81921269	81921269	G	A	NO	PLCG2	nonsynony	PLCG2:NM	0,99562	0,81508	Phospholi	600220	Autoinflam
chr16	81934517	81934517	C	-	NO	PLCG2	frameshift	PLCG2:NM	0,99562	0,81508	Phospholi	600220	Autoinflam
chr16	84733490	84733490	G	C	NO	USP10	nonsynony	USP10:NM	0,99411	-0,67408	Ubiquitin-	609818	NA
chr16	84759475	84759475	A	G	NO	USP10	0	0	0,99411	-0,67408	Ubiquitin-	609818	NA
chr16	85657520	85657520	G	T	NO	GSE1	nonsynony	GSE1:NM_	1	-3,3094	GSE1 coile	616886	NA
chr16	85665014	85665014	G	C	YES	GSE1	0	0	1	-3,3094	GSE1 coile	616886	NA
chr16	85788969	85788969	C	T	NO	EMC8	0	0	0,85233	0,6232	ER membr	604886	NA
chr16	87411652	87411652	G	C	NO	ZCCHC14	stopgain	ZCCHC14:	0,97852	0,81288	Zinc finger	620697	NA
chr16	88018670	88018670	A	G	NO	BANP	0	0	0,99388	2,4265	BTG3-asso	611564	NA
chr16	88428923	88428923	C	-	NO	ZNF469	frameshift	ZNF469:N	0,71866	0,77372	Zinc finger	612078	Brittle cor
chr16	88439180	88439180	C	-	NO	ZNF469	frameshift	ZNF469:N	0,71866	0,77372	Zinc finger	612078	Brittle cor
chr16	89291188	89291188	G	C	NO	ANKRD11	0	0	1	-0,55363	Ankyrin re	611192	KBG syndr
chr17	1538889	1538889	G	A	NO	PITPNA	stopgain	PITPNA:N	0,96513	2,4428	Phosphoti	600174	NA
chr17	1673741	1673741	C	A	NO	PRPF8	0	0	1	8,2838	Pre-mRNA	607300	Retinitis pi
chr17	1678758	1678758	T	C	NO	PRPF8	0	0	1	8,2838	Pre-mRNA	607300	Retinitis pi
chr17	2667200	2667200	-	A	YES	PAFAH1B1	0	0	0,99995	3,5284	Platelet-ac	601545	Subcortica
chr17	2964049	2964049	G	A	NO	RAP1GAP2	nonsynony	RAP1GAP2	0,96928	2,2279	RAP1 GTPa	618714	NA
chr17	4034163	4034163	G	A	NO	ZZEF1	nonsynony	ZZEF1:NM	0,83468	1,0464	Zinc finger	619459	NA

chr17	4074189	4074189	G	A	NO	ZZEF1	nonsynony	ZZEF1:NM	0,83468	1,0464	Zinc finger	619459	NA
chr17	4074229	4074229	C	T	NO	ZZEF1	stopgain	ZZEF1:NM	0,83468	1,0464	Zinc finger	619459	NA
chr17	4142606	4142606	A	G	NO	ZZEF1	nonsynony	ZZEF1:NM	0,83468	1,0464	Zinc finger	619459	NA
chr17	4209901	4209901	G	A	NO	ANKFY1	nonsynony	ANKFY1:N	0,95629	2,4638	Ankyrin re	607927	NA
chr17	4890946	4890946	C	A	NO	MINK1	0	0	1	4,0825	Misshapen	609426	NA
chr17	4897265	4897265	G	A	NO	MINK1	nonsynony	MINK1:NM	1	4,0825	Misshapen	609426	NA
chr17	5282523	5282523	G	T	NO	RABEP1	0	0	0,99851	1,8552	RAB GTPas	603616	NA
chr17	5362942	5362942	C	T	NO	RABEP1	nonsynony	RABEP1:N	0,99851	1,8552	RAB GTPas	603616	NA
chr17	7311819	7311819	T	G	NO	EIF5A	0	0	0,73709	2,407	Eukaryotic	600187	Faundes-B
chr17	7317937	7317937	G	A	NO	NEURL4	0	0	0,99999	2,9518	Neuralized	615865	NA
chr17	7462163	7462163	T	-	NO	ZBTB4	frameshift	ZBTB4:NM	0,99991	1,931	Zinc finger	612308	NA
chr17	7462348	7462348	G	-	NO	ZBTB4	stopgain	ZBTB4:NM	0,99991	1,931	Zinc finger	612308	NA
chr17	7466790	7466790	A	-	NO	ZBTB4	frameshift	ZBTB4:NM	0,99991	1,931	Zinc finger	612308	NA
chr17	7484771	7484771	G	T	NO	POLR2A	nonsynony	POLR2A:N	1	7,1307	Polymeras	180660	Neurodeve
chr17	7560108	7560108	C	T	NO	TNFSF12-T	stopgain	TNFSF13:N	7.6997e-0	9.8282e-0	|Tumor ne	|604472	NA
chr17	7560493	7560493	G	A	NO	TNFSF12-T	0	0	7.6997e-0	9.8282e-0	|Tumor ne	|604472	NA
chr17	7560747	7560747	G	T	NO	TNFSF12-T	nonsynony	TNFSF13:N	7.6997e-0	9.8282e-0	|Tumor ne	|604472	NA
chr17	7893934	7893934	C	T	NO	CHD3	nonsynony	CHD3:NM	1	6,1518	Chromodo	602120	Snijders Bl
chr17	8382778	8382778	A	T	NO	RPL26	0	0	0,9163	1,6397	Ribosomal	603704	?Diamond
chr17	8475947	8475947	G	A	NO	MYH10	nonsynony	MYH10:N	1	5,0058	Myosin, he	160776	NA
chr17	8478415	8478415	G	A	NO	MYH10	nonsynony	MYH10:N	1	5,0058	Myosin, he	160776	NA
chr17	12894953	12894953	G	A	NO	ARHGAP44	nonsynony	ARHGAP44	0,99988	2,3899	RHO GTPa	617716	NA
chr17	16080066	16080066	T	C	YES	NCOR1	0	0	1	3,9371	Nuclear re	600849	NA
chr17	17221379	17221379	T	G	NO	FLCN	stoploss	FLCN:NM_	0,78892	1,1301	Folliculin (	607273	Birt-Hogg-
chr17	17221490	17221490	C	T	NO	FLCN	stopgain	FLCN:NM_	0,78892	1,1301	Folliculin (	607273	Birt-Hogg-
chr17	17223929	17223929	G	A	NO	FLCN	nonsynony	FLCN:NM_	0,78892	1,1301	Folliculin (	607273	Birt-Hogg-
chr17	18184383	18184383	-	CGCCGCTG	NO	ALKBH5	frameshift	ALKBH5:N	0,94974	2,5626	AlkB homo	613303	NA
chr17	28720009	28720009	G	A	NO	RPL23A	nonsynony	RPL23A:N	0,90573	0,24236	Ribosomal	602326	NA
chr17	28720842	28720842	T	A	NO	RPL23A	nonsynony	RPL23A:N	0,90573	0,24236	Ribosomal	602326	NA
chr17	28749435	28749435	G	A	NO	TRAF4	nonsynony	TRAF4:NM	0,93602	2,4225	TNF recep	602464	NA
chr17	28917449	28917449	G	T	NO	PHF12	nonsynony	PHF12:NM	1	2,4291	PHD finger	618645	NA
chr17	29074800	29074801	TG	-	NO	MYO18A	frameshift	MYO18A:N	0,98723	2,454	Myosin XV	610067;60	NA
chr17	29093361	29093361	G	A	NO	MYO18A	nonsynony	MYO18A:N	0,98723	2,454	Myosin XV	610067;60	NA
chr17	29098951	29098951	G	A	NO	MYO18A	nonsynony	MYO18A:N	0,98723	2,454	Myosin XV	610067;60	NA
chr17	29118439	29118439	T	A	NO	MYO18A	nonsynony	MYO18A:N	0,98723	2,454	Myosin XV	610067;60	NA
chr17	29576679	29576680	AG	-	NO	GIT1	0	0	0,99978	3,0601	GIT ArfGap	608434	NA

chr17	29578742	29578742	T	G	NO	GIT1	nonsynony	GIT1:NM_	0,99978	3,0601	GIT ArfGap	608434	NA
chr17	30899216	30899216	G	A	NO	TEFM	stopgain	TEFM:NM	0,51424	0,58423	Transcripti	616422	Combined
chr17	31206254	31206254	G	T	NO	NF1	nonsynony	NF1:NM_0	0,90175	6,5427	Neurofibro	613113	Watson sy
chr17	31998955	31998958	AGAT	-	NO	SUZ12	frameshift	SUZ12:NM	0,99998	3,4007	SUZ12 pol	606245	Imagawa-
chr17	32175351	32175351	G	C	NO	RHOT1	nonsynony	RHOT1:NM	0,79851	2,4699	Ras homol	613888	NA
chr17	33023920	33023920	G	-	NO	ASIC2	frameshift	ASIC2:NM	0,99912	1,6707	Amiloride-	601784	NA
chr17	33023996	33023996	T	C	NO	ASIC2	nonsynony	ASIC2:NM	0,99912	1,6707	Amiloride-	601784	NA
chr17	35674234	35674234	G	A	NO	AP2B1	nonsynony	AP2B1:NM	0,9983	5,1667	Adaptor-re	601025	NA
chr17	37243532	37243532	T	A	NO	ACACA	stopgain	ACACA:NM	1	7,2382	Acetyl-Coe	200350	Acetyl-CoA
chr17	37520217	37520217	G	A	NO	SYNRG	0	0	0,93079	1,4376	Synergin, g	607291	NA
chr17	37520644	37520644	T	C	NO	SYNRG	nonsynony	SYNRG:NM	0,93079	1,4376	Synergin, g	607291	NA
chr17	37568788	37568788	T	C	NO	SYNRG	0	0	0,93079	1,4376	Synergin, g	607291	NA
chr17	38915148	38915148	T	G	YES	LASP1	0	0	0,9827	2,2064	LIM and S	602920	NA
chr17	39175243	39175243	G	A	NO	CACNB1	nonsynony	CACNB1:N	0,78689	2,1378	Calcium ch	114207	NA
chr17	39184868	39184868	G	C	NO	CACNB1	0	0	0,78689	2,1378	Calcium ch	114207	NA
chr17	39525860	39525860	A	G	NO	CDK12	0	0	1	2,5249	Cyclin-dep	615514	NA
chr17	40140781	40140781	-	GAGTGCG	YES	CASC3	0	0	0,61338	1,3171	MLN51 ge	606504	NA
chr17	41883122	41883122	C	G	NO	ACLY	nonsynony	ACLY:NM_	8,36E-02	3,2841	ATP citrate	108728	NA
chr17	41883214	41883214	A	C	NO	ACLY	nonsynony	ACLY:NM_	8,36E-02	3,2841	ATP citrate	108728	NA
chr17	42163606	42163606	C	T	NO	KCNH4	nonsynony	KCNH4:NM	0,99984	2,9416	Potassium	604528	NA
chr17	42219847	42219847	A	G	NO	STAT5B	0	0	0,99988	3,9952	Signal tran	604260	Growth ho
chr17	42224856	42224856	G	A	NO	STAT5B	nonsynony	STAT5B:N	0,99988	3,9952	Signal tran	604260	Growth ho
chr17	42333943	42333943	G	A	NO	STAT3	nonsynony	STAT3:NM	1	4,9943	Signal tran	102582	Hyper-IgE
chr17	42460911	42460911	G	A	NO	ATP6V0A1	nonsynony	ATP6V0A1	0,99797	3,7439	ATPase, H	192130	Neurodeve
chr17	42998791	42998791	T	A	NO	RPL27	nonsynony	RPL27:NM	0,85027	1,1412	Ribosomal	607526	?Diamond
chr17	44080806	44080806	T	C	NO	HDAC5	nonsynony	HDAC5:NM	1	3,3205	Histone de	605315	NA
chr17	44313099	44313099	C	T	NO	RUNDC3A	0	0	0,63623	2,6915	RUN doma	605448	NA
chr17	44558541	44558541	G	T	NO	FZD2	nonsynony	FZD2:NM_	0,65266	3,6675	Frizzled cla	600667	Omodyspl
chr17	45237631	45237631	T	C	NO	FMNL1	nonsynony	FMNL1:NM	0,99985	2,9981	Formin-lik	604656	NA
chr17	45245261	45245261	G	A	NO	FMNL1	nonsynony	FMNL1:NM	0,99985	2,9981	Formin-lik	604656	NA
chr17	45245399	45245399	G	A	NO	FMNL1	nonsynony	FMNL1:NM	0,99985	2,9981	Formin-lik	604656	NA
chr17	47122440	47122440	T	C	NO	CDC27	0	0	0,99675	3,1977	Cell divisio	116946	NA
chr17	47141924	47141924	G	-	NO	CDC27	frameshift	CDC27:NM	0,99675	3,1977	Cell divisio	116946	NA
chr17	47531346	47531346	T	-	NO	NPEPPS	frameshift	NPEPPS:N	1	3,9845	Aminopep	606793	NA
chr17	48550476	48550476	T	C	NO	HOXB3	nonsynony	HOXB3:NM	0,8917	0,11636	Homeobox	142966	NA
chr17	48555614	48555614	C	T	NO	HOXB3	0	0	0,8917	0,11636	Homeobox	142966	NA

chr17	49636811	49636811	T	C	NO	SPOP	0	0	0,99922	4,1412	Speckle-ty	602650	Nabais Sa-
chr17	50187011	50187011	A	G	NO	COL1A1	0	0	1	3,5319	Collagen I,	120150	Osteogene
chr17	50187968	50187968	G	A	NO	COL1A1	nonsynony	COL1A1:N	1	3,5319	Collagen I,	120150	Osteogene
chr17	50188630	50188630	C	T	NO	COL1A1	nonsynony	COL1A1:N	1	3,5319	Collagen I,	120150	Osteogene
chr17	50190328	50190328	G	A	NO	COL1A1	nonsynony	COL1A1:N	1	3,5319	Collagen I,	120150	Osteogene
chr17	50355824	50355824	G	A	NO	XYLT2	nonsynony	XYLT2:NM	0,92576	2,0916	Xylosyltran	608125	{Pseudoxa
chr17	50368247	50368247	G	A	NO	MRPL27	nonsynony	MRPL27:N	0,50212	-0,53905	Mitochond	611837	NA
chr17	50373163	50373163	G	T	NO	MRPL27	stopgain	MRPL27:N	0,50212	-0,53905	Mitochond	611837	NA
chr17	50619827	50619827	G	A	YES	CACNA1G	0	0	1	4,6386	Calcium ch	604065	Spinocere
chr17	51005259	51005259	G	A	NO	SPAG9	nonsynony	SPAG9:NM	1	3,1339	Sperm-ass	605430	NA
chr17	57116204	57116204	G	A	NO	AKAP1	nonsynony	AKAP1:NM	0,74238	1,18	A-kinase a	602449	NA
chr17	57258350	57258350	A	T	NO	MSI2	nonsynony	MSI2:NM_	0,99478	2,286	Musashi R	607897	NA
chr17	57979242	57979242	-	C	NO	VEZF1	frameshift	VEZF1:NM	0,99423	2,9212	Vascular e	606747	?Cardiomy
chr17	58492951	58492951	G	A	NO	MTMR4	nonsynony	MTMR4:N	0,99959	3,4935	Myotubula	603559	NA
chr17	59681478	59681478	-	GT	NO	CLTC	frameshift	CLTC:NM_	1	7,7637	Clathrin, h	118955	Intellectua
chr17	59681842	59681842	A	G	NO	CLTC	0	0	1	7,7637	Clathrin, h	118955	Intellectua
chr17	62481156	62481156	C	T	NO	TLK2	stopgain	TLK2:NM_	1	4,4909	Tousled-lik	608439	Intellectua
chr17	62677482	62677482	A	G	NO	MRC2	nonsynony	MRC2:NM	0,67472	2,7252	Mannose r	612264	NA
chr17	63437565	63437568	AAGA	-	NO	CYB561	0	0	0,61092	0,951	Cytochrom	600019	Orthostati
chr17	63550750	63550750	A	G	NO	DCAF7	nonsynony	DCAF7:NM	0,93284	2,7356	DDB1- and	605973	NA
chr17	63646070	63646070	A	G	NO	MAP3K3	nonsynony	MAP3K3:N	0,98535	2,042	Mitogen-a	602539	NA
chr17	64068191	64068191	G	C	NO	ERN1	stopgain	ERN1:NM_	0,99027	2,2663	Endoplasm	604033	NA
chr17	64504260	64504260	G	A	NO	DDX5	nonsynony	DDX5:NM	0,99998	2,7557	DEAD-box	180630	NA
chr17	67357583	67357583	A	G	NO	PSMD12	0	0	0,99984	1,8713	Proteasom	604450	Stankiewic
chr17	67875022	67875022	-	A	YES	BPTF	0	0	1	3,7096	Bromodom	601819	Neurodeve
chr17	67922986	67922986	G	A	NO	BPTF	nonsynony	BPTF:NM_	1	3,7096	Bromodom	601819	Neurodeve
chr17	72121820	72121820	-	AGGT	NO	SOX9	stopgain	SOX9:NM_	0,99772	1,6311	SRY (sex-d	608160	Campome
chr17	75231856	75231856	G	T	NO	NUP85	nonsynony	NUP85:NM	1	0,75518	Nucleopor	170285	Nephrotic
chr17	77089274	77089274	A	G	NO	SEC14L1	0	0	0,63264	2,4339	SEC14-like	601504	NA
chr17	77200634	77200634	C	T	NO	SEC14L1	stopgain	SEC14L1:N	0,63264	2,4339	SEC14-like	601504	NA
chr17	78358815	78358815	C	A	NO	SOCS3	nonsynony	SOCS3:NM	0,74613	1,8616	Suppresso	604176	NA
chr17	78709733	78709733	C	A	YES	CYTH1	0	0	0,99981	2,4385	Cytohesin	182115	NA
chr17	78782057	78782057	G	C	NO	CYTH1	0	0	0,99981	2,4385	Cytohesin	182115	NA
chr17	79784933	79784933	G	A	NO	CBX2	nonsynony	CBX2:NM_	0,99503	-0,26229	Chromobo	602770	?46XY sex
chr17	80144168	80144168	T	G	NO	EIF4A3	0	0	0,99985	4,021	Eukaryotic	608546	Robin sequ
chr17	82092697	82092697	C	A	NO	FASN	nonsynony	FASN:NM_	1	2,3643	Fatty acid	600212	NA

chr17	82255424	82255424	-	T	NO	CSNK1D	0	0	0,99577	2,7025	Casein kin	600864	Advanced
chr18	268006	268006	G	A	NO	THOC1	nonsynony	THOC1:NM	0,99955	2,5777	THO comp	606930	?Deafness
chr18	670719	670719	G	A	NO	TYMS	nonsynony	TYMS:NM	0,72247	2,5796	Thymidyla	188350	Dyskerato
chr18	2775839	2775839	C	T	NO	SMCHD1	nonsynony	SMCHD1:N	1	3,6318	Structural	614982	Bosma arh
chr18	3499307	3499307	G	A	NO	DLGAP1	nonsynony	DLGAP1:N	0,99875	3,2012	Discs large	605445	NA
chr18	8069779	8069779	G	A	NO	PTPRM	nonsynony	PTPRM:NM	0,99998	2,8441	Protein tyr	176888	NA
chr18	9550185	9550275	ACCTGGG	-	NO	PPP4R1	frameshift	PPP4R1:N	0,99958	2,0402	Protein ph	604908	NA
chr18	9562077	9562077	-	CAACAGCA	YES	PPP4R1	0	0	0,99958	2,0402	Protein ph	604908	NA
chr18	9588838	9588838	G	A	NO	PPP4R1	nonsynony	PPP4R1:N	0,99958	2,0402	Protein ph	604908	NA
chr18	10689669	10689669	G	A	NO	PIEZO2	nonsynony	PIEZO2:NM	8,10E-07	3,4445	PIEZO-type	613629	Arthrogryp
chr18	11851593	11851593	G	A	NO	CHMP1B	nonsynony	CHMP1B:N	0,73182	0,25667	Charged m	606486	NA
chr18	13387763	13387763	G	A	YES	LDLRAD4	0	0	0,62011	1,5738	Chromoso	606571	NA
chr18	21500586	21500586	G	A	NO	GREB1L	nonsynony	GREB1L:N	1	5,3659	GREB1-like	617782	Deafness,
chr18	21574022	21574023	TG	-	NO	ESCO1	frameshift	ESCO1:NM	0,99995	0,43144	Establishm	609674	NA
chr18	21575426	21575426	C	T	NO	ESCO1	0	0	0,99995	0,43144	Establishm	609674	NA
chr18	21815650	21815650	C	G	NO	MIB1	nonsynony	MIB1:NM_	3,36E-88	3,2115	Mindbomb	608677	Left ventri
chr18	33744172	33744172	G	-	NO	ASXL3	frameshift	ASXL3:NM	1	0,60715	ASXL trans	615115	Bainbridge
chr18	33746172	33746172	-	AAAGA	NO	ASXL3	frameshift	ASXL3:NM	1	0,60715	ASXL trans	615115	Bainbridge
chr18	34820914	34820914	G	A	NO	DTNA	nonsynony	DTNA:NM	0,63459	1,4204	Dystobrevi	601239	Left ventri
chr18	44876625	44876625	A	-	NO	SETBP1	frameshift	SETBP1:N	1	1,0965	SET-bindin	611060	Schinzel-G
chr18	46435284	46435284	T	G	NO	RNF165	0	0	0,97194	1,9384	NA	NA	NA
chr18	46818426	46818427	AT	-	NO	PIAS2	stopgain	PIAS2:NM	0,99718	2,0573	Protein inh	603567	NA
chr18	47231152	47231152	G	A	NO	SKOR2	stopgain	SKOR2:NM	0,75359	1,6321	SKI family	617138	NA
chr18	47244922	47244922	C	T	NO	SKOR2	stopgain	SKOR2:NM	0,75359	1,6321	SKI family	617138	NA
chr18	48950022	48950022	T	C	NO	SMAD7	nonsynony	SMAD7:N	0,54854	1,6522	SMAD fam	602932	{Colorecta
chr18	49491746	49491746	C	G	NO	RPL17	0	0	0,92412	1,3355	Ribosomal	603661	NA
chr18	50269822	50269822	C	G	NO	MBD1	0	0	0,99717	1,9926	Methyl=Cp	156535	NA
chr18	50273476	50273476	G	T	NO	MBD1	0	0	0,99717	1,9926	Methyl=Cp	156535	NA
chr18	50273866	50273866	A	T	NO	MBD1	0	0	0,99717	1,9926	Methyl=Cp	156535	NA
chr18	50986952	50986952	-	AGTT	NO	ELAC1	frameshift	ELAC1:NM	0,88118	0,43561	elaC ribon	608079	NA
chr18	57360004	57360007	AAAC	-	NO	ST8SIA3	frameshift	ST8SIA3:N	0,85912	1,4714	ST8 alpha-	609478	NA
chr18	58341111	58341111	G	A	NO	NEDD4L	nonsynony	NEDD4L:N	1	3,7252	Ubiquitin	606384	Periventric
chr18	58742014	58742018	GGTAC	-	NO	MALT1	frameshift	MALT1:NM	0,98651	2,8387	MALT1 par	604860	Immunode
chr18	62914940	62914940	G	T	NO	PHLPP1	nonsynony	PHLPP1:N	0,99927	1,7489	PH domain	609396	NA
chr18	63319169	63319169	C	T	NO	BCL2	0	0	0,55903	1,3975	B-cell CLL/	151430	Leukemia/
chr18	74140199	74140199	-	A	NO	FBXO15	0	0	0,71072	0,47878	F-box only	609093	NA

chr18	74634197	74634197	G	A	NO	ZNF407	nonsynony	ZNF407:N	1	0,85896	Zinc finger	615894	SIMHA syn
chr18	74877333	74877333	G	A	NO	ZNF407	nonsynony	ZNF407:N	1	0,85896	Zinc finger	615894	SIMHA syn
chr18	80137049	80137052	AGTC	-	NO	ADNP2	frameshift	ADNP2:NM	0,69573	0,34013	Activity-de	617422	NA
chr19	501742	501742	-	AC	NO	MADCAM1	frameshift	MADCAM1	0,53462	0,47605	Mucosal a	102670	NA
chr19	740467	740467	C	A	NO	PALM	stopgain	PALM:NM	0,51902	0,71581	Paralemm	608134	NA
chr19	804825	804825	G	A	NO	PTBP1	0	0	0,99961	1,6525	Polypyrimi	600693	NA
chr19	984387	984387	G	T	NO	WDR18	nonsynony	WDR18:N	0,99271	0,47932	WD repeat	620291	NA
chr19	1456303	1456303	C	A	NO	APC2	0	0	0,99994	0,75411	APC regula	612034	Cortical dy
chr19	1555345	1555345	-	A	NO	MEX3D	frameshift	MEX3D:N	0,92303	2,4708	Mex-3, C.	611009	NA
chr19	1816695	1816695	C	T	NO	REXO1	0	0	0,9864	-0,19765	RNA exonu	609614	NA
chr19	1979314	1979314	C	A	NO	CSNK1G2	0	0	0,99138	2,7969	Casein kin	602214	NA
chr19	1979623	1979623	G	A	NO	CSNK1G2	nonsynony	CSNK1G2:	0,99138	2,7969	Casein kin	602214	NA
chr19	1987497	1987497	C	T	NO	BTBD2	0	0	0,87534	1,787	BTB/POZ d	608531	NA
chr19	2226965	2226965	C	T	NO	DOT1L	stopgain	DOT1L:NM	1	2,7249	Dot1, yeas	607375	NA
chr19	3575537	3575537	C	T	NO	HMG20B	0	0	0,69112	2,2329	High mobi	605535	NA
chr19	3653285	3653285	C	A	NO	PIP5K1C	0	0	0,97704	2,2925	Phosphati	606102	Lethal con
chr19	4101143	4101143	T	C	NO	MAP2K2	nonsynony	MAP2K2:N	0,96673	1,8655	Mitogen-a	601263	Cardiofaci
chr19	4423376	4423376	G	A	NO	CHAF1A	nonsynony	CHAF1A:N	1	1,8498	Chromatin	601246	NA
chr19	4682692	4682692	T	C	NO	DPP9	0	0	0,98708	2,6594	Dipeptidyl	608258	Hatipoglu
chr19	4694621	4694621	T	C	NO	DPP9	stoploss	DPP9:NM_	0,98708	2,6594	Dipeptidyl	608258	Hatipoglu
chr19	5144400	5144400	-	GAGAGCA	NO	KDM4B	stopgain	KDM4B:N	1	3,4843	Lysine dem	609765	Intellectua
chr19	5150394	5150394	C	T	NO	KDM4B	nonsynony	KDM4B:N	1	3,4843	Lysine dem	609765	Intellectua
chr19	5587254	5587254	G	A	NO	SAFB2	nonsynony	SAFB2:NM	0,99979	0,48273	Scaffold at	608066	NA
chr19	5592891	5592891	T	C	NO	SAFB2	0	0	0,99979	0,48273	Scaffold at	608066	NA
chr19	6040240	6040240	G	A	NO	RFX2	stopgain	RFX2:NM_	0,9997	2,6556	Regulatory	142765	NA
chr19	6417042	6417042	G	A	NO	KHSRP	nonsynony	KHSRP:NM	1	3,83	KH-type sp	603445	NA
chr19	6712253	6712253	G	A	NO	C3	0	0	0,90421	2,7489	Compleme	120700	C3 deficien
chr19	7184524	7184524	G	A	NO	INSR	nonsynony	INSR:NM_	3,61E-05	3,8314	Insulin rec	147670	Rabson-M
chr19	7555644	7555644	G	A	NO	PNPLA6	nonsynony	PNPLA6:N	1,94E-13	4,3547	Patatin-lik	603197	Spastic par
chr19	7853187	7853187	G	A	NO	EVI5L	0	0	0,99985	4,2346	NA	NA	NA
chr19	8592874	8592874	C	A	NO	ADAMTS1	0	0	0,84288	3,628	ADAM me	608990	Weill-Mar
chr19	9838583	9838583	G	A	NO	PIN1	nonsynony	PIN1:NM_	0,74031	1,3483	Peptidyl-p	601052	NA
chr19	10142114	10142114	G	A	NO	DNMT1	nonsynony	DNMT1:N	1	4,9903	DNA meth	126375	Neuropath
chr19	10317554	10317554	G	T	NO	RAVER1	stopgain	RAVER1:N	0,91101	1,4571	Ribonucle	609950	NA
chr19	10320918	10320918	G	A	NO	RAVER1	nonsynony	RAVER1:N	0,91101	1,4571	Ribonucle	609950	NA
chr19	10463904	10463904	C	T	NO	PDE4A	stopgain	PDE4A:NM	0,98167	2,4163	Phosphodi	600126	NA

chr19	10798482	10798482	C	T	NO	DNM2	0	0	0,99998	3,4829	Dynamin-2	602378	Centronuc
chr19	11012946	11012946	C	A	NO	SMARCA4	0	0	1	6,8459	SWI/SNF-r	603254	Coffin-Siris
chr19	11041374	11041374	G	A	NO	SMARCA4	nonsynony	SMARCA4:	1	6,8459	SWI/SNF-r	603254	Coffin-Siris
chr19	12318716	12318716	G	A	NO	ZNF563	stopgain	ZNF563:N	0,7644	0,29217	NA	NA	NA
chr19	12319098	12319098	A	T	NO	ZNF563	stopgain	ZNF563:N	0,7644	0,29217	NA	NA	NA
chr19	12319763	12319763	G	A	NO	ZNF563	stopgain	ZNF563:N	0,7644	0,29217	NA	NA	NA
chr19	13959894	13959894	-	GGTGGAC	NO	DCAF15	frameshift	DCAF15:N	0,9942	2,2685	DDB1- and	620109	NA
chr19	14100893	14100893	A	G	NO	PRKACA	nonsynony	PRKACA:N	0,97142	2,9714	Protein kin	601639	Cushing sy
chr19	16392442	16392442	T	C	YES	EPS15L1	0	0	0,99964	1,9019	EPS15-like	616826	NA
chr19	16402486	16402486	C	G	YES	EPS15L1	0	0	0,99964	1,9019	EPS15-like	616826	NA
chr19	16578333	16578333	A	G	YES	MED26	0	0	0,88242	1,1281	Mediator c	605043	NA
chr19	16862894	16862894	G	A	NO	SIN3B	stopgain	SIN3B:NM	0,99999	3,8662	Sin3, yeast	607777	NA
chr19	17200834	17200834	G	A	NO	MYO9B	0	0	1	3,9717	Myosin IXB	602129	{Celiac dis
chr19	17202174	17202174	C	A	NO	MYO9B	stopgain	MYO9B:N	1	3,9717	Myosin IXB	602129	{Celiac dis
chr19	17271613	17271613	T	C	NO	BABAM1	nonsynony	BABAM1:N	0,96269	1,1667	Chromoso	612766	NA
chr19	17314345	17314345	G	A	NO	DDA1	nonsynony	DDA1:NM	0,93718	1,6583	NA	NA	NA
chr19	17327263	17327263	A	T	NO	ANO8	nonsynony	ANO8:NM	0,95634	2,5036	Transmem	610216	NA
chr19	17333765	17333765	G	A	NO	ANO8	nonsynony	ANO8:NM	0,95634	2,5036	Transmem	610216	NA
chr19	17762849	17762849	G	A	NO	FCHO1	nonsynony	FCHO1:NM	0,99845	1,4044	FCH doma	613437	Immunode
chr19	17784140	17784140	T	C	NO	FCHO1	nonsynony	FCHO1:NM	0,99845	1,4044	FCH doma	613437	Immunode
chr19	17784724	17784724	G	A	YES	FCHO1	0	0	0,99845	1,4044	FCH doma	613437	Immunode
chr19	18124151	18124151	G	A	NO	MAST3	0	0	0,99991	4,1363	Microtubu	612258	Developm
chr19	18929445	18929445	A	G	NO	HOMER3	stoploss	HOMER3:N	0,95363	1,1898	Homer sca	604800	NA
chr19	18932958	18932958	C	-	NO	HOMER3	frameshift	HOMER3:N	0,95363	1,1898	Homer sca	604800	NA
chr19	18939049	18939049	C	A	NO	HOMER3	0	0	0,95363	1,1898	Homer sca	604800	NA
chr19	18994380	18994380	G	A	NO	SUGP2	nonsynony	SUGP2:NM	0,99995	0,66247	SURP and	607993	NA
chr19	19276942	19276942	C	A	NO	SUGP1	0	0	0,99568	1,2098	SURP and	607992	NA
chr19	19302284	19302284	G	A	NO	SUGP1	nonsynony	SUGP1:NM	0,99568	1,2098	SURP and	607992	NA
chr19	19649667	19649667	T	C	NO	ATP13A1	0	0	0,98939	2,6636	ATPase 13	619118	NA
chr19	19655436	19655436	G	A	NO	ATP13A1	nonsynony	ATP13A1:N	0,98939	2,6636	ATPase 13	619118	NA
chr19	19655873	19655873	C	T	NO	ATP13A1	0	0	0,98939	2,6636	ATPase 13	619118	NA
chr19	30445133	30445133	G	A	NO	ZNF536	stopgain	ZNF536:N	0,99958	2,4037	Zinc finger	618037	NA
chr19	33301405	33301405	G	A	NO	CEBPA	nonsynony	CEBPA:NM	0,55365	0,56836	CCAAT/en	116897	Leukemia,
chr19	34454255	34454255	G	A	NO	UBA2	0	0	0,99851	2,6072	Ubiquitin-l	613295	ACCES syn
chr19	36748715	36748725	ATGTTGAA	-	NO	ZNF850	frameshift	ZNF850:N	0,72103	3,0016	NA	NA	NA
chr19	38714536	38714536	A	G	NO	ACTN4	nonsynony	ACTN4:NM	1	4,1552	Actinin, al	604638	Glomerulo

chr19	38725907	38725907	C	T	NO	ACTN4	0	0	1	4,1552	Actinin, al	604638	Glomerulo
chr19	38844003	38844003	T	C	NO	HNRNPL	0	0	0,99685	3,5416	Heterogen	603083	NA
chr19	38845848	38845848	C	T	NO	HNRNPL	0	0	0,99685	3,5416	Heterogen	603083	NA
chr19	39469258	39469258	G	A	NO	SUPT5H	0	0	1	4,561	SPT5 hom	602102	NA
chr19	39471624	39471624	G	A	NO	SUPT5H	nonsynony	SUPT5H:N	1	4,561	SPT5 hom	602102	NA
chr19	39836551	39836551	C	T	NO	FBL	0	0	0,99319	1,1955	Fibrillarin (	134795	NA
chr19	40238094	40238094	G	C	NO	AKT2	0	0	0,64432	2,4133	AKT serine	164731	Diabetes m
chr19	40572375	40572375	G	T	NO	SPTBN4	nonsynony	SPTBN4:N	1	3,9344	Spectrin, b	606214	Neurodeve
chr19	41272078	41272078	A	G	NO	HNRNPUL	0	0	0,99999	3,226	Heterogen	605800	NA
chr19	41902780	41902780	G	A	NO	ARHGEF1	0	0	0,76754	2,8274	Rho guani	601855	?Immunod
chr19	42042632	42042632	G	A	NO	GRIK5	nonsynony	GRIK5:NM	0,99425	3,3634	Glutamate	600283	NA
chr19	42062602	42062602	G	A	NO	GRIK5	nonsynony	GRIK5:NM	0,99425	3,3634	Glutamate	600283	NA
chr19	42117371	42117371	G	T	NO	POU2F2	0	0	0,99884	2,602	POU doma	164176	NA
chr19	42122576	42122576	T	C	NO	POU2F2	nonsynony	POU2F2:N	0,99884	2,602	POU doma	164176	NA
chr19	42292295	42292295	G	A	NO	CIC	0	0	0,99999	0,72576	Capicua tr	612082	Intellectua
chr19	42350148	42350148	G	A	NO	MEGF8	nonsynony	MEGF8:N	1,93E-05	3,4785	Multiple e	604267	Carpenter
chr19	42351229	42351229	A	G	NO	MEGF8	nonsynony	MEGF8:N	1,93E-05	3,4785	Multiple e	604267	Carpenter
chr19	45090292	45090292	-	CCGC	NO	GEMIN7	frameshift	GEMIN7:N	0,64543	0,34722	GEM-asso	607419	NA
chr19	45529091	45529100	GCGGCGG	-	NO	OPA3	frameshift	OPA3:NM	0,5657	5,20E-02	Outer mito	606580	3-methylgl
chr19	45529121	45529121	G	A	NO	OPA3	stopgain	OPA3:NM	0,5657	5,20E-02	Outer mito	606580	3-methylgl
chr19	45817976	45817976	G	A	NO	SYMPK	nonsynony	SYMPK:NM	1	2,8559	Symplekin	602388	NA
chr19	45847847	45847848	AG	-	NO	SYMPK	frameshift	SYMPK:NM	1	2,8559	Symplekin	602388	NA
chr19	45901284	45901284	G	A	NO	MYPOP	stopgain	MYPOP:N	0,66892	0,97505	MYB-relat	617861	NA
chr19	46390136	46390136	A	G	NO	PPP5C	0	0	0,99938	2,7341	Protein ph	600658	NA
chr19	47021742	47021742	G	A	NO	NPAS1	nonsynony	NPAS1:NM	0,97067	1,7971	Neuronal P	603346	NA
chr19	48491233	48491233	T	A	NO	LMTK3	nonsynony	LMTK3:NM	0,99997	4,3151	Lemur tyro	619624	NA
chr19	48499169	48499169	T	-	NO	LMTK3	frameshift	LMTK3:NM	0,99997	4,3151	Lemur tyro	619624	NA
chr19	49007321	49007321	G	C	NO	RUVBL2	nonsynony	RUVBL2:N	0,99992	3,1128	RUVB, E. c	604788	NA
chr19	49116410	49116410	G	C	NO	LIN7B	nonsynony	LIN7B:NM	0,85352	1,077	Lin7 homo	612331	NA
chr19	49134878	49134878	G	C	NO	PPFIA3	nonsynony	PPFIA3:NM	1	5,487	PTPRF-inte	603144	NA
chr19	49149320	49149320	G	A	NO	PPFIA3	nonsynony	PPFIA3:NM	1	5,487	PTPRF-inte	603144	NA
chr19	49474282	49474282	G	T	NO	FLT3LG	0	0	0,9404	0,89737	FMS-relate	600007	NA
chr19	49851073	49851073	-	C	NO	PTOV1	frameshift	PTOV1:NM	0,75737	-5,83E-02	PTOV1 ext	610195	NA
chr19	49857899	49857899	A	C	NO	PTOV1	0	0	0,75737	-5,83E-02	PTOV1 ext	610195	NA
chr19	50323898	50323898	T	C	NO	KCNC3	nonsynony	KCNC3:NM	0,20575	3,0401	Potassium	176264	Spinocere
chr19	50428208	50428208	C	T	NO	SPIB	nonsynony	SPIB:NM_	0,91231	1,2617	SPIB trans	606802	NA

chr19	53490998	53490998	C	T	NO	ZNF813	stopgain	ZNF813:N	0,63585	-0,64673	NA	NA	NA
chr19	53491213	53491213	C	G	NO	ZNF813	stopgain	ZNF813:N	0,63585	-0,64673	NA	NA	NA
chr19	53491348	53491348	C	A	NO	ZNF813	stopgain	ZNF813:N	0,63585	-0,64673	NA	NA	NA
chr19	53491580	53491580	C	T	NO	ZNF813	stopgain	ZNF813:N	0,63585	-0,64673	NA	NA	NA
chr19	54145644	54145644	G	A	NO	CNOT3	nonsynony	CNOT3:NM	1	3,7642	CCR4-NOT	604910	Intellectua
chr19	54200894	54200894	G	T	NO	RPS9	0	0	0,87956	2,7327	Ribosomal	603631	NA
chr19	54435666	54435666	G	C	NO	TTYH1	nonsynony	TTYH1:NM	0,99539	0,38422	Tweety fa	605784	NA
chr19	54456469	54456469	A	T	NO	LENG8	0	0	0,99938	1,3474	Leukocyte	616575	NA
chr19	55236908	55236908	C	T	NO	PPP6R1	0	0	0,98287	3,54E-02	SAPS dom	610875	NA
chr19	55239683	55239683	C	T	NO	PPP6R1	nonsynony	PPP6R1:N	0,98287	3,54E-02	SAPS dom	610875	NA
chr19	55302744	55302744	G	A	NO	BRSK1	nonsynony	BRSK1:NM	0,99982	3,9402	BR serine/	609235	NA
chr19	55401669	55401678	CCGCATAC	-	NO	UBE2S	frameshift	UBE2S:NM	0,63172	0,95911	Ubiquitin-	610309	NA
chr19	55688981	55688981	A	G	NO	EPN1	nonsynony	EPN1:NM_	0,78579	1,3433	Epsin 1 (EP	607262	NA
chr19	55694864	55694864	G	A	NO	EPN1	nonsynony	EPN1:NM_	0,78579	1,3433	Epsin 1 (EP	607262	NA
chr19	58393138	58393138	C	T	NO	RPS5	nonsynony	RPS5:NM_	0,94764	2,7926	Ribosomal	603630	NA
chr19	58393473	58393473	C	T	NO	RPS5	nonsynony	RPS5:NM_	0,94764	2,7926	Ribosomal	603630	NA
chr19	58550142	58550142	T	G	NO	TRIM28	0	0	1	2,3295	Tripartite	601742	NA
chr20	2656035	2656035	G	T	YES	NOP56	0	0	0,62098	0,60455	Nop56, S.	614154	Spinocere
chr20	3562422	3562422	G	T	NO	ATRN	nonsynony	ATRN:NM_	1	2,6863	Attractin (	603130	NA
chr20	3584086	3584086	A	G	NO	ATRN	0	0	1	2,6863	Attractin (	603130	NA
chr20	3784939	3784942	CTCA	-	NO	CENPB	frameshift	CENPB:NM	0,99634	2,7337	Centromer	117140	NA
chr20	6770442	6770442	G	A	NO	BMP2	nonsynony	BMP2:NM	0,98799	1,1385	Bone morp	112261	Short statu
chr20	8132703	8132703	G	A	NO	PLCB1	nonsynony	PLCB1:NM	0,98337	3,8345	Phospholi	607120	Developm
chr20	8716310	8716310	G	A	NO	PLCB1	nonsynony	PLCB1:NM	0,98337	3,8345	Phospholi	607120	Developm
chr20	10647005	10647005	T	C	NO	JAG1	nonsynony	JAG1:NM_	1	3,2459	Jagged 1 (J	601920	?Deafness
chr20	10649109	10649109	-	A	YES	JAG1	0	0	1	3,2459	Jagged 1 (J	601920	?Deafness
chr20	10673006	10673006	C	G	NO	JAG1	nonsynony	JAG1:NM_	1	3,2459	Jagged 1 (J	601920	?Deafness
chr20	19584971	19584971	C	T	NO	SLC24A3	nonsynony	SLC24A3:N	0,87946	2,0889	Solute carr	609839	NA
chr20	31721734	31721734	A	G	NO	BCL2L1	nonsynony	BCL2L1:N	0,93667	1,62	BCL2-like 1	600039	NA
chr20	31742540	31742540	G	C	NO	TPX2	0	0	0,99981	0,49021	Proliferati	605917	NA
chr20	31793966	31793966	G	C	NO	TPX2	nonsynony	TPX2:NM_	0,99981	0,49021	Proliferati	605917	NA
chr20	32215387	32215387	G	A	NO	POFUT1	nonsynony	POFUT1:N	0,96303	0,95225	Protein O-	607491	Dowling-D
chr20	33623158	33623158	G	A	NO	CBFA2T2	nonsynony	CBFA2T2:N	0,99585	2,475	Core-bindi	603672	NA
chr20	33629798	33629798	G	A	NO	CBFA2T2	nonsynony	CBFA2T2:N	0,99585	2,475	Core-bindi	603672	NA
chr20	34462202	34462202	C	T	NO	ITCH	nonsynony	ITCH:NM_	0,99997	3,5832	Itchy, mou	606409	Autoimmu
chr20	34861534	34861534	G	A	NO	GGT7	stopgain	GGT7:NM_	0,98393	2,304	Gamma-gl	612342	NA

chr20	35734179	35734182	CACC	-	NO	RBM39	0	0	0,99981	4,2603	RNA bindi	604739	NA
chr20	37403216	37403216	G	A	NO	SRC	nonsynony	SRC:NM_0	0,99691	3,8709	Protoonco	190090	?Thrombo
chr20	38558361	38558361	G	T	NO	RALGAPB	nonsynony	RALGAPB:	1	3,1771	Ral GTPase	618833	NA
chr20	41112816	41112816	G	A	NO	TOP1	nonsynony	TOP1:NM_	1	4,4734	Topoisom	126420	DNA topoi
chr20	41416791	41416791	G	A	NO	CHD6	nonsynony	CHD6:NM	1	4,0001	Chromodo	616114	NA
chr20	42104640	42104640	G	A	NO	PTPRT	nonsynony	PTPRT:NM	1	3,1351	Protein-tyr	608712	NA
chr20	47650992	47650992	T	C	NO	NCOA3	nonsynony	NCOA3:NM	0,54065	1,3278	Nuclear re	601937	NA
chr20	47651122	47651122	-	GCAG	NO	NCOA3	frameshift	NCOA3:NM	0,54065	1,3278	Nuclear re	601937	NA
chr20	47651127	47651127	-	GC	NO	NCOA3	frameshift	NCOA3:NM	0,54065	1,3278	Nuclear re	601937	NA
chr20	47651128	47651128	-	GCAACAG	NO	NCOA3	frameshift	NCOA3:NM	0,54065	1,3278	Nuclear re	601937	NA
chr20	48666354	48666354	C	T	NO	PREX1	nonsynony	PREX1:NM	1	3,695	Phosphati	606905	NA
chr20	48953559	48953559	C	T	NO	ARFGEF2	stopgain	ARFGEF2:N	0,99993	2,5971	ADP-ribos	605371	Periventric
chr20	48991052	48991052	G	A	NO	ARFGEF2	nonsynony	ARFGEF2:N	0,99993	2,5971	ADP-ribos	605371	Periventric
chr20	48991110	48991110	A	G	NO	ARFGEF2	nonsynony	ARFGEF2:N	0,99993	2,5971	ADP-ribos	605371	Periventric
chr20	49013648	49013648	G	A	NO	ARFGEF2	nonsynony	ARFGEF2:N	0,99993	2,5971	ADP-ribos	605371	Periventric
chr20	49032102	49032102	G	A	NO	ARFGEF2	nonsynony	ARFGEF2:N	0,99993	2,5971	ADP-ribos	605371	Periventric
chr20	49908442	49908442	G	A	NO	SPATA2	nonsynony	SPATA2:N	0,97889	0,67696	Spermatog	607662	NA
chr20	51608628	51608628	T	G	YES	ATP9A	0	0	0,99999	4,1543	ATPase, cl	609126	Neurodeve
chr20	51676127	51676127	C	T	NO	ATP9A	0	0	0,99999	4,1543	ATPase, cl	609126	Neurodeve
chr20	58898934	58898937	TTAA	-	NO	GNAS	0	0	0,68076	2,6546	GNAS com	139320	ACTH-inde
chr20	59605061	59605061	G	T	NO	PHACTR3	nonsynony	PHACTR3:	0,99687	0,97164	Phosphata	608725	NA
chr20	59881432	59881432	C	-	NO	SYCP2	0	0	1	0,25831	Synaptone	604105	Spermatog
chr20	59900741	59900741	T	C	NO	SYCP2	0	0	1	0,25831	Synaptone	604105	Spermatog
chr20	62124637	62124637	G	A	NO	LSM14B	nonsynony	LSM14B:N	0,9953	1,6895	LSM family	620689	NA
chr20	62896271	62896271	G	A	NO	DIDO1	nonsynony	DIDO1:NM	1	1,0989	Death-ass	604140	NA
chr20	64074031	64074031	G	C	NO	RGS19	nonsynony	RGS19:NM	0,96134	1,0612	Regulator	605071	NA
chr20	64205098	64205098	G	T	YES	MYT1	0	0	0,97791	2,5631	Myelin tra	600379	NA
chr21	14965269	14965269	-	CTTCTTTA	NO	NRIP1	stopgain	NRIP1:NM	0,99394	-0,45989	Nuclear re	602490	?Congenit
chr21	15847757	15847757	-	G	NO	USP25	frameshift	USP25:NM	0,7522	1,2369	Ubiquitin-	604736	NA
chr21	29327376	29327376	A	T	NO	BACH1	stopgain	BACH1:NM	0,57072	0,56056	BTB and C	602751	NA
chr21	31120802	31120802	G	A	NO	TIAM1	nonsynony	TIAM1:NM	0,95662	1,6982	T-cell lymp	600687	Neurodeve
chr21	31141153	31141153	G	A	NO	TIAM1	stopgain	TIAM1:NM	0,95662	1,6982	T-cell lymp	600687	Neurodeve
chr21	31217570	31217570	G	A	NO	TIAM1	nonsynony	TIAM1:NM	0,95662	1,6982	T-cell lymp	600687	Neurodeve
chr21	31217630	31217630	G	A	NO	TIAM1	stopgain	TIAM1:NM	0,95662	1,6982	T-cell lymp	600687	Neurodeve
chr21	31671401	31671401	A	T	NO	SCAF4	stoploss	SCAF4:NM	1	1,9418	SR-related	616023	Fliedner-Z
chr21	32762156	32762156	G	A	NO	PAXBP1	nonsynony	PAXBP1:N	0,99999	2,6647	PAX3- and	617621	NA

chr21	33555141	33555142	CA	-	NO	SON	frameshift	SON:NM_	1	1,541	SON DNA-	182465	ZTTK syndr
chr21	33772148	33772148	G	A	NO	ITSN1	nonsynony	ITSN1:NM	1	3,6138	Intersectin	602442	NA
chr21	33883649	33883649	C	T	NO	ITSN1	stopgain	ITSN1:NM	1	3,6138	Intersectin	602442	NA
chr21	36337001	36337001	-	T	NO	MORC3	frameshift	MORC3:N	0,99985	2,8899	MORC fam	610078	NA
chr21	36360075	36360075	C	G	NO	MORC3	nonsynony	MORC3:N	0,99985	2,8899	MORC fam	610078	NA
chr21	37478281	37478281	-	T	NO	DYRK1A	frameshift	DYRK1A:N	0,99967	3,3441	Dual speci	600855	Intellectua
chr21	38400646	38400646	C	T	YES	ERG	0	0	0,96413	2,528	ETS transc	165080	Lymphatic
chr21	38403680	38403680	G	A	NO	ERG	nonsynony	ERG:NM_0	0,96413	2,528	ETS transc	165080	Lymphatic
chr21	38805537	38805537	G	A	NO	ETS2	stopgain	ETS2:NM_	0,99888	2,2796	ETS protoo	164740	NA
chr21	39218272	39218272	T	C	NO	BRWD1	nonsynony	BRWD1:N	1	2,8458	Bromodom	617824	Ciliary dys
chr21	40312240	40312240	G	A	NO	DSCAM	nonsynony	DSCAM:N	1	3,2228	DS cell adh	602523	NA
chr21	43007494	43007494	C	T	NO	PKNOX1	stopgain	PKNOX1:N	0,99401	1,475	PBX/knott	602100	NA
chr21	45917627	45917627	G	C	NO	PCBP3	nonsynony	PCBP3:NM	0,84326	2,0153	Poly(rC)-bi	608502	NA
chr22	17791292	17791292	T	C	NO	MICAL3	nonsynony	MICAL3:N	0,99499	1,7451	Microtubu	608882	NA
chr22	17810776	17810776	A	G	NO	MICAL3	nonsynony	MICAL3:N	0,99499	1,7451	Microtubu	608882	NA
chr22	17871837	17871837	C	T	NO	MICAL3	nonsynony	MICAL3:N	0,99499	1,7451	Microtubu	608882	NA
chr22	18088051	18088051	-	T	NO	PEX26	frameshift	PEX26:NM	0,93189	-0,36755	Peroxisom	608666	Peroxisom
chr22	20087177	20087177	G	A	NO	DGCR8	nonsynony	DGCR8:NM	0,99973	2,9898	DGCR8 mi	609030	NA
chr22	20116272	20116273	CT	-	NO	RANBP1;T	frameshift	RANBP1:N	8.2235e-0	1.9929e+0	RAN-bindi	601180|61	NA
chr22	20429293	20429293	C	T	NO	SCARF2	nonsynony	SCARF2:N	0,9998	3,0165	Scavenger	613619	Van den E
chr22	20727360	20727360	G	A	NO	PI4KA	nonsynony	PI4KA:NM	3,12E-12	3,5271	Phosphati	600286	Spastic par
chr22	20727845	20727845	T	A	NO	PI4KA	nonsynony	PI4KA:NM	3,12E-12	3,5271	Phosphati	600286	Spastic par
chr22	20734119	20734119	G	A	NO	PI4KA	nonsynony	PI4KA:NM	3,12E-12	3,5271	Phosphati	600286	Spastic par
chr22	20803253	20803253	A	G	NO	PI4KA	nonsynony	PI4KA:NM	3,12E-12	3,5271	Phosphati	600286	Spastic par
chr22	23289622	23289622	G	T	YES	BCR	0	0	0,98761	0,88811	BCR activa	151410	Leukemia,
chr22	23292558	23292558	G	A	NO	BCR	nonsynony	BCR:NM_0	0,98761	0,88811	BCR activa	151410	Leukemia,
chr22	27798944	27798944	-	C	NO	MN1	frameshift	MN1:NM_	0,99999	2,1584	MN1 proto	156100	CEBALID s
chr22	28108197	28108197	G	A	NO	TTC28	nonsynony	TTC28:NM	1	4,1651	Tetratricop	615098	NA
chr22	29351734	29351734	G	A	NO	AP1B1	nonsynony	AP1B1:NM	0,99828	3,1177	Adaptor-re	600157	Keratitis-ic
chr22	29978530	29978530	A	G	NO	MTMR3	nonsynony	MTMR3:N	0,99937	1,995	Myotubula	603558	NA
chr22	30244047	30244047	C	A	NO	LIF	stopgain	LIF:NM_00	0,5242	0,39763	Leukemia i	159540	NA
chr22	30556095	30556095	T	A	YES	GAL3ST1	nonsynony	GAL3ST1:N	0,66559	1,7304	Galactose-	602300	NA
chr22	31439888	31439888	G	A	NO	EIF4ENIF1	stopgain	EIF4ENIF1:	0,99999	1,9865	Eukaryotic	607445	NA
chr22	32859328	32859328	G	A	NO	TIMP3	nonsynony	TIMP3:NM	0,63037	1,8285	Tissue inhi	188826	Sorsby fun
chr22	36292132	36292132	G	A	NO	MYH9	nonsynony	MYH9:NM	1	3,473	Myosin, he	160775	Macrothro
chr22	37632616	37632616	C	T	NO	GGA1	nonsynony	GGA1:NM	0,99992	1,2245	Golgi asso	606004	NA

chr22	39231617	39231617	C	T	NO	PDGFB	0	0	0,77006	1,0803	Platelet-de	190040	Meningiom
chr22	39313729	39313729	C	T	NO	RPL3	nonsynony	RPL3:NM_	0,99441	1,5583	Ribosomal	604163	NA
chr22	39318520	39318520	G	A	NO	RPL3	nonsynony	RPL3:NM_	0,99441	1,5583	Ribosomal	604163	NA
chr22	39663762	39663762	A	G	NO	CACNA1I	nonsynony	CACNA1I:N	1	5,0511	Calcium ch	608230	Neurodeve
chr22	39664763	39664763	G	A	NO	CACNA1I	nonsynony	CACNA1I:N	1	5,0511	Calcium ch	608230	Neurodeve
chr22	41164075	41164075	G	A	NO	EP300	nonsynony	EP300:NM	1	2,0341	E1A-bindin	602700	Menke-He
chr22	41646986	41646986	-	T	NO	XRCC6	frameshift	XRCC6:NM	0,99904	3,0983	X-ray repa	152690	NA
chr22	43207531	43207531	C	T	NO	SCUBE1	0	0	0,96076	1,9781	Signal pep	611746	NA
chr22	43833695	43833695	C	G	NO	SULT4A1	nonsynony	SULT4A1:N	0,96563	2,6586	Sulfotrans	608359	NA
chr22	46369758	46369758	G	T	NO	CELSR1	nonsynony	CELSR1:N	0,99994	1,2028	Cadherin E	604523	Lymphatic
chr22	46676672	46676672	G	A	NO	GRAMD4	0	0	0,99998	2,5559	Gram dom	613691	NA
chr22	49798689	49798689	G	T	NO	BRD1	0	0	0,99949	2,7601	BR140-like	604589	NA
chr22	50276910	50276910	T	C	NO	PLXNB2	0	0	0,99234	3,3625	Plexin B2 (	604293	NA
chr22	50281610	50281610	G	A	NO	PLXNB2	nonsynony	PLXNB2:N	0,99234	3,3625	Plexin B2 (	604293	NA
chr22	50294715	50294715	A	T	NO	PLXNB2	0	0	0,99234	3,3625	Plexin B2 (	604293	NA
chr22	50448395	50448395	G	A	NO	SBF1	nonsynony	SBF1:NM_	0,99933	0,5271	SET-bindin	603560	Charcot-M
chr22	50457029	50457029	G	C	NO	SBF1	0	0	0,99933	0,5271	SET-bindin	603560	Charcot-M
chr22	50460693	50460693	T	C	NO	SBF1	nonsynony	SBF1:NM_	0,99933	0,5271	SET-bindin	603560	Charcot-M
chr22	50464530	50464530	G	A	NO	SBF1	0	0	0,99933	0,5271	SET-bindin	603560	Charcot-M
chr22	50675190	50675190	A	G	NO	SHANK3	nonsynony	SHANK3:N	1	3,7434	SH3 and m	606230	Phelan-Mc
chr22	50721585	50721597	TGCCCAGC	-	NO	SHANK3	frameshift	SHANK3:N	1	3,7434	SH3 and m	606230	Phelan-Mc
chr22	50721898	50721899	TG	-	NO	SHANK3	frameshift	SHANK3:N	1	3,7434	SH3 and m	606230	Phelan-Mc
chrX	9743599	9743599	G	A	NO	GPR143	stopgain	GPR143:N	0,92526	0,87866	G protein-	300808	Ocular albi
chrX	13751253	13751253	C	T	NO	OFD1	stopgain	OFD1:NM_	0,95968	0,31954	OFD1 cent	300170	Simpson-G
chrX	14530969	14530969	C	T	NO	GLRA2	0	0	0,97334	2,7002	Glycine rec	305990	Intellectua
chrX	14914498	14914498	C	T	NO	MOSPD2	0	0	0,99105	1,6602	Motile spe	301086	NA
chrX	15852371	15852371	G	A	NO	AP1S2	stopgain	AP1S2:NM	0,86702	2,012	Adaptor-re	300629	Pettigrew
chrX	18564524	18564524	-	AG	YES	CDKL5	0	0	0,99932	2,7357	Cyclin-dep	300203	Developm
chrX	31126647	31126647	T	A	NO	DMD	stopgain	DMD:NM_	1	-2,4322	Dystrophin	300377	Becker mu
chrX	32411750	32411750	G	A	YES	DMD	0	0	1	-2,4322	Dystrophin	300377	Becker mu
chrX	41136799	41136799	A	G	NO	USP9X	0	0	1	6,4105	Ubiquitin-	300072	Intellectua
chrX	41342831	41342831	G	T	NO	DDX3X	stopgain	DDX3X:NM	0,99986	4,3295	DEAD-box	300160	Intellectua
chrX	45062750	45062750	T	G	YES	KDM6A	0	0	1	2,946	Lysine dem	300128	Kabuki syn
chrX	45062751	45062751	G	A	NO	KDM6A	0	0	1	2,946	Lysine dem	300128	Kabuki syn
chrX	48523959	48523959	G	A	NO	EBP	nonsynony	EBP:NM_0	0,93645	1,4942	Emopamil-	300205	MEND syn
chrX	51900286	51900286	-	CATTGCAG	NO	MAGED1	frameshift	MAGED1:N	0,99831	2,3263	Melanoma	300224	NA

chrX	53250732	53250754	ACAGAGC	-	NO	IQSEC2	frameshift	IQSEC2:NM	0,99998	5,1894	IQ motif- a	300522	Intellectua
chrX	54182866	54182866	-	T	NO	FAM120C	frameshift	FAM120C:	0,99998	3,6347	Family wit	300741	NA
chrX	54182869	54182869	-	GCGGC	NO	FAM120C	frameshift	FAM120C:	0,99998	3,6347	Family wit	300741	NA
chrX	54551747	54551747	G	A	NO	GNL3L	0	0	0,99558	1,3581	Guanine n	300873	NA
chrX	70133720	70133720	C	T	NO	IGBP1	0	0	0,98274	1,3042	Immunogl	300139	?Corpus ca
chrX	71132210	71132210	G	A	NO	MED12	0	0	1	6,5797	Mediator c	300188	Lujan-Fryn
chrX	71141374	71141374	G	T	NO	MED12	0	0	1	6,5797	Mediator c	300188	Lujan-Fryn
chrX	71250729	71250729	G	A	NO	ZMYM3	0	0	1	4,306	Zinc finger	300061	Intellectua
chrX	72474698	72474698	A	G	NO	HDAC8	stoploss	HDAC8:NM	0,97707	2,8212	Histone de	300269	Cornelia d
chrX	77895441	77895442	TT	-	NO	MAGT1	frameshift	MAGT1:N	0,96138	1,0616	Magnesiu	300715	Immunode
chrX	1,01E+08	1,01E+08	T	G	NO	GLA	0	0	0,99675	1,8759	Galactosid	300644	Fabry dise
chrX	1,01E+08	1,01E+08	GGGG	-	NO	ARMCX4	frameshift	ARMCX4:N	1	2,6373	Armadillo	301046	NA
chrX	1,01E+08	1,01E+08	C	-	NO	ARMCX4	frameshift	ARMCX4:N	1	2,6373	Armadillo	301046	NA
chrX	1,04E+08	1,04E+08	-	C	NO	TCEAL4	frameshift	TCEAL4:N	0,74827	0,9526	NA	NA	NA
chrX	1,08E+08	1,08E+08	C	T	YES	COL4A6	0	0	0,89901	1,4821	Collagen IV	303631	?Deafness
chrX	1,09E+08	1,09E+08	G	A	NO	IRS4	nonsynony	IRS4:NM_	0,57796	8,93E-02	Insulin rec	300904	Hypothyro
chrX	1,12E+08	1,12E+08	A	C	NO	ALG13	stoploss	ALG13:NM	0,99998	1,1475	ALG13 UD	300776	Developm
chrX	1,15E+08	1,15E+08	G	A	NO	LRCH2	nonsynony	LRCH2:NM	0,99713	1,9411	NA	NA	NA
chrX	1,25E+08	1,25E+08	-	A	YES	TENM1	0	0	1	3,4329	Teneurin t	300588	NA
chrX	1,25E+08	1,25E+08	T	C	NO	TENM1	0	0	1	3,4329	Teneurin t	300588	NA
chrX	1,3E+08	1,3E+08	C	T	YES	OCRL	0	0	0,99999	2,9631	OCRL inosi	300535	Dent disea
chrX	1,35E+08	1,35E+08	C	T	YES	FAM122B	0	0	0,83835	1,2604	NA	NA	NA
chrX	1,35E+08	1,35E+08	G	A	NO	FAM122B	stopgain	FAM122B:	0,83835	1,2604	NA	NA	NA
chrX	1,54E+08	1,54E+08	G	A	NO	MECP2	stopgain	MECP2:NM	0,89382	-1,2125	Methyl-Cp	300005	Rett syndr
chrX	1,54E+08	1,54E+08	G	A	NO	MECP2	stopgain	MECP2:NM	0,89382	-1,2125	Methyl-Cp	300005	Rett syndr
chrX	1,54E+08	1,54E+08	ACTC	-	NO	RPL10	frameshift	RPL10:NM	0,90915	2,6137	Ribosomal	312173	{Autism, s
chrX	1,55E+08	1,55E+08	G	A	NO	FAM3A	stopgain	FAM3A:N	0,66899	0,92684	Family wit	300492	NA
chrY	2935448	2935448	T	G	NO	ZFY	0	0	0,69359	1,5119	Zinc finger	490000	NA
chrY	12912740	12912740	G	A	NO	DDX3Y	nonsynony	DDX3Y:NM	0,96494	2,1024	DEAD-box	400010	NA
chrY	12912800	12912800	C	T	NO	DDX3Y	nonsynony	DDX3Y:NM	0,96494	2,1024	DEAD-box	400010	NA

Sfari_Gene	Sfari_Scor	Sfari_Synd	SABE1171	SABE1171	SABE1171	gnomad40	gnomad40	gnomad40	gnomad40	gnomad40	gnomad40	gnomad40	gnomad40
NA	NA	NA	0	0	0	2,00E-04	2,00E-04	2,00E-04	2,00E-04	4,00E-04	1,00E-04	1,00E-04	8,43E-05
NA	NA	NA	0	0	0	6,57E-06	6,57E-06	1,29E-05	0	2,00E-04	0	0	.
Rare Single	1	NO	0	0	0	0	0	0	0	0	0	0	0
NA	NA	NA	1	0,000427	2342	1,97E-05	1,97E-05	0	4,03E-05	7,23E-05	5,24E-06	2,45E-06	1,03E-05
NA	NA	NA	0	0	0	0	0	0	0	0	0	0	0
NA	NA	NA	0	0	0	1,00E-04	1,00E-04	9,00E-05	2,00E-04	5,00E-04	8,67E-05	7,26E-05	3,00E-04
NA	NA	NA	0	0	0	0	0	0	0	0	0	0	0
NA	NA	NA	0	0	0	0	0	0	0	0	0	0	0
NA	NA	NA	0	0	0	0	0	0	0	0	0	0	0
NA	NA	NA	0	0	0	2,64E-05	2,63E-05	0	5,39E-05	3,00E-04	8,16E-06	5,15E-06	5,39E-05
NA	NA	NA	0	0	0	0	0	0	0	0	0	0	0
NA	NA	NA	1	0,000427	2342	3,00E-04	3,00E-04	3,00E-04	3,00E-04	7,00E-04	2,00E-04	2,00E-04	2,00E-04
Rare Single	1	NO	1	0,000427	2342	2,00E-04	2,00E-04	3,00E-04	1,00E-04	6,00E-04	1,00E-04	1,00E-04	2,00E-04
Rare Single	1	NO	1	0,000427	2342	1,00E-04	1,00E-04	2,00E-04	6,73E-05	2,00E-04	8,68E-05	7,27E-05	9,91E-05
Rare Single	1	NO	0	0	0	0	0	0	0	0	0	0	0
NA	NA	NA	0	0	0	0	0	0	0	0	0	0	0
NA	NA	NA	1	0,000427	2342	3,29E-05	3,28E-05	2,57E-05	4,04E-05	4,82E-05	1,26E-05	7,98E-06	2,99E-06
NA	NA	NA	0	0	0	2,63E-05	2,63E-05	1,29E-05	4,03E-05	2,00E-04	8,14E-06	5,14E-06	.
NA	NA	NA	0	0	0	6,58E-06	6,57E-06	0	1,35E-05	2,42E-05	0	0	.
NA	NA	NA	0	0	0	0	0	0	0	0	0	0	0
NA	NA	NA	0	0	0	1,97E-05	1,97E-05	0	4,04E-05	4,41E-05	5,24E-06	2,45E-06	6,25E-06
NA	NA	NA	0	0	0	0	0	0	0	0	0	0	0
NA	NA	NA	0	0	0	1,31E-05	1,31E-05	2,57E-05	0	2,94E-05	2,18E-06	8,20E-07	1,83E-06
NA	NA	NA	0	0	0	4,00E-04	4,00E-04	3,00E-04	4,00E-04	1,40E-03	3,00E-04	3,00E-04	1,00E-03
Rare Single	1	NO	0	0	0	0	0	0	0	0	0	0	0
Rare Single	1	NO	0	0	0	0	0	0	0	0	0	0	0
NA	NA	NA	0	0	0	6,57E-06	6,57E-06	1,29E-05	0	1,47E-05	0	0	.
NA	NA	NA	0	0	0	0	0	0	0	0	0	0	0
NA	NA	NA	0	0	0	4,60E-05	4,59E-05	2,57E-05	6,71E-05	1,00E-04	2,11E-05	1,53E-05	3,34E-05
NA	NA	NA	0	0	0	6,57E-06	6,57E-06	1,29E-05	0	2,00E-04	0	0	.
NA	NA	NA	0	0	0	0	0	0	0	0	0	0	0
NA	NA	NA	0	0	0	0	0	0	0	0	0	0	0
NA	NA	NA	1	0,000427	2342	0	0	0	0	0	0	0	0
NA	NA	NA	0	0	0	3,29E-05	3,94E-05	3,85E-05	2,69E-05	1,00E-04	1,26E-05	7,98E-06	3,05E-05

NA	NA	NA	0	0	0	0	0	0	0	0	0	0	0
NA	NA	NA	0	0	0	0	0	0	0	0	0	0	0
NA	NA	NA	0	0	0	0	0	0	0	0	0	0	0
Rare Single	3	NO	0	0	0	1,31E-05	1,31E-05	1,28E-05	1,35E-05	2,00E-04	2,18E-06	8,20E-07	.
Rare Single	3	NO	0	0	0	1,31E-05	1,31E-05	0	2,69E-05	2,41E-05	2,18E-06	8,20E-07	.
Rare Single	3	NO	0	0	0	0	0	0	0	0	0	0	0
NA	NA	NA	0	0	0	0	0	0	0	0	0	0	0
NA	NA	NA	0	0	0	6,57E-06	6,57E-06	1,28E-05	0	1,47E-05	0	0	.
NA	NA	NA	0	0	0	0	0	0	0	0	0	0	0
NA	NA	NA	0	0	0	0	0	0	0	0	0	0	0
NA	NA	NA	0	0	0	1,32E-05	1,31E-05	1,29E-05	1,35E-05	6,55E-05	2,19E-06	8,20E-07	.
NA	NA	NA	1	0,000427	2342	1,31E-05	1,31E-05	1,29E-05	1,34E-05	2,94E-05	2,18E-06	8,20E-07	1,83E-06
NA	NA	NA	1	0,000427	2342	0	0	0	0	0	0	0	0
NA	NA	NA	0	0	0	0	0	0	0	0	0	0	0
NA	NA	NA	0	0	0	0	0	0	0	0	0	0	0
NA	NA	NA	0	0	0	1,97E-05	1,97E-05	0	4,04E-05	2,94E-05	5,24E-06	2,46E-06	1,83E-06
NA	NA	NA	0	0	0	6,68E-06	6,62E-06	1,30E-05	0	1,48E-05	0	0	.
NA	NA	NA	0	0	0	1,31E-05	1,31E-05	0	2,69E-05	2,94E-05	2,18E-06	8,20E-07	1,83E-06
NA	NA	NA	0	0	0	0	0	0	0	0	0	0	0
NA	NA	NA	0	0	0	0	0	0	0	0	0	0	0
NA	NA	NA	0	0	0	0	0	0	0	0	0	0	0
NA	NA	NA	0	0	0	2,04E-05	5,00E-04	3,99E-05	0	6,76E-05	5,43E-06	2,51E-06	.
NA	NA	NA	0	0	0	0	0	0	0	0	0	0	0
NA	NA	NA	0	0	0	6,57E-06	6,57E-06	1,29E-05	0	1,47E-05	0	0	.
NA	NA	NA	0	0	0	6,57E-06	6,57E-06	0	1,34E-05	1,47E-05	0	0	.
NA	NA	NA	0	0	0	0	0	0	0	0	0	0	0
Rare Single	3	NO	0	0	0	0	0	0	0	0	0	0	0
NA	NA	NA	0	0	0	3,00E-04	3,00E-04	4,00E-04	3,00E-04	1,00E-03	3,00E-04	2,00E-04	4,00E-04
NA	NA	NA	0	0	0	0	0	0	0	0	0	0	0
NA	NA	NA	0	0	0	1,32E-05	1,31E-05	1,29E-05	1,35E-05	6,55E-05	2,19E-06	8,20E-07	.
NA	NA	NA	0	0	0	2,63E-05	2,63E-05	5,15E-05	0	9,67E-05	8,15E-06	5,15E-06	1,92E-05
NA	NA	NA	1	0,000427	2342	0	0	0	0	0	0	0	0
NA	NA	NA	0	0	0	0	0	0	0	0	0	0	0
NA	NA	NA	0	0	0	2,63E-05	2,63E-05	3,86E-05	1,34E-05	4,00E-04	8,14E-06	5,14E-06	2,86E-05
Rare Single	2	NO	0	0	0	1,31E-05	1,31E-05	2,57E-05	0	2,94E-05	2,18E-06	8,20E-07	1,83E-06
Rare Single	2	NO	1	0,000427	2342	5,00E-04	5,00E-04	6,00E-04	4,00E-04	8,00E-04	4,00E-04	4,00E-04	6,00E-04

Rare Single	2	NO	0	0	0	6,62E-06	1,32E-05	0	1,36E-05	2,44E-05	0	0	.
NA	NA	NA	0	0	0	1,97E-05	1,97E-05	1,28E-05	2,69E-05	1,00E-04	5,24E-06	2,45E-06	9,07E-06
NA	NA	NA	0	0	0	6,57E-06	6,57E-06	1,29E-05	0	1,47E-05	0	0	.
NA	NA	NA	0	0	0	0	0	0	0	0	0	0	0
NA	NA	NA	1	0,000427	2342	6,57E-06	6,57E-06	0	1,35E-05	2,41E-05	0	0	.
NA	NA	NA	2	0,000854	2342	7,00E-04	7,00E-04	6,00E-04	7,00E-04	1,10E-03	6,00E-04	5,00E-04	8,00E-04
NA	NA	NA	0	0	0	3,95E-05	3,94E-05	5,15E-05	2,69E-05	3,00E-04	1,72E-05	1,13E-05	5,39E-05
NA	NA	NA	0	0	0	1,00E-04	1,00E-04	1,00E-04	9,41E-05	3,00E-04	7,58E-05	6,29E-05	1,00E-04
NA	NA	NA	0	0	0	6,58E-06	6,57E-06	0	1,35E-05	1,47E-05	0	0	.
NA	NA	NA	1	0,000427	2340	2,00E-04	2,00E-04	3,00E-04	1,00E-04	5,00E-04	1,00E-04	1,00E-04	3,00E-04
NA	NA	NA	0	0	0	0	0	0	0	0	0	0	0
NA	NA	NA	0	0	0	9,86E-05	9,85E-05	1,00E-04	6,73E-05	2,00E-04	6,01E-05	4,88E-05	8,88E-05
NA	NA	NA	0	0	0	4,60E-05	4,59E-05	5,14E-05	4,03E-05	2,00E-04	2,11E-05	1,53E-05	2,83E-05
NA	NA	NA	0	0	0	6,57E-06	6,56E-06	1,29E-05	0	1,47E-05	0	0	.
NA	NA	NA	0	0	0	6,58E-06	6,57E-06	0	1,35E-05	.	0	0	.
NA	NA	NA	0	0	0	0	0	0	0	0	0	0	0
NA	NA	NA	2	0,000854	2342	2,63E-05	2,63E-05	3,86E-05	1,35E-05	1,00E-04	8,15E-06	5,14E-06	9,09E-06
NA	NA	NA	0	0	0	0	0	0	0	0	0	0	0
NA	NA	NA	2	0,000854	2342	2,63E-05	2,63E-05	3,86E-05	1,35E-05	4,41E-05	8,14E-06	5,14E-06	6,25E-06
NA	NA	NA	0	0	0	0	0	0	0	0	0	0	0
NA	NA	NA	0	0	0	0	0	0	0	0	0	0	0
Rare Single	3	NO	0	0	0	0	0	0	0	0	0	0	0
NA	NA	NA	0	0	0	0	0	0	0	0	0	0	0
NA	NA	NA	0	0	0	6,57E-06	6,57E-06	1,29E-05	0	1,47E-05	0	0	.
NA	NA	NA	4	0,001708	2342	2,63E-05	3,28E-05	2,57E-05	2,69E-05	2,00E-04	8,14E-06	5,14E-06	2,84E-05
NA	NA	NA	0	0	0	0	0	0	0	0	0	0	0
NA	NA	NA	0	0	0	0	0	0	0	0	0	0	0
NA	NA	NA	0	0	0	1,97E-05	1,97E-05	0	4,04E-05	.	5,24E-06	2,45E-06	.
NA	NA	NA	0	0	0	0	0	0	0	0	0	0	0
NA	NA	NA	0	0	0	1,32E-05	1,31E-05	0	2,69E-05	1,47E-05	2,19E-06	8,20E-07	.
NA	NA	NA	0	0	0	7,89E-05	7,88E-05	1,00E-04	5,38E-05	1,00E-04	4,50E-05	3,51E-05	4,29E-05
NA	NA	NA	3	0,001281	2342	0,0012	0,0012	0,0012	0,0011	3,90E-03	0,001	0,001	3,20E-03
NA	NA	NA	0	0	0	6,58E-06	6,57E-06	0	1,35E-05	1,47E-05	0	0	.
NA	NA	NA	0	0	0	0	0	0	0	0	0	0	0
NA	NA	NA	0	0	0	3,63E-05	3,31E-05	2,80E-05	4,53E-05	3,00E-04	1,36E-05	8,71E-06	3,25E-05
NA	NA	NA	0	0	0	0	0	0	0	0	0	0	0

Rare Single	1	NO	0	0	0	0	0	0	0	0	0	0	0
NA	NA	NA	2	0,000854	2342	0,0016	0,0016	0,0019	0,0013	2,70E-03	0,0015	0,0014	2,20E-03
NA	NA	NA	0	0	0	1,00E-04	1,00E-04	1,00E-04	1,00E-04	6,00E-04	8,70E-05	7,28E-05	9,08E-05
NA	NA	NA	0	0	0	1,00E-04	1,00E-04	1,00E-04	1,00E-04	4,00E-04	9,74E-05	8,25E-05	1,00E-04
NA	NA	NA	0	0	0	0	0	0	0	0	0	0	0
Rare Single	1	NO	0	0	0	6,57E-06	6,57E-06	0	1,35E-05	2,42E-05	0	0	.
NA	NA	NA	0	0	0	3,29E-05	3,28E-05	2,57E-05	4,03E-05	2,00E-04	1,26E-05	7,98E-06	1,83E-06
NA	NA	NA	0	0	0	6,57E-06	6,57E-06	1,29E-05	0	2,41E-05	0	0	.
NA	NA	NA	0	0	0	0	0	0	0	0	0	0	0
NA	NA	NA	0	0	0	6,58E-05	6,57E-05	5,14E-05	8,08E-05	2,00E-04	3,52E-05	2,62E-05	6,97E-05
NA	NA	NA	0	0	0	6,58E-06	6,57E-06	1,29E-05	0	1,47E-05	0	0	.
NA	NA	NA	0	0	0	1,97E-05	1,97E-05	3,85E-05	0	4,41E-05	5,24E-06	2,45E-06	6,25E-06
NA	NA	NA	0	0	0	2,00E-04	2,00E-04	2,00E-04	3,00E-04	6,20E-03	2,00E-04	1,00E-04	3,90E-03
NA	NA	NA	0	0	0	0	6,57E-06	0	0	.	0	0	.
NA	NA	NA	0	0	0	0	0	0	0	0	0	0	0
NA	NA	NA	2	0,000854	2342	3,94E-05	3,94E-05	3,86E-05	4,04E-05	9,66E-05	1,72E-05	1,13E-05	1,92E-05
NA	NA	NA	0	0	0	6,58E-06	6,57E-06	1,29E-05	0	2,42E-05	0	0	.
NA	NA	NA	0	0	0	0	0	0	0	0	0	0	0
NA	NA	NA	0	0	0	0	0	0	0	0	0	0	0
NA	NA	NA	1	0,000427	2342	0,0018	0,0018	0,0021	0,0015	3,20E-03	0,0016	0,0016	2,70E-03
NA	NA	NA	0	0	0	0	0	0	0	0	0	0	0
NA	NA	NA	1	0,000427	2342	9,00E-04	9,00E-04	9,00E-04	9,00E-04	3,00E-03	8,00E-04	7,00E-04	2,40E-03
NA	NA	NA	1	0,000427	2342	0	0	0	0	0	0	0	0
NA	NA	NA	0	0	0	0	0	0	0	0	0	0	0
NA	NA	NA	0	0	0	3,94E-05	3,94E-05	2,57E-05	5,37E-05	4,00E-04	1,72E-05	1,13E-05	3,03E-05
NA	NA	NA	0	0	0	1,32E-05	1,31E-05	1,29E-05	1,35E-05	2,42E-05	2,19E-06	8,20E-07	.
NA	NA	NA	2	0,000854	2342	2,63E-05	2,63E-05	1,29E-05	4,04E-05	6,56E-05	8,14E-06	5,14E-06	.
NA	NA	NA	0	0	0	7,89E-05	7,88E-05	1,00E-04	4,04E-05	1,00E-04	4,50E-05	3,51E-05	5,99E-05
NA	NA	NA	0	0	0	3,28E-05	3,28E-05	2,57E-05	4,03E-05	1,00E-03	1,26E-05	7,97E-06	2,00E-04
NA	NA	NA	0	0	0	0	0	0	0	0	0	0	0
NA	NA	NA	0	0	0	3,29E-05	3,29E-05	2,57E-05	4,05E-05	2,00E-04	1,26E-05	7,99E-06	2,84E-05
NA	NA	NA	0	0	0	0	0	0	0	0	0	0	0
NA	NA	NA	0	0	0	0	0	0	0	0	0	0	0
NA	NA	NA	1	0,000427	2342	1,32E-05	1,31E-05	0	2,69E-05	4,83E-05	2,18E-06	8,20E-07	2,99E-06
NA	NA	NA	0	0	0	0	0	0	0	0	0	0	0
NA	NA	NA	0	0	0	3,94E-05	3,94E-05	5,14E-05	2,69E-05	1,00E-04	1,72E-05	1,13E-05	4,30E-05

NA	NA	NA	0	0	0	1,31E-05	1,31E-05	1,28E-05	1,35E-05	2,00E-04	2,18E-06	8,20E-07	.
NA	NA	NA	0	0	0	6,57E-06	6,57E-06	0	1,35E-05	1,47E-05	0	0	.
NA	NA	NA	0	0	0	0	0	0	0	0	0	0	0
NA	NA	NA	0	0	0	1,97E-05	1,97E-05	1,28E-05	2,69E-05	7,23E-05	5,24E-06	2,45E-06	1,03E-05
NA	NA	NA	0	0	0	3,00E-04	3,00E-04	1,00E-04	6,00E-04	9,80E-03	3,00E-04	2,00E-04	6,80E-03
NA	NA	NA	0	0	0	0	0	0	0	0	0	0	0
NA	NA	NA	0	0	0	0	0	0	0	0	0	0	0
NA	NA	NA	0	0	0	4,60E-05	4,59E-05	6,43E-05	2,69E-05	3,00E-04	2,11E-05	1,53E-05	8,28E-05
NA	NA	NA	2	0,000854	2342	3,00E-04	3,00E-04	3,00E-04	3,00E-04	1,00E-03	2,00E-04	2,00E-04	5,00E-04
NA	NA	NA	0	0	0	2,00E-04	2,00E-04	2,00E-04	3,00E-04	1,40E-03	2,00E-04	1,00E-04	8,00E-04
NA	NA	NA	0	0	0	1,97E-05	1,97E-05	1,29E-05	2,69E-05	2,94E-05	5,24E-06	2,46E-06	1,83E-06
NA	NA	NA	0	0	0	0	0	0	0	0	0	0	0
NA	NA	NA	0	0	0	5,25E-05	6,56E-05	5,14E-05	5,37E-05	1,00E-04	2,56E-05	1,83E-05	2,57E-05
NA	NA	NA	0	0	0	3,00E-04	3,00E-04	3,00E-04	4,00E-04	6,00E-04	3,00E-04	2,00E-04	4,00E-04
NA	NA	NA	0	0	0	1,31E-05	1,31E-05	0	2,69E-05	2,00E-04	2,18E-06	8,20E-07	.
NA	NA	NA	2	0,000854	2342	6,57E-06	6,57E-06	0	1,35E-05	6,55E-05	0	0	.
NA	NA	NA	1	0,000427	2342	0	0	0	0	0	0	0	0
NA	NA	NA	0	0	0	0	0	0	0	0	0	0	0
NA	NA	NA	0	0	0	7,88E-05	7,88E-05	6,42E-05	9,41E-05	2,00E-04	4,50E-05	3,51E-05	7,01E-05
NA	NA	NA	0	0	0	0	0	0	0	0	0	0	0
NA	NA	NA	0	0	0	0	0	0	0	0	0	0	0
NA	NA	NA	1	0,000427	2342	2,63E-05	2,63E-05	5,15E-05	0	7,23E-05	8,14E-06	5,14E-06	1,03E-05
NA	NA	NA	0	0	0	7,89E-05	7,88E-05	1,00E-04	4,04E-05	2,00E-04	4,50E-05	3,51E-05	6,96E-05
NA	NA	NA	3	0,001281	2342	4,00E-04	4,00E-04	6,00E-04	3,00E-04	1,60E-03	3,00E-04	3,00E-04	1,10E-03
NA	NA	NA	0	0	0	0	0	0	0	0	0	0	0
NA	NA	NA	0	0	0	6,57E-06	6,57E-06	1,29E-05	0	6,55E-05	0	0	.
NA	NA	NA	0	0	0	5,91E-05	5,91E-05	5,14E-05	6,72E-05	1,70E-03	3,08E-05	2,21E-05	7,00E-04
NA	NA	NA	0	0	0	2,00E-04	2,00E-04	2,00E-04	2,00E-04	6,00E-04	1,00E-04	9,69E-05	2,00E-04
NA	NA	NA	0	0	0	0	0	0	0	0	0	0	0
Rare Single	1	NO	1	0,000427	2342	0,0012	0,0012	0,001	0,0014	3,24E-02	0,0011	0,001	2,68E-02
NA	NA	NA	0	0	0	0	6,57E-06	0	0	.	0	0	.
NA	NA	NA	0	0	0	0	0	0	0	0	0	0	0
NA	NA	NA	0	0	0	0	0	0	0	0	0	0	0
NA	NA	NA	0	0	0	3,28E-05	3,28E-05	2,57E-05	4,03E-05	2,00E-04	1,26E-05	7,97E-06	6,25E-06
Rare Single	2	NO	0	0	0	6,57E-06	6,57E-06	1,29E-05	0	2,41E-05	0	0	.
NA	NA	NA	0	0	0	0	0	0	0	0	0	0	0

NA	NA	NA	0	0	0	1,97E-05	1,97E-05	3,85E-05	0	2,00E-04	5,23E-06	2,45E-06	.
NA	NA	NA	0	0	0	0	0	0	0	0	0	0	0
NA	NA	NA	0	0	0	0	0	0	0	0	0	0	0
NA	NA	NA	0	0	0	0	0	0	0	0	0	0	0
NA	NA	NA	0	0	0	0	0	0	0	0	0	0	0
NA	NA	NA	0	0	0	0	0	0	0	0	0	0	0
NA	NA	NA	1	0,000427	2342	4,00E-04	4,00E-04	4,00E-04	4,00E-04	7,00E-04	3,00E-04	3,00E-04	5,00E-04
NA	NA	NA	0	0	0	1,97E-05	1,97E-05	1,29E-05	2,69E-05	6,00E-04	5,24E-06	2,45E-06	8,36E-05
NA	NA	NA	3	0,001281	2342	0,001	0,001	9,00E-04	0,0011	3,60E-03	9,00E-04	8,00E-04	3,00E-03
NA	NA	NA	0	0	0	6,57E-06	6,57E-06	1,29E-05	0	1,47E-05	0	0	.
NA	NA	NA	0	0	0	0	0	0	0	0	0	0	0
NA	NA	NA	1	0,000427	2342	6,58E-06	6,57E-06	1,29E-05	0	1,47E-05	0	0	.
NA	NA	NA	0	0	0	0	0	0	0	0	0	0	0
NA	NA	NA	0	0	0	6,64E-06	6,59E-06	1,30E-05	0	1,48E-05	0	0	.
NA	NA	NA	1	0,000427	2342	5,00E-04	5,00E-04	5,00E-04	5,00E-04	1,60E-03	4,00E-04	3,00E-04	1,20E-03
Rare Single	3	NO	0	0	0	2,63E-05	2,63E-05	3,86E-05	1,34E-05	4,00E-04	8,14E-06	5,14E-06	3,04E-05
Rare Single	3	NO	0	0	0	0	0	0	0	0	0	0	0
NA	NA	NA	0	0	0	6,60E-06	6,58E-06	0	1,35E-05	2,00E-04	0	0	.
NA	NA	NA	0	0	0	0	0	0	0	0	0	0	0
NA	NA	NA	0	0	0	2,00E-04	2,00E-04	2,00E-04	2,00E-04	6,00E-04	1,00E-04	1,00E-04	4,00E-04
NA	NA	NA	1	0,000427	2342	9,20E-05	9,19E-05	1,00E-04	6,72E-05	2,00E-04	5,53E-05	4,36E-05	8,87E-05
NA	NA	NA	0	0	0	2,00E-04	2,00E-04	2,00E-04	2,00E-04	6,00E-04	1,00E-04	1,00E-04	4,00E-04
NA	NA	NA	0	0	0	5,25E-05	5,25E-05	2,57E-05	8,06E-05	4,00E-04	2,56E-05	1,83E-05	1,00E-04
NA	NA	NA	1	0,000427	2342	2,00E-04	2,00E-04	2,00E-04	2,00E-04	4,00E-04	1,00E-04	1,00E-04	1,00E-04
NA	NA	NA	0	0	0	0	0	0	0	0	0	0	0
NA	NA	NA	0	0	0	0	0	0	0	0	0	0	0
NA	NA	NA	0	0	0	6,57E-06	6,57E-06	0	1,34E-05	2,41E-05	0	0	.
NA	NA	NA	0	0	0	9,85E-05	9,85E-05	1,00E-04	5,38E-05	2,00E-04	6,01E-05	4,88E-05	7,89E-05
NA	NA	NA	0	0	0	0	0	0	0	0	0	0	0
NA	NA	NA	0	0	0	0	0	0	0	0	0	0	0
NA	NA	NA	0	0	0	1,32E-05	1,31E-05	1,29E-05	1,35E-05	2,42E-05	2,19E-06	8,20E-07	.
NA	NA	NA	0	0	0	0	0	0	0	0	0	0	0
NA	NA	NA	0	0	0	3,29E-05	3,28E-05	2,57E-05	4,04E-05	1,00E-04	1,26E-05	7,98E-06	3,05E-05
NA	NA	NA	1	0,000427	2342	0	0	0	0	0	0	0	0
NA	NA	NA	1	0,000427	2342	1,97E-05	1,97E-05	1,29E-05	2,69E-05	6,00E-04	5,24E-06	2,45E-06	8,37E-05
NA	NA	NA	0	0	0	3,00E-04	3,00E-04	3,00E-04	3,00E-04	1,10E-03	2,00E-04	2,00E-04	7,00E-04

NA	NA	NA	0	0	0	6,00E-04	6,00E-04	5,00E-04	6,00E-04	2,00E-03	5,00E-04	5,00E-04	1,50E-03
NA	NA	NA	0	0	0	0	0	0	0	0	0	0	0
NA	NA	NA	1	0,000427	2342	2,00E-04	2,00E-04	3,00E-04	1,00E-04	2,00E-04	2,00E-04	1,00E-04	1,00E-04
NA	NA	NA	0	0	0	6,57E-06	6,57E-06	0	1,35E-05	2,00E-04	0	0	.
NA	NA	NA	0	0	0	0	0	0	0	0	0	0	0
NA	NA	NA	0	0	0	0	0	0	0	0	0	0	0
NA	NA	NA	0	0	0	5,92E-05	5,91E-05	6,42E-05	5,38E-05	5,00E-04	3,08E-05	2,21E-05	2,00E-04
NA	NA	NA	1	0,000427	2342	2,63E-05	2,63E-05	3,85E-05	1,35E-05	6,55E-05	8,14E-06	5,14E-06	.
NA	NA	NA	0	0	0	6,57E-06	6,57E-06	0	1,35E-05	.	0	0	.
NA	NA	NA	0	0	0	0	0	0	0	0	0	0	0
NA	NA	NA	0	0	0	0	0	0	0	0	0	0	0
NA	NA	NA	0	0	0	1,32E-05	1,31E-05	1,29E-05	1,35E-05	4,83E-05	2,18E-06	8,20E-07	2,99E-06
NA	NA	NA	0	0	0	0	0	0	0	0	0	0	0
NA	NA	NA	0	0	0	1,32E-05	1,31E-05	2,57E-05	0	2,94E-05	2,18E-06	8,20E-07	1,83E-06
NA	NA	NA	0	0	0	0	0	0	0	0	0	0	0
NA	NA	NA	2	0,000854	2342	5,00E-04	5,00E-04	4,00E-04	5,00E-04	1,50E-03	4,00E-04	3,00E-04	1,10E-03
Genetic As	3	NO	0	0	0	0	0	0	0	0	0	0	0
NA	NA	NA	0	0	0	1,31E-05	1,31E-05	0	2,69E-05	4,00E-04	2,18E-06	8,20E-07	2,86E-05
NA	NA	NA	0	0	0	0,0011	0,0011	0,001	0,0011	1,60E-03	9,00E-04	9,00E-04	1,30E-03
NA	NA	NA	0	0	0	0	0	0	0	0	0	0	0
NA	NA	NA	0	0	0	6,57E-06	6,57E-06	1,28E-05	0	1,47E-05	0	0	.
NA	NA	NA	1	0,000427	2342	6,58E-06	6,57E-06	0	1,35E-05	1,47E-05	0	0	.
NA	NA	NA	0	0	0	2,64E-05	2,63E-05	3,87E-05	1,35E-05	6,57E-05	8,17E-06	5,16E-06	6,25E-06
NA	NA	NA	0	0	0	6,57E-06	6,57E-06	1,29E-05	0	1,47E-05	0	0	.
Rare Single	2	NO	0	0	0	1,00E-04	1,00E-04	1,00E-04	1,00E-04	1,20E-03	8,16E-05	6,72E-05	7,00E-04
Rare Single	3	NO	0	0	0	0	0	0	0	0	0	0	0
NA	NA	NA	3	0,001281	2342	7,00E-04	7,00E-04	8,00E-04	6,00E-04	1,20E-03	6,00E-04	6,00E-04	1,00E-03
NA	NA	NA	0	0	0	0	0	0	0	0	0	0	0
NA	NA	NA	0	0	0	1,00E-04	1,00E-04	2,00E-04	9,42E-05	2,00E-04	8,17E-05	6,73E-05	1,00E-04
Rare Single	3	NO	2	0,000854	2342	4,00E-04	4,00E-04	4,00E-04	3,00E-04	1,20E-03	3,00E-04	3,00E-04	8,00E-04
Rare Single	1	NO	0	0	0	0	0	0	0	0	0	0	0
Rare Single	3	YES	0	0	0	2,00E-04	2,00E-04	9,00E-05	2,00E-04	4,40E-03	1,00E-04	8,71E-05	2,60E-03
Rare Single	3	YES	0	0	0	0	0	0	0	0	0	0	0
NA	NA	NA	0	0	0	3,94E-05	3,94E-05	6,42E-05	1,35E-05	1,00E-04	1,72E-05	1,13E-05	4,30E-05
NA	NA	NA	0	0	0	3,95E-05	3,94E-05	5,15E-05	2,69E-05	7,35E-05	1,72E-05	1,13E-05	1,86E-05
NA	NA	NA	0	0	0	0	0	0	0	0	0	0	0

Rare Single	3	NO	3	0,001281	2342	7,00E-04	7,00E-04	7,00E-04	7,00E-04	2,40E-03	6,00E-04	5,00E-04	1,90E-03
Rare Single	3	NO	0	0	0	0	0	0	0	0	0	0	0
Rare Single	3	NO	0	0	0	0	0	0	0	0	0	0	0
Rare Single	1	NO	1	0,000427	2342	2,63E-05	2,63E-05	2,57E-05	2,69E-05	2,00E-04	8,14E-06	5,14E-06	2,84E-05
Rare Single	1	NO	0	0	0	6,58E-06	6,57E-06	0	1,35E-05	2,42E-05	0	0	.
Rare Single	3	NO	0	0	0	6,58E-06	6,57E-06	0	1,35E-05	2,42E-05	0	0	.
NA	NA	NA	1	0,000427	2342	5,00E-04	5,00E-04	6,00E-04	4,00E-04	1,60E-03	4,00E-04	4,00E-04	1,20E-03
Rare Single	1	NO	0	0	0	0	0	0	0	0	0	0	0
Rare Single	3	NO	0	0	0	0	0	0	0	0	0	0	0
Rare Single	3	NO	0	0	0	0	0	0	0	0	0	0	0
Rare Single	3	NO	0	0	0	0	0	0	0	0	0	0	0
NA	NA	NA	0	0	0	0	0	0	0	0	0	0	0
NA	NA	NA	0	0	0	0	0	0	0	0	0	0	0
NA	NA	NA	0	0	0	0	0	0	0	0	0	0	0
Genetic As	3	NO	0	0	0	1,97E-05	1,97E-05	2,57E-05	1,34E-05	1,00E-04	5,24E-06	2,45E-06	9,07E-06
Rare Single	3	NO	0	0	0	1,97E-05	1,97E-05	2,57E-05	1,34E-05	7,22E-05	5,24E-06	2,45E-06	1,03E-05
Rare Single	3	NO	0	0	0	7,88E-05	7,87E-05	1,00E-04	4,03E-05	1,00E-04	4,49E-05	3,51E-05	5,09E-05
NA	NA	NA	0	0	0	0	0	0	0	0	0	0	0
NA	NA	NA	0	0	0	5,26E-05	5,25E-05	6,42E-05	4,04E-05	1,00E-04	2,56E-05	1,83E-05	4,24E-05
NA	NA	NA	0	0	0	0	0	0	0	0	0	0	0
NA	NA	NA	0	0	0	6,57E-06	6,57E-06	0	1,35E-05	1,47E-05	0	0	.
Rare Single	1	NO	0	0	0	6,58E-06	6,57E-06	1,29E-05	0	2,42E-05	0	0	.
NA	NA	NA	0	0	0	1,32E-05	1,97E-05	1,29E-05	1,35E-05	2,95E-05	2,19E-06	8,20E-07	1,83E-06
NA	NA	NA	0	0	0	4,00E-04	4,00E-04	3,00E-04	4,00E-04	4,00E-04	3,00E-04	3,00E-04	2,00E-04
NA	NA	NA	0	0	0	0,0014	0,0014	0,0014	0,0013	4,50E-03	0,0012	0,0012	3,70E-03
Rare Single	2	NO	0	0	0	0	0	0	0	0	0	0	0
NA	NA	NA	0	0	0	1,31E-05	1,31E-05	2,57E-05	0	6,54E-05	2,18E-06	8,20E-07	.
Rare Single	3	YES	0	0	0	0	0	0	0	0	0	0	0
NA	NA	NA	0	0	0	0	0	0	0	0	0	0	0
NA	NA	NA	0	0	0	3,00E-04	3,00E-04	2,00E-04	4,00E-04	1,00E-03	2,00E-04	2,00E-04	7,00E-04
NA	NA	NA	0	0	0	0	0	0	0	0	0	0	0
NA	NA	NA	0	0	0	2,00E-04	2,00E-04	1,00E-04	2,00E-04	6,00E-04	1,00E-04	8,73E-05	3,00E-04
NA	NA	NA	0	0	0	0	0	0	0	0	0	0	0
Rare Single	3	NO	0	0	0	0	0	0	0	0	0	0	0
Rare Single	3	NO	0	0	0	5,26E-05	5,25E-05	5,14E-05	5,38E-05	8,82E-05	2,56E-05	1,83E-05	2,57E-05
NA	NA	NA	2	0,000854	2342	3,00E-04	3,00E-04	3,00E-04	2,00E-04	4,00E-04	2,00E-04	2,00E-04	2,00E-04

NA	NA	NA	2	0,000854	2342	1,31E-05	1,31E-05	0	2,69E-05	6,54E-05	2,18E-06	8,20E-07	.
NA	NA	NA	0	0	0	0	0	0	0	0	0	0	0
NA	NA	NA	0	0	0	0	0	0	0	0	0	0	0
NA	NA	NA	0	0	0	0	0	0	0	0	0	0	0
NA	NA	NA	0	0	0	0	0	0	0	0	0	0	0
NA	NA	NA	0	0	0	2,00E-04	2,00E-04	2,00E-04	2,00E-04	6,00E-04	2,00E-04	1,00E-04	2,00E-04
NA	NA	NA	0	0	0	3,00E-04	3,00E-04	4,00E-04	2,00E-04	1,10E-03	2,00E-04	2,00E-04	8,00E-04
NA	NA	NA	1	0,000427	2342	2,00E-04	2,00E-04	2,00E-04	2,00E-04	5,00E-04	1,00E-04	9,24E-05	3,00E-04
NA	NA	NA	0	0	0	3,94E-05	3,94E-05	3,85E-05	4,03E-05	7,35E-05	1,72E-05	1,13E-05	1,86E-05
Rare Single	1	NO	0	0	0	0	0	0	0	0	0	0	0
NA	NA	NA	0	0	0	6,57E-06	6,57E-06	1,28E-05	0	2,41E-05	0	0	.
NA	NA	NA	0	0	0	0	0	0	0	0	0	0	0
NA	NA	NA	0	0	0	3,00E-04	3,00E-04	4,00E-04	2,00E-04	6,00E-04	2,00E-04	2,00E-04	4,00E-04
Rare Single	1	NO	0	0	0	0	0	0	0	0	0	0	0
Rare Single	1	NO	0	0	0	0	0	0	0	0	0	0	0
NA	NA	NA	0	0	0	3,00E-04	3,00E-04	2,00E-04	3,00E-04	9,40E-03	2,00E-04	2,00E-04	6,20E-03
NA	NA	NA	0	0	0	6,57E-06	6,57E-06	0	1,35E-05	2,41E-05	0	0	.
NA	NA	NA	0	0	0	0	0	0	0	0	0	0	0
NA	NA	NA	0	0	0	0	0	0	0	0	0	0	0
NA	NA	NA	0	0	0	6,57E-06	6,57E-06	0	1,35E-05	6,55E-05	0	0	.
Rare Single	1	NO	2	0,000854	2342	2,63E-05	2,63E-05	2,57E-05	2,69E-05	2,00E-04	8,15E-06	5,14E-06	1,83E-06
NA	NA	NA	0	0	0	0	0	0	0	0	0	0	0
Rare Single	3	NO	0	0	0	5,26E-05	5,25E-05	5,14E-05	5,38E-05	1,00E-04	2,56E-05	1,83E-05	1,13E-05
NA	NA	NA	0	0	0	0,0013	0,0013	0,001	0,0017	3,32E-02	0,0011	0,0011	2,76E-02
NA	NA	NA	0	0	0	0	0	0	0	0	0	0	0
Rare Single	2	NO	5	0,002135	2342	5,00E-04	5,00E-04	5,00E-04	4,00E-04	8,00E-04	4,00E-04	4,00E-04	6,00E-04
NA	NA	NA	0	0	0	0	0	0	0	0	0	0	0
NA	NA	NA	0	0	0	0	0	0	0	0	0	0	0
Rare Single	1	NO	0	0	0	6,57E-06	6,57E-06	1,29E-05	0	2,41E-05	0	0	.
NA	NA	NA	0	0	0	0	0	0	0	0	0	0	0
Rare Single	3	NO	0	0	0	0	0	0	0	0	0	0	0
Rare Single	3	NO	0	0	0	1,97E-05	1,97E-05	0	4,03E-05	4,00E-04	5,24E-06	2,45E-06	3,04E-05
Rare Single	3	NO	0	0	0	0	0	0	0	0	0	0	0
Rare Single	3	NO	0	0	0	0	0	0	0	0	0	0	0
Rare Single	1	NO	0	0	0	6,57E-06	6,57E-06	0	1,35E-05	1,47E-05	0	0	.
NA	NA	NA	0	0	0	1,31E-05	1,31E-05	1,29E-05	1,35E-05	4,83E-05	2,18E-06	8,20E-07	2,99E-06

NA	NA	NA	0	0	0	3,29E-05	3,28E-05	3,86E-05	2,69E-05	9,66E-05	1,26E-05	7,98E-06	1,92E-05
NA	NA	NA	0	0	0	9,85E-05	9,84E-05	9,00E-05	1,00E-04	3,00E-04	6,00E-05	4,88E-05	1,00E-04
NA	NA	NA	0	0	0	4,61E-05	4,60E-05	7,72E-05	1,35E-05	7,36E-05	2,11E-05	1,53E-05	1,86E-05
NA	NA	NA	1	0,000427	2342	7,00E-04	7,00E-04	8,00E-04	6,00E-04	2,50E-03	6,00E-04	5,00E-04	1,50E-03
NA	NA	NA	0	0	0	7,27E-05	7,01E-05	8,40E-05	6,05E-05	3,00E-04	3,87E-05	2,97E-05	1,00E-04
NA	NA	NA	0	0	0	1,97E-05	1,97E-05	2,57E-05	1,35E-05	1,00E-04	5,24E-06	2,45E-06	9,07E-06
NA	NA	NA	0	0	0	0	0	0	0	0	0	0	0
NA	NA	NA	2	0,000855	2340	8,77E-05	8,67E-05	9,91E-05	7,56E-05	6,00E-04	4,97E-05	3,88E-05	4,55E-05
NA	NA	NA	0	0	0	0	0	0	0	0	0	0	0
NA	NA	NA	1	0,000427	2342	6,57E-06	6,57E-06	0	1,35E-05	6,55E-05	0	0	.
NA	NA	NA	0	0	0	0	0	0	0	0	0	0	0
NA	NA	NA	0	0	0	1,98E-05	1,97E-05	1,29E-05	2,70E-05	4,00E-04	5,25E-06	2,46E-06	2,86E-05
NA	NA	NA	1	0,000427	2342	1,00E-04	1,00E-04	1,00E-04	1,00E-04	6,00E-04	8,66E-05	7,26E-05	2,00E-04
NA	NA	NA	0	0	0	0	0	0	0	0	0	0	0
NA	NA	NA	0	0	0	0	0	0	0	0	0	0	0
NA	NA	NA	0	0	0	0	0	0	0	0	0	0	0
NA	NA	NA	0	0	0	6,57E-06	6,57E-06	1,28E-05	0	2,41E-05	0	0	.
NA	NA	NA	0	0	0	6,57E-06	6,57E-06	1,28E-05	0	1,47E-05	0	0	.
Rare Single	1	NO	0	0	0	3,00E-04	3,00E-04	3,00E-04	2,00E-04	1,00E-03	2,00E-04	2,00E-04	6,00E-04
NA	NA	NA	0	0	0	6,58E-06	6,57E-06	1,29E-05	0	2,42E-05	0	0	.
NA	NA	NA	0	0	0	0	0	0	0	0	0	0	0
NA	NA	NA	0	0	0	4,61E-05	4,60E-05	6,43E-05	2,69E-05	1,00E-04	2,11E-05	1,53E-05	1,13E-05
Rare Single	2	NO	0	0	0	1,31E-05	1,31E-05	2,57E-05	0	2,00E-04	2,18E-06	8,20E-07	.
NA	NA	NA	0	0	0	0	0	0	0	0	0	0	0
NA	NA	NA	0	0	0	7,00E-04	7,00E-04	6,00E-04	9,00E-04	8,00E-04	6,00E-04	6,00E-04	6,00E-04
NA	NA	NA	1	0,000427	2342	3,00E-04	3,00E-04	3,00E-04	3,00E-04	1,10E-03	2,00E-04	2,00E-04	8,00E-04
NA	NA	NA	0	0	0	2,63E-05	2,63E-05	2,57E-05	2,69E-05	8,00E-04	8,14E-06	5,14E-06	2,00E-04
NA	NA	NA	0	0	0	1,32E-05	1,31E-05	0	2,70E-05	2,94E-05	2,19E-06	8,20E-07	1,83E-06
NA	NA	NA	0	0	0	0	0	0	0	0	0	0	0
NA	NA	NA	0	0	0	5,26E-05	5,25E-05	5,14E-05	5,38E-05	2,00E-04	2,56E-05	1,83E-05	5,59E-05
NA	NA	NA	0	0	0	1,00E-04	1,00E-04	1,00E-04	1,00E-04	5,00E-04	9,76E-05	8,27E-05	3,00E-04
NA	NA	NA	0	0	0	3,56E-05	7,00E-04	3,48E-05	3,66E-05	1,00E-04	1,13E-05	6,61E-06	1,45E-05
NA	NA	NA	0	0	0	5,91E-05	5,91E-05	5,14E-05	6,73E-05	8,82E-05	3,08E-05	2,21E-05	2,57E-05
NA	NA	NA	0	0	0	7,00E-04	7,00E-04	7,00E-04	7,00E-04	1,20E-03	6,00E-04	5,00E-04	9,00E-04
NA	NA	NA	0	0	0	6,57E-06	6,57E-06	1,29E-05	0	1,47E-05	0	0	.
NA	NA	NA	1	0,000427	2342	5,91E-05	5,91E-05	5,14E-05	6,71E-05	6,00E-04	3,08E-05	2,21E-05	2,00E-04

NA	NA	NA	0	0	0	5,26E-05	5,25E-05	3,85E-05	6,72E-05	8,82E-05	2,56E-05	1,83E-05	2,57E-05
Rare Single	2	NO	1	0,000427	2342	5,91E-05	5,91E-05	7,71E-05	4,04E-05	1,00E-04	3,08E-05	2,21E-05	3,34E-05
Rare Single	2	NO	0	0	0	6,58E-06	1,31E-05	1,29E-05	0	6,55E-05	0	0	.
Rare Single	1	NO	0	0	0	0	0	0	0	0	0	0	0
Rare Single	1	NO	0	0	0	0	0	0	0	0	0	0	0
NA	NA	NA	0	0	0	0	0	0	0	0	0	0	0
NA	NA	NA	1	0,000427	2342	3,29E-05	3,94E-05	1,29E-05	5,38E-05	3,00E-04	1,26E-05	7,98E-06	5,39E-05
NA	NA	NA	0	0	0	6,58E-06	6,57E-06	1,29E-05	0	1,47E-05	0	0	.
NA	NA	NA	0	0	0	3,29E-05	3,29E-05	1,29E-05	5,39E-05	2,00E-04	1,26E-05	7,99E-06	1,13E-05
NA	NA	NA	0	0	0	0	0	0	0	0	0	0	0
NA	NA	NA	0	0	0	8,00E-04	7,00E-04	8,00E-04	7,00E-04	2,60E-03	6,00E-04	6,00E-04	2,10E-03
NA	NA	NA	0	0	0	8,00E-04	7,00E-04	8,00E-04	7,00E-04	2,60E-03	6,00E-04	6,00E-04	2,10E-03
NA	NA	NA	0	0	0	3,30E-05	3,29E-05	3,86E-05	2,70E-05	2,00E-04	1,26E-05	8,00E-06	2,84E-05
NA	NA	NA	0	0	0	2,00E-04	2,00E-04	2,00E-04	3,00E-04	1,30E-03	2,00E-04	2,00E-04	7,00E-04
NA	NA	NA	0	0	0	0	0	0	0	0	0	0	0
NA	NA	NA	0	0	0	0	0	0	0	0	0	0	0
NA	NA	NA	2	0,000854	2342	0,0016	0,0016	0,0018	0,0015	5,70E-03	0,0015	0,0014	4,90E-03
Rare Single	1	NO	0	0	0	0	0	0	0	0	0	0	0
Rare Single	1	NO	0	0	0	0	0	0	0	0	0	0	0
Rare Single	2	NO	0	0	0	6,58E-06	6,57E-06	0	1,35E-05	.	0	0	.
NA	NA	NA	0	0	0	6,00E-04	6,00E-04	5,00E-04	6,00E-04	1,80E-03	5,00E-04	4,00E-04	1,40E-03
NA	NA	NA	0	0	0	1,32E-05	1,31E-05	1,29E-05	1,35E-05	1,47E-05	2,19E-06	8,20E-07	.
NA	NA	NA	0	0	0	0	0	0	0	0	0	0	0
NA	NA	NA	0	0	0	6,58E-06	6,57E-06	1,29E-05	0	1,47E-05	0	0	.
NA	NA	NA	0	0	0	0	0	0	0	0	0	0	0
NA	NA	NA	7	0,002989	2342	0,0015	0,0015	0,0016	0,0014	5,10E-03	0,0014	0,0013	4,30E-03
NA	NA	NA	0	0	0	0	0	0	0	0	0	0	0
Rare Single	3	NO	0	0	0	0	0	0	0	0	0	0	0
NA	NA	NA	0	0	0	6,57E-06	6,57E-06	1,29E-05	0	6,55E-05	0	0	.
NA	NA	NA	0	0	0	3,94E-05	3,94E-05	3,85E-05	4,03E-05	1,00E-04	1,72E-05	1,13E-05	9,07E-06
NA	NA	NA	0	0	0	1,31E-05	1,31E-05	2,57E-05	0	1,47E-05	2,18E-06	8,20E-07	.
NA	NA	NA	0	0	0	0	0	0	0	0	0	0	0
NA	NA	NA	0	0	0	6,57E-06	6,57E-06	0	1,35E-05	1,47E-05	0	0	.
NA	NA	NA	0	0	0	0	0	0	0	0	0	0	0
NA	NA	NA	0	0	0	1,32E-05	1,31E-05	2,57E-05	0	2,42E-05	2,18E-06	8,20E-07	.
NA	NA	NA	0	0	0	0	0	0	0	0	0	0	0

NA	NA	NA	0	0	0	0	0	0	0	0	0	0	0
NA	NA	NA	1	0,000427	2342	3,29E-05	3,28E-05	2,57E-05	4,04E-05	4,41E-05	1,26E-05	7,98E-06	6,25E-06
NA	NA	NA	0	0	0	1,00E-04	1,00E-04	7,71E-05	2,00E-04	1,40E-03	9,74E-05	8,26E-05	4,00E-04
NA	NA	NA	0	0	0	0	0	0	0	0	0	0	0
NA	NA	NA	1	0,000427	2342	0	0	0	0	0	0	0	0
NA	NA	NA	0	0	0	6,58E-06	6,57E-06	1,29E-05	0	2,42E-05	0	0	.
NA	NA	NA	0	0	0	6,57E-06	6,57E-06	1,29E-05	0	2,41E-05	0	0	.
NA	NA	NA	0	0	0	0	0	0	0	0	0	0	0
Rare Single	3	YES	0	0	0	0	0	0	0	0	0	0	0
NA	NA	NA	1	0,000427	2342	6,58E-06	6,57E-06	0	1,35E-05	2,00E-04	0	0	.
NA	NA	NA	0	0	0	6,00E-04	6,00E-04	6,00E-04	6,00E-04	2,00E-03	5,00E-04	5,00E-04	1,60E-03
NA	NA	NA	0	0	0	0	0	0	0	0	0	0	0
NA	NA	NA	0	0	0	6,57E-06	6,57E-06	1,28E-05	0	1,47E-05	0	0	.
Rare Single	3	NO	1	0,000427	2342	0	0	0	0	0	0	0	0
NA	NA	NA	0	0	0	0	0	0	0	0	0	0	0
Rare Single	3	NO	0	0	0	6,57E-06	6,57E-06	0	1,35E-05	2,41E-05	0	0	.
NA	NA	NA	0	0	0	0	0	0	0	0	0	0	0
NA	NA	NA	0	0	0	0	0	0	0	0	0	0	0
NA	NA	NA	0	0	0	1,97E-05	1,97E-05	1,29E-05	2,69E-05	4,41E-05	5,24E-06	2,45E-06	6,25E-06
NA	NA	NA	1	0,000427	2342	4,00E-04	4,00E-04	3,00E-04	5,00E-04	1,02E-02	3,00E-04	3,00E-04	7,10E-03
Rare Single	3	NO	0	0	0	8,55E-05	8,53E-05	1,00E-04	6,73E-05	2,50E-03	4,96E-05	3,97E-05	1,10E-03
NA	NA	NA	1	0,000427	2342	7,00E-04	7,00E-04	7,00E-04	6,00E-04	2,40E-03	6,00E-04	5,00E-04	1,80E-03
NA	NA	NA	0	0	0	0	0	0	0	0	0	0	0
NA	NA	NA	1	0,000427	2342	6,00E-04	6,00E-04	4,00E-04	8,00E-04	3,50E-03	5,00E-04	5,00E-04	1,80E-03
NA	NA	NA	0	0	0	2,00E-04	2,00E-04	2,00E-04	2,00E-04	2,20E-03	1,00E-04	1,00E-04	1,00E-03
NA	NA	NA	0	0	0	2,00E-04	2,00E-04	2,00E-04	2,00E-04	2,20E-03	1,00E-04	1,00E-04	1,00E-03
NA	NA	NA	6	0,002566	2338	0,0016	0,0018	0,0016	0,0016	5,20E-03	0,0015	0,0014	4,30E-03
NA	NA	NA	6	0,002566	2338	0,0016	0,0018	0,0017	0,0015	5,00E-03	0,0014	0,0014	4,20E-03
NA	NA	NA	8	0,003419	2340	0,0016	0,0016	0,0015	0,0017	2,10E-03	0,0014	0,0014	1,50E-03
NA	NA	NA	8	0,003431	2332	0,0017	0,0017	0,0016	0,0019	2,20E-03	0,0016	0,0015	1,50E-03
NA	NA	NA	6	0,002795	2147	0,0022	0,0028	0,002	0,0023	8,20E-03	0,002	0,0019	7,10E-03
NA	NA	NA	0	0	0	1,97E-05	1,97E-05	2,57E-05	1,35E-05	2,94E-05	5,25E-06	2,46E-06	1,83E-06
NA	NA	NA	0	0	0	1,31E-05	1,97E-05	1,29E-05	1,34E-05	4,00E-04	2,18E-06	8,20E-07	2,86E-05
NA	NA	NA	1	0,000427	2342	3,00E-04	3,00E-04	4,00E-04	2,00E-04	1,00E-03	2,00E-04	2,00E-04	7,00E-04
NA	NA	NA	0	0	0	0	0	0	0	0	0	0	0
NA	NA	NA	0	0	0	0	0	0	0	0	0	0	0

NA	NA	NA	0	0	0	5,26E-05	5,25E-05	6,43E-05	4,03E-05	2,00E-04	2,56E-05	1,83E-05	4,29E-05
NA	NA	NA	0	0	0	0	0	0	0	0	0	0	0
NA	NA	NA	1	0,000427	2342	8,55E-05	8,54E-05	1,00E-04	6,73E-05	2,00E-04	4,96E-05	3,96E-05	7,01E-05
NA	NA	NA	0	0	0	1,32E-05	1,31E-05	1,29E-05	1,35E-05	2,42E-05	2,19E-06	8,20E-07	.
NA	NA	NA	0	0	0	0	0	0	0	0	0	0	0
NA	NA	NA	0	0	0	0	0	0	0	0	0	0	0
NA	NA	NA	0	0	0	0	0	0	0	0	0	0	0
NA	NA	NA	1	0,000427	2342	5,91E-05	5,91E-05	6,42E-05	5,37E-05	1,00E-04	3,08E-05	2,21E-05	3,34E-05
NA	NA	NA	0	0	0	0	0	0	0	0	0	0	0
NA	NA	NA	3	0,001281	2342	0,0018	0,0018	0,002	0,0016	3,20E-03	0,0016	0,0016	2,70E-03
NA	NA	NA	1	0,000427	2342	6,58E-06	6,57E-06	0	1,35E-05	6,55E-05	0	0	.
NA	NA	NA	1	0,000427	2342	9,86E-05	9,85E-05	7,71E-05	1,00E-04	3,00E-04	6,01E-05	4,88E-05	1,00E-04
NA	NA	NA	0	0	0	6,57E-06	6,57E-06	1,29E-05	0	2,41E-05	0	0	.
NA	NA	NA	0	0	0	0	0	0	0	0	0	0	0
Rare Single	1	NO	0	0	0	6,57E-06	6,57E-06	0	1,35E-05	.	0	0	.
Rare Single	1	NO	0	0	0	0	0	0	0	0	0	0	0
Rare Single	1	NO	0	0	0	2,63E-05	2,63E-05	2,57E-05	2,69E-05	5,88E-05	8,14E-06	5,14E-06	1,13E-05
NA	NA	NA	0	0	0	1,32E-05	1,31E-05	2,57E-05	0	2,94E-05	2,18E-06	8,20E-07	1,83E-06
NA	NA	NA	0	0	0	1,00E-04	1,00E-04	5,14E-05	2,00E-04	3,00E-04	8,66E-05	7,25E-05	8,88E-05
NA	NA	NA	0	0	0	0	0	0	0	0	0	0	0
NA	NA	NA	0	0	0	0	0	0	0	0	0	0	0
NA	NA	NA	0	0	0	0	0	0	0	0	0	0	0
NA	NA	NA	0	0	0	4,00E-04	4,00E-04	4,00E-04	4,00E-04	1,40E-03	3,00E-04	3,00E-04	1,00E-03
Rare Single	1	NO	0	0	0	0	0	0	0	0	0	0	0
Rare Single	1	NO	0	0	0	0	0	0	0	0	0	0	0
Rare Single	1	NO	0	0	0	1,31E-05	1,31E-05	1,29E-05	1,35E-05	2,94E-05	2,18E-06	8,20E-07	1,83E-06
NA	NA	NA	0	0	0	0	0	0	0	0	0	0	0
NA	NA	NA	0	0	0	0	0	0	0	0	0	0	0
Rare Single	3	NO	0	0	0	0	0	0	0	0	0	0	0
Rare Single	3	NO	0	0	0	1,31E-05	1,31E-05	1,28E-05	1,34E-05	1,47E-05	2,18E-06	8,20E-07	.
Rare Single	3	NO	0	0	0	0	0	0	0	0	0	0	0
NA	NA	NA	0	0	0	0	0	0	0	0	0	0	0
NA	NA	NA	3	0,001281	2342	0,0016	0,0016	0,0018	0,0015	5,80E-03	0,0015	0,0014	5,00E-03
NA	NA	NA	0	0	0	0	0	0	0	0	0	0	0
Genetic As	3	NO	0	0	0	0	0	0	0	0	0	0	0
NA	NA	NA	0	0	0	6,57E-06	6,57E-06	1,28E-05	0	6,54E-05	0	0	.

NA	NA	NA	0	0	0	0	0	0	0	0	0	0	0
NA	NA	NA	2	0,000854	2342	0,0018	0,0017	0,0017	0,002	8,20E-03	0,0016	0,0015	6,60E-03
NA	NA	NA	0	0	0	1,97E-05	1,97E-05	1,29E-05	2,70E-05	2,00E-04	5,25E-06	2,46E-06	1,83E-06
Rare Single	1	NO	1	0,000427	2342	5,00E-04	5,00E-04	6,00E-04	4,00E-04	1,70E-03	4,00E-04	4,00E-04	1,30E-03
Rare Single	1	NO	0	0	0	1,31E-05	1,31E-05	1,29E-05	1,35E-05	2,94E-05	2,18E-06	8,20E-07	1,83E-06
Rare Single	1	NO	0	0	0	3,94E-05	3,94E-05	2,57E-05	5,38E-05	9,65E-05	1,72E-05	1,13E-05	1,91E-05
NA	NA	NA	0	0	0	5,91E-05	5,91E-05	2,57E-05	9,41E-05	1,70E-03	3,08E-05	2,21E-05	7,00E-04
NA	NA	NA	0	0	0	6,57E-06	6,57E-06	1,29E-05	0	2,41E-05	0	0	.
NA	NA	NA	0	0	0	1,97E-05	1,97E-05	2,57E-05	1,35E-05	2,00E-04	5,24E-06	2,45E-06	.
NA	NA	NA	0	0	0	0	0	0	0	0	0	0	0
NA	NA	NA	0	0	0	0	0	0	0	0	0	0	0
NA	NA	NA	0	0	0	6,58E-06	6,57E-06	1,29E-05	0	6,55E-05	0	0	.
NA	NA	NA	1	0,000427	2342	6,57E-05	6,57E-05	2,57E-05	1,00E-04	1,00E-04	3,52E-05	2,62E-05	2,57E-05
Rare Single	1	NO	0	0	0	2,63E-05	2,63E-05	3,86E-05	1,35E-05	2,94E-05	8,15E-06	5,14E-06	1,83E-06
NA	NA	NA	0	0	0	6,57E-06	6,57E-06	0	1,35E-05	1,47E-05	0	0	.
NA	NA	NA	0	0	0	0	0	0	0	0	0	0	0
NA	NA	NA	0	0	0	1,31E-05	1,31E-05	1,29E-05	1,35E-05	1,47E-05	2,18E-06	8,20E-07	.
NA	NA	NA	3	0,001281	2342	2,00E-04	2,00E-04	2,00E-04	2,00E-04	1,10E-03	1,00E-04	1,00E-04	6,00E-04
NA	NA	NA	1	0,000427	2342	3,94E-05	3,94E-05	3,85E-05	4,04E-05	7,35E-05	1,72E-05	1,13E-05	1,86E-05
NA	NA	NA	0	0	0	2,00E-04	2,00E-04	2,00E-04	2,00E-04	1,00E-04	2,00E-04	1,00E-04	3,34E-05
NA	NA	NA	0	0	0	1,97E-05	1,97E-05	3,86E-05	0	6,55E-05	5,25E-06	2,46E-06	1,83E-06
NA	NA	NA	0	0	0	0	0	0	0	0	0	0	0
NA	NA	NA	0	0	0	5,00E-04	5,00E-04	4,00E-04	6,00E-04	1,00E-03	4,00E-04	4,00E-04	7,00E-04
NA	NA	NA	0	0	0	6,58E-06	6,57E-06	1,29E-05	0	1,47E-05	0	0	.
NA	NA	NA	0	0	0	6,58E-06	6,57E-06	1,29E-05	0	6,55E-05	0	0	.
NA	NA	NA	1	0,000427	2342	6,57E-06	6,57E-06	1,28E-05	0	.	0	0	.
NA	NA	NA	3	0,001281	2342	6,00E-04	6,00E-04	5,00E-04	7,00E-04	2,30E-03	5,00E-04	5,00E-04	1,80E-03
NA	NA	NA	0	0	0	6,66E-06	6,60E-06	1,30E-05	0	2,44E-05	0	0	.
NA	NA	NA	0	0	0	6,58E-06	6,57E-06	1,29E-05	0	1,47E-05	0	0	.
NA	NA	NA	0	0	0	6,58E-06	6,57E-06	0	1,35E-05	2,00E-04	0	0	.
NA	NA	NA	0	0	0	0	0	0	0	0	0	0	0
NA	NA	NA	0	0	0	0	0	0	0	0	0	0	0
NA	NA	NA	0	0	0	0	0	0	0	0	0	0	0
NA	NA	NA	0	0	0	0	0	0	0	0	0	0	0
NA	NA	NA	1	0,000427	2342	3,00E-04	3,00E-04	3,00E-04	4,00E-04	1,20E-03	3,00E-04	2,00E-04	8,00E-04
NA	NA	NA	0	0	0	1,31E-05	1,31E-05	2,57E-05	0	6,55E-05	2,18E-06	8,20E-07	.

NA	NA	NA	0	0	0	3,29E-05	3,28E-05	3,86E-05	2,69E-05	5,88E-05	1,26E-05	7,98E-06	1,12E-05
NA	NA	NA	0	0	0	0	0	0	0	0	0	0	0
NA	NA	NA	0	0	0	2,02E-05	4,64E-05	2,62E-05	1,39E-05	7,43E-05	5,38E-06	2,49E-06	1,05E-05
NA	NA	NA	0	0	0	0	0	0	0	0	0	0	0
NA	NA	NA	0	0	0	8,00E-04	8,00E-04	8,00E-04	8,00E-04	2,90E-03	7,00E-04	7,00E-04	2,30E-03
NA	NA	NA	0	0	0	3,95E-05	3,94E-05	2,57E-05	5,39E-05	8,82E-05	1,72E-05	1,13E-05	2,57E-05
NA	NA	NA	1	0,000427	2342	7,00E-04	6,00E-04	7,00E-04	6,00E-04	1,00E-03	5,00E-04	5,00E-04	8,00E-04
NA	NA	NA	0	0	0	0	0	0	0	0	0	0	0
NA	NA	NA	0	0	0	1,00E-04	1,00E-04	1,00E-04	9,42E-05	2,00E-04	7,58E-05	6,28E-05	7,01E-05
NA	NA	NA	0	0	0	1,97E-05	1,97E-05	1,29E-05	2,69E-05	6,55E-05	5,24E-06	2,45E-06	1,83E-06
NA	NA	NA	1	0,000427	2342	5,26E-05	5,25E-05	2,57E-05	8,07E-05	3,00E-04	2,56E-05	1,83E-05	5,28E-05
NA	NA	NA	4	0,001708	2342	5,26E-05	5,25E-05	7,71E-05	2,69E-05	2,00E-04	2,56E-05	1,83E-05	2,84E-05
NA	NA	NA	0	0	0	0	0	0	0	0	0	0	0
NA	NA	NA	1	0,000427	2342	1,31E-05	1,31E-05	2,57E-05	0	2,94E-05	2,18E-06	8,20E-07	1,83E-06
NA	NA	NA	0	0	0	1,32E-05	1,31E-05	1,29E-05	1,35E-05	2,41E-05	2,18E-06	8,20E-07	.
NA	NA	NA	0	0	0	8,54E-05	8,53E-05	9,00E-05	8,06E-05	2,00E-04	4,95E-05	3,96E-05	7,01E-05
NA	NA	NA	0	0	0	0	0	0	0	0	0	0	0
NA	NA	NA	0	0	0	0	0	0	0	0	0	0	0
NA	NA	NA	5	0,002135	2342	0,0018	0,0019	0,0017	0,002	6,60E-03	0,0017	0,0016	5,70E-03
NA	NA	NA	0	0	0	0	0	0	0	0	0	0	0
NA	NA	NA	1	0,000427	2342	3,00E-04	3,00E-04	3,00E-04	3,00E-04	6,00E-04	2,00E-04	2,00E-04	4,00E-04
NA	NA	NA	1	0,000427	2342	3,00E-04	3,00E-04	3,00E-04	3,00E-04	6,00E-04	2,00E-04	2,00E-04	4,00E-04
NA	NA	NA	0	0	0	6,57E-06	6,57E-06	1,29E-05	0	2,41E-05	0	0	.
NA	NA	NA	0	0	0	0	0	0	0	0	0	0	0
NA	NA	NA	1	0,000427	2342	6,57E-06	6,57E-06	1,29E-05	0	6,55E-05	0	0	.
NA	NA	NA	0	0	0	0	0	0	0	0	0	0	0
NA	NA	NA	0	0	0	0	0	0	0	0	0	0	0
NA	NA	NA	0	0	0	0	0	0	0	0	0	0	0
NA	NA	NA	0	0	0	0	0	0	0	0	0	0	0
NA	NA	NA	0	0	0	0	0	0	0	0	0	0	0
NA	NA	NA	0	0	0	0	0	0	0	0	0	0	0
NA	NA	NA	1	0,000427	2342	0	0	0	0	0	0	0	0
NA	NA	NA	0	0	0	3,28E-05	3,94E-05	1,29E-05	5,37E-05	6,00E-04	1,26E-05	7,97E-06	8,99E-05
Rare Single	3	YES	1	0,000427	2342	0	0	0	0	0	0	0	0
NA	NA	NA	0	0	0	0	0	0	0	0	0	0	0
NA	NA	NA	0	0	0	2,63E-05	2,63E-05	2,57E-05	2,69E-05	7,24E-05	8,14E-06	5,14E-06	1,03E-05

NA	NA	NA	0	0	0	6,57E-06	6,57E-06	0	1,34E-05	1,47E-05	0	0	.
NA	NA	NA	0	0	0	0,0015	0,0015	0,0016	0,0014	5,40E-03	0,0013	0,0013	4,60E-03
Rare Single	2	NO	0	0	0	0	0	0	0	0	0	0	0
NA	NA	NA	0	0	0	6,57E-06	6,57E-06	1,28E-05	0	1,47E-05	0	0	.
NA	NA	NA	1	0,000427	2342	6,57E-06	6,57E-06	1,29E-05	0	2,41E-05	0	0	.
NA	NA	NA	0	0	0	6,57E-06	6,56E-06	0	1,34E-05	1,47E-05	0	0	.
NA	NA	NA	0	0	0	4,00E-04	4,00E-04	3,00E-04	4,00E-04	4,00E-04	3,00E-04	3,00E-04	2,00E-04
NA	NA	NA	0	0	0	4,60E-05	4,59E-05	5,14E-05	4,03E-05	9,63E-05	2,11E-05	1,53E-05	1,91E-05
NA	NA	NA	0	0	0	7,92E-05	7,88E-05	1,00E-04	4,06E-05	2,00E-04	4,52E-05	3,53E-05	2,84E-05
NA	NA	NA	2	0,000854	2342	9,00E-04	9,00E-04	9,00E-04	9,00E-04	1,60E-03	8,00E-04	7,00E-04	1,20E-03
NA	NA	NA	0	0	0	2,00E-04	2,00E-04	2,00E-04	1,00E-04	3,00E-04	1,00E-04	9,70E-05	2,00E-04
NA	NA	NA	0	0	0	6,57E-06	6,57E-06	1,29E-05	0	1,47E-05	0	0	.
NA	NA	NA	1	0,000427	2342	2,00E-04	2,00E-04	2,00E-04	2,00E-04	1,20E-03	1,00E-04	1,00E-04	6,00E-04
NA	NA	NA	0	0	0	1,31E-05	1,31E-05	1,29E-05	1,35E-05	1,00E-04	2,18E-06	8,20E-07	9,07E-06
NA	NA	NA	0	0	0	2,63E-05	2,63E-05	2,57E-05	2,69E-05	2,00E-04	8,14E-06	5,14E-06	2,99E-06
NA	NA	NA	0	0	0	6,57E-06	6,57E-06	1,29E-05	0	1,47E-05	0	0	.
NA	NA	NA	0	0	0	0	0	0	0	0	0	0	0
NA	NA	NA	0	0	0	0	0	0	0	0	0	0	0
NA	NA	NA	0	0	0	0	0	0	0	0	0	0	0
NA	NA	NA	0	0	0	0	0	0	0	0	0	0	0
NA	NA	NA	0	0	0	0	0	0	0	0	0	0	0
NA	NA	NA	0	0	0	0	0	0	0	0	0	0	0
NA	NA	NA	0	0	0	0	0	0	0	0	0	0	0
NA	NA	NA	0	0	0	0	0	0	0	0	0	0	0
NA	NA	NA	6	0,002564	2340	0,0024	0,0024	0,0026	0,0022	8,20E-03	0,0022	0,0021	7,20E-03
NA	NA	NA	6	0,002562	2342	0,0024	0,0024	0,0025	0,0022	8,20E-03	0,0022	0,0021	7,20E-03
NA	NA	NA	2	0,000854	2342	4,00E-04	4,00E-04	4,00E-04	4,00E-04	1,10E-03	3,00E-04	3,00E-04	8,00E-04
NA	NA	NA	0	0	0	5,91E-05	5,91E-05	6,42E-05	5,38E-05	5,00E-04	3,08E-05	2,21E-05	2,00E-04
NA	NA	NA	0	0	0	6,57E-06	6,57E-06	0	1,35E-05	2,00E-04	0	0	.
NA	NA	NA	0	0	0	0	0	0	0	0	0	0	0
NA	NA	NA	0	0	0	0	0	0	0	0	0	0	0
NA	NA	NA	0	0	0	3,94E-05	3,94E-05	2,57E-05	5,37E-05	4,00E-04	1,71E-05	1,13E-05	1,00E-04
NA	NA	NA	0	0	0	7,88E-05	7,87E-05	1,00E-04	4,03E-05	4,00E-04	4,49E-05	3,51E-05	2,85E-05
NA	NA	NA	0	0	0	0	0	0	0	0	0	0	0
NA	NA	NA	0	0	0	0	0	0	0	0	0	0	0
NA	NA	NA	0	0	0	0	0	0	0	0	0	0	0

NA	NA	NA	1	0,000427	2342	8,00E-04	8,00E-04	9,00E-04	8,00E-04	5,00E-04	7,00E-04	7,00E-04	3,00E-04
Rare Single	1	NO	0	0	0	0	0	0	0	0	0	0	0
NA	NA	NA	1	0,000427	2342	3,94E-05	3,94E-05	5,14E-05	2,69E-05	1,00E-04	1,72E-05	1,13E-05	3,05E-05
NA	NA	NA	0	0	0	0	0	0	0	0	0	0	0
NA	NA	NA	0	0	0	2,63E-05	2,62E-05	3,85E-05	1,34E-05	2,00E-04	8,13E-06	5,14E-06	1,03E-05
NA	NA	NA	0	0	0	2,63E-05	2,63E-05	2,57E-05	2,69E-05	2,00E-04	8,14E-06	5,14E-06	2,84E-05
NA	NA	NA	0	0	0	1,00E-04	1,00E-04	7,71E-05	1,00E-04	1,00E-03	6,51E-05	5,32E-05	3,00E-04
NA	NA	NA	0	0	0	0	0	0	0	0	0	0	0
NA	NA	NA	0	0	0	0	0	0	0	0	0	0	0
NA	NA	NA	0	0	0	6,57E-06	6,56E-06	1,29E-05	0	1,47E-05	0	0	.
NA	NA	NA	0	0	0	0	0	0	0	0	0	0	0
NA	NA	NA	1	0,000427	2342	6,57E-05	6,57E-05	8,99E-05	4,04E-05	2,00E-04	3,52E-05	2,62E-05	9,94E-05
NA	NA	NA	1	0,000427	2342	2,00E-04	2,00E-04	2,00E-04	1,00E-04	6,00E-04	1,00E-04	1,00E-04	4,00E-04
NA	NA	NA	0	0	0	0	0	0	0	0	0	0	0
NA	NA	NA	1	0,000427	2342	1,32E-05	1,31E-05	1,29E-05	1,35E-05	2,42E-05	2,19E-06	8,20E-07	.
NA	NA	NA	1	0,000427	2342	0	0	0	0	0	0	0	0
NA	NA	NA	0	0	0	0	0	0	0	0	0	0	0
NA	NA	NA	1	0,000427	2342	1,31E-05	1,31E-05	1,28E-05	1,35E-05	1,00E-04	2,18E-06	8,20E-07	9,07E-06
NA	NA	NA	0	0	0	2,63E-05	2,63E-05	1,29E-05	4,03E-05	1,00E-04	8,14E-06	5,14E-06	9,07E-06
NA	NA	NA	0	0	0	2,00E-04	2,00E-04	2,00E-04	1,00E-04	4,00E-04	1,00E-04	1,00E-04	2,00E-04
NA	NA	NA	0	0	0	0	0	0	0	0	0	0	0
NA	NA	NA	3	0,001281	2342	0,002	0,002	0,0021	0,002	4,30E-03	0,0019	0,0018	3,00E-03
NA	NA	NA	0	0	0	2,63E-05	2,63E-05	3,85E-05	1,35E-05	4,41E-05	8,14E-06	5,14E-06	6,25E-06
NA	NA	NA	0	0	0	1,32E-05	1,31E-05	1,29E-05	1,35E-05	2,42E-05	2,19E-06	8,20E-07	.
NA	NA	NA	0	0	0	1,31E-05	1,31E-05	1,29E-05	1,34E-05	6,55E-05	2,18E-06	8,20E-07	.
NA	NA	NA	0	0	0	0	0	0	0	0	0	0	0
NA	NA	NA	0	0	0	1,97E-05	1,97E-05	1,29E-05	2,69E-05	6,53E-05	5,24E-06	2,45E-06	1,83E-06
NA	NA	NA	1	0,000427	2343	1,00E-04	1,00E-04	9,00E-05	2,00E-04	4,00E-04	9,74E-05	8,26E-05	2,00E-04
Rare Single	3	NO	0	0	0	0	0	0	0	0	0	0	0
Rare Single	3	NO	1	0,000427	2342	1,32E-05	1,31E-05	0	2,69E-05	2,42E-05	2,19E-06	8,20E-07	.
Rare Single	3	NO	0	0	0	0	0	0	0	0	0	0	0
Rare Single	3	NO	0	0	0	1,31E-05	1,31E-05	1,29E-05	1,35E-05	1,47E-05	2,18E-06	8,20E-07	.
Rare Single	3	NO	0	0	0	1,97E-05	1,97E-05	2,57E-05	1,35E-05	6,55E-05	5,24E-06	2,45E-06	1,83E-06
Rare Single	3	NO	0	0	0	0	0	0	0	0	0	0	0
Rare Single	3	NO	2	0,000854	2342	6,56E-06	6,56E-06	0	1,34E-05	6,54E-05	0	0	.
Rare Single	1	NO	0	0	0	0	6,58E-06	0	0	.	0	0	.

NA	NA	NA	0	0	0	2,63E-05	2,63E-05	2,57E-05	2,69E-05	9,66E-05	8,15E-06	5,15E-06	1,92E-05
NA	NA	NA	0	0	0	0	0	0	0	0	0	0	0
Rare Single	1	NO	0	0	0	0	0	0	0	0	0	0	0
Rare Single	1	NO	2	0,000854	2342	1,97E-05	1,97E-05	2,57E-05	1,35E-05	2,94E-05	5,25E-06	2,46E-06	1,83E-06
NA	NA	NA	0	0	0	0	0	0	0	0	0	0	0
NA	NA	NA	2	0,000854	2342	5,00E-04	5,00E-04	6,00E-04	4,00E-04	1,20E-03	4,00E-04	4,00E-04	7,00E-04
NA	NA	NA	1	0,000427	2342	6,58E-06	6,57E-06	1,29E-05	0	2,42E-05	0	0	.
NA	NA	NA	0	0	0	0	0	0	0	0	0	0	0
NA	NA	NA	0	0	0	0	0	0	0	0	0	0	0
NA	NA	NA	0	0	0	0	0	0	0	0	0	0	0
NA	NA	NA	0	0	0	0	0	0	0	0	0	0	0
NA	NA	NA	0	0	0	0	0	0	0	0	0	0	0
NA	NA	NA	0	0	0	2,00E-04	2,00E-04	1,00E-04	3,00E-04	2,00E-03	1,00E-04	1,00E-04	1,20E-03
NA	NA	NA	0	0	0	0	0	0	0	0	0	0	0
NA	NA	NA	0	0	0	0	0	0	0	0	0	0	0
NA	NA	NA	0	0	0	0	0	0	0	0	0	0	0
NA	NA	NA	0	0	0	5,00E-04	5,00E-04	4,00E-04	5,00E-04	1,20E-03	4,00E-04	3,00E-04	9,00E-04
NA	NA	NA	0	0	0	1,31E-05	1,31E-05	0	2,69E-05	4,81E-05	2,18E-06	8,20E-07	2,98E-06
NA	NA	NA	0	0	0	0	0	0	0	0	0	0	0
NA	NA	NA	0	0	0	0	0	0	0	0	0	0	0
NA	NA	NA	0	0	0	0	0	0	0	0	0	0	0
NA	NA	NA	0	0	0	6,92E-05	6,62E-05	8,08E-05	5,69E-05	2,00E-04	3,69E-05	2,84E-05	8,67E-05
NA	NA	NA	0	0	0	6,90E-05	6,63E-05	8,06E-05	5,67E-05	2,00E-04	3,68E-05	2,84E-05	8,66E-05
Rare Single	2	NO	0	0	0	3,95E-05	3,94E-05	5,14E-05	2,69E-05	8,83E-05	1,72E-05	1,13E-05	2,58E-05
Rare Single	2	NO	0	0	0	0	0	0	0	0	0	0	0
NA	NA	NA	2	0,000854	2342	1,00E-04	1,00E-04	1,00E-04	1,00E-04	4,00E-04	7,58E-05	6,29E-05	2,00E-04
Rare Single	1	NO	0	0	0	3,00E-04	3,00E-04	4,00E-04	3,00E-04	5,00E-04	3,00E-04	2,00E-04	3,00E-04
NA	NA	NA	0	0	0	6,57E-06	6,57E-06	1,28E-05	0	1,47E-05	0	0	.
NA	NA	NA	1	0,000427	2342	0,0022	0,0022	0,0021	0,0022	7,60E-03	0,002	0,0019	6,70E-03
NA	NA	NA	0	0	0	0	0	0	0	0	0	0	0
NA	NA	NA	2	0,000854	2342	6,57E-05	6,56E-05	7,71E-05	5,37E-05	1,00E-04	3,51E-05	2,61E-05	4,24E-05
NA	NA	NA	0	0	0	0	0	0	0	0	0	0	0
NA	NA	NA	4	0,001708	2342	8,00E-04	8,00E-04	8,00E-04	8,00E-04	4,60E-03	7,00E-04	7,00E-04	2,70E-03
NA	NA	NA	0	0	0	6,57E-06	6,57E-06	0	1,35E-05	1,47E-05	0	0	.
NA	NA	NA	0	0	0	0	0	0	0	0	0	0	0
NA	NA	NA	0	0	0	0,0012	0,0012	0,0012	0,0012	4,30E-03	0,001	0,001	3,60E-03

Rare Single	3	NO	0	0	0	0	0	0	0	0	0	0	0
NA	NA	NA	0	0	0	0	0	0	0	0	0	0	0
NA	NA	NA	0	0	0	0	1,57E-05	0	0	.	0	0	.
NA	NA	NA	0	0	0	1,97E-05	1,97E-05	1,29E-05	2,69E-05	4,41E-05	5,24E-06	2,45E-06	6,25E-06
NA	NA	NA	1	0,000427	2342	0,0017	0,0017	0,002	0,0015	6,20E-03	0,0016	0,0015	5,30E-03
NA	NA	NA	0	0	0	6,00E-04	6,00E-04	7,00E-04	4,00E-04	1,50E-03	5,00E-04	4,00E-04	1,10E-03
NA	NA	NA	0	0	0	1,31E-05	1,31E-05	2,57E-05	0	6,55E-05	2,18E-06	8,20E-07	.
NA	NA	NA	2	0,000854	2342	6,57E-06	6,57E-06	0	1,35E-05	6,54E-05	0	0	.
NA	NA	NA	13	0,005551	2342	0,0038	0,0038	0,0041	0,0035	6,70E-03	0,0035	0,0034	5,90E-03
NA	NA	NA	13	0,005551	2342	0,0039	0,0039	0,0041	0,0036	6,70E-03	0,0036	0,0035	5,90E-03
NA	NA	NA	0	0	0	0	0	0	0	0	0	0	0
NA	NA	NA	0	0	0	0	0	0	0	0	0	0	0
NA	NA	NA	1	0,000427	2342	6,66E-06	6,60E-06	1,30E-05	0	6,62E-05	0	0	.
NA	NA	NA	0	0	0	0	0	0	0	0	0	0	0
Rare Single	3	NO	0	0	0	2,67E-05	2,63E-05	0	5,47E-05	2,00E-04	8,25E-06	5,21E-06	2,85E-05
Rare Single	3	NO	0	0	0	0	0	0	0	0	0	0	0
NA	NA	NA	0	0	0	0	0	0	0	0	0	0	0
NA	NA	NA	1	0,000427	2342	1,00E-04	1,00E-04	2,00E-04	1,00E-04	4,00E-04	9,76E-05	8,27E-05	2,00E-04
Rare Single	NA	YES	0	0	0	4,61E-05	4,60E-05	6,44E-05	2,70E-05	4,00E-04	2,11E-05	1,53E-05	2,86E-05
NA	NA	NA	0	0	0	6,61E-06	6,59E-06	1,29E-05	0	2,44E-05	0	0	.
NA	NA	NA	0	0	0	5,26E-05	5,25E-05	6,42E-05	4,04E-05	7,24E-05	2,56E-05	1,83E-05	1,03E-05
NA	NA	NA	0	0	0	1,00E-04	1,00E-04	3,86E-05	2,00E-04	8,00E-04	7,58E-05	6,28E-05	2,00E-04
NA	NA	NA	0	0	0	0	0	0	0	0	0	0	0
NA	NA	NA	0	0	0	6,57E-06	6,57E-06	1,29E-05	0	1,47E-05	0	0	.
NA	NA	NA	0	0	0	2,63E-05	2,63E-05	2,57E-05	2,69E-05	4,41E-05	8,14E-06	5,14E-06	6,25E-06
NA	NA	NA	0	0	0	0	0	0	0	0	0	0	0
NA	NA	NA	1	0,000427	2342	5,26E-05	5,25E-05	7,71E-05	2,69E-05	8,82E-05	2,56E-05	1,83E-05	2,57E-05
Rare Single	1	NO	0	0	0	2,63E-05	2,63E-05	2,57E-05	2,69E-05	6,54E-05	8,14E-06	5,14E-06	6,25E-06
Genetic As	3	NO	6	0,002562	2342	5,00E-04	5,00E-04	5,00E-04	6,00E-04	1,90E-03	4,00E-04	4,00E-04	1,40E-03
NA	NA	NA	0	0	0	2,63E-05	2,63E-05	2,57E-05	2,69E-05	7,22E-05	8,14E-06	5,14E-06	1,03E-05
NA	NA	NA	1	0,000427	2342	4,00E-04	4,00E-04	4,00E-04	4,00E-04	5,00E-04	3,00E-04	3,00E-04	4,00E-04
NA	NA	NA	1	0,000427	2342	8,00E-04	8,00E-04	8,00E-04	8,00E-04	3,00E-03	7,00E-04	7,00E-04	2,40E-03
NA	NA	NA	0	0	0	0	0	0	0	0	0	0	0
Rare Single	3	NO	1	0,000427	2342	1,32E-05	1,31E-05	0	2,69E-05	6,56E-05	2,19E-06	8,20E-07	.
NA	NA	NA	0	0	0	0	0	0	0	0	0	0	0
NA	NA	NA	1	0,000427	2342	2,00E-04	2,00E-04	2,00E-04	2,00E-04	5,00E-04	1,00E-04	1,00E-04	3,00E-04

NA	NA	NA	0	0	0	0	0	0	0	0	0	0	0
NA	NA	NA	1	0,000427	2342	4,60E-05	5,26E-05	5,14E-05	4,04E-05	7,35E-05	2,11E-05	1,53E-05	1,86E-05
NA	NA	NA	0	0	0	1,97E-05	1,97E-05	1,29E-05	2,69E-05	2,94E-05	5,24E-06	2,45E-06	1,83E-06
NA	NA	NA	4	0,001708	2342	7,00E-04	7,00E-04	0,001	5,00E-04	1,30E-03	6,00E-04	6,00E-04	1,00E-03
Rare Single	3	NO	0	0	0	2,00E-04	2,00E-04	2,00E-04	2,00E-04	1,40E-03	1,00E-04	9,96E-05	5,00E-04
Rare Single	3	NO	0	0	0	2,00E-04	2,00E-04	2,00E-04	2,00E-04	1,40E-03	1,00E-04	1,00E-04	5,00E-04
NA	NA	NA	0	0	0	8,54E-05	8,53E-05	5,14E-05	1,00E-04	2,00E-04	4,96E-05	3,96E-05	5,09E-05
Rare Single	3	YES	0	0	0	2,63E-05	2,62E-05	1,29E-05	4,03E-05	6,00E-04	8,14E-06	5,14E-06	8,39E-05
Rare Single	NA	YES	0	0	0	0	0	0	0	0	0	0	0
NA	NA	NA	0	0	0	9,85E-05	1,00E-04	1,00E-04	9,40E-05	1,00E-04	6,00E-05	4,88E-05	5,09E-05
NA	NA	NA	2	0,000854	2342	8,25E-05	8,10E-05	1,00E-04	4,24E-05	2,00E-04	4,69E-05	3,67E-05	7,22E-05
Rare Single	2	NO	0	0	0	0	0	0	0	0	0	0	0
Rare Single	1	NO	0	0	0	0	0	0	0	0	0	0	0
Rare Single	1	NO	0	0	0	0	0	0	0	0	0	0	0
Rare Single	1	NO	0	0	0	7,02E-06	6,64E-06	1,36E-05	0	.	0	0	.
NA	NA	NA	0	0	0	0	0	0	0	0	0	0	0
NA	NA	NA	2	0,000854	2342	0	0	0	0	0	0	0	0
Rare Single	1	NO	0	0	0	7,24E-05	7,23E-05	9,00E-05	5,39E-05	2,00E-04	3,98E-05	3,13E-05	7,02E-05
NA	NA	NA	0	0	0	0	0	0	0	0	0	0	0
Rare Single	3	NO	1	0,000427	2342	0,0035	0,0035	0,0029	0,0042	4,10E-03	0,0033	0,0032	3,60E-03
NA	NA	NA	2	0,000854	2342	6,00E-04	6,00E-04	7,00E-04	5,00E-04	1,00E-03	5,00E-04	5,00E-04	7,00E-04
NA	NA	NA	0	0	0	1,97E-05	1,97E-05	1,28E-05	2,69E-05	7,24E-05	5,24E-06	2,45E-06	1,03E-05
NA	NA	NA	1	0,000427	2342	0	0	0	0	0	0	0	0
NA	NA	NA	0	0	0	9,86E-05	9,85E-05	1,00E-04	8,07E-05	2,00E-04	6,01E-05	4,88E-05	7,89E-05
NA	NA	NA	0	0	0	0	0	0	0	0	0	0	0
Rare Single	2	NO	0	0	0	0	0	0	0	0	0	0	0
Rare Single	2	NO	0	0	0	0	0	0	0	0	0	0	0
Rare Single	2	NO	1	0,000427	2342	1,00E-04	1,00E-04	1,00E-04	1,00E-04	2,10E-03	7,09E-05	5,75E-05	9,00E-04
NA	NA	NA	1	0,000427	2342	2,00E-04	2,00E-04	3,00E-04	6,73E-05	5,00E-04	1,00E-04	1,00E-04	3,00E-04
NA	NA	NA	0	0	0	0	0	0	0	0	0	0	0
NA	NA	NA	0	0	0	0	0	0	0	0	0	0	0
NA	NA	NA	0	0	0	0	0	0	0	0	0	0	0
NA	NA	NA	0	0	0	3,29E-05	3,28E-05	3,86E-05	2,69E-05	5,88E-05	1,26E-05	7,98E-06	1,12E-05
NA	NA	NA	2	0,000854	2342	3,00E-04	3,00E-04	3,00E-04	3,00E-04	1,10E-03	2,00E-04	2,00E-04	8,00E-04
NA	NA	NA	1	0,000427	2342	2,63E-05	2,63E-05	2,57E-05	2,69E-05	4,41E-05	8,15E-06	5,14E-06	6,25E-06
NA	NA	NA	0	0	0	3,29E-05	3,28E-05	3,85E-05	2,69E-05	2,00E-04	1,26E-05	7,98E-06	1,03E-05

NA	NA	NA	0	0	0	0	0	0	0	0	0	0	0
NA	NA	NA	0	0	0	0	0	0	0	0	0	0	0
NA	NA	NA	0	0	0	6,57E-06	6,57E-06	0	1,35E-05	6,55E-05	0	0	.
NA	NA	NA	1	0,000427	2342	6,00E-04	6,00E-04	4,00E-04	9,00E-04	1,10E-03	5,00E-04	5,00E-04	6,00E-04
NA	NA	NA	0	0	0	0	0	0	0	0	0	0	0
NA	NA	NA	0	0	0	5,00E-04	5,00E-04	5,00E-04	4,00E-04	1,70E-03	4,00E-04	4,00E-04	1,30E-03
Rare Single	3	NO	0	0	0	0	0	0	0	0	0	0	0
NA	NA	NA	0	0	0	6,57E-06	6,57E-06	0	1,35E-05	1,47E-05	0	0	.
NA	NA	NA	0	0	0	0	0	0	0	0	0	0	0
NA	NA	NA	0	0	0	0	0	0	0	0	0	0	0
NA	NA	NA	0	0	0	0	0	0	0	0	0	0	0
NA	NA	NA	0	0	0	0	0	0	0	0	0	0	0
NA	NA	NA	0	0	0	0	0	0	0	0	0	0	0
NA	NA	NA	0	0	0	0	0	0	0	0	0	0	0
NA	NA	NA	0	0	0	3,00E-04	3,00E-04	3,00E-04	3,00E-04	5,00E-04	2,00E-04	2,00E-04	3,00E-04
NA	NA	NA	0	0	0	0	0	0	0	0	0	0	0
NA	NA	NA	0	0	0	1,31E-05	1,31E-05	1,28E-05	1,35E-05	1,47E-05	2,18E-06	8,20E-07	.
NA	NA	NA	0	0	0	0	0	0	0	0	0	0	0
Rare Single	3	NO	2	0,000854	2342	3,29E-05	3,28E-05	2,57E-05	4,04E-05	5,88E-05	1,26E-05	7,98E-06	1,12E-05
Genetic As	2	NO	1	0,000427	2342	0	0	0	0	0	0	0	0
Genetic As	2	NO	0	0	0	0	0	0	0	0	0	0	0
Genetic As	2	NO	0	0	0	0	0	0	0	0	0	0	0
NA	NA	NA	0	0	0	1,32E-05	1,31E-05	2,57E-05	0	2,94E-05	2,19E-06	8,20E-07	1,83E-06
NA	NA	NA	0	0	0	9,85E-05	9,84E-05	1,00E-04	9,40E-05	2,90E-03	6,00E-05	4,88E-05	1,40E-03
NA	NA	NA	0	0	0	2,00E-04	2,00E-04	2,00E-04	2,00E-04	2,00E-04	1,00E-04	1,00E-04	8,88E-05
NA	NA	NA	0	0	0	6,57E-06	6,57E-06	1,28E-05	0	1,47E-05	0	0	.
Rare Single	2	YES	0	0	0	0	0	0	0	0	0	0	0
NA	NA	NA	0	0	0	0	0	0	0	0	0	0	0
Rare Single	3	NO	0	0	0	7,23E-05	7,22E-05	5,14E-05	9,41E-05	2,00E-04	3,97E-05	3,13E-05	9,92E-05
NA	NA	NA	2	0,000854	2342	0	0	0	0	0	0	0	0
NA	NA	NA	0	0	0	0	0	0	0	0	0	0	0
NA	NA	NA	0	0	0	6,57E-06	6,57E-06	1,29E-05	0	1,47E-05	0	0	.
NA	NA	NA	0	0	0	0	0	0	0	0	0	0	0
NA	NA	NA	0	0	0	0	0	0	0	0	0	0	0
NA	NA	NA	0	0	0	0	0	0	0	0	0	0	0
NA	NA	NA	0	0	0	0	0	0	0	0	0	0	0

Rare Single		3	NO	0	0	0	0	0	0	0	0	0	0	0
NA	NA		NA	0	0	0	1,00E-04	1,00E-04	1,00E-04	1,00E-04	2,00E-04	8,17E-05	6,73E-05	6,25E-06
NA	NA		NA	0	0	0	0	0	0	0	0	0	0	0
NA	NA		NA	0	0	0	3,94E-05	3,94E-05	2,57E-05	5,38E-05	2,00E-04	1,72E-05	1,13E-05	2,84E-05
NA	NA		NA	0	0	0	6,00E-04	6,00E-04	6,00E-04	5,00E-04	2,10E-03	5,00E-04	4,00E-04	1,60E-03
NA	NA		NA	1	0,000427	2342	6,58E-06	6,57E-06	1,29E-05	0	6,55E-05	0	0	.
NA	NA		NA	16	0,006832	2342	0,0093	0,0094	0,0099	0,0087	3,12E-02	0,0089	0,0088	2,92E-02
Rare Single		3	YES	1	0,000427	2342	5,25E-05	5,25E-05	1,29E-05	9,40E-05	2,00E-04	2,56E-05	1,83E-05	6,94E-05
Rare Single		3	YES	2	0,000854	2342	3,00E-04	3,00E-04	4,00E-04	3,00E-04	1,00E-03	2,00E-04	2,00E-04	3,00E-04
NA	NA		NA	0	0	0	0	0	0	0	0	0	0	0
Rare Single		3	NO	0	0	0	0	0	0	0	0	0	0	0
Rare Single		3	NO	0	0	0	0	0	0	0	0	0	0	0
NA	NA		NA	0	0	0	0	0	0	0	0	0	0	0
NA	NA		NA	0	0	0	0	0	0	0	0	0	0	0
NA	NA		NA	0	0	0	1,00E-04	1,00E-04	1,00E-04	9,40E-05	2,00E-04	7,08E-05	5,74E-05	7,01E-05
NA	NA		NA	0	0	0	6,57E-06	6,57E-06	0	1,35E-05	1,47E-05	0	0	.
Rare Single		1	NO	0	0	0	0	0	0	0	0	0	0	0
Rare Single		1	NO	0	0	0	0	0	0	0	0	0	0	0
NA	NA		NA	0	0	0	0	0	0	0	0	0	0	0
NA	NA		NA	2	0,000854	2342	6,00E-04	6,00E-04	5,00E-04	6,00E-04	1,80E-03	5,00E-04	4,00E-04	1,10E-03
NA	NA		NA	0	0	0	0	0	0	0	0	0	0	0
NA	NA		NA	0	0	0	0	0	0	0	0	0	0	0
NA	NA		NA	0	0	0	8,53E-05	8,53E-05	7,71E-05	9,40E-05	2,00E-04	4,95E-05	3,96E-05	4,24E-05
NA	NA		NA	0	0	0	1,00E-04	1,00E-04	1,00E-04	1,00E-04	4,00E-03	9,14E-05	7,70E-05	2,30E-03
NA	NA		NA	0	0	0	0	0	0	0	0	0	0	0
NA	NA		NA	3	0,001281	2342	3,00E-04	3,00E-04	2,00E-04	3,00E-04	2,50E-03	2,00E-04	2,00E-04	1,10E-03
NA	NA		NA	0	0	0	2,63E-05	2,63E-05	1,29E-05	4,04E-05	2,00E-04	8,15E-06	5,15E-06	2,84E-05
NA	NA		NA	0	0	0	6,58E-06	6,57E-06	0	1,35E-05	1,47E-05	0	0	.
NA	NA		NA	0	0	0	0	0	0	0	0	0	0	0
NA	NA		NA	0	0	0	5,00E-04	5,00E-04	6,00E-04	4,00E-04	9,00E-04	4,00E-04	4,00E-04	7,00E-04
NA	NA		NA	0	0	0	5,91E-05	5,91E-05	5,14E-05	6,73E-05	6,00E-04	3,08E-05	2,21E-05	8,98E-05
NA	NA		NA	0	0	0	0	0	0	0	0	0	0	0
NA	NA		NA	0	0	0	0	0	0	0	0	0	0	0
NA	NA		NA	0	0	0	1,00E-04	1,00E-04	1,00E-04	9,41E-05	1,00E-03	7,58E-05	6,28E-05	5,00E-04
NA	NA		NA	0	0	0	0	0	0	0	0	0	0	0
NA	NA		NA	0	0	0	0	0	0	0	0	0	0	0

Rare Single		1	NO	0	0	0	0	0	0	0	0	0	0	0
NA	NA		NA	0	0	0	0	0	0	0	0	0	0	0
NA	NA		NA	0	0	0	6,57E-06	6,57E-06	1,29E-05	0	6,54E-05	0	0	.
Rare Single		3	NO	1	0,000427	2342	1,00E-04	1,00E-04	7,71E-05	2,00E-04	1,20E-03	8,16E-05	6,72E-05	4,00E-04
Rare Single		3	NO	0	0	0	2,63E-05	2,63E-05	1,29E-05	4,03E-05	8,00E-04	8,14E-06	5,14E-06	2,00E-04
Rare Single		3	NO	0	0	0	7,23E-05	7,22E-05	9,01E-05	5,38E-05	4,00E-04	3,97E-05	3,13E-05	1,00E-04
NA	NA		NA	2	0,000854	2342	0	0	0	0	0	0	0	0
Rare Single		1	NO	0	0	0	0	0	0	0	0	0	0	0
NA	NA		NA	1	0,000427	2342	2,63E-05	2,63E-05	2,57E-05	2,69E-05	6,54E-05	8,14E-06	5,14E-06	1,83E-06
NA	NA		NA	0	0	0	0	0	0	0	0	0	0	0
NA	NA		NA	0	0	0	0	0	0	0	0	0	0	0
NA	NA		NA	0	0	0	6,57E-06	6,57E-06	1,29E-05	0	2,42E-05	0	0	.
NA	NA		NA	0	0	0	9,19E-05	9,19E-05	1,00E-04	5,37E-05	3,00E-04	5,53E-05	4,36E-05	2,00E-04
NA	NA		NA	0	0	0	1,31E-05	1,31E-05	1,28E-05	1,34E-05	2,94E-05	2,18E-06	8,20E-07	1,83E-06
NA	NA		NA	0	0	0	6,57E-06	6,56E-06	1,29E-05	0	2,00E-04	0	0	.
NA	NA		NA	1	0,000427	2342	9,85E-05	9,84E-05	1,00E-04	6,71E-05	2,00E-04	6,00E-05	4,88E-05	1,13E-05
NA	NA		NA	0	0	0	0	0	0	0	0	0	0	0
Rare Single		1	NO	0	0	0	0	0	0	0	0	0	0	0
Rare Single		1	NO	0	0	0	1,00E-04	1,00E-04	8,40E-05	2,00E-04	7,00E-04	3,15E-05	1,67E-05	.
NA	NA		NA	2	0,000854	2342	4,00E-04	4,00E-04	5,00E-04	4,00E-04	1,50E-03	4,00E-04	3,00E-04	1,10E-03
NA	NA		NA	0	0	0	0	0	0	0	0	0	0	0
NA	NA		NA	1	0,000427	2342	2,00E-04	2,00E-04	2,00E-04	2,00E-04	6,00E-04	1,00E-04	1,00E-04	4,00E-04
NA	NA		NA	0	0	0	0	0	0	0	0	0	0	0
NA	NA		NA	0	0	0	3,94E-05	3,94E-05	3,86E-05	4,03E-05	6,00E-04	1,72E-05	1,13E-05	8,99E-05
NA	NA		NA	0	0	0	0	0	0	0	0	0	0	0
NA	NA		NA	0	0	0	9,20E-05	9,19E-05	1,00E-04	6,73E-05	2,00E-04	5,53E-05	4,37E-05	7,01E-05
NA	NA		NA	0	0	0	0	0	0	0	0	0	0	0
NA	NA		NA	0	0	0	6,57E-06	6,57E-06	1,28E-05	0	1,47E-05	0	0	.
NA	NA		NA	8	0,003416	2342	0,0023	0,0023	0,0024	0,0021	7,90E-03	0,0021	0,002	7,00E-03
NA	NA		NA	1	0,000427	2342	0,001	0,001	0,001	0,0011	3,30E-03	9,00E-04	8,00E-04	2,70E-03
NA	NA		NA	0	0	0	5,91E-05	5,91E-05	7,71E-05	4,04E-05	6,00E-04	3,08E-05	2,21E-05	2,00E-04
NA	NA		NA	0	0	0	0	0	0	0	0	0	0	0
NA	NA		NA	1	0,000427	2342	0	0	0	0	0	0	0	0
NA	NA		NA	1	0,000427	2342	6,57E-06	6,57E-06	1,29E-05	0	1,47E-05	0	0	.
NA	NA		NA	0	0	0	0	0	0	0	0	0	0	0
NA	NA		NA	0	0	0	1,97E-05	1,97E-05	3,85E-05	0	4,41E-05	5,24E-06	2,45E-06	6,25E-06

NA	NA	NA	0	0	0	8,54E-05	8,54E-05	7,71E-05	9,42E-05	3,00E-04	4,96E-05	3,96E-05	1,00E-04
NA	NA	NA	0	0	0	1,31E-05	1,31E-05	1,29E-05	1,34E-05	2,94E-05	2,18E-06	8,20E-07	1,83E-06
NA	NA	NA	0	0	0	8,54E-05	8,53E-05	6,43E-05	1,00E-04	3,00E-04	4,96E-05	3,96E-05	5,28E-05
Genetic As	2	NO	1	0,000427	2342	0,001	0,001	9,00E-04	0,0011	3,40E-03	8,00E-04	8,00E-04	2,70E-03
Genetic As	2	NO	9	0,003843	2342	0,0019	0,0019	0,0019	0,0019	6,40E-03	0,0017	0,0016	5,50E-03
NA	NA	NA	4	0,001708	2342	0,001	0,001	0,0011	0,001	3,80E-03	9,00E-04	8,00E-04	3,10E-03
NA	NA	NA	0	0	0	0	0	0	0	0	0	0	0
NA	NA	NA	0	0	0	0	0	0	0	0	0	0	0
NA	NA	NA	0	0	0	5,26E-05	5,25E-05	3,86E-05	6,73E-05	3,00E-04	2,56E-05	1,83E-05	5,39E-05
NA	NA	NA	0	0	0	0	0	0	0	0	0	0	0
NA	NA	NA	1	0,000427	2342	1,31E-05	1,31E-05	1,28E-05	1,35E-05	2,00E-04	2,18E-06	8,20E-07	.
NA	NA	NA	0	0	0	6,57E-06	6,57E-06	1,29E-05	0	2,41E-05	0	0	.
NA	NA	NA	1	0,000427	2342	2,00E-04	2,00E-04	2,00E-04	1,00E-04	5,00E-04	1,00E-04	1,00E-04	3,00E-04
NA	NA	NA	0	0	0	2,63E-05	2,63E-05	2,57E-05	2,69E-05	3,00E-04	8,14E-06	5,14E-06	5,28E-05
NA	NA	NA	2	0,000854	2342	1,00E-04	1,00E-04	1,00E-04	1,00E-04	4,00E-04	7,58E-05	6,28E-05	2,00E-04
NA	NA	NA	0	0	0	3,29E-05	3,28E-05	5,14E-05	1,35E-05	7,35E-05	1,26E-05	7,98E-06	1,86E-05
Syndromic	NA	YES	0	0	0	0	0	0	0	0	0	0	0
NA	NA	NA	3	0,001281	2342	4,00E-04	4,00E-04	5,00E-04	3,00E-04	1,40E-03	3,00E-04	3,00E-04	1,00E-03
Rare Single	1	NO	0	0	0	0	0	0	0	0	0	0	0
NA	NA	NA	0	0	0	3,29E-05	3,28E-05	3,85E-05	2,69E-05	7,35E-05	1,26E-05	7,98E-06	1,86E-05
NA	NA	NA	0	0	0	1,31E-05	1,31E-05	2,57E-05	0	4,83E-05	2,18E-06	8,20E-07	2,99E-06
NA	NA	NA	2	0,000854	2342	8,00E-04	9,00E-04	9,00E-04	8,00E-04	7,00E-04	7,00E-04	7,00E-04	3,00E-04
NA	NA	NA	0	0	0	0	0	0	0	0	0	0	0
NA	NA	NA	0	0	0	0	0	0	0	0	0	0	0
NA	NA	NA	0	0	0	6,57E-05	6,57E-05	7,71E-05	5,38E-05	2,00E-04	3,52E-05	2,62E-05	3,34E-05
NA	NA	NA	0	0	0	1,31E-05	1,31E-05	1,28E-05	1,34E-05	2,41E-05	2,18E-06	8,20E-07	.
NA	NA	NA	0	0	0	1,97E-05	1,97E-05	2,57E-05	1,35E-05	4,41E-05	5,25E-06	2,46E-06	6,25E-06
NA	NA	NA	0	0	0	4,60E-05	4,59E-05	2,57E-05	6,71E-05	6,00E-04	2,11E-05	1,53E-05	9,00E-05
NA	NA	NA	0	0	0	0	0	0	0	0	0	0	0
NA	NA	NA	1	0,000427	2342	2,00E-04	2,00E-04	1,00E-04	2,00E-04	3,70E-03	1,00E-04	8,71E-05	2,00E-03
NA	NA	NA	0	0	0	6,57E-06	6,57E-06	1,29E-05	0	2,42E-05	0	0	.
NA	NA	NA	1	0,000427	2342	6,57E-06	6,57E-06	1,29E-05	0	1,47E-05	0	0	.
NA	NA	NA	0	0	0	0	0	0	0	0	0	0	0
NA	NA	NA	0	0	0	0	0	0	0	0	0	0	0
NA	NA	NA	0	0	0	0	0	0	0	0	0	0	0
NA	NA	NA	0	0	0	6,57E-06	6,57E-06	1,28E-05	0	1,47E-05	0	0	.

Rare	Single	3	NO	0	0	0	0	0	0	0	0	0	0	0
Rare	Single	3	NO	3	0,001281	2342	5,00E-04	5,00E-04	4,00E-04	5,00E-04	8,00E-04	4,00E-04	3,00E-04	5,00E-04
Rare	Single	2	NO	0	0	0	0	0	0	0	0	0	0	0
NA	NA		NA	0	0	0	0	0	0	0	0	0	0	0
NA	NA		NA	0	0	0	1,97E-05	1,97E-05	1,28E-05	2,69E-05	2,94E-05	5,24E-06	2,45E-06	1,83E-06
NA	NA		NA	1	0,000427	2342	1,31E-05	1,31E-05	0	2,69E-05	2,00E-04	2,18E-06	8,20E-07	.
NA	NA		NA	0	0	0	1,32E-05	1,31E-05	2,57E-05	0	2,94E-05	2,18E-06	8,20E-07	1,83E-06
NA	NA		NA	0	0	0	1,31E-05	1,31E-05	1,28E-05	1,34E-05	1,47E-05	2,18E-06	8,20E-07	.
NA	NA		NA	0	0	0	5,27E-05	5,26E-05	1,29E-05	9,43E-05	1,00E-04	2,56E-05	1,83E-05	1,13E-05
NA	NA		NA	0	0	0	0	0	0	0	0	0	0	0
NA	NA		NA	1	0,000427	2342	6,57E-06	6,57E-06	0	1,35E-05	6,55E-05	0	0	.
NA	NA		NA	0	0	0	7,00E-04	7,00E-04	6,00E-04	8,00E-04	1,10E-03	6,00E-04	6,00E-04	8,00E-04
NA	NA		NA	0	0	0	1,00E-04	1,00E-04	9,00E-05	2,00E-04	5,00E-04	8,67E-05	7,26E-05	2,00E-04
NA	NA		NA	0	0	0	0	0	0	0	0	0	0	0
NA	NA		NA	0	0	0	1,00E-04	1,00E-04	2,00E-04	9,41E-05	1,00E-04	8,66E-05	7,26E-05	5,99E-05
NA	NA		NA	0	0	0	0	0	0	0	0	0	0	0
NA	NA		NA	2	0,000854	2342	3,28E-05	3,28E-05	6,42E-05	0	6,00E-04	1,26E-05	7,97E-06	8,37E-05
NA	NA		NA	0	0	0	3,29E-05	3,28E-05	5,14E-05	1,35E-05	1,00E-04	1,26E-05	7,98E-06	9,08E-06
NA	NA		NA	0	0	0	1,97E-05	1,97E-05	3,86E-05	0	1,47E-05	5,25E-06	2,46E-06	.
NA	NA		NA	0	0	0	0	0	0	0	0	0	0	0
Rare	Single	2	NO	0	0	0	1,97E-05	1,97E-05	1,29E-05	2,69E-05	7,24E-05	5,24E-06	2,45E-06	1,03E-05
NA	NA		NA	0	0	0	0,002	0,002	0,0021	0,0019	3,30E-03	0,0018	0,0017	2,80E-03
NA	NA		NA	1	0,000427	2342	6,58E-06	6,57E-06	1,29E-05	0	1,47E-05	0	0	.
NA	NA		NA	0	0	0	0	0	0	0	0	0	0	0
Rare	Single	1	NO	1	0,000427	2342	5,00E-04	5,00E-04	6,00E-04	4,00E-04	1,90E-03	4,00E-04	4,00E-04	1,50E-03
Rare	Single	3	NO	0	0	0	0	0	0	0	0	0	0	0
NA	NA		NA	0	0	0	6,58E-06	6,57E-06	1,29E-05	0	1,47E-05	0	0	.
NA	NA		NA	0	0	0	2,63E-05	2,63E-05	2,57E-05	2,69E-05	7,24E-05	8,15E-06	5,14E-06	1,03E-05
NA	NA		NA	0	0	0	1,32E-05	1,31E-05	2,57E-05	0	1,00E-04	2,18E-06	8,20E-07	9,07E-06
NA	NA		NA	0	0	0	0	0	0	0	0	0	0	0
NA	NA		NA	0	0	0	6,57E-06	6,57E-06	0	1,35E-05	6,55E-05	0	0	.
NA	NA		NA	0	0	0	1,97E-05	1,97E-05	3,86E-05	0	6,55E-05	5,25E-06	2,46E-06	1,83E-06
Rare	Single	2	YES	0	0	0	6,57E-06	6,57E-06	0	1,35E-05	6,55E-05	0	0	.
Rare	Single	2	YES	0	0	0	3,29E-05	3,28E-05	1,29E-05	5,38E-05	4,81E-05	1,26E-05	7,98E-06	6,25E-06
Rare	Single	2	YES	1	0,000427	2342	1,00E-04	1,00E-04	2,00E-04	1,00E-04	1,70E-03	9,14E-05	7,70E-05	7,00E-04
NA	NA		NA	1	0,000427	2342	2,00E-04	2,00E-04	3,00E-04	1,00E-04	8,00E-04	2,00E-04	1,00E-04	5,00E-04

NA	NA	NA	0	0	0	0	0	0	0	0	0	0	0
NA	NA	NA	0	0	0	0	0	0	0	0	0	0	0
NA	NA	NA	0	0	0	0	0	0	0	0	0	0	0
NA	NA	NA	0	0	0	6,61E-06	6,57E-06	1,29E-05	0	2,42E-05	0	0	.
Rare Single	1	NO	0	0	0	0	0	0	0	0	0	0	0
Rare Single	2	NO	1	0,000427	2342	4,60E-05	4,60E-05	3,85E-05	5,38E-05	1,00E-04	2,11E-05	1,53E-05	9,08E-06
NA	NA	NA	0	0	0	4,60E-05	4,60E-05	3,86E-05	5,38E-05	2,00E-04	2,11E-05	1,53E-05	2,84E-05
NA	NA	NA	0	0	0	1,31E-05	1,31E-05	1,29E-05	1,35E-05	1,00E-04	2,18E-06	8,20E-07	9,07E-06
NA	NA	NA	0	0	0	6,57E-06	6,57E-06	1,28E-05	0	2,00E-04	0	0	.
NA	NA	NA	0	0	0	1,97E-05	1,97E-05	2,57E-05	1,35E-05	4,41E-05	5,25E-06	2,46E-06	6,25E-06
NA	NA	NA	0	0	0	6,57E-06	6,57E-06	1,29E-05	0	6,55E-05	0	0	.
NA	NA	NA	1	0,000427	2342	0	0	0	0	0	0	0	0
NA	NA	NA	0	0	0	0	0	0	0	0	0	0	0
NA	NA	NA	0	0	0	0	0	0	0	0	0	0	0
NA	NA	NA	0	0	0	0	0	0	0	0	0	0	0
NA	NA	NA	0	0	0	0	0	0	0	0	0	0	0
NA	NA	NA	0	0	0	0	0	0	0	0	0	0	0
NA	NA	NA	0	0	0	6,57E-05	6,57E-05	5,14E-05	8,07E-05	2,00E-04	3,52E-05	2,62E-05	5,09E-05
NA	NA	NA	1	0,000427	2342	7,23E-05	7,22E-05	7,71E-05	6,72E-05	1,00E-04	3,97E-05	3,13E-05	4,24E-05
NA	NA	NA	0	0	0	3,94E-05	3,94E-05	5,14E-05	2,69E-05	2,00E-04	1,72E-05	1,13E-05	2,84E-05
NA	NA	NA	0	0	0	0	0	0	0	0	0	0	0
NA	NA	NA	0	0	0	0	0	0	0	0	0	0	0
NA	NA	NA	0	0	0	6,00E-04	6,00E-04	6,00E-04	6,00E-04	1,10E-03	5,00E-04	5,00E-04	8,00E-04
NA	NA	NA	0	0	0	1,31E-05	1,31E-05	2,57E-05	0	4,82E-05	2,18E-06	8,20E-07	2,99E-06
NA	NA	NA	0	0	0	0	0	0	0	0	0	0	0
Rare Single	3	YES	0	0	0	2,63E-05	2,63E-05	2,57E-05	2,69E-05	9,65E-05	8,14E-06	5,14E-06	1,91E-05
NA	NA	NA	0	0	0	0	0	0	0	0	0	0	0
NA	NA	NA	0	0	0	0	0	0	0	0	0	0	0
NA	NA	NA	0	0	0	1,97E-05	1,97E-05	2,57E-05	1,35E-05	2,00E-04	5,24E-06	2,45E-06	1,83E-06
NA	NA	NA	0	0	0	0	0	0	0	0	0	0	0
NA	NA	NA	0	0	0	0	0	0	0	0	0	0	0
NA	NA	NA	0	0	0	0	0	0	0	0	0	0	0
NA	NA	NA	0	0	0	0	0	0	0	0	0	0	0
Rare Single	1	NO	0	0	0	1,97E-05	1,97E-05	1,29E-05	2,69E-05	1,00E-04	5,24E-06	2,45E-06	9,08E-06
Rare Single	1	NO	0	0	0	0	0	0	0	0	0	0	0
NA	NA	NA	0	0	0	2,00E-04	2,00E-04	2,00E-04	2,00E-04	6,00E-04	1,00E-04	1,00E-04	4,00E-04

NA	NA	NA	0	0	0	0	0	0	0	0	0	0	0
NA	NA	NA	0	0	0	1,97E-05	1,97E-05	2,57E-05	1,35E-05	.	5,24E-06	2,45E-06	.
NA	NA	NA	0	0	0	0	0	0	0	0	0	0	0
Rare Single	2	NO	0	0	0	6,57E-05	6,57E-05	5,14E-05	8,07E-05	1,00E-04	3,52E-05	2,62E-05	3,34E-05
NA	NA	NA	0	0	0	0	0	0	0	0	0	0	0
NA	NA	NA	0	0	0	2,00E-04	2,00E-04	2,00E-04	1,00E-04	3,00E-04	1,00E-04	1,00E-04	2,00E-04
NA	NA	NA	0	0	0	0	0	0	0	0	0	0	0
Rare Single	NA	YES	0	0	0	0	0	0	0	0	0	0	0
NA	NA	NA	0	0	0	0	0	0	0	0	0	0	0
NA	NA	NA	0	0	0	1,97E-05	1,97E-05	3,86E-05	0	4,41E-05	5,24E-06	2,45E-06	6,25E-06
NA	NA	NA	1	0,000427	2342	0	0	0	0	0	0	0	0
NA	NA	NA	0	0	0	0	0	0	0	0	0	0	0
NA	NA	NA	1	0,000427	2342	0	0	0	0	0	0	0	0
NA	NA	NA	0	0	0	4,60E-05	4,60E-05	2,57E-05	6,73E-05	1,00E-04	2,11E-05	1,53E-05	4,30E-05
NA	NA	NA	2	0,000854	2342	3,00E-04	3,00E-04	3,00E-04	2,00E-04	5,00E-04	2,00E-04	2,00E-04	3,00E-04
NA	NA	NA	0	0	0	0	0	0	0	0	0	0	0
NA	NA	NA	0	0	0	2,63E-05	2,63E-05	3,86E-05	1,35E-05	9,66E-05	8,15E-06	5,14E-06	1,92E-05
NA	NA	NA	0	0	0	1,32E-05	1,31E-05	0	2,70E-05	.	2,19E-06	8,20E-07	.
Rare Single	2	NO	0	0	0	6,58E-06	6,57E-06	1,29E-05	0	2,42E-05	0	0	.
Rare Single	2	NO	0	0	0	0	0	0	0	0	0	0	0
Rare Single	2	NO	0	0	0	0	0	0	0	0	0	0	0
NA	NA	NA	3	0,001281	2342	3,00E-04	3,00E-04	3,00E-04	3,00E-04	9,00E-04	2,00E-04	2,00E-04	6,00E-04
NA	NA	NA	1	0,000427	2342	0	0	0	0	0	0	0	0
NA	NA	NA	0	0	0	6,57E-06	6,57E-06	1,29E-05	0	2,41E-05	0	0	.
NA	NA	NA	0	0	0	0	0	0	0	0	0	0	0
NA	NA	NA	0	0	0	3,94E-05	3,94E-05	5,14E-05	2,69E-05	1,00E-04	1,71E-05	1,13E-05	3,05E-05
NA	NA	NA	0	0	0	0	0	0	0	0	0	0	0
NA	NA	NA	0	0	0	0	0	0	0	0	0	0	0
Rare Single	1	NO	0	0	0	0	0	0	0	0	0	0	0
NA	NA	NA	0	0	0	6,07E-05	6,62E-05	7,91E-05	4,14E-05	1,00E-04	3,15E-05	2,36E-05	5,16E-05
NA	NA	NA	0	0	0	0	0	0	0	0	0	0	0
NA	NA	NA	0	0	0	0	0	0	0	0	0	0	0
NA	NA	NA	0	0	0	0	0	0	0	0	0	0	0
NA	NA	NA	0	0	0	0	0	0	0	0	0	0	0
NA	NA	NA	1	0,000427	2342	6,57E-05	6,57E-05	8,99E-05	4,04E-05	7,24E-05	3,52E-05	2,62E-05	1,13E-05
NA	NA	NA	0	0	0	1,31E-05	1,31E-05	0	2,69E-05	4,83E-05	2,18E-06	8,20E-07	2,99E-06

NA	NA		NA	2	0,000854	2342	4,00E-04	4,00E-04	5,00E-04	3,00E-04	8,00E-04	3,00E-04	3,00E-04	6,00E-04
NA	NA		NA	0	0	0	0	0	0	0	0	0	0	0
NA	NA		NA	0	0	0	0	0	0	0	0	0	0	0
NA	NA		NA	0	0	0	3,00E-04	3,00E-04	2,00E-04	4,00E-04	2,70E-03	2,00E-04	2,00E-04	1,80E-03
NA	NA		NA	0	0	0	0	0	0	0	0	0	0	0
NA	NA		NA	0	0	0	0	0	0	0	0	0	0	0
NA	NA		NA	0	0	0	0,0012	0,0012	0,0011	0,0012	1,70E-03	0,001	0,001	1,40E-03
NA	NA		NA	0	0	0	2,00E-04	2,00E-04	1,00E-04	2,00E-04	5,00E-04	1,00E-04	8,71E-05	3,00E-04
NA	NA		NA	1	0,000427	2342	2,00E-04	2,00E-04	2,00E-04	1,00E-04	2,00E-04	1,00E-04	8,71E-05	1,00E-04
NA	NA		NA	0	0	0	0	0	0	0	0	0	0	0
NA	NA		NA	0	0	0	0	0	0	0	0	0	0	0
NA	NA		NA	0	0	0	1,97E-05	1,97E-05	1,29E-05	2,69E-05	2,94E-05	5,24E-06	2,45E-06	1,83E-06
NA	NA		NA	1	0,000427	2342	3,29E-05	3,28E-05	2,57E-05	4,04E-05	1,00E-04	1,26E-05	7,98E-06	3,05E-05
NA	NA		NA	3	0,001281	2342	0,0016	0,0016	0,0018	0,0015	3,10E-03	0,0015	0,0014	2,70E-03
NA	NA		NA	0	0	0	0	0	0	0	0	0	0	0
NA	NA		NA	0	0	0	7,88E-05	7,87E-05	8,99E-05	6,71E-05	2,10E-03	4,49E-05	3,51E-05	9,00E-04
NA	NA		NA	1	0,000427	2342	1,32E-05	1,31E-05	2,57E-05	0	4,83E-05	2,18E-06	8,20E-07	2,99E-06
NA	NA		NA	0	0	0	0	0	0	0	0	0	0	0
NA	NA		NA	1	0,000427	2342	1,33E-05	1,32E-05	1,30E-05	1,36E-05	2,98E-05	2,21E-06	8,30E-07	1,85E-06
NA	NA		NA	0	0	0	0	0	0	0	0	0	0	0
NA	NA		NA	0	0	0	3,29E-05	3,94E-05	2,57E-05	4,04E-05	3,00E-04	1,26E-05	7,98E-06	5,39E-05
NA	NA		NA	0	0	0	7,22E-05	7,22E-05	5,14E-05	9,40E-05	5,00E-04	3,97E-05	3,12E-05	2,00E-04
NA	NA		NA	0	0	0	0	0	0	0	0	0	0	0
Rare	Single	3	NO	0	0	0	6,58E-06	6,57E-06	0	1,35E-05	6,55E-05	0	0	.
NA	NA		NA	0	0	0	0	0	0	0	0	0	0	0
NA	NA		NA	0	0	0	0	0	0	0	0	0	0	0
NA	NA		NA	0	0	0	6,62E-06	6,58E-06	1,29E-05	0	1,47E-05	0	0	.
Rare	Single	3	NO	0	0	0	0	0	0	0	0	0	0	0
Rare	Single	3	NO	0	0	0	0	0	0	0	0	0	0	0
Rare	Single	3	NO	0	0	0	0	0	0	0	0	0	0	0
Rare	Single	3	NO	0	0	0	0	0	0	0	0	0	0	0
NA	NA		NA	0	0	0	0	0	0	0	0	0	0	0
NA	NA		NA	0	0	0	1,97E-05	1,97E-05	2,57E-05	1,35E-05	2,00E-04	5,24E-06	2,45E-06	1,83E-06
NA	NA		NA	0	0	0	4,60E-05	4,60E-05	6,43E-05	2,69E-05	8,82E-05	2,11E-05	1,53E-05	2,57E-05
NA	NA		NA	2	0,000854	2342	1,00E-04	1,00E-04	1,00E-04	2,00E-04	5,00E-04	8,67E-05	7,26E-05	2,00E-04
Rare	Single	1	NO	0	0	0	0	0	0	0	0	0	0	0

Rare Single	1	NO	0	0	0	6,57E-06	1,31E-05	1,29E-05	0	2,41E-05	0	0	.
Rare Single	1	NO	0	0	0	3,29E-05	3,29E-05	2,57E-05	4,04E-05	9,67E-05	1,26E-05	7,99E-06	1,92E-05
Rare Single	1	NO	0	0	0	1,97E-05	1,97E-05	3,86E-05	0	4,83E-05	5,24E-06	2,46E-06	3,00E-06
NA	NA	NA	0	0	0	3,00E-04	3,00E-04	4,00E-04	2,00E-04	3,00E-04	2,00E-04	2,00E-04	2,00E-04
NA	NA	NA	0	0	0	5,26E-05	5,25E-05	6,42E-05	4,03E-05	7,35E-05	2,56E-05	1,83E-05	1,86E-05
NA	NA	NA	1	0,000427	2342	6,57E-06	6,56E-06	0	1,34E-05	2,00E-04	0	0	.
|Rare Sing	NA	|NO	0	0	0	7,22E-05	7,22E-05	7,71E-05	6,71E-05	1,20E-03	3,97E-05	3,13E-05	3,00E-04
Rare Single	NA	YES	0	0	0	0	0	0	0	0	0	0	0
NA	NA	NA	0	0	0	6,57E-06	6,57E-06	0	1,35E-05	.	0	0	.
NA	NA	NA	0	0	0	2,00E-04	2,00E-04	3,00E-04	1,00E-04	6,00E-04	1,00E-04	1,00E-04	4,00E-04
NA	NA	NA	0	0	0	0	0	0	0	0	0	0	0
NA	NA	NA	0	0	0	6,57E-05	6,57E-05	1,00E-04	1,35E-05	1,00E-04	3,52E-05	2,62E-05	4,24E-05
NA	NA	NA	3	0,001281	2342	0	0	0	0	0	0	0	0
NA	NA	NA	0	0	0	1,32E-05	1,31E-05	0	2,69E-05	6,54E-05	2,18E-06	8,20E-07	.
NA	NA	NA	0	0	0	0	0	0	0	0	0	0	0
NA	NA	NA	0	0	0	5,91E-05	5,91E-05	3,85E-05	8,07E-05	3,00E-04	3,08E-05	2,21E-05	8,29E-05
NA	NA	NA	0	0	0	2,63E-05	2,63E-05	3,85E-05	1,35E-05	9,66E-05	8,14E-06	5,14E-06	1,92E-05
Rare Single	1	NO	0	0	0	3,28E-05	3,28E-05	2,57E-05	4,03E-05	7,35E-05	1,26E-05	7,98E-06	1,86E-05
NA	NA	NA	0	0	0	1,31E-05	1,31E-05	2,57E-05	0	2,94E-05	2,18E-06	8,20E-07	1,83E-06
NA	NA	NA	1	0,000427	2342	0	0	0	0	0	0	0	0
NA	NA	NA	1	0,000427	2342	0	0	0	0	0	0	0	0
NA	NA	NA	3	0,001281	2342	5,00E-04	5,00E-04	5,00E-04	4,00E-04	1,00E-03	4,00E-04	3,00E-04	5,00E-04
NA	NA	NA	0	0	0	2,08E-05	2,76E-05	2,70E-05	1,42E-05	6,00E-04	5,52E-06	2,53E-06	8,54E-05
NA	NA	NA	0	0	0	6,57E-06	6,57E-06	0	1,34E-05	1,47E-05	0	0	.
NA	NA	NA	0	0	0	1,97E-05	1,97E-05	0	4,04E-05	2,00E-04	5,24E-06	2,45E-06	2,84E-05
NA	NA	NA	0	0	0	0	0	0	0	0	0	0	0
NA	NA	NA	0	0	0	0	0	0	0	0	0	0	0
NA	NA	NA	0	0	0	1,97E-05	1,97E-05	2,57E-05	1,35E-05	4,83E-05	5,24E-06	2,45E-06	2,99E-06
NA	NA	NA	0	0	0	0	0	0	0	0	0	0	0
NA	NA	NA	0	0	0	0	6,57E-06	0	0	.	0	0	.
NA	NA	NA	0	0	0	3,29E-05	3,28E-05	3,85E-05	2,69E-05	1,00E-04	1,26E-05	7,98E-06	3,05E-05
NA	NA	NA	1	0,000427	2342	2,00E-04	2,00E-04	1,00E-04	2,00E-04	6,00E-04	1,00E-04	9,70E-05	3,00E-04
NA	NA	NA	0	0	0	0	0	0	0	0	0	0	0
NA	NA	NA	0	0	0	1,97E-05	1,97E-05	1,29E-05	2,69E-05	1,47E-05	5,25E-06	2,46E-06	.
NA	NA	NA	0	0	0	0	0	0	0	0	0	0	0
NA	NA	NA	0	0	0	0	0	0	0	0	0	0	0

NA	NA	NA	0	0	0	0	0	0	0	0	0	0	0
Rare Single	2	NO	1	0,000427	2342	0	0	0	0	0	0	0	0
NA	NA	NA	1	0,000427	2342	3,95E-05	3,94E-05	5,15E-05	2,70E-05	5,88E-05	1,72E-05	1,13E-05	1,13E-05
NA	NA	NA	0	0	0	0	0	0	0	0	0	0	0
NA	NA	NA	0	0	0	0	0	0	0	0	0	0	0
NA	NA	NA	1	0,000427	2342	2,63E-05	2,63E-05	2,57E-05	2,69E-05	2,00E-04	8,14E-06	5,14E-06	1,83E-06
NA	NA	NA	0	0	0	2,63E-05	2,63E-05	1,29E-05	4,03E-05	9,65E-05	8,14E-06	5,14E-06	1,91E-05
NA	NA	NA	0	0	0	1,97E-05	1,97E-05	2,57E-05	1,34E-05	4,41E-05	5,24E-06	2,45E-06	6,25E-06
NA	NA	NA	0	0	0	6,57E-06	6,57E-06	0	1,35E-05	1,47E-05	0	0	.
NA	NA	NA	1	0,000427	2342	1,31E-05	1,31E-05	1,29E-05	1,35E-05	2,94E-05	2,18E-06	8,20E-07	1,83E-06
NA	NA	NA	0	0	0	1,31E-05	1,31E-05	2,57E-05	0	6,54E-05	2,18E-06	8,20E-07	.
NA	NA	NA	0	0	0	0	0	0	0	0	0	0	0
NA	NA	NA	0	0	0	0	0	0	0	0	0	0	0
NA	NA	NA	0	0	0	1,00E-04	1,00E-04	1,00E-04	9,40E-05	4,00E-04	7,09E-05	5,74E-05	2,00E-04
NA	NA	NA	0	0	0	6,94E-06	6,78E-06	0	1,44E-05	1,49E-05	0	0	.
NA	NA	NA	0	0	0	5,92E-05	5,91E-05	6,43E-05	5,38E-05	1,00E-04	3,08E-05	2,21E-05	5,09E-05
NA	NA	NA	0	0	0	2,00E-04	2,00E-04	2,00E-04	2,00E-04	6,00E-04	1,00E-04	1,00E-04	4,00E-04
NA	NA	NA	2	0,000854	2342	3,00E-04	3,00E-04	2,00E-04	3,00E-04	1,00E-03	2,00E-04	2,00E-04	5,00E-04
NA	NA	NA	0	0	0	0	0	0	0	0	0	0	0
Rare Single	1	NO	0	0	0	0	0	0	0	0	0	0	0
NA	NA	NA	0	0	0	1,32E-05	1,31E-05	2,57E-05	0	1,47E-05	2,19E-06	8,20E-07	.
NA	NA	NA	0	0	0	0	0	0	0	0	0	0	0
NA	NA	NA	4	0,001708	2342	3,00E-04	3,00E-04	2,00E-04	5,00E-04	2,70E-03	3,00E-04	2,00E-04	1,90E-03
NA	NA	NA	1	0,000427	2342	9,20E-05	9,19E-05	7,71E-05	1,00E-04	1,00E-03	5,53E-05	4,36E-05	3,00E-04
NA	NA	NA	0	0	0	1,00E-04	1,00E-04	1,00E-04	1,00E-04	3,00E-04	6,51E-05	5,32E-05	2,00E-04
NA	NA	NA	0	0	0	0	0	0	0	0	0	0	0
NA	NA	NA	0	0	0	1,97E-05	1,97E-05	1,29E-05	2,69E-05	6,55E-05	5,24E-06	2,45E-06	2,99E-06
Rare Single	2	NO	0	0	0	1,00E-04	1,00E-04	2,00E-04	8,07E-05	2,00E-04	8,67E-05	7,26E-05	9,90E-05
Rare Single	2	NO	0	0	0	0	0	0	0	0	0	0	0
Rare Single	2	NO	0	0	0	0	0	0	0	0	0	0	0
Rare Single	3	NO	0	0	0	1,00E-04	1,00E-04	1,00E-04	2,00E-04	3,00E-04	9,75E-05	8,26E-05	2,00E-04
Rare Single	3	NO	0	0	0	1,97E-05	1,97E-05	0	4,04E-05	2,94E-05	5,25E-06	2,46E-06	1,83E-06
Rare Single	2	NO	0	0	0	0	0	0	0	0	0	0	0
NA	NA	NA	0	0	0	0	0	0	0	0	0	0	0
NA	NA	NA	0	0	0	0	0	0	0	0	0	0	0
NA	NA	NA	0	0	0	0	0	0	0	0	0	0	0

NA	NA	NA	2	0,000854	2342	0,001	0,001	0,001	0,001	2,70E-03	9,00E-04	8,00E-04	1,90E-03
NA	NA	NA	0	0	0	1,97E-05	1,97E-05	2,57E-05	1,35E-05	6,55E-05	5,24E-06	2,45E-06	1,83E-06
NA	NA	NA	0	0	0	0	0	0	0	0	0	0	0
NA	NA	NA	2	0,000854	2342	4,00E-04	4,00E-04	4,00E-04	3,00E-04	1,10E-03	3,00E-04	3,00E-04	7,00E-04
Rare Single	2	NO	1	0,000427	2342	6,57E-06	6,57E-06	0	1,35E-05	2,00E-04	0	0	.
Rare Single	2	NO	0	0	0	0	0	0	0	0	0	0	0
NA	NA	NA	0	0	0	0	0	0	0	0	0	0	0
NA	NA	NA	0	0	0	0	0	0	0	0	0	0	0
NA	NA	NA	2	0,000854	2342	3,00E-04	3,00E-04	2,00E-04	3,00E-04	5,00E-04	2,00E-04	2,00E-04	3,00E-04
Rare Single	3	NO	0	0	0	6,57E-06	6,57E-06	1,29E-05	0	2,41E-05	0	0	.
Rare Single	3	NO	0	0	0	0	0	0	0	0	0	0	0
NA	NA	NA	0	0	0	1,32E-05	1,31E-05	1,29E-05	1,35E-05	1,00E-04	2,18E-06	8,20E-07	9,07E-06
NA	NA	NA	0	0	0	0	0	0	0	0	0	0	0
NA	NA	NA	0	0	0	0	0	0	0	0	0	0	0
NA	NA	NA	0	0	0	6,59E-06	6,57E-06	0	1,35E-05	1,47E-05	0	0	.
NA	NA	NA	0	0	0	6,58E-06	6,57E-06	0	1,35E-05	1,47E-05	0	0	.
NA	NA	NA	0	0	0	6,57E-06	6,57E-06	1,29E-05	0	1,47E-05	0	0	.
NA	NA	NA	5	0,002135	2342	0,0022	0,0022	0,0023	0,0021	7,80E-03	0,002	0,0019	6,80E-03
NA	NA	NA	0	0	0	1,97E-05	1,97E-05	1,29E-05	2,69E-05	6,55E-05	5,24E-06	2,45E-06	.
NA	NA	NA	0	0	0	0	0	0	0	0	0	0	0
Rare Single	3	YES	0	0	0	0	0	0	0	0	0	0	0
NA	NA	NA	0	0	0	0	0	0	0	0	0	0	0
NA	NA	NA	0	0	0	6,57E-06	6,57E-06	1,29E-05	0	6,54E-05	0	0	.
NA	NA	NA	0	0	0	0	0	0	0	0	0	0	0
NA	NA	NA	0	0	0	0	0	0	0	0	0	0	0
NA	NA	NA	0	0	0	0	0	0	0	0	0	0	0
NA	NA	NA	2	0,000854	2342	2,00E-04	2,00E-04	2,00E-04	1,00E-04	3,00E-04	1,00E-04	9,71E-05	9,90E-05
NA	NA	NA	0	0	0	0	0	0	0	0	0	0	0
NA	NA	NA	0	0	0	0	0	0	0	0	0	0	0
NA	NA	NA	0	0	0	3,94E-05	3,94E-05	2,57E-05	5,38E-05	2,00E-04	1,72E-05	1,13E-05	2,84E-05
NA	NA	NA	0	0	0	6,58E-06	6,57E-06	0	1,35E-05	6,55E-05	0	0	.
NA	NA	NA	0	0	0	1,00E-04	1,00E-04	1,00E-04	1,00E-04	5,00E-04	8,66E-05	7,25E-05	2,00E-04
NA	NA	NA	0	0	0	0	0	0	0	0	0	0	0
NA	NA	NA	0	0	0	0	0	0	0	0	0	0	0
Rare Single	3	YES	0	0	0	0	0	0	0	0	0	0	0
NA	NA	NA	0	0	0	0	0	0	0	0	0	0	0

NA	NA	NA	0	0	0	0	0	0	0	0	0	0	0
NA	NA	NA	0	0	0	2,00E-04	2,00E-04	1,00E-04	2,00E-04	2,20E-03	1,00E-04	9,55E-05	1,00E-03
Rare Single	1	NO	0	0	0	5,91E-05	5,91E-05	5,14E-05	6,72E-05	2,00E-04	3,08E-05	2,21E-05	6,96E-05
NA	NA	NA	0	0	0	6,57E-06	6,57E-06	0	1,35E-05	1,47E-05	0	0	.
NA	NA	NA	0	0	0	0	0	0	0	0	0	0	0
Rare Single	1	NO	0	0	0	5,26E-05	5,26E-05	7,71E-05	2,69E-05	1,00E-04	2,56E-05	1,83E-05	4,30E-05
NA	NA	NA	0	0	0	3,94E-05	3,94E-05	5,14E-05	2,69E-05	5,88E-05	1,72E-05	1,13E-05	1,12E-05
NA	NA	NA	0	0	0	3,94E-05	3,94E-05	5,14E-05	2,69E-05	1,00E-04	1,72E-05	1,13E-05	3,05E-05
NA	NA	NA	0	0	0	0	0	0	0	0	0	0	0
NA	NA	NA	0	0	0	6,57E-06	6,57E-06	1,29E-05	0	2,41E-05	0	0	.
NA	NA	NA	0	0	0	0	0	0	0	0	0	0	0
NA	NA	NA	0	0	0	0	0	0	0	0	0	0	0
NA	NA	NA	0	0	0	6,58E-06	6,57E-06	1,29E-05	0	2,42E-05	0	0	.
NA	NA	NA	0	0	0	0	0	0	0	0	0	0	0
NA	NA	NA	0	0	0	0	0	0	0	0	0	0	0
NA	NA	NA	1	0,000427	2342	6,57E-06	6,57E-06	0	1,35E-05	2,41E-05	0	0	.
NA	NA	NA	0	0	0	0	0	0	0	0	0	0	0
NA	NA	NA	0	0	0	6,57E-06	6,57E-06	1,29E-05	0	1,47E-05	0	0	.
NA	NA	NA	1	0,000427	2342	2,63E-05	2,63E-05	1,28E-05	4,03E-05	4,41E-05	8,14E-06	5,14E-06	6,25E-06
NA	NA	NA	0	0	0	6,57E-06	6,57E-06	1,29E-05	0	1,47E-05	0	0	.
Rare Single	2	NO	0	0	0	3,29E-05	3,28E-05	3,85E-05	2,69E-05	5,88E-05	1,26E-05	7,98E-06	1,12E-05
Rare Single	2	NO	0	0	0	0	0	0	0	0	0	0	0
Rare Single	2	NO	0	0	0	0	0	0	0	0	0	0	0
Rare Single	2	NO	0	0	0	6,00E-04	6,00E-04	6,00E-04	6,00E-04	3,30E-03	5,00E-04	5,00E-04	1,50E-03
NA	NA	NA	0	0	0	6,57E-05	6,57E-05	6,42E-05	6,72E-05	2,00E-04	3,52E-05	2,62E-05	8,45E-05
NA	NA	NA	0	0	0	3,94E-05	3,94E-05	3,86E-05	4,04E-05	8,82E-05	1,72E-05	1,13E-05	2,57E-05
NA	NA	NA	0	0	0	3,94E-05	3,94E-05	3,85E-05	4,04E-05	8,82E-05	1,72E-05	1,13E-05	2,57E-05
NA	NA	NA	0	0	0	0	0	0	0	0	0	0	0
NA	NA	NA	0	0	0	2,63E-05	2,63E-05	3,86E-05	1,35E-05	6,55E-05	8,15E-06	5,15E-06	6,25E-06
NA	NA	NA	0	0	0	3,29E-05	3,29E-05	5,15E-05	1,35E-05	6,56E-05	1,26E-05	7,99E-06	1,13E-05
NA	NA	NA	0	0	0	0	0	0	0	0	0	0	0
Rare Single	3	NO	0	0	0	0	0	0	0	0	0	0	0
NA	NA	NA	0	0	0	0	0	0	0	0	0	0	0
NA	NA	NA	2	0,000854	2342	5,25E-05	5,25E-05	3,85E-05	6,71E-05	2,00E-04	2,56E-05	1,83E-05	5,58E-05
NA	NA	NA	0	0	0	0	0	0	0	0	0	0	0
Rare Single	1	NO	0	0	0	0	0	0	0	0	0	0	0

Rare Single	1	NO	2	0,000854	2342	0,0018	0,0018	0,0016	0,0019	6,20E-03	0,0016	0,0015	5,30E-03
NA	NA	NA	0	0	0	0	0	0	0	0	0	0	0
NA	NA	NA	0	0	0	6,57E-06	6,56E-06	0	1,34E-05	2,00E-04	0	0	.
NA	NA	NA	0	0	0	0	0	0	0	0	0	0	0
NA	NA	NA	0	0	0	2,00E-04	2,00E-04	2,00E-04	3,00E-04	1,00E-03	2,00E-04	2,00E-04	5,00E-04
NA	NA	NA	0	0	0	1,00E-04	1,00E-04	1,00E-04	1,00E-04	8,00E-04	8,66E-05	7,25E-05	2,00E-04
NA	NA	NA	0	0	0	3,94E-05	3,94E-05	3,86E-05	4,03E-05	2,00E-04	1,72E-05	1,13E-05	2,84E-05
NA	NA	NA	1	0,000427	2342	6,57E-06	6,57E-06	0	1,35E-05	1,47E-05	0	0	.
NA	NA	NA	0	0	0	1,00E-04	1,00E-04	1,00E-04	2,00E-04	5,00E-04	9,75E-05	8,26E-05	3,00E-04
NA	NA	NA	0	0	0	0	0	0	0	0	0	0	0
NA	NA	NA	0	0	0	0	0	0	0	0	0	0	0
NA	NA	NA	0	0	0	1,00E-04	1,00E-04	9,00E-05	1,00E-04	3,10E-03	6,51E-05	5,32E-05	1,60E-03
NA	NA	NA	1	0,000427	2342	7,22E-05	7,22E-05	6,42E-05	8,06E-05	4,00E-04	3,97E-05	3,13E-05	2,87E-05
NA	NA	NA	0	0	0	0	0	0	0	0	0	0	0
NA	NA	NA	0	0	0	4,60E-05	4,60E-05	0	9,42E-05	5,00E-04	2,11E-05	1,53E-05	2,00E-04
Rare Single	3	NO	0	0	0	7,00E-04	7,00E-04	7,00E-04	7,00E-04	5,20E-03	6,00E-04	6,00E-04	3,10E-03
NA	NA	NA	3	0,001281	2342	7,00E-04	7,00E-04	7,00E-04	7,00E-04	1,70E-03	6,00E-04	6,00E-04	1,00E-03
NA	NA	NA	0	0	0	1,32E-05	1,31E-05	0	2,69E-05	4,83E-05	2,18E-06	8,20E-07	2,99E-06
NA	NA	NA	0	0	0	1,97E-05	1,97E-05	3,86E-05	0	4,83E-05	5,24E-06	2,45E-06	2,99E-06
NA	NA	NA	1	0,000427	2342	6,58E-06	6,57E-06	1,29E-05	0	2,42E-05	0	0	.
NA	NA	NA	0	0	0	1,32E-05	1,31E-05	1,29E-05	1,35E-05	2,42E-05	2,18E-06	8,20E-07	.
NA	NA	NA	0	0	0	8,53E-05	8,53E-05	7,71E-05	9,40E-05	6,00E-04	4,95E-05	3,96E-05	2,00E-04
NA	NA	NA	0	0	0	0	0	0	0	0	0	0	0
NA	NA	NA	1	0,000427	2342	1,97E-05	1,97E-05	1,28E-05	2,69E-05	1,00E-04	5,24E-06	2,45E-06	9,07E-06
NA	NA	NA	0	0	0	0	0	0	0	0	0	0	0
NA	NA	NA	0	0	0	0	0	0	0	0	0	0	0
NA	NA	NA	0	0	0	0	0	0	0	0	0	0	0
NA	NA	NA	0	0	0	0	0	0	0	0	0	0	0
NA	NA	NA	0	0	0	1,98E-05	1,97E-05	3,87E-05	0	6,56E-05	5,26E-06	2,46E-06	1,83E-06
NA	NA	NA	0	0	0	0	0	0	0	0	0	0	0
NA	NA	NA	0	0	0	0	0	0	0	0	0	0	0
Rare Single	3	NO	0	0	0	0	0	0	0	0	0	0	0
NA	NA	NA	0	0	0	0	0	0	0	0	0	0	0
NA	NA	NA	1	0,000427	2342	1,31E-05	1,31E-05	2,57E-05	0	2,00E-04	2,18E-06	8,20E-07	.
NA	NA	NA	0	0	0	6,56E-06	6,56E-06	1,28E-05	0	1,47E-05	0	0	.
NA	NA	NA	0	0	0	1,00E-04	1,00E-04	1,00E-04	2,00E-04	6,00E-04	8,67E-05	7,26E-05	2,00E-04

NA	NA		NA	0	0	0	3,29E-05	3,28E-05	3,86E-05	2,69E-05	2,00E-04	1,26E-05	7,98E-06	1,92E-05
NA	NA		NA	0	0	0	0	0	0	0	0	0	0	0
NA	NA		NA	0	0	0	0	0	0	0	0	0	0	0
Rare	Single	1	NO	0	0	0	0	0	0	0	0	0	0	0
NA	NA		NA	0	0	0	5,27E-05	5,26E-05	7,72E-05	2,70E-05	2,00E-04	2,56E-05	1,84E-05	.
Rare	Single	3	NO	0	0	0	0	0	0	0	0	0	0	0
NA	NA		NA	0	0	0	0	0	0	0	0	0	0	0
NA	NA		NA	0	0	0	0	0	0	0	0	0	0	0
NA	NA		NA	0	0	0	0	0	0	0	0	0	0	0
NA	NA		NA	0	0	0	9,20E-05	9,20E-05	5,14E-05	1,00E-04	9,00E-04	5,53E-05	4,37E-05	4,00E-04
NA	NA		NA	3	0,00128	2343	5,00E-04	5,00E-04	6,00E-04	4,00E-04	1,60E-03	4,00E-04	4,00E-04	9,00E-04
NA	NA		NA	0	0	0	0	0	0	0	0	0	0	0
NA	NA		NA	0	0	0	0	0	0	0	0	0	0	0
NA	NA		NA	0	0	0	2,65E-05	2,63E-05	5,16E-05	0	4,86E-05	8,18E-06	5,17E-06	3,01E-06
NA	NA		NA	0	0	0	0	0	0	0	0	0	0	0
NA	NA		NA	0	0	0	0	0	0	0	0	0	0	0
NA	NA		NA	0	0	0	0	0	0	0	0	0	0	0
NA	NA		NA	1	0,000427	2342	9,85E-05	9,84E-05	1,00E-04	6,72E-05	1,20E-03	6,00E-05	4,88E-05	3,00E-04
NA	NA		NA	3	0,001281	2342	0,0015	0,0015	0,0018	0,0012	2,60E-03	0,0013	0,0013	2,10E-03
Rare	Single	3	NO	1	0,000427	2342	7,88E-05	7,88E-05	6,43E-05	9,40E-05	3,00E-04	4,50E-05	3,51E-05	5,39E-05
NA	NA		NA	1	0,000427	2342	1,31E-05	1,31E-05	2,57E-05	0	2,94E-05	2,18E-06	8,20E-07	1,83E-06
NA	NA		NA	0	0	0	0	0	0	0	0	0	0	0
NA	NA		NA	0	0	0	0	0	0	0	0	0	0	0
NA	NA		NA	0	0	0	0	0	0	0	0	0	0	0
NA	NA		NA	0	0	0	7,88E-05	7,87E-05	7,71E-05	8,06E-05	2,30E-03	4,49E-05	3,51E-05	1,00E-03
NA	NA		NA	0	0	0	6,57E-05	6,56E-05	3,86E-05	9,40E-05	1,30E-03	3,52E-05	2,62E-05	4,00E-04
NA	NA		NA	1	0,000427	2342	2,00E-04	2,00E-04	1,00E-04	2,00E-04	2,00E-04	1,00E-04	1,00E-04	1,00E-04
NA	NA		NA	0	0	0	2,00E-04	2,00E-04	3,00E-04	2,00E-04	6,00E-04	2,00E-04	2,00E-04	3,00E-04
NA	NA		NA	0	0	0	1,31E-05	1,31E-05	0	2,69E-05	4,00E-04	2,18E-06	8,20E-07	2,86E-05
NA	NA		NA	0	0	0	0	0	0	0	0	0	0	0
NA	NA		NA	0	0	0	6,57E-06	6,57E-06	0	1,35E-05	1,47E-05	0	0	.
NA	NA		NA	0	0	0	6,57E-06	6,57E-06	1,29E-05	0	1,47E-05	0	0	.
NA	NA		NA	3	0,001281	2342	0,0018	0,0018	0,0019	0,0017	6,50E-03	0,0016	0,0016	5,60E-03
NA	NA		NA	0	0	0	0	0	0	0	0	0	0	0
NA	NA		NA	0	0	0	3,29E-05	3,28E-05	3,85E-05	2,69E-05	2,00E-04	1,26E-05	7,98E-06	1,91E-05
NA	NA		NA	0	0	0	0	0	0	0	0	0	0	0

NA	NA	NA	0	0	0	6,57E-06	6,57E-06	0	1,35E-05	2,41E-05	0	0	.
NA	NA	NA	0	0	0	1,97E-05	1,97E-05	3,86E-05	0	4,41E-05	5,24E-06	2,45E-06	6,25E-06
NA	NA	NA	0	0	0	0	0	0	0	0	0	0	0
NA	NA	NA	0	0	0	7,88E-05	7,87E-05	2,57E-05	1,00E-04	2,00E-04	4,49E-05	3,51E-05	4,29E-05
Rare Single	1	NO	5	0,002135	2342	3,00E-04	3,00E-04	4,00E-04	3,00E-04	8,00E-04	3,00E-04	2,00E-04	3,00E-04
Rare Single	1	NO	0	0	0	0	0	0	0	0	0	0	0
NA	NA	NA	3	0,001281	2342	9,00E-04	9,00E-04	9,00E-04	9,00E-04	3,20E-03	8,00E-04	7,00E-04	2,60E-03
NA	NA	NA	0	0	0	1,31E-05	1,31E-05	1,29E-05	1,34E-05	4,00E-04	2,18E-06	8,20E-07	2,86E-05
NA	NA	NA	3	0,001281	2342	9,00E-04	9,00E-04	8,00E-04	0,001	3,30E-03	8,00E-04	8,00E-04	2,70E-03
NA	NA	NA	0	0	0	6,00E-04	6,00E-04	5,00E-04	6,00E-04	2,90E-03	5,00E-04	4,00E-04	2,00E-03
NA	NA	NA	0	0	0	0	0	0	0	0	0	0	0
Rare Single	2	NO	0	0	0	6,57E-06	6,57E-06	1,29E-05	0	1,47E-05	0	0	.
NA	NA	NA	0	0	0	6,57E-06	6,57E-06	1,29E-05	0	1,47E-05	0	0	.
NA	NA	NA	2	0,000854	2342	0	0	0	0	0	0	0	0
NA	NA	NA	0	0	0	1,31E-05	1,31E-05	1,28E-05	1,35E-05	1,00E-04	2,18E-06	8,20E-07	9,07E-06
NA	NA	NA	0	0	0	4,00E-04	4,00E-04	4,00E-04	3,00E-04	1,30E-03	3,00E-04	3,00E-04	9,00E-04
NA	NA	NA	0	0	0	0	0	0	0	0	0	0	0
Rare Single	1	NO	0	0	0	6,58E-06	6,57E-06	0	1,35E-05	2,42E-05	0	0	.
Rare Single	1	NO	0	0	0	2,63E-05	2,63E-05	3,85E-05	1,35E-05	7,23E-05	8,14E-06	5,14E-06	1,03E-05
NA	NA	NA	0	0	0	0	0	0	0	0	0	0	0
NA	NA	NA	0	0	0	0	0	0	0	0	0	0	0
NA	NA	NA	0	0	0	0	0	0	0	0	0	0	0
NA	NA	NA	0	0	0	8,53E-05	8,53E-05	8,99E-05	8,05E-05	1,00E-04	4,95E-05	3,96E-05	4,24E-05
NA	NA	NA	0	0	0	0	0	0	0	0	0	0	0
NA	NA	NA	0	0	0	6,59E-06	6,57E-06	1,29E-05	0	1,47E-05	0	0	.
NA	NA	NA	0	0	0	4,00E-04	4,00E-04	4,00E-04	3,00E-04	7,00E-04	3,00E-04	3,00E-04	3,00E-04
NA	NA	NA	1	0,000427	2342	7,22E-05	7,22E-05	5,14E-05	9,40E-05	2,10E-03	3,97E-05	3,13E-05	9,00E-04
NA	NA	NA	0	0	0	0	0	0	0	0	0	0	0
NA	NA	NA	0	0	0	0	0	0	0	0	0	0	0
NA	NA	NA	0	0	0	0	0	0	0	0	0	0	0
NA	NA	NA	0	0	0	6,57E-05	6,57E-05	6,42E-05	6,72E-05	2,00E-04	3,52E-05	2,62E-05	3,34E-05
NA	NA	NA	0	0	0	0	0	0	0	0	0	0	0
Rare Single	2	NO	0	0	0	0	0	0	0	0	0	0	0
Rare Single	2	NO	0	0	0	0	0	0	0	0	0	0	0
Rare Single	2	NO	0	0	0	0	0	0	0	0	0	0	0
Rare Single	1	NO	1	0,000427	2342	3,29E-05	3,28E-05	1,29E-05	5,39E-05	5,88E-05	1,26E-05	7,99E-06	1,13E-05

Rare Single	NA	YES	0	0	0	1,31E-05	1,31E-05	0	2,69E-05	6,55E-05	2,18E-06	8,20E-07	.
Rare Single	NA	YES	0	0	0	1,32E-05	1,97E-05	2,57E-05	0	4,83E-05	2,19E-06	8,20E-07	2,99E-06
Rare Single	3	NO	1	0,000427	2342	2,00E-04	2,00E-04	3,00E-04	1,00E-04	5,00E-04	2,00E-04	2,00E-04	3,00E-04
NA	NA	NA	1	0,000427	2342	8,00E-04	8,00E-04	8,00E-04	8,00E-04	1,40E-03	7,00E-04	6,00E-04	8,00E-04
NA	NA	NA	0	0	0	0	0	0	0	0	0	0	0
NA	NA	NA	0	0	0	6,57E-06	6,57E-06	0	1,35E-05	1,47E-05	0	0	.
NA	NA	NA	0	0	0	0	0	0	0	0	0	0	0
NA	NA	NA	0	0	0	1,32E-05	1,31E-05	0	2,69E-05	2,00E-04	2,19E-06	8,20E-07	.
NA	NA	NA	0	0	0	6,57E-06	6,57E-06	0	1,35E-05	1,47E-05	0	0	.
NA	NA	NA	0	0	0	9,86E-05	9,84E-05	6,43E-05	1,00E-04	3,00E-04	6,01E-05	4,88E-05	5,39E-05
NA	NA	NA	0	0	0	6,57E-06	6,57E-06	1,28E-05	0	1,47E-05	0	0	.
NA	NA	NA	0	0	0	0	0	0	0	0	0	0	0
NA	NA	NA	0	0	0	0	0	0	0	0	0	0	0
Rare Single	3	NO	1	0,000427	2342	1,97E-05	1,97E-05	2,57E-05	1,35E-05	2,94E-05	5,24E-06	2,45E-06	1,83E-06
Rare Single	3	NO	0	0	0	0	0	0	0	0	0	0	0
NA	NA	NA	0	0	0	6,58E-06	6,57E-06	1,29E-05	0	1,47E-05	0	0	.
NA	NA	NA	6	0,002562	2342	4,00E-04	4,00E-04	4,00E-04	4,00E-04	1,30E-03	3,00E-04	3,00E-04	9,00E-04
NA	NA	NA	1	0,000427	2342	1,00E-04	1,00E-04	1,00E-04	1,00E-04	8,00E-04	8,66E-05	7,25E-05	2,00E-04
NA	NA	NA	0	0	0	0	0	0	0	0	0	0	0
Rare Single	1	NO	0	0	0	0	0	0	0	0	0	0	0
NA	NA	NA	1	0,000427	2342	0	0	0	0	0	0	0	0
NA	NA	NA	0	0	0	0	0	0	0	0	0	0	0
NA	NA	NA	0	0	0	6,58E-06	6,57E-06	1,29E-05	0	2,00E-04	0	0	.
Rare Single	2	NO	0	0	0	0	0	0	0	0	0	0	0
NA	NA	NA	1	0,000427	2342	3,00E-04	3,00E-04	2,00E-04	4,00E-04	4,80E-03	2,00E-04	2,00E-04	2,90E-03
NA	NA	NA	0	0	0	6,68E-06	6,61E-06	1,30E-05	0	1,48E-05	0	0	.
Rare Single	2	NO	0	0	0	0	0	0	0	0	0	0	0
Rare Single	2	NO	0	0	0	0	0	0	0	0	0	0	0
NA	NA	NA	1	0,000427	2342	5,92E-05	5,91E-05	2,57E-05	9,42E-05	5,00E-04	3,08E-05	2,21E-05	2,00E-04
NA	NA	NA	0	0	0	1,00E-04	1,00E-04	7,71E-05	1,00E-04	2,00E-04	7,09E-05	5,75E-05	9,90E-05
NA	NA	NA	2	0,000854	2342	1,31E-05	1,31E-05	1,29E-05	1,35E-05	4,83E-05	2,18E-06	8,20E-07	2,99E-06
NA	NA	NA	0	0	0	0	0	0	0	0	0	0	0
NA	NA	NA	0	0	0	0	0	0	0	0	0	0	0
NA	NA	NA	0	0	0	6,57E-06	6,57E-06	0	1,35E-05	1,47E-05	0	0	.
NA	NA	NA	1	0,000427	2342	2,00E-04	2,00E-04	3,00E-04	2,00E-04	8,00E-04	2,00E-04	2,00E-04	5,00E-04
NA	NA	NA	0	0	0	0	0	0	0	0	0	0	0

NA	NA	NA	0	0	0	0	0	0	0	0	0	0	0
NA	NA	NA	0	0	0	1,97E-05	1,97E-05	1,29E-05	2,69E-05	4,41E-05	5,24E-06	2,45E-06	6,25E-06
NA	NA	NA	0	0	0	0	0	0	0	0	0	0	0
NA	NA	NA	0	0	0	6,57E-06	6,57E-06	0	1,35E-05	1,47E-05	0	0	.
NA	NA	NA	0	0	0	1,31E-05	1,31E-05	1,28E-05	1,35E-05	2,41E-05	2,18E-06	8,20E-07	.
NA	NA	NA	0	0	0	0	0	0	0	0	0	0	0
NA	NA	NA	0	0	0	4,60E-05	6,56E-05	3,86E-05	5,37E-05	1,20E-03	2,11E-05	1,53E-05	3,00E-04
NA	NA	NA	1	0,000427	2342	1,32E-05	1,31E-05	2,57E-05	0	6,56E-05	2,19E-06	8,20E-07	.
NA	NA	NA	0	0	0	0	0	0	0	0	0	0	0
NA	NA	NA	0	0	0	0	0	0	0	0	0	0	0
NA	NA	NA	0	0	0	2,63E-05	2,62E-05	2,57E-05	2,68E-05	4,41E-05	8,13E-06	5,14E-06	6,25E-06
NA	NA	NA	0	0	0	0	0	0	0	0	0	0	0
NA	NA	NA	1	0,000427	2342	5,91E-05	5,91E-05	2,57E-05	9,40E-05	2,00E-04	3,08E-05	2,21E-05	6,95E-05
NA	NA	NA	0	0	0	2,63E-05	2,62E-05	5,14E-05	0	3,00E-04	8,14E-06	5,14E-06	5,28E-05
NA	NA	NA	0	0	0	1,31E-05	1,31E-05	1,28E-05	1,35E-05	2,41E-05	2,18E-06	8,20E-07	.
NA	NA	NA	0	0	0	0	0	0	0	0	0	0	0
NA	NA	NA	0	0	0	6,58E-06	6,57E-06	1,29E-05	0	6,55E-05	0	0	.
NA	NA	NA	2	0,000854	2342	1,00E-04	1,00E-04	2,00E-04	9,42E-05	5,00E-04	9,16E-05	7,71E-05	2,00E-04
NA	NA	NA	0	0	0	0	0	0	0	0	0	0	0
NA	NA	NA	0	0	0	2,00E-04	2,00E-04	2,00E-04	2,00E-04	4,00E-04	1,00E-04	1,00E-04	8,88E-05
NA	NA	NA	0	0	0	6,58E-06	6,57E-06	0	1,35E-05	6,55E-05	0	0	.
NA	NA	NA	0	0	0	3,00E-04	3,00E-04	1,00E-04	5,00E-04	1,04E-02	3,00E-04	2,00E-04	7,30E-03
NA	NA	NA	0	0	0	1,97E-05	1,97E-05	2,57E-05	1,35E-05	2,00E-04	5,24E-06	2,45E-06	1,83E-06
NA	NA	NA	6	0,002562	2342	0	0	0	0	0	0	0	0
NA	NA	NA	1	0,000427	2342	1,32E-05	1,31E-05	2,57E-05	0	6,56E-05	2,19E-06	8,20E-07	.
NA	NA	NA	1	0,000427	2342	2,63E-05	2,63E-05	1,29E-05	4,04E-05	7,25E-05	8,15E-06	5,15E-06	1,03E-05
NA	NA	NA	0	0	0	6,57E-05	6,56E-05	7,72E-05	5,37E-05	4,00E-04	3,52E-05	2,62E-05	3,04E-05
NA	NA	NA	0	0	0	7,22E-05	7,22E-05	9,00E-05	5,37E-05	3,00E-04	3,97E-05	3,13E-05	1,00E-04
NA	NA	NA	1	0,000427	2342	3,00E-04	3,00E-04	3,00E-04	3,00E-04	1,00E-03	2,00E-04	2,00E-04	7,00E-04
NA	NA	NA	1	0,000427	2342	4,00E-04	4,00E-04	4,00E-04	4,00E-04	7,00E-04	3,00E-04	3,00E-04	5,00E-04
NA	NA	NA	0	0	0	1,97E-05	1,97E-05	2,57E-05	1,35E-05	4,41E-05	5,24E-06	2,45E-06	6,25E-06
NA	NA	NA	1	0,000427	2342	0	0	0	0	0	0	0	0
NA	NA	NA	0	0	0	0	0	0	0	0	0	0	0
NA	NA	NA	0	0	0	0	0	0	0	0	0	0	0
NA	NA	NA	1	0,000427	2342	0	0	0	0	0	0	0	0
NA	NA	NA	0	0	0	4,01E-05	3,96E-05	6,52E-05	1,37E-05	7,40E-05	1,74E-05	1,15E-05	1,87E-05

NA	NA	NA	4	0,001708	2342	3,00E-04	3,00E-04	3,00E-04	3,00E-04	6,00E-04	2,00E-04	2,00E-04	3,00E-04
NA	NA	NA	0	0	0	0,0011	0,0011	0,0011	0,0011	2,90E-03	0,001	9,00E-04	1,40E-03
NA	NA	NA	0	0	0	6,61E-06	6,60E-06	0	1,35E-05	1,48E-05	0	0	.
NA	NA	NA	0	0	0	1,31E-05	1,31E-05	1,29E-05	1,35E-05	2,94E-05	2,18E-06	8,20E-07	1,83E-06
NA	NA	NA	0	0	0	1,31E-05	1,31E-05	0	2,69E-05	1,00E-04	2,18E-06	8,20E-07	9,07E-06
Rare Single	1	NO	0	0	0	1,31E-05	1,31E-05	1,28E-05	1,35E-05	2,41E-05	2,18E-06	8,20E-07	.
Rare Single	1	NO	3	0,001281	2342	6,00E-04	6,00E-04	6,00E-04	6,00E-04	2,20E-03	5,00E-04	5,00E-04	1,70E-03
NA	NA	NA	0	0	0	1,97E-05	1,97E-05	2,57E-05	1,35E-05	2,94E-05	5,25E-06	2,46E-06	1,83E-06
NA	NA	NA	0	0	0	0	0	0	0	0	0	0	0
NA	NA	NA	0	0	0	6,57E-06	6,57E-06	1,29E-05	0	1,47E-05	0	0	.
Rare Single	1	NO	0	0	0	0	0	0	0	0	0	0	0
Rare Single	1	NO	0	0	0	0,0011	0,0011	0,0011	0,0011	3,80E-03	0,001	9,00E-04	3,10E-03
NA	NA	NA	0	0	0	1,00E-04	4,00E-04	1,00E-04	1,00E-04	4,00E-04	2,07E-05	8,34E-06	2,60E-05
NA	NA	NA	0	0	0	1,32E-05	1,31E-05	0	2,69E-05	2,42E-05	2,19E-06	8,20E-07	.
NA	NA	NA	0	0	0	3,29E-05	3,28E-05	5,14E-05	1,35E-05	6,55E-05	1,26E-05	7,98E-06	1,12E-05
NA	NA	NA	0	0	0	0	0	0	0	0	0	0	0
Rare Single	2	NO	0	0	0	0	0	0	0	0	0	0	0
NA	NA	NA	0	0	0	0	0	0	0	0	0	0	0
NA	NA	NA	0	0	0	0	0	0	0	0	0	0	0
NA	NA	NA	0	0	0	6,57E-06	6,57E-06	0	1,35E-05	2,00E-04	0	0	.
NA	NA	NA	0	0	0	0	0	0	0	0	0	0	0
NA	NA	NA	0	0	0	0	0	0	0	0	0	0	0
NA	NA	NA	2	0,000854	2342	8,00E-04	8,00E-04	9,00E-04	6,00E-04	2,20E-03	6,00E-04	6,00E-04	1,40E-03
NA	NA	NA	3	0,001281	2342	0,0016	0,0016	0,0019	0,0014	2,90E-03	0,0015	0,0014	2,40E-03
NA	NA	NA	0	0	0	6,58E-06	6,57E-06	0	1,35E-05	2,42E-05	0	0	.
NA	NA	NA	0	0	0	3,28E-05	3,28E-05	3,86E-05	2,69E-05	2,00E-04	1,26E-05	7,98E-06	6,25E-06
NA	NA	NA	0	0	0	1,97E-05	1,97E-05	1,29E-05	2,69E-05	2,94E-05	5,24E-06	2,45E-06	1,83E-06
NA	NA	NA	3	0,001281	2342	0,0012	0,0012	0,0012	0,0013	1,90E-03	0,0011	0,001	1,50E-03
NA	NA	NA	0	0	0	2,63E-05	2,63E-05	2,57E-05	2,69E-05	4,41E-05	8,14E-06	5,14E-06	6,25E-06
NA	NA	NA	0	0	0	0	0	0	0	0	0	0	0
NA	NA	NA	1	0,000427	2342	6,57E-05	6,56E-05	7,71E-05	5,37E-05	1,00E-04	3,52E-05	2,62E-05	6,00E-05
NA	NA	NA	0	0	0	0	0	0	0	0	0	0	0
NA	NA	NA	0	0	0	2,00E-04	2,00E-04	2,00E-04	1,00E-04	3,00E-04	1,00E-04	9,24E-05	2,00E-04
Rare Single	1	NO	0	0	0	0	0	0	0	0	0	0	0
Rare Single	1	NO	0	0	0	0	0	0	0	0	0	0	0
NA	NA	NA	1	0,000427	2342	2,00E-04	2,00E-04	1,00E-04	2,00E-04	1,20E-03	1,00E-04	9,70E-05	6,00E-04

NA	NA	NA	0	0	0	0	0	0	0	0	0	0	0
NA	NA	NA	0	0	0	6,57E-06	6,57E-06	1,29E-05	0	.	0	0	.
NA	NA	NA	0	0	0	0	0	0	0	0	0	0	0
NA	NA	NA	0	0	0	3,29E-05	3,28E-05	3,85E-05	2,69E-05	1,00E-04	1,26E-05	7,98E-06	3,05E-05
NA	NA	NA	2	0,000854	2342	2,00E-04	2,00E-04	2,00E-04	1,00E-04	8,00E-04	1,00E-04	9,70E-05	4,00E-04
NA	NA	NA	0	0	0	0	0	0	0	0	0	0	0
Rare Single	3	NO	0	0	0	0	0	0	0	0	0	0	0
NA	NA	NA	0	0	0	1,00E-04	1,00E-04	2,00E-04	5,38E-05	3,00E-04	9,75E-05	8,26E-05	2,00E-04
NA	NA	NA	1	0,000427	2342	2,00E-04	2,00E-04	2,00E-04	2,00E-04	7,00E-04	1,00E-04	1,00E-04	4,00E-04
NA	NA	NA	1	0,000427	2342	3,95E-05	5,25E-05	2,58E-05	5,39E-05	4,00E-04	1,72E-05	1,13E-05	2,87E-05
NA	NA	NA	0	0	0	0	0	0	0	0	0	0	0
NA	NA	NA	0	0	0	1,31E-05	1,31E-05	1,29E-05	1,35E-05	2,94E-05	2,18E-06	8,20E-07	1,83E-06
NA	NA	NA	1	0,000427	2342	3,30E-05	3,29E-05	3,87E-05	2,71E-05	2,00E-04	1,27E-05	8,01E-06	2,84E-05
NA	NA	NA	0	0	0	6,57E-06	6,57E-06	0	1,35E-05	6,55E-05	0	0	.
NA	NA	NA	0	0	0	0	0	0	0	0	0	0	0
NA	NA	NA	0	0	0	1,00E-04	1,00E-04	2,00E-04	1,00E-04	4,00E-04	9,74E-05	8,25E-05	2,00E-04
NA	NA	NA	0	0	0	0	6,56E-06	0	0	.	0	0	.
NA	NA	NA	0	0	0	3,00E-04	3,00E-04	3,00E-04	4,00E-04	1,20E-03	3,00E-04	2,00E-04	9,00E-04
NA	NA	NA	0	0	0	0	0	0	0	0	0	0	0
NA	NA	NA	0	0	0	0	0	0	0	0	0	0	0
NA	NA	NA	0	0	0	6,59E-06	6,57E-06	0	1,35E-05	2,00E-04	0	0	.
NA	NA	NA	0	0	0	9,00E-04	9,00E-04	9,00E-04	8,00E-04	1,50E-03	7,00E-04	7,00E-04	1,20E-03
NA	NA	NA	0	0	0	0	0	0	0	0	0	0	0
NA	NA	NA	2	0,000854	2342	0	0	0	0	0	0	0	0
NA	NA	NA	0	0	0	5,25E-05	5,25E-05	3,85E-05	6,71E-05	2,00E-04	2,56E-05	1,83E-05	5,57E-05
NA	NA	NA	0	0	0	0	0	0	0	0	0	0	0
NA	NA	NA	0	0	0	0	0	0	0	0	0	0	0
NA	NA	NA	0	0	0	0	0	0	0	0	0	0	0
NA	NA	NA	0	0	0	0	0	0	0	0	0	0	0
Rare Single	1	NO	0	0	0	1,31E-05	1,31E-05	0	2,69E-05	6,55E-05	2,18E-06	8,20E-07	.
NA	NA	NA	0	0	0	0	0	0	0	0	0	0	0
NA	NA	NA	0	0	0	0	0	0	0	0	0	0	0
NA	NA	NA	0	0	0	0	0	0	0	0	0	0	0
NA	NA	NA	1	0,000427	2342	6,58E-06	6,57E-06	0	1,35E-05	1,47E-05	0	0	.
NA	NA	NA	0	0	0	1,33E-05	1,31E-05	0	2,73E-05	2,45E-05	2,21E-06	8,30E-07	.
NA	NA	NA	0	0	0	6,57E-06	6,57E-06	1,29E-05	0	1,47E-05	0	0	.

NA	NA	NA	0	0	0	3,00E-04	3,00E-04	2,00E-04	3,00E-04	6,20E-03	2,00E-04	2,00E-04	3,90E-03
NA	NA	NA	0	0	0	0	0	0	0	0	0	0	0
NA	NA	NA	0	0	0	0	0	0	0	0	0	0	0
NA	NA	NA	0	0	0	2,63E-05	2,63E-05	2,57E-05	2,69E-05	7,24E-05	8,14E-06	5,14E-06	1,03E-05
NA	NA	NA	1	0,000427	2342	6,57E-06	6,57E-06	1,29E-05	0	2,41E-05	0	0	.
NA	NA	NA	0	0	0	3,29E-05	3,28E-05	5,14E-05	1,35E-05	7,35E-05	1,26E-05	7,98E-06	1,86E-05
NA	NA	NA	0	0	0	0	0	0	0	0	0	0	0
NA	NA	NA	0	0	0	1,32E-05	1,31E-05	1,29E-05	1,35E-05	2,42E-05	2,19E-06	8,20E-07	.
NA	NA	NA	3	0,001281	2342	0	0	0	0	0	0	0	0
NA	NA	NA	0	0	0	3,94E-05	3,94E-05	5,14E-05	2,69E-05	3,00E-04	1,72E-05	1,13E-05	5,39E-05
NA	NA	NA	0	0	0	0	0	0	0	0	0	0	0
NA	NA	NA	0	0	0	0	0	0	0	0	0	0	0
NA	NA	NA	0	0	0	1,32E-05	1,31E-05	0	2,70E-05	1,00E-04	2,19E-06	8,20E-07	9,09E-06
NA	NA	NA	1	0,000427	2342	6,57E-06	6,57E-06	0	1,34E-05	1,47E-05	0	0	.
NA	NA	NA	0	0	0	0	0	0	0	0	0	0	0
NA	NA	NA	2	0,000854	2342	7,00E-04	7,00E-04	7,00E-04	7,00E-04	2,50E-03	6,00E-04	5,00E-04	2,00E-03
NA	NA	NA	0	0	0	6,57E-06	6,57E-06	1,29E-05	0	1,47E-05	0	0	.
Rare Single	1	NO	0	0	0	0	0	0	0	0	0	0	0
NA	NA	NA	0	0	0	0	0	0	0	0	0	0	0
Rare Single	2	NO	0	0	0	1,00E-04	1,00E-04	8,99E-05	1,00E-04	7,00E-04	7,08E-05	5,74E-05	3,00E-04
Rare Single	2	NO	0	0	0	0	0	0	0	0	0	0	0
NA	NA	NA	0	0	0	0	0	0	0	0	0	0	0
Rare Single	2	NO	2	0,000854	2342	7,23E-05	7,22E-05	6,43E-05	8,06E-05	2,10E-03	3,97E-05	3,13E-05	9,00E-04
NA	NA	NA	2	0,000854	2342	3,00E-04	3,00E-04	3,00E-04	2,00E-04	9,00E-04	2,00E-04	2,00E-04	6,00E-04
NA	NA	NA	2	0,000854	2342	6,00E-04	6,00E-04	6,00E-04	6,00E-04	2,00E-03	5,00E-04	5,00E-04	1,30E-03
NA	NA	NA	1	0,000427	2342	0	0	0	0	0	0	0	0
NA	NA	NA	0	0	0	0	0	0	0	0	0	0	0
NA	NA	NA	0	0	0	7,88E-05	7,87E-05	7,71E-05	8,05E-05	4,00E-04	4,49E-05	3,51E-05	3,34E-05
NA	NA	NA	0	0	0	0	0	0	0	0	0	0	0
NA	NA	NA	1	0,000427	2342	6,00E-04	6,00E-04	3,00E-04	0,001	8,00E-04	5,00E-04	5,00E-04	4,00E-04
Rare Single	1	NO	1	0,000427	2342	9,20E-05	9,19E-05	1,00E-04	8,07E-05	3,00E-04	5,53E-05	4,37E-05	1,00E-04
NA	NA	NA	3	0,001281	2342	0,0011	0,0011	0,0011	0,001	3,80E-03	9,00E-04	9,00E-04	3,10E-03
NA	NA	NA	0	0	0	3,29E-05	3,29E-05	1,29E-05	5,40E-05	6,57E-05	1,26E-05	8,00E-06	3,00E-06
NA	NA	NA	0	0	0	1,32E-05	1,31E-05	1,29E-05	1,35E-05	6,55E-05	2,19E-06	8,20E-07	.
NA	NA	NA	0	0	0	0	0	0	0	0	0	0	0
NA	NA	NA	0	0	0	1,31E-05	1,31E-05	0	2,69E-05	6,54E-05	2,18E-06	8,20E-07	.

NA	NA	NA	0	0	0	0	0	0	0	0	0	0	0
NA	NA	NA	0	0	0	1,97E-05	1,97E-05	3,85E-05	0	6,55E-05	5,24E-06	2,45E-06	.
Rare Single	1	NO	0	0	0	0	0	0	0	0	0	0	0
NA	NA	NA	0	0	0	7,22E-05	7,22E-05	2,57E-05	1,00E-04	1,50E-03	3,97E-05	3,13E-05	5,00E-04
NA	NA	NA	0	0	0	0	0	0	0	0	0	0	0
NA	NA	NA	0	0	0	0	0	0	0	0	0	0	0
NA	NA	NA	4	0,001708	2342	0,0015	0,0015	0,0016	0,0014	2,60E-03	0,0014	0,0013	2,20E-03
NA	NA	NA	0	0	0	1,00E-04	1,00E-04	1,00E-04	1,00E-04	4,00E-04	7,58E-05	6,29E-05	2,00E-04
NA	NA	NA	0	0	0	0	0	0	0	0	0	0	0
NA	NA	NA	0	0	0	1,97E-05	1,97E-05	3,86E-05	0	7,24E-05	5,24E-06	2,45E-06	1,03E-05
NA	NA	NA	1	0,000427	2342	1,00E-04	1,00E-04	1,00E-04	2,00E-04	4,00E-04	8,66E-05	7,25E-05	1,00E-04
NA	NA	NA	1	0,000427	2342	6,57E-06	6,57E-06	1,28E-05	0	1,47E-05	0	0	.
NA	NA	NA	0	0	0	6,61E-06	6,58E-06	0	1,36E-05	1,48E-05	0	0	.
Rare Single	3	NO	0	0	0	9,20E-05	9,19E-05	3,86E-05	1,00E-04	2,50E-03	5,53E-05	4,37E-05	1,10E-03
Rare Single	3	NO	1	0,000427	2342	1,00E-04	1,00E-04	1,00E-04	2,00E-04	2,70E-03	9,76E-05	8,27E-05	1,30E-03
NA	NA	NA	0	0	0	2,63E-05	2,62E-05	2,57E-05	2,68E-05	4,00E-04	8,13E-06	5,14E-06	3,03E-05
NA	NA	NA	0	0	0	8,78E-05	7,14E-05	4,65E-05	1,00E-04	2,00E-04	4,04E-05	2,84E-05	2,99E-05
NA	NA	NA	0	0	0	1,31E-05	1,31E-05	0	2,69E-05	1,00E-04	2,18E-06	8,20E-07	9,08E-06
NA	NA	NA	1	0,000427	2342	0,0013	0,0013	0,0015	0,0012	2,30E-03	0,0012	0,0011	1,90E-03
NA	NA	NA	0	0	0	6,58E-06	6,57E-06	0	1,35E-05	1,47E-05	0	0	.
NA	NA	NA	1	0,000427	2342	0,0019	0,0019	0,002	0,0018	3,10E-03	0,0017	0,0016	2,60E-03
NA	NA	NA	0	0	0	7,24E-05	7,22E-05	5,15E-05	9,42E-05	2,00E-04	3,98E-05	3,13E-05	4,24E-05
NA	NA	NA	0	0	0	0	0	0	0	0	0	0	0
NA	NA	NA	2	0,000854	2342	2,00E-04	2,00E-04	2,00E-04	2,00E-04	4,00E-04	2,00E-04	2,00E-04	3,00E-04
NA	NA	NA	0	0	0	4,60E-05	4,60E-05	7,71E-05	1,34E-05	1,00E-04	2,11E-05	1,53E-05	4,30E-05
NA	NA	NA	0	0	0	0	0	0	0	0	0	0	0
NA	NA	NA	0	0	0	1,97E-05	1,97E-05	2,57E-05	1,34E-05	4,41E-05	5,23E-06	2,45E-06	6,25E-06
NA	NA	NA	0	0	0	0	0	0	0	0	0	0	0
NA	NA	NA	0	0	0	0	0	0	0	0	0	0	0
NA	NA	NA	0	0	0	4,00E-04	4,00E-04	4,00E-04	4,00E-04	7,00E-04	3,00E-04	3,00E-04	5,00E-04
NA	NA	NA	0	0	0	6,00E-04	6,00E-04	6,00E-04	6,00E-04	1,20E-03	5,00E-04	4,00E-04	9,00E-04
NA	NA	NA	1	0,000427	2342	2,00E-04	2,00E-04	2,00E-04	2,00E-04	2,90E-03	1,00E-04	1,00E-04	1,40E-03
NA	NA	NA	0	0	0	2,00E-04	2,00E-04	2,00E-04	3,00E-04	8,00E-04	2,00E-04	1,00E-04	5,00E-04
NA	NA	NA	0	0	0	3,34E-05	3,29E-05	2,61E-05	4,11E-05	7,44E-05	1,28E-05	8,10E-06	1,88E-05
NA	NA	NA	0	0	0	1,97E-05	1,97E-05	1,29E-05	2,69E-05	2,00E-04	5,24E-06	2,46E-06	2,84E-05
NA	NA	NA	0	0	0	0	0	0	0	0	0	0	0

NA	NA	NA	5	0,002135	2342	0,0036	0,0036	0,0038	0,0034	1,27E-02	0,0034	0,0033	1,15E-02
NA	NA	NA	1	0,000427	2342	1,00E-04	1,00E-04	1,00E-04	1,00E-04	2,00E-04	7,09E-05	5,75E-05	6,95E-05
NA	NA	NA	1	0,000427	2342	1,97E-05	1,97E-05	2,57E-05	1,35E-05	4,83E-05	5,24E-06	2,45E-06	2,99E-06
NA	NA	NA	0	0	0	1,98E-05	1,97E-05	1,29E-05	2,70E-05	4,00E-04	5,26E-06	2,46E-06	2,89E-05
NA	NA	NA	0	0	0	0	0	0	0	0	0	0	0
NA	NA	NA	0	0	0	1,97E-05	1,97E-05	1,28E-05	2,69E-05	7,23E-05	5,24E-06	2,45E-06	1,03E-05
NA	NA	NA	0	0	0	1,97E-05	1,97E-05	1,29E-05	2,69E-05	7,24E-05	5,24E-06	2,45E-06	1,03E-05
NA	NA	NA	0	0	0	6,61E-06	6,60E-06	0	1,35E-05	1,48E-05	0	0	.
Rare Single	3	NO	0	0	0	1,00E-04	1,00E-04	1,00E-04	1,00E-04	2,00E-04	8,17E-05	6,72E-05	1,00E-04
NA	NA	NA	0	0	0	2,63E-05	2,63E-05	2,57E-05	2,69E-05	5,88E-05	8,15E-06	5,14E-06	1,12E-05
NA	NA	NA	0	0	0	1,31E-05	1,31E-05	0	2,69E-05	2,94E-05	2,18E-06	8,20E-07	1,83E-06
NA	NA	NA	0	0	0	0	0	0	0	0	0	0	0
Rare Single	1	NO	0	0	0	5,29E-05	5,25E-05	2,59E-05	8,12E-05	2,00E-04	2,57E-05	1,84E-05	1,86E-05
NA	NA	NA	0	0	0	0	0	0	0	0	0	0	0
NA	NA	NA	1	0,000427	2342	1,97E-05	1,97E-05	1,29E-05	2,69E-05	6,55E-05	5,24E-06	2,46E-06	.
NA	NA	NA	0	0	0	0	0	0	0	0	0	0	0
Rare Single	1	NO	0	0	0	0	0	0	0	0	0	0	0
NA	NA	NA	0	0	0	6,57E-06	6,57E-06	1,29E-05	0	1,47E-05	0	0	.
NA	NA	NA	0	0	0	0	0	0	0	0	0	0	0
NA	NA	NA	0	0	0	6,57E-06	6,57E-06	0	1,35E-05	2,41E-05	0	0	.
NA	NA	NA	0	0	0	4,60E-05	4,59E-05	1,29E-05	8,06E-05	4,00E-04	2,11E-05	1,53E-05	1,00E-04
NA	NA	NA	0	0	0	0	0	0	0	0	0	0	0
NA	NA	NA	0	0	0	1,32E-05	1,31E-05	1,29E-05	1,35E-05	2,00E-04	2,19E-06	8,20E-07	.
Syndromic	1	NO	1	0,000427	2342	1,31E-05	1,31E-05	1,28E-05	1,35E-05	2,94E-05	2,18E-06	8,20E-07	1,83E-06
NA	NA	NA	1	0,000427	2342	2,00E-04	2,00E-04	9,00E-05	3,00E-04	1,60E-03	1,00E-04	1,00E-04	1,00E-03
NA	NA	NA	0	0	0	0	0	0	0	0	0	0	0
NA	NA	NA	0	0	0	0	0	0	0	0	0	0	0
NA	NA	NA	0	0	0	0	0	0	0	0	0	0	0
NA	NA	NA	2	0,000854	2342	0,0013	0,0013	0,0012	0,0013	4,30E-03	0,0011	0,0011	3,60E-03
NA	NA	NA	0	0	0	0	0	0	0	0	0	0	0
NA	NA	NA	0	0	0	0	0	0	0	0	0	0	0
NA	NA	NA	0	0	0	0	0	0	0	0	0	0	0
NA	NA	NA	14	0,005978	2342	0,009	0,0089	0,0097	0,0082	3,12E-02	0,0086	0,0084	2,92E-02
NA	NA	NA	0	0	0	1,31E-05	1,31E-05	1,28E-05	1,35E-05	2,41E-05	2,18E-06	8,20E-07	.
NA	NA	NA	2	0,000854	2342	1,00E-04	1,00E-04	1,00E-04	2,00E-04	3,90E-03	9,15E-05	7,70E-05	2,10E-03
NA	NA	NA	0	0	0	0	0	0	0	0	0	0	0

NA	NA	NA	0	0	0	0	0	0	0	0	0	0	0
NA	NA	NA	0	0	0	0	0	0	0	0	0	0	0
NA	NA	NA	0	0	0	0	0	0	0	0	0	0	0
NA	NA	NA	0	0	0	2,63E-05	2,63E-05	2,57E-05	2,69E-05	5,88E-05	8,14E-06	5,14E-06	1,12E-05
Rare Single	2	NO	0	0	0	6,58E-06	6,57E-06	1,29E-05	0	2,41E-05	0	0	.
NA	NA	NA	0	0	0	0	0	0	0	0	0	0	0
NA	NA	NA	0	0	0	0	0	0	0	0	0	0	0
NA	NA	NA	0	0	0	0	0	0	0	0	0	0	0
NA	NA	NA	0	0	0	1,00E-04	1,00E-04	7,71E-05	2,00E-04	4,00E-04	8,17E-05	6,72E-05	2,00E-04
NA	NA	NA	0	0	0	1,97E-05	1,97E-05	2,57E-05	1,35E-05	6,55E-05	5,24E-06	2,45E-06	1,83E-06
NA	NA	NA	0	0	0	4,60E-05	4,60E-05	5,14E-05	4,04E-05	1,00E-04	2,11E-05	1,53E-05	3,34E-05
NA	NA	NA	0	0	0	0	0	0	0	0	0	0	0
NA	NA	NA	0	0	0	9,20E-05	9,19E-05	5,14E-05	1,00E-04	1,00E-04	5,53E-05	4,36E-05	6,00E-05
NA	NA	NA	0	0	0	0	0	0	0	0	0	0	0
NA	NA	NA	0	0	0	0	0	0	0	0	0	0	0
Rare Single	3	NO	0	0	0	0	0	0	0	0	0	0	0
Rare Single	1	NO	0	0	0	0	0	0	0	0	0	0	0
Rare Single	1	NO	0	0	0	0	0	0	0	0	0	0	0
NA	NA	NA	2	0,000854	2342	0,0012	0,0011	0,001	0,0013	1,20E-03	0,001	0,001	8,00E-04
Rare Single	1	NO	2	0,000854	2342	0	0	0	0	0	0	0	0
NA	NA	NA	0	0	0	9,86E-05	9,85E-05	1,00E-04	9,42E-05	1,00E-04	6,01E-05	4,88E-05	5,09E-05
NA	NA	NA	0	0	0	7,00E-04	7,00E-04	7,00E-04	7,00E-04	1,30E-03	6,00E-04	6,00E-04	1,00E-03
NA	NA	NA	1	0,000427	2342	2,00E-04	2,00E-04	2,00E-04	2,00E-04	7,00E-04	2,00E-04	1,00E-04	4,00E-04
NA	NA	NA	0	0	0	0	0	0	0	0	0	0	0
NA	NA	NA	0	0	0	2,00E-04	2,00E-04	1,00E-04	4,00E-04	7,30E-03	2,00E-04	2,00E-04	4,80E-03
NA	NA	NA	1	0,000427	2342	9,00E-04	9,00E-04	0,001	7,00E-04	3,00E-03	7,00E-04	7,00E-04	2,40E-03
Rare Single	3	NO	2	0,000854	2342	2,00E-04	2,00E-04	2,00E-04	8,06E-05	3,00E-04	1,00E-04	8,71E-05	1,00E-04
Rare Single	3	NO	0	0	0	0	0	0	0	0	0	0	0
Rare Single	3	NO	0	0	0	0	0	0	0	0	0	0	0
NA	NA	NA	0	0	0	1,31E-05	1,31E-05	1,29E-05	1,35E-05	2,41E-05	2,18E-06	8,20E-07	.
NA	NA	NA	0	0	0	0	0	0	0	0	0	0	0
NA	NA	NA	0	0	0	0	0	0	0	0	0	0	0
NA	NA	NA	0	0	0	6,57E-06	6,57E-06	1,28E-05	0	2,41E-05	0	0	.
NA	NA	NA	0	0	0	0	0	0	0	0	0	0	0
NA	NA	NA	0	0	0	2,00E-04	2,00E-04	2,00E-04	3,00E-04	7,00E-04	2,00E-04	1,00E-04	5,00E-04
NA	NA	NA	0	0	0	0	0	0	0	0	0	0	0

NA	NA	NA	0	0	0	1,31E-05	1,31E-05	1,28E-05	1,35E-05	4,83E-05	2,18E-06	8,20E-07	2,99E-06
NA	NA	NA	1	0,000427	2342	1,97E-05	1,97E-05	1,29E-05	2,69E-05	2,94E-05	5,24E-06	2,45E-06	1,83E-06
NA	NA	NA	0	0	0	1,32E-05	1,31E-05	1,29E-05	1,35E-05	2,41E-05	2,18E-06	8,20E-07	.
NA	NA	NA	0	0	0	0	6,71E-06	0	0	.	0	0	.
NA	NA	NA	0	0	0	2,00E-04	2,00E-04	2,00E-04	2,00E-04	4,00E-04	2,00E-04	1,00E-04	3,00E-04
NA	NA	NA	1	0,000427	2342	9,00E-04	9,00E-04	0,0011	6,00E-04	3,00E-03	8,00E-04	7,00E-04	2,40E-03
NA	NA	NA	0	0	0	0	0	0	0	0	0	0	0
NA	NA	NA	0	0	0	0	0	0	0	0	0	0	0
NA	NA	NA	0	0	0	0	0	0	0	0	0	0	0
NA	NA	NA	0	0	0	6,59E-06	6,57E-06	0	1,35E-05	1,47E-05	0	0	.
NA	NA	NA	0	0	0	0	0	0	0	0	0	0	0
NA	NA	NA	0	0	0	0	0	0	0	0	0	0	0
NA	NA	NA	0	0	0	0	0	0	0	0	0	0	0
NA	NA	NA	0	0	0	0	0	0	0	0	0	0	0
NA	NA	NA	1	0,000427	2342	5,00E-04	5,00E-04	4,00E-04	5,00E-04	1,70E-03	4,00E-04	4,00E-04	1,30E-03
NA	NA	NA	0	0	0	0	0	0	0	0	0	0	0
NA	NA	NA	0	0	0	0	0	0	0	0	0	0	0
NA	NA	NA	0	0	0	1,31E-05	1,31E-05	0	2,69E-05	2,94E-05	2,18E-06	8,20E-07	1,83E-06
NA	NA	NA	0	0	0	0	0	0	0	0	0	0	0
NA	NA	NA	0	0	0	0	0	0	0	0	0	0	0
Rare Single	3	NO	1	0,000427	2344	2,00E-04	2,00E-04	2,00E-04	3,00E-04	9,00E-04	2,00E-04	1,00E-04	5,00E-04
Rare Single	3	NO	2	0,000854	2342	2,00E-04	2,00E-04	2,00E-04	1,00E-04	3,00E-04	1,00E-04	9,71E-05	1,00E-04
NA	NA	NA	0	0	0	3,29E-05	3,28E-05	3,86E-05	2,69E-05	6,55E-05	1,26E-05	7,98E-06	6,25E-06
NA	NA	NA	3	0,001281	2342	1,97E-05	1,97E-05	0	4,04E-05	6,55E-05	5,24E-06	2,45E-06	.
NA	NA	NA	1	0,000427	2342	1,31E-05	1,31E-05	0	2,69E-05	1,00E-04	2,18E-06	8,20E-07	9,07E-06
NA	NA	NA	0	0	0	0	0	0	0	0	0	0	0
NA	NA	NA	0	0	0	0	0	0	0	0	0	0	0
NA	NA	NA	0	0	0	0	0	0	0	0	0	0	0
NA	NA	NA	0	0	0	0	0	0	0	0	0	0	0
NA	NA	NA	2	0,000854	2342	5,00E-04	5,00E-04	5,00E-04	4,00E-04	1,60E-03	4,00E-04	3,00E-04	1,20E-03
NA	NA	NA	3	0,001281	2342	5,91E-05	5,91E-05	7,71E-05	4,03E-05	1,00E-04	3,08E-05	2,21E-05	4,24E-05
NA	NA	NA	0	0	0	0	0	0	0	0	0	0	0
NA	NA	NA	0	0	0	4,60E-05	4,60E-05	3,86E-05	5,38E-05	9,66E-05	2,11E-05	1,53E-05	1,92E-05
NA	NA	NA	0	0	0	6,57E-06	6,57E-06	1,29E-05	0	2,41E-05	0	0	.
NA	NA	NA	0	0	0	2,63E-05	2,63E-05	2,57E-05	2,69E-05	9,65E-05	8,14E-06	5,14E-06	1,92E-05
NA	NA	NA	0	0	0	0	0	0	0	0	0	0	0

NA	NA	NA	0	0	0	0	0	0	0	0	0	0	0
Rare Single	1	NO	7	0,002989	2342	0,0024	0,0024	0,0026	0,0023	3,90E-03	0,0022	0,0021	3,30E-03
Rare Single	1	NO	0	0	0	0	0	0	0	0	0	0	0
NA	NA	NA	1	0,000427	2342	1,97E-05	1,97E-05	2,57E-05	1,35E-05	4,83E-05	5,24E-06	2,45E-06	2,99E-06
NA	NA	NA	0	0	0	6,57E-05	6,56E-05	3,86E-05	9,40E-05	2,00E-04	3,52E-05	2,62E-05	8,43E-05
NA	NA	NA	0	0	0	6,58E-06	6,57E-06	0	1,35E-05	2,42E-05	0	0	.
NA	NA	NA	0	0	0	2,07E-05	2,02E-05	4,03E-05	0	1,52E-05	5,50E-06	2,52E-06	.
NA	NA	NA	0	0	0	0	0	0	0	0	0	0	0
NA	NA	NA	0	0	0	0	0	0	0	0	0	0	0
NA	NA	NA	0	0	0	0	0	0	0	0	0	0	0
NA	NA	NA	0	0	0	6,00E-04	6,00E-04	6,00E-04	6,00E-04	7,00E-04	5,00E-04	5,00E-04	5,00E-04
NA	NA	NA	1	0,000427	2342	7,00E-04	7,00E-04	7,00E-04	7,00E-04	1,70E-03	6,00E-04	5,00E-04	1,30E-03
Rare Single	2	NO	0	0	0	8,54E-05	8,54E-05	5,14E-05	1,00E-04	3,00E-04	4,96E-05	3,96E-05	1,00E-04
Rare Single	2	NO	0	0	0	0	0	0	0	0	0	0	0
NA	NA	NA	0	0	0	0	0	0	0	0	0	0	0
NA	NA	NA	0	0	0	6,57E-06	6,57E-06	0	1,35E-05	2,00E-04	0	0	.
NA	NA	NA	1	0,000427	2342	4,60E-05	4,60E-05	3,85E-05	5,38E-05	3,00E-04	2,11E-05	1,53E-05	8,29E-05
NA	NA	NA	0	0	0	6,57E-06	6,57E-06	1,29E-05	0	1,47E-05	0	0	.
NA	NA	NA	0	0	0	0	0	0	0	0	0	0	0
NA	NA	NA	0	0	0	6,57E-06	6,57E-06	1,29E-05	0	2,00E-04	0	0	.
NA	NA	NA	0	0	0	0	0	0	0	0	0	0	0
NA	NA	NA	0	0	0	3,94E-05	3,94E-05	6,42E-05	1,35E-05	1,00E-04	1,72E-05	1,13E-05	4,30E-05
NA	NA	NA	0	0	0	3,00E-04	3,00E-04	3,00E-04	3,00E-04	7,70E-03	2,00E-04	2,00E-04	5,20E-03
NA	NA	NA	0	0	0	0	0	0	0	0	0	0	0
NA	NA	NA	1	0,000427	2342	3,00E-04	3,00E-04	3,00E-04	4,00E-04	1,10E-03	2,00E-04	2,00E-04	5,00E-04
NA	NA	NA	0	0	0	5,25E-05	5,25E-05	3,85E-05	6,71E-05	4,00E-04	2,56E-05	1,83E-05	5,28E-05
NA	NA	NA	0	0	0	2,00E-04	2,00E-04	1,00E-04	2,00E-04	2,00E-04	1,00E-04	9,70E-05	7,90E-05
NA	NA	NA	3	0,001281	2342	1,00E-04	1,00E-04	1,00E-04	1,00E-04	1,10E-03	9,15E-05	7,70E-05	6,00E-04
NA	NA	NA	1	0,000427	2342	6,57E-06	6,57E-06	1,29E-05	0	2,41E-05	0	0	.
NA	NA	NA	0	0	0	0	0	0	0	0	0	0	0
NA	NA	NA	0	0	0	1,31E-05	1,31E-05	0	2,69E-05	2,94E-05	2,18E-06	8,20E-07	1,83E-06
NA	NA	NA	0	0	0	0	0	0	0	0	0	0	0
NA	NA	NA	0	0	0	0	0	0	0	0	0	0	0
NA	NA	NA	0	0	0	0	0	0	0	0	0	0	0
NA	NA	NA	0	0	0	0	0	0	0	0	0	0	0
Rare Single	3	NO	0	0	0	6,57E-06	6,57E-06	1,29E-05	0	1,47E-05	0	0	.

Rare	Single	3	NO	1	0,000427	2342	3,00E-04	3,00E-04	3,00E-04	4,00E-04	5,00E-04	2,00E-04	2,00E-04	3,00E-04
NA	NA		NA	0	0	0	1,97E-05	1,97E-05	2,57E-05	1,34E-05	7,22E-05	5,24E-06	2,45E-06	1,03E-05
NA	NA		NA	0	0	0	0	0	0	0	0	0	0	0
NA	NA		NA	3	0,001281	2342	9,85E-05	9,85E-05	1,00E-04	5,38E-05	5,00E-04	6,01E-05	4,88E-05	2,00E-04
NA	NA		NA	1	0,000427	2342	6,57E-06	6,57E-06	0	1,35E-05	.	0	0	.
NA	NA		NA	0	0	0	0	0	0	0	0	0	0	0
NA	NA		NA	1	0,000427	2342	1,00E-04	1,00E-04	2,00E-04	8,05E-05	2,00E-04	9,14E-05	7,70E-05	1,00E-04
NA	NA		NA	0	0	0	0	0	0	0	0	0	0	0
NA	NA		NA	0	0	0	0	0	0	0	0	0	0	0
NA	NA		NA	0	0	0	2,00E-04	2,00E-04	2,00E-04	2,00E-04	4,00E-04	2,00E-04	1,00E-04	2,00E-04
Rare	Single	2	NO	0	0	0	0	0	0	0	0	0	0	0
Rare	Single	2	NO	0	0	0	0	0	0	0	0	0	0	0
NA	NA		NA	0	0	0	6,57E-06	6,57E-06	0	1,35E-05	2,42E-05	0	0	.
NA	NA		NA	0	0	0	0	0	0	0	0	0	0	0
Rare	Single	1	NO	0	0	0	7,88E-05	7,87E-05	5,14E-05	1,00E-04	8,00E-04	4,49E-05	3,51E-05	2,00E-04
NA	NA		NA	0	0	0	0	0	0	0	0	0	0	0
NA	NA		NA	0	0	0	1,97E-05	1,97E-05	2,57E-05	1,35E-05	2,94E-05	5,25E-06	2,46E-06	1,83E-06
NA	NA		NA	0	0	0	0	0	0	0	0	0	0	0
NA	NA		NA	0	0	0	6,57E-06	6,57E-06	0	1,35E-05	6,55E-05	0	0	.
NA	NA		NA	0	0	0	0	0	0	0	0	0	0	0
NA	NA		NA	0	0	0	0	0	0	0	0	0	0	0
NA	NA		NA	0	0	0	0	0	0	0	0	0	0	0
NA	NA		NA	0	0	0	0	0	0	0	0	0	0	0
Rare	Single	1	NO	0	0	0	6,57E-06	6,57E-06	1,29E-05	0	1,47E-05	0	0	.
NA	NA		NA	0	0	0	0	0	0	0	0	0	0	0
NA	NA		NA	0	0	0	0	0	0	0	0	0	0	0
NA	NA		NA	0	0	0	0	0	0	0	0	0	0	0
NA	NA		NA	0	0	0	0	0	0	0	0	0	0	0
Rare	Single	3	NO	0	0	0	0	0	0	0	0	0	0	0
NA	NA		NA	2	0,000854	2342	0	0	0	0	0	0	0	0
NA	NA		NA	0	0	0	0	0	0	0	0	0	0	0
NA	NA		NA	0	0	0	2,63E-05	2,63E-05	2,57E-05	2,69E-05	5,88E-05	8,14E-06	5,14E-06	1,12E-05
NA	NA		NA	0	0	0	0	0	0	0	0	0	0	0
NA	NA		NA	3	0,001281	2342	7,23E-05	7,22E-05	7,71E-05	6,72E-05	3,00E-04	3,97E-05	3,13E-05	1,00E-04
NA	NA		NA	0	0	0	0	0	0	0	0	0	0	0
NA	NA		NA	0	0	0	3,29E-05	3,28E-05	3,85E-05	2,69E-05	1,00E-04	1,26E-05	7,98E-06	9,07E-06

NA	NA	NA	0	0	0	1,97E-05	1,97E-05	2,57E-05	1,35E-05	4,41E-05	5,24E-06	2,46E-06	6,25E-06
NA	NA	NA	0	0	0	0	0	0	0	0	0	0	0
NA	NA	NA	0	0	0	2,00E-04	2,00E-04	3,00E-04	1,00E-04	4,00E-04	1,00E-04	1,00E-04	2,00E-04
NA	NA	NA	0	0	0	1,32E-05	1,32E-05	2,58E-05	0	2,43E-05	2,19E-06	8,20E-07	.
Rare Single	1	NO	0	0	0	0	0	0	0	0	0	0	0
NA	NA	NA	0	0	0	1,31E-05	1,31E-05	1,28E-05	1,35E-05	2,00E-04	2,18E-06	8,20E-07	.
NA	NA	NA	1	0,000427	2342	6,57E-06	6,57E-06	1,29E-05	0	6,55E-05	0	0	.
NA	NA	NA	2	0,000854	2342	8,00E-04	8,00E-04	7,00E-04	9,00E-04	1,30E-03	7,00E-04	7,00E-04	1,00E-03
NA	NA	NA	0	0	0	1,31E-05	1,31E-05	0	2,69E-05	2,41E-05	2,18E-06	8,20E-07	.
NA	NA	NA	0	0	0	5,25E-05	5,25E-05	2,57E-05	8,06E-05	1,00E-04	2,56E-05	1,83E-05	9,06E-06
NA	NA	NA	0	0	0	0	0	0	0	0	0	0	0
NA	NA	NA	0	0	0	0	0	0	0	0	0	0	0
NA	NA	NA	0	0	0	0	0	0	0	0	0	0	0
NA	NA	NA	0	0	0	6,57E-06	6,57E-06	1,29E-05	0	1,47E-05	0	0	.
NA	NA	NA	0	0	0	0	0	0	0	0	0	0	0
NA	NA	NA	0	0	0	1,31E-05	1,31E-05	1,28E-05	1,35E-05	2,41E-05	2,18E-06	8,20E-07	.
NA	NA	NA	0	0	0	1,32E-05	1,31E-05	2,57E-05	0	2,42E-05	2,19E-06	8,20E-07	.
NA	NA	NA	0	0	0	0	0	0	0	0	0	0	0
NA	NA	NA	0	0	0	0	0	0	0	0	0	0	0
NA	NA	NA	1	0,000427	2342	1,00E-04	1,00E-04	1,00E-04	1,00E-04	4,00E-04	8,16E-05	6,72E-05	2,00E-04
NA	NA	NA	0	0	0	0	0	0	0	0	0	0	0
NA	NA	NA	0	0	0	4,60E-05	4,59E-05	5,14E-05	4,03E-05	1,40E-03	2,11E-05	1,53E-05	5,00E-04
Rare Single	2	NO	0	0	0	0	0	0	0	0	0	0	0
Rare Single	2	NO	0	0	0	6,57E-06	6,57E-06	1,29E-05	0	6,54E-05	0	0	.
NA	NA	NA	0	0	0	0	0	0	0	0	0	0	0
NA	NA	NA	0	0	0	2,00E-04	2,00E-04	1,00E-04	3,00E-04	7,00E-04	1,00E-04	1,00E-04	4,00E-04
NA	NA	NA	1	0,000427	2342	3,00E-04	3,00E-04	3,00E-04	2,00E-04	9,00E-04	2,00E-04	2,00E-04	6,00E-04
NA	NA	NA	0	0	0	1,31E-05	1,31E-05	0	2,69E-05	6,54E-05	2,18E-06	8,20E-07	.
NA	NA	NA	1	0,000427	2342	0	0	0	0	0	0	0	0
NA	NA	NA	0	0	0	0	0	0	0	0	0	0	0
NA	NA	NA	0	0	0	1,32E-05	1,31E-05	1,29E-05	1,35E-05	1,47E-05	2,19E-06	8,20E-07	.
NA	NA	NA	0	0	0	6,00E-04	6,00E-04	7,00E-04	5,00E-04	2,10E-03	5,00E-04	5,00E-04	1,60E-03
NA	NA	NA	1	0,000427	2342	7,88E-05	7,87E-05	6,42E-05	9,40E-05	8,00E-04	4,49E-05	3,51E-05	2,00E-04
NA	NA	NA	0	0	0	2,63E-05	2,63E-05	3,86E-05	1,35E-05	2,00E-04	8,14E-06	5,14E-06	9,08E-06
NA	NA	NA	0	0	0	0	0	0	0	0	0	0	0
NA	NA	NA	2	0,000854	2342	1,97E-05	1,97E-05	2,57E-05	1,35E-05	7,25E-05	5,25E-06	2,46E-06	1,03E-05

NA	NA	NA	0	0	0	0	0	0	0	0	0	0	0
NA	NA	NA	0	0	0	6,57E-06	1,31E-05	0	1,35E-05	.	0	0	.
Rare Single	2	NO	0	0	0	1,00E-04	1,00E-04	1,00E-04	1,00E-04	6,00E-04	9,15E-05	7,71E-05	2,00E-04
NA	NA	NA	0	0	0	0	0	0	0	0	0	0	0
NA	NA	NA	0	0	0	6,58E-06	6,57E-06	0	1,35E-05	2,00E-04	0	0	.
Rare Single	3	NO	1	0,000427	2342	9,20E-05	9,20E-05	1,00E-04	6,73E-05	2,00E-04	5,53E-05	4,37E-05	7,89E-05
NA	NA	NA	0	0	0	0	0	0	0	0	0	0	0
NA	NA	NA	4	0,001707	2343	0,0013	0,0014	0,0012	0,0014	5,20E-03	0,0012	0,0011	3,20E-03
NA	NA	NA	1	0,000427	2342	1,32E-05	1,31E-05	1,29E-05	1,35E-05	4,00E-04	2,19E-06	8,20E-07	2,86E-05
NA	NA	NA	1	0,000427	2342	0	0	0	0	0	0	0	0
Rare Single	2	NO	0	0	0	0	0	0	0	0	0	0	0
NA	NA	NA	0	0	0	0	0	0	0	0	0	0	0
NA	NA	NA	0	0	0	6,57E-06	6,57E-06	0	1,35E-05	6,55E-05	0	0	.
NA	NA	NA	0	0	0	0	0	0	0	0	0	0	0
NA	NA	NA	0	0	0	6,58E-06	6,57E-06	0	1,35E-05	1,47E-05	0	0	.
NA	NA	NA	0	0	0	4,60E-05	4,60E-05	3,86E-05	5,38E-05	3,00E-04	2,11E-05	1,53E-05	8,29E-05
NA	NA	NA	0	0	0	5,26E-05	5,25E-05	5,14E-05	5,38E-05	7,35E-05	2,56E-05	1,83E-05	1,86E-05
NA	NA	NA	0	0	0	0	0	0	0	0	0	0	0
NA	NA	NA	0	0	0	6,61E-06	6,58E-06	1,29E-05	0	2,00E-04	0	0	.
Rare Single	3	NO	0	0	0	0	0	0	0	0	0	0	0
NA	NA	NA	0	0	0	2,63E-05	2,63E-05	5,15E-05	0	3,00E-04	8,15E-06	5,15E-06	5,39E-05
NA	NA	NA	0	0	0	0	0	0	0	0	0	0	0
NA	NA	NA	0	0	0	6,57E-06	6,57E-06	1,29E-05	0	2,00E-04	0	0	.
NA	NA	NA	0	0	0	1,31E-05	1,31E-05	1,28E-05	1,35E-05	4,83E-05	2,18E-06	8,20E-07	2,99E-06
NA	NA	NA	1	0,000427	2342	3,29E-05	3,28E-05	3,86E-05	2,69E-05	6,56E-05	1,26E-05	7,99E-06	.
NA	NA	NA	0	0	0	0	0	0	0	0	0	0	0
NA	NA	NA	0	0	0	0	0	0	0	0	0	0	0
NA	NA	NA	0	0	0	0	0	0	0	0	0	0	0
NA	NA	NA	0	0	0	7,23E-05	7,22E-05	6,43E-05	8,07E-05	1,00E-03	3,97E-05	3,13E-05	2,00E-04
NA	NA	NA	0	0	0	0	0	0	0	0	0	0	0
NA	NA	NA	0	0	0	4,00E-04	4,00E-04	3,00E-04	5,00E-04	1,00E-03	3,00E-04	3,00E-04	5,00E-04
NA	NA	NA	0	0	0	0	0	0	0	0	0	0	0
NA	NA	NA	2	0,000854	2342	3,94E-05	3,94E-05	3,86E-05	4,03E-05	7,22E-05	1,72E-05	1,13E-05	1,03E-05
NA	NA	NA	0	0	0	0	0	0	0	0	0	0	0
Syndromic	2	NO	0	0	0	6,57E-06	6,57E-06	0	1,35E-05	2,41E-05	0	0	.
NA	NA	NA	0	0	0	1,00E-04	1,00E-04	1,00E-04	1,00E-04	5,00E-04	6,51E-05	5,32E-05	2,00E-04

Rare Single		1	NO	1	0,000427	2342	1,00E-04	1,00E-04	2,00E-04	8,24E-05	4,00E-04	7,72E-05	6,40E-05	2,00E-04
Rare Single		3	NO	0	0	0	1,97E-05	1,97E-05	3,85E-05	0	2,94E-05	5,24E-06	2,45E-06	1,83E-06
Rare Single		3	NO	0	0	0	0	0	0	0	0	0	0	0
NA	NA		NA	0	0	0	0	0	0	0	0	0	0	0
NA	NA		NA	0	0	0	0	0	0	0	0	0	0	0
Rare Single		1	NO	0	0	0	0	0	0	0	0	0	0	0
Genetic As		3	NO	1	0,000427	2342	4,60E-05	4,60E-05	3,85E-05	5,38E-05	4,00E-04	2,11E-05	1,53E-05	1,00E-04
Genetic As		3	NO	0	0	0	0	0	0	0	0	0	0	0
NA	NA		NA	0	0	0	0	0	0	0	0	0	0	0
NA	NA		NA	0	0	0	2,00E-04	2,00E-04	2,00E-04	1,00E-04	3,00E-04	1,00E-04	8,71E-05	1,00E-04
Rare Single		1	NO	0	0	0	3,00E-04	3,00E-04	3,00E-04	2,00E-04	5,00E-04	2,00E-04	2,00E-04	3,00E-04
NA	NA		NA	0	0	0	0	0	0	0	0	0	0	0
NA	NA		NA	0	0	0	6,57E-06	6,57E-06	1,29E-05	0	1,47E-05	0	0	.
NA	NA		NA	0	0	0	0	0	0	0	0	0	0	0
NA	NA		NA	1	0,000427	2342	7,00E-04	7,00E-04	7,00E-04	7,00E-04	1,30E-03	6,00E-04	6,00E-04	1,00E-03
NA	NA		NA	0	0	0	0	0	0	0	0	0	0	0
NA	NA		NA	2	0,000854	2342	3,00E-04	3,00E-04	3,00E-04	3,00E-04	1,20E-03	3,00E-04	2,00E-04	8,00E-04
NA	NA		NA	0	0	0	6,57E-06	6,57E-06	0	1,35E-05	1,47E-05	0	0	.
NA	NA		NA	0	0	0	0	0	0	0	0	0	0	0
NA	NA		NA	0	0	0	6,58E-06	6,57E-06	0	1,35E-05	2,00E-04	0	0	.
NA	NA		NA	2	0,000854	2342	1,97E-05	1,97E-05	2,57E-05	1,35E-05	4,41E-05	5,24E-06	2,45E-06	6,25E-06
NA	NA		NA	0	0	0	0	0	0	0	0	0	0	0
NA	NA		NA	0	0	0	6,57E-06	6,57E-06	1,29E-05	0	1,47E-05	0	0	.
NA	NA		NA	0	0	0	0	0	0	0	0	0	0	0
NA	NA		NA	0	0	0	0	0	0	0	0	0	0	0
NA	NA		NA	0	0	0	0	0	0	0	0	0	0	0
NA	NA		NA	3	0,001281	2342	0,0012	0,0012	0,0013	0,0011	3,50E-03	0,001	0,001	2,80E-03
NA	NA		NA	6	0,002562	2342	2,00E-04	2,00E-04	3,00E-04	2,00E-04	4,00E-04	2,00E-04	2,00E-04	2,00E-04
NA	NA		NA	0	0	0	6,57E-06	6,57E-06	1,28E-05	0	2,00E-04	0	0	.
NA	NA		NA	0	0	0	0	0	0	0	0	0	0	0
NA	NA		NA	0	0	0	0	0	0	0	0	0	0	0
NA	NA		NA	0	0	0	0	0	0	0	0	0	0	0
NA	NA		NA	0	0	0	6,57E-06	6,57E-06	1,29E-05	0	1,47E-05	0	0	.
NA	NA		NA	0	0	0	1,00E-04	1,00E-04	1,00E-04	2,00E-04	6,00E-04	9,14E-05	7,70E-05	1,00E-04
Rare Single		2	NO	1	0,000427	2342	0,0012	0,0012	0,0013	0,001	2,00E-03	0,001	0,001	1,60E-03
NA	NA		NA	0	0	0	2,63E-05	2,63E-05	1,29E-05	4,04E-05	6,54E-05	8,14E-06	5,14E-06	2,99E-06

NA	NA	NA	4	0,001708	2342	7,23E-05	7,22E-05	8,99E-05	5,38E-05	1,00E-04	3,97E-05	3,13E-05	5,09E-05
NA	NA	NA	0	0	0	0	0	0	0	0	0	0	0
NA	NA	NA	0	0	0	2,00E-04	2,00E-04	2,00E-04	3,00E-04	3,00E-04	2,00E-04	1,00E-04	2,00E-04
Rare Single	3	NO	0	0	0	0	0	0	0	0	0	0	0
Rare Single	3	NO	2	0,000854	2342	2,00E-04	2,00E-04	3,00E-04	1,00E-04	4,00E-04	2,00E-04	1,00E-04	2,00E-04
Rare Single	1	NO	2	0,000854	2342	4,60E-05	4,60E-05	5,14E-05	4,04E-05	8,82E-05	2,11E-05	1,53E-05	2,57E-05
Rare Single	3	NO	0	0	0	0	0	0	0	0	0	0	0
NA	NA	NA	0	0	0	2,63E-05	2,63E-05	2,57E-05	2,69E-05	5,88E-05	8,14E-06	5,14E-06	1,12E-05
NA	NA	NA	0	0	0	0	0	0	0	0	0	0	0
NA	NA	NA	0	0	0	0	0	0	0	0	0	0	0
NA	NA	NA	2	0,000854	2342	8,00E-04	8,00E-04	9,00E-04	7,00E-04	2,70E-03	7,00E-04	6,00E-04	2,20E-03
NA	NA	NA	0	0	0	6,57E-06	6,57E-06	0	1,35E-05	6,54E-05	0	0	.
NA	NA	NA	0	0	0	2,00E-04	2,00E-04	2,00E-04	2,00E-04	4,00E-04	2,00E-04	1,00E-04	3,00E-04
NA	NA	NA	1	0,000427	2342	4,60E-05	4,60E-05	5,14E-05	4,03E-05	1,00E-04	2,11E-05	1,53E-05	3,34E-05
NA	NA	NA	0	0	0	0	0	0	0	0	0	0	0
Rare Single	2	NO	0	0	0	5,26E-05	5,25E-05	6,42E-05	4,03E-05	1,00E-04	2,56E-05	1,83E-05	4,30E-05
Rare Single	2	NO	0	0	0	0,0025	0,0025	0,0028	0,0022	4,40E-03	0,0023	0,0022	3,80E-03
Rare Single	2	NO	0	0	0	1,97E-05	1,97E-05	1,29E-05	2,69E-05	4,41E-05	5,24E-06	2,45E-06	6,25E-06
Rare Single	2	NO	0	0	0	1,31E-05	1,97E-05	1,29E-05	1,34E-05	4,00E-04	2,18E-06	8,20E-07	3,03E-05
Rare Single	1	NO	2	0,000854	2342	0	0	0	0	0	0	0	0
Rare Single	1	NO	0	0	0	0	0	0	0	0	0	0	0
Rare Single	1	NO	0	0	0	0	0	0	0	0	0	0	0
NA	NA	NA	0	0	0	0	0	0	0	0	0	0	0
Rare Single	3	NO	0	0	0	0	0	0	0	0	0	0	0
Rare Single	3	NO	1	0,000427	2340	0,0011	0,0012	0,0011	0,001	3,90E-03	9,00E-04	9,00E-04	3,10E-03
NA	NA	NA	0	0	0	3,60E-05	3,49E-05	5,15E-05	0	7,56E-05	1,14E-05	6,66E-06	1,49E-05
Rare Single	NA	YES	0	0	0	0	0	0	0	0	0	0	0
Rare Single	1	NO	1	0,000427	2340	1,00E-04	2,00E-04	9,16E-05	2,00E-04	.	6,58E-05	5,10E-05	.
Rare Single	NA	YES	0	0	0	5,43E-05	5,23E-05	6,43E-05	3,06E-05	2,00E-04	2,35E-05	1,58E-05	5,84E-05
Rare Single	NA	YES	1	0,000427	2342	6,00E-04	7,00E-04	6,00E-04	6,00E-04	1,80E-03	5,00E-04	5,00E-04	1,00E-03
Rare Single	1	YES	0	0	0	0	0	0	0	0	0	0	0
Rare Single	1	NO	0	0	0	0	0	0	0	0	0	0	0
Rare Single	2	NO	0	0	0	0	0	0	0	0	0	0	0
Rare Single	2	NO	4	0,001708	2342	0,0027	0,0028	0,0029	0,0023	4,70E-03	0,0025	0,0024	4,10E-03
NA	NA	NA	0	0	0	5,41E-05	5,22E-05	5,14E-05	6,03E-05	1,00E-04	2,34E-05	1,57E-05	3,36E-05
NA	NA	NA	2	0,000854	2342	4,50E-05	4,35E-05	6,43E-05	0	9,54E-05	1,71E-05	1,06E-05	8,16E-06

Rare Single	1	NO	0	0	0	0	0	0	0	0	0	0	0
NA	NA	NA	0	0	0	0,0012	0,0012	9,00E-04	0,0018	1,10E-03	0,001	0,001	8,00E-04
NA	NA	NA	0	0	0	0,0011	0,0011	9,00E-04	0,0016	1,00E-03	0,001	9,00E-04	7,00E-04
NA	NA	NA	1	0,000427	2342	2,68E-05	3,48E-05	1,29E-05	5,83E-05	1,88E-05	7,11E-06	2,97E-06	.
NA	NA	NA	0	0	0	2,00E-04	2,00E-04	2,00E-04	8,74E-05	3,00E-04	1,00E-04	9,18E-05	9,40E-05
NA	NA	NA	0	0	0	4,45E-05	4,35E-05	3,85E-05	5,81E-05	6,47E-05	1,70E-05	1,05E-05	8,15E-06
NA	NA	NA	0	0	0	0	0	0	0	0	0	0	0
NA	NA	NA	0	0	0	7,00E-04	7,00E-04	7,00E-04	6,00E-04	2,20E-03	5,00E-04	5,00E-04	1,70E-03
Rare Single	NA	YES	0	0	0	0	0	0	0	0	0	0	0
NA	NA	NA	0	0	0	1,00E-04	1,00E-04	2,00E-04	1,00E-04	4,00E-04	8,88E-05	7,18E-05	7,63E-05
NA	NA	NA	0	0	0	8,89E-06	8,70E-06	0	2,89E-05	3,23E-05	0	0	.
NA	NA	NA	3	0,001285	2334	5,25E-05	1,00E-04	7,34E-05	0	3,00E-04	2,03E-05	1,31E-05	4,83E-06
NA	NA	NA	0	0	0	1,90E-05	2,73E-05	2,69E-05	0	4,01E-05	3,15E-06	1,18E-06	2,49E-06
NA	NA	NA	0	0	0	2,68E-05	2,61E-05	2,57E-05	2,93E-05	2,00E-04	7,12E-06	2,98E-06	1,37E-05
NA	NA	NA	0	0	0	0	0	0	0	0	0	0	0
NA	NA	NA	0	0	0	0	0	0	0	0	0	0	0
NA	NA	NA	0	0	0	9,00E-04	9,00E-04	0,001	6,00E-04	1,50E-03	7,00E-04	7,00E-04	1,20E-03
NA	NA	NA	0	0	0	4,00E-04	4,00E-04	4,00E-04	3,00E-04	6,00E-04	3,00E-04	2,00E-04	4,00E-04
NA	NA	NA	0	0	0	0,001	0,001	0,001	0,001	1,50E-03	8,00E-04	8,00E-04	1,10E-03
NA	NA	NA	1	0,000427	2342	1,78E-05	2,61E-05	2,57E-05	0	9,41E-05	2,96E-06	1,11E-06	.
Rare Single	NA	YES	0	0	0	8,00E-04	9,00E-04	8,00E-04	8,00E-04	2,90E-03	7,00E-04	6,00E-04	2,20E-03
NA	NA	NA	0	0	0	0	0	0	0	0	0	0	0
NA	NA	NA	0	0	0	9,06E-06	8,72E-06	1,29E-05	0	1,89E-05	0	0	.
Rare Single	1	NO	0	0	0	0	0	0	0	0	0	0	0
Rare Single	1	NO	0	0	0	0	0	0	0	0	0	0	0
Rare Single	3	NO	0	0	0	0	0	0	0	0	0	0	0
NA	NA	NA	0	0	0	0	0	0	0	0	0	0	0
NA	NA	NA	0	0	0	0	0	0	0	0	0	0	0
NA	NA	NA	0	0	0	0	0	0	0	0	0	0	0
NA	NA	NA	0	0	0	2,98E-05	2,71E-05	NA	2,98E-05	1,00E-04	0	0	.

gnomad40	gnomad40	gnomad40	gnomad40	gnomad40	gnomad40	gnomad40	gnomad40	gnomad40	gnomad40	gnomAD3.	gnomAD3.	gnomAD3.	gnomAD_
2,00E-04	0	6,54E-05	0	4,00E-04	9,42E-05	0	2,00E-04	0	2,00E-04	26	0,000171	152204	2,00E-04
0	0	0	0	2,00E-04	0	0	0	0	0	1	6,57E-06	152170	4,07E-06
0	0	0	0	0	0	0	0	0	0	0	0	0	0
7,23E-05	0	0	0	0	0	0	0	0	0	3	1,97E-05	152232	8,36E-06
0	0	0	0	0	0	0	0	0	0	0	0	0	0
5,00E-04	0	0	0	0	0	0	0	0	0	20	0,000131	152154	2,48E-05
0	0	0	0	0	0	0	0	0	0	0	0	0	0
0	0	0	0	0	0	0	0	0	0	0	0	0	0
0	0	0	0	0	0	0	0	0	0	0	0	0	0
0	0	3,00E-04	0	0	0	0	0	0	0	4	2,64E-05	151776	8,13E-06
0	0	0	0	0	0	0	0	0	0	0	0	0	0
1,00E-04	0	4,00E-04	0	0	0	0,0107	4,00E-04	0	7,00E-04	45	0,000309	145620	3,00E-04
9,63E-05	0	2,00E-04	0	0	0	0	3,00E-04	0	6,00E-04	30	0,000197	152104	9,83E-05
7,24E-05	0	2,00E-04	0	0	0	0	2,00E-04	0	0	20	0,000132	151826	5,59E-05
0	0	0	0	0	0	0	0	0	0	0	0	0	0
0	0	0	0	0	0	0	0	0	0	0	0	0	1,22E-05
4,82E-05	0	0	0	0	0	0	2,94E-05	5,00E-04	0	5	3,28E-05	152210	3,32E-05
0	0	0	0	0	9,41E-05	0,0034	1,47E-05	0	2,00E-04	4	2,63E-05	152204	2,45E-05
2,42E-05	0	0	0	0	0	0	0	0	0	1	6,58E-06	151916	8,21E-06
0	0	0	0	0	0	0	0	0	0	0	0	0	0
0	0	0	0	0	0	0	4,41E-05	0	0	3	1,97E-05	152154	1,22E-05
0	0	0	0	0	0	0	0	0	0	0	0	0	0
0	0	0	0	0	0	0	2,94E-05	0	0	2	1,31E-05	152242	1,22E-05
0,0014	0	0	0	0	0	0	0	0	0	50	0,000329	152114	1,00E-04
0	0	0	0	0	0	0	0	0	0	0	0	0	0
0	0	0	0	0	0	0	0	0	0	0	0	0	0
0	0	0	0	0	0	0	1,47E-05	0	0	1	6,57E-06	152202	7,29E-05
0	0	0	0	0	0	0	0	0	0	0	0	0	0
0	0	0	0	0	0	0	1,00E-04	0	0	7	4,60E-05	152234	9,08E-05
0	0	0	0	2,00E-04	0	0	0	0	0	1	6,57E-06	152194	8,22E-06
0	0	0	0	0	0	0	0	0	0	0	0	0	0
0	0	0	0	0	0	0	0	0	0	0	0	0	0
0	0	0	0	0	0	0	0	0	0	0	0	0	4,06E-06
1,00E-04	0	0	0	0	0	0	0	0	0	5	3,29E-05	152154	2,45E-05

0	0	0	0	0	0	0	0	0	0	0	0	0	4,07E-06
0	0	0	0	0	0	0	0	0	0	0	0	0	0
0	0	0	0	0	0	0	0	0	0	0	0	0	0
0	0	0	0	0	0	0	1,47E-05	0	2,00E-04	2	1,31E-05	152232	2,44E-05
2,41E-05	0	0	0	0	0	0	1,47E-05	0	0	2	1,31E-05	152184	1,22E-05
0	0	0	0	0	0	0	0	0	0	0	0	0	4,06E-06
0	0	0	0	0	0	0	0	0	0	0	0	0	0
0	0	0	0	0	0	0	1,47E-05	0	0	1	6,57E-06	152208	1,22E-05
0	0	0	0	0	0	0	0	0	0	0	0	0	0
0	0	0	0	0	0	0	0	0	0	0	0	0	0
0	0	6,55E-05	0	0	0	0	1,47E-05	0	0	2	1,32E-05	152068	8,41E-06
0	0	0	0	0	0	0	2,94E-05	0	0	2	1,31E-05	152194	2,10E-05
0	0	0	0	0	0	0	0	0	0	0	0	0	0
0	0	0	0	0	0	0	0	0	0	0	0	0	0
0	0	0	0	0	0	0	0	0	0	0	0	0	0
2,42E-05	0	0	0	0	0	0	2,94E-05	0	0	3	1,97E-05	152096	6,38E-06
0	0	0	0	0	0	0	1,48E-05	0	0	1	6,68E-06	149682	8,37E-06
0	0	0	0	0	0	0	2,94E-05	0	0	2	1,31E-05	152172	0
0	0	0	0	0	0	0	0	0	0	0	0	0	0
0	0	0	0	0	0	0	0	0	0	0	0	0	0
0	0	0	0	0	0	0	0	0	0	0	0	0	0
2,52E-05	0	6,76E-05	0	0	0	0	1,51E-05	0	0	0	0	0	0
0	0	0	0	0	0	0	0	0	0	0	0	0	0
0	0	0	0	0	0	0	1,47E-05	0	0	1	6,57E-06	152130	1,22E-05
0	0	0	0	0	0	0	1,47E-05	0	0	1	6,57E-06	152248	4,07E-06
0	0	0	0	0	0	0	0	0	0	0	0	0	0
0	0	0	0	0	0	0	0	0	0	0	0	0	0
9,63E-05	0	6,54E-05	0	0	9,42E-05	0	6,00E-04	0	0,001	53	0,000348	152164	3,00E-04
0	0	0	0	0	0	0	0	0	0	0	0	0	0
2,42E-05	0	6,55E-05	0	0	0	0	0	0	0	2	1,32E-05	152070	3,25E-05
9,67E-05	0	0	0	0	0	0	0	0	0	4	2,63E-05	151902	2,84E-05
0	0	0	0	0	0	0	0	0	0	0	0	0	0
0	0	0	0	0	0	0	0	0	0	0	0	0	0
0	0	0	0	4,00E-04	0	0	2,94E-05	0	0	4	2,63E-05	152152	6,90E-05
0	0	0	0	0	0	0	2,94E-05	0	0	2	1,31E-05	152192	1,23E-05
3,00E-04	0	6,55E-05	0	2,00E-04	9,42E-05	0	8,00E-04	5,00E-04	8,00E-04	75	0,000493	152078	9,00E-04

2,44E-05	0	0	0	0	0	0	0	0	0	1	6,63E-06	150892	4,15E-06
0	0	1,00E-04	0	0	0	0	1,47E-05	0	0	3	1,97E-05	152246	4,06E-06
0	0	0	0	0	0	0	1,47E-05	0	0	1	6,57E-06	152118	1,22E-05
0	0	0	0	0	0	0	0	0	0	0	0	0	4,50E-06
2,41E-05	0	0	0	0	0	0	0	0	0	1	6,57E-06	152222	0
1,00E-04	0	0,0011	0	0	2,00E-04	0	0,0011	0,0014	0	101	0,000666	151688	6,00E-04
2,41E-05	0	3,00E-04	0	0	0	0	1,47E-05	0	0	6	3,95E-05	151916	1,24E-05
0	0	0	0	0	0	0	3,00E-04	0	0	18	0,000118	151964	1,76E-05
0	0	0	0	0	0	0	1,47E-05	0	0	1	6,58E-06	152052	2,03E-05
5,00E-04	0	0	0	0	0	0	1,00E-04	5,00E-04	2,00E-04	31	0,000206	150354	1,00E-04
0	0	0	0	0	0	0	0	0	0	0	0	0	0
2,41E-05	0	6,55E-05	0	0	0	0	2,00E-04	0	0	15	9,86E-05	152202	1,00E-04
0	0	2,00E-04	0	0	0	0	4,41E-05	5,00E-04	0	7	4,60E-05	152224	5,29E-05
0	0	0	0	0	0	0	1,47E-05	0	0	1	6,57E-06	152102	4,16E-06
0	0	0	0	0	0	0	0	5,00E-04	0	1	6,58E-06	152072	4,11E-06
0	0	0	0	0	0	0	0	0	0	0	0	0	0
0	0	1,00E-04	0	0	0	0	2,94E-05	0	0	4	2,63E-05	152038	8,23E-06
0	0	0	0	0	0	0	0	0	0	0	0	0	0
0	0	0	0	0	0	0	4,41E-05	5,00E-04	0	4	2,63E-05	152126	3,26E-05
0	0	0	0	0	0	0	0	0	0	0	0	0	0
0	0	0	0	0	0	0	0	0	0	0	0	0	4,09E-06
0	0	0	0	0	0	0	0	0	0	0	0	0	0
0	0	0	0	0	0	0	0	0	0	0	0	0	0
0	0	0	0	0	0	0	1,47E-05	0	0	1	6,57E-06	152190	4,07E-06
0	0	2,00E-04	0	0	0	0	0	5,00E-04	0	4	2,63E-05	152142	4,13E-06
0	0	0	0	0	0	0	0	0	0	0	0	0	0
0	0	0	0	0	0	0	0	0	0	0	0	0	0
0	0	0	6,00E-04	0	0	0	0	5,00E-04	0	3	1,97E-05	152110	3,47E-05
0	0	0	0	0	0	0	0	0	0	0	0	0	0
0	0	0	0	0	0	0	1,47E-05	5,00E-04	0	2	1,32E-05	152070	1,00E-04
1,00E-04	0	6,54E-05	0	0	0	0	5,88E-05	5,00E-04	0	12	7,89E-05	152074	9,97E-05
0,0039	0	8,00E-04	0	0	0	0	2,94E-05	5,00E-04	0	176	0,001156	152232	2,00E-04
0	0	0	0	0	0	0	1,47E-05	0	0	1	6,57E-06	152100	4,14E-06
0	0	0	0	0	0	0	0	0	0	0	0	0	0
2,71E-05	0	2,00E-04	0	0	0	0	0	0	3,00E-04	4	2,91E-05	137692	0
0	0	0	0	0	0	0	0	0	0	0	0	0	2,12E-05

0	0	0	0	0	0	0	0	0	0	0	0	0	0
9,00E-04	0,0077	3,00E-04	3,00E-04	0	9,00E-04	0	0,0027	9,00E-04	0,0012	249	0,001637	152134	0,0022
9,68E-05	0	2,00E-04	0	2,00E-04	2,00E-04	0	1,00E-04	0	6,00E-04	20	0,000132	151484	2,00E-04
2,41E-05	0	4,00E-04	0	0	0	0	2,00E-04	5,00E-04	0	22	0,000145	152172	1,00E-04
0	0	0	0	0	0	0	0	0	0	0	0	0	0
2,42E-05	0	0	0	0	0	0	0	0	0	1	6,57E-06	152110	8,12E-06
2,41E-05	0	6,54E-05	0	2,00E-04	0	0	2,94E-05	0	0	5	3,28E-05	152226	2,03E-05
2,41E-05	0	0	0	0	0	0	0	0	0	1	6,57E-06	152144	0
0	0	0	0	0	0	0	0	0	0	0	0	0	0
2,00E-04	0	0	0	0	0	0	2,94E-05	0	0	10	6,58E-05	152070	5,33E-05
0	0	0	0	0	0	0	1,47E-05	0	0	1	6,58E-06	151950	1,63E-05
0	0	0	0	0	0	0	4,41E-05	0	0	3	1,97E-05	152188	4,06E-06
2,41E-05	0	0	0	0,0062	0	0	0	5,00E-04	0	34	0,000223	152166	5,00E-04
0	0	0	0	0	0	0	0	0	0	0	0	0	0
0	0	0	0	0	0	0	0	0	0	0	0	0	0
9,66E-05	0	6,55E-05	0	0	0	0	1,47E-05	0	0	6	3,94E-05	152118	1,22E-05
2,42E-05	0	0	0	0	0	0	0	0	0	1	6,58E-06	152090	4,06E-06
0	0	0	0	0	0	0	0	0	0	0	0	0	0
0	0	0	0	0	0	0	0	0	0	0	0	0	0
8,00E-04	0,0022	0,001	0	0	0	0	0,0032	0,0019	8,00E-04	275	0,001808	152064	0,0017
0	0	0	0	0	0	0	0	0	0	0	0	0	0
0,003	0	5,00E-04	0	0	0	0	0	0,0014	0	133	0,000874	152220	3,00E-04
0	0	0	0	0	0	0	0	0	0	0	0	0	0
0	0	0	0	0	0	0	0	0	0	0	0	0	0
2,41E-05	0	0	0	0	9,45E-05	0	2,94E-05	0	4,00E-04	6	3,94E-05	152118	8,13E-05
2,42E-05	0	0	0	0	0	0	1,47E-05	0	0	2	1,32E-05	152068	8,13E-06
2,41E-05	0	6,56E-05	0	0	0	0	1,47E-05	5,00E-04	0	4	2,63E-05	152170	2,35E-05
4,83E-05	0	0	0	0	0	0	1,00E-04	0	0	12	7,89E-05	152146	6,54E-05
0	0	0	0	0,001	0	0	0	0	0	5	3,29E-05	152206	4,87E-05
0	0	0	0	0	0	0	0	0	0	0	0	0	0
0	0	2,00E-04	0	0	0	0	2,94E-05	0	0	5	3,29E-05	151878	2,85E-05
0	0	0	0	0	0	0	0	0	0	0	0	0	0
0	0	0	0	0	0	0	0	0	0	0	0	0	0
4,83E-05	0	0	0	0	0	0	0	0	0	2	1,31E-05	152148	0
0	0	0	0	0	0	0	0	0	0	0	0	0	0
1,00E-04	0	0	0	0	0	0	0	0	0	6	3,94E-05	152144	1,63E-05

2,41E-05	0	0	0	0	0	0	0	0	2,00E-04	2	1,31E-05	152226	0
0	0	0	0	0	0	0	1,47E-05	0	0	1	6,57E-06	152164	0
0	0	0	0	0	0	0	0	0	0	0	0	0	0
7,23E-05	0	0	0	0	0	0	0	0	0	3	1,97E-05	152260	8,13E-06
7,23E-05	0	0	0	0	0	0	0	0	0,0098	50	0,000329	152022	0,0012
0	0	0	0	0	0	0	0	0	0	0	0	0	4,16E-06
0	0	0	0	0	0	0	0	0	0	0	0	0	0
0	0	3,00E-04	0	2,00E-04	0	0	1,47E-05	0	0	7	4,60E-05	152146	4,06E-05
4,82E-05	0	0,001	0	0	0	0	4,00E-04	0	0	43	0,000283	152116	3,00E-04
2,00E-04	0	0,0014	0	0	0	0	5,88E-05	0	2,00E-04	34	0,000224	152096	2,00E-04
2,41E-05	0	0	0	0	0	0	2,94E-05	0	0	3	1,97E-05	152092	1,63E-05
0	0	0	0	0	0	0	0	0	0	0	0	0	0
0	0	1,00E-04	0	0	0	0	8,82E-05	0	0	8	5,26E-05	152168	4,23E-06
9,63E-05	0	3,00E-04	0	0	3,00E-04	0	6,00E-04	0	0	53	0,000348	152164	2,00E-04
0	0	0	0	0	0	0	1,47E-05	0	2,00E-04	2	1,31E-05	152192	1,22E-05
0	0	6,55E-05	0	0	0	0	0	0	0	1	6,57E-06	152114	0
0	0	0	0	0	0	0	0	0	0	0	0	0	0
0	0	0	0	0	0	0	0	0	0	0	0	0	0
0	0	6,54E-05	0	0	0	0	2,00E-04	0	0	12	7,88E-05	152206	5,29E-05
0	0	0	0	0	0	0	0	0	0	0	0	0	2,61E-05
0	0	0	0	0	0	0	0	0	0	0	0	0	0
7,23E-05	0	0	0	0	0	0	0	5,00E-04	0	4	2,63E-05	151996	8,12E-06
2,00E-04	0	0	0	0	0	0	5,88E-05	0	0	12	7,89E-05	152156	4,25E-05
0,0016	0	6,55E-05	0	0	0	0	0	0	0	66	0,000434	152078	9,09E-05
0	0	0	0	0	0	0	0	0	0	0	0	0	4,18E-06
0	0	6,55E-05	0	0	0	0	0	0	0	1	6,57E-06	152200	0
0	0	0	0	0,0017	0	0	0	0	0	9	5,92E-05	152142	1,00E-04
1,00E-04	0	6,00E-04	0	0	2,00E-04	0	1,00E-04	0	0	25	0,000164	152226	2,00E-04
0	0	0	0	0	0	0	0	0	0	0	0	0	0
0	0	3,00E-04	0	0,0324	0	0	7,35E-05	9,00E-04	8,00E-04	182	0,001197	152098	0
0	0	0	0	0	0	0	0	0	0	0	0	0	1,22E-05
0	0	0	0	0	0	0	0	0	0	0	0	0	0
0	0	0	0	0	0	0	0	0	0	0	0	0	0
2,41E-05	0	0	0	0	0	0	4,41E-05	0	2,00E-04	5	3,29E-05	152184	3,66E-05
2,41E-05	0	0	0	0	0	0	0	0	0	1	6,57E-06	152154	0
0	0	0	0	0	0	0	0	0	0	0	0	0	0

2,40E-05	0	0	0	2,00E-04	9,41E-05	0	0	0	0	3	1,97E-05	152266	4,08E-06
0	0	0	0	0	0	0	0	0	0	0	0	0	0
0	0	0	0	0	0	0	0	0	0	0	0	0	0
0	0	0	0	0	0	0	0	0	0	0	0	0	0
0	0	0	0	0	0	0	0	0	0	0	0	0	0
0	0	0	0	0	0	0	0	0	0	0	0	0	0
1,00E-04	0	1,00E-04	0	2,00E-04	6,00E-04	0	7,00E-04	0	0	60	0,000394	152224	3,00E-04
0	0	0	0	6,00E-04	0	0	0	0	0	3	1,97E-05	152204	0
0,0036	0	2,00E-04	0	0	0	0	2,94E-05	0	0	154	0,001013	152052	0
0	0	0	0	0	0	0	1,47E-05	0	0	1	6,57E-06	152174	1,22E-05
0	0	0	0	0	0	0	0	0	0	0	0	0	0
0	0	0	0	0	0	0	1,47E-05	0	0	1	6,57E-06	152100	0
0	0	0	0	0	0	0	0	0	0	0	0	0	0
0	0	0	0	0	0	0	1,48E-05	0	0	1	6,64E-06	150530	0
0,0016	0	6,57E-05	0	0	0	0	1,47E-05	0	2,00E-04	70	0,000461	151834	7,76E-05
4,82E-05	0	0	0	0	0	0	0	0	4,00E-04	4	2,63E-05	152010	1,63E-05
0	0	0	0	0	0	0	0	0	0	0	0	0	9,10E-06
0	0	0	0	2,00E-04	0	0	0	0	0	1	6,60E-06	151488	8,61E-06
0	0	0	0	0	0	0	0	0	0	0	0	0	0
6,00E-04	0	6,53E-05	0	0	0	0	1,47E-05	0	0	29	0,000191	152192	3,25E-05
2,41E-05	0	0	0	0	0	0	2,00E-04	0	0	14	9,20E-05	152256	6,10E-05
6,00E-04	0	6,54E-05	0	0	0	0	0	5,00E-04	0	28	0,000184	152224	2,84E-05
0	0	4,00E-04	0	0	0	0	2,94E-05	0	0	8	5,26E-05	152166	3,26E-05
9,63E-05	0	4,00E-04	0	0	0	0,0034	3,00E-04	9,00E-04	0	30	0,000197	152080	1,00E-04
0	0	0	0	0	0	0	0	0	0	0	0	0	0
0	0	0	0	0	0	0	0	0	0	0	0	0	0
2,41E-05	0	0	0	0	0	0	0	0	0	1	6,57E-06	152242	4,06E-06
7,23E-05	0	0	0	0	0	0	2,00E-04	0	0	15	9,85E-05	152234	6,66E-05
0	0	0	0	0	0	0	0	0	0	0	0	0	4,16E-06
0	0	0	0	0	0	0	0	0	0	0	0	0	4,44E-06
2,42E-05	0	0	0	0	0	0	1,47E-05	0	0	2	1,32E-05	152022	8,13E-06
0	0	0	0	0	0	0	0	0	0	0	0	0	0
1,00E-04	0	0	0	0	0	0	0	0	0	5	3,29E-05	152122	8,16E-06
0	0	0	0	0	0	0	0	0	0	0	0	0	0
0	0	0	0	6,00E-04	0	0	0	0	0	3	1,97E-05	152106	2,21E-05
0,0011	0	6,53E-05	0	0	0	0	0	5,00E-04	0	46	0,000302	152204	9,34E-05

0,002	0	3,00E-04	0	0	0	0	0	5,00E-04	0	88	0,000578	152182	1,00E-04
0	0	0	0	0	0	0	0	0	0	0	0	0	6,98E-05
1,00E-04	0	6,53E-05	0,0029	2,00E-04	0	0	2,00E-04	0	0	33	0,000217	152252	2,00E-04
0	0	0	0	0	0	0	0	0	2,00E-04	1	6,57E-06	152188	0
0	0	0	0	0	0	0	0	0	0	0	0	0	0
0	0	0	0	0	0	0	0	0	0	0	0	0	0
0	0	5,00E-04	3,00E-04	0	0	0	1,47E-05	0	0	9	5,92E-05	152144	2,00E-04
2,41E-05	0	6,55E-05	0	0	0	0	1,47E-05	5,00E-04	0	4	2,63E-05	152166	6,61E-06
0	0	0	0	0	9,44E-05	0	0	0	0	1	6,57E-06	152104	8,12E-06
0	0	0	0	0	0	0	0	0	0	0	0	0	0
0	0	0	0	0	0	0	0	0	0	0	0	0	0
4,83E-05	0	0	0	0	0	0	0	0	0	2	1,31E-05	152144	0
0	0	0	0	0	0	0	0	0	0	0	0	0	0
0	0	0	0	0	0	0	2,94E-05	0	0	2	1,31E-05	152142	0
0	0	0	0	0	0	0	0	0	0	0	0	0	0
0,0015	0	3,00E-04	0	0	0	0	1,47E-05	5,00E-04	0	70	0,00046	152170	0
0	0	0	0	0	0	0	0	0	0	0	0	0	0
0	0	0	0	4,00E-04	0	0	0	0	0	2	1,31E-05	152172	8,61E-06
2,00E-04	0	0,0014	3,00E-04	0	0,0012	0	0,0016	0,0014	8,00E-04	164	0,001078	152070	0,0011
0	0	0	0	0	0	0	0	0	0	0	0	0	0
0	0	0	0	0	0	0	1,47E-05	0	0	1	6,57E-06	152204	4,66E-06
0	0	0	0	0	0	0	1,47E-05	0	0	1	6,58E-06	152074	4,47E-05
0	0	6,57E-05	0	0	0	0	4,41E-05	0	0	4	2,64E-05	151484	2,85E-05
0	0	0	0	0	0	0	1,47E-05	0	0	1	6,57E-06	152238	4,06E-06
0	0	0,0012	0	0	0	0	0	0	0	19	0,000125	152200	1,00E-04
0	0	0	0	0	0	0	0	0	0	0	0	0	0
3,00E-04	0	1,00E-04	0	0	9,00E-04	0	0,0012	5,00E-04	0	109	0,000716	152174	6,00E-04
0	0	0	0	0	0	0	0	0	0	0	0	0	0
7,24E-05	0	0	0	0	0	0	2,00E-04	0	0	19	0,000125	152096	5,29E-05
0,0012	0	5,00E-04	0	0	0	0	0	0	0	54	0,000355	152142	1,00E-04
0	0	0	0	0	0	0	0	0	0	0	0	0	0
0	0	0	0	0,0044	0	0	0	0	0	23	0,000151	152156	2,00E-04
0	0	0	0	0	0	0	0	0	0	0	0	0	0
1,00E-04	0	0	0	0	0	0	0	0	0	6	3,94E-05	152226	1,69E-05
0	0	6,56E-05	0	0	0	0	7,35E-05	0	0	6	3,95E-05	151952	5,02E-05
0	0	0	0	0	0	0	0	0	0	0	0	0	0

0,0024	0	3,00E-04	0	0	0	0	0	5,00E-04	0	105	0,00069	152174	0
0	0	0	0	0	0	0	0	0	0	0	0	0	0
0	0	0	0	0	0	0	0	0	0	0	0	0	0
2,41E-05	0	2,00E-04	0	0	0	0	0	0	0	4	2,63E-05	152098	0
2,42E-05	0	0	0	0	0	0	0	0	0	1	6,58E-06	151994	8,19E-06
2,42E-05	0	0	0	0	0	0	0	0	0	1	6,58E-06	152006	0
0,0016	0	1,00E-04	0	0	0	0	1,47E-05	0,0014	0	71	0,000467	152150	1,00E-04
0	0	0	0	0	0	0	0	0	0	0	0	0	0
0	0	0	0	0	0	0	0	0	0	0	0	0	0
0	0	0	0	0	0	0	0	0	0	0	0	0	0
0	0	0	0	0	0	0	0	0	0	0	0	0	0
0	0	0	0	0	0	0	0	0	0	0	0	0	0
0	0	0	0	0	0	0	0	0	0	0	0	0	0
0	0	0	0	0	0	0	0	0	0	0	0	0	0
0	0	1,00E-04	0	0	0	0	1,47E-05	0	0	3	1,97E-05	152240	1,22E-05
7,22E-05	0	0	0	0	0	0	0	0	0	3	1,97E-05	152096	1,63E-05
4,81E-05	0	6,53E-05	0	0	0	0	1,00E-04	0	0	12	7,89E-05	152186	4,47E-05
0	0	0	0	0	0	0	0	0	0	0	0	0	0
0	0	0	0	0	0	0	1,00E-04	0	0	8	5,26E-05	152190	0
0	0	0	0	0	0	0	0	0	0	0	0	0	0
0	0	0	0	0	0	0	1,47E-05	0	0	1	6,57E-06	152126	0
2,42E-05	0	0	0	0	0	0	0	0	0	1	6,58E-06	152028	2,03E-05
0	0	0	0	0	0	0	2,95E-05	0	0	2	1,32E-05	151528	2,86E-05
7,24E-05	0	2,00E-04	0	0	0,0022	0	4,00E-04	0	0	54	0,000356	151810	5,00E-04
0,0045	0	5,00E-04	0	0	0	0	1,00E-04	0,0029	0	207	0,001367	151408	3,00E-04
0	0	0	0	0	0	0	0	0	0	0	0	0	0
0	0	6,54E-05	0	0	0	0	1,47E-05	0	0	2	1,31E-05	152236	0
0	0	0	0	0	0	0	0	0	0	0	0	0	0
0	0	0	0	0	0	0	0	0	0	0	0	0	0
0,001	0	6,56E-05	0	0	0	0	0	0,001	0	46	0,000303	152004	2,23E-05
0	0	0	0	0	0	0	0	0	0	0	0	0	0
6,00E-04	0	0	0	0	0	0	0	0	0	24	0,000158	151948	2,68E-05
0	0	0	0	0	0	0	0	0	0	0	0	0	0
0	0	0	0	0	0	0	0	0	0	0	0	0	1,98E-05
4,83E-05	0	0	0	0	0	0	8,82E-05	0	0	8	5,26E-05	152146	1,32E-05
2,00E-04	0	6,56E-05	0	0	5,00E-04	0,0068	4,00E-04	0	0	42	0,000277	151714	2,00E-04

0	0	6,54E-05	0	0	0	0	1,47E-05	0	0	2	1,32E-05	152062	2,12E-05
0	0	0	0	0	0	0	0	0	0	0	0	0	0
0	0	0	0	0	0	0	0	0	0	0	0	0	4,07E-06
0	0	0	0	0	0	0	0	0	0	0	0	0	0
0	0	0	0	0	0	0	0	0	0	0	0	0	0
2,00E-04	0	6,00E-04	0	0	0	0	2,00E-04	5,00E-04	0	32	0,00021	152106	2,00E-04
0,0011	0	0	0	0	0	0	0	0	0	46	0,000303	151698	7,77E-05
5,00E-04	0	2,00E-04	0	0	0	0	0	5,00E-04	0	24	0,000158	152202	1,00E-04
0	0	6,54E-05	0	0	0	0	7,35E-05	0	0	6	3,94E-05	152226	5,84E-05
0	0	0	0	0	0	0	0	0	0	0	0	0	0
2,41E-05	0	0	0	0	0	0	0	0	0	1	6,57E-06	152216	4,07E-06
0	0	0	0	0	0	0	0	0	0	0	0	0	0
0	0	6,55E-05	0	0	9,41E-05	0	6,00E-04	0	0	45	0,000296	152190	2,00E-04
0	0	0	0	0	0	0	0	0	0	0	0	0	0
0	0	0	0	0	0	0	0	0	0	0	0	0	0
2,54E-05	0	0	0	0,0094	0	0	0	0	0	38	0,00027	140734	5,00E-04
2,41E-05	0	0	0	0	0	0	0	0	0	1	6,57E-06	152152	4,08E-06
0	0	0	0	0	0	0	0	0	0	0	0	0	0
0	0	0	0	0	0	0	0	0	0	0	0	0	0
0	0	6,55E-05	0	0	0	0	0	0	0	1	6,57E-06	152174	0
2,41E-05	0	0	0	2,00E-04	0	0	2,94E-05	0	0	4	2,63E-05	152100	1,28E-05
0	0	0	0	0	0	0	0	0	0	0	0	0	0
4,81E-05	0	1,00E-04	0	0	0	0	5,88E-05	0	0	8	5,26E-05	152040	4,47E-05
2,00E-04	0	3,00E-04	0	0,0332	4,00E-04	0	1,47E-05	0,0019	6,00E-04	196	0,001288	152154	0
0	0	0	0	0	0	0	0	0	0	0	0	0	0
1,00E-04	0	5,00E-04	3,00E-04	0	0	0	8,00E-04	0	0	70	0,000475	147520	4,00E-04
0	0	0	0	0	0	0	0	0	0	0	0	0	7,07E-06
0	0	0	0	0	0	0	0	0	0	0	0	0	0
2,41E-05	0	0	0	0	0	0	0	0	0	1	6,57E-06	152174	4,07E-06
0	0	0	0	0	0	0	0	0	0	0	0	0	0
0	0	0	0	0	0	0	0	0	0	0	0	0	0
0	0	0	0	0	0	0	1,47E-05	0	4,00E-04	3	1,97E-05	152008	3,61E-05
0	0	0	0	0	0	0	0	0	0	0	0	0	0
0	0	0	0	0	0	0	0	0	0	0	0	0	0
0	0	0	0	0	0	0	1,47E-05	0	0	1	6,57E-06	152188	4,06E-06
4,83E-05	0	0	0	0	0	0	0	0	0	2	1,31E-05	152170	0

9,66E-05	0	0	0	0	0	0	1,47E-05	0	0	5	3,29E-05	152138	0
3,00E-04	0	6,54E-05	3,00E-04	0	0	0	1,47E-05	0	2,00E-04	13	8,54E-05	152144	3,66E-05
0	0	6,56E-05	0	0	0	0	7,36E-05	5,00E-04	0	7	4,61E-05	151786	4,71E-05
0,002	0	3,00E-04	0	6,00E-04	0	0	1,47E-05	5,00E-04	0,0025	104	0,000685	151786	3,00E-04
3,00E-04	0	0	0	0	0	0	0	0	0	10	7,28E-05	137450	8,57E-06
2,41E-05	0	1,00E-04	0	0	0	0	0	0	0	3	1,97E-05	152210	4,07E-06
0	0	0	0	0	0	0	0	0	0	0	0	0	0
7,71E-05	0	8,05E-05	0	6,00E-04	0	0	6,32E-05	5,00E-04	3,00E-04	12	8,77E-05	136830	0
0	0	0	0	0	0	0	0	0	0	0	0	0	0
0	0	6,55E-05	0	0	0	0	0	0	0	1	6,57E-06	152184	0
0	0	0	0	0	0	0	0	0	0	0	0	0	0
0	0	0	0	4,00E-04	0	0	1,47E-05	0	0	3	1,97E-05	151928	3,66E-05
2,41E-05	0	6,00E-04	0	0	0	0	1,00E-04	0,0014	0	20	0,000131	152128	2,00E-04
0	0	0	0	0	0	0	0	0	0	0	0	0	0
0	0	0	0	0	0	0	0	0	0	0	0	0	0
0	0	0	0	0	0	0	0	0	0	0	0	0	0
2,41E-05	0	0	0	0	0	0	0	0	0	1	6,57E-06	152182	1,22E-05
0	0	0	0	0	0	0	1,47E-05	0	0	1	6,57E-06	152232	0
0,001	0	6,54E-05	0	0	0	0	0	0	0	41	0,000269	152200	6,13E-05
2,42E-05	0	0	0	0	0	0	0	0	0	1	6,58E-06	152074	0
0	0	0	0	0	0	0	0	0	0	0	0	0	0
2,42E-05	0	1,00E-04	0	0	0	0	5,88E-05	0	0	7	4,61E-05	152000	3,66E-05
0	0	0	0	2,00E-04	0	0	1,47E-05	0	0	2	1,31E-05	152256	1,63E-05
0	0	0	0	0	0	0	0	0	0	0	0	0	0
4,81E-05	0	0	0	0	0,0052	0	8,00E-04	0	0	110	0,000723	152188	0,0011
0,0011	0	6,53E-05	0	0	0	0	0	0	0	46	0,000302	152212	1,00E-04
0	0	0	0	8,00E-04	0	0	0	0	0	4	2,63E-05	152122	1,65E-05
0	0	0	0	0	0	0	2,94E-05	0	0	2	1,32E-05	151970	0
0	0	0	0	0	0	0	0	0	0	0	0	0	4,07E-06
2,00E-04	0	6,54E-05	0	0	0	0	0	0	0	8	5,26E-05	152192	1,22E-05
5,00E-04	0	6,56E-05	0	0	0	0	0	5,00E-04	0	22	0,000145	151898	2,04E-05
1,00E-04	0	0	0	0	0	0	1,95E-05	0	0	0	0	0	0
4,83E-05	0	6,54E-05	0	0	0	0	8,82E-05	0	0	9	5,91E-05	152184	7,73E-05
2,00E-04	0,0011	6,00E-04	0	0	9,43E-05	0	0,0012	0	2,00E-04	103	0,000677	152062	6,00E-04
0	0	0	0	0	0	0	1,47E-05	0	0	1	6,57E-06	152180	6,69E-06
0	0	6,00E-04	0	0	0	0	0	0	0	9	5,91E-05	152208	1,22E-05

2,41E-05	0	0	0	0	9,41E-05	0	8,82E-05	0	0	8	5,26E-05	152216	1,00E-04
4,83E-05	0	0	0	0	0	0	1,00E-04	0	0	9	5,91E-05	152198	2,84E-05
0	0	6,55E-05	0	0	0	0	0	0	0	1	6,58E-06	151992	0
0	0	0	0	0	0	0	0	0	0	0	0	0	4,80E-06
0	0	0	0	0	0	0	0	0	0	0	0	0	4,07E-06
0	0	0	0	0	0	0	0	0	0	0	0	0	0
0	0	3,00E-04	0	0	0	0	1,47E-05	0	0	5	3,29E-05	152186	2,45E-05
0	0	0	0	0	0	0	1,47E-05	0	0	1	6,58E-06	152004	4,31E-06
0	0	0	0	2,00E-04	0	0	5,88E-05	0	0	5	3,29E-05	152016	5,71E-05
0	0	0	0	0	0	0	0	0	0	0	0	0	0
0,0026	0	2,00E-04	0	0	0	0	3,01E-05	0	0	114	0,000771	147872	0
0,0026	0	2,00E-04	0	0	0	0	3,01E-05	0	0	114	0,00077	148038	0
0	0	2,00E-04	0	0	0	0	1,47E-05	5,00E-04	0	5	3,29E-05	151746	3,25E-05
0	0	0,0013	0,0046	0	0	0	2,94E-05	0	0	38	0,00025	152190	5,00E-04
0	0	0	0	0	0	0	0	0	0	0	0	0	0
0	0	0	0	0	0	0	0	0	0	0	0	0	0
0,0057	0	3,00E-04	0	0	0	0	2,95E-05	0,0019	0	246	0,001622	151630	3,00E-04
0	0	0	0	0	0	0	0	0	0	0	0	0	0
0	0	0	0	0	0	0	0	0	0	0	0	0	0
0	0	0	0	0	9,46E-05	0	0	0	0	1	6,58E-06	151944	8,13E-06
0,0018	0	7,00E-04	0	0	0	0	1,47E-05	0	0	83	0,000546	152052	2,00E-04
0	0	0	0	0	0	0	1,47E-05	5,00E-04	0	2	1,32E-05	151940	1,22E-05
0	0	0	0	0	0	0	0	0	0	0	0	0	8,20E-06
0	0	0	0	0	0	0	1,47E-05	0	0	1	6,58E-06	151908	0
0	0	0	0	0	0	0	0	0	0	0	0	0	0
0,0051	0	8,00E-04	0	0	0	0	0	0,0038	0	232	0,001525	152100	3,00E-04
0	0	0	0	0	0	0	0	0	0	0	0	0	0
0	0	0	0	0	0	0	0	0	0	0	0	0	0
0	0	6,55E-05	0	0	0	0	0	0	0	1	6,57E-06	152202	2,03E-05
2,41E-05	0	1,00E-04	0	0	0	0	1,47E-05	0,001	0	6	3,94E-05	152220	4,07E-06
0	0	0	0	0	0	0	1,47E-05	5,00E-04	0	2	1,31E-05	152222	8,22E-06
0	0	0	0	0	0	0	0	0	0	0	0	0	0
0	0	0	0	0	0	0	1,47E-05	0	0	1	6,57E-06	152252	0
0	0	0	0	0	0	0	0	0	0	0	0	0	8,15E-06
2,42E-05	0	0	0	0	0	0	1,47E-05	0	0	2	1,31E-05	152132	3,66E-05
0	0	0	0	0	0	0	0	0	0	0	0	0	0

0	0	0	0	0	0	0	0	0	0	0	0	0	0
2,41E-05	0	0	0	0	0	0	4,41E-05	5,00E-04	0	5	3,29E-05	152172	6,09E-05
3,00E-04	0	0	0	0,0014	0	0	2,94E-05	0	0	22	0,000145	152102	1,00E-04
0	0	0	0	0	0	0	0	0	0	0	0	0	0
0	0	0	0	0	0	0	0	0	0	0	0	0	0
2,42E-05	0	0	0	0	0	0	0	0	0	1	6,58E-06	152086	6,07E-06
2,41E-05	0	0	0	0	0	0	0	0	0	1	6,57E-06	152166	8,14E-06
0	0	0	0	0	0	0	0	0	0	0	0	0	0
0	0	0	0	0	0	0	0	0	0	0	0	0	0
0	0	0	0	0	0	0	0	0	2,00E-04	1	6,58E-06	152078	4,06E-06
0,002	0	0	0,0017	0	0	0	5,88E-05	9,00E-04	0	98	0,000644	152160	2,00E-04
0	0	0	0	0	0	0	0	0	0	0	0	0	0
0	0	0	0	0	0	0	1,47E-05	0	0	1	6,57E-06	152226	8,85E-06
0	0	0	0	0	0	0	0	0	0	0	0	0	0
0	0	0	0	0	0	0	0	0	0	0	0	0	0
2,41E-05	0	0	0	0	0	0	0	0	0	1	6,57E-06	152168	1,22E-05
0	0	0	0	0	0	0	0	0	0	0	0	0	0
0	0	0	0	0	0	0	0	0	0	0	0	0	0
0	0	0	0	0	0	0	4,41E-05	0	0	3	1,97E-05	152132	4,06E-06
2,42E-05	0	1,00E-04	0	0	0	0	8,83E-05	0	0,0102	58	0,000382	151736	0,0013
0	0	6,55E-05	0	0	0	0	0	0	0,0025	13	8,56E-05	151878	3,00E-04
0,0024	0	6,54E-05	0	0	0	0	0	5,00E-04	2,00E-04	100	0,000658	152082	1,00E-04
0	0	0	0	0	0	0	0	0	0	0	0	0	0
9,63E-05	0	2,00E-04	3,00E-04	0	0,0053	0	2,00E-04	5,00E-04	0,0035	94	0,000618	152112	9,00E-04
3,00E-04	0	1,00E-04	0	0,0022	0	0	6,07E-05	5,00E-04	2,00E-04	30	0,000207	145154	4,14E-05
3,00E-04	0	1,00E-04	0	0,0022	0	0	4,52E-05	5,00E-04	2,00E-04	29	0,000199	146036	3,09E-05
0,0052	0	0,0012	0	4,00E-04	0	0	2,00E-04	5,00E-04	4,00E-04	236	0,001614	146188	1,03E-05
0,005	0	0,0012	0	4,00E-04	0	0	2,00E-04	5,00E-04	4,00E-04	229	0,00159	144026	1,03E-05
6,00E-04	0	0,0021	0,0024	0	0,0047	0	0,0018	0,0015	4,00E-04	229	0,001623	141108	4,00E-04
8,00E-04	0	0,0022	0,0027	0	0,0047	0	0,0019	0,0016	2,00E-04	236	0,001742	135462	4,00E-04
0,0082	0	5,00E-04	0	0	0	0	0	6,00E-04	0	258	0,002159	119516	0
2,42E-05	0	0	0	0	0	0	2,94E-05	0	0	3	1,97E-05	152070	0
0	0	0	0	4,00E-04	0	0	0	0	0	2	1,31E-05	152110	4,80E-05
0,001	0	6,54E-05	0	0	0	0	2,94E-05	0	0	45	0,000296	152208	6,94E-05
0	0	0	0	0	0	0	0	0	0	0	0	0	0
0	0	0	0	0	0	0	0	0	0	0	0	0	0

1,00E-04	0	0	0	0	0	0	1,47E-05	0	2,00E-04	8	5,26E-05	152130	2,86E-05
0	0	0	0	0	0	0	0	0	0	0	0	0	4,07E-06
2,42E-05	0	6,55E-05	0	0	0	0	2,00E-04	0	0	13	8,55E-05	152126	1,00E-04
2,42E-05	0	0	0	0	0	0	1,47E-05	0	0	2	1,32E-05	152060	4,08E-06
0	0	0	0	0	0	0	0	0	0	0	0	0	0
0	0	0	0	0	0	0	0	0	0	0	0	0	0
0	0	0	0	0	0	0	0	0	0	0	0	0	0
0	0	6,54E-05	0	0	0	0	1,00E-04	5,00E-04	0	9	5,91E-05	152184	6,21E-05
0	0	0	0	0	0	0	0	0	0	0	0	0	0
7,00E-04	0,0197	1,00E-04	0	0	6,00E-04	0	0,0032	0	0	274	0,0018	152224	0,0013
0	0	6,55E-05	0	0	0	0	0	0	0	1	6,58E-06	152084	8,13E-06
3,00E-04	0	2,00E-04	0	0	0	0	0	0	0	15	9,86E-05	152190	4,48E-05
2,41E-05	0	0	0	0	0	0	0	0	0	1	6,57E-06	152186	0
0	0	0	0	0	0	0	0	0	0	0	0	0	0
0	0	0	0	0	0	0	0	5,00E-04	0	1	6,57E-06	152200	0
0	0	0	0	0	0	0	0	0	0	0	0	0	4,14E-06
0	0	0	0	0	0	0	5,88E-05	0	0	4	2,63E-05	152144	4,89E-05
0	0	0	0	0	0	0	2,94E-05	0	0	2	1,31E-05	152112	4,08E-06
0	0	3,00E-04	0	0	0	0	2,00E-04	9,00E-04	0	20	0,000131	152196	2,00E-04
0	0	0	0	0	0	0	0	0	0	0	0	0	0
0	0	0	0	0	0	0	0	0	0	0	0	0	0
0	0	0	0	0	0	0	0	0	0	0	0	0	0
0,0014	0	2,00E-04	0	0	0	0	0	0	0	63	0,000414	152184	1,00E-04
0	0	0	0	0	0	0	0	0	0	0	0	0	1,22E-05
0	0	0	0	0	0	0	0	0	0	0	0	0	0
0	0	0	0	0	0	0	2,94E-05	0	0	2	1,31E-05	152180	8,32E-06
0	0	0	0	0	0	0	0	0	0	0	0	0	0
0	0	0	0	0	0	0	0	0	0	0	0	0	0
0	0	0	0	0	0	0	0	0	0	0	0	0	0
0	0	0	0	0	0	0	1,47E-05	5,00E-04	0	2	1,31E-05	152248	1,23E-05
0	0	0	0	0	0	0	0	0	0	0	0	0	0
0	0	0	0	0	0	0	0	0	0	0	0	0	0
0,0058	0	3,00E-04	0	0	0	0	1,47E-05	9,00E-04	0	250	0,001643	152176	4,00E-04
0	0	0	0	0	0	0	0	0	0	0	0	0	0
0	0	0	0	0	0	0	0	0	0	0	0	0	0
0	0	6,54E-05	0	0	0	0	0	0	0	1	6,57E-06	152220	4,06E-06

0	0	0	0	0	0	0	0	0	0	0	0	0	0
0,0082	0	0,0014	0	0	0	0,0074	2,00E-04	0	0	144	0,001822	79022	6,54E-05
0	0	0	0	2,00E-04	0	0	2,94E-05	0	0	3	1,97E-05	152004	0
0,0017	0	3,00E-04	0	0	0	0	0	0,0014	0	80	0,000526	152230	1,00E-04
0	0	0	0	0	0	0	2,94E-05	0	0	2	1,31E-05	152164	4,06E-06
9,65E-05	0	0	0	0	0	0	2,94E-05	0	0	6	3,94E-05	152212	8,12E-06
0	0	0	0	0,0017	0	0	0	0	0	9	5,92E-05	152096	8,54E-05
2,41E-05	0	0	0	0	0	0	0	0	0	1	6,57E-06	152126	1,22E-05
2,41E-05	0	0	0	0	0	0	1,47E-05	0	2,00E-04	3	1,97E-05	152126	1,22E-05
0	0	0	0	0	0	0	0	0	0	0	0	0	4,07E-06
0	0	0	0	0	0	0	0	0	0	0	0	0	0
0	0	6,56E-05	0	0	0	0	0	0	0	1	6,58E-06	152074	0
0	0	1,00E-04	0	0	0	0	8,82E-05	0,001	0	10	6,57E-05	152184	4,47E-05
2,42E-05	0	0	0	0	9,45E-05	0	2,94E-05	0	0	4	2,63E-05	152060	3,66E-05
0	0	0	0	0	0	0	1,47E-05	0	0	1	6,57E-06	152194	0
0	0	0	0	0	0	0	0	0	0	0	0	0	0
0	0	0	0	0	0	0	1,47E-05	5,00E-04	0	2	1,31E-05	152196	4,06E-06
4,81E-05	0	0,0011	3,00E-04	0	0	0,0034	1,00E-04	5,00E-04	0	30	0,000197	152208	2,00E-04
0	0	0	0	0	0	0	7,35E-05	5,00E-04	0	6	3,94E-05	152166	2,05E-05
7,24E-05	0,0241	0	0	0	0	0	1,00E-04	0	0	32	0,00021	152236	6,92E-05
0	0	6,55E-05	0	0	0	0	2,94E-05	0	0	3	1,97E-05	152040	0
0	0	0	0	0	0	0	0	0	0	0	0	0	0
7,22E-05	0	3,00E-04	0	0	4,00E-04	0	0,001	5,00E-04	0	77	0,000506	152078	4,00E-04
0	0	0	0	0	0	0	1,47E-05	0	0	1	6,58E-06	152054	4,07E-06
0	0	6,55E-05	0	0	0	0	0	0	0	1	6,58E-06	152016	2,03E-05
0	0	0	0	0	0	0	0	5,00E-04	0	1	6,57E-06	152242	5,42E-06
0,0023	0	2,00E-04	0	0	0	0	0	0	0	96	0,000631	152236	2,00E-04
2,44E-05	0	0	0	0	0	0	0	0	0	1	6,66E-06	150236	1,43E-05
0	0	0	0	0	0	0	1,47E-05	0	0	1	6,58E-06	151918	4,10E-06
0	0	0	0	2,00E-04	0	0	0	0	0	1	6,58E-06	152056	0
0	0	0	0	0	0	0	0	0	0	0	0	0	4,07E-06
0	0	0	0	0	0	0	0	0	0	0	0	0	0
0	0	0	0	0	0	0	0	0	0	0	0	0	0
0	0	0	0	0	0	0	0	0	0	0	0	0	0
0,0012	0	6,54E-05	0	0	0	0	0	0	0	50	0,000329	152188	3,02E-05
0	0	6,55E-05	0	0	0	0	1,47E-05	0	0	2	1,31E-05	152156	3,66E-05

2,41E-05	0	0	0	0	0	0	5,88E-05	0	0	5	3,29E-05	152166	0
0	0	0	0	0	0	0	0	0	0	0	0	0	0
7,43E-05	0	0	0	0	0	0	0	0	0	3	2,02E-05	148314	0
0	0	0	0	0	0	0	0	0	0	0	0	0	0
0,0029	0	3,00E-04	0	0	0	0	0	0	0	121	0,000796	152046	2,00E-04
0	0	0	0	0	0	0	8,82E-05	0	0	6	3,94E-05	152102	4,87E-05
1,00E-04	0	6,54E-05	0,004	0	6,00E-04	0	0,001	5,00E-04	0	99	0,000651	152116	8,00E-04
0	0	0	0	0	0	0	0	0	0	0	0	0	0
2,41E-05	0	1,00E-04	0	0	0	0	2,00E-04	0,0019	0	18	0,000118	152176	4,50E-05
0	0	6,55E-05	0	0	0	0	2,94E-05	0	0	3	1,97E-05	152142	8,14E-06
0	0	3,00E-04	0	0	0	0	5,88E-05	0	0	8	5,26E-05	152220	8,60E-05
4,83E-05	0	2,00E-04	0	0	0	0	4,42E-05	0	0	8	5,26E-05	152102	4,10E-05
0	0	0	0	0	0	0	0	0	0	0	0	0	0
0	0	0	0	0	0	0	2,94E-05	0	0	2	1,31E-05	152156	1,26E-05
2,41E-05	0	0	0	0	9,41E-05	0	0	0	0	2	1,31E-05	152146	1,22E-05
2,41E-05	0	0	0	0	0	0,0034	2,00E-04	0	0	13	8,54E-05	152194	2,00E-04
0	0	0	0	0	0	0	0	0	0	0	0	0	0
0	0	0	0	0	0	0	0	0	0	0	0	0	0
0,0066	0	3,00E-04	0	0	0	0	1,47E-05	5,00E-04	0	281	0,001849	151982	5,00E-04
0	0	0	0	0	0	0	0	0	0	0	0	0	0
1,00E-04	0	0	0	2,00E-04	0	0	6,00E-04	0	2,00E-04	45	0,000296	152142	3,00E-04
1,00E-04	0	0	0	2,00E-04	0	0	6,00E-04	0	2,00E-04	45	0,000296	152080	3,00E-04
2,41E-05	0	0	0	0	0	0	0	0	0	1	6,57E-06	152176	0
0	0	0	0	0	0	0	0	0	0	0	0	0	0
0	0	6,55E-05	0	0	0	0	0	0	0	1	6,57E-06	152174	0
0	0	0	0	0	0	0	0	0	0	0	0	0	4,06E-06
0	0	0	0	0	0	0	0	0	0	0	0	0	1,63E-05
0	0	0	0	0	0	0	0	0	0	0	0	0	0
0	0	0	0	0	0	0	0	0	0	0	0	0	1,22E-05
0	0	0	0	0	0	0	0	0	0	0	0	0	0
0	0	0	0	0	0	0	0	0	0	0	0	0	4,07E-06
0	0	0	0	0	0	0	0	0	0	0	0	0	0
0	0	0	0	0	0	0	2,94E-05	0	6,00E-04	5	3,28E-05	152222	2,00E-04
0	0	0	0	0	0	0	0	0	0	0	0	0	4,35E-06
0	0	0	0	0	0	0	0	0	0	0	0	0	0
7,24E-05	0	0	0	0	0	0	1,47E-05	0	0	4	2,63E-05	152212	8,13E-06

0	0	0	0	0	0	0	1,47E-05	0	0	1	6,57E-06	152238	0
0,0054	0	1,00E-04	0	0	0	0	0	0,0019	0	227	0,001492	152190	4,00E-04
0	0	0	0	0	0	0	0	0	0	0	0	0	0
0	0	0	0	0	0	0	1,47E-05	0	0	1	6,57E-06	152222	8,19E-06
2,41E-05	0	0	0	0	0	0	0	0	0	1	6,57E-06	152132	0
0	0	0	0	0	0	0	1,47E-05	0	0	1	6,57E-06	152138	1,22E-05
2,00E-04	0	0	0	0	0,0021	0	4,00E-04	0	0	56	0,000368	152142	5,00E-04
9,63E-05	0	0	0	0	0	0	4,41E-05	0	0	7	4,60E-05	152154	3,66E-05
9,68E-05	0	2,00E-04	0	0	0	0	5,89E-05	5,00E-04	0	12	7,92E-05	151490	8,03E-05
6,00E-04	0	1,00E-04	0	0	0	0	0,0016	5,00E-04	0	135	0,000888	151998	8,00E-04
2,41E-05	0	2,00E-04	0	0	0	0	3,00E-04	0	0	25	0,000164	152198	1,00E-04
0	0	0	0	0	0	0	1,47E-05	0	0	1	6,57E-06	152198	0
2,00E-04	0	0,0012	0	0	0	0	7,36E-05	0	0	31	0,000204	151710	0,001
0	0	1,00E-04	0	0	0	0	0	0	0	2	1,31E-05	152196	0
4,83E-05	0	0	0	2,00E-04	0	0	1,47E-05	0	0	4	2,63E-05	152176	2,06E-05
0	0	0	0	0	0	0	1,47E-05	0	0	1	6,57E-06	152168	4,08E-06
0	0	0	0	0	0	0	0	0	0	0	0	0	1,22E-05
0	0	0	0	0	0	0	0	0	0	0	0	0	0
0	0	0	0	0	0	0	0	0	0	0	0	0	0
0	0	0	0	0	0	0	0	0	0	0	0	0	4,29E-06
0	0	0	0	0	0	0	0	0	0	0	0	0	2,03E-05
0	0	0	0	0	0	0	0	0	0	0	0	0	0
0	0	0	0	0	0	0	0	0	0	0	0	0	0
0	0	0	0	0	0	0	0	0	0	0	0	0	0
0,0082	0	0,0012	0	0	0	0	0	0,0019	2,00E-04	361	0,002402	150290	0
0,0082	0	0,0012	0	0	0	0	0	0,0019	2,00E-04	361	0,002387	151264	0
0,0011	0	4,00E-04	0	0	0	0,0068	0	9,00E-04	0	57	0,000374	152228	1,00E-04
2,41E-05	0	5,00E-04	0	0	0	0	0	5,00E-04	0	9	5,91E-05	152178	8,35E-06
0	0	0	0	2,00E-04	0	0	0	0	0	1	6,57E-06	152174	4,06E-06
0	0	0	0	0	0	0	0	0	0	0	0	0	4,15E-06
0	0	0	0	0	0	0	0	0	0	0	0	0	3,41E-05
0	0	4,00E-04	0	0	0	0	0	0	0	6	3,94E-05	152204	1,37E-05
2,41E-05	0	2,00E-04	0	4,00E-04	9,42E-05	0	7,35E-05	0	0	12	7,89E-05	152182	9,96E-05
0	0	0	0	0	0	0	0	0	0	0	0	0	0
0	0	0	0	0	0	0	0	0	0	0	0	0	0
0	0	0	0	0	0	0	0	0	0	0	0	0	0

2,41E-05	0	5,00E-04	0,0236	2,00E-04	0	0,0034	5,00E-04	9,00E-04	0	129	0,000848	152164	0
0	0	0	0	0	0	0	0	0	0	0	0	0	0
1,00E-04	0	0	0	0	0	0	1,47E-05	0	0	6	3,94E-05	152180	2,84E-05
0	0	0	0	0	0	0	0	0	0	0	0	0	0
7,21E-05	0	0	0	2,00E-04	0	0	0	0	0	4	2,63E-05	152262	1,22E-05
0	0	2,00E-04	0	0	0	0	1,47E-05	0	0	4	2,63E-05	152142	0
0	0	0	9,00E-04	0	0	0	1,00E-04	0	0,001	16	0,000105	152140	3,00E-04
0	0	0	0	0	0	0	0	0	0	0	0	0	0
0	0	0	0	0	0	0	0	0	0	0	0	0	0
0	0	0	0	0	0	0	1,47E-05	0	0	1	6,57E-06	152206	4,07E-06
0	0	0	0	0	0	0	0	0	0	0	0	0	0
2,00E-04	0	0	0	0	0	0	0	0	0	10	6,57E-05	152190	4,07E-06
6,00E-04	0	1,00E-04	0	0	0	0	0	0	2,00E-04	28	0,000184	152140	6,42E-05
0	0	0	0	0	0	0	0	0	0	0	0	0	0
2,42E-05	0	0	0	0	0	0	1,47E-05	0	0	2	1,32E-05	151992	5,47E-06
0	0	0	0	0	0	0	0	0	0	0	0	0	4,07E-06
0	0	0	0	0	0	0	0	0	0	0	0	0	4,07E-06
0	0	1,00E-04	0	0	0	0	0	0	0	2	1,31E-05	152226	0
0	0	1,00E-04	0	0	0	0	2,94E-05	0	0	4	2,63E-05	152212	7,31E-05
4,83E-05	0	6,54E-05	0	0	0	0	4,00E-04	0	0	28	0,000184	152200	2,00E-04
0	0	0	0	0	0	0	0	0	0	0	0	0	0
7,00E-04	0	6,00E-04	0	0	8,00E-04	0	0,0036	0,0014	0,0043	311	0,002044	152170	0,0029
2,41E-05	0	0	0	0	0	0	4,41E-05	0	0	4	2,63E-05	152210	1,98E-05
2,42E-05	0	0	0	0	0	0	1,47E-05	0	0	2	1,32E-05	151634	9,75E-06
2,41E-05	0	6,55E-05	0	0	0	0	0	0	0	2	1,32E-05	152036	1,22E-05
0	0	0	0	0	0	0	0	0	0	0	0	0	0
0	0	6,53E-05	0	0	0	0	2,94E-05	0	0	3	1,97E-05	152186	1,22E-05
4,00E-04	0	3,00E-04	0	0	0	0	0	5,00E-04	2,00E-04	21	0,000138	152100	3,66E-05
0	0	0	0	0	0	0	0	0	0	0	0	0	0
2,42E-05	0	0	0	0	0	0	0	5,00E-04	0	2	1,32E-05	152014	1,63E-05
0	0	0	0	0	0	0	0	0	0	0	0	0	4,10E-06
0	0	0	0	0	9,42E-05	0	1,47E-05	0	0	2	1,31E-05	152196	1,22E-05
0	0	6,55E-05	0	0	0	0	2,94E-05	0	0	3	1,97E-05	152160	0
0	0	0	0	0	0	0	0	0	0	0	0	0	0
0	0	6,54E-05	0	0	0	0	0	0	0	1	6,57E-06	152234	8,19E-06
0	0	0	0	0	0	0	0	0	0	0	0	0	0

9,66E-05	0	0	0	0	0	0	0	0	0	4	2,63E-05	152002	1,34E-05
0	0	0	0	0	0	0	0	0	0	0	0	0	0
0	0	0	0	0	0	0	0	0	0	0	0	0	0
2,42E-05	0	0	0	0	0	0	2,94E-05	0	0	3	1,97E-05	151960	1,24E-05
0	0	0	0	0	0	0	0	0	0	0	0	0	0
2,00E-04	0	0,0012	0	0	0	0	7,00E-04	5,00E-04	0	75	0,000493	152140	3,00E-04
2,42E-05	0	0	0	0	0	0	0	0	0	1	6,58E-06	152068	2,44E-05
0	0	0	0	0	0	0	0	0	0	0	0	0	4,48E-06
0	0	0	0	0	0	0	0	0	0	0	0	0	1,22E-05
0	0	0	0	0	0	0	0	0	0	0	0	0	8,13E-06
0	0	0	0	0	0	0	0	0	0	0	0	0	0
0	0	0	0	0	0	0	0	0	0	0	0	0	4,06E-06
2,41E-05	0	0,002	0	0	0	0	0	0	0	31	0,000204	152170	4,88E-05
0	0	0	0	0	0	0	0	0	0	0	0	0	4,07E-06
0	0	0	0	0	0	0	0	0	0	0	0	0	4,20E-06
0	0	0	0	0	0	0	0	0	0	0	0	0	0
0,0012	0	0,0012	0	0	0	0	0	5,00E-04	0	71	0,000467	152122	1,00E-04
4,81E-05	0	0	0	0	0	0	0	0	0	2	1,31E-05	152150	3,25E-05
0	0	0	0	0	0	0	0	0	0	0	0	0	4,08E-06
0	0	0	0	0	0	0	0	0	0	0	0	0	1,22E-05
0	0	0	0	0	0	0	0	0	0	0	0	0	0
2,00E-04	0	6,84E-05	0	0	0	0	0	0	0	10	6,92E-05	144560	0
2,00E-04	0	6,82E-05	0	0	0	0	0	0	0	10	6,90E-05	145006	0
0	0	0	0	0	0	0	8,83E-05	0	0	6	3,95E-05	152034	1,23E-05
0	0	0	0	0	0	0	0	0	0	0	0	0	0
4,00E-04	0	6,55E-05	0	0	0	0	0	0	0	18	0,000118	152060	0
3,00E-04	0	6,68E-05	0,0012	0	0	0	5,00E-04	5,00E-04	0	49	0,000337	145298	0
0	0	0	0	0	0	0	1,47E-05	0	0	1	6,57E-06	152232	8,12E-06
0,0076	0	3,00E-04	0	0	0	0	4,41E-05	0,0033	0	331	0,002175	152204	5,00E-04
0	0	0	0	0	0	0	0	0	0	0	0	0	0
2,41E-05	0	6,53E-05	0	0	0	0	1,00E-04	0	0	10	6,57E-05	152198	9,10E-05
0	0	0	0	0	0	0	0	0	0	0	0	0	0
0,002	0	6,00E-04	0	0,0046	0	0	5,88E-05	0,0019	0	125	0,000821	152220	5,00E-04
0	0	0	0	0	0	0	1,47E-05	0	0	1	6,57E-06	152230	1,36E-05
0	0	0	0	0	0	0	0	0	0	0	0	0	0
0,0043	0	3,00E-04	0	0	0	0	0	0	0	173	0,001138	152052	2,00E-04

0	0	0	0	0	0	0	0	0	0	0	0	0	0
0	0	0	0	0	0	0	0	0	0	0	0	0	0
0	0	0	0	0	0	0	0	0	0	0	0	0	0
0	0	0	0	0	0	0	4,41E-05	0	0	3	1,97E-05	152174	6,13E-06
0,0062	0	5,00E-04	0	0	0	0	0	9,00E-04	0	266	0,001747	152224	4,00E-04
0,0015	0	6,00E-04	0	0	9,00E-04	0	2,94E-05	0,0014	0	86	0,000565	152232	3,00E-04
0	0	6,55E-05	0	0	0	0	1,47E-05	0	0	2	1,31E-05	152242	2,03E-05
0	0	6,54E-05	0	0	0	0	0	0	0	1	6,57E-06	152192	0
0,0012	0,0022	9,00E-04	0	0	0,005	0	0,0067	0,0014	9,00E-04	575	0,0038	151308	0,0023
0,0015	0,0022	9,00E-04	0	0	0,0049	0	0,0067	0,0014	8,00E-04	588	0,003873	151822	0,0023
0	0	0	0	0	0	0	0	0	0	0	0	0	0
0	0	0	0	0	0	0	0	0	0	0	0	0	5,59E-06
0	0	6,62E-05	0	0	0	0	0	0	0	1	6,66E-06	150244	0
0	0	0	0	0	0	0	0	0	0	0	0	0	0
0	0	2,00E-04	0	0	0	0	1,49E-05	0	0	4	2,68E-05	149478	0
0	0	0	0	0	0	0	0	0	0	0	0	0	4,07E-06
0	0	0	0	0	0	0	0	0	0	0	0	0	0
4,00E-04	0	2,00E-04	0	0	0	0	2,94E-05	0	0	22	0,000145	152024	1,00E-04
2,41E-05	0	0	0	4,00E-04	0	0	4,41E-05	5,00E-04	0	7	4,61E-05	151774	8,23E-05
2,44E-05	0	0	0	0	0	0	0	0	0	1	6,61E-06	151204	8,48E-06
7,24E-05	0	6,55E-05	0	0	9,41E-05	0	4,41E-05	0	0	8	5,26E-05	152156	5,71E-05
0	0	1,00E-04	0	4,00E-04	9,41E-05	0	1,00E-04	0	8,00E-04	18	0,000118	152084	2,00E-04
0	0	0	0	0	0	0	0	0	0	0	0	0	0
0	0	0	0	0	0	0	1,47E-05	0	0	1	6,57E-06	152144	8,14E-06
2,41E-05	0	0	0	0	0	0	4,41E-05	0	0	4	2,63E-05	152234	0
0	0	0	0	0	0	0	0	0	0	0	0	0	0
0	0	6,55E-05	0	0	0	0	8,82E-05	5,00E-04	0	8	5,26E-05	152226	3,53E-05
0	0	6,54E-05	0	0	0	0	4,41E-05	0	0	4	2,63E-05	152182	8,13E-06
0,0019	0	6,54E-05	0	0	0	0	0	0	0	78	0,000513	152184	1,00E-04
7,22E-05	0	6,55E-05	0	0	0	0	0	0	0	3	1,97E-05	152070	2,49E-05
2,41E-05	0	3,00E-04	0,0052	0	0	0	5,00E-04	5,00E-04	2,00E-04	63	0,000414	152112	4,00E-04
0,003	0	6,55E-05	0	0	0	0	1,47E-05	0	2,00E-04	128	0,000841	152128	3,00E-04
0	0	0	0	0	0	0	0	0	0	0	0	0	4,08E-06
0	0	6,56E-05	0	0	0	0	1,47E-05	0	0	2	1,32E-05	152074	8,14E-06
0	0	0	0	0	0	0	0	0	0	0	0	0	0
5,00E-04	0	1,00E-04	0	0	3,00E-04	0	1,47E-05	0	0	26	0,000171	152094	7,76E-05

0	0	0	0	0	0	0	0	0	0	0	0	0	0
2,42E-05	0	6,55E-05	0	0	0	0	7,35E-05	0	0	7	4,60E-05	152058	3,25E-05
2,41E-05	0	0	0	0	0	0	2,94E-05	0	0	3	1,97E-05	152126	2,19E-05
4,00E-04	0	5,00E-04	0	0	2,00E-04	0	0,0013	5,00E-04	0	113	0,000743	152096	7,00E-04
0	0	2,00E-04	3,00E-04	0,0014	0	0	2,00E-04	5,00E-04	0	25	0,000169	148092	2,77E-05
0	0	2,00E-04	3,00E-04	0,0014	0	0	2,00E-04	5,00E-04	0	25	0,000171	146574	2,82E-05
2,41E-05	0	0	0	2,00E-04	9,42E-05	0	1,00E-04	0	2,00E-04	13	8,54E-05	152158	6,13E-05
2,41E-05	0	0	0	6,00E-04	0	0	0	0	0	4	2,63E-05	152216	3,26E-05
0	0	0	0	0	0	0	0	0	0	0	0	0	0
9,62E-05	0	0	0	0	9,41E-05	0	1,00E-04	5,00E-04	0	15	9,86E-05	152202	2,00E-04
2,50E-05	0	0	0	0	0	0	2,00E-04	0	0	12	8,25E-05	145470	8,47E-05
0	0	0	0	0	0	0	0	0	0	0	0	0	0
0	0	0	0	0	0	0	0	0	0	0	0	0	1,63E-05
0	0	0	0	0	0	0	0	0	0	0	0	0	4,61E-06
0	0	0	0	0	1,00E-04	0	0	0	0	0	0	0	8,60E-06
0	0	0	0	0	0	0	0	0	0	0	0	0	0
0	0	0	0	0	0	0	0	0	0	0	0	0	0
0	0	0	0	0	0	0	2,00E-04	0	0	11	7,24E-05	151948	5,84E-05
0	0	0	0	0	0	0	0	0	0	0	0	0	0
4,00E-04	0,0187	3,00E-04	0	0	0,0203	0	0,0041	0,0019	0	537	0,003533	151994	0
2,00E-04	0	8,00E-04	0	0	9,41E-05	0	0,001	9,00E-04	2,00E-04	91	0,000598	152222	8,00E-04
7,24E-05	0	0	0	0	0	0	0	0	0	3	1,97E-05	152224	4,07E-06
0	0	0	0	0	0	0	0	0	0	0	0	0	0
4,83E-05	0	0	0	0	0	0	2,00E-04	0	2,00E-04	15	9,86E-05	152162	3,25E-05
0	0	0	0	0	0	0	0	0	0	0	0	0	0
0	0	0	0	0	0	0	0	0	0	0	0	0	0
0	0	0	0	0	0	0	0	0	0	0	0	0	0
2,41E-05	0	0	0	0,0021	0	0	5,88E-05	5,00E-04	0	17	0,000112	152136	1,00E-04
5,00E-04	0	6,55E-05	0	2,00E-04	0	0	1,47E-05	5,00E-04	0	26	0,000171	152022	5,28E-05
0	0	0	0	0	0	0	0	0	0	0	0	0	0
0	0	0	0	0	0	0	0	0	0	0	0	0	0
0	0	0	0	0	0	0	0	0	0	0	0	0	0
2,42E-05	0	0	0	0	0	0	5,88E-05	0	0	5	3,29E-05	152120	2,85E-05
0,0011	0	6,83E-05	0	0	0	0	0	0	0	46	0,000309	149034	6,93E-05
2,42E-05	0	0	0	0	0	0	4,41E-05	0	0	4	2,63E-05	152062	1,22E-05
7,24E-05	0	0	0	2,00E-04	0	0	1,47E-05	0	0	5	3,29E-05	152204	1,63E-05

0	0	0	0	0	0	0	0	0	0	0	0	0	0
0	0	0	0	0	0	0	0	0	0	0	0	0	0
0	0	6,55E-05	0	0	0	0	0	0	0	1	6,57E-06	152168	0
4,81E-05	0	0,0011	0	0	0,0056	0	2,00E-04	0,0014	0	93	0,000611	152264	6,00E-04
0	0	0	0	0	0	0	0	0	0	0	0	0	1,22E-05
0,0017	0	6,59E-05	0	0	0	0	0	0	0	72	0,000476	151294	1,00E-04
0	0	0	0	0	0	0	0	0	0	0	0	0	4,91E-05
0	0	0	0	0	0	0	1,47E-05	0	0	1	6,57E-06	152120	2,84E-05
0	0	0	0	0	0	0	0	0	0	0	0	0	0
0	0	0	0	0	0	0	0	0	0	0	0	0	0
0	0	0	0	0	0	0	0	0	0	0	0	0	7,72E-05
0	0	0	0	0	0	0	0	0	0	0	0	0	0
0	0	0	0	0	0	0	0	0	0	0	0	0	0
0	0	0	0	0	0	0	0	0	0	0	0	0	1,22E-05
3,00E-04	0	0	0	0	9,41E-05	0	5,00E-04	0	0	45	0,000296	152206	3,00E-04
0	0	0	0	0	0	0	0	0	0	0	0	0	4,07E-06
0	0	0	0	0	0	0	1,47E-05	5,00E-04	0	2	1,31E-05	152236	0
0	0	0	0	0	0	0	0	0	0	0	0	0	0
2,41E-05	0	0	0	0	0	0	5,88E-05	0	0	5	3,29E-05	152124	4,94E-05
0	0	0	0	0	0	0	0	0	0	0	0	0	1,63E-05
0	0	0	0	0	0	0	0	0	0	0	0	0	0
0	0	0	0	0	0	0	0	0	0	0	0	0	0
0	0	0	0	0	0	0	2,94E-05	0	0	2	1,32E-05	152058	1,62E-05
0	0	0	0	2,00E-04	0	0	0	0	0,0029	15	9,85E-05	152234	2,00E-04
9,64E-05	0	0	0	0	8,00E-04	0	2,00E-04	5,00E-04	0	26	0,000171	152080	3,00E-04
0	0	0	0	0	0	0	1,47E-05	0	0	1	6,57E-06	152208	2,44E-05
0	0	0	0	0	0	0	0	0	0	0	0	0	0
0	0	0	0	0	0	0	0	0	0	0	0	0	0
2,00E-04	0	0	0	0	0	0	0	0	2,00E-04	10	6,58E-05	152084	3,28E-05
0	0	0	0	0	0	0	0	0	0	0	0	0	0
0	0	0	0	0	0	0	0	0	0	0	0	0	4,12E-06
0	0	0	0	0	0	0	1,47E-05	0	0	1	6,57E-06	152176	4,06E-06
0	0	0	0	0	0	0	0	0	0	0	0	0	0
0	0	0	0	0	0	0	0	0	0	0	0	0	0
0	0	0	0	0	0	0	0	0	0	0	0	0	4,08E-06
0	0	0	0	0	0	0	0	0	0	0	0	0	0

0	0	0	0	0	0	0	0	0	0	0	0	0	0
0	0	0	0	0	0,0014	0	4,41E-05	0	2,00E-04	19	0,000125	152016	7,31E-05
0	0	0	0	0	0	0	0	0	0	0	0	0	0
0	0	2,00E-04	0	0	0	0	2,94E-05	5,00E-04	0	6	3,94E-05	152118	8,18E-06
0,0021	0	6,54E-05	0	0	0	0	0	5,00E-04	0	89	0,000585	152190	1,00E-04
0	0	6,55E-05	0	0	0	0	0	0	0	1	6,58E-06	152042	4,09E-06
0,0312	0	0,0052	0	0	0	0,0068	3,00E-04	0,009	6,00E-04	1416	0,009319	151940	0
2,00E-04	0	0	0	0	0	0	0	0	0	2	1,31E-05	152194	8,42E-06
2,00E-04	0	6,00E-04	0,002	8,00E-04	0	0	2,00E-04	0,0014	0,001	47	0,000309	152186	4,00E-04
0	0	0	0	0	0	0	0	0	0	0	0	0	2,00E-04
0	0	0	0	0	0	0	0	0	0	0	0	0	0
0	0	0	0	0	0	0	0	0	0	0	0	0	8,13E-06
0	0	0	0	0	0	0	0	0	0	0	0	0	4,16E-06
0	0	0	0	0	0	0	0	0	0	0	0	0	0
4,81E-05	0	1,00E-04	0	0	0	0	2,00E-04	5,00E-04	2,00E-04	17	0,000112	152228	6,95E-05
0	0	0	0	0	0	0	1,47E-05	0	0	1	6,57E-06	152156	4,08E-06
0	0	0	0	0	0	0	0	0	0	0	0	0	0
0	0	0	0	0	0	0	0	0	0	0	0	0	0
0	0	0	0	0	0	0	0	0	0	0	0	0	2,94E-05
1,00E-04	0	0,0018	0,0023	0	9,45E-05	0	6,00E-04	5,00E-04	4,00E-04	85	0,000559	152092	8,00E-04
0	0	0	0	0	0	0	0	0	0	0	0	0	0
0	0	0	0	0	0	0	0	0	0	0	0	0	0
2,41E-05	0	1,00E-04	0	2,00E-04	0	0	1,00E-04	0	2,00E-04	13	8,54E-05	152230	2,73E-05
0	0	0	0	0,004	0	0	0	0	0	21	0,000138	152238	1,00E-04
0	0	0	0	0	0	0	0	0	0	0	0	0	0
2,41E-05	0	2,00E-04	0,0035	0	0	0,0034	2,00E-04	5,00E-04	0,0025	42	0,000276	152174	7,00E-04
2,42E-05	0	2,00E-04	0	0	0	0	0	0	0	4	2,63E-05	151994	5,31E-05
0	0	0	0	0	0	0	1,47E-05	0	0	1	6,58E-06	151962	8,94E-06
0	0	0	0	0	0	0	0	0	0	0	0	0	4,08E-06
2,00E-04	0	2,00E-04	3,00E-04	0	0	0	9,00E-04	0	2,00E-04	76	0,000499	152212	5,00E-04
2,41E-05	0	2,00E-04	0	0	0	0	2,94E-05	0	6,00E-04	9	5,91E-05	152186	4,47E-05
0	0	0	0	0	0	0	0	0	0	0	0	0	0
0	0	0	0	0	0	0	0	0	0	0	0	0	0
0	0	0,001	0	0	0	0	0	9,00E-04	0	18	0,000118	152050	0
0	0	0	0	0	0	0	0	0	0	0	0	0	0
0	0	0	0	0	0	0	0	0	0	0	0	0	5,20E-06

0	0	0	0	0	0	0	0	0	0	0	0	0	1,24E-05
0	0	0	0	0	0	0	0	0	0	0	0	0	9,95E-06
0	0	6,54E-05	0	0	0	0	0	0	0	1	6,57E-06	152190	0
4,81E-05	0	1,00E-04	0	0	0	0	1,00E-04	5,00E-04	0,0012	19	0,000125	152182	3,00E-04
0	0	0	0	8,00E-04	0	0	0	0	0	4	2,63E-05	152108	8,32E-06
9,64E-05	0	4,00E-04	0	0	0	0	1,47E-05	0	0	10	6,58E-05	151948	3,33E-05
0	0	0	0	0	0	0	0	0	0	0	0	0	0
0	0	0	0	0	0	0	0	0	0	0	0	0	4,06E-06
2,41E-05	0	6,54E-05	0	0	0	0	2,94E-05	0	0	4	2,63E-05	152194	1,62E-05
0	0	0	0	0	0	0	0	0	0	0	0	0	4,06E-06
0	0	0	0	0	0	0	0	0	0	0	0	0	0
2,42E-05	0	0	0	0	0	0	0	0	0	1	6,57E-06	152116	1,28E-05
3,00E-04	0	0	0	0	0	0	0	0	0	14	9,20E-05	152162	2,85E-05
0	0	0	0	0	0	0	2,94E-05	0	0	2	1,31E-05	152260	3,66E-05
0	0	0	0	0	0	0	0	0	2,00E-04	1	6,58E-06	152026	9,92E-06
2,41E-05	0	1,00E-04	0	2,00E-04	6,00E-04	0	5,88E-05	0	2,00E-04	15	9,86E-05	152190	1,00E-04
0	0	0	0	0	0	0	0	0	0	0	0	0	4,30E-06
0	0	0	0	0	0	0	0	0	0	0	0	0	0
0	0	3,00E-04	0	7,00E-04	0	0	0	0,0027	0	3	0,000119	25200	1,00E-04
0,0015	0	3,00E-04	0	0	0	0	0	5,00E-04	0	67	0,000441	152066	6,63E-05
0	0	0	0	0	0	0	0	0	0	0	0	0	5,79E-06
6,00E-04	0	6,54E-05	0	0	0	0	1,47E-05	9,00E-04	0	30	0,000197	152170	5,83E-05
0	0	0	0	0	0	0	0	0	0	0	0	0	0
4,82E-05	0	0	0	0	0	0	1,47E-05	0	6,00E-04	5	3,29E-05	152142	6,18E-05
0	0	0	0	0	0	0	0	0	0	0	0	0	0
4,83E-05	0	6,55E-05	0	0	0	0	2,00E-04	0	0	14	9,20E-05	152138	1,36E-05
0	0	0	0	0	0	0	0	0	0	0	0	0	0
0	0	0	0	0	0	0	1,47E-05	0	0	1	6,57E-06	152266	1,62E-05
0,0079	0	5,00E-04	0	0	0	0	4,41E-05	0,0014	0	342	0,002248	152166	6,00E-04
0,0033	0	7,00E-04	0	6,00E-04	0	0	5,88E-05	5,00E-04	0	156	0,001025	152188	3,00E-04
0	0	6,00E-04	0	0	0	0	0	0	0	9	5,91E-05	152204	5,69E-05
0	0	0	0	0	0	0	0	0	0	0	0	0	0
0	0	0	0	0	0	0	0	0	0	0	0	0	0
0	0	0	0	0	0	0	1,47E-05	0	0	1	6,57E-06	152168	1,63E-05
0	0	0	0	0	0	0	0	0	0	0	0	0	0
0	0	0	0	0	0	0	4,41E-05	0	0	3	1,97E-05	152222	2,52E-05

3,00E-04	0	6,55E-05	0	0	0	0	0	0	0	13	8,54E-05	152160	2,03E-05
0	0	0	0	0	0	0	2,94E-05	0	0	2	1,31E-05	152222	0
2,41E-05	0	3,00E-04	0	0	0	0	1,00E-04	0	0	13	8,55E-05	152134	9,75E-05
0,0034	0	1,00E-04	0	0	0	0	0	0,0019	0	146	0,000961	151948	1,00E-04
0,0064	0	0,0011	0	0	0	0,0034	0	0,0019	0	285	0,001874	152084	4,00E-04
0,0038	0	0	0	0	0	0	0	5,00E-04	0	157	0,001032	152114	2,00E-04
0	0	0	0	0	0	0	0	0	0	0	0	0	0
0	0	0	0	0	0	0	0	0	0	0	0	0	4,06E-06
0	0	3,00E-04	0	0	0	0	5,88E-05	0	0	8	5,26E-05	152138	1,22E-05
0	0	0	0	0	0	0	0	0	0	0	0	0	0
0	0	0	0	0	0	0	1,47E-05	0	2,00E-04	2	1,31E-05	152184	1,63E-05
2,41E-05	0	0	0	0	0	0	0	0	0	1	6,57E-06	152194	8,12E-06
5,00E-04	0	5,00E-04	0	0	0	0	1,47E-05	0	0	28	0,000184	152212	6,50E-05
0	0	3,00E-04	0	0	0	0	0	0	0	4	2,63E-05	152240	1,00E-04
4,00E-04	0	0	0	0	0	0	0	0	0	18	0,000118	152154	4,47E-05
0	0	0	0	0	0	0	7,35E-05	0	0	5	3,29E-05	152190	3,66E-05
0	0	0	0	0	0	0	0	0	0	0	0	0	0
0,0014	0	6,53E-05	0	0	0	0	1,47E-05	0	0	62	0,000407	152232	0
0	0	0	0	0	0	0	0	0	0	0	0	0	4,06E-06
0	0	0	0	0	0	0	7,35E-05	0	0	5	3,28E-05	152218	0
4,83E-05	0	0	0	0	0	0	0	0	0	2	1,31E-05	152178	0
1,00E-04	0,0923	7,00E-04	0,0029	0	0	0	3,00E-04	5,00E-04	0	129	0,000848	152034	4,00E-04
0	0	0	0	0	0	0	0	0	0	0	0	0	4,06E-06
0	0	0	0	0	0	0	0	0	0	0	0	0	0
2,41E-05	0	6,54E-05	0	2,00E-04	0	0	1,00E-04	0	0	10	6,57E-05	152164	5,29E-05
2,41E-05	0	0	0	0	0	0	1,47E-05	0	0	2	1,31E-05	152242	2,69E-05
0	0	0	0	0	0	0	4,41E-05	0	0	3	1,97E-05	152074	1,52E-05
2,41E-05	0	0	0	2,00E-04	0	0	2,94E-05	0	6,00E-04	7	4,60E-05	152154	4,11E-05
0	0	0	0	0	0	0	0	0	0	0	0	0	0
0	0	0	0	0,0037	0	0	5,88E-05	0	0	23	0,000151	152234	2,00E-04
2,42E-05	0	0	0	0	0	0	0	0	0	1	6,57E-06	152122	0
0	0	0	0	0	0	0	1,47E-05	0	0	1	6,57E-06	152134	1,16E-05
0	0	0	0	0	0	0	0	0	0	0	0	0	0
0	0	0	0	0	0	0	0	0	0	0	0	0	0
0	0	0	0	0	0	0	0	0	0	0	0	0	0
0	0	0	0	0	0	0	1,47E-05	0	0	1	6,57E-06	152220	1,44E-05

0	0	0	0	0	0	0	0	0	0	0	0	0	0
7,22E-05	0	8,00E-04	0	0	0	0	7,00E-04	9,00E-04	0	69	0,000453	152224	4,00E-04
0	0	0	0	0	0	0	0	0	0	0	0	0	0
0	0	0	0	0	0	0	0	0	0	0	0	0	4,08E-06
2,41E-05	0	0	0	0	0	0	2,94E-05	0	0	3	1,97E-05	152238	1,71E-05
0	0	0	0	0	0	0	1,47E-05	0	2,00E-04	2	1,31E-05	152210	1,62E-05
0	0	0	0	0	0	0	2,94E-05	0	0	2	1,31E-05	152136	1,62E-05
0	0	0	0	0	0	0,0034	1,47E-05	0	0	2	1,31E-05	152274	8,97E-06
2,42E-05	0	1,00E-04	0	0	9,46E-05	0	5,89E-05	0	0	8	5,27E-05	151916	1,00E-04
0	0	0	0	0	0	0	0	0	0	0	0	0	0
0	0	6,55E-05	0	0	0	0	0	0	0	1	6,57E-06	152150	4,10E-06
3,00E-04	0	6,55E-05	9,00E-04	0	0,0016	0	0,0011	0	0,001	112	0,000737	152032	8,00E-04
5,00E-04	0	6,55E-05	0	0	0	0	0	0	0	20	0,000131	152150	4,48E-05
0	0	0	0	0	0	0	0	0	0	0	0	0	2,07E-05
0	0	0	0,0029	0	0	0	1,00E-04	0	0	20	0,000131	152216	1,00E-04
0	0	0	0	0	0	0	0	0	0	0	0	0	0
2,41E-05	0	0	0	6,00E-04	0	0	1,47E-05	0	0	5	3,29E-05	152200	1,22E-05
0	0	1,00E-04	0	0	0	0	2,94E-05	5,00E-04	0	5	3,29E-05	152132	1,63E-05
0	0	0	0	0	0	0	1,47E-05	0,001	0	3	1,97E-05	152006	4,27E-06
0	0	0	0	0	0	0	0	0	0	0	0	0	0
7,24E-05	0	0	0	0	0	0	0	0	0	3	1,97E-05	152212	0
6,00E-04	0,0186	2,00E-04	3,00E-04	0	0,0025	0	0,0033	9,00E-04	8,00E-04	304	0,001997	152256	0,0018
0	0	0	0	0	0	0	1,47E-05	0	0	1	6,58E-06	152064	4,09E-06
0	0	0	0	0	0	0	0	0	0	0	0	0	0
0,0019	0	0	0	0	0	0	1,47E-05	0	0	80	0,000526	152188	1,00E-04
0	0	0	0	0	0	0	0	0	0	0	0	0	8,13E-06
0	0	0	0	0	0	0	1,47E-05	0	0	1	6,58E-06	152068	2,16E-05
7,24E-05	0	0	0	0	0	0	1,47E-05	0	0	4	2,63E-05	152110	1,22E-05
0	0	1,00E-04	0	0	0	0	0	0	0	2	1,31E-05	152136	0
0	0	0	0	0	0	0	0	0	0	0	0	0	0
0	0	6,55E-05	0	0	0	0	0	0	0	1	6,57E-06	152112	0
0	0	6,55E-05	0	0	0	0	2,94E-05	0	0	3	1,97E-05	152022	1,81E-05
0	0	6,55E-05	0	0	0	0	0	0	0	1	6,57E-06	152118	4,07E-06
4,81E-05	0	0	0	0	0	0	4,41E-05	0	0	5	3,29E-05	152104	2,46E-05
2,41E-05	0	6,53E-05	0,0023	0,0017	0	0	2,94E-05	0	0	21	0,000138	152194	4,00E-04
8,00E-04	0	0	0	0	0	0	0	0	0	32	0,00021	152146	7,73E-05

0	0	0	0	0	0	0	0	0	0	0	0	0	1,22E-05
0	0	0	0	0	0	0	0	0	0	0	0	0	0
0	0	0	0	0	0	0	0	0	0	0	0	0	0
2,42E-05	0	0	0	0	0	0	0	0	0	1	6,61E-06	151292	0
0	0	0	0	0	0	0	0	0	0	0	0	0	8,13E-06
4,83E-05	0	1,00E-04	0	0	0	0	4,41E-05	0	0	7	4,60E-05	152172	2,08E-05
2,42E-05	0	2,00E-04	0	0	0	0	4,41E-05	0	0	7	4,60E-05	152140	6,09E-05
0	0	1,00E-04	0	0	0	0	0	0	0	2	1,31E-05	152150	0
0	0	0	0	2,00E-04	0	0	0	0	0	1	6,57E-06	152200	1,22E-05
0	0	0	0	0	0	0	4,41E-05	0	0	3	1,97E-05	152026	1,64E-05
0	0	6,55E-05	0	0	0	0	0	0	0	1	6,57E-06	152118	1,22E-05
0	0	0	0	0	0	0	0	0	0	0	0	0	4,55E-06
0	0	0	0	0	0	0	0	0	0	0	0	0	0
0	0	0	0	0	0	0	0	0	0	0	0	0	0
0	0	0	0	0	0	0	0	0	0	0	0	0	0
0	0	0	0	0	0	0	0	0	0	0	0	0	0
0	0	0	0	0	0	0	0	0	0	0	0	0	0
0	0	0	0	0	0	0	1,00E-04	0	2,00E-04	10	6,57E-05	152166	7,72E-05
4,82E-05	0	6,54E-05	0	0	0	0	1,00E-04	0	0	11	7,23E-05	152086	8,53E-05
0	0	2,00E-04	0	0	0	0	4,41E-05	0	0	6	3,94E-05	152182	2,03E-05
0	0	0	0	0	0	0	0	0	0	0	0	0	8,12E-06
0	0	0	0	0	0	0	0	0	0	0	0	0	0
2,00E-04	0	3,00E-04	0	0	0	0	0,0011	0	0	90	0,000594	151632	5,00E-04
4,82E-05	0	0	0	0	0	0	0	0	0	2	1,31E-05	152226	4,13E-06
0	0	0	0	0	0	0	0	0	0	0	0	0	0
9,65E-05	0	0	0	0	0	0	0	0	0	4	2,63E-05	152252	8,54E-06
0	0	0	0	0	0	0	0	0	0	0	0	0	0
0	0	0	0	0	0	0	0	0	0	0	0	0	0
0	0	0	0	0	0	0	2,94E-05	0	2,00E-04	3	1,97E-05	152122	2,44E-05
0	0	0	0	0	0	0	0	0	0	0	0	0	0
0	0	0	0	0	0	0	0	0	0	0	0	0	4,06E-06
0	0	0	0	0	0	0	0	0	0	0	0	0	0
0	0	0	0	0	0	0	0	0	0	0	0	0	0
2,42E-05	0	1,00E-04	0	0	0	0	0	0	0	3	1,97E-05	152108	7,77E-05
0	0	0	0	0	0	0	0	0	0	0	0	0	0
6,00E-04	0	6,53E-05	0	0	0	0	1,47E-05	0	0	26	0,000171	152196	6,06E-05

0	0	0	0	0	0	0	0	0	0	0	0	0	0
0	0	0	9,00E-04	0	0	0	0	0	0	3	1,97E-05	152210	3,86E-05
0	0	0	0	0	0	0	0	0	0	0	0	0	0
7,24E-05	0	0	0	0	0	0	1,00E-04	0	0	10	6,57E-05	152164	1,00E-04
0	0	0	0	0	0	0	0	0	0	0	0	0	0
1,00E-04	0	0	0	2,00E-04	0	0	3,00E-04	0	0	29	0,000191	152228	2,00E-04
0	0	0	0	0	0	0	0	0	0	0	0	0	0
0	0	0	0	0	0	0	0	0	0	0	0	0	0
0	0	0	0	0	0	0	0	0	0	0	0	0	0
0	0	0	0	0	0	0	4,41E-05	0	0	3	1,97E-05	152194	1,64E-05
0	0	0	0	0	0	0	0	0	0	0	0	0	0
0	0	0	0	0	0	0	0	0	0	0	0	0	0
0	0	0	0	0	0	0	0	0	0	0	0	0	4,06E-06
1,00E-04	0	6,55E-05	0	0	0	0	0	0	0	7	4,60E-05	152162	2,03E-05
9,63E-05	0	6,54E-05	0	0	0	0	5,00E-04	0	0	39	0,000256	152210	2,00E-04
0	0	0	0	0	0	0	0	0	0	0	0	0	0
9,66E-05	0	0	0	0	0	0	0	0	0	4	2,63E-05	152086	8,12E-06
0	0	0	0	0	2,00E-04	0	0	0	0	2	1,32E-05	151938	4,06E-05
2,42E-05	0	0	0	0	0	0	0	0	0	1	6,57E-06	152092	1,22E-05
0	0	0	0	0	0	0	0	0	0	0	0	0	4,06E-06
0	0	0	0	0	0	0	0	0	0	0	0	0	0
9,00E-04	0	3,00E-04	0	0	0	0	0	9,00E-04	0	44	0,000289	152086	0
0	0	0	0	0	0	0	0	0	0	0	0	0	4,06E-06
2,41E-05	0	0	0	0	0	0	0	0	0	1	6,57E-06	152138	0
0	0	0	0	0	0	0	0	0	0	0	0	0	0
1,00E-04	0	6,53E-05	0	0	0	0	0	0	0	5	3,28E-05	152234	0
0	0	0	0	0	0	0	0	0	0	0	0	0	0
0	0	0	0	0	0	0	0	0	0	0	0	0	4,22E-06
0	0	0	0	0	0	0	0	0	0	0	0	0	0
0	0	0	0	0	0	0	1,00E-04	0	0	9	6,07E-05	148256	0
0	0	0	0	0	0	0	0	0	0	0	0	0	0
0	0	0	0	0	0	0	0	0	0	0	0	0	4,29E-06
0	0	0	0	0	0	0	0	0	0	0	0	0	0
0	0	0	0	0	0	0	0	0	0	0	0	0	0
7,24E-05	0	0	6,00E-04	0	0	0	5,88E-05	5,00E-04	0	10	6,57E-05	152130	4,07E-05
4,83E-05	0	0	0	0	0	0	0	0	0	2	1,31E-05	152204	0

9,64E-05	0,0044	0	0	0	0	0	8,00E-04	0	0	62	0,000408	151978	4,00E-04
0	0	0	0	0	0	0	0	0	0	0	0	0	0
0	0	0	0	0	0	0	0	0	0	0	0	0	0
0	0	0,0027	0	0	0	0	2,94E-05	0	0	43	0,000283	152208	4,47E-05
0	0	0	0	0	0	0	0	0	0	0	0	0	0
0	0	0	0	0	0	0	0	0	0	0	0	0	0
3,00E-04	0,0208	3,00E-04	0	0	0,0021	0	0,0017	0,001	0	177	0,001163	152204	9,00E-04
5,00E-04	0	6,54E-05	0	0	0	0	0	0	0	20	0,000131	152220	3,71E-05
4,81E-05	0	6,53E-05	0	0	3,00E-04	0	2,00E-04	0	0	23	0,000151	152190	1,00E-04
0	0	0	0	0	0	0	0	0	0	0	0	0	0
0	0	0	0	0	0	0	0	0	0	0	0	0	0
2,41E-05	0	0	0	0	0	0	2,94E-05	0	0	3	1,97E-05	152194	2,52E-05
1,00E-04	0	0	0	0	0	0	0	0	0	5	3,29E-05	152180	1,22E-05
1,00E-04	0,0022	6,00E-04	9,00E-04	0	0,0012	0	0,0031	0,0019	0	250	0,001643	152198	0,0015
0	0	0	0	0	0	0	0	0	0	0	0	0	4,06E-06
0	0	0	0	0,0021	0	0	0	0	2,00E-04	12	7,88E-05	152198	1,00E-04
4,83E-05	0	0	0	0	0	0	0	0	0	2	1,31E-05	152124	0
0	0	0	0	0	0	0	0	0	0	0	0	0	0
0	0	0	0	0	0	0	2,98E-05	0	0	2	1,33E-05	150378	4,06E-05
0	0	0	0	0	0	0	0	0	0	0	0	0	4,06E-06
0	0	3,00E-04	0	0	0	0	1,47E-05	0	0	5	3,29E-05	152174	2,05E-05
0	0	5,00E-04	0	2,00E-04	0	0	2,94E-05	0	0	11	7,23E-05	152246	2,00E-04
0	0	0	0	0	0	0	0	0	0	0	0	0	0
0	0	6,55E-05	0	0	0	0	0	0	0	1	6,58E-06	152072	0
0	0	0	0	0	0	0	0	0	0	0	0	0	0
0	0	0	0	0	0	0	0	0	0	0	0	0	0
0	0	0	0	0	0	0	1,47E-05	0	0	1	6,62E-06	151152	9,63E-06
0	0	0	0	0	0	0	0	0	0	0	0	0	0
0	0	0	0	0	0	0	0	0	0	0	0	0	0
0	0	0	0	0	0	0	0	0	0	0	0	0	0
0	0	0	0	0	0	0	0	0	0	0	0	0	7,71E-06
0	0	0	0	0	0	0	0	0	0	0	0	0	0
0	0	0	0	2,00E-04	0	0	2,94E-05	0	0	3	1,97E-05	152176	8,42E-06
2,41E-05	0	0	0	0	0	0	8,82E-05	0	0	7	4,60E-05	152182	4,09E-06
0	0	5,00E-04	0	0	0	0	2,00E-04	0	2,00E-04	20	0,000131	152162	8,94E-05
0	0	0	0	0	0	0	0	0	0	0	0	0	0

2,41E-05	0	0	0	0	0	0	0	0	0	1	6,57E-06	152160	0
9,67E-05	0	0	0	0	0	0	1,47E-05	0	0	5	3,29E-05	152014	1,65E-05
4,83E-05	0	0	0	0	0	0	1,47E-05	0	0	3	1,97E-05	152080	3,66E-05
2,00E-04	0	3,00E-04	0,002	2,00E-04	0	0	3,00E-04	5,00E-04	2,00E-04	46	0,000302	152146	0
7,23E-05	0	0	0	0	0	0	7,35E-05	0	0	8	5,26E-05	152236	1,22E-05
0	0	0	0	0	0	0	0	0	2,00E-04	1	6,57E-06	152134	3,25E-05
0	0	1,00E-04	0	0,0012	0	0	2,94E-05	0	2,00E-04	11	7,23E-05	152208	2,00E-04
0	0	0	0	0	0	0	0	0	0	0	0	0	0
0	0	0	0	0	9,42E-05	0	0	0	0	1	6,57E-06	152196	0
6,00E-04	0	1,00E-04	0	0	0	0	2,94E-05	0	0	30	0,000198	151844	6,50E-05
0	0	0	0	0	0	0	0	0	0	0	0	0	0
2,41E-05	0	0	3,00E-04	0	0	0	1,00E-04	0	0	10	6,57E-05	152142	2,86E-05
0	0	0	0	0	0	0	0	0	0	0	0	0	1,18E-05
0	0	6,54E-05	0	0	0	0	1,47E-05	0	0	2	1,31E-05	152142	0
0	0	0	0	0	0	0	0	0	0	0	0	0	4,08E-06
0	0	3,00E-04	0	0	0	0	5,88E-05	0	0	9	5,91E-05	152198	2,44E-05
9,66E-05	0	0	0	0	0	0	0	0	0	4	2,63E-05	152144	2,46E-05
0	0	0	0	0	0	0	7,35E-05	0	0	5	3,28E-05	152232	2,03E-05
0	0	0	0	0	0	0	2,94E-05	0	0	2	1,31E-05	152160	4,06E-06
0	0	0	0	0	0	0	0	0	0	0	0	0	4,06E-06
0	0	0	0	0	0	0	0	0	0	0	0	0	8,23E-06
2,00E-04	0	0,001	0	0	0	0	6,00E-04	0,0019	0	68	0,000447	152042	5,00E-04
0	0	0	0	6,00E-04	0	0	0	0	0	3	2,08E-05	144558	1,80E-05
0	0	0	0	0	0	0	1,47E-05	0	0	1	6,57E-06	152268	0
0	0	2,00E-04	0	0	0	0	0	0	0	3	1,97E-05	152168	4,88E-05
0	0	0	0	0	0	0	0	0	0	0	0	0	2,94E-05
0	0	0	0	0	0	0	0	0	0	0	0	0	0
4,83E-05	0	0	0	0	0	0	1,47E-05	0	0	3	1,97E-05	152172	4,06E-06
0	0	0	0	0	0	0	0	0	0	0	0	0	0
0	0	0	0	0	0	0	0	0	0	0	0	0	0
1,00E-04	0	0	0	0	0	0	0	0	0	5	3,29E-05	152198	0
6,00E-04	0	0	0	0	0	0	0	0	2,00E-04	25	0,000164	152162	4,21E-06
0	0	0	0	0	0	0	0	0	0	0	0	0	0
0	0	0	0	0	2,00E-04	0	1,47E-05	0	0	3	1,97E-05	151992	8,32E-06
0	0	0	0	0	0	0	0	0	0	0	0	0	4,07E-06
0	0	0	0	0	0	0	0	0	0	0	0	0	0

0	0	0	0	0	0	0	0	0	0	0	0	0	0
0	0	0	0	0	0	0	0	0	0	0	0	0	0
4,84E-05	0	0	0	0	0	0	5,88E-05	0	0	6	3,95E-05	151926	2,85E-05
0	0	0	0	0	0	0	0	0	0	0	0	0	0
0	0	0	0	0	0	0	0	0	0	0	0	0	0
2,41E-05	0	0	0	0	0	0	2,94E-05	0	2,00E-04	4	2,63E-05	152222	3,23E-05
9,65E-05	0	0	0	0	0	0	0	0	0	4	2,63E-05	152210	1,68E-05
0	0	0	0	0	0	0	4,41E-05	0	0	3	1,97E-05	152236	4,10E-06
0	0	0	0	0	0	0	1,47E-05	0	0	1	6,57E-06	152180	1,22E-05
0	0	0	0	0	0	0	2,94E-05	0	0	2	1,31E-05	152180	5,69E-05
0	0	6,54E-05	0	0	0	0	1,47E-05	0	0	2	1,31E-05	152172	4,54E-05
0	0	0	0	0	0	0	0	0	0	0	0	0	4,54E-05
0	0	0	0	0	0	0	0	0	0	0	0	0	0
4,00E-04	0	6,54E-05	0	0	0	0	0	5,00E-04	0	17	0,000112	152164	6,10E-05
0	0	0	0	0	0	0	1,49E-05	0	0	1	6,94E-06	144060	0
0	0	0	0	0	0	0	1,00E-04	0	0	9	5,91E-05	152156	1,00E-04
6,00E-04	0	6,54E-05	0	0	0	0	0	0	0	27	0,000177	152156	3,59E-05
4,82E-05	0	0,001	0	0	0	0	3,00E-04	0	0	39	0,000257	152006	1,00E-04
0	0	0	0	0	0	0	0	0	0	0	0	0	0
0	0	0	0	0	0	0	0	0	0	0	0	0	0
0	0	0	0	0	0	0	1,47E-05	5,00E-04	0	2	1,32E-05	151868	0
0	0	0	0	0	0	0	0	0	0	0	0	0	4,06E-06
2,41E-05	0	0,0027	0	2,00E-04	0	0	8,82E-05	5,00E-04	0	51	0,000335	152162	2,00E-04
2,00E-04	0	0	0	0	0	0	1,47E-05	0	0,001	14	9,20E-05	152120	3,66E-05
3,00E-04	0	6,54E-05	0	0	0	0	0	5,00E-04	0	16	0,000105	152200	3,66E-05
0	0	0	0	0	0	0	0	0	0	0	0	0	6,61E-06
4,83E-05	0	6,55E-05	0	0	0	0	0	0	0	3	1,97E-05	152162	0
9,65E-05	0	0	6,00E-04	0	0	0	2,00E-04	0	0	20	0,000131	152188	1,00E-04
0	0	0	0	0	0	0	0	0	0	0	0	0	8,29E-06
0	0	0	0	0	0	0	0	0	0	0	0	0	0
0	0	0	0	0	9,42E-05	0	3,00E-04	0	0	22	0,000145	152188	2,00E-04
2,42E-05	0	0	0	0	0	0	2,94E-05	0	0	3	1,97E-05	152008	2,03E-05
0	0	0	0	0	0	0	0	0	0	0	0	0	0
0	0	0	0	0	0	0	0	0	0	0	0	0	0
0	0	0	0	0	0	0	0	0	0	0	0	0	0
0	0	0	0	0	0	0	0	0	0	0	0	0	4,33E-06

2,00E-04	0	0,0027	9,00E-04	0	0	0	0,0014	0,0014	4,00E-04	154	0,001012	152130	0,0011
0	0	6,55E-05	0	0	0	0	2,94E-05	0	0	3	1,97E-05	152158	8,13E-06
0	0	0	0	0	0	0	0	0	0	0	0	0	0
0,0011	0	4,00E-04	0	0	0	0	0	0,0014	0	53	0,000348	152104	5,28E-05
0	0	0	0	0	0	0	0	0	2,00E-04	1	6,57E-06	152156	1,63E-05
0	0	0	0	0	0	0	0	0	0	0	0	0	0
0	0	0	0	0	0	0	0	0	0	0	0	0	0
0	0	0	0	0	0	0	0	0	0	0	0	0	0
4,81E-05	0	2,00E-04	0	0	0	0	5,00E-04	9,00E-04	0	40	0,000263	152194	2,00E-04
2,41E-05	0	0	0	0	0	0	0	0	0	1	6,57E-06	152104	5,29E-05
0	0	0	0	0	0	0	0	0	0	0	0	0	0
0	0	1,00E-04	0	0	0	0	0	0	0	2	1,31E-05	152144	8,14E-06
0	0	0	0	0	0	0	0	0	0	0	0	0	8,49E-06
0	0	0	0	0	0	0	0	0	0	0	0	0	0
0	0	0	0	0	0	0	1,47E-05	0	0	1	6,59E-06	151722	4,43E-06
0	0	0	0	0	0	0	1,47E-05	0	0	1	6,58E-06	152046	4,19E-06
0	0	0	0	0	0	0	1,47E-05	0	0	1	6,57E-06	152142	4,31E-06
0,0078	0	7,00E-04	0	0	0	0	1,47E-05	9,00E-04	0	326	0,002142	152182	5,00E-04
2,41E-05	0	6,55E-05	0	0	0	0	1,47E-05	0	0	3	1,97E-05	152166	4,77E-06
0	0	0	0	0	0	0	0	0	0	0	0	0	0
0	0	0	0	0	0	0	0	0	0	0	0	0	4,11E-06
0	0	0	0	0	0	0	0	0	0	0	0	0	0
0	0	6,54E-05	0	0	0	0	0	0	0	1	6,57E-06	152176	0
0	0	0	0	0	0	0	0	0	0	0	0	0	0
0	0	0	0	0	0	0	0	0	0	0	0	0	0
0	0	0	0	0	0	0	0	0	0	0	0	0	0
1,00E-04	0	3,00E-04	0	0	0	0	2,00E-04	5,00E-04	0	25	0,000164	151980	1,00E-04
0	0	0	0	0	0	0	0	0	0	0	0	0	4,06E-06
0	0	0	0	0	0	0	0	0	0	0	0	0	8,20E-06
2,41E-05	0	2,00E-04	0	0	0	0	2,94E-05	0	0	6	3,94E-05	152158	6,50E-05
0	0	6,55E-05	0	0	0	0	0	0	0	1	6,57E-06	152100	0
5,00E-04	0	0	0	0	9,41E-05	0	0	0	0	20	0,000131	152132	6,92E-05
0	0	0	0	0	0	0	0	0	0	0	0	0	0
0	0	0	0	0	0	0	0	0	0	0	0	0	0
0	0	0	0	0	0	0	0	0	0	0	0	0	4,28E-06
0	0	0	0	0	0	0	0	0	0	0	0	0	4,26E-06

0	0	0	0	0	0	0	0	0	0	0	0	0	0
1,00E-04	0	6,71E-05	0	0,0022	0	0	8,95E-05	0	2,00E-04	24	0,000163	147060	3,73E-05
2,00E-04	0	6,54E-05	0	0	0	0	0	0	0	9	5,91E-05	152232	1,63E-05
0	0	0	0	0	0	0	1,47E-05	0	0	1	6,57E-06	152200	0
0	0	0	0	0	0	0	0	0	0	0	0	0	0
1,00E-04	0	0	0	0	0	0	2,94E-05	0	0	8	5,26E-05	152092	4,07E-05
4,83E-05	0	0	0	0	0	0	5,88E-05	0	0	6	3,94E-05	152126	8,14E-06
1,00E-04	0	0	0	0	0	0	0	5,00E-04	0	6	3,94E-05	152218	4,10E-05
0	0	0	0	0	0	0	0	0	0	0	0	0	0
2,41E-05	0	0	0	0	0	0	0	0	0	1	6,57E-06	152116	4,21E-06
0	0	0	0	0	0	0	0	0	0	0	0	0	0
0	0	0	0	0	0	0	0	0	0	0	0	0	0
2,42E-05	0	0	0	0	0	0	0	0	0	1	6,58E-06	152064	8,13E-06
0	0	0	0	0	0	0	0	0	0	0	0	0	0
0	0	0	0	0	0	0	0	0	0	0	0	0	4,06E-06
2,41E-05	0	0	0	0	0	0	0	0	0	1	6,57E-06	152140	1,71E-05
0	0	0	0	0	0	0	0	0	0	0	0	0	0
0	0	0	0	0	0	0	1,47E-05	0	0	1	6,57E-06	152194	8,20E-06
0	0	0	0	0	0	0	4,41E-05	5,00E-04	0	4	2,63E-05	152232	1,23E-05
0	0	0	0	0	0	0	1,47E-05	0	0	1	6,57E-06	152172	0
2,41E-05	0	0	0	0	0	0	5,88E-05	0	0	5	3,29E-05	152152	2,33E-05
0	0	0	0	0	0	0	0	0	0	0	0	0	4,10E-06
0	0	0	0	0	0	0	0	0	0	0	0	0	0
0,0015	0	9,00E-04	3,00E-04	0	0	0	9,95E-05	0,0018	0,0033	75	0,000615	122034	0
2,00E-04	0	6,54E-05	0	0	0	0	0	0	0	10	6,57E-05	152218	1,23E-05
0	0	0	0	0	0	0	8,82E-05	0	0	6	3,94E-05	152160	1,02E-05
0	0	0	0	0	0	0	8,82E-05	0	0	6	3,94E-05	152150	0
0	0	0	0	0	0	0	0	0	0	0	0	0	0
0	0	6,56E-05	0	0	0	0	4,41E-05	0	0	4	2,63E-05	152018	2,02E-05
0	0	6,56E-05	0	0	0	0	5,88E-05	0	0	5	3,29E-05	151912	5,38E-05
0	0	0	0	0	0	0	0	0	0	0	0	0	0
0	0	0	0	0	0	0	0	0	0	0	0	0	2,07E-05
0	0	0	0	0	0	0	0	0	0	0	0	0	0
2,00E-04	0	0	0	0	0	0	0	0	2,00E-04	8	5,26E-05	152194	2,44E-05
0	0	0	0	0	0	0	0	0	0	0	0	0	4,06E-06
0	0	0	0	0	0	0	0	0	0	0	0	0	0

0,0062	0	4,00E-04	0	0	0	0,0034	1,47E-05	5,00E-04	0	266	0,001752	151832	4,00E-04
0	0	0	0	0	0	0	0	0	0	0	0	0	0
0	0	0	0	0	0	0	0	0	2,00E-04	1	6,58E-06	152066	8,13E-06
0	0	0	0	0	0	0	0	0	0	0	0	0	0
0	0	0,001	9,00E-04	0	6,00E-04	0	2,00E-04	0	0	37	0,000243	152248	2,00E-04
4,81E-05	0	6,54E-05	0	8,00E-04	0	0	1,00E-04	0	6,00E-04	20	0,000131	152208	2,00E-04
0	0	2,00E-04	0	0	0	0,0034	1,47E-05	5,00E-04	0	6	3,95E-05	152028	3,31E-05
0	0	0	0	0	0	0	1,47E-05	0	0	1	6,57E-06	152204	4,06E-06
5,00E-04	0	6,54E-05	0	0	0	0	0	0	2,00E-04	22	0,000145	152134	3,87E-05
0	0	0	0	0	0	0	0	0	0	0	0	0	0
0	0	0	0	0	0	0	0	0	0	0	0	0	0
0	0	0	0	0,0031	0	0	0	0	0	16	0,000105	152162	3,00E-04
2,41E-05	0	1,00E-04	0	4,00E-04	0	0	7,35E-05	5,00E-04	0	11	7,23E-05	152220	1,00E-04
0	0	0	0	0	0	0	0	0	0	0	0	0	0
0	0	5,00E-04	0	0	0	0	0	0	0	7	4,60E-05	152098	1,47E-05
2,00E-04	0	0,0019	0	2,00E-04	0	0	6,00E-04	0,0019	0,0052	109	0,000716	152140	0,0011
2,00E-04	0	0,0017	0	0	2,00E-04	0	0,0011	5,00E-04	2,00E-04	109	0,000717	152008	7,00E-04
4,83E-05	0	0	0	0	0	0	0	0	0	2	1,31E-05	152148	6,99E-06
4,83E-05	0	0	0	0	0	0	1,47E-05	0	0	3	1,97E-05	152144	1,22E-05
2,42E-05	0	0	0	0	0	0	0	0	0	1	6,58E-06	152058	4,07E-06
2,42E-05	0	0	0	0	0	0	1,47E-05	0	0	2	1,31E-05	152136	1,22E-05
4,81E-05	0	6,00E-04	0	0	0	0	0	9,00E-04	0	13	8,54E-05	152222	7,74E-05
0	0	0	0	0	0	0	0	0	0	0	0	0	0
0	0	1,00E-04	0	0	0	0	1,47E-05	0	0	3	1,97E-05	152214	2,07E-05
0	0	0	0	0	0	0	0	0	0	0	0	0	0
0	0	0	0	0	0	0	0	0	0	0	0	0	0
0	0	0	0	0	0	0	0	0	0	0	0	0	0
0	0	0	0	0	0	0	0	0	0	0	0	0	0
0	0	6,56E-05	0	0	0	0	2,95E-05	0	0	3	1,98E-05	151708	0
0	0	0	0	0	0	0	0	0	0	0	0	0	0
0	0	0	0	0	0	0	0	0	0	0	0	0	0
0	0	0	0	0	0	0	0	0	0	0	0	0	0
0	0	0	0	0	0	0	0	0	0	0	0	0	0
2,41E-05	0	0	0	0	0	0	0	0	2,00E-04	2	1,31E-05	152210	4,08E-06
0	0	0	0	0	0	0	1,47E-05	0	0	1	6,57E-06	152238	0
1,00E-04	0	6,00E-04	0	0	0	0,0034	4,41E-05	5,00E-04	0	20	0,000132	152030	1,00E-04

9,66E-05	0	0	0	0	0	0	0	0	2,00E-04	5	3,29E-05	152112	7,31E-05
0	0	0	0	0	0	0	0	0	0	0	0	0	0
0	0	0	0	0	0	0	0	0	0	0	0	0	0
0	0	0	0	0	0	0	0	0	0	0	0	0	0
0	0,0066	0	0	0	0	0	1,47E-05	0	2,00E-04	8	5,27E-05	151758	2,90E-05
0	0	0	0	0	0	0	0	0	0	0	0	0	0
0	0	0	0	0	0	0	0	0	0	0	0	0	0
0	0	0	0	0	0	0	0	0	0	0	0	0	0
0	0	0	0	0	0	0	0	0	0	0	0	0	0
0	0	9,00E-04	0	0	0	0	0	0	0	14	9,20E-05	152140	1,63E-05
6,00E-04	0	0,0016	0	0	0	0	3,00E-04	0	4,00E-04	71	0,000486	146028	0
0	0	0	0	0	0	0	0	0	0	0	0	0	0
0	0	0	0	0	0	0	0	0	0	0	0	0	1,06E-05
4,86E-05	0	0	0	0	0	0	2,95E-05	0	0	4	2,64E-05	151230	6,81E-06
0	0	0	0	0	0	0	0	0	0	0	0	0	8,13E-06
0	0	0	0	0	0	0	0	0	0	0	0	0	2,85E-05
0	0	0	0	0	0	0	0	0	0	0	0	0	0
2,41E-05	0	2,00E-04	0	0,0012	0	0	7,35E-05	0	0	15	9,86E-05	152132	1,00E-04
6,00E-04	0	0,0011	0	0	9,00E-04	0	0,0026	0,0019	0	229	0,001505	152124	0,0012
2,41E-05	0	3,00E-04	0	0	0	0	7,35E-05	9,00E-04	0	12	7,89E-05	152114	8,82E-05
0	0	0	0	0	0	0	2,94E-05	0	0	2	1,31E-05	152196	1,82E-05
0	0	0	0	0	0	0	0	0	0	0	0	0	0
0	0	0	0	0	0	0	0	0	0	0	0	0	0
0	0	0	0	0	0	0	0	0	0	0	0	0	0
0	0	0	0	0,0023	0	0	0	0	0	12	7,88E-05	152218	9,76E-05
0	0	0	0	0,0013	0	0	4,41E-05	0	0	10	6,57E-05	152154	5,69E-05
9,66E-05	0	2,00E-04	0	0	3,00E-04	0	2,00E-04	5,00E-04	0	26	0,000171	152142	1,00E-04
7,21E-05	0	0	0	0	3,00E-04	0	4,00E-04	0	6,00E-04	38	0,00025	152220	3,00E-04
0	0	0	0	4,00E-04	0	0	0	0	0	2	1,31E-05	152184	8,13E-06
0	0	0	0	0	0	0	0	0	0	0	0	0	0
0	0	0	0	0	0	0	1,47E-05	0	0	1	6,57E-06	152158	0
0	0	0	0	0	0	0	1,47E-05	0	0	1	6,57E-06	152128	0
0,0065	0	2,00E-04	0	0	0	0	4,41E-05	5,00E-04	0	275	0,001807	152202	0
0	0	0	0	0	0	0	0	0	0	0	0	0	0
9,65E-05	0	0	0	0	0	0	0	0	2,00E-04	5	3,29E-05	152190	8,13E-06
0	0	0	0	0	0	0	0	0	0	0	0	0	0

2,41E-05	0	0	0	0	0	0	0	0	0	1	6,57E-06	152128	4,25E-06
0	0	0	0	0	0	0	4,41E-05	0	0	3	1,97E-05	152120	2,06E-05
0	0	0	0	0	0	0	0	0	0	0	0	0	1,28E-05
1,00E-04	0	2,00E-04	0	0	0	0,0034	2,94E-05	0	0	12	7,88E-05	152208	8,12E-06
7,23E-05	0	5,00E-04	0	0	9,43E-05	0	5,00E-04	9,00E-04	8,00E-04	51	0,000335	152062	3,00E-04
0	0	0	0	0	0	0	0	0	0	0	0	0	0
0,0032	0	1,00E-04	0	0	0	0	1,47E-05	5,00E-04	0	136	0,000893	152252	2,00E-04
0	0	0	0	4,00E-04	0	0	0	0	0	2	1,31E-05	152100	2,03E-05
0,0033	0	1,00E-04	0	0	0	0	0	5,00E-04	0	140	0,00092	152208	2,00E-04
2,00E-04	0	0,0029	0	0	0,0013	0	2,00E-04	0,0024	0	86	0,000565	152220	3,00E-04
0	0	0	0	0	0	0	0	0	0	0	0	0	8,27E-06
0	0	0	0	0	0	0	1,47E-05	0	0	1	6,57E-06	152112	5,30E-05
0	0	0	0	0	0	0	1,47E-05	0	0	1	6,57E-06	152134	0
0	0	0	0	0	0	0	0	0	0	0	0	0	1,63E-05
0	0	1,00E-04	0	0	0	0	0	0	0	2	1,31E-05	152214	1,37E-05
0,0013	0	1,00E-04	0	0	0	0	0	0	0	57	0,000374	152222	9,11E-05
0	0	0	0	0	0	0	0	0	0	0	0	0	0
2,42E-05	0	0	0	0	0	0	0	0	0	1	6,58E-06	152060	0
7,23E-05	0	6,55E-05	0	0	0	0	0	0	0	4	2,63E-05	152228	4,07E-06
0	0	0	0	0	0	0	0	0	0	0	0	0	0
0	0	0	0	0	0	0	0	0	0	0	0	0	0
0	0	0	0	0	0	0	0	0	0	0	0	0	4,07E-06
1,00E-04	0	0	0	0	0	0	1,00E-04	0	0	13	8,54E-05	152250	3,27E-05
0	0	0	0	0	0	0	0	0	0	0	0	0	4,06E-06
0	0	0	0	0	0	0	1,47E-05	0	0	1	6,59E-06	151798	0
9,63E-05	0	7,00E-04	0	0	0	0	5,00E-04	0,0014	0	54	0,000355	152166	2,00E-04
0	0	0	0	0,0021	0	0	0	0	0	11	7,23E-05	152160	5,87E-05
0	0	0	0	0	0	0	0	0	0	0	0	0	0
0	0	0	0	0	0	0	0	0	0	0	0	0	0
0	0	0	0	0	0	0	0	0	0	0	0	0	4,83E-06
0	0	2,00E-04	0	0	0	0	1,00E-04	0	0	10	6,57E-05	152226	8,53E-05
0	0	0	0	0	0	0	0	0	0	0	0	0	0
0	0	0	0	0	0	0	0	0	0	0	0	0	0
0	0	0	0	0	0	0	0	0	0	0	0	0	0
0	0	0	0	0	0	0	0	0	0	0	0	0	0
0	0	0	0	0	0	0	5,88E-05	5,00E-04	0	5	3,29E-05	152032	2,44E-05

0	0	6,55E-05	0	0	0	0	0	5,00E-04	0	2	1,31E-05	152196	1,63E-05
4,83E-05	0	0	0	0	0	0	0	0	0	2	1,32E-05	152072	4,07E-06
1,00E-04	0	0	0	0	0	0	5,00E-04	0	0	37	0,000243	152184	0
4,00E-04	0	0,0014	0,0014	0	0	0	0,0011	0,0014	0	119	0,000782	152114	7,00E-04
0	0	0	0	0	0	0	0	0	0	0	0	0	0
0	0	0	0	0	0	0	1,47E-05	0	0	1	6,57E-06	152154	2,44E-05
0	0	0	0	0	0	0	0	0	0	0	0	0	8,12E-06
2,42E-05	0	0	0	2,00E-04	0	0	0	0	0	2	1,32E-05	152048	1,22E-05
0	0	0	0	0	0	0	1,47E-05	0	0	1	6,57E-06	152156	1,63E-05
9,63E-05	0	3,00E-04	0	0	0	0	1,00E-04	0	0	15	9,86E-05	152084	1,00E-04
0	0	0	0	0	0	0	1,47E-05	0	0	1	6,57E-06	152254	4,50E-06
0	0	0	0	0	0	0	0	0	0	0	0	0	0
0	0	0	0	0	0	0	0	0	0	0	0	0	1,22E-05
2,41E-05	0	0	0	0	0	0	2,94E-05	0	0	3	1,97E-05	152192	2,03E-05
0	0	0	0	0	0	0	0	0	0	0	0	0	0
0	0	0	0	0	0	0	1,47E-05	0	0	1	6,58E-06	152002	0
0,0013	0	3,00E-04	0	6,00E-04	0	0	0	5,00E-04	8,00E-04	65	0,000427	152182	4,00E-04
4,81E-05	0	6,54E-05	0,0014	2,00E-04	0	0	1,00E-04	0	8,00E-04	20	0,000131	152198	3,00E-04
0	0	0	0	0	0	0	0	0	0	0	0	0	4,06E-06
0	0	0	0	0	0	0	0	0	0	0	0	0	0
0	0	0	0	0	0	0	0	0	0	0	0	0	0
0	0	0	0	0	0	0	0	0	0	0	0	0	0
0	0	0	0	2,00E-04	0	0	0	0	0	1	6,58E-06	151986	4,09E-05
0	0	0	0	0	0	0	0	0	0	0	0	0	0
3,00E-04	0	0	0	0,0048	0	0	0	0	0,0014	45	0,000296	152170	0
0	0	0	0	0	0	0	1,48E-05	0	0	1	6,68E-06	149802	1,37E-05
0	0	0	0	0	0	0	0	0	0	0	0	0	0
0	0	0	0	0	0	0	0	0	0	0	0	0	0
0	0	5,00E-04	0	0	0	0	1,47E-05	5,00E-04	0	9	5,92E-05	152124	5,29E-05
2,41E-05	0	1,00E-04	0	0	0	0	2,00E-04	0	0	17	0,000112	152198	1,00E-04
4,83E-05	0	0	0	0	0	0	0	0	0	2	1,31E-05	152158	4,06E-06
0	0	0	0	0	0	0	0	0	0	0	0	0	0
0	0	0	0	0	0	0	0	0	0	0	0	0	0
0	0	0	0	0	0	0	1,47E-05	0	0	1	6,57E-06	152220	0
8,00E-04	0	2,00E-04	0	0	0	0	0	5,00E-04	0	38	0,00025	152160	7,11E-05
0	0	0	0	0	0	0	0	0	0	0	0	0	0

0	0	0	0	0	0	0	0	0	0	0	0	0	0
0	0	0	0	0	0	0	4,41E-05	0	0	3	1,97E-05	152150	8,13E-06
0	0	0	0	0	0	0	0	0	0	0	0	0	0
0	0	0	0	0	0	0	1,47E-05	0	0	1	6,57E-06	152154	2,02E-05
2,41E-05	0	0	0	0	0	0	1,47E-05	0	0	2	1,31E-05	152236	1,17E-05
0	0	0	0	0	0	0	0	0	0	0	0	0	0
0	0	0	0	0,0012	0	0	0	0	2,00E-04	7	4,60E-05	152162	1,00E-04
0	0	6,56E-05	0	0	0	0	1,47E-05	0	0	2	1,32E-05	151940	2,03E-05
0	0	0	0	0	0	0	0	0	0	0	0	0	0
0	0	0	0	0	0	0	0	0	0	0	0	0	0
2,40E-05	0	0	0	0	0	0	4,41E-05	0	0	4	2,63E-05	152274	1,00E-04
0	0	0	0	0	0	0	0	0	0	0	0	0	0
2,00E-04	0	0	0	0	0	0	0	5,00E-04	0	8	5,26E-05	152178	0
0	0	3,00E-04	0	0	0	0	0	0	0	4	2,63E-05	152226	5,33E-05
2,41E-05	0	0	0	0	0	0	1,47E-05	0	0	2	1,31E-05	152242	2,50E-05
0	0	0	0	0	0	0	0	0	0	0	0	0	4,06E-06
0	0	6,55E-05	0	0	0	0	0	0	0	1	6,58E-06	152030	0
5,00E-04	0	1,00E-04	0	0	0	0	0	0	0	21	0,000138	152096	2,44E-05
0	0	0	0	0	0	0	0	0	0	0	0	0	0
7,22E-05	0	6,54E-05	0,0029	0	0	0	2,00E-04	5,00E-04	4,00E-04	30	0,000197	152182	5,00E-04
0	0	6,55E-05	0	0	0	0	0	0	0	1	6,58E-06	152086	1,22E-05
0	0	0	0	0	0	0	0	5,00E-04	0,0104	51	0,000335	152238	0,0011
0	0	0	0	0	0	0	2,94E-05	0	2,00E-04	3	1,97E-05	152154	3,66E-05
0	0	0	0	0	0	0	0	0	0	0	0	0	4,06E-06
0	0	6,56E-05	0	0	0	0	1,47E-05	0	0	2	1,31E-05	152108	5,28E-05
7,25E-05	0	0	0	0	0	0	1,47E-05	0	0	4	2,63E-05	151876	4,69E-06
9,64E-05	0	0	0	0	0	0	5,88E-05	0	4,00E-04	10	6,58E-05	152048	6,91E-05
3,00E-04	0	0	0	0	0	0	0	0	0	11	7,23E-05	152168	2,03E-05
0,001	0	1,00E-04	0	0	0	0	4,41E-05	9,00E-04	0	49	0,000322	152162	8,12E-05
2,00E-04	0,0011	3,00E-04	0	0	0	0	7,00E-04	9,00E-04	0	63	0,000414	152034	5,00E-04
0	0	0	0	0	0	0	4,41E-05	0	0	3	1,97E-05	152106	3,25E-05
0	0	0	0	0	0	0	0	0	0	0	0	0	0
0	0	0	0	0	0	0	0	0	0	0	0	0	0
0	0	0	0	0	0	0	0	0	0	0	0	0	0
0	0	0	0	0	0	0	0	0	0	0	0	0	8,13E-06
2,48E-05	0	0	0	0	0	0	7,40E-05	0	0	6	4,01E-05	149598	2,45E-05

0	0	4,00E-04	0	0	9,41E-05	0	4,00E-04	5,00E-04	6,00E-04	40	0,000263	152208	4,00E-04
0,0014	0	0,0014	0	0,0012	9,45E-05	0,0034	9,00E-04	0,0014	0,0029	166	0,001096	151502	0,001
0	0	0	0	0	0	0	1,48E-05	0	0	1	6,61E-06	151322	1,47E-05
0	0	0	0	0	0	0	2,94E-05	0	0	2	1,31E-05	152202	4,07E-06
0	0	1,00E-04	0	0	0	0	0	0	0	2	1,31E-05	152262	1,19E-05
2,41E-05	0	0	0	0	0	0	1,47E-05	0	0	2	1,31E-05	152246	2,03E-05
0,0022	0	6,54E-05	0	0	0	0	0	0	0	94	0,000618	152116	1,00E-04
2,42E-05	0	0	0	0	0	0	2,94E-05	0	0	3	1,97E-05	151994	3,39E-05
0	0	0	0	0	0	0	0	0	0	0	0	0	0
0	0	0	0	0	0	0	1,47E-05	0	0	1	6,57E-06	152196	0
0	0	0	0	0	0	0	0	0	0	0	0	0	0
0,0038	0	5,00E-04	0	0	0	0	4,41E-05	5,00E-04	0	169	0,00111	152204	3,00E-04
4,00E-04	0	0	0	0	0	0	0	0	0	0	0	0	0
2,42E-05	0	0	0	0	0	0	1,47E-05	0	0	2	1,31E-05	152094	2,04E-05
0	0	6,55E-05	0	0	0	0	5,88E-05	0	0	5	3,29E-05	152146	0
0	0	0	0	0	0	0	0	0	0	0	0	0	0
0	0	0	0	0	0	0	0	0	0	0	0	0	4,07E-06
0	0	0	0	0	0	0	0	0	0	0	0	0	0
0	0	0	0	0	0	0	0	0	0	0	0	0	0
0	0	0	0	0	0	0	0	0	2,00E-04	1	6,57E-06	152222	4,23E-06
0	0	0	0	0	0	0	0	0	0	0	0	0	0
0	0	0	0	0	0	0	0	0	0	0	0	0	0
3,00E-04	0	0,0022	9,00E-04	0	0	0	9,00E-04	0,0014	0	115	0,000755	152236	7,00E-04
6,00E-04	0	3,00E-04	0	0	0,0025	0	0,0029	0	0	251	0,001651	152030	0,0018
2,42E-05	0	0	0	0	0	0	0	0	0	1	6,58E-06	152090	0
2,41E-05	0	0	0	2,00E-04	0	0	4,41E-05	0	0	5	3,29E-05	152144	3,26E-05
0	0	0	0	0	0	0	2,94E-05	5,00E-04	0	3	1,97E-05	152204	3,25E-05
4,00E-04	0	6,00E-04	6,00E-04	0	0,0027	0	0,0019	0	2,00E-04	186	0,001228	151446	0,0012
2,41E-05	0	0	0	0	0	0	4,41E-05	0	0	4	2,63E-05	152156	8,66E-05
0	0	0	0	0	0	0	0	0	0	0	0	0	0
0	0	0	0	0	0	0	1,00E-04	0	0	10	6,57E-05	152154	1,63E-05
0	0	0	0	0	0	0	0	0	0	0	0	0	5,89E-06
2,41E-05	0	6,55E-05	0	0	0	0	3,00E-04	0	0	24	0,000158	152174	2,00E-04
0	0	0	0	0	0	0	0	0	0	0	0	0	0
0	0	0	0	0	0	0	0	0	0	0	0	0	0
4,81E-05	0	0,0012	0	0	0	0	7,35E-05	0	0	25	0,000164	152136	1,00E-04

0	0	0	0	0	0	0	0	0	0	0	0	0	0
0	0	0	0	0	0	0	0	5,00E-04	0	1	6,57E-06	152188	0
0	0	0	0	0	0	0	0	0	0	0	0	0	0
1,00E-04	0	0	0	0	0	0	0	0	0	5	3,29E-05	152122	4,06E-06
4,81E-05	0	8,00E-04	0	0	0	0,0034	1,00E-04	9,00E-04	0	25	0,000164	152186	7,72E-05
0	0	0	0	0	0	0	0	0	0	0	0	0	0
0	0	0	0	0	0	0	0	0	0	0	0	0	0
4,83E-05	0	0	0	2,00E-04	0	0	3,00E-04	0	0	22	0,000145	152162	9,75E-05
7,00E-04	0	6,55E-05	0	0	0	0	0	0	0	30	0,000197	152200	4,06E-05
9,65E-05	0	0	0	4,00E-04	0	0	0	0	0	6	3,95E-05	151780	5,32E-05
0	0	0	0	0	0	0	0	0	0	0	0	0	4,06E-06
0	0	0	0	0	0	0	2,94E-05	0	0	2	1,31E-05	152166	2,03E-05
2,43E-05	0	2,00E-04	0	0	0	0	1,47E-05	0	0	5	3,30E-05	151480	4,53E-05
0	0	6,55E-05	0	0	0	0	0	0	0	1	6,57E-06	152174	2,03E-05
0	0	0	0	0	0	0	0	0	0	0	0	0	0
4,00E-04	0	1,00E-04	0	0	0	0,0068	0	0	0	21	0,000138	152192	6,70E-05
0	0	0	0	0	0	0	0	0	0	0	0	0	0
0,0012	0	6,55E-05	0	0	0	0	0	0	0	52	0,000342	152112	6,11E-05
0	0	0	0	0	0	0	0	0	0	0	0	0	0
0	0	0	0	0	0	0	0	0	0	0	0	0	0
0	0	0	0	2,00E-04	0	0	0	0	0	1	6,59E-06	151764	8,19E-06
2,00E-04	0	3,00E-04	0	0	0,0014	0	0,0015	0	0	132	0,000867	152220	0,0012
0	0	0	0	0	0	0	0	0	0	0	0	0	0
0	0	0	0	0	0	0	0	0	0	0	0	0	0
2,00E-04	0	0	0	0	0	0	1,47E-05	0	0	8	5,26E-05	152216	4,47E-05
0	0	0	0	0	0	0	0	0	0	0	0	0	0
0	0	0	0	0	0	0	0	0	0	0	0	0	0
0	0	0	0	0	0	0	0	0	0	0	0	0	0
0	0	0	0	0	0	0	0	0	0	0	0	0	0
0	0	6,55E-05	0	0	0	0	0	5,00E-04	0	2	1,31E-05	152196	8,16E-06
0	0	0	0	0	0	0	0	0	0	0	0	0	0
0	0	0	0	0	0	0	0	0	0	0	0	0	0
0	0	0	0	0	0	0	0	0	0	0	0	0	1,23E-05
0	0	0	0	0	0	0	1,47E-05	0	0	1	6,58E-06	152054	4,07E-06
2,45E-05	0	0	0	0	0	0	1,48E-05	0	0	2	1,33E-05	150436	8,20E-06
0	0	0	0	0	0	0	1,47E-05	0	0	1	6,57E-06	152128	4,06E-06

0	0	6,54E-05	0	0,0062	9,42E-05	0	2,94E-05	0	0,001	41	0,000269	152200	5,00E-04
0	0	0	0	0	0	0	0	0	0	0	0	0	0
0	0	0	0	0	0	0	0	0	0	0	0	0	0
7,24E-05	0	0	0	0	0	0	1,47E-05	0	0	4	2,63E-05	152180	2,03E-05
2,41E-05	0	0	0	0	0	0	0	0	0	1	6,57E-06	152170	6,42E-06
0	0	0	0	0	0	0	7,35E-05	0	0	5	3,29E-05	152156	8,14E-06
0	0	0	0	0	0	0	0	0	0	0	0	0	0
2,42E-05	0	0	0	0	0	0	1,47E-05	0	0	2	1,32E-05	152080	8,12E-06
0	0	0	0	0	0	0	0	0	0	0	0	0	0
0	0	3,00E-04	0	0	0	0	2,94E-05	0	0	6	3,94E-05	152218	6,91E-05
0	0	0	0	0	0	0	0	0	0	0	0	0	0
0	0	0	0	0	0	0	0	0	0	0	0	0	0
0	0	1,00E-04	0	0	0	0	0	0	0	2	1,32E-05	151842	4,26E-05
0	0	0	0	0	0	0	1,47E-05	0	0	1	6,57E-06	152240	2,53E-05
0	0	0	0	0	0	0	0	0	0	0	0	0	0
0,0025	0	6,54E-05	0	0	0	0	0	0	0	106	0,000696	152224	2,00E-04
0	0	0	0	0	0	0	1,47E-05	0	0	1	6,57E-06	152164	0
0	0	0	0	0	0	0	0	0	0	0	0	0	1,64E-05
0	0	0	0	0	0	0	0	0	0	0	0	0	0
0	0	7,00E-04	0	2,00E-04	2,00E-04	0	4,41E-05	0	0	17	0,000112	152234	1,00E-04
0	0	0	0	0	0	0	0	0	0	0	0	0	4,06E-06
0	0	0	0	0	0	0	0	0	0	0	0	0	0
0	0	0	0	0	0	0	1,47E-05	0	0,0021	11	7,23E-05	152058	3,00E-04
9,00E-04	0	1,00E-04	0	0	0	0	0	5,00E-04	0	40	0,000264	151246	8,43E-05
1,00E-04	0	0,002	0	0	0	0	8,00E-04	9,00E-04	0	90	0,000591	152200	5,00E-04
0	0	0	0	0	0	0	0	0	0	0	0	0	3,27E-05
0	0	0	0	0	0	0	0	0	0	0	0	0	0
2,41E-05	0	0	0	0	0	0,0034	1,00E-04	5,00E-04	4,00E-04	12	7,88E-05	152226	6,67E-05
0	0	0	0	0	0	0	0	0	0	0	0	0	0
0	0	0	0	0	0,0048	0	6,00E-04	5,00E-04	8,00E-04	99	0,00065	152202	7,00E-04
3,00E-04	0	1,00E-04	0	0	0	0	0	0	0	14	9,20E-05	152172	3,40E-05
0,0038	0	3,00E-04	0	0	0	0	0	0,0019	2,00E-04	163	0,001071	152154	3,00E-04
4,84E-05	0	6,57E-05	0	0	0	0	2,94E-05	0	0	5	3,29E-05	151838	8,17E-06
2,42E-05	0	6,55E-05	0	0	0	0	0	0	0	2	1,32E-05	152072	1,63E-05
0	0	0	0	0	0	0	0	0	0	0	0	0	0
0	0	6,54E-05	0	0	0	0	1,47E-05	0	0	2	1,31E-05	152246	0

0	0	0	0	0	0	0	0	0	0	0	0	0	0
0	0	6,55E-05	0	0	0	0	1,47E-05	5,00E-04	0	3	1,97E-05	152192	4,07E-06
0	0	0	0	0	0	0	0	0	0	0	0	0	0
4,81E-05	0	0	0	0	0	0	2,94E-05	0	0,0015	11	7,23E-05	152174	4,00E-04
0	0	0	0	0	0	0	0	0	0	0	0	0	0
0	0	0	0	0	0	0	0	0	0	0	0	0	0
3,00E-04	0	0,001	0,0029	0	5,00E-04	0	0,0026	0,0024	4,00E-04	230	0,001512	152164	0,0018
4,00E-04	0	0	0	0	0	0	2,94E-05	0	0	18	0,000118	152102	4,47E-05
0	0	0	0	0	0	0	0	0	0	0	0	0	0
7,24E-05	0	0	0	0	0	0	0	0	0	3	1,97E-05	152152	8,13E-06
2,41E-05	0	4,00E-04	0	0	0	0	2,00E-04	5,00E-04	2,00E-04	20	0,000131	152186	1,00E-04
0	0	0	0	0	0	0	1,47E-05	0	0	1	6,57E-06	152254	0
0	0	0	0	0	0	0	1,48E-05	0	0	0	0	0	0
0	0	0	0	0	0	0	2,94E-05	0	0,0025	14	9,21E-05	152018	3,00E-04
2,41E-05	0	2,00E-04	0	0,0027	0	0	2,95E-05	9,00E-04	0	22	0,000145	151858	4,00E-04
4,81E-05	0	0	0	0	0	0	0	0	4,00E-04	4	2,63E-05	152250	4,51E-05
5,06E-05	0	2,00E-04	0	0	0	0	1,00E-04	0	0	7	8,78E-05	79716	0
0	0	1,00E-04	0	0	0	0	0	0	0	2	1,31E-05	152164	8,15E-06
4,00E-04	0	0,0012	3,00E-04	0	7,00E-04	0,0034	0,0023	9,00E-04	6,00E-04	204	0,00134	152216	0,0014
0	0	0	0	0	0	0	1,47E-05	0	0	1	6,58E-06	151950	6,21E-06
4,00E-04	0	0,0027	0	0	0,0013	0	0,0031	0,0019	0	283	0,001859	152198	0,0016
0	0	1,00E-04	0	0	0	0	1,00E-04	0	2,00E-04	11	7,24E-05	151858	6,91E-05
0	0	0	0	0	0	0	0	0	0	0	0	0	0
9,65E-05	0	2,00E-04	0	0	0	0	4,00E-04	5,00E-04	0	36	0,000237	152154	2,00E-04
1,00E-04	0	0	0	0	0	0	1,47E-05	0	0	7	4,60E-05	152230	1,22E-05
0	0	0	0	0	0	0	0	0	0	0	0	0	0
0	0	0	0	0	0	0	4,41E-05	0	0	3	1,97E-05	152272	4,07E-06
0	0	0	0	0	0	0	0	0	0	0	0	0	0
0	0	0	0	0	0	0	0	0	0	0	0	0	4,06E-06
4,81E-05	0	1,00E-04	0	0	0,0011	0	7,00E-04	5,00E-04	0	62	0,000407	152218	4,00E-04
9,62E-05	0	0	0	0	0	0	0,0012	5,00E-04	0	84	0,000552	152224	6,00E-04
2,00E-04	0	6,54E-05	0	0,0029	0	0	0	0,0014	0	28	0,000184	152162	2,00E-04
8,00E-04	0	6,54E-05	0	0	0	0	0	5,00E-04	0	33	0,000217	152140	2,86E-05
0	0	0	0	0	0	0	7,44E-05	0	0	5	3,34E-05	149570	7,76E-06
0	0	2,00E-04	0	0	0	0	0	0	0	3	1,97E-05	152088	1,24E-05
0	0	0	0	0	0	0	0	0	0	0	0	0	0

0,0127	0	0,001	0	0	0	0	4,41E-05	0,0014	0	550	0,003613	152208	0
2,00E-04	0	6,54E-05	0	0	0	0	1,00E-04	0	0	17	0,000112	152142	6,55E-05
4,83E-05	0	0	0	0	0	0	1,47E-05	0	0	3	1,97E-05	152164	2,86E-05
0	0	0	0	4,00E-04	0	0	1,48E-05	0	0	3	1,98E-05	151434	1,22E-05
0	0	0	0	0	0	0	0	0	0	0	0	0	0
7,24E-05	0	0	0	0	0	0	0	0	0	3	1,97E-05	152270	3,66E-05
7,24E-05	0	0	0	0	0	0	0	0	0	3	1,97E-05	152156	2,03E-05
0	0	0	0	0	0	0	1,48E-05	0	0	1	6,61E-06	151272	0
0	0	0	0,0012	0	0	0	2,00E-04	0	0	19	0,000125	152228	2,00E-04
0	0	0	0	0	0	0	5,88E-05	0	0	4	2,63E-05	152074	1,22E-05
0	0	0	0	0	0	0	2,94E-05	0	0	2	1,31E-05	152242	8,12E-06
0	0	0	0	0	0	0	0	0	0	0	0	0	0
2,45E-05	0	6,56E-05	0	0	0	0	7,37E-05	0	2,00E-04	8	5,30E-05	151046	4,11E-06
0	0	0	0	0	0	0	0	0	0	0	0	0	0
2,42E-05	0	6,55E-05	0	0	0	0	1,47E-05	0	0	3	1,97E-05	152082	1,64E-05
0	0	0	0	0	0	0	0	0	0	0	0	0	4,13E-06
0	0	0	0	0	0	0	0	0	0	0	0	0	0
0	0	0	0	0	0	0	1,47E-05	0	0	1	6,57E-06	152154	0
0	0	0	0	0	0	0	0	0	0	0	0	0	0
2,41E-05	0	0	0	0	0	0	0	0	0	1	6,57E-06	152234	8,20E-06
0	0	4,00E-04	0	0	0	0	0	5,00E-04	0	7	4,60E-05	152102	8,13E-06
0	0	0	0	0	0	0	0	0	0	0	0	0	0
0	0	0	0	2,00E-04	0	0	0	5,00E-04	0	2	1,32E-05	152074	4,06E-06
0	0	0	0	0	0	0	2,94E-05	0	0	2	1,31E-05	152210	1,64E-05
0	0	0,0016	0	0	0	0	2,94E-05	0,001	0	29	0,000191	152108	1,00E-04
0	0	0	0	0	0	0	0	0	0	0	0	0	0
0	0	0	0	0	0	0	0	0	0	0	0	0	0
0	0	0	0	0	0	0	0	0	0	0	0	0	0
0,0043	0	9,00E-04	0	0	0	0	1,47E-05	0	0	192	0,001262	152144	4,00E-04
0	0	0	0	0	0	0	0	0	0	0	0	0	0
0	0	0	0	0	0	0	0	0	0	0	0	0	0
0	0	0	0	0	0	0	0	0	0	0	0	0	0
0,0312	0	0,0031	0	0,001	0	0,0037	2,00E-04	0,0045	2,00E-04	1321	0,008951	147574	0
2,41E-05	0	0	0	0	0	0	1,47E-05	0	0	2	1,31E-05	152228	8,16E-06
0	0	0	0	0,0039	0	0	0	0	2,00E-04	21	0,000138	152118	2,00E-04
0	0	0	0	0	0	0	0	0	0	0	0	0	0

0	0	0	0	0	0	0	0	0	0	0	0	0	0
0	0	0	0	0	0	0	0	0	0	0	0	0	1,31E-05
0	0	0	0	0	0	0	0	0	0	0	0	0	0
0	0	0	0	0	0	0	5,88E-05	0	0	4	2,63E-05	152130	1,23E-05
2,41E-05	0	0	0	0	0	0	0	0	0	1	6,57E-06	152092	0
0	0	0	0	0	0	0	0	0	0	0	0	0	8,13E-06
0	0	0	0	0	0	0	0	0	0	0	0	0	0
0	0	0	0	0	0	0	0	0	0	0	0	0	0
4,00E-04	0	6,54E-05	0	2,00E-04	0	0	1,47E-05	0	0	19	0,000125	152134	2,86E-05
0	0	6,55E-05	0	0	0	0	2,94E-05	0	0	3	1,97E-05	152168	2,03E-05
0	0	0	0	0	0	0	1,00E-04	0	0	7	4,60E-05	152194	2,86E-05
0	0	0	0	0	0	0	0	0	0	0	0	0	8,12E-06
9,63E-05	0	0	0	0	0	0	1,00E-04	0	0	14	9,20E-05	152130	1,00E-04
0	0	0	0	0	0	0	0	0	0	0	0	0	0
0	0	0	0	0	0	0	0	0	0	0	0	0	0
0	0	0	0	0	0	0	0	0	0	0	0	0	0
0	0	0	0	0	0	0	0	0	0	0	0	0	0
0	0	0	0	0	0	0	0	0	0	0	0	0	0
0,0012	0	1,00E-04	0	0	0,0052	0	0,001	0,001	0	173	0,00114	151730	0,001
0	0	0	0	0	0	0	0	0	0	0	0	0	0
9,65E-05	0	1,00E-04	0	0	0	0	1,00E-04	0	0	15	9,86E-05	152158	9,35E-05
7,22E-05	0	4,00E-04	6,00E-04	0	0	0	0,0013	5,00E-04	0,0012	107	0,000704	151958	6,00E-04
7,00E-04	0	6,54E-05	0	0	0	0	4,41E-05	5,00E-04	2,00E-04	35	0,00023	152014	7,57E-05
0	0	0	0	0	0	0	0	0	0	0	0	0	0
0	0	0	0	0,0073	0	0	0	0	0	37	0,000244	151398	2,00E-04
0,003	0	3,00E-04	0	0	0	0	0	5,00E-04	0	132	0,000867	152194	2,00E-04
9,62E-05	0	0	0	0	0	0	3,00E-04	5,00E-04	0	23	0,000151	152208	2,00E-04
0	0	0	0	0	0	0	0	0	0	0	0	0	0
0	0	0	0	0	0	0	0	0	0	0	0	0	0
2,41E-05	0	0	0	0	0	0	1,47E-05	0	0	2	1,31E-05	152172	8,14E-06
0	0	0	0	0	0	0	0	0	0	0	0	0	0
0	0	0	0	0	0	0	0	0	0	0	0	0	8,26E-06
2,41E-05	0	0	0	0	0	0	0	0	0	1	6,57E-06	152202	0
0	0	0	0	0	0	0	0	0	0	0	0	0	4,07E-06
7,00E-04	0	6,54E-05	0	0	0	0	0	0	0	32	0,00021	152202	0
0	0	0	0	0	0	0	0	0	0	0	0	0	0

4,83E-05	0	0	0	0	0	0	0	0	0	2	1,31E-05	152212	0
2,41E-05	0	0	0	0	0	0	2,94E-05	0	0	3	1,97E-05	152178	8,12E-06
2,41E-05	0	0	0	0	0	0	1,47E-05	0	0	2	1,31E-05	152118	0
0	0	0	0	0	0	0	0	0	0	0	0	0	0
2,41E-05	0	0	0,0014	0	0	0	4,00E-04	0	0	34	0,000223	152246	2,00E-04
0,003	0	3,00E-04	0	0	0	0	0	9,00E-04	0	133	0,000874	152212	2,00E-04
0	0	0	0	0	0	0	0	0	0	0	0	0	4,39E-06
0	0	0	0	0	0	0	0	0	0	0	0	0	0
0	0	0	0	0	0	0	0	0	0	0	0	0	0
0	0	0	0	0	0	0	1,47E-05	0	0	1	6,59E-06	151792	1,22E-05
0	0	0	0	0	0	0	0	0	0	0	0	0	0
0	0	0	0	0	0	0	0	0	0	0	0	0	0
0	0	0	0	0	0	0	0	0	0	0	0	0	0
0	0	0	0	0	0	0	0	0	0	0	0	0	0
0,0017	0	6,54E-05	0	0	0	0	0	5,00E-04	0	72	0,000473	152180	1,00E-04
0	0	0	0	0	0	0	0	0	0	0	0	0	0
0	0	0	0	0	0	0	0	0	0	0	0	0	0
0	0	0	0	0	0	0	2,94E-05	0	0	2	1,31E-05	152164	4,06E-06
0	0	0	0	0	0	0	0	0	0	0	0	0	0
0	0	0	0	0	0	0	0	0	0	0	0	0	0
9,00E-04	0	0	0	0	0	0	5,37E-05	0	0	19	0,000245	77590	0
3,00E-04	0	0	0	0	2,00E-04	0	1,00E-04	0	0	25	0,000164	152142	4,69E-05
2,41E-05	0	6,55E-05	0	0	0	0	4,41E-05	0	0	5	3,29E-05	152120	1,65E-05
0	0	6,55E-05	0	0	0	0	1,47E-05	5,00E-04	0	3	1,97E-05	152184	3,26E-05
0	0	1,00E-04	0	0	0	0	0	0	0	2	1,31E-05	152224	1,49E-05
0	0	0	0	0	0	0	0	0	0	0	0	0	0
0	0	0	0	0	0	0	0	0	0	0	0	0	0
0	0	0	0	0	0	0	0	0	0	0	0	0	8,16E-06
0	0	0	0	0	0	0	0	0	0	0	0	0	0
0,0016	0	1,00E-04	0	2,00E-04	0	0	0	5,00E-04	0	70	0,00046	152226	9,75E-05
2,41E-05	0	0	0	0	0	0	1,00E-04	0	0	9	5,91E-05	152206	9,77E-05
0	0	0	0	0	0	0	0	0	0	0	0	0	0
9,66E-05	0	0	0	0	0	0	4,41E-05	0	0	7	4,60E-05	152138	2,44E-05
2,41E-05	0	0	0	0	0	0	0	0	0	1	6,57E-06	152178	1,64E-05
9,65E-05	0	0	0	0	0	0	0	0	0	4	2,63E-05	152160	2,08E-05
0	0	0	0	0	0	0	0	0	0	0	0	0	0

0	0	0	0	0	0	0	0	0	0	0	0	0	0
3,00E-04	0,0559	7,00E-04	0,0023	2,00E-04	7,00E-04	0,0034	0,0039	0,0028	0,0015	369	0,002425	152138	0,0026
0	0	0	0	0	0	0	0	0	0	0	0	0	0
4,83E-05	0	0	0	0	0	0	1,47E-05	0	0	3	1,97E-05	152124	2,84E-05
2,00E-04	0	6,54E-05	0	0	0	0	0	0	0	10	6,57E-05	152150	3,25E-05
2,42E-05	0	0	0	0	0	0	0	0	0	1	6,57E-06	152096	8,12E-06
0	0	0	0	0	0	0	1,52E-05	0,001	0	3	2,07E-05	145038	0
0	0	0	0	0	0	0	0	0	0	0	0	0	4,06E-06
0	0	0	0	0	0	0	0	0	0	0	0	0	0
0	0	0	0	0	0	0	0	0	0	0	0	0	0
0	0	1,00E-04	0,0014	0	0,0035	0	7,00E-04	0	0	92	0,000605	151980	7,00E-04
0,0017	0	5,00E-04	0,006	0	0	0	8,82E-05	0	0	106	0,000696	152208	5,00E-04
3,00E-04	0	0	0	0	0	0	0	0	0	13	8,54E-05	152244	2,03E-05
0	0	0	0	0	0	0	0	0	0	0	0	0	0
0	0	0	0	0	0	0	0	0	0	0	0	0	4,07E-06
0	0	0	0	0	0	0	0	0	2,00E-04	1	6,57E-06	152226	2,44E-05
0	0	3,00E-04	0	0	0	0	0	0,001	0	7	4,60E-05	152218	0
0	0	0	0	0	0	0	1,47E-05	0	0	1	6,57E-06	152122	0
0	0	0	0	0	0	0	0	0	0	0	0	0	0
0	0	0	0	2,00E-04	0	0	0	0	0	1	6,57E-06	152206	0
0	0	0	0	0	0	0	0	0	0	0	0	0	0
1,00E-04	0	0	0	0	0	0	0	0	0	6	3,94E-05	152190	1,29E-05
0	0	6,53E-05	0	0,0077	0	0	0	0	0	41	0,000269	152236	4,00E-04
0	0	0	0	0	0	0	0	0	0	0	0	0	0
6,00E-04	0	0,0011	0	0	0	0	1,00E-04	0	0	48	0,000316	151690	0
2,41E-05	0	3,00E-04	0	4,00E-04	0	0	1,47E-05	0	0	8	5,26E-05	152234	6,09E-05
4,83E-05	0	1,00E-04	0	0	8,00E-04	0	2,00E-04	5,00E-04	0	25	0,000164	152186	1,00E-04
4,82E-05	0	0,0011	0	0	0	0	2,94E-05	0	0	21	0,000138	152222	2,03E-05
2,41E-05	0	0	0	0	0	0	0	0	0	1	6,57E-06	152128	0
0	0	0	0	0	0	0	0	0	0	0	0	0	8,13E-06
0	0	0	0	0	0	0	2,94E-05	0	0	2	1,31E-05	152156	1,01E-05
0	0	0	0	0	0	0	0	0	0	0	0	0	0
0	0	0	0	0	0	0	0	0	0	0	0	0	1,24E-05
0	0	0	0	0	0	0	0	0	0	0	0	0	0
0	0	0	0	0	0	0	0	0	0	0	0	0	0
0	0	0	0	0	0	0	1,47E-05	0	0	1	6,57E-06	152198	4,06E-06

2,00E-04	0	5,00E-04	0	4,00E-04	9,41E-05	0	5,00E-04	0	0	49	0,000322	152236	3,00E-04
7,22E-05	0	0	0	0	0	0	0	0	0	2	1,31E-05	152200	1,62E-05
0	0	0	0	0	0	0	0	0	0	0	0	0	4,06E-05
2,41E-05	0	5,00E-04	0	0	0	0	7,35E-05	5,00E-04	0	15	9,85E-05	152230	1,00E-04
0	0	0	0	0	0	0	0	5,00E-04	0	1	6,57E-06	152234	4,06E-06
0	0	0	0	0	0	0	0	0	0	0	0	0	8,15E-06
2,40E-05	0	6,53E-05	0	0	9,41E-05	0,0034	2,00E-04	5,00E-04	2,00E-04	20	0,000131	152252	1,00E-04
0	0	0	0	0	0	0	0	0	0	0	0	0	4,06E-06
0	0	0	0	0	0	0	0	0	0	0	0	0	0
1,00E-04	0	0	0	2,00E-04	0	0	4,00E-04	0	0	33	0,000217	152154	1,00E-04
0	0	0	0	0	0	0	0	0	0	0	0	0	0
0	0	0	0	0	0	0	0	0	0	0	0	0	1,62E-05
2,42E-05	0	0	0	0	0	0	0	0	0	1	6,57E-06	152128	1,68E-05
0	0	0	0	0	0	0	0	0	0	0	0	0	0
2,41E-05	0	0	0	0	0	0	1,00E-04	0	8,00E-04	12	7,88E-05	152212	1,00E-04
0	0	0	0	0	0	0	0	0	0	0	0	0	0
0	0	0	0	0	0	0	2,94E-05	5,00E-04	0	3	1,97E-05	152032	4,28E-05
0	0	0	0	0	0	0	0	0	0	0	0	0	0
0	0	6,55E-05	0	0	0	0	0	0	0	1	6,57E-06	152158	0
0	0	0	0	0	0	0	0	0	0	0	0	0	0
0	0	0	0	0	0	0	0	0	0	0	0	0	5,39E-06
0	0	0	0	0	0	0	0	0	0	0	0	0	0
0	0	0	0	0	0	0	0	0	0	0	0	0	0
0	0	0	0	0	0	0	1,47E-05	0	0	1	6,57E-06	152140	1,22E-05
0	0	0	0	0	0	0	0	0	0	0	0	0	0
0	0	0	0	0	0	0	0	0	0	0	0	0	0
0	0	0	0	0	0	0	0	0	0	0	0	0	0
0	0	0	0	0	0	0	0	0	0	0	0	0	0
0	0	0	0	0	0	0	0	0	0	0	0	0	4,07E-06
0	0	0	0	0	0	0	0	0	0	0	0	0	0
0	0	0	0	0	0	0	0	0	0	0	0	0	0
0	0	0	0	0	0	0	5,88E-05	0	0	4	2,63E-05	152232	0
0	0	0	0	0	0	0	0	0	0	0	0	0	0
3,00E-04	0	0	0	0	0	0	0	0	0	11	7,23E-05	152216	2,86E-05
0	0	0	0	0	0	0	0	0	0	0	0	0	0
0	0	1,00E-04	0	0	0	0	4,41E-05	0	0	5	3,28E-05	152218	1,58E-05

0	0	0	0	0	0	0	4,41E-05	0	0	3	1,97E-05	152092	4,88E-05
0	0	0	0	0	0	0	0	0	0	0	0	0	8,13E-06
2,45E-05	0	6,61E-05	0	0	0	0	4,00E-04	5,00E-04	0	30	0,000199	150450	1,00E-04
2,43E-05	0	0	0	0	0	0	1,47E-05	0	0	2	1,32E-05	151560	1,63E-05
0	0	0	0	0	0	0	0	0	0	0	0	0	0
2,41E-05	0	0	0	2,00E-04	0	0	0	0	0	2	1,31E-05	152212	0
0	0	6,55E-05	0	0	0	0	0	0	0	1	6,57E-06	152122	3,73E-05
4,82E-05	0	1,00E-04	9,00E-04	0	0,0029	0	0,0013	0	0	124	0,000816	152018	6,00E-04
2,41E-05	0	0	0	0	0	0	1,47E-05	0	0	2	1,31E-05	152264	1,63E-05
2,41E-05	0	1,00E-04	0	0	0	0	2,94E-05	0,0014	0	8	5,26E-05	152148	5,03E-05
0	0	0	0	0	0	0	0	0	0	0	0	0	4,09E-06
0	0	0	0	0	0	0	0	0	0	0	0	0	0
0	0	0	0	0	0	0	0	0	0	0	0	0	0
0	0	0	0	0	0	0	1,47E-05	0	0	1	6,57E-06	152134	8,82E-06
0	0	0	0	0	0	0	0	0	0	0	0	0	4,06E-06
2,41E-05	0	0	0	0	0	0	1,47E-05	0	0	2	1,31E-05	152216	4,10E-06
2,42E-05	0	0	0	0	0	0	1,47E-05	0	0	2	1,32E-05	152068	4,07E-05
0	0	0	0	0	0	0	0	0	0	0	0	0	0
0	0	0	0	0	0	0	0	0	0	0	0	0	4,06E-06
4,00E-04	0	2,00E-04	0	0	0	0	0	5,00E-04	0	16	0,000105	152176	4,09E-05
0	0	0	0	0	0	0	0	0	0	0	0	0	0
0	0	0	0	0,0014	0	0	0	0	0	7	4,60E-05	152100	1,69E-05
0	0	0	0	0	0	0	0	0	0	0	0	0	0
0	0	6,54E-05	0	0	0	0	0	0	0	1	6,57E-06	152168	0
0	0	0	0	0	0	0	0	0	0	0	0	0	0
7,00E-04	0	0	0	0	0	0	1,47E-05	0	0	29	0,000191	152214	2,44E-05
9,00E-04	0	0	0	0	0	0	0	0	0	39	0,000256	152206	8,79E-05
0	0	6,54E-05	0	0	0	0	1,47E-05	0	0	2	1,31E-05	152206	1,63E-05
0	0	0	0	0	0	0	0	0	0	0	0	0	0
0	0	0	0	0	0	0	0	0	0	0	0	0	0
0	0	0	0	0	0	0	1,47E-05	5,00E-04	0	2	1,32E-05	152086	8,13E-06
0,0021	0	0	0	0	0	0	4,41E-05	5,00E-04	0	92	0,000605	152110	2,00E-04
2,41E-05	0	6,53E-05	0	4,00E-04	0	0	5,88E-05	0	8,00E-04	12	7,88E-05	152218	2,00E-04
0	0	1,00E-04	0	0	0	0	1,47E-05	0	2,00E-04	4	2,63E-05	152146	3,66E-05
0	0	0	0	0	0	0	0	0	0	0	0	0	8,13E-06
7,25E-05	0	0	0	0	0	0	0	0	0	3	1,97E-05	152064	0

0	0	0	0	0	0	0	0	0	0	0	0	0	7,16E-06
0	0	0	0	0	0	0	0	5,00E-04	0	1	6,57E-06	152168	1,10E-05
0	0	6,00E-04	0	0	0	0	2,00E-04	5,00E-04	0	21	0,000138	152054	2,00E-04
0	0	0	0	0	0	0	0	0	0	0	0	0	4,06E-06
0	0	0	0	2,00E-04	0	0	0	0	0	1	6,58E-06	152076	3,88E-05
0	0	6,55E-05	0	0	0	0	2,00E-04	0	2,00E-04	14	9,20E-05	152118	8,53E-05
0	0	0	0	0	0	0	0	0	0	0	0	0	0
0,0039	0	3,00E-04	0	0	0	0	1,00E-04	0	0,0052	197	0,001325	148710	2,00E-04
0	0	0	0	4,00E-04	0	0	0	0	0	2	1,32E-05	152072	0
0	0	0	0	0	0	0	0	0	0	0	0	0	0
0	0	0	0	0	0	0	0	0	0	0	0	0	0
0	0	0	0	0	0	0	0	0	0	0	0	0	0
0	0	6,55E-05	0	0	0	0	0	0	0	1	6,57E-06	152174	4,07E-06
0	0	0	0	0	0	0	0	0	0	0	0	0	0
0	0	0	0	0	0	0	1,47E-05	0	0	1	6,58E-06	152078	2,44E-05
0	0	3,00E-04	0	0	0	0	2,94E-05	0	0	7	4,60E-05	152036	2,84E-05
7,24E-05	0	0	0	0	0	0	7,35E-05	0	0	8	5,26E-05	152182	3,66E-05
0	0	0	0	0	0	0	0	0	0	0	0	0	4,08E-06
0	0	0	0	2,00E-04	0	0	0	0	0	1	6,61E-06	151342	0
0	0	0	0	0	0	0	0	0	0	0	0	0	0
0	0	3,00E-04	0	0	0	0	0	0	0	4	2,63E-05	151910	0
0	0	0	0	0	0	0	0	0	0	0	0	0	0
0	0	0	0	2,00E-04	0	0	0	0	0	1	6,57E-06	152116	8,16E-06
4,83E-05	0	0	0	0	0	0	0	0	0	2	1,31E-05	152196	0
0	0	6,56E-05	0	0	0	0	1,47E-05	0,0014	0	5	3,29E-05	152022	2,85E-05
0	0	0	0	0	0	0	0	0	0	0	0	0	0
0	0	0	0	0	0	0	0	0	0	0	0	0	1,22E-05
0	0	0	0	0	0	0	0	0	0	0	0	0	0
7,23E-05	0	0	0	0,001	0	0	0	0	6,00E-04	11	7,24E-05	151964	2,00E-04
0	0	0	0	0	0	0	0	0	0	0	0	0	4,09E-06
1,00E-04	0	0,001	0	0	7,00E-04	0	5,00E-04	5,00E-04	0	59	0,000388	152018	7,00E-04
0	0	0	0	0	0	0	0	0	0	0	0	0	0
7,23E-05	0	0	0	0	9,43E-05	0	2,94E-05	0	0	6	3,95E-05	152090	2,85E-05
0	0	0	0	0	0	0	0	0	0	0	0	0	4,06E-06
2,41E-05	0	0	0	0	0	0	0	0	0	1	6,57E-06	152180	8,14E-06
4,82E-05	0	5,00E-04	0	2,00E-04	0	0	4,41E-05	9,00E-04	0	16	0,000105	152094	1,00E-04

4,00E-04	0	6,67E-05	0	0	0	0	0	5,00E-04	0	18	0,000121	149274	0
0	0	0	0	0	0	0	2,94E-05	5,00E-04	0	3	1,97E-05	152230	2,03E-05
0	0	0	0	0	0	0	0	0	0	0	0	0	0
0	0	0	0	0	0	0	0	0	0	0	0	0	0
0	0	0	0	0	0	0	0	0	0	0	0	0	0
0	0	0	0	0	0	0	0	0	0	0	0	0	0
0	0	4,00E-04	0	0	0	0	1,47E-05	0	0	7	4,60E-05	152186	2,46E-05
0	0	0	0	0	0	0	0	0	0	0	0	0	4,06E-06
0	0	0	0	0	0	0	0	0	0	0	0	0	0
2,41E-05	0	2,00E-04	0	0	9,42E-05	0	3,00E-04	0	0	23	0,000151	152202	2,00E-04
1,00E-04	0	0	0	0	2,00E-04	0	5,00E-04	5,00E-04	0	44	0,000289	152116	3,00E-04
0	0	0	0	0	0	0	0	0	0	0	0	0	0
0	0	0	0	0	0	0	1,47E-05	0	0	1	6,57E-06	152116	0
0	0	0	0	0	0	0	0	0	0	0	0	0	8,17E-06
2,00E-04	0	4,00E-04	0	0	2,00E-04	0	0,0013	0	0	107	0,000703	152186	5,00E-04
0	0	0	0	0	0	0	0	0	0	0	0	0	0
0,0012	0	6,53E-05	0	0	0	0	0	0	0	50	0,000329	152190	1,00E-04
0	0	0	0	0	0	0	1,47E-05	0	0	1	6,57E-06	152218	4,08E-06
0	0	0	0	0	0	0	0	0	0	0	0	0	0
0	0	0	0	0	0	0	0	0	2,00E-04	1	6,58E-06	152058	0
0	0	0	0	0	0	0	4,41E-05	0	0	3	1,97E-05	152198	5,01E-05
0	0	0	0	0	0	0	0	0	0	0	0	0	0
0	0	0	0	0	0	0	1,47E-05	0	0	0	0	0	4,62E-06
0	0	0	0	0	0	0	0	0	0	0	0	0	0
0	0	0	0	0	0	0	0	0	0	0	0	0	0
0	0	0	0	0	0	0	0	0	0	0	0	0	0
0,0035	0	0,0014	0	2,00E-04	0	0	1,00E-04	0,0024	0	175	0,001157	151226	1,00E-04
4,81E-05	0,0055	4,00E-04	0	0	0	0	3,00E-04	0,0014	0	38	0,00025	152190	2,00E-04
0	0	0	0	0	0	0	0	0	2,00E-04	1	6,57E-06	152210	8,13E-06
0	0	0	0	0	0	0	0	0	0	0	0	0	0
0	0	0	0	0	0	0	0	0	0	0	0	0	2,10E-05
0	0	0	0	0	0	0	0	0	0	0	0	0	4,78E-05
0	0	0	0	0	0	0	1,47E-05	0	0	1	6,57E-06	152174	1,63E-05
2,41E-05	0	0	0	0	9,42E-05	0	2,00E-04	0	6,00E-04	21	0,000138	152190	2,00E-04
6,00E-04	0,0011	0,0011	0	0	0	0	0,002	0,0014	0	178	0,00117	152196	0,001
4,83E-05	0	6,54E-05	0	0	0	0	1,47E-05	0	0	4	2,63E-05	152184	1,22E-05

0	0	6,54E-05	0	0	0	0	1,00E-04	5,00E-04	0	11	7,23E-05	152224	1,00E-04
0	0	0	0	0	0	0	0	0	0	0	0	0	0
4,83E-05	0,0077	0	0	0	4,00E-04	0	3,00E-04	0	0	32	0,00021	152158	1,00E-04
0	0	0	0	0	0	0	0	0	0	0	0	0	8,13E-06
0	0	2,00E-04	0,0015	0	0	0	4,00E-04	0	0	32	0,000222	143980	2,00E-04
0	0	0	0	0	0	0	8,82E-05	5,00E-04	0	7	4,60E-05	152142	5,69E-05
0	0	0	0	0	0	0	0	0	0	0	0	0	0
0	0	0	0	0	0	0	5,88E-05	0	0	4	2,63E-05	152250	8,13E-06
0	0	0	0	0	0	0	0	0	0	0	0	0	0
0	0	0	0	0	0	0	0	0	0	0	0	0	0
0,0027	0	4,00E-04	0	0	0	0	1,47E-05	9,00E-04	0	123	0,000808	152260	1,00E-04
0	0	6,54E-05	0	0	0	0	0	0	0	1	6,57E-06	152216	0
4,82E-05	0	1,00E-04	0	0	9,41E-05	0	4,00E-04	5,00E-04	0	35	0,00023	152108	2,00E-04
0	0	0	0	0	0	0	1,00E-04	0	0	7	4,60E-05	152222	3,71E-05
0	0	0	0	0	0	0	0	0	0	0	0	0	0
1,00E-04	0	0	0	0	0	0	2,94E-05	0	0	8	5,26E-05	152228	5,71E-05
6,00E-04	0,011	7,00E-04	0,0049	0	0,0011	0	0,0044	0,0024	2,00E-04	381	0,002505	152124	0,0022
0	0	0	0	0	0	0	4,41E-05	0	0	3	1,97E-05	152162	8,13E-06
0	0	0	0	0	0	0	0	0	4,00E-04	2	1,31E-05	152178	1,66E-05
0	0	0	0	0	0	0	0	0	0	0	0	0	0
0	0	0	0	0	0	0	0	0	0	0	0	0	0
0	0	0	0	0	0	0	0	0	0	0	0	0	0
0	0	0	0	0	0	0	0	0	0	0	0	0	0
0	0	0	0	0	0	0	0	0	0	0	0	0	0
0,0039	0	2,00E-04	0	0	0	0	0	7,00E-04	0	121	0,001091	110915	0
0	0	0	0	0	0	0	7,56E-05	0	0	4	3,60E-05	111180	1,25E-05
0	0	0	0	0	0	0	0	0	0	0	0	0	0
0	0	0	0	0	0,0027	0	0	0	0	12	0,000114	104938	2,00E-04
2,00E-04	0	0	0	0	0	0	0	0	0	6	5,43E-05	110413	5,63E-06
2,00E-04	0	0,0018	4,00E-04	0	0	0	8,00E-04	7,00E-04	0	71	0,000637	111443	6,00E-04
0	0	0	0	0	0	0	0	0	0	0	0	0	0
0	0	0	0	0	0	0	0	0	0	0	0	0	0
0	0	0	0	0	0	0	0	0	0	0	0	0	0
4,00E-04	0	5,00E-04	0,0019	0	0,0052	0	0,0047	0,0013	0	307	0,002737	112161	0,0027
0	0	0	0	0	0	0	1,00E-04	0	0	6	5,41E-05	110918	3,92E-05
0	0	9,54E-05	0	0	0	0	5,66E-05	7,00E-04	0	5	4,50E-05	111191	5,73E-05

0	0	0	0	0	0	0	0	0	0	0	0	0	0
6,66E-05	0	3,00E-04	0	0	0,0121	0	0,0011	0	0	125	0,001192	104826	8,00E-04
6,47E-05	0	3,00E-04	0	0	0,0109	0	0,001	0	0	124	0,001112	111521	0,001
0	0	0	8,00E-04	0	0	0	1,88E-05	0	0	3	2,67E-05	112157	5,62E-05
3,00E-04	0	9,44E-05	0	0	0	0	2,00E-04	0	0	19	0,000169	112177	0
6,47E-05	0	0	0	0	0	0	5,64E-05	0	0	5	4,45E-05	112278	1,14E-05
0	0	0	0	0	0	0	0	0	0	0	0	0	0
0,0022	0	4,00E-04	0	0	0	0	1,88E-05	0	0	74	0,00066	112081	3,00E-04
0	0	0	0	0	0	0	0	0	0	0	0	0	2,92E-05
6,44E-05	0	4,00E-04	0	0	3,00E-04	0	2,00E-04	0	0	16	0,000142	112731	2,00E-04
3,23E-05	0	0	0	0	0	0	0	0	0	1	8,89E-06	112477	5,60E-06
7,80E-05	0	0	0	3,00E-04	0	0	4,30E-05	0	0	5	5,25E-05	95268	0
0	0	0	0	0	0	0	4,01E-05	0	0	2	1,90E-05	105417	2,86E-05
0	0	2,00E-04	0	0	0	0	0	7,00E-04	0	3	2,68E-05	111931	9,53E-06
0	0	0	0	0	0	0	0	0	0	0	0	0	0
0	0	0	0	0	0	0	0	0	0	0	0	0	0
4,00E-04	0	4,00E-04	0	0	0	0	0,0015	0	4,00E-04	97	0,000866	111960	7,00E-04
2,00E-04	0	2,00E-04	0	0	0	0	6,00E-04	0,0013	0	41	0,000367	111630	3,00E-04
4,00E-04	0	3,00E-04	0	0	0,0024	0	0,0015	0,0013	0	109	0,00098	111279	8,00E-04
0	0	9,41E-05	0	0	0	0	1,88E-05	0	0	2	1,78E-05	112082	1,12E-05
0,0029	0	2,00E-04	0	0	0	0	0	7,00E-04	0	88	0,000797	110468	2,00E-04
0	0	0	0	0	0	0	0	0	0	0	0	0	0
0	0	0	0	0	0	0	1,89E-05	0	0	1	9,06E-06	110426	0
0	0	0	0	0	0	0	0	0	0	0	0	0	0
0	0	0	0	0	0	0	0	0	0	0	0	0	0
0	0	0	0	0	0	0	0	0	0	0	0	0	0
0	0	0	0	0	0	0	0	0	0	0	0	0	0
0	0	0	0	0	0	0	0	0	0	0	0	0	0
0	0	0	0	0	0	0	0	0	0	0	0	0	0
1,00E-04	0	0	0	0	0	0	0	0	0	1	2,98E-05	33517	1,49E-05

gnomAD_	gnomAD_	gnomAD_	gnomAD_	gnomAD_	gnomAD_	gnomAD_	gnomAD_	gnomAD_g	gnomAD_g	gnomAD_g	gnomAD_g	gnomAD_g	gnomAD_g
3,00E-04	3,00E-04	0	3,00E-04	1,00E-04	2,00E-04	2,00E-04	6,50E-05	2,00E-04	2,00E-04	0	0	0	0
6,55E-05	0	0	0	0	0	0	0	0	0	0	0	0	0
0	0	0	0	0	0	0	0	0	0	0	0	0	0
0	0	0	0	0	1,86E-05	0	0	9,70E-05	3,00E-04	0	0	0	0
0	0	0	0	0	0	0	0	0	0	0	0	0	0
3,00E-04	3,00E-05	0	0	0	0	0	0	2,00E-04	7,00E-04	0	0	0	0
0	0	0	0	0	0	0	0	0	0	0	0	0	0
0	0	0	0	0	0	0	0	0	0	0	0	0	0
0	0	0	0	0	0	0	0	0	0	0	0	0	0
0	5,96E-05	0	0	0	0	0	0	0	0	0	0	0	0
0	0	0	0	0	0	0	0	0	0	0	0	0	0
3,00E-04	1,00E-04	2,00E-04	0	0	4,00E-04	2,00E-04	4,00E-04	1,00E-04	1,00E-04	0,0012	0	0	0
6,75E-05	6,19E-05	1,00E-04	5,91E-05	0	1,00E-04	0	1,00E-04	3,24E-05	0	0	0	0	3,00E-04
6,71E-05	6,19E-05	0	5,89E-05	0	8,53E-05	0	0	0	0	0	0	0	0
0	0	0	0	0	0	0	0	0	0	0	0	0	0
0	0	0	0	0	1,80E-05	0	3,25E-05	0	0	0	0	0	0
0	8,35E-05	0	0	0	3,47E-05	3,00E-04	0	6,47E-05	1,00E-04	0	0	0	0
0	0	0	0	4,53E-05	1,81E-05	4,00E-04	3,25E-05	0	0	0	0	0	0
0	0	0	5,80E-05	0	8,98E-06	0	0	0	0	0	0	0	0
0	0	0	0	0	0	0	0	0	0	0	0	0	0
0	0	0	0	0	2,69E-05	0	0	0	0	0	0	0	0
0	0	0	0	0	0	0	0	0	0	0	0	0	0
0	0	0	5,80E-05	0	1,79E-05	0	0	0	0	0	0	0	0
0,0018	0	0	0	0	0	0	0	4,00E-04	0,0015	0	0	0	0
0	0	0	0	0	0	0	0	0	0	0	0	0	0
0	0	0	0	0	0	0	0	0	0	0	0	0	0
0	1,00E-04	0	0	0	1,11E-05	2,00E-04	3,00E-04	0	0	0	0	0	0
0	0	0	0	0	0	0	0	0	0	0	0	0	0
0	2,00E-04	0	0	0	1,00E-04	0	3,29E-05	0	0	0	0	0	0
0	0	0	1,00E-04	0	0	0	0	0	0	0	0	0	0
0	0	0	0	0	0	0	0	0	0	0	0	0	0
0	0	0	0	0	0	0	0	0	0	0	0	0	0
0	2,98E-05	0	0	0	0	0	0	0	0	0	0	0	0
1,00E-04	0	0	5,83E-05	0	2,71E-05	0	0	6,47E-05	2,00E-04	0	0	0	0

0	0	0	0	0	0	0	3,25E-05	0	0	0	0	0	0
0	0	0	0	0	0	0	0	0	0	0	0	0	0
0	0	0	0	0	0	0	0	0	0	0	0	0	0
0	2,98E-05	0	5,80E-05	4,48E-05	8,96E-06	0	6,50E-05	0	0	0	0	0	0
6,55E-05	2,98E-05	0	0	0	8,97E-06	0	0	0	0	0	0	0	0
0	0	0	0	0	8,97E-06	0	0	0	0	0	0	0	0
0	0	0	0	0	0	0	0	0	0	0	0	0	0
0	0	0	0	0	1,79E-05	0	3,25E-05	0	0	0	0	0	0
0	0	0	0	0	0	0	0	0	0	0	0	0	0
0	0	0	0	0	0	0	0	0	0	0	0	0	0
0	0	1,00E-04	0	0	0	0	3,44E-05	0	0	0	0	0	0
0	0	0	0	0	4,66E-05	0	0	0	0	0	0	0	0
0	0	0	0	0	0	0	0	0	0	0	0	0	0
0	0	0	0	0	0	0	0	0	0	0	0	0	0
0	0	0	0	0	0	0	0	0	0	0	0	0	0
1,00E-04	0	0	0	0	0	0	0	3,23E-05	0	0	0	0	0
0	0	0	0	4,58E-05	9,31E-06	0	0	0	0	0	0	0	0
0	0	0	0	0	0	0	0	0	0	0	0	0	0
0	0	0	0	0	0	0	0	0	0	0	0	0	0
0	0	0	0	0	0	0	0	0	0	0	0	0	0
0	0	0	0	0	0	0	0	0	0	0	0	0	0
0	0	0	0	0	0	0	0	3,36E-05	0	0	0	0	0
0	0	0	0	0	0	0	0	0	0	0	0	0	0
0	5,96E-05	0	0	0	0	0	3,25E-05	0	0	0	0	0	0
0	0	0	0	0	8,97E-06	0	0	0	0	0	0	0	0
0	0	0	0	0	0	0	0	0	0	0	0	0	0
0	0	0	0	0	0	0	0	0	0	0	0	0	0
1,00E-04	2,98E-05	0	0	2,00E-04	5,00E-04	4,00E-04	4,00E-04	5,00E-04	0	0	0	0	3,00E-04
0	0	0	0	0	0	0	0	0	0	0	0	0	0
0	1,00E-04	0	0	0	0	0	9,75E-05	3,23E-05	1,00E-04	0	0	0	0
1,00E-04	2,98E-05	0	0	0	2,69E-05	0	3,25E-05	6,46E-05	2,00E-04	0	0	0	0
0	0	0	0	0	0	0	0	0	0	0	0	0	0
0	0	0	0	0	0	0	0	0	0	0	0	0	0
0	5,96E-05	2,00E-04	3,00E-04	0	5,37E-05	2,00E-04	0	0	0	0	0	0	0
0	0	0	0	0	2,72E-05	0	0	0	0	0	0	0	0
6,53E-05	1,00E-04	2,00E-04	0	4,49E-05	0,0015	7,00E-04	0,0011	6,00E-04	1,00E-04	0	0	0	0

6,55E-05	0	0	0	0	0	0	0	3,25E-05	1,00E-04	0	0	0	0
0	0	0	0	0	0	0	3,25E-05	0	0	0	0	0	0
0	0	0	0	0	2,69E-05	0	0	3,23E-05	0	0	0	0	0
0	0	0	0	0	1,01E-05	0	0	0	0	0	0	0	0
0	0	0	0	0	0	0	0	3,24E-05	1,00E-04	0	0	0	0
6,55E-05	3,00E-04	0,0013	0	4,52E-05	0,001	9,00E-04	3,26E-05	9,00E-04	1,00E-04	0	0	0	6,00E-04
0	6,06E-05	0	0	0	9,03E-06	0	0	3,27E-05	0	0	0	0	0
0	0	0	0	0	3,25E-05	0	0	6,50E-05	0	0	0	0	0
0	2,98E-05	0	0	0	3,59E-05	0	0	0	0	0	0	0	0
0	0	0	0	0	0	0	6,00E-04	1,00E-04	4,00E-04	0	0	0	0
0	0	0	0	0	0	0	0	0	0	0	0	0	0
0	5,98E-05	0	0	0	2,00E-04	0	0	0	0	0	0	0	0
6,54E-05	5,96E-05	0	0	0	8,97E-05	0	0	0	0	0	0	0	0
0	0	0	0	0	9,09E-06	0	0	0	0	0	0	0	0
0	0	0	0	0	9,03E-06	0	0	0	0	0	0	0	0
0	0	0	0	0	0	0	0	0	0	0	0	0	0
0	6,14E-05	0	0	0	0	0	0	3,23E-05	0	0	0	0	0
0	0	0	0	0	0	0	0	0	0	0	0	0	0
0	2,98E-05	0	0	0	6,29E-05	0	0	0	0	0	0	0	0
0	0	0	0	0	0	0	0	0	0	0	0	0	0
0	0	0	0	0	9,07E-06	0	0	0	0	0	0	0	0
0	0	0	0	0	0	0	0	0	0	0	0	0	0
0	0	0	0	0	0	0	0	0	0	0	0	0	0
0	0	0	0	0	8,97E-06	0	0	0	0	0	0	0	0
0	0	0	0	0	0	2,00E-04	0	0	0	0	0	0	0
0	0	0	0	0	0	0	0	0	0	0	0	0	0
0	0	0	0	0	0	0	0	0	0	0	0	0	0
0	0	7,00E-04	0	0	9,62E-06	0	0	0	0	0	0	0	0
0	0	0	0	0	0	0	0	0	0	0	0	0	0
0	0	0	0	0	2,00E-04	0	9,79E-05	0	0	0	0	0	0
1,00E-04	2,00E-04	0	0	0	1,00E-04	4,00E-04	9,94E-05	6,47E-05	0	0	0	0	0
0,0035	1,00E-04	0	0	0	1,82E-05	0	0	0,001	0,0034	0	0	0	0
0	0	0	0	0	9,28E-06	0	0	3,24E-05	0	0	0	0	0
0	0	0	0	0	0	0	0	0	0	0	0	0	0
0	0	0	0	0	0	0	0	3,29E-05	1,00E-04	0	0	0	0
0	1,00E-04	0	0	0	1,26E-05	0	0	0	0	0	0	0	0

0	0	0	0	0	0	0	0	0	0	0	0	0	0
0,001	6,00E-04	4,00E-04	0	0,0012	0,0037	0,0018	0,0013	0,0016	8,00E-04	0,0012	0	0	0,0014
1,00E-04	3,00E-04	0	1,00E-04	0	2,00E-04	0	0	2,00E-04	2,00E-04	0	0	0	6,00E-04
0	4,23E-05	0	0	0	3,00E-04	3,00E-04	0	2,00E-04	0	0	0	0	0
0	0	0	0	0	0	0	0	0	0	0	0	0	0
6,53E-05	0	0	0	0	8,95E-06	0	0	0	0	0	0	0	0
0	0	0	0	0	4,48E-05	0	0	3,23E-05	1,00E-04	0	0	0	0
0	0	0	0	0	0	0	0	0	0	0	0	0	0
0	0	0	0	0	0	0	0	0	0	0	0	0	0
1,00E-04	0	0	0	2,00E-04	5,43E-05	0	0	2,00E-04	5,00E-04	0	0	0	0
0	0	0	0	0	3,59E-05	0	0	0	0	0	0	0	0
0	0	0	0	0	8,96E-06	0	0	0	0	0	0	0	0
0	0	0	0,0062	0	3,10E-05	2,00E-04	4,55E-05	3,00E-04	0	0	0	0,0062	0
0	0	0	0	0	0	0	0	0	0	0	0	0	0
0	0	0	0	0	0	0	0	0	0	0	0	0	0
6,53E-05	0	0	0	0	1,79E-05	0	0	3,23E-05	1,00E-04	0	0	0	0
6,53E-05	0	0	0	0	0	0	0	0	0	0	0	0	0
0	0	0	0	0	0	0	0	0	0	0	0	0	0
0	0	0	0	0	0	0	0	0	0	0	0	0	0
7,00E-04	9,00E-04	0	0	0	0,0032	0	4,00E-04	0,0013	2,00E-04	0	0	0	0
0	0	0	0	0	0	0	0	0	0	0	0	0	0
0,0038	2,00E-04	0	5,80E-05	0	8,96E-06	0	3,25E-05	0,001	0,0037	0	0	0	0
0	0	0	0	0	0	0	0	0	0	0	0	0	0
0	0	0	0	0	0	0	0	0	0	0	0	0	0
0	0	0	0	0	1,00E-04	0	2,00E-04	3,23E-05	0	0	0	0	0
0	2,98E-05	0	0	0	8,96E-06	0	0	3,23E-05	0	0	0	0	0
7,62E-05	3,55E-05	0	6,62E-05	0	2,08E-05	0	0	3,23E-05	0	0	0	0	0
0	0	0	0	0	1,00E-04	0	0	9,73E-05	0	0	0	0	0
0	0	0	7,00E-04	0	0	0	0	0	0	0	0	0	0
0	0	0	0	0	0	0	0	0	0	0	0	0	0
0	5,96E-05	0	0	0	3,58E-05	2,00E-04	0	9,70E-05	0	0,0012	0	0	0
0	0	0	0	0	0	0	0	0	0	0	0	0	0
0	0	0	0	0	0	0	0	0	0	0	0	0	0
0	0	0	0	0	0	0	0	0	0	0	0	0	0
0	0	0	0	0	0	0	0	0	0	0	0	0	0
2,00E-04	0	0	0	0	8,96E-06	0	0	3,23E-05	1,00E-04	0	0	0	0

0	0	0	0	0	0	0	0	0	0	0	0	0	0
0	0	0	0	0	0	0	0	0	0	0	0	0	0
0	0	0	0	0	0	0	0	0	0	0	0	0	0
6,55E-05	0	0	0	0	0	0	3,25E-05	3,23E-05	1,00E-04	0	0	0	0
6,53E-05	2,98E-05	0	5,80E-05	0	8,06E-05	7,00E-04	0,0094	3,23E-05	0	0,0012	0	0	0
0	0	0	0	0	9,09E-06	0	0	0	0	0	0	0	0
0	0	0	0	0	0	0	0	0	0	0	0	0	0
0	1,00E-04	0	0	0	4,48E-05	0	0	3,23E-05	0	0	0	6,00E-04	0
6,56E-05	9,00E-04	0	0	0	3,00E-04	2,00E-04	0	2,00E-04	1,00E-04	0	0	0	0
3,00E-04	6,00E-04	1,00E-04	2,00E-04	0	9,85E-05	2,00E-04	9,75E-05	6,47E-05	1,00E-04	0	0	0	0
0	5,96E-05	0	0	0	1,79E-05	0	0	0	0	0	0	0	0
0	0	0	0	0	0	0	0	0	0	0	0	0	0
0	3,21E-05	0	0	0	0	0	0	0	0	0	0	0	0
6,54E-05	5,96E-05	0	0	4,49E-05	4,00E-04	0	3,25E-05	5,00E-04	1,00E-04	0	0	0	0
0	2,98E-05	0	5,80E-05	0	8,96E-06	0	0	0	0	0	0	0	0
0	0	0	0	0	0	0	0	0	0	0	0	0	0
0	0	0	0	0	0	0	0	0	0	0	0	0	0
0	0	0	0	0	0	0	0	0	0	0	0	0	0
0	0	0	0	0	1,00E-04	2,00E-04	0	1,00E-04	0	0	0	0	0
0	2,00E-04	0	0	0	0	0	0	0	0	0	0	0	0
0	0	0	0	0	0	0	0	0	0	0	0	0	0
6,53E-05	0	0	0	0	8,95E-06	0	0	0	0	0	0	0	0
2,00E-04	0	0	0	0	4,73E-05	0	6,90E-05	9,69E-05	3,00E-04	0	0	0	0
0,0014	0	0	0	0	9,97E-06	0	0	5,00E-04	0,0018	0	0	0	0
0	0	0	5,98E-05	0	0	0	0	0	0	0	0	0	0
0	0	0	0	0	0	0	0	0	0	0	0	0	0
0	0	0	0,0018	0	0	2,00E-04	0	2,00E-04	0	0	0	0,0037	0
3,00E-04	1,00E-04	2,00E-04	4,00E-04	2,00E-04	8,47E-05	4,00E-04	2,00E-04	9,71E-05	0	0	0	0	6,00E-04
0	0	0	0	0	0	0	0	0	0	0	0	0	0
0	0	0	0	0	0	0	0	0,0016	0	0	0	0,029	0
0	0	0	5,80E-05	0	1,79E-05	0	0	0	0	0	0	0	0
0	0	0	0	0	0	0	0	0	0	0	0	0	0
0	0	0	0	0	0	0	0	0	0	0	0	0	0
0	8,93E-05	0	5,80E-05	0	3,58E-05	0	3,25E-05	3,23E-05	0	0	0	0	0
0	0	0	0	0	0	0	0	0	0	0	0	0	0
0	0	0	0	0	0	0	0	0	0	0	0	0	0

0	0	0	0	0	9,01E-06	0	0	3,23E-05	0	0	0	0	3,00E-04
0	0	0	0	0	0	0	0	0	0	0	0	0	0
0	0	0	0	0	0	0	0	0	0	0	0	0	0
0	0	0	0	0	0	0	0	0	0	0	0	0	0
0	0	0	0	0	0	0	0	0	0	0	0	0	0
0	0	0	0	0	0	0	0	0	0	0	0	0	0
1,00E-04	0	0	9,77E-05	7,00E-04	6,00E-04	3,00E-04	0	3,00E-04	2,00E-04	0	0	0	0,0011
0	0	0	0	0	0	0	0	0	0	0	0	0	0
0	0	0	0	0	0	0	0	0,0012	0,0043	0	0	0	0
0	2,98E-05	0	5,80E-05	4,49E-05	0	0	0	3,23E-05	0	0	0	0	0
0	0	0	0	0	0	0	0	0	0	0	0	0	0
0	0	0	0	0	0	0	0	0	0	0	0	0	0
0	0	0	0	0	0	0	0	0	0	0	0	0	0
0	0	0	0	0	0	0	0	0	0	0	0	0	0
0,0012	0	0	0	0	0	2,00E-04	0	6,00E-04	0,0021	0	0	0	0
0	0	0	5,80E-05	0	8,97E-06	0	6,50E-05	0	0	0	0	0	0
0	0	0	0	0	1,92E-05	0	0	0	0	0	0	0	0
0	0	0	1,00E-04	0	0	0	0	0	0	0	0	0	0
0	0	0	0	0	0	0	0	0	0	0	0	0	0
5,00E-04	0	0	0	0	0	0	0	1,00E-04	2,00E-04	0	0	0	0
0	0	0	0	4,49E-05	1,00E-04	0	0	0	0	0	0	0	0
5,00E-04	0	0	0	0	0	0	0	1,00E-04	5,00E-04	0	0	0	0
0	2,00E-04	0	0	0	1,80E-05	0	0	0	0	0	0	0	0
6,55E-05	1,00E-04	1,00E-04	0	0	3,00E-04	2,00E-04	0	6,47E-05	0	0	0	0	0
0	0	0	0	0	0	0	0	0	0	0	0	0	0
0	0	0	0	0	0	0	0	0	0	0	0	0	0
6,54E-05	0	0	0	0	0	0	0	0	0	0	0	0	0
0	0	0	0	0	1,00E-04	0	0	9,69E-05	0	0	0	0	0
0	3,10E-05	0	0	0	0	0	0	0	0	0	0	0	0
0	0	0	0	0	0	0	3,65E-05	0	0	0	0	0	0
0	0	0	0	0	8,96E-06	2,00E-04	0	3,24E-05	1,00E-04	0	0	0	0
0	0	0	0	0	0	0	0	0	0	0	0	0	0
6,56E-05	2,99E-05	0	0	0	0	0	0	6,46E-05	2,00E-04	0	0	0	0
0	0	0	0	0	0	0	0	0	0	0	0	0	0
0	0	0	3,00E-04	0	0	0	0	0	0	0	0	0	0
0,0014	0	0	0	0	1,79E-05	0	0	3,00E-04	0,0011	0	0	0	0

0,0017	1,00E-04	0	0	0	8,97E-06	4,00E-04	6,50E-05	7,00E-04	0,0024	0,0012	0	0	0
0	0	0	9,00E-04	8,97E-05	0	0	0	0	0	0	0	0	0
6,54E-05	0	0,0017	0	0	3,00E-04	4,00E-04	1,00E-04	9,69E-05	0	0	0,0066	0	0
0	0	0	0	0	0	0	0	0	0	0	0	0	0
0	0	0	0	0	0	0	0	0	0	0	0	0	0
0	0	0	0	0	0	0	0	0	0	0	0	0	0
0	0,0013	0	0	0	1,87E-05	6,00E-04	0	3,23E-05	0	0,0012	0	0	0
0	0	0	0	0	0	3,00E-04	0	6,46E-05	0	0	0	0	3,00E-04
0	0	0	0	4,48E-05	0	0	3,25E-05	0	0	0	0	0	0
0	0	0	0	0	0	0	0	0	0	0	0	0	0
0	0	0	0	0	0	0	0	0	0	0	0	0	0
0	0	0	0	0	0	0	0	3,23E-05	1,00E-04	0	0	0	0
0	0	0	0	0	0	0	0	0	0	0	0	0	0
0	0	0	0	0	0	0	0	0	0	0	0	0	0
0	0	0	0	0	0	0	0	0	0	0	0	0	0
0	0	0	0	0	0	0	0	4,00E-04	0,0014	0	0	0	0
0	0	0	0	0	0	0	0	0	0	0	0	0	0
0	0	0	6,10E-05	0	9,30E-06	0	0	3,23E-05	0	0	0	6,00E-04	0
2,00E-04	2,00E-04	0	0	0,0014	0,0016	0,0015	0,0016	0,0011	0	0	0,0033	0	9,00E-04
0	0	0	0	0	0	0	0	0	0	0	0	0	0
0	0	0	0	0	9,81E-06	0	0	0	0	0	0	0	0
0	5,96E-05	0	5,80E-05	0	7,17E-05	0	0	0	0	0	0	0	0
0	0	0	0	0	6,29E-05	0	0	0	0	0	0	0	0
0	0	0	0	0	8,95E-06	0	0	0	0	0	0	0	0
6,55E-05	7,00E-04	0	0	0	1,79E-05	0	0	0	0	0	0	0	0
0	0	0	0	0	0	0	0	0	0	0	0	0	0
6,53E-05	5,96E-05	0	0	0,0013	0,0011	4,00E-04	0	5,00E-04	2,00E-04	0	0	0	6,00E-04
0	0	0	0	0	0	0	0	0	0	0	0	0	0
0	2,98E-05	0	0	0	1,00E-04	0	0	2,00E-04	2,00E-04	0	0	0	0
0,001	2,00E-04	0	0	0	2,69E-05	0	0	3,00E-04	0,001	0	0	0	0
0	0	0	0	0	0	0	0	0	0	0	0	0	0
0	0	0	0,0022	0	0	2,00E-04	0	0	0	0	0	0	0
0	0	0	0	0	0	0	0	0	0	0	0	0	0
3,00E-04	0	0	0	0	0	0	0	0	0	0	0	0	0
0	0	0	0	0	9,14E-05	0	6,98E-05	9,69E-05	0	0	0	0	0
0	0	0	0	0	0	0	0	0	0	0	0	0	0

0	0	0	0	0	0	0	0	7,00E-04	0,0025	0	0	0	0
0	0	0	0	0	0	0	0	0	0	0	0	0	0
0	0	0	0	0	0	0	0	0	0	0	0	0	0
0	0	0	0	0	0	0	0	0	0	0	0	0	0
0	0	0	0	0	8,98E-06	0	3,32E-05	3,23E-05	1,00E-04	0	0	0	0
0	0	0	0	0	0	0	0	0	0	0	0	0	0
0,0017	1,00E-04	0	0	0	8,99E-06	0	0	4,00E-04	0,0013	0	0	0	0
0	0	0	0	0	0	0	0	0	0	0	0	0	0
0	0	0	0	0	0	0	0	0	0	0	0	0	0
0	0	0	0	0	0	0	0	0	0	0	0	0	0
0	0	0	0	0	0	0	0	0	0	0	0	0	0
0	0	0	0	0	0	0	0	0	0	0	0	0	0
0	0	0	0	0	0	0	0	0	0	0	0	0	0
0	0	0	0	0	0	0	0	0	0	0	0	0	0
0	5,96E-05	0	0	0	8,95E-06	0	0	3,23E-05	0	0	0	0	0
6,54E-05	0	0	0	0	2,69E-05	0	0	3,23E-05	1,00E-04	0	0	0	0
0	1,00E-04	0	0	0	4,48E-05	2,00E-04	3,25E-05	6,46E-05	0	0	0	0	0
0	0	0	0	0	0	0	0	0	0	0	0	0	0
0	0	0	0	0	0	0	0	3,23E-05	0	0	0	0	0
0	0	0	0	0	0	0	0	0	0	0	0	0	0
0	0	0	0	0	0	0	0	0	0	0	0	0	0
0	0	0	0	0	3,59E-05	0	3,25E-05	0	0	0	0	0	0
0	2,99E-05	0	5,81E-05	0	4,52E-05	0	0	0	0	0	0	0	0
1,00E-04	1,00E-04	1,00E-04	5,80E-05	0,0029	4,00E-04	5,00E-04	0	9,00E-04	0	0,0012	0	0	0,0026
0,004	9,14E-05	0	0	0	1,00E-04	4,00E-04	3,36E-05	0,0014	0,0043	0	0	0	0
0	0	0	0	0	0	0	0	0	0	0	0	0	0
0	0	0	0	0	0	0	0	0	0	0	0	0	0
0	0	0	0	0	0	0	0	0	0	0	0	0	0
0	0	0	0	0	0	0	0	0	0	0	0	0	0
4,00E-04	0	0	0	0	0	0	0	9,70E-05	3,00E-04	0	0	0	0
0	0	0	0	0	0	0	0	0	0	0	0	0	0
4,00E-04	0	0	0	0	0	0	0	1,00E-04	5,00E-04	0	0	0	0
0	0	0	0	0	0	0	0	0	0	0	0	0	0
0	0	0	0	0	3,44E-05	0	4,42E-05	0	0	0	0	0	0
0	4,18E-05	0	9,77E-05	0	0	0	0	0	0	0	0	0	0
0	2,00E-04	3,00E-04	2,00E-04	4,00E-04	3,00E-04	4,00E-04	6,72E-05	3,00E-04	3,00E-04	0	0	6,00E-04	6,00E-04

0	1,00E-04	0	0	0	9,16E-06	0	0	0	0	0	0	0	0
0	0	0	0	0	0	0	0	0	0	0	0	0	0
0	0	1,00E-04	0	0	0	0	0	0	0	0	0	0	0
0	0	0	0	0	0	0	0	0	0	0	0	0	0
0	0	0	0	0	0	0	0	0	0	0	0	0	0
2,00E-04	5,00E-04	0	0	5,62E-05	1,00E-04	6,00E-04	3,34E-05	2,00E-04	3,00E-04	0	0	0	0
0,0012	2,98E-05	0	0	0	0	0	0	2,00E-04	8,00E-04	0	0	0	0
5,00E-04	3,00E-04	1,00E-04	5,00E-04	0	0	0	9,78E-05	1,00E-04	5,00E-04	0	0	0	0
0	2,00E-04	0	0	0	6,48E-05	0	3,25E-05	3,23E-05	0	0	0	0	0
0	0	0	0	0	0	0	0	0	0	0	0	0	0
6,54E-05	0	0	0	0	0	0	0	0	0	0	0	0	0
0	0	0	0	0	0	0	0	0	0	0	0	0	0
0	0	0	0	3,00E-04	3,00E-04	2,00E-04	0	7,00E-04	0	0	0	0	0
0	0	0	0	0	0	0	0	0	0	0	0	0	0
0	0	0	0	0	0	0	0	0	0	0	0	0	0
0	0	0	0,0065	0	0	7,00E-04	4,25E-05	5,00E-04	0	0	0	0,0089	0
6,57E-05	0	0	0	0	0	0	0	3,24E-05	1,00E-04	0	0	0	0
0	0	0	0	0	0	0	0	0	0	0	0	0	0
0	0	0	0	0	0	0	0	0	0	0	0	0	0
0	0	0	0	0	0	0	0	0	0	0	0	0	0
0	0	0	2,00E-04	0	0	0	0	6,46E-05	0	0	0	0	0
0	0	0	0	0	0	0	0	0	0	0	0	0	0
6,53E-05	2,98E-05	0	0	0	8,06E-05	0	0	0	0	0	0	0	0
0	0	0	0	0	0	0	0	0,0024	2,00E-04	0	0	0,0426	6,00E-04
0	0	0	0	0	0	0	0	0	0	0	0	0	0
1,00E-04	8,00E-04	0	0	4,48E-05	7,00E-04	2,00E-04	0	2,00E-04	0	0,0012	0	0	0
0	0	0	0	0	0	3,00E-04	0	0	0	0	0	0	0
0	0	0	0	0	0	0	0	0	0	0	0	0	0
0	0	0	0	0	8,97E-06	0	0	0	0	0	0	0	0
0	0	0	0	0	0	0	0	0	0	0	0	0	0
0	0	0	0	0	0	0	0	0	0	0	0	0	0
0	0	0	0	0	1,99E-05	0	2,00E-04	0	0	0	0	0	0
0	0	0	0	0	0	0	0	0	0	0	0	0	0
0	0	0	0	0	0	0	0	0	0	0	0	0	0
0	2,98E-05	0	0	0	0	0	0	0	0	0	0	0	0
0	0	0	0	0	0	0	0	0	0	0	0	0	0

0	0	0	0	0	0	0	0	0	0	0	0	0	0
2,00E-04	0	0	0	0	2,69E-05	0	9,75E-05	6,46E-05	1,00E-04	0	0	0	0
7,08E-05	4,26E-05	0	0	0	6,89E-05	0	4,08E-05	3,25E-05	0	0	0	0	0
0,0018	0	0	6,80E-05	0	0	0	0,0019	7,00E-04	0,0023	0	0	0,0012	0
0	0	0	0	0	0	3,00E-04	0	6,84E-05	2,00E-04	0	0	0	0
6,54E-05	0	0	0	0	0	0	0	0	0	0	0	0	0
0	0	0	0	0	0	0	0	0	0	0	0	0	0
0	0	0	0	0	0	0	0	1,00E-04	1,00E-04	0	0	0,0019	0
0	0	0	0	0	0	0	0	0	0	0	0	0	0
0	0	0	0	0	0	0	0	0	0	0	0	0	0
0	0	0	0	0	0	0	0	0	0	0	0	0	0
0	5,96E-05	0	2,00E-04	0	8,99E-06	0	6,50E-05	6,47E-05	0	0	0	0,0012	0
0	8,00E-04	0	0	0	9,62E-05	0,0011	0	6,47E-05	0	0,0012	0	0	0
0	0	0	0	0	0	0	0	0	0	0	0	0	0
0	0	0	0	0	0	0	0	0	0	0	0	0	0
0	0	0	0	0	0	0	0	0	0	0	0	0	0
6,60E-05	0	0	0	0	0	2,00E-04	3,25E-05	0	0	0	0	0	0
0	0	0	0	0	0	0	0	0	0	0	0	0	0
0,001	0	0	0	0	0	0	0	4,00E-04	0,0014	0	0	0	0
0	0	0	0	0	0	0	0	0	0	0	0	0	0
0	0	0	0	0	0	0	0	0	0	0	0	0	0
0	2,99E-05	0	0	0	7,17E-05	0	0	3,24E-05	0	0	0	0	0
0	0	0	0	0	3,60E-05	0	0	0	0	0	0	0	0
0	0	0	0	0	0	0	0	0	0	0	0	0	0
6,59E-05	0	0	0	0,0053	0,0013	5,00E-04	0	0,0011	0	0	0	0	0,0057
0,0014	5,96E-05	0	5,80E-05	0	8,97E-06	0	0	1,00E-04	5,00E-04	0	0	0	0
0	0	0	2,00E-04	0	0	0	0	3,23E-05	0	0	0	6,00E-04	0
0	0	0	0	0	0	0	0	6,48E-05	0	0	0	0	0
0	0	0	0	0	8,98E-06	0	0	0	0	0	0	0	0
0	8,94E-05	0	0	0	0	0	0	3,23E-05	1,00E-04	0	0	0	0
3,00E-04	0	0	0	0	0	0	0	2,00E-04	8,00E-04	0	0	0	0
0	0	0	0	0	0	0	0	0	0	0	0	0	0
6,56E-05	8,94E-05	1,00E-04	0	0	1,00E-04	2,00E-04	0	6,47E-05	0	0	0	0	0
6,55E-05	1,00E-04	0	0	4,49E-05	0,001	0	0,0011	7,00E-04	5,00E-04	0	0	0	3,00E-04
0	4,17E-05	0	0	0	0	0	0	3,23E-05	0	0	0	0	0
0	8,95E-05	0	0	0	0	0	0	0	0	0	0	0	0

6,59E-05	5,96E-05	0	0	2,00E-04	2,00E-04	4,00E-04	0	2,00E-04	0	0	0	0	3,00E-04
6,53E-05	0	0	0	0	4,48E-05	0	3,25E-05	9,68E-05	1,00E-04	0	0	0	0
0	0	0	0	0	0	0	0	0	0	0	0	0	0
0	0	0	0	0	1,09E-05	0	0	0	0	0	0	0	0
0	2,98E-05	0	0	0	0	0	0	0	0	0	0	0	0
0	0	0	0	0	0	0	0	0	0	0	0	0	0
0	1,00E-04	0	0	0	1,80E-05	0	0	0	0	0	0	0	0
0	0	0	0	0	9,65E-06	0	0	3,23E-05	0	0	0	0	0
0	0	0	5,81E-05	8,97E-05	9,87E-05	0	0	3,23E-05	0	0	0	0	0
0	0	0	0	0	0	0	0	0	0	0	0	0	0
0	0	0	0	0	0	0	0	7,00E-04	0,0023	0	0	0	0
0	0	0	0	0	0	0	0	7,00E-04	0,0023	0	0	0	0
0	2,00E-04	0	0	0	8,96E-06	0	0	3,24E-05	0	0	0	0	0
0	0,001	0,0059	0	0	8,49E-05	0	0	2,00E-04	0	0	0,0166	0	0
0	0	0	0	0	0	0	0	0	0	0	0	0	0
0	0	0	0	0	0	0	0	0	0	0	0	0	0
0,0046	2,00E-04	0	0	0	3,44E-05	0	0	0,0014	0,005	0,0012	0	0	0
0	0	0	0	0	0	0	0	0	0	0	0	0	0
0	0	0	0	0	0	0	0	0	0	0	0	0	0
0	0	0	0	8,97E-05	0	0	0	0	0	0	0	0	0
0,0023	8,44E-05	0	0	0	1,99E-05	6,00E-04	4,44E-05	5,00E-04	0,0016	0	0	0	0
6,54E-05	0	0	0	0	8,96E-06	0	3,25E-05	0	0	0	0	0	0
0	0	0	0	0	1,80E-05	0	0	0	0	0	0	0	0
0	0	0	0	0	0	0	0	3,25E-05	0	0	0	0	0
0	0	0	0	0	0	0	0	0	0	0	0	0	0
0,0047	3,00E-04	0	0	0	0	6,00E-04	4,61E-05	0,0013	0,0045	0	0	0	0
0	0	0	0	0	0	0	0	0	0	0	0	0	0
0	0	0	0	0	0	0	0	0	0	0	0	0	0
0	2,98E-05	0	5,80E-05	0	8,95E-06	2,00E-04	3,25E-05	0	0	0	0	0	0
0	0	0	0	0	0	2,00E-04	0	3,23E-05	0	0	0	0	0
0	3,03E-05	0	0	0	9,05E-06	0	0	0	0	0	0	0	0
0	0	0	0	0	0	0	0	0	0	0	0	0	0
0	0	0	0	0	0	0	0	3,23E-05	0	0	0	0	0
0	0	0	0	0	1,80E-05	0	0	0	0	0	0	0	0
0	0	1,00E-04	5,80E-05	0	6,27E-05	0	0	0	0	0	0	0	0
0	0	0	0	0	0	0	0	0	0	0	0	0	0

0	0	0	0	0	0	0	0	0	0	0	0	0	0
0	5,96E-05	1,00E-04	0	0	1,00E-04	0	0	3,23E-05	1,00E-04	0	0	0	0
2,00E-04	0	0	9,00E-04	0	5,49E-05	0	0	2,00E-04	2,00E-04	0	0	0,0025	0
0	0	0	0	0	0	0	0	0	0	0	0	0	0
0	0	0	0	0	0	0	0	0	0	0	0	0	0
9,80E-05	0	0	0	0	0	0	0	0	0	0	0	0	0
0	2,98E-05	0	0	0	0	2,00E-04	0	0	0	0	0	0	0
0	0	0	0	0	0	0	0	0	0	0	0	0	0
0	0	0	0	0	0	0	0	0	0	0	0	0	0
0	0	0	0	0	8,96E-06	0	0	0	0	0	0	0	0
0,0022	3,00E-05	3,00E-04	0	0	2,70E-05	2,00E-04	0	5,00E-04	0,0018	0	0	0	0
0	0	0	0	0	0	0	0	0	0	0	0	0	0
0	3,09E-05	0	0	0	9,92E-06	0	0	0	0	0	0	0	0
0	0	0	0	0	0	0	0	0	0	0	0	0	0
0	0	0	0	0	0	0	0	0	0	0	0	0	0
0	2,98E-05	1,00E-04	0	0	8,97E-06	0	0	0	0	0	0	0	0
0	0	0	0	0	0	0	0	0	0	0	0	0	0
0	0	0	0	0	0	0	0	0	0	0	0	0	0
0	0	0	0	0	8,96E-06	0	0	0	0	0	0	0	0
0	1,00E-04	0	5,91E-05	0	1,00E-04	0,0013	0,0097	6,51E-05	0	0	0	0	0
0	0	0	0	0	3,60E-05	2,00E-04	0,002	0	0	0	0	0	0
0,0022	2,98E-05	0	0	0	8,96E-06	0	3,25E-05	5,00E-04	0,0016	0	0	0	0
0	0	0	0	0	0	0	0	0	0	0	0	0	0
1,00E-04	2,00E-04	0	5,81E-05	0,0054	2,00E-04	9,00E-04	0,0021	6,00E-04	1,00E-04	0	0	0	0,0037
0	0	0	0	0	2,77E-05	0	2,00E-04	3,00E-04	0	0,0014	0	0,0033	0
0	0	0	0	0	2,75E-05	0	1,00E-04	3,00E-04	0	0,0014	0	0,0033	0
0	5,17E-05	0	0	0	0	0	0	0,0019	0,0062	0	0	0	4,00E-04
0	5,18E-05	0	0	0	0	0	0	0,0019	0,0062	0	0	0	4,00E-04
6,00E-04	2,00E-04	9,00E-04	0	0,0015	3,00E-04	7,00E-04	5,20E-05	0,0017	0,0014	0	0	0	0,0033
7,00E-04	2,00E-04	9,00E-04	0	0,0015	3,00E-04	8,00E-04	5,29E-05	0,0019	0,002	0	0	0	0,0033
0	0	0	0	0	0	0	0	0,0023	0,0076	0	0	0	0
0	0	0	0	0	0	0	0	0	0	0	0	0	0
0	0	0	5,00E-04	0	0	0	0	0	0	0	0	0	0
9,00E-04	2,98E-05	0	0	0	1,81E-05	2,00E-04	0	2,00E-04	8,00E-04	0	0	0	0
0	0	0	0	0	0	0	0	0	0	0	0	0	0
0	0	0	0	0	0	0	0	0	0	0	0	0	0

1,00E-04	0	0	5,81E-05	0	3,62E-05	0	0	6,46E-05	2,00E-04	0	0	0	0
0	2,98E-05	0	0	0	0	0	0	0	0	0	0	0	0
0	4,17E-05	0	0	5,94E-05	3,00E-04	3,00E-04	0	1,00E-04	0	0	0	0	0
0	0	0	0	0	9,05E-06	0	0	0	0	0	0	0	0
0	0	0	0	0	0	0	0	0	0	0	0	0	0
0	0	0	0	0	0	0	0	0	0	0	0	0	0
0	0	0	0	0	0	0	0	0	0	0	0	0	0
0	1,00E-04	0	0	0	9,72E-05	0	0	9,69E-05	1,00E-04	0	0	0	0
0	0	0	0	0	0	0	0	0	0	0	0	0	0
7,00E-04	2,00E-04	0	0	0	0,0033	6,00E-04	0	0,0021	0,0011	0	0	0	0,0014
0	2,98E-05	0	0	0	8,97E-06	0	0	0	0	0	0	0	0
5,00E-04	8,95E-05	0	0	0	0	2,00E-04	0	9,69E-05	3,00E-04	0	0	0	0
0	0	0	0	0	0	0	0	3,23E-05	1,00E-04	0	0	0	0
0	0	0	0	0	0	0	0	0	0	0	0	0	0
0	0	0	0	0	0	0	0	0	0	0	0	0	0
0	2,98E-05	0	0	0	0	0	0	0	0	0	0	0	0
6,55E-05	0	0	3,00E-04	0	2,70E-05	0	9,78E-05	3,23E-05	0	0	0	0	0
0	0	0	0	0	9,04E-06	0	0	0	0	0	0	0	0
0	4,00E-04	0	0	0	2,00E-04	2,00E-04	0	9,68E-05	0	0,0012	0	0	0
0	0	0	0	0	0	0	0	0	0	0	0	0	0
0	0	0	0	0	0	0	0	0	0	0	0	0	0
0	0	0	0	0	0	0	0	0	0	0	0	0	0
0,0018	6,40E-05	0	0	0	9,48E-06	4,00E-04	0	4,00E-04	0,0013	0	0	0	0
0	2,98E-05	0	0	0	8,98E-06	2,00E-04	0	0	0	0	0	0	0
0	0	0	0	0	0	0	0	0	0	0	0	0	0
0	3,02E-05	0	0	0	9,21E-06	0	0	0	0	0	0	0	0
0	0	0	0	0	0	0	0	0	0	0	0	0	0
0	0	0	0	0	0	0	0	0	0	0	0	0	0
0	0	0	0	0	0	0	0	0	0	0	0	0	0
0	0	0	0	0	2,71E-05	0	0	0	0	0	0	0	0
0	0	0	0	0	0	0	0	0	0	0	0	0	0
0	0	0	0	0	0	0	0	0	0	0	0	0	0
0,0057	3,00E-04	0	0	0	3,59E-05	2,00E-04	0	0,0015	0,0055	0	0	0	0
0	0	0	0	0	0	0	0	0	0	0	0	0	0
0	0	0	0	0	0	0	0	0	0	0	0	0	0
0	2,98E-05	0	0	0	0	0	0	0	0	0	0	0	0

0	0	0	0	0	0	0	0	0	0	0	0	0	0
8,00E-04	1,00E-04	0	0	0	9,45E-06	0	0	0,0017	0,0081	0	0	0	0
0	0	0	0	0	0	0	0	0	0	0	0	0	0
0,0018	0	0	0	0	9,00E-06	2,00E-04	0	5,00E-04	0,0017	0	0	0	0
0	0	0	0	0	0	0	3,25E-05	0	0	0	0	0	0
6,53E-05	0	0	0	0	8,95E-06	0	0	6,46E-05	1,00E-04	0	0	0	0
0	0	0	0,0012	0	0	0	0	0	0	0	0	0	0
0	8,93E-05	0	0	0	0	0	0	0	0	0	0	0	0
0	0	0	0	0	8,95E-06	2,00E-04	3,25E-05	3,23E-05	1,00E-04	0	0	0	0
0	0	0	0	0	0	2,00E-04	0	0	0	0	0	0	0
0	0	0	0	0	0	0	0	0	0	0	0	0	0
0	0	0	0	0	0	0	0	0	0	0	0	0	0
0	8,93E-05	0	0	0	7,16E-05	0	0	0	0	0	0	0	0
0	0	0	0	4,49E-05	7,17E-05	0	0	0	0	0	0	0	0
0	0	0	0	0	0	0	0	0	0	0	0	0	0
0	0	0	0	0	0	0	0	0	0	0	0	0	0
0	2,98E-05	0	0	0	0	0	0	0	0	0	0	0	0
0	5,00E-04	5,00E-04	0	0	2,00E-04	0,0013	0	9,68E-05	0	0	0	0	0
0	3,00E-05	0	0	0	3,60E-05	0	0	6,46E-05	0	0	0	0	0
0	2,98E-05	0	0	0	1,00E-04	0	1,00E-04	1,00E-04	3,00E-04	0	0	0	0
0	0	0	0	0	0	0	0	6,47E-05	0	0	0	0	0
0	0	0	0	0	0	0	0	0	0	0	0	0	0
0	1,00E-04	0	0	8,00E-04	6,00E-04	2,00E-04	0	0,001	0	0	0	0	6,00E-04
0	0	0	0	0	8,97E-06	0	0	0	0	0	0	0	0
0	1,00E-04	0	0	0	8,97E-06	0	0	0	0	0	0	0	0
0	0	0	0	0	1,20E-05	0	0	0	0	0	0	0	0
0,0031	0	0	0	0	0	0	0	8,00E-04	0,0027	0	0	0	0
0	0	0	0	0	2,97E-05	0	0	0	0	0	0	0	0
0	3,04E-05	0	0	0	0	0	0	0	0	0	0	0	0
0	0	0	0	0	0	0	0	0	0	0	0	0	0
0	0	0	0	0	8,97E-06	0	0	0	0	0	0	0	0
0	0	0	0	0	0	0	0	0	0	0	0	0	0
0	0	0	0	0	0	0	0	0	0	0	0	0	0
0	0	0	0	0	0	0	0	0	0	0	0	0	0
4,00E-04	6,88E-05	0	0	0	0	0	0	2,00E-04	7,00E-04	0	0	0	0
0	0	0	0	4,49E-05	6,28E-05	0	3,26E-05	0	0	0	0	0	0

0	0	0	0	0	0	0	0	3,23E-05	0	0	0	0	0
0	0	0	0	0	0	0	0	0	0	0	0	0	0
0	0	0	0	0	0	0	0	0	0	0	0	0	0
0	0	0	0	0	0	0	0	0	0	0	0	0	0
0,0024	3,11E-05	0	0	0	0	0	0	9,00E-04	0,0033	0	0	0	0
0	2,98E-05	0	0	4,48E-05	8,95E-05	0	0	0	0	0	0	0	0
4,00E-04	3,00E-04	0,0039	0	6,00E-04	0,0011	5,00E-04	0	5,00E-04	2,00E-04	0	0,0033	0	6,00E-04
0	0	0	0	0	0	0	0	0	0	0	0	0	0
0	1,00E-04	0	0	0	6,30E-05	0	0	9,68E-05	0	0	0	0	0
0	0	0	0	0	1,79E-05	0	0	0	0	0	0	0	0
0	4,00E-04	0	0	0	4,86E-05	4,00E-04	0	3,23E-05	0	0,0012	0	0	0
0	2,00E-04	0	0	0	3,63E-05	0	0	6,46E-05	0	0	0	0	0
0	0	0	0	0	0	0	0	0	0	0	0	0	0
0	3,03E-05	0	0	0	1,87E-05	0	0	0	0	0	0	0	0
0	0	0	0	8,97E-05	0	2,00E-04	0	6,46E-05	0	0	0	0	3,00E-04
0	2,00E-04	0	0	0	3,00E-04	4,00E-04	0	3,23E-05	0	0	0	0	0
0	0	0	0	0	0	0	0	0	0	0	0	0	0
0	0	0	0	0	0	0	0	0	0	0	0	0	0
0,0078	5,98E-05	0	0	0	2,76E-05	0	6,54E-05	0,0019	0,0065	0,0012	0	0	0
0	0	0	0	0	0	0	0	0	0	0	0	0	0
1,00E-04	2,98E-05	0	0	0	6,00E-04	0	6,50E-05	4,00E-04	2,00E-04	0	0	6,00E-04	0
1,00E-04	2,98E-05	0	0	0	6,00E-04	0	6,50E-05	4,00E-04	2,00E-04	0	0	6,00E-04	0
0	0	0	0	0	0	0	0	0	0	0	0	0	0
0	0	0	0	0	0	0	0	0	0	0	0	0	0
0	0	0	0	0	0	0	0	3,23E-05	0	0	0	0	0
0	0	0	0	0	8,95E-06	0	0	0	0	0	0	0	0
0	0	0	0	0	8,97E-06	0	9,75E-05	0	0	0	0	0	0
0	0	0	0	0	0	0	0	0	0	0	0	0	0
0	0	0	2,00E-04	0	0	0	0	0	0	0	0	0	0
0	0	0	0	0	0	0	0	0	0	0	0	0	0
0	0	0	0	0	8,97E-06	0	0	0	0	0	0	0	0
0	0	0	0	0	0	0	0	0	0	0	0	0	0
0	0	0	0	0	4,48E-05	4,00E-04	0,0011	3,23E-05	0	0	0	0	0
0	0	0	0	0	9,20E-06	0	0	0	0	0	0	0	0
0	0	0	0	0	0	0	0	0	0	0	0	0	0
0	2,98E-05	0	0	0	8,96E-06	0	0	3,23E-05	1,00E-04	0	0	0	0

0	0	0	0	0	0	0	0	3,23E-05	0	0	0	0	0
0,0065	0	0	0	0	8,96E-05	2,00E-04	0	0,0013	0,0046	0	0	0	0
0	0	0	0	0	0	0	0	0	0	0	0	0	0
0	5,96E-05	0	0	0	0	0	0	0	0	0	0	0	0
0	0	0	0	0	0	0	0	0	0	0	0	0	0
0	0	0	0	0	2,69E-05	0	0	0	0	0	0	0	0
4,00E-04	2,98E-05	0	5,80E-05	0,0022	4,00E-04	2,00E-04	2,00E-04	3,00E-04	1,00E-04	0	0	0	6,00E-04
2,00E-04	2,98E-05	0	0	0	3,60E-05	0	3,25E-05	3,23E-05	1,00E-04	0	0	0	0
0	2,00E-04	0	0	1,00E-04	4,16E-05	0	2,00E-04	2,00E-04	4,00E-04	0,0014	0	0	0
7,00E-04	2,00E-04	0	0	4,49E-05	0,0016	6,00E-04	3,26E-05	9,00E-04	3,00E-04	0,0012	0	0	0
0	8,94E-05	0	0	0	2,00E-04	2,00E-04	1,00E-04	1,00E-04	0	0	0	0	0
0	0	0	0	0	0	0	0	0	0	0	0	0	0
2,00E-04	0,0059	0	5,84E-05	0	2,00E-04	2,00E-04	2,00E-04	3,00E-04	3,00E-04	0,006	0	0	0
0	0	0	0	0	0	0	0	0	0	0	0	0	0
0	0	0	1,00E-04	0	2,73E-05	0	0	3,24E-05	0	0	0	0	0
0	0	0	0	0	9,01E-06	0	0	0	0	0	0	0	0
0	9,09E-05	0	0	0	0	0	0	0	0	0	0	0	0
0	0	0	0	0	0	0	0	0	0	0	0	0	0
0	0	0	0	0	0	0	0	0	0	0	0	0	0
0	0	0	6,21E-05	0	0	0	0	0	0	0	0	0	0
6,54E-05	0	0	0	0	3,58E-05	0	0	0	0	0	0	0	0
0	0	0	0	0	0	0	0	0	0	0	0	0	0
0	0	0	0	0	0	0	0	0	0	0	0	0	0
0	0	0	0	0	0	0	0	0	0	0	0	0	0
0	0	0	0	0	0	0	0	0,0023	0,0082	0	0	0	0
0	0	0	0	0	0	0	0	0,0023	0,0081	0	0	0	0
9,00E-04	3,00E-04	0	0	0	0	0	4,55E-05	5,00E-04	0,0017	0	0	0	0
0	3,00E-05	0	0	0	0	2,00E-04	0	0	0	0	0	0	0
0	0	0	0	0	8,95E-06	0	0	3,23E-05	0	0	0	6,00E-04	0
0	0	0	5,84E-05	0	0	0	0	0	0	0	0	0	0
0	1,00E-04	0	0	0	3,73E-05	0	0	3,23E-05	0	0	0	0	0
0	8,35E-05	0	0	0	0	0	0	3,23E-05	0	0,0012	0	0	0
0	4,00E-04	0	5,84E-05	0	8,31E-05	4,00E-04	0	6,46E-05	0	0	0	6,00E-04	0
0	0	0	0	0	0	0	0	0	0	0	0	0	0
0	0	0	0	0	0	0	0	0	0	0	0	0	0
0	0	0	0	0	0	0	0	0	0	0	0	0	0

0	0	0	0	0	0	0	0	6,00E-04	0	0	0,0331	0	0
0	0	0	0	0	0	0	0	0	0	0	0	0	0
6,54E-05	0	0	0	0	4,48E-05	2,00E-04	0	3,24E-05	1,00E-04	0	0	0	0
0	0	0	0	0	0	0	0	0	0	0	0	0	0
2,00E-04	0	0	0	0	0	0	0	0	0	0	0	0	0
0	0	0	0	0	0	0	0	0	0	0	0	0	0
6,54E-05	8,94E-05	0,0016	2,00E-04	0	1,00E-04	4,00E-04	0,0014	6,46E-05	0	0	0,0033	0	0
0	0	0	0	0	0	0	0	0	0	0	0	0	0
0	0	0	0	0	0	0	0	0	0	0	0	0	0
0	0	0	0	0	9,00E-06	0	0	0	0	0	0	0	0
0	0	0	0	0	0	0	0	0	0	0	0	0	0
6,56E-05	0	0	0	0	0	0	0	6,47E-05	2,00E-04	0	0	0	0
0,001	0	0	0	0	0	0	0	1,00E-04	5,00E-04	0	0	0	0
0	0	0	0	0	0	0	0	0	0	0	0	0	0
0	0	0	0	0	1,29E-05	0	0	0	0	0	0	0	0
0	0	0	0	0	8,96E-06	0	0	0	0	0	0	0	0
0	0	0	0	0	8,97E-06	0	0	0	0	0	0	0	0
0	0	0	0	0	0	0	0	0	0	0	0	0	0
0	1,00E-04	0	5,80E-05	4,49E-05	9,85E-05	0	3,25E-05	3,23E-05	0	0	0	0	0
6,54E-05	2,98E-05	0	0	0	5,00E-04	0	0	2,00E-04	1,00E-04	0	0	0	0
0	0	0	0	0	0	0	0	0	0	0	0	0	0
4,00E-04	5,00E-04	5,00E-04	0	0,0016	0,0041	0,0026	0,0056	0,0018	7,00E-04	0,0012	0	0	3,00E-04
0	0	0	0	0	5,19E-05	0	0	0	0	0	0	0	0
0	0	0	0	0	2,17E-05	0	0	3,30E-05	1,00E-04	0	0	0	0
6,54E-05	0	0	0	4,49E-05	8,96E-06	0	0	0	0	0	0	0	0
0	0	0	0	0	0	0	0	0	0	0	0	0	0
0	2,99E-05	0	0	0	1,80E-05	0	0	0	0	0	0	0	0
5,00E-04	2,99E-05	0	0	0	8,98E-06	0	0	2,00E-04	6,00E-04	0	0	0	0
0	0	0	0	0	0	0	0	0	0	0	0	0	0
6,55E-05	5,98E-05	0	0	0	8,99E-06	0	0	0	0	0	0	0	0
0	0	0	0	0	9,08E-06	0	0	0	0	0	0	0	0
0	0	0	0	0	2,70E-05	0	0	3,23E-05	0	0	0	0	0
0	0	0	0	0	0	0	0	3,23E-05	0	0	0	0	0
0	0	0	0	0	0	0	0	0	0	0	0	0	0
0	3,03E-05	0	0	0	9,00E-06	0	0	0	0	0	0	0	0
0	0	0	0	0	0	0	0	0	0	0	0	0	0

1,00E-04	0	0	6,50E-05	0	0	0	0	6,46E-05	2,00E-04	0	0	0	0
0	0	0	0	0	0	0	0	0	0	0	0	0	0
0	0	0	0	0	0	0	0	0	0	0	0	0	0
0	6,06E-05	0	0	0	9,18E-06	0	0	0	0	0	0	0	0
0	0	0	0	0	0	0	0	0	0	0	0	0	0
6,54E-05	3,00E-04	0	0	0	5,00E-04	4,00E-04	0	3,00E-04	2,00E-04	0,0012	0	0	0
0	2,99E-05	0	5,82E-05	0	1,80E-05	0	6,50E-05	3,23E-05	1,00E-04	0	0	0	0
0	0	0	0	0	9,59E-06	0	0	0	0	0	0	0	0
0	2,98E-05	0	0	0	1,79E-05	0	0	0	0	0	0	0	0
0	2,98E-05	0	0	0	0	2,00E-04	0	0	0	0	0	0	0
0	0	0	0	0	0	0	0	0	0	0	0	0	0
0	0	0	0	0	8,95E-06	0	0	0	0	0	0	0	0
0	3,00E-04	0	0	0	8,99E-06	2,00E-04	0	3,23E-05	1,00E-04	0	0	0	0
6,54E-05	0	0	0	0	0	0	0	0	0	0	0	0	0
0	0	0	0	0	9,34E-06	0	0	0	0	0	0	0	0
0	0	0	0	0	0	0	0	0	0	0	0	0	0
0,0015	1,00E-04	0	0	0	0	0	0	2,00E-04	8,00E-04	0	0	0	0
6,54E-05	1,00E-04	0	0	0	2,69E-05	0	0	0	0	0	0	0	0
0	0	0	0	0	9,00E-06	0	0	0	0	0	0	0	0
0	5,96E-05	0	0	0	8,96E-06	0	0	3,23E-05	0	0	0	0	0
0	0	0	0	0	0	0	0	0	0	0	0	0	0
0	0	0	0	0	0	0	0	0	0	0	0	0	0
0	0	0	0	0	0	0	0	0	0	0	0	0	0
0	0	0	5,81E-05	0	9,03E-06	0	3,26E-05	1,00E-04	0	0	0	0	0
0	0	0	0	0	0	0	0	0	0	0	0	0	0
0	0	0	0	0	0	0	0	9,74E-05	3,00E-04	0	0	0	0
0	0	0	0	0	0	0	0	2,00E-04	0	0	0	0	0
0	0	0	0	0	1,79E-05	0	0	6,46E-05	0	0	0	0	0
0,0077	8,94E-05	0	0	0	1,79E-05	4,00E-04	0	0,0018	0,0063	0	0	0	0
0	0	0	0	0	0	0	0	0	0	0	0	0	0
0	0	0	0	5,31E-05	2,00E-04	0	0	6,47E-05	0	0	0	0	0
0	0	0	0	0	0	0	0	0	0	0	0	0	0
0,0021	1,00E-04	0	0,0031	0	5,38E-05	7,00E-04	4,00E-04	9,00E-04	0,0016	0	0	0,0074	0
0	4,24E-05	0	0	0	1,77E-05	0	0	0	0	0	0	0	0
0	0	0	0	0	0	0	0	0	0	0	0	0	0
0,0062	0	0	0	0	0	0	0	0,001	0,0036	0	0	0	0

0	0	0	0	0	0	0	0	0	0	0	0	0	0
0	0	0	0	0	0	0	0	0	0	0	0	0	0
0	0	0	0	0	0	0	0	0	0	0	0	0	0
0	0	0	0	0	1,46E-05	0	0	9,71E-05	0	0	0	0	0
0,0066	2,00E-04	0	0	0	0	2,00E-04	0	0,0016	0,0056	0	0	0	0
0,0013	4,00E-04	0	0	0,0014	0	0	0	4,00E-04	0,001	0	0	0	6,00E-04
0	0	0	0	0	4,48E-05	0	0	0	0	0	0	0	0
0	0	0	0	0	0	0	0	0	0	0	0	0	0
9,00E-04	7,00E-04	3,00E-04	0	0,0027	0,0039	0,0012	0,001	0,0037	0,0013	0	0	0	0,0054
0,0011	7,00E-04	3,00E-04	0	0,0028	0,004	0,0012	9,00E-04	0,0038	0,0015	0	0	0	0,0054
0	0	0	0	0	0	0	0	0	0	0	0	0	0
0	0	0	0	0	1,32E-05	0	0	0	0	0	0	0	0
0	0	0	0	0	0	0	0	0	0	0	0	0	0
0	0	0	0	0	0	0	0	0	0	0	0	0	0
0	0	0	0	0	0	0	0	0	0	0	0	0	0
0	0	0	0	0	0	0	3,25E-05	0	0	0	0	0	0
0	0	0	0	0	0	0	0	0	0	0	0	0	0
4,00E-04	3,00E-04	0	0	0	9,64E-05	2,00E-04	0	1,00E-04	5,00E-04	0	0	0	0
0	0	0	4,00E-04	0	5,81E-05	2,00E-04	2,00E-04	0	0	0	0	0	0
1,00E-04	0	0	0	0	0	0	0	0	0	0	0	0	0
6,54E-05	3,00E-04	0	0	2,00E-04	0	0	0	9,69E-05	2,00E-04	0	0	0	3,00E-04
0	0	0	0	0	3,00E-04	6,00E-04	7,00E-04	6,48E-05	0	0	0	0	0
0	0	0	0	0	0	0	0	0	0	0	0	0	0
0	0	0	0	0	8,97E-06	2,00E-04	0	0	0	0	0	0	0
0	0	0	0	0	0	0	0	0	0	0	0	0	0
0	0	0	0	0	0	0	0	0	0	0	0	0	0
0	0	0	0	0	6,68E-05	2,00E-04	0	3,23E-05	0	0	0	0	0
0	0	0	0	0	1,79E-05	0	0	0	0	0	0	0	0
0,0016	1,00E-04	0	0	0	9,09E-06	0	0	6,00E-04	0,0023	0	0	0	0
6,62E-05	0	0	0	4,64E-05	9,24E-06	2,00E-04	6,53E-05	6,46E-05	1,00E-04	0,0012	0	0	0
0	1,00E-04	0,0039	0	0	4,00E-04	4,00E-04	0	5,00E-04	0	0	0,0132	0	0
0,0042	5,96E-05	0	0	4,49E-05	2,69E-05	0	6,50E-05	0,001	0,0032	0,0012	0	0	0
0	0	0	0	0	9,01E-06	0	0	0	0	0	0	0	0
0	0	0	0	0	8,98E-06	0	3,25E-05	0	0	0	0	0	0
0	0	0	0	0	0	0	0	0	0	0	0	0	0
3,00E-04	0	0	0	5,00E-04	2,71E-05	4,00E-04	0	2,00E-04	3,00E-04	0	0	0	3,00E-04

0	0	0	0	0	0	0	0	0	0	0	0	0	0
0	0	0	1,00E-04	0	4,48E-05	2,00E-04	0	3,23E-05	1,00E-04	0	0	0	0
9,63E-05	0	0	0	0	3,96E-05	0	0	3,23E-05	0	0	0	0	0
7,00E-04	0,0011	1,00E-04	0	4,48E-05	0,001	0,0016	3,25E-05	7,00E-04	8,00E-04	0,0012	0	0	3,00E-04
0	0	0	2,00E-04	0	3,69E-05	0	0	1,00E-04	0	0,0013	0	6,00E-04	0
0	0	0	2,00E-04	0	3,79E-05	0	0	1,00E-04	0	0,0013	0	6,00E-04	0
0	2,98E-05	0	0	0	1,00E-04	0	3,25E-05	3,23E-05	0	0	0	0	0
0	0	0	3,00E-04	0	8,98E-06	2,00E-04	0	0	0	0	0	0	0
0	0	0	0	0	0	0	0	0	0	0	0	0	0
1,00E-04	2,00E-04	0	0	2,00E-04	3,00E-04	2,00E-04	8,04E-05	6,47E-05	1,00E-04	0	0	0	0
1,00E-04	6,11E-05	0	0	0	2,00E-04	0	0	9,96E-05	0	0	0	0	0
0	0	0	0	0	0	0	0	0	0	0	0	0	0
0	5,97E-05	0	5,80E-05	0	0	0	3,25E-05	0	0	0	0	0	0
0	0	0	0	0	0	2,00E-04	0	0	0	0	0	0	0
0	0	0	0	0	9,59E-06	0	3,38E-05	0	0	0	0	0	0
0	0	0	0	0	0	0	0	0	0	0	0	0	0
0	0	0	0	0	0	0	0	0	0	0	0	0	0
0	0	1,00E-04	0	2,00E-04	9,55E-05	0	0	3,23E-05	0	0	0	0	0
0	0	0	0	0	0	0	0	0	0	0	0	0	0
0	0	0	0	0	0	0	0	0,0056	1,00E-04	0	0	0	0,0203
1,00E-04	9,00E-04	2,00E-04	0	2,00E-04	0,0013	9,00E-04	3,00E-04	7,00E-04	0	0,0012	0	0	0
0	2,98E-05	0	0	0	0	0	0	0	0	0	0	0	0
0	0	0	0	0	0	0	0	0	0	0	0	0	0
0	0	0	0	0	5,37E-05	0	6,50E-05	3,23E-05	1,00E-04	0	0	0	0
0	0	0	0	0	0	0	0	0	0	0	0	0	0
0	0	0	0	0	0	0	0	0	0	0	0	0	0
0	0	0	0	0	0	0	0	0	0	0	0	0	0
1,00E-04	0	0	0,0013	0	2,70E-05	0	0	0	0	0	0	0	0
5,00E-04	8,94E-05	0	5,80E-05	0	8,96E-06	0	0	1,00E-04	5,00E-04	0	0	0	0
0	0	0	0	0	0	0	0	0	0	0	0	0	0
0	0	0	0	0	0	0	0	0	0	0	0	0	0
0	0	0	0	0	0	0	0	0	0	0	0	0	0
0	3,00E-05	0	0	0	5,38E-05	0	0	3,23E-05	1,00E-04	0	0	0	0
9,00E-04	6,01E-05	0	0	0	9,00E-06	0	0	4,00E-04	0,0014	0	0	0	0
0	0	0	0	0	1,80E-05	0	3,25E-05	0	0	0	0	0	0
0	2,99E-05	0	0	0	0	2,00E-04	6,53E-05	3,23E-05	1,00E-04	0	0	0	0

0	0	0	0	0	0	0	0	0	0	0	0	0	0
0	0	0	0	0	0	0	0	0	0	0	0	0	0
0	0	0	0	0	0	0	0	0	0	0	0	0	0
6,55E-05	5,96E-05	0	0	0,0045	3,00E-04	4,00E-04	6,50E-05	6,00E-04	0	0	0	0	0,0043
0	2,98E-05	0	0	0	8,96E-06	2,00E-04	0	0	0	0	0	0	0
0,0017	0	0	0	0	0	0	0	7,00E-04	0,0022	0,0012	0	0	0
0	3,00E-04	0	0	0	0	2,00E-04	0	0	0	0	0	0	0
0	2,98E-05	1,00E-04	2,00E-04	0	1,79E-05	0	0	0	0	0	0	0	0
0	0	0	0	0	0	0	0	0	0	0	0	0	0
0	0	0	0	0	0	0	0	0	0	0	0	0	0
0	0	0	1,00E-04	0	1,79E-05	0	5,00E-04	0	0	0	0	0	0
0	0	0	0	0	0	0	0	0	0	0	0	0	0
0	0	0	0	0	0	0	0	0	0	0	0	0	0
0	0	0	0	0	2,69E-05	0	0	0	0	0	0	0	0
2,00E-04	0	0	0	2,00E-04	7,00E-04	0	0	2,00E-04	0	0	0	0	3,00E-04
0	2,98E-05	0	0	0	0	0	0	0	0	0	0	0	0
0	0	0	0	0	0	0	0	0	0	0	0	0	0
0	0	0	0	0	0	0	0	0	0	0	0	0	0
0	2,00E-04	0	0	0	4,54E-05	4,00E-04	0	0	0	0	0	0	0
0	0	0	0	0	3,58E-05	0	0	0	0	0	0	0	0
0	0	0	0	0	0	0	0	0	0	0	0	0	0
0	0	0	0	0	0	0	0	0	0	0	0	0	0
0	2,98E-05	0	0	4,48E-05	1,79E-05	0	0	3,23E-05	0	0	0	0	0
0	3,01E-05	0	0	0	0	0	0,0014	0	0	0	0	0	0
3,00E-04	0	0	4,00E-04	0,0014	2,00E-04	4,00E-04	3,25E-05	4,00E-04	3,00E-04	0	0	0	0,002
6,54E-05	0	0	0	4,49E-05	1,79E-05	0	6,50E-05	0	0	0	0	0	0
0	0	0	0	0	0	0	0	0	0	0	0	0	0
0	0	0	0	0	0	0	0	0	0	0	0	0	0
1,00E-04	0	1,00E-04	1,00E-04	0	8,99E-06	0	6,50E-05	3,23E-05	1,00E-04	0	0	0	0
0	0	0	0	0	0	0	0	0	0	0	0	0	0
0	0	0	0	0	9,16E-06	0	0	0	0	0	0	0	0
0	0	0	0	0	8,96E-06	0	0	0	0	0	0	0	0
0	0	0	0	0	0	0	0	0	0	0	0	0	0
0	0	0	0	0	0	0	0	0	0	0	0	0	0
0	0	0	0	0	0	0	3,29E-05	0	0	0	0	0	0
0	0	0	0	0	0	0	0	0	0	0	0	0	0

0	0	0	0	0	0	0	0	0	0	0	0	0	0
0	0	0	0	7,00E-04	8,95E-06	0	6,50E-05	2,00E-04	0	0	0	0	0,0011
0	0	0	0	0	0	0	0	0	0	0	0	0	0
0	4,26E-05	0	0	0	0	0	0	0	0	0	0	0	0
0,0022	0	0	0	0	0	0	0	6,00E-04	0,0021	0	0	0	0
0	0	0	0	0	0	2,00E-04	0	0	0	0	0	0	0
0	0	0	0	0	0	0	0	0,0095	0,0327	0,006	0	0	0
6,65E-05	3,18E-05	0	0	0	0	0	0	6,46E-05	2,00E-04	0	0	0	0
2,00E-04	5,00E-04	0,0015	0,0019	0	1,00E-04	0,0016	3,00E-04	2,00E-04	1,00E-04	0,0012	0	6,00E-04	0
8,37E-05	1,00E-04	2,00E-04	0	4,00E-04	1,00E-04	0	4,00E-04	0	0	0	0	0	0
0	0	0	0	0	0	0	0	0	0	0	0	0	0
6,53E-05	2,98E-05	0	0	0	0	0	0	0	0	0	0	0	0
0	0	1,00E-04	0	0	0	0	0	0	0	0	0	0	0
0	0	0	0	0	0	0	0	0	0	0	0	0	0
0	1,00E-04	0	5,81E-05	0	9,95E-05	0	0	1,00E-04	0	0	0	0	0
0	0	0	0	0	0	0	3,26E-05	0	0	0	0	0	0
0	0	0	0	0	0	0	0	0	0	0	0	0	0
0	0	0	0	0	0	0	0	0	0	0	0	0	0
0	2,00E-04	0	0	0	1,85E-05	0	0	3,23E-05	0	0	0	0	0
0	0,0016	0,0022	0	0	7,00E-04	0,0015	4,00E-04	4,00E-04	0	0,0012	0,0033	0	0
0	0	0	0	0	0	0	0	0	0	0	0	0	0
0	0	0	0	0	0	0	0	0	0	0	0	0	0
0	1,00E-04	0	0	0	0	0	0	6,47E-05	0	0	0	0	0
0	0	0	0,0017	0	0	0	3,47E-05	0	0	0	0	0	0
0	0	0	0	0	0	0	0	0	0	0	0	0	0
0	3,00E-04	0,0019	0	0	4,00E-04	0,0014	0,0016	6,48E-05	0	0	0	0	0
0	3,00E-04	0	0	0	0	2,00E-04	9,77E-05	0	0	0	0	0	0
0	0	0	0	0	1,94E-05	0	0	0	0	0	0	0	0
0	0	0	0	0	9,02E-06	0	0	0	0	0	0	0	0
1,00E-04	3,00E-04	4,00E-04	0	8,98E-05	7,00E-04	4,00E-04	5,00E-04	3,00E-04	0	0,0012	0	0	0
0	1,00E-04	0	0	0	8,96E-06	0	2,00E-04	3,24E-05	0	0,0012	0	0	0
0	0	0	0	0	0	0	0	0	0	0	0	0	0
0	0	0	0	0	0	0	0	0	0	0	0	0	0
0	0	0	0	0	0	0	0	0	0	0	0	0	0
0	0	0	0	0	0	0	0	0	0	0	0	0	0
9,84E-05	0	0	0	0	0	0	0	0	0	0	0	0	0

0	0	0	0	0	0	0	9,80E-05	0	0	0	0	0	0
0	0	0	2,00E-04	0	0	0	0	0	0	0	0	0	0
0	0	0	0	0	0	0	0	0	0	0	0	0	0
6,54E-05	5,99E-05	2,00E-04	0	0	2,00E-04	2,00E-04	0,0015	6,46E-05	1,00E-04	0	0	0	0
0	0	0	1,00E-04	0	0	0	0	3,23E-05	0	0	0	6,00E-04	0
4,00E-04	2,99E-05	0	5,84E-05	0	0	2,00E-04	0	3,23E-05	0	0	0	0	0
0	0	0	0	0	0	0	0	0	0	0	0	0	0
0	0	0	0	0	8,96E-06	0	0	0	0	0	0	0	0
0	2,98E-05	0	0	0	1,79E-05	0	3,25E-05	3,23E-05	0	0	0	0	0
0	0	0	0	0	8,97E-06	0	0	0	0	0	0	0	0
0	0	0	0	0	0	0	0	0	0	0	0	0	0
0	0	0	0	0	2,78E-05	0	0	0	0	0	0	0	0
4,00E-04	2,98E-05	0	0	0	0	0	0	6,46E-05	2,00E-04	0	0	0	0
0	0	0	0	0	2,69E-05	0	2,00E-04	6,46E-05	0	0	0	0	0
0	0	0	0	0	1,11E-05	0	3,75E-05	0	0	0	0	0	0
0	3,00E-04	0	0	6,00E-04	1,00E-04	0	7,60E-05	6,48E-05	0	0,0012	0	0	3,00E-04
0	0	0	0	0	9,70E-06	0	0	0	0	0	0	0	0
0	0	0	0	0	0	0	0	0	0	0	0	0	0
0	7,00E-04	0	3,00E-04	0	1,83E-05	0	4,44E-05	3,46E-05	0	0,0012	0	0	0
9,00E-04	6,21E-05	0	0	0	0	0	0	3,00E-04	9,00E-04	0	0	0	0
0	0	0	0	0	1,28E-05	0	0	0	0	0	0	0	0
6,00E-04	3,18E-05	0	0	0	2,72E-05	0	3,41E-05	2,00E-04	7,00E-04	0	0	0	0
0	0	0	0	0	0	0	0	0	0	0	0	0	0
0	5,97E-05	0	5,00E-04	0	2,76E-05	0	6,51E-05	0	0	0	0	0	0
0	0	0	0	0	0	0	0	0	0	0	0	0	0
0	0	0	0	0	3,49E-05	0	0	9,69E-05	0	0	0	0	0
0	0	0	0	0	0	0	0	0	0	0	0	0	0
0	0	0	1,00E-04	0	1,79E-05	0	0	0	0	0	0	0	0
0,0084	8,93E-05	0	0	0	4,48E-05	4,00E-04	0	0,0022	0,0078	0,0012	0	0	0
0,0022	5,00E-04	0	1,00E-04	0	1,00E-04	5,00E-04	2,00E-04	7,00E-04	0,0024	0	0	0	0
0	3,00E-04	0	0	0	2,69E-05	0	0	0	0	0	0	0	0
0	0	0	0	0	0	0	0	0	0	0	0	0	0
0	0	0	0	0	0	0	0	0	0	0	0	0	0
6,55E-05	0	0	0	0	2,70E-05	0	0	0	0	0	0	0	0
0	0	0	0	0	0	0	0	0	0	0	0	0	0
0	0	0	0	0	4,72E-05	2,00E-04	0	3,23E-05	0	0	0	0	0

2,00E-04	2,98E-05	0	0	0	0	0	3,25E-05	9,69E-05	3,00E-04	0	0	0	0
0	0	0	0	0	0	0	0	0	0	0	0	0	0
6,53E-05	2,98E-05	0	0	0	1,00E-04	4,00E-04	2,00E-04	3,23E-05	0	0	0	0	0
0,0022	4,23E-05	0	0	0	0	0	0	0,001	0,0037	0	0	0	0
0,0069	3,00E-04	0	0	0	2,28E-05	0	9,15E-05	0,0019	0,0069	0	0	0	0
0,0037	5,96E-05	0	0	0	8,95E-06	0	0	9,00E-04	0,0033	0	0	0	0
0	0	0	0	0	0	0	0	0	0	0	0	0	0
0	0	0	5,80E-05	0	0	0	0	0	0	0	0	0	0
0	0	0	0	0	2,69E-05	0	0	0	0	0	0	0	0
0	0	0	0	0	0	0	0	0	0	0	0	0	0
0	0	0	0	0	8,97E-06	0	9,75E-05	0	0	0	0	0	0
6,53E-05	0	0	0	0	8,95E-06	0	0	0	0	0	0	0	0
5,00E-04	2,00E-04	0	5,80E-05	0	0	2,00E-04	0	1,00E-04	3,00E-04	0	0	0	0
0	7,00E-04	0	0	0	8,96E-06	4,00E-04	0	3,23E-05	0	0	0	0	0
5,00E-04	5,96E-05	0	0	0	0	2,00E-04	0	3,23E-05	1,00E-04	0	0	0	0
0	0	0	0	0	8,10E-05	0	0	6,46E-05	0	0	0	0	0
0	0	0	0	0	0	0	0	0	0	0	0	0	0
0	0	0	0	0	0	0	0	3,00E-04	0,001	0	0	0	0
0	0	0	0	0	0	2,00E-04	0	0	0	0	0	0	0
0	0	0	0	0	0	0	0	0	0	0	0	0	0
0	0	0	0	0	0	0	0	0	0	0	0	0	0
3,00E-04	8,00E-04	0,0034	0	0	3,00E-04	5,00E-04	0	3,00E-04	0	0,0012	0,0066	0	0
0	2,98E-05	0	0	0	0	0	0	0	0	0	0	0	0
0	0	0	0	0	0	0	0	0	0	0	0	0	0
0	5,96E-05	0	1,00E-04	0	6,28E-05	0	6,50E-05	3,24E-05	1,00E-04	0	0	0	0
0	0	0	9,68E-05	6,63E-05	3,51E-05	0	0	0	0	0	0	0	0
0	0	0	0	0	2,16E-05	3,00E-04	0	3,25E-05	0	0	0	0	0
0	0	0	0	0	5,44E-05	0	1,00E-04	0	0	0	0	0	0
0	0	0	0	0	0	0	0	0	0	0	0	0	0
0	0	0	0,003	0	0	0	3,59E-05	3,00E-04	0	0	0	0,0056	0
0	0	0	0	0	0	0	0	0	0	0	0	0	0
0	0	0	7,85E-05	0	1,42E-05	0	0	0	0	0	0	0	0
0	0	0	0	0	0	0	0	0	0	0	0	0	0
0	0	0	0	0	0	0	0	0	0	0	0	0	0
0	0	0	0	0	0	0	0	0	0	0	0	0	0
0	0	0	0	0	3,07E-05	0	0	0	0	0	0	0	0

0	0	0	0	0	0	0	0	0	0	0	0	0	0
6,54E-05	7,00E-04	0	0	0	7,00E-04	2,00E-04	9,75E-05	4,00E-04	1,00E-04	0,0012	0	0	0
0	0	0	0	0	0	0	0	0	0	0	0	0	0
0	2,98E-05	0	0	0	0	0	0	0	0	0	0	0	0
0	0	0	0	0	2,85E-05	0	3,36E-05	0	0	0	0	0	0
6,54E-05	0	0	0	0	8,95E-06	0	6,50E-05	0	0	0	0	0	0
0	0	0	0	0	3,58E-05	0	0	0	0	0	0	0	0
0	0	0	0	0	1,99E-05	0	0	0	0	0	0	0	0
0	0	0	0	0	3,00E-04	0	0	1,00E-04	1,00E-04	0	0	0	3,00E-04
0	0	0	0	0	0	0	0	0	0	0	0	0	0
0	0	0	0	0	9,02E-06	0	0	0	0	0	0	0	0
3,00E-04	1,00E-04	0,0015	0	0,0019	0,001	5,00E-04	0,001	9,00E-04	3,00E-04	0	0	0	0,0011
7,00E-04	0	0	0	0	0	0	0	2,00E-04	6,00E-04	0	0	0	0
0	0	0	0	0	4,57E-05	0	0	0	0	0	0	0	0
0	6,14E-05	0,0023	0	0	7,26E-05	6,00E-04	0	9,69E-05	0	0	0,0099	0	0
0	0	0	0	0	0	0	0	0	0	0	0	0	0
6,54E-05	0	0	5,80E-05	0	9,00E-06	0	0	0	0	0	0	0	0
0	0	0	0	0	2,69E-05	2,00E-04	0	3,23E-05	0	0	0	0	0
0	3,27E-05	0	0	0	0	0	0	0	0	0	0	0	0
0	0	0	0	0	0	0	0	0	0	0	0	0	0
0	0	0	0	0	0	0	0	0	0	0	0	0	0
7,00E-04	4,00E-04	0	0	0,0025	0,0031	0,0015	6,00E-04	0,002	5,00E-04	0	0	0	0,0017
0	0	0	0	0	9,02E-06	0	0	0	0	0	0	0	0
0	0	0	0	0	0	0	0	0	0	0	0	0	0
0,002	2,98E-05	0	0	0	1,79E-05	0	0	7,00E-04	0,0025	0	0	0	0
0	0	0	0	0	1,79E-05	0	0	0	0	0	0	0	0
0	0	0	6,21E-05	0	3,71E-05	0	0	0	0	0	0	0	0
0	2,99E-05	0	0	4,49E-05	8,96E-06	0	0	3,23E-05	0	0	0	0	0
0	0	0	0	0	0	0	0	0	0	0	0	0	0
0	0	0	0	0	0	0	0	0	0	0	0	0	0
0	0	0	0	0	0	0	0	0	0	0	0	0	0
0	3,04E-05	0	0	0	3,06E-05	0	0	3,24E-05	0	0	0	0	0
0	2,98E-05	0	0	0	0	0	0	0	0	0	0	0	0
6,54E-05	2,98E-05	0	5,80E-05	0	1,83E-05	0	3,25E-05	3,24E-05	0	0	0	0	0
0	1,00E-04	0,004	0,0019	0	1,00E-04	2,00E-04	3,25E-05	2,00E-04	0	0	0,0033	0,0025	0
0,0011	2,98E-05	1,00E-04	0	0	0	0	0	9,69E-05	3,00E-04	0	0	0	0

0	0	0	2,00E-04	0	0	0	0	0	0	0	0	0	0
0	0	0	0	0	0	0	0	0	0	0	0	0	0
0	0	0	0	0	0	0	0	0	0	0	0	0	0
0	0	0	0	0	0	0	0	3,34E-05	1,00E-04	0	0	0	0
6,54E-05	0	1,00E-04	0	0	0	0	0	0	0	0	0	0	0
0	3,11E-05	0	0	0	3,65E-05	0	0	3,23E-05	0	0	0	0	0
0	2,98E-05	0	0	0	9,85E-05	0	9,75E-05	6,46E-05	0	0	0	0	0
0	0	0	0	0	0	0	0	0	0	0	0	0	0
0	0	0	1,00E-04	0	0	0	3,25E-05	0	0	0	0	0	0
0	3,03E-05	0	0	0	2,70E-05	0	0	0	0	0	0	0	0
0	2,98E-05	0	0	0	1,79E-05	0	0	0	0	0	0	0	0
0	3,19E-05	0	0	0	0	0	0	0	0	0	0	0	0
0	0	0	0	0	0	0	0	0	0	0	0	0	0
0	0	0	0	0	0	0	0	0	0	0	0	0	0
0	0	0	0	0	0	0	0	0	0	0	0	0	0
0	0	0	0	0	0	0	0	0	0	0	0	0	0
0	0	0	0	0	0	0	0	0	0	0	0	0	0
6,53E-05	0	0	0	0	1,00E-04	4,00E-04	3,25E-05	1,00E-04	0	0	0	0	0
0	0	0	0	0	2,00E-04	0	3,25E-05	3,23E-05	0	0	0	0	0
0	2,98E-05	0	0	0	3,58E-05	0	0	0	0	0	0	0	0
0	0	0	1,00E-04	0	0	0	0	0	0	0	0	0	0
0	0	0	0	0	0	0	0	0	0	0	0	0	0
2,00E-04	4,00E-04	0	0	0	0,001	0	8,55E-05	4,00E-04	1,00E-04	0	0	0	0
6,57E-05	0	0	0	0	0	0	0	0	0	0	0	0	0
0	0	0	0	0	0	0	0	0	0	0	0	0	0
1,00E-04	0	0	0	0	0	0	0	0	0	0	0	0	0
0	0	0	0	0	0	0	0	0	0	0	0	0	0
0	0	0	0	0	0	0	0	0	0	0	0	0	0
0	0	1,00E-04	0	0	4,48E-05	0	0	9,69E-05	2,00E-04	0	0	0	0
0	0	0	0	0	0	0	0	0	0	0	0	0	0
0	0	0	5,80E-05	0	0	0	0	0	0	0	0	0	0
0	0	0	0	0	0	0	0	0	0	0	0	0	0
0	0	0	0	0	0	0	0	0	0	0	0	0	0
0	5,00E-04	0	0	0	0	2,00E-04	0	6,47E-05	0	0,0024	0	0	0
0	0	0	0	0	0	0	0	0	0	0	0	0	0
8,00E-04	3,02E-05	0	0	0	0	2,00E-04	0	2,00E-04	7,00E-04	0	0	0	0

0	0	0	0	0	0	0	0	0	0	0	0	0	0
0	0	6,00E-04	0	0	2,59E-05	0	0	0	0	0	0	0	0
0	0	0	0	0	0	0	0	0	0	0	0	0	0
0	5,96E-05	0	0	8,99E-05	2,00E-04	2,00E-04	0	0	0	0	0	0	0
0	0	0	0	0	0	0	0	0	0	0	0	0	0
0	3,74E-05	0	0	4,74E-05	4,00E-04	0	1,00E-04	2,00E-04	2,00E-04	0	0	6,00E-04	0
0	0	0	0	0	0	0	0	0	0	0	0	0	0
0	0	0	0	0	0	0	0	0	0	0	0	0	0
0	0	0	0	0	0	0	0	0	0	0	0	0	0
0	0	0	5,82E-05	0	9,03E-06	0	6,53E-05	0	0	0	0	0	0
0	0	0	0	0	0	0	0	0	0	0	0	0	0
0	0	0	0	0	0	0	0	0	0	0	0	0	0
6,53E-05	0	0	0	0	0	0	0	0	0	0	0	0	0
3,00E-04	0	0	0	0	0	0	0	6,46E-05	2,00E-04	0	0	0	0
6,53E-05	5,96E-05	0	0	0	4,00E-04	5,00E-04	6,50E-05	2,00E-04	1,00E-04	0	0	0	0
0	0	0	0	0	0	0	0	0	0	0	0	0	0
1,00E-04	0	0	0	0	0	0	0	3,23E-05	1,00E-04	0	0	0	0
0	0	0	0	4,00E-04	1,79E-05	0	0	6,46E-05	1,00E-04	0	0	0	0
0	2,98E-05	0	0	0	0	0	6,50E-05	0	0	0	0	0	0
0	2,98E-05	0	0	0	0	0	0	0	0	0	0	0	0
0	0	0	0	0	0	0	0	0	0	0	0	0	0
0	0	0	0	0	0	0	0	4,00E-04	0,0011	0,0024	0	0	0
0	0	0	0	0	0	0	3,25E-05	0	0	0	0	0	0
0	0	0	0	0	0	0	0	0	0	0	0	0	0
0	0	0	0	0	0	0	0	0	0	0	0	0	0
0	0	0	0	0	0	0	0	6,47E-05	2,00E-04	0	0	0	0
0	0	0	0	0	0	0	0	0	0	0	0	0	0
0	2,98E-05	0	0	0	0	0	0	0	0	0	0	0	0
0	0	0	0	0	0	0	0	0	0	0	0	0	0
0	0	0	0	0	0	0	0	3,51E-05	0	0	0	0	0
0	0	0	0	0	0	0	0	0	0	0	0	0	0
0	0	0	0	0	9,33E-06	0	0	0	0	0	0	0	0
0	0	0	0	0	0	0	0	0	0	0	0	0	0
0	0	0	0	0	0	0	0	0	0	0	0	0	0
6,55E-05	1,00E-04	2,00E-04	0	0	2,69E-05	0	0	3,24E-05	0	0	0	0	0
0	0	0	0	0	0	0	0	3,23E-05	1,00E-04	0	0	0	0

2,00E-04	3,01E-05	0	0	3,00E-04	9,00E-04	4,00E-04	0	2,00E-04	0	0	0	0	3,00E-04
0	0	0	0	0	0	0	0	0	0	0	0	0	0
0	0	0	0	0	0	0	0	0	0	0	0	0	0
0	3,00E-04	0	0	0	8,96E-06	0	3,25E-05	0	0	0	0	0	0
0	0	0	0	0	0	0	0	0	0	0	0	0	0
0	0	0	0	0	0	0	0	0	0	0	0	0	0
4,00E-04	4,00E-04	0	0	0,0014	0,0013	9,00E-04	3,00E-04	9,00E-04	2,00E-04	0	0	0	3,00E-04
4,00E-04	9,01E-05	0	0	0	0	0	0	1,00E-04	5,00E-04	0	0	0	0
1,00E-04	2,98E-05	0	0	4,00E-04	2,00E-04	0	0	2,00E-04	1,00E-04	0	0	0	3,00E-04
0	0	0	0	0	0	0	0	0	0	0	0	0	0
0	0	0	0	0	0	0	0	0	0	0	0	0	0
0	0	0	0	0	3,70E-05	2,00E-04	3,42E-05	0	0	0	0	0	0
1,00E-04	2,98E-05	0	0	0	0	0	0	9,70E-05	1,00E-04	0	0	0	0
0	8,94E-05	0,002	0	9,00E-04	0,0029	0,0018	0	0,0026	2,00E-04	0,0012	0	0	0,0014
0	0	0	0	0	8,96E-06	0	0	0	0	0	0	0	0
0	0	0	0,0019	0	0	0	0	9,69E-05	0	0	0	0,0018	0
0	0	0	0	0	0	0	0	0	0	0	0	0	0
0	0	0	0	0	0	0	0	0	0	0	0	0	0
6,54E-05	0	0	1,00E-04	0	8,95E-06	2,00E-04	2,00E-04	0	0	0	0	0	0
0	2,98E-05	0	0	0	0	0	0	0	0	0	0	0	0
0	5,99E-05	0	0	0	2,71E-05	0	0	0	0	0	0	0	0
0	4,00E-04	0,0016	0	0	3,43E-05	5,00E-04	4,38E-05	3,23E-05	0	0,0012	0	0	0
0	0	0	0	0	0	0	0	0	0	0	0	0	0
0	0	0	0	0	0	0	0	0	0	0	0	0	0
0	0	0	0	0	0	0	0	0	0	0	0	0	0
0	0	0	0	0	0	0	0	0	0	0	0	0	0
0	0	0	0	0	1,99E-05	0	0	3,24E-05	0	0	0	0	0
0	0	0	0	0	0	0	0	0	0	0	0	0	0
0	0	0	0	0	0	0	0	0	0	0	0	0	0
0	0	0	0	0	0	0	0	0	0	0	0	0	0
0	0	0	1,00E-04	0	0	0	0	0	0	0	0	0	0
0	0	0	0	0	0	0	0	0	0	0	0	0	0
0	0	0	0	0	9,19E-06	0	3,39E-05	0	0	0	0	0	0
0	0	0	0	0	9,01E-06	0	0	0	0	0	0	0	0
0	8,93E-05	0	0	0	2,00E-04	2,00E-04	3,25E-05	0	0	0	0	0	0
0	0	0	0	0	0	0	0	0	0	0	0	0	0

0	0	0	0	0	0	0	0	0	0	0	0	0	0
6,59E-05	3,08E-05	0	0	0	9,05E-06	2,00E-04	0	0	0	0	0	0	0
6,53E-05	2,00E-04	0	0	0	1,79E-05	0	0	0	0	0	0	0	0
0	0	0	0	0	0	0	0	2,00E-04	3,00E-04	0	0	0	0
0	2,98E-05	0	0	0	8,96E-06	0	3,25E-05	6,46E-05	1,00E-04	0	0	0	0
0	2,98E-05	0	5,80E-05	0	3,58E-05	0	6,50E-05	0	0	0	0	0	0
0	3,00E-04	1,00E-04	0,0021	0	4,58E-05	2,00E-04	6,50E-05	6,46E-05	0	0	0	0,0012	0
0	0	0	0	0	0	0	0	0	0	0	0	0	0
0	0	0	0	0	0	0	0	0	0	0	0	0	0
7,00E-04	5,96E-05	0	2,00E-04	0	8,97E-06	0	0	3,00E-04	8,00E-04	0	0	0	0
0	0	0	0	0	0	0	0	0	0	0	0	0	0
6,54E-05	0	0	0	0	5,44E-05	0	0	3,23E-05	0	0	0	0	0
0	4,94E-05	0	0	0	1,23E-05	0	0	0	0	0	0	0	0
0	0	0	0	0	0	0	0	0	0	0	0	0	0
0	0	0	0	0	9,00E-06	0	0	0	0	0	0	0	0
0	0	0	0	0	4,49E-05	2,00E-04	0	3,23E-05	0	0	0	0	0
1,00E-04	0	0	0	0	3,66E-05	0	0	3,23E-05	1,00E-04	0	0	0	0
0	5,96E-05	0	0	0	2,69E-05	0	0	0	0	0	0	0	0
0	0	0	0	0	8,96E-06	0	0	0	0	0	0	0	0
0	0	0	0	0	8,95E-06	0	0	0	0	0	0	0	0
0	2,98E-05	0	0	0	9,07E-06	0	0	0	0	0	0	0	0
0	0,0016	0	0	0	5,00E-04	6,00E-04	0	9,69E-05	2,00E-04	0	0	0	0
0	0	0	2,00E-04	0	0	0	0	0	0	0	0	0	0
0	0	0	0	0	0	0	0	0	0	0	0	0	0
0	3,00E-04	0	0	0	0	5,00E-04	0	0	0	0	0	0	0
1,00E-04	0	0	0	0	5,84E-05	0	0	0	0	0	0	0	0
0	0	0	0	0	0	0	0	0	0	0	0	0	0
6,53E-05	0	0	0	0	0	0	0	6,46E-05	0	0	0	0	0
0	0	0	0	0	0	0	0	0	0	0	0	0	0
0	0	0	0	0	0	0	0	0	0	0	0	0	0
0	0	0	0	0	0	0	0	3,23E-05	1,00E-04	0	0	0	0
6,79E-05	0	0	0	0	0	0	0	2,00E-04	6,00E-04	0	0	0	0
0	0	0	0	0	0	0	0	0	0	0	0	0	0
0	0	0	0	4,58E-05	9,08E-06	0	0	0	0	0	0	0	0
0	2,99E-05	0	0	0	0	0	0	0	0	0	0	0	0
0	0	0	0	0	0	0	0	0	0	0	0	0	0

0	0	0	0	0	0	0	0	0	0	0	0	0	0
0	0	0	0	0	0	0	0	0	0	0	0	0	0
0	5,96E-05	0	0	0	2,69E-05	2,00E-04	3,25E-05	3,23E-05	1,00E-04	0	0	0	0
0	0	0	0	0	0	0	0	0	0	0	0	0	0
0	0	0	0	0	0	0	0	0	0	0	0	0	0
0	3,18E-05	0	0	0	3,15E-05	0	1,00E-04	3,23E-05	1,00E-04	0	0	0	0
2,00E-04	0	0	0	0	9,34E-06	0	0	3,23E-05	1,00E-04	0	0	0	0
0	0	0	0	0	9,08E-06	0	0	6,46E-05	0	0	0	0	0
0	0	0	0	0	1,79E-05	2,00E-04	0	6,47E-05	0	0	0	0	0
0	0	0	0	4,49E-05	1,00E-04	0	3,25E-05	0	0	0	0	0	0
0	2,00E-04	0	0	0	1,80E-05	0	3,25E-05	3,24E-05	0	0	0	0	0
0	2,00E-04	0	5,80E-05	0	0	4,00E-04	0	0	0	0	0	0	0
0	0	0	0	0	0	0	0	0	0	0	0	0	0
7,00E-04	2,98E-05	0	0	0	2,69E-05	0	0	9,69E-05	2,00E-04	0,0012	0	0	0
0	0	0	0	0	0	0	0	3,33E-05	0	0	0	0	0
0	0	0	0	0	3,00E-04	2,00E-04	0	9,69E-05	0	0	0	0	0
3,00E-04	1,00E-04	0	0	0	0	0	0	3,00E-04	0,001	0	0	0	0
0	4,00E-04	0	0	0	2,00E-04	5,00E-04	0	9,74E-05	0	0,0012	0	0	0
0	0	0	0	0	0	0	0	0	0	0	0	0	0
0	0	0	0	0	0	0	0	0	0	0	0	0	0
0	0	0	0	0	0	0	0	0	0	0	0	0	0
0	0	0	0	0	0	0	3,25E-05	0	0	0	0	0	0
1,00E-04	0,0013	0	0	0	8,96E-05	5,00E-04	3,25E-05	3,23E-05	0	0,0012	0	0	0
1,00E-04	0	0	0	0	0	0	2,00E-04	9,69E-05	3,00E-04	0	0	0	0
5,00E-04	5,96E-05	0	0	0	0	0	0	1,00E-04	3,00E-04	0,0012	0	0	0
0	4,01E-05	0	0	0	0	0	0	0	0	0	0	0	0
0	0	0	0	0	0	0	0	3,23E-05	1,00E-04	0	0	0	0
1,00E-04	8,93E-05	0,0017	0	4,49E-05	6,27E-05	5,00E-04	0	4,00E-04	1,00E-04	0	0	0	0
0	3,00E-05	0	0	0	0	0	3,26E-05	0	0	0	0	0	0
0	0	0	0	0	0	0	0	0	0	0	0	0	0
0	0	0	0	2,00E-04	3,00E-04	0	0	9,69E-05	0	0	0	0	0
0	0	0	0	0	3,58E-05	0	3,25E-05	0	0	0	0	0	0
0	0	0	0	0	0	0	0	0	0	0	0	0	0
0	0	0	0	0	0	0	0	0	0	0	0	0	0
0	0	0	0	0	0	0	0	0	0	0	0	0	0
0	0	0	0	0	9,31E-06	0	0	0	0	0	0	0	0

3,00E-04	9,00E-04	0,0011	5,83E-05	2,00E-04	0,0018	0,002	6,00E-04	6,00E-04	3,00E-04	0,0012	0	0	0
0	0	0	0	0	1,80E-05	0	0	3,23E-05	0	0	0	0	0
0	0	0	0	0	0	0	0	0	0	0	0	0	0
6,00E-04	1,00E-04	0	0	0	0	0	0	4,00E-04	0,0014	0	0	0	0
0	0	0	5,80E-05	0	8,95E-06	0	6,50E-05	0	0	0	0	0	0
0	0	0	0	0	0	0	0	0	0	0	0	0	0
0	0	0	0	0	0	0	0	0	0	0	0	0	0
0	0	0	0	0	0	0	0	0	0	0	0	0	0
0	5,00E-04	0	0	0	4,00E-04	0	0	2,00E-04	0	0	0	0	0
0	5,98E-05	0	1,00E-04	0	8,07E-05	0	0	3,23E-05	1,00E-04	0	0	0	0
0	0	0	0	0	0	0	0	0	0	0	0	0	0
0	5,96E-05	0	0	0	0	0	0	0	0	0	0	0	0
6,71E-05	0	0	6,15E-05	0	0	0	0	0	0	0	0	0	0
0	0	0	0	0	0	0	0	0	0	0	0	0	0
0	0	0	0	0	9,63E-06	0	0	3,24E-05	0	0	0	0	0
0	0	0	0	0	9,36E-06	0	0	0	0	0	0	0	0
0	0	1,00E-04	0	0	0	0	0	0	0	0	0	0	0
0,0065	6,00E-04	0	0	0	1,82E-05	0	6,60E-05	0,002	0,0071	0	0	0	0
0	3,32E-05	0	0	0	0	0	0	0	0	0	0	0	0
0	0	0	0	0	0	0	0	0	0	0	0	0	0
0	0	0	0	0	9,05E-06	0	0	0	0	0	0	0	0
0	0	0	0	0	0	0	0	0	0	0	0	0	0
0	0	0	0	0	0	0	0	0	0	0	0	0	0
0	0	0	0	0	0	0	0	0	0	0	0	0	0
0	0	0	0	0	0	0	0	0	0	0	0	0	0
0	0	0	0	0	0	0	0	0	0	0	0	0	0
0	1,00E-04	0	0	4,48E-05	2,00E-04	0	0	2,00E-04	1,00E-04	0	0	0	0
0	0	0	0	0	8,95E-06	0	0	0	0	0	0	0	0
0	0	0	0	0	1,80E-05	0	0	0	0	0	0	0	0
0	4,00E-04	0	5,80E-05	0	0	0	6,50E-05	3,23E-05	0	0	0	0	0
0	0	0	0	0	0	0	0	0	0	0	0	0	0
9,00E-04	2,98E-05	0	0	8,98E-05	0	2,00E-04	0	2,00E-04	7,00E-04	0	0	0	0
0	0	0	0	0	0	0	0	0	0	0	0	0	0
0	0	0	0	0	0	0	0	0	0	0	0	0	0
0	0	0	0	0	9,22E-06	0	0	0	0	0	0	0	0
0	0	0	0	0	0	2,00E-04	0	0	0	0	0	0	0

0	0	0	0	0	0	0	0	0	0	0	0	0	0
0	0	0	3,00E-04	0	5,11E-05	0	0	0	0	0	0	0	0
1,00E-04	2,98E-05	0	0	0	9,00E-06	0	0	6,46E-05	2,00E-04	0	0	0	0
0	0	0	0	0	0	0	0	0	0	0	0	0	0
0	0	0	0	0	0	0	0	0	0	0	0	0	0
3,00E-04	5,96E-05	0	5,82E-05	0	2,69E-05	0	0	6,46E-05	2,00E-04	0	0	0	0
0	0	0	5,80E-05	0	8,98E-06	0	0	0	0	0	0	0	0
1,00E-04	2,99E-05	0	0	0	6,32E-05	0	0	3,23E-05	1,00E-04	0	0	0	0
0	0	0	0	0	0	0	0	0	0	0	0	0	0
0	3,22E-05	0	0	0	0	0	0	0	0	0	0	0	0
0	0	0	0	0	0	0	0	0	0	0	0	0	0
0	0	0	0	0	0	0	0	0	0	0	0	0	0
0	0	0	0	0	1,79E-05	0	0	3,34E-05	1,00E-04	0	0	0	0
0	0	0	0	0	0	0	0	0	0	0	0	0	0
0	2,98E-05	0	0	0	0	0	0	0	0	0	0	0	0
0	0	0	0	0	4,04E-05	0	0	0	0	0	0	0	0
0	0	0	0	0	0	0	0	0	0	0	0	0	0
0	0	0	0	0	1,82E-05	0	0	0	0	0	0	0	0
0	0	0	0	0	9,08E-06	0	6,53E-05	6,47E-05	0	0	0	0	0
0	0	0	0	0	0	0	0	0	0	0	0	0	0
0	4,95E-05	0	0	0	3,58E-05	0	0	3,23E-05	0	0	0	0	0
0	0	0	0	0	0	0	3,26E-05	0	0	0	0	0	0
0	0	0	0	0	0	0	0	0	0	0	0	0	0
0	0	0	0	0	0	0	0	4,00E-04	0,0015	0	0	0	0
2,00E-04	0	0	0	0	0	0	0	6,46E-05	2,00E-04	0	0	0	0
0	0	0	0	0	2,15E-05	0	0	0	0	0	0	0	0
0	0	0	0	0	0	0	0	0	0	0	0	0	0
0	0	0	0	0	0	0	0	0	0	0	0	0	0
0	0	0	0	6,68E-05	3,53E-05	0	0	3,23E-05	0	0	0	0	0
0	0	0	0	0	1,00E-04	0	0	3,23E-05	0	0	0	0	0
0	0	0	0	0	0	0	0	0	0	0	0	0	0
0	0	0	5,86E-05	0	3,61E-05	0	0	0	0	0	0	0	0
0	0	0	0	0	0	0	0	0	0	0	0	0	0
1,00E-04	0	0	0	0	8,98E-06	0	9,75E-05	0	0	0	0	0	0
0	2,98E-05	0	0	0	0	0	0	0	0	0	0	0	0
0	0	0	0	0	0	0	0	0	0	0	0	0	0

0,0051	2,00E-04	0	0	5,03E-05	2,55E-05	2,00E-04	0	0,0016	0,0056	0	0	0	0
0	0	0	0	0	0	0	0	0	0	0	0	0	0
0	0	0	0	0	8,98E-06	0	3,25E-05	0	0	0	0	0	0
0	0	0	0	0	0	0	0	0	0	0	0	0	0
0	6,00E-04	7,00E-04	0	2,00E-04	1,00E-04	9,00E-04	0	2,00E-04	0	0	0	0	6,00E-04
0	1,00E-04	0	9,00E-04	4,53E-05	1,00E-04	0	6,00E-04	6,46E-05	0	0	0	0,0012	0
0	3,10E-05	0	0	0	6,34E-05	0	0	3,23E-05	0	0	0	0	0
0	0	0	0	0	8,95E-06	0	0	0	0	0	0	0	0
8,00E-04	0	0	0	0	0	0	0	1,00E-04	5,00E-04	0	0	0	0
0	0	0	0	0	0	0	0	0	0	0	0	0	0
0	0	0	0	0	0	0	0	0	0	0	0	0	0
0	5,96E-05	0	0,0038	0	0	0	6,50E-05	2,00E-04	0	0	0	0,0037	0
1,00E-04	3,00E-04	0	2,00E-04	0	1,00E-04	0	9,79E-05	6,46E-05	1,00E-04	0	0	0	0
0	0	0	0	0	0	0	0	0	0	0	0	0	0
0	7,02E-05	0	0	0	1,09E-05	0	0	3,23E-05	0	0,0012	0	0	0
5,00E-04	7,00E-04	0	0	0	6,00E-04	0,0013	0,0055	3,00E-04	0	0	0	0	0
2,00E-04	8,00E-04	0	0	4,00E-04	0,001	0,0016	2,00E-04	7,00E-04	0	0	0	0	6,00E-04
2,00E-04	0	0	0	0	0	0	0	0	0	0	0	0	0
0	0	0	0	0	2,70E-05	0	0	0	0	0	0	0	0
0	3,00E-05	0	0	0	0	0	0	0	0	0	0	0	0
0	0	1,00E-04	0	0	1,79E-05	0	0	0	0	0	0	0	0
0	4,00E-04	2,00E-04	0	0	8,99E-06	4,00E-04	0	6,46E-05	0	0,0024	0	0	0
0	0	0	0	0	0	0	0	0	0	0	0	0	0
0	0	0	0	0	4,50E-05	0	0	0	0	0	0	0	0
0	0	0	0	0	0	0	0	0	0	0	0	0	0
0	0	0	0	0	0	0	0	0	0	0	0	0	0
0	0	0	0	0	0	0	0	0	0	0	0	0	0
0	0	0	0	0	0	0	0	0	0	0	0	0	0
0	0	0	0	0	0	0	0	0	0	0	0	0	0
0	0	0	0	0	0	0	0	0	0	0	0	0	0
0	0	0	0	0	0	0	0	0	0	0	0	0	0
0	0	0	0	0	0	0	0	0	0	0	0	0	0
0	0	0	0	0	0	0	0	0	0	0	0	0	0
0	2,98E-05	0	0	0	0	0	0	0	0	0	0	0	0
0	0	0	0	0	0	0	0	0	0	0	0	0	0
3,00E-04	5,00E-04	0	0	0	5,77E-05	8,00E-04	0	6,48E-05	1,00E-04	0	0	0	0

1,00E-04	2,98E-05	0	0	0	1,79E-05	2,00E-04	4,00E-04	0	0	0	0	0	0
0	0	0	0	0	0	0	0	0	0	0	0	0	0
0	0	0	0	0	0	0	0	0	0	0	0	0	0
0	0	0	0	0	0	0	0	0	0	0	0	0	0
0	3,11E-05	0	5,96E-05	0	1,81E-05	0	9,97E-05	0	0	0	0	0	0
0	0	0	0	0	0	0	0	0	0	0	0	0	0
0	0	0	0	0	0	0	0	0	0	0	0	0	0
0	0	0	0	0	0	0	0	0	0	0	0	0	0
0	0	0	0	0	0	0	0	0	0	0	0	0	0
0	8,93E-05	0	5,80E-05	0	0	0	0	0	0	0	0	0	0
0	0	0	0	0	0	0	0	3,44E-05	0	0	0	0	0
0	0	0	0	0	0	0	0	0	0	0	0	0	0
0	0	0	0	0	2,49E-05	0	0	0	0	0	0	0	0
0	0	0	0	6,00E-05	0	0	0	0	0	0	0	0	0
0	2,98E-05	0	0	4,49E-05	0	0	0	0	0	0	0	0	0
0	2,00E-04	0	0	0	0	0	0	0	0	0	0	0	0
0	0	0	0	0	0	0	0	0	0	0	0	0	0
7,24E-05	3,38E-05	0	5,00E-04	0	8,52E-05	0	3,00E-04	1,00E-04	0	0	0	0,0025	0
9,00E-04	4,00E-04	0	0	8,00E-04	0,0021	6,00E-04	3,27E-05	0,0017	0,001	0,0012	0	0	0,0014
6,90E-05	4,00E-04	0	0	0	5,78E-05	2,00E-04	0	6,47E-05	0	0,0024	0	0	0
0	0	0	0	0	3,81E-05	0	0	0	0	0	0	0	0
0	0	0	0	0	0	0	0	0	0	0	0	0	0
0	0	0	0	0	0	0	0	0	0	0	0	0	0
0	0	0	0	0	0	0	0	0	0	0	0	0	0
0	0	0	0,0014	0	0	0	0	0	0	0	0	0	0
0	0	0	5,00E-04	0	1,79E-05	2,00E-04	6,50E-05	0	0	0	0	0	0
0	1,00E-04	0	0	5,00E-04	2,00E-04	0	0	3,00E-04	1,00E-04	0,0012	0	0	3,00E-04
0	2,98E-05	0	0	8,97E-05	5,00E-04	0	7,00E-04	4,00E-04	1,00E-04	0	0	0	9,00E-04
0	0	0	5,80E-05	0	0	0	3,25E-05	0	0	0	0	0	0
0	0	0	0	0	0	0	0	0	0	0	0	0	0
0	0	0	0	0	0	0	0	0	0	0	0	0	0
0	0	0	0	0	0	0	0	0	0	0	0	0	0
0	0	0	0	0	0	0	0	0,002	0,007	0	0	0	0
0	0	0	0	0	0	0	0	0	0	0	0	0	0
0	2,98E-05	0	0	0	0	0	3,25E-05	0	0	0	0	0	0
0	0	0	0	0	0	0	0	0	0	0	0	0	0

6,64E-05	0	0	0	0	0	0	0	3,23E-05	1,00E-04	0	0	0	0
0	6,15E-05	0	5,93E-05	0	1,81E-05	0	0	0	0	0	0	0	0
0	0	0	0	0	1,88E-05	0	3,51E-05	0	0	0	0	0	0
6,54E-05	2,98E-05	0	0	0	0	0	0	6,46E-05	0	0	0	0	0
6,54E-05	4,00E-04	1,00E-04	0	0	4,00E-04	2,00E-04	7,00E-04	1,00E-04	0	0	0	0	0
0	0	0	0	0	0	0	0	0	0	0	0	0	0
0,0027	2,98E-05	0	0	0	8,95E-06	0	0	8,00E-04	0,0027	0	0	0	0
0	0	0	2,00E-04	0	0	2,00E-04	0	0	0	0	0	0	0
0,0034	9,30E-05	0	0	0	0	0	0	9,00E-04	0,0033	0	0	0	0
1,00E-04	2,00E-04	0	0	0,0017	2,00E-04	0,0013	0	4,00E-04	1,00E-04	0,0024	0	0	0,0014
0	0	0	0	0	9,12E-06	0	3,31E-05	0	0	0	0	0	0
0	0	0	0	0	1,00E-04	0	0	0	0	0	0	0	0
0	0	0	0	0	0	0	0	0	0	0	0	0	0
0	0	0	2,00E-04	4,49E-05	0	0	0	0	0	0	0	0	0
0	8,36E-05	0	0	0	0	0	0	0	0	0	0	0	0
0,0014	3,11E-05	0	0	0	0	0	0	4,00E-04	0,0014	0	0	0	0
0	0	0	0	0	0	0	0	0	0	0	0	0	0
0	0	0	0	0	0	0	0	0	0	0	0	0	0
6,55E-05	0	0	0	0	0	0	0	0	0	0	0	0	0
0	0	0	0	0	0	0	0	0	0	0	0	0	0
0	0	0	0	0	0	0	0	0	0	0	0	0	0
0	2,99E-05	0	0	0	0	0	0	0	0	0	0	0	0
6,60E-05	2,99E-05	0	0	0	5,42E-05	0	0	3,23E-05	0	0	0	0	0
0	0	0	0	0	8,96E-06	0	0	0	0	0	0	0	0
0	0	0	0	0	0	0	0	0	0	0	0	0	0
6,54E-05	1,00E-04	0	5,80E-05	0	4,00E-04	0	0	3,00E-04	2,00E-04	0,0012	0	0	0
0	0	0	6,00E-04	0	0	2,00E-04	0	6,46E-05	0	0	0	6,00E-04	0
0	0	0	0	0	0	0	0	0	0	0	0	0	0
0	0	0	0	0	0	0	0	0	0	0	0	0	0
0	3,46E-05	0	0	0	0	0	0	0	0	0	0	0	0
0	0	0	0	0	2,00E-04	0	3,25E-05	3,23E-05	0	0	0	0	0
0	0	0	0	0	0	0	0	0	0	0	0	0	0
0	0	0	0	0	0	0	0	0	0	0	0	0	0
0	0	0	0	0	0	0	0	0	0	0	0	0	0
0	0	0	0	0	0	0	0	0	0	0	0	0	0
0	0	0	0	0	5,39E-05	0	0	0	0	0	0	0	0

0	0	0	0	0	3,58E-05	0	0	0	0	0	0	0	0
6,54E-05	0	0	0	0	0	0	0	3,23E-05	1,00E-04	0	0	0	0
0	0	0	0	0	0	0	0	3,00E-04	1,00E-04	0	0	0	0
2,00E-04	5,00E-04	0,0017	0	8,97E-05	0,0011	0	6,50E-05	5,00E-04	1,00E-04	0	0	0	0
0	0	0	0	0	0	0	0	0	0	0	0	0	0
0	0	0	0	0	5,38E-05	0	0	0	0	0	0	0	0
0	0	0	0	0	1,79E-05	0	0	0	0	0	0	0	0
6,56E-05	0	0	0	0	0	2,00E-04	3,27E-05	3,23E-05	1,00E-04	0	0	0	0
0	2,98E-05	0	0	0	1,79E-05	2,00E-04	0	0	0	0	0	0	0
1,00E-04	5,00E-04	0	0	6,56E-05	1,00E-04	5,00E-04	0	6,46E-05	0	0,0012	0	0	0
0	0	0	0	0	9,65E-06	0	0	0	0	0	0	0	0
0	0	0	0	0	0	0	0	0	0	0	0	0	0
0	0	0	0	4,49E-05	8,98E-06	0	3,25E-05	0	0	0	0	0	0
0	5,96E-05	0	0	0	8,95E-06	0	6,50E-05	3,23E-05	0	0	0	0	0
0	0	0	0	0	0	0	0	0	0	0	0	0	0
0	0	0	0	0	0	0	0	0	0	0	0	0	0
0,0012	8,99E-05	0	8,00E-04	0	8,13E-05	4,00E-04	0,0019	5,00E-04	0,0016	0	0	0	0
0	2,00E-04	8,00E-04	0	0	1,00E-04	0	0,0011	3,23E-05	0	0	0,0033	0	0
0	0	0	0	0	8,96E-06	0	0	0	0	0	0	0	0
0	0	0	0	0	0	0	0	0	0	0	0	0	0
0	0	0	0	0	0	0	0	0	0	0	0	0	0
0	0	0	0	0	0	0	0	0	0	0	0	0	0
0	0	0	5,82E-05	9,00E-05	3,62E-05	0	9,81E-05	0	0	0	0	0	0
0	0	0	0	0	0	0	0	0	0	0	0	0	0
0	0	0	0	0	0	0	0	2,00E-04	1,00E-04	0	0	0,0025	0
0	3,72E-05	0	6,47E-05	0	9,69E-06	0	0	0	0	0	0	0	0
0	0	0	0	0	0	0	0	0	0	0	0	0	0
0	0	0	0	0	0	0	0	0	0	0	0	0	0
0	8,94E-05	0	0	0	8,98E-05	0	0	3,23E-05	0	0	0	0	0
0	2,98E-05	0	5,80E-05	8,97E-05	2,00E-04	0	6,50E-05	3,23E-05	0	0	0	0	0
6,53E-05	0	0	0	0	0	0	0	0	0	0	0	0	0
0	0	0	0	0	0	0	0	0	0	0	0	0	0
0	0	0	0	0	0	0	0	0	0	0	0	0	0
0	0	0	0	0	0	0	0	0	0	0	0	0	0
0,0011	0	0	0	0	0	0	0	4,00E-04	0,0013	0	0	0	0
0	0	0	0	0	0	0	0	0	0	0	0	0	0

0	0	0	0	0	0	0	0	0	0	0	0	0	0
0	0	0	0	0	1,79E-05	0	0	3,23E-05	0	0	0	0	0
0	0	0	0	0	0	0	0	0	0	0	0	0	0
0	0	1,00E-04	0	0	3,51E-05	0	0	0	0	0	0	0	0
0	3,82E-05	1,00E-04	0	0	0	0	0	3,23E-05	0	0	0	0	0
0	0	0	0	0	0	0	0	0	0	0	0	0	0
0	0	0	0,0015	0	0	0	1,00E-04	6,47E-05	0	0	0	0,0012	0
0	8,93E-05	0	0	0	0	4,00E-04	0	3,24E-05	0	0	0	0	0
0	0	0	0	0	0	0	0	0	0	0	0	0	0
0	0	0	0	0	0	0	0	0	0	0	0	0	0
0	0	0	0	0	2,00E-04	0	6,50E-05	3,23E-05	0	0	0	0	0
0	0	0	0	0	0	0	0	0	0	0	0	0	0
0	0	0	0	0	0	0	0	9,69E-05	3,00E-04	0	0	0	0
0	3,00E-04	0	0	0	1,80E-05	0	0	3,23E-05	0	0,0012	0	0	0
6,61E-05	0	0	0	0	4,54E-05	0	0	0	0	0	0	0	0
0	2,98E-05	0	0	0	0	0	0	0	0	0	0	0	0
0	0	0	0	0	0	0	0	0	0	0	0	0	0
3,00E-04	0	0	0	0	0	0	3,25E-05	2,00E-04	8,00E-04	0	0	0	0
0	0	0	0	0	0	0	0	0	0	0	0	0	0
6,53E-05	1,00E-04	0,0062	0	0	3,00E-04	9,00E-04	6,50E-05	3,00E-04	2,00E-04	0	0,0066	0	0
0	5,98E-05	0	0	0	0	2,00E-04	0	0	0	0	0	0	0
6,54E-05	0	0	0	0	3,59E-05	7,00E-04	0,0086	3,23E-05	0	0	0	0	0
0	2,98E-05	0	0	4,57E-05	2,69E-05	0	1,00E-04	6,47E-05	0	0	0	0	0
0	2,98E-05	0	0	0	0	0	0	0	0	0	0	0	0
0	4,00E-04	0	0	0	8,95E-06	0	0	3,23E-05	0	0,0012	0	0	0
0	0	0	0	0	1,05E-05	0	0	0	0	0	0	0	0
6,53E-05	2,98E-05	0	0	0	7,16E-05	2,00E-04	2,00E-04	9,69E-05	0	0	0	0	0
3,00E-04	0	0	0	0	0	0	0	9,69E-05	3,00E-04	0	0	0	0
8,00E-04	0	1,00E-04	0	0	4,48E-05	2,00E-04	0	2,00E-04	5,00E-04	0	0	0	0
3,00E-04	2,00E-04	0	0	1,00E-04	9,00E-04	0,0011	0	4,00E-04	3,00E-04	0	0	0	0
0	1,00E-04	0	0	0	2,69E-05	0	0	3,23E-05	0	0	0	0	0
0	0	0	0	0	0	0	0	0	0	0	0	0	0
0	0	0	0	0	0	0	0	0	0	0	0	0	0
0	0	0	0	0	0	0	0	0	0	0	0	0	0
0	2,98E-05	0	0	0	8,96E-06	0	0	0	0	0	0	0	0
0	0	0	5,81E-05	0	3,62E-05	0	3,25E-05	0	0	0	0	0	0

1,00E-04	4,00E-04	0	0	0	5,00E-04	2,00E-04	7,00E-04	9,70E-05	0	0	0	0	3,00E-04
8,00E-04	5,00E-04	0	0,0013	2,00E-04	9,00E-04	0,001	0,0026	0,001	0,0018	0,0012	0	0,0019	0
0	0	0	0	0	3,34E-05	0	0	0	0	0	0	0	0
0	0	0	0	0	0	0	3,25E-05	0	0	0	0	0	0
0	1,00E-04	0	0	0	0	0	0	0	0	0	0	0	0
0	2,98E-05	0	5,80E-05	0	2,69E-05	0	0	0	0	0	0	0	0
0,0023	0	0	0	0	0	2,00E-04	0	9,00E-04	0,0033	0	0	0	0
0	3,00E-05	0	2,00E-04	0	1,88E-05	2,00E-04	3,27E-05	3,24E-05	0	0	0	0	0
0	0	0	0	0	0	0	0	0	0	0	0	0	0
0	0	0	0	0	0	0	0	0	0	0	0	0	0
0	0	0	0	0	0	0	0	0	0	0	0	0	0
0,0038	2,00E-04	0	0	0	5,39E-05	2,00E-04	0	0,0011	0,0039	0	0	0	0
0	0	0	0	0	0	0	0	0	0	0	0	0	0
7,97E-05	0	0	0	0	3,44E-05	0	0	3,24E-05	0	0	0	0	0
0	0	0	0	0	0	0	0	6,46E-05	0	0	0	0	0
0	0	0	0	0	0	0	0	0	0	0	0	0	0
0	0	0	0	0	8,99E-06	0	0	0	0	0	0	0	0
0	0	0	0	0	0	0	0	0	0	0	0	0	0
0	0	0	0	0	0	0	0	0	0	0	0	0	0
0	0	0	0	0	9,30E-06	0	0	0	0	0	0	0	0
0	0	0	0	0	0	0	0	0	0	0	0	0	0
0	0	0	0	0	0	0	0	0	0	0	0	0	0
6,53E-05	0,001	0,0014	0	4,49E-05	0,001	0,0015	2,00E-04	5,00E-04	1,00E-04	0	0	0	0
5,00E-04	8,93E-05	0	0	0,0029	0,0031	0,002	0	0,0016	6,00E-04	0,0012	0	0	0,0029
0	0	0	0	0	0	0	0	6,47E-05	1,00E-04	0	0	0	0
0	8,94E-05	0	0	0	1,80E-05	0	9,75E-05	3,23E-05	0	0	0	0	0
0	4,23E-05	1,00E-04	0	0	4,47E-05	0	0	0	0	0	0	0	0
1,00E-04	6,00E-04	4,00E-04	1,00E-04	0,0033	0,0016	0,0013	6,00E-04	0,0011	2,00E-04	0	0	0	0,0023
0	3,11E-05	0	0	0	2,00E-04	0	0	3,23E-05	1,00E-04	0	0	0	0
0	0	0	0	0	0	0	0	0	0	0	0	0	0
0	0	0	0	0	3,62E-05	0	0	6,46E-05	0	0	0	0	0
0	0	0	0	0	1,37E-05	0	0	0	0	0	0	0	0
0	8,93E-05	0	0	0	3,00E-04	0	0	6,46E-05	0	0	0	0	0
0	0	0	0	0	0	0	0	0	0	0	0	0	0
0	0	0	0	0	0	0	0	0	0	0	0	0	0
0	3,00E-04	0	0	0	1,00E-04	4,00E-04	3,25E-05	6,46E-05	0	0,0012	0	0	0

0	0	0	0	0	0	0	0	0	0	0	0	0	0
0	0	0	0	0	0	0	0	0	0	0	0	0	0
0	0	0	0	0	0	0	0	0	0	0	0	0	0
6,53E-05	0	0	0	0	0	0	0	6,46E-05	2,00E-04	0	0	0	0
0	2,00E-04	0	0	4,49E-05	8,95E-05	4,00E-04	0	6,47E-05	0	0	0	0	0
0	0	0	0	0	0	0	0	0	0	0	0	0	0
0	0	0	0	0	0	0	0	0	0	0	0	0	0
0	0	0	5,80E-05	4,49E-05	2,00E-04	0	6,50E-05	5,00E-04	0	0	0	0	0
6,00E-04	2,98E-05	0	0	0	0	0	0	3,00E-04	9,00E-04	0	0	0	0
0	0	0	8,00E-04	0	0	0	0	6,49E-05	2,00E-04	0	0	0	0
0	0	0	0	0	8,96E-06	0	0	0	0	0	0	0	0
0	2,98E-05	0	0	0	3,59E-05	0	0	3,23E-05	0	0	0	0	0
6,56E-05	2,00E-04	0	0	0	1,83E-05	2,00E-04	0	0	0	0	0	0	0
0	1,00E-04	0	0	4,49E-05	0	0	0	0	0	0	0	0	0
0	0	0	0	0	0	0	0	0	0	0	0	0	0
5,00E-04	2,00E-04	0	0	0	0	0	0	9,71E-05	3,00E-04	0	0	0	0
0	0	0	0	0	0	0	0	0	0	0	0	0	0
9,00E-04	0	0	0	0	8,96E-06	0	0	3,00E-04	0,001	0	0	0	0
0	0	0	0	0	0	0	0	0	0	0	0	0	0
0	0	0	0	0	0	0	0	0	0	0	0	0	0
0	0	0	1,00E-04	0	0	0	0	3,23E-05	0	0	0	6,00E-04	0
2,00E-04	2,00E-04	0	0	0,0019	0,0021	7,00E-04	2,00E-04	0,0011	2,00E-04	0,0012	0	0	9,00E-04
0	0	0	0	0	0	0	0	0	0	0	0	0	0
0	0	0	0	0	0	0	0	0	0	0	0	0	0
3,00E-04	1,00E-04	0	0	0	0	0	3,25E-05	6,46E-05	1,00E-04	0	0	0	0
0	0	0	0	0	0	0	0	0	0	0	0	0	0
0	0	0	0	0	0	0	0	0	0	0	0	0	0
0	0	0	0	0	0	0	0	0	0	0	0	0	0
0	0	0	0	0	0	0	0	3,23E-05	0	0	0	0	0
0	0	0	5,80E-05	0	8,97E-06	0	0	0	0	0	0	0	0
0	0	0	0	0	0	0	0	0	0	0	0	0	0
0	0	0	0	0	0	0	0	0	0	0	0	0	0
0	5,96E-05	0	0	0	0	2,00E-04	0	0	0	0	0	0	0
0	0	0	0	0	8,99E-06	0	0	0	0	0	0	0	0
1,00E-04	0	0	0	0	0	0	0	3,27E-05	0	0	0	0	0
0	0	0	5,80E-05	0	0	0	0	0	0	0	0	0	0

0	0	0	0,0063	4,50E-05	1,80E-05	2,00E-04	3,00E-04	5,00E-04	0	0	0	0,0093	0
0	0	0	0	0	0	0	0	0	0	0	0	0	0
0	0	0	0	0	0	0	0	0	0	0	0	0	0
1,00E-04	5,96E-05	0	0	0	0	0	3,25E-05	0	0	0	0	0	0
0	4,14E-05	0	0	0	0	0	0	3,23E-05	1,00E-04	0	0	0	0
6,56E-05	0	0	0	0	0	0	3,25E-05	3,24E-05	0	0	0	0	0
0	0	0	0	0	0	0	0	0	0	0	0	0	0
0	0	0	0	0	8,95E-06	0	3,25E-05	0	0	0	0	0	0
0	0	0	0	0	0	0	0	0	0	0	0	0	0
0	2,00E-04	0	0	0	8,06E-05	0	0	0	0	0	0	0	0
0	0	0	0	0	0	0	0	0	0	0	0	0	0
0	0	0	0	0	0	0	0	0	0	0	0	0	0
0	1,00E-04	0	0	0	2,91E-05	0	8,44E-05	3,25E-05	0	0	0	0	3,00E-04
0	0	0	0	0	4,60E-05	0	3,76E-05	0	0	0	0	0	0
0	0	0	0	0	0	0	0	0	0	0	0	0	0
0,0027	8,94E-05	0	0	0	2,76E-05	2,00E-04	3,25E-05	5,00E-04	0,0016	0	0	0	0
0	0	0	0	0	0	0	0	0	0	0	0	0	0
0	0	0	0	0	1,80E-05	2,00E-04	3,27E-05	0	0	0	0	0	0
0	0	0	0	0	0	0	0	0	0	0	0	0	0
6,54E-05	5,00E-04	0	0	0	1,80E-05	9,00E-04	0	6,46E-05	0	0	0	0	0
0	0	0	0	0	0	0	3,25E-05	0	0	0	0	0	0
0	0	0	0	0	0	0	0	0	0	0	0	0	0
0	2,98E-05	0	0	0	2,69E-05	5,00E-04	0,0019	0	0	0	0	0	0
0,0012	6,08E-05	0	0	0	0	0	3,34E-05	3,00E-04	0,0012	0	0	0	0
1,00E-04	7,00E-04	0	0	0	8,00E-04	9,00E-04	0	6,00E-04	1,00E-04	0	0	0	3,00E-04
0	1,00E-04	0	0	0	2,73E-05	0	3,25E-05	3,23E-05	0	0	0	0	0
0	0	0	0	0	0	0	0	0	0	0	0	0	0
1,00E-04	0	0	0	0	1,00E-04	0	1,00E-04	3,23E-05	1,00E-04	0	0	0	0
0	0	0	0	0	0	0	0	0	0	0	0	0	0
0	2,98E-05	1,00E-04	5,80E-05	0,0044	5,00E-04	7,00E-04	6,00E-04	0,0015	0	0	0	0	0,0043
3,00E-04	6,08E-05	0	0	0	9,41E-06	0	0	1,00E-04	3,00E-04	0,0012	0	0	0
0,0042	0	0	0	0	0	2,00E-04	0	0,001	0,0036	0	0	0	0
0	0	0	0	0	9,00E-06	0	3,26E-05	9,71E-05	2,00E-04	0	0	0	0
0	2,98E-05	0	0	0	1,79E-05	0	3,25E-05	0	0	0	0	0	0
0	0	0	0	0	0	0	0	0	0	0	0	0	0
0	0	0	0	0	0	0	0	0	0	0	0	0	0

0	0	0	0	0	0	0	0	0	0	0	0	0	0
0	0	0	0	0	8,97E-06	0	0	0	0	0	0	0	0
0	0	0	0	0	0	0	0	0	0	0	0	0	0
0	0	0	1,00E-04	0	1,00E-04	0	0,0026	3,23E-05	1,00E-04	0	0	0	0
0	0	0	0	0	0	0	0	0	0	0	0	0	0
0	0	0	0	0	0	0	0	0	0	0	0	0	0
5,00E-04	9,00E-04	0,006	5,80E-05	9,00E-04	0,0027	0,0016	3,00E-04	0,0021	3,00E-04	0,0012	0	0	9,00E-04
5,00E-04	0	0	0	0	8,96E-06	0	6,50E-05	9,69E-05	3,00E-04	0	0	0	0
0	0	0	0	0	0	0	0	0	0	0	0	0	0
1,00E-04	0	0	0	0	0	0	0	3,23E-05	1,00E-04	0	0	0	0
0	2,98E-05	0	0	0	2,00E-04	2,00E-04	2,00E-04	3,23E-05	0	0	0	0	0
0	0	0	0	0	0	0	0	0	0	0	0	0	0
0	0	0	0	0	0	0	0	3,26E-05	0	0	0	0	0
0	0	0	0	0	4,50E-05	0,0013	0,0018	3,24E-05	0	0	0	0	0
1,00E-04	2,00E-04	0	0,0039	0	8,21E-05	0	6,52E-05	2,00E-04	0	0	0	0,0025	0
6,54E-05	0	0	3,00E-04	0	1,82E-05	0	9,86E-05	3,23E-05	0	0	0	0	0
0	0	0	0	0	0	0	0	9,42E-05	0	0	0	0	0
0	0	0	0	0	8,97E-06	2,00E-04	0	0	0	0	0	0	0
2,00E-04	9,00E-04	1,00E-04	0	5,00E-04	0,0026	0,0024	3,00E-04	0,0012	5,00E-04	0,0012	0,0033	0	6,00E-04
0	0	0	7,10E-05	0	0	0	0	3,24E-05	1,00E-04	0	0	0	0
7,00E-04	2,00E-04	0	0	0,0011	0,0031	0,0018	0	0,002	7,00E-04	0	0	0	0,0023
0	0	0	0	0	2,00E-04	0	0	9,73E-05	0	0	0	0	0
0	0	0	0	0	0	0	0	0	0	0	0	0	0
0	1,00E-04	0	0	0	3,00E-04	0	6,50E-05	2,00E-04	0	0,0012	0	0	0
2,00E-04	0	0	0	0	0	0	0	6,46E-05	2,00E-04	0	0	0	0
0	0	0	0	0	0	0	0	0	0	0	0	0	0
0	0	0	0	0	8,99E-06	0	0	0	0	0	0	0	0
0	0	0	0	0	0	0	0	0	0	0	0	0	0
0	0	0	0	0	8,96E-06	0	0	0	0	0	0	0	0
0	1,00E-04	1,00E-04	0	0,0019	5,00E-04	5,00E-04	6,50E-05	5,00E-04	0	0	0	0	0,0011
1,00E-04	0	0	0	4,48E-05	0,0013	2,00E-04	0	5,00E-04	1,00E-04	0	0	0	0
6,69E-05	3,27E-05	0	0,0025	0	0	2,00E-04	0	2,00E-04	1,00E-04	0	0	0,0025	0
3,00E-04	6,02E-05	0	0	0	0	0	0	2,00E-04	7,00E-04	0	0	0	0
0	0	0	0	0	2,15E-05	0	0	0	0	0	0	0	0
0	6,10E-05	0	0	0	9,09E-06	0	0	0	0	0	0	0	0
0	0	0	0	0	0	0	0	0	0	0	0	0	0

0	0	0	0	0	0	0	0	0,0037	0,0128	0,0012	0	0	0
4,00E-04	0	0	0	0	9,04E-05	0	0	3,24E-05	0	0	0	0	0
0	0	0	5,80E-05	1,00E-04	1,82E-05	0	3,25E-05	6,47E-05	1,00E-04	0	0	0	0
0	2,98E-05	0	5,80E-05	0	8,98E-06	0	0	0	0	0	0	0	0
0	0	0	0	0	0	0	0	0	0	0	0	0	0
0	1,00E-04	0	0	0	0	2,00E-04	9,75E-05	3,23E-05	1,00E-04	0	0	0	0
0	0	0	0	0	4,48E-05	0	0	3,23E-05	1,00E-04	0	0	0	0
0	0	0	0	0	0	0	0	0	0	0	0	0	0
0	1,00E-04	0,0015	0	0	2,00E-04	0	3,00E-04	9,70E-05	0	0	0	0	0
0	0	0	0	0	2,71E-05	0	0	0	0	0	0	0	0
0	0	0	0	0	0	0	6,50E-05	0	0	0	0	0	0
0	0	0	0	0	0	0	0	0	0	0	0	0	0
0	0	1,00E-04	0	0	0	0	0	6,54E-05	0	0	0	0	0
0	0	0	0	0	0	0	0	0	0	0	0	0	0
0	0	0	0	0	3,62E-05	0	0	0	0	0	0	0	0
0	3,01E-05	0	0	0	0	0	0	0	0	0	0	0	0
0	0	0	0	0	0	0	0	0	0	0	0	0	0
0	0	0	0	0	0	0	0	3,23E-05	0	0	0	0	0
0	0	0	0	0	0	0	0	0	0	0	0	0	0
1,00E-04	0	0	0	0	0	0	0	0	0	0	0	0	0
0	5,96E-05	0	0	0	0	0	0	0	0	0	0	0	0
0	0	0	0	0	0	0	0	0	0	0	0	0	0
0	0	0	0	4,49E-05	0	0	0	0	0	0	0	0	0
0	5,97E-05	0	0	0	1,81E-05	0	0	3,23E-05	0	0	0	0	0
0	6,00E-04	0	0	0	5,60E-05	0	0	6,47E-05	0	0,0012	0	0	0
0	0	0	0	0	0	0	0	0	0	0	0	0	0
0	0	0	0	0	0	0	0	0	0	0	0	0	0
0	0	0	0	0	0	0	0	0	0	0	0	0	0
0,0061	1,00E-04	0	0	0	0	6,00E-04	4,97E-05	0,001	0,0037	0	0	0	0
0	0	0	0	0	0	0	0	0	0	0	0	0	0
0	0	0	0	0	0	0	0	0	0	0	0	0	0
0	0	0	0	0	0	0	0	0	0	0	0	0	0
0	0	0	0	0	0	0	0	0,0092	0,0321	0	0	0	0
0	0	0	0	4,68E-05	8,97E-06	0	0	0	0	0	0	0	0
0	0	0	0,0028	0	3,58E-05	4,00E-04	3,25E-05	3,23E-05	0	0	0	6,00E-04	0
0	0	0	0	0	0	0	0	0	0	0	0	0	0

0	0	0	0	0	0	0	0	0	0	0	0	0	0
0	0	0	5,97E-05	0	9,79E-06	0	3,39E-05	0	0	0	0	0	0
0	0	0	0	0	0	0	0	0	0	0	0	0	0
0	0	0	0	0	2,70E-05	0	0	3,23E-05	0	0	0	0	0
0	0	0	0	0	0	0	0	3,24E-05	1,00E-04	0	0	0	0
0	0	0	0	0	1,79E-05	0	0	0	0	0	0	0	0
0	0	0	0	0	0	0	0	0	0	0	0	0	0
0	0	0	0	0	0	0	0	0	0	0	0	0	0
3,00E-04	0	0	0	0	1,80E-05	0	0	1,00E-04	5,00E-04	0	0	0	0
1,00E-04	5,96E-05	0	0	0	0	0	3,25E-05	0	0	0	0	0	0
0	0	0	0	0	6,33E-05	0	0	3,23E-05	0	0	0	0	0
0	0	0	0	0	1,79E-05	0	0	0	0	0	0	0	0
0	0	0	0	0	3,00E-04	3,00E-04	0	3,23E-05	0	0	0	0	0
0	0	0	0	0	0	0	0	0	0	0	0	0	0
0	0	0	0	0	0	0	0	0	0	0	0	0	0
0	0	0	0	0	0	0	0	0	0	0	0	0	0
0	0	0	0	0	0	0	0	0	0	0	0	0	0
0	0	0	0	0	0	0	0	0	0	0	0	0	0
0,0016	3,00E-04	0	5,80E-05	0,0048	7,00E-04	0,0013	3,25E-05	0,0014	9,00E-04	0	0	0	0,0043
0	0	0	0	0	0	0	0	0	0	0	0	0	0
1,00E-04	5,96E-05	0	0	0	2,00E-04	2,00E-04	0	1,00E-04	0	0	0	0	0
2,00E-04	2,00E-04	6,00E-04	0	9,11E-05	9,00E-04	6,00E-04	8,00E-04	7,00E-04	2,00E-04	0	0	0	0
5,00E-04	1,00E-04	0	0	0	5,88E-05	3,00E-04	0	2,00E-04	6,00E-04	0,0012	0	0	0
0	0	0	0	0	0	0	0	0	0	0	0	0	0
0	0	0	0,0047	0	0	0	0	5,00E-04	0	0	0	0,0093	0
0,0029	2,00E-04	0	0	0	0	0	0	0,001	0,0036	0	0	0	0
0	5,96E-05	0	0	0	3,00E-04	4,00E-04	3,00E-04	9,68E-05	1,00E-04	0	0	0	0
0	0	0	0	0	0	0	0	0	0	0	0	0	0
0	0	0	0	0	0	0	0	0	0	0	0	0	0
0	0	0	0	4,49E-05	8,98E-06	0	0	0	0	0	0	0	0
0	0	0	0	0	0	0	0	0	0	0	0	0	0
6,71E-05	0	0	0	0	9,10E-06	0	0	0	0	0	0	0	0
0	0	0	0	0	0	0	0	0	0	0	0	0	0
0	0	0	0	0	8,96E-06	0	0	0	0	0	0	0	0
0	0	0	0	0	0	0	0	3,00E-04	9,00E-04	0	0	0	0
0	0	0	0	0	0	0	0	0	0	0	0	0	0

0	0	0	0	0	0	0	0	0	0	0	0	0	0
0	2,98E-05	0	0	0	8,95E-06	0	0	0	0	0	0	0	0
0	0	0	0	0	0	0	0	0	0	0	0	0	0
0	0	0	0	0	0	0	0	0	0	0	0	0	0
0	3,00E-04	0,0013	0	0	3,00E-04	3,00E-04	0	2,00E-04	1,00E-04	0	0,0033	0	0
0,0026	5,96E-05	0	0	0	0	0	0	8,00E-04	0,0028	0	0	0	0
0	3,14E-05	0	0	0	0	0	0	0	0	0	0	0	0
0	0	0	0	0	0	0	0	0	0	0	0	0	0
0	0	0	0	0	0	0	0	0	0	0	0	0	0
0	0	0	0	0	2,71E-05	0	0	0	0	0	0	0	0
0	0	0	0	0	0	0	0	0	0	0	0	0	0
0	0	0	0	0	0	0	0	0	0	0	0	0	0
0	0	0	0	0	0	0	0	0	0	0	0	0	0
0	0	0	0	0	0	0	0	0	0	0	0	0	0
0,0023	2,00E-04	0	0	0	0	0	0	5,00E-04	0,0019	0	0	0	0
0	0	0	0	0	0	0	0	0	0	0	0	0	0
0	0	0	0	0	0	0	0	0	0	0	0	0	0
0	0	0	0	0	8,95E-06	0	0	3,23E-05	0	0	0	0	0
0	0	0	0	0	0	0	0	0	0	0	0	0	0
0	0	0	0	0	0	0	0	0	0	0	0	0	0
0	0	0	0	0	0	0	0	0	0	0	0	0	0
3,00E-04	0	0	0	2,00E-04	3,48E-05	0	0	5,00E-04	6,00E-04	0	0	0	3,00E-04
0	0	0	0	0	2,76E-05	0	3,25E-05	3,24E-05	0	0	0	0	0
0	8,95E-05	0	0	0	4,49E-05	0	0	0	0	0	0	0	0
0	1,00E-04	0	0	0	0	0	0	3,23E-05	0	0,0012	0	0	0
0	0	0	0	0	0	0	0	0	0	0	0	0	0
0	0	0	0	0	0	0	0	0	0	0	0	0	0
0	0	0	0	0	1,80E-05	0	0	0	0	0	0	0	0
0	0	0	0	0	0	0	0	0	0	0	0	0	0
0,0011	2,98E-05	0	2,00E-04	0	8,95E-06	0	3,25E-05	7,00E-04	0,0023	0	0	6,00E-04	0
0	0	0	0	0	2,00E-04	0	0	9,71E-05	1,00E-04	0	0	0	0
0	0	0	0	0	0	0	0	0	0	0	0	0	0
6,54E-05	2,98E-05	0	0	0	3,59E-05	0	0	6,46E-05	1,00E-04	0	0	0	0
1,00E-04	0	0	0	0	1,83E-05	0	0	0	0	0	0	0	0
9,15E-05	6,12E-05	0	0	0	1,42E-05	0	0	0	0	0	0	0	0
0	0	0	0	0	0	0	0	0	0	0	0	0	0

0	0	0	0	0	0	0	0	0	0	0	0	0	0
4,00E-04	7,00E-04	0,0037	0	0,0013	0,0045	0,0018	0,0013	0,002	5,00E-04	0	0,0066	0	6,00E-04
0	0	0	0	0	0	0	0	0	0	0	0	0	0
0	5,96E-05	0	2,00E-04	0	0	0	3,25E-05	0	0	0	0	0	0
5,00E-04	2,98E-05	0	0	0	0	0	0	0	0	0	0	0	0
0	0	0	5,80E-05	0	8,95E-06	0	0	0	0	0	0	0	0
0	0	0	0	0	0	0	0	0	0	0	0	0	0
0	0	0	5,80E-05	0	0	0	0	0	0	0	0	0	0
0	0	0	0	0	0	0	0	0	0	0	0	0	0
0	0	0	0	0	0	0	0	0	0	0	0	0	0
6,53E-05	5,97E-05	0,0011	0	0,0027	8,00E-04	9,00E-04	0	0,0011	0	0,0012	0	0	0,0032
0,0016	4,00E-04	0,0076	0	0	8,95E-05	7,00E-04	0	5,00E-04	0,0017	0,0012	0,0033	0	0
3,00E-04	2,98E-05	0	0	0	0	0	0	6,46E-05	2,00E-04	0	0	0	0
0	0	0	0	0	0	0	0	0	0	0	0	0	0
0	0	0	0	0	0	0	3,25E-05	0	0	0	0	0	0
0	2,98E-05	0	0	0	8,96E-06	2,00E-04	9,75E-05	0	0	0	0	0	0
0	0	0	0	0	0	0	0	0	0	0	0	0	0
0	0	0	0	0	0	0	0	3,24E-05	0	0	0	0	0
0	0	0	0	0	0	0	0	0	0	0	0	0	0
0	0	0	0	0	0	0	0	0	0	0	0	0	0
0	0	0	0	0	0	0	0	0	0	0	0	0	0
1,00E-04	0	0	0	0	9,47E-06	0	0	6,46E-05	2,00E-04	0	0	0	0
0	3,58E-05	0	0,0052	0	0	0	0	4,00E-04	0	0	0	0,0068	0
0	0	0	0	0	0	0	0	0	0	0	0	0	0
0	0	0	0	0	0	0	0	2,00E-04	6,00E-04	0	0	0	0
0	2,00E-04	0	2,00E-04	0	2,69E-05	5,00E-04	0	3,23E-05	0	0	0	6,00E-04	0
6,56E-05	2,98E-05	0	0	2,00E-04	2,00E-04	5,00E-04	0	2,00E-04	0	0,0012	0	0	0,0014
0	1,00E-04	0	0	0	8,95E-06	0	0	3,23E-05	0	0	0	0	0
0	0	0	0	0	0	0	0	0	0	0	0	0	0
0	0	0	5,80E-05	0	8,96E-06	0	0	0	0	0	0	0	0
0	0	0	0	0	2,19E-05	0	0	6,47E-05	0	0	0	0	0
0	0	0	0	0	0	0	0	0	0	0	0	0	0
0	0	0	2,00E-04	0	0	0	0	0	0	0	0	0	0
0	0	0	0	0	0	0	0	0	0	0	0	0	0
0	0	0	0	0	0	0	0	0	0	0	0	0	0
0	0	0	0	0	8,97E-06	0	0	3,23E-05	0	0	0	0	0

1,00E-04	2,00E-04	1,00E-04	5,80E-05	0	5,00E-04	2,00E-04	0	2,00E-04	1,00E-04	0	0	0	0
2,00E-04	2,98E-05	0	0	0	0	0	0	0	0	0	0	0	0
0	8,93E-05	0	0	0	0	2,00E-04	2,00E-04	0	0	0	0	0	0
0	5,00E-04	0	0	0	2,00E-04	4,00E-04	0	0	0	0	0	0	0
0	2,98E-05	0	0	0	0	0	0	3,23E-05	0	0	0	0	0
0	0	0	0	0	1,80E-05	0	0	0	0	0	0	0	0
0	7,06E-05	0	0	0	2,00E-04	2,00E-04	1,00E-04	2,00E-04	0	0	0	0	0
0	0	0	0	0	8,95E-06	0	0	0	0	0	0	0	0
0	0	0	0	0	0	0	0	0	0	0	0	0	0
0	0	0	5,82E-05	0	3,00E-04	2,00E-04	0	2,00E-04	2,00E-04	0	0	0	0
0	0	0	0	0	0	0	0	0	0	0	0	0	0
0	8,93E-05	0	5,80E-05	0	0	0	0	0	0	0	0	0	0
0	0	0	0	0	3,33E-05	0	0	0	0	0	0	0	0
0	0	0	0	0	0	0	0	0	0	0	0	0	0
1,00E-04	5,96E-05	3,00E-04	0	0	8,12E-05	4,00E-04	5,00E-04	3,23E-05	0	0	0	0	0
0	0	0	0	0	0	0	0	0	0	0	0	0	0
0	2,00E-04	0	0	0	3,04E-05	0	0	0	0	0	0	0	0
0	0	0	0	0	0	0	0	0	0	0	0	0	0
0	0	0	0	0	0	0	0	0	0	0	0	0	0
0	0	0	0	0	0	0	0	0	0	0	0	0	0
0	0	0	0	0	1,28E-05	0	0	0	0	0	0	0	0
0	0	0	0	0	0	0	0	0	0	0	0	0	0
0	0	0	0	0	0	0	0	0	0	0	0	0	0
0	0	0	0	0	2,69E-05	0	0	0	0	0	0	0	0
0	0	0	0	0	0	0	0	0	0	0	0	0	0
0	0	0	0	0	0	0	0	0	0	0	0	0	0
0	0	0	0	0	0	0	0	0	0	0	0	0	0
0	0	0	0	0	0	0	0	0	0	0	0	0	0
6,54E-05	0	0	0	0	0	0	0	0	0	0	0	0	0
0	0	0	0	0	0	0	0	0	0	0	0	0	0
0	0	0	0	0	0	0	0	0	0	0	0	0	0
0	0	0	0	0	0	0	0	0	0	0	0	0	0
0	0	0	0	0	0	0	0	0	0	0	0	0	0
5,00E-04	0	0	0	0	0	0	0	3,23E-05	1,00E-04	0	0	0	0
0	0	0	0	0	0	0	0	0	0	0	0	0	0
0	1,00E-04	0	0	0	0	0	0	9,70E-05	0	0,0024	0	0	0

0	2,98E-05	0	0	0	7,17E-05	0	9,75E-05	3,23E-05	0	0	0	0	0
0	2,98E-05	0	0	0	8,96E-06	0	0	0	0	0	0	0	0
0	8,93E-05	0	0	0	2,00E-04	4,00E-04	0	6,50E-05	0	0	0	0	0
0	2,98E-05	0	5,80E-05	0	8,98E-06	2,00E-04	0	0	0	0	0	0	0
0	0	0	0	0	0	0	0	0	0	0	0	0	0
0	0	0	0	0	0	0	0	0	0	0	0	0	0
0	0	0	0	0	7,33E-05	2,00E-04	0	3,24E-05	0	0,0012	0	0	0
0	2,00E-04	0,0014	0	0,0015	9,00E-04	0,001	0	0,0011	2,00E-04	0	0	0	0,002
0	0	0	0	0	2,69E-05	0	3,25E-05	3,23E-05	1,00E-04	0	0	0	0
0	1,00E-04	1,00E-04	0	0	5,56E-05	2,00E-04	0	3,24E-05	0	0,0012	0	0	0
0	0	0	0	0	9,06E-06	0	0	0	0	0	0	0	0
0	0	0	0	0	0	0	0	0	0	0	0	0	0
0	0	0	0	0	0	0	0	0	0	0	0	0	0
0	6,20E-05	0	0	0	0	0	0	0	0	0	0	0	0
0	0	0	0	0	8,95E-06	0	0	0	0	0	0	0	0
0	0	0	0	0	9,03E-06	0	0	0	0	0	0	0	0
0	5,96E-05	0	0	0	7,18E-05	0	0	3,24E-05	0	0	0	0	0
0	0	0	0	0	0	0	0	0	0	0	0	0	0
0	2,98E-05	0	0	0	0	0	0	0	0	0	0	0	0
5,00E-04	2,98E-05	0	0	0	0	0	3,25E-05	9,69E-05	3,00E-04	0	0	0	0
0	0	0	0	0	0	0	0	0	0	0	0	0	0
0	3,00E-05	0	2,00E-04	0	0	0	0	0	0	0	0	0	0
0	0	0	0	0	0	0	0	0	0	0	0	0	0
0	0	0	0	0	0	0	0	0	0	0	0	0	0
0	0	0	0	0	0	0	0	0	0	0	0	0	0
4,00E-04	0	0	0	0	0	0	0	3,00E-04	0,001	0	0	0	0
0,0013	2,98E-05	0	0	0	0	0	0	2,00E-04	8,00E-04	0	0	0	0
0	2,98E-05	0	0	0	2,69E-05	0	0	3,23E-05	0	0	0	0	0
0	0	0	0	0	0	0	0	0	0	0	0	0	0
0	0	0	0	0	0	0	0	0	0	0	0	0	0
0	2,99E-05	0	0	0	8,96E-06	0	0	0	0	0	0	0	0
0,0025	2,98E-05	0	0	0	8,96E-06	0	0	6,00E-04	0,0019	0	0	0	0
0	1,00E-04	0	2,00E-04	0	8,99E-05	4,00E-04	0,001	6,46E-05	1,00E-04	0	0	0	0
0	2,00E-04	0	0	0	0	0	6,50E-05	0	0	0	0	0	0
0	0	0	0	0	8,96E-06	2,00E-04	0	0	0	0	0	0	0
0	0	0	0	0	0	0	0	6,47E-05	2,00E-04	0	0	0	0

0	0	0	0	0	1,97E-05	0	0	0	0	0	0	0	0
0	0	0	0	0	1,27E-05	2,00E-04	0	0	0	0	0	0	0
1,00E-04	5,00E-04	0	0	0	2,00E-04	7,00E-04	0	1,00E-04	1,00E-04	0	0	0	0
0	0	0	0	0	8,95E-06	0	0	0	0	0	0	0	0
8,57E-05	0	0	0	0	7,44E-05	0	0	3,25E-05	0	0	0	6,00E-04	0
0	2,98E-05	0	0	0	2,00E-04	2,00E-04	3,25E-05	3,23E-05	0	0	0	0	0
0	0	0	0	0	0	0	0	0	0	0	0	0	0
0,0018	3,11E-05	0	6,17E-05	0	5,82E-05	0	8,00E-04	0,0013	0,0043	0	0	0	0
0	0	0	0	0	0	0	0	6,46E-05	0	0	0	0,0012	0
0	0	0	0	0	0	0	0	0	0	0	0	0	0
0	0	0	0	0	0	0	0	0	0	0	0	0	0
0	0	0	0	0	0	0	0	0	0	0	0	0	0
0	0	0	0	0	0	2,00E-04	0	0	0	0	0	0	0
0	0	0	0	0	0	0	0	0	0	0	0	0	0
0	0	0	0	0	4,48E-05	0	3,25E-05	3,23E-05	0	0	0	0	0
0	1,00E-04	0	0	0	1,79E-05	0	0	3,23E-05	0	0,0012	0	0	0
6,53E-05	0	0	5,80E-05	0	6,29E-05	0	0	3,23E-05	1,00E-04	0	0	0	0
0	0	0	0	0	9,03E-06	0	0	0	0	0	0	0	0
0	0	0	0	0	0	0	0	0	0	0	0	0	0
0	0	0	0	0	0	0	0	0	0	0	0	0	0
0	0	0	0	0	0	0	0	0	0	0	0	0	0
0	0	0	0	0	0	0	0	0	0	0	0	0	0
0	0	0	1,00E-04	0	0	0	0	0	0	0	0	0	0
0	0	0	0	0	0	0	0	0	0	0	0	0	0
0	8,99E-05	0	0	0	3,59E-05	0	0	0	0	0	0	0	0
0	0	0	0	0	0	0	0	0	0	0	0	0	0
0	0	0	0	0	1,79E-05	2,00E-04	0	0	0	0	0	0	0
0	0	0	0	0	0	0	0	0	0	0	0	0	0
1,00E-04	4,20E-05	0	0,0014	0	5,70E-05	0	2,00E-04	3,23E-05	0	0	0	6,00E-04	0
0	0	0	0	0	9,05E-06	0	0	0	0	0	0	0	0
1,00E-04	3,00E-04	1,00E-04	0	0,0011	0,0013	6,00E-04	0	3,00E-04	0	0	0	0	0,0014
0	0	0	0	0	0	0	0	0	0	0	0	0	0
6,54E-05	0	0	5,80E-05	0	2,69E-05	0	6,52E-05	3,23E-05	1,00E-04	0	0	0	0
0	0	1,00E-04	0	0	0	0	0	0	0	0	0	0	0
1,00E-04	0	0	0	0	0	0	0	0	0	0	0	0	0
0	4,00E-04	0	1,00E-04	8,97E-05	1,00E-04	0	2,00E-04	9,69E-05	0	0,0012	0	0	0

0	0	0	0	0	0	0	0	2,00E-04	5,00E-04	0,0016	0	0	0
0	2,98E-05	0	0	0	2,69E-05	0	3,25E-05	0	0	0	0	0	0
0	0	0	0	0	0	0	0	0	0	0	0	0	0
0	0	0	0	0	0	0	0	0	0	0	0	0	0
0	0	0	0	0	0	0	0	0	0	0	0	0	0
0	0	0	0	0	0	0	0	0	0	0	0	0	0
0	1,00E-04	0	0	0	0	0	6,57E-05	0	0	0	0	0	0
0	0	0	0	0	8,96E-06	0	0	0	0	0	0	0	0
0	0	0	0	0	0	0	0	0	0	0	0	0	0
6,62E-05	2,00E-04	0	0	0	3,00E-04	2,00E-04	0	2,00E-04	0	0,0012	0	0	3,00E-04
2,00E-04	3,00E-04	0	0	1,00E-04	4,00E-04	5,00E-04	0	2,00E-04	1,00E-04	0	0	0	0
0	0	0	0	0	0	0	0	0	0	0	0	0	0
0	0	0	0	0	0	0	0	0	0	0	0	0	0
0	2,99E-05	0	0	0	9,01E-06	0	0	0	0	0	0	0	0
0	3,00E-04	0	0	4,49E-05	0,0011	4,00E-04	0	7,00E-04	3,00E-04	0,0024	0	0	0
0	0	0	0	0	0	0	0	0	0	0	0	0	0
0,0015	1,00E-04	0	0	0	8,96E-06	2,00E-04	0	3,00E-04	0,0011	0	0	0	0
0	0	0	0	0	8,99E-06	0	0	3,23E-05	0	0	0	0	0
0	0	0	0	0	0	0	0	0	0	0	0	0	0
0	0	0	0	0	0	0	0	0	0	0	0	0	0
0	2,00E-04	0	0	0	4,57E-05	2,00E-04	0	3,23E-05	0	0	0	0	0
0	0	0	0	0	0	0	0	0	0	0	0	0	0
0	0	0	0	0	1,04E-05	0	0	0	0	0	0	0	0
0	0	0	0	0	0	0	0	0	0	0	0	0	0
0	0	0	0	0	0	0	0	0	0	0	0	0	0
0	0	0	0	0	0	0	0	0	0	0	0	0	0
7,00E-04	5,00E-04	0	0	0	2,48E-05	0	7,39E-05	8,00E-04	0,0029	0	0	0	0
0	3,00E-04	0	0	0	4,00E-04	0	0	2,00E-04	1,00E-04	0,0012	0	0	0
0	0	0	0	0	8,96E-06	0	3,25E-05	0	0	0	0	0	0
0	0	0	0	0	0	0	0	0	0	0	0	0	0
0	8,95E-05	0	0	0	0	4,00E-04	0	0	0	0	0	0	0
0	0	0	6,00E-04	0	0	0	0	0	0	0	0	0	0
0	0	0	0	0	3,59E-05	0	0	3,23E-05	0	0	0	0	0
0	4,00E-04	0	0	9,12E-05	1,00E-04	2,00E-04	8,00E-04	2,00E-04	0	0	0	0	0
2,00E-04	7,00E-04	0	5,80E-05	0	0,0019	0,0011	2,00E-04	0,0012	0,0011	0,0024	0	0	3,00E-04
0	2,98E-05	0	0	0	1,79E-05	0	0	3,23E-05	1,00E-04	0	0	0	0

0	3,00E-04	0	8,61E-05	0	1,00E-04	5,00E-04	0	0	0	0	0	0	0
0	0	0	0	0	0	0	0	0	0	0	0	0	0
6,60E-05	0	1,00E-04	0	2,00E-04	2,00E-04	0	9,76E-05	2,00E-04	1,00E-04	0	0	0	6,00E-04
0	5,96E-05	0	0	0	0	0	0	0	0	0	0	0	0
0	5,99E-05	0,0012	5,84E-05	9,41E-05	4,00E-04	0	0	2,00E-04	0	0	0	0	0
6,53E-05	0	0	1,00E-04	0	8,95E-05	0	3,25E-05	3,23E-05	0	0	0	0	0
0	0	0	0	0	0	0	0	0	0	0	0	0	0
0	0	0	0	0	1,79E-05	0	0	3,23E-05	0	0	0	0	0
0	0	0	0	0	0	0	0	0	0	0	0	0	0
0	0	0	0	0	0	0	0	0	0	0	0	0	0
0,0028	0	0	0	0	0	0	0	7,00E-04	0,0024	0	0	0	0
0	0	0	0	0	0	0	0	0	0	0	0	0	0
0	3,00E-04	0	0	2,00E-04	4,00E-04	4,00E-04	7,23E-05	1,00E-04	1,00E-04	0	0	0	0
0	0	0	0	0	6,39E-05	2,00E-04	3,29E-05	6,47E-05	0	0	0	0	0
0	0	0	0	0	0	0	0	0	0	0	0	0	0
2,00E-04	5,96E-05	0	0	0	6,31E-05	2,00E-04	3,25E-05	3,23E-05	1,00E-04	0	0	0	0
0	4,00E-04	0,0031	0	0,0011	0,0039	0	0	0,0024	2,00E-04	0,0012	0,0132	0	6,00E-04
0	0	0	0	0	1,79E-05	0	0	3,23E-05	0	0	0	0	0
0	0	0	0	4,67E-05	9,16E-06	0	6,68E-05	0	0	0	0	0	0
0	0	0	0	0	0	0	0	0	0	0	0	0	0
0	0	0	0	0	0	0	0	0	0	0	0	0	0
0	0	0	0	0	0	0	0	0	0	0	0	0	0
0	0	0	0	0	0	0	0	0	0	0	0	0	0
0	0	0	0	0	0	0	0	0	0	0	0	0	0
0	0	0	0	0	0	0	0	0,0017	0,0062	0	0	0	0
0	0	0	0	0	2,68E-05	0	0	4,64E-05	0	0	0	0	0
0	0	0	0	0	0	0	0	0	0	0	0	0	0
0	0	0	0	0,0018	1,94E-05	3,00E-04	0	1,00E-04	0	0	0	0	0,0015
8,21E-05	0	0	0	0	0	0	0	0	0	0	0	0	0
8,17E-05	0,0019	1,00E-04	0	6,09E-05	6,00E-04	0,001	0	7,00E-04	3,00E-04	0,0065	0	0	0
0	0	0	0	0	0	0	0	0	0	0	0	0	0
0	0	0	0	0	0	0	0	0	0	0	0	0	0
0	0	0	0	0	0	0	0	0	0	0	0	0	0
6,00E-04	7,00E-04	0,0032	0	0,0056	0,0041	0,0017	2,00E-04	0,0029	5,00E-04	0	0	0	0,0058
0	0	0	0	0	7,49E-05	2,00E-04	0	0	0	0	0	0	0
0	1,00E-04	0	0	0	7,65E-05	3,00E-04	0	9,37E-05	2,00E-04	0	0	0	0

0	0	0	0	0	0	0	0	0	0	0	0	0	0
0	0	0	0	0,006	3,00E-04	0	0	0,0021	0	0	0	0	0,0099
0	0	0	0	0,006	3,00E-04	0	0	0,002	0	0	0	0	0,0094
0	3,77E-05	7,00E-04	0	0	3,76E-05	2,00E-04	0	9,30E-05	0	0	0,0054	0	0
0	0	0	0	0	0	0	0	2,00E-04	2,00E-04	0	0	0	0
0	0	0	0	0	1,27E-05	3,00E-04	0	4,61E-05	0	0	0	0	0
0	0	0	0	0	0	0	0	0	0	0	0	0	0
0,0028	3,00E-04	0	0	0	0	3,00E-04	0	7,00E-04	0,0025	0	0	0	0
0	0	0	8,20E-05	0	2,59E-05	0	1,00E-04	0	0	0	0	0	0
8,88E-05	2,00E-04	0	0	4,00E-04	2,00E-04	0	5,68E-05	1,00E-04	2,00E-04	0	0	0	0
0	0	0	0	0	0	2,00E-04	0	0	0	0	0	0	0
0	0	0	0	0	0	0	0	0	0	0	0	0	0
2,00E-04	6,16E-05	0	0	0	0	0	0	0	0	0	0	0	0
0	0	0	0	0	0	4,00E-04	0	0	0	0	0	0	0
0	0	0	0	0	0	0	0	0	0	0	0	0	0
0	0	0	0	0	0	0	0	0	0	0	0	0	0
2,00E-04	7,69E-05	0	7,86E-05	6,16E-05	0,0013	0,001	2,00E-04	6,00E-04	5,00E-04	0,0016	0	0	0
0	5,00E-04	0	0	0	5,00E-04	9,00E-04	0	1,00E-04	0	0	0	0	0
8,51E-05	2,00E-04	0	0	0,002	0,0012	8,00E-04	0	7,00E-04	2,00E-04	0	0	0	0,0028
0	0	0	0	0	2,51E-05	0	0	0	0	0	0	0	0
0,0028	0	0	0	0	1,25E-05	0	0	8,00E-04	0,003	0	0	0	0
0	0	0	0	0	0	0	0	0	0	0	0	0	0
0	0	0	0	0	0	0	0	0	0	0	0	0	0
0	0	0	0	0	0	0	0	0	0	0	0	0	0
0	0	0	0	0	0	0	0	0	0	0	0	0	0
0	0	0	0	0	0	0	0	0	0	0	0	0	0
0	0	0	0	0	0	0	0	0	0	0	0	0	0
0	0	0	0	0	0	0	0	0	0	0	0	0	0
0	0	0	0	0	0	0	0	0	0	0	0	0	0
3,00E-04	0	0	0	0	0	0	0	0	0	0	0	0	0

gnomAD_g	gnomAD_g	1000g201	CADD_raw	CADD_raw	CADD_phr	SpliceAI_in	SpliceAI_h	SpliceAI_p	PossibleDe	DeNovoGe	QUAL	FILTER	INFO
2,00E-04	0,001	0	0	0	0	NA	NA	NA	NA	NA	4157,62	PASS	AC=2;AF=0
0	0	0	0	0	0	NA	NA	NA	NA	NA	1985,62	PASS	AC=2;AF=0
0	0	0	9,452	0,999	50	SKI|0.00|0	Donor Gai	PP3	hiConfDeN	1	1494,1	PASS	AC=1;AF=0
0	0	0	4,59	0,919	32	TP73|0.99	Acceptor G	PP3	NA	NA	1185,05	PASS	AC=1;AF=0
0	0	0	0	0	0	KCNAB2|0	Donor Los	PP3	NA	NA	543,78	PASS	AC=2;AF=0
0	0	0	0	0	0	CHD5|0.00	Donor Gai	PP3	NA	NA	268,09	PASS	AC=1;AF=0
0	0	0	8,987	0,997	46	CHD5|0.66	Acceptor G	PP3	NA	NA	1975	PASS	AC=1;AF=0
0	0	0	0	0	0	NA	NA	NA	NA	NA	1297,94	PASS	AC=1;AF=0
0	0	0	0	0	0	ICMT|0.00	Donor Los	PP3	NA	NA	131	PASS	AC=1;AF=0
0	0	0	4,414	0,903	31	ACOT7|0.0	NA	NA	NA	NA	3569,7	PASS	AC=2;AF=0
0	0	0	0	0	0	DNAJC11|	NA	NA	NA	NA	18295,6	PASS	AC=2;AF=0
1,00E-04	0	0	0	0	0	PARK7|0.0	NA	NA	NA	NA	834,51	PASS	AC=3;AF=0
0	0	0	0	0	0	NA	NA	NA	NA	NA	6086,31	PASS	AC=2;AF=0
0	0	0	0	0	0	NA	NA	NA	NA	NA	1700,73	PASS	AC=2;AF=0
0	0	0	0	0	0	RERE|0.01	Acceptor L	PP3	NA	NA	401,64	PASS	AC=1;AF=0
0	0	0	4,405	0,902	31	TMEM201	NA	NA	NA	NA	920,7	PASS	AC=2;AF=0
6,68E-05	0	0	4,353	0,893	30	TMEM201	NA	NA	NA	NA	864,73	PASS	AC=2;AF=0
0	0	0,0002	0	0	0	PIK3CD|0.	Donor Gai	PP3	NA	NA	1638,69	PASS	AC=2;AF=0
0	0	0	4,366	0,896	31	CLSTN1|0.	NA	NA	NA	NA	2884,07	PASS	AC=1;AF=0
0	0	0	0	0	0	RBP7|0.17	Acceptor G	VUS	NA	NA	1751,63	PASS	AC=2;AF=0
0	0	0	6,983	0,97	37	UBE4B|0.0	Donor Los	BP7	NA	NA	2438	PASS	AC=1;AF=0
0	0	0	4,35	0,893	30	UBE4B|0.0	Acceptor L	BP7	NA	NA	498,75	PASS	AC=2;AF=0
0	0	0	4,388	0,899	31	KIF1B|0.00	NA	NA	NA	NA	3467,73	PASS	AC=2;AF=0
0	0	0,000399	4,826	0,928	33	PGD|0.00|	NA	NA	NA	NA	1170,69	PASS	AC=2;AF=0
0	0	0	4,415	0,903	31	CASZ1|0.0	NA	NA	NA	NA	3640,72	PASS	AC=2;AF=0
0	0	0	4,692	0,924	32	CASZ1|0.0	Donor Los	BP7	NA	NA	1815,04	PASS	AC=1;AF=0
0	0	0	4,417	0,903	31	KAZN|0.04	Acceptor G	BP7	NA	NA	1973,68	PASS	AC=2;AF=0
0	0	0	0	0	0	KAZN|0.00	Acceptor L	BP7	NA	NA	1144,62	PASS	AC=2;AF=0
0	0	0,0002	1,93	0,292	18,72	ZBTB17|0.	Acceptor G	PP3	NA	NA	388	PASS	AC=1;AF=0
0	0	0	0	0	0	FBXO42|0	Donor Los	VUS	NA	NA	409,97	PASS	AC=2;AF=0
0	0	0	0	0	0	RCC2|0.01	Acceptor G	BP7	NA	NA	86,94	PASS	AC=1;AF=0
0	0	0	4,386	0,899	31	RCC2|0.00	NA	NA	NA	NA	460	PASS	AC=1;AF=0
0	0	0	4,95	0,931	33	RCC2|0.00	NA	NA	NA	NA	2116,88	PASS	AC=2;AF=0
0	0	0	4,595	0,919	32	IFFO2|0.0	NA	NA	NA	NA	1403,68	PASS	AC=2;AF=0

0	0	0	4,716	0,925	32	UBR4|0.00	Acceptor L	BP7	NA	NA			396	PASS	AC=1;AF=0
0	0	0	7,379	0,977	38	ECE1|0.00	Donor Los	BP7	NA	NA			789	PASS	AC=1;AF=0
0	0	0	4,599	0,919	32	RAP1GAP|	Donor Gai	BP7	NA	NA			757	PASS	AC=1;AF=0
0	0	0	4,361	0,895	31	EPHB2|0.0	Donor Gai	BP7	NA	NA			3138,18	PASS	AC=2;AF=0
0	0	0	4,602	0,919	32	EPHB2|0.0	Acceptor G	BP7	NA	NA			1072,11	PASS	AC=1;AF=0
0	0	0	4,447	0,907	32	EPHB2|0.0	NA	NA	NA	NA			1617,03	PASS	AC=1;AF=0
0	0	0	4,491	0,912	32	KDM1A|0.	NA	NA	NA	NA			974	PASS	AC=1;AF=0
0	0	0	4,554	0,917	32	KDM1A|0.	NA	NA	NA	NA			778	PASS	AC=1;AF=0
0	0	0	0	0	0	LUZP1|0.0	NA	NA	NA	NA			1526,93	PASS	AC=1;AF=0
0	0	0	5,003	0,933	33	GRHL3|0.0	Donor Gai	VUS	NA	NA			2016,69	PASS	AC=2;AF=0
0	0	0	4,5	0,912	32	SRRM1|0.	NA	NA	NA	NA			421,06	PASS	AC=1;AF=0
0	0	0	4,559	0,917	32	RUNX3|0.	NA	NA	NA	NA			2383,97	PASS	AC=2;AF=0
0	0	0	0	0	0	HMGN2|0	Acceptor L	VUS	NA	NA			709,01	PASS	AC=1;AF=0
0	0	0	0	0	0	SLC9A1|0.	Donor Gai	PP3	NA	NA			1489,94	PASS	AC=1;AF=0
0	0	0	4,622	0,921	32	WASF2|0.	Donor Los	BP7	NA	NA			601,06	PASS	AC=1;AF=0
6,67E-05	0	0	4,428	0,905	31	STX12|0.0	NA	NA	NA	NA			1735,73	PASS	AC=2;AF=0
0	0	0	0	0	0	RPA2|0.00	Donor Gai	PP3	NA	NA			2048,11	PASS	AC=2;AF=0
0	0	0	5,33	0,941	33	EYA3|0.00	Donor Los	PP3	NA	NA			1861,69	PASS	AC=2;AF=0
0	0	0	0	0	0	GMEB1|0.	Acceptor G	PP3	NA	NA			154,1	PASS	AC=1;AF=0
0	0	0	0	0	0	YTHDF2|0.	Acceptor G	VUS	NA	NA			589,33	PASS	AC=1;AF=0
0	0	0	0	0	0	EIF3I|0.00	Donor Gai	PP3	NA	NA			1334	PASS	AC=1;AF=0
6,96E-05	0	0	0	0	0	ZBTB8OS|	Donor Los	PP3	NA	NA			248,68	PASS	AC=1;AF=0
0	0	0	0	0	0	ZMYM4|0.	Donor Los	PP3	NA	NA			542,13	PASS	AC=1;AF=0
0	0	0	0	0	0	PSMB2|0.	Acceptor L	PP3	NA	NA			2074,68	PASS	AC=2;AF=0
0	0	0	4,414	0,903	31	MAP7D1|	NA	NA	NA	NA			4857,68	PASS	AC=2;AF=0
0	0	0	4,522	0,914	32	MAP7D1|	Acceptor G	VUS	NA	NA			2896,68	PASS	AC=2;AF=0
0	0	0	0	0	0	NA	NA	NA	NA	NA			2095,62	PASS	AC=2;AF=0
8,00E-04	0,001	0	4,51	0,913	32	MACF1|0.	Acceptor G	BP7	NA	NA			2608,72	PASS	AC=2;AF=0
0	0	0	4,507	0,913	32	MACF1|0.	Donor Gai	BP7	NA	NA			258,01	PASS	AC=1;AF=0
0	0	0	4,358	0,894	30	MACF1|0.	NA	NA	NA	NA			597,08	PASS	AC=2;AF=0
0	0	0	4,491	0,912	32	MACF1|0.	NA	NA	NA	NA			884,7	PASS	AC=2;AF=0
0	0	0	4,415	0,903	31	MACF1|0.	NA	NA	NA	NA			840,68	PASS	AC=2;AF=0
0	0	0	4,398	0,901	31	MACF1|0.	Acceptor G	PP3	NA	NA			2213,69	PASS	AC=2;AF=0
0	0	0,000399	4,468	0,909	32	MACF1|0.	Acceptor G	BP7	NA	NA			1103	PASS	AC=1;AF=0
0	0	0	0	0	0	HIVEP3|0.	NA	NA	NA	NA			2230,05	PASS	AC=1;AF=0
0,0011	0,001	0	4,358	0,894	30	HIVEP3|0.	Donor Los	BP7	NA	NA			607,02	PASS	AC=1;AF=0

0	0	0	0	0	0	HIVEP3|0.	NA	NA	NA	NA	1309,69	PASS	AC=2;AF=0
0	0	0	4,389	0,899	31	RNF220|0.	Donor Los	BP7	NA	NA	1311,68	PASS	AC=2;AF=0
6,67E-05	0	0	4,712	0,924	32	RPS8|0.00	Donor Los	VUS	NA	NA	662	PASS	AC=1;AF=0
0	0	0	7,678	0,982	39	NASP|0.00	NA	NA	NA	NA	1083,71	PASS	AC=2;AF=0
0	0	0	4,775	0,926	33	LRRC41|0.	Donor Los	PP3	NA	NA	278,06	PASS	AC=2;AF=0
0,0016	0,002	0	0	0	0	FAF1|0.00	Donor Gai	VUS	NA	NA	413,71	PASS	AC=2;AF=0
6,70E-05	0	0,0002	5,43	0,944	34	OSBPL9|0.	Donor Los	PP3	NA	NA	1533,74	PASS	AC=2;AF=0
1,00E-04	0	0	0	0	0	OSBPL9|0.	Donor Los	PP3	NA	NA	508,65	PASS	AC=1;AF=0
0	0	0	0	0	0	LRP8|0.22	Acceptor G	PP3	NA	NA	1751,05	PASS	AC=1;AF=0
6,91E-05	0	0	0	0	0	NA	NA	NA	NA	NA	24692,1	PASS	AC=2;AF=0
0	0	0	0	0	0	NDC1|0.0	Acceptor L	VUS	NA	NA	412,74	PASS	AC=2;AF=0
0	0	0	8,523	0,994	43	DIO1|0.00	Donor Los	BP7	NA	NA	1154,49	PASS	AC=1;AF=0
0	0	0,0002	0	0	0	DAB1|0.08	Donor Los	PP3	NA	NA	501	PASS	AC=1;AF=0
0	0	0,0002	0	0	0	DNAJC6|0	Acceptor G	VUS	NA	NA	471	PASS	AC=1;AF=0
0	0	0	3,687	0,69	25,4	LEPR|0.02	Acceptor L	PP3	NA	NA	551,14	PASS	AC=1;AF=0
0	0	0	4,51	0,913	32	SERBP1|0.	NA	NA	NA	NA	188,02	PASS	AC=1;AF=0
6,67E-05	0	0	4,426	0,904	31	LRRC7|0.0	Acceptor L	BP7	NA	NA	498	PASS	AC=1;AF=0
0	0	0	0	0	0	LPAR3|0.0	NA	NA	NA	NA	990,99	PASS	AC=1;AF=0
0	0	0	4,391	0,9	31	LPAR3|0.0	Donor Los	BP7	NA	NA	1091	PASS	AC=1;AF=0
0	0	0	0	0	0	LMO4|0.0	Donor Los	PP3	NA	NA	243,91	PASS	AC=1;AF=0
0	0	0	0	0	0	ARHGAP29	NA	NA	NA	NA	1789,76	PASS	AC=2;AF=0
0	0	0	4,453	0,908	32	PTBP2|0.0	Donor Los	BP7	NA	NA	569	PASS	AC=1;AF=0
0	0	0	4,465	0,909	32	COL11A1|	Acceptor L	BP7	NA	NA	1005,01	PASS	AC=1;AF=0
0	0	0	0	0	0	COL11A1|	Acceptor L	VUS	NA	NA	1032	PASS	AC=1;AF=0
0	0	0	4,368	0,896	31	COL11A1|	NA	NA	NA	NA	572,7	PASS	AC=2;AF=0
0	0	0	0	0	0	NA	NA	NA	NA	NA	1940,63	PASS	AC=2;AF=0
0	0	0	5,564	0,947	34	SORT1|0.0	Donor Los	PP3	NA	NA	967,69	PASS	AC=2;AF=0
0	0	0	4,833	0,928	33	CSF1|0.00	Donor Gai	PP3	NA	NA	1843,72	PASS	AC=2;AF=0
0	0	0	0	0	0	SLC6A17|0	Acceptor G	PP3	NA	NA	916,04	PASS	AC=1;AF=0
0	0	0	4,427	0,905	31	RBM15|0.	NA	NA	NA	NA	1292,73	PASS	AC=2;AF=0
1,00E-04	0	0	0	0	0	NA	NA	NA	NA	NA	4323,68	PASS	AC=2;AF=0
6,67E-05	0,001	0,001398	4,638	0,921	32	CAPZA1|0	NA	NA	NA	NA	471	PASS	AC=1;AF=0
6,68E-05	0	0	0	0	0	RHOC|0.0	NA	NA	NA	NA	1716,95	PASS	AC=2;AF=0
0	0	0	5,318	0,941	33	MAN1A2|	Acceptor L	PP3	NA	NA	920,76	PASS	AC=2;AF=0
0	0	0	6,72	0,967	36	NOTCH2|0	Acceptor G	BP7	NA	NA	451,89	PASS	AC=2;AF=0
0	0	0	0	0	0	RPRD2|0.9	Acceptor G	PP3	NA	NA	433,81	PASS	AC=2;AF=0

0	0	0	4,414	0,903	31	POGZ|0.00	Donor Gai	PP3	NA	NA	653,04	PASS	AC=1;AF=0
0,0025	0,001	0,001198	6,881	0,969	36	RORC|0.00	Donor Los	BP7	NA	NA	124,01	PASS	AC=1;AF=0
6,75E-05	0	0	0	0	0	NA	NA	NA	NA	NA	2327,73	PASS	AC=1;AF=0
3,00E-04	0	0	0	0	0	KCNN3|0.	Donor Los	BP7	NA	NA	15547,2	PASS	AC=1;AF=0
0	0	0	4,453	0,908	32	CLK2|0.00	NA	NA	NA	NA	998	PASS	AC=1;AF=0
0	0	0	4,83	0,928	33	ASH1L|0.0	Acceptor G	BP7	NA	NA	941,68	PASS	AC=2;AF=0
0	0	0	4,481	0,911	32	ARHGEF2|	NA	NA	NA	NA	990	PASS	AC=1;AF=0
0	0	0	4,739	0,925	32	ARHGEF2|	Acceptor G	BP7	NA	NA	511,76	PASS	AC=2;AF=0
0	0	0	4,865	0,929	33	UBQLN4|0	Acceptor G	PP3	NA	NA	1943,68	PASS	AC=2;AF=0
6,68E-05	0	0	0	0	0	LMNA|0.9	Acceptor G	PP3	NA	NA	3097	PASS	AC=1;AF=0
0	0	0	0	0	0	LMNA|0.1	Acceptor G	VUS	NA	NA	1335,92	PASS	AC=2;AF=0
0	0	0	0	0	0	CCT3|0.00	NA	NA	NA	NA	576,96	PASS	AC=1;AF=0
0	0	0,001398	4,409	0,902	31	ARHGEF11	NA	NA	NA	NA	2638,68	PASS	AC=2;AF=0
0	0	0	4,621	0,92	32	ARHGEF11	Acceptor L	BP7	NA	NA	830	PASS	AC=1;AF=0
0	0	0	0	0	0	COPA|0.2	Acceptor G	PP3	NA	NA	162,01	PASS	AC=1;AF=0
0	0	0	4,432	0,905	31	VANGL2|0	Donor Los	BP7	NA	NA	1865	PASS	AC=1;AF=0
0	0	0	4,38	0,898	31	DEDD|0.0	Acceptor L	BP7	NA	NA	886,02	PASS	AC=1;AF=0
0	0	0	4,58	0,918	32	TOMM40L	Donor Gai	VUS	NA	NA	168	PASS	AC=1;AF=0
0	0	0	4,868	0,929	33	NOS1AP|0	Donor Los	PP3	NA	NA	1864,72	PASS	AC=2;AF=0
0,0025	0	0,000599	5,417	0,943	34	UHMK1|0.	Donor Los	BP7	NA	NA	691,01	PASS	AC=1;AF=0
0	0	0	4,953	0,931	33	DDR2|0.00	Acceptor L	BP7	NA	NA	3364,68	PASS	AC=2;AF=0
0	0	0,001398	0	0	0	UCK2|0.00	Donor Gai	PP3	NA	NA	1113,71	PASS	AC=2;AF=0
0	0	0	4,544	0,916	32	ILDR2|0.0	NA	NA	NA	NA	389,13	PASS	AC=1;AF=0
0	0	0	0	0	0	ZBTB37|0.	NA	NA	NA	NA	4006,66	PASS	AC=2;AF=0
6,67E-05	0	0,0002	4,548	0,916	32	RC3H1|0.0	Donor Los	BP7	NA	NA	1220,69	PASS	AC=2;AF=0
6,67E-05	0	0	4,477	0,91	32	RC3H1|0.0	NA	NA	NA	NA	1969,72	PASS	AC=2;AF=0
6,66E-05	0	0	4,5	0,912	32	CEP350|0.	Acceptor L	VUS	NA	NA	342	PASS	AC=1;AF=0
2,00E-04	0	0	0	0	0	DHX9|0.00	Acceptor L	VUS	NA	NA	1021,85	PASS	AC=2;AF=0
0	0	0,000599	4,518	0,914	32	LAMC1|0.	Donor Los	BP7	NA	NA	642	PASS	AC=1;AF=0
0	0	0	0	0	0	LAMC1|0.	NA	NA	NA	NA	7219,62	PASS	AC=2;AF=0
6,67E-05	0,001	0	4,421	0,904	31	RGL1|0.00	Acceptor L	VUS	NA	NA	1852,69	PASS	AC=2;AF=0
0	0	0	6,73	0,967	36	TRMT1L|0	Acceptor L	BP7	NA	NA	616,01	PASS	AC=1;AF=0
0	0	0	0	0	0	TRMT1L|0	Acceptor G	VUS	NA	NA	1505,9	PASS	AC=2;AF=0
0	0	0	4,387	0,899	31	TPR|0.00|	NA	NA	NA	NA	816,01	PASS	AC=1;AF=0
0	0	0	4,363	0,895	31	LHX9|0.02	Acceptor L	BP7	NA	NA	431,7	PASS	AC=2;AF=0
0	0	0	4,403	0,901	31	LMOD1|0.	NA	NA	NA	NA	1234	PASS	AC=1;AF=0

0	0	0	0	0	0	MYOG|0.0	Donor Los	PP3	NA	NA	4162,62	PASS	AC=2;AF=0
0	0	0	0	0	0	SOX13|0.0	Donor Gai	PP3	NA	NA	1030,02	PASS	AC=1;AF=0
0	0	0	4,489	0,911	32	PIK3C2B|0	Acceptor G	PP3	NA	NA	182,01	PASS	AC=1;AF=0
0	0	0	4,445	0,907	31	PIK3C2B|0	Donor Gai	VUS	NA	NA	1293,92	PASS	AC=1;AF=0
0	0	0,002995	4,618	0,92	32	PIK3C2B|0	Donor Gai	BP7	NA	NA	1491,7	PASS	AC=2;AF=0
0	0	0	4,394	0,9	31	NFASC|0.0	NA	NA	NA	NA	3941,13	PASS	AC=3;AF=0
0	0	0	2,589	0,432	22,6	NFASC|0.0	Acceptor L	VUS	NA	NA	2678,69	PASS	AC=2;AF=0
0	0	0,000599	4,363	0,895	31	DSTYK|0.0	Donor Los	BP7	NA	NA	862,07	PASS	AC=1;AF=0
3,00E-04	0	0,0002	5,321	0,941	33	DSTYK|0.0	Donor Los	PP3	NA	NA	348,15	PASS	AC=1;AF=0
6,68E-05	0	0,000599	4,364	0,895	31	MAPKAPK	NA	NA	NA	NA	1377	PASS	AC=1;AF=0
0	0	0	7,982	0,987	40	KCNH1|0.	NA	NA	NA	NA	745,01	PASS	AC=1;AF=0
0	0	0	4,409	0,902	31	PPP2R5A|	NA	NA	NA	NA	449,72	PASS	AC=2;AF=0
0	0	0	0	0	0	NA	NA	NA	NA	NA	2868,95	PASS	AC=1;AF=0
0,001	0,001	0,000799	4,624	0,921	32	RAB3GAP2	Donor Los	BP7	NA	NA	2667,7	PASS	AC=2;AF=0
0	0	0	4,522	0,914	32	CNIH3|0.0	Donor Los	BP7	NA	NA	1159,68	PASS	AC=2;AF=0
0	0	0	5,553	0,947	34	CDC42BPA	Donor Los	PP3	NA	NA	3519,72	PASS	AC=2;AF=0
0	0	0	0	0	0	TRIM67|0.	Acceptor L	VUS	NA	NA	1511,69	PASS	AC=2;AF=0
0	0	0	4,414	0,903	31	HEATR1|0	NA	NA	NA	NA	679,01	PASS	AC=1;AF=0
3,00E-04	0	0	4,389	0,899	31	HEATR1|0	Acceptor L	BP7	NA	NA	684,06	PASS	AC=1;AF=0
0	0	0	4,552	0,916	32	HEATR1|0	Acceptor L	BP7	NA	NA	829,01	PASS	AC=1;AF=0
0	0	0	0,39	0,079	5,313	ACTN2|0.0	Donor Los	BP7	NA	NA	1229,89	PASS	AC=2;AF=0
0	0	0,0002	4,363	0,895	31	ACTN2|0.0	NA	NA	NA	NA	615,09	PASS	AC=1;AF=0
0	0	0	4,654	0,922	32	RYR2|0.00	Donor Gai	BP7	NA	NA	405,03	PASS	AC=1;AF=0
0	0	0,000799	0	0	0	RYR2|0.03	Acceptor L	VUS	NA	NA	121,11	PASS	AC=1;AF=0
0	0	0	4,49	0,911	32	FMN2|0.0	NA	NA	NA	NA	2144,7	PASS	AC=2;AF=0
0	0	0	4,56	0,917	32	CEP170|0.	Donor Gai	BP7	NA	NA	350,1	PASS	AC=1;AF=0
0	0	0,000399	4,511	0,913	32	KIF26B|0.0	NA	NA	NA	NA	2332,52	PASS	AC=3;AF=0
6,68E-05	0	0	4,478	0,91	32	KIF26B|0.0	NA	NA	NA	NA	526,26	PASS	AC=1;AF=0
0	0	0	4,374	0,897	31	AHCTF1|0	Acceptor L	BP7	NA	NA	512,03	PASS	AC=1;AF=0
1,00E-04	0	0,006789	0	0	0	MYT1L|0.0	Acceptor L	VUS	NA	NA	1335,86	PASS	AC=1;AF=0
0	0	0	4,572	0,918	32	RPS7|0.00	NA	NA	NA	NA	1949	PASS	AC=1;AF=0
0	0	0	4,782	0,927	33	RPS7|0.00	NA	NA	NA	NA	1585	PASS	AC=1;AF=0
0	0	0	4,395	0,9	31	SOX11|0.0	NA	NA	NA	NA	2837,29	PASS	AC=2;AF=0
6,67E-05	0	0,0002	4,622	0,921	32	MBOAT2|	Acceptor L	BP7	NA	NA	218	PASS	AC=1;AF=0
0	0	0	0	0	0	ASAP2|0.0	Donor Los	PP3	NA	NA	1575,82	PASS	AC=2;AF=0
0	0	0	4,635	0,921	32	YWHAQ|0	NA	NA	NA	NA	1808,96	PASS	AC=2;AF=0

0	0	0,0002	5,529	0,946	34	HPCAL1|0	Donor Los	PP3	NA	NA	400,09	PASS	AC=1;AF=0
0	0	0	5,502	0,946	34	DDX1|0.35	Acceptor L	PP3	NA	NA	592	PASS	AC=1;AF=0
0	0	0	0	0	0	NA	NA	NA	NA	NA	2209,81	PASS	AC=2;AF=0
0	0	0	0	0	0	PUM2|0.0	Donor Los	PP3	NA	NA	678,02	PASS	AC=1;AF=0
0	0	0	0	0	0	PUM2|0.0	NA	NA	NA	NA	1660,97	PASS	AC=1;AF=0
0	0	0	0	0	0	ATAD2B|0	Acceptor L	PP3	NA	NA	365,74	PASS	AC=2;AF=0
3,00E-04	0	0	4,566	0,917	32	EFR3B|0.0	NA	NA	NA	NA	739	PASS	AC=1;AF=0
0	0	0	0	0	0	RAB10|0.0	Donor Los	VUS	NA	NA	1652,75	PASS	AC=2;AF=0
0	0	0	0	0	0	DPYSL5|0.	Donor Los	PP3	NA	NA	262,67	PASS	AC=1;AF=0
0	0,001	0	0	0	0	CAD|0.85|	Acceptor G	PP3	NA	NA	1946,7	PASS	AC=2;AF=0
0	0	0	7,846	0,985	39	ZNF513|0.	Acceptor L	BP7	NA	NA	2949,69	PASS	AC=2;AF=0
0	0	0	5,424	0,944	34	CCDC121|	Donor Gai	BP7	NA	NA	1127,68	PASS	AC=2;AF=0
0	0	0	4,341	0,891	29,9	CCDC121|	NA	NA	NA	NA	1711,03	PASS	AC=1;AF=0
0	0	0	0	0	0	PPP1CB|0.	Acceptor L	VUS	NA	NA	262,1	PASS	AC=1;AF=0
0	0,001	0,000399	4,402	0,901	31	WDR43|0.	NA	NA	NA	NA	4069,73	PASS	AC=2;AF=0
0	0	0,000399	4,741	0,925	32	BIRC6|0.0	Acceptor L	VUS	NA	NA	627,06	PASS	AC=1;AF=0
0	0	0	0	0	0	BIRC6|0.0	Donor Gai	PP3	NA	NA	342,04	PASS	AC=1;AF=0
0	0	0	0	0	0	CRIM1|0.0	Donor Gai	BP7	NA	NA	661,06	PASS	AC=1;AF=0
0	0	0	4,363	0,895	31	CRIM1|0.0	Acceptor L	BP7	NA	NA	464	PASS	AC=1;AF=0
1,00E-04	0	0,000399	1,924	0,291	18,67	HNRNPLL|	Acceptor G	BP7	NA	NA	636,06	PASS	AC=1;AF=0
0	0	0	0	0	0	HNRNPLL|	Donor Gai	VUS	NA	NA	1447,86	PASS	AC=2;AF=0
0	0	0	0	0	0	SRSF7|0.0	Donor Gai	PP3	NA	NA	379	PASS	AC=1;AF=0
0	0	0	0	0	0	SOS1|0.00	Donor Los	PP3	NA	NA	2072,88	PASS	AC=1;AF=0
1,00E-04	0	0,000399	4,952	0,931	33	SOS1|0.01	Acceptor L	PP3	NA	NA	580,03	PASS	AC=1;AF=0
0	0	0	4,515	0,914	32	SIX3|0.00|	NA	NA	NA	NA	3575,72	PASS	AC=2;AF=0
0	0	0	4,553	0,917	32	SIX2|0.00|	Donor Los	BP7	NA	NA	3183,79	PASS	AC=2;AF=0
0	0	0	4,728	0,925	32	SIX2|0.00|	Donor Los	BP7	NA	NA	508,06	PASS	AC=1;AF=0
2,00E-04	0	0	4,617	0,92	32	MSH2|0.0	NA	NA	NA	NA	921,84	PASS	AC=2;AF=0
0	0	0	0	0	0	PSME4|0.	Acceptor L	PP3	NA	NA	156	PASS	AC=1;AF=0
0	0	0	0	0	0	PSME4|0.	Acceptor L	PP3	NA	NA	470,33	PASS	AC=1;AF=0
0	0	0	4,622	0,921	32	SPTBN1|0.	NA	NA	NA	NA	1450,76	PASS	AC=2;AF=0
0	0	0	4,444	0,907	31	CCDC88A|	Donor Gai	PP3	NA	NA	1785,68	PASS	AC=2;AF=0
0	0	0	4,923	0,931	33	EFEMP1|0	NA	NA	NA	NA	449	PASS	AC=1;AF=0
0	0	0	4,603	0,92	32	USP34|0.0	NA	NA	NA	NA	189,06	PASS	AC=1;AF=0
0	0	0	4,422	0,904	31	USP34|0.2	Acceptor G	PP3	NA	NA	756,05	PASS	AC=1;AF=0
0	0	0,0002	4,485	0,911	32	ANTXR1|0	Acceptor L	BP7	NA	NA	685,68	PASS	AC=2;AF=0

0	0	0,000599	4,41	0,902	31	TGFA|0.00	NA	NA	NA	NA	470	PASS	AC=1;AF=0
0	0	0	4,587	0,919	32	CYP26B1|	NA	NA	NA	NA	3652,88	PASS	AC=2;AF=0
6,67E-05	0	0	4,522	0,914	32	CYP26B1|	NA	NA	NA	NA	1933	PASS	AC=1;AF=0
0	0	0	4,761	0,926	33	FBXO41|0	Acceptor G	BP7	NA	NA	1824,69	PASS	AC=2;AF=0
0	0	0	4,474	0,91	32	INO80B-W	Donor Los	BP7	NA	NA	43,54	PASS	AC=1;AF=0
0	0	0	0	0	0	INO80B-W	NA	NA	NA	NA	1783,07	PASS	AC=1;AF=0
0	0	0	4,425	0,904	31	DNAH6|0.	Acceptor G	PP3	NA	NA	226,03	PASS	AC=1;AF=0
6,66E-05	0	0	4,656	0,922	32	DNAH6|0.	Acceptor G	PP3	NA	NA	1589,68	PASS	AC=2;AF=0
0	0	0	4,674	0,923	32	SNRNP200	NA	NA	NA	NA	593,01	PASS	AC=1;AF=0
0	0	0	4,6	0,919	32	SNRNP200	NA	NA	hiConfDeN	1	744	PASS	AC=1;AF=0
0	0	0	4,41	0,902	31	INPP4A|0.	Donor Los	BP7	NA	NA	1967	PASS	AC=1;AF=0
0	0	0	0	0	0	INPP4A|0.	Acceptor L	PP3	NA	NA	1382,71	PASS	AC=2;AF=0
0	0	0	0	0	0	INPP4A|0.	Donor Gai	BP7	NA	NA	814,79	PASS	AC=2;AF=0
0	0	0	0,431	0,084	5,771	EIF5B|0.00	Donor Gai	VUS	NA	NA	1028,68	PASS	AC=2;AF=0
0	0	0	7,866	0,985	39	EIF5B|0.00	Donor Los	VUS	NA	NA	752,13	PASS	AC=1;AF=0
0	0	0	0	0	0	REV1|0.00	Donor Los	PP3	NA	NA	80,18	PASS	AC=1;AF=0
0	0	0	0	0	0	NPAS2|0.0	Acceptor L	BP7	NA	NA	1200,93	PASS	AC=1;AF=0
0	0	0,0002	4,411	0,903	31	MAP4K4|0	Donor Gai	BP7	NA	NA	1588,05	PASS	AC=1;AF=0
0,0021	0	0,000599	0	0	0	RANBP2|0	Donor Los	PP3	NA	NA	855,19	PASS	AC=1;AF=0
0	0	0	6,913	0,969	36	BCL2L11|0	Acceptor G	BP7	NA	NA	874,02	PASS	AC=1;AF=0
0	0	0	4,544	0,916	32	ANAPC1|0	Donor Los	BP7	NA	NA	1161,53	PASS	AC=2;AF=0
0	0	0	4,482	0,911	32	POLR1B|0	NA	NA	NA	NA	2215,69	PASS	AC=2;AF=0
0	0	0	4,409	0,902	31	GLI2|0.00	NA	NA	NA	NA	2882,08	PASS	AC=1;AF=0
0	0	0	4,765	0,926	33	GLI2|0.63	Acceptor G	PP3	NA	NA	3945,68	PASS	AC=2;AF=0
0	0	0,000799	4,581	0,918	32	CLASP1|0.	Acceptor L	BP7	NA	NA	2615,69	PASS	AC=2;AF=0
0	0	0	8,804	0,996	45	CNTNAP5|	Donor Los	BP7	NA	NA	1715,69	PASS	AC=2;AF=0
9,00E-04	0	0,000399	0	0	0	IWS1|0.00	Donor Gai	PP3	NA	NA	636,69	PASS	AC=2;AF=0
0	0	0	7,141	0,973	37	IWS1|0.00	NA	NA	hiConfDeN	0,999994	1182	PASS	AC=1;AF=0
3,00E-04	0	0	4,347	0,892	30	R3HDM1|	Acceptor L	VUS	NA	NA	994	PASS	AC=1;AF=0
0	0	0,0002	4,571	0,918	32	MCM6|0.0	NA	NA	NA	NA	1644,7	PASS	AC=2;AF=0
0	0	0	4,454	0,908	32	MBD5|0.0	Donor Los	VUS	NA	NA	2708,68	PASS	AC=2;AF=0
0	0	0,000599	0	0	0	KIF5C|0.00	NA	NA	NA	NA	1237,78	PASS	AC=2;AF=0
0	0	0	4,35	0,893	30	KIF5C|0.00	Donor Gai	PP3	NA	NA	1338,73	PASS	AC=2;AF=0
0	0	0	0	0	0	RIF1|0.14|	Acceptor G	VUS	NA	NA	2681,73	PASS	AC=2;AF=0
2,00E-04	0	0	4,565	0,917	32	RIF1|0.11|	Acceptor G	VUS	NA	NA	436,7	PASS	AC=2;AF=0
0	0	0	4,454	0,908	32	PRPF40A|	NA	NA	NA	NA	609,76	PASS	AC=2;AF=0

0	0	0,000399	0	0	0	GALNT13|	Acceptor L	PP3	NA	NA	1948,01	PASS	AC=3;AF=0
0	0	0	4,518	0,914	32	GALNT13|	Acceptor L	BP7	NA	NA	1091,73	PASS	AC=2;AF=0
0	0	0	0	0	0	GALNT13|	Acceptor L	PP3	NA	NA	748,12	PASS	AC=2;AF=0
0	0	0	8,565	0,994	44	BAZ2B|0.0	Acceptor G	BP7	NA	NA	284,02	PASS	AC=1;AF=0
0	0	0	6,672	0,966	36	BAZ2B|0.0	Donor Gai	BP7	NA	NA	945,69	PASS	AC=2;AF=0
0	0	0	5,195	0,937	33	SLC4A10|0	Acceptor G	PP3	NA	NA	267	PASS	AC=1;AF=0
0	0	0,000399	0	0	0	SCN3A|0.1	Acceptor G	VUS	NA	NA	1349,83	PASS	AC=2;AF=0
0	0	0	0	0	0	SCN1A|0.2	Acceptor G	PP3	loConfDeN	NA	180,17	PASS	AC=1;AF=0
0	0	0	4,355	0,894	30	LRP2|0.00	Acceptor L	VUS	NA	NA	1379,86	PASS	AC=2;AF=0
0	0	0	4,369	0,896	31	LRP2|0.00	Donor Los	BP7	NA	NA	814	PASS	AC=1;AF=0
0	0	0	0	0	0	NA	NA	NA	hiConfDeN	0,775606	889,96	PASS	AC=1;AF=0
0	0	0	4,52	0,914	32	GORASP2|	Donor Gai	VUS	NA	NA	927,69	PASS	AC=2;AF=0
0	0	0	4,458	0,908	32	TLK1|0.00	Acceptor L	BP7	NA	NA	1119,31	PASS	AC=3;AF=0
0	0	0	2,007	0,307	19,31	TLK1|0.00	NA	NA	NA	NA	805,02	PASS	AC=1;AF=0
6,66E-05	0	0	4,455	0,908	32	DLX2|0.00	Acceptor L	BP7	NA	NA	1014,04	PASS	AC=1;AF=0
0	0	0,0002	4,538	0,915	32	RAPGEF4|	Acceptor G	BP7	NA	NA	1768	PASS	AC=1;AF=0
1,00E-04	0	0,0002	4,62	0,92	32	RAPGEF4|	Donor Los	VUS	NA	NA	993,71	PASS	AC=2;AF=0
0	0	0	0	0	0	AGPS|0.05	Acceptor G	BP7	NA	NA	912,62	PASS	AC=2;AF=0
6,66E-05	0	0	0	0	0	OSBPL6|0.	Donor Los	PP3	NA	NA	428,22	PASS	AC=1;AF=0
0	0	0	4,756	0,926	32	SESTD1|0.	Donor Gai	PP3	NA	NA	1864,32	PASS	AC=3;AF=0
0	0	0	7,596	0,98	38	NEUROD1	Donor Gai	BP7	NA	NA	1440,99	PASS	AC=1;AF=0
0	0	0	4,432	0,905	31	NCKAP1|0	Acceptor G	BP7	NA	NA	223	PASS	AC=1;AF=0
0	0	0	4,426	0,905	31	CALCRL|0.	Acceptor G	PP3	NA	NA	1168,88	PASS	AC=2;AF=0
0,0011	0,001	0,0002	4,46	0,909	32	COL5A2|0	Donor Los	BP7	NA	NA	4132,68	PASS	AC=2;AF=0
2,00E-04	0,0011	0,001597	0	0	0	STAT1|0.0	Donor Gai	VUS	NA	NA	512,44	PASS	AC=1;AF=0
0	0	0	0	0	0	HECW2|0.	Acceptor G	VUS	NA	NA	1967,73	PASS	AC=2;AF=0
0	0	0	0	0	0	HSPD1|0.0	NA	NA	NA	NA	3090,63	PASS	AC=2;AF=0
0	0	0	6,573	0,965	36	SATB2|0.0	NA	NA	hiConfDeN	0,999998	654	PASS	AC=1;AF=0
0	0	0	0	0	0	SPATS2L|0	NA	NA	NA	NA	2655,63	PASS	AC=2;AF=0
0	0	0	0	0	0	INO80D|0	Acceptor L	VUS	NA	NA	506,03	PASS	AC=1;AF=0
0	0	0	8,094	0,989	41	ADAM23|	Donor Los	BP7	NA	NA	1821,7	PASS	AC=2;AF=0
0	0	0,0002	4,571	0,918	32	MAP2|0.0	Donor Los	VUS	NA	NA	1346,77	PASS	AC=2;AF=0
0	0	0	3,907	0,755	26,4	MAP2|0.0	Donor Los	PP3	NA	NA	845	PASS	AC=1;AF=0
0	0	0	4,419	0,904	31	UNC80|0.	NA	NA	NA	NA	241,01	PASS	AC=1;AF=0
0	0	0	4,379	0,898	31	UNC80|0.	NA	NA	NA	NA	500	PASS	AC=1;AF=0
3,00E-04	0	0	0	0	0	KANSL1L|0	NA	NA	NA	NA	690,77	PASS	AC=1;AF=0

0	0	0	4,373	0,897	31	KANSL1L|0	Acceptor L	PP3	NA	NA	1117,75	PASS	AC=2;AF=0
0	0	0	4,356	0,894	30	XRCC5|0.0	NA	NA	NA	NA	1464	PASS	AC=1;AF=0
0	0	0	4,616	0,92	32	ARPC2|0.0	NA	NA	NA	NA	862	PASS	AC=1;AF=0
0	0	0	4,57	0,918	32	USP37|0.0	Acceptor L	VUS	NA	NA	738,7	PASS	AC=2;AF=0
0	0	0	6,159	0,96	35	SPEG|0.01	Acceptor G	BP7	NA	NA	2885,76	PASS	AC=2;AF=0
1,00E-04	0	0,000599	4,785	0,927	33	SPEG|0.00	NA	NA	NA	NA	2039,75	PASS	AC=2;AF=0
0	0	0,0002	7,536	0,979	38	SPEG|0.00	NA	NA	NA	NA	7142,08	PASS	AC=4;AF=0
0	0	0	4,463	0,909	32	SPEG|0.00	NA	NA	NA	NA	642,21	PASS	AC=1;AF=0
6,68E-05	0	0	4,408	0,902	31	SPEG|0.01	Acceptor L	BP7	NA	NA	968,9	PASS	AC=2;AF=0
0	0	0	7,45	0,978	38	CUL3|0.03	Acceptor G	BP7	hiConfDeN	0,992499	205,08	PASS	AC=1;AF=0
0	0	0	0	0	0	AGFG1|0.	Acceptor G	PP3	NA	NA	505	PASS	AC=1;AF=0
0	0	0	8,694	0,995	44	SPHKAP|0	NA	NA	hiConfDeN	1	1175	PASS	AC=1;AF=0
0,0014	0	0	0	0	0	SPHKAP|0	Donor Los	PP3	NA	NA	375,01	PASS	AC=1;AF=0
0	0	0	4,396	0,9	31	TRIP12|0.	NA	NA	NA	NA	4128,86	PASS	AC=2;AF=0
0	0	0	4,38	0,898	31	TRIP12|0.	Donor Los	VUS	NA	NA	220	PASS	AC=1;AF=0
0	0	0,001597	5,064	0,934	33	ITM2C|0.0	NA	NA	NA	NA	244,47	PASS	AC=1;AF=0
0	0	0	4,545	0,916	32	ITM2C|0.0	NA	NA	NA	NA	836,73	PASS	AC=2;AF=0
0	0	0	0	0	0	B3GNT7|0	Acceptor G	BP7	NA	NA	5929,62	PASS	AC=2;AF=0
0	0	0	4,363	0,895	31	NCL|0.00|	Acceptor L	BP7	hiConfDeN	0,999995	211	PASS	AC=1;AF=0
0	0	0	0	0	0	NA	NA	NA	NA	NA	1302,27	PASS	AC=1;AF=0
6,66E-05	0,001	0	0	0	0	NA	NA	NA	NA	NA	1091,47	PASS	AC=1;AF=0
0	0	0	4,523	0,914	32	ATG16L1|	NA	NA	NA	NA	1819,73	PASS	AC=2;AF=0
0	0	0,0002	0	0	0	AGAP1|0.	Donor Gai	PP3	NA	NA	473,69	PASS	AC=2;AF=0
0	0	0,004992	0	0	0	COPS8|0.0	Donor Los	PP3	NA	NA	263,69	PASS	AC=2;AF=0
0	0	0	4,357	0,894	30	ILKAP|0.0	Donor Gai	BP7	NA	NA	621	PASS	AC=1;AF=0
3,00E-04	0,001	0,000599	0	0	0	PER2|0.16	Acceptor L	PP3	NA	NA	4072,68	PASS	AC=2;AF=0
0	0	0	6,518	0,964	36	SNED1|0.0	Donor Los	PP3	NA	NA	2375,88	PASS	AC=2;AF=0
0	0	0	3,141	0,552	23,7	SNED1|0.0	Donor Los	PP3	NA	NA	864,01	PASS	AC=1;AF=0
0	0	0	4,485	0,911	32	HDLBP|0.0	NA	NA	NA	NA	1690,68	PASS	AC=2;AF=0
0	0	0	8,277	0,991	42	LRRN1|0.0	NA	NA	NA	NA	3519,68	PASS	AC=2;AF=0
0	0	0	4,69	0,924	32	ITPR1|0.0	Donor Gai	PP3	NA	NA	520	PASS	AC=1;AF=0
0	0	0,0002	4,461	0,909	32	SRGAP3|0	NA	NA	NA	NA	1316,14	PASS	AC=1;AF=0
0	0	0	8,355	0,992	42	SRGAP3|0	Acceptor G	BP7	NA	NA	3249,97	PASS	AC=2;AF=0
0	0	0	4,408	0,902	31	SRGAP3|0	NA	NA	NA	NA	2455,79	PASS	AC=2;AF=0
0	0	0	4,531	0,915	32	SETD5|0.0	Donor Gai	PP3	NA	NA	2154,45	PASS	AC=3;AF=0
0	0	0	4,517	0,914	32	RAF1|0.00	NA	NA	NA	NA	2510,68	PASS	AC=2;AF=0

0	0	0	0	0	0	SLC6A6|0.	Acceptor G	PP3	NA	NA	346,48	PASS	AC=2;AF=0
6,67E-05	0	0	4,394	0,9	31	NR2C2|0.0	NA	NA	NA	NA	760,68	PASS	AC=2;AF=0
6,70E-05	0	0	0	0	0	ANKRD28|	Acceptor L	PP3	NA	NA	369,77	PASS	AC=1;AF=0
0	0	0,002196	0	0	0	ANKRD28|	Acceptor L	VUS	NA	NA	369,77	PASS	AC=1;AF=0
0	0	0	0	0	0	NKIRAS1|0	NA	NA	NA	NA	357,61	PASS	AC=1;AF=0
0	0	0	4,523	0,914	32	SLC4A7|0.	NA	NA	NA	NA	1387,9	PASS	AC=2;AF=0
0	0	0	0	0	0	SLC4A7|0.	Acceptor G	PP3	NA	NA	548,14	PASS	AC=1;AF=0
8,27E-05	0	0	0	0	0	EOMES|0.	NA	NA	NA	NA	1470,5	PASS	AC=1;AF=0
0	0	0	5,348	0,941	34	CLASP2|0.	Acceptor L	BP7	NA	NA	379,7	PASS	AC=2;AF=0
0	0	0	7,252	0,975	37	PDCD6IP|	NA	NA	NA	NA	1291,69	PASS	AC=2;AF=0
0	0	0	0	0	0	OXSR1|0.0	Donor Gai	BP7	NA	NA	1413,04	PASS	AC=1;AF=0
0	0	0	4,507	0,913	32	SCN5A|0.0	Donor Los	BP7	NA	NA	1177,71	PASS	AC=2;AF=0
6,69E-05	0	0,0002	0	0	0	SCN5A|0.2	Acceptor G	PP3	NA	NA	506,01	PASS	AC=1;AF=0
0	0	0	0	0	0	RPL14|0.0	Donor Gai	BP7	NA	NA	1970,08	PASS	AC=1;AF=0
0	0	0	7,063	0,972	37	SNRK|0.00	NA	NA	NA	NA	1766	PASS	AC=1;AF=0
0	0	0	0	0	0	SACM1L|0	Donor Los	PP3	NA	NA	1889,63	PASS	AC=2;AF=0
0	0	0	6,15	0,96	35	PTH1R|0.0	Donor Gai	BP7	NA	NA	3703,73	PASS	AC=2;AF=0
0	0	0	4,863	0,929	33	PTH1R|0.0	Acceptor G	BP7	NA	NA	3710,7	PASS	AC=2;AF=0
0	0	0,000599	0	0	0	SETD2|0.0	Donor Gai	VUS	NA	NA	1129,63	PASS	AC=2;AF=0
0	0	0	4,441	0,906	31	SMARCC1|	Acceptor L	BP7	NA	NA	458,1	PASS	AC=1;AF=0
0	0	0	4,547	0,916	32	MAP4|0.0	Donor Los	BP7	NA	NA	998,7	PASS	AC=2;AF=0
6,68E-05	0	0	5,049	0,934	33	MAP4|0.0	Donor Los	PP3	NA	NA	796,02	PASS	AC=1;AF=0
0	0	0	4,442	0,907	31	PLXNB1|0.	Acceptor G	BP7	NA	NA	843,03	PASS	AC=1;AF=0
0	0	0	5,125	0,936	33	CELSR3|0.	Acceptor L	PP3	NA	NA	1889,71	PASS	AC=2;AF=0
9,00E-04	0,002	0,000599	4,519	0,914	32	CELSR3|0.	Donor Los	BP7	NA	NA	2568,68	PASS	AC=2;AF=0
0	0	0,0002	0	0	0	CELSR3|0.	Donor Los	PP3	NA	NA	925,77	PASS	AC=2;AF=0
0	0	0	0	0	0	ARIH2|0.0	Acceptor L	BP7	NA	NA	1933,13	PASS	AC=3;AF=0
1,00E-04	0	0	0	0	0	USP19|0.1	Acceptor L	PP3	NA	NA	724,03	PASS	AC=2;AF=0
0	0	0	4,437	0,906	31	USP19|0.0	Donor Gai	BP7	NA	NA	4953,68	PASS	AC=2;AF=0
0	0	0	0	0	0	USP19|0.0	Donor Gai	PP3	NA	NA	1104,76	PASS	AC=2;AF=0
0	0	0	0	0	0	RBM6|0.0	Acceptor L	PP3	NA	NA	466,01	PASS	AC=1;AF=0
0	0	0	0	0	0	NA	NA	NA	loConfDeN	NA	13295,9	PASS	AC=1;AF=0
1,00E-04	0	0	4,409	0,902	31	CACNA2D2	Acceptor G	BP7	NA	NA	2046	PASS	AC=1;AF=0
0,0012	0	0	4,456	0,908	32	DOCK3|0.	Acceptor L	BP7	NA	NA	965	PASS	AC=1;AF=0
6,66E-05	0	0	4,802	0,927	33	DOCK3|0.	Acceptor L	BP7	NA	NA	619,99	PASS	AC=1;AF=0
0	0	0,0002	4,418	0,903	31	DOCK3|0.	Acceptor L	PP3	NA	NA	1004	PASS	AC=1;AF=0

2,00E-04	0,001	0	0	0	0	NISCH|0.0	Donor Los	VUS	NA	NA	3833,63	PASS	AC=2;AF=0
1,00E-04	0	0	4,468	0,909	32	CACNA1D|NA		NA	NA	NA	1226,7	PASS	AC=2;AF=0
0	0	0	4,405	0,902	31	CACNA1D|	Donor Gai	BP7	NA	NA	4302,7	PASS	AC=2;AF=0
0	0	0	0	0	0	CACNA2D3	Donor Gai	PP3	NA	NA	670,68	PASS	AC=2;AF=0
0	0	0	4,445	0,907	31	CACNA2D3	Acceptor L	VUS	NA	NA	461	PASS	AC=1;AF=0
0	0	0	0	0	0	ERC2|0.00	Acceptor L	PP3	NA	NA	451,94	PASS	AC=1;AF=0
0	0	0	0	0	0	ERC2|0.00	Donor Gai	VUS	NA	NA	1181,7	PASS	AC=2;AF=0
6,67E-05	0	0	0	0	0	APPL1|0.0	Acceptor L	BP7	NA	NA	300,67	PASS	AC=2;AF=0
6,67E-05	0	0	4,876	0,929	33	SLMAP|0.	Acceptor L	BP7	NA	NA	760,13	PASS	AC=2;AF=0
0	0	0	0	0	0	SLMAP|0.	Donor Los	PP3	NA	NA	1197,03	PASS	AC=2;AF=0
0	0	0	0	0	0	ATXN7|0.0	NA	NA	NA	NA	105,48	PASS	AC=1;AF=0
0	0	0	0	0	0	ATXN7|0.0	NA	NA	NA	NA	130,75	PASS	AC=1;AF=0
0	0,001	0	0	0	0	ATXN7|0.1	Acceptor G	VUS	NA	NA	1616	PASS	AC=1;AF=0
0	0	0	0	0	0	ATXN7|0.0	Acceptor L	PP3	NA	NA	1157,9	PASS	AC=2;AF=0
0	0	0	4,854	0,929	33	MAGI1|0.	Donor Los	BP7	NA	NA	1498,01	PASS	AC=1;AF=0
0	0	0	4,555	0,917	32	UBA3|0.00	Donor Los	VUS	NA	NA	101,05	PASS	AC=1;AF=0
0	0	0	5,454	0,944	34	UBA3|0.00	Donor Los	BP7	NA	NA	1196,96	PASS	AC=2;AF=0
0	0	0	4,888	0,93	33	FOXP1|0.0	Acceptor L	BP7	hiConfDeN	0,999998	470,02	PASS	AC=1;AF=0
0	0	0	7,748	0,983	39	FOXP1|0.0	Acceptor L	BP7	hiConfDeN	1	2016,03	PASS	AC=1;AF=0
0	0	0	4,466	0,909	32	ROBO2|0.	NA	NA	NA	NA	1153,78	PASS	AC=2;AF=0
6,67E-05	0	0,000599	0,029	0,044	1,525	EPHA6|0.0	NA	NA	NA	NA	2980,01	PASS	AC=2;AF=0
0	0	0	4,38	0,898	31	CBLB|0.00	NA	NA	NA	NA	697,8	PASS	AC=2;AF=0
0	0	0	4,388	0,899	31	CBLB|0.00	NA	NA	NA	NA	954,06	PASS	AC=2;AF=0
6,70E-05	0	0	0,829	0,129	9,684	BBX|0.00|	Donor Los	BP7	NA	NA	1740,69	PASS	AC=2;AF=0
0	0	0	4,987	0,932	33	LSAMP|0.	Acceptor L	PP3	NA	NA	473,95	PASS	AC=2;AF=0
0	0	0,000599	0	0	0	LSAMP|0.	Acceptor L	BP7	NA	NA	279,09	PASS	AC=1;AF=0
0	0	0	4,579	0,918	32	FSTL1|0.0	Donor Gai	PP3	NA	NA	416	PASS	AC=1;AF=0
0	0	0	4,442	0,906	31	ADCY5|0.0	NA	NA	NA	NA	1478,94	PASS	AC=2;AF=0
0	0	0	4,412	0,903	31	NA	NA	NA	NA	NA	1015	PASS	AC=1;AF=0
6,67E-05	0	0	4,424	0,904	31	PLXNA1|0	NA	NA	NA	NA	4763,7	PASS	AC=2;AF=0
0	0	0	0	0	0	PLXNA1|0	Acceptor G	PP3	NA	NA	324	PASS	AC=1;AF=0
0	0	0	4,466	0,909	32	PLXNA1|0	NA	NA	NA	NA	1644,72	PASS	AC=2;AF=0
6,67E-05	0	0	4,639	0,921	32	PLXNA1|0	NA	NA	NA	NA	457,06	PASS	AC=1;AF=0
0	0	0	4,449	0,907	32	PODXL2|0	NA	NA	NA	NA	1719,7	PASS	AC=2;AF=0
0	0	0	4,566	0,917	32	ISY1-RAB4	Donor Los	BP7	NA	NA	189	PASS	AC=1;AF=0
0	0	0	0	0	0	PLXND1|0	Acceptor G	PP3	NA	NA	1328	PASS	AC=1;AF=0

0	0	0	0	0	0	PLXND1|0	Acceptor	GVUS	NA	NA	1328	PASS	AC=1;AF=0
0	0	0	0	0	0	ASTE1|0.0	NA	NA	NA	NA	2299,93	PASS	AC=1;AF=0
6,67E-05	0	0,000599	4,503	0,913	32	DNAJC13|	NA	NA	NA	NA	391,77	PASS	AC=2;AF=0
0	0	0	9,069	0,997	47	DNAJC13|	Acceptor	GBP7	NA	NA	597,01	PASS	AC=1;AF=0
0	0	0	4,735	0,925	32	CDV3|0.00	NA	NA	NA	NA	372,01	PASS	AC=1;AF=0
0	0	0	0	0	0	TOPBP1|0	Acceptor L	PP3	NA	NA	1934,47	PASS	AC=2;AF=0
0	0	0	0	0	0	NA	NA	NA	NA	NA	1877,62	PASS	AC=2;AF=0
0	0	0	0	0	0	WWTR1|0	Donor Gai	PP3	NA	NA	809,06	PASS	AC=1;AF=0
0	0	0	4,405	0,902	31	MED12L|0	Acceptor	GBP7	NA	NA	143,1	PASS	AC=1;AF=0
0	0	0	4,517	0,914	32	GMPS|0.0	NA	NA	NA	NA	878,72	PASS	AC=2;AF=0
0	0	0,000599	4,404	0,902	31	MECOM|0	Acceptor L	PP3	NA	NA	777,72	PASS	AC=2;AF=0
0	0	0	6,359	0,962	35	TNIK|0.00	NA	NA	NA	NA	125,02	PASS	AC=1;AF=0
0	0	0	4,632	0,921	32	SOX2|0.00	NA	NA	NA	NA	3279,09	PASS	AC=1;AF=0
0	0	0	4,432	0,905	31	YEATS2|0.	Acceptor L	PP3	NA	NA	260,73	PASS	AC=2;AF=0
0	0	0	4,418	0,903	31	PSMD2|0.	Acceptor L	BP7	NA	NA	1563,68	PASS	AC=2;AF=0
0	0	0	4,449	0,907	32	EIF4G1|0.	NA	NA	NA	NA	1402,68	PASS	AC=2;AF=0
0	0	0	4,77	0,926	33	OPA1|0.01	Acceptor L	BP7	NA	NA	760,03	PASS	AC=1;AF=0
0	0	0	4,555	0,917	32	OPA1|0.00	Donor Los	BP7	NA	NA	345	PASS	AC=1;AF=0
0	0	0	4,553	0,917	32	OPA1|0.01	Acceptor L	BP7	NA	NA	1854,14	PASS	AC=3;AF=0
1,00E-04	0	0,000998	4,509	0,913	32	ATP13A3|	NA	NA	NA	NA	1267,14	PASS	AC=3;AF=0
0	0	0,000399	4,842	0,928	33	DLG1|0.01	Acceptor	GBP7	NA	NA	448,03	PASS	AC=1;AF=0
0	0	0,000599	0	0	0	PCGF3|0.0	Donor Los	VUS	NA	NA	431,01	PASS	AC=1;AF=0
0	0	0	0	0	0	ADD1|0.0	Acceptor L	BP7	NA	NA	1203,93	PASS	AC=1;AF=0
2,00E-04	0,001	0,000799	8,111	0,989	41	ADD1|0.0	NA	NA	NA	NA	1635	PASS	AC=1;AF=0
7,29E-05	0	0	0	0	0	HTT|0.00|	NA	NA	NA	NA	2865,87	PASS	AC=2;AF=0
7,24E-05	0	0	0	0	0	NA	NA	NA	NA	NA	3033,93	PASS	AC=2;AF=0
1,00E-04	0,0011	0	0	0	0	HTT|0.00|	NA	NA	NA	NA	6920,83	PASS	AC=1;AF=0
2,00E-04	0,0011	0	0	0	0	NA	NA	NA	NA	NA	7633,81	PASS	AC=1;AF=0
0,0019	0,0011	0	0	0	0	HTT|0.00|	NA	NA	NA	NA	75733,4	PASS	AC=2;AF=0
0,002	0,0011	0	0	0	0	HTT|0.00|	NA	NA	NA	NA	27059,5	PASS	AC=2;AF=0
0	0	0	0	0	0	NA	NA	NA	NA	NA	183462	PASS	AC=5;AF=0
0	0	0	4,537	0,915	32	HTT|0.00|	NA	NA	NA	NA	901	PASS	AC=1;AF=0
0	0	0	0	0	0	NA	NA	NA	NA	NA	2616,99	PASS	AC=2;AF=0
0	0	0	4,47	0,91	32	ADRA2C|0	Acceptor	GPP3	NA	NA	1867,75	PASS	AC=2;AF=0
0	0	0	6,205	0,96	35	TADA2B|0	NA	NA	NA	NA	768	PASS	AC=1;AF=0
0	0	0	0	0	0	NA	NA	NA	hiConfDeN	NA	155,65	PASS	AC=1;AF=0

0	0	0,0002	4,417	0,903	31	SLIT2|0.00	NA	NA	NA	NA	528,02	PASS	AC=1;AF=0
0	0	0	4,429	0,905	31	PDS5A|0.0	NA	NA	NA	NA	2124,72	PASS	AC=2;AF=0
3,00E-04	0	0	0	0	0	LIMCH1|0	Donor Los	BP7	NA	NA	1769,67	PASS	AC=2;AF=0
0	0	0	4,946	0,931	33	LIMCH1|0	Acceptor G	VUS	NA	NA	962,83	PASS	AC=2;AF=0
0	0	0	0	0	0	LIMCH1|0	Donor Los	BP7	NA	NA	1064,62	PASS	AC=1;AF=0
0	0	0	0	0	0	LIMCH1|0	Donor Los	VUS	NA	NA	324,01	PASS	AC=1;AF=0
0	0	0	2,505	0,414	22,4	LIMCH1|0	Donor Los	BP7	NA	NA	251,68	PASS	AC=1;AF=0
1,00E-04	0	0,0002	0	0	0	GABRB1|0	Acceptor G	PP3	NA	NA	1246	PASS	AC=1;AF=0
0	0	0	5,42	0,943	34	SLAIN2|0.	Acceptor L	PP3	NA	NA	504,12	PASS	AC=1;AF=0
0,0032	0,002	0,000399	0	0	0	USP46|0.1	Acceptor G	VUS	NA	NA	876,24	PASS	AC=1;AF=0
0	0	0	0	0	0	NA	NA	NA	NA	NA	6919,63	PASS	AC=2;AF=0
0	0	0	0	0	0	PDGFRA|0	Donor Gai	VUS	NA	NA	1079,71	PASS	AC=2;AF=0
0	0	0	6,963	0,97	36	RASGEF1B	Donor Los	BP7	NA	NA	2813,69	PASS	AC=2;AF=0
0	0	0	0	0	0	LIN54|0.0	Acceptor L	VUS	NA	NA	540,51	PASS	AC=2;AF=0
0	0	0	4,396	0,9	31	WDFY3|0.	NA	NA	NA	NA	285,01	PASS	AC=1;AF=0
0	0	0	4,369	0,896	31	WDFY3|0.	NA	NA	NA	NA	223	PASS	AC=1;AF=0
6,67E-05	0	0	0	0	0	WDFY3|0.	Acceptor G	VUS	NA	NA	133	PASS	AC=1;AF=0
0	0	0	6,03	0,958	35	MAPK10|0	Donor Los	PP3	NA	NA	626,39	PASS	AC=2;AF=0
1,00E-04	0	0,000399	4,715	0,924	32	BMPR1B|0	Donor Los	BP7	NA	NA	1930,87	PASS	AC=2;AF=0
0	0	0	0	0	0	NFKB1|0.0	Donor Gai	PP3	NA	NA	1383,76	PASS	AC=2;AF=0
0	0	0	0	0	0	INTS12|0.	Acceptor L	BP7	NA	NA	509,94	PASS	AC=1;AF=0
0	0	0	5,176	0,937	33	INTS12|0.	Donor Los	PP3	NA	NA	251,77	PASS	AC=2;AF=0
0	0	0,000399	0	0	0	ELOVL6|0.	Acceptor L	PP3	NA	NA	747	PASS	AC=1;AF=0
0	0	0	4,526	0,915	32	ANK2|0.00	NA	NA	NA	NA	491,69	PASS	AC=2;AF=0
0	0	0	0	0	0	NA	NA	NA	NA	NA	2782,66	PASS	AC=2;AF=0
0	0	0	4,732	0,925	32	ANK2|0.00	NA	NA	NA	NA	1571,69	PASS	AC=2;AF=0
0	0	0	4,417	0,903	31	CAMK2D|	NA	NA	NA	NA	578,7	PASS	AC=2;AF=0
0	0	0	6,717	0,967	36	JADE1|0.0	Donor Gai	BP7	hiConfDeN	0,999997	770	PASS	AC=1;AF=0
0	0	0	4,458	0,908	32	PCDH10|0	Acceptor L	BP7	NA	NA	3865,72	PASS	AC=2;AF=0
0	0	0	4,379	0,898	31	PCDH10|0	NA	NA	NA	NA	1268	PASS	AC=1;AF=0
0	0	0	0	0	0	PCDH10|0	NA	NA	NA	NA	2328,94	PASS	AC=1;AF=0
0	0	0	0	0	0	ELF2|0.63	Acceptor L	PP3	NA	NA	188,54	PASS	AC=2;AF=0
0	0	0,002796	4,528	0,915	32	HHIP|0.00	Donor Los	BP7	NA	NA	624,8	PASS	AC=2;AF=0
0	0	0	4,56	0,917	32	ABCE1|0.0	Acceptor L	BP7	NA	NA	1763,43	PASS	AC=2;AF=0
0	0	0	4,644	0,922	32	ZNF827|0.	NA	NA	NA	NA	2409,13	PASS	AC=3;AF=0
0	0	0	0	0	0	FNIP2|0.1	Acceptor G	VUS	NA	NA	495	PASS	AC=1;AF=0

0	0	0	0	0	0	NA	NA	NA	NA	NA	1118,04	PASS		AC=1;AF=0
0	0	0	0	0	0	NA	NA	NA	NA	NA	825,87	PASS		AC=1;AF=0
0	0	0	0	0	0	ADCY2|0.0	Donor Gai	PP3	NA	NA	996,7	PASS		AC=2;AF=0
0	0	0,000599	0	0	0	TRIO|0.00	Donor Gai	VUS	NA	NA	621,84	PASS		AC=2;AF=0
0	0	0	4,478	0,91	32	TRIO|0.00	Donor Los	VUS	NA	NA	352	PASS		AC=1;AF=0
6,67E-05	0	0	4,36	0,895	31	TRIO|0.00	NA	NA	NA	NA	3752,68	PASS		AC=2;AF=0
0	0	0,000799	4,864	0,929	33	CDH6|0.02	Acceptor	GBP7	NA	NA	816,68	PASS		AC=2;AF=0
0	0	0	0	0	0	PDZD2|0.0	Acceptor L	PP3	NA	NA	1904,68	PASS		AC=2;AF=0
0	0	0	4,408	0,902	31	PDZD2|0.0	Donor Gai	BP7	NA	NA	1751,68	PASS		AC=2;AF=0
0	0	0	4,695	0,924	32	PDZD2|0.0	Donor Gai	BP7	NA	NA	861,68	PASS		AC=2;AF=0
0	0	0	4,395	0,9	31	MTMR12|	Donor Los	BP7	NA	NA	448,69	PASS		AC=2;AF=0
0	0	0	5,349	0,941	34	MTMR12|	Donor Los	PP3	NA	NA	603	PASS		AC=1;AF=0
0	0	0	0	0	0	PRLR|0.00	Donor Los	PP3	NA	NA	520,69	PASS		AC=2;AF=0
0	0	0	4,461	0,909	32	NUP155|0	NA	NA	NA	NA	1201,01	PASS		AC=1;AF=0
0	0	0	4,462	0,909	32	PTGER4|0.	NA	NA	NA	NA	1000,88	PASS		AC=2;AF=0
0	0	0	4,577	0,918	32	NNT|0.02|	Acceptor	GBP7	NA	NA	661	PASS		AC=1;AF=0
0	0	0	4,742	0,925	32	ISL1|0.02|	Acceptor	GBP7	NA	NA	2367	PASS		AC=1;AF=0
2,00E-04	0	0,0002	7,568	0,98	38	SNX18|0.0	NA	NA	NA	NA	2490	PASS		AC=1;AF=0
1,00E-04	0	0	4,977	0,932	33	DDX4|0.00	Donor Los	PP3	NA	NA	325,01	PASS		AC=1;AF=0
6,66E-05	0	0	4,437	0,906	31	MAP3K1|0	NA	NA	NA	NA	487,76	PASS		AC=2;AF=0
6,67E-05	0,001	0	0	0	0	NA	NA	NA	NA	NA	344,23	PASS		AC=1;AF=0
0	0	0	4,415	0,903	31	PDE8B|0.0	Donor Los	BP7	NA	NA	3142,72	PASS		AC=2;AF=0
0,0019	0,001	0,0002	0	0	0	AP3B1|0.0	Donor Los	PP3	NA	NA	2415,78	PASS		AC=2;AF=0
0	0	0	8,524	0,994	43	AP3B1|0.0	Donor Los	PP3	NA	NA	2590,7	PASS		AC=2;AF=0
0	0	0	5,323	0,941	33	AP3B1|0.0	Donor Gai	BP7	NA	NA	1122,69	PASS		AC=2;AF=0
0	0	0	0	0	0	SCAMP1|0	Donor Los	VUS	NA	NA	345,89	PASS		AC=2;AF=0
0	0	0,000799	0	0	0	JMY|0.00|	Donor Gai	PP3	NA	NA	321,07	PASS		AC=1;AF=0
0	0	0	4,579	0,918	32	JMY|0.00|	Donor Los	VUS	NA	NA	788,41	PASS		AC=2;AF=0
0	0	0	4,351	0,893	30	RASGRF2|	NA	NA	NA	NA	486	PASS		AC=1;AF=0
0	0	0	4,592	0,919	32	VCAN|0.0	NA	NA	NA	NA	832	PASS		AC=1;AF=0
0	0	0	4,367	0,896	31	ELL2|0.00	Acceptor L	BP7	NA	NA	1583,7	PASS		AC=2;AF=0
0	0	0	0	0	0	ELL2|0.00	NA	NA	NA	NA	767,94	PASS		AC=1;AF=0
0	0	0	8,045	0,988	40	ELL2|0.00	NA	NA	NA	NA	685,11	PASS		AC=1;AF=0
0	0	0	4,453	0,908	32	PPIP5K2|0	Donor Los	BP7	NA	NA	701,72	PASS		AC=2;AF=0
0	0	0,000399	0	0	0	FER|0.08|	Donor Gai	PP3	NA	NA	60,09	PASS		AC=1;AF=0
0	0	0	4,428	0,905	31	FER|0.00|	NA	NA	NA	NA	379,01	PASS		AC=1;AF=0

6,66E-05	0	0	0	0	0	APC|0.00|	Donor Los	VUS	NA	NA	259,58	PASS		AC=2;AF=0
0	0	0	4,575	0,918	32	KCNN2|0.	NA	NA	NA	NA	2385	PASS		AC=1;AF=0
0	0	0	5,794	0,953	35	CCDC112|	Acceptor L	PP3	NA	NA	566,73	PASS		AC=2;AF=0
0	0	0	4,452	0,908	32	LOX|0.00|	Donor Gai	PP3	NA	NA	1170,09	PASS		AC=1;AF=0
0	0	0,001597	0	0	0	CSNK1G3|	Acceptor G	PP3	NA	NA	187,01	PASS		AC=1;AF=0
0	0	0	4,505	0,913	32	FBN2|0.00	NA	NA	NA	NA	5104,68	PASS		AC=2;AF=0
6,00E-04	0,001	0,000399	1,756	0,26	17,42	CTC-432M	NA	NA	NA	NA	1243,71	PASS		AC=2;AF=0
0	0	0	7,75	0,983	39	CTC-432M	Donor Los	PP3	NA	NA	1218,69	PASS		AC=2;AF=0
2,00E-04	0	0	4,405	0,902	31	CTC-432M	Acceptor L	BP7	NA	NA	184,01	PASS		AC=1;AF=0
0	0	0	4,524	0,914	32	CTC-432M	Donor Los	BP7	NA	NA	449	PASS		AC=1;AF=0
0	0	0	5,085	0,935	33	PDLIM4|0	Donor Los	BP7	NA	NA	1975,07	PASS		AC=1;AF=0
1,00E-04	0	0	4,547	0,916	32	KIF3A|0.0	Donor Gai	PP3	NA	NA	391,82	PASS		AC=2;AF=0
0	0	0	7,749	0,983	39	HSPA4|0.1	Acceptor G	VUS	NA	NA	1040,7	PASS		AC=2;AF=0
0	0	0	4,451	0,908	32	JADE2|0.1	Acceptor G	BP7	NA	NA	3307,7	PASS		AC=2;AF=0
6,66E-05	0	0	4,414	0,903	31	C5orf24|0	Donor Gai	BP7	NA	NA	2322	PASS		AC=1;AF=0
6,66E-05	0	0	0	0	0	HSPA9|0.0	Donor Gai	VUS	NA	NA	1191,72	PASS		AC=2;AF=0
0	0	0	0	0	0	NA	NA	NA	NA	NA	1045,21	PASS		AC=1;AF=0
0	0	0	4,364	0,895	31	ANKHD1-E	NA	NA	NA	NA	1305,55	PASS		AC=1;AF=0
0	0	0,0002	4,383	0,898	31	ANKHD1-E	Acceptor L	BP7	NA	NA	240,61	PASS		AC=1;AF=0
0	0	0	6,984	0,97	37	ANKHD1-E	NA	NA	NA	NA	831,65	PASS		AC=1;AF=0
6,00E-04	0	0	0	0	0	NA	NA	NA	NA	NA	1125,58	PASS		AC=1;AF=0
6,00E-04	0	0	0	0	0	NA	NA	NA	NA	NA	1131,57	PASS		AC=1;AF=0
0	0	0	4,801	0,927	33	NDUFA2|0	Acceptor L	PP3	NA	NA	3842,93	PASS		AC=4;AF=0
0	0	0	4,381	0,898	31	PCDHGA1|	NA	NA	NA	NA	1169,96	PASS		AC=2;AF=0
6,66E-05	0	0	4,762	0,926	33	DIAPH1|0.	Donor Los	BP7	NA	NA	1784,69	PASS		AC=2;AF=0
0	0	0	4,347	0,892	30	DIAPH1|0.	NA	NA	NA	NA	1677,68	PASS		AC=2;AF=0
0	0	0	4,613	0,92	32	NDFIP1|0.	Acceptor G	BP7	NA	NA	500,07	PASS		AC=1;AF=0
0	0	0	4,356	0,894	30	NR3C1|0.0	NA	NA	NA	NA	1446	PASS		AC=1;AF=0
0	0	0	0	0	0	YIPF5|0.01	Acceptor L	VUS	NA	NA	1295,68	PASS		AC=2;AF=0
0	0	0	4,412	0,903	31	NA	NA	NA	NA	NA	1297,01	PASS		AC=1;AF=0
0	0	0	4,856	0,929	33	FBXO38|0	Acceptor L	PP3	NA	NA	599	PASS		AC=1;AF=0
0	0	0	4,49	0,912	32	HMGXB3|	Acceptor L	BP7	NA	NA	1055	PASS		AC=1;AF=0
6,67E-05	0	0	0	0	0	PDGFRB|0	Acceptor L	VUS	NA	NA	1591,89	PASS		AC=2;AF=0
0	0	0	4,383	0,898	31	CAMK2A|	Acceptor G	PP3	NA	NA	3403,85	PASS		AC=2;AF=0
0	0	0	0	0	0	TCOF1|0.0	Donor Los	PP3	NA	NA	2330,86	PASS		AC=2;AF=0
0	0	0	4,94	0,931	33	DCTN4|0.0	NA	NA	NA	NA	662,69	PASS		AC=2;AF=0

6,67E-05	0	0	4,495	0,912	32	SPARC|0.0	NA	NA	NA	NA	455,04	PASS	AC=1;AF=0
0	0	0,001797	0	0	0	SPARC|0.0	Donor Gai	PP3	NA	NA	1633,7	PASS	AC=2;AF=0
0	0	0	6,684	0,966	36	GRIA1|0.0	Donor Los	BP7	NA	NA	1173,68	PASS	AC=2;AF=0
0	0	0	0	0	0	TENM2|0.	Donor Los	PP3	NA	NA	1084,07	PASS	AC=1;AF=0
0	0	0	8,632	0,995	44	SLIT3|0.01	Acceptor L	BP7	NA	NA	1122,27	PASS	AC=1;AF=0
0	0	0,0002	4,418	0,904	31	SLIT3|0.00	Acceptor L	BP7	NA	NA	547	PASS	AC=1;AF=0
3,00E-04	0	0	5,401	0,943	34	SLIT3|0.02	Acceptor G	BP7	NA	NA	1306,7	PASS	AC=2;AF=0
0	0	0	5,241	0,939	33	SLIT3|0.00	NA	NA	NA	NA	1042,01	PASS	AC=1;AF=0
1,00E-04	0	0	4,375	0,897	31	GPRIN1|0.	NA	NA	NA	NA	389,6	PASS	AC=1;AF=0
0,0016	0	0,000799	4,811	0,928	33	UNC5A|0.	Donor Gai	PP3	NA	NA	694,01	PASS	AC=1;AF=0
3,00E-04	0	0	4,404	0,902	31	PDLIM7|0	Donor Gai	BP7	NA	NA	1115,69	PASS	AC=2;AF=0
0	0	0	4,895	0,93	33	ADAMTS2	Donor Gai	BP7	NA	NA	6727,89	PASS	AC=4;AF=0
1,00E-04	0	0,0002	4,422	0,904	31	ADAMTS2	NA	NA	NA	NA	3301,12	PASS	AC=1;AF=0
0	0	0	5,869	0,955	35	ADAMTS2	Donor Los	PP3	NA	NA	604,08	PASS	AC=1;AF=0
0	0,001	0	1,99	0,304	19,18	MGAT4B|	Donor Los	PP3	NA	NA	983,72	PASS	AC=2;AF=0
0	0	0	0	0	0	FLT4|0.12	Acceptor G	VUS	NA	NA	833,05	PASS	AC=1;AF=0
0	0	0	4,364	0,895	31	GMDS|0.0	Donor Los	BP7	NA	NA	1497,69	PASS	AC=2;AF=0
0	0	0	0	0	0	CDYL|0.00	Donor Los	PP3	NA	NA	1114,9	PASS	AC=2;AF=0
0	0	0	0	0	0	CDYL|0.00	Donor Gai	VUS	NA	NA	1114,9	PASS	AC=2;AF=0
0	0	0	0	0	0	RANBP9|0	Acceptor G	PP3	NA	NA	788,84	PASS	AC=2;AF=0
0	0	0	0	0	0	KIF13A|0.	Donor Los	VUS	NA	NA	1040,01	PASS	AC=1;AF=0
0	0	0	5,163	0,937	33	KIF13A|0.	Acceptor L	PP3	NA	NA	385	PASS	AC=1;AF=0
0	0	0	0	0	0	E2F3|0.00	NA	NA	NA	NA	2968,9	PASS	AC=2;AF=0
0	0	0	6,688	0,966	36	E2F3|0.00	NA	NA	NA	NA	1404,29	PASS	AC=1;AF=0
0	0	0	0	0	0	SOX4|0.00	Donor Los	BP7	NA	NA	1780,09	PASS	AC=1;AF=0
0	0	0	0	0	0	NA	NA	NA	NA	NA	25857,6	PASS	AC=1;AF=0
0	0	0	0	0	0	NA	NA	NA	NA	NA	1909,44	PASS	AC=1;AF=0
0	0	0	5,025	0,933	33	DHX16|0.2	Acceptor G	PP3	NA	NA	1964,72	PASS	AC=2;AF=0
0	0	0	4,53	0,915	32	BAG6|0.00	Donor Los	BP7	NA	NA	1324,07	PASS	AC=1;AF=0
0	0	0	4,538	0,915	32	AGPAT1|0	Acceptor G	PP3	NA	NA	2136,7	PASS	AC=2;AF=0
6,67E-05	0	0	0	0	0	BRD2|0.00	Donor Los	PP3	NA	NA	449,45	PASS	AC=1;AF=0
0	0	0,0002	0	0	0	BRD2|0.00	Donor Los	PP3	NA	NA	1493,46	PASS	AC=1;AF=0
6,67E-05	0	0,0002	4,348	0,892	30	BRD2|0.00	NA	NA	NA	NA	485	PASS	AC=1;AF=0
0	0	0	4,406	0,902	31	BRD2|0.00	Acceptor L	BP7	NA	NA	1840	PASS	AC=1;AF=0
0	0	0	7,877	0,985	40	BRD2|0.00	Donor Los	BP7	NA	NA	3276,73	PASS	AC=2;AF=0
0	0	0	0	0	0	RING1|0.1	Acceptor L	PP3	NA	NA	856,03	PASS	AC=2;AF=0

5,00E-04	0	0	0	0	0	PFDN6|0.0	Donor Los	PP3	NA	NA		619,51	PASS	AC=1;AF=0
0	0	0	6,726	0,967	36	SYNGAP1|	Acceptor L	BP7	hiConfDeN		1	3036	PASS	AC=1;AF=0
0	0	0	4,437	0,906	31	ITPR3|0.0	Acceptor G	BP7	NA	NA		1404	PASS	AC=1;AF=0
0	0	0	4,37	0,896	31	ITPR3|0.0	Donor Gai	BP7	NA	NA		861,03	PASS	AC=1;AF=0
0	0	0,0002	4,486	0,911	32	GRM4|0.0	Acceptor G	BP7	NA	NA		992	PASS	AC=1;AF=0
0	0	0	4,805	0,927	33	NUDT3|0.	Acceptor L	BP7	NA	NA		1991,68	PASS	AC=2;AF=0
6,67E-05	0	0,0002	4,526	0,915	32	RPS10-NU	Acceptor L	BP7	NA	NA		1550,73	PASS	AC=2;AF=0
0	0	0	0	0	0	SNRPC|0.0	NA	NA	NA	NA		1022,67	PASS	AC=2;AF=0
0	0	0	7,512	0,979	38	SCUBE3|0.	Acceptor L	VUS	NA	NA		725,01	PASS	AC=1;AF=0
0	0	0,0002	4,684	0,923	32	SCUBE3|0.	Donor Los	BP7	NA	NA		1672,86	PASS	AC=2;AF=0
0	0	0	0	0	0	NA	NA	NA	loConfDeN	NA		232,94	PASS	AC=1;AF=0
0	0	0	7,196	0,974	37	DEF6|0.00	NA	NA	NA	NA		1963,68	PASS	AC=2;AF=0
0	0	0	0	0	0	RPL10A|0.	Acceptor G	PP3	NA	NA		68,18	PASS	AC=1;AF=0
0	0	0	0	0	0	NA	NA	NA	NA	NA		169,95	PASS	AC=1;AF=0
0	0	0	0	0	0	NA	NA	NA	NA	NA		1354,59	PASS	AC=1;AF=0
0	0	0	4,643	0,922	32	FKBP5|0.0	Acceptor L	PP3	NA	NA		695,7	PASS	AC=2;AF=0
0	0	0	4,582	0,918	32	BRPF3|0.0	NA	NA	NA	NA		276,02	PASS	AC=1;AF=0
0	0	0	4,505	0,913	32	MDGA1|0	Acceptor G	BP7	NA	NA		684,14	PASS	AC=1;AF=0
6,67E-05	0	0	4,492	0,912	32	TFEB|0.00	NA	NA	NA	NA		4280,68	PASS	AC=2;AF=0
3,00E-04	0	0	4,508	0,913	32	TFEB|0.03	Acceptor G	BP7	NA	NA		620	PASS	AC=1;AF=0
0	0	0	5,15	0,936	33	UBR2|0.31	Acceptor L	PP3	NA	NA		1890,71	PASS	AC=2;AF=0
0,0031	0,0031	0,001797	0	0	0	NA	NA	NA	NA	NA		2893,62	PASS	AC=2;AF=0
0	0	0	4,498	0,912	32	RP11-444E	NA	NA	NA	NA		3964,68	PASS	AC=2;AF=0
0	0	0	4,432	0,905	31	RUNX2|0.	NA	NA	NA	NA		4151,33	PASS	AC=2;AF=0
0	0	0,0002	4,445	0,907	31	TRAM2|0.	Acceptor L	BP7	NA	NA		335	PASS	AC=1;AF=0
0	0	0	0	0	0	TRAM2|0.	Acceptor G	PP3	NA	NA		1428,64	PASS	AC=2;AF=0
0	0	0	0	0	0	NA	NA	NA	NA	NA		238,98	PASS	AC=1;AF=0
0	0	0	0	0	0	ELOVL5|0.	NA	NA	NA	NA		37114,9	PASS	AC=1;AF=0
0	0	0	4,379	0,898	31	DST|0.00|	NA	NA	NA	NA		530,01	PASS	AC=1;AF=0
0	0	0	4,605	0,92	32	DST|0.06|	Acceptor G	BP7	NA	NA		2015,69	PASS	AC=2;AF=0
0	0	0	0,748	0,12	8,892	DST|0.00|	NA	NA	NA	NA		2029,11	PASS	AC=1;AF=0
6,66E-05	0	0	0	0	0	DST|0.00|	NA	NA	NA	NA		2465,94	PASS	AC=1;AF=0
6,66E-05	0	0	4,541	0,916	32	DST|0.00|	Donor Los	BP7	NA	NA		845	PASS	AC=1;AF=0
0	0	0	4,401	0,901	31	DST|0.00|	Acceptor L	BP7	NA	NA		240,73	PASS	AC=2;AF=0
0	0	0,0002	0	0	0	DST|0.47|	Acceptor G	PP3	NA	NA		879,02	PASS	AC=1;AF=0
0	0	0	4,484	0,911	32	PHF3|0.00	NA	NA	NA	NA		1514	PASS	AC=1;AF=0

0	0	0	4,48	0,911	32	FAM135A|Acceptor L	BP7	NA	NA	309,11	PASS	AC=2;AF=0
0	0	0	0	0	0	B3GAT2|0	Acceptor G	BP7	NA	NA	2098,97	PASS	AC=2;AF=0
0	0	0	4,418	0,904	31	RIMS1|0.0	NA	NA	NA	NA	522,02	PASS	AC=1;AF=0
0	0	0	4,415	0,903	31	RIMS1|0.0	Donor Los	BP7	NA	NA	2244	PASS	AC=1;AF=0
0	0	0	4,379	0,898	31	COL12A1|	NA	NA	NA	NA	1372,68	PASS	AC=2;AF=0
4,00E-04	0	0,000399	0	0	0	COL12A1|	Donor Gai	VUS	NA	NA	1385,99	PASS	AC=1;AF=0
0	0	0	4,662	0,922	32	COL12A1|	Donor Gai	BP7	NA	NA	1421	PASS	AC=1;AF=0
0	0	0	0	0	0	SNAP91|0	Donor Gai	VUS	NA	NA	274,7	PASS	AC=2;AF=0
0	0	0	4,463	0,909	32	PNISR|0.0	NA	NA	NA	NA	216	PASS	AC=1;AF=0
0	0	0	4,787	0,927	33	PRDM1|0.	Donor Gai	BP7	NA	NA	1275,09	PASS	AC=1;AF=0
0	0	0	0	0	0	CDK19|0.1	Acceptor G	VUS	NA	NA	2724,22	PASS	AC=3;AF=0
0	0	0	4,361	0,895	31	FYN|0.03|	Acceptor G	BP7	NA	NA	2485,68	PASS	AC=2;AF=0
0	0	0	4,585	0,919	32	VGLL2|0.0	NA	NA	NA	NA	1825,69	PASS	AC=2;AF=0
0	0	0	6,905	0,969	36	SLC35F1|0	NA	NA	NA	NA	268	PASS	AC=1;AF=0
0	0	0	0	0	0	KIAA0408|	Donor Los	PP3	NA	NA	737,71	PASS	AC=1;AF=0
0	0	0	0	0	0	NA	NA	NA	NA	NA	298,1	PASS	AC=2;AF=0
0	0	0,000399	4,535	0,915	32	PTPRK|0.0	Donor Los	PP3	NA	NA	321,05	PASS	AC=1;AF=0
0	0	0,0002	4,396	0,9	31	EPB41L2|0	Acceptor L	BP7	NA	NA	886,71	PASS	AC=2;AF=0
0	0	0	4,542	0,916	32	EPB41L2|0	NA	NA	NA	NA	634,72	PASS	AC=2;AF=0
6,66E-05	0	0	4,373	0,897	31	PDE7B|0.0	Donor Los	BP7	NA	NA	491	PASS	AC=1;AF=0
0	0	0	0	0	0	TNFAIP3|0	Acceptor G	VUS	NA	NA	695,03	PASS	AC=1;AF=0
0	0	0	0	0	0	HECA|0.00	NA	NA	NA	NA	428,93	PASS	AC=1;AF=0
0	0	0	0	0	0	NA	NA	NA	NA	NA	419,67	PASS	AC=1;AF=0
3,00E-04	0	0	4,614	0,92	32	STXBP5|0.	Acceptor L	VUS	NA	NA	340,68	PASS	AC=2;AF=0
0	0	0	4,371	0,896	31	STXBP5|0.	Donor Los	BP7	NA	NA	1098,77	PASS	AC=1;AF=0
0	0	0	0	0	0	SCAF8|0.0	Donor Gai	VUS	NA	NA	2114,24	PASS	AC=3;AF=0
4,00E-04	0	0	0	0	0	ARID1B|0.	NA	NA	NA	NA	51004,2	PASS	AC=2;AF=0
1,00E-04	0	0	5,604	0,949	34	TCP1|0.00	Donor Gai	PP3	NA	NA	1877,18	PASS	AC=3;AF=0
6,67E-05	0	0,002995	0	0	0	IGF2R|0.0	Donor Los	PP3	NA	NA	713,71	PASS	AC=2;AF=0
0	0	0	4,526	0,915	32	THBS2|0.0	NA	NA	NA	NA	578	PASS	AC=1;AF=0
1,00E-04	0	0,000599	4,376	0,897	31	THBS2|0.0	Donor Los	BP7	NA	NA	1214,72	PASS	AC=2;AF=0
0	0	0	5,812	0,954	35	GET4|0.66	Acceptor L	PP3	NA	NA	437	PASS	AC=1;AF=0
6,67E-05	0	0,001597	0	0	0	GET4|0.00	Donor Los	PP3	NA	NA	609,08	PASS	AC=1;AF=0
0	0	0	4,397	0,901	31	MAFK|0.0	NA	NA	NA	NA	1288,07	PASS	AC=1;AF=0
0	0	0	0	0	0	ELFN1|0.0	NA	NA	NA	NA	2286,17	PASS	AC=2;AF=0
0	0	0,000399	4,526	0,915	32	EIF3B|0.00	Donor Los	BP7	NA	NA	739,14	PASS	AC=1;AF=0

0	0	0	5,361	0,942	34	CARD11|0	Donor Gai	PP3	NA	NA	1333	PASS	AC=1;AF=0
0	0	0	4,472	0,91	32	FOXK1|0.0	NA	NA	NA	NA	1938,94	PASS	AC=2;AF=0
0	0	0	0	0	0	NA	NA	NA	NA	NA	3400,29	PASS	AC=1;AF=0
2,00E-04	0	0	4,376	0,897	31	TNRC18|0	Acceptor G	VUS	NA	NA	1477,03	PASS	AC=1;AF=0
0	0,001	0,001797	2,008	0,308	19,32	FSCN1|0.0	Donor Los	PP3	NA	NA	716,01	PASS	AC=1;AF=0
6,68E-05	0,001	0,0002	4,397	0,9	31	FSCN1|0.0	NA	NA	NA	NA	664	PASS	AC=1;AF=0
0	0	0	0	0	0	CYTH3|0.0	Donor Los	VUS	NA	NA	2172,68	PASS	AC=2;AF=0
0	0	0	0	0	0	THSD7A|0	Donor Los	PP3	NA	NA	1765	PASS	AC=1;AF=0
0,0055	0,001	0	0	0	0	MEOX2|0.	NA	NA	NA	NA	1631,97	PASS	AC=1;AF=0
0,0055	0,001	0	0	0	0	MEOX2|0.	NA	NA	NA	NA	1634,98	PASS	AC=1;AF=0
0	0	0	0	0	0	NA	NA	NA	NA	NA	1407,63	PASS	AC=2;AF=0
0	0	0	5,091	0,935	33	HDAC9|0.	Acceptor G	PP3	NA	NA	1129,78	PASS	AC=2;AF=0
0	0	0	5,524	0,946	34	SP4|0.00|	Donor Gai	VUS	NA	NA	2259,08	PASS	AC=1;AF=0
0	0	0	5,254	0,939	33	RAPGEF5|	Donor Los	PP3	NA	NA	243,74	PASS	AC=2;AF=0
0	0	0	0	0	0	MPP6|0.0	Donor Gai	PP3	NA	NA	795,74	PASS	AC=2;AF=0
0	0	0	4,577	0,918	32	MPP6|0.0	Donor Los	BP7	NA	NA	977,68	PASS	AC=2;AF=0
0	0	0	0	0	0	HNRNPA2	Acceptor G	VUS	NA	NA	1647,69	PASS	AC=2;AF=0
0	0	0	4,419	0,904	31	TBX20|0.0	Acceptor L	BP7	NA	NA	1484,7	PASS	AC=2;AF=0
0	0	0	4,595	0,919	32	CDK13|0.0	NA	NA	NA	NA	1137,96	PASS	AC=2;AF=0
0	0	0	0	0	0	GLI3|0.00	Donor Gai	VUS	NA	NA	1200,16	PASS	AC=2;AF=0
0	0	0	4,49	0,912	32	HECW1|0.	Acceptor L	BP7	NA	NA	813,7	PASS	AC=2;AF=0
1,00E-04	0	0,0002	0,514	0,093	6,654	GCK|0.00|	Donor Gai	BP7	NA	NA	576,71	PASS	AC=1;AF=0
0	0	0	8,22	0,99	41	ZMIZ2|0.0	Acceptor L	PP3	NA	NA	569,04	PASS	AC=1;AF=0
0	0	0	8,379	0,992	42	TNS3|0.00	NA	NA	NA	NA	482	PASS	AC=1;AF=0
0	0	0	4,61	0,92	32	VWC2|0.0	Donor Gai	BP7	NA	NA	316,36	PASS	AC=1;AF=0
0	0	0	0	0	0	NA	NA	NA	NA	NA	975,02	PASS	AC=1;AF=0
6,66E-05	0	0	4,461	0,909	32	TMEM248	NA	NA	NA	NA	1757,69	PASS	AC=2;AF=0
0	0	0	4,359	0,894	31	AUTS2|0.0	NA	NA	NA	NA	2549,75	PASS	AC=2;AF=0
0	0	0	4,77	0,926	33	CLIP2|0.68	Acceptor G	PP3	NA	NA	1325,27	PASS	AC=1;AF=0
0	0	0,0002	0	0	0	GTF2IRD1|	Donor Gai	PP3	NA	NA	586,77	PASS	AC=2;AF=0
5,00E-04	0,002	0,0002	0	0	0	HIP1|0.01	Acceptor L	VUS	NA	NA	529,69	PASS	AC=2;AF=0
6,67E-05	0	0,001997	4,377	0,897	31	HIP1|0.00	NA	NA	NA	NA	962,69	PASS	AC=2;AF=0
0	0	0	4,436	0,906	31	HGF|0.00|	NA	NA	NA	NA	1867,69	PASS	AC=2;AF=0
0	0	0	1,624	0,238	16,53	CACNA2D1	NA	NA	NA	NA	1397,69	PASS	AC=2;AF=0
0	0	0	4,36	0,895	31	PCLO|0.03	Acceptor G	BP7	NA	NA	403,68	PASS	AC=2;AF=0
6,67E-05	0	0	4,352	0,893	30	GRM3|0.0	NA	NA	NA	NA	3249,68	PASS	AC=2;AF=0

0	0	0	0	0	0	COL1A2|0	Donor Gai	BP7	NA	NA	1232,62	PASS	AC=2;AF=0
0	0	0	4,538	0,915	32	COL1A2|0	Donor Los	BP7	NA	NA	3499,69	PASS	AC=2;AF=0
6,66E-05	0	0,0002	4,409	0,902	31	COL1A2|0	NA	NA	NA	NA	534,02	PASS	AC=1;AF=0
9,00E-04	0	0	4,628	0,921	32	COL1A2|0	Donor Los	BP7	NA	NA	4816,8	PASS	AC=2;AF=0
7,09E-05	0	0	0	0	0	DLX6|0.00	NA	NA	NA	NA	596,33	PASS	AC=1;AF=0
7,21E-05	0	0	0	0	0	DLX6|0.00	NA	NA	NA	NA	596,36	PASS	AC=1;AF=0
0	0,001	0,0002	4,43	0,905	31	LMTK2|0.0	NA	NA	NA	NA	1959,02	PASS	AC=1;AF=0
0	0	0,0002	0	0	0	TRRAP|0.0	Donor Gai	PP3	NA	NA	560	PASS	AC=1;AF=0
0	0	0	4,563	0,917	32	ACTL6B|0.	Donor Gai	BP7	NA	NA	1740,68	PASS	AC=2;AF=0
6,68E-05	0	0	0	0	0	NA	NA	NA	NA	NA	1269,39	PASS	AC=1;AF=0
2,00E-04	0	0	0	0	0	SRRT|0.00	Donor Gai	PP3	NA	NA	604,15	PASS	AC=1;AF=0
0	0	0	4,548	0,916	32	CUX1|0.01	Acceptor G	BP7	NA	NA	305,94	PASS	AC=2;AF=0
0	0	0	4,389	0,899	31	RELN|0.00	Acceptor L	BP7	NA	NA	1574,71	PASS	AC=2;AF=0
0	0	0	4,614	0,92	32	KMT2E|0.	Acceptor G	VUS	NA	NA	463,11	PASS	AC=1;AF=0
0	0	0	0	0	0	KMT2E|0.	NA	NA	NA	NA	4385,37	PASS	AC=1;AF=0
0	0	0	6,931	0,97	36	SRPK2|0.0	Donor Gai	BP7	NA	NA	1067,02	PASS	AC=3;AF=0
0	0	0	0	0	0	SRPK2|0.0	Donor Gai	PP3	NA	NA	1163,69	PASS	AC=2;AF=0
6,67E-05	0	0	0	0	0	FOXP2|0.0	Acceptor L	BP7	NA	NA	2055,11	PASS	AC=2;AF=0
0	0	0	5,424	0,944	34	PTPRZ1|0.	Donor Los	PP3	NA	NA	1519,02	PASS	AC=1;AF=0
0,006	0,0102	0,000799	0	0	0	LRRC4|0.0	Acceptor L	VUS	NA	NA	375,85	PASS	AC=2;AF=0
0,0011	0,0031	0,000998	4,378	0,898	31	FLNC|0.00	Donor Gai	BP7	NA	NA	3060,7	PASS	AC=2;AF=0
0	0	0	4,511	0,913	32	FLNC|0.00	NA	NA	NA	NA	379,05	PASS	AC=1;AF=0
0	0	0	4,514	0,914	32	FLNC|0.01	Acceptor G	BP7	NA	NA	1186,01	PASS	AC=1;AF=0
0	0	0	4,409	0,902	31	FLNC|0.00	Acceptor L	BP7	NA	NA	2624	PASS	AC=1;AF=0
0	0	0	4,862	0,929	33	AHCYL2|0.	Donor Gai	BP7	NA	NA	755,7	PASS	AC=2;AF=0
0	0	0	0	0	0	PLXNA4|0	Donor Los	PP3	NA	NA	1465,73	PASS	AC=2;AF=0
0	0	0	0	0	0	PLXNA4|0	Donor Gai	BP7	NA	NA	231,96	PASS	AC=1;AF=0
0	0	0,0002	4,361	0,895	31	PLXNA4|0	Donor Los	BP7	NA	NA	550	PASS	AC=1;AF=0
0	0	0	4,528	0,915	32	CALD1|0.0	NA	NA	NA	NA	1986,68	PASS	AC=2;AF=0
0	0	0	4,662	0,922	32	CALD1|0.0	Acceptor L	VUS	NA	NA	1489,71	PASS	AC=2;AF=0
0	0	0	6,147	0,959	35	NUP205|0	Acceptor G	BP7	NA	NA	274	PASS	AC=1;AF=0
0	0	0	0	0	0	NUP205|0	Acceptor L	BP7	NA	NA	1880,66	PASS	AC=2;AF=0
0	0	0	5,001	0,932	33	NUP205|0	Donor Los	BP7	NA	NA	808,73	PASS	AC=2;AF=0
0	0	0	0	0	0	NUP205|0	Donor Los	PP3	NA	NA	367,94	PASS	AC=1;AF=0
0	0	0	4,641	0,921	32	DGKI|0.00	Donor Los	BP7	NA	NA	587	PASS	AC=1;AF=0
0	0	0	4,64	0,921	32	KIAA1549|	Donor Los	BP7	NA	NA	743,72	PASS	AC=2;AF=0

0	0	0	0	0	0	KIAA1549|Donor Los	PP3	NA	NA	493,01	PASS	AC=1;AF=0
0	0	0	4,367	0,896	31	HIPK2|0.0 Donor Los	BP7	NA	NA	506,01	PASS	AC=1;AF=0
0	0	0	5,677	0,95	34	FAM131B|Donor Los	PP3	NA	NA	1841,69	PASS	AC=2;AF=0
2,00E-04	0	0,000799	4,429	0,905	31	KCNH2|0. NA	NA	NA	NA	628,7	PASS	AC=2;AF=0
0	0	0	4,476	0,91	32	SLC4A2|0. Acceptor G	PP3	NA	NA	2575,68	PASS	AC=2;AF=0
0	0	0,000599	0	0	0	ACTR3B|0 Acceptor L	VUS	NA	NA	492,01	PASS	AC=1;AF=0
0	0	0	5,717	0,951	34	CSMD1|0. Donor Los	PP3	NA	NA	308,15	PASS	AC=1;AF=0
0	0	0	4,755	0,926	32	DLC1|0.00 NA	NA	NA	NA	783	PASS	AC=1;AF=0
0	0	0	4,453	0,908	32	INTS10|0. Donor Gai	BP7	NA	NA	414	PASS	AC=1;AF=0
0	0	0	4,533	0,915	32	XPO7|0.01 Acceptor G	BP7	NA	NA	2569,69	PASS	AC=2;AF=0
0	0	0	4,56	0,917	32	DMTN|0.0 NA	NA	NA	NA	1654,68	PASS	AC=2;AF=0
0	0	0	0	0	0	NA NA	NA	NA	NA	1291,59	PASS	AC=1;AF=0
0	0	0	8,167	0,99	41	EBF2|0.00 Donor Gai	BP7	NA	NA	1507	PASS	AC=1;AF=0
0	0	0	5,566	0,947	34	PTK2B|0.0 Donor Gai	PP3	NA	NA	865,69	PASS	AC=2;AF=0
3,00E-04	0	0	0	0	0	PTK2B|0.9 Acceptor L	PP3	NA	NA	2459,64	PASS	AC=2;AF=0
0	0	0	4,546	0,916	32	PTK2B|0.0 NA	NA	NA	NA	1688,69	PASS	AC=2;AF=0
0	0	0	0	0	0	DUSP4|0.0 NA	NA	NA	NA	2272,67	PASS	AC=2;AF=0
0	0	0	0	0	0	NA NA	NA	NA	NA	2047,01	PASS	AC=1;AF=0
0	0	0	4,967	0,932	33	UNC5D|0. Donor Los	BP7	NA	NA	789	PASS	AC=1;AF=0
0	0	0	4,416	0,903	31	FGFR1|0.0 NA	NA	NA	NA	3537,69	PASS	AC=2;AF=0
0	0	0	0	0	0	FGFR1|0.0 Donor Los	PP3	NA	NA	801,61	PASS	AC=1;AF=0
0	0	0	0	0	0	FGFR1|0.0 Donor Los	PP3	NA	NA	1342,95	PASS	AC=2;AF=0
6,67E-05	0	0	4,732	0,925	32	SFRP1|0.0 NA	NA	NA	NA	908,04	PASS	AC=1;AF=0
0	0	0,0002	4,622	0,921	32	SFRP1|0.0 NA	NA	NA	NA	1217,07	PASS	AC=1;AF=0
1,00E-04	0	0,0002	4,504	0,913	32	ANK1|0.00 NA	NA	NA	NA	2263,01	PASS	AC=1;AF=0
0	0	0	4,531	0,915	32	ANK1|0.00 NA	NA	NA	NA	918	PASS	AC=1;AF=0
0	0	0	5,048	0,934	33	KAT6A|0.0 Donor Gai	PP3	hiConfDeN	0,998482	381,01	PASS	AC=1;AF=0
0	0	0	4,466	0,909	32	VDAC3|0. Donor Los	BP7	NA	NA	518,15	PASS	AC=1;AF=0
0	0	0,000399	0	0	0	PRKDC|0.0 Acceptor L	VUS	NA	NA	1110,69	PASS	AC=2;AF=0
0	0	0	4,539	0,916	32	RB1CC1|0. Acceptor L	PP3	NA	NA	180,1	PASS	AC=1;AF=0
0	0	0	4,388	0,899	31	SOX17|0.0 NA	NA	NA	NA	2198,01	PASS	AC=1;AF=0
0	0	0	0	0	0	LYN|0.00| NA	NA	NA	NA	1751,63	PASS	AC=2;AF=0
0	0	0	6,571	0,965	36	MOS|0.00 NA	NA	hiConfDeN	0,999997	1495,14	PASS	AC=1;AF=0
0	0	0	5,369	0,942	34	GGH|0.00 Donor Los	VUS	NA	NA	645,21	PASS	AC=1;AF=0
0	0	0	8,918	0,997	46	NCOA2|0. Donor Los	VUS	NA	NA	466	PASS	AC=1;AF=0
0	0	0	0	0	0	JPH1|0.01 Acceptor L	PP3	NA	NA	435	PASS	AC=2;AF=0

0	0	0	0	0	0	NA	NA	NA	NA	NA	216,94	PASS	AC=1;AF=0
2,00E-04	0	0,0002	4,503	0,913	32	RUNX1T1|	Donor Los	BP7	NA	NA	462,01	PASS	AC=1;AF=0
0	0	0	0	0	0	NA	NA	NA	NA	NA	480,62	PASS	AC=2;AF=0
0	0	0	5,261	0,939	33	RUNX1T1|	Donor Los	PP3	NA	NA	1214,37	PASS	AC=2;AF=0
0	0	0,000399	0	0	0	CCNE2|0.0	Donor Los	VUS	NA	NA	710,72	PASS	AC=2;AF=0
0	0	0	4,515	0,914	32	GDF6|0.00	NA	NA	NA	NA	1296,89	PASS	AC=2;AF=0
1,00E-04	0,001	0,008986	0	0	0	YWHAZ|0.	Donor Los	PP3	NA	NA	2847,23	PASS	AC=2;AF=0
0	0	0	0	0	0	RIMS2|0.0	Donor Gai	VUS	NA	NA	556,83	PASS	AC=2;AF=0
1,00E-04	0	0,000399	0	0	0	RIMS2|0.0	NA	NA	NA	NA	899,57	PASS	AC=1;AF=0
0	0	0	0	0	0	OXR1|0.76	Acceptor L	PP3	NA	NA	24,52	PASS	AC=1;AF=0
0	0	0	8,229	0,99	41	EXT1|0.00	Acceptor L	BP7	NA	NA	295	PASS	AC=1;AF=0
0	0	0	4,387	0,899	31	EXT1|0.01	Acceptor G	BP7	NA	NA	1354	PASS	AC=1;AF=0
0	0	0	1,215	0,178	13,75	ATAD2|0.0	Acceptor L	BP7	NA	NA	166,2	PASS	AC=1;AF=0
0	0	0	0	0	0	ATAD2|0.0	Donor Los	VUS	NA	NA	582,97	PASS	AC=2;AF=0
2,00E-04	0,001	0,000399	4,366	0,896	31	TRIB1|0.0	NA	NA	NA	NA	2013,97	PASS	AC=2;AF=0
0	0	0	5,964	0,956	35	ASAP1|0.0	Donor Los	BP7	NA	NA	1494,68	PASS	AC=2;AF=0
0	0	0	4,513	0,913	32	KCNQ3|0.	Acceptor L	PP3	NA	NA	767,05	PASS	AC=1;AF=0
0	0	0	6,208	0,96	35	KCNQ3|0.	Donor Los	PP3	NA	NA	1320,36	PASS	AC=3;AF=0
6,67E-05	0	0	0	0	0	PTK2|0.00	Acceptor L	PP3	NA	NA	1008,68	PASS	AC=2;AF=0
5,00E-04	0,0031	0,000799	2,526	0,418	22,5	PTK2|0.00	Donor Los	BP7	NA	NA	284,04	PASS	AC=1;AF=0
0	0	0	0	0	0	ARC|0.00|	NA	NA	NA	NA	4740,63	PASS	AC=2;AF=0
0	0	0	0	0	0	FAM83H|0	Acceptor G	PP3	NA	NA	2340,63	PASS	AC=2;AF=0
1,00E-04	0	0	0	0	0	SCRIB|0.2	Acceptor G	PP3	NA	NA	879,18	PASS	AC=1;AF=0
0	0	0,001198	5,031	0,933	33	SCRIB|0.0	NA	NA	NA	NA	953,12	PASS	AC=1;AF=0
0	0	0	0	0	0	SCRIB|0.1	Acceptor G	VUS	NA	NA	1174	PASS	AC=1;AF=0
6,68E-05	0,001	0,0002	5,121	0,935	33	PUF60|0.0	NA	NA	NA	NA	744,26	PASS	AC=2;AF=0
0	0	0	4,376	0,897	31	PTPRD|0.0	Acceptor L	BP7	NA	NA	1529,68	PASS	AC=2;AF=0
0	0	0	10,02	0,999	53	PTPRD|0.2	Acceptor L	PP3	NA	NA	295,05	PASS	AC=1;AF=0
0	0	0	0	0	0	MLLT3|0.0	Donor Los	PP3	NA	NA	745,03	PASS	AC=2;AF=0
5,00E-04	0	0	4,361	0,895	31	NA	NA	NA	NA	NA	1295	PASS	AC=1;AF=0
0	0	0	4,514	0,914	32	NA	NA	NA	NA	NA	2063,68	PASS	AC=2;AF=0
0	0	0	8,935	0,997	46	UBAP1|0.0	Donor Gai	VUS	NA	NA	591,68	PASS	AC=2;AF=0
0	0	0	0	0	0	NA	NA	NA	NA	NA	297,94	PASS	AC=1;AF=0
0	0	0,0002	4,545	0,916	32	VCP|0.00|	Donor Los	PP3	NA	NA	1697,01	PASS	AC=1;AF=0
0	0	0	5,529	0,946	34	TLN1|0.59	Acceptor L	PP3	NA	NA	690	PASS	AC=1;AF=0
0	0	0	4,535	0,915	32	CLTA|0.00	Donor Gai	BP7	NA	NA	539,31	PASS	AC=2;AF=0

0	0	0	4,432	0,905	31	PAX5|0.00	Donor Los	BP7	NA	NA	1675,69	PASS	AC=2;AF=0
0	0	0	8,427	0,993	43	RP11-613	NA	NA	NA	NA	1575,45	PASS	AC=1;AF=0
0	0	0	5,373	0,942	34	APBA1|0.7	Acceptor G	PP3	NA	NA	1561	PASS	AC=1;AF=0
6,66E-05	0	0,000399	4,378	0,898	31	GDA|0.00	Acceptor L	BP7	NA	NA	272	PASS	AC=1;AF=0
0	0	0,000399	-0,062	0,037	0,995	GDA|0.00	Acceptor L	BP7	NA	NA	691,71	PASS	AC=2;AF=0
6,67E-05	0	0,000399	0	0	0	GDA|0.00	Donor Los	BP7	NA	NA	947,73	PASS	AC=2;AF=0
0	0	0	2,353	0,38	21,9	ZFAND5|0	Donor Los	PP3	NA	NA	342,2	PASS	AC=1;AF=0
0	0	0	4,411	0,903	31	RORB|0.0	NA	NA	NA	NA	808,7	PASS	AC=2;AF=0
6,66E-05	0	0,0002	4,518	0,914	32	TLE1|0.00	Donor Los	BP7	NA	NA	1311,68	PASS	AC=2;AF=0
0	0	0	4,507	0,913	32	TLE1|0.00	Donor Gai	PP3	NA	NA	314	PASS	AC=1;AF=0
0	0	0	0	0	0	TLE1|0.11	Acceptor G	VUS	NA	NA	2177,73	PASS	AC=2;AF=0
0	0	0	0	0	0	TLE1|0.05	Acceptor L	PP3	NA	NA	677,13	PASS	AC=1;AF=0
0	0	0,000399	0	0	0	DAPK1|0.0	Acceptor L	VUS	NA	NA	2834,93	PASS	AC=2;AF=0
1,00E-04	0	0	0	0	0	SEMA4D|0	Acceptor G	PP3	NA	NA	918,78	PASS	AC=2;AF=0
0	0	0,0002	4,57	0,918	32	BICD2|0.0	NA	NA	NA	NA	464,12	PASS	AC=1;AF=0
0	0	0	4,53	0,915	32	WNK2|0.0	Donor Gai	BP7	NA	NA	550,69	PASS	AC=2;AF=0
0	0	0	4,378	0,898	31	FAM120A	Acceptor G	BP7	NA	NA	5620,82	PASS	AC=2;AF=0
0	0	0	0	0	0	PHF2|0.00	NA	NA	NA	NA	1650,14	PASS	AC=1;AF=0
0	0	0	0	0	0	NA	NA	NA	NA	NA	5322,95	PASS	AC=1;AF=0
0	0	0,000599	0	0	0	NR4A3|0.0	Acceptor L	VUS	NA	NA	307,73	PASS	AC=2;AF=0
0	0	0	0	0	0	TEX10|0.1	Acceptor L	PP3	NA	NA	243,98	PASS	AC=2;AF=0
0	0	0,000399	4,442	0,906	31	SMC2|0.0	Acceptor G	BP7	NA	NA	441,01	PASS	AC=1;AF=0
0	0	0	7,395	0,977	38	EPB41L4B|	NA	NA	NA	NA	612,45	PASS	AC=3;AF=0
0	0	0	4,931	0,931	33	SVEP1|0.0	Donor Los	BP7	NA	NA	1008,7	PASS	AC=2;AF=0
0	0	0	0	0	0	SVEP1|0.0	NA	NA	NA	NA	2748,62	PASS	AC=2;AF=0
2,00E-04	0	0	0	0	0	SVEP1|0.0	Acceptor L	PP3	NA	NA	388	PASS	AC=1;AF=0
0	0	0	4,358	0,894	30	SVEP1|0.0	NA	NA	NA	NA	2921,84	PASS	AC=2;AF=0
0	0	0	5,11	0,935	33	COL27A1|	Donor Los	VUS	NA	NA	2065,69	PASS	AC=2;AF=0
0	0	0,000998	0	0	0	COL27A1|	Acceptor G	VUS	NA	NA	875	PASS	AC=1;AF=0
6,68E-05	0	0,001797	5,077	0,934	33	COL27A1|	Donor Los	VUS	NA	NA	328,08	PASS	AC=1;AF=0
0	0	0	0	0	0	COL27A1|	Donor Los	VUS	NA	NA	487	PASS	AC=1;AF=0
0	0	0	0	0	0	COL27A1|	Acceptor G	VUS	NA	NA	324,01	PASS	AC=1;AF=0
0	0	0	4,46	0,909	32	DAB2IP|0.	NA	NA	NA	NA	508,02	PASS	AC=1;AF=0
0	0	0	4,753	0,926	32	RABGAP1|	Donor Gai	BP7	NA	NA	2061,8	PASS	AC=2;AF=0
0	0	0	0	0	0	SCAI|0.00	Acceptor L	PP3	NA	NA	680,81	PASS	AC=2;AF=0
6,67E-05	0	0	0	0	0	PPP6C|0.0	NA	NA	NA	NA	1184,06	PASS	AC=1;AF=0

0	0	0	0	0	0	GAPVD1|0	Acceptor G	VUS	NA	NA	916,68	PASS	AC=2;AF=0
0	0	0	4,406	0,902	31	GAPVD1|0	Acceptor G	BP7	NA	NA	719	PASS	AC=1;AF=0
6,66E-05	0	0,0002	4,771	0,926	33	GAPVD1|0	Acceptor G	PP3	NA	NA	3386,88	PASS	AC=2;AF=0
0	0	0,000998	6,79	0,968	36	LMX1B|0.	Donor Los	VUS	NA	NA	458,03	PASS	AC=2;AF=0
0	0	0,002995	0	0	0	LMX1B|0.	Donor Los	PP3	NA	NA	1178,24	PASS	AC=1;AF=0
0	0	0,000799	0	0	0	GOLGA2|0	Acceptor L	PP3	NA	NA	1295,68	PASS	AC=2;AF=0
0	0	0	4,382	0,898	31	SPTAN1|0	NA	NA	NA	NA	716,01	PASS	AC=1;AF=0
0	0	0	4,556	0,917	32	SPTAN1|0	NA	NA	NA	NA	4677,69	PASS	AC=2;AF=0
0	0	0	5,736	0,952	34	SPTAN1|0	Donor Gai	PP3	NA	NA	3630,94	PASS	AC=2;AF=0
0	0	0	4,688	0,923	32	ZER1|0.02	Acceptor G	BP7	NA	NA	1845,99	PASS	AC=2;AF=0
0	0	0	5,267	0,939	33	NUP188|0	NA	NA	NA	NA	691	PASS	AC=1;AF=0
0	0	0	4,524	0,914	32	NUP188|0	Acceptor G	BP7	NA	NA	638	PASS	AC=1;AF=0
6,66E-05	0	0	0	0	0	NA	NA	NA	NA	NA	519,62	PASS	AC=2;AF=0
0	0,001	0	4,429	0,905	31	NUP188|0	Acceptor L	BP7	NA	NA	1444,68	PASS	AC=2;AF=0
0	0	0	0	0	0	FNBP1|0.0	Acceptor L	BP7	NA	NA	160,94	PASS	AC=1;AF=0
1,00E-04	0	0	0	0	0	RAPGEF1|	NA	NA	NA	NA	1100,88	PASS	AC=2;AF=0
0	0	0	4,416	0,903	31	NTNG2|0.	NA	NA	NA	NA	924,99	PASS	AC=1;AF=0
0	0	0	0	0	0	SETX|0.00	Donor Los	PP3	NA	NA	1373,1	PASS	AC=1;AF=0
0	0	0	4,396	0,9	31	TSC1|0.00	NA	NA	NA	NA	943,01	PASS	AC=1;AF=0
0	0	0	0	0	0	WDR5|0.0	Acceptor L	VUS	NA	NA	326,01	PASS	AC=1;AF=0
0	0	0	4,653	0,922	32	COL5A1|0	Acceptor L	VUS	NA	NA	585	PASS	AC=1;AF=0
3,00E-04	0,001	0,000399	4,392	0,9	31	COL5A1|0	Donor Gai	BP7	NA	NA	1345,69	PASS	AC=2;AF=0
0	0	0	4,477	0,91	32	COL5A1|0	Acceptor G	BP7	NA	NA	1269	PASS	AC=1;AF=0
0	0	0	4,364	0,895	31	OLFM1|0.	NA	NA	NA	NA	1362	PASS	AC=1;AF=0
0	0	0	0	0	0	RABL6|0.2	Acceptor G	PP3	NA	NA	701	PASS	AC=1;AF=0
0	0	0	4,387	0,899	31	RABL6|0.0	Acceptor G	BP7	NA	NA	2029,01	PASS	AC=1;AF=0
6,71E-05	0	0	0	0	0	NA	NA	NA	NA	NA	888,12	PASS	AC=1;AF=0
0	0	0	4,403	0,901	31	TRAF2|0.0	Donor Gai	PP3	NA	NA	703,01	PASS	AC=1;AF=0
0	0	0	0	0	0	NA	NA	NA	NA	NA	131,94	PASS	AC=1;AF=0
6,67E-05	0	0,000399	4,414	0,903	31	TRAF2|0.0	Donor Gai	PP3	NA	NA	835,11	PASS	AC=1;AF=0
0	0	0	7,592	0,98	38	TRAF2|0.0	Donor Gai	PP3	NA	NA	958,68	PASS	AC=2;AF=0
0	0	0	4,388	0,899	31	ABCA2|0.0	Acceptor G	BP7	NA	NA	715,09	PASS	AC=1;AF=0
0	0	0	0	0	0	ABCA2|0.0	Donor Los	BP7	NA	NA	2706,62	PASS	AC=2;AF=0
0	0	0	7,942	0,986	40	ABCA2|0.0	Acceptor L	VUS	NA	NA	1809,79	PASS	AC=2;AF=0
0	0	0	0	0	0	NA	NA	NA	NA	NA	4053,62	PASS	AC=2;AF=0
0	0	0	6,1	0,959	35	MRPL41|0	Acceptor G	PP3	NA	NA	2137,75	PASS	AC=2;AF=0

0	0	0	4,386	0,899	31	CACNA1B|	Acceptor G	BP7	NA	NA	1480,69	PASS	AC=2;AF=0
7,00E-04	0	0,000799	4,501	0,913	32	CACNA1B|	NA	NA	NA	NA	925,01	PASS	AC=1;AF=0
0	0	0	0	0	0	DIP2C|0.0	Donor Los	PP3	NA	NA	1859,08	PASS	AC=1;AF=0
0	0	0	5,058	0,934	33	ADARB2|0	Acceptor L	PP3	NA	NA	844,68	PASS	AC=2;AF=0
0	0	0	4,688	0,923	32	SFMBT2|0	Donor Los	BP7	NA	NA	864,09	PASS	AC=1;AF=0
0	0	0	4,422	0,904	31	SEPHS1|0.	Donor Los	BP7	NA	NA	989,73	PASS	AC=2;AF=0
0	0	0	4,401	0,901	31	FRMD4A|0	Donor Los	BP7	NA	NA	1551,68	PASS	AC=2;AF=0
0	0	0	0	0	0	HSPA14|0	Donor Los	PP3	NA	NA	885,16	PASS	AC=1;AF=0
1,00E-04	0	0	0	0	0	SUV39H2|	Donor Los	VUS	NA	NA	414,62	PASS	AC=1;AF=0
0	0	0	4,385	0,899	31	MLLT10|0	Donor Los	BP7	NA	NA	822,68	PASS	AC=2;AF=0
0	0	0	4,374	0,897	31	ARHGAP21	Acceptor L	BP7	NA	NA	231,73	PASS	AC=2;AF=0
0,0013	0,001	0,000799	4,397	0,901	31	ABI1|0.00	Acceptor L	BP7	NA	NA	731,71	PASS	AC=2;AF=0
0	0	0	4,395	0,9	31	ZEB1|0.00	NA	NA	NA	NA	1219	PASS	AC=1;AF=0
0	0	0	0	0	0	ZEB1|0.00	NA	NA	NA	NA	1765,69	PASS	AC=2;AF=0
0	0	0	2,89	0,497	23,2	ITGB1|0.0	Donor Gai	PP3	NA	NA	843,91	PASS	AC=2;AF=0
0	0	0	4,355	0,894	30	ITGB1|0.0	NA	NA	NA	NA	1861,69	PASS	AC=2;AF=0
0	0	0,0002	4,5	0,912	32	NRP1|0.00	Acceptor L	BP7	NA	NA	487	PASS	AC=1;AF=0
6,67E-05	0	0	4,418	0,903	31	PARD3|0.0	Donor Gai	VUS	NA	NA	1132	PASS	AC=1;AF=0
0	0	0	5,502	0,946	34	PARD3|0.0	Donor Los	PP3	NA	NA	278,04	PASS	AC=1;AF=0
0	0	0	0	0	0	CREM|0.0	Donor Los	VUS	NA	NA	211,66	PASS	AC=2;AF=0
0	0	0	4,498	0,912	32	WDFY4|0.	Acceptor L	BP7	NA	NA	1492,22	PASS	AC=3;AF=0
0,0034	0,002	0,000799	6,621	0,966	36	C10orf53|	NA	NA	NA	NA	1356,7	PASS	AC=2;AF=0
0	0	0	5,274	0,939	33	BICC1|0.0	Donor Gai	BP7	NA	NA	611,01	PASS	AC=1;AF=0
0	0	0	0	0	0	PHYHIPL|0	Donor Los	PP3	NA	NA	1894,72	PASS	AC=2;AF=0
6,66E-05	0	0	0	0	0	ANK3|0.00	Donor Los	PP3	NA	NA	1570,69	PASS	AC=2;AF=0
0	0	0	4,492	0,912	32	JMJD1C|0.	Donor Los	BP7	NA	NA	591,03	PASS	AC=1;AF=0
0	0	0	4,404	0,902	31	CCAR1|0.0	NA	NA	NA	NA	779,7	PASS	AC=2;AF=0
6,67E-05	0	0	0	0	0	VPS26A|0.	Acceptor L	BP7	NA	NA	730,96	PASS	AC=1;AF=0
0	0	0	0	0	0	HK1|0.33|	Acceptor G	PP3	NA	NA	418,7	PASS	AC=2;AF=0
0	0	0	4,791	0,927	33	PPP3CB|0.	NA	NA	NA	NA	1158,7	PASS	AC=2;AF=0
0	0	0	4,423	0,904	31	RP11-574K	Donor Los	BP7	NA	NA	725,16	PASS	AC=1;AF=0
6,69E-05	0	0	4,421	0,904	31	ZNF503|0.	NA	NA	NA	NA	1122,93	PASS	AC=2;AF=0
0	0	0	0	0	0	ZMIZ1|0.1	Acceptor G	VUS	NA	NA	646,08	PASS	AC=1;AF=0
6,68E-05	0	0,0002	4,375	0,897	31	ZMIZ1|0.0	Acceptor G	BP7	NA	NA	1079,24	PASS	AC=1;AF=0
6,67E-05	0	0,000399	4,366	0,896	31	ZMIZ1|0.0	Acceptor G	BP7	NA	NA	618,03	PASS	AC=1;AF=0
0	0	0,000399	4,378	0,898	31	CCSER2|0.	Donor Gai	PP3	NA	NA	1379,69	PASS	AC=2;AF=0

0	0	0	6,547	0,965	36	CCSER2|0.	NA	NA	NA	NA	1384,69	PASS		AC=2;AF=0
0	0	0	2,358	0,381	21,9	CCSER2|0.	Donor Los	BP7	NA	NA	2182,13	PASS		AC=3;AF=0
0	0	0	4,417	0,903	31	BMPR1A|0	NA	NA	NA	NA	733,71	PASS		AC=2;AF=0
0	0	0	4,462	0,909	32	GLUD1|0.	NA	NA	NA	NA	319,11	PASS		AC=2;AF=0
0	0	0	4,415	0,903	31	PTEN|0.00	Acceptor L	BP7	NA	NA	965,8	PASS		AC=2;AF=0
6,66E-05	0	0	4,488	0,911	32	BTAF1|0.0	Acceptor G	BP7	NA	NA	1343,77	PASS		AC=2;AF=0
1,00E-04	0	0	4,357	0,894	30	LGI1|0.00	Donor Los	VUS	NA	NA	2281,72	PASS		AC=2;AF=0
0	0	0	4,397	0,901	31	SLIT1|0.04	Acceptor G	BP7	NA	NA	1589,69	PASS		AC=2;AF=0
0	0	0	4,707	0,924	32	SLIT1|0.00	NA	NA	NA	NA	1092,68	PASS		AC=2;AF=0
0	0	0	4,463	0,909	32	SLIT1|0.00	Donor Los	BP7	NA	NA	1148,71	PASS		AC=2;AF=0
0	0	0	4,439	0,906	31	CHUK|0.0	Acceptor L	BP7	NA	NA	635	PASS		AC=1;AF=0
0	0	0	5,766	0,953	34	TLX1|0.69	Acceptor L	PP3	NA	NA	3674,9	PASS		AC=4;AF=0
0	0	0	4,802	0,927	33	PPRC1|0.3	Acceptor L	PP3	NA	NA	1337	PASS		AC=1;AF=0
0	0	0	0	0	0	PPRC1|0.0	Donor Gai	VUS	NA	NA	5355,62	PASS		AC=2;AF=0
0	0	0	0	0	0	NFKB2|0.0	Donor Gai	PP3	NA	NA	219,36	PASS		AC=2;AF=0
0	0	0	0	0	0	NFKB2|0.0	Acceptor L	PP3	NA	NA	1233,08	PASS		AC=1;AF=0
0	0	0	4,403	0,901	31	SUFU|0.01	Donor Gai	BP7	NA	NA	1867,99	PASS		AC=1;AF=0
3,00E-04	0	0	4,397	0,901	31	SUFU|0.00	Acceptor L	BP7	NA	NA	1348	PASS		AC=1;AF=0
6,67E-05	0	0	4,496	0,912	32	SUFU|0.00	NA	NA	NA	NA	5396,68	PASS		AC=2;AF=0
0	0	0	4,388	0,899	31	WBP1L|0.	Donor Los	BP7	NA	NA	1825,68	PASS		AC=2;AF=0
0	0	0	4,762	0,926	33	CNNM2|0	Acceptor L	VUS	NA	NA	884	PASS		AC=1;AF=0
0	0	0	0	0	0	SH3PXD2A	Donor Los	PP3	NA	NA	2105,68	PASS		AC=2;AF=0
7,00E-04	0	0,0002	0	0	0	SH3PXD2A	Acceptor G	PP3	NA	NA	1086	PASS		AC=1;AF=0
0	0	0	0	0	0	SORCS1|0.	Donor Los	PP3	NA	NA	1295,57	PASS		AC=1;AF=0
0	0	0	0	0	0	SMNDC1|	Acceptor G	PP3	NA	NA	729,16	PASS		AC=2;AF=0
0	0	0	0	0	0	SMC3|0.8	Acceptor G	PP3	NA	NA	891,02	PASS		AC=1;AF=0
0	0	0	4,357	0,894	30	VAX1|0.00	NA	NA	NA	NA	275,17	PASS		AC=1;AF=0
0	0	0	6,963	0,97	36	EIF3A|0.00	Donor Gai	BP7	hiConfDeN	0,999995	3235,01	PASS		AC=1;AF=0
0	0,001	0	4,471	0,91	32	EIF3A|0.00	Donor Gai	BP7	NA	NA	522	PASS		AC=1;AF=0
0	0	0	0	0	0	GRK5|0.00	NA	NA	NA	NA	875,62	PASS		AC=2;AF=0
0	0	0	0	0	0	FGFR2|0.1	Acceptor G	VUS	NA	NA	258,02	PASS		AC=1;AF=0
0	0	0	4,599	0,919	32	HMX3|0.0	NA	NA	NA	NA	1956,01	PASS		AC=1;AF=0
0	0	0	0	0	0	AP2A2|0.0	NA	NA	NA	NA	961,95	PASS		AC=1;AF=0
0	0	0	0	0	0	BRSK2|0.0	Donor Gai	VUS	NA	NA	507,03	PASS		AC=1;AF=0
0	0	0	0	0	0	BRSK2|0.0	Donor Los	BP7	hiConfDeN	0,995461	3293,97	PASS		AC=1;AF=0
0	0	0,0002	0	0	0	DUSP8|0.0	Donor Los	PP3	NA	NA	2867,98	PASS		AC=2;AF=0

0	0	0	1,436	0,209	15,32	CDKN1C|0	Donor Gai	PP3	NA	NA	1351,94	PASS	AC=2;AF=0
0	0	0	4,476	0,91	32	CDKN1C|0	NA	NA	NA	NA	1031,11	PASS	AC=1;AF=0
0	0	0	6,094	0,959	35	STIM1|0.0	Donor Los	PP3	NA	NA	2246,68	PASS	AC=2;AF=0
0	0	0	4,431	0,905	31	APBB1|0.0	Donor Gai	BP7	NA	NA	2435,7	PASS	AC=2;AF=0
0	0	0	4,426	0,904	31	TAF10|0.0	NA	NA	NA	NA	1019	PASS	AC=1;AF=0
2,00E-04	0	0	0	0	0	ARNTL|0.0	Acceptor L	PP3	NA	NA	718,95	PASS	AC=1;AF=0
0	0	0	0	0	0	GTF2H1|0	Acceptor L	PP3	NA	NA	521,91	PASS	AC=1;AF=0
0	0	0	5,327	0,941	33	SLC1A2|0.	Donor Los	BP7	NA	NA	527,72	PASS	AC=2;AF=0
0	0	0	0	0	0	TTC17|0.0	Acceptor L	PP3	NA	NA	592	PASS	AC=1;AF=0
0	0	0	4,643	0,922	32	MAPK8IP1	NA	NA	NA	NA	501,68	PASS	AC=2;AF=0
0	0	0	4,559	0,917	32	CREB3L1|0	NA	NA	NA	NA	553,13	PASS	AC=1;AF=0
0	0	0	0	0	0	AMBRA1|	Acceptor G	VUS	NA	NA	490,02	PASS	AC=1;AF=0
0	0	0	4,59	0,919	32	LRP4|0.00	Donor Gai	PP3	NA	NA	407,69	PASS	AC=2;AF=0
0	0	0	4,518	0,914	32	NR1H3|0.	Acceptor L	BP7	NA	NA	3224,69	PASS	AC=2;AF=0
4,00E-04	0	0	4,598	0,919	32	NR1H3|0.	NA	NA	NA	NA	1333,02	PASS	AC=1;AF=0
0	0	0	0	0	0	NA	NA	NA	NA	NA	694,97	PASS	AC=1;AF=0
0	0	0	0	0	0	NA	NA	NA	NA	NA	649,93	PASS	AC=1;AF=0
0	0,001	0	4,395	0,9	31	OSBP|0.00	NA	NA	NA	NA	2424,73	PASS	AC=2;AF=0
0	0	0	9,261	0,998	48	AHNAK|0.	NA	NA	NA	NA	1315,69	PASS	AC=2;AF=0
0	0	0	0	0	0	AHNAK|0.	NA	NA	NA	NA	1458,95	PASS	AC=1;AF=0
0	0	0	5,453	0,944	34	AHNAK|0.	NA	NA	NA	NA	395,99	PASS	AC=1;AF=0
0	0	0	0	0	0	GANAB|0.	Donor Los	VUS	NA	NA	92,58	PASS	AC=1;AF=0
0	0	0	0	0	0	NA	NA	NA	NA	NA	2261,94	PASS	AC=1;AF=0
0	0	0	0	0	0	STX5|0.94	Acceptor G	PP3	NA	NA	1209	PASS	AC=1;AF=0
0	0	0	4,4	0,901	31	MACROD1	Donor Los	PP3	NA	NA	1224,11	PASS	AC=1;AF=0
0	0	0	5,723	0,952	34	MACROD1	Donor Los	PP3	NA	NA	597,33	PASS	AC=1;AF=0
0	0	0	0	0	0	NA	NA	NA	NA	NA	2608,84	PASS	AC=2;AF=0
0	0	0	0	0	0	GPR137|0	Donor Gai	PP3	NA	NA	5523,81	PASS	AC=2;AF=0
0	0	0	6,03	0,958	35	NRXN2|0.	Donor Los	PP3	NA	NA	1109,14	PASS	AC=1;AF=0
6,96E-05	0	0	0	0	0	SF1|0.00|	NA	NA	NA	NA	769,81	PASS	AC=2;AF=0
0	0	0	4,438	0,906	31	SYVN1|0.0	Donor Los	BP7	NA	NA	1294	PASS	AC=1;AF=0
0	0	0	0	0	0	LTBP3|0.0	Donor Los	PP3	NA	NA	578,25	PASS	AC=2;AF=0
0	0	0	4,404	0,902	31	RBM4|0.0	NA	NA	NA	NA	811,8	PASS	AC=2;AF=0
0	0	0	6,126	0,959	35	PC|0.00|0	Donor Los	PP3	NA	NA	1481,02	PASS	AC=1;AF=0
6,68E-05	0	0	4,495	0,912	32	LRP5|0.00	NA	NA	NA	NA	5182,79	PASS	AC=2;AF=0
0	0	0	4,348	0,892	30	LRP5|0.00	NA	NA	NA	NA	1839,68	PASS	AC=2;AF=0

3,00E-04	0	0,0002	4,637	0,921	32	PPP6R3|0.	NA	NA	NA	NA	395,8	PASS	AC=2;AF=0
0	0	0	4,625	0,921	32	CCND1|0.	NA	NA	NA	NA	711,04	PASS	AC=1;AF=0
0	0	0	0	0	0	ANO1|0.0	NA	NA	NA	NA	850,95	PASS	AC=1;AF=0
0	0	0	4,463	0,909	32	ANO1|0.0	Donor Gai	PP3	NA	NA	1093,69	PASS	AC=2;AF=0
0	0	0	0	0	0	ANO1|0.0	Acceptor L	BP7	NA	NA	1015,93	PASS	AC=1;AF=0
0	0	0	5,549	0,947	34	FADD|0.00	NA	NA	NA	NA	617	PASS	AC=1;AF=0
0,0017	0	0	4,433	0,906	31	NUMA1|0	Acceptor L	BP7	NA	NA	1057	PASS	AC=1;AF=0
0	0	0	0	0	0	NUMA1|0	Donor Los	VUS	NA	NA	456,01	PASS	AC=1;AF=0
6,67E-05	0,002	0,0002	4,533	0,915	32	NUMA1|0	Acceptor G	BP7	NA	NA	937,01	PASS	AC=1;AF=0
0	0	0	4,36	0,894	31	NUMA1|0	Donor Los	VUS	NA	NA	933,7	PASS	AC=2;AF=0
0	0	0	7,506	0,979	38	ARHGEF17	Acceptor L	BP7	NA	NA	4546,73	PASS	AC=2;AF=0
0	0	0	4,349	0,893	30	ARHGEF17	NA	NA	NA	NA	1253,71	PASS	AC=2;AF=0
1,00E-04	0	0	4,543	0,916	32	ARHGEF17	Donor Los	BP7	NA	NA	785	PASS	AC=1;AF=0
0,0045	0,0051	0	4,604	0,92	32	ARHGEF17	Donor Los	BP7	NA	NA	439,74	PASS	AC=2;AF=0
0	0	0	8,247	0,991	42	ARHGEF17	NA	NA	NA	NA	657,69	PASS	AC=2;AF=0
0	0	0,000599	4,566	0,917	32	PPME1|0.	NA	NA	NA	NA	145,01	PASS	AC=1;AF=0
0	0	0	4,772	0,926	33	LIPT2|0.01	Acceptor G	BP7	NA	NA	839,16	PASS	AC=1;AF=0
0	0	0	0	0	0	ARRB1|0.0	Donor Los	PP3	NA	NA	237,06	PASS	AC=1;AF=0
0	0	0	4,734	0,925	32	TENM4|0.	NA	NA	NA	NA	1372	PASS	AC=1;AF=0
0	0	0	4,543	0,916	32	TENM4|0.	NA	NA	NA	NA	3064,08	PASS	AC=1;AF=0
0	0	0	4,677	0,923	32	TENM4|0.	Acceptor L	BP7	NA	NA	294	PASS	AC=1;AF=0
0	0	0,000399	4,379	0,898	31	TENM4|0.	NA	NA	NA	NA	1669,68	PASS	AC=2;AF=0
0	0	0	4,486	0,911	32	PCF11|0.0	NA	NA	NA	NA	2384,69	PASS	AC=2;AF=0
0	0	0	4,728	0,925	32	GRM5|0.0	NA	NA	NA	NA	2214,93	PASS	AC=2;AF=0
0	0	0	8,058	0,988	40	FAT3|0.00	NA	NA	NA	NA	732	PASS	AC=1;AF=0
0	0	0	5,288	0,94	33	FAT3|0.09	Acceptor L	VUS	NA	NA	1220,77	PASS	AC=2;AF=0
6,68E-05	0	0	0	0	0	DCUN1D5	Donor Gai	PP3	NA	NA	602,82	PASS	AC=2;AF=0
0	0	0	4,34	0,891	29,9	CADM1|0.	Donor Los	PP3	hiConfDeN	0,999995	225	PASS	AC=1;AF=0
0	0	0	0	0	0	PAFAH1B2	Donor Los	BP7	NA	NA	2047,94	PASS	AC=1;AF=0
0	0	0	0,735	0,118	8,776	NA	NA	NA	NA	NA	588,69	PASS	AC=1;AF=0
0	0	0	1,21	0,178	13,71	NA	NA	NA	NA	NA	1061,04	PASS	AC=2;AF=0
0	0	0	4,408	0,902	31	RNF214|0.	Acceptor L	BP7	NA	NA	2590	PASS	AC=1;AF=0
0	0	0	4,449	0,907	32	DSCAML1|	Acceptor G	BP7	NA	NA	640	PASS	AC=1;AF=0
0	0	0	0	0	0	DSCAML1|	Acceptor L	VUS	NA	NA	1683,79	PASS	AC=2;AF=0
0	0	0	4,361	0,895	31	DSCAML1|	NA	NA	NA	NA	932,01	PASS	AC=1;AF=0
0	0	0	4,414	0,903	31	KMT2A|0.	Acceptor G	PP3	NA	NA	1366,73	PASS	AC=2;AF=0

0	0	0	4,36	0,894	31	KMT2A|0.	NA	NA	NA	NA	1382,69	PASS	AC=2;AF=0
0	0	0	4,457	0,908	32	KMT2A|0.	Donor Los	BP7	NA	NA	2687,69	PASS	AC=2;AF=0
0	0	0	4,363	0,895	31	KMT2A|0.	Acceptor L	BP7	NA	NA	1006	PASS	AC=1;AF=0
6,66E-05	0,002	0	0	0	0	DDX6|0.00	Acceptor L	PP3	NA	NA	156,01	PASS	AC=1;AF=0
6,67E-05	0	0	4,448	0,907	32	HMBS|0.0	Donor Gai	BP7	NA	NA	3810,68	PASS	AC=2;AF=0
0	0	0,0002	4,352	0,893	30	C2CD2L|0.	NA	NA	NA	NA	864,05	PASS	AC=1;AF=0
0	0	0	0	0	0	MFRP|0.0	Donor Los	PP3	NA	NA	4647,95	PASS	AC=2;AF=0
0	0	0	0	0	0	HEPACAM	Acceptor G	BP7	NA	NA	3657,62	PASS	AC=2;AF=0
0	0	0	4,456	0,908	32	STT3A|0.0	Acceptor L	BP7	NA	NA	739	PASS	AC=1;AF=0
6,67E-05	0,001	0,000399	4,623	0,921	32	STT3A|0.0	NA	NA	NA	NA	1240,96	PASS	AC=2;AF=0
0	0	0	0	0	0	ETS1|0.13	Acceptor G	VUS	NA	NA	319	PASS	AC=1;AF=0
6,66E-05	0	0	4,522	0,914	32	PRDM10|0	Donor Los	BP7	NA	NA	992,02	PASS	AC=1;AF=0
0	0	0	4,394	0,9	31	IGSF9B|0.	NA	NA	NA	NA	963,96	PASS	AC=2;AF=0
0	0	0	0	0	0	IGSF9B|0.	Acceptor G	PP3	NA	NA	2506,7	PASS	AC=2;AF=0
0	0	0	4,377	0,897	31	WNK1|0.0	Donor Gai	BP7	NA	NA	296,02	PASS	AC=1;AF=0
6,66E-05	0	0	4,377	0,897	31	WNK1|0.0	NA	NA	NA	NA	3205,84	PASS	AC=2;AF=0
0	0	0	6,498	0,964	36	FBXL14|0.	Acceptor L	BP7	NA	NA	779,71	PASS	AC=1;AF=0
0	0	0	0	0	0	CACNA1C|	Acceptor L	PP3	NA	NA	541,03	PASS	AC=1;AF=0
0	0	0	4,377	0,897	31	TSPAN9|0	Acceptor L	BP7	NA	NA	1281,68	PASS	AC=2;AF=0
0	0	0	4,993	0,932	33	NOP2|0.0	NA	NA	NA	NA	1242	PASS	AC=1;AF=0
0	0	0	0	0	0	CHD4|0.06	Acceptor L	PP3	NA	NA	1110,01	PASS	AC=1;AF=0
6,67E-05	0	0,000399	0	0	0	CHD4|0.69	Acceptor G	PP3	NA	NA	1166,01	PASS	AC=1;AF=0
0	0	0	0	0	0	ZNF384|0.	NA	NA	NA	NA	3193120	PASS	AC=2;AF=0
0	0	0	4,412	0,903	31	PTPN6|0.0	Acceptor G	BP7	NA	NA	3976,69	PASS	AC=2;AF=0
0	0	0	4,355	0,894	30	CLSTN3|0.	Acceptor G	BP7	NA	NA	1563,06	PASS	AC=1;AF=0
0	0	0	0	0	0	CLSTN3|0.	NA	NA	NA	NA	686,26	PASS	AC=1;AF=0
0	0	0	0	0	0	PHC1|0.00	Donor Los	PP3	NA	NA	842,01	PASS	AC=1;AF=0
1,00E-04	0	0	4,361	0,895	31	GABARAPL	NA	NA	NA	NA	742,01	PASS	AC=1;AF=0
0	0	0	0	0	0	EPS8|0.00	Donor Los	PP3	NA	NA	1045,7	PASS	AC=2;AF=0
0	0	0	6,769	0,967	36	STRAP|0.0	Donor Los	PP3	NA	NA	2176,69	PASS	AC=2;AF=0
0	0	0	0	0	0	RASSF8|0.	Acceptor L	PP3	NA	NA	716,78	PASS	AC=2;AF=0
0	0	0	4,566	0,917	32	BHLHE41|	NA	NA	NA	NA	445,53	PASS	AC=1;AF=0
0	0	0	5,626	0,949	34	BHLHE41|	Donor Los	PP3	NA	NA	3420,68	PASS	AC=2;AF=0
0	0	0	4,99	0,932	33	STK38L|0.	Donor Los	PP3	NA	NA	68,01	PASS	AC=1;AF=0
0	0	0	0	0	0	STK38L|0.	Donor Los	PP3	NA	NA	258,73	PASS	AC=2;AF=0
0	0	0	4,382	0,898	31	DENND5B	NA	NA	NA	NA	539	PASS	AC=1;AF=0

0	0	0	0	0	0	SLC2A13|0	Acceptor L	PP3	NA	NA	1418,74	PASS	AC=2;AF=0
0	0	0	0	0	0	PRICKLE1|	NA	NA	NA	NA	2429,94	PASS	AC=1;AF=0
0	0	0	4,365	0,895	31	NELL2|0.0	Donor Los	PP3	NA	NA	556	PASS	AC=1;AF=0
0	0	0	7,273	0,975	37	SCAF11|0.	NA	NA	NA	NA	587	PASS	AC=1;AF=0
0	0	0	4,566	0,917	32	SLC38A2|0	NA	NA	NA	NA	213,03	PASS	AC=1;AF=0
0	0	0	4,35	0,893	30	HDAC7|0.	Donor Los	PP3	NA	NA	1472,74	PASS	AC=2;AF=0
0	0	0	0	0	0	HDAC7|0.	Acceptor L	VUS	NA	NA	621,03	PASS	AC=1;AF=0
1,00E-04	0	0	0	0	0	KCNH3|0.	Acceptor L	PP3	NA	NA	2312,95	PASS	AC=1;AF=0
1,00E-04	0	0	0	0	0	KCNH3|0.	Acceptor L	PP3	NA	NA	862	PASS	AC=1;AF=0
0	0	0	4,518	0,914	32	PRPF40B|	NA	NA	NA	NA	709	PASS	AC=1;AF=0
6,69E-05	0	0	4,547	0,916	32	NCKAP5L|	Acceptor L	BP7	NA	NA	1716,68	PASS	AC=2;AF=0
0	0	0	4,391	0,9	31	ASIC1|0.0	NA	NA	NA	NA	505	PASS	AC=1;AF=0
0	0	0	0	0	0	NA	NA	NA	hiConfDeN	0,995461	625,96	PASS	AC=1;AF=0
0	0	0	4,543	0,916	32	DIP2B|0.0	NA	NA	NA	NA	1921	PASS	AC=1;AF=0
6,82E-05	0	0	-0,097	0,035	0,84	SLC4A8|0.	Acceptor L	BP7	NA	NA	278,8	PASS	AC=2;AF=0
2,00E-04	0	0	4,855	0,929	33	KRT18|0.0	Acceptor L	BP7	NA	NA	2342,71	PASS	AC=2;AF=0
0	0	0	2,349	0,379	21,8	RARG|0.0	NA	NA	NA	NA	704,65	PASS	AC=1;AF=0
1,00E-04	0	0	0	0	0	RARG|0.0	NA	NA	NA	NA	1272,61	PASS	AC=2;AF=0
0	0	0	4,949	0,931	33	MAP3K12|	Donor Los	PP3	NA	NA	3119,01	PASS	AC=1;AF=0
0	0	0	7,117	0,972	37	SMARCC2|	Acceptor G	VUS	NA	NA	4512,69	PASS	AC=2;AF=0
0	0	0	0	0	0	ANKRD52|	Donor Gai	PP3	NA	NA	1054,12	PASS	AC=1;AF=0
0	0	0	4,747	0,926	32	PAN2|0.00	Donor Los	BP7	NA	NA	319	PASS	AC=1;AF=0
0	0	0,000599	4,581	0,918	32	PAN2|0.05	Acceptor G	BP7	NA	NA	529,69	PASS	AC=2;AF=0
0	0	0	4,355	0,894	30	PAN2|0.00	NA	NA	NA	NA	1480,69	PASS	AC=2;AF=0
0	0	0,0002	0	0	0	STAT2|0.0	Donor Los	VUS	NA	NA	1819,68	PASS	AC=2;AF=0
0	0	0	4,389	0,899	31	NAB2|0.00	NA	NA	NA	NA	1271,08	PASS	AC=1;AF=0
0	0	0	0	0	0	STAT6|0.0	Donor Los	PP3	NA	NA	612,99	PASS	AC=1;AF=0
8,00E-04	0	0	4,349	0,892	30	LRP1|0.00	NA	NA	NA	NA	5687,68	PASS	AC=2;AF=0
0	0	0	5,149	0,936	33	LRP1|0.00	Donor Gai	PP3	NA	NA	471,14	PASS	AC=1;AF=0
0	0	0	0	0	0	LRP1|0.00	Donor Los	PP3	NA	NA	2203,83	PASS	AC=1;AF=0
2,00E-04	0	0	4,551	0,916	32	MBD6|0.0	Acceptor L	BP7	NA	NA	1994,69	PASS	AC=2;AF=0
0	0	0	4,593	0,919	32	MBD6|0.8	Acceptor G	PP3	NA	NA	982	PASS	AC=1;AF=0
0	0	0	0	0	0	AGAP2|0.	Donor Gai	PP3	NA	NA	1098,15	PASS	AC=1;AF=0
0	0	0	0	0	0	PPM1H|0.	Donor Los	BP7	NA	NA	905,94	PASS	AC=1;AF=0
0	0	0	7,21	0,974	37	PPM1H|0.	Acceptor L	BP7	NA	NA	289	PASS	AC=1;AF=0
0	0	0	5,044	0,934	33	SRGAP1|0	Donor Gai	PP3	NA	NA	374,04	PASS	AC=1;AF=0

9,00E-04	0	0,000599	4,363	0,895	31	SRGAP1|0	Acceptor L	BP7	NA	NA	1239,68	PASS	AC=2;AF=0
6,67E-05	0	0	4,367	0,896	31	XPOT|0.00	NA	NA	NA	NA	674,05	PASS	AC=1;AF=0
0	0	0	0	0	0	TBC1D30|	Donor Los	BP7	NA	NA	890,95	PASS	AC=2;AF=0
0	0	0,0002	4,378	0,898	31	TBC1D30|	NA	NA	NA	NA	1802,66	PASS	AC=1;AF=0
0	0	0	4,489	0,911	32	GRIP1|0.0	NA	NA	NA	NA	1715,69	PASS	AC=2;AF=0
0	0	0	4,645	0,922	32	GRIP1|0.0	Acceptor L	BP7	NA	NA	1480,01	PASS	AC=1;AF=0
0	0	0	0	0	0	MDM2|0.	Acceptor L	PP3	NA	NA	548	PASS	AC=1;AF=0
0	0	0	5,256	0,939	33	CCT2|0.58	Acceptor G	PP3	NA	NA	448,71	PASS	AC=2;AF=0
5,00E-04	0	0,0002	4,492	0,912	32	CCT2|0.00	Donor Los	BP7	NA	NA	533,06	PASS	AC=1;AF=0
0	0	0	4,646	0,922	32	PTPRB|0.0	NA	NA	NA	NA	664,68	PASS	AC=2;AF=0
0	0	0	8,123	0,989	41	PTPRB|0.0	Acceptor L	BP7	NA	NA	300	PASS	AC=1;AF=0
0	0	0	7,334	0,976	37	NAV3|0.0	NA	NA	NA	NA	537,69	PASS	AC=2;AF=0
0	0	0	4,887	0,93	33	NAV3|0.0	Donor Los	BP7	NA	NA	100,11	PASS	AC=1;AF=0
0	0	0	7,677	0,982	39	NAV3|0.0	Acceptor L	BP7	NA	NA	998,08	PASS	AC=1;AF=0
6,67E-05	0	0	10,272	1	55	NAV3|0.0	Acceptor L	VUS	NA	NA	661,7	PASS	AC=2;AF=0
0	0	0	0	0	0	NAV3|0.0	Donor Gai	PP3	NA	NA	801,84	PASS	AC=2;AF=0
0	0	0	4,388	0,899	31	PPFIA2|0.	Donor Los	VUS	NA	NA	1078,68	PASS	AC=2;AF=0
0	0,001	0,003594	0	0	0	PPFIA2|0.	Acceptor G	PP3	NA	NA	1516,96	PASS	AC=1;AF=0
0	0	0	0	0	0	PPFIA2|0.	Donor Los	PP3	NA	NA	1950,8	PASS	AC=2;AF=0
0	0	0	0	0	0	EEA1|0.05	Acceptor L	VUS	NA	NA	795,75	PASS	AC=2;AF=0
0	0	0	4,65	0,922	32	ANKS1B|0	Donor Gai	PP3	NA	NA	555	PASS	AC=1;AF=0
0	0	0	4,627	0,921	32	HSP90B1|	Acceptor G	PP3	NA	NA	235,68	PASS	AC=2;AF=0
0	0	0	5,349	0,941	34	C12orf75|	Donor Los	PP3	NA	NA	790,68	PASS	AC=2;AF=0
0	0	0	4,398	0,901	31	POLR3B|0	Donor Los	BP7	NA	NA	689,1	PASS	AC=1;AF=0
0	0	0	4,682	0,923	32	RFX4|0.00	Donor Los	BP7	NA	NA	388,04	PASS	AC=1;AF=0
0	0	0	4,506	0,913	32	BTBD11|0	NA	NA	NA	NA	1334,13	PASS	AC=1;AF=0
3,00E-04	0	0,0002	4,553	0,917	32	BTBD11|0	Acceptor L	BP7	NA	NA	1102,68	PASS	AC=2;AF=0
0	0	0	4,444	0,907	31	SART3|0.0	NA	NA	NA	NA	1422,7	PASS	AC=2;AF=0
0	0	0	0	0	0	SART3|0.0	Acceptor L	VUS	NA	NA	1842,73	PASS	AC=2;AF=0
6,66E-05	0	0	4,526	0,915	32	SART3|0.0	Donor Gai	BP7	NA	NA	149	PASS	AC=1;AF=0
0	0	0	0	0	0	NA	NA	NA	NA	NA	1492,65	PASS	AC=2;AF=0
0	0	0,0002	6,82	0,968	36	TMEM119	NA	NA	NA	NA	1498,03	PASS	AC=1;AF=0
0	0	0	7,793	0,984	39	ANAPC7|0	Acceptor L	BP7	NA	NA	789,49	PASS	AC=1;AF=0
0	0	0	5,841	0,954	35	PPP1CC|0.	Acceptor L	PP3	NA	NA	313,29	PASS	AC=1;AF=0
0	0	0	4,998	0,932	33	CUX2|0.00	Donor Los	PP3	NA	NA	452,72	PASS	AC=2;AF=0
0	0	0	5,526	0,946	34	ATXN2|0.1	Acceptor L	PP3	hiConfDeN	0,999994	581,35	PASS	AC=1;AF=0

0	0	0	6,599	0,965	36	ATXN2|0.0	Donor Los	PP3	NA	NA	993,73	PASS	AC=2;AF=0
0	0	0	0	0	0	NA	NA	NA	NA	0,997725	50380,9	PASS	AC=1;AF=0
0	0	0	0	0	0	HECTD4|0	Acceptor G	PP3	NA	NA	3889,69	PASS	AC=2;AF=0
0	0	0	4,348	0,892	30	TBX3|0.00	NA	NA	NA	NA	1754,01	PASS	AC=1;AF=0
0	0	0	4,574	0,918	32	TBX3|0.00	NA	NA	NA	NA	2985	PASS	AC=1;AF=0
0	0	0	4,578	0,918	32	MED13L|0	Acceptor L	BP7	NA	NA	796,05	PASS	AC=1;AF=0
0	0	0	4,602	0,919	32	NOS1|0.02	Acceptor G	BP7	NA	NA	2277,11	PASS	AC=1;AF=0
0	0	0	4,524	0,914	32	KSR2|0.00	NA	NA	NA	NA	1199,69	PASS	AC=2;AF=0
0	0	0	4,537	0,915	32	CIT|0.00|0	NA	NA	NA	NA	1017	PASS	AC=1;AF=0
0	0	0	5,411	0,943	34	CIT|0.00|0	Donor Los	BP7	NA	NA	141,88	PASS	AC=1;AF=0
0	0	0	4,996	0,932	33	MSI1|0.31	Acceptor L	PP3	NA	NA	1386,7	PASS	AC=2;AF=0
0	0	0	0	0	0	SRSF9|0.8	Acceptor G	PP3	NA	NA	316,01	PASS	AC=1;AF=0
0	0	0	0	0	0	DYNLL1|0.	Acceptor G	VUS	NA	NA	646	PASS	AC=1;AF=0
0	0	0	0	0	0	UNC119B|	Acceptor L	BP7	NA	NA	1367,93	PASS	AC=1;AF=0
0	0	0	0	0	0	KDM2B|0.	Acceptor L	PP3	hiConfDeN	0,999997	1317	PASS	AC=1;AF=0
0	0	0	4,046	0,802	27,3	ZCCHC8|0	Donor Los	PP3	NA	NA	1319,05	PASS	AC=2;AF=0
0	0	0	0	0	0	NA	NA	NA	NA	NA	2039,11	PASS	AC=1;AF=0
0	0	0	4,467	0,909	32	PITPNM2|	NA	NA	NA	NA	1637,69	PASS	AC=2;AF=0
1,00E-04	0	0	4,443	0,907	31	NCOR2|0.	NA	NA	NA	NA	361,02	PASS	AC=1;AF=0
0	0	0	7,89	0,985	40	DHX37|0.0	Acceptor L	BP7	NA	NA	2849,68	PASS	AC=2;AF=0
6,67E-05	0	0	5,63	0,949	34	EP400|0.0	Donor Los	PP3	NA	NA	542,7	PASS	AC=2;AF=0
0	0	0	0	0	0	EP400|0.0	Donor Gai	PP3	NA	NA	830,07	PASS	AC=1;AF=0
0	0	0	0	0	0	EP400|0.0	Acceptor L	PP3	NA	NA	395,01	PASS	AC=1;AF=0
7,35E-05	0	0	0	0	0	EP400|0.0	Acceptor L	BP7	hiConfDeN	NA	133477	PASS	AC=1;AF=0
0	0	0	0	0	0	XPO4|0.00	Donor Los	VUS	NA	NA	142,02	PASS	AC=1;AF=0
0	0	0	0	0	0	XPO4|0.00	NA	NA	NA	NA	483,1	PASS	AC=1;AF=0
0	0	0	0	0	0	XPO4|0.00	NA	NA	NA	NA	483,05	PASS	AC=1;AF=0
0	0	0	4,515	0,914	32	FLT3|0.14	Acceptor G	VUS	NA	NA	549	PASS	AC=1;AF=0
6,67E-05	0	0	0	0	0	NA	NA	NA	NA	NA	1392,21	PASS	AC=1;AF=0
6,66E-05	0	0	-0,054	0,038	1,035	FLT1|0.00	NA	NA	NA	NA	319,2	PASS	AC=1;AF=0
0	0	0	5,695	0,951	34	POMP|0.3	Acceptor L	PP3	NA	NA	391,02	PASS	AC=1;AF=0
0	0	0	5,371	0,942	34	KATNAL1|	Donor Gai	PP3	NA	NA	601,75	PASS	AC=2;AF=0
0	0	0	4,683	0,923	32	HSPH1|0.6	Acceptor G	PP3	NA	NA	479,13	PASS	AC=1;AF=0
0	0	0,0002	4,452	0,908	32	FRY|0.00|	Acceptor L	BP7	NA	NA	395	PASS	AC=1;AF=0
0	0	0	4,398	0,901	31	FRY|0.01|	Acceptor L	BP7	NA	NA	1974,68	PASS	AC=2;AF=0
0	0	0	4,478	0,91	32	NBEA|0.00	Donor Los	BP7	NA	NA	645,71	PASS	AC=2;AF=0

0	0	0,001997	2,477	0,407	22,4	NBEA|0.11	Acceptor L	PP3	NA	NA	677,2	PASS	AC=2;AF=0
0	0	0	0	0	0	PROSER1|	Acceptor L	BP7	NA	NA	2234,63	PASS	AC=2;AF=0
0	0	0,0002	4,389	0,899	31	AKAP11|0	NA	NA	NA	NA	207,71	PASS	AC=2;AF=0
0	0	0	4,578	0,918	32	NA	NA	NA	NA	NA	1803,74	PASS	AC=2;AF=0
2,00E-04	0	0,000599	0	0	0	TPT1|0.00	Donor Los	PP3	NA	NA	1514,67	PASS	AC=2;AF=0
0	0	0,000799	0	0	0	TPT1|0.02	Donor Los	BP7	NA	NA	1362,63	PASS	AC=2;AF=0
6,67E-05	0	0	5,526	0,946	34	COG3|0.0	Donor Los	BP7	NA	NA	1189,82	PASS	AC=2;AF=0
0	0	0	4,417	0,903	31	COG3|0.0	Donor Los	BP7	NA	NA	765	PASS	AC=1;AF=0
0	0	0	4,394	0,9	31	LRCH1|0.0	NA	NA	NA	NA	2989,91	PASS	AC=2;AF=0
0	0	0	0	0	0	LRCH1|0.0	Acceptor L	PP3	NA	NA	666,96	PASS	AC=2;AF=0
0	0	0	5,058	0,934	33	FNDC3A|0	Donor Los	PP3	NA	NA	513,82	PASS	AC=2;AF=0
0	0	0,000998	4,443	0,907	31	FNDC3A|0	Donor Los	BP7	NA	NA	1634,68	PASS	AC=2;AF=0
6,67E-05	0	0,0002	4,526	0,915	32	PCDH17|0	NA	NA	NA	NA	4794,94	PASS	AC=2;AF=0
0	0	0	0	0	0	PCDH20|0	NA	NA	NA	NA	1757,95	PASS	AC=1;AF=0
0	0	0	0	0	0	PCDH20|0	NA	NA	NA	NA	886,17	PASS	AC=2;AF=0
5,00E-04	0,001	0,000998	4,355	0,894	30	PCDH9|0.0	Acceptor L	BP7	NA	NA	997,89	PASS	AC=4;AF=0
0,0013	0	0	4,383	0,898	31	DACH1|0.	Acceptor L	BP7	NA	NA	787	PASS	AC=1;AF=0
0	0	0	2,203	0,348	20,9	DACH1|0.	Donor Los	BP7	NA	NA	472,77	PASS	AC=1;AF=0
0	0	0	4,373	0,897	31	NA	NA	NA	NA	NA	226,68	PASS	AC=2;AF=0
0	0	0	4,365	0,896	31	SLAIN1|0.	NA	NA	NA	NA	339,01	PASS	AC=1;AF=0
0	0	0	4,438	0,906	31	SLAIN1|0.	Donor Los	BP7	NA	NA	1058	PASS	AC=1;AF=0
0	0	0,0002	0	0	0	NDFIP2|0.	Acceptor	GVUS	NA	NA	823,69	PASS	AC=2;AF=0
0	0	0	4,625	0,921	32	NDFIP2|0.	Acceptor L	PP3	NA	NA	1065,68	PASS	AC=2;AF=0
0	0	0	0	0	0	HS6ST3|0.	NA	NA	NA	NA	1464,93	PASS	AC=1;AF=0
0	0	0	4,369	0,896	31	RAP2A|0.0	NA	NA	NA	NA	1161,01	PASS	AC=1;AF=0
0	0	0	4,374	0,897	31	DOCK9|0.	NA	NA	NA	NA	1466	PASS	AC=1;AF=0
0	0	0	4,697	0,924	32	TM9SF2|0	NA	NA	NA	NA	327,03	PASS	AC=1;AF=0
0	0	0	0	0	0	NA	NA	NA	NA	NA	778,97	PASS	AC=2;AF=0
0	0	0	4,501	0,913	32	ZIC5|0.00|	NA	NA	NA	NA	710,45	PASS	AC=2;AF=0
0	0	0	4,397	0,901	31	ZIC2|0.00|	NA	NA	NA	NA	2673,13	PASS	AC=1;AF=0
0	0	0	4,402	0,901	31	ARGLU1|0	NA	NA	NA	NA	397,88	PASS	AC=2;AF=0
0	0	0	0	0	0	NA	NA	NA	NA	NA	798,63	PASS	AC=2;AF=0
0	0	0	6,154	0,96	35	COL4A1|0	Acceptor L	PP3	NA	NA	1270	PASS	AC=1;AF=0
0	0	0	4,567	0,917	32	MMP14|0	NA	NA	NA	NA	1417,01	PASS	AC=1;AF=0
0	0	0	4,356	0,894	30	AJUBA|0.0	NA	NA	NA	NA	1193,14	PASS	AC=1;AF=0
0	0,001	0,000599	4,416	0,903	31	ACIN1|0.0	NA	NA	NA	NA	155,35	PASS	AC=1;AF=0

0	0	0	5,321	0,941	33	ACIN1|0.0	Donor Gai	BP7	NA	NA	1216,68	PASS	AC=2;AF=0
0	0	0	0	0	0	IRF9|0.00|	Acceptor L	BP7	NA	NA	1715,14	PASS	AC=1;AF=0
0	0	0	4,624	0,921	32	NFATC4|0	Donor Los	VUS	NA	NA	430	PASS	AC=1;AF=0
0	0	0	4,425	0,904	31	FOXG1|0.0	NA	NA	hiConfDeN	1	2480	PASS	AC=1;AF=0
0	0	0	7,688	0,982	39	STRN3|0.0	Donor Los	PP3	NA	NA	294,72	PASS	AC=2;AF=0
0	0	0	4,402	0,901	31	ARHGAP5|	Acceptor G	BP7	NA	NA	929,8	PASS	AC=2;AF=0
0	0	0	0	0	0	AKAP6|0.0	NA	NA	NA	NA	388,94	PASS	AC=1;AF=0
0	0	0	0	0	0	AKAP6|0.0	Donor Los	PP3	NA	NA	820,7	PASS	AC=2;AF=0
0	0	0	7,412	0,977	38	NPAS3|0.0	Acceptor L	BP7	hiConfDeN	0,893028	416,04	PASS	AC=1;AF=0
0	0	0	4,758	0,926	32	NPAS3|0.0	Donor Gai	PP3	NA	NA	681	PASS	AC=1;AF=0
7,24E-05	0	0	0	0	0	NA	NA	NA	NA	NA	457821	PASS	AC=2;AF=0
0	0	0	7,958	0,987	40	ARF6|0.00	Donor Gai	PP3	NA	NA	2178,69	PASS	AC=2;AF=0
0	0	0	5,32	0,941	33	MAP4K5|0	Acceptor L	PP3	NA	NA	400,02	PASS	AC=1;AF=0
0	0	0	4,51	0,913	32	MAP4K5|0	NA	NA	NA	NA	622,26	PASS	AC=1;AF=0
0	0	0	4,687	0,923	32	FERMT2|0	Donor Los	PP3	NA	NA	601,01	PASS	AC=1;AF=0
0	0	0	6,271	0,961	35	DDHD1|0.	NA	NA	NA	NA	1718,71	PASS	AC=2;AF=0
0	0	0	0	0	0	DDHD1|0.	NA	NA	NA	NA	2938,05	PASS	AC=1;AF=0
0	0	0	4,425	0,904	31	SAMD4A|	NA	NA	NA	NA	261,02	PASS	AC=1;AF=0
0,0025	0	0,0002	0,494	0,091	6,438	SAMD4A|	NA	NA	NA	NA	895	PASS	AC=1;AF=0
0	0	0,000399	0	0	0	EXOC5|0.0	Acceptor L	PP3	NA	NA	364,08	PASS	AC=1;AF=0
0	0	0	4,74	0,925	32	PSMA3|0.	NA	NA	NA	NA	774,91	PASS	AC=2;AF=0
0	0	0	4,473	0,91	32	PPM1A|0.	Donor Gai	BP7	NA	NA	320,69	PASS	AC=2;AF=0
0	0	0	0	0	0	ZBTB1|0.0	NA	NA	NA	NA	3228,62	PASS	AC=2;AF=0
0	0	0	4,554	0,917	32	HSPA2|0.0	NA	NA	NA	NA	2796,68	PASS	AC=2;AF=0
0	0	0,000799	8,214	0,99	41	SPTB|0.00	NA	NA	NA	NA	1409,04	PASS	AC=1;AF=0
0	0	0,0002	4,427	0,905	31	SPTB|0.00	NA	NA	NA	NA	772,01	PASS	AC=1;AF=0
3,00E-04	0	0	0	0	0	SPTB|0.91	Acceptor G	PP3	NA	NA	2437,92	PASS	AC=2;AF=0
4,00E-04	0,001	0,0002	4,462	0,909	32	SPTB|0.03	Acceptor G	BP7	NA	NA	1261,75	PASS	AC=2;AF=0
0	0	0	4,577	0,918	32	ACTN1|0.0	Acceptor G	BP7	NA	NA	1142,69	PASS	AC=2;AF=0
0	0	0	4,372	0,897	31	ACTN1|0.0	NA	NA	hiConfDeN	0,999997	226	PASS	AC=1;AF=0
0	0	0	7,29	0,975	37	DCAF5|0.0	NA	NA	NA	NA	3270,84	PASS	AC=2;AF=0
0	0	0	7,33	0,976	37	SLC39A9|0	NA	NA	NA	NA	360,01	PASS	AC=1;AF=0
0	0,002	0,001198	0	0	0	SLC39A9|0	Donor Los	PP3	NA	NA	117,96	PASS	AC=2;AF=0
0	0	0	4,39	0,9	31	SRSF5|0.0	NA	NA	NA	NA	1180,69	PASS	AC=2;AF=0
0	0	0	4,369	0,896	31	SYNJ2BP-C	NA	NA	NA	NA	1116,68	PASS	AC=2;AF=0
0	0	0	4,549	0,916	32	SIPA1L1|0	Donor Los	PP3	NA	NA	591	PASS	AC=1;AF=0

0	0	0	4,402	0,901	31	RBM25|0.	Donor Los	BP7	NA	NA	385,03	PASS	AC=1;AF=0
0	0	0	4,358	0,894	30	YLPM1|0.0	Donor Los	BP7	NA	NA	1131,68	PASS	AC=2;AF=0
0	0	0	4,613	0,92	32	YLPM1|0.0	NA	NA	NA	NA	254	PASS	AC=1;AF=0
1,00E-04	0	0	4,362	0,895	31	TGFB3|0.0	NA	NA	NA	NA	1521,71	PASS	AC=2;AF=0
3,00E-04	0	0,0002	4,462	0,909	32	NRXN3|0.	NA	NA	NA	NA	286,07	PASS	AC=1;AF=0
0	0	0	1,845	0,276	18,07	NRXN3|0.	Donor Los	BP7	NA	NA	1012,54	PASS	AC=1;AF=0
0	0	0,0002	0	0	0	ZC3H14|0.	Acceptor	GVUS	NA	NA	1188,69	PASS	AC=2;AF=0
0	0	0	0,415	0,082	5,592	ZC3H14|0.	Acceptor L	PP3	NA	NA	743,01	PASS	AC=1;AF=0
0	0	0,001398	0	0	0	PSMC1|0.	Donor Los	PP3	NA	NA	415	PASS	AC=1;AF=0
3,00E-04	0	0,000399	4,502	0,913	32	TTC7B|0.0	NA	NA	NA	NA	1791,71	PASS	AC=2;AF=0
0	0	0	4,425	0,904	31	TTC7B|0.0	Donor Gai	BP7	NA	NA	1198,01	PASS	AC=1;AF=0
0	0	0	5,123	0,936	33	UNC79|0.	Acceptor L	BP7	NA	NA	1015,01	PASS	AC=1;AF=0
0	0	0	4,56	0,917	32	DDX24|0.0	Acceptor L	BP7	NA	NA	757,02	PASS	AC=1;AF=0
0	0	0	4,388	0,899	31	DDX24|0.0	Acceptor L	BP7	NA	NA	2626,02	PASS	AC=1;AF=0
0	0	0	4,466	0,909	32	CCDC85C|	NA	NA	NA	NA	1871,05	PASS	AC=2;AF=0
0	0	0	0	0	0	EML1|0.48	Acceptor L	PP3	NA	NA	232,96	PASS	AC=1;AF=0
0	0	0	4,507	0,913	32	EML1|0.00	Acceptor L	BP7	NA	NA	1871,02	PASS	AC=1;AF=0
0	0	0	4,488	0,911	32	DYNC1H1|	Donor Los	BP7	NA	NA	3682	PASS	AC=1;AF=0
0	0	0	0	0	0	NA	NA	NA	NA	NA	2162,16	PASS	AC=1;AF=0
0	0	0	0	0	0	HSP90AA1	Donor Los	BP7	NA	NA	2883,7	PASS	AC=2;AF=0
0	0	0	0	0	0	TECPR2|0.	Donor Gai	BP7	NA	NA	1302,63	PASS	AC=2;AF=0
0	0	0	0	0	0	TECPR2|0.	Donor Los	PP3	NA	NA	882,04	PASS	AC=2;AF=0
6,67E-05	0	0,0002	0	0	0	TRAF3|0.0	Donor Gai	PP3	NA	NA	867,83	PASS	AC=2;AF=0
0	0	0	4,493	0,912	32	KLC1|0.01	Acceptor G	BP7	NA	NA	679,03	PASS	AC=1;AF=0
0	0	0	4,566	0,917	32	KLC1|0.00	Donor Los	BP7	NA	NA	327,02	PASS	AC=1;AF=0
3,00E-04	0	0,000399	4,469	0,909	32	PPP1R13B	Donor Gai	VUS	NA	NA	4387,68	PASS	AC=2;AF=0
0	0,001	0,0002	0	0	0	PPP1R13B	Acceptor	GVUS	NA	NA	112,02	PASS	AC=1;AF=0
0	0	0	0	0	0	PPP1R13B	NA	NA	NA	NA	614,94	PASS	AC=1;AF=0
0	0	0	0	0	0	TMEM179	NA	NA	NA	NA	1886,89	PASS	AC=2;AF=0
0	0	0	8,022	0,987	40	INF2|0.00	Donor Los	BP7	NA	NA	944,04	PASS	AC=1;AF=0
6,67E-05	0	0	0	0	0	INF2|0.00	Donor Los	PP3	NA	NA	795	PASS	AC=1;AF=0
0	0	0	0	0	0	CEP170B|	Acceptor	GPP3	NA	NA	290,19	PASS	AC=1;AF=0
0	0	0	4,504	0,913	32	NA	NA	NA	hiConfDeN	0,999998	731	PASS	AC=1;AF=0
0	0	0	4,413	0,903	31	NA	NA	NA	NA	NA	803,01	PASS	AC=1;AF=0
0	0	0	4,472	0,91	32	CYFIP1|0.0	NA	NA	NA	NA	1670,69	PASS	AC=2;AF=0
0	0	0	4,402	0,901	31	UBE3A|0.0	Acceptor L	BP7	NA	NA	539	PASS	AC=1;AF=0

0	0	0	0	0	0	HERC2|0.0	Donor Gai	PP3	NA	NA	1417,68	PASS	AC=2;AF=0
0	0	0	5,258	0,939	33	HERC2|0.2	Acceptor L	PP3	NA	NA	1442,99	PASS	AC=1;AF=0
5,00E-04	0	0	0	0	0	APBA2|0.0	Donor Los	VUS	NA	NA	1262,12	PASS	AC=2;AF=0
9,00E-04	0	0,000799	4,358	0,894	30	TJP1|0.00	NA	NA	NA	NA	291	PASS	AC=1;AF=0
0	0	0	0	0	0	RYR3|0.00	Donor Los	PP3	NA	NA	734,01	PASS	AC=1;AF=0
0	0	0	4,485	0,911	32	RYR3|0.03	Acceptor L	BP7	NA	NA	567,99	PASS	AC=1;AF=0
0	0	0	5,148	0,936	33	RYR3|0.00	Donor Gai	PP3	NA	NA	1010,69	PASS	AC=2;AF=0
0	0	0	4,585	0,919	32	RYR3|0.00	Acceptor L	BP7	NA	NA	4168,7	PASS	AC=2;AF=0
0	0	0	4,54	0,916	32	RYR3|0.00	Acceptor L	VUS	NA	NA	1311	PASS	AC=1;AF=0
6,67E-05	0	0	4,515	0,914	32	RYR3|0.00	NA	NA	NA	NA	461	PASS	AC=1;AF=0
0	0	0	0	0	0	RYR3|0.00	Donor Gai	VUS	NA	NA	240,03	PASS	AC=1;AF=0
0	0	0	4,409	0,902	31	RYR3|0.00	Donor Gai	BP7	NA	NA	532,01	PASS	AC=1;AF=0
0	0	0	4,616	0,92	32	EMC4|0.0	Acceptor L	BP7	NA	NA	2589,68	PASS	AC=2;AF=0
6,66E-05	0	0	4,789	0,927	33	THBS1|0.0	Acceptor G	BP7	NA	NA	1199	PASS	AC=1;AF=0
0	0	0	7,004	0,971	37	THBS1|0.0	NA	NA	NA	NA	2868,68	PASS	AC=2;AF=0
0	0	0	0	0	0	NA	NA	NA	NA	NA	3239900	PASS	AC=1;AF=0
6,68E-05	0	0,001398	4,889	0,93	33	BAHD1|0.	NA	NA	NA	NA	2338,69	PASS	AC=2;AF=0
0	0	0,0002	4,892	0,93	33	SNAP23|0	Donor Los	BP7	NA	NA	717,01	PASS	AC=1;AF=0
0	0	0	4,452	0,908	32	TP53BP1|	NA	NA	NA	NA	4045	PASS	AC=1;AF=0
0	0	0	5,998	0,957	35	MAP1A|0.	Donor Los	PP3	NA	NA	767,05	PASS	AC=1;AF=0
0	0	0	4,364	0,895	31	PDIA3|0.0	Donor Los	BP7	NA	NA	425,01	PASS	AC=1;AF=0
0	0	0	4,438	0,906	31	SEMA6D|0	Acceptor L	BP7	NA	NA	1150	PASS	AC=1;AF=0
0	0	0	4,45	0,907	32	MYEF2|0.0	Acceptor G	BP7	NA	NA	488	PASS	AC=1;AF=0
0	0	0	0	0	0	FBN1|0.00	Donor Gai	PP3	NA	NA	591,02	PASS	AC=1;AF=0
0	0	0,001198	0	0	0	USP8|0.00	Donor Los	PP3	NA	NA	612,34	PASS	AC=2;AF=0
0	0	0	0	0	0	USP8|0.00	Acceptor L	VUS	NA	NA	1876,15	PASS	AC=2;AF=0
0	0	0	0	0	0	NA	NA	NA	NA	NA	3632,63	PASS	AC=2;AF=0
0	0	0	4,375	0,897	31	LEO1|0.00	NA	NA	NA	NA	1887,69	PASS	AC=2;AF=0
6,67E-05	0	0	4,492	0,912	32	RFX7|0.00	NA	NA	NA	NA	1607	PASS	AC=1;AF=0
6,66E-05	0	0	0	0	0	SLTM|0.14	Acceptor G	VUS	NA	NA	1519,69	PASS	AC=2;AF=0
0	0	0	4,503	0,913	32	FOXB1|0.0	Acceptor G	BP7	NA	NA	2945,68	PASS	AC=2;AF=0
0	0	0	4,401	0,901	31	TLN2|0.00	NA	NA	NA	NA	539	PASS	AC=1;AF=0
0	0	0	0	0	0	TLN2|0.00	NA	NA	NA	NA	188,96	PASS	AC=1;AF=0
0	0	0	4,512	0,913	32	TLN2|0.00	NA	NA	NA	NA	5239,82	PASS	AC=4;AF=0
0	0	0,000399	0	0	0	HERC1|0.0	Acceptor L	VUS	NA	NA	1320,02	PASS	AC=2;AF=0
0	0	0	4,644	0,922	32	HERC1|0.0	Acceptor L	PP3	NA	NA	331,14	PASS	AC=1;AF=0

0	0	0	0	0	0	HERC1|0.0	Donor Los	PP3	NA	NA	572,07	PASS	AC=1;AF=0
6,67E-05	0	0	4,383	0,898	31	SNX1|0.00	NA	NA	NA	NA	1370,68	PASS	AC=2;AF=0
0	0	0	4,547	0,916	32	RPL4|0.01	Donor Gai	BP7	NA	NA	584,02	PASS	AC=1;AF=0
0	0	0	0	0	0	SMAD3|0.	Acceptor G	BP7	NA	NA	552,94	PASS	AC=1;AF=0
6,67E-05	0	0	0	0	0	TLE3|0.00	Acceptor L	VUS	NA	NA	1696,82	PASS	AC=2;AF=0
0	0	0	4,533	0,915	32	TLE3|0.00	NA	NA	NA	NA	2336,73	PASS	AC=2;AF=0
0	0	0	1,677	0,247	16,87	TLE3|0.00	Donor Los	BP7	NA	NA	1358,71	PASS	AC=2;AF=0
6,69E-05	0	0	0	0	0	PKM|0.00	Donor Gai	PP3	NA	NA	1016,73	PASS	AC=2;AF=0
0	0	0	4,526	0,915	32	PARP6|0.4	Acceptor G	PP3	NA	NA	1142,68	PASS	AC=2;AF=0
0	0	0	4,522	0,914	32	PARP6|0.0	Acceptor L	VUS	NA	NA	1062,69	PASS	AC=2;AF=0
6,67E-05	0	0,0002	4,546	0,916	32	LOXL1|0.0	NA	NA	NA	NA	2403,68	PASS	AC=2;AF=0
0	0	0	0	0	0	CSK|0.00|	Donor Gai	PP3	NA	NA	2118	PASS	AC=1;AF=0
0	0	0	0	0	0	HMG20A|	Acceptor G	PP3	NA	NA	1950,94	PASS	AC=1;AF=0
0	0	0,0002	0	0	0	HMG20A|	Donor Los	VUS	NA	NA	546,13	PASS	AC=1;AF=0
0	0	0	4,42	0,904	31	IREB2|0.0	Donor Los	PP3	NA	NA	360,03	PASS	AC=1;AF=0
0	0	0	4,68	0,923	32	AP3B2|0.0	Donor Los	PP3	NA	NA	1110,69	PASS	AC=2;AF=0
0	0	0	0	0	0	AP3B2|0.0	Acceptor L	BP7	NA	NA	20603,1	PASS	AC=2;AF=0
0	0	0	4,493	0,912	32	BNC1|0.00	NA	NA	NA	NA	1392,01	PASS	AC=1;AF=0
0	0	0	4,365	0,895	31	AKAP13|0	Acceptor L	BP7	NA	NA	697	PASS	AC=1;AF=0
3,00E-04	0,001	0	4,349	0,893	30	AKAP13|0	NA	NA	NA	NA	2978,68	PASS	AC=2;AF=0
0	0	0	4,448	0,907	32	AKAP13|0	Acceptor L	VUS	NA	NA	325,03	PASS	AC=1;AF=0
0	0,001	0,002796	4,355	0,894	30	ACAN|0.0	NA	NA	NA	NA	2010,68	PASS	AC=2;AF=0
1,00E-04	0	0	0	0	0	IDH2|0.00	Donor Gai	PP3	NA	NA	1876,71	PASS	AC=2;AF=0
0	0	0	0	0	0	IDH2|0.11	Acceptor G	VUS	NA	NA	3766	PASS	AC=1;AF=0
0	0	0	4,481	0,911	32	IDH2|0.02	Acceptor G	BP7	NA	NA	763,02	PASS	AC=1;AF=0
0	0	0	4,52	0,914	32	IQGAP1|0.	NA	NA	NA	NA	977,05	PASS	AC=2;AF=0
2,00E-04	0	0,0002	4,567	0,917	32	IQGAP1|0.	NA	NA	NA	NA	1158,69	PASS	AC=2;AF=0
0	0	0,0002	4,786	0,927	33	IQGAP1|0.	Acceptor G	PP3	NA	NA	619,01	PASS	AC=1;AF=0
6,67E-05	0	0	4,52	0,914	32	CRTC3|0.0	Acceptor L	PP3	NA	NA	283	PASS	AC=1;AF=0
6,00E-04	0,001	0,000599	4,483	0,911	32	CRTC3|0.0	Donor Los	BP7	NA	NA	198,01	PASS	AC=1;AF=0
6,67E-05	0	0	4,806	0,927	33	CRTC3|0.2	Acceptor L	PP3	NA	NA	2577,69	PASS	AC=2;AF=0
0	0	0	0	0	0	NR2F2|0.0	Donor Los	PP3	NA	NA	991,7	PASS	AC=2;AF=0
0	0	0	7,864	0,985	39	IGF1R|0.0	Donor Gai	BP7	NA	NA	1975,05	PASS	AC=1;AF=0
0	0	0	4,379	0,898	31	MEF2A|0.	Acceptor L	BP7	NA	NA	2167,16	PASS	AC=3;AF=0
0	0	0	4,389	0,899	31	RAB11FIP3	Acceptor G	BP7	NA	NA	1617,68	PASS	AC=2;AF=0
0	0	0	4,372	0,897	31	CAPN15|0	Acceptor L	BP7	NA	NA	421,72	PASS	AC=2;AF=0

1,00E-04	0	0	0	0	0	UNKL|0.00	NA	NA	NA	NA	973,96	PASS	AC=1;AF=0
7,00E-04	0	0	0	0	0	NA	NA	NA	NA	NA	18768,5	PASS	AC=1;AF=0
0	0	0	0	0	0	MAPK8IP3	Donor Gai	PP3	NA	NA	17768,2	PASS	AC=1;AF=0
0	0	0	4,401	0,901	31	MAPK8IP3	Acceptor G	BP7	NA	NA	2506,73	PASS	AC=2;AF=0
0	0	0	9,239	0,998	48	ZNF598|0.	NA	NA	NA	NA	1179,09	PASS	AC=1;AF=0
0	0	0	4,37	0,896	31	TSC2|0.00	Acceptor L	BP7	NA	NA	1380,69	PASS	AC=2;AF=0
0	0	0,000399	4,671	0,923	32	TSC2|0.02	Acceptor G	BP7	NA	NA	1572,09	PASS	AC=1;AF=0
6,69E-05	0	0	0	0	0	PKD1|0.47	Acceptor G	PP3	NA	NA	2229,47	PASS	AC=2;AF=0
0	0	0	8,4	0,992	42	PKD1|0.00	Donor Gai	BP7	NA	NA	199,28	PASS	AC=1;AF=0
0	0	0	7,768	0,983	39	CASKIN1|0	Acceptor G	BP7	NA	NA	1903,11	PASS	AC=1;AF=0
0	0	0	4,366	0,896	31	CREBBP|0.	NA	NA	NA	NA	828,01	PASS	AC=1;AF=0
0	0	0,0002	0	0	0	CREBBP|0.	Donor Gai	PP3	NA	NA	1084,78	PASS	AC=2;AF=0
0	0	0	0	0	0	NA	NA	NA	loConfDeN	NA	4547,89	PASS	AC=1;AF=0
6,71E-05	0	0	0	0	0	CDIP1|0.0	Acceptor L	VUS	NA	NA	2871,06	PASS	AC=2;AF=0
6,66E-05	0,001	0	0	0	0	UBN1|0.1	Acceptor G	VUS	NA	NA	384,03	PASS	AC=1;AF=0
0	0	0	0	0	0	C16orf72|	Acceptor L	PP3	NA	NA	1327,35	PASS	AC=2;AF=0
0	0	0	4,486	0,911	32	GRIN2A|0.	NA	NA	NA	NA	178,01	PASS	AC=1;AF=0
0	0	0	0	0	0	GSPT1|0.0	Donor Gai	BP7	hiConfDeN	NA	134,48	PASS	AC=1;AF=0
0	0	0	0	0	0	GSPT1|0.0	Donor Gai	BP7	hiConfDeN	NA	125,14	PASS	AC=1;AF=0
0	0	0	2,708	0,458	22,8	MYH11|0.	Acceptor L	PP3	NA	NA	977,07	PASS	AC=1;AF=0
0	0	0	4,353	0,893	30	MYH11|0.	NA	NA	NA	NA	1319,01	PASS	AC=1;AF=0
0	0	0	0	0	0	MYH11|0.	NA	NA	NA	NA	2097,74	PASS	AC=2;AF=0
9,00E-04	0	0,0002	4,43	0,905	31	MYH11|0.	NA	NA	NA	NA	1750	PASS	AC=1;AF=0
0,0021	0,001	0,000599	4,379	0,898	31	MYH11|0.	NA	NA	NA	NA	1569	PASS	AC=1;AF=0
6,67E-05	0	0	7,004	0,971	37	XYLT1|0.0	Donor Los	BP7	NA	NA	837,02	PASS	AC=1;AF=0
6,67E-05	0	0,0002	4,445	0,907	31	NA	NA	NA	NA	NA	1283	PASS	AC=1;AF=0
0	0	0	1,199	0,176	13,63	NA	NA	NA	NA	NA	1229,74	PASS	AC=2;AF=0
0,0016	0,001	0	4,467	0,909	32	METTL9|0	Donor Los	BP7	NA	NA	1620,07	PASS	AC=2;AF=0
0	0	0	4,409	0,902	31	PLK1|0.00	Acceptor L	BP7	NA	NA	684,01	PASS	AC=1;AF=0
0	0	0	4,35	0,893	30	XPO6|0.01	Acceptor L	BP7	NA	NA	609,16	PASS	AC=1;AF=0
1,00E-04	0	0,0002	0	0	0	XPO6|0.00	Donor Los	VUS	NA	NA	930,8	PASS	AC=2;AF=0
0	0	0	0	0	0	AC009133.	Donor Los	PP3	NA	NA	2562,7	PASS	AC=2;AF=0
1,00E-04	0	0	0	0	0	AC009133.	Acceptor L	VUS	NA	NA	1709,7	PASS	AC=2;AF=0
0	0	0	4,248	0,869	29,1	RP11-2C24	Donor Los	BP7	NA	NA	840,02	PASS	AC=1;AF=0
0	0	0	0	0	0	NA	NA	NA	hiConfDeN	0,995461	1914,07	PASS	AC=1;AF=0
6,67E-05	0	0,000399	4,366	0,896	31	FUS|0.00|	NA	NA	NA	NA	1196,29	PASS	AC=1;AF=0

0	0	0	0	0	0	VPS35|0.0	Donor Los	PP3	NA	NA	2372,82	PASS	AC=2;AF=0
0	0	0	5,502	0,946	34	BRD7|0.00	Acceptor L	PP3	NA	NA	1216,94	PASS	AC=4;AF=0
0	0	0	0	0	0	RBL2|0.00	Donor Los	PP3	NA	NA	397,72	PASS	AC=2;AF=0
0	0	0	4,675	0,923	32	IRX3|0.00	Donor Gai	BP7	NA	NA	4258,7	PASS	AC=2;AF=0
1,00E-04	0	0,000399	4,503	0,913	32	MMP2|0.0	NA	NA	NA	NA	3007,7	PASS	AC=2;AF=0
0	0	0	0	0	0	HERPUD1|	Acceptor L	PP3	NA	NA	959,68	PASS	AC=2;AF=0
0	0	0	0	0	0	CDH11|0.0	Acceptor L	PP3	NA	NA	410,11	PASS	AC=1;AF=0
0,0011	0	0	4,617	0,92	32	DYNC1LI2|	Acceptor L	VUS	NA	NA	668,69	PASS	AC=2;AF=0
0	0	0	4,509	0,913	32	EDC4|0.00	Donor Gai	PP3	NA	NA	2629	PASS	AC=1;AF=0
0	0	0,0002	5,441	0,944	34	NFAT5|0.0	Acceptor L	PP3	NA	NA	821,02	PASS	AC=1;AF=0
0	0	0	4,621	0,92	32	AP1G1|0.0	Acceptor L	BP7	NA	NA	3176,69	PASS	AC=2;AF=0
6,66E-05	0	0	4,411	0,903	31	ZFHX3|0.0	NA	NA	hiConfDeN	0,999998	960,03	PASS	AC=1;AF=0
0	0	0	4,977	0,932	33	GLG1|0.01	Acceptor G	BP7	NA	NA	557,19	PASS	AC=1;AF=0
0	0	0	0	0	0	GLG1|0.00	Donor Los	VUS	NA	NA	884,04	PASS	AC=1;AF=0
0	0	0	7,89	0,985	40	BCAR1|0.0	NA	NA	hiConfDeN	0,999998	477,11	PASS	AC=1;AF=0
0	0	0	0	0	0	NA	NA	NA	NA	NA	679	PASS	AC=4;AF=0
0	0	0	4,568	0,917	32	BCAR1|0.0	Donor Los	BP7	NA	NA	472,18	PASS	AC=1;AF=0
0	0	0,0002	4,373	0,897	31	PLCG2|0.0	NA	NA	NA	NA	845,68	PASS	AC=2;AF=0
0	0	0	4,438	0,906	31	PLCG2|0.0	Donor Los	PP3	NA	NA	1039,69	PASS	AC=2;AF=0
0	0	0	0	0	0	PLCG2|0.0	Donor Los	VUS	NA	NA	1457,62	PASS	AC=2;AF=0
0	0	0	4,59	0,919	32	USP10|0.0	NA	NA	NA	NA	747,38	PASS	AC=3;AF=0
0,0017	0,001	0,0002	0	0	0	USP10|0.0	Donor Los	VUS	NA	NA	1458,69	PASS	AC=2;AF=0
0	0	0	4,501	0,912	32	GSE1|0.00	NA	NA	NA	NA	1500,09	PASS	AC=1;AF=0
0	0	0	5,686	0,951	34	GSE1|0.50	Acceptor L	PP3	NA	NA	806,03	PASS	AC=2;AF=0
6,67E-05	0	0,0002	0	0	0	EMC8|0.0	Donor Los	VUS	NA	NA	2462,71	PASS	AC=2;AF=0
0	0	0	7,648	0,981	39	ZCCHC14|	NA	NA	NA	NA	2973,68	PASS	AC=2;AF=0
0	0	0	0	0	0	BANP|0.00	Donor Gai	PP3	NA	NA	2129,79	PASS	AC=2;AF=0
0	0	0	0	0	0	ZNF469|0.	Acceptor G	BP7	NA	NA	1561,08	PASS	AC=1;AF=0
6,67E-05	0	0	0	0	0	ZNF469|0.	NA	NA	NA	NA	3172,03	PASS	AC=1;AF=0
0	0	0	0	0	0	ANKRD11|	Acceptor G	VUS	NA	NA	7876,83	PASS	AC=4;AF=0
0	0	0	7,669	0,982	39	PITPNA|0.	Donor Los	BP7	NA	NA	340,71	PASS	AC=2;AF=0
0	0	0	0	0	0	PRPF8|0.3	Donor Los	PP3	NA	NA	51,01	PASS	AC=1;AF=0
0	0	0	0	0	0	PRPF8|0.0	Donor Los	PP3	NA	NA	2165,69	PASS	AC=2;AF=0
0	0	0	0	0	0	PAFAH1B1	NA	NA	NA	NA	1099,01	PASS	AC=1;AF=0
6,75E-05	0	0	4,439	0,906	31	RAP1GAP2	NA	NA	NA	NA	1574,69	PASS	AC=2;AF=0
0	0	0	4,519	0,914	32	ZZEF1|0.1	Donor Los	PP3	NA	NA	910	PASS	AC=1;AF=0

0	0,001	0,000799	4,579	0,918	32	ZZEF1|0.0	NA	NA	NA	NA	568,11	PASS	AC=1;AF=0
0	0	0	8,209	0,99	41	ZZEF1|0.0	NA	NA	NA	NA	1769,79	PASS	AC=2;AF=0
0	0	0	4,362	0,895	31	ZZEF1|0.0	NA	NA	NA	NA	3190,92	PASS	AC=2;AF=0
0	0	0	4,395	0,9	31	ANKFY1|0	Acceptor G	BP7	NA	NA	430	PASS	AC=1;AF=0
0	0	0	0	0	0	MINK1|0.0	Acceptor L	PP3	NA	NA	2094,69	PASS	AC=2;AF=0
6,68E-05	0	0	4,49	0,911	32	MINK1|0.0	NA	NA	NA	NA	1034,72	PASS	AC=2;AF=0
0	0	0	0	0	0	RABEP1|0.	Donor Los	VUS	NA	NA	584,59	PASS	AC=1;AF=0
0	0	0	4,398	0,901	31	RABEP1|0.	Acceptor L	VUS	NA	NA	584	PASS	AC=1;AF=0
0	0	0	0	0	0	EIF5A|0.02	Acceptor L	PP3	NA	NA	422,19	PASS	AC=1;AF=0
0	0	0	0	0	0	NEURL4|0	Acceptor L	VUS	NA	NA	2342,68	PASS	AC=2;AF=0
0	0	0	0	0	0	ZBTB4|0.0	NA	NA	NA	NA	1369,94	PASS	AC=1;AF=0
0	0	0	0	0	0	ZBTB4|0.0	NA	NA	NA	NA	1893,95	PASS	AC=1;AF=0
0	0	0	0	0	0	ZBTB4|0.0	Acceptor L	BP7	NA	NA	1959,17	PASS	AC=2;AF=0
0	0	0	4,396	0,9	31	POLR2A|0	NA	NA	NA	NA	197,18	PASS	AC=1;AF=0
0	0	0	6,505	0,964	36	TNFSF12-T	Donor Gai	BP7	NA	NA	3349	PASS	AC=2;AF=0
0	0	0,000799	0	0	0	TNFSF12-T	Donor Los	VUS	NA	NA	1040,67	PASS	AC=1;AF=0
0	0	0	4,543	0,916	32	TNFSF12-T	Acceptor L	BP7	NA	NA	1322,04	PASS	AC=2;AF=0
0	0	0	4,518	0,914	32	CHD3|0.00	NA	NA	NA	NA	306,01	PASS	AC=1;AF=0
0	0	0	0	0	0	RP11-849F	Donor Gai	PP3	NA	NA	164,57	PASS	AC=1;AF=0
6,67E-05	0,001	0	4,519	0,914	32	MYH10|0.	Acceptor L	BP7	NA	NA	3842,76	PASS	AC=2;AF=0
0	0	0	4,822	0,928	33	MYH10|0.	Acceptor G	BP7	NA	NA	1164,69	PASS	AC=2;AF=0
0	0	0	4,631	0,921	32	ARHGAP44	Donor Los	BP7	NA	NA	591,75	PASS	AC=2;AF=0
0	0	0,000599	4,618	0,92	32	NCOR1|0.	Acceptor L	PP3	NA	NA	609,68	PASS	AC=2;AF=0
0	0	0,000399	0,217	0,061	3,322	FLCN|0.00	Donor Gai	BP7	NA	NA	2644	PASS	AC=1;AF=0
0,001	0,001	0,000799	0,024	0,044	1,492	FLCN|0.00	Acceptor L	BP7	NA	NA	3499,02	PASS	AC=1;AF=0
6,66E-05	0	0	4,396	0,9	31	FLCN|0.00	Donor Gai	PP3	NA	NA	3448,97	PASS	AC=2;AF=0
0	0	0	0	0	0	NA	NA	NA	NA	NA	1057,79	PASS	AC=2;AF=0
0	0	0	4,39	0,899	31	RPL23A|0.	Donor Gai	BP7	NA	NA	1071,02	PASS	AC=1;AF=0
0	0	0	4,352	0,893	30	RPL23A|0.	NA	NA	NA	NA	992,86	PASS	AC=2;AF=0
0,0017	0,0061	0,000399	4,473	0,91	32	TRAF4|0.0	NA	NA	NA	NA	1223	PASS	AC=1;AF=0
0	0	0	4,441	0,906	31	PHF12|0.0	NA	NA	NA	NA	671,01	PASS	AC=1;AF=0
0	0	0,001797	0	0	0	MYO18A|	NA	NA	NA	NA	1341,99	PASS	AC=2;AF=0
6,68E-05	0	0	4,356	0,894	30	MYO18A|	Donor Los	BP7	NA	NA	1980,69	PASS	AC=2;AF=0
0	0	0	4,648	0,922	32	MYO18A|	Acceptor G	BP7	NA	NA	1105,71	PASS	AC=2;AF=0
0	0	0	4,372	0,897	31	MYO18A|	Acceptor L	PP3	NA	NA	2278	PASS	AC=1;AF=0
0	0	0	0	0	0	GIT1|0.18	Acceptor G	VUS	NA	NA	2393,63	PASS	AC=2;AF=0

0	0	0	4,357	0,894	30	GIT1|0.00	Donor Los	BP7	NA	NA	1323	PASS	AC=1;AF=0
0	0	0	8,51	0,994	43	TEFM|0.00	NA	NA	NA	NA	217,02	PASS	AC=1;AF=0
0	0	0	4,519	0,914	32	NF1|0.00|	NA	NA	NA	NA	683,69	PASS	AC=2;AF=0
0	0	0	0	0	0	SUZ12|0.0	NA	NA	NA	NA	556,94	PASS	AC=1;AF=0
0	0	0	4,42	0,904	31	RHOT1|0.	Acceptor L	BP7	NA	NA	2021,82	PASS	AC=2;AF=0
0	0	0	0	0	0	ASIC2|0.0	NA	NA	NA	NA	643,94	PASS	AC=1;AF=0
0,0037	0,001	0,000399	4,45	0,907	32	ASIC2|0.0	Acceptor L	BP7	NA	NA	2434,7	PASS	AC=2;AF=0
0	0	0	4,348	0,892	30	AP2B1|0.0	NA	NA	NA	NA	722,01	PASS	AC=1;AF=0
0	0	0	7,899	0,986	40	ACACA|0.1	Acceptor G	VUS	NA	NA	611	PASS	AC=1;AF=0
0	0	0	0	0	0	SYNRG|0.0	Donor Los	PP3	NA	NA	555	PASS	AC=1;AF=0
6,67E-05	0	0,0002	4,388	0,899	31	SYNRG|0.0	Acceptor L	PP3	NA	NA	1327,85	PASS	AC=4;AF=0
0	0	0	0	0	0	SYNRG|0.0	Donor Gai	PP3	NA	NA	2221,69	PASS	AC=2;AF=0
6,74E-05	0	0	5,57	0,948	34	LASP1|0.0	Donor Los	PP3	NA	NA	26,1	PASS	AC=1;AF=0
6,69E-05	0	0,0002	4,409	0,902	31	CACNB1|0	Donor Gai	BP7	NA	NA	1600,01	PASS	AC=1;AF=0
6,72E-05	0	0,001398	0	0	0	CACNB1|0	Acceptor L	PP3	NA	NA	1531,53	PASS	AC=2;AF=0
0	0,001	0	0	0	0	CDK12|0.9	Acceptor G	PP3	NA	NA	780,11	PASS	AC=1;AF=0
2,00E-04	0	0	0	0	0	NA	NA	NA	NA	NA	585,63	PASS	AC=2;AF=0
0	0	0	5,723	0,952	34	ACLY|0.00	Donor Los	PP3	NA	NA	1098	PASS	AC=1;AF=0
0,0019	0	0,000399	4,352	0,893	30	ACLY|0.00	NA	NA	NA	NA	666	PASS	AC=1;AF=0
0	0	0	5,056	0,934	33	KCNH4|0.	Donor Los	PP3	NA	NA	228,76	PASS	AC=2;AF=0
0,0029	0,0041	0,001198	0	0	0	STAT5B|0.	Acceptor G	VUS	NA	NA	1650	PASS	AC=1;AF=0
2,00E-04	0	0	4,581	0,918	32	STAT5B|0.	NA	NA	NA	NA	820,73	PASS	AC=2;AF=0
0	0	0	4,438	0,906	31	STAT3|0.0	NA	NA	NA	NA	3480,87	PASS	AC=2;AF=0
3,00E-04	0,001	0	4,509	0,913	32	ATP6V0A1	NA	NA	NA	NA	980,69	PASS	AC=2;AF=0
0	0	0	4,725	0,925	32	RPL27|0.0	NA	NA	NA	NA	697,69	PASS	AC=2;AF=0
0	0	0	4,389	0,899	31	HDAC5|0.	Acceptor G	BP7	NA	NA	1554,69	PASS	AC=2;AF=0
0	0	0,0002	0	0	0	RUNDC3A	Acceptor G	VUS	NA	NA	2820,7	PASS	AC=2;AF=0
0	0	0	4,535	0,915	32	FZD2|0.00	NA	NA	NA	NA	981,68	PASS	AC=2;AF=0
0	0	0	4,432	0,905	31	FMNL1|0.	NA	NA	NA	NA	4603	PASS	AC=1;AF=0
7,00E-04	0,001	0,0002	4,498	0,912	32	FMNL1|0.	Acceptor G	BP7	NA	NA	2254,51	PASS	AC=2;AF=0
8,00E-04	0,001	0,000399	4,408	0,902	31	FMNL1|0.	Donor Los	BP7	NA	NA	619	PASS	AC=1;AF=0
0	0	0,000799	0	0	0	CDC27|0.0	Donor Gai	PP3	NA	NA	112,12	PASS	AC=1;AF=0
0	0	0	0	0	0	CDC27|0.0	NA	NA	NA	NA	339,01	PASS	AC=1;AF=0
0	0	0	0	0	0	NPEPPS|0.	NA	NA	NA	NA	4623,91	PASS	AC=2;AF=0
0	0	0	4,36	0,894	31	HOXB3|0.	NA	NA	NA	NA	1340,69	PASS	AC=2;AF=0
0	0	0	0	0	0	HOXB3|0.	Acceptor G	VUS	NA	NA	713,13	PASS	AC=1;AF=0

0	0,001	0,002995	0	0	0	SPOP|0.00	Acceptor L	PP3	NA	NA	426,95	PASS	AC=1;AF=0
6,69E-05	0	0,000399	0	0	0	COL1A1|0	Donor Los	VUS	NA	NA	711,01	PASS	AC=1;AF=0
6,68E-05	0	0	4,885	0,93	33	COL1A1|0	Acceptor G	BP7	NA	NA	2462,74	PASS	AC=2;AF=0
0	0	0,0002	4,392	0,9	31	COL1A1|0	NA	NA	NA	NA	1447,73	PASS	AC=2;AF=0
0	0	0	5,064	0,934	33	COL1A1|0	NA	NA	NA	NA	2788,69	PASS	AC=2;AF=0
0	0	0	4,375	0,897	31	XYLT2|0.0	NA	NA	NA	NA	1656,02	PASS	AC=1;AF=0
0	0	0	4,365	0,895	31	MRPL27|0	Acceptor G	BP7	NA	NA	1368,68	PASS	AC=2;AF=0
0	0	0	5,761	0,952	34	MRPL27|0	Donor Gai	BP7	NA	NA	385,01	PASS	AC=1;AF=0
2,00E-04	0	0	5,221	0,938	33	CACNA1G|	Donor Los	PP3	NA	NA	592,03	PASS	AC=1;AF=0
0	0	0	4,497	0,912	32	SPAG9|0.0	Acceptor L	BP7	NA	NA	764,01	PASS	AC=1;AF=0
0	0	0	4,523	0,914	32	AKAP1|0.0	NA	NA	NA	NA	1029,99	PASS	AC=1;AF=0
0	0	0	4,571	0,918	32	MSI2|0.00	Donor Los	BP7	NA	NA	908	PASS	AC=1;AF=0
1,00E-04	0	0	0	0	0	VEZF1|0.0	NA	NA	NA	NA	885,43	PASS	AC=1;AF=0
0	0	0	4,777	0,927	33	MTMR4|0	NA	NA	NA	NA	1227,7	PASS	AC=2;AF=0
0	0	0	0	0	0	NA	NA	NA	NA	NA	1703,63	PASS	AC=2;AF=0
0	0	0	0	0	0	CLTC|0.00	Donor Gai	VUS	NA	NA	680,01	PASS	AC=1;AF=0
0	0	0	6,405	0,963	36	TLK2|0.00	Acceptor L	BP7	hiConfDeN	0,999997	518	PASS	AC=1;AF=0
6,67E-05	0	0	4,526	0,915	32	MRC2|0.0	Donor Gai	VUS	NA	NA	662,94	PASS	AC=2;AF=0
0	0	0	0	0	0	CYB561|0.	Acceptor L	PP3	NA	NA	1211,06	PASS	AC=1;AF=0
0	0	0	4,414	0,903	31	DCAF7|0.0	Donor Gai	BP7	NA	NA	1618,24	PASS	AC=2;AF=0
0	0	0,0002	4,58	0,918	32	MAP3K3|0	Acceptor L	BP7	NA	NA	1043,69	PASS	AC=2;AF=0
0	0	0	4,883	0,929	33	ERN1|0.00	NA	NA	NA	NA	2238,71	PASS	AC=2;AF=0
0	0	0	4,394	0,9	31	DDX5|0.00	NA	NA	NA	NA	666	PASS	AC=1;AF=0
6,66E-05	0	0	0	0	0	PSMD12|0	Acceptor G	VUS	NA	NA	275	PASS	AC=1;AF=0
6,68E-05	0	0	0	0	0	BPTF|0.00	NA	NA	NA	NA	193,04	PASS	AC=1;AF=0
0	0	0	4,495	0,912	32	BPTF|0.00	NA	NA	NA	NA	387,03	PASS	AC=1;AF=0
0	0	0	0	0	0	NA	NA	NA	NA	NA	1617,19	PASS	AC=2;AF=0
0	0	0	4,491	0,912	32	NUP85|0.	Acceptor G	BP7	NA	NA	2673,69	PASS	AC=2;AF=0
0	0	0,001797	0	0	0	SEC14L1|0	Donor Gai	PP3	NA	NA	105,41	PASS	AC=1;AF=0
0	0	0	9,027	0,997	47	SEC14L1|0	Donor Los	PP3	NA	NA	809,68	PASS	AC=2;AF=0
0	0	0	4,536	0,915	32	SOCS3|0.0	NA	NA	NA	NA	2409,7	PASS	AC=2;AF=0
0	0	0	5,966	0,957	35	CYTH1|0.9	Acceptor L	PP3	NA	NA	1394,7	PASS	AC=2;AF=0
7,11E-05	0	0,006789	0	0	0	CYTH1|0.0	Donor Gai	VUS	NA	NA	195,41	PASS	AC=2;AF=0
0	0	0	4,462	0,909	32	CBX2|0.00	NA	NA	NA	NA	1134,7	PASS	AC=2;AF=0
0	0	0,000998	0	0	0	EIF4A3|0.0	Donor Gai	VUS	NA	NA	298	PASS	AC=1;AF=0
0	0	0	5,489	0,945	34	FASN|0.00	Donor Los	PP3	NA	NA	2343,01	PASS	AC=1;AF=0

0	0	0	0	0	0	CSNK1D|0	Donor Los	PP3	NA	NA	639,94	PASS	AC=1;AF=0
0	0	0	4,453	0,908	32	THOC1|0.	NA	NA	NA	NA	839,78	PASS	AC=2;AF=0
0	0	0	4,411	0,903	31	TYMS|0.03	Acceptor	GBP7	NA	NA	374	PASS	AC=1;AF=0
6,66E-05	0	0	4,357	0,894	30	SMCHD1|	Acceptor	GBP7	NA	NA	627	PASS	AC=1;AF=0
0	0	0	4,89	0,93	33	DLGAP1|0	NA	NA	NA	NA	1134,15	PASS	AC=1;AF=0
0	0	0	4,363	0,895	31	PTPRM|0.	NA	NA	NA	NA	1179,75	PASS	AC=2;AF=0
0	0	0	0	0	0	NA	NA	NA	NA	NA	1763,94	PASS	AC=1;AF=0
0	0	0	0	0	0	NA	NA	NA	NA	NA	132,96	PASS	AC=1;AF=0
0	0	0	4,503	0,913	32	PPP4R1|0.	NA	NA	NA	NA	1072,71	PASS	AC=2;AF=0
0	0	0	4,396	0,9	31	PIEZO2|0.	NA	NA	NA	NA	451	PASS	AC=1;AF=0
6,66E-05	0	0	4,503	0,913	32	CHMP1B|	NA	NA	NA	NA	2379,69	PASS	AC=2;AF=0
0	0	0	5,464	0,945	34	LDLRAD4|	Donor Los	PP3	NA	NA	615	PASS	AC=1;AF=0
6,67E-05	0	0	4,421	0,904	31	GREB1L|0.	Acceptor L	BP7	NA	NA	986,02	PASS	AC=1;AF=0
0	0	0	0	0	0	ESCO1|0.0	NA	NA	NA	NA	8171,51	PASS	AC=1;AF=0
0	0	0	0	0	0	ESCO1|0.0	Acceptor L	VUS	NA	NA	472,68	PASS	AC=1;AF=0
0	0	0	4,371	0,896	31	MIB1|0.51	Acceptor	GPP3	NA	NA	1822,68	PASS	AC=2;AF=0
0	0	0	0	0	0	ASXL3|0.0	NA	NA	hiConfDeN	0,698864	2665,98	PASS	AC=1;AF=0
0	0	0	0	0	0	NA	NA	NA	hiConfDeN	0,766198	2625,94	PASS	AC=1;AF=0
0,0013	0,001	0,001198	4,781	0,927	33	DTNA|0.0	Donor Los	BP7	NA	NA	1325,7	PASS	AC=2;AF=0
0	0	0	0	0	0	SETBP1|0.	NA	NA	NA	NA	1732,64	PASS	AC=2;AF=0
3,00E-04	0	0	0	0	0	RNF165|0.	Acceptor L	PP3	NA	NA	687	PASS	AC=1;AF=0
0,0013	0,001	0	0	0	0	PIAS2|0.0	Acceptor L	BP7	NA	NA	293,13	PASS	AC=1;AF=0
0	0	0,000399	-0,443	0,018	0,157	SKOR2|0.0	Acceptor L	BP7	NA	NA	1290,04	PASS	AC=2;AF=0
0	0	0	9,701	0,999	51	SKOR2|0.0	Donor Los	PP3	NA	NA	1305,68	PASS	AC=1;AF=0
0	0	0,003395	4,429	0,905	31	SMAD7|0.	NA	NA	NA	NA	1093,6	PASS	AC=1;AF=0
0	0,001	0,0002	0	0	0	RPL17|0.0	Donor Gai	PP3	NA	NA	151,11	PASS	AC=1;AF=0
1,00E-04	0	0	0	0	0	MBD1|0.1	Acceptor	GVUS	NA	NA	2430,02	PASS	AC=2;AF=0
0	0	0	0	0	0	MBD1|0.1	Acceptor L	PP3	NA	NA	5669,69	PASS	AC=2;AF=0
0	0	0	0	0	0	MBD1|0.1	Acceptor	GVUS	NA	NA	4338,82	PASS	AC=2;AF=0
0	0	0	0	0	0	NA	NA	NA	NA	NA	2547,62	PASS	AC=2;AF=0
0	0	0	0	0	0	ST8SIA3|0	Acceptor	GVUS	NA	NA	2046,67	PASS	AC=2;AF=0
0	0	0	4,545	0,916	32	NEDD4L|0	NA	NA	NA	NA	1157,69	PASS	AC=2;AF=0
0	0	0	0	0	0	NA	NA	NA	NA	NA	1232,82	PASS	AC=2;AF=0
0	0	0	4,449	0,907	32	PHLPP1|0.	NA	NA	NA	NA	151,01	PASS	AC=1;AF=0
0	0,001	0	0	0	0	BCL2|0.00	Donor Los	PP3	NA	NA	1284,7	PASS	AC=2;AF=0
0	0	0	0	0	0	FBXO15|0	Donor Los	PP3	NA	NA	1435,62	PASS	AC=2;AF=0

0	0	0	4,443	0,907	31	ZNF407|0.	NA	NA	NA	NA	1614,02	PASS	AC=1;AF=0
0	0	0	4,367	0,896	31	ZNF407|0.	Donor Los	BP7	NA	NA	367,01	PASS	AC=1;AF=0
0	0	0	0	0	0	ADNP2|0.	Acceptor G	BP7	NA	NA	7712,62	PASS	AC=2;AF=0
0	0	0	0	0	0	NA	NA	NA	loConfDeN	NA	2416,13	PASS	AC=1;AF=0
2,00E-04	0	0	8,75	0,996	45	PALM|0.0	Donor Los	VUS	NA	NA	948,69	PASS	AC=2;AF=0
0	0	0,000799	0	0	0	PTBP1|0.1	Acceptor G	VUS	NA	NA	3038,27	PASS	AC=3;AF=0
0	0	0	4,653	0,922	32	WDR18|0.	Donor Gai	BP7	NA	NA	654,12	PASS	AC=1;AF=0
0	0	0	0	0	0	APC2|0.32	Acceptor G	PP3	NA	NA	1067,04	PASS	AC=1;AF=0
0	0	0	0	0	0	MEX3D|0.	NA	NA	NA	NA	659,65	PASS	AC=2;AF=0
0	0	0	0	0	0	REXO1|0.0	Donor Los	VUS	NA	NA	2125,68	PASS	AC=2;AF=0
0	0	0	0	0	0	CSNK1G2|	Acceptor G	PP3	NA	NA	1550,83	PASS	AC=2;AF=0
0	0	0	4,834	0,928	33	CSNK1G2|	Donor Los	PP3	NA	NA	2443,69	PASS	AC=2;AF=0
0	0	0	0	0	0	BTBD2|0.0	Donor Los	VUS	NA	NA	378,08	PASS	AC=1;AF=0
0	0	0	8,917	0,997	46	DOT1L|0.0	Acceptor L	BP7	NA	NA	713,14	PASS	AC=1;AF=0
0	0	0	1,382	0,202	14,96	HMG20B|	Acceptor L	PP3	NA	NA	657,01	PASS	AC=1;AF=0
0	0	0	0	0	0	PIP5K1C|0	Donor Gai	PP3	NA	NA	1692,81	PASS	AC=2;AF=0
0	0	0	4,442	0,907	31	MAP2K2|0	Acceptor L	VUS	NA	NA	1300,7	PASS	AC=2;AF=0
6,66E-05	0	0	4,585	0,919	32	CHAF1A|0	Donor Los	BP7	NA	NA	310	PASS	AC=1;AF=0
0	0	0	0	0	0	DPP9|0.00	Donor Gai	PP3	NA	NA	1068,69	PASS	AC=2;AF=0
0	0	0	-0,088	0,035	0,877	DPP9|0.00	Donor Los	BP7	NA	NA	571,01	PASS	AC=1;AF=0
0	0	0	0	0	0	NA	NA	NA	NA	0,997725	305196	PASS	AC=1;AF=0
6,00E-04	0	0	4,439	0,906	31	KDM4B|0.	NA	NA	NA	NA	452,02	PASS	AC=1;AF=0
6,72E-05	0	0	4,425	0,904	31	SAFB2|0.0	NA	NA	NA	NA	1249,77	PASS	AC=2;AF=0
0	0	0	0	0	0	SAFB2|0.9	Acceptor G	PP3	NA	NA	220,09	PASS	AC=1;AF=0
0	0	0	7,811	0,984	39	RFX2|0.02	Acceptor G	BP7	NA	NA	248,7	PASS	AC=2;AF=0
0	0	0	4,376	0,897	31	KHSRP|0.0	Acceptor L	BP7	NA	NA	762	PASS	AC=1;AF=0
0	0	0	0	0	0	C3|0.00|0	Donor Gai	VUS	NA	NA	3939,69	PASS	AC=2;AF=0
0	0	0	4,506	0,913	32	INSR|0.00	Donor Los	BP7	NA	NA	1751,02	PASS	AC=1;AF=0
0	0	0	4,505	0,913	32	PNPLA6|0	Acceptor G	BP7	NA	NA	2349,69	PASS	AC=2;AF=0
0	0	0,0002	0	0	0	EVI5L|0.00	Donor Los	PP3	NA	NA	1627,74	PASS	AC=2;AF=0
1,00E-04	0	0	0	0	0	ADAMTS1	Acceptor G	VUS	NA	NA	1380,89	PASS	AC=2;AF=0
0	0	0	4,455	0,908	32	PIN1|0.00	NA	NA	NA	NA	3762,81	PASS	AC=2;AF=0
6,67E-05	0	0	4,446	0,907	32	DNMT1|0.	Donor Los	BP7	NA	NA	655	PASS	AC=1;AF=0
0	0	0	8,305	0,991	42	CTD-2369P	Acceptor G	BP7	NA	NA	3563,14	PASS	AC=1;AF=0
0	0	0	4,372	0,897	31	RAVER1|0	NA	NA	NA	NA	972,29	PASS	AC=2;AF=0
0	0	0	6,992	0,971	37	PDE4A|0.0	NA	NA	NA	NA	1295,68	PASS	AC=2;AF=0

0	0	0	0	0	0	DNM2|0.0	Acceptor L	VUS	NA	NA	1469	PASS	AC=1;AF=0
0,0035	0,001	0,000998	0	0	0	SMARCA4	Acceptor L	PP3	NA	NA	547	PASS	AC=1;AF=0
0	0	0	4,468	0,909	32	SMARCA4	NA	NA	NA	NA	2123,71	PASS	AC=2;AF=0
0	0	0	5,036	0,933	33	ZNF563|0.	NA	NA	NA	NA	1337	PASS	AC=1;AF=0
0	0	0,000399	5,157	0,936	33	ZNF563|0.	NA	NA	NA	NA	2036	PASS	AC=1;AF=0
0	0	0	4,245	0,868	29,1	ZNF563|0.	NA	NA	NA	NA	1005,14	PASS	AC=1;AF=0
0	0	0	0	0	0	NA	NA	NA	NA	NA	739185	PASS	AC=2;AF=0
0	0	0	4,378	0,898	31	PRKACA|0	NA	NA	NA	NA	198,56	PASS	AC=2;AF=0
0	0	0	6,061	0,958	35	EPS15L1|0	Acceptor L	PP3	NA	NA	746	PASS	AC=1;AF=0
0	0	0	5,151	0,936	33	EPS15L1|0	Acceptor L	PP3	NA	NA	748,68	PASS	AC=2;AF=0
0,0013	0,002	0	4,398	0,901	31	CTD-3222	Donor Los	BP7	NA	NA	986	PASS	AC=1;AF=0
0	0	0,000799	5,929	0,956	35	SIN3B|0.0	Acceptor L	PP3	NA	NA	506	PASS	AC=1;AF=0
0	0	0	0	0	0	MYO9B|0.	Donor Gai	PP3	NA	NA	3281,13	PASS	AC=3;AF=0
0	0	0	8,629	0,995	44	MYO9B|0.	Acceptor G	BP7	NA	NA	1621	PASS	AC=1;AF=0
0	0	0	4,424	0,904	31	BABAM1|	NA	NA	NA	NA	1744,68	PASS	AC=2;AF=0
0	0	0	4,561	0,917	32	DDA1|0.0	NA	NA	NA	NA	1501,69	PASS	AC=2;AF=0
0	0	0	4,348	0,892	30	ANO8|0.0	NA	NA	NA	NA	1115,06	PASS	AC=1;AF=0
6,68E-05	0	0	4,478	0,91	32	ANO8|0.0	Acceptor G	BP7	NA	NA	2242,71	PASS	AC=2;AF=0
0	0	0	4,39	0,899	31	FCHO1|0.0	NA	NA	NA	NA	305,01	PASS	AC=1;AF=0
0	0	0	4,467	0,909	32	FCHO1|0.0	Acceptor L	VUS	NA	NA	1621,68	PASS	AC=2;AF=0
0	0	0	4,742	0,925	32	FCHO1|0.8	Acceptor L	PP3	NA	NA	3887,68	PASS	AC=2;AF=0
0	0	0	0	0	0	MAST3|0.	Donor Los	VUS	NA	NA	2126	PASS	AC=1;AF=0
0	0	0,002196	1,483	0,216	15,63	AC002985.	Donor Los	BP7	NA	NA	2155,54	PASS	AC=3;AF=0
0	0	0	0	0	0	AC002985.	NA	NA	NA	NA	1103,47	PASS	AC=2;AF=0
1,00E-04	0	0	0	0	0	HOMER3|	Acceptor L	VUS	NA	NA	705,05	PASS	AC=1;AF=0
0	0	0,0002	4,562	0,917	32	SUGP2|0.0	NA	NA	NA	NA	977,68	PASS	AC=2;AF=0
6,68E-05	0	0	0	0	0	SUGP1|0.0	Donor Gai	PP3	NA	NA	3182,71	PASS	AC=2;AF=0
6,67E-05	0	0	4,353	0,893	30	SUGP1|0.0	Donor Gai	BP7	NA	NA	608	PASS	AC=1;AF=0
0	0	0	0	0	0	ATP13A1|	Acceptor L	PP3	NA	NA	978	PASS	AC=1;AF=0
0	0	0	4,547	0,916	32	ATP13A1|	Acceptor G	BP7	NA	NA	2351,68	PASS	AC=2;AF=0
1,00E-04	0	0	0	0	0	ATP13A1|	Donor Gai	PP3	NA	NA	4874,7	PASS	AC=2;AF=0
0	0	0	8,019	0,987	40	ZNF536|0.	NA	NA	hiConfDeN	0,999995	1156,02	PASS	AC=1;AF=0
0	0	0	4,545	0,916	32	CEBPA|0.0	NA	NA	NA	NA	350,09	PASS	AC=1;AF=0
0	0	0	0	0	0	UBA2|0.79	Acceptor G	PP3	NA	NA	269,31	PASS	AC=1;AF=0
0	0	0	0	0	0	NA	NA	NA	NA	NA	630,05	PASS	AC=2;AF=0
6,67E-05	0	0	4,699	0,924	32	ACTN4|0.0	NA	NA	NA	NA	2639,68	PASS	AC=2;AF=0

3,00E-04	0	0,0002	0	0	0	ACTN4|0.0	Donor Gai	PP3	NA	NA	598,73	PASS	AC=2;AF=0
0	0	0,0002	0	0	0	HNRNPL|0	Donor Gai	PP3	NA	NA	1240	PASS	AC=1;AF=0
0	0	0	0	0	0	HNRNPL|0	Donor Gai	PP3	NA	NA	745,04	PASS	AC=1;AF=0
0	0	0	0	0	0	SUPT5H|0	Acceptor L	VUS	NA	NA	412,7	PASS	AC=2;AF=0
0	0,001	0	4,45	0,907	32	SUPT5H|0	Acceptor G	BP7	NA	NA	640,04	PASS	AC=1;AF=0
0	0	0	0	0	0	FBL|0.00|	Donor Gai	VUS	NA	NA	401	PASS	AC=1;AF=0
3,00E-04	0	0,0002	0	0	0	AKT2|0.23	Acceptor G	PP3	NA	NA	3290,87	PASS	AC=2;AF=0
0	0	0	4,703	0,924	32	SPTBN4|0.	Donor Gai	BP7	NA	NA	136,24	PASS	AC=2;AF=0
0	0	0	0	0	0	HNRNPUL	Acceptor G	PP3	NA	NA	765,02	PASS	AC=1;AF=0
3,00E-04	0	0,0002	0	0	0	ARHGEF1|	Acceptor L	VUS	NA	NA	640,07	PASS	AC=1;AF=0
0	0	0	4,529	0,915	32	GRIK5|0.0	Donor Los	BP7	NA	NA	1076,06	PASS	AC=1;AF=0
0	0	0	4,513	0,913	32	GRIK5|0.0	Acceptor G	BP7	NA	NA	2335,68	PASS	AC=2;AF=0
0	0	0	1,41	0,206	15,15	POU2F2|0	Acceptor L	VUS	NA	NA	1307,13	PASS	AC=1;AF=0
0	0	0	4,573	0,918	32	POU2F2|0	Donor Gai	BP7	NA	NA	748,72	PASS	AC=2;AF=0
6,68E-05	0	0	0	0	0	CIC|1.00|0	Acceptor G	PP3	NA	NA	1641,01	PASS	AC=1;AF=0
0	0	0	5,455	0,944	34	MEGF8|0.	Acceptor L	PP3	NA	NA	1097,72	PASS	AC=2;AF=0
0	0	0	4,637	0,921	32	MEGF8|0.	Acceptor G	VUS	NA	NA	1908,01	PASS	AC=1;AF=0
0	0	0	0	0	0	NA	NA	NA	NA	NA	2164,66	PASS	AC=2;AF=0
0	0	0	0	0	0	NA	NA	NA	NA	NA	5347,91	PASS	AC=2;AF=0
0	0	0	6,18	0,96	35	NA	NA	NA	NA	NA	1614,12	PASS	AC=1;AF=0
0	0	0	4,779	0,927	33	SYMPK|0.	NA	NA	NA	NA	1609,71	PASS	AC=2;AF=0
0	0	0	0	0	0	SYMPK|0.	NA	NA	NA	NA	1480,93	PASS	AC=1;AF=0
0	0	0	6,89	0,969	36	MYPOP|0.	Donor Los	BP7	NA	NA	429,16	PASS	AC=1;AF=0
0	0	0	0	0	0	PPP5C|0.0	Donor Los	PP3	NA	NA	2497,68	PASS	AC=2;AF=0
0	0	0	4,629	0,921	32	NPAS1|0.0	NA	NA	NA	NA	1751,92	PASS	AC=2;AF=0
0	0	0	4,532	0,915	32	LMTK3|0.0	Acceptor L	BP7	NA	NA	1300,27	PASS	AC=2;AF=0
0	0	0	0	0	0	LMTK3|0.0	NA	NA	hiConfDeN	NA	194,06	PASS	AC=1;AF=0
0	0	0	4,568	0,917	32	RUVBL2|0	Acceptor L	VUS	NA	NA	2803	PASS	AC=1;AF=0
0	0	0	4,391	0,9	31	LIN7B|0.0	Acceptor G	BP7	NA	NA	794	PASS	AC=1;AF=0
0	0	0	4,355	0,894	30	PPFIA3|0.	NA	NA	NA	NA	850,7	PASS	AC=2;AF=0
0	0	0	4,377	0,897	31	PPFIA3|0.	Acceptor G	BP7	NA	NA	1275,03	PASS	AC=1;AF=0
0	0	0	0	0	0	CTD-3148I	Donor Los	VUS	NA	NA	287,92	PASS	AC=2;AF=0
0	0	0	0	0	0	NA	NA	NA	NA	NA	321,73	PASS	AC=2;AF=0
0	0	0	0	0	0	PTOV1|0.1	Acceptor G	VUS	NA	NA	4379,68	PASS	AC=2;AF=0
0	0	0	4,367	0,896	31	KCNC3|0.0	Donor Gai	BP7	NA	NA	1323,01	PASS	AC=1;AF=0
6,67E-05	0	0	4,683	0,923	32	CTD-2545	NA	NA	NA	NA	848,04	PASS	AC=1;AF=0

6,67E-05	0	0	4,868	0,929	33	ZNF813|0.	NA	NA	NA	NA	2811,69	PASS	AC=2;AF=0
0	0	0	5,134	0,936	33	ZNF813|0.	NA	NA	NA	NA	2114,01	PASS	AC=1;AF=0
1,00E-04	0	0	4,337	0,89	29,9	ZNF813|0.	NA	NA	NA	NA	3409,71	PASS	AC=2;AF=0
0	0	0	5,129	0,936	33	ZNF813|0.	NA	NA	NA	NA	2274,06	PASS	AC=1;AF=0
0	0	0	4,464	0,909	32	CNOT3|0.	Acceptor L	BP7	NA	NA	677	PASS	AC=1;AF=0
0	0	0	0	0	0	RPS9|0.00	Donor Los	PP3	NA	NA	109,52	PASS	AC=1;AF=0
0	0	0	4,651	0,922	32	TTYH1|0.0	Acceptor G	BP7	NA	NA	877,04	PASS	AC=1;AF=0
0,0016	0	0	0	0	0	LENG8|0.0	Donor Los	VUS	NA	NA	1041,14	PASS	AC=1;AF=0
0	0	0	0	0	0	PPP6R1|0.	Donor Los	PP3	NA	NA	2427,99	PASS	AC=1;AF=0
0	0	0,000399	4,71	0,924	32	PPP6R1|0.	Acceptor G	VUS	NA	NA	1805	PASS	AC=1;AF=0
0	0	0	4,502	0,913	32	BRSK1|0.0	NA	NA	NA	NA	1077,04	PASS	AC=1;AF=0
0	0	0	0	0	0	NA	NA	NA	NA	NA	2188,94	PASS	AC=1;AF=0
0	0	0	4,737	0,925	32	EPN1|0.00	NA	NA	NA	NA	1616,93	PASS	AC=2;AF=0
0	0	0	4,473	0,91	32	EPN1|0.75	Acceptor G	PP3	NA	NA	1913,82	PASS	AC=2;AF=0
0	0	0	4,398	0,901	31	RPS5|0.00	Donor Los	BP7	NA	NA	2575,03	PASS	AC=1;AF=0
0	0	0	4,381	0,898	31	RPS5|0.00	NA	NA	NA	NA	699,01	PASS	AC=1;AF=0
6,68E-05	0	0	0	0	0	TRIM28|0.	Acceptor G	VUS	NA	NA	1079,02	PASS	AC=1;AF=0
0	0	0	6,377	0,963	35	NOP56|0.	Donor Los	PP3	NA	NA	6756,68	PASS	AC=2;AF=0
0	0	0	4,35	0,893	30	ATRN|0.00	Donor Gai	BP7	NA	NA	1225,68	PASS	AC=2;AF=0
0	0	0,001398	0	0	0	ATRN|0.01	Donor Los	PP3	NA	NA	147,15	PASS	AC=1;AF=0
0	0	0	0	0	0	CENPB|0.0	Acceptor G	BP7	NA	NA	2615,62	PASS	AC=2;AF=0
0	0	0,000399	4,513	0,914	32	BMP2|0.0	NA	NA	NA	NA	427,31	PASS	AC=1;AF=0
0	0	0	4,384	0,899	31	PLCB1|0.0	NA	NA	NA	NA	5933,81	PASS	AC=2;AF=0
0	0	0	4,553	0,916	32	PLCB1|0.0	Acceptor L	BP7	NA	NA	863,88	PASS	AC=2;AF=0
0	0	0	4,376	0,897	31	JAG1|0.00	Donor Gai	VUS	NA	NA	2604	PASS	AC=1;AF=0
0	0	0	0	0	0	JAG1|0.00	NA	NA	NA	NA	700,63	PASS	AC=2;AF=0
0	0	0,000399	4,358	0,894	30	JAG1|0.04	Acceptor G	BP7	NA	NA	4315,77	PASS	AC=2;AF=0
6,67E-05	0	0	4,609	0,92	32	SLC24A3|0	NA	NA	NA	NA	612,68	PASS	AC=2;AF=0
0	0	0	4,496	0,912	32	BCL2L1|0.	NA	NA	NA	NA	2488,7	PASS	AC=2;AF=0
0	0	0	0	0	0	TPX2|0.13	Acceptor L	PP3	NA	NA	328,02	PASS	AC=1;AF=0
0	0	0	4,522	0,914	32	TPX2|0.49	Acceptor G	PP3	NA	NA	369	PASS	AC=1;AF=0
6,67E-05	0	0,000399	4,465	0,909	32	POFUT1|0	NA	NA	NA	NA	329	PASS	AC=1;AF=0
6,66E-05	0	0,0002	4,396	0,9	31	CBFA2T2|	NA	NA	NA	NA	720,01	PASS	AC=1;AF=0
0	0	0	4,46	0,909	32	CBFA2T2|	NA	NA	NA	NA	1031,68	PASS	AC=2;AF=0
0	0	0	4,44	0,906	31	ITCH|0.00	NA	NA	NA	NA	1696,83	PASS	AC=2;AF=0
0	0	0	7,112	0,972	37	GGT7|0.00	Acceptor L	BP7	NA	NA	1316,88	PASS	AC=2;AF=0

0	0	0	0	0	0	RBM39|0.	Donor Los	PP3	NA	NA			817,94	PASS	AC=2;AF=0
0	0	0	4,618	0,92	32	SRC|0.00|	Acceptor L	BP7	NA	NA			1330,82	PASS	AC=2;AF=0
2,00E-04	0	0,0002	4,352	0,893	30	RALGAPB|	NA	NA	NA	NA			305	PASS	AC=1;AF=0
0	0	0	4,506	0,913	32	TOP1|0.00	NA	NA	NA	NA			975	PASS	AC=1;AF=0
0	0	0	4,44	0,906	31	CHD6|0.00	NA	NA	NA	NA			879,73	PASS	AC=2;AF=0
6,66E-05	0	0	4,422	0,904	31	PTPRT|0.0	Donor Gai	BP7	NA	NA			397,99	PASS	AC=1;AF=0
0	0	0	4,441	0,906	31	NCOA3|0.	Acceptor L	BP7	NA	NA			2285,74	PASS	AC=2;AF=0
2,00E-04	0	0	0	0	0	NA	NA	NA	NA	NA			425795	PASS	AC=1;AF=0
0	0	0	0	0	0	NA	NA	NA	NA	NA			2187,51	PASS	AC=2;AF=0
0	0	0	0	0	0	NA	NA	NA	NA	NA			3991,29	PASS	AC=2;AF=0
0	0	0	4,575	0,918	32	PREX1|0.1	Acceptor G	VUS	NA	NA			1007	PASS	AC=1;AF=0
0	0	0	7,269	0,975	37	ARFGEF2|	Acceptor L	BP7	NA	NA			2136,74	PASS	AC=2;AF=0
0	0	0	4,393	0,9	31	ARFGEF2|	Acceptor G	BP7	NA	NA			579,01	PASS	AC=1;AF=0
0	0	0	4,4	0,901	31	ARFGEF2|	Acceptor L	BP7	NA	NA			718	PASS	AC=1;AF=0
6,67E-05	0	0	4,499	0,912	32	ARFGEF2|	Donor Los	BP7	NA	NA			1114,68	PASS	AC=2;AF=0
0	0	0,0002	4,43	0,905	31	ARFGEF2|	Acceptor L	BP7	NA	NA			617,14	PASS	AC=3;AF=0
0	0	0	4,453	0,908	32	SPATA2|0.	Acceptor G	BP7	NA	NA			639,7	PASS	AC=2;AF=0
0	0	0	5,415	0,943	34	ATP9A|0.0	Acceptor L	PP3	NA	NA			41,09	PASS	AC=1;AF=0
0	0	0	0	0	0	ATP9A|0.0	Donor Los	PP3	NA	NA			565	PASS	AC=1;AF=0
0	0	0	0	0	0	GNAS|0.0	Acceptor L	VUS	NA	NA			3307,64	PASS	AC=2;AF=0
0	0	0	4,391	0,9	31	PHACTR3|	NA	NA	NA	NA			4403,85	PASS	AC=2;AF=0
0	0	0	0	0	0	SYCP2|0.0	Donor Los	PP3	NA	NA			439,06	PASS	AC=1;AF=0
0	0	0	0	0	0	SYCP2|0.0	Donor Los	PP3	NA	NA			2661,7	PASS	AC=2;AF=0
0	0	0	4,463	0,909	32	LSM14B|0	Acceptor G	PP3	NA	NA			272	PASS	AC=1;AF=0
0	0	0	4,509	0,913	32	DIDO1|0.0	Acceptor L	BP7	NA	NA			760	PASS	AC=1;AF=0
0	0	0	4,372	0,897	31	RGS19|0.0	NA	NA	NA	NA			1041,21	PASS	AC=1;AF=0
0	0	0	5,581	0,948	34	MYT1|0.0	Donor Los	PP3	NA	NA			853	PASS	AC=1;AF=0
0	0	0	0	0	0	NA	NA	NA	NA	NA			2111,94	PASS	AC=1;AF=0
0	0	0	0	0	0	USP25|0.0	Donor Los	PP3	NA	NA			707,91	PASS	AC=2;AF=0
0	0	0	7,782	0,984	39	BACH1|0.0	NA	NA	NA	NA			4750,95	PASS	AC=2;AF=0
2,00E-04	0,001	0	4,468	0,909	32	TIAM1|0.0	Acceptor L	VUS	NA	NA			1737,68	PASS	AC=2;AF=0
0	0	0	9,037	0,997	47	TIAM1|0.0	Donor Los	VUS	NA	NA			915,13	PASS	AC=1;AF=0
0	0	0,000399	4,451	0,908	32	TIAM1|0.0	Donor Los	BP7	NA	NA			520,68	PASS	AC=2;AF=0
0	0	0	6,927	0,97	36	TIAM1|0.0	NA	NA	NA	NA			967,68	PASS	AC=2;AF=0
0	0	0	1,438	0,21	15,34	SCAF4|0.0	NA	NA	NA	NA			889	PASS	AC=1;AF=0
1,00E-04	0	0,0002	4,409	0,902	31	PAXBP1|0	NA	NA	NA	NA			940	PASS	AC=1;AF=0

0	0	0	0	0	0	NA	NA	NA	NA	NA	1370,83	PASS	AC=3;AF=0
0	0	0	4,363	0,895	31	AP000304.	NA	NA	NA	NA	1510,7	PASS	AC=2;AF=0
0	0	0	8,182	0,99	41	AP000304.	Acceptor G	BP7	NA	NA	172	PASS	AC=1;AF=0
0	0	0	0	0	0	MORC3|0.	Donor Gai	BP7	NA	NA	173,95	PASS	AC=1;AF=0
0	0	0	4,82	0,928	33	MORC3|0.	Donor Los	PP3	NA	NA	1476,68	PASS	AC=2;AF=0
0	0	0	0	0	0	DYRK1A|0	Donor Los	BP7	NA	NA	3122,94	PASS	AC=1;AF=0
0	0	0	5,4	0,943	34	ERG|0.02|	Acceptor L	PP3	NA	NA	201	PASS	AC=1;AF=0
0	0	0	4,701	0,924	32	ERG|0.00|	Acceptor L	BP7	NA	NA	1432	PASS	AC=1;AF=0
0	0	0	0	0	0	ETS2|0.00	Donor Gai	BP7	NA	NA	534,88	PASS	AC=1;AF=0
3,00E-04	0	0,0002	4,549	0,916	32	BRWD1|0.	Acceptor G	BP7	NA	NA	688,69	PASS	AC=2;AF=0
4,00E-04	0	0	4,422	0,904	31	DSCAM|0.	NA	NA	NA	NA	1151	PASS	AC=1;AF=0
0	0	0	7,851	0,985	39	PKNOX1|0	NA	NA	NA	NA	421	PASS	AC=1;AF=0
0	0	0	4,843	0,928	33	PCBP3|0.0	Donor Gai	BP7	NA	NA	1665,73	PASS	AC=2;AF=0
0	0	0	4,693	0,924	32	MICAL3|0.	Acceptor G	BP7	NA	NA	1960,69	PASS	AC=2;AF=0
0,0011	0,001	0	4,439	0,906	31	MICAL3|0.	NA	NA	NA	NA	603	PASS	AC=1;AF=0
0	0	0	5,792	0,953	35	MICAL3|0.	Donor Los	PP3	NA	NA	1612,71	PASS	AC=2;AF=0
0	0	0	0	0	0	PEX26|0.0	Acceptor G	BP7	NA	NA	5375,62	PASS	AC=2;AF=0
6,66E-05	0	0	4,377	0,897	31	DGCR8|0.	Acceptor L	BP7	NA	NA	842,7	PASS	AC=2;AF=0
0	0	0	0	0	0	TRMT2A|0	Acceptor G	BP7	NA	NA	1983,05	PASS	AC=1;AF=0
0	0	0	4,452	0,908	32	SCARF2|0.	NA	NA	NA	NA	2025	PASS	AC=1;AF=0
6,67E-05	0	0	4,448	0,907	32	PI4KA|0.0	Acceptor L	BP7	NA	NA	2525,69	PASS	AC=2;AF=0
0	0	0	4,423	0,904	31	PI4KA|0.0	NA	NA	NA	NA	1791,68	PASS	AC=2;AF=0
0	0	0	4,52	0,914	32	PI4KA|0.0	NA	NA	NA	NA	2127,71	PASS	AC=2;AF=0
0	0	0	4,548	0,916	32	PI4KA|0.0	NA	NA	NA	NA	356	PASS	AC=1;AF=0
0	0	0	5,427	0,944	34	BCR|0.00|	Donor Los	PP3	NA	NA	1979,01	PASS	AC=1;AF=0
0	0	0	4,476	0,91	32	BCR|0.00|	NA	NA	NA	NA	1536,68	PASS	AC=2;AF=0
0	0	0	0	0	0	MN1|0.00	NA	NA	NA	NA	1245,04	PASS	AC=1;AF=0
1,00E-04	0,001	0	4,403	0,901	31	TTC28|0.0	NA	NA	NA	NA	1065,99	PASS	AC=1;AF=0
0	0	0	4,638	0,921	32	AP1B1|0.0	NA	NA	NA	NA	1129,68	PASS	AC=2;AF=0
0	0	0	4,532	0,915	32	MTMR3|0	Donor Gai	BP7	NA	NA	249,02	PASS	AC=1;AF=0
0	0	0	0,711	0,115	8,556	LIF|0.00|0	NA	NA	NA	NA	800,24	PASS	AC=1;AF=0
0	0	0	4,807	0,927	33	GAL3ST1|	Acceptor L	PP3	NA	NA	2529,92	PASS	AC=2;AF=0
6,66E-05	0	0	7,62	0,981	38	EIF4ENIF1	NA	NA	NA	NA	784	PASS	AC=1;AF=0
3,00E-04	0	0,0002	4,547	0,916	32	SYN3|0.00	NA	NA	NA	NA	1699,69	PASS	AC=2;AF=0
0,0017	0	0,000599	4,361	0,895	31	MYH9|0.0	NA	NA	NA	NA	832	PASS	AC=1;AF=0
0	0	0	4,447	0,907	32	GGA1|0.0	Acceptor G	BP7	NA	NA	2120,69	PASS	AC=2;AF=0

0	0	0	0	0	0	PDGFB|0.0	Donor Gai	PP3	NA	NA	1186,01	PASS	AC=2;AF=0
0	0	0	5,106	0,935	33	RPL3|0.01	Acceptor G	BP7	NA	NA	3119,68	PASS	AC=2;AF=0
1,00E-04	0	0	4,866	0,929	33	RPL3|0.00	Acceptor L	BP7	NA	NA	1782,7	PASS	AC=2;AF=0
0	0	0	4,453	0,908	32	CACNA1I|	NA	NA	NA	NA	1779,03	PASS	AC=1;AF=0
4,00E-04	0	0	4,445	0,907	31	CACNA1I|	Acceptor G	BP7	NA	NA	1041,1	PASS	AC=1;AF=0
6,66E-05	0	0	4,36	0,895	31	EP300|0.0	Acceptor L	BP7	NA	NA	1231	PASS	AC=1;AF=0
0	0	0	0	0	0	XRCC6|0.0	NA	NA	NA	NA	635,62	PASS	AC=2;AF=0
6,67E-05	0	0	0	0	0	SCUBE1|0.	Donor Los	VUS	NA	NA	1743,68	PASS	AC=2;AF=0
0	0	0	4,415	0,903	31	SULT4A1|	NA	NA	NA	NA	927	PASS	AC=1;AF=0
0	0	0	4,445	0,907	31	CELSR1|0.	Acceptor L	BP7	NA	NA	1735,7	PASS	AC=2;AF=0
0	0	0,0002	0	0	0	GRAMD4|	Acceptor G	PP3	NA	NA	563,01	PASS	AC=1;AF=0
0	0	0	0	0	0	BRD1|0.25	Acceptor G	PP3	NA	NA	1128,32	PASS	AC=2;AF=0
2,00E-04	0	0,0002	0	0	0	PLXNB2|0.	Acceptor G	PP3	NA	NA	1260	PASS	AC=1;AF=0
1,00E-04	0	0	4,424	0,904	31	PLXNB2|0.	Donor Gai	VUS	NA	NA	4204,76	PASS	AC=2;AF=0
0	0	0	0	0	0	PLXNB2|0.	Donor Gai	PP3	NA	NA	1644,02	PASS	AC=1;AF=0
0	0	0	4,515	0,914	32	SBF1|0.09	Acceptor G	BP7	NA	NA	804,04	PASS	AC=1;AF=0
0,0041	0,0031	0,000599	0	0	0	SBF1|0.00	Donor Gai	VUS	NA	NA	1362,02	PASS	AC=2;AF=0
6,68E-05	0	0	4,398	0,901	31	SBF1|0.04	Acceptor G	BP7	NA	NA	1286	PASS	AC=1;AF=0
0	0	0,0002	0	0	0	SBF1|0.00	Donor Gai	PP3	NA	NA	2598,83	PASS	AC=2;AF=0
0	0	0	4,389	0,899	31	SHANK3|0	Acceptor L	BP7	NA	NA	1788,3	PASS	AC=2;AF=0
0	0	0	0	0	0	NA	NA	NA	loConfDeN	NA	447,07	PASS	AC=1;AF=0
0	0	0	0	0	0	SHANK3|0	NA	NA	hiConfDeN	0,799488	5806,94	PASS	AC=1;AF=0
0	0	0	6,805	0,968	36	GPR143|0	Donor Los	VUS	NA	0,999337	3214,15	PASS	AC=3;AF=0
0	0	0	5,833	0,954	35	OFD1|0.00	Acceptor L	BP7	NA	NA	502,35	PASS	AC=1;AF=0
0	0	0,001589	0	0	0	GLRA2|0.0	Acceptor L	VUS	NA	NA	470,4	PASS	AC=3;AF=0
9,50E-05	0	0	0	0	0	MOSPD2|	Acceptor L	PP3	NA	NA	4326,48	PASS	AC=1;AF=0
0	0	0	6,185	0,96	35	AP1S2|0.0	Donor Gai	PP3	NA	NA	1591,59	PASS	AC=5;AF=0
0	0	0	0	0	0	NA	NA	NA	NA	NA	303,45	PASS	AC=3;AF=0
0	0	0	8,937	0,997	46	DMD|0.00	Donor Los	BP7	NA	NA	1595,12	PASS	AC=3;AF=0
9,00E-04	0	0,00053	2,846	0,487	23,1	DMD|0.00	Donor Los	BP7	NA	NA	515,01	PASS	AC=1;AF=0
0	0	0	0	0	0	USP9X|0.1	Acceptor G	VUS	NA	NA	1203,96	PASS	AC=2;AF=0
0	0	0	7,207	0,974	37	DDX3X|0.0	Donor Los	BP7	hiConfDeN	0,99924	914,03	PASS	AC=1;AF=0
0	0	0	4,829	0,928	33	KDM6A|0.	Donor Los	PP3	NA	NA	1302,27	PASS	AC=3;AF=0
0,004	0,0041	0,002119	0	0	0	KDM6A|0.	Donor Los	PP3	NA	NA	112,83	PASS	AC=2;AF=0
0	0	0	4,43	0,905	31	EBP|0.22|	Acceptor G	PP3	NA	NA	171,21	PASS	AC=2;AF=0
9,59E-05	0	0	0	0	0	NA	NA	NA	NA	NA	704,93	PASS	AC=2;AF=0

0	0	0	0	0	0	NA	NA	NA	hiConfDeN	0,994141	1711,98	PASS	AC=1;AF=0
0,0016	0,0046	0	0	0	0	FAM120C|	NA	NA	NA	NA	828,36	PASS	AC=2;AF=0
0,0015	0,0043	0	0	0	0	NA	NA	NA	NA	NA	361467	PASS	AC=2;AF=0
9,57E-05	0	0	0	0	0	GNL3L|0.0	Donor Los	PP3	NA	0,987567	2021,68	PASS	AC=2;AF=0
3,00E-04	0	0	0	0	0	IGBP1|0.0	Acceptor L	VUS	NA	NA	382,27	PASS	AC=1;AF=0
9,49E-05	0	0	0	0	0	MED12|0.	Donor Los	PP3	NA	5,506262	1479,71	PASS	AC=2;AF=0
0	0	0	0	0	0	MED12|0.	Donor Los	VUS	NA	0,99895	4403,64	PASS	AC=3;AF=0
0	0	0,001325	0	0	0	ZMYM3|0.	Acceptor L	VUS	NA	0,997493	1532,84	PASS	AC=2;AF=0
0	0	0	0,513	0,093	6,643	HDAC8|0.	Acceptor G	VUS	NA	NA	1670,13	PASS	AC=2;AF=0
9,46E-05	0,0014	0	0	0	0	MAGT1|0.	NA	NA	NA	NA	1680,94	PASS	AC=1;AF=0
0	0	0	0	0	0	GLA|0.00|	Acceptor L	PP3	NA	NA	937,12	PASS	AC=1;AF=0
0	0	0	0	0	0	ARMCX4|0	NA	NA	NA	NA	1925,75	PASS	AC=1;AF=0
0	0	0	0	0	0	ARMCX4|0	NA	NA	NA	NA	707,65	PASS	AC=1;AF=0
0	0	0	0	0	0	NA	NA	NA	NA	NA	492,66	PASS	AC=1;AF=0
0	0	0	4,392	0,9	31	COL4A6|0	Donor Los	PP3	NA	NA	1793,13	PASS	AC=3;AF=0
0	0	0	4,386	0,899	31	IRS4|0.00|	NA	NA	NA	NA	1912,14	PASS	AC=3;AF=0
9,00E-04	0	0,00053	0,868	0,134	10,1	NA	NA	NA	NA	NA	241,71	PASS	AC=1;AF=0
3,00E-04	0	0,000265	4,348	0,892	30	LRCH2|0.0	Acceptor	GBP7	NA	0,99373	2791,88	PASS	AC=2;AF=0
7,00E-04	0,0014	0	0	0	0	TENM1|0.	Acceptor	GBP7	NA	NA	381,99	PASS	AC=1;AF=0
0	0	0	0	0	0	TENM1|0.	Donor Los	VUS	NA	NA	772,05	PASS	AC=1;AF=0
0	0	0,001325	0	0	0	OCRL|0.04	Acceptor	GBP7	NA	0,999206	1877,67	PASS	AC=2;AF=0
0	0	0	5,377	0,942	34	FAM122B|	Acceptor L	PP3	NA	NA	571,2	PASS	AC=2;AF=0
0	0	0	7,258	0,975	37	FAM122B|	NA	NA	NA	NA	258,02	PASS	AC=1;AF=0
0	0	0	7,299	0,975	37	MECP2|0.	Acceptor G	VUS	hiConfDeN	6,341678	3370,02	PASS	AC=1;AF=0
0	0	0	6,819	0,968	36	MECP2|0.	NA	NA	hiConfDeN	0,999999	1673,09	PASS	AC=1;AF=0
0	0	0	0	0	0	RPL10|0.0	NA	NA	NA	NA	1958,6	PASS	AC=2;AF=0
0	0	0	6,164	0,96	35	FAM3A|0.	Donor Los	BP7	NA	0,905178	1142,67	PASS	AC=2;AF=0
0	0	0	0	0	0	ZFY|0.00|	Donor Los	PP3	NA	NA	1695,82	PASS	AC=2;AF=0
0	0	0	7,404	0,977	38	DDX3Y|0.0	Acceptor	GBP7	NA	NA	3609,74	PASS	AC=4;AF=0
0	0	0,000811	9,051	0,997	47	DDX3Y|0.0	NA	NA	NA	NA	3870,71	PASS	AC=4;AF=0

DeNovo	is_pathoge	haplotype	transmissi	status	id
NA	no	3	paternal	proband; f	P_375; F_375
NA	no	1	maternal	proband;	P_289;
M_289 2	yes	0	DNV	proband	P_115
NA	no	unphased	paternal	father	F_210
NA	no	3	paternal	proband; f	P_226; F_226
NA	no	unphased	maternal	mother	M_100
NA	yes	unphased	paternal	father	F_397
NA	yes	unphased	paternal	father	F_329
NA	no	unphased	paternal	father	F_322
NA	no	1	maternal	proband;	P_72; M_72
NA	no	1	paternal	proband; f	P_375; F_375
NA	no	1	paternal	proband; f	P_101; F_101; S_101
NA	no	1	maternal	proband;	P_343; M_343
NA	no	1	maternal	proband;	P_343; M_343
NA	no	unphased	paternal	father	F_338
NA	no	1	paternal	proband; f	P_262; F_262
NA	no	unphased	NA	cousin	O1_136
NA	no	unphased	paternal	proband; f	P_41; F_41
NA	no	unphased	maternal	mother	M_243
NA	no	1	paternal	proband; f	P_165; F_165
NA	no	unphased	paternal	father	F_336
NA	no	1	maternal	proband;	P_96; M_96
NA	no	0	maternal	proband;	P_100; M_100
NA	no	0	paternal	proband; f	P_203; F_203
NA	no	0	paternal	proband; f	P_153; F_153
NA	no	unphased	NA	proband	P_64
NA	no	unphased	maternal	proband;	P_164; M_164
NA	no	1	paternal	proband; f	P_313; F_313
NA	no	unphased	maternal	mother	M_161
NA	no	3	maternal	proband;	P_283; M_283
NA	no	unphased	maternal	mother	M_360
NA	no	unphased	paternal	father	F_258
NA	no	2	maternal	proband;	P_103; M_103
NA	no	2	paternal	proband; f	P_63; F_63

NA	no	unphased	maternal	mother	M_141
NA	no	unphased	maternal	mother	M_223
NA	no	1	NA	proband	P_214
NA	no	0	maternal	proband;	P_421; M_421
NA	no	unphased	paternal	father	F_223
NA	no	unphased	paternal	father	F_138
NA	no	unphased	paternal	father	F_291
NA	no	unphased	maternal	mother	M_371
NA	no	unphased	paternal	father	F_85
NA	no	unphased	paternal	proband; f	P_306; F_306
NA	no	unphased	paternal	father	F_81
NA	no	1	paternal	proband; f	P_44; F_44
NA	no	unphased	maternal	mother	M_425
NA	no	unphased	paternal	father	F_301
NA	no	unphased	maternal	mother	M_180
NA	no	0	maternal	mother; pr	M_438; P_438
NA	no	1	paternal	proband; f	P_86; F_86
NA	no	1	paternal	proband; f	P_122; F_122
NA	no	unphased	maternal	mother	M_389
NA	no	unphased	paternal	father	F_174
NA	no	unphased	maternal	mother	M_62
NA	no	unphased	maternal	mother	M_25
NA	no	unphased	maternal	mother	M_266
NA	no	1	paternal	proband; f	P_344; F_344
NA	no	1	paternal	proband; f	P_303; F_303
NA	no	0	paternal	proband; f	P_220; F_220
NA	no	1	paternal	proband; f	P_290; F_290
NA	no	1	paternal	proband; f	P_358; F_358
NA	no	unphased	maternal	mother	M_416
NA	no	unphased	paternal	father	F_286
NA	no	2	paternal	proband; f	P_441; F_441
NA	no	1	maternal	mother; pr	M_151; P_151
NA	no	unphased	maternal	proband;	P_111; M_111
NA	no	unphased	paternal	father	F_213
NA	no	unphased	paternal	father	F_335
NA	no	unphased	paternal	father	F_265

NA	no	1	paternal	proband; f	P_327; F_327
NA	no	1	maternal	proband;	P_134; M_134
NA	no	unphased	paternal	father	F_41
NA	no	unphased	paternal	father	F_260
NA	no	0	NA	proband	P_137
NA	no	unphased	NA	proband	P_236
NA	no	1	maternal	proband;	P_50; M_50
NA	no	unphased	maternal	mother	M_83
NA	no	unphased	paternal	father	F_291
NA	no	1	paternal	proband; f	P_52; F_52
NA	no	1	maternal	proband;	P_379; M_379
NA	no	unphased	paternal	father	F_144
NA	no	unphased	paternal	father	F_110
NA	no	unphased	maternal	mother	M_424
NA	no	unphased	paternal	father	F_342
NA	no	unphased	maternal	mother	M_23
NA	no	unphased	paternal	father	F_19
NA	no	unphased	paternal	father	F_133
NA	no	unphased	paternal	father	F_44
NA	no	unphased	paternal	father	F_125
NA	no	1	maternal	proband;	P_336; M_336
NA	no	unphased	paternal	father	F_407
NA	no	unphased	paternal	father	F_252
NA	no	unphased	maternal	mother	M_427
NA	no	2	maternal	proband;	P_287; M_287
NA	no	0	paternal	proband; f	P_133; F_133
NA	no	1	maternal	proband;	P_1; M_1
NA	no	1	maternal	proband;	P_397; M_397
NA	no	unphased	NA	proband	P_235
NA	no	1	paternal	proband; f	P_131; F_131
NA	no	1	paternal	proband; f	P_215; F_215
NA	no	unphased	maternal	mother	M_198
NA	no	1	paternal	proband; f	P_282; F_282
NA	no	1	maternal	proband;	P_429; M_429
NA	no	unphased	NA	proband	P_242
NA	no	1	maternal	proband;	P_240; M_240

NA	no	unphased	NA	proband	P_167
NA	no	unphased	paternal	father	F_267
NA	no	unphased	paternal	father	F_320
NA	no	unphased	maternal	mother	M_401
NA	no	unphased	maternal	mother	M_168
NA	risk	1	maternal	proband;	P_204; M_204
NA	no	unphased	maternal	mother	M_115
NA	no	unphased	NA	mother	M_264
NA	no	1	paternal	proband; f	P_203; F_203
NA	no	unphased	maternal	mother	M_97
NA	no	1	paternal	proband; f	P_228; F_228
NA	no	unphased	NA	uncle	O3_359
NA	no	3	paternal	proband; f	P_321; F_321
NA	no	unphased	paternal	father	F_314
NA	no	unphased	paternal	father	F_390
NA	no	unphased	paternal	father	F_270
NA	no	unphased	paternal	father	F_438
NA	no	unphased	paternal	father	F_350
NA	no	1	maternal	proband;	P_50; M_50
NA	no	unphased	maternal	mother	M_163
NA	no	0	paternal	proband; f	P_24; F_24
NA	no	0	maternal	proband;	P_282; M_282
NA	no	unphased	paternal	father	F_97
NA	no	1	maternal	proband;	P_416; M_416
NA	no	0	paternal	proband; f	P_40; F_40
NA	no	0	paternal	proband; f	P_153; F_153
NA	no	unphased	maternal	mother	M_257
NA	no	1	paternal	proband; f	P_33; F_33
NA	no	unphased	paternal	father	F_250
NA	no	0	maternal	proband;	P_173; M_173
NA	no	0	paternal	proband; f	P_206; F_206
NA	no	unphased	maternal	mother	M_156
NA	no	1	paternal	proband; f	P_33; F_33
NA	no	unphased	maternal	mother	M_62
NA	no	1	maternal	proband;	P_361; M_361
NA	no	unphased	maternal	mother	M_87

NA	no	unphased	maternal	mother; si	M_357; S_357
NA	no	unphased	paternal	father	F_291
NA	no	unphased	paternal	father	F_361
NA	no	unphased	maternal	mother	M_13
NA	no	1	maternal	proband;	P_174; M_174
NA	no	unphased	NA	cousin; au	O1_370; O2_370
NA	no	1	maternal	proband;	P_414; M_414
NA	no	unphased	paternal	father	F_251
NA	no	unphased	maternal	mother	M_338
NA	no	1	NA	proband	P_158
NA	no	unphased	paternal	father	F_277
NA	no	1	paternal	proband; f	P_418; F_418
NA	no	unphased	maternal	mother	M_78
NA	no	0	maternal	proband;	P_70; M_70
NA	no	1	maternal	proband;	P_228; M_228
NA	no	2	paternal	proband; f	P_67; F_67
NA	no	1	maternal	proband;	P_185; M_185
NA	no	unphased	maternal	mother	M_193
NA	no	unphased	maternal	mother	M_239
NA	no	unphased	maternal	mother	M_396
NA	no	3	maternal	proband;	P_272; M_272
NA	no	unphased	maternal	mother	M_48
NA	no	unphased	paternal	father	F_76
NA	no	unphased	maternal	mother	M_105
NA	no	1	maternal	proband;	P_52; M_52
NA	no	unphased	paternal	father	F_251
NA	no	3	paternal	proband; f	P_412; F_412; S_412
NA	no	unphased	paternal	father	F_381
NA	no	unphased	paternal	father	F_232
NA	no	unphased	paternal	father	F_384
NA	no	unphased	NA	father	F_342
NA	no	unphased	NA	mother	M_131
NA	no	2	maternal	proband;	P_69; M_69
NA	no	unphased	paternal	father	F_17
NA	no	1	maternal	proband;	P_401; M_401
NA	no	unphased	paternal	proband; f	P_327; F_327

NA	no	unphased	maternal	mother	M_119
NA	no	unphased	maternal	mother	M_426
NA	no	1	maternal	proband;	P_5; M_5
NA	no	unphased	paternal	father	F_63
NA	no	unphased	paternal	father	F_136
NA	no	1	NA	proband	P_286
NA	no	unphased	maternal	mother	M_126
NA	no	3	maternal	mother; pr	M_68; P_68
NA	no	unphased	paternal	father	F_338
NA	no	1	maternal	proband;	P_277; M_277
NA	no	unphased	paternal	proband; f	P_67; F_67
NA	no	1	maternal	proband;	P_88; M_88
NA	no	1	NA	proband	P_186
NA	no	unphased	paternal	father	F_33
NA	no	0	maternal	proband;	P_69; M_69
NA	no	unphased	maternal	mother	M_170
NA	no	unphased	paternal	father	F_318
NA	no	unphased	NA	mother	M_80
NA	no	unphased	maternal	mother	M_334
NA	no	unphased	maternal	mother	M_148
NA	no	1	paternal	proband; f	P_81; F_81
NA	no	unphased	maternal	mother	M_188
NA	no	unphased	paternal	father	F_119
NA	no	unphased	maternal	mother	M_369
NA	no	1	paternal	proband; f	P_366; F_366
NA	no	0	maternal	proband;	P_242; M_242
NA	no	unphased	paternal	father	F_362
NA	no	unphased	paternal	proband; f	P_362; F_362
NA	no	unphased	paternal	father	F_356
NA	no	unphased	maternal	mother	M_317
NA	no	1	maternal	proband;	P_273; M_273
NA	no	1	paternal	proband; f	P_401; F_401
NA	no	unphased	maternal	mother	M_288
NA	no	unphased	paternal	father	F_278
NA	no	unphased	maternal	mother	M_149
NA	no	unphased	paternal	father	F_44

NA	no	unphased	paternal	father	F_393
NA	no	3	paternal	proband; f	P_149; F_149
NA	no	unphased	paternal	father	F_332
NA	no	2	maternal	proband;	P_172; M_172
NA	no	unphased	NA	mother	M_378
NA	no	unphased	NA	mother	M_157
NA	no	unphased	maternal	mother	M_146
NA	no	1	paternal	proband; f	P_248; F_248
NA	no	unphased	maternal	mother	M_225
2	no	unphased	DNV	proband	P_292
NA	no	unphased	paternal	father	F_69
NA	no	2	paternal	proband; f	P_99; F_99
NA	no	1	maternal	proband;	P_310; M_310
NA	no	1	maternal	proband;	P_336; M_336
NA	no	unphased	maternal	mother	M_411
NA	no	unphased	paternal	father	F_258
NA	no	unphased	NA	father	F_425
NA	no	unphased	maternal	mother	M_66
NA	no	unphased	maternal	mother	M_236
NA	no	unphased	maternal	mother	M_141
NA	no	unphased	NA	cousin; un	O1_136; O3_136
NA	no	1	paternal	proband; f	P_248; F_248
NA	no	unphased	paternal	father	F_408
NA	no	unphased	paternal	father; sibl	F_357; S_357
NA	no	unphased	maternal	proband;	P_371; M_371
NA	no	1	maternal	proband;	P_132; M_132
NA	no	1	paternal	proband; f	P_174; F_174
2	no	unphased	DNV	proband	P_3
NA	no	unphased	paternal	father	F_28
NA	no	0	maternal	proband;	P_12; M_12
NA	no	0	maternal	proband;	P_100; M_100
NA	no	3	paternal	proband; f	P_348; F_348
NA	no	1	paternal	proband; f	P_76; F_76
NA	no	0	maternal	proband;	P_75; M_75
NA	no	1	maternal	proband;	P_286; M_286
NA	no	1	paternal	proband; f	P_274; F_274

NA	no	0	maternal	proband;	P_357; M_357; S_357
NA	no	1	maternal	proband;	P_39; M_39
NA	no	2	maternal	proband;	P_238; M_238
NA	no	unphased	maternal	mother	M_85
NA	yes	0	maternal	proband;	P_17; M_17
NA	no	unphased	NA	mother	M_269
NA	no	0	maternal	proband;	P_48; M_48
2	yes	1	NA	proband	P_71
NA	no	unphased	NA	proband	P_175
NA	no	unphased	maternal	mother	M_281
2	no	unphased	DNV	proband	P_203
NA	no	2	paternal	proband; f	P_215; F_215
NA	no	1	maternal	proband; s	P_62; S_62; M_62
NA	no	unphased	maternal	mother	M_256
NA	no	unphased	paternal	father	F_247
NA	no	unphased	maternal	mother	M_315
NA	no	1	maternal	proband;	P_434; M_434
NA	no	2	maternal	proband;	P_83; M_83
NA	no	unphased	maternal	mother	M_212
NA	no	1	maternal	proband; s	P_339; S_339; M_339
NA	no	unphased	paternal	father	F_300
NA	no	unphased	maternal	mother	M_288
NA	no	2	maternal	proband;	P_2; M_2
NA	no	unphased	paternal	father; sibl	F_426; S_426
NA	no	unphased	paternal	father	F_12
NA	no	unphased	maternal	mother; si	M_357; S_357
NA	no	1	paternal	proband; f	P_16; F_16
2	no	unphased	DNV	proband	P_24
NA	no	1	maternal	proband;	P_182; M_182
NA	no	unphased	maternal	mother	M_415
NA	no	1	maternal	proband;	P_43; M_43
NA	no	1 0	maternal	proband;	P_211; M_211
NA	no	unphased	paternal	father	F_212
NA	no	unphased	NA	uncle	O2_398
NA	no	unphased	maternal	mother	M_424
NA	no	unphased	paternal	father	F_203

NA	no	1	maternal	proband;	P_290; M_290
NA	no	unphased	paternal	father	F_178
NA	no	1	NA	proband	P_218
NA	no	2	maternal	proband;	P_203; M_203
NA	no	3	maternal	proband;	P_352; M_352
NA	no	0	maternal	proband;	P_103; M_103
NA	no	unphased	paternal	father	F_79
NA	no	unphased	paternal	father	F_43
NA	no	unphased	maternal	sibling; mo	S_62; M_62
2	yes	1	DNV	proband	P_332
NA	no	unphased	paternal	father	F_163
2	no	1	DNV	proband	P_320
NA	no	unphased	maternal	mother	M_241
NA	no	2 1	maternal	proband;	P_161; M_161
NA	no	unphased	paternal	father	F_269
NA	no	unphased	maternal	mother	M_219
NA	no	unphased	maternal	proband;	P_44; M_44
NA	no	unphased	maternal	proband;	P_20; M_20
2	no	1	DNV	proband	P_281
NA	no	unphased	paternal	father	F_240
NA	no	unphased	paternal	father	F_39
NA	no	1	paternal	proband; f	P_432; F_432
NA	no	1	paternal	proband; f	P_385; F_385
NA	no	unphased	paternal	father	F_381
NA	no	unphased	maternal	mother	M_63
NA	no	unphased	maternal	proband;	P_333; M_333
NA	no	0	maternal	proband;	P_156; M_156
NA	no	unphased	maternal	mother	M_328
NA	no	1	maternal	proband;	P_372; M_372
NA	no	1	maternal	proband;	P_266; M_266
NA	no	unphased	paternal	father	F_42
NA	no	unphased	NA	proband	P_189
NA	no	1	maternal	proband;	P_184; M_184
NA	no	0	maternal	proband;	P_420; M_420
NA	no	1	paternal	proband; s	P_61; S1_61; F_61
NA	no	unphased	maternal	proband;	P_108; M_108

NA	no	unphased	paternal	father	F_360
NA	no	0	paternal	proband; f	P_177; F_177
NA	no	unphased	paternal	father	F_9
NA	no	unphased	NA	uncle	O3_136
NA	no	unphased	maternal	mother	M_405
NA	no	1	paternal	proband; f	P_406; F_406
NA	no	unphased	maternal	mother	M_102
NA	no	unphased	maternal	mother	M_45
NA	no	1	paternal	proband; f	P_390; F_390
NA	no	1	NA	proband	P_361
NA	no	unphased	paternal	father	F_241
NA	no	0	maternal	proband;	P_270; M_270
NA	no	unphased	paternal	father	F_402
NA	no	unphased	maternal	mother	M_179
NA	no	unphased	NA	mother	M_190
NA	no	1	paternal	proband; f	P_77; F_77
NA	no	unphased	paternal	father	F_440
NA	no	1	maternal	proband;	P_248; M_248
NA	no	0	paternal	proband; f	P_334; F_334
NA	no	unphased	maternal	mother	M_153
NA	no	0	paternal	proband; f	P_87; F_87
NA	no	unphased	paternal	father	F_229
NA	no	unphased	maternal	mother	M_35
NA	no	1	paternal	proband; f	P_1; F_1
NA	no	unphased	maternal	proband;	P_101; M_101
NA	no	0	maternal	proband;	P_297; M_297
NA	no	3	maternal	sibling; pro	S_166; P_166; M_166
NA	no	1	maternal	proband;	P_309; M_309
NA	no	1	maternal	proband;	P_156; M_156
NA	no	0	maternal	proband;	P_9; M_9
NA	no	unphased	paternal	father	F_277
NA	no	unphased	NA	proband	P_14
NA	no	unphased	maternal	mother	M_319
NA	no	unphased	paternal	father	F_292
NA	no	unphased	paternal	father	F_407
NA	no	unphased	paternal	father	F_313

NA	no	1	maternal	proband;	P_67; M_67
NA	no	3	maternal	proband;	P_113; M_113
NA	no	unphased	paternal	father	F_430
NA	no	1	maternal	proband;	P_130; M_130
NA	no	unphased	paternal	father	F_441
NA	no	unphased	paternal	father	F_91
NA	no	1	paternal	proband; f	P_32; F_32
NA	no	unphased	maternal	mother	M_47
NA	no	1	paternal	proband; f	P_223; F_223
NA	no	1	maternal	proband;	P_416; M_416
NA	no	unphased	maternal	mother	M_176
NA	no	unphased	maternal	mother	M_176
NA	no	unphased	maternal	mother	M_323
NA	no	1	paternal	proband; f	P_360; F_360
NA	no	unphased	paternal	father	F_416
NA	no	unphased	maternal	mother	M_138
NA	no	0	paternal	proband; f	P_216; F_216
2	yes	unphased	DNV	proband	P_238
2	yes	unphased	DNV	proband	P_333
NA	no	1	maternal	proband;	P_219; M_219
NA	no	0 1	maternal	proband;	P_162; M_162
NA	no	1	maternal	proband;	P_153; M_153
NA	no	1	paternal	proband; f	P_5; F_5
NA	no	1	paternal	proband; f	P_17; F_17
NA	no	1	paternal	proband; f	P_294; F_294
NA	no	unphased	paternal	father	F_258
NA	no	unphased	maternal	mother	M_30
NA	no	2	maternal	proband;	P_430; M_430
NA	no	unphased	paternal	father	F_118
NA	no	1	maternal	proband;	P_127; M_127
NA	no	unphased	maternal	mother	M_190
NA	no	1	paternal	proband; f	P_134; F_134
NA	no	unphased	maternal	mother	M_71
NA	no	1	paternal	proband; f	P_253; F_253
NA	no	unphased	maternal	mother	M_70
NA	no	unphased	paternal	father	F_220

NA	no	unphased	paternal	father	F_220
NA	no	unphased	maternal	mother	M_261
NA	no	0	paternal	proband; f	P_427; F_427
NA	no	unphased	paternal	father	F_403
NA	no	unphased	paternal	father	F_13
NA	no	unphased	maternal	proband;	P_75; M_75
NA	no	1	maternal	proband;	P_190; M_190
NA	no	unphased	paternal	father	F_130
NA	no	unphased	maternal	mother	M_310
NA	no	3	paternal	proband; f	P_390; F_390
NA	no	1 0	maternal	proband;	P_270; M_270
NA	no	unphased	maternal	mother	M_321
NA	no	unphased	paternal	father	F_340
NA	no	unphased	NA	proband	P_288
NA	no	1	paternal	proband; f	P_281; F_281
NA	no	1	maternal	proband;	P_43; M_43
NA	no	unphased	maternal	mother	M_255
NA	no	unphased	paternal	father	F_220
NA	no	unphased	maternal	mother; si	M_234; S_234; P_234
NA	no	unphased	NA	cousin; au	O1_370; O2_370
NA	no	unphased	paternal	father	F_174
NA	no	unphased	paternal	father	F_108
NA	no	unphased	paternal	father	F_362
NA	no	unphased	paternal	father	F_210
NA	no	3	paternal	proband; f	P_381; F_381
NA	no	3	paternal	proband; f	P_381; F_381
NA	no	unphased	paternal	father	F_236
NA	no	unphased	paternal	father	F_236
NA	no	0	NA	proband	P_185
NA	no	0	maternal	proband;	P_185; M_185
NA	no	unphased	paternal	father	F_203
NA	no	unphased	maternal	mother	M_276
NA	no	3	maternal	proband;	P_80; M_80
NA	no	0	maternal	proband;	P_48; M_48
NA	no	unphased	paternal	father	F_344
2	no	1	NA	proband	P_135

NA	no	unphased	paternal	father	F_102
NA	no	1	maternal	proband;	P_175; M_175
NA	no	1	maternal	proband;	P_291; M_291
NA	no	1	maternal	proband;	P_157; M_157
NA	no	unphased	paternal	father	F_407
NA	no	unphased	maternal	mother	M_39
NA	no	unphased	maternal	mother	M_187
NA	no	unphased	paternal	father	F_332
NA	no	unphased	paternal	father	F_13
NA	no	unphased	maternal	mother	M_242
NA	no	1	maternal	proband;	P_75; M_75
NA	no	unphased	NA	proband	P_335
NA	no	1	maternal	proband;	P_427; M_427
NA	no	1	maternal	proband;	P_89; M_89
NA	no	unphased	maternal	mother	M_4
NA	no	unphased	maternal	mother	M_248
NA	no	unphased	paternal	father	F_326
NA	no	1	maternal	proband;	P_372; M_372
NA	no	1	maternal	proband;	P_44; M_44
NA	no	1	paternal	proband; f	P_75; F_75
NA	no	unphased	NA	half-sibling	S1_395
NA	no	1	paternal	proband; f	P_397; F_397
NA	no	unphased	paternal	father	F_269
NA	no	1	paternal	proband; f	P_317; F_317
NA	no	0	maternal	proband;	P_288; M_288
NA	no	1	paternal	proband; f	P_102; F_102
NA	no	1	maternal	proband;	P_404; M_404
2	no	3	DNV	proband	P_213
NA	no	1	maternal	proband;	P_97; M_97
NA	no	unphased	paternal	father	F_286
NA	no	unphased	paternal	father	F_173
NA	no	unphased	NA	mother	M_9
NA	no	1	paternal	proband; f	P_20; F_20
NA	no	2	paternal	proband; f	P_168; F_168
NA	no	1	maternal	proband; s	P_150; S_150; M_150
NA	no	unphased	maternal	mother	M_198

NA	no	unphased	paternal	father	F_58
NA	no	1	NA	proband	P_106
NA	no	2	paternal	proband; f	P_21; F_21
NA	no	0	maternal	proband;	P_290; M_290
NA	no	unphased	maternal	mother	M_225
NA	no	0	paternal	proband; f	P_8; F_8
NA	no	3	maternal	proband;	P_381; M_381
NA	no	1	maternal	proband;	P_401; M_401
NA	no	unphased	maternal	proband;	P_425; M_425
NA	no	1	paternal	proband; f	P_262; F_262
NA	no	1	maternal	proband;	P_136; M_136
NA	no	unphased	maternal	mother	M_117
NA	no	1	paternal	proband; f	P_155; F_155
NA	no	unphased	paternal	father	F_92
NA	no	1	NA	proband	P_242
NA	no	unphased	NA	mother	M_212
NA	no	unphased	paternal	father	F_17
NA	no	unphased	maternal	mother	M_390
NA	no	unphased	paternal	father	F_242
NA	no	1	paternal	proband; f	P_284; F_284
NA	no	unphased	paternal	father	F_126
NA	no	1	maternal	proband;	P_308; M_308
NA	no	1	maternal	proband;	P_173; M_173
NA	no	1	maternal	proband;	P_292; M_292
NA	no	1	paternal	proband; f	P_386; F_386
NA	no	1	paternal	proband; f	P_422; F_422
NA	no	unphased	paternal	father	F_172
NA	no	3	maternal	proband;	P_174; M_174
NA	no	unphased	paternal	father	F_229
NA	no	unphased	maternal	mother	M_165
NA	no	3	maternal	mother; pr	M_68; P_68
NA	no	unphased	maternal	mother	M_209
NA	no	unphased	maternal	mother	M_2
NA	no	unphased	maternal	proband;	P_240; M_240
NA	no	unphased	maternal	mother	M_100
NA	no	unphased	maternal	mother	M_54

NA	no	1	NA	proband	P_104
NA	no	unphased	maternal	mother	M_319
NA	no	unphased	maternal	proband;	P_63; M_63
NA	no	unphased	paternal	father	F_93
NA	no	unphased	maternal	mother	M_254
NA	no	1	paternal	proband; f	P_252; F_252
NA	no	1	paternal	proband; f	P_271; F_271
NA	no	1	paternal	proband; f	P_165; F_165
NA	no	unphased	maternal	mother	M_99
NA	no	unphased	maternal	mother	M_263
NA	no	unphased	maternal	mother	M_253
NA	no	0	paternal	proband; f	P_262; F_262
NA	no	unphased	paternal	proband; f	P_431; F_431
NA	no	1	maternal	proband;	P_337; M_337
NA	no	unphased	maternal	mother	M_15
NA	no	1	paternal	proband; f	P_207; F_207
NA	no	unphased	maternal	mother	M_337
NA	no	unphased	maternal	mother	M_229
NA	no	unphased	maternal	mother	M_216
NA	no	unphased	maternal	mother	M_180
NA	no	unphased	paternal	father	F_86
NA	no	unphased	paternal	father	F_86
NA	no	0	maternal	proband;	P_359; M_359; O1_359; O2_359
NA	no	3	maternal	proband;	P_216; M_216
NA	no	1	paternal	proband; f	P_401; F_401
NA	no	unphased	maternal	mother; u	M_359; O3_359
NA	no	1	NA	proband	P_199
NA	no	unphased	paternal	father	F_421
NA	no	3	paternal	proband; f	P_181; F_181
NA	no	unphased	NA	father	F_116
NA	no	unphased	paternal	father	F_184
NA	no	1	NA	proband	P_117
NA	no	1	maternal	proband;	P_267; M_267
NA	no	1	paternal	father; pro	F_173; P_173
NA	no	2	paternal	proband; f	P_148; F_148
NA	no	1 2	maternal	proband;	P_167; M_167

NA	no	unphased	maternal	mother	M_100
NA	no	1	paternal	proband; f	P_335; F_335
NA	no	1	paternal	proband; f	P_238; F_238
NA	no	unphased	paternal	father	F_175
NA	no	unphased	maternal	mother	M_156
NA	no	unphased	maternal	mother	M_278
NA	no	1	paternal	proband; f	P_27; F_27
NA	no	unphased	maternal	mother	M_408
NA	no	unphased	paternal	father	F_346
NA	no	unphased	paternal	father	F_212
NA	no	1	paternal	proband; f	P_380; F_380
NA	no	1	maternal	proband;	P_359; M_359; O1_359; O3_359
NA	no	unphased	paternal	father	F_345
NA	no	unphased	paternal	father	F_411
NA	no	1	maternal	proband;	P_277; M_277
NA	no	unphased	maternal	mother	M_372
NA	no	1	maternal	proband;	P_431; M_431
NA	no	0	paternal	proband; f	P_89; F_89
NA	no	0	paternal	proband; f	P_143; F_143
NA	no	unphased	paternal	proband; f	P_171; F_171
NA	no	unphased	maternal	mother	M_63
NA	no	unphased	paternal	father	F_50
NA	no	1	paternal	proband; f	P_38; F_38
NA	no	unphased	paternal	father	F_277
NA	no	unphased	maternal	mother	M_204
NA	no	unphased	maternal	mother	M_204
NA	no	unphased	maternal	mother	M_13
NA	no	1	maternal	proband;	P_50; M_50
NA	no	unphased	paternal	father	F_172
NA	no	0	paternal	proband; f	P_160; F_160
NA	no	unphased	paternal	father	F_35
NA	no	unphased	paternal	father	F_251
NA	no	unphased	maternal	mother	M_298
NA	no	unphased	paternal	father	F_223
NA	no	1	maternal	proband;	P_50; M_50
NA	no	1	maternal	proband;	P_184; M_184

NA	no	unphased	maternal	mother	M_372
2	yes	1	DNV	proband	P_300
NA	no	unphased	maternal	mother	M_403
NA	no	unphased	paternal	father	F_39
NA	no	unphased	maternal	mother	M_387
NA	no	1	paternal	proband; f	P_367; F_367
NA	no	1	paternal	proband; f	P_55; F_55
NA	no	1	maternal	proband;	P_372; M_372
NA	no	unphased	maternal	mother	M_342
NA	no	unphased	paternal	proband; f	P_190; F_190
2	no	unphased	NA	proband	P_432
NA	no	0	maternal	proband;	P_42; M_42
NA	no	unphased	paternal	father	F_123
NA	no	unphased	paternal	father	F_397
NA	no	unphased	paternal	father	F_102
NA	no	1	maternal	proband;	P_299; M_299
NA	no	unphased	paternal	father	F_20
NA	no	unphased	maternal	mother	M_9
NA	no	1	maternal	proband;	P_360; M_360
NA	no	unphased	maternal	mother	M_359
NA	no	1	paternal	proband; f	P_78; F_78
NA	no	3 1	maternal	proband;	P_152; M_152
NA	no	unphased	paternal	proband; f	P_338; F_338
NA	no	1	NA	proband	P_240
NA	no	unphased	paternal	father	F_129
NA	no	1	paternal	proband; f	P_92; F_92
NA	no	unphased	paternal	father	F_118
NA	no	unphased	paternal	father	F_11
NA	no	unphased	maternal	mother	M_284
NA	no	0	maternal	proband;	P_31; M_31
NA	no	unphased	maternal	mother	M_256
NA	no	unphased	paternal	father	F_112
NA	no	unphased	paternal	father	F_424
NA	no	0	maternal	proband;	P_317; M_317
NA	no	unphased	maternal	mother	M_426
NA	no	unphased	paternal	father	F_68

NA	no	unphased	paternal	father	F_358
NA	no	1	maternal	proband;	P_306; M_306
NA	no	unphased	maternal	mother	M_48
NA	no	unphased	maternal	mother	M_67
NA	no	unphased	maternal	proband;	P_49; M_49
NA	no	unphased	NA	proband	P_152
NA	no	unphased	maternal	mother	M_67
NA	no	1	paternal	proband; f	P_312; F_312
NA	no	unphased	paternal	father	F_322
NA	no	unphased	paternal	father	F_334
NA	no	unphased	maternal	mother; si	M_234; S_234; P_234
NA	no	unphased	NA	aunt; uncl	O2_359; O3_359
NA	no	1	maternal	proband;	P_16; M_16
NA	no	unphased	paternal	father	F_11
NA	no	unphased	maternal	mother	M_161
NA	no	unphased	paternal	father	F_82
0	no	0	NA	proband	P_65
NA	no	1	paternal	proband; f	P_153; F_153
NA	no	0	maternal	proband;	P_114; M_114
NA	no	unphased	paternal	father	F_44
NA	no	unphased	maternal	mother	M_201
NA	no	unphased	paternal	father	F_159
NA	no	unphased	paternal	father	F_159
NA	no	1	maternal	proband;	P_239; M_239
NA	no	unphased	maternal	mother	M_437
NA	no	0	maternal	proband;	P_154; M_154; S_154
NA	no	2	paternal	proband; f	P_210; F_210
NA	no	1	paternal	proband; f	P_267; F_267; S_267
NA	no	unphased	NA	proband	P_160
NA	no	unphased	paternal	father	F_114
NA	no	1	maternal	proband;	P_127; M_127
NA	no	unphased	maternal	mother	M_285
NA	no	unphased	maternal	mother	M_411
NA	no	unphased	maternal	mother	M_97
NA	no	1	maternal	proband;	P_73; M_73
NA	no	unphased	maternal	mother	M_346

NA	no	unphased	paternal	father	F_269
NA	no	0	maternal	proband;	P_171; M_171
NA	no	unphased	paternal	father	F_259
NA	no	unphased	NA	proband	P_195
NA	no	unphased	maternal	mother	M_133
NA	no	unphased	maternal	mother	M_158
NA	no	1	maternal	proband;	P_360; M_360
NA	no	unphased	paternal	father	F_392
NA	no	unphased	paternal	father	F_256
NA	no	unphased	paternal	father	F_256
NA	no	1	paternal	proband; f	P_176; F_176
NA	no	1	maternal	proband;	P_263; M_263
NA	no	unphased	NA	mother	M_42
NA	no	unphased	paternal	father; sibl	F_328; S_328
NA	no	1	maternal	proband;	P_40; M_40
NA	no	1	maternal	proband;	P_157; M_157
NA	no	2 1	maternal	proband;	P_189; M_189
NA	no	0	paternal	proband; f	P_171; F_171
NA	no	1	maternal	proband;	P_200; M_200
NA	no	unphased	NA	cousin 1; c	O1_176; O2_176
NA	no	unphased	maternal	proband;	P_87; M_87
NA	no	unphased	maternal	mother	M_4
NA	no	unphased	paternal	father	F_321
NA	no	unphased	paternal	father	F_292
NA	no	unphased	NA	proband	P_420
NA	no	unphased	maternal	mother	M_293
NA	no	1	paternal	proband; f	P_223; F_223
NA	no	2	paternal	proband; f	P_392; F_392
NA	no	unphased	paternal	father	F_116
NA	no	unphased	NA	cousin; un	O1_136; O3_136
NA	no	1	NA	proband	P_361
NA	no	0	paternal	proband; f	P_297; F_297
NA	no	1	paternal	proband; f	P_49; F_49
NA	no	1	paternal	proband; f	P_244; F_244
NA	no	1	paternal	proband; f	P_383; F_383
NA	no	0	paternal	proband; f	P_237; F_237

NA	no	0	maternal	proband;	P_351; M_351
NA	no	1	paternal	proband; f	P_105; F_105
NA	no	unphased	maternal	mother	M_317
NA	no	1	paternal	proband; f	P_282; F_282
NA	no	unphased	paternal	father	F_146
NA	no	unphased	paternal	father	F_146
NA	no	unphased	paternal	father	F_270
NA	no	unphased	paternal	father	F_381
NA	no	1	maternal	proband;	P_120; M_120
NA	no	unphased	paternal	father	F_97
NA	no	unphased	maternal	mother	M_27
NA	no	1	maternal	proband;	P_94; M_94
NA	no	2	paternal	proband; f	P_46; F_46
NA	no	unphased	maternal	mother	M_203
NA	no	unphased	paternal	father	F_140
NA	no	1	paternal	proband; f	P_328; F_328; S_328
NA	no	1	maternal	proband;	P_205; M_205
NA	no	1	maternal	proband;	P_69; M_69
NA	no	unphased	paternal	father	F_288
NA	no	1	paternal	proband; f	P_112; F_112
NA	no	1	paternal	proband; f	P_114; F_114
NA	no	unphased	maternal	mother	M_349
NA	no	unphased	paternal	father	F_39
NA	no	unphased	paternal	father	F_10
NA	no	1	paternal	proband; f	P_112; F_112
NA	no	1	paternal	proband; f	P_47; F_47
NA	no	unphased	paternal	father	F_362
NA	no	unphased	paternal	father	F_80
NA	no	unphased	maternal	half-sibling	S1_395; M_395
NA	no	1	maternal	proband;	P_140; M_140
NA	no	unphased	maternal	mother	M_406
NA	no	unphased	maternal	proband;	P_122; M_122
NA	no	unphased	paternal	proband; f	P_138; F_138
NA	no	unphased	maternal	mother	M_85
NA	no	unphased	maternal	mother	M_169
NA	no	1	paternal	proband; f	P_77; F_77

NA	no	unphased	maternal	mother	M_167
NA	no	unphased	paternal	father	F_80
NA	no	1	paternal	proband; f	P_105; F_105
NA	no	unphased	maternal	mother	M_432
NA	no	1	paternal	proband; f	P_409; F_409
NA	no	unphased	paternal	father	F_47
NA	no	unphased	NA	mother	M_67
NA	no	unphased	paternal	father	F_366
NA	no	unphased	maternal	mother	M_105
NA	no	1	paternal	proband; f	P_318; F_318
NA	no	3	maternal	proband;	P_272; M_272
NA	no	unphased	maternal	mother	M_237
NA	no	unphased	maternal	mother	M_75
NA	no	1	paternal	proband; f	P_51; F_51
NA	no	1	paternal	proband; f	P_284; F_284
NA	no	0	maternal	proband;	P_150; M_150
NA	no	1	paternal	proband; f	P_47; F_47
NA	no	unphased	NA	proband	P_64
NA	no	unphased	NA	aunt	O2_359
NA	no	unphased	paternal	proband; f	P_244; F_244
NA	no	unphased	paternal	father	F_62
NA	no	1	maternal	proband;	P_110; M_110
NA	no	unphased	NA	father	F_358
NA	no	unphased	NA	father	F_72
NA	no	unphased	maternal	mother	M_341
NA	no	unphased	NA	father	F_16
2	yes	unphased	DNV	proband	P_301
NA	no	unphased	maternal	mother	M_167
NA	no	0	paternal	proband; f	P_112; F_112
NA	no	unphased	paternal	father	F_351
NA	no	unphased	NA	proband	P_170
NA	no	1	maternal	proband;	P_13; M_13
2	no	1	DNV	proband	P_240
NA	no	unphased	maternal	mother	M_163
NA	no	unphased	maternal	mother	M_137
NA	no	unphased	paternal	father; sibl	F_101; S_101

NA	no	unphased	paternal	father	F_364
NA	no	unphased	NA	mother	M_351
NA	no	unphased	paternal	father	F_371
NA	no	1	maternal	proband;	P_4; M_4
NA	no	0	paternal	proband; f	P_409; F_409
NA	no	unphased	paternal	proband; f	P_13; F_13
NA	no	0	maternal	proband;	P_355; M_355
NA	no	0	maternal	proband;	P_116; M_116
NA	no	unphased	paternal	father	F_221
NA	no	unphased	maternal	mother	M_94
NA	no	1	NA	proband	P_214
NA	no	unphased	paternal	father	F_359
NA	no	unphased	maternal	mother	M_300
NA	no	1	paternal	proband; f	P_110; F_110
NA	no	1	maternal	proband;	P_153; M_153
NA	no	unphased	maternal	mother; si	M_328; S_328
NA	no	unphased	maternal	mother	M_42
NA	risk	unphased	maternal	proband;	P_357; M_357; S_357
NA	no	1	maternal	proband;	P_30; M_30
NA	no	unphased	maternal	mother	M_313
NA	no	0	paternal	proband; f	P_177; F_177
NA	no	1	maternal	proband;	P_13; M_13
NA	no	unphased	maternal	mother	M_48
NA	no	unphased	paternal	father	F_213
NA	no	unphased	paternal	father	F_20
NA	no	1	maternal	proband;	P_231; M_231
NA	no	1	paternal	proband; f	P_232; F_232
NA	no	unphased	maternal	mother	M_83
NA	no	unphased	paternal	proband; f	P_32; F_32
NA	no	unphased	maternal	mother	M_232
NA	no	1	maternal	proband;	P_53; M_53
NA	no	unphased	maternal	proband;	P_201; M_201
NA	no	unphased	paternal	father	F_239
NA	no	unphased	paternal	father	F_31
NA	no	unphased	maternal	mother	M_16
NA	no	1	paternal	proband; f	P_239; F_239

NA	no	0	paternal	proband; f	P_225; F_225
NA	no	unphased	NA	aunt	O2_136
NA	no	unphased	paternal	father	F_392
NA	no	unphased	maternal	mother	M_4
NA	no	unphased	paternal	proband; f	P_302; F_302
NA	no	unphased	NA	proband	P_156
NA	no	unphased	NA	proband	P_399
NA	no	unphased	NA	proband	P_405
NA	no	1	paternal	proband; f	P_229; F_229
NA	no	unphased	paternal	father	F_238
NA	no	unphased	paternal	proband; f	P_221; F_221
NA	no	unphased	maternal	mother	M_43
NA	no	0	maternal	proband;	P_108; M_108
NA	no	1	paternal	proband; f	P_35; F_35
NA	no	unphased	paternal	father	F_267
NA	no	1	maternal	proband;	P_312; M_312
NA	no	1	maternal	proband;	P_206; M_206
NA	yes	unphased	NA	proband	P_399
NA	no	unphased	paternal	father	F_243
NA	no	0	paternal	proband; f	P_20; F_20
NA	no	unphased	maternal	mother	M_339
NA	no	unphased	paternal	father	F_195
NA	no	unphased	paternal	father	F_395
NA	no	1	paternal	proband; f	P_227; F_227
NA	no	1	paternal	proband; f	P_109; F_109
NA	no	unphased	paternal	father	F_164
NA	no	1	maternal	proband;	P_205; M_205
NA	no	3	maternal	proband;	P_123; M_123
NA	no	unphased	paternal	father	F_423
NA	no	unphased	paternal	father	F_251
NA	no	unphased	paternal	father	F_325
NA	no	unphased	NA	sibling	S_339
NA	no	unphased	paternal	father	F_282
NA	no	1	paternal	proband; f	P_390; F_390
NA	no	2	paternal	proband; f	P_104; F_104
NA	no	unphased	paternal	father	F_259

NA	no	0	maternal	proband;	P_428; M_428
NA	no	unphased	NA	father	F_51
NA	no	1	paternal	proband; f	P_67; F_67
NA	no	0	paternal	proband; f	P_156; F_156
NA	no	unphased	paternal	father	F_195
NA	no	0	maternal	proband;	P_111; M_111
NA	no	unphased	maternal	mother	M_58
NA	no	unphased	paternal	proband; f	P_9; F_9
NA	no	unphased	paternal	proband; f	P_255; F_255
NA	no	1	maternal	proband;	P_429; M_429
NA	no	unphased	maternal	mother	M_35
NA	no	unphased	maternal	mother	M_17
NA	no	unphased	NA	proband	P_4
NA	no	1	paternal	proband; f	P_78; F_78
NA	no	unphased	paternal	father	F_323
NA	no	1	maternal	proband;	P_205; M_205
NA	no	unphased	paternal	father	F_299
NA	no	unphased	paternal	father	F_355
NA	no	unphased	paternal	father	F_404
NA	no	unphased	maternal	mother	M_181
NA	no	unphased	maternal	mother	M_187
NA	no	1	maternal	proband;	P_193; M_193
NA	no	unphased	maternal	mother	M_132
NA	no	unphased	NA	father	F_190
NA	no	unphased	maternal	mother	M_345
NA	no	unphased	maternal	mother	M_352
NA	no	unphased	NA	mother	M_301
NA	no	unphased	paternal	father	F_421
NA	no	unphased	paternal	father	F_239
NA	no	unphased	maternal	mother	M_213
NA	no	1	paternal	proband; f	P_228; F_228
NA	no	unphased	paternal	father	F_249
NA	no	1	maternal	proband;	P_325; M_325
NA	no	1	maternal	proband;	P_400; M_400
NA	no	1	maternal	proband;	P_232; M_232
NA	no	1	maternal	proband;	P_212; M_212

NA	no	3	maternal	proband;	P_164; M_164
NA	no	unphased	paternal	father	F_291
NA	no	unphased	maternal	mother	M_420
NA	no	1	paternal	proband; f	P_94; F_94
NA	no	unphased	paternal	father	F_165
NA	no	1	paternal	proband; f	P_118; F_118
NA	no	1	maternal	proband;	P_248; M_248
NA	no	unphased	paternal	father	F_178
NA	no	unphased	NA	cousin 2	O2_176
NA	no	unphased	maternal	proband;	P_389; M_389
NA	no	1	maternal	proband;	P_298; M_298
NA	no	1	maternal	proband;	P_90; M_90
NA	no	unphased	NA	mother	M_125
NA	no	unphased	maternal	proband;	P_111; M_111
NA	no	1	paternal	proband; f	P_259; F_259
NA	no	1	maternal	proband;	P_164; M_164
NA	no	unphased	maternal	mother	M_292
NA	no	unphased	paternal	father	F_165
NA	no	unphased	paternal	father	F_366
NA	no	1	paternal	proband; f	P_353; F_353
NA	no	0	maternal	proband;	P_328; M_328; S_328
NA	no	1	paternal	proband; f	P_22; F_22
NA	no	unphased	maternal	mother	M_149
NA	no	1	maternal	proband;	P_387; M_387
NA	no	0	paternal	proband; f	P_182; F_182
NA	no	1	NA	proband	P_330
NA	no	1	paternal	proband; f	P_94; F_94
NA	no	unphased	NA	half-sibling	S1_396
NA	no	unphased	paternal	father	F_385
NA	no	unphased	maternal	mother; si	M_368; S2_368
NA	no	unphased	paternal	father	F_286
NA	no	unphased	paternal	proband; f	P_372; F_372
NA	no	unphased	maternal	mother	M_104
NA	no	unphased	maternal	mother	M_290
NA	no	unphased	paternal	father	F_324
NA	no	0	maternal	proband;	P_45; M_45

NA	no	3	maternal	mother; pr	M_68; P_68
NA	no	unphased	maternal	mother; si	M_234; S_234; P_234
NA	no	1	maternal	proband;	P_187; M_187
NA	no	1	NA	proband	P_337
NA	no	1	paternal	proband; f	P_40; F_40
NA	no	1	maternal	proband;	P_210; M_210
NA	no	1	paternal	proband; f	P_17; F_17
NA	no	unphased	NA	cousin 1; c	O1_176; O2_176
NA	no	3	maternal	proband;	P_149; M_149
NA	no	1	paternal	proband; f	P_228; F_228
NA	no	unphased	paternal	father	F_428
NA	no	1	maternal	proband;	P_79; M_79
NA	no	unphased	maternal	mother	M_392
NA	no	3	paternal	father; pro	F_159; P_159
NA	no	unphased	NA	proband	P_238
NA	no	unphased	NA	proband	P_189
NA	no	unphased	paternal	father	F_173
NA	no	unphased	paternal	father	F_294
NA	no	1	paternal	father; pro	F_173; P_173
NA	no	1	paternal	proband; f	P_372; F_372
NA	no	unphased	maternal	mother	M_294
NA	no	2	maternal	proband;	P_120; M_120
NA	no	unphased	paternal	father	F_30
NA	no	unphased	paternal	father	F_257
NA	no	0	paternal	proband; f	P_404; F_404
NA	no	unphased	paternal	father	F_338
NA	no	unphased	paternal	father	F_246
2	no	1	DNV	proband	P_429
NA	no	unphased	paternal	father	F_229
NA	no	1	maternal	proband;	P_221; M_221
NA	no	unphased	maternal	mother	M_438
NA	no	unphased	maternal	mother	M_117
NA	no	unphased	maternal	mother	M_3
NA	no	unphased	maternal	mother	M_186
2	yes	unphased	DNV	proband	P_16
NA	no	0	maternal	proband;	P_191; M_191

NA	no	1	maternal	proband;	P_402; M_402
NA	no	unphased	paternal	father	F_230
NA	no	1	maternal	proband;	P_171; M_171
NA	no	1	maternal	proband;	P_429; M_429
NA	no	unphased	maternal	mother	M_22
NA	no	unphased	maternal	mother	M_116
NA	no	unphased	NA	cousin	O1_359
NA	no	1	paternal	proband; f	P_26; F_26
NA	no	unphased	maternal	mother	M_250
NA	no	2	maternal	proband;	P_327; M_327
NA	no	unphased	maternal	mother	M_73
NA	no	unphased	maternal	mother	M_231
NA	no	unphased	maternal	proband;	P_363; M_363
NA	no	1	paternal	proband; f	P_49; F_49
NA	no	unphased	paternal	father	F_25
NA	no	unphased	paternal	father	F_306
NA	no	unphased	paternal	father	F_41
NA	no	1	paternal	proband; f	P_228; F_228
NA	no	3	NA	proband	P_381
NA	no	unphased	maternal	mother	M_171
NA	no	unphased	maternal	mother	M_385
NA	no	unphased	maternal	mother	M_343
NA	no	unphased	maternal	mother	M_142
NA	no	unphased	paternal	father	F_94
NA	no	unphased	maternal	mother	M_386
NA	no	unphased	NA	proband	P_387
NA	no	1	maternal	proband;	P_371; M_371
NA	no	1	paternal	proband; f	P_282; F_282
NA	no	unphased	maternal	mother	M_252
NA	no	unphased	maternal	mother	M_78
NA	no	unphased	maternal	mother	M_438
NA	no	1	paternal	proband; f	P_78; F_78
NA	no	1	paternal	proband; f	P_406; F_406
NA	no	unphased	maternal	mother	M_145
NA	no	1	paternal	father; pro	F_173; P_173
NA	no	1	maternal	proband;	P_116; M_116

NA	no	1	NA	proband	P_426
NA	no	unphased	maternal	mother	M_211
NA	no	1	NA	proband	P_330
NA	no	0	paternal	proband; f	P_109; F_109
NA	no	unphased	maternal	mother	M_231
NA	no	unphased	paternal	father	F_13
NA	no	unphased	maternal	mother	M_30
NA	no	unphased	maternal	mother	M_384
NA	no	unphased	NA	father	F_51
NA	no	1	paternal	proband; f	P_82; F_82
NA	no	1	paternal	proband; f	P_4; F_4
NA	no	1	maternal	proband;	P_425; M_425
NA	no	unphased	maternal	mother	M_42
NA	no	1	NA	proband	P_378
NA	no	1	maternal	proband;	P_14; M_14
NA	no	unphased	paternal	father	F_381
NA	no	unphased	maternal	mother	M_34
NA	no	unphased	paternal	father	F_349
NA	no	unphased	NA	proband	P_218
NA	no	unphased	maternal	mother	M_271
NA	no	unphased	maternal	mother	M_121
NA	no	unphased	paternal	proband; f	P_203; F_203
NA	no	1	paternal	proband; f	P_44; F_44
NA	no	1	maternal	proband;	P_281; M_281
NA	no	unphased	maternal	mother	M_143
NA	no	unphased	NA	cousin; au	O1_370; O2_370
NA	no	1	maternal	proband;	P_260; M_260
NA	no	unphased	NA	cousin	O1_370
NA	no	unphased	maternal	mother	M_360
NA	no	unphased	paternal	father	F_148
NA	no	1	maternal	mother; pr	M_151; P_151
NA	no	unphased	maternal	mother	M_432
NA	no	unphased	paternal	father	F_228
NA	no	unphased	NA	sibling	S_150
NA	no	unphased	maternal	mother	M_304
NA	no	1	maternal	proband;	P_45; M_45

NA	no	1	paternal	proband; f	P_253; F_253
NA	no	0	maternal	mother; pr	M_66; P_66
NA	no	unphased	NA	proband	P_161
NA	no	unphased	NA	father	F_112
NA	no	1	paternal	proband; f	P_249; F_249
NA	no	unphased	maternal	mother	M_329
NA	no	3	paternal	proband; f	P_381; F_381
NA	yes	3	paternal	proband; f	P_149; F_149
NA	no	unphased	maternal	mother	M_295
NA	no	0	maternal	proband;	P_381; M_381
NA	no	unphased	maternal	mother	M_117
NA	no	unphased	maternal	mother	M_434
NA	no	2	paternal	proband; f	P_130; F_130
NA	no	1	maternal	proband;	P_379; M_379
NA	no	unphased	maternal	mother	M_162
NA	no	1	maternal	proband;	P_5; M_5
NA	no	unphased	paternal	father	F_48
NA	no	unphased	paternal	father	F_44
NA	no	1	paternal	proband; f	P_333; F_333
NA	no	unphased	maternal	mother	M_52
NA	no	unphased	maternal	mother	M_104
NA	no	unphased	maternal	mother	M_121
NA	no	unphased	maternal	mother	M_80
NA	no	3	paternal	father; pro	F_245; P_245
NA	no	unphased	paternal	father	F_67
NA	no	unphased	paternal	father	F_203
NA	no	unphased	NA	proband	P_353
NA	no	unphased	paternal	father	F_2
NA	no	1	maternal	proband;	P_177; M_177
NA	no	1	maternal	proband;	P_88; M_88
NA	no	0	paternal	proband; f	P_335; F_335
NA	no	unphased	maternal	mother	M_333
NA	no	1	maternal	proband;	P_118; M_118
NA	no	unphased	paternal	father	F_268
NA	no	1	paternal	proband; f	P_360; F_360
NA	no	unphased	NA	mother	M_174

NA	no	1	paternal	proband; f	P_136; F_136
NA	no	unphased	paternal	father	F_140
NA	no	unphased	maternal	mother	M_375
NA	no	unphased	maternal	mother	M_363
NA	no	unphased	paternal	father	F_259
NA	no	2	maternal	proband;	P_16; M_16
NA	no	unphased	paternal	father	F_124
NA	no	unphased	paternal	father	F_99
NA	no	unphased	paternal	father	F_363
NA	no	unphased	NA	proband	P_199
NA	no	1	maternal	proband;	P_187; M_187
NA	no	1	NA	proband	P_185
NA	no	unphased	NA	sibling	S2_368
NA	no	unphased	paternal	father	F_159
NA	no	1	NA	proband	P_18
NA	no	1	paternal	proband; f	P_129; F_129
NA	no	unphased	maternal	mother	M_53
NA	no	1	maternal	proband;	P_180; M_180
NA	no	unphased	paternal	father	F_289
NA	yes	1	paternal	proband; f	P_71; F_71
NA	no	unphased	paternal	father	F_7
NA	no	unphased	NA	cousin	O1_398
NA	no	1	maternal	proband;	P_384; M_384
NA	no	unphased	maternal	mother; si	M_412; S_412
NA	no	0	paternal	proband; f	P_87; F_87
NA	no	unphased	maternal	mother	M_408
NA	no	unphased	paternal	father	F_373
NA	no	1	paternal	father; pro	F_159; P_159
NA	no	unphased	paternal	father	F_148
NA	no	1	NA	proband	P_233
NA	no	1	paternal	proband; f	P_139; F_139
NA	no	unphased	paternal	father	F_206
NA	no	unphased	NA	father	F_206
NA	no	unphased	paternal	father	F_92
NA	no	unphased	maternal	mother	M_53
NA	no	unphased	paternal	father	F_102

NA	no	1	paternal	proband; f	P_260; F_260
NA	no	unphased	paternal	father	F_302
NA	no	1	maternal	proband;	P_254; M_254
NA	no	unphased	paternal	father	F_40
NA	no	1	paternal	proband; f	P_122; F_122
NA	no	unphased	maternal	mother	M_421
NA	no	unphased	paternal	father	F_28
NA	no	1	paternal	proband; f	P_139; F_139
NA	no	unphased	NA	mother	M_329
NA	no	1	maternal	proband;	P_127; M_127
NA	no	1	NA	proband	P_189
NA	no	1	paternal	proband; f	P_118; F_118
NA	no	unphased	paternal	father	F_250
NA	no	unphased	maternal	mother	M_220
NA	no	1	NA	proband	P_35
NA	no	1	paternal	proband; f	P_435; F_435
NA	no	0	paternal	proband; f	P_287; F_287
NA	no	unphased	paternal	father	F_31
NA	no	1	paternal	proband; f	P_230; F_230
NA	no	1	paternal	proband; f	P_336; F_336
NA	no	unphased	paternal	father	F_105
NA	no	0	NA	proband	P_385
NA	no	2	maternal	proband;	P_113; M_113
NA	no	unphased	maternal	mother	M_272
NA	no	unphased	maternal	mother	M_441
NA	no	unphased	paternal	father	F_262
NA	no	1	paternal	proband; f	P_230; F_230
NA	no	1	paternal	proband; f	P_172; F_172
NA	no	1	maternal	proband;	P_273; M_273
NA	no	unphased	maternal	mother	M_329
NA	no	unphased	paternal	proband; f	P_287; F_287
NA	no	unphased	maternal	mother	M_201
NA	no	unphased	maternal	mother	M_179
NA	no	unphased	maternal	mother	M_28
NA	no	1	maternal	proband;	P_422; M_422
2	no	unphased	DNV	proband	P_176

NA	no	1	paternal	proband; f	P_335; F_335
NA	no	unphased	maternal	mother	M_213
NA	no	0	paternal	proband; f	P_393; F_393
NA	no	unphased	paternal	father	F_374
NA	no	unphased	paternal	father	F_360
NA	no	unphased	paternal	father	F_38
NA	no	unphased	maternal	mother	M_437
NA	no	1	paternal	proband; f	P_97; F_97
NA	no	unphased	maternal	mother	M_21
NA	no	unphased	paternal	father	F_414
NA	no	2	maternal	proband;	P_278; M_278
NA	no	unphased	paternal	father	F_172
NA	no	unphased	paternal	father	F_14
NA	no	unphased	maternal	mother	M_379
2	no	1	DNV	proband	P_129
NA	no	2	paternal	proband; f	P_175; F_175
NA	no	unphased	maternal	mother	M_13
NA	no	unphased	NA	father	F_79
NA	no	unphased	NA	cousin	O1_398
NA	no	1	maternal	proband;	P_315; M_315
NA	no	1	paternal	proband; f	P_363; F_363
NA	no	unphased	paternal	father	F_87
NA	no	unphased	paternal	father	F_308
NA	no	unphased	paternal	father	F_104
NA	no	unphased	maternal	mother	M_289
NA	no	unphased	maternal	mother	M_55
NA	no	unphased	maternal	mother	M_55
NA	no	unphased	paternal	father	F_258
NA	no	unphased	paternal	father	F_116
NA	no	unphased	maternal	mother	M_369
NA	no	unphased	maternal	mother	M_91
NA	no	1	maternal	proband;	P_240; M_240
NA	no	unphased	maternal	mother	M_242
NA	no	unphased	NA	proband	P_235
NA	no	unphased	maternal	proband;	P_375; M_375
NA	no	1	paternal	proband; f	P_240; F_240

NA	no	1	maternal	proband;	P_422; M_422
NA	no	2	paternal	proband; f	P_326; F_326
NA	no	unphased	NA	proband	P_307
NA	no	0	maternal	proband;	P_114; M_114
NA	no	1	paternal	proband; f	P_83; F_83
NA	no	2	maternal	proband;	P_215; M_215
NA	no	1	maternal	proband;	P_50; M_50
NA	no	unphased	maternal	mother	M_85
NA	no	0	maternal	proband;	P_188; M_188
NA	no	1	maternal	proband;	P_255; M_255
NA	no	2	paternal	proband; f	P_141; F_141
NA	no	3	maternal	proband;	P_80; M_80
NA	no	unphased	maternal	proband;	P_223; M_223
NA	no	unphased	paternal	father	F_56
NA	no	1	paternal	proband; f	P_429; F_429
NA	no	0	maternal	mother; pr	M_433; P_433
NA	no	unphased	maternal	mother	M_211
NA	no	unphased	maternal	mother	M_395
NA	no	1	paternal	proband; f	P_377; F_377
NA	no	unphased	NA	mother	M_231
NA	no	unphased	maternal	mother	M_96
NA	no	1	paternal	proband; f	P_48; F_48
NA	no	1	paternal	proband; f	P_83; F_83
NA	no	unphased	maternal	mother	M_349
NA	no	3	NA	proband	P_217
NA	no	unphased	maternal	mother	M_393
NA	no	unphased	maternal	mother	M_210
NA	no	1	paternal	proband; f	P_379; F_379
NA	no	1	paternal	proband; f	P_128; F_128
NA	no	unphased	maternal	mother	M_177
NA	no	1	paternal	proband; f	P_349; F_349
NA	no	0	paternal	proband; f	P_329; F_329
NA	no	unphased	maternal	mother	M_295
NA	no	unphased	paternal	father	F_321
NA	no	unphased	paternal	father	F_295
NA	no	unphased	NA	uncle	O3_359

NA	no	0	maternal	proband;	P_125; M_125
NA	no	unphased	paternal	father	F_289
NA	no	unphased	maternal	mother	M_320
2	yes	1	DNV	proband	P_374
NA	no	unphased	paternal	father	F_344
NA	no	2	maternal	proband;	P_257; M_257
NA	no	unphased	paternal	father	F_301
NA	no	1	maternal	proband;	P_112; M_112
2	no	1	DNV	proband	P_114
NA	no	unphased	paternal	father	F_285
2	no	unphased	NA	proband	P_21
NA	no	unphased	maternal	mother	M_258
NA	no	1	NA	proband	P_339
NA	no	unphased	paternal	father	F_148
NA	no	unphased	paternal	father	F_441
NA	no	unphased	maternal	mother	M_432
NA	no	unphased	paternal	father	F_265
NA	no	unphased	paternal	father	F_169
NA	no	unphased	maternal	mother	M_409
NA	no	unphased	maternal	mother	M_210
NA	no	1	maternal	proband;	P_13; M_13
NA	no	1	maternal	proband;	P_341; M_341
NA	no	2	maternal	proband;	P_265; M_265
NA	no	1	paternal	proband; f	P_322; F_322
NA	no	unphased	paternal	father	F_149
NA	no	unphased	maternal	mother	M_213
NA	no	1	maternal	proband;	P_183; M_183
NA	no	unphased	NA	cousin; au	O1_136; O2_136
NA	no	unphased	paternal	father; sibl	F_412; S_412
2	no	1	DNV	proband	P_274
NA	no	1	paternal	proband; f	P_293; F_293
NA	no	unphased	NA	proband	P_150
NA	no	unphased	maternal	mother	M_61
NA	no	1	paternal	proband; f	P_215; F_215
NA	no	0	maternal	proband;	P_184; M_184
NA	no	unphased	paternal	father	F_119

NA		no	unphased	paternal	father	F_304
NA		no	1	maternal	proband;	P_104; M_104
NA		no	unphased	paternal	father	F_169
NA		no	0	maternal	proband;	P_177; M_177
NA		no	unphased	paternal	father	F_76
NA		no	unphased	paternal	father	F_273
NA		no	unphased	paternal	proband; f	P_138; F_138
NA		no	unphased	paternal	father	F_181
NA		no	unphased	maternal	mother	M_221
NA		no	1	paternal	proband; f	P_157; F_157
NA		no	unphased	maternal	mother	M_59
NA		no	unphased	maternal	mother	M_190
NA		no	unphased	paternal	father	F_407
NA		no	unphased	maternal	mother	M_233
NA		no	1	maternal	proband;	P_428; M_428
NA		no	unphased	paternal	father	F_141
NA		no	unphased	maternal	mother	M_75
NA		no	unphased	paternal	father	F_344
NA		no	unphased	maternal	mother	M_256
NA		no	0	paternal	proband; f	P_24; F_24
NA		no	0	paternal	proband; f	P_215; F_215
NA		no	unphased	paternal	proband; f	P_262; F_262
NA		no	1	maternal	proband;	P_35; M_35
NA		no	unphased	maternal	mother	M_172
NA		no	unphased	paternal	father	F_124
NA		no	1	paternal	proband; f	P_255; F_255
NA		no	unphased	maternal	mother	M_381
NA		no	unphased	NA	proband	P_330
NA		no	3	maternal	proband;	P_393; M_393
NA		no	unphased	maternal	mother	M_428
NA		no	unphased	paternal	father	F_228
NA		no	unphased	paternal	father	F_244
	2	no	unphased	DNV	proband	P_248
NA		no	unphased	NA	mother	M_363
NA		no	1	paternal	proband; f	P_83; F_83
NA		no	unphased	maternal	mother	M_9

NA	no	1	paternal	proband; f	P_130; F_130
NA	no	unphased	maternal	mother	M_33
NA	no	1	maternal	proband;	P_351; M_351
NA	no	unphased	maternal	mother	M_343
NA	no	unphased	maternal	mother	M_138
NA	no	unphased	paternal	father	F_206
NA	no	unphased	paternal	proband; f	P_35; F_35
NA	no	1	maternal	proband;	P_75; M_75
NA	no	unphased	paternal	father	F_76
NA	no	unphased	maternal	mother	M_51
NA	no	unphased	maternal	mother	M_94
NA	no	unphased	NA	aunt	O2_370
NA	no	1	paternal	proband; f	P_103; F_103
NA	no	unphased	NA	father	F_269
NA	no	1	maternal	mother; pr	M_437; P_437
NA	no	1	NA	proband	P_330
NA	no	0	maternal	proband;	P_207; M_207
NA	no	unphased	paternal	father	F_142
NA	no	unphased	maternal	mother	M_173
NA	no	unphased	maternal	mother	M_14
NA	no	unphased	paternal	father	F_138
NA	no	unphased	paternal	father	F_243
NA	no	unphased	paternal	father	F_380
NA	no	unphased	paternal	father	F_212
NA	no	0	maternal	proband;	P_45; M_45
NA	no	1	paternal	father; pro	F_54; P_54
NA	no	1	maternal	proband;	P_46; M_46
NA	no	2	paternal	proband; f	P_116; F_116
NA	no	unphased	NA	mother	M_129
NA	no	1	paternal	proband; f	P_344; F_344
NA	no	1	paternal	proband; f	P_98; F_98
NA	no	1	NA	proband	P_214
NA	no	unphased	paternal	father	F_382
NA	no	1	paternal	proband; f	P_400; F_400; S1_400; S2_400
NA	no	0	paternal	proband; f	P_220; F_220
NA	no	unphased	maternal	mother	M_93

NA	no	unphased	maternal	mother	M_51
NA	no	1	paternal	proband; f	P_18; F_18
NA	no	unphased	paternal	father	F_395
NA	no	1	NA	proband	P_152
NA	no	1	maternal	proband;	P_49; M_49
NA	no	1	maternal	proband;	P_241; M_241
NA	no	unphased	paternal	sibling; fat	S_166; F_166
NA	no	1	paternal	proband; f	P_27; F_27
NA	no	1	paternal	proband; f	P_312; F_312
NA	no	1	maternal	proband;	P_320; M_320
NA	no	unphased	NA	sibling; pro	S_234; P_234
NA	no	unphased	paternal	father	F_156
NA	no	unphased	paternal	father	F_298
NA	no	unphased	maternal	mother	M_161
NA	no	unphased	paternal	father	F_332
NA	no	0	paternal	proband; f	P_34; F_34
NA	no	2	paternal	proband; f	P_223; F_223
NA	no	unphased	paternal	father	F_141
NA	no	unphased	maternal	mother	M_68
NA	no	unphased	paternal	proband; f	P_4; F_4
NA	no	unphased	NA	proband	P_201
NA	no	1	paternal	proband; f	P_174; F_174
NA	no	1	paternal	proband; f	P_232; F_232
NA	no	unphased	paternal	father	F_144
NA	no	unphased	paternal	father	F_154
NA	no	1 0	maternal	proband;	P_205; M_205
NA	no	1	maternal	proband;	P_189; M_189
NA	no	unphased	NA	father	F_275
NA	no	unphased	maternal	mother	M_206
NA	no	unphased	maternal	mother	M_307
NA	no	0	paternal	proband; f	P_230; F_230
NA	no	0	paternal	proband; f	P_31; F_31
NA	no	unphased	maternal	mother	M_124
NA	no	1	maternal	proband;	P_28; M_28; S_28
NA	no	1	paternal	proband; f	P_257; F_257
NA	no	unphased	paternal	father	F_250

NA	no	unphased	paternal	father	F_139
NA	no	unphased	maternal	mother	M_186
NA	no	unphased	paternal	father	F_22
NA	no	0	paternal	proband; f	P_182; F_182
NA	no	unphased	paternal	father	F_148
NA	no	1	maternal	proband;	P_168; M_168
NA	no	unphased	maternal	mother	M_180
NA	no	1	maternal	proband;	P_185; M_185
NA	no	unphased	maternal	mother	M_354
NA	no	unphased	maternal	mother	M_182
NA	yes	1	NA	proband	P_167
NA	no	0	maternal	proband;	P_45; M_45
NA	no	unphased	maternal	mother	M_275
NA	no	0	maternal	proband;	P_46; M_46
NA	no	unphased	maternal	mother	M_220
NA	no	unphased	paternal	proband; f	P_53; F_53
NA	no	unphased	NA	proband	P_279
NA	no	unphased	NA	sibling	S_376
NA	no	unphased	NA	sibling	S_376
NA	no	unphased	paternal	father	F_333
NA	no	unphased	NA	proband	P_234
NA	no	1	paternal	proband; f	P_184; F_184
NA	no	unphased	NA	proband	P_283
NA	no	unphased	maternal	mother	M_297
NA	no	unphased	paternal	father	F_428
NA	no	unphased	maternal	mother	M_313
NA	no	1	paternal	proband; f	P_408; F_408
NA	no	1	maternal	proband;	P_77; M_77
NA	no	unphased	maternal	mother	M_226
NA	no	unphased	maternal	mother	M_48
NA	no	1	paternal	proband; f	P_85; F_85
NA	no	1	maternal	proband;	P_90; M_90
NA	no	1	maternal	proband;	P_230; M_230
NA	yes	1	NA	proband	P_219
2	yes	1	DNV	proband	P_172
NA	no	unphased	paternal	father	F_272

NA	no	1	maternal	proband;	P_27; M_27
NA	no	1	maternal a	proband;	P_188; M_188; F_188
NA	no	unphased	maternal	proband;	P_341; M_341
NA	no	1	maternal	proband;	P_167; M_167
NA	no	2	maternal	mother; pr	M_59; P_59
NA	no	1	maternal	proband;	P_298; M_298
NA	no	unphased	NA	father	F_16
NA	no	1	paternal	proband; f	P_313; F_313
NA	no	0	NA	proband	P_331
NA	no	unphased	paternal	father	F_348
NA	no	1	paternal	proband; f	P_82; F_82
2	no	1	DNV	proband	P_295
NA	no	unphased	NA	proband	P_204
NA	no	unphased	paternal	father	F_143
2	no	1	DNV	proband	P_243
NA	no	unphased	paternal	father	F_395
NA	no	1	NA	proband	P_358
NA	no	0	paternal	proband; f	P_109; F_109
NA	no	3	maternal	proband;	P_352; M_352
NA	no	0	maternal	proband;	P_122; M_122
NA	no	unphased	NA	proband; s	P_412; S_412
NA	no	1	paternal	proband; f	P_139; F_139
NA	no	unphased	maternal	mother	M_231
NA	no	unphased	maternal	proband;	P_297; M_297
NA	no	1	maternal	proband;	P_415; M_415
NA	no	1	paternal	proband; f	P_353; F_353
NA	no	1	paternal	proband; f	P_102; F_102
NA	no	unphased	maternal	mother	M_285
NA	no	unphased	maternal	mother	M_16
NA	no	1	paternal	proband; f	P_440; F_440
NA	no	unphased	NA	proband	P_374
NA	no	unphased	NA	mother	M_397
NA	no	1	paternal	proband; f	P_171; F_171
NA	no	unphased	paternal	father	F_358
NA	no	1	maternal	proband;	P_375; M_375
NA	no	unphased	maternal	mother	M_201

NA	no	unphased	paternal	father	F_226
NA	no	unphased	maternal	proband;	P_421; M_421
NA	no	1	paternal	proband; f	P_271; F_271
NA	no	unphased	maternal	mother	M_102
NA	no	0 1	maternal	proband;	P_196; M_196
NA	no	0	maternal	proband;	P_36; M_36
NA	no	unphased	maternal	mother	M_161
NA	no	unphased	maternal	mother	M_334
NA	no	unphased	maternal	mother	M_372
NA	no	0	maternal	proband;	P_364; M_364
NA	no	unphased	maternal	mother	M_293
NA	no	unphased	paternal	father	F_14
NA	no	1	paternal	proband; f	P_266; F_266
NA	no	unphased	paternal	father	F_413
NA	no	2	paternal	proband; f	P_141; F_141
NA	no	unphased	paternal	father	F_258
NA	no	1	maternal	proband;	P_45; M_45
NA	no	unphased	paternal	father	F_60
NA	no	unphased	NA	proband	P_161
NA	no	1	paternal	father; pro	F_159; P_159
NA	no	1	paternal	proband; f	P_207; F_207
NA	no	1	paternal	proband; f	P_329; F_329
NA	no	unphased	paternal	proband; f	P_277; F_277
NA	no	unphased	paternal	father	F_133
NA	no	unphased	paternal	father	F_269
NA	no	1	maternal	proband;	P_64; M_64
NA	no	1	maternal	proband;	P_185; M_185
NA	no	unphased	paternal	father	F_60
NA	no	1	maternal	proband;	P_372; M_372
NA	no	unphased	maternal	mother	M_372
NA	no	unphased	paternal	father	F_297
NA	no	0	paternal	proband; f	P_36; F_36
NA	no	1	maternal	proband;	P_164; M_164
NA	no	2	maternal	proband;	P_83; M_83
NA	no	unphased	maternal	mother	M_66
NA	no	2	maternal	proband;	P_47; M_47

NA	no	unphased	maternal	mother	M_129
NA	no	unphased	maternal	mother	M_328
NA	no	1	paternal	proband; f	P_47; F_47
NA	no	unphased	paternal	father	F_130
NA	no	unphased	maternal	proband;	P_63; M_63
NA	no	unphased	paternal	father	F_187
NA	no	1	paternal	father; pro	F_159; P_159
NA	no	unphased	maternal	mother	M_48
NA	no	unphased	paternal	father	F_37
NA	no	unphased	maternal	mother	M_204
NA	no	1	maternal	proband;	P_440; M_440
NA	no	1	maternal	proband;	P_253; M_253
NA	no	unphased	maternal	mother	M_437
NA	no	1	NA	proband	P_218
NA	no	1	paternal	proband; f	P_404; F_404
NA	no	unphased	paternal	father	F_10
NA	no	0	paternal	proband; f	P_425; F_425
NA	no	unphased	paternal	father	F_178
NA	no	unphased	maternal	mother	M_294
NA	no	0	maternal	proband;	P_78; M_78
NA	no	unphased	maternal	mother	M_332
NA	no	1	maternal	proband;	P_225; M_225
NA	no	0	maternal	proband;	P_408; M_408
NA	no	1	maternal	proband;	P_397; M_397
NA	no	0	paternal	proband; f	P_137; F_137
NA	no	unphased	NA	cousin 1; c	O1_176; O2_176
NA	no	1	paternal	proband; f	P_386; F_386
NA	no	1	paternal	proband; f	P_404; F_404
NA	no	unphased	paternal	father	F_59
NA	no	1	maternal	proband;	P_225; M_225
NA	no	unphased	paternal	father	F_165
NA	no	unphased	maternal	mother	M_413
NA	no	unphased	NA	mother	M_97
NA	no	1	maternal	proband;	P_313; M_313
NA	no	1	paternal	proband; f	P_110; F_110
NA	no	unphased	paternal	father	F_227

NA	no	unphased	NA	proband	P_61
NA	no	unphased	paternal	father	F_406
NA	no	1	paternal	proband; f	P_144; F_144
NA	no	unphased	paternal	sibling; fat	S_166; F_166
NA	no	1	maternal	proband;	P_228; M_228
NA	no	unphased	maternal	mother	M_227
NA	no	1	maternal	proband;	P_50; M_50
NA	no	unphased	NA	proband	P_233
NA	no	unphased	paternal	father	F_171
NA	no	unphased	paternal	father	F_245
NA	no	unphased	maternal	mother	M_260
NA	no	unphased	NA	aunt	O2_359
NA	no	unphased	paternal	father	F_13
NA	no	1	maternal	proband;	P_308; M_308
NA	no	1	maternal	proband;	P_248; M_248
NA	no	unphased	paternal	father	F_276
2	yes	unphased	DNV	proband	P_132
NA	no	1	paternal	proband; f	P_419; F_419
NA	no	unphased	paternal	father	F_51
NA	no	0	maternal	proband;	P_36; M_36
NA	no	1	paternal	proband; f	P_262; F_262
NA	no	1	paternal	proband; f	P_291; F_291
NA	no	unphased	NA	cousin	O1_398
NA	no	unphased	maternal	mother	M_406
NA	no	unphased	maternal	mother	M_9
NA	no	unphased	NA	father	F_422
NA	no	1	maternal	proband;	P_277; M_277
NA	no	1	paternal	proband; f	P_303; F_303
NA	no	unphased	paternal	father	F_298
NA	no	1	maternal	proband;	P_337; M_337
NA	no	0	paternal	proband; f	P_277; F_277
NA	no	1	paternal	proband; f	P_265; F_265
NA	no	0	NA	proband	P_156
NA	no	1	maternal	proband;	P_439; M_439
NA	no	unphased	NA	proband	P_200
NA	no	unphased	paternal	father	F_268

NA	no	unphased	paternal	father	F_53
NA	no	1	NA	proband	P_133
NA	no	unphased	NA	mother	M_120
NA	no	unphased	paternal	father	F_313
NA	no	unphased	paternal	father	F_203
NA	no	unphased	paternal	father; pro	F_213; P_213
NA	no	unphased	paternal	father	F_350
NA	no	unphased	paternal	father	F_350
NA	no	1	paternal	proband; f	P_333; F_333
NA	no	unphased	NA	father	F_327
NA	no	1	paternal	proband; f	P_105; F_105
NA	no	unphased	NA	cousin	O1_398
NA	no	unphased	maternal	mother	M_19
NA	no	unphased	NA	proband	P_341
NA	no	unphased	maternal	mother	M_179
NA	no	1	maternal	proband;	P_227; M_227
2	yes	1	DNV	proband	P_21
2	yes	1	DNV	proband	P_120
NA	no	unphased	maternal	proband;	P_43; M_43
NA	no	unphased	maternal	proband;	P_41; M_41
NA	no	unphased	NA	father	F_6
NA	no	unphased	maternal	mother	M_39
NA	no	unphased	paternal	proband; f	P_111; F_111
NA	no	unphased	NA	cousin	O_417
NA	no	unphased	maternal	mother	M_66
NA	no	unphased	maternal	mother	M_360
NA	no	1	maternal	proband;	P_160; M_160
NA	no	1	maternal	proband;	P_406; M_406
NA	no	1	paternal	proband; f	P_17; F_17
NA	no	1	maternal	proband;	P_56; M_56
NA	no	1	paternal	proband; f	P_303; F_303
NA	no	1	maternal	proband;	P_46; M_46
NA	no	unphased	maternal	proband;	P_40; M_40
NA	no	unphased	maternal	mother	M_91
NA	no	0	maternal	proband;	P_431; M_431
NA	no	1	paternal	proband; f	P_39; F_39

NA	no	unphased	maternal	mother	M_152
NA	no	unphased	paternal	father	F_277
NA	no	1	maternal	proband;	P_374; M_374
2	no	unphased	NA	proband	P_373
NA	no	1	paternal	proband; f	P_107; F_107
NA	no	0	maternal	proband;	P_61; M_61; S2_61
NA	no	unphased	paternal	father	F_350
NA	no	unphased	paternal	father	F_226
NA	no	unphased	paternal	father	F_40
NA	no	1	paternal	proband; f	P_206; F_206
NA	no	1	maternal	proband;	P_276; M_276
NA	no	1	maternal	proband;	P_148; M_148
NA	no	1	NA	proband	P_167
NA	no	unphased	maternal	mother	M_41
NA	no	unphased	paternal	father	F_108
NA	no	1	maternal	proband;	P_8; M_8
NA	no	1	maternal	proband;	P_291; M_291
NA	no	unphased	paternal	father	F_376
NA	no	3	maternal	proband;	P_352; M_352
NA	no	unphased	maternal	mother	M_297
NA	no	1	NA	proband	P_345
NA	no	unphased	maternal	mother	M_46
NA	no	1	maternal	proband;	P_212; M_212
NA	no	unphased	paternal	father	F_349
NA	no	unphased	maternal	mother	M_425
NA	no	unphased	maternal	mother	M_289
NA	no	1	maternal	proband;	P_255; M_255
NA	no	unphased	paternal	father	F_58
NA	no	1	maternal	proband;	P_312; M_312
NA	no	0	paternal	proband; f	P_37; F_37
NA	no	1	maternal	proband;	P_220; M_220
NA	no	0	maternal	proband;	P_91; M_91
NA	no	unphased	paternal	father	F_139
NA	no	unphased	maternal	mother	M_393
NA	no	unphased	maternal	proband;	P_138; M_138
NA	no	1	maternal	proband;	P_13; M_13

NA	no	unphased	paternal	father	F_292
NA	no	unphased	paternal	father	F_44
NA	no	1	paternal	proband; f	P_125; F_125
NA	no	unphased	paternal	father	F_348
NA	no	unphased	NA	proband	P_341
NA	no	unphased	paternal	father	F_87
NA	no	unphased	NA	proband; s	P_367; S1_368
NA	no	1	NA	proband	P_363
NA	no	unphased	paternal	father	F_264
NA	no	1	maternal	proband;	P_341; M_341
NA	no	unphased	paternal	father	F_303
NA	no	unphased	paternal	father	F_281
NA	no	unphased	paternal	half-sibling	S1_395; F_395; S2_395
NA	no	unphased	maternal	mother	M_110
NA	no	1	paternal	proband; f	P_378; F_378
NA	no	0	paternal	proband; f	P_413; F_413
NA	no	1	NA	proband	P_195
NA	no	1	paternal	proband; f	P_259; F_259
NA	no	unphased	maternal	mother	M_397
NA	no	3	maternal	proband;	P_272; M_272
NA	no	1	paternal	proband; f	P_127; F_127
NA	no	unphased	maternal	mother	M_289
NA	no	3	paternal	proband; f	P_412; F_412; S_412
NA	no	1	maternal	proband;	P_209; M_209
NA	no	unphased	maternal	mother	M_210
NA	no	1	paternal	proband; f	P_85; F_85
NA	no	1	paternal	proband; f	P_206; F_206
NA	no	unphased	paternal	father	F_146
NA	no	unphased	maternal	mother	M_413
NA	no	2	paternal	proband; f	P_179; F_179
NA	no	1	maternal	proband;	P_269; M_269
2	no	1	DNV	proband	P_320
NA	no	unphased	maternal	mother	M_149
NA	no	unphased	paternal	father	F_91
NA	no	1	maternal	proband;	P_359; M_359
NA	no	1	paternal	proband; f	P_67; F_67

NA	no	1	maternal	proband;	P_376; M_376
NA	no	unphased	paternal	father	F_9
NA	no	unphased	maternal	mother	M_160
NA	no	1	maternal	proband;	P_361; M_361
NA	no	unphased	paternal	father	F_46
NA	no	unphased	maternal	mother	M_346
NA	no	1	paternal	proband; f	P_333; F_333
NA	no	unphased	maternal	mother	M_352
NA	no	unphased	maternal	mother	M_39
NA	no	unphased	paternal	father	F_267
NA	no	0	NA	proband	P_162
NA	no	1	maternal	proband;	P_278; M_278
NA	no	unphased	paternal	father	F_296
NA	no	1	paternal	proband; f	P_94; F_94
NA	no	unphased	paternal	father	F_98
NA	no	0	maternal	proband;	P_15; M_15
NA	no	unphased	maternal	mother	M_22
NA	no	1	maternal	proband;	P_167; M_167
NA	no	1	paternal	proband; f	P_25; F_25
NA	no	unphased	maternal	mother	M_344
NA	no	unphased	maternal	proband;	P_348; M_348
NA	no	unphased	maternal	mother	M_275
NA	no	unphased	maternal	mother	M_313
NA	no	1	paternal	proband; f	P_239; F_239
NA	no	unphased	NA	cousin; un	O1_136; O3_136
NA	no	1	paternal	proband; f	P_176; F_176
2	no	1	NA	proband	P_122
NA	no	unphased	paternal	father	F_336
NA	no	unphased	NA	proband	P_117
NA	no	2	paternal	proband; f	P_309; F_309
NA	no	unphased	maternal	mother	M_122
NA	no	1	maternal	proband;	P_282; M_282
NA	no	1	NA	proband	P_72
NA	no	0	paternal	proband; f	P_323; F_323
NA	no	unphased	maternal	mother	M_187
NA	no	unphased	paternal	father	F_359

NA	no	2	maternal	proband;	P_13; M_13
NA	no	unphased	paternal	father	F_212
NA	no	1	paternal	proband; f	P_220; F_220
NA	no	unphased	maternal	mother	M_165
NA	no	unphased	maternal	mother	M_434
NA	no	0	NA	proband	P_196
NA	no	unphased	maternal	mother	M_120
NA	no	unphased	maternal	mother	M_336
NA	no	unphased	paternal	father	F_7
NA	no	unphased	paternal	father	F_104
NA	no	unphased	paternal	father	F_216
NA	no	unphased	paternal	father	F_16
NA	no	1	maternal	proband;	P_374; M_374
NA	no	2	paternal	proband; f	P_27; F_27
NA	no	unphased	maternal	mother	M_115
NA	no	unphased	maternal	mother	M_91
NA	no	unphased	paternal	father	F_207
NA	no	unphased	maternal	mother	M_433
NA	no	1	maternal	proband;	P_12; M_12
NA	no	unphased	paternal	father	F_349
NA	no	0	maternal	proband;	P_382; M_382
NA	no	unphased	maternal	mother	M_251
NA	no	1	paternal	proband; f	P_329; F_329
NA	no	1	paternal	proband; f	P_344; F_344
NA	no	unphased	maternal	mother	M_31
NA	no	1	maternal	proband;	P_184; M_184
NA	no	1	paternal	proband; f	P_216; F_216
NA	no	2	maternal	proband;	P_122; M_122
NA	no	1	maternal	proband;	P_51; M_51
NA	no	unphased	NA	cousin	O1_398
NA	no	unphased	paternal	father	F_427
NA	no	unphased	paternal	father	F_108
NA	no	unphased	NA	proband	P_200
NA	no	1	maternal	proband;	P_175; M_175
NA	no	2	maternal	proband;	P_256; M_256
NA	no	1	maternal	proband;	P_220; M_220

NA	no	unphased	NA	proband; s	P_339; S_339
NA	no	1	maternal	proband;	P_138; M_138
NA	no	unphased	paternal	father	F_210
NA	no	unphased	paternal	father	F_408
NA	no	1	maternal	proband;	P_25; M_25
NA	no	unphased	maternal	mother	M_388
NA	no	unphased	paternal	proband; f	P_134; F_134
NA	no	unphased	paternal	father	F_304
NA	no	1	paternal	proband; f	P_2; F_2
NA	no	1	paternal	proband; f	P_2; F_2
NA	no	unphased	paternal	father	F_178
NA	no	1	paternal	proband; f	P_144; F_144
NA	no	unphased	maternal	mother	M_56
NA	no	unphased	paternal	father	F_352
NA	no	0	maternal	proband;	P_318; M_318
NA	no	1	paternal	father; pro	F_396; P_396; S1_396
NA	no	1	maternal	proband;	P_261; M_261
NA	no	unphased	NA	proband	P_417
NA	no	unphased	paternal	father	F_237
NA	no	unphased	paternal	father; sibl	F_267; S_267
NA	no	1	maternal	proband;	P_169; M_169
NA	no	unphased	paternal	father	F_181
NA	no	2	maternal	proband;	P_441; M_441
NA	no	unphased	paternal	father	F_423
NA	no	unphased	maternal	mother	M_87
NA	no	unphased	paternal	father	F_11
NA	no	unphased	paternal	father	F_174
NA	no	unphased	paternal	father	F_198
NA	no	1	maternal	proband;	P_51; M_51
NA	no	unphased	maternal	proband;	P_72; M_72
NA	no	1	paternal	proband; f	P_299; F_299
NA	no	unphased	NA	father	F_231
NA	no	1	maternal	proband;	P_20; M_20
NA	no	1	paternal	proband; f	P_145; F_145
NA	no	unphased	paternal	father	F_243
NA	no	unphased	paternal	father	F_34

NA	no	unphased	paternal	father	F_111
NA	no	1	maternal	proband;	P_106; M_106
NA	no	unphased	paternal	father	F_177
NA	no	0	NA	proband	P_235
NA	no	unphased	maternal	proband;	P_277; M_277
NA	yes	unphased	NA	cousin	O_417
NA	no	unphased	maternal	mother	M_129
NA	no	unphased	maternal	mother	M_118
NA	no	unphased	maternal	mother	M_30
NA	no	1	maternal	proband;	P_273; M_273
NA	no	unphased	paternal	father	F_340
NA	no	unphased	paternal	father	F_203
NA	no	1	paternal	proband; f	P_212; F_212
NA	no	3	maternal	proband;	P_190; M_190
NA	no	unphased	maternal	mother	M_28
NA	no	2	maternal	proband;	P_43; M_43
NA	no	unphased	paternal	proband; f	P_287; F_287
NA	no	1	maternal	proband;	P_135; M_135
NA	no	unphased	paternal	father	F_313
NA	no	unphased	paternal	father	F_287
NA	no	1	maternal	proband;	P_396; M_396
NA	no	1	maternal	proband;	P_97; M_97
NA	no	1	paternal	proband; f	P_228; F_228
NA	no	unphased	paternal	father	F_168
NA	no	unphased	maternal	mother	M_173
NA	no	unphased	maternal	proband;	P_73; M_73
NA	no	unphased	maternal	mother	M_47
NA	no	unphased	paternal	father	F_380
NA	no	unphased	maternal a	mother; fa	M_24; F_24
NA	no	unphased	paternal	father	F_187
NA	no	unphased	paternal	father	F_39
NA	no	3	maternal	proband;	P_260; M_260
NA	no	unphased	paternal	father	F_108
NA	no	1	paternal	proband; f	P_2; F_2
NA	no	unphased	NA	mother	M_33
NA	no	0	paternal	proband; f	P_287; F_287

NA	no	1	maternal	proband;	P_23; M_23
NA	no	1	paternal	proband; f	P_77; F_77
NA	no	1	paternal	proband; f	P_136; F_136
NA	no	unphased	NA	cousin	O_417
NA	no	unphased	paternal	father	F_122
NA	no	unphased	maternal	mother	M_282
NA	no	1	paternal	proband; f	P_104; F_104
NA	no	1	maternal	proband;	P_62; M_62
NA	no	unphased	paternal	father	F_213
NA	no	unphased	maternal	mother	M_433
NA	no	unphased	NA	proband	P_161
NA	no	0	paternal	proband; f	P_128; F_128
NA	no	unphased	maternal	mother	M_434
NA	no	1	paternal	proband; f	P_116; F_116
NA	no	3	NA	proband	P_359
NA	no	unphased	NA	father	F_82
NA	no	1	maternal	proband;	P_47; M_47
NA	no	unphased	paternal	father	F_273
NA	no	1	maternal	proband;	P_215; M_215
NA	no	2	paternal	proband; f	P_93; F_93
0	yes	unphased	NA	proband	P_365
2	yes	unphased	DNV	proband	P_264
NA	no	unphased	maternal	proband;	P_340; M_340
NA	no	unphased	NA	mother	M_226
NA	no	unphased	maternal	proband;	P_12; M_12
NA	no	unphased	NA	mother	M_429
NA	yes	unphased	NA	cousin; mo	O1_398; M_398; O2_398
NA	no	unphased	NA	mother	M_304
NA	no	unphased	maternal	proband;	P_140; M_140
NA	no	unphased	NA	mother	M_187
NA	no	unphased	NA	father	F_210
2	yes	unphased	NA	proband	P_39
NA	no	unphased	maternal	proband;	P_290; M_290
NA	no	unphased	NA	proband	P_329
NA	no	unphased	NA	father	F_377
NA	no	unphased	maternal	proband;	P_172; M_172

	2 yes	unphased	NA	proband	P_258
NA	no	unphased	NA	father	F_294
NA	no	unphased	NA	father	F_294
NA	no	unphased	NA	father	F_392
NA	no	unphased	NA	mother	M_366
NA	no	unphased	NA	father	F_59
NA	no	unphased	maternal	proband;	P_141; M_141
NA	no	unphased	NA	father	F_134
NA	no	unphased	maternal	proband;	P_205; M_205
NA	no	unphased	NA	mother	M_229
NA	no	unphased	NA	mother	M_16
NA	no	unphased	NA	father	F_181
NA	no	unphased	NA	mother	M_413
NA	no	unphased	NA	mother	M_390
NA	no	unphased	maternal	proband;	P_226; M_226
NA	no	unphased	maternal	proband;	P_286; M_286
NA	no	unphased	NA	mother	M_329
NA	no	unphased	NA	father	F_173
NA	no	unphased	NA	mother	M_84
NA	no	unphased	NA	mother	M_216
NA	no	unphased	NA	father	F_127
NA	no	unphased	NA	proband	P_385
NA	no	unphased	NA	mother	M_248
	2 yes	unphased	NA	proband	P_65
	2 yes	unphased	NA	proband	P_128
NA	no	unphased	NA	father	F_244
NA	no	unphased	NA	father	F_245
NA	no	unphased	NA	father	F_365
NA	no	unphased	paternal	proband; f	P_255; F_255
NA	no	unphased	paternal	proband; f	P_413; F_413
