# Supplementary material for: Genomic landscape of autism spectrum disorder in Brazil
Source: Genet Mol Biol. 2026 Sep 7;49(Suppl 1):e20250247. doi: 10.1590/1678-4685-GMB-2025-0247 (PMC13561403; doi:10.1590/1678-4685-GMB-2025-0247)
Supplement: Supplementary file 10 [file 1415-4757-GMB-49-s1-e20250247-s6.pdf]

Genetics and Molecular Biology
https://doi.org/10.1590/1678-4685-GMB-2025-0247

Supplementary Material to "Genomic landscape of autism spectrum disorder in Brazil"

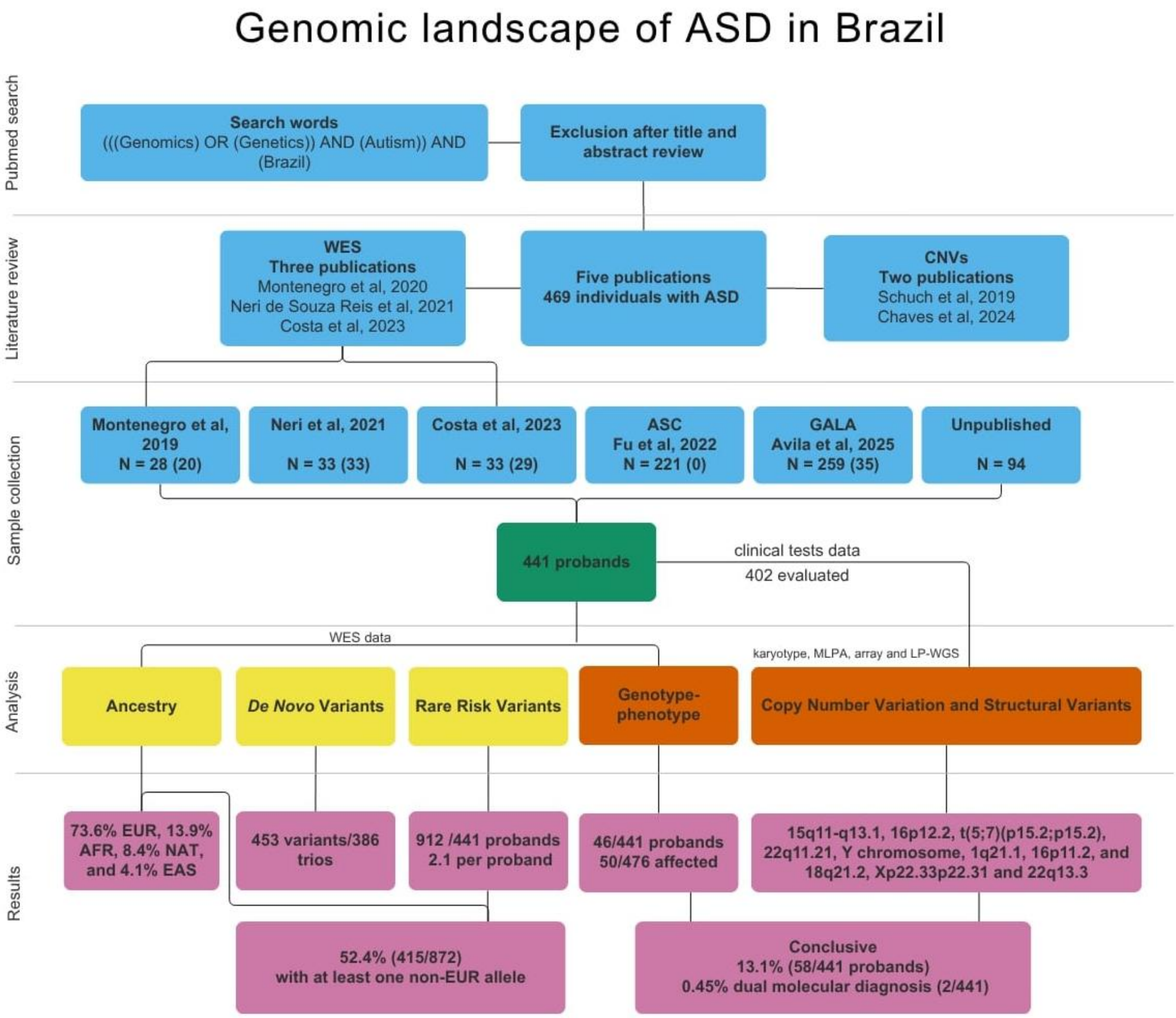

Figure S1 - Study flowchart describing the main steps of the study. The numbers in parentheses in the sample collection section represent exclusive cases from each study, while yellow and orange boxes in the analysis section denote exploratory and clinical analyses, respectively. ASC, Autism Sequencing Consortium. GALA, Genomics of Autism in Latin Ancestries Consortium.
