# Supplementary material for: Genomic landscape of autism spectrum disorder in Brazil
Source: Genet Mol Biol. 2026 Sep 7;49(Suppl 1):e20250247. doi: 10.1590/1678-4685-GMB-2025-0247 (PMC13561403; doi:10.1590/1678-4685-GMB-2025-0247)
Supplement: Supplementary file 11 [file 1415-4757-GMB-49-s1-e20250247-s7.pdf]

Genetics and Molecular Biology
https://doi.org/10.1590/1678-4685-GMB-2025-0247

Supplementary Material to “Genomic landscape of autism spectrum disorder in Brazil”

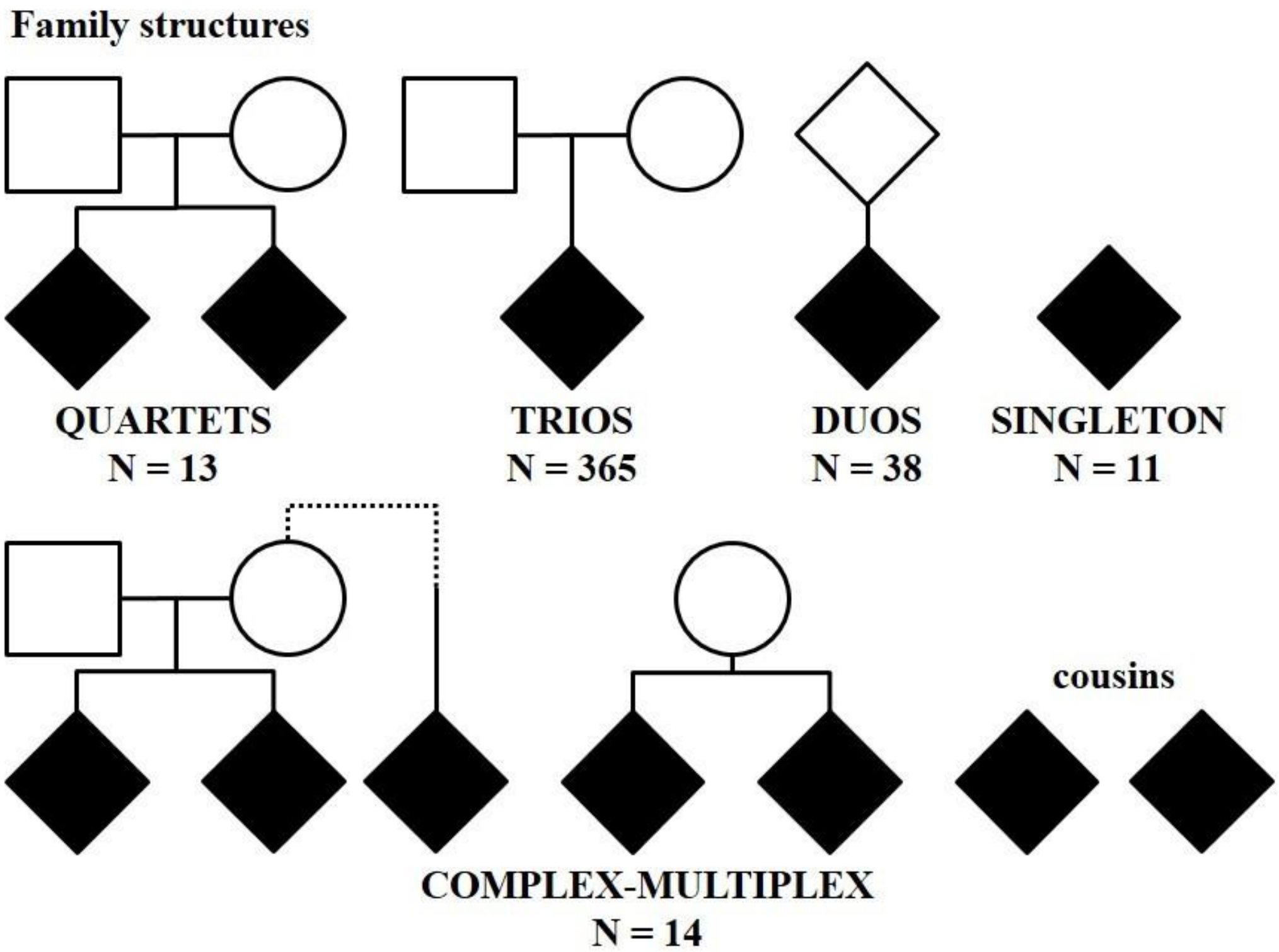

Figure S2 - Diagram of the different family structure types included in the study, such as quartets, trios, duos, singleton, and multiplex families.
